# Systematic analysis of the regulatory roles of microRNAs in postnatal maturation and metergasis of liver of breeder cocks

**DOI:** 10.1038/s41598-017-18674-3

**Published:** 2018-01-08

**Authors:** Shengru Wu, Wei Guo, Saisai Liang, Hong Lu, Wenqiang Sun, Xiaochun Ren, Qingzhu Sun, Xiaojun Yang

**Affiliations:** 1 College of Animal Science and Technology, Northwest A&F University, Yangling, 712100 Shaanxi China; 2 Dazhou Institute of Agricultural Sciences, Dazhou, 635000 Sichuan China

## Abstract

The liver function of chickens is intensively remodeled from birth to adult, which was validated by metabolomics research in the present study. In order to understand the roles of microRNAs (miRNA) in liver maturation and metergasis, miRNA expression profiles in livers of 20 male chicks aged one day and five adult cocks aged 35 weeks were determined. A total of 191 differentially expressed miRNAs with the criteria of P < 0.05 and fold changes either >1.5 or <0.67 and 32 differentially expressed miRNAs with the criteria of false discovery value (FDR) < 0.05 and fold changes either >1.5 or <0.67 were detected. Subsequently, Gene Ontology and Kyoto Encyclopedia of Genes and Genomes analyses of the targets revealed that candidate miRNAs may involve in the regulation of hepatic metabolism and immune functions, and some pathways including cell cycle which were implicated in postnatal liver development. Furthermore, 1211 differentially expressed mRNAs (messenger RNA) in livers between the postnatal and matured chickens were used to define the roles of differentially expressed miRNAs in regulating the expression of target genes. Our results revealed the first miRNA profile related to the adaption of mature liver functions after birth in breeder cock.

## Introduction

To date, most research in the organogenesis and development of chicken livers have focused on the fetal stage^1^. However, the liver, which is mainly hematopoietic in the embryo^2^ but converts to a major metabolism-regulatory tissue in the adult, is extensively remodeled after birth to adapt to and perform adult functions^1,3^. After birth, hepatocytes undergo a process of acquisition of various functions of the mature liver and the main metabolic functions are obtained^3,4^, which means there are substantial changes in postnatal liver maturation and metergasis. Most gene regulation studies in the liver have focused on several post-transcriptional mechanisms, such as alternative pre-messenger RNA splicing and long non-coding RNAs, which have essential roles in sequential replacement of fetal-to-adult and postnatal liver maturation processes^5,6^. However, the main microRNAs (miRNA) in chicken livers and differentially expressed miRNAs, which are involved in postnatal liver maturation and metergasis, remain unknown.

The miRNAs are a series of endogenous non-coding RNAs, about 22 nucleotides (nt) long, which repress gene expression by binding to the target mRNAs (messenger RNA)^7^. Furthermore, miRNAs could regulate gene expression at either transcriptional^8^ or post-transcriptional levels^9^, guide the remodeling of chromatin^10^, and result in de novo DNA methylation^11^. Since miRNAs could regulate multiple aspects of biological process, it is not surprising that they are involved in liver maturation. For example, miR-122 could contribute to the liver development by regulating the balance between proliferation and differentiation of hepatocytes by targeting CCAAT displacement protein (CUTL1, also known as CDP)^12,13^. Moreover, the spatial and temporal patterns of miRNA expression are suggestive of functional roles in hepatic development and function^14^. Meanwhile, hepatocyte apoptosis and hepatocyte regeneration process also could be regulated by miRNAs^15^. Herein, as a most studied post-transcriptional mechanism in animals, miRNAs should also play important roles in postnatal liver maturation and metergasis.

As an important breeder animal, one breeder cock produces around 1000 broilers per year^16^. There are several serious metabolic diseases occurred during the feeding of breeder cocks, such as fatty liver and ascites syndrome^17^, which are induced by incorrect metabolic regulation and could further influence the usability of breeder cocks. It’s important to clarify the postnatal liver maturation process and the hepatic function changes after birth so that the hepatic metabolic or immune condition of breeder cocks could be better regulated and the breeder cocks could be used maximally. Hence, we tried to identify the functional differences between immature and mature livers of breeder cocks and study the potential regulatory roles of miRNAs in postnatal liver metergasis and maturation.

We hypothesized that postnatal liver maturation and metergasis of breeder cocks were related to their differential miRNAs expression profiles. To test this hypothesis, we firstly identified differential metabolites and studied the functional changes between immature and mature livers by using a gas chromatography-mass spectrometer (GC-MS)-based metabolomic study. Furthermore, the miRNAs in the livers of one-day-old and 35-week-old Arbor Acres breeder cocks were identified to find out the differentially expressed miRNAs related to postnatal liver maturation and metergasis by using 6 livers miRNA transcriptome libraries. Moreover, in order to test the roles of differentially expressed miRNAs, the differentially expressed mRNAs screened out by 6 mRNA transcriptome libraries from the same livers samples were used to perform the integrated analyses between differentially expressed miRNAs and mRNAs. Taken together, these expression profiles could especially clarify the roles of miRNAs in postnatal liver maturation and acquirement of various functions of the mature liver.

## Results and Discussion

### Metabolic changes in the liver from postnatal to matured breeder cocks

**Figure 1:**
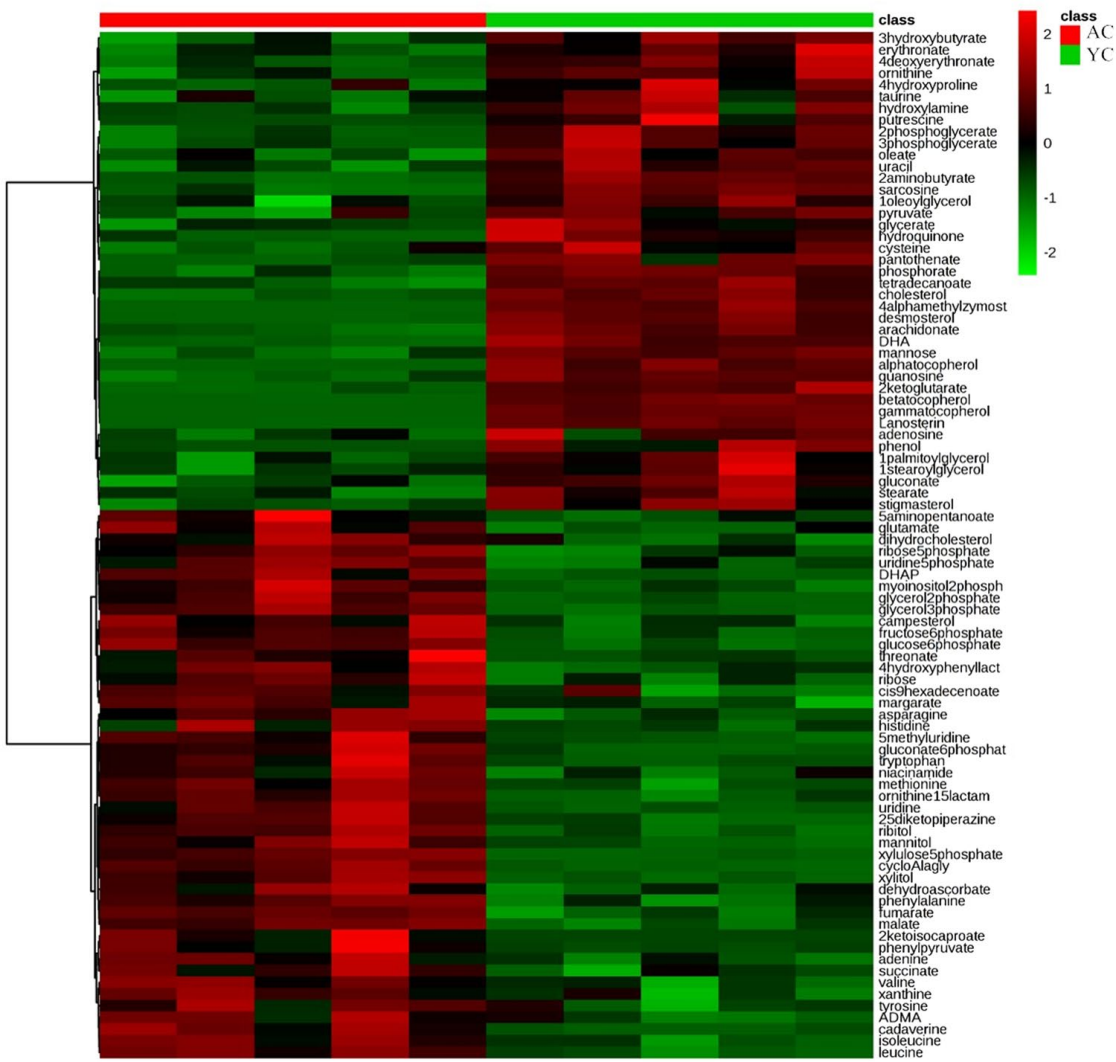
The heat map of differential metabolites in livers between the postnatal young chicks (YC) and adult chickens (AC). Notes: The up-regulated metabolites are depicted in red color whereas the down-regulated metabolites are depicted in green color.

The metabolic changes in the liver between the postnatal and matured chickens were examined in this study. The principal component analysis (PCA; R^2^X = 0.597, Figure S1a) showed that the postnatal and matured livers distributed in two separate areas, indicating a markedly different metabolome between the postnatal and matured livers. Furthermore, the partial least squares discriminant analysis (PLS-DA) score plot (R^2^Y = 0.998, Q^2^ = 0.963) also found a dramatic difference between the two aged livers (Figure S1b). To identify the metabolites that were mainly contributing to the metabolomic distinctions between two ages, we carried out supervised models the orthogonal projection of latent structures–discriminant analysis (OPLS-DA; R^2^Y = 0.998, Q^2^ = 0.966) to distinguish the differential metabolites (Figure S1c). With the criteria of the variable importance in the projection (VIP) value of OPLS-DA model larger than 1 and P values of univariate statistical analysis lower than 0.05, a total of 88 significantly differential metabolites were identified (Fig. 1), which indicated massive metabolic changes from the postnatal to maturation processes. The involved metabolic pathways were further mapped with identified significantly altered metabolites. As results, 12 metabolic pathways involved in amino acid metabolism, steroid metabolism, and glycometabolism were standing out (Table 1).

**Table 1:** The significantly enriched pathway of differential metabolites with P < 0.05.

Pathway	Total	Hits	P value	=−LOG(p)	Impact
Pentose phosphate pathway	20	7	<0.000	9.8799	0.48901
Aminoacyl-tRNA biosynthesis	44	10	<0.000	9.4682	0
Valine, leucine and isoleucine biosynthesis	10	5	<0.000	9.3031	0
Phenylalanine, tyrosine and tryptophan biosynthesis	4	3	<0.000	7.3815	1
Alanine, aspartate and glutamate metabolism	23	6	0.001	6.8106	0.34333
Butanoate metabolism	18	5	0.002	6.1264	0
Phenylalanine metabolism	8	3	0.007	4.904	0.58975
Glutathione metabolism	26	5	0.012	4.4353	0.04452
Steroid biosynthesis	28	5	0.016	4.1202	0.34782
Citrate cycle (TCA cycle)	20	4	0.021	3.8552	0.16093
D-Glutamine and D-glutamate metabolism	5	2	0.027	3.6113	1
Taurine and hypotaurine metabolism	6	2	0.039	3.2419	0.5
Glycolysis or Gluconeogenesis	26	4	0.050	2.9783	0.17596

Note: (1) the Total is the total number of compounds in the pathway; (2) the Hits is the actually matched number from the uploaded data; (3) the Impact is the pathway impact value calculated from pathway topology analysis.

According to our results, we found that the matured livers had higher metabolic efficiencies in pentose phosphate pathway, Citrate cycle (TCA cycle), and glycolysis/gluconeogenesis, which concluded from the increased concentrations of fumaric acid, succinic acid, malic acid, glucose-6-phosphate, fructose-6-phosphate, dihydroxyacetone phosphate, and so on. Similarly, the metabolic efficiency of some amino acid metabolism pathways, including biosynthesis of valine, leucine, isoleucine, phenylalanine, tyrosine, and tryptophan, as well as the metabolism of tyrosine, tryptophan, glycine, serine, threonine, phenylalanine, cysteine and methionine, were also significantly increased. Meanwhile, the concentrations of metabolites involved in purine and pyrimidine metabolism were also increased, which indicated an enhanced function in nucleic acid metabolism. However, the concentrations of metabolites related to lipid metabolic process, including fatty acid metabolism, as well as lipid and steroid biosynthesis, were significantly decreased. Besides, the concentrations of arginine and proline metabolism were also decreased, which indicated that the lipid metabolism, arginine and proline metabolism were crucial for the development of the postnatal liver and chicks.

To sum up, livers underwent hypertrophic growth and maturation via large-scale changes in metabolic functions after birth^3^. Diverse genetic mechanisms including miRNAs, which ensure these changes occur precisely to initiate proper cell growth, differentiation, as well as functional changes, have been involved in postnatal liver maturation and metergasis^3–5^. Herein, based on the functional changes found in the metabolomics studies, potential roles of miRNAs in regulating postnatal liver maturation were further studied by miRNA sequencing in the present study.

### The miRNA expression profiles of liver tissues

In the present study, six miRNA expression profiles of the livers from one-day-old male chicks and adult cocks were gathered. These six transcriptomes provided a total of 11 to 20 million raw reads separately (Table S1). After filtering for invalid reads (see Methods), a total of 6,429,847, 8,813,361, and 11,416,566 valid reads from three duplicate samples of the postnatal chicks and 7,554,585, 5,484,721, and 4,097,655 valid reads from three duplicate samples from adult cocks were obtained (Table S1). The discovery of mRNA and tRNA indicated that the prepared RNA for sequencing was of good quality. Length distribution analyses showed that most of the total clean reads had lengths of 21 to 24 nt (Figure S2), which is consistent with characteristics of miRNAs^18^.

### Identification of known and novel miRNAs in chicken liver tissues

According to a series of bioinformatic analyses, we identified 1,757 kinds of miRNAs. In order to distinguish the expressed miRNAs into known and potentially novel miRNAs, we carried out advanced bioinformatic analyses and divided the clean reads from those 1,757 miRNAs into five groups (Table S2). All miRNAs in group 4 were novel miRNAs, and miRNAs in group 2, 3, and 4 were also new miRNAs in chicken (Table S2). In total, there were 1,152 known and 605 novel miRNAs in those six liver libraries (Table S3 and Table S4). Through Venn diagrams analysis, 627 miRNAs could be identified in all three postnatal livers and 586 miRNAs could be identified in all three matured livers. Furthermore, the Venn diagrams analysis between two age groups demonstrating that 498 miRNAs were both expressed in the postnatal and matured livers (Figure S3); 129 miRNAs were only shared in the postnatal livers and 88 were only shared in matured livers. Specifically, those 129 miRNAs and 88 miRNAs could also play crucial roles in regulating the special functions of liver related to different ages.

### Differentially expressed miRNAs in immature and mature livers

**Figure 2:**
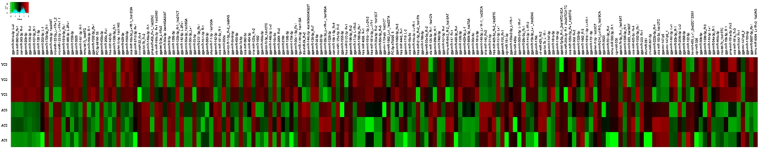
The heat map of differentially expressed miRNAs for livers between the postnatal chicks (YC) and adult chickens (AC). Notes: The up-regulated miRNAs are depicted in red color whereas the down-regulated miRNAs are depicted in green color. YC1, YC2, and YC3 represent the results of 3 replications from the postnatal young chicks using RNA sequencing and AC1, AC2, and AC3 represent the results of 3 replications from adult chickens using RNA sequencing.

Based on the criteria of *P* < 0.05 and fold changes either >1.5 or <0.67, we gathered 191 differentially expressed miRNAs; of these, 72 miRNAs were up-regulated and 119 miRNAs were down-regulated in matured livers relative to the postnatal liver (Fig. 2). Those 56 potentially novel miRNAs and 135 known miRNAs could play important roles in regulating the postnatal liver maturation in chicken. Among those differentially expressed miRNAs, the gga-miR-122 was the highest one. Several studies have demonstrated that miR-122 was one of the tissue-specific miRNA and highest expressed miRNAs in liver^19^. This miRNA can contribute to the liver development by regulating the balance between proliferation and differentiation of hepatocytes, at least by targeting CUTL1^12^. Meanwhile, it can also influence liver polyploidization and further liver maturation, by directly influencing the targets expression of miR-122, which was related to cytokinesis process^13^. In our experiment, the gga-miR-122 was significantly increased in matured liver, which could indicate that miR-122 plays crucial roles in postnatal liver maturation. In additional, of the top 10 abundantly differentially expressed miRNAs identified in this study, some of them, such as miR-21^20^, miR-30c^14^, miR-191^21^, let-7a and miR-22^22^ have also been reported to play important roles in the liver development. Moreover, we also found that miRNA-30 family and let-7 family were significantly differentially expressed between the postnatal and matured livers and were abundant in the liver, whose important roles in the liver development have been proved^14,22^.

Furthermore, in order to reduce the false positives of our identified differentially expressed miRNAs, we further screened out the differentially expressed miRNAs by using the criteria of the false discovery value (FDR) < 0.05 and fold changes either >1.5 or <0.67. As results, a total of 32 differentially expressed miRNAs were identified between the immature and mature livers, of these, 11 miRNAs were up-regulated and 21 miRNAs were down-regulated in matured livers relative to the postnatal immature livers (Table 2). Among those miRNAs, miR-125b was the highest differentially expressed miRNAs in the present study and was proved to take part in the functional changes of matured livers^23^. Similarly, some other highly expressed differential miRNAs (FDR < 0.05), such as miR-107-3p, let-7b, and miR-181b, have all been proved to take part in the regulation of postnatal liver development or metergasis process^22,24,25^. Those previous results suggested the important roles of our identified differentially expressed miRNAs in regulating postnatal liver maturation and metergasis.

**Table 2:** Summary of differentially expressed miRNAs which selected with the criteria of FDR < 0.05 and fold changes either >1.5 or <0.67 in livers between the postnatal (YC) and matured chickens (AC).

Name	Sequence	AC1	AC2	AC3	YC1	YC2	YC3	Fold Changes (AC/YC)	FDR value
gga-miR-125b-5p	TCCCTGAGACCCTAACTTGTGA	58677.75	55626.50	56667.41	32650.48	26424.53	23446.84	2.07	0.03
aca-miR-191-5p_R-1	CAACGGAATCCCAAAAGCAGCT	57152.94	75715.60	64057.34	16422.00	14792.69	11220.11	4.64	0.04
gga-miR-107-3p_R-1	AGCAGCATTGTACAGGGCTATC	6369.26	7804.42	7933.42	17972.62	16911.23	17611.13	0.42	0.02
gga-let-7b_R + 2	TGAGGTAGTAGGTTGTGTGGTTTT	2391.77	2699.59	2956.35	504.96	423.82	342.41	6.33	0.02
aca-miR-363-3p_R + 1	AATTGCACGGTATCCATCTGTA	2108.82	3097.80	2316.88	10026.60	9279.55	9535.19	0.26	0.02
oan-miR-363-3p_R + 1	AATTGCACGGTATCCATCTGTA	2108.82	3097.80	2316.88	10026.60	9279.55	9535.19	0.26	0.02
gga-miR-181b-5p_R + 1	AACATTCATTGCTGTCGGTGGGT	1989.16	1916.79	2396.51	5621.88	5157.39	4870.29	0.40	0.03
gga-miR-20b-5p	CAAAGTGCTCATAGTGCAGGTAG	1832.73	2452.51	2603.00	8999.41	10104.73	9234.32	0.24	0.02
gga-miR-458a-3p	ATAGCTCTTTGAATGGTACTGC	1085.15	1143.90	984.38	211.99	166.56	198.19	5.57	0.02
gga-miR-456-3p_1ss22AT	CAGGCTGGTTAGATGGTTGTCT	857.08	899.48	633.92	2657.60	2921.09	3110.16	0.28	0.02
gga-miR-1729-5p_R-1	ATCCCTTACTCACATGAGTAGT	706.42	675.66	753.52	278.68	361.75	353.20	2.15	0.03
rno-miR-122-5p_L + 1 R + 1_2	CTGGAGTGTGACAATGGTGTTTGT	582.92	585.29	541.57	312.62	363.88	361.05	1.65	0.03
rno-miR-122-5p_L + 1 R + 1_1	CTGGAGTGTGACAATGGTGTTTGA	582.92	585.29	541.57	312.62	363.88	361.05	1.65	0.03
gga-miR-146a-3p_R-1	ACCCATGGGGCTCAGTTCTTCA	469.30	520.09	579.09	161.97	138.97	125.58	3.68	0.03
mmu-let-7j_1ss8TG	TGAGGTAGTAGTTTGTGCTGTTAT	450.36	394.26	459.49	145.89	143.22	63.77	3.70	0.04
gga-miR-1451-3p_R-1	CGTAACTCGCTGCTGTGAGAGG	97.15	94.56	85.29	169.11	188.83	186.41	0.51	0.03
gga-miR-456-3p_R-1	CAGGCTGGTTAGATGGTTGTC	55.16	58.34	37.52	167.33	159.66	166.79	0.31	0.02
gga-miR-106-3p_R + 1	ACTGCAGTATAAGCACTTCTGGC	54.34	57.19	63.81	307.26	285.90	288.45	0.20	0.00
gga-miR-130b-5p_R-1	CCTCTTTCCCTGTTGCACTAC	46.93	57.96	39.12	176.26	141.63	164.83	0.30	0.04
gga-miR-301a-5p_L + 2	GCTCTGACAATGTTGCACTACT	26.35	44.23	33.99	226.28	214.29	233.51	0.16	0.02
cin-miR-33_R + 4	GTGCATTGTAGTTGCATTGCAAT	23.05	22.12	22.45	0.00	3.18	3.92	9.52	0.02
aca-miR-18a-3p_L + 1R-1_1ss12GA	TACTGCCCTAAATGCTCCTTCT	21.41	28.22	20.52	86.94	107.15	98.11	0.24	0.03
gga-miR-1559-3p	AGTTACATGTATGCATCGAGCA	11.53	8.39	10.90	25.01	27.58	29.43	0.38	0.03
gga-miR-128-1-5p	CGGGGCCGTAACACTGTCTGAGA	8.23	12.96	13.47	38.11	42.43	39.24	0.29	0.02
aca-miR-363-5p	GTGGATCACGATGCAATTTTGA	4.94	2.54	3.95	25.21	27.94	34.34	0.13	0.04
pma-miR-456_R + 1_1ss22AC	CAGGCTGGTTAGATGGTTGTCCC	4.94	2.29	3.85	13.10	13.79	13.74	0.27	0.03
gga-let-7j-3p_L-1R + 2	TATACAGTCTATTGCCTTCCTTT	3.17	2.94	3.00	0.95	0.34	0.25	5.92	0.03
gga-miR-3529	AGGCAGACTGTGACTTGTTGT	1.65	4.58	3.85	16.08	14.85	13.74	0.23	0.03
PC-3p-68190_58	CTGATGAGGATCTTAAAA	1.65	0.00	0.00	13.10	10.61	13.74	0.04	0.03
gga-miR-202-5p_L-1	TTCCTATGCATATACTTCTTT	0.00	0.76	1.28	7.15	6.37	5.89	0.11	0.03
gga-miR-489-3p_L-2R + 1	TGACATCATATGTACGGCTGCT	0.00	0.76	0.00	9.53	8.49	7.85	0.03	0.02
gga-miR-6710-3p_R + 1	AAACTGTTCTCTTCCATCTAGT	0.00	0.38	1.28	4.76	4.24	4.91	0.12	0.04

### Validation of differentially expressed miRNAs

**Figure 3:**
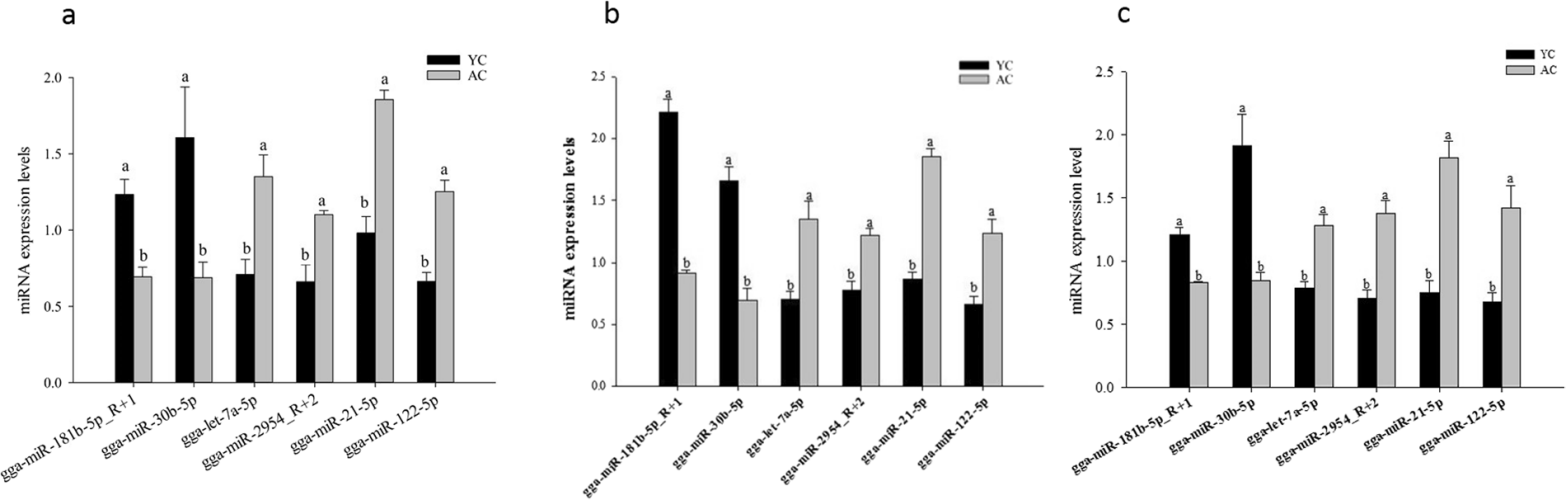
Eight differentially expressed miRNAs, which were validated by reverse-transcription quantitative polymerase chain reaction. Notes: 1. AC represent the livers of adult chickens, YC represent the livers of the postnatal young chickens; 2. Superscripts (**a**,**b**) on bars denote significantly different expression levels in the same miRNAs (*P* < 0.05). 3. U6 (**a**), 5 S (**b**), 18 S (**c**) were respectively used as internal control gene for normalization in our experiments. The data are all presented as means ± SE (for postnatal chicks: n = 20; for adult chickens: n = 5).

The expression levels of eight differentially expressed miRNAs between the postnatal and matured livers were assessed by stem-loop qRT-PCR. By using three different endogenous control miRNAs (U6, 5S, and 18S), the results have all showed that two miRNAs (gga-miR-181b-5p_R + 1 and gga-miR-30b-5p) had significantly lower expression levels in matured livers than the postnatal livers, and the expression level of another four miRNAs (gga-let-7a-5p, gga-miR-2954_R + 2, gga-miR-21-5p, and gga-miR-122-5p) were significantly increased in matured livers (Fig. 3), which were consistent with the miRNA-seq data. Moreover, we could detect the expression of those two novel miRNAs (PC-5p-479025_5 and PC-3p-68190_58) in the postnatal livers but could not detect their expression in the matured livers, which were also consistent with the sequencing results. According to the results of the stem-loop qRT-PCR, two novel miRNAs could be both detected, and the different expression trends observed in the miRNA-seq data and in the qRT-PCR were uniform in the postnatal and matured livers, suggesting that the miRNA expression profiles could represent actual miRNA expression levels in the present study.

### Target genes of differentially expressed miRNAs

A total of 5,074 target genes were predicted based on those 191 differentially expressed miRNAs with the criteria of P < 0.05 (Tables S5), and 2,837 target genes were predicted based on those 32 differentially expressed miRNAs with the criteria of FDR < 0.05 (Table S5). As expected, most of the miRNAs targeted multi genes, and most of the targets were regulated by multi miRNA. Among these target genes, serine and arginine rich splicing factor 3 (SRSF3), and epithelial splicing regulatory protein 2 (ESRP2) were proved to take part in the hepatocyte differentiation, metabolic functional change, as well as postnatal liver maturation in previous studies^3,5^. In our experiment, the SRSF3 could regulated by seven differentially expressed miRNAs (with the criteria of P < 0.05), including cgr-miR-500, gga-miR-1467-3p_L + 1, gga-miR-1668-3p, gga-miR-223_1ss15AG, gga-miR-223_1ss19CT, gga-miR-223_R + 1, and mmu-miR-500-3p_R-1, which suggested their potential regulatory roles in postnatal liver maturation by targeting SRSF3. Meanwhile, the ESRP2 could be regulated by 21 differentially expressed miRNAs (Table S5). Among these 21 miRNAs, the gga-miR-30a and gga-miR-30e were highly expressed and reported to regulate the liver development^14^, which indicated the roles of gga-miR-30a and gga-miR-30e in the regulation of postnatal liver maturation by targeting ESRP2. Specifically, the gga-miR-146a is an only differentially expressed miRNAs with the criteria of FDR < 0.05 by targeting ESRP2, which indicated that miR-146a could influence the postnatal liver maturation process by targeting ESRP2 genes^3^. Moreover, gga-miR-122 was the identified differentially expressed miRNAs which proved by miRNAs sequencing with the criteria of P value < 0.05 and the qRT-PCR validation. Several previous reported target genes of gga-miR-122 involved in cytokinesis and further liver development have also been detected in the present study, including microtubule affinity-regulating kinase 3 (MARK3), mitogen-activated protein kinase kinase kinase 4 (MAP3K4), RAD21 cohesin complex component (RAD21), transforming growth factor beta 3 (TGFβ3), neural precursor cell expressed, developmentally down-regulated 4-like, E3 ubiquitin protein ligase (NEDD4L), and microtubule-associated protein, RP/EB family, member 1 (MAPRE1)^13^, which again confirmed the roles of miR-122 in postnatal liver maturation. Meanwhile, these target genes could also be regulated by other differentially expressed miRNAs (Table S5). Those previous results demonstrated the high reliability of target identification in the present research.

### Functional annotation of differentially expressed miRNAs

To gain a better understanding of the functional roles of these differentially expressed miRNA, further Gene Ontology (GO) and Kyoto Encyclopedia of Genes and Genomes (KEGG) analyses were performed based on those target genes of differentially expressed miRNAs. Based on the target genes of differentially expressed miRNAs which selected by the criteria of P value < 0.05, there were 148, 1,067 and 948 target genes of differentially expressed miRNAs mapped to several significantly enriched GO terms (P < 0.05) for 17 biological process, 15 cell component topology, and 26 molecular function, respectively (Table S6). Meanwhile, KEGG pathway annotation showed 1,576 target genes that were annotated for 23 KEGG pathways with P < 0.05 (Table S7). Similarly, based on the differentially expressed miRNAs which selected by the criteria of FDR value <0.05, further GO and KEGG analyses (with P < 0.05) have identified 43 significantly enriched GO terms (Table S8) and 15 significantly enriched KEGG pathways (Table S9).

As the most important metabolic organ, the main function of liver takes part in a series of metabolism pathways, which are directly corrected with postnatal liver maturation^7^. Combined with the results of GO and KEGG analysis (Tables S6, S7, S8, and S9), we firstly focused on the functional changes between the postnatal and matured livers and found that differentially expressed miRNAs mainly regulated many metabolic changes in liver, including metabolic process (GO: 0008152), nucleotide metabolic process (GO: 0009117), nitrogen compound metabolic process(GO:0006807), fatty acid metabolism (ko00071), Fatty acid elongation(ko00062), arginine and proline metabolisms (ko00330), valine, leucine and isoleucine degradation (ko00280), pyruvate metabolism (ko00620), glycolysis/gluconeogenesis (ko00010), protein processing in endoplasmic reticulum (ko04141), ribosome biogenesis in eukaryotes(ko03008), mammalian Target of Rapamycin (mTOR) signaling pathway(ko04150), propanoate metabolism (ko00640), Peroxisome proliferator activated receptor (PPAR) signaling pathway (ko03320), glutathione metabolism (ko00480), and cysteine and methionine metabolism (ko00270). Considering the results obtained from metabolomic researches, we could ensure that many metabolic changes were intensively remodeled during postnatal liver maturation to rapidly adapt and perform adult functions^3^, and those metabolic functions changes could be regulated by these differentially expressed miRNAs.

Meanwhile, the putative target genes of differentially expressed miRNAs (screened out with P < 0.05 or FDR < 0.05) have both enriched natural killer cell (NK cell) mediated cytotoxicity pathway (Tables S7 and S9), which is an important change in immune function along with the postnatal liver development. Recent studies have indicated that some special cells in liver contribute to the foundation of hepatic innate immunity^26–28^, including the NK cells. NK cells are innate lymphoid cells widely renowned for their roles in eliminating transformed and virus-infected cells^29^, and NK cell mediated cytotoxicity is the mark of NK cell maturation and is crucial for liver function of anti-inflammatory and anti-tumor^28,30^. The discovery of NK cell mediated cytotoxicity in our experiment represented the improvement of immune function in mature liver, and the relevant differentially expressed miRNAs could contribute to the regulation of NK cell mediated cytotoxicity in liver. Moreover, several epigenetic pathways, such as protein polyubiquitination^31^, histone deacetylation^32^, and so on, could be regulated by the differentially expressed miRNAs and involved in the regulation of liver development, which suggested that those differentially expressed miRNAs may regulate liver maturation by changing other epigenetic modifications.

**Figure 4:**
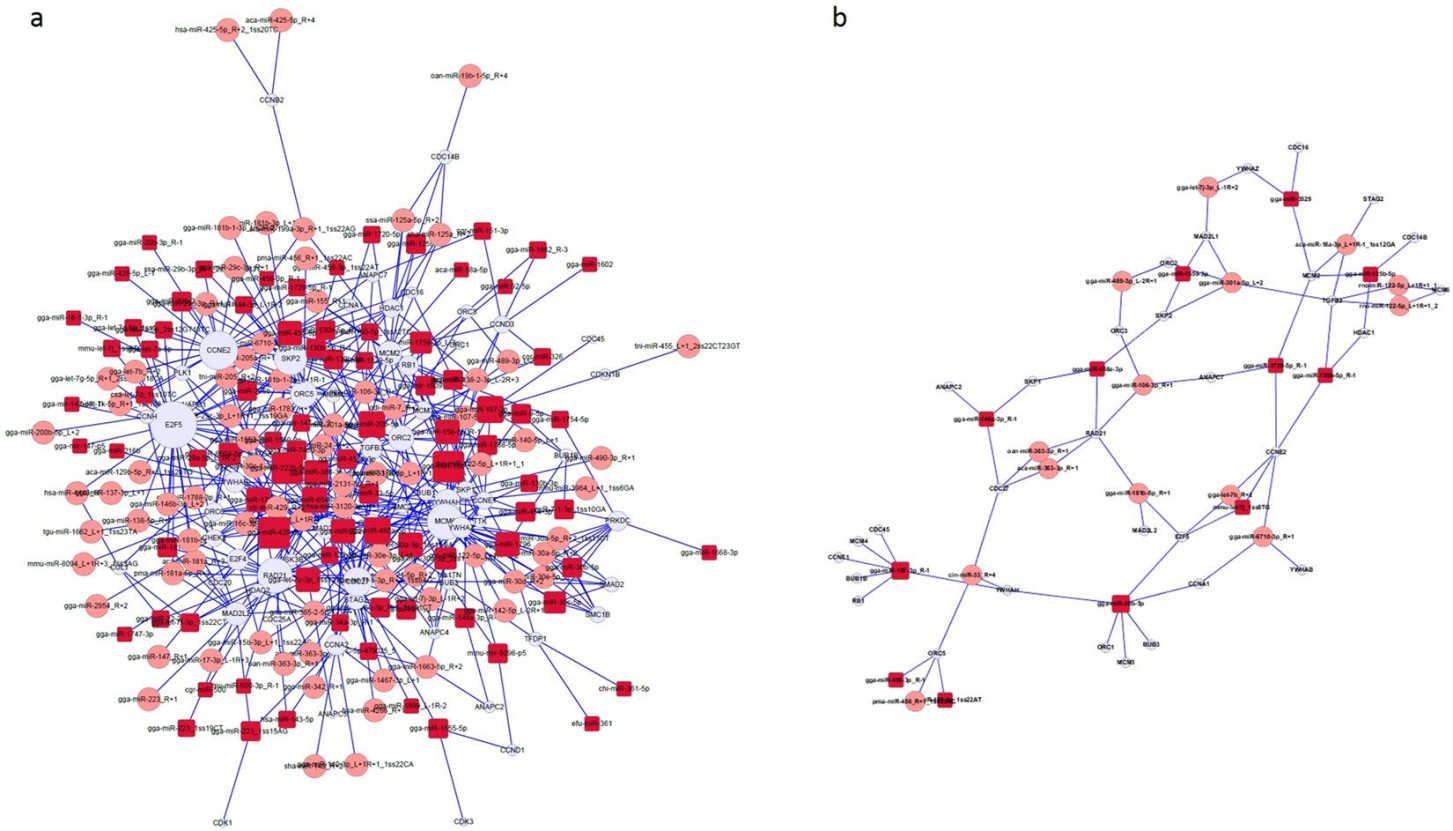
The miRNAs-genes network of cell cycle pathway (ko04110). Note: 1. (**a**) the miRNAs-genes network of cell cycle pathway (ko04110) constructed based on differentially expressed miRNAs (screened out with the criteria of P < 0.05 and fold changes either >1.5 or <0.67) and their targets; (**b**) the miRNAs-genes network of cell cycle pathway (ko04110) constructed based on differentially expressed miRNAs (screened out with the criteria of FDR < 0.05 and fold changes either >1.5 or <0.67) and their targets. 2. White circular nodes represent potential target genes which were involved in the cell cycle pathway, and red rectangular and pink rounded rectangle nodes represent identified differentially expressed miRNAs which were involved in the cell cycle pathway. The size of the nodes represents the power of the interrelation among the nodes, and edges between two nodes represent interactions between genes. The more edges a gene has, the more miRNAs that interact with it, and the more central a role it had within the network.

Additionally, several differentially expressed miRNAs were enriched in cell cycle (ko04110), focal adhesion (ko04510), cytokinesis (GO: 0000910), and positive regulation of cell growth (GO: 0030307), which could influence cell division and growth of liver and further influence the liver maturation process. Previous studies have proved that liver development requires the cell cycle regulator, such as RAD21, TGFβ3, ubiquitin-like with PHD and ring finger domains 1 (UHRF1), and cyclin-dependent kinase (CDK)^13,33,34^, which could also been detected in our study and indicated that the cell cycle control is crucial for the liver maturation^35^. In order to clarify the relationship between differentially expressed miRNAs and their potential target genes involved in the regulation of cell cycle pathway in chicken liver, the miRNAs-genes interaction network have been built (Fig. 4). According to the miRNAs-genes network, several differentially expressed miRNAs including miR-122 and their potential target genes were involved in the regulation of cell cycle^13^, which indicated that those differentially expressed miRNAs in our study could regulate the postnatal liver development by influencing the cell cycle pathway. Moreover, miR-122 and its target genes, which were related to cytokinesis, have been indicted to regulate the liver cell polyploidization^13^. These results indicated that the liver cell polyploidization and cytokinesis process were crucial for formation of binuclear hepatocytes and further liver development process^36^. Meanwhile, positive regulation of cell growth and focal adhesion processes have also been linked to the regulation of liver maturation^37,38^.

### The gga-miR-122–5p targets the 3′ Untranslated Regions (3′UTRs) of TGFβ3 or RAD21

**Figure 5:**
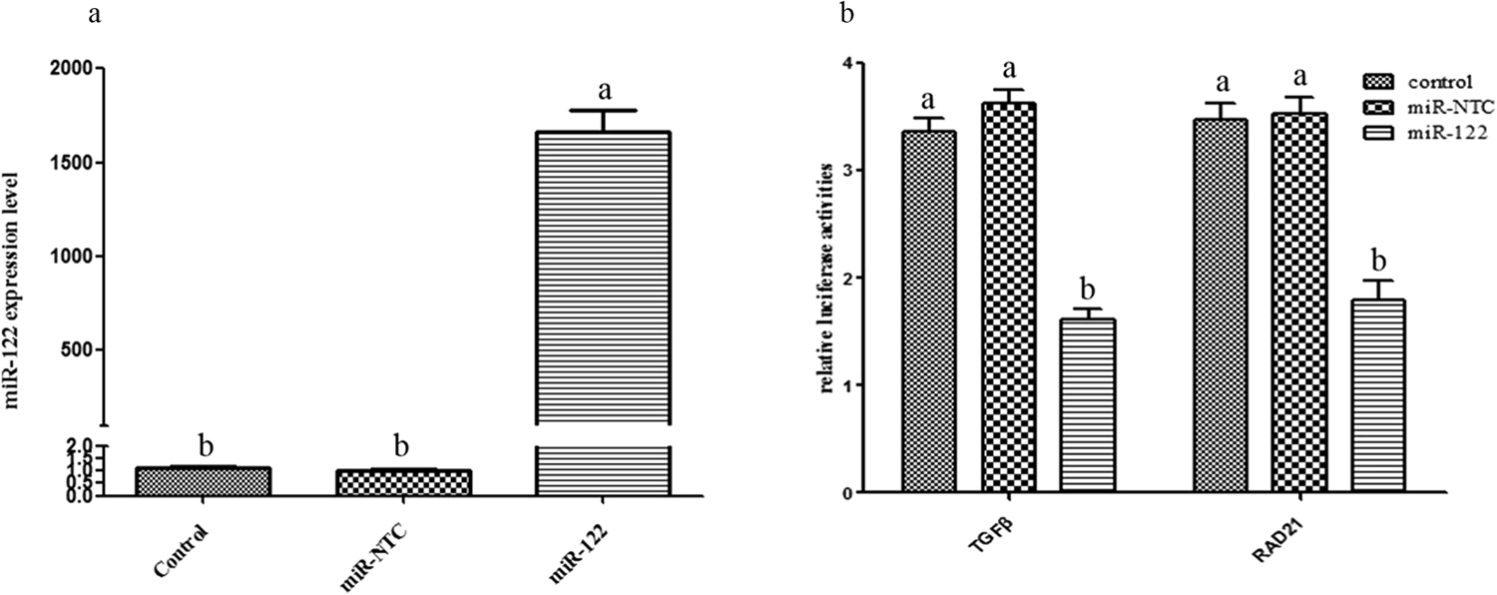
MiR-122–5p mimic or negative control (miR-NTC) and luciferase reporter genes with TGFβ3 or RAD21 3′UTRs in psiCHECK^TM^-2 vectors were co-transfected into HEK293T cells. Note: (**a**) The overexpression of miR-122–5p was detected at 48 h in miR-122-5p group after transfection. (**b**) Renilla luciferase activity was normalized to firefly luciferase. All measurements shown are the means ± SE (n = 4). Superscripts (**a**,**b**) denote significantly different expression levels in the same microRNAs (P < 0.05).

The gga-miR-122-5p was the highest differentially expressed miRNAs in the present study, and TGFβ3 or RAD21 were the targets of gga-miR-122-5p involved in the cell cycle pathway. In order to further validate the interaction of gga-miR-122-5p with TGFβ3 or RAD21 *in vitro*, we constructed luciferase reporter genes with TGFβ3 or RAD21 3′UTRs in psiCHECK^TM^-2 vectors respectively and then co-transfected the vectors and gga-miR-122-5p mimic or miR-NTC into HEK293T cells respectively. The significant changes of gga-miR-122-5p expression levels in the gga-miR-122-5p groups showed that our co-transfections were successful (Fig. 5a). Furthermore, compared with the control and miR-NTC groups, luciferase reporter assays manifested a significant decrease in luciferase activity (*P* < 0.05) when TGFβ3 or RAD21 3′UTR were co-transfected with gga-miR-122-5p mimic respectively (Fig. 5b). These results demonstrated that TGFβ3 or RAD21 were targeted by gga-miR-122-5p and the target predictions of miRNAs were accurate.

### Identification of the potential way of differentially expressed miRNAs to regulate the target genes expression

Previous studies suggested that miRNAs may affect their target genes expression by repressing target translation or triggering target degradation^39,40^. In order to validate the roles of differentially expressed miRNAs in affecting their target genes expression, we performed mRNA sequencing by using the same samples for miRNA sequencing. As results, 1211 differentially expressed mRNAs between the postnatal and matured livers met the criteria of FDR values < 0.05 and |log2foldchenge| > 1 (Table S10); of these, 669 mRNAs were up-regulated whereas 542 down-regulated in the matured livers relative to the postnatal livers.

Then we screened out the mutual genes between those identified differentially expressed mRNAs and the target genes of our identified differentially expressed miRNAs. Among our identified 5,074 target genes of the 191 differentially expressed miRNAs, 579 genes were found to be differentially expressed genes by mRNA sequencing analysis and were designated as “mutual mRNAs” (Table S11). Furthermore, as a result of triggering target mRNAs degradation, a total of 188 differentially expressed miRNAs could regulate the expression levels of the 422 differentially expressed mRNAs in the present study (Table S12). Similarly, the mutual genes between those identified differentially expressed mRNAs and the target genes of our identified 32 differentially expressed miRNAs (FDR < 0.05) were also obtained. Among our identified 2,837 target genes of the 32 differentially expressed miRNAs, 297 genes were found to be differentially expressed genes by mRNA sequencing analysis and were also designated as “intersection mRNAs” (Table S13). Furthermore, as a result of triggering target mRNAs degradation, all 32 differentially expressed miRNAs could regulate the expression levels of the 190 differentially expressed mRNAs in the present study (Table S14), which proved that those differentially expressed miRNAs could regulate expression levels of their target mRNAs at mRNA levels^39^. As for many other differentially expressed miRNAs, they could regulate their potential targets genes expressions by repressing target translation^40^. To sum up, these results provided evidence that miRNAs have a potential to regulate their target gene expression at both post-transcriptional and translation levels.

Among those “intersection mRNAs”, several genes related to the liver development, such as RAD21^13^, sorting nexin^41^, von Willebrand factor^42^, as well as cytochrome P450 gene isoforms^43^, could be regulated by the differentially expressed miRNAs (including those miRNAs selected both with the criteria of P < 0.05 and the criteria of FDR < 0.05; Tables S12 and S14) at mRNA levels, meanwhile, the cyclin-dependent kinase 1 (CDK1)^33^, which was also proved to be involved in the regulation of liver development, also could be regulated by the differentially expressed miRNAs which selected with the criteria of P < 0.05 (Table S12). Some other previous reported genes involved in postnatal liver maturation, such as TGFβ3, SRSF3, ESRP2, NEDD4L, MAPRE1, and UHRF1, could be regulated by our identified differentially expressed miRNAs by influencing translation process. Additionally, combined with the results of luciferase reporter assay, the miR-122 could regulate targets expression by repressing target translation or triggering target degradation, For example, the expression of RAD21 might be regulated in triggering target mRNA degradation and TGFβ3 might be regulated in repressing target translation, which indicated the miR-122 has a potential roles in regulating postnatal liver maturation process.

### Roles of differentially expressed miRNAs in regulating postnatal hepatic metabolic changes

The hepatic function of chickens is intensively remodeled from birth to adult, specifically, livers underwent large-scale changes in metabolic functions after birth^3^. In order to reveal the roles of differentially expressed miRNAs and their targets which were associated with the postnatal hepatic metabolic functional changes, an association study based on the pathway analyses of differentially expressed miRNAs and metabolites were performed. As results, we can found that our identified changed metabolic pathways, including TCA cycle, glycolysis/gluconeogenesis, the biosynthesis or degradation of valine/leucine/isoleucine, arginine and proline metabolism, glutathione metabolism, taurine and hypotaurine metabolism, cysteine and methionine metabolism, steroid biosynthesis, and fatty acid metabolic process, could be all regulated by the identified differential expression of miRNAs and their intersection targets mRNAs (Table 3). This study have identified 30 intersection genes, of which 9 are involved in glycometabolism pathways, 16 are involved in different amino acids metabolism pathways, and 9 are involved in lipid metabolism pathways. These intersection genes were the key metabolic enzymic genes involved in differential metabolic pathways, which could further induce the changes of the metabolites in different metabolic process. Meanwhile, we can found that these intersection target mRNAs could be regulated by the differentially expressed genes in the present study, which indicated that our identified differentially expressed miRNAs could really regulate the remodeled hepatic function of chickens from birth to adult.

**Table 3:** Differentially expressed miRNAs and their transcriptional dependent target mRNAs which were associated with the postnatal hepatic metabolic functional changes.

Item	Metabolic Pathway	Differentially expressed metabolites	Related pathway based on differentially expressed miRNAs	Differentially expressed miRNAs^1^	Intersection targets mRNAs
glycometabolism	Citrate cycle (TCA cycle)	fumaric acid, 2-ketoglutaric acid, succinic acid, malic acid, pyruvic acid	Citrate cycle (TCA cycle) or Pyruvate metabolism	gga-let-7b_L-1R-1/ola-miR-126-5p_R + 1_1ss1CN	ALDH3A2
				gga-let-7c-3p_1ss12TC/gga-miR-106-3p_R + 1(FDR < 0.05)/gga-miR-130b-3p/gga-miR-1467-3p_L + 1/gga-miR-17-3p_L-1R + 3/gga-miR-20b-5p (FDR < 0.05)/gga-miR-454-3p	LDHA
				hsa-miR-3120-3p_R + 1	PDHA1
				gga-miR-1329-5p/gga-miR-9-5p/rno-miR-122-5p_L + 1 R + 1_1 (FDR < 0.05)/rno-miR-122-5p_L + 1 R + 1_2 (FDR < 0.05)	SUCLG1
				aca-miR-363-3p_R + 1	ACO2
	Glycolysis/Gluconeogenesis	pyruvic acid, 2-phosphoglyceric acid, 3-phosphoglyceric acid, glucose 6-phosphate, fructose 6-phosphate, dihydroxyacetone phosphate	Glycolysis/Gluconeogenesis	gga-let-7b_L-1R-1/ola-miR-126-5p_R + 1_1ss1CN	ALDH3A2
				cin-miR-33_R + 4 (FDR < 0.05)/gga-miR-214_L + 1R-3_1ss19GA/gga-miR-22-3p/gga-miR-29b-3p_R-1/gga-miR-29c-3p_R + 1/gga-miR-29c-3p_R + 1_1ss18GA/gga-miR-33-5p/hsa-miR-3120-3p_R + 1/ola-miR-199a-3p_R + 1_1ss22AG/ssa-miR-29b-3p_R-1_2ss20TC21TC	BPGM
				gga-let-7c-3p_1ss12TC/gga-miR-106-3p_R + 1 (FDR < 0.05)/gga-miR-130b-3p/gga-miR-1467-3p_L + 1/gga-miR-17-3p_L-1R + 3/gga-miR-20b-5p (FDR < 0.05)/gga-miR-454-3p	LDHA
				hsa-miR-3120-3p_R + 1	PDHA1
				gga-mir-147-p3_2	PFKL
				gga-miR-22-3p	TPI1
amino acid or protein metabolism	Valine, leucine and isoleucine biosynthesis/degradation	Isoleucine, leucine, valine, 2-ketoisocaproic acid	Valine, leucine and isoleucine degradation	aca-miR-18a-3p_L + 1R-1_1ss12GA (FDR < 0.05)/csa-let-7d_1ss10TC/gga-miR-130b-3p/gga-miR-1699_L-1R-2/gga-miR-1788-3p_R + 1/gga-miR-1788-5p/gga-miR-216b/gga-miR-301a-5p_L + 2 (FDR < 0.05)/gga-miR-3529 (FDR < 0.05)/gga-miR-383-3p/gga-miR-454-3p/gga-miR-460a-5p/gga-miR-6548-5p/mmu-mir-5098-p5/oha-miR-7-1-3p_1ss10GA	ACADSB
				gga-let-7b_L-1R-1/ola-miR-126-5p_R + 1_1ss1CN	ALDH3A2
				gga-miR-142-5p_L-2R + 1/mmu-miR-3964_L + 1_1ss6GA	HIBADH
				gga-miR-1731-5p/gga-miR-1759-3p_L-3/gga-miR-1788-3p_R + 1/gga-miR-6548-5p	HMGCL
	Arginine and proline metabolism	4-hydroxyproline, 5-aminopentanoic acid, putrescine, ornithine, glutamic acid, sarcosine	Arginine and proline metabolism	gga-let-7b_L-1R-1/ola-miR-126-5p_R + 1_1ss1CN	ALDH3A2
				ola-miR-199a-3p_R + 1_1ss22AG	ALDH4A1
				gga-let-7a-5p/gga-let-7a-5p_2ss12GT18TC/gga-let-7b_R + 2(FDR < 0.05)/gga-let-7c-5p_1ss17AG/gga-let-7g-5p_R + 1_2ss14TG18CA/gga-let-7k-5p_R + 1_1ss16AT/gga-miR-22-3p/mmu-let-7j_1ss8TG (FDR < 0.05)	GATM
				gga-miR-20b-3p_R-1	GLS
				dre-miR-24_R + 2/gga-miR-9-5p	GOT1
				gga-miR-1747-3p/gga-miR-1796/gga-miR-20b-3p_R-1	P4HA1
				aca-miR-425-5p_R + 4/dre-miR-24_R + 2/gga-miR-200b-5p_L + 2/gga-miR-223_1ss15AG/gga-miR-223_1ss19CT/gga-miR-223_R + 1/hsa-miR-425-5p_R + 2_1ss20TC	SRM
	Glutathione metabolism	Putrescine, dehydroascorbic acid, cadaverine	Glutathione metabolism	gga-miR-17-3p_L-1R + 3/gga-miR-20b-3p_R-1/gga-miR-34a-3p_R-1/gga-miR-6604-5p_1ss7GA/mmu-miR-8094_L + 1 R + 3_1ss3AG	GSTA
				gga-miR-489-3p_L-2R + 1 (FDR < 0.05)	GSTO1
				gga-let-7f-3p_1ss22CT/gga-miR-1329-5p/hsa-miR-3120-3p_R + 1	RRM1
				aca-miR-425-5p_R + 4/dre-miR-24_R + 2/gga-miR-200b-5p_L + 2/gga-miR-223_1ss15AG/gga-miR-223_1ss19CT/gga-miR-223_R + 1/hsa-miR-425-5p_R + 2_1ss20TC	SRM
	Taurine and hypotaurine metabolism/Cysteine and methionine metabolism	Taurine, 2-aminobutyric acid, methionine, cysteine	Cysteine and methionine metabolism	gga-miR-214_L + 1R-3_1ss19GA	CTH
				dre-miR-24_R + 2/gga-miR-9-5p	GOT1
				gga-let-7c-3p_1ss12TC/gga-miR-106-3p_R + 1 (FDR < 0.05)/gga-miR-130b-3p/gga-miR-1467-3p_L + 1/gga-miR-17-3p_L-1R + 3/gga-miR-20b-5p (FDR < 0.05)/gga-miR-454-3p	LDHA
				aca-miR-425-5p_R + 4/dre-miR-24_R + 2/gga-miR-200b-5p_L + 2/gga-miR-223_1ss15AG/gga-miR-223_1ss19CT/gga-miR-223_R + 1/hsa-miR-425-5p_R + 2_1ss20TC	SRM
				aca-miR-18a-3p_L + 1R-1_1ss12GA (FDR < 0.05)/gga-miR-107-5p_R + 1/gga-miR-342_R + 1	TAT
Lipid metabolism	Steroid biosynthesis	Lanosterin, 4α-methylzymosterol, campesterol, desmosterol, dihydrocholesterol, stigmasterol, cholesterol	PPAR signaling pathway	aca-miR-363-3p_R + 1 (FDR < 0.05)/gga-miR-106-3p_R + 1 (FDR < 0.05)/gga-miR-1783_R + 1/gga-miR-181b-5p_R + 1 (FDR < 0.05)/gga-miR-205a_R + 1/gga-miR-205b/gga-miR-460a-5p/ggo-miR-342_R + 1/oan-miR-363-3p_R + 1 (FDR < 0.05)/tni-miR-205_R + 2	ACSBG2
				gga-miR-1662_R-3/tgu-miR-1662_L + 1_1ss23TA	CYP7A1
				gga-miR-1716_L-2	FABP3
				gga-miR-1559-3p (FDR < 0.05)/gga-miR-16-1-3p_R-1/gga-miR-20b-5p (FDR < 0.05)/gga-miR-456-3p_1ss22AT (FDR < 0.05)/gga-miR-456-3p_R-1 (FDR < 0.05)/oan-miR-363-3p_R + 1 (FDR < 0.05)/pma-miR-456_R + 1_1ss22AC (FDR < 0.05)/aca-miR-363-5p(FDR < 0.05)	FABP5
				hsa-miR-3120-3p_R + 1/oan-let-7f-1-3p_R + 2_1ss8AG	FABP7
				cgr-miR-151-3p/gga-miR-1467-3p_L + 1	FADS2
				gga-miR-2954_R + 2/gga-miR-458a-3p(FDR < 0.05)	LPL
	Fatty acid metabolism	arachidonic acid, docosahexaenoic acid, margaric acid, oleic acid, stearic acid, tetradecanoic acid, cis-9-hexadecenoic acid	Fatty acid metabolism	aca-miR-18a-3p_L + 1R-1_1ss12GA (FDR < 0.05)/csa-let-7d_1ss10TC/gga-miR-130b-3p /gga-miR-1699_L-1R-2/gga-miR-1788-3p_R + 1/gga-miR-1788-5p/gga-miR-216b/gga-miR-301a-5p_L + 2 (FDR < 0.05)/gga-miR-3529 (FDR < 0.05)/gga-miR-383-3p/gga-miR-454-3p/gga-miR-460a-5p/gga-miR-6548-5p/mmu-mir-5098-p5/oha-miR-7-1-3p_1ss10GA	ACADSB
				gga-miR-106-3p_R + 1(FDR < 0.05)/gga-miR-1783_R + 1/gga-miR-181b-5p_R + 1(FDR < 0.05)/gga-miR-205a_R + 1/gga-miR-205b/gga-miR-460a-5p/gga-miR-342_R + 1/oan-miR-363-3p_R + 1(FDR < 0.05)/tni-miR-205_R + 2/aca-miR-363-3p_R + 1(FDR < 0.05)	ACSBG2
				gga-let-7b_L-1R-1/ola-miR-126-5p_R + 1_1ss1CN	ALDH3A2

Note: ^1^The FDR < 0.05 followed by those differentially expressed miRNAs represented that those miRNAs were selected based on the criteria of FDR < 0.05 and fold changes either >1.5 or <0.67; other differentially miRNAs without the following label of FDR <0.05 were selected based on the criteria of P < 0.05 and fold changes either >1.5 or <0.67.

In conclusion, based on the comparison of miRNAs expression profiles of the immature and matured livers, 191 differentially expressed miRNAs with the criteria of P < 0.05 and 32 differentially expressed miRNAs with the criteria of FDR < 0.05 were obtained in this study. Furthermore, we also performed functional annotations for their targets separately, suggesting that they were likely to play regulatory roles in postnatal liver maturation and could be a potential regulatory strategy for chicken liver function and metergasis, especially the metabolic functions changes.

## Materials and Methods

### Study design and sample collections

All animals in this study were managed according to the No. 5 Proclamation from the Ministry of Agriculture of China and animal protocols were approved by the Institutional Animal Care and Use Committee of the Northwest A&F University (Yangling, Shaanxi, China). Twenty one-day-old healthy Arbor Acres breeder cocks and five adult healthy Arbor Acres breeder cocks (35 week age) were randomly selected from Experimental Center for Animal Science of the Northwest A&F University. Here, the Arbor Acres breeder cock in 35 week age were both somatic and sexual matured, which ensured their livers were assuredly matured.

These randomly selected chickens were killed and immediately dissected. The left side liver samples were collected into 2 mL Eppendorf tubes, and frozen immediately in liquid nitrogen. All liver samples were stored at −80 °C until being analyzed. The livers of three adult cocks and 12 one-day-old male chicks were randomly selected for next generation sequencing analyses of miRNAs and mRNAs, and all liver samples from five adult cocks and 20 one-day-old male chicks were used for metabolomics study as well as used to extract total RNA and validate the reliability of the sequencing data.

### Metabolite extraction, sample derivatization, and GC-MS analysis

Four livers samples of one-day-old breeder cocks were mixed equally together as a pooled liver sample according to the weights of livers, so five pooled liver samples were created from the total of 20 male chicks. Then, five pooled liver samples from one-day-old male chicks and five directly used liver samples from adult breeder cocks were used for GC-MS analyses. Approximately 50 mg of liver sample were homogenized in 800 μL chloroform/methanol/water solvent (2:5:2). After centrifugation at 13,000 rpm for a min, the supernatant was collected. Then 640 μL ice-cold methanol was added to the residue for another repeated extraction. The supernatants from the two extractions were pooled. Then 100 μL of the solution was taken into a glass vial, 10 μL of the internal standards (containing 0.05 mg/mL of ^13^C_6_-L-leucine and ^13^C_6_-^15^N L-isoleucine) was added, mixed and then dried under gentle nitrogen stream. The dried residuary sample was added with 30 μL of 20 mg/mL methoxyamine hydrochloride in pyridine, vortex-mixed for 30 s and incubated at 37 °C for 90 min. A 30 μL of N,O-bis(trimethylsilyl) trifluroacetamide (BSTFA) with 1% trimethylchlorosilane (TMCS) was added into the mixture and incubated at 70 °C for 60 min.

The derivatives in the sample were analyzed using an Agilent 7890 A gas chromatography system coupled to an Agilent 5975 C Mass-Spring-Damper system (Agilent Technologies, CA, USA). A HP-5ms fused-silica capillary column (30 m × 0.25 mm × 0.25 μm; Agilent J&W Scientific, Folsom, CA) was used to separate the derivatives. Helium (>99.999%) was used as a carrier gas at a constant flow rate of 1 mL/min through the column. Injection volume was 1 μL in splitless mode, and the solvent delay time was set for 6 min. The over temperature program was set as: initial temperature 70 °C for 2 min, increased to 160 °C at a ramp rate of 6 °C/min, then up to 240 °C at a ramp rate of 10 °C/min, and finally to 300 °C at a ramp rate of 20 °C/min and held for 6 min to clean the column. The temperatures of injector, transfer line, and electron impact ion source were set to 250 °C, 260 °C, and 230 °C, respectively. The impact energy was 70 eV. Mass data was acquired in a full scan mode (m/z 50–600).

### Data preprocessing and statistical analysis for GC-MS analysis

The acquired GC-MS data were processed as described in the previous study^44^. For statistical analysis, the normalized data were imported to SIMCA software (version 13.0, Umetrics, Umeå, Sweden). The model quality is described by the R^2^X or R^2^Y and Q^2^ values as described in the previous study^44^. In order to avoid model over-fitting, a default 7-round cross validation in SIMCA-P software was performed throughout to determine the optimal number of principal components. The variables with VIP values of OPLS-DA model larger than 1 and p values of univariate statistical analysis lower than 0.05 were identified as significantly differential metabolites. The fold change was calculated as binary logarithm of average normalized peak intensity ratio between two age groups. Then, the AMDIS software was applied to deconvolute mass spectra from raw GC-MS data. The pathway analyses based on differential metabolites were performed by MetaboAnalyst^44^.

### RNA isolation for RNA sequencing

Total RNA from 3 adult cocks’ and 12 one-day-old male chicks’ liver were extracted using Trizol reagent (Invitrogen, CA, USA). Specifically, the DNaseI was used to avoid contamination with genomic DNA during the RNA isolation process. The purity and quantity of total RNA were analyzed by Nanodrop equipment, and the integrity of RNA was accessed with Bioanalyzer 2100 and RNA Nano6000 LabChip Kit (Agilent, CA, USA). Only when the OD260/280 > 1.8, OD260/230 > 2.0, and the RNA Integrity Number (RIN) > 7.0, RNA samples will be used for further sequencing. Four RNA samples from one-day-old chicks’ liver were mixed equally together as a pooled RNA sample according to the purity and quantity of total RNA, and the RNA samples of individual adult cock’ liver were directly used. In total, we gathered three RNA samples from three individual adult cocks and three pooled RNA samples from 12 one-day-ole male chicks for further library construction.

### Small RNA sequencing and screening of the differentially expressed miRNAs

Approximately 1 μg of total RNA from each prepared RNA samples were used to prepare small RNA libraries respectively by using TruSeq Small RNA Sample Prep™ Kits (Illumina, San Diego, USA). Briefly, the total RNAs were purified by polyacrylamide gel electrophoresis to enrich 15 to 35 nt molecules, and then proprietary adapters were ligated to the 5′ and 3′ termini of the RNA using T4 RNA ligase, and the samples were used as templates for complementary DNA (cDNA) synthesis. Sequencing libraries were produced by amplifying the cDNA using polymerase chain reaction (PCR). The PCR products were purified on 6% polyacrylamide Tris-borate-EDTA gels.

Then the single-end sequencing (1*50 bp) were performed on an Illumina Hiseq. 2500 at the LC-BIO (Hangzhou, China) and our sequencing data (for each sequencing sample, the sequencing data >10 M) were processed according to the procedures described in a previous study^45^. Moreover, raw sequencing reads were filtered to obtain mappable sequences using ACGT101-4.2 (LC Sciences, Hangzhou, China)^46^. Briefly, the raw reads were firstly processed through FastQC to obtain the clean data, by removing the reads that contain 3′ adaptor (3′ ADT) sequence or poly-N, the low quality reads whose Q value were less than 20, the reads smaller than 18 nt or longer than 26 nt, and the redundancy reads. At the same time, Q20 and Q30 of the clean data were calculated (Table S15). The clean reads were mapped to the genomes of gallus gallus (Gallus gallus 4 Ensembl 85; http://www.ensembl.org/Gallus_gallus/Info/Index) and other selected species (with the exclusion of specific species) to analyze their expression and distribution^47^. The matched sequences were blasted against the NCBI Rfam database (http://rfam.janelia.org) and GenBank database (http://blast.ncbi.nlm.nih.gov/) to identify and remove the rRNA, tRNA, snRNA, scRNA, srpRNA, and snoRNA sequences.

Subsequently, unique sequences with lengths from 18 to 26 nt were mapped to specific species precursors in miRBase21.0 (ftp://mirbase.org/pub/mirbase/CURRENT/) and the genomes of gallus gallus (Gallus gallus 4 Ensembl 85; http://www.ensembl.org/Gallus_gallus/Info/Index) or other selected species (with the exclusion of specific species) by using Bowtie v2.0.6^47^ to identify known miRNAs and novel 3p- and 5p- derived miRNAs. Length variations at both 3 ft. and 5 ft. ends and one mismatch inside the sequence were allowed in the alignment. The unique sequences mapping to specific species mature miRNAs in hairpin arms were identified as known miRNAs. The unique sequences mapping to the other arm of known specific species precursor hairpin opposite the annotated mature miRNA-containing arm were considered to be novel 5p- or 3p- derived miRNA candidates. The remaining sequences were mapped to other selected species precursors (with the exclusion of specific species) in miRBase 21.0 by Bowtie v2.0.6 and mapped pre-miRNAs were further BLASTed against the specific species genomes to determine their genomic locations. We defined the above two as known miRNAs. The unmapped sequences were BLASTed against the specific genomes and the hairpin RNA structures containing sequences were predicated from the flank at 80nt sequences using RNAfold software (http://rna.tbi.univie.ac.at/cgi-bin/RNAfold.cgi/). Specifically, the identification and classification of miRNAs were obeying the following principles: (1) gp1a: Reads map to Gallus gallus pre-miRNAs in miRbase and the pre-miRNAs further map to the genome & EST. (2) gp1b: Reads map to other vertebrata pre-miRNAs in miRbase we selected and the pre-miRNAs further map to the genome & EST. (3) gp2: Reads map to all vertebrata pre-miRNAs we selected in miRbase. The mapped pre-miRNAs do not map to the genome; however, the reads (and of course the miRNAs) map to genome. The extended genome sequences from the genome loci may form hairpins. (4) gp3: Reads map to all vertebrata pre-miRNAs in miRbase we selected. Both the mapped pre-miRNAs and the reads do not map to the genome. (5) gp4: Reads do not map to all of the vertebrata pre-miRNAs in miRbase we selected; However, the reads map to genome & the extended genome sequences from genome may form hairpins. Here, the identified miRNAs in gp1, gp2, and gp3 were known miRNAs and the identified miRNAs in gp4 were novel miRNAs.

Modified reads per million reads was used to quantify the normalized reads^46^. The differential expression of miRNAs based on normalized counts was analyzed using Student’s t test and the significance threshold was set as *P* < 0.05 and the fold-change >1.5 or <0.67. Furthermore, in order to reduce the false positives of our detected differentially expressed miRNAs, FDR was further used to screen out the differentially expressed miRNAs. Specifically, a Fold Change cut-off of >1.5 or <0.67, representing the size of the change and a p-value with FDR cutoff of <=0.05 representing the significance of the change was used to get the differentially expressed miRNAs. The fold-change, *P* value and the FDR value for each differentially expressed miRNA was calculated by using the formula as previously described^48^.

### Target prediction of differentially expressed miRNAs and further functional analyses

To predict the genes targeted by differentially expressed miRNAs, TargetScan 50 and Miranda 3.3a were used to identify miRNA binding sites. The overlaps predicted by both algorithms were used as predicted target genes. Functional annotation of predicted miRNA targets were performed based on GO database and enriched pathways were analyzed using KEGG database. GO enrichment analysis for the screened differentially expressed mRNAs was carried out using GOseq platform^49^. The KEGG pathway enrichment analysis for the differentially expressed mRNAs was performed by using KOBAS software^50^. In these two analyses, *P* < 0.05 were defined as significantly enriched GO terms or KEGG pathways. Additionally, the miRNA-pathway interaction network was constructed by Cytoscape v2.8.3 software (http://www.cytoscape.org/).

### Sequencing for mRNA and screening of differentially expressed mRNAs

Approximately 3 μg of total RNA was used to prepare an mRNA library. According to protocol of Epicentre Ribo-zero™ Gold Kit (Illumine, San Diego, USA), ribosomal RNA was removed and the rRNA-depleted RNA (Poly A^+^ and Poly A^−^ RNA) were collected^51,52^. Subsequently, high strand-specificity libraries were generated using the rRNA-depleted RNA and a NEBNext Ultra Directional RNA Library Prep Kit for Illumina (NEB, Ipswich, MA, USA) following the manufacturer’s recommendations. Briefly, the rRNA-depleted RNA was fragmented using divalent cations under elevated temperature in NEBNext. First-strand cDNA was synthesized using random hexamer primers and M-MuLV reverse transcriptase (RNase H^−^). Subsequently, second-strand cDNA synthesis was performed using second-strand synthesis reaction buffer, DNA polymerase I, and RNase H. Remaining overhangs were converted into blunt ends by exonuclease/polymerase activity. After adenylation of the 3′ ends of the DNA fragments, NEBNext adaptors with hairpin loop structures were ligated to the fragments to prepare them for hybridization. To select cDNA fragments that are 150–200 bp in length, the fragments in each of the library were purified with an AMPure XP system (Beckman Coulter, Brea, CA, USA). Then 3 μl USER Enzyme (NEB, Ipswich, MA, USA) was used with size-selected, adaptor-ligated cDNA at 37 °C for 15 min followed by 5 min at 95 °C before PCR. The qPCRs were performed with Phusion High-Fidelity DNA polymerase, Universal PCR primers, and Index (X) Primer. The PCR products were purified (AMPure XP system) and library quality was assessed on an Agilent Bioanalyzer 2100 system. Clustering of the index-coded samples was performed on a cBot Cluster Generation System using a TruSeq PE Cluster Kit v3-cBot-HS (Illumina, San Diego, CA, USA) according to the manufacturer’s instructions. After cluster generation, the paired-end sequencing (2*125 bp) were performed (for each sequencing sample, the sequencing data >10 G) on an Illumina Hiseq. 2500 at the LC-BIO (Hangzhou, China).

The 125 bp paired-end raw reads were firstly processed through FastQC to obtain the clean data, by removing the reads that contain sequencing adapter contaminations or poly-N and the low quality reads whose Q value were less than 20. At the same time, Q20, Q30 and GC content of the clean data were calculated (Table S15). Index of the reference genome was built using Bowtie v2.0.6^47^ and the clean reads from six cDNA libraries were merged and mapped to the Gallus gallus 4 (Ensembl 85; http://www.ensembl.org/Gallus_gallus/Info/Index) using TopHat^53^. The mapped reads were assembled into transcripts using Cufflinks^54^. Briefly, ribosomal RNA in sequencing data was removed. We aligned all reads to ribosomal RNA of chicken download from ensemble by bowtie2, then those aligned reads were removed from fastq files.

Expression levels of all mRNAs were quantified as FPKM using the Cuffdiff program from the Cufflinks package^54^. Based on Negative binomial distribution, differential gene expression was determined using DESeq with a *FDR* value < 0.05 and log_2_foldchange >1 or <−1^55^.

### Integrated analysis of differentially expressed microRNAs and mRNAs

In order to test the roles of differentially expressed miRNAs in gene expression regulation, we firstly screened out the mutual genes between our identified differentially expressed mRNAs and the predicted target genes of our identified differentially expressed miRNAs and defined these genes as the “intersection mRNAs”, including positive and negative relationships between mRNA and miRNA, by using the Venn diagrams analysis. Then, we further integrated the expression trends of the differentially expressed miRNAs with the expression trends of the differentially expressed mRNAs and selected out the transcriptional dependent target mRNAs. Briefly, according to the expression change trends of our identified mutual genes between the differentially expressed mRNAs and the target genes of differentially expressed miRNAs, we defined a mutual gene as the transcriptional dependent target mRNAs if the change trends of its expression levels was contrary with the change trends of the expression levels of our identified differentially expressed miRNAs. For example, of all miRNAs up-regulated in mature livers, the down-regulated expressed target mutual genes we named the relevance target genes; contrapose down-regulated miRNAs in mature livers, the target mutual genes which up-regulated were referred to as the related target genes^56^.

### Real-time Quantitative PCR

We selected 8 miRNAs (6 known miRNAs and 2 novel miRNAs) represent different expression levels for further stem-loop RT-qPCR. Briefly, 2 of these selected miRNAs were differentially expressed miRNAs based on the criteria of FDR < 0.05 and fold changes either >1.5 or <0.67, another 6 miRNAs were selected based on the criteria of P < 0.05 and fold changes either >1.5 or <0.67. Total RNAs from 5 adult cocks’ and 20 one-day-old male chicks’ liver were extracted using Trizol reagent (Takara, Dalian, China). About 500 ng of total RNA was reverse transcribed using Bulge-Loop™ miRNA miR-Reverse Primer Set (RIBO Bio, Guangzhou, China). The miRNA quantification was performed with iCycler IQ^TM^5 (Bio-Rad Laboratories, Hercules, CA, USA) using SYBR Advantage qPCR Premix (Takara, Dalian, China). The U6, 5S, and 18S were used as the internal normalization control^57^. The specific primers were all designed and provided by RIBO Bio Co., Ltd (Guangzhou, China). All data were analyzed using the 2^−ΔΔCt^ method^58^ and statistical analyzed by Student’s t test using SPSS 20.0 statistical software. All data were expressed as means with standard error (SE). Differences were considered to be statistically significant at *P* < 0.05.

### 3′UTR luciferase reporter assays

The 3′UTR of transforming growth factor beta 3(TGFβ3) or RAD21 cohesin complex component (RAD21) mRNA, containing the binding site of gga-miR-122-5p, were amplified from total RNA extracted from breeder cocks’ livers by RT-PCR and further PCR with primers shown in Table S16. All constructs were further confirmed by sequencing. The fragments were cloned into psiCHECKTM-2 Vectors (Promega, Madison, WI, USA) at the 3′-end of the Renilla gene using XhoI and NotI restriction sites.

Luciferase reporter experiments were performed in HEK-293T cells. More than 2.0 × 10^4^ HEK293T cells were seeded in 24-well plates and cultured in high glucose Dulbecco’s modified Eagle’s medium (DMEM) with 10% fetal bovine serum. 24 hours later, 500 ng of vector constructs were co-transfected with either 50 nM of gga-miR-122-5p mimic or miR-NTC (RIBO Bio, Guangzhou, China) using 2.0 μL X-tremeGENE siRNA Transfection Reagent (Roche, USA), and the cells in control group were without any treatment. The expression level of gga-miR-122-5p from cells in four groups were measured by qRT-PCR; the methods and data process were described as before. Luciferase activity was measured at 48 h after transfection by the DualGlo Luciferase Assay System (Promega). Renill luciferase activity was measured and normalized to corresponding firefly luciferase activity.

### Data Availability

The authors confirm that all data underlying the findings are fully available without restriction.

## Electronic supplementary material

Supplementary Information
