# Supplementary material for: Systematic analysis of the regulatory roles of microRNAs in postnatal maturation and metergasis of liver of breeder cocks
Source: Sci Rep. 2018 Jan 8;8:61. doi: 10.1038/s41598-017-18674-3 (PMC5758705; doi:10.1038/s41598-017-18674-3)

Title: Systematic analysis of the regulatory roles of microRNAs in postnatal maturation and metergasis of liver of breeder cocks

Authors: Shengru Wu*, Wei Guo*, Saisai Liang*, Hong Lu*, Wenqiang Sun*, Xiaochun Ren*[†], Qingzhu Sun *, and Xiaojun Yang*

Supplementary Information

Table S1. Overview of reads from raw data to cleaned sequences in livers from chicken. Notes: (1) ADT & length filter: reads without 3' Adaptor and with length of <18nt or >26nt were removed (for animals). (2) Junk reads: the reads contained more than 2 discontinued or 7 consecutive adenine or 8 consecutive cytosine or 6 consecutive guanylic acid or 7 consecutive thymine or 10 dimer repeats or 6 trimer repeats or 5 tetramer repeats will be regarded as junk reads and further filtered out. (3) Rfam: Collection of many common non-coding RNA families except micro RNA (http://rfam.janelia.org), including rRNA, snoRNA, snRNA, tRNA. (4) Repeats: Prototypic sequences representing repetitive DNA from different eukaryotic species (http://www.girinst.org/repbase). There are redundant sequences between mRNA, Rfam and Repeats.

Table S2. Summary of known and predicted miRNA. Notes: (1) gp1a: Reads map to specific miRNAs/pre-miRNAs in miRbase and the pre-miRNAs further map to the genome & EST, (2) gp1b: Reads map to selected (except for specific) miRNAs/pre-miRNAs in miRbase and the pre-miRNAs further map to the genome & EST., (3) gp2: Reads map to selected miRNAs/pre-miRNAs in miRbase. The mapped pre-miRNAs do not map to the genome; however, the reads (and of course the

miRNAs of the pre-miRNAs) map to genome. The extended genome sequences from the genome loci may form hairpins, (4) gp3: Reads map to selected miRNA/pre-miRNAs in miRbase. The mapped pre-miRNAs do not map to the genome and the reads do not map to the genome, (5) gp4: Reads do not map to selected pre-miRNAs in miRbase. However, the reads map to genome & the extended genome sequences from genome may form hairpins.

Table S3. Summary of all expressed known miRNAs of chicken livers in this study.

Table S4. Summary of all expressed novel miRNAs in the livers of chicken.

Table S5. Target genes of the 191 differentially expressed miRNAs related to postnatal liver maturation which were selected with the criteria of P < 0.05 and fold changes either > 1.5 or < 0.67 and the target genes of the 32 differentially expressed miRNAs which were selected with the criteria of FDR < 0.05 and fold changes either > 1.5 or < 0.67.

Table S6. The significantly enriched GO terms (P < 0.05) of predicted targets of differentially expressed miRNAs which selected with the criteria of P < 0.05 and fold changes either > 1.5 or < 0.67. Note: S gene number: the number of significant differentially expressed target genes which match to a GO term; TS gene number: the number of significant differentially expressed target genes; B gene number: the number of target genes which match to a GO term; TB gene number: the number of all target genes

Table S7. The significantly enriched KEGG pathways (P < 0.05) of predicted

targets of differentially expressed miRNAs which selected with the criteria of *P* < 0.05 and fold changes either > 1.5 or < 0.67. Note: S gene number: the number of significant differentially expressed target genes which match to a KEGG term; TS gene number: the number of significant differentially expressed target genes; B gene number: the number of target genes which match to a KEGG term; TB gene number: the number of all target genes.

Table S8. The significantly enriched GO terms (P < 0.05) of predicted targets of differentially expressed miRNAs which selected with the criteria of FDR < 0.05 and fold changes either > 1.5 or < 0.67. Note: S gene number: the number of significant differentially expressed target genes which match to a GO term; TS gene number: the number of significant differentially expressed target genes; B gene number: the number of target genes which match to a GO term; TB gene number: the number of all target genes.

Table S9. The significantly enriched KEGG pathways (P < 0.05) of predicted targets of differentially expressed miRNAs which selected with the criteria of FDR < 0.05 and fold changes either > 1.5 or < 0.67. Note: S gene number: the number of significant differentially expressed target genes which match to a KEGG term; TS gene number: the number of significant differentially expressed target genes; B gene number: the number of target genes which match to a KEGG term; TB gene number: the number of all target genes.

Table S10. Summary of differentially expressed mRNAs between young chicks group (immature livers) and adult chickens group (mature livers).

Table S11. Mutual genes between the differentially expressed mRNAs and the target genes of 191 differentially expressed miRNAs which were selected with the criteria of P < 0.05 and fold changes either > 1.5 or < 0.67.

Table S12. Intersection target differentially expressed genes of 191 differentially expressed miRNAs (which were selected with the criteria of P < 0.05 and fold changes either > 1.5 or < 0.67.) in mRNA levels.

Table S13. Mutual genes between the differentially expressed mRNAs and the target genes of 32 differentially expressed miRNAs which were selected with the criteria of FDR < 0.05 and fold changes either > 1.5 or < 0.67.

Table S14. Intersection target differentially expressed genes of 32 differentially expressed miRNAs (which were selected with the criteria of FDR < 0.05 and fold changes either > 1.5 or < 0.67.) in mRNA levels.

Table S15. Quality control parameters of the miRNA sequencing and mRNA sequencing in the present study.

Table S16. Primer sequences for PCR reactions in luciferase reporter study.

Figure S1. (a) The principal component analysis (PCA), (b) the partial least squares discriminant analysis (PLS-DA), and (c) the orthogonal projection of latent structures–discriminant analysis (OPLS-DA) of liver samples of 5 postnatal young (YC) and 5 mature chickens (AC). Note: For every panel (a, b, and c), the left part belongs to livers from adult chickens and the right part belongs to livers from the postnatal young chicks. t(1) = first principal component; t(2) = second principal component; to(1) = orthogonal component.

Figure S2. Size distribution of small RNAs in the libraries for livers of the postnatal chicks (YC) and adult chickens (AC).

Notes: a. the size distribution of the total small RNAs; b. the size distribution of unique miRNAs. nt = nucleotides. YC1, YC2, and YC3 represent the results of 3 replications from the postnatal young chicks using RNA sequencing and AC1, AC2, and AC3 represent the results of 3 replications from adult chickens using RNA sequencing.

Figure. S3. Venn diagrams of miRNAs detected from livers of the postnatal chicks (YC) and adult chickens (AC).

Table S1. Overview of reads from raw data to cleaned sequences in livers from chicken.

	Group AC						Group YC				
	AC1		AC2		AC3		YC1		YC2		Y
	Total	unique	Total	unique	Total	unique	Total	unique	Total	unique	Total
	19,344,974	6,066,338	13,551,037	1,192,170	14,692,026	996,267	11,844,048	3,129,976	16,760,492	5,156,931	11,262,577
er	10,903,585	3,324,188	4,087,873	731,790	2,710,576	594,271	4,630,425	1,635,192	5,838,433	2,121,437	5,295,456
	24,501	19,189	7,400	4,337	6,919	4,444	17,194	13,126	34,395	27,222	16,770
	532,309	70,904	459,933	48,985	407,219	32,381	425,684	44,996	627,500	59,746	470,081
	1,512,504	1,078,030	204,298	146,518	165,090	127,932	1,323,549	772,073	2,759,891	1,484,847	1,415,792
	4,577	1,252	7,950	245	3,071	161	5,457	872	4,857	1,548	4,529
	6,429,847	1,586,455	8,813,361	265,945	11,416,566	241,187	5,484,721	673,438	7,554,585	1,476,665	4,097,655
	251,313	26,597	260,608	22,939	225,816	15,122	227,092	17,357	250,895	20,080	280,630
	108,015	15,234	104,268	14,580	95,388	6,813	95,660	9,375	211,695	12,888	77,320
	54,270	10,818	50,934	3,512	45,432	3,761	28,716	5,478	41,316	8,214	21,111
	44,909	6,455	8,647	2,506	5,774	2,230	24,861	4,145	45,404	6,668	21,300
[illegible]	73,802	11,800	35,476	5,448	34,809	4,455	49,355	8,641	78,190	11,896	69,720

Table S2. Summary of known and predicted miRNA.

	AC libraries						YC libraries					
	AC1		AC2		AC3		YC1		YC2		YC3	
Criteria	Pre-miRNA	Unique miRNA	Pre-miRNA	Unique miRNA	Pre-miRNA	Unique miRNA	Pre-miRNA	Unique miRNA	Pre-miRNA	Unique miRNA	Pre-miRNA	Unique miRNA
gp1a	580	680	481	577	460	560	537	647	559	684	484	579
gp1b	2	2	1	1	1	1	1	1	2	2	2	2
gp2	28	30	26	28	32	33	32	31	31	32	33	32
gp3	257	270	244	258	267	292	279	321	301	339	229	248
gp4	14	16	8	8	9	9	13	15	14	16	14	16

Table S3. Summary of all expressed known miRNAs of chicken livers in this study.

miR_name	miR_seq	len	genome ID	group	SS	AC1(norm)	AC2(norm)	AC3(norm)	YC1(norm)	YC2(norm)	YC3(norm)
gga-miR-122-5p	TGGAGTGTGACAATGGTGTTTGT	23	JH375727.1	gp1a	(((((((((..(((((((.((((((((((((.................))))))))))))).)))))))..))))))).))))....	1422861.04	1084522.12	1061818.31	679923.77	668513.47	614622.64
gga-miR-99a-5p_R-1_1ss16TA	AACCCGTAGATCCGAACTTGT	21	1	gp1a	...((((((..((.((((((((((((((((.(((((((((..(((.(((((((	322573.23	321839.62	518657.44	372452.77	511597.38	401989.06

					......<br>.....)<br>))))))<br>.)))..<br>))))))<br>))).))<br>))))))<br>).))))<br>.....)<br>)))..)<br>).....<br>.)))))<br>)...						
gga-miR-21-5p	TAGCTT<br>ATCAGA<br>CTGATG<br>TTGA	22	19	gp1a	...(((<br>(((...<br>((((((<br>((((((<br>((((.(<br>((((.(<br>((((.(<br>..(...<br>.....)<br>..))))<br>)).)))<br>))))))<br>))))))	316266.54	219846.8<br>3	319748.01	120421.6<br>6	131154.<br>60	93681.4<br>1

					))))))...))))))...........						
gga-miR-26a-5p_R+1	TTCAAGTAATCCAGGATAGGCT	22	2	gp1a	...((((((.((..((.((((((((.((((((((((((.......))).))))))))).))))))))).))..)).)))))))....	206511.19	218724.67	308579.28	177700.38	190039.98	168976.61
gga-miR-148a-3p	TCAGTGCACTACAGAACTTTGT	22	2	gp1a	.((((((((.((..(((((((((((.(((	162432.69	160011.94	222250.52	160954.44	208377.01	186399.37

					(..(((((((.......))).))))))))...))))))))))))..)).))))))))).						
gga-miR-2954_R+2	CATCCCCATTCCACTCCTAGCAGT	24	Z	gp1a	(((((..((((..(((((..((((((..(((..((......))))).))))))..)).)))..)))))))))).....	133994.95	157136.95	125983.02	68414.09	62999.40	75618.97
gga-miR-126-3p_R-2	TCGTACCGTGAGTAATAA	21	17	gp1a	((.((((((..(((((.(	116851.62	100334.90	150579.77	112467.31	137130.45	108984.95

	TGC				((((((((((..(((((((.....(((....)))....)))))))..))))))))).)))))..))))))))).						
gga-let-7a-5p	TGAGGTAGTAGGTTGTATAGTT	22	1	gp1a	...(..(((..((.((((((..(((...(..(((((((((((((((.(((((.....))))))..........)))))	97185.61	95599.55	92748.41	67997.26	59785.52	54486.59

					))))))))))).)..)))..))).))).))))..)...						
gga-miR-30c-5p	TGTAAACATCCTACACTCTCAGCT	24	3	gp1a	...(((((..((.(((..((..((...((((....(((((((.(((...(((((((((((....))......)))))))))).)).)))))))....))))).))..)).....)	93427.94	95842.82	155736.39	192695.62	221097.83	225634.42

					)).))..)))))...						
gga-miR-451	AAACCGTTACCATTACTGAGTTT	23	19	gp1a	...((((((.(((((.((((((((((((((((((....)))))))))))))))))).))).))))))...	74236.99	197537.05	181132.50	42360.29	37895.65	30364.73
gga-miR-22-3p	AAGCTGCCAGTTGAAGAACTGT	22	19	gp1a	.....(((((((....((((((((((((((((((.(((((((.((.((.....))..)))))))))))	60921.31	74684.18	57599.53	26406.92	25200.82	31176.12

					)))).))))))))))))))...)))))))...						
gga-miR-125b-5p	TCCCTGAGACCCTAACTTGTGA	22	24	gp1a	...(((..((((..(((.((.(((.((((.(((((..(((..((((((...))))))).)))..)))))).)))))))))..)).)))..))))..)))...	58677.75	55626.50	56667.41	32650.48	26424.53	23446.84
gga-miR-101-3p_R-1	GTACAGTACTGTGATAAC	21	Z	gp1a	((.((((((.(((((((((	57076.37	53644.89	76897.28	57763.43	97572.96	76760.99

	TGA				((((((..((((..(((((.......................))))).)))).)))))))))))))).))))))..)).........						
gga-miR-100-5p_R+1	AACCCGTAGATCCGAACTTGTGA	23	24	gp1a	...((((.(((..(((((.(((.((((((...........))))).))).)))))..)).))))..........	54193.08	57611.16	113714.47	52414.88	77573.55	54413.00

gga-let-7a-5p_2ss12GT18TC	TGAGGTAGTAGTTTGTACAGTT	22	12	gp1a	......(((((.((((((((.((((((((.(..((((((((.....((((.((.((((....))))))..))))))))))))))..).))))))))))))))))))).))...	47997.54	45865.64	41181.18	32424.19	32269.36	25957.53
gga-miR-199-3p_L-1R+2	ACAGTAGTCTGCACATTGGTT	21	8	gp1a	(((((((.(((...(((((((.((((((((.(((....((((	44241.52	42156.75	48371.99	44364.65	41828.27	32330.90

					....))))..))))))))))).))))))).
..))).))))).))...........						
gga-let-7k-5p_1ss16AT	TGAGGTAGTAGATTGTATAGTT	22	26	gp1a	...(((((((((.(((..(((..(((((((((.(((((((((((((.......))))......)).)))))).))))))))))..))).))).))).)	43308.69	41910.81	47164.76	30632.41	35809.99	28326.94

					)))))...						
gga-let-7f-5p	TGAGGTAGTAGATTGTATAGTT	22	12	gp1a	...((((.((((...((((((((((((((((((((((((((((... ..))))))).........)))))))))))))))))))))))))). .))))))))...	43308.69	41910.81	47164.76	30632.41	35809.99	28326.94
gga-miR-30a-5p_R+2_1ss13CT	TGTAAACATCCTTGACTGGAAGCT	24	3	gp1a	....((.((((..((..(((((((((((..((((((((((((	40998.43	45620.08	80963.07	108958.78	160514.01	144441.70

					................))))))))))))))))))))))..))..))).))						
gga-miR-30e-5p_R+5	TGTAAACATCCTTGACTGGAAGCT	24	23	gp1a	..((((((((...((.((((((((((((((..((((((((((((((((...).))).)...))))))))))))))))))))))).)).)))))))..	40998.43	45620.08	80963.07	108958.78	160514.01	144441.70
gga-miR-126-5p	CATTAT	21	17	gp1a	((.(((	38836.37	39769.44	29838.66	26208.03	26051.6	27918.8

	TACTTT				(((..(					3	0
	TGGTAC				((((.(						
	GCG				((((((						
					(((..(						
					((((((						
					.....(						
					((....						
					)))...						
					.)))))						
					))..))						
					))))))						
					)).)))						
					))..))						
					))))))						
					.						
gga-miR-30d_R+2	TGTAAA	24	2	gp1a	....((	31188.48	37704.33	58387.36	65466.49	89124.2	76212.5
	CATCCC				(.....					4	4
	CGACTG				...)))						
	GAAGCT				...(((						
					.(((((						
					((((..						
					.(((((						
					((((((						
					......						
					.....)						
					))))))						

					)))).)))))))).)))..					
gga-miR-199-5p	CCCAGTGTTCAGACTACCTGTTC	23	8	gp1a	(((((((.(((...(((((((.((((((((.(((....((((....))))..)))))))))).)))))))...))).))))).)).............	24760.76	23561.20	33587.97	32527.21	29102.68	23899.14
gga-miR-92-3p_R+1	TATTGCACTTGTCCCGGCCTGT	22	1	gp1a	..(((((..((((..((.((((((((..(.	24128.44	28452.50	19172.70	37086.17	31919.27	30980.88

					.......)..))))))))))..))))..)))))....						
gga-miR-146c-5p_R-1	TGAGAACTGAATTCCATGGACT	22	4	gp1a	.......((((..((.((((..((((((((((.((((((((((...((......)).. .))))))))))).))))))))))).)))).))))))...............	23361.10	30905.39	37196.20	31470.24	39494.90	35402.79

gga-miR-1662	TTGACATCATCATACTTGGGAT	22	2	gp1a	((.((((..((((((((((...((((((((............))))))))..)).))))))))..)))))).	22239.73	17582.44	19045.08	15354.32	12825.31	12699.64
gga-miR-140-3p_L+1	ACCACAGGGTAGAACCACGGAC	22	11	gp1a	.........(((..(((((((.((((((((((((((.(((((((..(((......))))))))))).))))))))))))))).)))	20502.51	27061.52	18272.32	14764.20	15051.54	14118.34

					))..).))						
gga-let-7c-5p_1ss17AG	TGAGGTAGTAGGTTGTGTGGTT	22	1	gp1a	...((.((((..(((((((((.(((..(..(((.(((.((((((((..(((..((.(..(.....)..).)))))..))))))))).))).)))..)..))))))))).)..))).)))))))...	19167.89	18545.98	15820.34	9757.45	7405.89	6696.15
gga-let-7i_R+1	TGAGGTAGTAGTTTGTGC	22	1	gp1a	((((..(((((.((((((	18328.10	12008.24	8960.77	15547.25	15111.48	11896.10

	TGTT				(((((((((((. (((((. (((((. ....)))))))... .....))).))))))))))))))))))))))..)))).....						
gga-miR-30b-5p	TGTAAA CATCCT ACACTC AGCT	22	2	gp1a	...... ((((((((((.((.(((((((... ((.... .))..))))))) ..))))))))))))....	15391.29	18800.69	24639.71	25686.40	34467.4 7	35720.6 7

gga-miR-146c-5p_4ss18GA20AG22TG23GT	TGAGAACTGAATTCCATAGGCGT	23	6	gp1a	...((((((..(((...((((((((((.(((((((((((.........)))).))))))))))))))))..)))..)))))))...	13965.29	11828.26	16926.89	1924.57	2776.28	1773.87
gga-miR-221-3p	AGCTACATTGTCTGCTGGGTTTC	23	1	gp1a	.....(((((((....((...(((((..(((.(((((((((....((((((((..)))).)))))	13561.85	13708.83	13718.19	22204.66	19081.23	18735.50

					))))))<br>)).)))<br>..))))<br>)))...<br>.)))))<br>))...						
gga-miR-2188-5p	AAGGTC<br>CAACCT<br>CACATG<br>TCCT	22	22	gp1a	....((<br>(((((.<br>(((.((<br>((((((<br>(.((((<br>(..(((<br>.((.((<br>(((((.<br>......<br>......<br>.)))))<br>)).)).<br>)))..)<br>)))).)<br>))))))<br>))..))<br>))))))<br>))...	13454.82	38290.00	19959.56	11405.72	6892.43	7040.53
gga-let-7k-5p_R+1_1<br>ss16AT	TGAGGT<br>AGTAGA	23	12	gp1a	...(((<br>(.((.(	10494.96	12078.39	16548.85	4459.51	4702.81	3596.79

	TTGTATAGTTT				((((.(((..((((((((((((((((.....(((...((((....))))).)))))))))))))))))))..)))))))).))..))))...						
gga-miR-16-5p	TAGCAGCACGTAAATATTGGTG	22	1	gp1a	...((..(((.((((..((((((((((.((((((((........................))))	10286.66	13703.11	12573.16	14577.82	17447.50	15101.42

					)))).)).))))))))..)))).)))..))...						
gga-miR-15b-5p_R-1	TAGCAGCACATCATGGTTTGC	21	4	gp1a	...((((((((..(((..(((((.((..((((((((((..........))))))))))..)).)))))..)))..)))))))...	9703.74	14757.78	8983.85	18425.18	19742.68	15419.31
gga-miR-17-5p_R-1	CAAAGTGCTTACAGTGCAGGTAG	23	1	gp1a	...((..(((((((..((((((..((((((	9541.55	11238.01	13546.64	14374.16	16699.06	14971.92

					((.((.<br>(((((.<br>..((..<br>......<br>..))..<br>.)))))<br>.)).))<br>))))))<br>...)))<br>)))..)<br>)))).)<br>)..)).<br>..						
gga-miR-29c-3p_R+1	TAGCAC<br>CATTTG<br>AAATCG<br>GTT	21	1	gp1a	...(((<br>((((((<br>(((...<br>((((((<br>(.((((<br>......<br>.....)<br>))))))<br>))))..<br>.)))))<br>))))))<br>)...	9433.69	11859.91	15923.91	1633.98	3640.88	2853.10
gga-miR-142-5p_L-2R	CATAAA	21	19	gp1a	...(((	9100.24	10595.52	8178.39	2400.95	3197.44	2225.18

+1	GTAGAA<br>AGCACT<br>ACT				((..((<br>((((((<br>.(((.(<br>((((((<br>((((.(<br>((((((<br>((....<br>..((..<br>.))...<br>..))))<br>))))).<br>))))))<br>))))).<br>))).))<br>))))))<br>...)).<br>)))...						
gga-miR-10a-5p	TACCCT<br>GTAGAT<br>CCGAAT<br>TTGT	22	27	gp1a	...(((<br>(..(((<br>(((((.<br>((((.(<br>((((.(<br>((((((<br>(((...<br>......<br>......	8826.07	10452.16	44593.18	9390.64	11878.4<br>9	8297.35

					)))))))))).)))))))).))))))))).)))))...						
gga-miR-10b-5p_1ss12AT	TACCCTGTAGATCCGAATTTGT	22	7	gp1a	...(((((((((((...(((((((.........(((.((.((((((.((((((.((((.(((.(((((((((((((...(((.....)))..)))))))))).))))))))).))))	8826.07	10452.16	44593.18	9390.64	11878.49	8297.35

					)))).))))).).))))))))))..)))))).)))..						
gga-miR-34a-5p_R-1	TGGCAGTGTCTTAGCTGGTTGT	22	21	gp1a	...((((((((((((..(((((..(((((((((((.(((((((((((...(((....)))......))))))))))))).)))))))..)))))..)).)))).)))))...	7943.47	8772.92	7657.02	8605.80	10705.18	9287.30

gga-miR-200a-3p_R+1	TAACACTGTCTGGTAACGATGTT	23	21	gp1a	...((((((((((.(..((((((((((((((((((.((((...............)))).)))))))))))))).))))).).))))).)).)))...	7599.32	4474.92	10392.13	5899.96	7020.80	7874.48
gga-miR-214_R+1	ACAGCAGGCACAGACAGGCAGT	22	8	gp1a	...((((((.....(((((((((((.(((((((((((...((((((((((((......	7567.21	8825.54	4155.57	5903.54	4364.39	3306.38

					....))<br>))))))<br>))))..<br>.)))))<br>))))).<br>))))))<br>))))))<br>))))).<br>....					
gga-miR-142-3p	TGTAGT<br>GTTTCC<br>TACTTT<br>ATGG	22	19	gp1a	...(((<br>((..((<br>((((((<br>.(((.(<br>((((((<br>((((.(<br>((((((<br>((....<br>..((..<br>.))...<br>..))))<br>))))).<br>))))))<br>))))).<br>))).))<br>))))))<br>...)).	7444.53	8309.64	10241.43	1281.46	2406.57	1624.74

					)))...						
gga-miR-223_R+1	TGTCAGTTTGTCAAATACCCCA	22	4	gp1a	...(((\(...((\(((..(\(((....(((((\((((((\(((......(((\((((.(.....).))))))))))))))))))))))..\..))))..)))))).))))...	7377.02	11165.18	10796.14	4799.53	5064.56	3690.98
gga-miR-146a-5p_R+1	TGAGAACTGAATTCCATGGGTTG	23	13	gp1a	..((((..(((((((((..((((((((((.....(((.....	7270.81	6184.66	10156.46	3270.94	3691.28	3236.72

					...))))))))))))..))))))))).))))..						
gga-miR-130b-3p	CAGTGCAATAATGAAAGGGCGT	22	15	gp1a	...(((((((..(((((((((.((((.......))))......)))))))))..))))))))........	6982.64	7980.58	6456.84	34825.15	24491.63	25181.47
gga-miR-122-3p_R-1	AACGCCATTATCACACTAAAT	21	JH375727.1	gp1a	(((((((((..((((((((.(((((((((((.............))))))	6431.01	5199.77	7872.50	6867.01	9106.46	9160.73

					))))).						
					))))))						
					)..)))						
					))).))						
					)....						
gga-miR-24-3p_R+1	TGGCTC	23	Z	gp1a	......	6418.66	6360.82	6863.10	6513.30	6383.22	5823.94
	AGTTCA				((((((						
	GCAGGA				(.((((						
	ACAGT				(...((						
					(.....						
					(((((.						
					...)))						
					))..))						
					)..(((						
					.(((((						
					((((..						
					(((((.						
					.((...						
					...)).						
					))))).						
					))))))						
					))).))						
					))))))						
					.))))..						
					))))..						
					.						

gga-miR-107-3p_R-1	AGCAGCATTGTACAGGGCTATC	22	6	gp1a	...((((..((((((.(((.((((.((((((((((.(((.........(...)))))))))))))).))))))...))))))..)))))..	6369.26	7804.42	7933.42	17972.62	16911.23	17611.13
gga-miR-193b-3p_R-2	AACTGGCCCACAAAGTCCCGCT	22	14	gp1a	...((((..((((..((((.((((.(((.(((((....))).....)).)).)))).)))	6318.22	11609.02	7542.23	5019.85	3994.15	5148.93

					)..)))<br>)..)))<br>))...						
gga-miR-375_R-1	TTTGTT<br>CGTTCG<br>GCTCGC<br>GTT	21	7	gp1a	...(((<br>.(((((<br>(((..(<br>(.((((<br>((..((<br>(..(((<br>(((...<br>......<br>..))))<br>))..))<br>)..)))<br>))))).<br>.)))))<br>))).))<br>)...	6249.06	5190.24	1689.16	7831.68	7077.02	9365.79
gga-miR-27b-3p	TTCACA<br>GTGGCT<br>AAGTTC<br>TGC	21	Z	gp1a	.(((((<br>(((...<br>.(((((<br>((((((<br>((((((<br>.((((.<br>((((((<br>(.....	6132.15	6571.30	5107.25	8511.72	8723.48	7313.28

					......<br>....))<br>))))))<br>))))))<br>))))))<br>))))))<br>))..))<br>))).))<br>)..						
gga-miR-16c-5p	TAGCAG<br>CACGTA<br>AATACT<br>GGAG	22	4	gp1a	...(((<br>((...(<br>((..((<br>(((((.<br>...(((<br>((((((<br>(...((<br>(....)<br>))....<br>..))))<br>))))))<br>....))<br>))))).<br>.)))..<br>.)))))<br>...	5761.65	8677.21	6691.24	13066.50	13147.8<br>2	10985.6<br>2
gga-miR-222a	AGCTAC	24	1	gp1a	...((.	5517.94	7114.27	5313.74	12914.66	10177.9	11061.1

	ATCTGG				((.((.					3	6
	CTACTG				.(((((						
	GGTCTC				...(((						
					((((((						
					((((((						
					(((...						
					(((...						
					......						
					.))))..						
					.)))))						
					.)))))						
					))))))						
					))...)						
					)))).)						
					).))))						
					...						
gga-miR-103-3p	AGCAGC	23	13	gp1a	..((((	5218.25	7421.21	6468.07	6151.25	6507.34	5305.91
	ATTGTA				((((((						
	CAGGGC				.(((.(						
	TATGA				((((((						
					((((.(						
					((....						
					......						
					..))))						
					))))))						
					)))).)						

					)))))).<br>.))))))<br>))..						
gga-miR-181a-5p	AACATT<br>CAACGC<br>TGTCGG<br>TGAGT	23	17	gp1a	...(((<br>((((((<br>((((((<br>...(((<br>.(((((<br>(..(((<br>((((.(<br>(((...<br>......<br>...)))<br>))))))<br>))))))<br>)).)))<br>...)))<br>))))).<br>))).))<br>))...	5131.80	5165.45	5042.16	12080.40	9325.52	9223.52
gga-miR-29b-3p_R-1	TAGCAC<br>CATTTG<br>AAATCA<br>GTGT	22	1	gp1a	((((((<br>((((((<br>(.((((<br>((..((<br>((((..<br>......	4976.19	4412.39	6965.71	807.46	2309.50	1889.64

					.....)						
					))))))						
					))))).						
					))))))						
					)))).)						
					))..						
gga-miR-200b-3p	TAATAC	23	21	gp1a	..((((	4881.51	4142.43	8794.99	3948.59	4113.50	4397.39
	TGCCTG				(..(((						
	GTAATG				(...((						
	ATGAT				((((((						
					..((((						
					(.((((						
					((....						
					......						
					...)))						
					))).))						
					)))..)						
					))).))						
					))...)						
					)))..)						
					))))..						
gga-miR-7_R+2	TGGAAG	24	Z	gp1a	......	4753.07	6787.11	17647.70	2023.42	3545.94	2287.98
	ACTAGT				((((((						
	GATTTT				((....						
	GTTGTT				...(((						
					...)))						

					...((.<br>...(((<br>(((.((<br>.(..((<br>(((.((<br>(((.((<br>((.(((<br>((((((<br>(((((.<br>...)))<br>))..))<br>))))))<br>))))))<br>)))).)<br>))))..<br>).))))<br>))))..<br>))))))<br>))))..<br>..						
gga-miR-130c-3p_1ss<br>10GA	CAGTGC<br>AATATT<br>AAAAGG<br>GCAT	22	19	gp1a	..((.(<br>((.(((<br>..((((<br>((((.(<br>..((((<br>(((.((	4385.87	6250.25	9812.08	8512.31	12879.9<br>5	10885.5<br>4

gga-miR-130a-3p	CAGTGCAATATTAAAAGGGCAT	22	15	gp1a	.(((......)))..)).))))))))..).)))))))))..))).)))))...(((((((((((((((...(((..(((((((((..((((((((.((..((....))...)).))))))))))..))))))))..)))...))))...)))).	4385.87	6250.25	9812.08	8512.31	12879.95	10885.54

					....)))))).))						
gga-miR-365-3p	TAATGCCCCTAAAAATCCTTAT	22	18	gp1a	..........(((((((.((((((((..(((.((((((((((((.(((.....)).))))).....))))))))))..))))))))).)))))))...	3556.78	5451.04	4853.94	3368.01	3784.10	4599.50
gga-miR-454-3p	TAGTGCAATATTGCTTATAGGGT	23	15	gp1a	..........((((((((..(((((((((((((((((((((	3434.92	4766.99	13161.55	24796.76	32794.49	23958.99

					(((..(						
					......						
					.)..))						
					)).)).						
					))))))						
					)))...						
					))))))						
					)))..)						
					)))).)						
					)))...						
					......						
gga-miR-128-3p	TCACAG	21	2	gp1a	((.(((	3313.07	3617.76	3241.73	4819.18	3871.09	3802.83
	TGAACC				(..(((						
	GGTCTC				((((..						
	TTT				.(((((						
					(..(((						
					(.((..						
					...)).						
					.)))))						
					))))).						
					..))))						
					)))..)						
					))).))						
gga-miR-140-5p_L+1	CAGTGG	22	11	gp1a	......	2805.90	3318.82	4100.42	1691.15	1869.24	1646.32
	TTTTAC				...(((						
	CCTATG				..((((						

	GTAG				(((.((((((((((((.((((((((..(((......)))))))))).)))))))))))))).))))..).))						
gga-miR-30a-3p_1ss18GA	CTTTCAGTCGGATGTTTACAGC	22	3	gp1a	....((.((((..((..(((((((((((..((((((((((((..................)))))))))))))))))))))	2748.27	2673.67	3722.38	6094.68	7595.79	6265.44

					)))..))..))))).))						
gga-miR-30e-3p_L+1	CTTTCAGTCGGATGTTTACAGC	22	23	gp1a	..((((((((...((.(((((((((((((..(((((((((((((((((...).))).)...))))))))))))))))))))))).)).))))))))..	2748.27	2673.67	3722.38	6094.68	7595.79	6265.44
gga-miR-32-5p	TATTGCACATTACTAAGTTGC	21	2	gp1a	....(((.(((.((.(((((..((((((((((.((((((((((	2573.72	2578.34	5155.98	1637.56	3651.49	2421.41

					((((..<br>((....<br>))..))<br>.)))))<br>))))))<br>))))))<br>))))..<br>))))).<br>..)).)<br>)).)))<br>..						
gga-let-7b_R+2	TGAGGT<br>AGTAGG<br>TTGTGT<br>GGTTTT	24	1	gp1a	((....<br>(((.((<br>(.((((<br>..((((<br>((((((<br>((((((<br>(((((.<br>..((((<br>((....<br>.)))))<br>)..(((<br>....))<br>))))))<br>))))))<br>))))))	2391.77	2699.59	2956.35	504.96	423.82	342.41

					))))).)<br>))).))<br>))))))<br>....						
gga-miR-429-3p	TAATAC<br>TGTCTG<br>GTAATG<br>CCGT	22	21	gp1a	...((.<br>(((((.<br>((((((<br>.(..((<br>((((((<br>((..((<br>(((((.<br>.((...<br>...)))<br>))))))<br>..))))<br>))))))<br>..).).<br>))))))<br>)).)))<br>)...	2327.55	2675.95	2677.07	3804.49	4335.22	3991.20
gga-miR-30c-1-3p_R+1	TGGGAG<br>AGGATT<br>GTTTAC<br>GCCT	22	23	gp1a	..((((<br>.((..(<br>((.((.<br>.(..((<br>.((...<br>..((.(	2007.27	2135.27	1895.66	3413.86	2878.12	3359.36

					((((..						
					((((..						
					.(((((						
					((((((						
					((...(						
					((((((						
					(..(..						
					......						
					.)..))						
					))))))						
					)))).)						
					))))))						
					))...)						
					))))))						
					))))..						
					.)).))						
					..)..)						
					))))..						
					))))))						
					..						
gga-miR-181b-5p_R+1	AACATT	23	17	gp1a	.(.(((	1989.16	1916.79	2396.51	5621.88	5157.39	4870.29
	CATTGC				(.(((.						
	TGTCGG				((.(((						
	TGGGT				((....						
					..((((						
					((((..						

gga-miR-23b-3p	ATCACATTGCCAGGGATTACC	21	Z	gp1a	...(((((((((..((((((((((((.................))))))))))))))))))))).....))))).))))))))))..))))))))...((..(((((((.(((((((((((((((.((((((.(((...))))))))))))))).))))))))	1844.25	2544.02	2347.13	2156.81	2770.98	2062.32

					))..)))).))))))..)).						
gga-miR-20b-5p	CAAAGTGCTCATAGTGCAGGTAG	23	4	gp1a	(((.((.(((.(((((((((..(((((((((((.(((((.(((.((........)).))))))))).))))))))))))..)))))))))))).))))).	1832.73	2452.51	2603.00	8999.41	10104.73	9234.32
gga-miR-1454_L-1R+1	TACAATGATGAGACTTTGGCTCCC	24	3	gp1a	...(((((((..(((.......(((.....	1744.63	237.55	272.23	403.73	705.47	480.75

					.)))......)).)))))))........................						
gga-miR-1559-5p	TTCGATGCTTGTATGCTACTCC	22	chr7	gp1a	(((.(((..((((((((.(..(((..(((((.........................)))))..)))..).))))))))..))).)))	1738.87	1557.99	1870.65	2653.44	2650.04	2980.65
gga-miR-1451-5p	TCGCACAGGAGCAAGTTACCGC	22	3	gp1a	...((((((((.((((((((((((((((.(((((.(	1612.08	2078.84	1178.69	1669.71	1332.45	1346.10

gga-miR-33-5p	GTGCAT<br>TGTAGT<br>TGCATT<br>GC	20	14	gp1a	((.(((<br>(((.((<br>(.....<br>......<br>))).))<br>)))).)<br>)).)))<br>)).)))<br>))))))<br>))))))<br>))))..<br>...)))<br>))...<br>...(((<br>((((((<br>.(((.(<br>((((.(<br>(((..(<br>((((((<br>((...(<br>(.....<br>))..))<br>))))))<br>)..)))<br>).))))<br>).))).	1506.69	1547.69	1370.44	242.36	551.65	620.07

					))))))<br>)))...						
gga-miR-190a-5p_R+1	TGATAT<br>GTTTGA<br>TATATT<br>AGGTT	23	10	gp1a	......<br>((((((<br>(.((((<br>(.((((<br>((((((<br>((((((<br>..((((<br>(.....<br>.....)<br>))))))<br>))))))<br>))))))<br>))..))<br>))).))<br>))))).<br>......<br>...	1267.92	1676.95	3182.73	1385.07	2444.23	1970.09
gga-miR-19a-3p	TGTGCA<br>AATCTA<br>TGCAAA<br>ACTGA	23	1	gp1a	.(..((<br>((((((<br>((((((<br>((((((<br>((((..<br>.....)<br>)))...	1174.07	1926.32	9687.99	2705.84	6002.37	5041.00

					))))))<br>..))))<br>))))))<br>))))..)..						
gga-miR-194_R+1	TGTAAC<br>AGCAAC<br>TCCATG<br>TGGAC	23	3	gp1a	...(((<br>((((((<br>.(((((<br>(.((((<br>......<br>.....)<br>))))))<br>))).))<br>))))))<br>).....	1086.79	92.27	110.94	57.17	160.19	96.15
gga-miR-458a-3p	ATAGCT<br>CTTTGA<br>ATGGTA<br>CTGC	22	13	gp1a	...((.<br>.(((((<br>(.((((<br>(((..(<br>(((((.<br>.(((((<br>(((..(<br>(((...<br>..))))<br>.)))))<br>)))..)	1085.15	1143.90	984.38	211.99	166.56	198.19

					))))).)))))))).))))))).						
gga-miR-455-5p	TATGTGCCCTTGGACTACATCG	22	17	gp1a	...((.((..(..((.((((((((((.((((((.(((.(.(((.........))).))).))))).))))))))).)).).)..)).))...	864.49	770.22	868.31	1339.82	1611.45	1455.98
gga-miR-460a-5p	CCTGCATTGTACACACTGTGTG	22	2	gp1a	..(((((..((.((((.(((((((....(((.......))	861.20	957.44	791.35	1408.30	1311.76	1604.13

					))))))))).)))).)).).)))))...........						
gga-miR-456-3p_1ss22AT	CAGGCTGGTTAGATGGTTGTCT	22	3	gp1a	((((..(((...(((((((....(..((((....))))..))))))))..(((((..((.....))..)))))))))..))))	857.08	899.48	633.92	2657.60	2921.09	3110.16
gga-miR-219b_R-1	CACAAGAATTGCGTTTGGACA	21	17	gp1a	...((((((((((...((((((((((.(((((((((	732.76	722.94	985.02	649.07	753.21	722.11

gga-miR-199-3p_L-1R<br>+4	ACAGTA<br>GTCTGC<br>ACATTG<br>GTTTT	23	chr8	gp1a	(...((<br>(.....<br>....))<br>)..)))<br>))))))<br>.)))))<br>))).))<br>)...))<br>))))))<br>))...<br>(((.((<br>.((((.<br>(..(((<br>((((.(<br>((((((<br>(.((((<br>((....<br>......<br>..))))<br>))))))<br>)))).)<br>))))))<br>..).))<br>)).)).<br>)))...<br>.....	718.77	746.96	1237.05	319.77	332.58	218.79

gga-miR-1729-5p_R-1	ATCCCTTACTCACATGAGTAGT	22	15	gp1a	...((((((...((((..(((((((((((.(((((((((((.(((((((((....(((((....)))))))))))))))))))))))))))))).)))))))..)).)).))))))...	706.42	675.66	753.52	278.68	361.75	353.20
gga-miR-146b-3p_L+2	TGCCCTATGGATTCAGTTCTGC	22	6	gp1a	...((((((..(((...(((((((((.((((((((((...)......	694.89	626.85	506.62	90.51	71.08	62.79

					...))).)))))))))))))))..)))..))))))...						
gga-miR-22-5p_R-1	AGTTCTTCAGTGGCAAGCTTT	21	19	gp1a	.....((((((....((((((((((((((((((.((((((.((.((.....))..))))))))))).))))))))))))))...)))))))...	650.43	622.28	559.21	438.27	475.27	457.20
gga-miR-130b-5p	CCTCTTTCCCTG	22	15	gp1a	...((((((((..	641.37	720.65	451.79	2942.24	2274.49	3155.29

	TTGCAC				((((((						
	TACT				(((.((						
					((....						
					...)))						
					).....						
					))))))						
					)))..)						
					))))))						
					......						
					.						
gga-miR-455-3p_L+1	ATGCAG	23	17	gp1a	...((.	630.67	768.70	667.58	894.40	1332.45	1206.78
	TCCATG				((..(.						
	GGCATA				.((.((						
	TACAC				((((((						
					((.(((						
					(((.((						
					(.(((.						
					......						
					.))).)						
					)).)))						
					))).))						
					))))))						
					)).)).						
					.)..))						
					.))...						
gga-miR-460a-3p	CACAGC	22	2	gp1a	..((((	592.80	597.11	493.79	1034.94	1355.78	1652.21

	GCATACAATGTGGATT				(..((.((((.((((((((....(((......)))))))))))).)))).)).))))))..........						
gga-miR-101-2-5p_R-1	TCAGTTATCACAGTGCTGATGC	22	8	gp1a	.........(((((((.....(((((((((((((((.(((((............))))))))))))))))))))))).....)	535.16	713.03	1352.48	326.32	571.81	408.15

					))))))...						
gga-miR-218-5p	TTGTGCTTGATCTAACCATGT	21	13	gp1a	.((...((((((((..(((((((((.......................)))))))).)))))))))))...))	507.99	476.24	642.25	634.18	622.73	520.98
gga-miR-146a-3p_R-1	ACCCATGGGGCTCAGTTCTTCA	22	13	gp1a	..((((..(((((((((.(((((((((((.....(((.........)))))))))))))..))))))))).))	469.30	520.09	579.09	161.97	138.97	125.58

					))..						
gga-miR-221-5p_L-1R<br>-4	ACCTGG<br>CATACA<br>ATGTAG<br>ATTT	22	1	gp1a	.....(<br>((((((<br>....((<br>...(((<br>((..((<br>(.((((<br>(((((.<br>...(((<br>(((((.<br>..))))<br>.)))))<br>))))))<br>)).)))<br>..))))<br>)))...<br>.)))))<br>))...	462.71	407.23	1162.02	884.88	1333.51	1232.29
gga-miR-1a-3p_R+1	TGGAAT<br>GTAAAG<br>AAGTAT<br>GTAT	22	2	gp1a	...((.<br>.(((((<br>.(((((<br>.(((((<br>((((((<br>((..(.<br>.(((((<br>......	461.89	186.07	640.33	512.11	423.82	380.68

gga-miR-21-3p_R-1	CAACAA<br>CAGTCG<br>GTAGGC<br>TGT	21	19	gp1a	......<br>..))))<br>).)..)<br>))))))<br>))))))<br>..))))<br>))))))<br>..))..<br>.<br>...(((<br>(((...<br>((((((<br>((((((<br>((((.(<br>((((.(<br>((((.(<br>..(...<br>.....)<br>..))))<br>)).)))<br>))))))<br>))))))<br>))))))<br>...)))<br>)))...<br>......	429.78	339.36	339.24	307.26	402.07	392.45

gga-miR-33-3p_L+1R+1_2	NAATGTTCCTGCAGTGCAGTAT	22	chr1	gp1a	..(((((.((((((((((..((((((((((((((.....)).)).))))))))))..))))))))))).)))))))	424.84	423.24	219.96	53.59	85.93	82.41
gga-miR-33-3p_L+1R+1_1	CAATGTTCCTGCAGTGCAGTAT	22	1	gp1a	.((.....)).....(((((.(((((((((((.(((((((((((((.....)).)).)))))))))..)))))))))).)	424.84	423.24	219.96	53.59	85.93	82.41

					))))..<br>.						
gga-let-7d_R+1	AGAGGT<br>AGTGGG<br>TTGCAT<br>AGTT	22	12	gp1a	...(((<br>.(((((<br>(.((((<br>((((..<br>(((((.<br>((((((<br>...(((<br>(((...<br>..))))<br>))....<br>......<br>))))))<br>.)))))<br>..))))<br>))))))<br>))))))<br>)...	409.19	397.31	236.64	339.42	279.01	162.87
gga-miR-135a-5p	TATGGC<br>TTTTTA<br>TTCCTA<br>TGTGA	23	1	gp1a	......<br>.(((((<br>....((<br>((((((<br>(((((.<br>(..(((<br>((((..	397.67	424.77	1039.85	237.59	238.16	230.56

					(((...<br>......<br>....))<br>)..)))<br>))))..<br>))))))<br>))))))<br>))...)<br>))))..<br>.						
gga-miR-1552-5p_R+1	TTAGTG<br>CGCGGT<br>AAGCTA<br>GGGTGT	24		gp1a	((((((<br>.((((.<br>.(((((<br>((((..<br>(((.((<br>(((.((<br>((....<br>.....)<br>))).))<br>))))))<br>..))))<br>))))).<br>.)))))<br>)))))	375.44	383.59	439.28	676.46	606.81	623.99
gga-miR-140-3p_L+1R<br>+1_1ss22CA	ACCACA<br>GGGTAG	23	chr11	gp1a	..((((<br>((((((	363.91	486.54	519.13	161.97	171.86	165.81

	AACCACGGAAA				(((.((.((((((((((((.(((((((..(((......))))))))))).))))))))))))))))))).)))))))))))))..					
gga-mir-6651-p5_1ss4TG	CGGGGAGCCGAGGCACACCAGGT	23	Z	gp1a	.(((.(((....))).)))...(((((..(((..(((((..((((((((....(((....))).))))))))	352.38	42.71	22.45	72.65	133.67	76.53

					..))))<br>)))).)<br>))))..<br>.((((.<br>......<br>...)))<br>).						
gga-miR-155_R+1	TTAATG<br>CTAATC<br>GTGATA<br>GGGGT	23	1	gp1a	...(((<br>((((..<br>((.(((<br>(.((((<br>..((((<br>((((((<br>((.(((<br>(.((((<br>((((((<br>((....<br>)))).)<br>))))))<br>))))))<br>))))))<br>))))).<br>.)))).<br>..))))<br>..))..<br>))))))	350.74	420.19	239.20	26.20	15.91	21.58

					)...						
gga-miR-125b-3p_R-1_1ss21CT	ACAAGTCAGGCTCTTGGGACT	21	1	gp1a	...((...(((..((((((..((..(((.(((.((((((((....((((....))))))))))).))).)))..))..))))).)))..))...	314.51	392.74	382.21	896.78	765.94	735.84
gga-miR-1456-5p_L-1	AAAGGACGGAGGCGGCCCGCGC	22	Z	gp1a	.((((..((....((((.(((((.(((((.((((((.((((((((.(((..((.	312.86	330.20	158.40	314.41	297.04	313.96

					...)).						
					).))))						
					))))))						
					.)))))						
					).))))						
					).))))						
					).))))						
					...)).						
					.)))).						
					..						
gga-miR-17-3p_L-1R+	CTGCAG	22	1	gp1a	...((.	294.75	328.68	307.18	543.67	691.15	620.07
3	TGAAGG				.(((((						
	CACTTG				((..((						
	TAGC				((((..						
					((((((						
					((.((.						
					(((((.						
					..((..						
					......						
					..))..						
					.)))))						
					.)).))						
					))))))						
					...)))						
					)))..)						
					)))).)						

					)..)).<br>..						
gga-miR-9-5p	TCTTTG<br>GTTATC<br>TAGCTG<br>TATGA	23	Z	gp1a	.....(<br>(((.((<br>(((.((<br>(..(((<br>((((((<br>((((((<br>.(((((<br>(..(..<br>..)..)<br>))))).<br>))))))<br>))))))<br>)))..)<br>)).)))<br>))))))<br>......	288.16	288.26	257.16	148.87	95.48	86.34
gga-miR-146c-3p_L-1	GTCCAT<br>GGTATT<br>CAGTTC<br>TCT	21	4	gp1a	......<br>.((((.<br>.((.((<br>((..((<br>((((((<br>(((.((<br>((((((<br>(((...	286.52	465.57	329.30	287.02	305.00	302.19

					((......))...)))))))))))).)))))))))))).)))).))))))....................						
gga-miR-101-1-5p_R-1	TCGGTTATCATGGTACCGGTGC	22	Z	gp1a	((.((((((.((((((((((((((..((((..((((((...........))))).)))).)))))))))))))).)))))))..))....	265.11	326.39	352.71	160.78	291.74	233.51

					.....						
gga-miR-26a-3p_R-1	CCTATTCTTGGTTACTTGCACT	22	2	gp1a	...(((((((.((..((.(((((((((.((((((((((((.......))).)))))))))).)))))).))..)).)))))))... .	223.95	205.90	268.70	246.53	215.36	260.98
gga-miR-181a-3p	ACCATCGACCGTTGATTGTACC	22	17	gp1a	...(((((((((((((((...(((.(((((((..((((((((.((((............	216.54	187.22	195.91	336.44	274.23	350.26

					...))))))))))))))).)))...))))))))))).))).))))...						
gga-let-7k-3p_1ss22CT	CTATACAATCTACTGTCTTTCT	22	26	gp1a	...(((((((((.(((..(((..(((((((((.((((((((((......))))......)).))))))))))))))))..)).))).))).))).)))))...	201.72	235.64	428.06	217.35	275.82	222.71

gga-miR-147_R+1	GTGTGC GGAAAT GCTTCT GCT	21	10	gp1a	...((. ((((((..(((. (((..(((.((. ((((((((.... ...... ((((.. ..)))))))))))).)).)))..))).))) ..))))))..)) ...	201.72	289.79	205.53	57.17	86.99	66.72
gga-miR-30c-2-3p_L+1	CTGGGA GAAGGC TGTTTA CTCT	22	3	gp1a	...(((((..((.(((.. ((..((...((((....(((((((.(((.. .(((((	181.13	188.36	215.47	271.54	250.36	233.51

gga-miR-133c-3p_1ss<br>22CT	TTGGTC<br>CCCTTC<br>AACCAG<br>CTGT	22	20	gp1a	((((((<br>....))<br>......<br>))))))<br>))).))<br>).))))<br>)))...<br>.)))).<br>))..))<br>.....)<br>)).)).<br>.)))))<br>...<br>...(((<br>((...(<br>(((.((<br>(((((.<br>.((.((<br>.(((((<br>(((...<br>......<br>...)))<br>))))).<br>)).)).<br>.)))))<br>)).)))	179.49	149.47	131.14	138.75	86.46	90.26

					)...))<br>)))...						
tgu-miR-2954-5p_L+1	TGCTGA<br>GAGGGC<br>TTGGGG<br>AGAGGA	24	Z	gp1a	(((((.<br>.((((.<br>.(((((<br>..((((<br>((..((<br>(..((.<br>.....)<br>)))).)<br>))))).<br>.)).))<br>)..)))<br>))))))<br>.....	177.84	210.48	160.32	259.63	254.61	206.04
gga-miR-99a-3p_R+2	CAAGCT<br>CGCTTC<br>TATGGG<br>TCTGT	23	1	gp1a	...(((<br>(((..(<br>(.((((<br>((((((<br>((((((<br>(.((((<br>(((((.<br>.(((.(<br>((((((<br>......<br>.....)	174.55	193.70	166.74	173.88	105.03	72.60

					)))))).)))..)))))))))).))))))))).))))).....))))..))......)))))))...						
gga-miR-29a-5p_L+1	ACTGATTTCTTTTGGTGTTCAGA	23	1	gp1a	...((((((((((((...(((((((.((((...........)))))))))))...)))))))))))))...	149.02	171.97	266.78	44.07	85.40	70.64
gga-miR-30a-3p_2ss10GA20AT	CTTTCAGTCAGATGTTTG	22	2	gp1a	....(((........)))	148.20	166.25	220.92	322.75	378.20	340.45

	CTGC				...(((<br>.(((((<br>((((..<br>.(((((<br>((((((<br>......<br>.....)<br>))))))<br>)))).)<br>))))))<br>)).)))<br>..						
gga-miR-135a-5p_R-1	TATGGC<br>TTTTTA<br>TTCCTA<br>TGTG	22	26	gp1a	...(((<br>((((((<br>((((((<br>((..((<br>(((..(<br>(((((.<br>......<br>))).))<br>)..)))<br>)).)))<br>))))))<br>))))))<br>))...	148.20	186.07	418.44	85.15	79.56	73.58
gga-miR-2131-5p	ATGCAG	24	Z	gp1a	((..(.	148.20	270.72	477.12	312.03	388.28	645.58

	AAGTGC<br>ACGGAA<br>ACAGCT				.(((((<br>(((.((<br>((((..<br>.((((.<br>((((((<br>(....(<br>((....<br>...)))<br>.)))))<br>)).)))<br>).))))<br>))))))<br>))))..<br>)..))						
gga-let-7f-3p_1ss22<br>CT	CTATAC<br>AATCTA<br>TTGCCT<br>TCCT	22	12	gp1a	...(((<br>(.((((<br>...(((<br>((((((<br>((((((<br>((((((<br>((((((<br>(((...<br>..))))<br>)))...<br>......<br>))))))	139.97	183.79	187.58	83.37	107.15	129.51

					)))))))))))))))))..))))))))...						
gga-miR-34a-3p_R-1	CAATCAGCAAGTATACTGCCCT	22	21	gp1a	...((((((((((..(((((..(((((((((((.(((((((((((...(((....)))......))))))))))))).))))))))..)))))..)).)))).....)))))...	136.67	172.35	219.32	258.44	403.13	386.56
gga-miR-214_R+2	ACAGCAGGCACA	23	chr8	gp1a	..(((...(((.	136.67	224.20	119.92	61.93	54.10	21.58

	GACAGG				..((.(						
	CAGAA				((((((						
					((.(((						
					...(((						
					((((((						
					(((...						
					......						
					....))						
					))))))						
					))))..						
					.))).)						
					))))))						
					)).)).						
					.)))..						
					)))..						
gga-miR-204	TTCCCT	22	Z	gp1a	...(((	135.03	357.66	147.50	225.09	401.01	2299.75
	TTGTCA				((...)						
	TCCTAT				))))..						
	GCCT				.(((((						
					...(((						
					((.(((						
					((((((						
					(.((((						
					(.((((						
					......						
					......						

					...))))))))).))))))))))).))))).)))))))..						
gga-miR-10a-3p_L+1R-1	CAAATTCGTATCTAGGGGAAT	21	27	gp1a	...((((..((((((((.((((.(((((.(((((((((((...............))))))))))).))))))))).))))))))).))))..	130.09	158.62	266.14	176.26	165.49	153.05
gga-miR-3538_R+3	GTTCGGTGATGAAACCAT	24	1	gp1a	....((((....))))..	126.79	15.25	28.86	86.94	238.69	107.92

	GGAATA				((((((<br>(...((<br>((((..<br>..))))<br>))..((<br>(.(((.<br>...)))<br>))).))<br>))))).						
gga-let-7k-3p	CTATAC<br>AATCTA<br>CTGTCT<br>TTCC	22	1	gp1a	...(..<br>(((..(<br>(.((((<br>((..((<br>(...(.<br>.(((((<br>((((((<br>((((.<br>((((.<br>....))<br>)))...<br>......<br>.)))))<br>))))))<br>))))).<br>.)..))<br>)..)))	124.32	134.98	262.29	105.99	119.35	111.85

					.))).)))))..)...					
gga-miR-1434_R+3	GTGCGTGATGATGGAAAATTTCT	23	28	gp1a	...(((().....))))).((((...((((..(((((((((((.((..((.......))..)).))))))))))...))))..)))))....	124.32	11.06	9.94	19.65	36.60	29.43
gga-miR-144-3p_L-1R-2	TACAGTATAGATGATGTAC	19	12	gp1a	...((....((((((((.......)).))))))..))...	123.50	199.04	178.28	348.95	356.45	504.30

gga-miR-1563_R+2	GCACAT<br>GATGAG<br>GAAGCA<br>CTGAAA<br>CT	26	12	gp1a	((((..<br>....((<br>(((...<br>.((((.<br>..((((<br>..((..<br>.(((((<br>(((((.<br>.....)<br>))))..<br>((((..<br>..))))<br>.)))))<br>...)).<br>.)))))<br>))))))<br>))))))	121.85	10.30	11.22	22.63	21.22	20.60
gga-miR-3523	CCGCGC<br>AGTGCC<br>TCGTCC<br>TCGA	22	13	gp1a	(((...<br>..))).<br>.....(<br>(....)<br>).((((<br>((((((<br>(((...<br>(((..(<br>((((((	116.91	129.64	51.94	225.09	221.72	219.77

					((((((((.((.(((....)))))).)).))))))))))))..))).......((..................))))))))))))))))					
gga-miR-34b-5p_L-1R+1	AGGCAGTGTAGTTAGCTGATTGT	23	24	gp1a	...(((.....(((.(((.(((((((..(((((.(((.((((((.(((((.(((((((((....))..)	115.27	30.50	14.11	17.86	23.34	31.40

					)))))))))).)))).)).))))..))))))))))))..)))...						
gga-miR-1784-5p	TTCTGCTCTTATTGAAATCAGT	22	3	gp1a	...((((((((((.(((((((((((((((((((.((.((((((((....))..))))))).)).)))))))))))))))).)))))))))...	107.03	56.05	81.12	1.19	2.12	1.96
tgu-miR-7-1-3p_R+1	CAACAAATCACA	22	Z	gp1a	......((((((	103.74	179.97	212.27	54.78	66.83	58.87

	GTCTGC				((....						
	CATA				...(((						
					...)))						
					...((.						
					...(((						
					(((.((						
					.(..((						
					(((.((						
					(((.((						
					((.(((						
					((((((						
					(((((.						
					...)))						
					))..))						
					))))))						
					))))))						
					)))).)						
					))))..						
					).))))						
					))))..						
					))))))						
					))))..						
					..						
gga-miR-15b-5p_L-1R	AGCAGC	22	chr9	gp1a	.....(	102.51	157.76	142.99	127.02	169.83	124.72
+1	ACATCA				((((((						
	TGGTTT				...((.						

	GCAA				.(((((<br>((.((.<br>((((((<br>(((((.<br>((....<br>...)))<br>))))))<br>)))).)<br>).))))<br>)))..)<br>)...))<br>))))).<br>.						
gga-miR-130a-5p	GCCCTT<br>TTTCTG<br>TTGTAC<br>TACT	22	15	gp1a	.(((((<br>((((((<br>(((((.<br>..(((.<br>.(((((<br>(((..(<br>((((((<br>((.((.<br>.((...<br>.))...<br>)).)))<br>))))))<br>..))))	98.80	160.91	153.27	151.85	112.45	187.39

					))))..						
					)))...						
					))))..						
					.)))).						
					....))						
					)))).)						
					)						
gga-miR-130c-5p_1ss	GCCCTT	22	19	gp1a	......	98.80	160.91	153.27	151.85	112.45	187.39
10AC	TTTCTG				..((.(						
	TTGTAC				((.(((						
	TACT				..((((						
					((((.(						
					..((((						
					(((.((						
					.(((..						
					....))						
					)..)).						
					))))))						
					)..).)						
					))))))						
					)..)))						
					.)))))						
					..						
gga-let-7a-5p_R+2_2	TGAGGT	24	chr26	gp1a	..((((	97.15	120.49	228.94	58.36	83.81	41.21
	AGTAGG				((((((						
	TTGTAT				..((((						

	AGTTAG				(((((.						
					..((..						
					((((((						
					...)))						
					)).)..						
					))....						
					..))))						
					))))).						
					.)))))						
					))))).						
					.....						
gga-let-7a-5p_R+2_1	TGAGGT	24	24	gp1a	...(((	97.15	120.49	228.94	58.36	83.81	41.21
	AGTAGG				.(((((						
	TTGTAT				((((..						
	AGTTAA				(((((.						
					.((((.						
					..((((						
					(((..(						
					((.(((						
					.(((((						
					((((((						
					((....						
					......						
					......						
					.....)						
					))))))						

					)))))).))).)))..)))))))..))))))))))..)).))).)))).))).						
gga-miR-222b-3p_R+1	AGCTACATCTGATTACTGGGTCACT	25	4	gp1a	...(((.((.((((..((((((((((((((((((..((((.......................))))))))).))))))))))))..))).)))))...	97.15	153.28	73.11	46.45	47.74	41.21
gga-miR-1451-3p_R-1	CGTAAC	22	3	gp1a	...(((	97.15	94.56	85.29	169.11	188.83	186.41

	TCGCTG				(((((.						
	CTGTGA				((((((						
	GAGG				((((((						
					((((.(						
					((((.(						
					((.(((						
					(((.((						
					(.....						
					......						
					))).))						
					)))).)						
					)).)))						
					)).)))						
					))))))						
					))))))						
					))))..						
					...)))						
					))...						
gga-miR-153-3p_R+2	TTGCAT	22	2	gp1a	.((((.	95.51	106.76	237.92	139.34	221.72	166.79
	AGTCAC				.....(						
	AAAAGT				(((((.						
	GATC				((((((						
					((((((						
					((((((						
					(..(((						
					((((.(						

					(.....<br>..))..<br>))))))<br>)..)))<br>))))))<br>)))).)<br>))))).<br>))))))<br>......<br>)))).						
gga-miR-187-3p_R+3	TCGTGT<br>CTTGTG<br>TTGCAG<br>CCAGT	23	2	gp1a	......<br>.((((.<br>..((((<br>.((((.<br>((((((<br>((((((<br>((((..<br>......<br>......<br>))))))<br>))))))<br>)))).)<br>))).))<br>))..))<br>))...	95.51	114.39	57.07	35.73	31.83	115.77
gga-miR-1329-5p	TACAGT	22	3	gp1a	...(((	87.27	109.05	81.44	47.64	65.77	27.47

	GATCAC				..(...					
	GTTACG				.((..(					
	ATGG				((.(((					
					(..(((					
					((((.(					
					((((((					
					.((...					
					......					
					......					
					.)))))					
					)))).)					
					))))))					
					..))))					
					.))))..					
					)))..)					
					))...					
gga-miR-190a-3p_L+1	ACTATA	22	10	gp1a	......	79.04	103.33	204.57	102.42	152.76	143.24
	TATCAA				((((((					
	ACATAT				(.((((					
	TCCT				(.((((					
					((((((					
					((((((					
					..((((					
					(.....					
					.....)					
					))))))					

					))))))						
					))))))						
					))..))						
					))).))						
					))))).						
					......						
					...						
gga-let-7g-3p_1ss22	CTGTAC	22	12	gp1a	......	75.75	78.55	75.67	54.78	97.60	119.70
CT	AGGCCA				(((((.						
	CTGCCT				((((((						
	TGCT				((.(((						
					(((((.						
					(..(((						
					((((((						
					.....(						
					(((.((						
					.((((.						
					...)))						
					)))..)						
					))))))						
					))))))						
					..).))						
					))))))						
					))))))						
					))))).						
					))...						

gga-miR-1663-3p	TGGCATCCAGAACAGCGGTAC	21	5	gp1a	......((((((..(((((((((.((((.(((((((((..(((.......)))...))))))))))).)))).)))))))))..)))))).....	74.10	48.81	37.19	25.01	39.25	39.24
gga-miR-1729-3p	CTACTCGGTGAGTAAGGATAGC	22	15	gp1a	...(((((...((((..(((((((((.(((((((((.((((((((((....(((((....)	74.10	67.87	65.41	69.08	76.38	78.49

gga-miR-7b_R+3	TGGAAG<br>ACTAGT<br>GATTTT<br>TGTTGT<br>T	25	JH3762<br>10.1	gp1a	))))))<br>))))))<br>))))))<br>))))))<br>.)))))<br>))..))<br>.)).))<br>)))...<br>...(((<br>((...(<br>(((((.<br>((.(..<br>(((((.<br>(((((.<br>(((((.<br>.(((((<br>((((((<br>((....<br>))))).<br>.)))))<br>))))))<br>))))))<br>).))))<br>)..).)<br>))))))<br>)....)	68.34	89.22	168.66	1113.54	2554.03	1073.35

					)))).......						
gga-miR-3525	CAGCCATTCTGCGATTCTGTGA	22	chr1	gp1a	((((((..((((((..((((((((..((((((.((((.........)))).))))))..))))))))..))))))..))))))	67.51	73.21	44.25	48.83	58.88	41.21
gga-miR-2188-3p_L-1R+1	ATATATGTGGTCAGACCTATCT	22	22	gp1a	....(((((((.(((.((((((((.(((((..(((.((.(((((((............	64.22	120.49	125.69	105.99	101.84	78.49

					.)))))<br>)).)).<br>)))..)<br>)))).)<br>))))))<br>))..))<br>))))))<br>))...						
gga-miR-1699_L-1R+1	CAGAGG<br>GACATG<br>GCAGGG<br>CAAT	22	11	gp1a	...(((<br>(.((.(<br>(((..(<br>((((.(<br>(....(<br>(....)<br>)..)).<br>))))).<br>.)))).<br>))))))<br>.....	60.10	80.45	35.27	113.14	69.49	61.81
gga-miR-1416-5p	TCCTTA<br>ACTCAT<br>GCCGCT<br>GTG	21	chrZ	gp1a	.(.(((<br>((((((<br>(((...<br>((((((<br>((((((<br>...(((<br>(....(	59.28	35.08	31.42	53.59	35.01	29.43

					(.....<br>))...)<br>)))...<br>))))))<br>))))))<br>...)))<br>))))))<br>)))).						
gga-miR-3536_R+1	CTGCAT<br>ACGAGT<br>AGACCC<br>TTTCT	23	25	gp1a	...(((<br>.(((((<br>((((((<br>.((...<br>((((((<br>..((((<br>......<br>..))))<br>..))))<br>))...)<br>)..)))<br>))))).<br>..))).<br>)))...	59.28	7.63	8.34	23.82	24.40	13.74
gga-miR-200b-5p_L+2	CATCTT<br>ACTGGG<br>CAGCAT<br>TGGA	22	21	gp1a	..((((<br>(..(((<br>(...((<br>((((((	57.63	44.23	64.77	20.25	18.03	41.21

gga-miR-456-3p_R-1	CAGGCT<br>GGTTAG<br>ATGGTT<br>GTC	21	2	gp1a	..((((<br>(.((((<br>((....<br>......<br>...)))<br>))).))<br>)))..)<br>))).))<br>))...)<br>)))..)<br>))))..<br>...((.<br>.(((((<br>..((..<br>..(((.<br>......<br>.)))..<br>......<br>...(((<br>(...((<br>(((...<br>...)))<br>))..))<br>))....<br>..))..<br>))))).	55.16	58.34	37.52	167.33	159.66	166.79

					.))...					
gga-miR-106-3p_R+1	ACTGCAGTATAAGCACTTCTGGC	23	4	gp1a	.(.((..(((..(((((((((...(((((.(((.((...)).)))))))))...))))))))))...)))..)))..	54.34	57.19	63.81	307.26	285.90	288.45
tgu-miR-9-3p	ATAAAGCTAGATAACCGAAAGT	22	Z	gp1a	.....((((.(((((.(((..((((((((((((((.(((((((..(....)..)))))).))))))))))))	51.05	44.23	48.74	39.30	31.83	29.43

					)))..)<br>)).)))<br>))))))<br>......						
gga-mir-3528-p3_2	CTGTAG<br>TGCCAT<br>GTTTGT<br>G	19	Z	gp1a	...(((<br>((.(((<br>.....)<br>))))))<br>).....<br>..	49.40	1.53	2.57	19.65	13.26	11.77
gga-mir-3528-p3_3	GCAGAA<br>AGGAAA<br>AGGCTG<br>TGTCT	23	17	gp1a	...(((<br>.(((((<br>(....(<br>((..((<br>((((((<br>(((...<br>...)))<br>))))))<br>))..))<br>)..)).<br>))))..<br>..)))(<br>(((((.<br>..))))<br>))..	49.40	1.53	2.57	19.65	13.26	11.77
gga-mir-3528-p3_1	AAAGGA	19	4	gp1a	(((.((	49.40	1.53	2.57	19.65	13.26	11.77

	AAAGGC<br>TGTGTC<br>T				((((((<br>.((((.<br>......<br>......<br>((((((<br>((....<br>((..((<br>(((.((<br>((....<br>.....)<br>))))))<br>))..))<br>))))))<br>))))))<br>))).))<br>))).))<br>)...						
gga-miR-148a-5p	AAAGTT<br>CTGTGA<br>CACTCA<br>GACT	22	2	gp1a	.(((((<br>(((.((<br>..((((<br>((((((<br>((.(((<br>(..(((<br>((((..<br>.....)<br>)).)))	47.75	54.14	66.05	202.46	431.77	437.58

					))))).					
					..))))					
					))))))					
					))..))					
					.)))))					
					))).					
gga-miR-1786	ATTCTT	21	14	gp1a	...(((	47.75	42.71	72.47	92.30	77.44	134.41
	TTCTGC				(....(						
	TGTGTT				((.(((						
	ACT				..(((.						
					(((((.						
					((((((						
					..((((						
					(((.((						
					(((((.						
					....((						
					(.....						
					......						
					)))...						
					))))))						
					).))))						
					)))..)						
					))))).						
					))))).						
					.))))..						
					))))))						

					..))))<br>...						
gga-miR-1805-3p_R+1	TGTATT<br>GGAACA<br>CTACAG<br>CTCC	22	1	gp1a	...(((<br>((((((<br>((....<br>...(((<br>(..(((<br>.(((((<br>((((((<br>(.((((<br>((((((<br>.....(<br>(.....<br>.))...<br>)))).)<br>))))).<br>))))))<br>))))))<br>.))).)<br>))))))<br>))).))<br>)))...	47.75	52.62	73.11	85.75	136.85	102.04
gga-miR-6553-3p_L+1	AAACAC<br>TACCCT<br>GAGCTG<br>CTCC	22	1	gp1a	......<br>..((((<br>((.(((<br>(((.((	47.75	77.02	36.87	74.43	53.04	64.75

					(((.........))))).((.(((......))).))....)))).))))))))).						
gga-miR-130b-5p_R-1	CCTCTTTCCCTGTTGCACTAC	21	2	gp1a	((.(((((.....(((((((.....))))))))))).))	46.93	57.96	39.12	176.26	141.63	164.83
gga-miR-3064-5p_L-2R+2	GCTGTTGTGGTGTGCAAAACT	21	18	gp1a	..............(((((((.(((((..(((((..((((((((((....((.....)).	44.46	1.14	0	10.12	18.57	19.62

					)))))).<br>)))))).<br>.)))))<br>.....)<br>))))..<br>.)))))<br>))....<br>......<br>......<br>.....						
gga-miR-551-3p_R+1	GCGACC<br>CATACT<br>TGGTTT<br>CAGT	22	9	gp1a	...(((<br>((((..<br>(((((.<br>((((((<br>(((..(<br>((((..<br>...(((<br>((((..<br>...)).<br>))))).<br>.)))))<br>..))))<br>))))).<br>))))).<br>.)).))<br>)))...	42.81	83.12	81.44	125.05	133.67	111.85

gga-miR-101-3p_L-1R+2	TACAGTACTGTGATAACTGAAGG	23	Z	gp1a	...(((.((((((.(((((((((((((((..((((..(((((...........)))))).))))..)))))))))))))).))))))..)))...	41.99	30.50	58.68	42.28	46.15	42.19
gga-miR-16-1-3p_R-1	CCAGTATTAACTGTGCTGCTGA	22	1	gp1a	...((..(((.((((..(((((((((.((((((((....................))))	39.52	22.88	36.55	59.55	73.20	74.57

					))))．)<br>)．))))<br>))))．．<br>))))．)<br>))．．))<br>．．．						
gga-miR-200a-5p	CATCTT<br>ACTAGA<br>CAGTGC<br>TGGA	22	21	gp1a	．．．(((<br>((((((<br>．(．．((<br>((((((<br>((((((<br>((((．(<br>(((．．．<br>．．．．．．<br>．．．．．)<br>)))．))<br>))))))<br>)))))．<br>)))))．<br>．)．)))<br>)．))．)<br>))．．．	39.52	51.09	67.98	32.16	30.77	35.32
gga-mir-3538-p3	GCTGGG<br>CACAGC<br>GCCTGA<br>CATCC	23	1	gp1a	．．．．((<br>((．．．．<br>))))．．<br>((((((	39.52	0	0	4.76	9.02	1.96

gga-let-7c-3p_1ss12TC	CTGTACAACCTCCTAGCTTTCC	22	1	gp1a	(...((((((....))))))..(((.(((....))))).))))))....((.((((..(((((((((.(((..(..(((.(((.((((((((..(((..((.(..(.....)..).)))))..)))))))).))).)))..)..)))))))))).	37.87	54.14	60.28	82.18	95.48	100.07

					..))).						
					.)))))						
					)...						
gga-miR-6516-3p_L+1	TCATGT	24	18	gp1a	((((..	37.87	9.15	7.05	20.25	31.83	13.74
	ATGATA				(((...						
	CTGCAC				..))).						
	ACAACG				.)))).						
					..((((						
					(((.((						
					...(((						
					(.((((						
					.(((..						
					.)))..						
					.)))).						
					))))..						
					.)).))						
					))))).						
					......						
					...						
gga-miR-138-5p_R+7	AGCTGG	24	11	gp1a	...(((	37.05	47.28	40.08	299.52	265.75	212.90
	TGTTGT				(.((.(						
	GAATCA				((..(.						
	GGCCGT				.(((((						
					((.(((						
					((...(						
					((((..						

					(((....)))..........))))..)))).))))))).)..))).))))))...						
gga-miR-203a	GTGAAATGTTTAGGACCACTTG	22	5	gp1a	...(((..(((((..((((..(((((((((.((((.((((.(((((........)))))))))).))).)))))))))..)))..))))..))	36.23	38.89	39.76	17.86	24.40	102.04

					)...						
gga-miR-223_R-2	TGTCAG<br>TTTGTC<br>AAATAC<br>C	19	1	gp1a	...(((<br>(((...<br>......<br>))))))<br>...	36.23	47.66	54.19	23.82	39.25	24.53
gga-miR-1618-5p	TGCATC<br>CTGGCT<br>CCTGGC<br>GTCC	22	8	gp1a	.((.((<br>(.((((<br>(((((.<br>(((.((<br>(.((..<br>..))..<br>..))).<br>.))).)<br>))))))<br>)).)))<br>.))...	36.23	44.23	0.64	28.58	26.52	0
gga-miR-499-5p_R-2	TTAAGA<br>CTTGTA<br>GTGATG<br>TTT	21	20	gp1a	((..((<br>((((..<br>((((((<br>(((((.<br>.(((..<br>..))).<br>.)))))<br>))))))<br>..))))	34.58	22.12	32.06	756.25	167.62	104.00

					))..))<br>...						
gga-miR-1781-3p	TTTAAA<br>TCATGC<br>AGCTGT<br>TGA	21	14	gp1a	...(((<br>(.(((.<br>((((.(<br>((((((<br>((((((<br>..((((<br>((((((<br>(...((<br>((....<br>))))))<br>))))))<br>)))..)<br>))))))<br>))))))<br>.)))))<br>))))))<br>...	34.58	56.43	53.87	33.35	31.83	19.62
gga-miR-1662_R-3	TTGACA<br>TCATCA<br>TACTTG<br>G	19	1	gp1a	......<br>......<br>.((((.<br>((((..<br>....))<br>))..))<br>))...	33.76	27.45	20.20	6.55	7.96	9.81

gga-miR-460b-3p_R-3_1ss11GA	CACAGCGCATACAATGTGGA	20	4	gp1a	..(((..(((((((((...(((((.(((((((..((((((((.............))))))))))..))))))))).)))))...))))))).))))))	32.93	38.13	36.55	48.83	38.19	31.40
gga-miR-1306-5p_L-1R-3	CCACCTCCCCTGCAAACGTCC	21	15	gp1a	...(((....((..(((((((......((.((((((((...((......))..))))))))	31.29	39.27	13.47	44.07	35.01	35.32

					).))..<br>.)))))<br>))..))<br>....))<br>)...						
gga-miR-1552-3p_R+1	CTAGCT<br>GCTCTG<br>CACTGA<br>CTGT	22		gp1a	((((((<br>.((((.<br>.(((((<br>((((..<br>(((.((<br>(((.((<br>((....<br>.....)<br>))).))<br>))))))<br>..))))<br>))))).<br>.)))))<br>)))))	29.64	24.40	28.22	77.41	62.59	51.02
gga-miR-1306-3p_R-1	TGGACG<br>TTGGCT<br>CTGGTG<br>GTGA	22	15	gp1a	...(((<br>....((<br>..((((<br>(((...<br>...((.<br>((((((<br>((...(	27.99	23.64	25.33	63.12	82.22	53.96

					(.....<br>.))..)<br>))))))<br>).))..<br>.)))))<br>))..))<br>....))<br>)...						
gga-miR-2131-3p_R+1	CTGTTA<br>CTGTTC<br>TTCTGA<br>TGGT	22	Z	gp1a	((..(.<br>.(((((<br>(((.((<br>((((..<br>.((((.<br>((((((<br>(....(<br>((....<br>...)))<br>.)))))<br>)).)))<br>).))))<br>))))))<br>))))..<br>)..))	27.99	38.13	41.04	65.50	80.63	66.72
gga-miR-27b-5p_R-1	AGAGCT<br>TAGCTG<br>ATTGGT	22	Z	gp1a	.(((((<br>(((...<br>.(((((	26.35	20.59	19.88	45.26	36.07	29.43

	GAAC				((((((<br>((((((<br>.((((.<br>((((((<br>(.....<br>......<br>....))<br>))))))<br>))))))<br>))))))<br>))))))<br>))..))<br>))).))<br>)..						
gga-miR-301a-5p_L+2	GCTCTG<br>ACAATG<br>TTGCAC<br>TACT	22	15	gp1a	..((((<br>(.((((<br>...(((<br>.(((((<br>((((((<br>((((.(<br>(.....<br>......<br>...)).<br>))))))<br>...)))<br>))))))	26.35	44.23	33.99	226.28	214.29	233.51

					.)))..<br>.)))).<br>))))).<br>.						
gga-miR-301a-3p_R+1	CAGTGC<br>AATAAT<br>ATTGTC<br>AAAGCA<br>TT	26	15	gp1a	..((((<br>(.((((<br>...(((<br>.(((((<br>((((((<br>((((.(<br>(.....<br>......<br>...)).<br>))))))<br>...)))<br>))))))<br>.)))..<br>.)))).<br>))))).<br>.	26.35	49.19	107.42	70.27	209.52	149.13
gga-miR-92-5p	AGGTTG<br>GGATCA<br>GTTGCA<br>ATGCT	23	1	gp1a	..((((<br>(..(((<br>(..((.<br>((((((<br>((..(.<br>......	24.70	24.40	21.80	73.84	57.29	72.60

					.)..))<br>))))))<br>))..))<br>))..))<br>)))...<br>.						
gga-miR-107-5p_R+1	AGCTTC<br>TTTACA<br>GTGTTG<br>CCTTGT	24	6	gp1a	...(((<br>((..((<br>((((.(<br>((.(((<br>.(((((<br>((((((<br>.(((..<br>......<br>....))<br>))))))<br>))))))<br>.)))))<br>)...))<br>))))..<br>))))).<br>..	24.70	16.78	27.58	71.46	110.33	105.96
gga-miR-190a-5p_R+2<br>_2ss15T-16A-	TGATAT<br>GTTTGA<br>TATTAG<br>GTTG	22	25	gp1a	...(((<br>(((((.<br>((((..<br>((((((	24.70	17.92	33.99	22.63	18.03	19.62

gga-miR-383-5p	AGATCAGAAGGTGATTGTGGCT	22	4	gp1a	(((((((((..(((((((......))).))))..))))))))))))).)..)))).)))))))).......(((((..((......((((((.(((((.((.((((((..((((.(((((((((..(......)..)).))))))))))))	24.70	67.87	48.10	38.11	84.87	52.98

					..))))						
					)).)).						
					.)))))						
					.))).)						
					))....						
					.))..)						
					))))..						
					.						
gga-miR-3607-3p_R-4	CTGTAA	19	Z	gp1a	.(..((	24.70	3.81	5.13	9.53	19.10	11.77
	ACGCTT				(.....						
	TCTGAT				.(((..						
	G				...(((						
					((((((						
					.....(						
					((((((						
					((((((						
					(.(((.						
					.....)						
					)).)))						
					))))))						
					).))))						
					....))						
					))))))						
					)...))						
					)..)))						
					..)...						

gga-miR-7_R+3	TGGAAG<br>ACTAGT<br>GATTTT<br>GTTGTT<br>A	25	10	gp1a	...(((<br>(((.((<br>(.(((.<br>.(..((<br>(((..(<br>((....<br>.....(<br>((((..<br>((((((<br>(..((.<br>(((((.<br>((((.(<br>((((((<br>(.((..<br>((....<br>..))))<br>.)))))<br>))))))<br>))))))<br>.))..)<br>))))))<br>.)))))<br>)))..)<br>))))..<br>).))).<br>))).))	23.88	41.94	109.34	13.70	19.63	16.68

					)).))						
gga-miR-19b-5p	AGTTTTGCAGGTTTGCATCCAGC	23	chr1	gp1a	.(((((((((((((((((((((..((((..(((...)))..))))))))))).)).)))))))))))))..	21.41	38.89	48.10	59.55	71.61	57.89
gga-miR-137-3p_L+1	TTATTGCTTAAGAATACGCGTAG	23	8	gp1a	.......((((((((((..(((.((((((((..(..(.(((((((.....)).))))))..)..))))))).))).	21.41	19.83	28.86	53.59	53.04	37.28

					.))))))))))))...						
gga-mir-1662-p3	CCCAAGCAGGCTGATGTCAGGC	22	2	gp1a	((.((((..(((((((((...((((((((......... ...))))))))..)).))))))))..))))))).	21.41	27.45	18.60	28.58	27.58	58.87
gga-miR-29b-2-5p_L-2	CTGGTTTCACATGGTGGCTTAGA	23	26	gp1a	...((((.(((((((((((((((((.((((((.((.((((.................))))))))))))	19.76	46.52	42.97	3.57	0	1.96

					.)))))<br>))))).<br>))))))<br>.)).))<br>))...						
gga-mir-147-p5	TAGTGG<br>AATCAC<br>TTCTGC<br>ACA	21	10	gp1a	...((.<br>((((((<br>..(((.<br>(((..(<br>((.((.<br>((((((<br>((....<br>......<br>((((..<br>..))))<br>))))))<br>)).)).<br>)))..)<br>)).)))<br>..))))<br>))..))<br>...	19.76	22.12	11.54	2.38	2.12	3.92
gga-mir-147-p3_1	TAGTGG<br>AATCAC<br>TTCTGC<br>ACA	21	2	gp1a	...(((<br>((..((<br>.....(<br>((((..	19.76	22.12	11.54	2.38	2.12	3.92

gga-mir-1563-p3	ATTCAA<br>TCCAAT<br>GCTGAG<br>C	19	12	gp1a	..))))<br>)(((((<br>..((((<br>...(((<br>(....)<br>)))...<br>((((..<br>..))))<br>))))..<br>))))).<br>....))<br>..))))<br>)...<br>((((..<br>....((<br>(((...<br>.((((.<br>..((((<br>..((..<br>.(((((<br>(((((.<br>.....)<br>))))..<br>((((..<br>..))))<br>.)))))	19.76	4.96	3.21	5.36	7.43	9.81

					...)).<br>.)))))<br>))))))<br>))))))						
gga-mir-1563-p5_1	ATTCAA<br>TCCAAT<br>GCTGAG<br>C	19	11	gp1a	......<br>((((.(<br>((...)<br>)).)))<br>)...	19.76	4.96	3.21	5.36	7.43	9.81
gga-mir-6582-p5	TCACTC<br>TGGGTG<br>TTTCTC<br>TTGC	22	3	gp1a	..(((.<br>.(((((<br>((((((<br>((.((.<br>((((..<br>(.....<br>.)..))<br>)).)).<br>))))))<br>))))))<br>)..)))<br>...((.<br>.(((.(<br>(.((..<br>....))<br>.)))))<br>..))..	19.76	19.06	18.60	15.48	31.83	25.51

gga-miR-34b-3p	AATCAC<br>TAAATT<br>CACTGC<br>CATC	22	24	gp1a	.....<br>...(((<br>(((..(<br>(((..(<br>((((((<br>...(((<br>(.((((<br>(((...<br>......<br>...)))<br>))))))<br>))...)<br>))))))<br>..))))<br>...)))<br>)))...	18.11	3.05	1.92	2.38	3.18	7.85
gga-miR-214_L+1R-3	TACAGC<br>AGGCAC<br>AGACAG<br>G	19	14	gp1a	......<br>...(((<br>(((.((<br>..(((.<br>..(...<br>..)..)<br>))..))<br>))))))<br>...	17.29	12.20	5.77	7.15	5.30	5.89
gga-miR-100-3p_L+1	CAAGCT	22	24	gp1a	...(((	16.47	15.63	28.22	22.63	37.13	27.47

	TGTATC<br>TATAGG<br>TATG				(.(((.<br>.(((((<br>.(((.(<br>(((((.<br>......<br>....))<br>)))).)<br>)).)))<br>))..))<br>).))))<br>......<br>.						
gga-miR-1467-3p_L+1	TTCACA<br>CCAGAG<br>TAACTG<br>GGATC	23	2	gp1a	...(((<br>(.((.(<br>(((((.<br>...(((<br>(((.((<br>(.((((<br>(((.((<br>(((((.<br>......<br>...)))<br>.)))).<br>))))).<br>))))).<br>))))))	16.47	17.16	8.34	41.68	73.73	76.53

					....))						
					)))).)						
					).....						
					.)))).						
					..						
gga-miR-1560-3p_R+1	GCATCT	23	11	gp1a	.(((((	16.47	9.15	19.88	25.01	24.40	21.58
	CTGGAC				..((..						
	GCGCTC				.((((.						
	GTTCT				((((..						
					((((((						
					(((...						
					......						
					.)))))						
					))))..						
					))))..						
					))))..						
					.))..)						
					)).)).						
					......						
					...						
gga-miR-1677-3p_L+1	TTGACT	23	3	gp1a	((.((.	16.47	10.68	22.45	71.46	37.13	25.51
	TCAGTA				.(((((						
	GGAGCA				((.(((						
	GGATT				((((((						
					((((((						
					(.....						

					...))))))))))))).)).)))))))..)).))						
gga-miR-3539	CAGAGGAAGACTGATGCTAGTT	22	1	gp1a	...((((((.((((((.((.(((((..((.....)).))))))).))).))).))))))...	16.47	6.86	8.98	2.38	13.79	7.85
gga-miR-146c-5p_L-1	GAGAACTGAATTCCATGGACTG	22	4	gp1a	...((((((.((((.(((((((((((.(((((((((....((......))...	15.64	27.45	24.05	25.01	42.96	36.30

					.)))))<br>))))).<br>))))))<br>))))).<br>.)))).<br>))..))<br>))...						
gga-miR-222b-5p_R-4	TGCTCA<br>GTAGTC<br>AGTGTA<br>GGAT	22	4	gp1a	...(((<br>.((.((<br>((..((<br>((((((<br>((((((<br>((((..<br>.((((.<br>......<br>......<br>..))))<br>))))).<br>))))))<br>))))))<br>)..)))<br>).))))<br>)...	14.82	19.83	11.54	3.57	8.49	5.89
gga-mir-1636-p5	TGCCCC<br>GTCACT<br>CCTGAT	22	15	gp1a	...(((<br>((((.(<br>(((.((	14.82	9.15	3.85	3.57	1.06	3.92

	ACCG				.(((((<br>((((..<br>((((.(<br>((((((<br>.....)<br>))..))<br>))))))<br>...)))<br>))))))<br>.)).))<br>))....<br>))))))<br>)...						
gga-mir-3538-p5_1	ATCCGT<br>GAGGAG<br>TCGTTC<br>CATGCT<br>GC	26	1	gp1a	((....<br>(((.((<br>(....)<br>))))).<br>..((((<br>(((..(<br>(((((.<br>(((...<br>......<br>.(((..<br>..))))<br>))..))<br>))))..	14.82	1.53	0	0	2.12	1.96

					)))))))))..						
gga-mir-3538-p5_2	GCTGGGCACAGCGCCTGAC	19	11	gp1a	((.((.(((((((((.(((.((((((..((((..((((.........))))......))))..)))).))).)))......)))))))))).)))).	14.82	1.53	0	0	2.12	1.96
gga-mir-147-p3_2	TGGAATCACTTCTGCACAA	19	2	gp1a	...((((((((.....))))))).....	13.17	9.91	6.41	1.19	1.06	1.96
gga-miR-184-3p	TGGACGGAGAACTGATAA	22	10	gp1a	((.((((((((.(((.(((	13.17	48.04	28.22	34.54	38.19	115.77

	GGGT				((((((<br>.(((((<br>(...((<br>(((.((<br>......<br>..))..<br>))))).<br>.)))))<br>).))))<br>))))).<br>))).))<br>))))))<br>).						
gga-miR-216a_R+1	TAATCT<br>CAGCTG<br>GCAACT<br>GTGA	22	3	gp1a	......<br>.(((..<br>((((((<br>..((((<br>(.((((<br>(((((.<br>..((.(<br>((((((<br>(.((((<br>...)))<br>))))))<br>))).))<br>..))))	13.17	5.34	2.57	7.15	12.73	5.89

					))))).)))))).))))))...)))...						
gga-miR-1651-3p_R+1	TTGCTTTTGTTGGCCTCTGCTGT	23	1	gp1a	...((((((((..(((.(((..((.(..(((((((((((((...........))))))))))))..).))..))).)))..)))))))...	13.17	21.35	19.88	11.91	12.73	15.70
gga-miR-1744-3p	ACTTCAACAGGAGCAAGACTGA	22	10	gp1a	...((((..((((((((((.((((.(((......	13.17	0.76	0.64	17.86	9.55	0

					..))).<br>)))).)<br>))))))<br>).))))<br>..))))<br>......<br>......<br>..						
gga-miR-3528_R+1	CATGCC<br>CCAGTC<br>GTGTTG<br>CAGAT	23	17	gp1a	...(((<br>.(((((<br>(....(<br>((..((<br>((((((<br>(((...<br>...)))<br>))))))<br>))..))<br>)..)).<br>))))..<br>..)))(<br>(((((.<br>..))))<br>))..	13.17	6.86	3.21	7.15	15.91	9.81
gga-miR-20b-3p_R-1	ACTGTA<br>ATGTGG<br>GCACTT	21	4	gp1a	(((.((<br>.(((.(<br>((((((	11.53	10.68	6.41	34.54	42.43	23.55

	ACA				(((..(<br>((((((<br>((((.(<br>((((.(<br>((.((.<br>......<br>)).)))<br>))))).<br>))))))<br>))))).<br>..))))<br>))))))<br>))).))<br>))).						
gga-miR-24-5p	GTGCCT<br>ACTGAG<br>CTGATA<br>TCAGT	23	Z	gp1a	......<br>((((((<br>(.((((<br>(...((<br>(.....<br>(((((.<br>...)))<br>))..))<br>)..(((<br>.(((((<br>((((..<br>(((((.	11.53	19.83	18.60	17.86	20.16	15.70

					.((......)).))))).)))))))))).)))))))))).))..))))...						
gga-miR-29c-3p_L+1R +1_1ss19GA	CTAGCACCATTTGAAATCAGTT	22	26	gp1a	...((((.(((((((((((((((((.((((((.((.((((.............))))))))))))).)))))))))).))))))).)).))))...	11.53	17.16	12.51	1.79	3.71	5.89
gga-miR-135a-2-3p_L	TGTAGG	22	1	gp1a	......	11.53	16.01	18.60	9.53	4.24	3.92

-1R+1	GATGGA<br>AGCCAT<br>GAAT				.(((((<br>....((<br>((((((<br>((((.<br>(..(((<br>((((..<br>(((...<br>......<br>....))<br>)..)))<br>))))..<br>))))))<br>))))))<br>))...)<br>))))..<br>.						
gga-miR-196-5p_R+2	TAGGTA<br>GTTTCA<br>TGTTGT<br>TGGGT	23	JH3755<br>93.1	gp1a	......<br>..((((<br>(((..(<br>(..((.<br>((((((<br>((((.(<br>((((..<br>(((.((<br>(.....<br>))))))	11.53	12.96	17.31	3.57	6.37	0

					..))))<br>).))))<br>))))))<br>.))..)<br>))))))<br>)).(((<br>((....<br>.)))))<br>.						
gga-miR-429-5p_L-1	GTCTTA<br>CCAGGC<br>AAAGTT<br>AGA	21	21	gp1a	...((.<br>(((((.<br>((((((<br>.(..((<br>((((((<br>((..((<br>(((((.<br>.((...<br>...)))<br>))))))<br>..))))<br>))))))<br>..).).<br>))))))<br>)).)))<br>)...	11.53	10.68	22.45	32.16	46.68	52.98
gga-miR-1456-3p_R+1	CGGGGT	22	Z	gp1a	.((((.	11.53	8.39	3.21	16.67	7.43	7.85

	CGTCGC				.((...						
	CGTCCC				.((((.						
	TTCC				(((((.						
					(((((.						
					((((((						
					.(((((						
					(((.((						
					(..((.						
					...)).						
					).))))						
					))))))						
					.)))))						
					).))))						
					).))))						
					).))))						
					...)).						
					.)))).						
					..						
gga-miR-1559-3p	AGTTAC	22	chr7	gp1a	(((.((	11.53	8.39	10.90	25.01	27.58	29.43
	ATGTAT				(..(((						
	GCATCG				(((((.						
	AGCA				(..(((						
					..((((						
					(.....						
					......						
					...)))						

					))..)))..).))))))))..))).)))						
gga-miR-1618-3p_L+1	AGAGCCCGGAGCCAGGCTGCTGT	23	8	gp1a	.((.(((.(((((((((.(((.(((.((....))....))).....))).))))))))).))).))...	11.53	6.10	4.49	5.95	5.30	7.85
gga-miR-1663-5p_R+2	TACCACTGTCCTGGGTGCCTGG	22	5	gp1a	......((((((..(((((((((.((((.(((((((((..(((.......))).....))))	11.53	12.20	16.67	23.82	19.10	23.55

					))))).)))).)))))))))..))))))...						
gga-miR-1684b-3p	AAGTATGAGGAAATGGAGATCT	22	AADN03024942.1	gp1a	......(((..(....((..((.(((((((.((((..(((((((((((.((..(((.......))).)).))))))))))..))))).))))).))..)).)..))).	11.53	69.40	4.49	11.91	33.95	11.77
gga-miR-1731-5p	ACTTGA	23	12	gp1a	...(((	11.53	5.34	10.26	38.11	63.65	41.21

	CTGCAG				((....						
	GCACTG				.(((((						
	CTGCT				(..(..						
					((.((.						
					.(((..						
					.(((.(						
					(((((.						
					.((((.						
					..))))						
					..))))						
					)).)))						
					..))).						
					.)))).						
					.)..))						
					))))..						
					....))						
					)))...						
gga-miR-1747-5p_R+1	TGCACT	23	2	gp1a	((.(((	11.53	12.20	7.05	8.34	2.12	5.89
	TGAATG				(((((.						
	GAGTTC				((..((						
	TGGGT				(.(((.						
					((((..						
					..))))						
					))))))						
					..))))						
					))))))						

					.))..						
gga-miR-6582-3p_R+1	CACTCT<br>GGGTGT<br>TTCTCT<br>TGCAGT	24	3	gp1a	..(((.<br>.(((((<br>((((((<br>((.((.<br>((((..<br>(.....<br>.)..))<br>)).)).<br>))))))<br>))))))<br>)..)))<br>...((.<br>.(((.(<br>(.((..<br>....))<br>.)))))<br>..))..<br>.....	11.53	14.11	7.05	2.38	15.91	3.92
gga-miR-7475-5p_L-3<br>R+1	CCGCCG<br>CCGCGC<br>CCTCCC	18		gp1a	(((.((<br>(((((.<br>((((((<br>((.(((<br>.(((..<br>......<br>))).))	11.53	9.91	0	3.57	3.18	7.85

					).))).))))))))))).)))).						
gga-miR-130b-5p_R-3	CCTCTTTCCCTGTTGCACT	19	21	gp1a	..................((((((..((((....((((((.....)))))..((((((.....)))))........)))).).))))))	10.70	16.40	3.85	57.17	32.36	34.34
gga-miR-217-5p_R-1	TACTGCATCAGGAACTGATTGGA	23	3	gp1a	...(((...(((...(((((.((((....(((((.((((((	10.70	9.91	3.85	4.17	11.14	6.87

					(((..(((........)))...))))))))).))))))))).....)))))..)))..)))......						
gga-miR-15c-3p	CAGACCATTCTGGGCTGCCTCA	22	4	gp1a	...((((((((..(((..(((((.((..((((((((((...........))))))))))..)).)))))..)))..))))))	9.88	16.01	13.47	13.10	15.91	27.47

					)...						
gga-miR-29b-2-5p_L-2_2ss12CT20CT	CTGGTTTCATATGGTGGTTTAGA	23	1	gp1a	((((((((((((.((((((..((((((........... .))))))))))))).)))))).))))...	9.88	18.30	27.58	11.91	8.49	17.66
gga-miR-193b-5p	CGGGGTTTTGGGGGCGAGATGA	22	14	gp1a	...((((..((((..((((.((((.((.(((((....))).....)).)).)))).)))))..))))..)))))...	9.88	5.34	3.85	2.38	8.49	7.85

gga-miR-215-3p_R-1	CCTGTCATTTCTATAGGCCAAT	22	3	gp1a	...((((.(((..((((.(((((...((.((((((((.((((((...((.........)))))))).)))).))))).))...))))))..)))).))))))...	9.88	0	1.92	0	0	1.96
gga-miR-451_L-5	GTTACCATTACTGAGTTT	18	3	gp1a	...((((((......((....)).)))))))...	9.88	20.59	7.70	7.74	3.71	2.94
gga-miR-3530-5p	GCTCTGCTCGCA	21	chr7	gp1a	((.((((((..((	9.88	4.58	3.85	8.34	8.49	5.89

	CCATTG<br>TGG				((((((<br>((.(((<br>((((((<br>((((((<br>.(....<br>......<br>.)))))<br>))))))<br>))))).<br>))).))<br>))))).<br>...)))<br>)).))						
gga-mir-3539-p3_1ss<br>22GT	CTGCCT<br>CATGTC<br>CTCCTC<br>TAGT	22	1	gp1a	...(((<br>(((.((<br>((((.(<br>(.((((<br>(..((.<br>....))<br>.)))))<br>)).)))<br>.))).)<br>))))).<br>..	9.88	6.10	3.85	1.19	8.49	7.85
gga-miR-6603-3p_L+2<br>_1ss3AT	GATAGG<br>ACAAGA	25	chr5	gp1a	.(((..<br>((((..	9.88	0.76	0	1.19	0	0

	GGAAAT<br>GGTTTC<br>A				.(((((<br>(((((.<br>.((..(<br>((((.(<br>((((((<br>(.((((<br>......<br>..)).)<br>).))))<br>)))).)<br>))))..<br>))..))<br>))))))<br>).)..)<br>)))..)<br>))....<br>..					
gga-miR-7451-3p	TTGCTT<br>CTTCTT<br>ATCCCA<br>CAGT	22	1	gp1a	...(((<br>((((((<br>..((((<br>((.(((<br>((((..<br>......<br>))))))<br>)..)))<br>))))))	9.88	3.81	2.57	1.19	3.18	0

					))).))<br>)...						
gga-let-7a-2-3p_1ss<br>22CT	CTGTAC<br>AACCTC<br>CTAGCT<br>TTCT	22	24	gp1a	...(((<br>.(((((<br>((((..<br>(((((.<br>.((((.<br>..((((<br>(((..(<br>((.(((<br>.(((((<br>((((((<br>((....<br>......<br>......<br>.....)<br>))))))<br>))))))<br>.))).)<br>))..))<br>))))).<br>.)))))<br>))))..<br>)).)))<br>.)))).<br>))).	9.06	5.72	6.41	6.55	10.61	9.81

gga-miR-1782	ACATTC ATTGGA GCAGGG ACA	21	2	gp1a	(((... ....((..(((. ((..((.(((.. ((((((.((((. .((((. ((((.(((((((.((((((..... ...)))))).))))))))))).))))..))))..)))))).. .))).))..)). .)))..))...)))....	9.06	16.40	6.41	9.53	12.73	12.75
gga-miR-23b-5p_L+1	TGGGTT CCTGGC	22	Z	gp1a	.((..(((((((	8.23	1.53	1.92	4.76	2.12	5.89

	ATGATGATTT				.((((((((((((((((.((((((.(((.......))))))))))).))))))))))..))).))))))))..))).						
gga-miR-128-1-5p	CGGGGCCGTAACACTGTCTGAGA	23	7	gp1a	...(((((.((((((...(((((((((...((((((..((((..(....).).))))))))))...))))))..)))))))...))))))	8.23	12.96	13.47	38.11	42.43	39.24

					.))))..						
gga-miR-135a-3-3p_R+1	ATGTAGGGCGAAAAGCCATGGGT	23	26	gp1a	...(((((((((((((((((..(((((..((((((.......))).)))..)))))).))))))))))))))...	8.23	3.81	5.77	3.57	4.24	3.92
gga-miR-153-5p_L+1	GTCATTTTTGTGATGTTGCAGCT	23	2	gp1a	.((((......((((((.((((((((((((((((((..(((((((.((.......))..))))))	8.23	3.05	8.98	7.15	5.30	3.92

					)..))))))))))))).)))))).))))))......)))).					
gga-miR-205a_R+1	TCCTTCATTCCACCGGAGTCTGT	23	26	gp1a	...((.((((((...(((.((((.(((((((((((.((((((((.........))))))).)))))))))))).)))).))))))))..))...	8.23	11.44	5.77	390.63	340.54	353.20
gga-miR-1569_R+1	TGTTTGGGACGT	23	2	gp1a	..((((.....(	8.23	0.76	0.64	0	0	0

	TGCTCTGCAGT				(((.........))))(((..(((((..(((((...........)))))..)))))..))).))))).						
gga-miR-1684a-3p_L+1R-1	TAAGTATGAGGAAATGGAGCTC	22	AADN03024942.1	gp1a	...((..((.(((((((.((((..((((((((((.((..(((.......)))..)).))))))))))..))))).)))))).))	8.23	0	0	3.57	5.30	1.96

					..))..<br>.						
gga-miR-1703-5p	AGAGGC<br>TGTAGG<br>TCCCGT<br>GCTTT	23	5	gp1a	((.(((<br>(((.((<br>((....<br>.(((((<br>.(((((<br>.((.((<br>(((...<br>......<br>...)))<br>)).)).<br>))))))<br>))))..<br>...)))<br>))))))<br>))).	8.23	1.53	2.57	8.34	6.37	7.85
gga-miR-1731-3p	TGGCAG<br>TTCCTT<br>GGTCTT<br>GTCC	22	12	gp1a	...(((<br>((....<br>.(((((<br>(..(..<br>((.((.<br>.(((..<br>.(((.(<br>((((.<br>.((((.	8.23	2.29	1.92	7.15	8.49	5.89

					..))))						
					..))))						
					)).)))						
					..))).						
					.)))).						
					.)..))						
					))))..						
					....))						
					)))...						
gga-miR-6516-5p_R+1	ATGCAG	23	18	gp1a	((((..	8.23	6.86	5.13	7.15	8.49	15.70
	TAACAG				(((...						
	GTGTGA				..))).						
	GCATC				.)))).						
					..((((						
					(((.((						
					...(((						
					(.((((						
					.(((..						
					.)))..						
					.)))).						
					))))..						
					.)).))						
					))))).						
					......						
					...						
gga-miR-6669-3p_L-1	TGCAGC	22	Z	gp1a	......	8.23	2.29	3.85	8.34	4.24	3.92

R+1	CTGGCCGTATCTCAGT				.((((..(((...((..(((((.((((((((.....((((((((.....((((....))))..))))))))..))))))))))))..))...)))....))))						
gga-miR-1b-3p_R+1	TGGAATGTTAAGAAGTATGTAT	22	23	gp1a	...(((((.((((((((((((((((.((((..(((((....(((....)))..))	7.41	4.96	27.25	26.80	13.26	10.79

					))).))<br>)).)))<br>))))))<br>)))...<br>..))).<br>))))).<br>..						
gga-miR-15b-3p_L+1_<br>1ss22AC	CGAATC<br>ATTATT<br>TGCTGC<br>TTTC	22	chr9	gp1a	.....(<br>((((((<br>...((.<br>.(((((<br>((.((.<br>((((((<br>(((((.<br>((....<br>...)))<br>))))))<br>)))).)<br>).))))<br>)))..)<br>)...))<br>))))).<br>.	7.41	13.41	18.66	58.95	104.50	89.28
gga-miR-181b-1-3p_L<br>+2R-2	GCTCAC<br>TGAACA<br>ATGAAT	21	chr8	gp1a	.....(<br>((((((<br>.....(	7.41	1.53	7.37	18.46	17.50	12.75

	GCA				((((((<br>((...(<br>((((((<br>((((..<br>((....<br>))..))<br>))))))<br>))).))<br>))))))<br>)....)<br>))))))<br>....						
gga-miR-6544-5p	TTCAGA<br>AAAGGA<br>TATGAA<br>TTGT	22	Z	gp1a	...(((<br>(((...<br>......<br>.)))))<br>)...	7.41	4.19	13.15	10.72	21.22	19.62
gga-miR-138-2-3p_L+<br>2R-1	GCTATT<br>TCACTA<br>CACCAG<br>GGT	21	11	gp1a	...(((<br>(.((.(<br>((..(.<br>.(((((<br>((.(((<br>((...(<br>((((..<br>(((...<br>.))))..	6.59	15.25	7.70	14.29	15.91	9.81

					......<br>..))))<br>)..)))<br>)).)))<br>)))).)<br>..))).<br>))))))<br>...						
gga-miR-460b-5p_R-1	TCCTCA<br>TTGTAC<br>ATGCTG<br>TGT	21	4	gp1a	..(((.<br>.(((((<br>((((..<br>.(((((<br>.(((((<br>((..((<br>((((((<br>(.....<br>......<br>))))))<br>)))..)<br>))))))<br>.)))))<br>...)))<br>))).))<br>))))	6.59	98.38	57.07	83.37	49.86	52.98
gga-miR-1329-3p	CCTCGT<br>AGCTTG	22	3	gp1a	...(((<br>..(...	6.59	9.91	8.34	2.38	4.24	1.96

	ATCACGATAT				.((..(((.(((..(((((((.(((((((.((...............)))))))))).))))))..)))).)))..)))..))...						
gga-miR-1563_R-5	GCACATGATGAGGAAGCAC	19	AADN03019808.1	gp1a	...((((..(((((..((.......(((....))).......))..))))).))))...........	6.59	0.38	0	1.19	0.53	0

					..						
gga-miR-1590	AGGGACAAATTGCTCTCTGGAAG	23	6	gp1a	...((.(((..((.((((..(((((..(((..((((((((.(((((((((((((..(((((..((((....))))).)))))))))))))))).)))))))))..))))))))))))).))..))).))...	6.59	3.81	10.90	5.95	2.12	1.96
gga-mir-1630-p5_1ss5AG	TTCTGAAGGGGGAGGAAA	18	6	gp1a	...(((.((((..((..(	6.59	0	0	2.38	2.12	1.96

gga-miR-1655-5p	TCAGCT<br>GGTCTT<br>GGGTAA<br>ATGC	22	8	gp1a	((...(<br>((...(<br>((((..<br>......<br>.(((((<br>....))<br>)))...<br>.((...<br>.))..)<br>))))..<br>)))..)<br>))..))<br>...)))<br>))))..<br>((.(((<br>(....(<br>((((((<br>(.((((<br>((((((<br>(((..(<br>(.((((<br>((((((<br>((...(<br>(....)<br>))))))<br>))))))	6.59	3.81	3.85	1.19	5.30	5.89

					).))..<br>))))))<br>))))))<br>).))))<br>))))..<br>)))).)<br>)						
gga-miR-1736-3p	AGCACC<br>TTCGGA<br>ACTGTC<br>CTCA	22	1	gp1a	..(((.<br>(((.((<br>....((<br>(((..(<br>(.(..(<br>((((((<br>(((((.<br>((((.(<br>(.((((<br>((((((<br>((....<br>.....)<br>))....<br>))))))<br>))))))<br>))).))<br>))))))<br>))))..<br>).))..	6.59	31.27	14.11	3.57	11.67	17.66

					))))).))))))))...					
gga-miR-3533_L-2R+1	GAAGTGTGATGTGGATATC	19	10	gp1a	...((((((..(((((..((..((((((....((((((((........)))))).))).))))))..))....)))))...))))))...	6.59	1.53	0.32	4.17	7.43	5.89
gga-mir-3536-p3	GGGGACAATGTATGCAAC	18	25	gp1a	...(((.((((((((((((.((...((((((..((((........))))	6.59	0	0	0	0	0

					..))))						
					))...)						
					)..)))						
					))))).						
					..))).						
					)))...						
gga-miR-6548-3p	AGAGGT	22	chr14	gp1a	.((((.	6.59	0.76	1.28	5.95	11.67	9.81
	GCCCCG				((((..						
	CTGTTG				..(((.						
	CTGT				(((...						
					......						
					..))))						
					))....						
					))))))						
					))....						
					......						
					((((((						
					.((((.						
					...((.						
					...)).						
					..))))						
					.)))))						
					).....						
					.....						
gga-miR-6552-5p	GTCTGT	21	chr5	gp1a	...(((	6.59	1.53	0.64	2.38	4.24	1.96
	CCAGGT				...(((						

	CTTGTTGGA				(.((((((((((((((..((((((((((.(((.....))).))))))))))..)))))))))))))))).)))).....)))...........						
gga-miR-6578-5p_L-1R+2	AGCTGTGGCAGAGTTCTCCACC	22	3	gp1a	...(((((..(((((.((((((((((.(((((..((.....)).)))).))))))))).)	6.59	0	0	4.76	11.67	5.89

					).))).))..)))))....(((......)))...						
gga-mir-6619-p3	AATGACGTGTGGCGTGGAT	19	JH376175.1	gp1a	.....(((((((....)))..((((((((((((....((.(((((((..........))))))).))))))).)))))))))))...((((.......))))))))).....	6.59	0.76	0	0	0	9.81
gga-miR-7455-3p_R-1	CTTCCC	22		gp1a	..((((	6.59	4.58	1.92	7.15	9.55	3.92

_1ss22AT	TCCGTC<br>GGCGCG<br>CACT				(.((((<br>((((((<br>(.(((.<br>(((...<br>..))))<br>))))))<br>))))).<br>)).)))<br>))....						
gga-miR-7_R+1	TGGAAG<br>ACTAGT<br>GATTTT<br>GTTGA	23	Z	gp1a	((.((.<br>...(((<br>(((.((<br>.(..((<br>(((.((<br>(((.((<br>((.(((<br>((((((<br>(((((.<br>...)))<br>))..))<br>))))))<br>))))))<br>)))).)<br>))))..<br>).))))<br>))))..	5.76	13.73	50.34	2.98	1.59	3.92

					)).))						
gga-miR-29c-3p_R+1_1ss18GA	TAGCACCATTTGAAATCAGTT	21	1	gp1a	...(((.((((((.......................))))))))))..............	4.94	5.72	6.73	0	0	0
gga-miR-133b_L+1_1ss22AT	TTTGGTCCCCTTCAACCAGCTT	22	3	gp1a	...((.(((((.((((((((..((.((.(((((((.....((....)))))))))).)).))..))))))).)))))).))...	4.94	0.38	1.60	0.60	0.53	0

gga-miR-187-5p_L-1R+2	GCTACAACACAGGACATGGGAGT	23	2	gp1a	.......((((...((((.((((.((((((((((((((((.................)))))))))))))))))).))))).))))..))))...	4.94	3.81	1.28	2.38	1.06	1.96
gga-miR-214_L-1	CAGCAGGCACAGACAGGCAG	20	4	gp1a	...(((...(((((.....((((.(((((.((((((((((((((.....((.(((((...))))	4.94	5.72	0.96	2.98	1.59	0.98

					)).)).....))))))))))))).))....)))))).))))..)))))..))).....						
gga-miR-221-3p_R-4	AGCTACATTGTCTGCTGGG	19	Z	gp1a	((((((((((...................)))))))).)))....	4.94	7.24	2.24	12.50	6.37	6.87
gga-miR-383-3p	CCACAGCACTGCCTGGTCAGA	21	4	gp1a	...(((((..((......((((((.(((((.((.((((((..((((.(	4.94	4.58	3.21	7.15	8.49	7.85

					((((((<br>(((..(<br>......<br>)..)).<br>))))))<br>))))))<br>..))))<br>)).)).<br>.)))))<br>.))).)<br>))....<br>.))..)<br>))))..<br>.						
gga-miR-449a	TGGCAG<br>TGTATG<br>TTAGCT<br>GGT	21	Z	gp1a	.(((((<br>(..(..<br>((.(((<br>(((((.<br>.(((((<br>((((((<br>..(...<br>..)..)<br>)..)))<br>))))))<br>..)).)<br>))))).	4.94	2.29	0	1.19	1.06	3.92

					))..).<br>.)))))<br>).						
gga-miR-455-3p_L-2R+3	CAGTCCATGGGCATATACACCTT	23	chr17	gp1a	((.(((...(((.((((((((((.(((((((.(((.(((.........)).))).)))))).)))))))))).)))...))).))	4.94	0.76	2.57	0	1.06	0
gga-mir-1457-p3_1	CTCCTTGGGCAACCTGTTC	19	9	gp1a	...(((((.....((((((((....((((....))))..)))))...)..))))	4.94	0	0	0.60	0	0

					.....)))))...						
gga-mir-1457-p5_5	TTGGGCAACCTGTTCCAGT	19	3	gp1a	...(((..((((((((((((((((....)))))......))))))..)))))))..)))....	4.94	0	0	0.60	0	0
gga-mir-1457-p3_2	CTGTTCCAGTGCGTCACC	18	4	gp1a	..........(((...)))...	4.94	0	0	0.60	0	0
gga-mir-1457-p3_4	TTGGGCAACCTGTTCCAGT	19	13	gp1a	...((((((((.......((....))......)))))))))...	4.94	0	0	0.60	0	0

gga-mir-1457-p3_3	TGGGCA ACCTGT TCCAGT	18	6	gp1a	...((. .(((((((.... .... ((.(((((((.. ....)))).))) ..)).((....)).....)))))))..)). ..	4.94	0	0	0.60	0	0
gga-mir-1594-p5	GTCGGT GGGCAT GGTGGG GATGGG T	25	Z	gp1a	..(((. ..))). (((((. (((((((....(((.(((((..(((((((. .(...) ..))))	4.94	0	0	0	0	0

					)))..))))))))))))))))))))						
gga-mir-1635-p3_1ss 5GT	CTGGTG GCACGC TCAGCT	18	AADN03 022998 .1	gp1a	...((((..(((..(((((.(((((.((.. ....)) .))))) ....((((...(((...... ....))) ..))))))))). .))).))))...	4.94	0	0	0	1.06	1.96
gga-miR-1727_R-1	AAGCTG CTCTAA TGAACT GAAG	22	1	gp1a	((.(((.(((((..((((((((((	4.94	0.76	0.64	0	1.06	3.92

gga-miR-1756a	TCCAGT<br>GATTCA<br>CACCAG<br>CTGT	22	Z	gp1a	(((((.<br>((..((<br>((((..<br>.(((((<br>......<br>...)))<br>))...)<br>))))).<br>.)).))<br>))).))<br>))))))<br>))))))<br>)....)<br>)))).<br>....((<br>((((((<br>(.((.(<br>((((((<br>(((.((<br>(((...<br>((((..<br>.(((((<br>.....)<br>))))))<br>))...)<br>)))).)	4.94	4.58	1.28	3.57	1.06	0

					))))))<br>))).))<br>.)))))<br>))))						
gga-miR-1781-5p_R-1	AACAGC<br>TGAGTG<br>ATTTAA<br>AGC	21	14	gp1a	...(((<br>(.(((.<br>((((.(<br>((((((<br>((((((<br>..((((<br>((((((<br>(...((<br>((....<br>))))))<br>))))))<br>)))..)<br>))))))<br>))))))<br>.)))))<br>))))))<br>...	4.94	9.15	5.13	2.38	1.06	1.96
gga-mir-1797-p5	AGTCTC<br>TGCCCA<br>GGACCA<br>ACTGC	23	26	gp1a	...((.<br>((((((<br>...(((<br>..((((<br>.(((..	4.94	0	0	1.19	2.12	0

					(((((((.((......)).))).))))..))).))))..))).....)))))))).					
gga-miR-3525_R-2	CAGCCATTCTGCGATTCTGT	20	1	gp1a	...((.((((..(((((((..((((..................))))..)))))))))))).))...	4.94	3.81	1.92	0	3.18	1.96
gga-miR-3533_R-2	ATGAAGTGTGATGTGGAT	18	12	gp1a	...((((.((((((..............))))))))	4.94	0	0	0.60	1.59	0

					))...						
gga-miR-3540	ATGGTG GAAGAA CAAGGC CTGC	22	10	gp1a	....((((((.(((. .((((((((((. ....))))))).)))..))) .))))))...	4.94	3.81	5.77	3.57	3.18	7.85
gga-miR-3607-5p_R+1	TACATA TGATGA GCTTTG CAGTA	23	Z	gp1a	.(..(((..... .(((.. ...(((((((((.....((((((((((((((.(((. .....))).)))))))))).)))) ....))	4.94	5.34	4.49	14.29	18.03	25.51

					))))))						
					)...))						
					)..)))						
					..)...						
gga-miR-6546-5p	AGGAGC	22	7	gp1a	......	4.94	3.05	4.49	5.95	19.10	19.62
	GCCTCT				(((..(						
	GAGCTC				(((...						
	CCCT				.)))).						
					))).((						
					(((.((						
					((((((						
					(.((((						
					(..(((						
					((((((						
					((.(((						
					....))						
					))))))						
					))))))						
					..))))						
					).))))						
					))))).						
					.)))))						
					...						
gga-miR-6548-5p	AACAAC	22	chr14	gp1a	.((((.	4.94	16.01	10.90	34.54	50.92	47.09
	AGCTGC				((((..						
	GTGCCA				..(((.						

	TGCC				(((.........)))))).....)))))))).........((((((.((((....((....))...)))).))))))..........						
gga-mir-6578-p3	TGGGGCACTCTGCTGACC	18	3	gp1a	...(((((..(((((.((((((((((.(((((..((.....)).))))).)))))))).)).))).	4.94	1.53	0	1.19	13.79	7.85

					))..))<br>)))...<br>.(((..<br>....))<br>)...						
gga-miR-6631-5p	GAAGAG<br>AATGCT<br>GTGGTT<br>CTGC	22	3	gp1a	((.(((<br>..((.(<br>((((((<br>((((((<br>(..(((<br>((..((<br>(((((.<br>....((<br>(.((((<br>(..(((<br>((((.(<br>(..(..<br>......<br>.)..))<br>..))))<br>))))))<br>))))))<br>))))))<br>..))))<br>).))))<br>).....	4.94	2.29	3.21	7.15	0	3.92

					..)))))).)))..))..))))).						
gga-miR-6648-3p	TCCGGCATTCTGAACGCTCCT	21	15	gp1a	...(((((((((((((((((((((((.(((....)))..)))))))))))))))))))))).......	4.94	1.14	2.89	1.19	4.77	2.94
gga-mir-6681-p3	AAGGATAATGCTGAATGACTCT	22	20	gp1a	........(((((.(((...(((((....(((((((((..(((((((((...((....)).	4.94	0	0	1.19	0	0

					..))))<br>))))).<br>.))).)<br>))))..<br>.)).))<br>).....<br>))).))<br>)))...<br>.						
gga-miR-6691-5p_L-2R+1	GCATGATGAACCTGCTGAGCT	21	7	gp1a	...(((<br>((((((<br>(((((.<br>...(((<br>(....(<br>((....<br>..))).<br>))))..<br>..))))<br>.)))))<br>)))))(<br>(((((.<br>.....)<br>))))).<br>...	4.94	0	0	0.60	0	0.98
gga-mir-6700-p5_1ss4GA	TGAAGGAGCTGG	20	3	gp1a	...(((<br>((...(	4.94	0	0	0	0	0

	GATTGTTC				(..((...((((((..((((((.......))))))))))))...))..))..)))))...						
gga-mir-7475-p5_1ss17GC	CGCCGCCGCGCCCTCCCC	18	9	gp1a	..((((..(((........)))...........))))...	4.94	9.15	0.64	1.79	5.83	6.87
gga-miR-30c-5p_L+2	CATGTAAACATCCTACACTCTCAGCT	26	23	gp1a	..((((.((..(((.((..(..((.((.....((.(((((..((((...(((((	4.12	6.10	5.13	0	6.37	2.94

					((((((<br>((...(<br>((((((<br>(..(..<br>......<br>.)..))<br>))))))<br>)))).)<br>))))))<br>))...)<br>))))))<br>))))..<br>.)).))<br>..)..)<br>))))..<br>))))))<br>..						
gga-miR-1434_R-1	GTGCGT<br>GATGAT<br>GGAAAA<br>T	19	Z	gp1a	.....(<br>((((.(<br>((((((<br>..((((<br>(..(((<br>(....)<br>))).((<br>(((((.<br>..(((.	4.12	0.38	0.32	1.79	1.59	3.92

					.....)))..)))))))........))))).. ..))))))))))))))..						
gga-mir-6668-p5_1ss16CG_1	GCTTGGGTTGGAAGGGACC	19	7	gp1a	...((((....((((..((((...(((..(((((......)))))....)))....))))..))))....))))....	4.12	0	0	0	0.53	0
gga-mir-6668-p3_1ss16CG_2	GCTTGGGTTGGAAGGGACC	19	26	gp1a	...(((((.(((((.(..((.((.(((((	4.12	0	0	0	0.53	0

					.......)))))...(((.....)))))))..))))))).)))))...						
gga-mir-6668-p3	GAATAGCTTGGGTTGGAAGG	20	9	gp1a	...(((.((((.(((......(((((((((((.....(..(((.................)))..)...)).)))..))))))...))).)))))))))...	4.12	0	0	0	0	0
gga-mir-6668-p3_1ss	GCTTGG	18	JH3763	gp1a	...((.	4.12	0	0	0	0.53	0

16CG_1	GTTGGAAGGGAC		30.1		(((.........)).))...						
gga-miR-34b-5p_L-1R+2	AGGCAGTGTAGTTAGCTGATTGTT	24	24	gp1a	...((((((..((((..(((((((...((((.(((((((.........…)))))))))))...)))))))..))))...))))))...	3.29	0.76	0	1.19	0	0
gga-miR-124a-3p_L-1	TAAGGCACGCGGTGAATGCCA	21	2	gp1a	....((((.((((..(..(((((.(((..((((((((..	3.29	0.76	0.64	3.57	3.18	6.87

gga-miR-140-5p_L+1R<br>+1_1ss2AN	CNGTGG<br>TTTTAC<br>CCTATG<br>GTAGA	23	chr11	gp1a	......<br>...)))<br>))))).<br>.))).)<br>))))..<br>)..)))<br>))))).<br>)))...<br>......<br>...(((<br>((((((<br>((....<br>)))...<br>......<br>...(((<br>((((..<br>...(((<br>((((((<br>(.((((<br>((((((<br>(((.((<br>(((((.<br>.(((..<br>....))<br>))))))<br>)).)))	3.29	0	1.28	1.19	1.06	0.98

					))))))<br>))))))<br>.)))))<br>))).))<br>))))).<br>....))<br>))))))						
gga-miR-144-5p_R-3	GGATAT<br>CATCAT<br>ATACTG<br>T	19	Z	gp1a	((.(((<br>(....)<br>))))).<br>.	3.29	12.20	11.54	3.57	5.30	3.92
gga-miR-451_L-1R-4	AACCGT<br>TACCAT<br>TACTGA	18	19	gp1a	((.(((<br>(((.((<br>(..(((<br>((((((<br>((((((<br>((....<br>..))))<br>))))))<br>))))))<br>)..)))<br>.)))))<br>).))	3.29	0.76	1.28	0	0	0
gga-miR-458a-5p_R-1	AGCGCC<br>ATTTTC<br>AGAGCT	20	13	gp1a	...((.<br>.(((((<br>(.((((	3.29	12.20	4.49	2.38	0	0

	AT				(((..(<br>(((((.<br>.(((((<br>(((..(<br>(((...<br>..))))<br>.)))))<br>)))..)<br>))))).<br>.)))))<br>)).)))<br>))))).						
gga-miR-499-3p	AACATC<br>ACTTTA<br>AGTCTG<br>TGCT	22	20	gp1a	((..((<br>((((..<br>((((((<br>(((((.<br>.(((..<br>..))).<br>.)))))<br>))))))<br>..))))<br>))..))<br>...	3.29	0.76	0.64	20.25	4.24	5.89
gga-mir-1457-p5_1ss<br>16CT	CAACCT<br>GTTCCA<br>GTGTGT	22	2	gp1a	...(((<br>((.((.<br>.(((((	3.29	0	0	0	0	1.96

	CACC				(.((.. ....((((....))))(((((..... .))))) .))))))))...))..)))))...						
gga-mir-1457-p3_1ss4AG	ACAGCCTCCTTGGGCAACC	19	3	gp1a	....((((. ...))))....	3.29	0.38	0	0	0	0
gga-mir-1557-p3	TGAGCGGCTGCAATTGCCTCAC	22	2	gp1a	...(((....((..((.(((((((((((.(((((((((((((((((((......	3.29	1.53	0	0	2.12	0

					..)))))))))))))))))).))))))))))).))...))....)))...						
gga-mir-1575-p5	CGTGCTGTGCTTCGTGGTGCT	21	4	gp1a	..((((((((((((........))..))))))))))((((((((.((........)).)))).)))).....	3.29	0	0	0	1.06	0
gga-miR-1603_R+2	AGTGGTTGGTTTGGTGCTGTCGA	23	7	gp1a	...((.(((((((.((((((((((((.(((.	3.29	0.76	0.64	0	1.06	0

					.((((((.((((.((....)).))))))))))).))))))))).))))).))))))))...						
gga-miR-1612_L+1_1ss23GT	GGAAGGAAAAGGAGCTGACTGGT	23	chr9	gp1a	(((..((((.((((.(..((((((...((((((((((.........)))))))))))))))))))))..).))))..))))..)))	3.29	0	0	0	0	0
gga-mir-1619-p5_1ss	TGTATT	21	20	gp1a	...(((	3.29	0	0.64	0	0	3.92

16CG	GCTACA<br>AATGCT<br>CTT				.(((((<br>.....(<br>((((((<br>((((((<br>((((((<br>((((..<br>(((..(<br>(((((.<br>((....<br>....))<br>.)))))<br>))))..<br>))))))<br>))))))<br>))))))<br>))))).<br>.)))))<br>.)))..<br>.						
gga-mir-1626-p5	ATGGCA<br>GACTTT<br>CTAGGC<br>TGAAGA<br>TT	26	1	gp1a	((.(..<br>((((((<br>(((..(<br>(((.((<br>((.((.<br>((((((<br>.....(	3.29	0	0	0	0	0

					(((((...)))))))))))))))))).))))..))))))).)))..))).						
gga-miR-1627-5p_L+2R+2	CTGAAGTGACATGTGGTTGATGGCG	25	4	gp1a	............(((((...((((.................)))))))))...	3.29	0	0	0	0	0
gga-miR-1630_R-4_1ss6GA	AAAGAAGAAACTGAAGCT	18	chr9	gp1a	....(((((..((((((((((((.........)))))))))))))..	3.29	0	0	0	0	0

					))))).......						
gga-mir-1647-p3	AGAGTTGCCCCGTAGGCATG	20	28	gp1a	....((((((((((((((.((.((((.(..(((((..(((((((((.........)))).))))))..))))))..).))))).)).))))).)))).)).)))....	3.29	0	0	0	0	0
gga-mir-1648-p5_1ss11TG	GCGGCTCGGCGCGGCTCCG	19	20	gp1a	..((((((((((..((((((.((((((((((.(((((	3.29	0.76	0	1.19	0	0

gga-miR-1653_R+2	CAGGAG<br>CTGTGC<br>TGCATG<br>CAGGTT	24	9	gp1a	.((((.<br>...)))<br>)....)<br>)))).)<br>))))))<br>)).)))<br>))).))<br>))))..<br>))))..<br>((.(((<br>((((((<br>((..((<br>(....(<br>((....<br>)))..)<br>))...)<br>))))))<br>))))..<br>.))...<br>((((((<br>....))<br>))))((<br>((((((<br>(.((..<br>..)).)<br>))))))	3.29	0.76	1.28	0	0	3.92

					)).						
gga-miR-1656_R+1	TCACCAGCGGGCATTGGCATCAC	23	1	gp1a	...((.((((..((((((..((.(((((((((.(((...))))))))))))))))).)))))))..))))))...	3.29	0.76	0	1.19	4.24	5.89
gga-miR-1668-3p	TGCTGTGTCCTCACTCACCTT	21	LGE22C19W28_E50C23	gp1a	....((.(((.((.(((((((.((((..(((((.(((..(........).)..))))))))..))))))))))).)).)	3.29	6.10	2.57	14.29	20.16	27.47

					))..))						
gga-mir-1700-p5_1ss12CG	AGGATGGACAGGTGGTGTC	19	17	gp1a	.........(((..((((((...(((((((((((((..((((.........(((((.....))))))))...)))))))))))))))).))....)))...	3.29	0.38	0	3.57	3.71	2.94
gga-mir-1700-p3_1ss12CG	AGGATGGACAGGTGGTGTC	19	1	gp1a	............(((((..(((.(((....((((.(((.(((((((.	3.29	0.38	0	3.57	3.71	2.94

					......<br>...)))<br>)))).)<br>)).)))<br>)...))<br>).))).<br>.)))))<br>...						
gga-miR-1712-3p_1ss<br>22GT	TTCAGT<br>TATCAG<br>TGGAGT<br>TTGT	22	3	gp1a	..((((<br>((.(((<br>(..(((<br>(((...<br>.(((((<br>..(((.<br>((((..<br>..))))<br>..))).<br>.)))))<br>....))<br>))))..<br>)))).)<br>).))))<br>..	3.29	1.53	1.92	7.15	25.46	5.89
gga-mir-1723-p5	GAGCTG<br>CACATT<br>TCGTGT	22	2	gp1a	((((.(<br>((((((<br>((((((	3.29	2.29	0	1.19	2.12	0

	CCCA				(((.((						
					(((...						
					......						
					..))))						
					))))))						
					))))))						
					))).))						
					.)))).						
					......						
					......						
					.						
gga-miR-1723_L-3R+2	GAGCGG	22	2	gp1a	((((.(	3.29	1.53	1.28	9.53	9.02	0.98
	AATGTG				((((((						
	CAGCCT				((((((						
	CACT				(((.((						
					(((...						
					......						
					..))))						
					))))))						
					))))))						
					))).))						
					.)))).						
					......						
					......						
					.						
gga-miR-3532-3p	TTGGAG	22	5	gp1a	...(((	3.29	3.81	3.85	3.57	4.24	9.81

	GCTGCA<br>GTGTCA<br>TGGT				((((..<br>(((..(<br>((((((<br>(((((.<br>.((..(<br>((.(((<br>......<br>.)))))<br>)..)).<br>))))))<br>)))).)<br>)..)))<br>..))))<br>)))...						
gga-miR-6542-3p_R-1	ACGGGA<br>CAGTGC<br>TGAAGA<br>CT	20	3	gp1a	......<br>......<br>.(((((<br>.(((((<br>((((..<br>((((((<br>...(((<br>..(..(<br>......<br>..)..)<br>....))<br>)...))	3.29	0	1.28	3.57	4.24	3.92

					)))).))))).))))).)))))...						
gga-miR-6553-5p	AGCAGCCCTGTGATAGCTGTTTGC	24	1	gp1a	........((((((.((((((.(((((.........))))).((.(((......))).))....)).)))))))))).	3.29	6.86	8.34	3.57	8.49	7.85
gga-mir-6584-p5	AAGTTCTGCAGATGATGATACC	22	2	gp1a	...(((....(((.(((((.....((((((((..(((((((((((.........	3.29	0	0	1.19	3.18	0

					.)))))<br>))))))<br>......<br>))))))<br>))....<br>))))).<br>.)))..<br>....))<br>)..						
gga-mir-6584-p3	CAAAGA<br>TGTGCG<br>AGCTCA<br>AAGACC	24	2	gp1a	...(((<br>....((<br>(.((((<br>(.....<br>((((((<br>((..((<br>((((((<br>(((...<br>......<br>.)))))<br>))))))<br>......<br>))))))<br>))....<br>))))).<br>.)))..<br>....))	3.29	0	0	2.38	0	7.85

					)..						
gga-miR-6602-5p_L+5R-2	TGAGGACAGGGATTGCAGAGGTTGG	25	26	gp1a	((....))..(((((..((((((...((((((..((.....))..)))))).)))))).))))))...((((((.....((((..((((((((..(((((....))).))...))))))..))))))))))	3.29	0	0	0	2.12	0
gga-mir-6602-p3	CATTCTGCTGTACATTGCT	19	26	gp1a	((....))..(((((..((((((.	3.29	0	0	1.79	0.53	0.98

					..(((((..((.....))..)))))).))))).))))...((((((.....((((..((((((..(((((....))).))...))))))..))))))))))						
gga-mir-6602-p5	CATTCTGCTGTACATTGCT	19	1	gp1a	...(..((..((((((((((.........))))))).)))).)..))..).	3.29	0	0	1.79	0.53	0.98

					..					
gga-miR-6615-5p_R-1	TTGGGGACACCATCAGAACAGCC	23	28	gp1a	.((((((((((.((((((....((((((..((.....)).)))))))).)))))))))))).))).))..	3.29	4.58	10.26	11.91	19.10	11.77
gga-mir-6627-p5	CATTTCCCTCAGGCCTGTGGTGTT	24	4	gp1a	(((..(((..(..(((((..((......)).....)))))..)..))))..))).....((..((..((..((..((.((((((	3.29	0	0	0	1.06	0

gga-mir-6627-p3	AAGGTG<br>ATGCTG<br>TGGATG<br>GGCACC	24	4	gp1a	((..((<br>(...((<br>(....)<br>))..))<br>)...))<br>))))).<br>.))..)<br>)..)).<br>.))...<br>(((..(<br>((..(.<br>.(((((<br>..((..<br>....))<br>.....)<br>))))..<br>)..)))<br>..))).<br>...((.<br>.((..(<br>(..((.<br>.(((((<br>((..((<br>(...((<br>(....)<br>))..))	3.29	0	0	0	0	0

					)...))<br>))))).<br>.))..)<br>)..)).<br>.))...						
gga-miR-6664-3p	CAGGAA<br>TGGTGC<br>TGGAGG<br>CTGA	22	20	gp1a	(..(((<br>(...((<br>((((((<br>((((((<br>(.....<br>....))<br>))...(<br>(((...<br>.....(<br>((....<br>.)))..<br>......<br>)))).)<br>))))))<br>))))..<br>..))))<br>..)...	3.29	0	0	0	0	0
gga-mir-6668-p3_1ss<br>5AG_1	GAATGG<br>CTTGGG<br>TTGGAA<br>G	19	21	gp1a	...(((<br>((...(<br>(((((.<br>...(((	3.29	0	0	1.19	0	0

					(((..(((((((((..((((...(((.........))...))))...).))))..))))...)).)))).))))))....)))))..						
gga-mir-6668-p5_1ss5AG	GAATGGCTTGGGTTGGAAGG	20	1	gp1a	.....((.((..((..(((((..((((...(((((........))))).....)	3.29	0	0	1.19	0	0

					))))．)))))．．))．．))))．．						
gga-mir-6668-p3_1ss5AG_2	GAATGGCTTGGGTTGGAAGG	20	4	gp1a	...((((((((((((((...))))).)))))))))..	3.29	0	0	1.19	0	0
gga-mir-6703-p5_1ss18CG	AGTGCATGAGCTTTCACG	18	1	gp1a	..........(((((.........))))))...	3.29	0	0	0	0	0
gga-miR-7462-5p_R+1	CAACCTCATTTCTAGCTTCTGGT	23	3	gp1a	...((((((.(((((((((((((.(((((.((((((..(.((.........)	3.29	3.81	1.92	0	0	1.96

					))..))))))..)))))..)))))))))))))).))))))...						
gga-miR-7472-3p_1ss22CA	CAGCTCCTCTCCATCACTCTGA	22	chr6	gp1a	..(((.((((((.((.((((.((.......................)).))))))..)))))).))).....	3.29	0	0	0	1.06	0
gga-let-7j-3p_L-1R+2	TATACAGTCTATTGCCTTCCTTT	23	chr26	gp1a	..((((((((((..((((((((((...((..((((((...))))).)..	3.17	2.94	3.00	0.95	0.34	0.25

					))......))))))))).)))))))))).).....						
gga-let-7b_L-1R-1	GAGGTAGTAGGTTGTGTGGT	20	21	gp1a	...((.((.((.(((..((((....)))).))).))))))...	2.47	3.81	3.85	1.19	1.59	0.98
gga-let-7a-2-3p_R-2	CTGTACAACCTCCTAGCTTT	20	3	gp1a	.....((..((((((((......((((..((....))..)))).......)))))).)).))...	2.47	1.14	0.64	0.60	0	0
gga-miR-206	TGGAAT	22	3	gp1a	((((.(	2.47	0	0.64	3.57	4.24	3.92

	GTAAGG				(..(((						
	AAGTGT				((.(((						
	GTGG				......						
					((..((						
					((...(						
					((((..						
					(((..(						
					((((((						
					((((..						
					(((((.						
					......						
					......						
					))))).						
					))))))						
					))))).						
					.)))..						
					))))).						
					.)))).						
					.))...						
					.))).)						
					))))))						
					))))..						
					.						
gga-miR-221-3p_R-5	AGCTAC	18	1	gp1a	......	2.47	1.14	0	1.19	2.65	0.98
	ATTGTC				.(((((						
	TGCTGG				((.(((						

					......<br>.)))..<br>......<br>...(((<br>(.....<br>......<br>......<br>.....)<br>)))..)<br>))))))<br>...						
gga-miR-1467-5p	TCTCAG<br>CTACAT<br>CGGTGT<br>AAATC	23	2	gp1a	...(((<br>(.((.(<br>((((.<br>...(((<br>(((.((<br>(.((((<br>(((.((<br>((((.<br>......<br>...)))<br>.)))).<br>))))).<br>))))).<br>))))))<br>....))	2.47	3.05	2.24	4.17	9.02	4.91

					))))．))..... .))))． ..						
gga-miR-1648-5p_R-2 _1ss10TG	CGGCTC GGCGCG GCTCCG C	19	Z	gp1a	...(((((((.. ((((.. ....)))))))))))...	2.47	0.76	0.32	2.38	0	3.92
gga-mir-3533-p3_3	ATGGAG TCCTGT GGTATC C	19	24	gp1a	...((((((((((((.(. .(....)..)..)))))))))))) ...	2.47	0	0	0	0	0
gga-mir-3533-p3_2	ATGAAA CTACCT TCAACT C	19	7	gp1a	..((((((.... .....(((((.. ((((((....))))))．.)))))．	2.47	0	0	0	0	0

					.((((((.........))))))........))))))............						
gga-mir-3533-p3_4	TACCTTCAACTCCATCATG	19	12	gp1a	.(((((.(((((((..((......((((....))))...)))))))))..)))))....	2.47	0	0	0	0	0
gga-miR-6592-3p_R+1	AGCGTCTCCCGCCTGACGGCCA	22	19	gp1a	...((((((.(((..((((((((((...((((..((((((.((.	2.47	0	0.64	0	2.12	0

					.....)<br>).))))<br>)..)))<br>)...))<br>))))))<br>)..)))<br>))))))<br>...						
gga-mir-6603-p5_1ss<br>18GA	TTTCCC<br>CTTGTC<br>CTGTCA	18	2	gp1a	......<br>..((((<br>......<br>))))..<br>.	2.47	0	0	0.60	0.53	0
gga-mir-6672-p3	CAGGAC<br>AAGGGG<br>CAGTGG<br>GCACA	23	5	gp1a	...(((<br>(((..(<br>((((..<br>((....<br>...)).<br>.)))))<br>.....)<br>))))).<br>..	2.47	0	0	1.19	0	0
gga-miR-9-3p	TAAAGC<br>TAGAGA<br>ACCGAA<br>TGT	21	28	gp1a	...(((<br>((((..<br>.(((((<br>((.(((	1.65	0	1.28	5.95	1.06	1.96

gga-miR-10b-3p_L+2R-1	ACAGATTCGATTCTAGGGGAAT	22	7	gp1a	((((.(((((.(((.....)))))))).)))))))).))))))))...))))))).......((((((((((...(((((((.........(((.((.(((((.((((((.((((.((((.((((((((((...(((....)))..))))))))	1.65	0	0	2.38	1.06	1.96

					)))))<br>))))))<br>).))))<br>)))).)<br>)))).)<br>).))))<br>))))))<br>..))))<br>)).)))<br>)..						
gga-miR-16c-3p_L+1R<br>-1	TCCAGT<br>ATTGCA<br>TTGCTG<br>CTTT	22	4	gp1a	...(((<br>((...(<br>((..((<br>(((((.<br>...(((<br>((((((<br>(...((<br>(....)<br>))....<br>..))))<br>))))))<br>....))<br>))))).<br>.)))..<br>.)))))<br>...	1.65	2.29	1.92	8.34	8.49	11.77

gga-mir-17-p5	ACTGCA GTGAAG GCACTT G	19	1	gp1a	.((((((((((((..((((.(((((....))))))))).)))))))))).))	1.65	1.14	0.32	1.79	3.18	2.94
gga-miR-17-3p_R-2	ACTGCA GTGAAG GCACTT	18	2	gp1a	...((. .((((((....((....((((((((...((((((.. ....)))))).)))))))))))))))))). ..	1.65	0.38	0.32	1.19	0.53	0
gga-miR-17-5p_R-4_1 ss20GT	CAAAGT GCTTAC AGTGCA GT	20	2	gp1a	....(((((((((((((. ((....	1.65	3.05	0.96	0	1.59	3.92

					)).)))<br>))))).<br>.)))))<br>...						
gga-miR-26a-5p_R-2_<br>1ss19GT	TTCAAG<br>TAATCC<br>AGGATA<br>T	19	3	gp1a	((((..<br>(((..(<br>((....<br>.))).)<br>)).)))<br>)...	1.65	4.58	1.60	0	0	0
gga-miR-29a-5p_L-1R<br>-1	TGATTT<br>CTTTTG<br>GTGTTC<br>AG	20	Z	gp1a	......<br>...(((<br>(((((.<br>((((((<br>((....<br>....((<br>(((((.<br>...)).<br>))))).<br>......<br>.)))))<br>......<br>...)))<br>))))))<br>))...	1.65	2.29	0.32	0	1.06	0
gga-miR-30e-5p_R-1_	TGTAAA	18	2	gp1a	......	1.65	0	0	0	0	0

1ss18GT	CATCCT<br>TGACTT				......<br>(((((.<br>.(..((<br>(((...<br>......<br>))))).<br>.)))))<br>)...						
gga-miR-34c-3p_L-1R+1	ATCACT<br>AACCAC<br>ACAGCC<br>AGGT	22	24	gp1a	...(((<br>.....(<br>((.(((<br>.(((((<br>((..((<br>(((.((<br>(.((((<br>.(((((<br>.(((((<br>(((...<br>.))..)<br>))))))<br>)))).)<br>))).))<br>).))))<br>)..)))<br>))))))<br>))))..	1.65	0	0.64	0	0	1.96

					)))...						
gga-miR-133c-5p_L+1R-1_2ss12AT20GA	AGCTGGTAAAATGGAACCAAAT	22	20	gp1a	...((((...((((.(((((((.((.((.((((((((............)))))))))).)).)).)))))))).))))...)))))...	1.65	0.76	3.85	0	1.06	0.98
gga-miR-144-3p_R-3	CTACAGTATAGATGATGTA	19	17	gp1a	...((((((((.((((((((((....))))...(((..((.(((((..(......)..))	1.65	1.53	1.92	0	1.06	0

					)))))..)))..)))))).))))))).))...						
gga-miR-144-3p_L-1R-3	TACAGTATAGATGATGTA	18	7	gp1a	...((((((.((((..((.(((........))).)))))).)))))))..........	1.65	0.76	1.28	2.38	1.06	1.96
gga-miR-146c-3p_R-1	AGTCCATGGTATTCAGTTCTC	21	4	gp1a	...((((((.((((.(((((((((((.(((((((((((....(((......))....)))))	1.65	7.24	4.17	4.76	3.71	3.92

					))))).)))))))))))..)))).))..))))...						
gga-miR-148a-3p_L-1R-2_1ss5TA	CAGAGCACTACAGAACTTT	19	9	gp1a	..(((.(((((.((........((((((((......))))))))...((((((.((((((.(((......)))).)))))).))))))...)))))))))))..	1.65	0	0	0	0	0
gga-miR-181b-1-3p_L+1R-1	CTCACTGAACAATGAATG	21	17	gp1a	.(.((((.(((.((.(((	1.65	4.58	7.05	22.63	13.79	13.74

	CAA				((....<br>..((((<br>((((..<br>...(((<br>((((((<br>..((((<br>((((((<br>(.....<br>......<br>.)))))<br>))))))<br>))))))<br>)))...<br>..))))<br>).))))<br>))))))<br>..))))<br>))))..						
gga-miR-193a-3p_R-2<br>_1ss2AG	AGCTGG<br>CCTACA<br>AAGTCC<br>CA	20	Z	gp1a	.(((((<br>....))<br>.)))..<br>...	1.65	0.76	0	0.60	0	0
gga-miR-214_1ss8GA	ACAGCA<br>GACACA<br>GACAGG<br>CAG	21	5	gp1a	......<br>.....(<br>((..((<br>((....	1.65	0.38	0	0.60	0	0

					..)).))..)))...					
gga-miR-214_R-2_1ss19CA	ACAGCAGGCACAGACAGGA	19	4	gp1a	((.((((((.(((((.((((..((.((...(((..(((((......)))).)))..)).)).)))))))))...........)))))).))......	1.65	0	0	0	0	0
gga-miR-214_L+1R-3_1ss19GA	TACAGCAGGCACAGACAGA	19	14	gp1a	...((((((((....)))))))..............	1.65	1.91	0.96	0.60	0.53	0

gga-miR-214_L-2R+1	AGCAGGCACAGACAGGCAGT	20	10	gp1a	...((.((...((...(((....)))..)).....)).)).....	1.65	3.05	0	1.19	0	0
gga-miR-214_R+1_1ss13GA	ACAGCAGGCACAAACAGGCAGT	22	8	gp1a	...((((((((((((((((..(((.((((......))))))...))))))..).))))).....)))))))....	1.65	0.38	0	0.60	0.53	0
gga-miR-216b	AAATCTCTGCAGGCAAATGTGA	22	3	gp1a	.(.((((..((..((((.((((.((((((	1.65	5.34	2.57	8.34	10.61	11.77

gga-miR-217-3p_L-1	CATCAG<br>TTCCTA<br>ATGCAT<br>TGCC	22	3	gp1a	(((((.<br>((.(((<br>((((.(<br>(....)<br>)))...<br>))))).<br>)).)))<br>))))))<br>))))).<br>)).)))<br>)..)).<br>.)))).<br>).<br>...(((<br>...(((<br>...(((<br>((.(((<br>(....(<br>((((.(<br>((((((<br>(((..(<br>((....<br>....))<br>)...))<br>))))))<br>)).)))	1.65	0.76	0	0	0	1.96

					)))))).....)))))..)))..))).......						
gga-miR-221-3p_L-1R-2	GCTACATTGTCTGCTGGGTT	20	Z	gp1a	...((((((.((((((((((.......(((((......)))))..((((..(((((..........)))))..))))...)))).)))))).))))))...	1.65	3.81	2.57	11.31	3.71	4.91
gga-miR-221-3p_L-4	ACATTGTCTGCTGGGTTT	19	2	gp1a	.......((.((((((((	1.65	1.53	0.32	2.38	0.53	3.92

	C				((.((..((((((((....)))))..)))...))))))))))))).						
gga-miR-221-5p_L-1R-5_2ss21TA22TA	ACCTGGCATACAATGTAGAAA	21	chr1	gp1a	..(((...((..(((((.(((..(((.((((((((((....((((((((...))))).))))))))))))).)))..)))..)))))..))))).	1.65	0.38	1.60	0	0	0
gga-miR-223_L-4R+2	AGTTTGTCAAAT	19	3	gp1a	...(((......	1.65	0	0	0	0	0.98

	ACCCCAT				(((((((....))))))).....)))...						
gga-miR-223_L-2R+1	TCAGTTTGTCAAATACCCCA	20	Z	gp1a	...((((((......))))))................	1.65	2.67	2.57	2.98	2.65	0
gga-miR-449b-5p_R-1	AGGCAGTGTGCTGTTAGCGGCT	22	Z	gp1a	((((..((.((..(((((((((....(((.((((((((................))))))).))).))))))))).))).))..)))))..........	1.65	0	0	1.19	0	0

					....						
gga-mir-449b-p3_1ss8GC	CCACACTCCCACCTGCTGC	19	Z	gp1a	((((..((.((..(((((((((....(((.((((((((.....................)))))))).)))).))))))))))).))).))..)))).........................	1.65	0	0	0	0	0
gga-mir-456-p5	TGTGTGTGTGCGTGTGTGA	19	2	gp1a	...(((((((..(.(((.(((.((((((((((.........)))).))))))))	1.65	0	0	0	0	0

					.)))))<br>).)..)<br>).))))<br>)...						
gga-mir-456-p5_1ss1<br>0CT	GTGTGT<br>GTGTGT<br>GTGTGA	18	6	gp1a	...((.<br>(((.((<br>(((((.<br>((((((<br>(.....<br>...(((<br>(.(((.<br>....))<br>))))).<br>.....(<br>(.....<br>))..))<br>))))).<br>))))..<br>.....)<br>)).)))<br>.))..	1.65	0	0	0	0	0
gga-mir-456-p3_1ss1<br>0CT	GTGTGT<br>GTGTGT<br>GTGTGA	18	3	gp1a	......<br>((((((<br>..((((<br>.((...<br>......	1.65	0	0	0	0	0

					......<br>......<br>......<br>......<br>))))))<br>..))))<br>))...						
gga-mir-456-p3	CTGTGT<br>GTGTGC<br>GTGTGT<br>GA	20	5	gp1a	...(((<br>(.....<br>.(((.(<br>((....<br>)))...<br>)))...<br>...)))<br>).....<br>....	1.65	0	0	0	1.06	1.96
gga-miR-551-5p	GAAATC<br>AAGGGT<br>GGGTAA<br>GACCT	23	9	gp1a	...(((<br>((((..<br>(((((.<br>((((((<br>(((..(<br>((((..<br>...(((<br>((((..<br>...)).<br>))))).	1.65	2.29	5.77	11.91	3.18	1.96

					.))))) ..))))))))).))))). .)).)))))...						
gga-miR-1451-5p_R+1 _1ss1TN	NCGCAC AGGAGC AAGTTA CCGCA	23	chr3	gp1a	..((((((((.((((((((((((((((.(((((.(((.((((((.(((...... .....))).)))))).))).))))).)))))))))))))))))))... ..))))).	1.65	0	0	0	1.06	0
gga-miR-1451-3p_2ss	CGTAAN	23	chr3	gp1a	..((((	1.65	0	0	0	1.06	0

6CN23CT	TCGCTG<br>CTGTGA<br>GAGGT				((((.(<br>((((((<br>((((((<br>(((.((<br>(((.((<br>(.((((<br>((.(((<br>......<br>.....)<br>)).)))<br>))).))<br>).))))<br>).))))<br>))))))<br>))))))<br>)))...<br>..))))<br>).						
gga-miR-1452_L-1	TGAGAT<br>AAGACA<br>GAGGAT<br>ATT	21	Z	gp1a	.(((((<br>((....<br>((((((<br>(((.((<br>((((((<br>(((...<br>......<br>......	1.65	0	0.64	1.19	2.12	3.92

					)))))))).))).))))))))).))))))))						
gga-miR-1453	TGGGCAGCCTCTCAGTGAGCTCT	23	20	gp1a	....(((.((((((((((..((((((((((((....))))....)))))))))))))....((.((((((....(((((....((.((((...)..))))))..))))))))))))))).(((......)))	1.65	8.39	2.57	4.76	8.49	5.89

					..))))))))))..						
gga-mir-1457-p3_1ss19TA	ACCTCCTTGGGCAACCTGA	19	2	gp1a	..((((((...........((((((....(((....))).....))).)))..((.((((.........))).))........)))))).......	1.65	0	0	0	1.06	0
gga-mir-1457-p5_1ss13GA	CAACCTCCTTGGACAACC	18	24	gp1a	.........((((....))))...	1.65	0	0	1.19	0	0
gga-mir-1457-p5_1	ACCTGTTCCAGT	20	4	gp1a	..........((	1.65	0	0	0.60	0	0.98

	GCGTCACC				(...)))...						
gga-mir-1457-p5_3	TCCTTGGGCAACCTGTTCC	19	Z	gp1a	...(((..((....))......)))...	1.65	0	0	0.60	0	0.98
gga-miR-1457	CACTACAGGCTGCAAAAGGGTT	22	2	gp1a	.................((((((.(((((....)))))).((((((..(((......))))))))).)))))...	1.65	0	1.28	0	0	0
gga-mir-1457-p5_1ss4AG	ACAGCCTCCTTGGGCAACCT	20	14	gp1a	....((((.....((....)).((((.....)))))	1.65	0.38	0	0	1.06	1.96

					)))...						
gga-mir-1457-p5_4	TGGGCAACCTGTTCCAGTGCGTC	23	AADN03011115.1	gp1a	...((((((.(((((...(((......))).....)))))........)))))))...	1.65	0	0	0.60	0	0.98
gga-mir-1457-p5_1ss8AG	ATCCACAGCCTCCTTGGGCA	20	3	gp1a	.......((((....))))....	1.65	0	0	0	0	0
gga-mir-1457-p3_1ss16AG	CAACCTCCTTGGGCAGCCT	19	1	gp1a	...(((((..(((....(((..(.....)..))).((....))....))))..)))))....	1.65	0	0	0	0	0

gga-mir-1457-p5_2	CAACCTCCTTGGGCAACC	18	4	gp1a	...((((((..(((((((((.((((((((..........))))))).((((..(((.....))).)))).))))))).)).........))))))...	1.65	0	0	0.60	0	0.98
gga-mir-1457-p5_1ss3CT	TCTTTGGGCAACCTGTTCC	19	9	gp1a	.....((....)).(((((....((((((.............))))))..))	1.65	0	0	1.19	1.06	0

					)))...						
gga-mir-1462-p3	GAGTGAGTGCTGCTGTGTCTCT	22	4	gp1a	...(((((.(((...(((((..((((((.(((((((.((......................)).))))))))..)).)))..))))...))).)))))......	1.65	0	0	0	0	0
gga-miR-1464_R-1	TGCTGTTTGCAGGGCCGCCTCGG	23	15	gp1a	.(((((((.((.((.((((((.(((....)))))).))))).))....((((..((	1.65	0	0	0	1.06	1.96

					((.((((.(((....))))))).))))..))))).)).)))))))......					
gga-miR-1550-3p	CTTCACTTACACTGCTCACTGGT	23	12	gp1a	...(((((.((((((((((..((((((((((((.((((...........)))).))).)))))).))))..))))))))))))))...	1.65	0	0.64	0	0	1.96
gga-mir-1563-p5_2	GAAACTGACTCT	23	3	gp1a	........((.(	1.65	1.14	1.28	0.60	1.06	1.96

	GTATGAAGTGC				(..((....((..((((((....))))))..))....))..))).))...						
gga-mir-1570-p5_1ss17GA	AGAAGGGTGTGCAGAAAAA	19	3	gp1a	....(((.(((((((((.....((((((((((((......)))))).((.......))....))))))).....)))))).))))))...	1.65	0	0	0	1.06	0
gga-miR-1570_L-4R-1	GGTGTG	18	chr7	gp1a	......	1.65	0	0	0	0	0

_1ss12AT	CTGAAG<br>AATGTG				..((.(<br>(..(((<br>((((((<br>((((((<br>.(((..<br>(((.((<br>(..(((<br>((...)<br>))))))<br>).))).<br>.))).)<br>))))))<br>)).)))<br>))).))<br>.))...<br>....						
gga-mir-1575-p3	TAGTTC<br>TGTCCT<br>GACTCT<br>ACACT	23	4	gp1a	..((((<br>((((((<br>((....<br>....))<br>..))))<br>))))))<br>((((((<br>((.((.<br>......<br>.)).))	1.65	0	0	0	1.06	0

					)).)))<br>).....					
gga-miR-1579_L+1R-1<br>_1ss21CA	CCGTGG<br>CGGGAA<br>GGGATG<br>GGA	21	6	gp1a	((((((<br>((((((<br>(((((.<br>.((...<br>.))..)<br>))))))<br>.....)<br>)).)))<br>))))..<br>......<br>..	1.65	0	0	0	0	0
gga-miR-1583	CGTAGG<br>GCCGTG<br>GGACGG<br>CA	20	Z	gp1a	.(((((<br>(((((.<br>..((((<br>((.(((<br>.((.((<br>((((((<br>((((((<br>((((..<br>..))).<br>))))))<br>))))))<br>))).))<br>...)))	1.65	1.53	0	0	1.06	0

					.)))))<br>).))))<br>.)))))<br>).						
gga-miR-1592_R+2	CACTGC<br>CTCAGA<br>CAACGG<br>GTGTGT	24	19	gp1a	...(((<br>(((...<br>((((((<br>..((..<br>((((((<br>(((((.<br>.....(<br>((((..<br>.....(<br>((((..<br>((((((<br>(.((.(<br>((((((<br>(.....<br>......<br>..))))<br>)))).)<br>))))))<br>.)))))<br>))))))<br>)....)<br>))))..	1.65	0.76	0	1.19	2.12	0

					))))))<br>..))..<br>))))))<br>...)))<br>))).						
gga-mir-1602-p5	TGGGGG<br>TGCTGC<br>GAAGCC<br>TGCAGC	24	4	gp1a	...(((<br>(.((.(<br>((((..<br>((((((<br>.(((..<br>((((..<br>((((((<br>((....<br>))).).<br>))))))<br>))..))<br>).))))<br>))..))<br>))))).<br>))))..<br>.	1.65	0	0	2.38	0	0
gga-mir-1604-p5_1ss<br>13GA	AGGGCT<br>GTGCTG<br>AAGACA	18	21	gp1a	......<br>..((..<br>.(((((<br>(....)<br>))))))	1.65	0	0	0	1.06	0

					).						
gga-mir-1612-p3_1ss1CT	TTCTTTTCTTTCTTTCTTT	19	Z	gp1a	((((((.((((((.....)))))).(((.((((((((((.....(((.....)))...))))))....)))))).))))))))................	1.65	0	0	1.19	0	0
gga-mir-1612-p5_1ss5AG	GAGGGAGGAAAAGGAGCT	18	9	gp1a	...........(((.((((.(((..(((..(..(((.(((((((((((	1.65	0	0	1.19	0	0

					.(((((....)).))).)))))))))))..))).)..)))))))))))))))						
gga-mir-1613-p3_1ss19GA	ACTGATGTAGTGAAGATGA	19	5	gp1a	...(((.(((((....))))).)))).............	1.65	0.38	0	0	0	0
gga-miR-1619_L+1R-2	AAGAATTCATAGCAATACGGA	21	20	gp1a	...(((.(((((.....(((((((((((((((((((((((((..(((..((((((.((....	1.65	1.53	0	0	0	0

					....))<br>.)))))<br>))))..<br>))))))<br>))))))<br>))))))<br>))))).<br>.)))))<br>.)))..<br>.						
gga-miR-1626-5p_L-1R-5_1ss11AT	TTCCATGGCTGACTTTCT	18	chr1	gp1a	((.(..((((((((..((((.((((.((.((((((.....(((((...))))))))))))))))).))))..))))).)))..)))	1.65	0	0	0	0	0
gga-mir-1630-p3_1ss	CTTTCT	20	9	gp1a	......	1.65	0	0	1.79	0.53	1.96

7AG_2	GAAGGG	......
	GGAGGA	....((
	AA	((((((
		(..(((
		((....
		.)))))
		...(((
		(((...
		..)).)
		)))...
		))))))
		)))(((
		(((((.
		.....(
		((((.(
		....).
		......
		(((((.
		.....)
		))))))
		))).((
		(((...
		......
		))))).
		))))))
		))..

gga-mir-1630-p3_1ss7AG_1	CTTTCTGAAGGGGGAGGA	18	11	gp1a	((.(((..(....)..)))))..	1.65	0	0	1.79	0.53	1.96
gga-mir-1630-p5_1ss7AG_2	CTTTCTGAAGGGGGAGGAAA	20	9	gp1a	...((((..((((((((((((.........))))))))))))..)))))...	1.65	0	0	1.79	0.53	1.96
gga-miR-1635_R-3	TGCCCAGGCTGTGCTGTGCTCT	22	21	gp1a	...(((((.(((((.(..(((((((((((..(((((..((((((..............)))))).))))))))))))	1.65	0	0.64	5.95	2.12	3.92

					)))))).<br>.).)))<br>.)).))<br>)))...<br>....						
gga-mir-1639-p5	GCACTG<br>AGCACA<br>GGGGAA	18	2	gp1a	......<br>((((((<br>((.(((<br>((....<br>.(..((<br>.(((((<br>....))<br>)))..)<br>)..)..<br>.)))))<br>......<br>...(((<br>(((...<br>.)))))<br>).....<br>))))))<br>)).	1.65	0.76	0	0	1.06	0
gga-miR-1647_R+1	TCTCTG<br>CCTGAG<br>GGGTAC<br>TCTA	22	28	gp1a	....((<br>((((((<br>((((((<br>.((.((	1.65	0	0	1.19	1.06	0

					((.(..(((((..(((((((((.........)))).))))))..))))) ..).))))).)).)))))).))).)).)))....					
gga-miR-1648-5p_L-1R-1_1ss17CG	GGCTCGGCTCGGCTCGGCT	19	1	gp1a	...((((((((((.(((.((((......))))..((((....))))...))).))))))))))...	1.65	0	0	0	0	0
gga-miR-1648-5p_L-1	GGCTCG	18	12	gp1a	...((.	1.65	0	0	1.19	0	0

R-2_1ss10TG	GCGCGGCTCCGC				.((((((((((.((((((((...)))))).))))))))))..))...						
gga-miR-1649-5p_R+2	TCCTGCAGAAGGTGCGGCTGTG	22	3	gp1a	...(((((..(((((...((((((((..(..(((((((....))))))......))))))).))..)..))))))))..))))).)))))....	1.65	0	0	0	0	0
gga-miR-1651-5p	GCTAAGACTGATAAAAGC	22	1	gp1a	...(((((((..(((.((	1.65	3.05	1.92	3.57	3.18	0

	AAGC				(..((.<br>(..(((<br>((((((<br>(((...<br>......<br>...)))<br>))))))<br>)))..)<br>.))..)<br>)).)))<br>..))))<br>)))...						
gga-mir-1653-p5_1ss23GT	TGTATGCCCAGCACTGGTCCTGT	23	9	gp1a	((.(((<br>((((((<br>((..((<br>(....(<br>((....<br>)))..)<br>))...)<br>))))))<br>))))..<br>.))...<br>((((((<br>....))<br>))))((<br>((((((	1.65	0.76	2.57	1.19	0	1.96

					(.((..<br>..)).)<br>))))))<br>)).						
gga-mir-1673-p3_1ss<br>14GA	CACTCT<br>CACTGC<br>GAGGCC	18	15	gp1a	...(((<br>(.(((.<br>.(((((<br>(((((.<br>.(....<br>...)..<br>))))))<br>).))).<br>.)))))<br>))....<br>.(((((<br>((.(((<br>......<br>......<br>..))).<br>))))..<br>...)))	1.65	0	0	0	0.53	0
gga-mir-1676-p5	GCTCCT<br>CTCTGG<br>CAGCAC<br>CGAGAG<br>CG	26	5	gp1a	..((((<br>((((..<br>(((..(<br>((.(((<br>(((((.	1.65	0	0	0	0	0

gga-miR-1677-5p	TCCTGC<br>ACCGCT<br>GAAGTC<br>AAT	21	3	gp1a	....((<br>(((((.<br>((((((<br>((.((.<br>.((...<br>.))..)<br>))))))<br>))).))<br>))))).<br>.....)<br>))))))<br>)...))<br>).....<br>)))..)<br>))).))<br>))..<br>((.((.<br>.(((((<br>((.(((<br>((((((<br>((((((<br>(.....<br>...)))<br>))))))<br>))))).<br>)).)))	1.65	1.53	1.92	10.72	2.12	1.96

					))))..<br>)).))						
gga-miR-1683_L+1	TTCTGG<br>GACAGT<br>CACAGC<br>ATCTTT	24	1	gp1a	...(((<br>(((.((<br>..(((.<br>..((..<br>(.((((<br>((((..<br>(((((.<br>((.(((<br>(.(((.<br>....))<br>))))).<br>))))))<br>)....)<br>))))))<br>).)..)<br>)....)<br>))..))<br>))))))<br>...	1.65	0	0.64	3.57	0	1.96
gga-miR-1684a-5p	AGCTCT<br>GCTTCC<br>TCATAC<br>ATAC	22	AADN03<br>024942<br>.1	gp1a	......<br>(((..(<br>....((<br>..((.(<br>((((((	1.65	8.39	0	1.19	6.37	0

					.((((.<br>.(((((<br>(((((.<br>((..((<br>(.....<br>..))).<br>.)).))<br>))))))<br>))..))<br>)).)))<br>)))).)<br>)..)).<br>.)..))<br>).						
gga-miR-1685-5p_R+1	TGGAGT<br>CACTAC<br>CAGTGC<br>TGTGT	23	2	gp1a	...(((<br>(((((.<br>...(((<br>(((.((<br>(((.((<br>(((((.<br>...((.<br>......<br>))...)<br>))))))<br>.)))))<br>)).)))	1.65	0.76	3.21	0	2.12	0

					)...))))))))...						
gga-miR-1689-3p_L+1R+2	CCCGGTGACTGGAAGCAGCGTTGACT	26	19	gp1a	...((((((.((.(((((.((((..((((((...((....))...))))))..)))).))))))....))))))))..	1.65	0	0	0	1.06	0
gga-mir-1693-p3	AATGGTGTCTGGGCAAAGGATGAAGC	26	1	gp1a	..(((((((..(((.(.(((((....((((...((.(((((((....)).))))	1.65	0	0	0	0	0

					).))..))))))))))))))))))))......						
gga-mir-1700-p5	CCTTTGCTATGCCTATGATGGGT	23	1	gp1a	............(((((..(((.(((....((((.(((.(((((((.........))))))).))).))))...)).))).)))))...	1.65	0	0	0	1.06	0
gga-miR-1710_L+1R-2	TTCATCTGCTGCATAACCATC	21	5	gp1a	(((((.(((((.((((((((((...	1.65	1.53	0	1.19	3.18	1.96

					((((.((..... ..)).))))...))).))))))))))).)))))						
gga-mir-1716-p3_1ss 22CT	CCCGGC TTCCTG CCGCCC TGGT	22	5	gp1a	..(((((.((.. (((((. .(((((((((.. .((((((.(((((.....).)))) .))))))...))))))))).))))).)).)))))).. .	1.65	5.34	0	6.55	1.06	2.94
gga-miR-1717	ACTCTC TAACCT	23	3	gp1a	...(((.((.((	1.65	0	1.92	1.19	0	0

	GACAGA<br>AAACT				((....<br>((((((<br>(.((.(<br>((((((<br>((....<br>.(((((<br>.(((((<br>(((((.<br>.....)<br>))).))<br>)))).)<br>))))..<br>...)))<br>))))))<br>))..))<br>))))).<br>.)))).<br>))))).<br>..						
gga-miR-1720-5p	TGATCA<br>CCTCGG<br>ACGTTG<br>CTTT	22	chr10	gp1a	(((.((<br>((((((<br>(...((<br>((((((<br>((....<br>......<br>....))	1.65	0	0	2.38	5.30	5.89

					))))))<br>)).)))<br>)).)))<br>).)))						
gga-mir-1724-p5_1ss<br>21CT	AACCCG<br>GCTCCG<br>CTCAGG<br>TCT	21	23	gp1a	.((.((<br>(.((((<br>(..(((<br>...)))<br>..))))<br>).....<br>...(((<br>((.(((<br>..((.(<br>((.(((<br>(((((.<br>(.((..<br>....))<br>.)))))<br>))))))<br>)..(((<br>(.....<br>.)))).<br>.))..)<br>)).)))<br>))..))<br>)))...	1.65	0.76	0	3.57	3.18	3.92

gga-miR-1737	CAGCAC TGCTGC GCTCGG TG	20	10	gp1a	... .(((.(((.(((.((.((((((((((.((((. ...)).)))))))..... .....(((((.. ..)))))..... ...))))).))))).))) .)))	1.65	3.05	3.85	5.36	3.18	1.96
gga-miR-1740-5p_L+1 R-1	ATGTAG TTTAAG AGGATA GGTCT	23	3	gp1a	...((((((((. .(((.((((((. .((((. ((((..	1.65	0	0.64	1.19	3.18	3.92

					..))))))))..)))))).)))..))))))))...						
gga-miR-1751-5p	TGAGCTGCTCTGTTCCTGCATCC	23	4	gp1a	...(((((.(((((((.(((..((((((((..((((.((((((((..................)))))))).))))..))))))..)).))))).))))))...	1.65	3.05	0.64	4.76	7.43	3.92
gga-miR-1758_L-1R-2	CACCAAGCACTTGTCGGGCT	20	24	gp1a	.........((((....))))(((	1.65	0	0	0	0	0

					(((((.(((((............(((((((......))))))))))))))).))))))))...						
gga-mir-1766-p5	TTGGCTGTCTTTCTCTTGT	19	2	gp1a	...((..(.........)..)).((((((.((..(((.((((......)))).)))...))..))))))...	1.65	0	0	0	0	0
gga-miR-1769-3p_R-1	AGTGTGAAATCTGCCTGA	22	15	gp1a	...((((((((((((((((	1.65	8.39	6.41	2.38	7.43	3.92

	AAGT				(((... (((..((((.(((((((. ....((((((((((.... ....))))....)))))) .....))))))) .)))). .)))))))))).. ....))))))). .))))) ...						
gga-mir-1771-p5	CAGAGA GTCTCA GAGGCA CT	20	5	gp1a	((((.. (((..((((.(((..... .((.(((((..(((..((	1.65	0	0	0	0	0

gga-miR-1772-3p_R+1	TCGTTGTCTGTTCGGTGGCAGT	22	6	gp1a	(((((.((....((((((.((((..(........).)))))))))).))..)))))))..))).)))))))..))))))))..)))....)))).....((((((((((((((.((((((.......... ..)))))).))))).....)))	1.65	0	0	1.19	0	0

					))))))..						
gga-mir-1784-p5_1ss11TC	AGGAGCAGAACTGGGACC	18	8	gp1a	...(((..((((((....(((((.....(((..(((((..(..(.(((......(((((.....))))).......)))..)..)..)))))..)))..)))))....))))))..)))	1.65	0	0	0	1.06	0
gga-miR-1787_L-2R+1	TTGTTTACGGAAGGGGGAA	19	chr12	gp1a	(((.((..((((((((((((.(((((...	1.65	0	0	0	0	0

					.((..((..((.......))..)).))....)))))..))))))))))...))..)))						
gga-mir-1787-p5	TTTCCCCCTTCCCTAAACCTCT	22	12	gp1a	....((((((((.(((((....((..((..((.......))..)).))....)))))).)))))))))...	1.65	0	0.64	0	0	0
gga-miR-1788-5p	GGCTTGTTTTCCGTTGCCTGCG	22	7	gp1a	(..((((((((((((((.((((	1.65	0.76	0.64	8.34	13.79	11.77

					((((.(((...........)))))))))))))))))))))))..)						
gga-mir-1797-p3_1ss19TA	GCAGGAACTGCACAGCTGA	19	26	gp1a	...((.((((((...(((..((((.(((..(((((((.((......)).))).))))..))).)))..))).....))))))).	1.65	0	0	0	1.06	1.96
gga-miR-1805-5p_L-1	AGTTGTAGTCTT	21	1	gp1a	...(((((((((	1.65	0	0	1.19	1.06	0

	TCAAAC<br>AGA				((....<br>...(((<br>(..(((<br>.(((((<br>((((((<br>(.((((<br>((((((<br>.....(<br>(.....<br>.))...<br>)))).)<br>))))).<br>))))))<br>))))))<br>.))).)<br>))))))<br>))).))<br>)))...						
gga-miR-2128_R+3	TGCAGT<br>GACGTC<br>TCTTCC<br>CCGCA	23	10	gp1a	..((((<br>((((.(<br>((((((<br>(.((((<br>(((((.<br>......<br>))).))<br>)))).)	1.65	0.76	0	0	0	0

					)).)))))..)))))))..						
gga-mir-2129-p5	AGTGGGACAAGGACCAGGATACTGC	25	14	gp1a	..........((..((((((.((((....((((..(((((...)))))))))..))))))))))..)).	1.65	0	0	0	1.06	1.96
gga-miR-2129_L-1	AGCAGGACTGGCTTTGTTACGA	22	14	gp1a	..........((..((((((.((((....((((..(((((...)))))))))..))))))	1.65	0	0	1.19	0	0

					))))..<br>)).						
gga-miR-2130_R-2	TCCCAG<br>TGGAGC<br>TCTGCA<br>AGG	21	JH3762<br>68.1	gp1a	((((.(<br>((((((<br>.(((..<br>.(((((<br>(..(((<br>......<br>.)))))<br>))))..<br>))).))<br>.)))))<br>.))))	1.65	0	0	1.19	0	0
gga-mir-3523-p5	GAGTGC<br>GAGGCG<br>CTGCGG<br>GGGCT	23	13	gp1a	(((...<br>..))).<br>.....(<br>(....)<br>).((((<br>((((((<br>(((...<br>(((..(<br>((((((<br>((((((<br>(.((.(<br>((....<br>.)))))	1.65	0.76	0.64	1.19	5.30	3.92

					.)).)))))))))))))..))).......((..................)))))))))))))))))						
gga-mir-3524a-p5	ATTCTATGATTCTCTGAT	18	4	gp1a	....(((((....(((........))))))))...	1.65	0	0	0	0	0
gga-mir-3524b-p5_1ss9AG	ATTCTATGGTTCTGTGATG	19	1	gp1a	........(((((.(((((((((.....))))..))))).))))...	1.65	0	0	0	0	0
gga-mir-3524a-p3	ATTCTA	18	4	gp1a	.(((((	1.65	0	0	0	0	0

	TGATTCTCTGAT				(((....))))).))..						
gga-mir-3524b-p5_1ss3TC	AGCCATTCTATGATTCTGTG	20	1	gp1a	...(((...(((((...........))))).)))...	1.65	0	0	0	0	1.96
gga-mir-3525-p5_1ss18TC	AGAGAATCATAGAATGGCC	19	4	gp1a	............(((((....((((((.(.....))))))).........(((((.((.......))))))..)))))...	1.65	0	0	0	0	0.98
gga-miR-3529	AGGCAGACTGTG	21	10	gp1a	...(((((((.(	1.65	4.58	3.85	16.08	14.85	13.74

	ACTTGT<br>TGT				((((..<br>((.(((<br>((((((<br>.(((((<br>((.(((<br>((....<br>.)).))<br>).))))<br>)))..)<br>))).))<br>))).))<br>..))))<br>)..)))<br>))))..<br>.						
gga-miR-3531-5p	TCCTTG<br>TTTGGG<br>TTGTAA<br>TGA	21	23	gp1a	...(((<br>((((((<br>((((.(<br>((((.(<br>((((.(<br>((((((<br>.(((..<br>(.....<br>.)..))<br>).))))<br>))).))	1.65	1.53	0	0	1.06	0

					))))))<br>))...)<br>))))))<br>))))))<br>...						
gga-miR-3532-5p_R-4<br>_1ss18CA	GTTGCA<br>CTGCAG<br>CTGCTA	18	chr5	gp1a	.(((((<br>(..(((<br>..((((<br>((((((<br>((..((<br>..(((.<br>(((...<br>....))<br>))))..<br>)).)))<br>))))))<br>).))..<br>)))..)<br>))))).	1.65	0	0	0	0	0
gga-mir-3594-p5	ATGTCA<br>GAGCAG<br>TGACCC<br>C	19	JH3763<br>03.1	gp1a	......<br>..((.(<br>(.((.(<br>((...(<br>(..(((<br>((((((<br>((.(((	1.65	0	0	0	2.12	0

gga-mir-6542-p5	TGGCGCCAGTGTCTGCCAGCATCAGC	26	3	gp1a	((.((((.......))))....)))))))....))))))))))..)).. .))))))).)).........................(((((.(((((((((..((((((...(((..(..(........)..)....)))...)))))).))))).)))))).))	1.65	1.53	0	1.19	1.06	0

					)))...						
gga-mir-6543-p3_1ss12TG	CTGAGAGAAGAGGCTGACTC	20	Z	gp1a	...(((((((((..((((((.(((.(((.....))).)))......(((((....))))).................))).))))).))))))...........	1.65	0	0	0	0	0
gga-miR-6543-3p_L+4R+4	CACAGCCTCCTTTCAGGTCACTGTAG	26	AADN03022228.1	gp1a	......((((..(..(((((((.((.(((((((((..((.((((..((	1.65	0	0	1.19	0	0

					.....)<br>)...))<br>)).)))<br>))))))<br>)).)).<br>)))).)<br>))..).<br>))))..<br>......<br>......						
gga-mir-6546-p5_1ss1TC	CGTGCTGCTGCAGGGCTCTG	20	13	gp1a	...(((<br>(.((((<br>..((((<br>.(((((<br>((((((<br>(.....<br>..(((.<br>....))<br>).((((<br>....))<br>))..))<br>))))))<br>)..)))<br>.)))).<br>))))))<br>))...	1.65	0	0	0	0	0

gga-mir-6546-p3_1ss1TC	CGTGCTGCTGCAGGGCTCTG	20	15	gp1a	...(((...(((((((..(((((....))))) ..(((((....)))))....)))))))))))	1.65	0	0	0	0	0
gga-mir-6548-p3_1ss11CT	TTTTTGCCAGTGATGAACA	19	7	gp1a	...((((.((.(((.... .((((. .((((((.((((((((((((.... .))..))))))..)))).)).))))..)))) .))).)).)))	1.65	0	0	0	0	0

					)...						
gga-miR-6549-5p	AGCCTT CTGTTG TGCATC TGAGA	23	8	gp1a	...(((((((.. .(((.((..((((((.((((((((((.((((.(((. ((((.. .(((((...))))).)))).))).))))))))))))))...)))))). .)).)))..... ...)))))))... .	1.65	0	0	0	1.06	1.96
gga-miR-6550-3p_L+1	GGCTCC ACCCTC GGCTGC	23	10	gp1a	..((((((((((((((((	1.65	2.29	0.64	2.38	4.24	0

	TTTGA				..(..(						
					.(((..						
					..((.(						
					(((.((						
					((((((						
					((((((						
					(((.((						
					(....(						
					(..(..						
					......						
					)..)).						
					....))						
					).))))						
					))))))						
					))))))						
					).))))						
					.)))))						
					)..)..						
					)))).)						
					))))))						
					))))).						
					..						
gga-miR-6551-5p	CGGTGC	22	18	gp1a	...(((	1.65	0.76	0	0	1.06	0
	ATCTCT				(((...						
	GCAGCC				...(((						
	CGCA				((((((						

					((((.((..((((((((((((((.(((...((......))..)))))))))).))))))))..))))))))))))))......))))))...						
gga-mir-6554-p5_1ss6TA	GATACACACTGATTCTCC	18	1	gp1a	...((((.(((((....((((....))))....))))))))...	1.65	0	0	1.19	2.12	5.89
gga-miR-6555-3p_1ss21GT	AATGTGGCCTCA	21	12	gp1a	...........(	1.65	0	0	0	0	0

	ACAGAGCAT				((((((((..........))))))))))..					
gga-miR-6558-5p_L-1R-2	AGTGAAACGTTGAGGTGGCTT	21	9	gp1a	..........((.(((...))).))(((((((..((((.(((..((((((((.....))))))))))))..))))..))))))...	1.65	0	0	0	1.06	0
gga-mir-6561-p3	TGTGGCCCTCTGCTGGACTCT	21	2	gp1a	...(((...((......(((((......)))))........))	1.65	0	0	0	0	0

					.))).....					
gga-miR-6561-3p_L+1R-1_1ss19AC	AGGGAGCCCAGAACTGGACACA	22	chr8	gp1a	....((.((((..(((((((.(((((((...((.((((((((...................))))))))))))....)))))))).)))...))))..)))))).........	1.65	0	0	0	0	0
gga-miR-6564-3p_R+1	TGGACGGAGGAAGCGCGGGCAGT	23	20	gp1a	...((((..(..(((((.((((((((((((((..((	1.65	0	0	0	1.06	0

gga-miR-6570-3p_R+2	AGAGGA<br>GAGCCT<br>GTGGGC<br>CAGAGA	24	chr1	gp1a	((....<br>......<br>))))..<br>))))))<br>))))))<br>).))))<br>)).)..<br>))))..<br>..<br>......<br>(((((.<br>.(((((<br>..((.(<br>((((.(<br>(.(((.<br>..((((<br>(((...<br>.)))))<br>))..))<br>).)).)<br>))))))<br>...)))<br>)).)))<br>))....<br>((....<br>.)).	1.65	0	0	0	0	0

gga-miR-6572-5p_R-1	TTTTCCCTCTCTGTGCAGTTCT	22	2	gp1a	............((((((((..((((.(((((.((..((((((.....................))))))).)))))).)))).))))))))).....	1.65	0.76	1.28	0	5.30	3.92
gga-mir-6572-p3_1ss22AT	GACAGATAGGGTGAGGAGGACTGACA	26	2	gp1a	............((((((((..((((.(((((.((..((((((.....................))	1.65	0	0	1.19	2.12	1.96

					))))).))))).)))).))))))))))).......						
gga-miR-6590-5p_R-4_1ss5AG	TGGGGTCAGGTGCTGAAAT	19	chr5	gp1a	(((((((..(((....)))...))))))).(((((((..(.(((.(((((...........))))).))).)..))))))))....((((.........)))).	1.65	0	0	0	0	0
gga-miR-6590-3p_R-1	TTACTTCTGTTC	21	5	gp1a	((((((((((..	1.65	1.53	3.21	8.34	2.12	0

	CTGATCATC				(.(((.(((((...........))))).))).)..))))))))).)))...						
gga-mir-6592-p3	GAGGCCGTCAGCCTGGAGC	19	9	gp1a	...(((((((.((((.....))))...))))))))...	1.65	0	0	0	0	0
gga-mir-6592-p5	GAGGCCGTCAGCCTGGAGC	19	19	gp1a	...((((((.(((..(((((((((...((((..((((((.((......)).))))))..)))	1.65	0	0	0	0	0

					)...))))))))..)))))))))...						
gga-mir-6593-p5_1ss10GC	GGGTGCTGGCAGGGCTCT	18	15	gp1a	...(((((((((.((((((....((.....))..))))))....))))))))))....	1.65	0	0	0	0	0
gga-mir-6594-p3_1ss22GT	TCAGCTCATGTCATCCCACAGT	22	4	gp1a	..(((((((.(((...(((....((((.....((..((((((((((((((((.(..(((((.....	1.65	2.29	3.21	5.95	11.67	5.89

					.)))))						
					..).))						
					))))))						
					))))))						
					))))..						
					))))..						
					))))))						
					.)))))						
					))..						
gga-miR-6598-5p_L+2	GCTGCA	21	chr5	gp1a	...(((	1.65	0	0	0	0	0
R-2	GCAAGG				.....(						
	GAGAAG				(((...						
	TCT				((((((						
					((..((						
					(((..(						
					((.(((						
					((((..						
					.....(						
					((....						
					.)))..						
					..))))						
					).)).)						
					))..))						
					)))..)						
					))))))						
					))))).						

					....)))。						
gga-miR-6601-3p_R+1	GATGTCTGTGGCTCTGGGCAGA	22	2	gp1a	...((((((((.((((.....(((((((.........)))))))....))))).))))))))...	1.65	0	0	0	0	0
gga-mir-6603-p5_1ss19AG	AAAAGAGAGGAGATTTAGGT	20	2	gp1a	...((((((..(((..(((((....)))...))..)))..)))))).....	1.65	0	0	0	0	0
gga-mir-6603-p5_1ss13AG	AAACTAAAAGAGGGGAGAT	19	4	gp1a	...(((((((((.....((....	1.65	0	0	0	0	0

					...)).......))).)))))))...						
gga-mir-6603-p3_1ss12AG	AACTAAAAGAGGGGAGATTTA	21	4	gp1a	...(((((((((((.....((.......)).......))).)))))))...	1.65	0	0	1.19	1.06	0
gga-miR-6604-5p_1ss7GA	TGGCACAGTGCTAGGGATTTCTGT	24	6	gp1a	.(((((((..(....(((.((..((((.(((((...(((((((.((...((((....)))))))))))))..	1.65	1.53	3.21	5.95	9.55	9.81

gga-miR-6609-3p_L-1<br>R-3_1ss5CT	CAGTCC<br>CTGTGC<br>TCTGAC<br>C	19	chr1	gp1a	...)))<br>)).)))<br>)..)))<br>))..).<br>.)))))<br>))....<br>..((((<br>..((((<br>(...((<br>(((...<br>..))))<br>)..)).<br>.)))..<br>))))..<br>.(((..<br>((((((<br>.(..((<br>(...((<br>((((((<br>((.((.<br>((((((<br>(((...<br>...)))<br>))..))<br>))..))<br>.)))).	1.65	0	0	0	0	0

					))))))...)))..).))))))......))........						
gga-mir-6610-p3	AGCTGTGGGTGGCTTTGGAGAT	22	2	gp1a	...((((..((.(..((.((((((((((...(..(.(((((((((.........))....)))))))..)....)))))))))..).))..))))...	1.65	0	0	0	1.06	0
gga-miR-6615-3p	TGGCACTGATGT	22	28	gp1a	.((((((((((.	1.65	0.76	5.77	5.95	12.73	9.81

	GTTCTC<br>CACA				((((((<br>....((<br>(((((.<br>.((...<br>..)).)<br>))))))<br>.)))))<br>))))))<br>.))).)<br>)..						
gga-miR-6634-5p_R+1<br>_1ss10TC	TCTCAG<br>GGTCCT<br>GAAGGA<br>GCTGGC<br>T	25	chr3	gp1a	.(((((<br>((....<br>))).))<br>))....<br>(((((.<br>......<br>(((.((<br>((.(((<br>((.((.<br>......<br>)).)))<br>))..))<br>)).)))<br>......<br>...(((<br>((....	1.65	0	0	0	0	0

					))))).)))))..					
gga-mir-6635-3-p3_1ss6GT	GAAGTTCTGTGCTGTTTGTC	20	Z	gp1a	...((((.((((..((((((.......))))).)))))..)))))..........	1.65	0	0	0	0	0
gga-miR-6639-5p_L+1	TTACAGAACTTCGGCATGATTGCT	24	19	gp1a	.......(((.((((((...(((.....)))..((((.(((((((((..........(((((...(((((.(((((....)))))	1.65	0	0	1.19	0	0

					))))).<br>))))).<br>......<br>..))))<br>))))))<br>))))))<br>)).)))<br>....						
gga-mir-6641-p3	AAAAGC<br>TATGTG<br>CAAGGT<br>GACAG	23	1	gp1a	..((.(<br>..((((<br>.((...<br>((((((<br>(((.((<br>((((((<br>....((<br>(((...<br>))))).<br>.)))))<br>))))))<br>))))))<br>...)).<br>))))..<br>))).	1.65	0	0	0	0	0
gga-miR-6643-5p_L-1	CAGGGC<br>TGGCAG<br>GGGAGG	20	14	gp1a	...(((<br>(((((.<br>..((((	1.65	0.76	0	2.38	0	1.96

	GT				((((((<br>((((((<br>(..(((<br>......<br>.....)<br>)).)))<br>))))))<br>))))))<br>))..))<br>.)))))<br>)...						
gga-miR-6645-5p	TGGGAG<br>GTAGCA<br>GTGGTG<br>GTGA	22	5	gp1a	...(((<br>((....<br>.(((((<br>(..(((<br>(((((.<br>(((((.<br>..(((.<br>((((..<br>..))))<br>.)))..<br>..))))<br>).))))<br>))))..<br>))))))<br>))))).	1.65	0.76	0.64	0	0	0

					..<br>...((.						
gga-mir-6649-p5	TTTATC<br>ACATCA<br>ATGCCA<br>AGC	21	Z	gp1a	.(((..<br>...)))<br>..))((<br>(.....<br>)))...<br>.(((..<br>((((((<br>((((((<br>(((..(<br>(((((.<br>((.(((<br>((.(((<br>(((((.<br>......<br>..))))<br>))))))<br>))).))<br>.)))))<br>)...))<br>))))))<br>)).)))<br>))..))<br>)	1.65	0.76	1.92	2.38	8.49	0
gga-miR-6649-5p_L-2	TATCAC	23	Z	gp1a	...((.	1.65	0.76	2.57	0	0	1.96

R+2	ATCAAT<br>GCCAAG<br>CAGGT				.(((..<br>...)))<br>..))((<br>(.....<br>)))...<br>.(((..<br>((((((<br>((((((<br>(((..(<br>(((((.<br>((.(((<br>((.(((<br>(((((.<br>......<br>..))))<br>))))))<br>))).))<br>.)))))<br>)...))<br>))))))<br>)).)))<br>))..))<br>)						
gga-mir-6654-p3	ACGGAG<br>CCGCAG<br>CTGTGT	22	26	gp1a	...(((<br>((((..<br>((((((	1.65	0.76	0	0	0	0

	GAGT				(.((((<br>(((((.<br>((((((<br>..(((.<br>...)))<br>..))..<br>.)))).<br>))))).<br>))))..<br>))))..)<br>))))))<br>)))...						
gga-miR-6655-5p	TCTGCT<br>AGGAGG<br>CTGTGT<br>ATCCT	23	3	gp1a	...(((<br>((.(((<br>....((<br>((....<br>))))..)<br>)).)))<br>))..	1.65	0	0	0	0	0
gga-miR-6657-3p	ACATGG<br>TGGGAG<br>AAGGGT<br>GAC	21	chr6	gp1a	.((((.<br>((((.(<br>(((..(<br>(.((((<br>(.(((.<br>(((..(<br>((((..	1.65	0.76	0.64	0	0	0

					((((..(.........).)))))..)))))..))).)))....))))))..))))))))..)))))...						
gga-mir-6657-p5	TGCCCCCTCTTTCACCAGGGTCT	23	6	gp1a	..(((..((....)).)))......(((((..((((..(.........)..))))).))))))...	1.65	0	0	1.19	0	0
gga-mir-6668-p5_1ss9AC	GGTGATTCCATGATTCTA	20	12	gp1a	...((.((((.....(((	1.65	0	0	0	0	0

	TG				((((....((((((....((((((...((((.....))))...........(((((......)))))..))))))......))))))..))))).)))...)))))))..					
gga-mir-6668-p5_1ss1CT	TTTGGGTTGGAAGGCACCT	19	27	gp1a	...((..((((......))))..))...	1.65	0	0	0	0	0
gga-miR-6669-5p_R+2	GGTGATACGGGAGCAAGG	26	Z	gp1a	.......((((..(((..	1.65	0	0	0	0	0

	CTGCAT<br>AT				.((..(<br>((((.(<br>((((((<br>.....(<br>((((((<br>(.....<br>((((..<br>..))))<br>..))))<br>))))..<br>))))))<br>))))))<br>..))..<br>.)))..<br>..))))						
gga-miR-6688-3p_L+3R-2	GCTTGA<br>GTCACC<br>TGAGGA<br>GGCTT	23	chr4	gp1a	......<br>.((((.<br>..(((.<br>((((..<br>..((((<br>(....(<br>((((.(<br>((((((<br>(((((.<br>.....)<br>))))))	1.65	0	0	1.19	0	0

					)))))).<br>))))))<br>)))))..<br>......<br>.)))).<br>))).))<br>))....<br>..						
gga-mir-6688-p5_1ss10GA	TTTCCTCTGATGGCTCAG	18	4	gp1a	........((((<br>(...((<br>....))<br>.)))))<br>...	1.65	0	0	1.19	0	0
gga-miR-6690-5p_L-2R-2	AGAAGGGGATGGCTTTGGC	19	3	gp1a	...(((<br>((((((<br>(((..(<br>(((.((<br>((((((<br>(.....<br>))))))<br>.)))..<br>.)))).<br>.)))))<br>)))).)<br>))...	1.65	0	0	0	0	0

gga-mir-6691-1-p5	TTGTTGTAGTGCATGATGAACC	22	7	gp1a	...((((((((((((((....(((.......))).))))....))))).))))))))))((((((......)))))).....	1.65	0	0	0	0.53	0
gga-mir-6698-p5	TTCTTCTCAGTATCTTCCTCTGACC	25	2	gp1a	..((....))..((((((((..((((((((...(((..(.((((((.................))))))	1.65	0	0	0	2.12	0

					..))).))))))).))..))))))))...						
gga-mir-6700-p3_1ss19CT	GAGGGAGCTGGGATTGTTT	19	AADN03022365.1	gp1a	...(((((.((((((.....((((..((((........))))...))))..)))))).)))))..	1.65	0.76	0	0	0	0
gga-mir-6700-p5	GCTGAGGGAGCTGGGATT	18	1	gp1a	...((((((((..(..((....))..)......)))))))))...	1.65	0	0	1.19	0	0
gga-mir-6700-p3_1ss7GA	GAGGGAACTGGG	19	1	gp1a	...((((((((.	1.65	0	0	0	0	0

	ATTGTT C				.(..((....)) ..)... ..)))))))).. .						
gga-miR-6707-5p_R-3 _1ss12CT	ACTGGA ACAAGT TGCCTG GAG	21	chr2	gp1a	....(((. (((... .((((((((..(((..(((...(((.((.. ((.((((((... ...)))))).)) ..)).)))...)))..)))))))))))...))).))).	1.65	0	0	0	0	0
gga-mir-6709-p3	GCAGGG	21	11	gp1a	...(((	1.65	0	0	1.19	0	0

	CTGCTT<br>TTGCGA<br>TCC				(.((..<br>.....(<br>((((((<br>(((((.<br>((....<br>(((.((<br>((((.(<br>(..((.<br>....))<br>..))))<br>)))).)<br>))..))<br>.)))))<br>))))))<br>).)).)<br>)))...						
gga-miR-7441-5p_R+2	CTGAGG<br>AGGGTT<br>TGTAAC<br>ATGGGT	24	17	gp1a	...(((<br>((((((<br>(...((<br>((((((<br>......<br>......<br>....))<br>))))))<br>..))))<br>))))))	1.65	0.76	0.64	0	0	0

					....						
gga-miR-7449-3p_L+1R-1	GAGGGTTGGACGAGATGGTCTT	22	chr1	gp1a	((..((((.((((((..(((((..(((..........))).))).)))))))))).))))..))..	1.65	1.53	0.64	1.19	0	1.96
gga-mir-7449-p3_1ss5TC	AACTCCCCTGCAATGAACA	19	1	gp1a	...((((((..(((((.......))))))...)).))))...	1.65	0	0	0	1.59	0.98
gga-mir-7449-p5_1ss5TC	AACTCCCCTGCAATGAACA	19	3	gp1a	......(((((.......)))))..	1.65	0	0	0	1.59	0.98
gga-miR-7469-3p_1ss	TTTGTG	23	4	gp1a	...(((	1.65	0	0	0	0	1.96

23GT	TGCTTG				(...((						
	CATTGC				((((.(						
	AAAGT				(((((.						
					......						
					.(((((						
					((((((						
					((...(						
					((((.(						
					((((((						
					(....)						
					))))..						
					..))))						
					))))..						
					.)))))						
					))))))						
					))..))						
					))))..						
					.)))))						
					)..)))						
					)...						
gga-miR-7472-5p	ATGTGC	22	chr6	gp1a	..(((.	1.65	2.29	5.77	4.76	9.55	15.70
	TGGAGA				((((((						
	TGAAGC				.((.((						
	TGGA				((.((.						
					......						
					......						

					..)).)))))).)))))).))).....						
gga-miR-7476-3p_L+2R-2	TTTCACAACAGTGGGCTGAAT	21	15	gp1a	...((((.(((((.(((((((((..((((((((.(((.((((((.(((.........)))...)))))))).)))))))))))).)))))).))))))))))..))))....	1.65	0	0	0	0	0
gga-mir-7481-p5_1ss9AT	GAGCCAGGTAGA	18	Z	gp1a	(((((((...((	1.65	0	0	0	0	0

	AAGACA				(.(((((..(((.....)))..).)))))).))))))).						
gga-mir-7482-2-p3_1ss15CA	AGGGCAACTTTCTGAAGA	18	Z	gp1a	.((.((((((....((..(((((((.....)))))))..))...))))))..)).	1.65	0	0	0	0	0
hsa-miR-3074-3p_L-1R+2	ATATCAGCTCAGTAGGCACCGGA	23	Z	gp1b	(((((((.((((.((.((((((((.((((..((((.((......))..)))))..	1.65	0	0	0	0	0

					.)))).<br>))))))<br>)).)).<br>))))..<br>))))))<br>)						
hsa-miR-3591-5p_R-1	TTTAGT<br>GTGATA<br>ATGGCG<br>TTTG	22	Z	gp1b	(((.((<br>((....<br>((((((<br>((((((<br>(.((((<br>(.....<br>......<br>.)))))<br>.)))))<br>))))))<br>))....<br>))))))<br>)	1.65	0	1.28	2.38	1.06	1.96
gga-let-7d_R-3	AGAGGT<br>AGTGGG<br>TTGCAT	18	7	gp1a	...(((<br>(((...<br>((....<br>..))..<br>..))))<br>))...	0.82	0	0.64	0	0	0
gga-miR-29a-5p_L-1R	TGATTT	19	2	gp1a	...(((	0.82	0.38	0.64	0	0	0

-2	CTTTTGGTGTTCA				((.((((....)))).(((......)))).)))))...						
gga-miR-30e-5p_R+1_1ss3TC	TGCAAACATCCTTGACTGGA	20	3	gp1a	..((.((((((((.(((......)))))))))).)))..	0.82	0.38	0.64	0.60	0	1.96
gga-miR-30c-5p_R-5_1ss1TG	GGTAAACATCCTACACTCT	19	3	gp1a	...((((((..(((((((.........(((...(((...........))).(..)))....(((.........))).))))))).)	0.82	0	0	0	0	0

					))))...						
gga-miR-103-2-5p_R+1_1ss1AG	GGCTTCTTTACAGTGCTGCCTTGT	24	13	gp1a	..((((((((((.(((.(((((((((((.(((.........))))))))))))))).))))))..)))))))..	0.82	0.38	2.89	2.98	2.65	0
gga-miR-103-3p_R-2_1ss1AG	GGCAGCATTGTACAGGGCTAT	21	27	gp1a	(((((((((((.....(((....)))..))).)))).)))))..	0.82	0.76	0.32	2.38	1.06	2.94
gga-miR-128-3p_L-1R+1_1ss21TC	CACAGTGAACCGGTCTCT	21	2	gp1a	.((((((......(((((	0.82	0	0	0	0	0

	TCT				((((.(<br>((.(((<br>..((..<br>(((.((<br>(.((.(<br>((((.(<br>((....<br>......<br>.....)<br>))))))<br>))))))<br>)))...<br>.))...<br>)))...<br>)))..)<br>))))).<br>)))...<br>..))))<br>))...							
gga-miR-130b-5p_R-3_1ss10CT	CCTCTTTCCTTGTTGCACT	19	18	gp1a	...(((<br>((((((<br>......<br>..((((<br>((....<br>))).))<br>).....	0.82	0	0	0	0	0

					..))))<br>))..))<br>)...						
gga-miR-130b-3p_R-3	CAGTGC<br>AATAAT<br>GAAAGG<br>G	19	1	gp1a	..((((<br>..((((<br>((..((<br>((((..<br>((((((<br>(.....<br>..((((<br>(((.((<br>((...(<br>((...)<br>))))))<br>))))))<br>))))))<br>))..))<br>))))..<br>......<br>))))))<br>..))))<br>..	0.82	1.14	0.32	4.76	2.12	2.94
gga-miR-142-5p_L-2_<br>1ss16GA	CATAAA<br>GTAGAA<br>AACACT<br>AC	20	19	gp1a	...(((<br>((.((.<br>((((((<br>((((((	0.82	0	0	0	0	0

gga-miR-147_L-3R+4	TGCGGA<br>AATGCT<br>TCTGCT<br>ACC	21	27	gp1a	((((((<br>.(((((<br>(.....<br>(((...<br>.)))..<br>.)))))<br>).))))<br>))))))<br>))))))<br>)).)).<br>))))).<br>..<br>...(((<br>((..((<br>((((((<br>(.((((<br>......<br>.(((((<br>(.((((<br>(.....<br>......<br>.)))))<br>..))))<br>))...)<br>))))))<br>)).)))	0.82	0	0.32	0	0	0

					)....)))))...						
gga-miR-214_R-1_1ss18GC	ACAGCAGGCACAGACAGCCA	20	6	gp1a	......(((.((..((((.......)))).)).)))...	0.82	0.38	0	0	0	0.98
gga-miR-217-5p_R-4	TACTGCATCAGGAACTGATT	20	15	gp1a	.....((((((...((..(....)..))..))))))...	0.82	0	0	0.60	0.53	0.98
gga-miR-221-3p_R-3_1ss20TA	AGCTACATTGTCTGCTGGGA	20	2	gp1a	...((((((((((((..((((.(((.....((..(((..(((((((.(((....	0.82	3.43	1.92	1.19	0.53	0.98

					.)))..<br>))))).<br>))..))<br>)..)).<br>......<br>))))))<br>......<br>)..)))<br>).)).)<br>))))).<br>..						
gga-miR-223_1ss19CT	TGTCAG<br>TTTGTC<br>AAATAC<br>TCC	21	12	gp1a	...(((<br>(((((.<br>((..((<br>(((.(.<br>.(((((<br>......<br>....))<br>)))...<br>.)))))<br>)..)))<br>))))).<br>))...	0.82	1.53	0.96	0.60	0	0
gga-miR-223_1ss15AG	TGTCAG<br>TTTGTC<br>AAGTAC	21	11	gp1a	......<br>(((((.<br>..(((.	0.82	0.76	0.64	0	0	0

	CCC				(((.(((((.....))))).((..((((...))))..))..)))))))))))...						
gga-mir-365b-p5_1ss17CA	ATCTGCCCCCAAAAGTACC	19	5	gp1a	...(((.(((((((.....((.....))..))))))))))...	0.82	0	0	0	0	0
gga-mir-365b-p3_1ss17CA	ATCTGCCCCCAAAAGTACC	19	14	gp1a	...(((......((((((((.((((((....(((.((((.......))	0.82	0	0	0	0	0

					))))))))))).)))))))))))..						
gga-miR-451_R-5_1ss18GA	AAACCGTTACCATTACTA	18	3	gp1a	...((((..((..((((.......((.....)).........((....))..............))))..))..))))...	0.82	0	0	0	0	0
gga-miR-1454_L-3R-1	CAATGATGAGACTTTGGCTC	20	2	gp1a	.......(((((((..((((((((((((((.........)))	0.82	0	0.32	0	0	0

					)))))). ..((.. ..)).))).))) ..)))))))...						
gga-miR-1555-5p	AGCAAA CCCTTC ACCCTG CC	20	6	gp1a	...((. .((((. ((((.. ....)))).))))..))...	0.82	0.76	0	1.79	2.65	2.94
gga-miR-1591-5p	TGATTC ATTGCC TGGCTC TGC	21	7	gp1a	...(((((..((..(..(((.(((..(.(((((((. ....))))))))..))).))) ..)..))...))	0.82	0	0	0	0	0

					)))...						
gga-mir-1591-p5_1ss12TA	CATTGCCTGGCACTGCAAA	19	1	gp1a	...((((..(((((..(((...((.((((...))))))....)))..)))))))))...	0.82	0	0	0	0	0
gga-mir-1630-p5_1ss6AG	TTTCTGAAGGGGGAGGAAA	19	Z	gp1a	...((((((((.((..(((((((..((..(((((.((....)).)))))..))..))))).))..)).)))))))))...	0.82	0	0	0.60	1.59	0.98
gga-mir-1635-p3	CTGGCA	21	JH3754	gp1a	...((.	0.82	0	0.32	0.60	1.06	0

	CACAGCCTGGGAGCA		56.1		.((.(((((....(((...(((((......))))))..))).......))))))...))...						
gga-miR-1662_R-4	TTGACATCATCATACTTG	18	2	gp1a	...((((((((..((((((.((...((((.((....)).))))..)).))).))))))))))...	0.82	0	0.96	0	0	0
gga-miR-1698_R+1	CGAGGCTGCGGCAATCCCTGCGC	23	19	gp1a	...(((((((.(((((((.(((((	0.82	0	0.32	0.60	1.06	0

					((((((...(((...(((((.....))))).)))...))).)))))))))))))).)))).)))..					
gga-miR-1699_L-1R-1	CAGAGGGACATGGCAGGGCA	20	19	gp1a	(((..((.((((((((...((.(((..(((....((((.....(((((((..((((.(((((.(((..((..((....)).))	0.82	1.53	0.64	0.60	4.24	1.96

					..))).						
					...)))						
					))..))						
					))...)						
					))))))						
					)..)))						
					).))).						
					.))).)						
					)...))						
					)))...						
					))))).						
					)))...						
gga-miR-1768	GAGCAG	22	2	gp1a	...(((	0.82	0	0	1.19	0	1.96
	AGGATT				..((..						
	GTCGTG				.....(						
	CTGA				((.((.						
					..((((						
					((((((						
					((((((						
					((((((						
					((((((						
					(((((.						
					......						
					...)).						
					))))))						
					))))))						

					))))))<br>))))))<br>))))))<br>)..)))<br>))))..)))...						
gga-miR-1768_L-1	AGCAGA<br>GGATTG<br>TCGTGC<br>TGA	21	2	gp1a	...(((<br>((.(((<br>((((((<br>(.((((<br>((((((<br>((((..<br>...((.<br>...)).<br>.)))))<br>))))))<br>))).))<br>))))))<br>)).)))<br>))...	0.82	0	0	0	0	1.96
gga-miR-1782_L+2R-1	AAACAT<br>TCATTG<br>GAGCAG<br>GGAC	22	3	gp1a	......<br>.....(<br>(((..(<br>((((((<br>....))<br>...)))	0.82	5.72	1.28	3.57	2.12	2.94

					))..))<br>))						
gga-mir-3064-p3_2	CTGTTG<br>TGGTGT<br>GCAAAA<br>C	19	1	gp1a	...(((<br>(((...<br>..))))<br>))....<br>....	0.82	0	0	0	0	0
gga-mir-3064-p5_7	TTGTCT<br>AACGTT<br>TATCTT<br>C	19	4	gp1a	..((((<br>((((((<br>..((((<br>((....<br>.(((((<br>..(...<br>..)...<br>))))).<br>.....)<br>))))).<br>..))))<br>))))))<br>..	0.82	0	0	0	1.06	0
gga-mir-3064-p3_6	TGTGAA<br>ATTTGT<br>CATCAA<br>TT	20	15	gp1a	...(((<br>.(((((<br>((.(((<br>.((...<br>....))<br>.)))))	0.82	0	0	0	0	0

					)))))))).....						
gga-miR-3064-3p_L-2	GCCACACTGCAACACTTTACA	21	14	gp1a	...((((((....(((.((......)).)))......))))))...	0.82	0	0	0	0	0
gga-mir-3064-p5_4	GATTTGGCTGTTGTGGTGT	19	1	gp1a	...(((..((.((.(((((((...............)))))..)).)).))..)))...	0.82	0	0	0	1.06	0
gga-mir-3064-p5_3	GATGTGGAAGATGTGAAATT	20	3	gp1a	.((.(((.(((((.(..(((......(((..((((.	0.82	0	0	0	1.06	0

					.....))))..)))...)))..).))))))))))).......						
gga-mir-3064-p5_2	CAGATGTGGAAGATGTGAA	19	6	gp1a	...((((((((.((............))))))))))....	0.82	0	0	0	1.06	0
gga-mir-3064-p5_5	GCTGTTGTGGTGTGCAAAAC	20	W	gp1a	.....((((((.......))))))....	0.82	0	0	0	1.06	0
gga-mir-3064-p5_1	CAGATGTGGAAGATGTGA	18	3	gp1a	...((((((.....(((((((((((((((((((((	0.82	0	0	0	1.06	0

gga-mir-3064-p3_7	TTTTGC<br>CACACT<br>GCAACA<br>CT	20	18	gp1a	.((((.<br>...)))<br>)...))<br>).))))<br>))....<br>......<br>))))).<br>......<br>...)))<br>))))).<br>.)))))<br>)...<br>......<br>......<br>..((((<br>(((.((<br>(((..(<br>((((..<br>((((((<br>((((..<br>..((..<br>...)).<br>))))).<br>))))).<br>.)))))<br>.....)	0.82	0	0	0	0	0

					))))...)))))))........................						
gga-mir-3064-p3_1	AAAACTTTGTACCTTGCT	18	4	gp1a	...((.........))).......	0.82	0	0	0	0	0
gga-mir-3064-p3_3	GATGTGGAAGATGTGAAATTT	21	2	gp1a	...((..(((((((..(((((((..........))).))..))))))..))...	0.82	0	0	0	0	0
gga-mir-3064-p5_6	TGTTGTGGTGTGCAAAACT	19	10	gp1a	...((((.((..((.((....)).))..)).))))..	0.82	0	0	0	1.06	0

gga-mir-3064-p3_4	TACAGATGTGGAAGATGTG	19	12	gp1a	...(((((.....(((....))).....)))))...	0.82	0	0	0	0	0
gga-mir-3064-p3_5	TGGAAGATGTGAAATTTGTC	20	8	gp1a	...(((((..(((((.((.........((((((....((..((.((((((.........))))))).))..))...))))))).........)))))).))))...	0.82	0	0	0	0	0
gga-miR-3524a	CAGAATCGCAGA	22	20	gp1a	...(((((..((	0.82	0.38	0.96	0.60	0	0.98

	ATGGCT GAGG				(...((((.(((.....))).))))))).. .))))) ...						
gga-mir-3524a-p3_1s s14AT	CTTTAA GGTCCC TTCCAA CC	20	27	gp1a	..((((((..((((((.. ((((.. ....)))).... .(((.. .....))).... ....))))))....))))))	0.82	0	0	0	3.18	1.96
gga-mir-3524b-p5_1s s10AG	CATTCT ATGGTT	19	7	gp1a	....((	0.82	0	0	0.60	0	0

	CTGTGA<br>T				((.(((<br>(....)<br>))).))<br>))..						
gga-mir-3524b-p3_1s<br>s10AG	CATTCT<br>ATGGTT<br>CTGTGA<br>T	19	1	gp1a	...(((<br>((....<br>((....<br>.))...<br>))))).<br>....	0.82	0	0	0.60	0	0
gga-miR-3525_L+1R-3	TCAGCC<br>ATTCTG<br>CGATTC<br>TG	20	20	gp1a	...(((<br>((....<br>.)))))<br>......	0.82	0	0.32	0.60	0	0
gga-mir-3526-p3_1	ATAAAG<br>CAACTC<br>CAGTGT<br>CA	20	1	gp1a	...(((<br>(.....<br>......<br>......<br>......<br>))))..<br>.	0.82	0	0	0	0	0
gga-mir-3526-p3_5	TTTCCT<br>CTTTGT<br>AGTTAT<br>A	19	4	gp1a	...(((<br>(.((..<br>..((..<br>.(((((<br>......	0.82	0	0	0	0	0

gga-mir-3526-p5_7	TTATTT<br>TTCCTC<br>TTTGTA<br>G	19	Z	gp1a	))))).<br>..))..<br>..)).)<br>)))...<br>...(((<br>((....<br>((((((<br>.(((((<br>(.....<br>(((((.<br>....((<br>((((((<br>(.((((<br>.((((.<br>..((((<br>(.(((.<br>....))<br>)..)))<br>))...)<br>))).))<br>))...)<br>))))..<br>.)))).<br>....))<br>)))...<br>))))))	0.82	0	0	0.60	0	0.98

					.))))))......))))).						
gga-mir-3526-p3_2	GTGTCAGAATTGAAGATGAA	20	2	gp1a	...((((..((((..((.((.(((......))).)).))...))))........))))....	0.82	0	0	0	0	0
gga-mir-3526-p5_5	TAAAGCAACTCCAGTGTCA	19	1	gp1a	..((((((((((......))...))))))))..	0.82	0	0	0.60	0	0.98
gga-mir-3526-p5_2	ATAAAGCAACTCCAGTGTC	19	8	gp1a	....((((....((((..((((.(((....	0.82	0	0	0.60	0	0.98

					.))).))))..))))...))))...						
gga-mir-3526-p5_1	AGAATTGAAGATGAAGTTG	19	1	gp1a	...(((...((((((......))))))...)))...	0.82	0	0	0.60	0	0.98
gga-mir-3526-p3_3	TGTTTGTCTTCTCGCAGCT	19	14	gp1a	...(((((((((.((....((.....))....)))))))))))))...	0.82	0	0	0	0	0
gga-mir-3526-p3_7	TTTTGGAGTAGCTGTTTGTC	20	3	gp1a	...(((((...((.....))....)))))..........	0.82	0	0	0	0	0

gga-mir-3526-p5_3	GAGTAG CTGTTT GTCTTC T	19	2	gp1a	.......(((((((.... .)))))))...	0.82	0	0	0.60	0	0.98
gga-miR-3526_L+1R-1	ATTGAA GATGAA GTTGGT G	19	5	gp1a	...((((..(((((((((((.(((((....))))).)))))..)))))..)))..))))...	0.82	0	0	0	0	0.98
gga-mir-3526-p5_9	TTTTCC TCTTTG TAGTTA T	19	1	gp1a	...((((((... ...(((...(((((..((((((.. ....))) ....))))..))))).	0.82	0	0	0.60	0	0.98

					...)))......)).))))...						
gga-mir-3526-p5_4	GTCTTCTCGCAGCTTTCCC	19	4	gp1a	...((((..(((((((.. .(((..((((....(((....)))))))..)))...).)).))))...))))...	0.82	0	0	0.60	0	0.98
gga-mir-3526-p5_8	TTTGGAGTAGCTGTTTGTCT	20	5	gp1a	...((((((.((.(((((...(((...((((((..............	0.82	0	0	0.60	0	0.98

gga-mir-3526-p5_10	TTTTGG<br>AGTAGC<br>TGTTTG	18	3	gp1a	......<br>.)))))<br>)...))<br>).....<br>..))))<br>).)).)<br>))))).<br>..<br>...(((<br>..((((<br>((((((<br>(((..(<br>(((((.<br>((((..<br>....((<br>((...(<br>(((((.<br>......<br>...)))<br>)))...<br>))))..<br>....))<br>)).)))<br>....))<br>)...))<br>))))))	0.82	0	0	0.60	0	0.98

					))))).<br>.)))..<br>.						
gga-mir-3526-p3_4	TGTTTG<br>TCTTCT<br>CGCAGC<br>TTTCCC	24	3	gp1a	...(((<br>..((((<br>((((((<br>(((..(<br>(((((.<br>((((..<br>....((<br>((...(<br>(((((.<br>......<br>...)))<br>)))...<br>))))..<br>....))<br>)).)))<br>....))<br>)...))<br>))))))<br>))))).<br>.)))..<br>.	0.82	0	0	0	0	0
gga-mir-3526-p3_6	TTTTGG<br>AGTAGC	18	2	gp1a	...(((<br>(((.((	0.82	0	0	0.60	0	0.98

	TGTTTG				......<br>)).)))<br>)))...<br>.....						
gga-mir-3526-p5_6	TCTTCT<br>CGCAGC<br>TTTCCC<br>A	19	17	gp1a	...(((<br>((((((<br>......<br>......<br>....))<br>))).))<br>))...	0.82	0	0	0.60	0	0.98
gga-mir-3533-p5_2	CATGAA<br>GTGTGA<br>TGTGGA	18	12	gp1a	...(((<br>(.((((<br>((....<br>......<br>....))<br>))))))<br>))...	0.82	0	0	0.60	0	0
gga-mir-3533-p5_1	ATCCGT<br>AAGGAT<br>CTGTAT<br>GCC	21	22	gp1a	..((..<br>......<br>......<br>......<br>((.((.<br>...)))<br>).(((.<br>...)))	0.82	0	0	0.60	0	0

gga-mir-3533-p5_3	GAAGTG<br>TGATGT<br>GGATAT<br>CCGTA	23	14	gp1a	((((((<br>(((...<br>....((<br>(.((.(<br>(((.((<br>.((...<br>...)).<br>))))))<br>)).)))<br>.....)<br>))))))<br>)).))<br>..((((<br>((((((<br>.(((((<br>.((...<br>)).)))<br>))....<br>.....(<br>(((((.<br>....))<br>)))).)<br>))).))<br>))))..<br>..((((<br>(((.((	0.82	0	0	0.60	0	0

gga-mir-3533-p3_1	ATCCGT<br>AAGGAT<br>CTGTAT<br>GCC	21	14	gp1a	....((<br>((..((<br>.(..(.<br>(((((.<br>.....)<br>))))).<br>.)))..<br>))))..<br>.)))))<br>))))..<br>.<br>..((((<br>((((((<br>.(((((<br>.((...<br>)).)))<br>))....<br>.....(<br>(((((.<br>....))<br>)))).)<br>))).))<br>))))..<br>..((((<br>(((.((<br>....((	0.82	0	0	0.60	0	0

					((..((.(..(.(((((......))))))).))).)))))).......))))))))).....						
gga-mir-3533-p5_4	GTATCCATGAAACTACCT	18	5	gp1a	((((((((........((......))........))).)))))....((((....))))........	0.82	0	0	0.60	0	0
gga-mir-3533-p3_1ss1TC	CATCCGTAAGGATCTGTATGC	21	5	gp1a	((((((((........((......	0.82	0	0	0	0	0

					))........)).)))))....((((....))))).......						
gga-mir-3533-p5_6	TGTGATGTGGATATCCGTA	19	15	gp1a	...((((((((((((.((....)).))))))...))))))))...	0.82	0	0	0.60	0	0
gga-mir-3533-p5_5	TCATGAAGTGTGATGTGGAT	20	LGE64	gp1a	...(((((.(((((.....))))).((((((....)).))).......))))...	0.82	0	0	0.60	0	0
gga-miR-3535_L+1R-7	TGGATA	19	4	gp1a	.(..(.	0.82	0	0	1.79	0	0

	TGATGACTGATTA				((((((((((.....(((......)))....(((((((..(((.....)))....)))))))).......)))))))))))..).						
gga-miR-3535_L+1R-8_1ss18TA	TGGATATGATGACTGATA	18	12	gp1a	((.((((.((...(((........))).)))))).))	0.82	0	0.32	0.60	1.59	0
gga-mir-6548-p5_1ss19CT_1	CTGTCACCACTGCTGAAATGCACC	24	25	gp1a	...........(((((..((.(((..(((.	0.82	0	0	0	0	0

					.....)<br>)).)))<br>))..))<br>)))...						
gga-mir-6548-p5_1ss<br>19CT_2	CTGTCA<br>CCACTG<br>CTGAAA<br>TGCACC<br>A	25	25	gp1a	...(((<br>.....(<br>((....<br>..))).<br>......<br>(((..(<br>((((((<br>(.(((.<br>......<br>...)))<br>...)))<br>))))).<br>)))...<br>....))<br>)...	0.82	0	0	0	0	0
gga-miR-6592-3p_L-1<br>_1ss14TA	GCGTCT<br>CCCGCC<br>AGACGG<br>CC	20	Z	gp1a	...(((<br>(.((((<br>...(((<br>((..((<br>(((.((<br>......<br>((((..	0.82	0	0	0	0	0

					...)))<br>).))))<br>)))..)<br>))))..<br>.)))).<br>..))))<br>...						
gga-miR-6608-3p	AGCACT<br>GCAAGA<br>GGGAGA<br>GAGAT	23	1	gp1a	...(((<br>..((((<br>(((..(<br>(((.((<br>.(((((<br>((((..<br>((((((<br>.((((.<br>......<br>..))))<br>))))))<br>...)))<br>))))))<br>)).)))<br>)..)))<br>))))..<br>)))...	0.82	0.38	0	0	0.53	0
gga-miR-6608-3p_R-1	AGCACT<br>GCAAGA	22	1	gp1a	...(((<br>..((((	0.82	0	0	0	0	0

	GGGAGA<br>GAGA				(((..(<br>(((...<br>.(((((<br>((((..<br>((((((<br>.((((.<br>......<br>..))))<br>))))))<br>...)))<br>))))))<br>...)))<br>)..)))<br>))))..<br>)))...						
gga-mir-6626-p3_1ss<br>22GT	TGCTTT<br>CCTTTC<br>TCCCTC<br>CAGT	22	21	gp1a	...(((<br>(((((.<br>(..((.<br>(((...<br>....))<br>)..)).<br>.).)))<br>))))).<br>((((((<br>((..((<br>(.....	0.82	0.76	0.64	0	1.06	0

					...)))<br>..))).<br>))))).						
gga-mir-6635-3-p5	ATGTCC<br>GGCTGT<br>GAAGGA<br>TC	20	15	gp1a	.....(<br>..(.((<br>(((((.<br>.(((.(<br>((((..<br>((((..<br>...)))<br>))))))<br>.)))..<br>..))))<br>))).).<br>.)...	0.82	0	0	0	0.53	0
gga-mir-6668-p3_1ss 15CG	CTTGGG<br>TTGGAA<br>GGGACC	18	21	gp1a	...(((<br>((..((<br>(((..(<br>(((..(<br>......<br>....).<br>.)))))<br>).))).<br>.)))))<br>...	0.82	0	0	0.60	0.53	0
gga-mir-6668-p5_1ss	GCTTGG	20	3	gp1a	....((	0.82	0	0	0	0	0

16CG_2	GTTGGAAGGGACCT				(((......))))).((((((..........((.((((....))))).(((..((((..................)))..))).))))))...						
gga-mir-6668-p5_1ss11GA	GAATAGCTTGAGTTGGAAG	19	23	gp1a	...((((...(((.((((((....(((((...(((..(((((.(((((((..........	0.82	0	0	0	0	0

					....))<br>)).)))<br>))))).<br>.)))..<br>))))))<br>))))).<br>))).))<br>))...						
gga-mir-6672-p3_1ss<br>7GC	GACAAG<br>CGGCAG<br>TGGGCA<br>C	19	13	gp1a	.(((((<br>(((...<br>......<br>...)))<br>))))).<br>......<br>.	0.82	0	0	0	0	0
gga-mir-6681-p5_1	CAGAGA<br>GACGGT<br>CTGGTG<br>CTTCT	23	20	gp1a	......<br>..((((<br>(.(((.<br>..((((<br>(....(<br>((((((<br>(..(((<br>((((((<br>...((.<br>...)).<br>..))))	0.82	0	0	0	0	0

					))))).)))).)))))...)).)))).....))).)))))....					
gga-mir-6681-p5_2	GCATGTGCTGGAAGGATAAT	20	1	gp1a	...(((((..((..(.((((((((..................))))))))).)..)).)))))...	0.82	0	0	0	0	0
gga-mir-6705-p5	GAAGATCATTCTGGGAAGC	19	1	gp1a	...(((((..(((((((.........))))).)).	0.82	0	0	0	1.06	0

					.))))))...						
gga-miR-7444-3p_R-1	AGTGTTAGGTGCAGCCTTGCT	21	1	gp1a	..........((((...(((((.(((.((..((((((.......)))))..)).))))))))))))...	0.82	0	0.32	0	0	0
gga-miR-7449-5p_R-4_1ss3GA	AGATCATCTAGTCCAACT	18	4	gp1a	...((....(((((((....((((((....)))))).)))))))...))...	0.82	0	0	0	0.53	0
gga-miR-21-5p_L+1_2ss6TG23AT	ATAGCGTATCAGACTGAT	23	chr19	gp1a	.((((((...((((((((	0.62	0.35	0.24	0	0	0

	GTTGT				((((((<br>((.(((<br>((.(((<br>((.(..<br>(.....<br>...)..<br>))))))<br>.)))))<br>))))))<br>))))))<br>))))..<br>.)))))<br>).....<br>.						
gga-miR-148a-3p_2ss<br>19TG21GT	TCAGTG<br>CACTAC<br>AGAACT<br>GTTT	22	chr2	gp1a	((..((<br>((((((<br>((((.(<br>(((..(<br>((((((<br>......<br>.))).)<br>))))))<br>)...))<br>))))))<br>))))..<br>))	0.60	1.25	0.86	0.79	0.92	0.89

gga-let-7g-5p_R+1_2ss14TG18CA	TGAGGTAGTAGTTGGTAAAGTT	22	chr12	gp1a	.(((.(((((((((.(..((((((((((....(((((.((.((((....))))))..))))))))))))..).))))))))))))	0.38	0.23	0.26	0.09	0.16	0
gga-miR-458a-3p_2ss14TC17TG	ATAGCTCTTTGAACGGGACTGC	22	chr13	gp1a	.(((.((..((..((..(((((.(((((((..((((((..(((((((((..((((.....)))).)))))))	0.33	0.11	0	0	0	0

					)..))))))..)))))))).))))))).......))..)).)))))						
gga-miR-2188-5p_R-1_2ss8AG18GT	AAGGTCCGACCTCACATTTCC	21	chr22	gp1a	((((((((((((((..(..((((((((((.(((((((((.(((.(((((((((((.(((((.(((.((.((((((((...............)))))))).)).)))..)))	0.33	0.46	0.26	0.48	0.21	0

					)).)))<br>))))))<br>..))))<br>))))))<br>.)).))<br>)....)<br>))))))<br>..)..)<br>))))))<br>))))))<br>)						
gga-miR-30a-5p_R+2_<br>2ss15AC22GA	TGTAAA<br>CATCCT<br>CGCCTG<br>GAAACT	24	chr3	gp1a	((..((<br>((((((<br>((((..<br>((((((<br>(((((.<br>......<br>......<br>...)))<br>))))))<br>))))))<br>))))))<br>))..))	0.26	0.21	0.36	0.40	0.35	0.33
gga-miR-10a-5p_R+1_<br>1ss18TG	TACCCT<br>GTAGAT<br>CCGAAG	23	chr27	gp1a	((((((<br>((.(((<br>(.((((	0.14	0.06	0.40	0.10	0.04	0.25

	TTGTT				(.((((((((((...................))))))))).)))))))).))))))))						
gga-miR-30b-5p_2ss8AC22TC	TGTAAACCTCCTACACTCAGCC	22	chr2	gp1a	((((...((((((...((((((((((.((.(((((((...((.....))..)))))))..))))))))))))...))))))).))))).	0.12	0.08	0.11	0.17	0.08	0
gga-miR-146a-5p_R+2	NGAGAA	24	chr13	gp1a	..((((	0.11	0.05	0.09	0	0	0

_1ss1TN	CTGAATTCCATGGGTTGC				((..((((..((((((((..((((((((((.....(((........))))))))))))))..)))))))))).)))).)))))).........						
gga-miR-301b-3p_R-2_1ss12TG	CAGTGCAATAGGATTGTCAAAGC	23	chr19	gp1a	....((((..(..(((((.((((.((((((((((.((((........)).)).))))))	0.09	0.04	0.22	0.35	0.23	0.11

					))))....)))).)))))..)..))))))..						
gga-miR-15c-5p_R+1_1ss6GN	TAGCANCACATCATGGTTTGTAT	23	chr4	gp1a	.((((..(((..(((((.((..((((((((((.............))))))))))..)).)))))..)))..))))).	0.07	0	0.06	0	0	0
gga-let-7b_R-1_2ss9TA18TA	TGAGGTAGAAGGTTGTGAGGT	21	chr1	gp1a	((((..((((((((((((((((((((...((((((.....))))))).	0.06	0.45	0.08	0	0	0

					.(((....))))))))))))))))))))))).)))))						
gga-miR-200b-3p_L-1R-3_1ss6CA	AATAATGCCTGGTAATGAT	19	chr21	gp1a	(((((...((((((((..(((((.((((((.............)))))).)))))..))).))))...))))))...........	0.05	0.04	0	0.04	0	0
gga-miR-1a-1-5p_2ss15AG17GA	ACATACTTCTTTATGTACCCATA	23	2	gp1a	...((..(((((.(((((.(((((	0	0	0.64	0	0	0

gga-miR-1b-5p_R-1_1ss12CT	ACATACTTCTTTATATGCCCAT	22	23	gp1a	((((((((..(..(((((..................))))).)..)))))))))))))..))))))))))..))...(((((.(((((((((((((((.((((..(((((....(((....)))..))))).)))).))))))))))))...	0	0	0	0	0.53	0

					..))).))))))..						
gga-miR-1a-1-5p_R-1	ACATACTTCTTTATATGCCCAT	22	20	gp1a	...((..(((((((.(((((((((((((((((..(((((.((.........))))))).))))))))))))))).))))))))..))...	0	0	0	0	0.53	0
gga-let-7g-3p_2ss6CN22CT	CTGTANAGGCCACTGCCTTGCT	22	chr12	gp1a	.(((.(((((((.(..(((((((((.....((((.((.((((....)	0	0	0	0	0.08	0

					))))).)))))))))))))..).)))))))))))						
gga-let-7b_L+1R-5	ATGAGGTAGTAGGTTGTG	18	3	gp1a	...((((((((((.........))))))...))))...	0	0.76	0	0	0	0
gga-let-7k-5p_R+1_1ss6TN	TGAGGNAGTAGATTGAATAGTTC	23	chr26	gp1a	((..(((..(((((((((.((((((((((((.......))))......)).))))).))))))))..)))	0	0.04	0	0	0	0

					.))........						
gga-let-7a-5p_R+3_1ss1TN	NGAGGTAGTAGGTTGTATAGTTTTT	25	chr1	gp1a	(((..((..((((((((((((((((.(((((.....))))).........)))))))))))))))..)).. .)))	0	0	0.09	0	0	0.07
oha-miR-7-1-3p_1ss10GA	CAACAAATCACAGTCTACCATA	22	JH376210.1	gp1a	...(((((...(((((.((.(..(((((.(((((.(((((..((((((((((.....))))).	0	0	1.28	58.36	78.50	70.64

					.))))))))))))))))))).)))))..).)))))))).....))))).......						
gga-miR-15a_L+2R+1	CATAGCAGCACATAATGGTTTGTG	24	chr1	gp1a	(((((.......'...((((((((..((((..(((............)))..))))..))))))))................)))))	0	0	0	0	1.06	0
gga-miR-16c-5p_R-1_1ss9CT	TAGCAGCATGTAAATACT	21	Z	gp1a	...((.(((((((.....	0	0	0.64	0	0	0

	GGA				((((........(((....)))))))))))))).))....						
gga-miR-16-2-3p_R+1_1ss2CN	CNAATATTATTGTGCTGCTTAAT	23	chr9	gp1a	.....((((..(((...(((((((((..((((((((.((..(((.........)))).)).)))))))))..))))))))))...)))..))))....	0	0	0	0	0	0.98
gga-miR-16c-5p_R-4	TAGCAGCACGTAAATACT	18	8	gp1a	...((..((((...((..	0	1.14	1.28	2.98	2.65	0.98

					((((((((((.........)))).)))))..))...))))..))...						
gga-miR-17-3p_1ss20TG	ACTGCAGTGAAGGCACTTGG	20	1	gp1a	.((((((((((((..((((.(((((.....)))))))).))))))))).))	0	0	0	0.60	0	0.98
gga-miR-17-3p_R+2_1ss2CG	AGTGCAGTGAAGGCACTTGTAT	22	chr1	gp1a	((((..(((((((..((((((((.((.(((((...((.................))...))	0	0	0	0	0	0.15

					))).)).)))))))...))))))..)))))						
gga-miR-17-5p_R-5_1ss19GT	CAAAGTGCTTACAGTGCAT	19	1	gp1a	...(((((((..(((((...............)))))..))).))))........	0	0.38	0	0	1.06	0
gga-miR-17-3p_L-1	CTGCAGTGAAGGCACTTGT	19	10	gp1a	..(((..((((........))))..)))........	0	1.53	0.32	0.60	0	0
gga-miR-18b-5p_L+1R+2	TTAAGGTGCATCTAGTGCAGTTAG	25	chr4	gp1a	.......(((((((.((((.((((	0	0	0	0.10	0	0

	A										
gga-miR-18a-5p_L+2	AATAAG<br>GTGCAT<br>CTAGTG<br>CAGATA	24	chr1	gp1a	.(((.(<br>((((.(<br>(..(((<br>...)))<br>.....)<br>))))))<br>.))).)<br>))).))<br>))...)<br>))))))<br>..((((<br>(((((.<br>((((((<br>.(((..<br>(((.((<br>((.(((<br>....((<br>......<br>))..))<br>))))).<br>)))..)<br>)).)))<br>..))).<br>))))))<br>)))...<br>...	0	0	0	0	0.53	0

gga-miR-18b-5p_R-2_1ss2AG	TGAGGTGCATCTAGTGCAGT	20	9	gp1a	...((((((((((..((((...((((((...(((((..(((((...........)))))..))))).. ..)))))...)))).......)).))))).))))...	0	0.76	0	0	0	1.96
gga-miR-20b-5p_R+1_1ss13AC	CAAAGTGCTCATCGTGCAGGTAGA	24	chr4	gp1a	((((((((((..(((((((((((.(((((.(((.((........)).))))))))	0	0	0.09	0	0.14	0.13

					.)))))<br>))))))<br>...)))<br>))))))<br>).....<br>.						
gga-miR-21-5p_R-4_1<br>ss18GC	TAGCTT<br>ATCAGA<br>CTGATC	18	14	gp1a	...(((<br>((....<br>)).)))<br>...	0	0.38	0.32	0	0	0
gga-miR-22-3p_R-3_1<br>ss7CT	AAGCTG<br>TCAGTT<br>GAAGAA<br>C	19	Z	gp1a	....((<br>((((..<br>.(((((<br>......<br>(((...<br>((((((<br>(...))<br>))))).<br>)))...<br>..))))<br>)...((<br>(..(((<br>(.....<br>.)))).<br>.)))..<br>))))))	0	0.38	0	0	0	0

					...						
gga-miR-22-3p_R-3_1ss1AT	TAGCTGCCAGTTGAAGAAC	19	2	gp1a	...((((((......))))))..........	0	1.14	0	0	0	0
gga-miR-22-5p_R+1_1ss6TN	AGTTCNTCAGTGGCAAGCTTTAA	23	chr19	gp1a	.....((((((....(((((((((((((((((.((((((.((.((.....))..)))))))))))).))))))))))))))...))))))).....	0	0.06	0	0	0	0
gga-miR-26a-5p_R-1_1ss5AG	TTCAGGTAATCC	20	1	gp1a	...((((((((.	0	0	0.64	0	0	0

	AGGATAGG				..(((..((.((.........((......))......)).))...))).)))))))...						
gga-miR-26a-5p_R+1_2ss15GA17TA	TTCAAGTAATCCAGAAAAGGCT	22	2	gp1a	...(((((.((((..((((((....))))))))))).)))))..	0	0	0	0	1.06	1.96
gga-miR-29c-3p_1ss6CT	TAGCATCATTTGAAATCGGT	20	4	gp1a	...(((((.(((((((((.......((((..(((.........	0	1.14	0.32	0	0	0

gga-miR-29a-5p_L-1R-2_1ss20AT	TGATTTCTTTTGGTGTTCT	19	7	gp1a	..........)))..))))....(((....))).)))))))))).))))).......(((((..((((((((...(((.((.((((((((...((((((((.((((..((....(.....)....))..)))).)))))))))))))	0	0	0.32	0	0	0

					.....)) ...)) .)))..)))))) ....)) ..)))))...						
gga-miR-29a-5p_L+1R -4	ACTGAT TTCTTT TGGTGT T	19	2	gp1a	....(((((..(.((((. ....)))).)..))))). ..	0	0.38	0.32	0	0.53	0
gga-mir-29c-p3_1ss1 1AG	AGGCTG ACCGGT TTCTCT T	19	5	gp1a	...(((((((.((....(((....))))))))))))	0	0	0	0	0.53	0
gga-miR-29b-3p_R-2_ 1ss3GA	TAACAC CATTTG AAATCA GTG	21	14	gp1a	...((((..((((..((. (((.((((..((	0	0.38	0	0	0	0

					.(((((......))))).((........))...........)...))))).)))..))..))))...))))..						
gga-miR-29a-5p_L+1R-3_1ss20CA	ACTGATTTCTTTTGGTGTTA	20	9	gp1a	...(((..(((((((..((((((.....)))))).....))))))).)..)))...	0	0	0.64	0	0	0
gga-miR-29b-3p_R-4_1ss19GA	TAGCACCATTTGAAATCA	19	4	gp1a	...((((((..((((((((	0	0.38	0	0	0.53	0

	A				.((...<br>.(((((<br>......<br>...)))<br>))..))<br>.)).))<br>)))..)<br>)).)))<br>...						
gga-miR-30c-5p_L-3R<br>-1_1ss23CT	AAACAT<br>CCTACA<br>CTCTCA<br>GT	20	1	gp1a	......<br>..((((<br>((((.(<br>((.((.<br>.....)<br>)..(((<br>(.....<br>....))<br>))....<br>......<br>......<br>......<br>..))))<br>)).)))<br>))...	0	0.38	0	0	0	0.98
gga-miR-30c-1-3p_R+<br>1_1ss6GN	TGGGAN<br>AGGATT	22	chr23	gp1a	.(((((<br>..((((	0	0.76	0	1.19	1.06	0

	GTTTAC<br>GCCA				...(((<br>((((((<br>((((..<br>.(((((<br>(((..(<br>......<br>...)..<br>))))))<br>))))))<br>.)))))<br>))))..<br>.)))))<br>)))).					
gga-miR-30c-1-3p_R+<br>1_1ss11TG	TGGGAG<br>AGGAGT<br>GTTTAC<br>GCCT	22	2	gp1a	...((.<br>.((..(<br>((((((<br>((..((<br>(.....<br>((((((<br>(((...<br>.)))))<br>)).)).<br>.....)<br>))..))<br>))))))<br>)...))	0	0	0.32	0	0	0

					..))...						
gga-miR-30c-5p_R-5_1ss17TC	TGTAAACATCCTACACCCT	19	4	gp1a	...(((....)))..........	0	0	0.32	0	0	0
gga-miR-30c-5p_L-1R-1_1ss2GA	ATAAACATCCTACACTCTCAGC	22	1	gp1a	...(((...((((.((((.((...(((((.........))))))..)).))))))))...)))...................	0	0	0	0	0.53	0
gga-mir-30c-1-p5_1ss1AC	CCACTCTCAGCTGTGAACT	19	23	gp1a	..........((((........))))...	0	0	0	0	0.53	0
gga-miR-30c-5p_R-5_	TGTAAA	19	4	gp1a	...(((	0	0	0.32	0	0	0

1ss11CT	CATCTT ACACTC T				((((.. ...))))))).. .						
gga-mir-30c-1-p5_1ss19CA	CTGGGA GAGGAT TGTTTA A	19	2	gp1a	...(((..(((. ..(((. ((((.. ......)))).))).... .)))..)))...	0	0	0.32	0.60	0	0
gga-mir-30b-p3	TATAAC AAGTGG TAGGGC	18	2	gp1a	...... ((((((((((.((.(((((((... ((.... .))..))))))) ..))))))))))))....	0	0.76	0	1.19	0	0
gga-miR-30c-5p_R-5_1ss7CT	TGTAAA TATCCT	19	1	gp1a	...((((.((((	0	0.38	0	0	0	0

	ACACTCT				(....(((((.((..((....)).)))))))....)))))))))...						
gga-miR-30e-5p_1ss3TG	TGGAAACATCCTTGACTGG	19	Z	gp1a	....((((..(((((........)))))..))))...	0	0	0	0	0.53	0
gga-miR-31-5p	AGGCAAGATGTTGGCATAGCTG	22	Z	gp1a	..((((.(((((((((.(((..(..(((((.(((((((((((((.(((........))).).)))))	0	9.15	1.28	0	2.12	7.85

					))))))<br>)).)))<br>))..).<br>.))).)<br>)...))<br>))))).<br>))))						
gga-miR-31-3p	TGCTAT<br>GCCAAC<br>ATATTG<br>TCATC	23	Z	gp1a	..((((<br>.(((((<br>((((.(<br>((..(.<br>.(((((<br>.(((((<br>((((((<br>((.(((<br>......<br>..))).<br>.)))))<br>))))))<br>)).)))<br>))..).<br>.))).)<br>)...))<br>))))).<br>))))	0	0	0	0	0	1.96
gga-miR-33-3p_L-1R-	ATGTTC	18	6	gp1a	....((	0	0.76	0	0	0	0

1	CTGCAGTGCAGT				((((...)))).))...						
gga-miR-34a-3p_2ss1CN23AT	NAATCAGCAAGTATACTGCCCTT	23	chr21	gp1a	.((((((((((((..(((((..((((((((((.((((((((((...(((....)))......)))))))))))))).))))))))..)))))..)).))))..))))).....	0	0	0	0	0.07	0
gga-miR-100-3p_L+1R-3	CAAGCTTGTATCTATAGGT	19	1	gp1a	....((((......(((..((((((	0	0.38	0	0	0	0

					.(((((....)))))...((((((.....))))))).)))))))..))).,...))))...						
gga-miR-103-3p_1ss4AT	AGCTGCATTGTACAGGGCTATGA	23	18	gp1a	.((((((..((((((((.....)))))))))))))))..	0	0.38	0.32	0	0	0
gga-miR-103-3p_R-2_2ss16GT20AT	AGCAGCATTGTACAGTGCTTT	21	12	gp1a	...((((((((..((((((((..((...((.((..((((.((((.(((((...	0	0.76	0	0	0	0

					.))))))))))))))..)).))....))..).))))))))..)))))))))...						
gga-miR-106-5p_R-2_1ss7GT	AAAAGTTCTTACAGTGCAGG	20	11	gp1a	...(((((((........((....))..............(((......))).......))))))))..........	0	0	0	0	0.53	0
gga-miR-106-3p_R-2	ACTGCAGTATAAGCACTT	20	9	gp1a	.........(((((((((	0	0	0.32	1.19	0.53	1.96

	CT				(((((.<br>....((<br>((.(((<br>....))<br>).))))<br>.)))))<br>...(((<br>......<br>.)))..<br>......<br>))))..<br>))))).<br>..						
gga-miR-106-5p_R-2_1ss20GA	AAAAGTGCTTACAGTGCAGA	20	4	gp1a	...(((<br>(((...<br>......<br>...(((<br>((....<br>))))).<br>.....(<br>(....)<br>)...))<br>))))..<br>.	0	0	0	0.60	0	0
gga-miR-106-5p_R+1_1ss21TG	AAAAGTGCTTAC	23	chr4	gp1a	((..(.<br>((..((	0	0.22	0.27	0.13	1.21	0.28

	AGTGCAGGGAA				(..(((((((((...(((((.(((.((...))..))))))))...)))))))))...)))..)))..))						
gga-mir-122b-p5_2ss6TG19GA	TTGGGGGTTTGGGGGAGCA	19	1	gp1a	...((((..(((((..(((((((....))))....(((((((....)))))))............)))..)))))..))))...	0	0	0	0	0	1.96

gga-miR-124a-5p_L+3	CGTGTT CACAGC GGACCT TGAT	22	2	gp1a	...(((.((((. ((((.. (..(((((.(((..((((((((.. ....)))))))). .))).)))))..)..)))))))).)))...	0	0	0	0.60	0	0.98
gga-miR-124b_L-1R+1	TAAGGC ACGCAG TGAATG CCAT	22	23	gp1a	...((((...(((.((.(((((((((.... (((((((..(((.(..((((((((...((((((((.	0	0	0.64	0	0	0

gga-miR-125b-3p_R-1_2ss4AG21CT	ACAGGTCAGGCTCTTGGGACT	21	chr1	gp1a	..........))))))))...)))))))).).)))..)))))))......)))))))))..)))))....))))...((((..............(((((.......)))))((((.........((..(((((..((..(((.(((.((((	0	0.76	0	1.19	0	0

gga-miR-125b-5p_R-3_1ss8GA	TCCCTGAAACCCTAACTTG	19	1	gp1a	((((..............))))))))).))).)))..))..))))).))((....))).))))...)))).(((((....(((.((((((((..((....))...((((....)))).))))))))).)))....))))).........	0	0	0	0	1.06	0

gga-miR-128-3p_R-1_1ss15TG	TCACAGTGAACCGGGCTCTT	20	3	gp1a	...((((.....((((.(((((....((((((.((.......)).))))))))))).))))......))))....	0	0.38	0.32	0.60	0	0
gga-miR-128-2-5p_R-2	GGGGGCCGTTACACTGTAAGA	21	2	gp1a	((.((((..(((((((...(((((..(((((.((.....)).
.))))))))))...)))))))..)))).))	0	0	0	0	0	1.96
gga-miR-130b-5p_L-1	CTCTTT	19	2	gp1a	...(((	0	0	0	0.60	0	0.98

R-2_1ss15GT	CCCTGTTTCACTA				(((..........(((((..((....))..)))))........)))))))....						
gga-miR-130b-5p_R-2_1ss20AG	CCTCTTTCCCTGTTGCACTG	20	22	gp1a	...(((((.(((((((....................................))))))))...))))))...	0	0	0.32	0	0	0
gga-miR-130b-3p_R-4	CAGTGCAATAATGAAAGG	18	11	gp1a	...((.((((.(((.......)))))))).))............	0	0.38	0.64	0.60	0.53	0.98

					.					
gga-miR-130b-5p_L-3	CTTTCCCTGTTGCACTACT	19	17	gp1a	....((.(((((((........))))))).))...	0	0	0	0	1.06	0.98
gga-miR-130b-5p_L-1R-3	CTCTTTCCCTGTTGCACT	18	27	gp1a	...(((..(((...........((((((((((........))))))).))).........)))..)))).....	0	0.76	0	1.79	0	0.98
gga-miR-130b-5p_R-3_1ss1CT	TCTCTTTCCCTGTTGCACT	19	6	gp1a	...(((((.(((.((((........)))))))..))	0	0	0	0.60	0	0

					)))........						
gga-miR-130b-5p_R-3_1ss19TC	CCTCTTTCCCTGTTGCACC	19	4	gp1a	...(((((((.(((((((((.......((..(((.......))).))......)))))))).))))))))...	0	0	0	1.19	0	0
gga-miR-130b-5p_L-4	TTTCCCTGTTGCACTACT	18	24	gp1a	.......(((((.........)))))...	0	0.38	0	1.19	0	0
gga-miR-135a-1-3p	ATATAGGGATTGAAGCCGTGCA	22	12	gp1a	..(((((((.........(((((((((((..((((((((((((	0	0	0.64	1.19	0	0

					..............))))))))))).))))))))))))))))))..						
gga-miR-135b_R+2	TATGGCTTTTTATTCCTTTTGT	22	chr3	gp1a	((..(((((..(((...(((((((((.......)))).))))...)))..).....))))..))	0	0	0.64	0	0	0
gga-miR-137-3p_R+1_1ss1TN	NATTGCTTAAGAATACGCGTAGA	23	chr8	gp1a	.((((((((((((((..(((.(((((((..(..(.	0	0	0	0	0.07	0

					(((((((.....)).))))))..)..))))))))).)))..))))))))))))..)))....						
gga-mir-138-1-p5_1ss9GC	AGCAGCTGCTGTTGTGAA	18	1	gp1a	...((..((((...((.....))..)))).))...	0	0	0	0.60	0	0
gga-miR-142-3p_L-1_1ss11CT	GTAGTGTTTTCTACTTTATGG	21	19	gp1a	...(((((.((.((((((((((((((((((.((((((.....(((....)))...)))))	0	0.38	0.64	0	0.53	0

					).))))))))))))))))).)).)))))..						
gga-miR-144-3p_L-1R-1_1ss3AG	TGCAGTATAGATGATGTACT	20	9	gp1a	...((((((((((.....))))))))))........	0	0	0.64	0	0	1.96
gga-miR-144-3p_L-1R-1_1ss12AC	TACAGTATAGCTGATGTACT	20	2	gp1a	...(((((((.....)))))))....	0	0.76	0	0	0	0
gga-miR-144-5p_L+1R+2_1ss13TG	TGGATATCATCAGATACTGTAAGTT	25	chr19	gp1a	...((.((((.(((....((((((((.(((((((((..(((.....))).....)	0	1.53	1.92	0	2.12	1.96

					))))))))))))))))......))).)))).))						
gga-miR-144-3p_L-1R+4	TACAGTATAGATGATGTACTCCACT	25	chr19	gp1a	...((.((((.(((....((((((((.((((((((..(((.....)))....)))))))))))))))))......))).)))).))	0	0	0	0	1.06	0
gga-miR-144-3p_L-1R-2_1ss13TC	TACAGTATAGACGATGTAC	19	4	gp1a	..............(((.(((((((.....	0	0	0	0	2.12	0

					(((.......)))..((....)).....))))))).))))...						
gga-miR-144-3p_L-1R-1_1ss10AG	TACAGTATGGATGATGTACT	20	5	gp1a	.......(((..((((......))))).......)))...	0	5.34	1.28	3.57	2.12	1.96
gga-mir-144-p5	CATATACTGTAAGTTCACT	19	Z	gp1a	...((.((((.(((.(((((.........))))))))))).))...	0	0	0	0	1.06	0
gga-miR-144-5p_R-2_1ss19TC	GGATATCATCATATACTGCA	20	1	gp1a	.................((((((.(	0	0.76	0.64	1.19	0	1.96

					(....)<br>))))))<br>...						
gga-miR-146c-5p_R-2<br>_1ss2GC	TCAGAA<br>CTGAAT<br>TCCATG<br>GAC	21	AADN03<br>024437<br>.1	gp1a	...(((<br>(..(((<br>.(((((<br>(.....<br>((((..<br>((((((<br>(((...<br>.)))))<br>))...)<br>).))))<br>..))).<br>))...)<br>.)))))<br>))...	0	0	0	0.60	0	0.98
gga-miR-146b-5p_R+2<br>_1ss2GN	TNAGAA<br>CTGAAT<br>TCCATA<br>GGCGTA	24	chr6	gp1a	((.(((<br>((((((<br>..(((.<br>..((((<br>((((((<br>.(((((<br>((((((<br>......<br>......	0	0.76	0.32	0	0.53	0

					)))).)<br>))))))<br>))))))<br>))))..<br>)))..)<br>))))).<br>......<br>....))<br>)))						
gga-miR-148a-3p_2ss<br>18TA19TC	TCAGTG<br>CACTAC<br>AGAACA<br>CTGT	22	12	gp1a	...(((<br>((((..<br>(((((.<br>...)))<br>))....<br>......<br>.((.((<br>..((((<br>......<br>...)))<br>)..)))<br>).....<br>..))))<br>)))...	0	0.76	0.64	0	0	0
gga-miR-155_2ss7CA2<br>2GA	TTAATG<br>ATAATC<br>GTGATA	22	chr1	gp1a	.(((((<br>((((((<br>.((((.	0	0	0.03	0	0	0

	GGGA				((((((((((((....))))).))))))))))))))))))))).						
gga-miR-181a-3p_R-1_2ss16TG19TG	ACCATCGACCGTTGAGTGGAC	21	chr8	gp1a	..(((((((..(((..((((((((((..(((((.((((..((...))....)))))))))))))))))))))..)))..)))))).....	0	0	0	1.19	0	0
gga-miR-190a-3p_L+1R-1_2ss5TA21CT	ACTAAATATCAA	21	25	gp1a	...((((((((.	0	0.38	0.64	0	0	0

	ACATAT<br>TCT				((((..<br>((((((<br>((((((<br>(((..(<br>((((((<br>......<br>))).))<br>))..))<br>))))))<br>))))))<br>.)..))<br>)).)))<br>))))).<br>..						
gga-miR-190a-5p_R+2<br>_1ss2GN	TNATAT<br>GTTTGA<br>TATATT<br>AGGTTT	24	chr10	gp1a	.(((((<br>((.(((<br>((.(((<br>((((((<br>((((((<br>(..(((<br>((....<br>......<br>))))))<br>))))))<br>))))))<br>)))..)	0	0	0.13	0	0	0

					)))).))))))).						
gga-miR-190a-3p_L+1_1ss22TA	NCTATATATCAAACATATTCCA	22	chr10	gp1a	.(((((((.(((((.((((((((((((((((..(((((............))))))))))))))))))))..))))).))))))))).	0	0	0	0	0	0.13
gga-miR-193a-3p_R-3_1ss19CA	AACTGGCCTACAAAGTCCA	19	2	gp1a	...(((((.((((...........((((..(((((((((..........	0	1.14	1.28	0.60	0.53	0

					.....))))..)))))))))...))))))))...						
gga-miR-193a-5p_L+2R-2	GTTGGGTCTTTGCGGGCGAGAT	22		gp1a	(((((((..(((((.(((((..(((.((.(((.(((......)))))).)).)))..))))).)))))..))))))))	0	0	0	0	0.13	0
gga-miR-193b-3p_R-2_2ss16TG20GC	AACTGGCCCACAAAGGCCCCCT	22	chr14	gp1a	.((..((((((..((((..((((.((((.((.((((((....)	0	0.06	0.15	0.10	0	0

					))....<br>.)).))<br>.)))).<br>))))..<br>))))..<br>))))).<br>)..))						
gga-miR-196-5p_R-1_<br>1ss12AC	TAGGTA<br>GTTTCC<br>TGTTGT<br>TG	20	2	gp1a	..((((<br>(....(<br>(..((.<br>((((((<br>((((.(<br>(((((.<br>((((..<br>......<br>...)))<br>).))))<br>)).)))<br>))))))<br>).))..<br>))..))<br>)))..	0	0.76	0	0.60	0	0
gga-miR-199-3p_R-2_<br>1ss13CA	TACAGT<br>AGTCTG<br>AACATT	18	1	gp1a	...(((<br>((((((<br>(.....<br>..))))	0	0	0	0	1.06	0

					))..))<br>))...						
gga-miR-199-3p_L-1R<br>+2_1ss2AT	TCAGTA<br>GTCTGC<br>ACATTG<br>GGT	21	3	gp1a	...(((<br>((.(((<br>((((((<br>((((..<br>......<br>....))<br>))))))<br>..))))<br>)...))<br>)))...	0	0	0	0	1.06	0
gga-miR-200b-3p_R-4<br>_1ss1TC	CAATAC<br>TGCCTG<br>GTAATG<br>A	19	3	gp1a	.....(<br>(.((((<br>(((((.<br>..))))<br>).))))<br>.))...	0	0	0.64	0	0	0
gga-miR-200a-3p_R+1<br>_2ss18GT21GA	TAACAC<br>TGTCTG<br>GTAACT<br>ATATT	23	chr21	gp1a	((((((<br>((.(..<br>((((((<br>((((((<br>((((((<br>.((((.<br>......<br>......	0	0.03	0.02	0	0	0

					.)))).)))))))))))))).))))..).))))).)).))					
gga-miR-202-5p_L-1	TTCCTATGCATATACTTCTTT	21	6	gp1a	...((((..((.((((((((((((.(((.(((((((.(((......))))))))).))).)))))))))))).))..))))...	0	0.76	1.28	7.15	6.37	5.89
gga-miR-204_R+1	TTCCCTTTGTCATCCTATGCCTA	23	10	gp1a	...(((((.....(((...(((((.(((((	0	4.19	0.96	0	0	0.98

hsa-miR-204-3p	GCTGGG AAGGCA AAGGGA CGT	21	Z	gp1a	((((.. ((((.(((((.. ....)))))))))..))))))))).)))))...)))))))) ... ...(((((...))))).. .(((((...(((((.((((((((((.(((((.((((...... ....))))))))) .)))))	0	1.53	0	0	0	0

					)))))).<br>)))))).<br>..))))<br>)..						
gga-miR-205b	CCCTTC<br>ATTCCA<br>CCGGAA<br>TCTG	22	1	gp1a	...(((<br>(..(((<br>(((..(<br>((((((<br>.(((.(<br>((((((<br>(...((<br>......<br>))))))<br>)))).)<br>)).)))<br>))))..<br>))))))<br>..))))<br>...	0	0	0	30.96	31.83	39.24
gga-mir-205b-p3	GATTTC<br>AGTGAA<br>ATGAAG<br>CCC	21	1	gp1a	...(((<br>(..(((<br>(((..(<br>((((((<br>.(((.(<br>((((((<br>(...((	0	0	0	1.19	1.06	0

					......<br>))))))<br>)))).)<br>)).)))<br>))))..<br>))))))<br>..))))<br>...						
gga-miR-214_1ss19CT	ACAGCAGGCACAGACAGGTAG	21	Z	gp1a	...(((<br>(...((<br>.(((((<br>(..(((<br>(.....<br>))))..<br>))))))<br>.))...<br>..))))<br>......<br>..	0	1.53	0.32	0	0	0
gga-miR-214_R-1_1ss9CT	ACAGCAGGTACAGACAGGCA	20	4	gp1a	...(((<br>((((..<br>......<br>...((.<br>((((..<br>((((..<br>....))	0	0.38	0	0	0	0

					))...(<br>((((((<br>((....<br>))))).<br>))))..<br>..))))<br>.)))))<br>))))..<br>......<br>...						
gga-miR-214_L-2	AGCAGG<br>CACAGA<br>CAGGCA<br>G	19	3	gp1a	...(((<br>...(((<br>(.....<br>(((((.<br>..(((.<br>.((..(<br>((((((<br>(.....<br>......<br>......<br>..))))<br>))))..<br>.))..)<br>.))...<br>.)))))<br>))))..	0	0.76	0.32	0	0	0

					.)))...						
gga-mir-214-p3	AGAACATCCTCTCACCTGT	19	12	gp1a	...(((((((((..((((.(((........).)).)))).)).)))).)))...	0	0	0	0.60	0	0
gga-miR-214_L+1_1ss11AT	TACAGCAGGCTCAGACAGGCAG	22	18	gp1a	....((..(((((....)).)))..))...	0	0.38	0	0	0.53	0
gga-miR-214_L+1R-3_1ss19GT	TACAGCAGGCACAGACAGT	19	4	gp1a	...((((((((....((((((((((...)))).((((((.............))))).....)	0	0.38	0	0.60	0	0.98

					)))))...))))))))...						
gga-mir-214-p5_1ss21AT	TGTCTACACTTGCTGTGCAGT	21	1	gp1a	...(((....((((((..(((((...(((...)))...)))))..))))))...((((.....))))).)))...	0	0	0	0	0	0.98
gga-mir-216c-p3	TCACATTTGCCTGCAGAGATTT	22	3	gp1a	...(((((((..((((((((.(((((((.(((((..((........))..))))	0	1.53	1.28	0	0	0

					).))))))).))))))))..)))))))...						
gga-miR-219a	TGATTGTCCAAACGCAATTCT	21	17	gp1a	...((((((((((((...(((.(((((.(((((((((((((((((...).)))))....)))))))))))).))))))))...))))))))))))...	0	0	0.64	0	1.06	0
gga-miR-221-3p_R-1_1ss5AG	AGCTGCATTGTCTGCTGGGTTT	22	JH376175.1	gp1a	....(((..(.(((((((..((.	0	0.38	0	1.79	0.53	0

					((((((...))))))(((.((((. ..)))) .(((((((((.. ..)))))))))))).)). .)))))))))..)))...						
gga-miR-221-3p_L-3_1ss23CT	TACATTGTCTGCTGGGTTTT	20	4	gp1a	....(((((((...(((((.. ..))))))))))))...	0	0.38	0.32	0	0	0
gga-miR-223_L+1R-2	GTGTCAGTTTGTCAAATACC	20	4	gp1a	....((((...((..... ...)). .)))).	0	0.76	0.64	0	1.06	0

					..						
gga-miR-223_R-1_1ss18CT	TGTCAGTTTGTCAAATATCC	20	3	gp1a	.......(((.....(((((((.........)))))))......)))...	0	0.76	0	0	0.53	0
gga-mir-301a-p5_1ss1GA	ACAGTGCAATAATATTGTC	19	3	gp1a	...((((((......)))))).....	0	0.38	0	0	0	0
gga-miR-301a-3p_R-3_1ss22GA	CAGTGCAATAATATTGTCAAAA	22	3	gp1a	...((((((......)))))).....	0	0.38	0	0	0.53	0
gga-miR-301a-3p_L-7R+1	ATAATATTGTCAAAGCATT	19	3	gp1a	........((((.....((((((.........))))	0	0.38	0	0	0	0

					))......))))….						
gga-miR-301a-3p_R-6_1ss19AT	CAGTGCAATAATATTGTCT	19	8	gp1a	...(((((((..((.....))...)).)))))...	0	0	0.32	0	0	0
gga-miR-302d_R-2	TAAGTGCTTCCATGTTTTAGT	21	4	gp1a	...(((((.((..(((((..(((((((((((((((((.............))))))))))))))))..))))).)).))))...	0	0	0	0	1.06	0
gga-miR-302c-3p_L-1_1ss23GT	AAGTGCTTCCAT	22	chr4	gp1a	((((..((..((	0	0	0	0	0.35	0

	GTTTCA<br>GTGT				(((((.<br>.((((.<br>((((.(<br>(.....<br>))..))<br>)).)))<br>)..)))<br>))))..))..))<br>))						
gga-miR-302a_L+1R-2	AAAGTG<br>CTTCCA<br>TGTTTT<br>AGT	21	4	gp1a	...(((<br>((((.(<br>((..((<br>(.(((.<br>.((((.<br>(..(((<br>.((((.<br>......<br>)))).)<br>))..).<br>))))..))).))<br>)..)))<br>.))).)<br>)))...	0	0	0	0	2.12	0
gga-miR-365-2-5p_L-	AGGGAC	23	18	gp1a	......	0	3.05	3.85	30.96	16.97	29.43

1R+1	TTTCAGGGGCAGCTGTG				....((((((((.((((((((..(((.((((((((((((.(((.....)).))))).....)))))))))))..)))))))).))))))))...						
gga-miR-365-1-5p_L-1R+1	AGGGACTTTTGGGGGCAGATGTG	23	14	gp1a	..(((((..(((.(((..((....(((.((((((..((((((((..((((((((((((((((..	0	0.76	1.92	11.91	9.55	0

gga-miR-365-3p_R+4	TAATGC<br>CCCTAA<br>AAATCC<br>TTATTT<br>CT	26	14	gp1a	.....)<br>))).))<br>)....)<br>))))))<br>)))).)<br>))))))<br>)..)))<br>))).))<br>).....<br>..))..<br>))).))<br>)..)))<br>))...<br>..((((<br>(..(((<br>.(((..<br>((....<br>(((.((<br>((((..<br>((((((<br>((..((<br>((((((<br>((((((<br>((((..<br>.....)<br>))).))	0	0	0.64	0	0	0

					)....)<br>))))))<br>)))).)<br>))))))<br>)..)))<br>))).))<br>).....<br>..))..<br>))).))<br>)..)))<br>))...						
gga-mir-449a-p3_1ss11CT	TCAGCTAACATGCAGTTGCTAAC	23	Z	gp1a	.(((((<br>(..(..<br>((.(((<br>(((((.<br>.(((((<br>((((((<br>..(...<br>..)..)<br>)..)))<br>))))))<br>..)).)<br>))))).<br>))..).<br>.)))))<br>).	0	0	0	0	1.06	0

gga-miR-449c-5p	TGGCAGTGCGTGTTAGCTGGCTGT	24	Z	gp1a	(..((((((.(((((.(((.(((.((((((((.((...))...)))))))))))).))))))))))))..)	0	0	0.64	0	0	1.96
gga-mir-451-p5	TTTAGTAATGGTAACGGTT	19	3	gp1a	...((.((.(((.((((((..(((((((((...((((...((((((...(.(((((((..((((((((.((..(((...)	0	0	0	0	0	0.98

					)))...<br>....))<br>.)))))<br>))))..<br>))....<br>.)))))<br>......<br>))))))<br>..))))<br>......<br>.)))))<br>))))..<br>....))<br>))))))<br>))).))						
gga-miR-451_R-4_1ss13TG	AAACCGTTACCAGTACTGA	19	5	gp1a	..((((<br>.(((((<br>((..((<br>..((((<br>((....<br>...(((<br>(..(((<br>((....<br>(((((.<br>....((<br>((..((	0	0	0.32	0	0	0

					(((... ..)))))..))))..... ..)))))....)).))). .)))). ...)))))).)) ..)))))))))))..						
gga-miR-454-3p_L-7R +2	ATATTG CTTATA GGGTCT	18	4	gp1a	(((((. ....)))))...	0	0	0	1.19	0	1.96
gga-miR-454-3p_R-2_ 1ss5GT	TAGTTC AATATT GCTTAT AGG	21	13	gp1a	....(((...(((((... ..)))))..))) ...	0	0	0	0.60	0.53	0
gga-miR-454-3p_L-4R	GCAATA	18	1	gp1a	((((((	0	0	0.32	0.60	0.53	0

-1	TTGCTT<br>ATAGGG				(.(((.<br>...)))<br>))))..<br>..))).						
gga-miR-454-5p_L-2R<br>+1	CTAACC<br>TTAAGG<br>ATGAGA	18	3	gp1a	...(((<br>((...(<br>((((((<br>..((.(<br>((((((<br>((((..<br>....((<br>(.....<br>...)))<br>..((..<br>..))..<br>))))))<br>.).)))<br>)))..)<br>)))).)<br>)..)))<br>))...	0	0.76	0	0	0.53	0
gga-miR-456-5p_L+1R<br>-6	GCAGGC<br>ATCTTC<br>TCAGCC<br>TA	20	1	gp1a	...(((<br>..((((<br>((((((<br>(((..(<br>((.((.	0	0	0	0.60	1.06	0

					...)).)))))).))))))))))..)))...						
gga-miR-456-5p_L+1R-4	GCAGGCATCTTCTCAGCCTACA	22	3	gp1a	((((..(((...(((((((....(..((((....))))..))))))))..(((((..((.....))..))))))))..))))	0	0.76	0.32	4.76	3.71	11.77
gga-miR-456-5p_L+1R-5	GCAGGCATCTTCTCAGCCTAC	21	7	gp1a	...(((((((.(((......))).)))))))).....	0	0	0.32	0.60	2.65	0

gga-miR-458a-3p_R-4_1ss15GT	ATAGCTCTTTGAATTGTA	18	5	gp1a	....((((..(((((..(((.(((((((.((.......(..((((.((((..(((....((..................))......)))..))))))))..).....))))))).)))).)))..)))))..))))...	0	0	0	0	1.06	0
gga-miR-460a-5p_R-3	CCTGCATTGTACACACTG	19	4	gp1a	........(((.((((((	0	0.38	0	1.79	0.53	0.98

	T				((((((<br>..(((.<br>......<br>......<br>....))<br>))))))<br>)...))<br>))))))<br>)...						
gga-miR-489-5p	TGGTCG<br>TATGTA<br>TGACGT<br>CATT	22	2	gp1a	...(((<br>(..(..<br>(..(.(<br>(((..(<br>(((((.<br>.(((((<br>.(((((<br>((.((.<br>......<br>..))))<br>))))).<br>))))).<br>.)))))<br>)..)))<br>).)..)<br>..)...<br>.)))).	0	0	0	3.57	5.30	0

					..						
gga-miR-489-3p_L-2R<br>+1	TGACAT<br>CATATG<br>TACGGC<br>TGCT	22	2	gp1a	...(((<br>(..(..<br>(..(.(<br>(((..(<br>(((((.<br>.(((((<br>.(((((<br>((.((.<br>......<br>..))))<br>))))).<br>))))).<br>.)))))<br>)..)))<br>).)..)<br>..)...<br>.)))).<br>..	0	0.76	0	9.53	8.49	7.85
gga-miR-490-5p	CCATGG<br>ATCTCC<br>AGGTGG<br>GT	20	1	gp1a	...((.<br>.(..((<br>((((((<br>.(((((<br>((((((<br>((((.(<br>((....	0	0	0.64	5.95	1.06	1.96

					(((...<br>..))).<br>.))).)<br>))))))<br>))..))<br>)))).)<br>))))))<br>)..)..<br>))...						
gga-miR-490-3p_R+1	CAACCT<br>GGAGGA<br>CTCCAT<br>GCTGT	23	1	gp1a	...((.<br>.(..((<br>((((((<br>.(((((<br>((((((<br>((((.(<br>((....<br>(((...<br>..))).<br>.))).)<br>))))))<br>))..))<br>)))).)<br>))))))<br>)..)..<br>))...	0	1.53	2.57	9.53	6.37	11.77
gga-miR-1306-5p_L-1	CCACCT	19	11	gp1a	......	0	0.38	0	0	0	0

R-5	CCCCTGCAAACGT				.....(((........)))..........((((..((((((((.....))))))).....))..)))))						
gga-miR-1306-3p_R-4	TGGACGTTGGCTCTGGTGG	19	19	gp1a	...((((....((((((.....)))))).......))))...	0	0	0.32	0	0.53	0.98
gga-miR-1397-5p_R+1	TGCATTGCGACGGGTTATATCAC	23	1	gp1a	(((..((((..(((((((((((..........))))))))	0	0	0	1.19	0	0

					)))..))))))))...						
gga-mir-1434-p5_1ss18GA	AAATTTGAGTATCTGACA	18	2	gp1a	...(((((...(((((((((.....)))))))))).......))))))...	0	0	0	0	0	2.94
gga-mir-1452-p3	ATATCCTTCTTTCTTGTCTTTT	22	Z	gp1a	.(((((((....(((((((((.((((((((((.............)))))))).))).)))))))))).))))))	0	0	0.64	0	1.06	3.92
gga-mir-1454-p5	GACTTTGGCTCC	19	3	gp1a	...(((((((..	0	0.38	0	0	0	0

	CTCTAC<br>T				...(((<br>(((...<br>..))).<br>)))...<br>...)))<br>))))..<br>.						
gga-mir-1457-p5_1ss<br>4GA	TGGACA<br>ACCTGT<br>TCCAGT	18	8	gp1a	...(((<br>((((..<br>...(((<br>....((<br>.((((.<br>.(((..<br>...(((<br>.(((((<br>(.((((<br>.....)<br>).))))<br>))))..<br>..))).<br>.....)<br>))..))<br>))....<br>))....<br>)))...<br>))))))	0	0	0	0	1.06	0

					)...						
gga-mir-1457-p3_1ss17GA	TGGGCAACCTGTTCCAATG	19	1	gp1a	...((((.....)))).....	0	0	0	0	0.53	0
gga-mir-1457-p5_1ss11AG	TCCTTGGGCAGCCTGTTCC	19	9	gp1a	...(((..((((..(((((.((((((((..(((.....))).............((((........))))..........)))))).....))))))).))))..)))..	0	0	0	0.60	1.06	0
gga-mir-1457-p3_1ss11AG	TCCTTGGGCAGC	19	11	gp1a	...(((((...(	0	0	0	0.60	1.06	0

	CTGTTC C				((.... .))))))))...						
gga-miR-1458	TTCCTG TGATGC TCATGA GAA	21	9	gp1a	....(((((((((((..(......)..).))))).. .))))) ..	0	0	0.64	0	4.24	7.85
gga-miR-1463_L-5R-2 _1ss16GT	ATGATG CTTTTC TGAGAA	18	chr5	gp1a	..((((((.(((.((.(((((... ..((.(((((((...(((((((.. ((((.. ..)))) ...))))))).. .....))...))	0	0	0	0	1.06	0

					))).)))))))).))))).))))))..						
gga-miR-1551-5p	CTAGCAGCAAAAAGAACTTCAGA	23	14	gp1a	..((((((((...(((((.((((.((..............))))))))))))...))))))))).......	0	0	0	9.53	4.24	3.92
gga-miR-1551-3p	TGAGTTTGTGTTGCTGCTTTCT	22	14	gp1a	..((((((((...(((((.((((.((..............))))))))))))...))))))))	0	1.53	0.64	2.38	1.06	1.96

					......						
gga-mir-1553-p5	CCAGTT	20	7	gp1a	..((.(	0	0	0	0	1.06	0
	GGCAGC				(((..(						
	TCTCAC				((((.(						
	AC				(((.((						
					((((((						
					(.((((						
					((.((.						
					......						
					......						
					..))))						
					))))))						
					))))).						
					))))))						
					.)))))						
					.)))))						
					).						
gga-miR-1554_R+1	GCAGGT	22	14	gp1a	((.(((	0	0	0	0	2.12	0
	GATGAG				((....						
	GCTTTG				...(((						
	CTGC				((((..						
					.(((..						
					((((..						
					((((..						
					(((..(						
					((((((						

					......<br>..))))<br>...)))<br>.))).)<br>)))...<br>))))..<br>)))...<br>))))))<br>).))))<br>).))						
gga-mir-1554-p3	GCATTG<br>GCTGGC<br>ACATCT<br>GATT	22	14	gp1a	((.(((<br>((....<br>...(((<br>((((..<br>.(((..<br>((((..<br>((((..<br>(((..(<br>((((((<br>......<br>..))))<br>...)))<br>.))).)<br>)))...<br>))))..<br>)))...	0	0	0	1.19	4.24	0

					))))))<br>).))))<br>).))						
gga-miR-1555-3p	CAGGGT<br>TATTGG<br>TTTTGT<br>GTGA	22	1	gp1a	...(((<br>(((((.<br>((((((<br>(((.((<br>...(((<br>(((((.<br>.((...<br>......<br>))..))<br>))))))<br>....))<br>.)))))<br>.)))).<br>))))))<br>))...	0	0	1.92	0	2.12	1.96
gga-miR-1556	TGCAGG<br>CTGGAA<br>GTAGGA<br>GTGT	22	Z	gp1a	...((.<br>.((..(<br>(((.((<br>(((.((<br>((...(<br>(.((.(<br>(((((.<br>....))	0	1.53	1.28	0	1.06	0

					)))).)<br>)))...<br>)))).)<br>)))).)<br>)))..)<br>)..)).<br>..					
gga-mir-1557-p5	CCAGCC<br>GCTCAG<br>CCGACG<br>GGCA	22	2	gp1a	...(((<br>....((<br>..((.(<br>((((((<br>((((.(<br>((((((<br>((((((<br>((((((<br>......<br>..))))<br>))))))<br>))))))<br>))).))<br>))))))<br>))).))<br>...)).<br>...)))<br>...	0	0.76	0	0	0	0
gga-miR-1560-3p_L+1	AGCATC	24	chr11	gp1a	(((..(	0	0	0.64	0	0	0

R+1_1ss7TG	GCTGGA				((..(.						
	CGCGCT				.((..(						
	CGTTCT				..(..(						
					((((((						
					(..(((						
					(((...						
					(((((.						
					......						
					...)))						
					)).)))						
					)))..)						
					)))).)						
					))..).						
					.)..))						
					..)...						
					.))).)						
					))						
gga-miR-1561_L-3	GGGTTT	18	18	gp1a	...(((	0	0	0.64	3.57	4.24	0
	GCGGCC				((((((						
	GCTTTT				(((.((						
					(..((.						
					.....(						
					(((((.						
					.....)						
					))))).						
					.....)						

					)..))))))))))))))...						
gga-miR-1562-3p	TGTGCCCCGTGTATGCAGTGCA	22	8	gp1a	...((((...(((.((((((((((......(((((.(((.(((((........))))))))))))......))))))))).))))...))))...	0	0	0	0	0	1.96
gga-miR-1564-5p_L+3R-1	GCTGCAATGAACACGGCAGTAGAGG	25	14	gp1a	...((((((((..(.(.....).)..))))))))).(	0	0.76	0	1.19	1.06	0

					((((((.((.(((.........))))))))))))..						
gga-mir-1564-p3	GGTAGCGCTGTGGTTGGAAGC	21	14	gp1a	...((((((((..(.(.....).)..)))))))).(((((((.((.(((.........))))))))))))..	0	0.76	0	1.19	0	0
gga-miR-1565_L-2R-3_1ss10TG	AGGGTCGGGCCTGGTTTT	18	chr28	gp1a	((((((((((((((((.(((.(((.(((.(((((((((.........))	0	0.76	0	0	0	0

					))))..)))))))))))..))))))))))))))))						
gga-miR-1572	GAACCTGACACACGGGCCTGC	21	12	gp1a	.((((((((((...((((.((((.(((((((...(((.((.........)))..)))..))))))).))))).)))))..))))))))).))	0	0.76	0.64	0	0	0
gga-mir-1572-p3_1ss2AC	TCTTCCTGCTTGGGAACC	18	28	gp1a	...(((((((((((((..((((((..(.((	0	0	0	1.19	0	0

					......)))..))))))).))))...(((....))).))))))))))...........						
gga-miR-1573_R+3	TGTTCTGCAGGTGCCAGTCTGAG	23	4	gp1a	...((((.((.(((((((((.((..(((.(.(((..(((.......))) ..))))).))).)).))))))))).)).))))...	0	0	0	0	0	1.96
gga-miR-1574-3p_R+1	ACAGGAGGATGT	23	chr10	gp1a	((((....((((	0	0	0	0	0	1.96

	CAGGAA<br>GCTTT				(.((((<br>((((((<br>(.((((<br>(.((..<br>..))..<br>.)))))<br>))))))<br>).))))<br>))).))<br>....))<br>))						
gga-mir-1579-p3_1ss<br>20GA	GTGGCG<br>GGAAGG<br>GATGGG<br>CA	20	6	gp1a	((((((<br>((((((<br>(((((.<br>.((...<br>.))..)<br>))))))<br>.....)<br>)).)))<br>))))..<br>......<br>..	0	3.81	0	1.19	1.06	0
gga-miR-1580	CGGCTT<br>CTCGGT<br>ACCTGC<br>GTT	21	18	gp1a	...(((<br>..((((<br>..(.((<br>(..(((	0	0	0	1.19	0	0

					(((((.(((...............))))))))))).))).).)))).)))... .						
gga-miR-1585_R+1	AGTGCCCAGGTGTGGTTAATCCTGT	25	19	gp1a	...((((((...(((((..((..(.....(((.(((((.....)))))).)))).))..)))))...))))))...	0	0.76	2.57	1.19	0	0
gga-miR-1587_R-2	GGGCTGGGCTGGGCTGGG	18	1	gp1a	...(((..((..((((((	0	0.38	0	0	0	0

					(.....<br>...)))<br>))).).<br>.))..)<br>))....<br>....						
gga-miR-1587_L+2R-1	ACGGGC<br>TGGGCT<br>GGGCTG<br>GGC	21	chr19	gp1a	((((..<br>((.(((<br>(((.((<br>(..(((<br>..((((<br>.(((.(<br>((((..<br>((((..<br>..))))<br>.)))))<br>))))))<br>)...))<br>)...))<br>))))))<br>)..)).<br>..))))	0	0	0	0	1.06	0
gga-mir-1590-p5	TCCAGA<br>GAACAG<br>TTTGTC<br>CCACT	23	6	gp1a	...((.<br>(((..(<br>(.((((<br>..((((	0	0.76	0	0	0	0

gga-miR-1593_L+1	CACCAT<br>CTGATT<br>TGGACA<br>CGCA	22	1	gp1a	(..(((<br>..((((<br>((((.(<br>((((((<br>((((..<br>(((((.<br>.((((.<br>...)))<br>).))))<br>))))))<br>))))))<br>.)))))<br>)))..)<br>))))))<br>))))).<br>))..))<br>).))..<br>.<br>...(((<br>(....(<br>(((((.<br>((((..<br>.(((((<br>((..((<br>((((((<br>..((..	0	0	0.64	0	1.06	0

					...)).)))))))).))))))).))))...)))))).....))))...						
gga-mir-1594-p3_1ss17CT	GATCTTGGAGGTCTTTTCC	19	Z	gp1a	..(((...))).(((((.(((((((....(((.((((..(((((((((..(...)..)))))))..)))))))))))))))))))))	0	0	0	0	0	1.96
gga-mir-1595-p5_1ss16GC_2	AACAAGCACGAGTGTCGT	20	8	gp1a	......((((.....)))	0	0.38	0	0	0.53	0.98

	GG				)...					
gga-mir-1595-p5_1ss16GC_1	AACAAGCACGAGTGTCGT	18	8	gp1a	.....((.((.....)).(((((((...)))).))))).....)).	0	0.38	0	0	0.53	0.98
gga-mir-1595-p3_1ss23CT	GCTCATGGAGCCTCCCCTTAGCT	23	8	gp1a	.....((.((.....)).(((((((...)))).))))).....)).	0	0	0.32	0	0	0
gga-miR-1595-3p_L+1R-1_1ss14CG	TTAGCCCCGCTGTGCTACTGCT	22	8	gp1a	....((((.....(..((((.(((...((......))...)))).))))..)...	0	0	0.32	2.38	0	0

					..))))					
gga-miR-1596-5p	ATTCTC<br>CTTCAA<br>TCCATC<br>TTTGT	23	chr1	gp1a	.(((((<br>(.((((<br>(((((.<br>(((...<br>..((((<br>((((((<br>(.....<br>......<br>...).)<br>))))))<br>)))...<br>.))).)<br>))))))<br>)).)))<br>..))).	0	0	0	0	0	1.96
gga-miR-1597-5p	ATGTTT<br>ACAGGG<br>CTCTTC<br>ATT	21	2	gp1a	...(..<br>((((((<br>((.(((<br>((((.(<br>((((((<br>((((((<br>(((...<br>....))<br>)..)))<br>))))))	0	0	0	1.19	0	3.92

					)))).)<br>))))))<br>.)))))<br>)).)..<br>)...						
gga-miR-1602	TGGGCT<br>CTGCAT<br>CACCCC<br>ATGG	22	4	gp1a	...(((<br>(.((.(<br>((((..<br>((((((<br>.(((..<br>((((..<br>((((((<br>((....<br>))).).<br>))))))<br>))..))<br>).))))<br>))..))<br>))))).<br>))))..<br>.	0	0.76	0	2.38	3.18	1.96
gga-mir-1603-p5	TACAGC<br>ACGCAG<br>TCCCAG<br>CCAGCT	24	7	gp1a	...((.<br>((((((<br>(.((((<br>((((((<br>(.(((.	0	0	0.64	0	1.06	0

					.(((((((.((((.((....)).))))))))))).))))))))).)))).)))))))))...						
gga-miR-1604	TGGGCCCAGGGCTGTGCTGGAGA	23	1	gp1a	...((((((((..((.(((.((((((.((((((..((......))..))))))))))).))).))..)))))))...	0	1.53	0	1.19	4.24	1.96
gga-miR-1609	AGGCTGAGGCTG	22	13	gp1a	..((((((((((	0	0	0	0	0	0.98

	TGCTGG<br>TTGT				((....<br>((.(((<br>(((((.<br>((((((<br>(((...<br>...)))<br>.)).))<br>))))))<br>)).)))<br>)....)<br>))))))<br>))))).<br>.						
gga-mir-1609-p3	AAGCTG<br>CGCAGA<br>CCCACA<br>GGC	21	13	gp1a	..((((<br>((((((<br>((....<br>((.(((<br>(((((.<br>((((((<br>(((...<br>...)))<br>.)).))<br>))))))<br>)).)))<br>)....)<br>))))))	0	0	0	0.60	1.06	0.98

					))))))..						
gga-miR-1610_L-2R+2	GCTTGTGGTGGAACGGGCGGC	21	8	gp1a	...(((((.((.((((((((.(((....(((.((((((((((((.(((((((((...((..((.......))..))((....)).))))))).)))).))))))).)))).)))))))))))))))..))))))...	0	0	0.64	1.19	0	0
gga-mir-1612-p5_1ss1CT	TTCTTTTCTTTC	22	1	gp1a	...(((((((((	0	0	0	0	1.06	0

	TTTCTT				.((...						
	TTTT				((((((						
					(.....						
					.((..(						
					.....(						
					((((..						
					.(((..						
					((((((						
					((...(						
					(((..(						
					..(...						
					..)..)						
					..))))						
					.)))))						
					))).))						
					)...))						
					)))...						
					)..)).						
					))))))						
					)...))						
					))))))						
					)))...						
gga-mir-1613-p5	ACTGAT	18	2	gp1a	...(((	0	0.38	0	0	0	0
	GTAGTG				((((.(						
	AAGATG				(.((..						
					(((((.						

					..((((<br>(((((.<br>......<br>......<br>))))))<br>).))..<br>))))).<br>.)))).<br>.)))))<br>))...						
gga-miR-1613_R+1	TACATG<br>CGTATA<br>TTCTGG<br>CAGT	22	2	gp1a	...(((<br>((((.(<br>(.((..<br>(((((.<br>..((((<br>(((((.<br>......<br>......<br>))))))<br>).))..<br>))))).<br>.)))).<br>.)))))<br>))...	0	0	0	1.19	4.24	1.96
gga-miR-1614-5p	GGCATG<br>GCAGAC	21	chr20	gp1a	((((((<br>((((((	0	0	0	0	0	1.96

	TCACCCTGC				(.((.((((((..((.((((((...((((...))))))))))).))..)))))))..)))))))))))))						
gga-miR-1616_L-1R-2_1ss12GA	GGATCCTGCAATTTGCTTT	19	11	gp1a	...((((((((..(((..((((((((((.((..((((.(((((.((.....)))).)))))))))).))))))))))..))).))).	0	0	0	0	1.06	0

					)))))).<br>..						
gga-miR-1622	CACATA<br>TGATGA<br>GTACTG<br>ATG	21	2	gp1a	...(((<br>((((..<br>.(((((<br>(((((.<br>.(((((<br>..((((<br>(.((((<br>((((((<br>.....)<br>))).))<br>.))))<br>..)).)<br>))..))<br>)))...<br>...)))<br>))..))<br>)))...<br>...)))<br>))))..<br>.	0	0	0	1.19	0	1.96
gga-miR-1625-5p	TGGACC<br>AGGGCT<br>CTTCCT<br>GCTGGC	25	15	gp1a	(((..(<br>((((..<br>....((<br>((((.(	0	1.53	0.64	1.19	4.24	1.96

	T				(..((....)).)))))))).....))))))))...(((.(..((((((........))))))..)))).....						
gga-miR-1626-3p_R+1	TCTGGAAGTTGCCCTGGACGTGC	23	1	gp1a	((.(..((((((((((..((((.((((.((.((((((((.....((((((...))))))))))))))))).))))..)))	0	0.76	0	0	1.06	0

					))).))<br>)..)))<br>.						
gga-mir-1628-p5_1ss13AG	TCACAACAGCTCGACTTCCTTC	22	4	gp1a	((.(((((((((......(((.((((((......)).)))).)))......)))))))))).))	0	0	0	1.19	0	1.96
gga-miR-1628_L-1	AGAGCTCTTCCTGTTGTGACC	21	4	gp1a	((.(((((((((......(((.((((((......)).)))).)))......)))))))))).))	0	0	0	1.19	0	0
gga-mir-1630-p5_1ss	CTTTCT	19	23	gp1a	...(((	0	0	0	0.60	0.53	0

7AG_1	GAAGGG<br>GGAGGA<br>A				(..(((<br>(...((<br>(((...<br>.(((((<br>.((.((<br>(....(<br>(.(((.<br>..((((<br>(.....<br>...)))<br>))...)<br>))))..<br>))).))<br>.)))))<br>.)))))<br>))))..<br>))))						
gga-miR-1631_L+4R-1	AGCGAA<br>ACTGGC<br>GACGTG<br>GGACGG<br>T	25	Z	gp1a	((((((<br>((...(<br>(((...<br>.((((.<br>((.(((<br>..((((<br>...)))<br>)...))<br>))))))	0	0	0	1.19	0	1.96

					)......))))..)))))))))						
gga-miR-1636_R+1	TGCAGGTGATGGCGGGGCTGT	21	15	gp1a	...((((((((.((((.((.(((((((((..((((.((((((((.....)))..))))))))...))))))))).)).))))....)))))))...	0	0	0	1.19	0	0
gga-miR-1641_R+1	TGAGGATTAATGACTGTCTGGGA	23	chr3	gp1a	...((((((.(((((((((.(((((((((((((..(((	0	0	0	1.19	0	0

					(((.........))))))..))))))))))).)))))))).)))))).........						
gga-miR-1643-5p_L+1R+1	TTCTTATCACGAGGAGCACTTTTT	24	11	gp1a	...(((((.((...((((((.((((.((.((((((((.(((((....)))))).))))))))).)).))))))))))...)).))))...	0	0	0	0	2.12	0
gga-miR-1646_L-2R-3	TGACCT	18	10	gp1a	...(((	0	0	0	0	0	1.96

_1ss5CA	GCAGCA<br>CTGAGA				(((.((<br>(((...<br>.((((.<br>....))<br>))))))<br>)..)))<br>)))...						
gga-miR-1648-3p_L+1<br>_1ss22CT	GTGGGA<br>GTGGAG<br>CGGAGC<br>TGTT	22	20	gp1a	..((((<br>((((((<br>..((((<br>((.(((<br>((((((<br>.(((((<br>.((((.<br>...)))<br>)....)<br>)))).)<br>))))))<br>)).)))<br>))).))<br>))))..<br>))))..	0	0	0	0	1.06	0
gga-miR-1649-3p_R+2	CTCCGT<br>GCACTC<br>TGCAGG<br>CACC	22	3	gp1a	...(((<br>((..((<br>(((...<br>((((((	0	0	0	0	1.06	0

					((..(..(((((((.............))))). .))..)..))))))))...)))))).)))))...						
gga-mir-1650-p3	AACTCCTCTGAGCTCAGC	18	JH376281.1	gp1a	...((.((((((((((((....(((((.................))))))))))).)))))).))...	0	0	0	0	0.53	0
gga-mir-1650-p5	AGGCCAGGGAGCAGCCACAGCAG	23	2	gp1a	.......(((.(((.(((((((((..(((	0	0.76	0	2.38	0	0

					((((((.(((.......)))..)))).))).))))))))).)))).))).)))...						
gga-miR-1650	TCCTCTGAGCTCAGCTTCGCCTCT	24	2	gp1a	.......(((.(((.(((((((((..(((((((((.(((.......)))..)))).))).))))))))).)))).))).)))...	0	0.76	0	0	1.59	1.96
gga-miR-1655-3p_L-1	TTTACC	22	8	gp1a	((.(((	0	2.29	0.64	1.19	3.18	0

R+1	CATGGT<br>GGGCTG<br>ATAC				(....(<br>((((((<br>(.((((<br>((((((<br>(((..(<br>(.((((<br>((((((<br>((...(<br>(....)<br>))))))<br>))))))<br>).))..<br>))))))<br>))))))<br>).))))<br>))))..<br>)))).)<br>)						
gga-mir-1655-p3_1ss<br>1AG	GCACTG<br>TGTCAG<br>CTGGTC	18	23	gp1a	((.(((<br>((...(<br>((((..<br>..))))<br>)...))<br>))))).	0	0	0	0.60	0	1.96
gga-mir-1656-p3	ATGGCT<br>GTGATG	19	1	gp1a	...((.<br>((((..	0	0	0	0	1.06	0

	TCATGA G				((((((..((.(((((((((.(((...))))))))))))).)))))))..))))))...					
gga-miR-1658-5p_1ss23CT	TATACCACCCCCAGGAGTTCTGT	23	chr9	gp1a	((((((((((....((((((..((((..((((((.(((((....)))))....)))))))).))))).))))))....))))))..))))	0	0	0	0	0	1.96
gga-miR-1664-3p_L+1	TTCTGT	22	1	gp1a	...(((	0	1.53	1.28	5.95	8.49	0

	GACCTC<br>ATTTAC<br>CTCC				(((.((<br>(((((.<br>.(((((<br>((((((<br>((((((<br>((((((<br>((.(((<br>.....)<br>)).)))<br>))))))<br>))))))<br>))))))<br>))))..<br>....))<br>))))).<br>))))))<br>...						
gga-miR-1665	TAAGGC<br>CCAGGA<br>GCGCTG<br>CAGC	22	8	gp1a	...(((<br>((((((<br>((((((<br>((.(..<br>(((((.<br>((((..<br>......<br>.....)<br>))).))	0	0.76	0	0	0	0

					)))..).)))))))))))))...))))))...						
gga-miR-1666_R+3	TAACGCCACGGGGCTGAGGCTGAGT	25	19	gp1a	...((((((.(((...((((((...(((((((((............................)))))))))..))))))...))).))))))...	0	0	0	0	1.06	0
gga-mir-1666-p3	CAGCTTCAGAGCGTGGCT	18	19	gp1a	...((((((.(((...((((((...(((((((((.............	0	0	0	1.19	0	0

					......<br>.)))))<br>))))..))))))<br>...)))<br>.)))))<br>)...						
gga-miR-1668-5p	GTGGGC<br>TGGGAG<br>CACAGG<br>CACT	22	LGE22C<br>19W28_<br>E50C23	gp1a	....((<br>.(((.(<br>(.((((<br>(((.((<br>((..((<br>(((.((<br>(..(..<br>......<br>.)..))<br>))))))<br>..))))<br>))))))<br>).)).)<br>))..))<br>......	0	0	0	0	3.18	1.96
gga-mir-1669-p5	TGCGCT<br>GCTCTG<br>CGCCCA<br>AACGGA	24	26	gp1a	...(((<br>(((((.<br>.(((..<br>...(((	0	0	0	0	1.06	0

					((..((<br>((((((<br>((.(((<br>((((((<br>.((((.<br>.((((.<br>..(((.<br>...)))<br>..))))<br>)))).)<br>))))))<br>.)).))<br>))))))<br>))...)<br>))))..<br>.)))..<br>.)))))<br>.)))..<br>.						
gga-miR-1669_R+3	GTGGGC<br>TCAGAG<br>CAGGCA<br>CAGCT	23	26	gp1a	...(((<br>(((((.<br>.(((..<br>...(((<br>((..((<br>((((((<br>((.(((	0	0	0	0	1.06	0

gga-mir-1670-p5_1ss<br>6GC	CAGTGC<br>TGTGCT<br>TCTGCC	18	17	gp1a	((((((<br>.((((.<br>.((((.<br>..(((.<br>...)))<br>..))))<br>)))).)<br>))))))<br>.)).))<br>))))))<br>))...)<br>))))..<br>.)))..<br>.)))))<br>.)))..<br>.<br>((.((.<br>...(((<br>((((((<br>(((.((<br>((.(((<br>((....<br>......<br>))))))<br>))).))<br>))))))	0	0	0	0	2.12	0

					).)))...)))).						
gga-miR-1674_R+1	GGGCTATGATGCTGGATTTTCTGAGC	26	6	gp1a	...((((.((((...((..((..(((((((..(((....((((((..(((..((((.... ..))). .)..)))...))))))....))). .))).))))..))..)).)))).))))...	0	0	0	0	2.12	0
gga-miR-1680-5p	TTCCTCCTGGGTACAACT	22	chr10	gp1a	(((((((((.((((.(((	0	0	0	0	1.06	0

	CTGC				(((((.<br>(((..(<br>.((((.<br>......<br>......<br>......<br>.)))))<br>..))))<br>))))))<br>)..)))<br>).))))<br>)))))						
gga-miR-1682_R+1	TGGGTC<br>AGATGG<br>AGCTGA<br>GGGT	22	14	gp1a	...(((<br>(..((.<br>((....<br>.)).))<br>..))))<br>.	0	0	0	4.76	0	7.85
gga-miR-1685-3p	ACAGTA<br>ATGGTA<br>TGAACT<br>CAGA	22	2	gp1a	...(((<br>(((((.<br>...(((<br>(((.((<br>(((.((<br>(((((.<br>...((.<br>......	0	0	1.28	0	1.06	0

					))...))))))).))))))).))))...))))))))...						
gga-miR-1687-3p	TTGCTGGCTGTTTCTCAGTGA	21	chr20	gp1a	((((..((((((.(((..((((((((((...(((((((..((..........))..))))))).))))))..)))..)))))))))))))	0	0	0.64	0	1.06	0
gga-miR-1689-5p	TCTCTGCTTACAGTGTCT	22	19	gp1a	...((((((.((.(((((	0	0	0	0	0	3.92

	GGGG				(.((((..((((((...((....))...))))))..)))).))))))))))....)))))))))..						
gga-mir-1690-p3	GGCAGAGCTGCTTTCTCTGTGA	22	1	gp1a	..((((((((....((((((((((((((((..(((.(((.((((((((..........)))))).))).))))))))).))).)))	0	0	0	0	1.06	0

					))))))<br>))..))<br>)))).)<br>).						
gga-mir-1690-p3_1ss<br>7GC	GGCAGA<br>CCTGCT<br>TTCTCT	18	13	gp1a	...(((<br>((((((<br>(((((.<br>.(.(((<br>((((..<br>..((..<br>..))))<br>)))))(<br>(.....<br>))....<br>)..)))<br>))..))<br>))).))<br>))...	0	0	0	0	0	1.96
gga-miR-1691_R+1	TCAGCT<br>GACCTG<br>GGCCTG<br>CAGTGC	24	1	gp1a	...(((<br>((..((<br>(..(..<br>((((..<br>(((((.<br>......<br>.)))))<br>.)))).	0	0	1.28	0	0	0

					.)..)))..)))))...					
gga-miR-1692_R-1_1ss12TC	TGTAGCTCAGTCGGTAGAG	19	16	gp1a	(((((((..((..(....)..)).)))))))))......	0	0.76	0	0	0	0
gga-miR-1692_L+1R-2	GTGTAGCTCAGTTGGTAGA	19	9	gp1a	............((((((((((...((....))..))))))))))...	0	0.76	0	0	0	1.96
gga-mir-1696-p5_1ss16GA	CAGCTTGCCTTGTGCAGATA	20	22	gp1a	...((((((..((((.(((....((.((((((.......))))))).))	0	0	0	1.19	0	0

					......<br>))).))<br>))))))<br>))...						
gga-mir-1698-2-p5	TGCAGG<br>GATGCT<br>CCTGCC<br>CTCC	22	19	gp1a	...(((<br>((((.(<br>((((((<br>.(((((<br>((((((<br>...(((<br>...(((<br>((....<br>.)))))<br>.)))..<br>.))).)<br>))))))<br>))))))<br>)).)))<br>).))).<br>..	0	0	0	0	0.53	0
gga-miR-1699_L-1R-2	CAGAGG<br>GACATG<br>GCAGGG<br>C	19	8	gp1a	....((<br>(((...<br>....))<br>)))...	0	0	0	0.60	0.53	0.98
gga-mir-1699-p5	CCCTTG<br>GCATGC	22	11	gp1a	...(((<br>(.((.(	0	0	0.64	3.57	2.12	5.89

	TCCTCT<br>CGGC				(((..(<br>((((.(<br>(....(<br>(....)<br>)..)).<br>))))).<br>.)))).<br>))))))<br>.....						
gga-miR-1699_L-2R-1	AGAGGG<br>ACATGG<br>CAGGGC<br>A	19	1	gp1a	...((.<br>((((..<br>((((..<br>..((..<br>.((((.<br>.....(<br>(((...<br>.(((..<br>..)))(<br>(((...<br>.....)<br>)))...<br>......<br>.)))).<br>))))..<br>.))..)<br>)))..)	0	0.38	0	0	0	0

					))).))...						
gga-miR-1704	ACAGCAGATTGATGGGGCACA	21	9	gp1a	...((((.((((((...((..((((((((..........)))))))..))))))))))))...	0	0	0	1.19	0	0
gga-mir-1705-p3	TGACAAGAAATCTGGAAGTCAGC	23	17	gp1a	...((((...((((((((((((.((((.(((((......)))))).))))))))))))))))..))))...	0	0	0	0	1.06	1.96
gga-miR-1706	TGTGGA	22	2	gp1a	...(((	0	0.76	0.64	2.38	0	1.96

	GGTGGG<br>ATGAGG<br>TTCC				(((..(<br>((((((<br>(((.((<br>((((((<br>(....(<br>(.....<br>.))...<br>)))).)<br>)))).)<br>))))))<br>)))..)<br>))))).<br>..						
gga-mir-1707-p5	AGATGC<br>AGAGCT<br>CGCTGA<br>AATGC	23	17	gp1a	...(((<br>(..(((<br>(((((.<br>((((((<br>.(((.(<br>((....<br>......<br>..))))<br>)).)))<br>))).))<br>))).))<br>)..)))<br>)...	0	0	0.64	0	0	0

gga-miR-1707_R-1	TTTGAGCGGGATCTGTTATCGT	22	17	gp1a	...((((..((((((((.((((((.(((.(((.............))))).)))))).)))).)))..))))...	0	0.76	3.21	0	1.06	3.92
gga-miR-1708	TGGGGTGTGGAATGAATCCTT	21	1	gp1a	...(((((..(..(((((((.((((((.((((((((..((......)..))))))).))..)))))))))))..	0	0.76	0.64	0	4.24	3.92

					).))))<br>)...						
gga-mir-1708-p3	AGGATTGATTGTTCACCCTCATT	23	1	gp1a	...(((((..(..(((((((.((((((.((((((..((......))..)))))))).))..))))))))))))..).))))<br>)...	0	0	0	0	1.06	0
gga-miR-1712-5p_1ss17AG	GGCTCCAGTCTTAACTGCAGGC	22	3	gp1a	..((((((.((((..((((((....(((((..(((.((((....))))..))).	0	0	0	1.19	0	0

					.)))))<br>....))<br>))))..<br>)))).)<br>).))))<br>..						
gga-miR-1716_L-2	CGGGGC<br>GGCTGT<br>GAGCTG<br>AGCT	22	5	gp1a	..((((<br>(.((..<br>(((((.<br>.(((((<br>((((..<br>.(((((<br>(.((((<br>(.....<br>).))))<br>.)))))<br>)...))<br>))))))<br>).))))<br>).)).)<br>))))..<br>.	0	2.29	0.64	5.95	6.37	9.81
gga-mir-1716-p5_1ss<br>21GT	CCCGGC<br>TTCCTG<br>CCGCCC<br>TGT	21	6	gp1a	...(((<br>((((((<br>((.((.<br>((....	0	0	0	0.60	0	0.98

					.))..)<br>))))).<br>))))))<br>)...						
gga-mir-1717-p3	TTTCCT<br>GTCTGC<br>TCAGGG<br>AGTT	22	3	gp1a	...(((<br>.((.((<br>((....<br>((((((<br>(.((.(<br>((((((<br>((....<br>.(((((<br>.(((((<br>(((((.<br>.....)<br>))).))<br>)))).)<br>))))..<br>...)))<br>))))))<br>))..))<br>))))).<br>.)))).<br>))))).<br>..	0	0.76	0	0	1.06	0
gga-miR-1719	AAAGAT	21	12	gp1a	......	0	0	0	0	0	5.89

	GATGAG TTGCTG ATG				(((..((..((. ...)). .))..)))..						
gga-miR-1720-3p	AAGCAA CGAGAG GTCGGT CTGA	22	chr10	gp1a	(((.(((((((((...((((((((((.... ....)))))))))).))))).)))).)))	0	0	0	4.76	0	0
gga-miR-1722-3p_1ss22GT	TAGAGC ATCACC AAGGGG CAAT	22	chr10	gp1a	(.((((((((.(((((.. ..((((..((.((((((((((((. .....))).)))))))))	0	0	0	1.19	0	0

					))....<br>))))..<br>.)))))<br>.))..)<br>))))))<br>..						
gga-mir-1723-p3_1ss15GA	ACTGCTGTACTGAAACACT	19	2	gp1a	...........(((.......)))..	0	0	0	0	0.53	0.98
gga-miR-1725_R+1	TATGATATAGTTAGAGCTGCTT	22	chr5	gp1a	.(((....(((((((.(((((((((.((((.............)))).))..))))))))))))))).	0	0	0	1.19	0	0
gga-miR-1728-5p_1ss23AT	TGGTTGATCCATTGGAAG	23	4	gp1a	.(((((((((.(((....(	0	0	0	1.19	0	1.96

	ACTGT				(.((((<br>(((((.<br>(((.((<br>((((((<br>((....<br>(((((.<br>(((.((<br>((....<br>......<br>.)))).<br>..))).<br>))))).<br>..))))<br>))))))<br>.))).)<br>))))))<br>))))..<br>..))))<br>))).))<br>))...						
gga-miR-1731-5p_R-4	ACTTGA<br>CTGCAG<br>GCACTG<br>C	19	3	gp1a	...(((<br>((.(((<br>(..(((<br>(((...<br>.)))..<br>.)))..	0	0	0	1.19	0	0

					.)))))))))...						
gga-miR-1734	ATATGCCCCAATGTATAAGCT	21	3	gp1a	...(((((((((((((((((((.((((..((((((.....(((....)))......))))))..))))).)))))))))))))))))...	0	5.34	2.57	3.57	4.24	0
gga-mir-1734-p3	TGCTTATGTATTGAGGCATATT	22	3	gp1a	...(((((((((((((((((((.((((..((((((.....(((....)	0	1.53	2.57	0	1.06	0

gga-miR-1736-5p_R+2	AGGGCA<br>ATTCCT<br>GATAGG<br>TGCTGA	24	1	gp1a	)).... .)))))<br>)..)))<br>).))))<br>))))))<br>))))))<br>)))...<br>..(((.<br>(((.((<br>....((<br>(((..(<br>(.(..(<br>((((((<br>(((((.<br>((((.(<br>(.((((<br>((((((<br>((....<br>.....)<br>))....<br>))))))<br>))))))<br>))).))<br>))))))<br>))))..<br>).))..	0	0.76	1.28	0	1.06	0

					)))))).))))))))...						
gga-mir-1737-p5	TCCCACTGCCCCTCAGCAG	19	14	gp1a	...((..((((((.((((......))))....))))))..))...	0	0.38	0	0	0.53	0
gga-mir-1738-p5_1ss17GC	TGCTCAGAGGGATTGGCCT	19	1	gp1a	...((((((..((((((.((((.(((((.(((...((((.(((....)))...))))....))).)))))).))))...))))))....))	0	0	0	0	1.06	0

					))))...						
gga-mir-1738-p3	GGGAAGTCTGTGTCCATTGCT	21	1	gp1a	...((((((..((((((.((((.(((((.(((...((((.(((((....)))...))))...).))).)))))).))))...))))))....))))))...	0	0	0	0	1.06	0
gga-miR-1740-3p_R+1	TCATCCAATTAAACCACAAAT	21	3	gp1a	...((((((((.(((.((((((((..((((..((((......	0	0	0	0	1.06	0

					........))))))))..)))))).)))..))))))))...						
gga-miR-1742_R+2	AATCGTCGTTCGGGATCCGCCC	22	1	gp1a	...((((((..(((((((((..((((((.........))))))..))))))))..))))))...	0	0.76	0	0	1.06	0
gga-miR-1744-5p_L-1R-3_1ss12AG	GCTCTGTTGAGCTCAAAA	18	chr10	gp1a	.((((((((...((((..((((((((((((.((((.(((........)	0	0	0	0	0	1.96

					)).))))).)))))))).)))))..))))...))))))))...						
gga-miR-1745_R-1	TGCTCTGCTTGGCAAACTGCT	21	24	gp1a	...(((((...(((((((((..((((((..(((((.(((((..................))).)))))..))))))..))))))))..)))))...	0	0.76	0.64	0	1.06	1.96
gga-mir-1745-p3_1ss21TC	TCAGGTTGCCGTGCAGAT	22	24	gp1a	...(((((...((((((	0	0	0.64	0	2.12	0

	TACC				(..((((..(((((.(((.......................))).))))))..))))..))))))))..)))))....						
gga-miR-1747-3p	CCTGAATCCCTTCAAGTGGACA	22	2	gp1a	((.((((((((.((..(((.(((.((((....))))))))))..))))))))))).))..	0	0	0.64	1.19	1.06	1.96
gga-mir-1750-p3_1ss2AG	AGGTGGTGCTCTGTGCCT	20	5	gp1a	.((.((..(...)..)).	0	0	0	0	0.53	0

	CT				))						
gga-mir-1751-p3	ATGAAG<br>GACGAG<br>GGCAGA<br>CCGC	22	4	gp1a	...(((<br>(.((((<br>(((.((<br>(..(((<br>((((..<br>((((.(<br>((((((<br>......<br>......<br>))))))<br>).))))<br>..))))<br>)))..)<br>)).)))<br>)).)))<br>)))...	0	2.29	0.64	4.76	4.24	1.96
gga-miR-1754-5p	TCGTGC<br>TGGAGC<br>TGTTCT<br>GG	20	9	gp1a	...(((<br>((((((<br>((((((<br>..((((<br>...(((<br>......<br>)))...<br>.)))).<br>.)))))	0	3.05	1.92	15.48	14.85	21.58

					).))))<br>))))).<br>..						
gga-miR-1754-5p_R+3	TCGTGC<br>TGGAGC<br>TGTTCT<br>GGCAA	23	chr9	gp1a	...(((<br>((((((<br>((((((<br>..((((<br>...(((<br>......<br>)))...<br>.)))).<br>.)))))<br>).))))<br>))))).<br>..	0	0	0	0	1.06	0
gga-miR-1754-3p_L+3<br>R-1	CCCAGG<br>GCAGCT<br>CACAGC<br>ACGGG	23	9	gp1a	...(((<br>((((((<br>((((((<br>..((((<br>...(((<br>......<br>)))...<br>.)))).<br>.)))))<br>).))))<br>))))).	0	0	0	1.19	0	0

gga-miR-1756b_R-1	TTCTCTGATTTACACTAGCTGA	22	chr3	gp1a	...((((((.(..(.((((((((((((((((...(((((..((....))....))))..)))))))))))))))).)..).))))).	0	0.76	0	0	0	0
gga-miR-1759-5p	TAGGGTAGGAGGAGAGAGGTGT	22	2	gp1a	...(((((...((((((...(((..((((((((((((((((........))).)))).)	0	0	0	10.72	5.30	1.96

					))))).)))).)))...)))))))))))...						
gga-miR-1759-3p_L-3	TCCTCTCCCATCCTCTCCTTCT	22	2	gp1a	...((((...(((((...(((..(((((((((((((((.........))).))))).))))))..)))).)))...)))))))))))...	0	0	0	1.19	2.12	1.96
gga-miR-1759-3p_L-3_1ss5CG	TGCTCTCCCATCCTCTCCTTCT	22	chr2	gp1a	((((((((((((.(((((((((((	0	0	0.64	0	0	0

					((((((........))).)))).))))).)))).)).)..)))))))))						
gga-miR-1764-5p_R+1	GCTCAGGCAGCAGGAGGCTTGT	22	15	gp1a	.......(((.((((..((..(((((.(((((((..(((((...((((....))))))))))..))))))).)))))..))..)))))))	0	0	0.64	0	0	0
gga-miR-1764-3p_R+1	AGCTGCTTGTTG	22	15	gp1a	.......(((.(	0	0	0.64	0	0	0

	GCTGGGGAGC				(((..((..((((((.(((((((..(((((...((((....)))))))))))..))))))).)))))..))..)))))))						
gga-miR-1765_L+1_1ss21GT	GCGGAGCGCGGCCCTGCGCTT	21	18	gp1a	..((((((..((.((((((((((.((.((..((((.....((.((((((....)))))).))..)))..)).)).)))	0	0.76	0	1.19	0	0

					))).))<br>)))..)<br>))))).<br>.						
gga-miR-1765_R+1	CGGAGC<br>GCGGCC<br>CTGCGC<br>TGT	21	JH3763<br>49.1	gp1a	.(((((<br>((((((<br>((.(((<br>((((((<br>.(((((<br>((....<br>.).)).<br>)))).)<br>))))))<br>)).)))<br>))))))<br>)).))	0	0	0	0	2.12	1.96
gga-mir-1768-p5	CATCAG<br>CATGAC<br>AATCCT<br>CTGC	22	2	gp1a	...(((<br>..((..<br>.....(<br>((.((.<br>..((((<br>((((((<br>((((((<br>((((((<br>((((((<br>(((((.	0	0.38	0	0	0	0

gga-miR-1769-5p	CTTCAG<br>GCTTTT<br>CTCACA<br>CCTG	22	15	gp1a	......<br>...)).<br>))))))<br>))))))<br>))))))<br>))))))<br>))))))<br>)..)))<br>))))..<br>)))...<br>...(((<br>((((((<br>((((((<br>(((...<br>(((..(<br>(((.((<br>(((((.<br>....((<br>((((((<br>((....<br>....))<br>))....<br>))))))<br>.....)<br>))))))<br>.)))).	0	3.81	0.64	0	1.06	0

					.)))))))))....)))))))..)))))...						
gga-miR-1771_L+1	CCTCCAAGGCTGTTTCCTCTGA	22	5	gp1a	((((..(((..((((.(((.....((.(((((..(((..(((((((.((....((((((.((((..(.......).....))))))))))..))..))))))))..))).....)))))))..))	0	0	0	2.38	0	1.96

					)))))..)))....))))...						
gga-miR-1774	CCCCTGCTGAGGTTCTGCTTC	21	4	gp1a	...(((....((((.......))).)))...	0	0	0.64	0.60	0	0
gga-mir-1775-p3_1ss8AT	AGCCAGATGATTCTGATC	18	1	gp1a	...((((((((.......))))))))...	0	0	0	1.19	1.06	0
gga-miR-1778_L+4R-1	GTTGGAGGGAAGAGAAATGGTCTGC	25	13	gp1a	...(((.((((((((...((((((((((..(..((........))...)..))))))))))...))	0	0	0	0	2.12	0

					))))))<br>.)))..<br>.						
gga-mir-1778-p3	CTGGAG<br>GACTGT<br>TTATTT<br>AAATTG<br>GC	26	13	gp1a	...(((<br>.(((((<br>(((...<br>((((((<br>((((..<br>(..((.<br>......<br>.))...<br>)..)))<br>))))))<br>)...))<br>))))))<br>.)))..<br>.	0	0	0	0	1.06	0
gga-miR-1779	AGACGT<br>GGACTG<br>GAACAC<br>CTGAG	23	Z	gp1a	((((((<br>(..(((<br>(((((.<br>((.(((<br>((..((<br>(..((.<br>((..((<br>(.....<br>.))))..	0	0	0.64	0	0	1.96

					.)))).<br>.)))..<br>))))).<br>)).)))<br>))))).<br>.)))))<br>))						
gga-miR-1783_R+1	TGCTCT<br>GAACGA<br>GCTGAG<br>AGGCAT	24	12	gp1a	...(((<br>((((((<br>(...((<br>..(((.<br>.(((((<br>(.((.(<br>((....<br>......<br>......<br>.))).)<br>))))))<br>)..)))<br>..))..<br>))))))<br>)))).. .	0	0	0	1.19	1.06	1.96
gga-mir-1783-p3	CATCCA<br>GCTCTC<br>TCATTG	21	12	gp1a	...(((<br>((((((<br>(...((	0	0	0	0	1.06	0

	CTC				..(((..(((((.((.(((.............))).)))))))))..)))..))..))))))))))....						
gga-mir-1784-p3_1ss20AT	TGACTTAAATAGGAGCAGAT	20	8	gp1a	...(((((((((.(((((((((((((.....((....)).)))))))))).......)))))))))))))...	0	0.38	0.32	0	0	0

gga-mir-1784-p5_1ss20AT	TGACTT<br>AAATAG<br>GAGCAG<br>AT	20	1	gp1a	......<br>....((<br>((.(((<br>(...((<br>((((((<br>(.(((.<br>....))<br>))))))<br>))))..))))..<br>.)))).<br>..	0	0.38	0.32	0	0	0
gga-miR-1784-5p_R-2_1ss19CT	TTCTGC<br>TCTTAT<br>TGAAAT<br>TA	20	3	gp1a	...(((<br>(((...<br>......<br>((((((<br>((((((<br>((.(((<br>......<br>...)))<br>.)))))<br>))))))<br>))))))<br>)))...	0	0.38	0.32	0	0	0
gga-miR-1786_L-1	TTCTTT<br>TCTGCT	20	7	gp1a	...(((<br>((.(((	0	0	0	0	0	0.98

	GTGTTA<br>CT				(((...<br>..))))<br>)).)))<br>))....<br>......<br>.						
gga-mir-1786-p5	TAACAC<br>AACAGG<br>AAACAA<br>ATTC	22	14	gp1a	...(((<br>(....(<br>((.(((<br>..(((.<br>(((((.<br>((((((<br>..((((<br>(((.((<br>(((((.<br>....((<br>(.....<br>......<br>)))...<br>))))))<br>).))))<br>)))..)<br>))))).<br>))))).<br>.)))..<br>))))))	0	1.53	1.92	0	2.12	0

					..)))) ...						
gga-miR-1786_R-2	ATTCTT TTCTGC TGTGTT A	19	3	gp1a	.... ((((.. ..((((..(((. ...))) .)))). .)))). .	0	0	0	0.60	0	0
gga-mir-1787-p3	CTTCCA TGGTGC GGTGGT C	19	12	gp1a	....(((((((((.(((((....((..((. .((... ....)) ..)).))....))))).)))))))))...	0	0	0	0	0	3.92
gga-miR-1788-3p_R+1	CAGGCA GCGAAA GCAAGT	22	7	gp1a	(..(((((((((((((((	0	0	0.64	1.19	2.12	1.96

	CTGT				(.((((<br>((((.(<br>((....<br>......<br>.)))))<br>))))))<br>))))))<br>))))))<br>))))..<br>)						
gga-miR-1790_L+1	GCGGGT<br>GAGGGC<br>GGTGGG<br>GGA	21	4	gp1a	((((.(<br>(((((.<br>(((.((<br>((((((<br>((((((<br>(....)<br>))).((<br>(((...<br>))))).<br>..)).)<br>))))))<br>))..((<br>(.....<br>.)))..<br>.....)<br>)).)))	0	0.76	0.64	0	0	0

					)))..))))...						
gga-miR-1794_R-1	GCCAGAATGGACATGGGCAGCA	22	10	gp1a	...((((((((.(((.....((((..((((((........))))))).))))((.......)).))).)))))))).......	0	0.76	0	0	0	0
gga-miR-1795	GCAAGATGAAGAACAGGCCTGT	22	chr21	gp1a	(((((..(..((((((...(((((..((((((....))))))...)))))..........	0	0	0	1.19	0	0

					.))))))...)..)))))					
gga-miR-1796	GAACTGCTGAAGGAGGGCTGA	21	2	gp1a	...((((((((((.(((((((...(((((.((((...((((((...........)))))).)))).)))).))))))))))))))))))((((.....(((.....)))....)))).....	0	1.53	0.64	3.57	3.18	1.96
gga-miR-1798-3p_L-3R+1	TTTTCAGAAGTG	20	20	gp1a	...((((((((((	0	0	0	1.19	1.06	0

	TAGCGTTT				..(((...(((.((((((((....((((..((((((..((((((((.........................))))))))..))))))..))))))...))))))))))))......)))..)))))))))))...						
gga-mir-1801-p3_1ss20CT	CAGTGCGGCATGCTGGGGGTT	21	8	gp1a	((((((((((((((((((((.(((((...)))))..)	0	0	0	0	0	3.92

					)))))))))))))))))))....						
gga-miR-1803_R+2	ATTGACTTCAGCGGGGTTTGGTT	23	chr2	gp1a	(((...(((((.(((((((((.(((..((.((((((((((((....))))))))))))).)))..))).))))))))).)))))....)))	0	0	0	1.19	0	0
gga-mir-1804-p5	AGTTGGTTAACAGCTCTGTACT	22	4	gp1a	....(((((.....((((((((((...((((((((.......	0	0	0	0	0	1.96

					)))..)<br>)))).)<br>)).)))<br>)))...<br>))))).<br>..						
gga-miR-1808_L-2R+2	TGTTGG<br>GAATGA<br>ATACAT<br>ATTGT	23	5	gp1a	((.(((<br>((((..<br>.((.((<br>(((((.<br>....((<br>((((((<br>(.....<br>)))).)<br>))))..<br>..))))<br>))).))<br>...)))<br>))))))<br>.	0	0	3.85	7.15	3.18	11.77
gga-mir-1808-p3	CATATG<br>GTCAAT<br>TCCCAT<br>CACC	22	5	gp1a	((.(((<br>((((..<br>.((.((<br>(((((.<br>....((<br>((((((	0	0	0	1.19	1.06	0

					(.....)))).)))))....))))))).))...))))))))))).						
gga-mir-1813-1-p5	TTCTCATTGCATGTTAACAGC	21	2	gp1a	...(((((((((((.(((((((((((.(((((.(..(.(((..((((.......))))...))))..)))))).)))))))))))....)).)))).))))...	0	0	0	0	2.12	1.96

gga-mir-3524b-p5_1ss1AT	TAGAATCACAGAATCACAGA	20	2	gp1a	...(((((((((((...((.........(((((((..(((.....)))...))))))))))).(((((...))))).....))...)))))))))))...	0	0	0	0	0.53	0
gga-mir-3524b-p3_1ss1AT_1	TAGAATCACAGAATCACA	18	Z	gp1a	...((.((...)).))...	0	0	0	0	0	0.98
gga-mir-3524b-p5_1ss19GC	ATTCTATGATTCTGTGATC	19	2	gp1a	.......(((((((.(((((.(((((....	0	0	0	0	0.53	0

					..)))))).(((....)))..))...))))))))))...						
gga-mir-3524a-p5_1ss11CG	CTATGATTCTGTGATTCTA	19	3	gp1a	...(((((....(((((.((((((.........))))))..))))))......))))...	0	0	0	0	1.06	0
gga-miR-3524a_1ss22GT	CAGAATCGCAGAATGGCTGAGT	22	chr1	gp1a	(..(((.((((.....(((.((..((.(((((.(((((((..((((((.((((.	0	0	0	0.60	0	0

					.........)))).))))))..))))))))).)).))..)).)))....))))..)))..						
gga-mir-3524b-p3_1ss19GC	ATTCTATGATTCTGTGATC	19	1	gp1a	...((((((((((.....((((((.........(((.(((....))).)))...(((..((((........))))..))).))))))))	0	0	0	0	0.53	0

					))))))<br>)...						
gga-mir-3524a-p5_1ss12AT	TTAAGGTCCCTTCCAACC	18	7	gp1a	...(((....((((..(((((((.(((.....(((.........)))...)))))))))).)))))....)))...	0	0.76	0	0	0.53	0.98
gga-mir-3524b-p3_1ss1AT_2	TAGAATCACAGAATCACAGA	20	3	gp1a	...((.((((((((.((....))..)))).))))))).....	0	0	0	0	0.53	0
gga-mir-3524b-p3_1ss11AG	AAGTCATTCTGTGATTCTGTGA	22	2	gp1a	...((([...((((.((.((((...	0	0	0	0	1.06	0

					((.....))))))))).))))..)))...						
gga-mir-3524a-p3_1ss12AT	TTAAGGTCCCTTCCAACC	18	5	gp1a	((((..((((.((.....(((((((.(((((.((((((....))))))))))......((((.......))))..))))))))......)).))))...))))...	0	0.76	0	0	0.53	0.98
gga-mir-3524a-p3_1ss11CG	CTATGATTCTGTGATTCT	19	2	gp1a	...((((((...((((..	0	0	0	0	1.06	0

	A				..)))) ...))))))... .						
gga-mir-3524a-p3_1ss16AT	ATCTTTAAGGTCCCTTCCA	19	27	gp1a	...((((((((.......((..(((....)))..)).. ..)))))))...	0	0	0	0	0.53	0
gga-miR-3525_L-1R+1_1ss12CT	AGCCATTCTGTGATTCTGTGAT	22	1	gp1a	...........((((......))))...	0	0	0	1.19	0	0
gga-mir-3525-p5_1ss14CT	TCATAGAATGGTCTGGGTTG	20	1	gp1a	...........((((...(..(.......)..)..)))))...	0	0	0.64	0	0	0
gga-mir-3525-p5_1ss8CT	CATTCTGTGATT	20	9	gp1a	...((((((((.	0	0	0.64	1.19	0	0

	CTGTGA<br>TA				.((((.<br>...(((<br>.....)<br>))...(<br>((((((<br>......<br>.)))))<br>))....<br>..))))<br>.)))))<br>)))...						
gga-miR-3529_1ss1AT	TGGCAG<br>ACTGTG<br>ACTTGT<br>TGT	21	Z	gp1a	...(((<br>.(((((<br>((((((<br>((((((<br>...(((<br>(.....<br>.)))).<br>))))))<br>)).)))<br>).))))<br>).))).<br>..	0	0	0	0.60	0	0
gga-miR-3530-3p_L+1<br>R+1	ACAATG<br>GTGTGA<br>GCTGGG	23	7	gp1a	....((<br>(((.((<br>((.(((	0	0.76	0.64	0	1.06	0

	ATGGT				(((((...((((.((..((((...)))).. .)).))))....)))))))))))))))).....						
gga-miR-3531-3p	CCTTGCAACACAAACAGGAGACA	23	23	gp1a	...(((((((((((((.(((((.(((((.((((((.(((..(......)..))).))))))).))))))))))...))))))))))))	0	0	0.64	0	0	0

gga-mir-3533-p5_1ss2CT	CTTTCAACTCCATCATGA	18	11	gp1a	......((((((((((((...((.(((......)))..)).....))))))..)))))))....	0	0	0	0	0.53	0
gga-mir-3534-p3	CCGAGCTCTGCTGCCCCACATC	22	JH375605.1	gp1a	((((.....((((..((((..(((.........)))..)))..))))).(((...)))..))))....	0	0	0.64	0	0	0
gga-mir-3535-p3_2	TAGATAGTCAAGTGCTGA	20	9	gp1a	...(((((((((.......(	0	0	0	0	0	0.98

	TC				((((((((((....((((...(((.....)))...)))....))))))))))).))....))))))))...						
gga-mir-3535-p3_3	TCTGAAATGCTGATGAAAC	19	1	gp1a	...((..(((((((...)...))))))))..)).........	0	0	0	0	0	0.98
gga-miR-3535_L+2R-8	CTGGATATGATGACTGATT	19	2	gp1a	..(((((((........))))))).............	0	0.38	0.32	0.60	0.53	0

gga-mir-3535-p3_1	ATTATCTGAAATGCTGATG	19	1	gp1a	...((.((....((((.......(((((...((.((....)).))..)))))......)))))).))....	0	0	0	0	0	0.98
gga-mir-3535-p3_4	TTATCTGAAATGCTGATGAAA	21	3	gp1a	...((((((.....(((((((((((....(..((.....((((.....))))......))..)....)))).))))))).....	0	0	0	0	0	0.98

					))))))...						
gga-mir-3540-p3_1ss22GA	TGCCCGCGAGGTAAGGGCAGGACCT	25	10	gp1a	......(((((((.(((..((((((((((.........))))))).)))..))).))))))...	0	0.76	0	1.19	0	0
gga-miR-6542-3p_L-1R-2_1ss4GT	CGTGACAGTGCTGAAGAC	18	chr3	gp1a	((((......))))((((.((((.......(((((.((((((((..((((((...(((..(..(........)..).	0	0	0	0	1.06	0

					...)))...))))).))))).))))).)))))....))))).....))))						
gga-mir-6543-p3	CATTCCCTTTTGTCCTGTT	19	2	gp1a	...(((((..........(((..(((.........)))..)))...((((((((......)))))..))......)))))....	0	0.38	0	0	0	0
gga-miR-6543-5p_R-1_1ss20TC	GCTGTTACCTGA	23	AADN03022228	gp1a	......((((..	0	0	0	5.95	4.24	1.96

	GAGAAGACGCT		.1		(..((((((((.((.(((((((((..((.((((..((.....))...))))).))))))))))).)).)))).)))..).)))).............						
gga-miR-6544-3p_L-1R-2	AGTTGTATTTCTTTTCTGACA	21	15	gp1a	....((((..((...........))..))))...........	0	0	0	0.60	0	0
gga-miR-6544-3p_L-1	AGTTGTATTTCTTTTCTG	23	5	gp1a	..((((((((((((..(	0	2.29	1.28	1.79	5.30	5.89

	ACAGT				.(((((<br>((....<br>..)).)<br>)))).)<br>..)).)<br>))))))<br>))))..<br>..						
gga-miR-6546-3p_L+1	GGGAGT<br>TCAGCT<br>GTGCTG<br>CTGC	22	7	gp1a	......<br>(((..(<br>(((...<br>.)))).<br>))).((<br>(((.((<br>((((((<br>(.((((<br>(..(((<br>((((((<br>((.(((<br>....))<br>))))))<br>))))))<br>..))))<br>).))))<br>))))).<br>.)))))	0	0.76	1.92	0	0	0

					...						
gga-mir-6547-p5_1ss7TC	ACGGGGCGACGGGGAGCGCGGTGTCC	26	27	gp1a	..(((((((..((((...........(((....)))))))..))))))).((((((((((.....)).........)))))))).(((((.((.......)).)))))	0	0.76	0	1.19	0	1.96
gga-miR-6549-3p	ACAGCTGTAAGCAGAAGGCACCC	23	8	gp1a	...((((((((...(((.((..((((((.(((((((((.(((	0	0	0	0	0	1.96

					(.(((.((((...(((((...))))).))).))).))))))))))))))...))))))).)).)))).........)))))))...						
gga-miR-6550-5p_L+3R-1	TTCAAAGCAGCCCAGGAAGCGC	22	10	gp1a	..((((((((((((((((..(..(.(((....((.((((.((((((((((((((((.(((....(	0	0	0	1.19	0	0

gga-miR-6552-3p_R+1	CAACAA<br>GAAGTG<br>GACAGA<br>CTGT	22	5	gp1a	(..(..<br>......<br>)..)).<br>....))<br>).))))<br>))))))<br>))))))<br>).))))<br>.)))))<br>)..)..<br>)))).)<br>))))))<br>))))).<br>..<br>......<br>.((((.<br>.(((..<br>.(((((<br>(...))<br>))))..<br>((((((<br>(.....<br>......<br>.....)<br>))))))<br>....))	0	0	1.92	0	0	0

					)..)))<br>)...						
gga-miR-6553-3p_L+1R-3	AAACACTACCCTGAGCTGC	19	13	gp1a	...........(((((((((.......)))))).)))...	0	0	0.32	0.60	0	0
gga-miR-6556-5p	AAGGCTGCTCGGCCCGCGCTGC	22	chr9	gp1a	((((.((.((((.....((((((((..((((((((((.(((((((((.....(((.....)))))))).))).)))))))))))).)))))))))))).))))	0	0	0	0	1.06	0

					))..						
gga-miR-6557-5p_L+1	CCCGGAGGAGCCCGGCGCTGC	21	2	gp1a	.(((...((((((((((((((((.((((((.(((.........)))....))))))...).))))))))))))))))))	0	0	0	0	1.06	0
gga-miR-6557-3p_1ss25CT	CGCGCCGATTGTCTCCTCCGGGCAT	25	2	gp1a	.(((...((((((((((((((((.((((((.(((.........)))....))))))...).)))))	0	0	0.64	2.38	0	0

					))))))<br>))))))<br>)						
gga-miR-6559-5p	TGGGAG<br>AAAGAG<br>TCATTA<br>CCTGA	23	1	gp1a	..((((<br>...(((<br>((((..<br>((((((<br>..(((.<br>......<br>)))..)<br>))).))<br>))))))<br>))))).<br>..((((<br>((....<br>....))<br>))))	0	0	0	2.38	0	0
gga-miR-6559-3p	AGAGTT<br>GACTCT<br>TCCTTG<br>CAGT	22	1	gp1a	..((((<br>...(((<br>((((..<br>((((((<br>..(((.<br>......<br>)))..)<br>))).))<br>))))))	0	0.76	0.64	1.19	0	0

					))))).<br>..((((<br>((....<br>....))<br>))))						
gga-miR-6560-3p_R-3<br>_1ss21CT	TACCAG<br>GATGGA<br>CCGCTG<br>CAT	21	chr6	gp1a	......<br>....((<br>(((..(<br>(((.((<br>.((((.<br>.(((((<br>((.((.<br>((((.(<br>((..((<br>......<br>)).)))<br>.)))))<br>))))))<br>..))..<br>)))).)<br>).))))<br>.)))))<br>......<br>..	0	0	0	1.19	0	0
gga-miR-6561-3p_L-2<br>R-1_1ss18AC	GAGCCC<br>AGAACT	19	3	gp1a	......<br>...(((	0	0	0	0	0.53	0

	GGACACA				((...((((......)))).....)))))...						
gga-miR-6565-5p_L+1	AATGAGATAGCAAAGCACACTTC	23	Z	gp1a	...((((.....(((....(((((...((.(((((((..(((((......)))))..))))))))...))))))))......))))...........	0	0	0	0	1.06	0
gga-miR-6565-3p_R+1	TCTGTGCTTGTGTACTCA	22	Z	gp1a	...((((.....(((...	0	0	0.64	1.19	3.18	0

	TAGT				.(((((...((. (((((((..(((((.... ..)))))..))))))))) ...)))))))). ....))))....						
gga-miR-6566-5p_L+1	TGAGGC CCGATG TGTCAT TCCT	22	AADN03 010670 .1	gp1a	..((.. ....((((((.(.((((((((.(((((.(((.(((. ...((((((... ....))))))..)))	0	0	1.28	2.38	2.12	0

					))).))<br>))).))<br>))))))<br>))))))<br>)....)<br>).						
gga-miR-6568-5p_R+1	GCGGCC<br>GCCATC<br>TTGCTT<br>GCGG	22	3	gp1a	.(((((<br>((.(((<br>((.(((<br>.(((((<br>(((((.<br>(((.((<br>......<br>..)).)<br>)).)))<br>))))))<br>).))))<br>).))).<br>))))).<br>))	0	0.76	0	1.19	1.06	0
gga-miR-6568-3p	ACAACC<br>AAGATG<br>GCGCCC<br>GGGC	22	3	gp1a	.(((((<br>((.(((<br>((.(((<br>.(((((<br>(((((.<br>(((.((	0	2.29	2.57	10.72	10.61	3.92

					........)).))).))))))))).)))).))).))))).))						
gga-miR-6575-5p	TTGTCAGCTTGGGGAAGCTCTT	22	10	gp1a	...(((((.((((((...((((((((((((((((((....((.((....)).))))))))))))))))))))..)))))).)))))...	0	0.76	0.64	0	2.12	5.89
gga-miR-6579-5p	CAACGCCGTGCA	21	chr5	gp1a	...(((..((.(	0	0	0	0	0	1.96

	ACGCACCTG				(((..(((((((..((((.(((.((.((..(((........)))..)).)).)))).))..)))))))))))).))..))).((((......))))....						
gga-miR-6582-3p_L-2R+1	CTCTGGGTGTTTCTCTTGCAGT	22	23	gp1a	...(((.((.(((.((((...((...(((....)))...)).)))).))).))	0	0.38	0	0	0	0

					......))))...						
gga-mir-6585-p5_1	TGTGAGCTACCTGTTTAT	18	2	gp1a	...((((((.....))))))...	0	0	0	0	0.53	0.98
gga-mir-6585-p3_1	TGTGAGCTACCTGTTTATT	19	2	gp1a	...((((((.....))))))...	0	0	0	0	0.53	0.98
gga-mir-6585-p5_2	TGTGAGCTACCTGTTTATT	19	19	gp1a	.(((((((.(((...((.(((.(((...((((((((((..((((((((((((((((..(((.((((((((.....))))))))..)))...))	0	0	0	0	0.53	0.98

					)....)))))))).....)))))))..))))))......)))).))).))).))...))).)))).)))						
gga-mir-6585-p3_2	TTTTGCTGTAGTGAGGAGCTTATTTT	26	19	gp1a	.(((((((.(((...((.(((.(((...((((((((((..((((((((((((((((((..(((.((((((.....))))))..))	0	0	0	0	0	1.96

					)...))<br>)....)<br>))))))<br>.....)<br>))))))<br>..))))<br>))....<br>..))))<br>.))).)<br>)).)).<br>..))).<br>)))).)<br>))						
gga-miR-6586-5p	TGCTGC<br>CAGATA<br>GAAGTT<br>CACCT	23	4	gp1a	...(((<br>(((((.<br>.(((((<br>.((.((<br>(((.((<br>(((...<br>......<br>....))<br>))))))<br>)).)).<br>))))).<br>..))))<br>))))..	0	0.38	1.28	1.19	3.18	0.98

					.						
gga-miR-6587-3p_L-1R+3_1ss2GT	TCTGGAAGTGGTGCTGGAGGCTGGA	25	5	gp1a	...((((.((((((((((....(((((.(((((((...)))))))))))..((....))......)))))))))))))))......	0	0.76	0	0	0	0
gga-mir-6592-p3_1ss1GC	CGCCGTCAGCCTGGAGCA	18	3	gp1a	...((((..(((((....(((((((.((........)).).))))))))))).))))...	0	0	0	0	0	1.96
gga-miR-6594-5p_R+1	TGGGGG	22	4	gp1a	..((((	0	2.29	1.28	2.38	3.18	3.92

	TGGCGT				(((.((						
	GAGTTG				(...((						
	TGGC				(....(						
					(((...						
					..((..						
					((((((						
					((((((						
					((((.(						
					..((((						
					(.....						
					.)))))						
					..).))						
					))))))						
					))))))						
					))))..						
					))))..						
					))))))						
					.)))))						
					))..						
gga-miR-6599-5p_R-1	AAGGGA	23	18	gp1a	...(((	0	0	0	1.19	0	0
	GGCTCC				(..(((						
	GGTCCG				((((((						
	CTCTG				(((.((						
					((((.(						
					(..(((						
					(((((.						

					....))						
					).(((.						
					..))).						
					.)))))						
					..))))						
					)))).)						
					))...)						
					))))))						
					))...)						
					)))...						
gga-miR-6599-3p	TGACGG	22	18	gp1a	...(((	0	0	0	4.76	1.06	1.96
	ATCCTG				(..(((						
	GCTCCC				((((((						
	TCCG				(((.((						
					((((.(						
					(..(((						
					(((((.						
					....))						
					).(((.						
					..))).						
					.)))))						
					..))))						
					)))).)						
					))...)						
					))))))						
					))...)						

					)))...						
gga-miR-6603-3p_L-1R-1_1ss10AG	AGGACAAGGGGAAATGGTTTC	21	2	gp1a	...((((...(((....((((......)))) ((((...(((((.....((((((((........))).))))).......)))))).)))).)))......))))....	0	0	0	0	1.06	0
gga-mir-6605-p3_1ss6AC	TGAGACTTTCTGCATTCC	18	3	gp1a	...((...((((((.((((((..(((((.((((((...((..	0	0	0	0	1.06	0

					..))...))))))))))))..))))))))))))...))...)						
gga-miR-6606-5p	GAGGAGCGGGAGGAGCGGGA	20	AADN03014199.1	gp1a	...((((.(((((.......)))))....))))...	0	0	0	18.46	2.12	15.70
gga-miR-6606-5p_L-1R-1	AGGAGCGGGAGGAGCGGG	18	JH376151.1	gp1a	...((((.(((((.......)))))....))))...	0	0	0	1.79	0	0.98
gga-mir-6606-p3_1ss2GA	CAGGAGGAGCGGGAGGAG	18	1	gp1a	...((((((((((((((.((((...(((.....((....	0	0	0	3.57	0	0.98

					))....)))...))))..)).)).)))))))))))).))....\..						
gga-mir-6606-p5_1ss16GA	GCGGGAGGAGCGGGAAGAG	19	7	gp1a	((.((((((.....(((.....((((((((.........))))))))...................(((((.((.......)).))))))))....)))))))).	0	0	0	0.60	0	0

gga-miR-6606-5p_R+1	GAGGAGCGGGAGGAGCGGGAA	21	chr1	gp1a	.....((((((.....(((.((((....)).))))).((..(..((((((...))))))..).))....))))))....(((((...((((....))))...)))))...	0	0	0	11.91	1.06	9.81
gga-miR-6606-5p_1ss20AT	GAGGAGCGGGAGGAGCGGGT	20	10	gp1a	...((((.((.(((((((((.(((.(((..((....))..)))	0	0	0	3.57	0	0

					.))))))).)))))..)).))))...						
gga-mir-6608-2-p5_1ss24GT	TGTTCTTCCTCACCAGTGCCTCATT	25	1	gp1a	...(((..(((((((..((((.((.(((((((((..((((((.((((.........))))))))))...))))))))).)))..))))))..)))...	0	0	0	0.60	0	0
gga-miR-6609-3p_R+1	ACAGCCCCTGTGCTCTGACCGCAT	24	1	gp1a	...(((.(((.....(((((.....	0	0	0	0	1.06	1.96

gga-mir-6614-p3	AAGGCG<br>GCTGCG<br>GGTTTC<br>TGT	21	4	gp1a	.(((((<br>(.(..(<br>((...(<br>((((((<br>(((.((<br>.(((((<br>((((..<br>....))<br>)))..)<br>)))..)<br>).))))<br>.)))))<br>)...))<br>)..).)<br>))))).<br>....))<br>))))).<br>)))...<br>...((.<br>.((..(<br>((((..<br>....((<br>(.((((<br>((((..<br>((..((<br>......	0	0	0.64	0	0	1.96

gga-miR-6615-3p_L-4<br>_1ss12TA	ACTGAT<br>GAGTTC<br>TCCACA	18	chr28	gp1a	))...)<br>)..)))<br>))))))<br>))....<br>.)))))<br>..))..<br>))....<br>......<br>....<br>.(((..<br>...)))<br>......<br>(((..(<br>((((((<br>((((((<br>.(((((<br>(....(<br>((((((<br>..((..<br>...)).<br>))))))<br>).))))<br>))))))<br>))...)<br>))))))<br>..))).	0	0	0	0	0	1.96

gga-mir-6616-p5	TTCTGCTGGCGGGGAAGGA	19	1	gp1a	.............(((((..(((.((((((((((......)))......))))))).)).)..))))...	0	0	0	0	1.06	0
gga-miR-6620-5p_L+2R-4	GCAGAGATGGAAGCGCTGCGC	21	chr25	gp1a	...((.(((..((((((.....(((((....((.((.((((((((((((.......(((......)))..)))))))))))))).)))))	0	0	0	1.19	0	0

					)))))).))).))............						
gga-miR-6621-5p_L+1R+1	CACAGGGATTACAGGGCTCTGAT	23	1	gp1a	...((((((..(((((((.(((((....((((...))))..)))))).)).))))))..)))))))....	0	0	1.28	1.19	0	3.92
gga-mir-6621-p3	TTCAGTGTCCCTGTTCTCCTTGC	23	1	gp1a	...((((((..(((((((.(((((....((((...))))..)))))).)).)))))	0	0	0	2.38	3.18	0

					)..))))))....						
gga-mir-6630-p3	AGGAAACAGCCTCCTGCAC	19	2	gp1a	...((((......))))........((((.((....)).)))...(((((((((......)))))))))))	0	0	0	0	0	1.96
gga-mir-6635-3-p5_1ss17CT	GTGCTGTGCTGTTTGTTT	18	20	gp1a	...(((.....(((((((......((..(((((.((((((((((((.....)))))....	0	0	0	0	1.06	0

gga-mir-6635-3-p5_1<br>ss1TA	AGCTGT<br>GCTGTT<br>TGTCTT	18	2	gp1a	(((((.<br>......<br>....))<br>)))...<br>))))))<br>).))))<br>)..)))<br>))))))<br>....))<br>)...<br>...(((<br>(.(((.<br>.(((((<br>((((..<br>((((((<br>((((((<br>.((((.<br>..))))<br>......<br>.)))))<br>)..)))<br>)))..)<br>)).)))<br>)))..)<br>))..))<br>))...	0	0	0	0.60	0	0

gga-mir-6635-3-p3_1ss1TA	AGCTGTGCTGTTTGTCTT	18	3	gp1a	...((.(((((((.((((..((((...(((((((....)))))))..((((((.((((((((.....))))))).)))))))..)))).))))...))))))))).))...	0	0	0	0.60	0	0
gga-mir-6640-p5_1ss13AT	GAATTGTAGTAGTGTGTATT	20	3	gp1a	(((..((((((((((....((((((.(((....)))))))))))))))))	0	0	0	0	1.06	0

					)).)))<br>.((((.<br>...)))<br>).(.((<br>(.((..<br>....))<br>..))).<br>).						
gga-mir-6640-p3	GAAGGA<br>CGACAC<br>GACGGA<br>C	19	3	gp1a	(((..(<br>((((((<br>(((...<br>.(((((<br>.(((..<br>..))))<br>))))))<br>))))))<br>)).)))<br>.((((.<br>...)))<br>).(.((<br>(.((..<br>....))<br>..))).<br>).	0	0	0	0	3.18	0
gga-miR-6641-5p	ACAGCT<br>TTTTTA	22	1	gp1a	..((.(<br>..((((	0	0	0	0	1.06	0

	TTTCCA<br>GTTA				.((...<br>((((((<br>(((.((<br>((((((<br>....((<br>(((...<br>))))).<br>.)))))<br>))))))<br>))))))<br>...)).<br>))))..<br>))).						
gga-mir-6643-p3_1ss<br>21CT	CACCCC<br>TGCCAG<br>CCTTGG<br>GAT	21	14	gp1a	...(((<br>(((((.<br>..((((<br>((((((<br>((((((<br>(..(((<br>......<br>.....)<br>)).)))<br>))))))<br>))))))<br>))..))<br>.)))))	0	0	1.28	2.38	0	1.96

					)...						
gga-mir-6645-p3_1ss22GA	ACCCCCACTGCTCTCTCCCAGA	22	5	gp1a	...(((((.....((((((..((((((((.(((((...(((.((((....)))).)))....)))))).)))))))))..)))))))))))))..	0	0.76	0	0	0	0
gga-mir-6655-p5_1ss3CT	TGTCCAGGTGGTTCTGCT	18	4	gp1a	(..((((((......))))))..)	0	0	0	0	0.53	0
gga-miR-6656-5p	TGCAGGGCGAGTAGAGAAAGGCAT	24	1	gp1a	.....((((((..(((((((.((((...(	0	0.76	0	0	1.06	0

gga-mir-6659-p5	TTATAG<br>TTAACC<br>TTTAGC	18	1	gp1a	((((.(<br>((.(((<br>(.....<br>......<br>.)))).<br>))).))<br>)))..)<br>))))))<br>))))).<br>.)))))<br>)...<br>.(((((<br>(....(<br>(((((.<br>.((((.<br>.(((..<br>..((((<br>((((((<br>((((((<br>...(((<br>(((..(<br>(((((.<br>.((...<br>.....)<br>)..)))<br>)))..)	0	0	0.64	0	0	0

					))))).						
					..))))						
					))))))						
					))))))						
					.....)						
					))..))						
					))..))						
					)))).)						
					))))).						
					..						
gga-miR-6659-3p_L+1	CTTTGG	23	1	gp1a	.(((((	0	0	0	0	0	3.92
	CTGCCA				(....(						
	TCGGAC				(((((.						
	CTGGT				.((((.						
					.(((..						
					..((((						
					((((((						
					((((((						
					...(((						
					(((..(						
					(((((.						
					.((...						
					.....)						
					)..)))						
					)))..)						
					))))).						

					..))))))))))))))))).....)))..))))..)))))).)))))))...						
gga-miR-6660-3p	TCTGACATGGCTCTGCTCCGCAGT	24	10	gp1a	...((((((((((((..(((((((((((....))))))).)))..)).))))))))))...	0	3.05	0.64	2.38	2.12	3.92
gga-miR-6662-3p_1ss22CT	AATGGTTGACCAGTGGCTGAAT	22	25	gp1a	...((.(..((((.((((..((((.((((.((........))	0	1.53	0	0	0	0

					))))．)<br>)))..)<br>))).))<br>))..).<br>))...(<br>(((..(<br>((...)<br>))..))<br>)).....<br>..						
gga-miR-6663-5p_R+1	ATTGCT<br>GTGTGA<br>GGAGTG<br>CTGT	22	27	gp1a	...(((<br>(((((.<br>((((((<br>((...(<br>(((.((<br>((((..<br>......<br>)).)))<br>)..)))<br>)....)<br>))))))<br>))))))<br>)))...	0	1.91	0.32	1.19	2.65	0.98
gga-mir-6663-p3_1ss22AT	GCTTCT<br>CCACCC<br>CACAGC	22	27	gp1a	...(((<br>(((((.<br>((((((	0	0.76	0	0.60	0	0.98

	AAGT				((...(((((.((((((.. ......)).))))..))))....))))))))))))))))...						
gga-miR-6665-5p	TGCCTG AAGGAT TTTGAT GCCC	22	8	gp1a	...(((((..(((((((((((((. ((((((..((((((((.. ....))))).))) ..)))))).)))))..)))))))) ..))))	0	0	0.64	1.19	0	0

					))....						
gga-miR-6666-3p	TCGGGTGATGAGGACAGGAGGA	22	28	gp1a	...((((((..(((((.(..((..((((((.....))))..))..))..)))))))..)))))))...	0	0	0	0	1.06	0
gga-mir-6666-p3_1ss8GT	AATTTAGTGGACTCCTGCAC	20	2	gp1a	...............(((((.....((......))..)))))....	0	0	0	1.19	0	0
gga-miR-6670-5p_L-1	TGGAATGATGATCTATATTCTGAGT	25	3	gp1a	...((.((((((((.....(((((((((((((((((	0	0	0.64	0	1.06	0

					((.(((((.........)))))..)))))))).))).)))))))).)).))))).))....						
gga-mir-6671-p3_1ss7AG	TGCAGGGACTGCTGCAAG	18	AADN03014490.1	gp1a	...(((((..((.(((..(((((.((((...(((((....))))))..))))).)))))...)))))))))))...	0	0	0	1.19	0	0
gga-mir-6673-p3	GGCACCTGAGAA	19	15	gp1a	..((((((..((	0	0.76	0	0	1.06	0

	TGAGAT<br>G				..((((<br>((...(<br>((((((<br>(..(((<br>((((((<br>(((...<br>.(((((<br>((((((<br>((..((<br>......<br>.))..)<br>)).)))<br>))))))<br>)....)<br>))))))<br>))))).<br>.)))..<br>))))).<br>..))))<br>))....<br>.))...<br>))))))<br>...						
gga-miR-6674-3p_L-1<br>R+3	AGGTGT<br>CTGTGG<br>CAGCCT	23	chr20	gp1a	(((...<br>(((...<br>.))).. 	0	0	0.64	0	0	0

	CACCC				))).((<br>(.(((.<br>(((.((<br>((((..<br>(((.((<br>(...((<br>((....<br>......<br>.)))).<br>..))).<br>)))..)<br>))))).<br>))))))<br>...)))<br>......<br>..						
gga-miR-6675-5p	ATACGT<br>GAGGTT<br>CATTGG<br>GTTC	22	24	gp1a	...(((<br>((..((<br>((((((<br>(.((((<br>(..(((<br>((((((<br>......<br>..))))<br>)..)).<br>))..))	0	0	0	1.19	0	0

					))).))))))))..)))))...						
gga-miR-6675-3p	TCTCTGGACGTCACGTGTGAGG	22	24	gp1a	...(((((..((((((((((.((((((..((((((((((........)))))..)).))..))))).))))))))..)))))...	0	0	1.28	2.38	4.24	0
gga-miR-6677-5p_L+1R-4	TCTCACTGCCTGCCCTGAGCAC	22	20	gp1a	...((((((..(((((((.((((.((..((((((..((((........	0	0	0	0	1.06	0

					))))))<br>)))).)<br>).).))<br>))))))<br>))..))<br>.)))).<br>..						
gga-miR-6677-3p_R-1	TGCTCA<br>GACACG<br>TCAGGA<br>GG	20	20	gp1a	...(((<br>(((..(<br>((((((<br>.((((.<br>((..((<br>((((..<br>((((..<br>......<br>))))))<br>)))).)<br>).).))<br>))))))<br>))..))<br>.)))).<br>..	0	0	0	1.19	0	0
gga-miR-6680-3p	GTTGCT<br>GATGAA<br>GATACT<br>GAAT	22	11	gp1a	......<br>.....(<br>(((.(.<br>.(.(((	0	0	0	0	1.06	1.96

					(.....<br>....))<br>)))..)<br>))))..<br>.						
gga-miR-6685-5p_L+1R+1	CCAGCAATGCACGTGTACTCAGT	23	5	gp1a	.(((((<br>.(((((<br>((((.(<br>((((((<br>.(((.(<br>(.....<br>.)))))<br>)).)))<br>)).)))<br>))))))<br>))))).	0	0.76	1.28	0	0	0
gga-miR-6685-3p_L-1R+2	GGTGACGCGGGCATTGCTGCTT	22	5	gp1a	.(((((<br>.(((((<br>((((.(<br>((((((<br>.(((.(<br>(.....<br>.)))))<br>)).)))<br>)).)))<br>))))))	0	0.76	0	0	0	5.89

					))))).						
gga-mir-6687-p3	GCCGGATCGGGCGGGAGTCACGAG	24	3	gp1a	...(((...(((....))).((((((((.((((((.((..(((((((....))).)).)).))))))))...)))))))))	0	0	0.64	0	0	1.96
gga-mir-6691-1-p5_1ss2AC	GCGCTGCTGCTGCTGCTGA	19	6	gp1a	.......(((((..((((((((......(((((....((((((..((((....))))...))))).	0	0.38	0	0	0	0

					.)))))<br>..))))<br>))))..<br>))))).<br>.						
gga-mir-6691-1-p3_1ss2AC	GCGCTGCTGCTGCTGCTGA	19	17	gp1a	(..(((<br>((((((<br>(..(((<br>(....)<br>).))..<br>).))))<br>.)))))<br>..)	0	0.38	0	0	0	0
gga-miR-6694-3p	TTAAGAGTAGGGATTCTGTTCC	22	6	gp1a	...(((<br>(.((((<br>(((.((<br>..((((<br>.(((((<br>...((.<br>......<br>.....)<br>)..)))<br>))))))<br>..)).)<br>))))))<br>.)))).	0	0.76	0	0	2.12	1.96

gga-miR-6696-5p_R+1	AGCTGT<br>GATCTC<br>AAGCCC<br>CCACT	23	17	gp1a	.<br>..((.(<br>(((((.<br>..((..<br>((((((<br>((.(((<br>.((((.<br>.(((((<br>......<br>......<br>))))).<br>..))))<br>...)))<br>.)))))<br>)))..)<br>)..)))<br>).)).)<br>).	0	0	0	0	0	1.96
gga-miR-6696-3p_L-1<br>R+2	GGAGAC<br>TTGCCT<br>GATAAC<br>AGCTGC	24	17	gp1a	..((.(<br>(((((.<br>..((..<br>((((((<br>((.(((<br>.((((.<br>.(((((<br>......	0	0	0.64	1.19	0	1.96

					......)))))...))))...))).))))))))..))..))))).)).).).						
gga-mir-6697-p3_1ss1TG	GGAAGCTGATGTCCTTTG	18	6	gp1a	...(((((((........)))))))...	0	0	0	0	1.06	0
gga-miR-6698-3p	TTAGGAGAGAGTGAGAAGTAAGT	23	2	gp1a	..((....))..((((((((..((((((((...(((..(.((((((..................))))))..))).	0	0	0	0	2.12	1.96

					))))))).))..))))))))...						
gga-miR-6699-5p	TGAAGACCTCGTGCATTGTAGC	22	3	gp1a	..(((..((((.((.(((.((((((((.......))))))))).))).)).))))...)))...	0	0	0.64	1.19	0	0
gga-miR-6701-3p	ATTATTTTACAGACAGATCGC	21	2	gp1a	...((((..(((((((((.(((.((((((..((..............))..))))))).)).)))	0	2.29	0	1.19	3.18	0

					))))))<br>)..)))<br>)...						
gga-miR-6705-5p_R+1	AAGCAA<br>TGGGGT<br>AAGGAA<br>CAGT	22	4	gp1a	.((((.<br>(((((.<br>((((((<br>.((...<br>..((.(<br>((....<br>.)))))<br>.....)<br>).))))<br>)).(((<br>(((((.<br>..(...<br>...)..<br>.)))))<br>)))..)<br>))))))<br>))....<br>.	0	0	0.64	2.38	3.18	0
gga-mir-6705-p3	ATATGA<br>AGATCA<br>TTCTGG<br>G	19	2	gp1a	(((((.<br>.(((((<br>(.....<br>.....)<br>))))).	0	0	0	0.60	0	0

					..))))<br>).....<br>......<br>.						
gga-mir-6706-p3	GTGTCA<br>TGTTTC<br>ATGCTG<br>CAACA	23	5	gp1a	...(((<br>(((..(<br>(((.((<br>((.(((<br>......<br>))).))<br>))))))<br>..))))<br>))....<br>.....(<br>((..((<br>(.....<br>...)))<br>..))).<br>...	0	0	0	0	0	3.92
gga-miR-6708-5p_R+2	TATGGG<br>AGGAGC<br>GGTGAG<br>CAGCAG<br>C	25	1	gp1a	.(((((<br>.(((((<br>(((.((<br>((...(<br>(((.((<br>(.....<br>....))	0	0	0	0	2.12	0

					).))))<br>))))))<br>)...))<br>))))))<br>)).						
gga-mir-6710-p5	CGGTAG<br>CAGACC<br>TGAAGT	18	2	gp1a	.((.((<br>(...((<br>(...(.<br>.(((((<br>((...(<br>((((((<br>(....(<br>((((..<br>..))))<br>)...))<br>))))))<br>.)))))<br>))..).<br>....))<br>)..)))<br>))...	0	0	0	0	1.06	0
gga-miR-6710-3p_R+1	AAACTG<br>TTCTCT<br>TCCATC<br>TAGT	22	2	gp1a	.((.((<br>(...((<br>(...(.<br>.(((((<br>((...(	0	0.38	1.28	4.76	4.24	4.91

					((((((<br>(....(<br>((((..<br>..))))<br>)...))<br>))))))<br>.)))))<br>))..).<br>....))<br>)..)))<br>))...						
gga-miR-6710-3p_L-1<br>R+1	AACTGT<br>TCTCTT<br>CCATCT<br>AGT	21	3	gp1a	...(((<br>((....<br>......<br>..((((<br>......<br>......<br>......<br>))))))<br>)))...	0	0.38	0	0.60	0	0
gga-miR-6710-3p	AAACTG<br>TTCTCT<br>TCCATC<br>TAG	21	Z	gp1a	.((((.<br>..((((<br>.(((((<br>((....<br>......<br>...)))	0	0	0	1.79	0	0.98

					))))..<br>))))..<br>.))))						
gga-miR-7438-5p	TTTTTC<br>AAGGGG<br>ACAACT<br>CAGA	22	Z	gp1a	...(((<br>(.(..(<br>((((((<br>((((((<br>..((..<br>....))<br>..)).)<br>))))))<br>))))..<br>).))))<br>.....	0	0	0	0	1.06	1.96
gga-mir-7439-p3_1ss<br>16CG	AGTTTG<br>CTTCTC<br>CTTGTC<br>C	19	Z	gp1a	..(((.<br>.(((((<br>...(((<br>..(((.<br>...(((<br>((((..<br>....))<br>))))).<br>.)))..<br>)))...<br>.)))))<br>..))).	0	0	0	1.19	0	1.96

					..						
gga-miR-7441-3p_L+1	CATGTTGGGATCCTCTTCTGCA	22	17	gp1a	...((((((((((...((((((((.............))))))))..)))))))))))....	0	0	0	0	1.06	0
gga-mir-7444-p5	CTCCTTGCACTCTCTGGGACCTGGA	25	19	gp1a	...(((((.(((.((..((((((((((((...(((((((((((...(((((((((((.....((.....))....)))).)))))	0	0	0	1.19	0	0

					)...))<br>))))))<br>))))..<br>))))))<br>))...)<br>))...)<br>).))).<br>))))).<br>..						
gga-miR-7445-3p_L+1	TGGATG<br>AGTCTG<br>TTTAAC<br>TTTAT	23	chr1	gp1a	((((((<br>((((((<br>((((((<br>((((..<br>((((..<br>..))))<br>))))))<br>))))))<br>))))))<br>))))..	0	0	0.64	0	0	0
gga-mir-7446-p3	GAATGG<br>CGGACG<br>TGTGAG<br>GAG	21	1	gp1a	.((.((<br>(.((.(<br>(((((.<br>((((((<br>(.((((<br>((((.(<br>(.(((.	0	0.76	0	1.19	1.06	0

					((...(<br>((((.(<br>((((..<br>.((.((<br>(((...<br>..))))<br>)))...<br>.)))))<br>.))).)<br>)))..)<br>)).)))<br>))))))<br>)....)<br>))))))<br>.)))))<br>).)).)<br>))))..<br>.						
gga-miR-7447-5p_R+1	AAAGTT<br>GGTAGT<br>GATTGC<br>TGATGT	24	1	gp1a	..((((<br>.(((..<br>((.(((<br>...(((<br>((...(<br>((((..<br>((((((<br>..((((	0	0	0	1.19	0	0

gga-miR-7447-3p_L+2R-2	CCTGAGCACTCAGCCCAACTCC	22	1	gp1a	((....<br>....((<br>(((.((<br>(....(<br>(.((..<br>...)).<br>))))))<br>))))))<br>)))).)<br>))).))<br>..))))<br>)...))<br>)))...<br>))))).<br>.))).)<br>)))...<br>..((((<br>.(((..<br>((.(((<br>...(((<br>((...(<br>((((..<br>((((((<br>..((((<br>((....<br>....((	0	0	0	0	0	1.96

gga-miR-7456-5p_L-1_1ss21AG	CTGCAGCTGCATTTTCCCTGTG	22	2	gp1a	(((.(((....((.((.....)).))))))))))))))).))))).))..)))))...))))...)))))).))).))))...)...(((((.(((((((((((((.((((((((.((((.(((..........))).)))))))))).)))))))	0	0	0.64	0	0	0

					))))))))).)))...						
gga-miR-7456-3p_R-1	CAGGGAGATGTACCTGTAGGA	21	2	gp1a	...(((((.((((((((((((((.((((((((.((((.(((..........))).))))))))))).))))))))))))))).)))...	0	0	3.21	0	5.30	3.92
gga-miR-7460-3p	CCTGACTGAGCTCTGCTTTCTC	22	13	gp1a	.((((.((..((((((.((.......(((.((((....))))...(((	0	0	1.28	2.38	3.18	1.96

					((((((((.....)))).....)))))))...)))......))))))))..))))))...						
gga-miR-7462-5p_R+1_1ss17TG	CAACCTCATTTCTAGCGTCTGGT	23	chr3	gp1a	((((((.(((((.(((((((..(.((.................)))..))))))).)))))).)))))))..	0	0	0.64	0	0	0
gga-miR-7462-3p_L+1R-1	TGGAAGCTTGAAATAAGGTTGA	22	3	gp1a	...((((((.((((((((((((((.(((((	0	3.05	0.64	0	0	0

					.((((((..(.((..........)))..)))))).)))).)))))))))))).)))))))...						
gga-mir-7464-p3_1ss8CA	CTGGTGAACAATGGCACTG	19	8	gp1a	...(((((.((((........))))))))).. .	0	0	0	0	1.06	0
gga-miR-7464-5p_L-2R-3_1ss15AC	ATGGCTCAGTGCCGTGCC	18	chr3	gp1a	((((((((((((((((((((((..(((...))))))))))))))))))))))))	0	0	0	1.19	0	0

					)..						
gga-miR-7465-3p	AGAGTG TCTGTT ACCTGA CC	20	3	gp1a	...(((((.((((.(((((((... ....))))))).)))).)))))...	0	0	0.64	0	1.06	0
gga-mir-7466-p5	GGCCAC TCTGGG AGGCTC CAGCAT T	25	4	gp1a	(((... ...(((((....))))). ..((((((....)))))))))	0	0	0	1.19	0	0
gga-miR-7466-3p_R-5	TTTTCC TGTAGA GGAAGC	18	4	gp1a	(((... ...(((((....))))). ..((((((....))))))	0	0	0	0	1.06	0

					)))						
gga-miR-7467-5p_L-1R-1	CCAGCGCGGTGGTGATGGTGC	21	4	gp1a	...((((.((.((.((.((((((.((((((..(((((...((.(((....))))).)))))..))))))).))))))).)))).))))).))...	0	0.76	0	0	0	0
gga-miR-7469-5p_R+1	TTTGCAATGCAATTTTACAGTAGA	24	4	gp1a	...((((...((((((.((((((.......(((((((((((((...(((((.(	0	0	0.64	1.19	2.12	0

					((((((<br>(....)<br>))))..<br>..))))<br>))))..<br>.)))))<br>))))))<br>))..))<br>))))..<br>.)))))<br>)..)))<br>)...						
gga-miR-7472-5p_1ss1AC	CTGTGCTGGAGATGAAGCTGGA	22	11	gp1a	......((((((.(((((.....))))))))))).. .	0	0	0.64	0	0	0
gga-mir-7472-p5_1ss13GT	GAGATGAAGCTGTAGCTC	18	1	gp1a	........((((((.((......)))))))...	0	0	0.64	0	0	0
gga-miR-7473-3p	ACTTAG	23	chr7	gp1a	.(((((	0	0	0.64	0	0	1.96

	GCACGTAAATCTCTCGT				(((((.(((.(((.((((((............)))))).))).))).)))))))))))...						
gga-mir-7475-p5_1ss18GC	CCGCCGCCGCGCCCTCCCC	19	Z	gp1a	...((((((.(((((((.............(((....))).))).)))).))))))...	0	2.29	0	0.60	2.65	0.98
gga-mir-7475-p5	GCCGGCAGCGCTGTGAGGA	19	1	gp1a	((((((((.((((((....)))).))))..	0	1.53	0	0	0	0

					.((..(((....)))..))...).))))))).............((......))..............						
gga-mir-7476-p5	CATCTGATGTGAAGCTGTGCAAGAAA	26	15	gp1a	...((((.((((((.((((((((((((..((((((((.(((.((((((.(((((....)....)))...)))))))..))))))))))))))))))).	0	0	0.64	0	1.06	3.92

					.)))))<br>).))))<br>)))))).<br>))))..<br>.						
gga-miR-7477-3p	TACCGC<br>CCCGTT<br>CTGCGC<br>TCGGA	23	JH3762<br>68.1	gp1a	..((((<br>((((.(<br>(((((.<br>((((((<br>.((...<br>...)).<br>)))).)<br>))))))<br>).))))<br>))))..<br>..	0	0	0	1.19	2.12	0
hsa-miR-3074-5p_L+1<br>R+1	TGTTCC<br>TGCTGA<br>ACTGAG<br>CCAGT	23	Z	gp1b	((((((<br>(.((((<br>.((.((<br>((((((<br>.((((.<br>.((((.<br>((....<br>..))..<br>))))..<br>.)))).	0	0.76	0	0	1.06	0

))))))
)).)).
))))..
))))))
)

Table S4. Summary of all expressed novel miRNAs in the livers of chicken.

miR_name	miR_seq	len	genome ID	group	Secondary Stracture	AC1 (norm)	AC2 (norm)	AC3 (norm)	YC1 (norm)	YC2 (norm)	YC3 (norm)
hsa-miR-143-3p_R+1	TGAGATGAAGCACTGTAGCTCT	22	13	gp2	...((((((..(((.((.(((((((((.((((((.((((......)).)).)))))).))))))))).)).)))..))))))...	132328.53	114940.16	150282.21	189276.41	160778.17	150890.61
aca-miR-191-5p_R-1	CAACGGAATCCCAAAAGCAGCT	22		gp3	((..((.((..((.((((((((((.((..(((((((.................)))))))..)))))))))))).)). .)).))..))..	57152.94	75715.60	64057.34	16422.00	14792.69	11220.11
aca-miR-425-5p_R+4	AATGACACGATCACTCCCGCTGAGC	25		gp3	...((((((((((((((..(((((((.((..(((((((.((.......))..))))...)))..))))))))))..))))))))))))))...	19106.97	23883.02	14462.09	6989.09	6583.19	5116.55
aca-miR-20a-5p_R+1	TAAAGTGCTTATAGTGCAGGTAG	23		gp3	...((((.(((.((((((((..((((((((.(((...........)))))))))))..)))))))..))).))))........	6349.50	6770.15	11474.95	8750.80	11094.51	8972.36
ssa-miR-20a-5p	TAAAGTGCTTATAGTGCAGGTAG	23		gp3	..(((((((((((.(((((.(((............)))))))).)))))))))))).....	6349.50	6770.15	11474.95	8750.80	11094.51	8972.36
aca-miR-148b-3p_1ss20TG	TCAGTGCATCACAGAACTTGGT	22		gp3	.......(((((.((((((((..(((((((((.((.((((..((((............)))))))).)).)))))))))..))))))).))).))..	5713.89	7325.13	6983.99	10558.96	15619.10	10349.85
ggo-miR-148b_R+1_1ss20TG	TCAGTGCATCACAGAACTTGGT	22		gp3	...........((..((..(((.((((((((..(((((((((.((.((((..((((............)))))))).)).))))))))))..)))))))).))).))..))	5713.89	7325.13	6983.99	10558.96	15619.10	10349.85
aca-miR-150-5p_1ss12TC	TCTCCCAACCCCTGTACCAGTG	22		gp3	...((((((((((.((((.(((.((..((((((((((...((....))...)))))))))))..)).))).))))).)))......))))).)))...	5605.22	6685.69	4064.19	1878.72	1912.74	1625.72
bta-miR-139	TCTACAGTGCA	2		gp	...((((((((((.((.(((((((((((((..........)))))))))))))).)))).))))))..	4019	3655	4693	3378	3133	3197

	CGTGTCTCCAGT	3		3		.49	.51	.61	.72	.79	.48
eca-miR-139-5p_R+	TCTACAGTGCA	2		gp		4019	3655	4693	3378	3133	3197
1	CGTGTCTCCAGT	3		3	((((((((((.((.(((((((((((((.((....))..)))))))))))))).)))).))))))	.49	.51	.61	.72	.79	.48
	TTCAAGTAATC										
hsa-miR-26a-5p_R+	CAGGATAGGCT	2		gp	((((((..(..((..(((((((((.((((((((((.............))))))))))).)))))))))..))..)..	3933	5443	8356	1126	1327	1071
3	TTT	5		3	))))))	.04	.42	.35	.04	.14	.38
	TTCAAGTAATC										
	CAGGATAGGCT	2		gp	.(.(((...(((((..((((((.(((((((((.((.((((((((.((.......))...))))))).)).)))))))	3933	5443	8356	1126	1327	1071
ola-miR-26_R+4	TTT	5		3	)).)))))))..)))))....))).).	.04	.42	.35	.04	.14	.38
oan-miR-15a-5p_R+	TAGCAGCACAT	2		gp	((((((((((((..........((((((((..((((..((((..........)))).)))))..))))))))........	3757	4359	5046	3042	3794	3382
2	AATGGTTTGTGT	3		3	..)))))...)).))))))	.67	.77	.97	.28	.71	.91
mmu-miR-6240_R-7	ACAAAGCATCG	1		gp	..((((...(((....((....(((.((.(((((.((....))....(((((..(((.((.....)))))..)))))..)	3426	416.	271.	715.	708.	2896
_1ss1CA	CGAAGGCC	9		3	)))).))..)))......))......)))...))))..	.69	38	27	76	66	.27
	TTCACAGTGGC	2		gp	((((.(((((.(..((((((..(.((((((((..((.....))......)))))))))..))))))..).)))))..	3364	2566	2753	2910	3234	3469
cgr-miR-27a-3p	TAAGTTCCGC	1		3	))))	.12	.77	.81	.09	.04	.57
	TTCACAGTGGC	2		gp		3364	2566	2753	2910	3234	3469
aca-miR-27a-3p	TAAGTTCCGC	1		3	(((.(..(..(((((((...(((((((((....(((......)))))))))))).))))))))..)..).))).	.12	.77	.81	.09	.04	.57
	TCCCTGAGACC										
	CTAACTTGTGA	2		gp	((((.............(((((.......)))))((((...........((..(((((..((..(((.(((.(((((((	2263	2658	2998	1510	1318	1007
sha-miR-125a_R+2	AA	4		3	(...............)))))))).))).)))..))..)))))..))((....)).))))...)))).	.33	.41	.36	.12	.12	.61
mmu-miR-215-5p_R	ATGACCTATGA	2		gp	...((((.(((..((((..(((((...((.((((((((.((((((...((..........)))))))).))).))))	2150	166.	212.	216.	533.	351.
+1_1ss12TA	ATTGACAGACT	2	3	2	).))...)))))..)))).))).))))...	.53	25	91	75	61	24
aca-miR-363-3p_R+	AATTGCACGGT	2		gp	.....((..(((.(((..(((((((((..((.((((((((((.................)))))))))))..)))))))	2108	3097	2316	1002	9279	9535
1	ATCCATCTGTA	2		3	))..))).)))..))....	.82	.80	.88	6.60	.55	.19
oan-miR-363-3p_R+	AATTGCACGGT	2		gp	((..(((.(((..(((((((((..((.(((((((((..((..........))..))))))))))))..))))))))).	2108	3097	2316	1002	9279	9535
1	ATCCATCTGTA	2		3	.))).)))..))	.82	.80	.88	6.60	.55	.19

hsa-miR-7641_L+1R+5_1ss2TC	TCTGATCTCGGAAGCTAAGCAGGGT	25		gp3	.((..((((..(..(((((.((((......)))).))))).)..))))..)).	2101.13	823.99	495.08	711.00	664.10	1081.20
oan-miR-19b-3p	TGTGCAAATCCATGCAAAACTGA	23		gp3	((((.((((..((((((((((((((((((((((((...(((((.........)))))))))).)).)))))))))))))))..)))...)))).))))	2044.87	3776.38	16085.41	4222.91	7711.60	7294.31
aca-miR-19b_R+2	TGTGCAAATCCATGCAAAACTGA	23		gp3	.((((((((((((((((((((..((((..(((...)))..)))))))))).)).)))))))))))))..	2044.87	3776.38	16085.41	4222.91	7711.60	7294.31
bbe-miR-100-5p_R+3	AACCCGTAGATCCGAACTTGTGTTT	25		gp3	((((...(((...((.((((.(((((((((..(((.((((((..((....)).)))))).)))..))))))))).))).))....)))...))))....	2008.92	2136.03	4412.09	974.00	1584.40	1096.24
dre-miR-92a-3p_R+2	TATTGCACTTGTCCCGGCCTGTAT	24		gp3	.....(((((....(((((..(((((..((.((((((((............))))))))))..))))).)))))))))).....	1643.36	2523.43	2680.92	1682.22	1682.53	1448.14
aca-miR-23a-3p_R+2	ATCACATTGCCAGGGATTTCCAT	23		gp3	.(((((......(..((((((((..((((((((((............))))))))))..))))))))..).......))))).((....)).	1529.74	2145.19	2710.10	1040.89	1240.15	1161.65
csa-let-7d_1ss10TC	TGAGGTAGTCGGTTGTATTGTT	22		gp3	..(((...((((((.(((((((((((((((((((.(((.....((...))....))).))))))))))))))).))))))))))))..)))...............	1248.44	1201.64	1018.37	1929.74	2307.73	1620.81
tgu-miR-2970-5p_1ss21GT	GACAGTCAGCAGTTGGTCTGT	21		gp3	((((((((((.(..((((.((...(((...(((..((((((((.(..(..(((((((..((.(((((((((((.....)))....)))))))).))..))))))..)..).))).)))))..))))))...)).))))..)))))))))))	1223.47	1541.21	835.60	1063.52	983.42	812.37
hsa-miR-143-5p	GGTGCAGTGCTGCATCTCTGGT	22	13	gp2	...((((((..(((.((.(((((((((.((((((.((((......)).)).)))))).))))))))).)).)))..))))))...	1118.08	742.01	1019.65	1940.06	2356.18	2266.39
hsa-miR-425-5p_R+2_1ss20TC	AATGACACGATCACTCCCGCTG	25		gp3	......(((((.....((((((((.(((((((((((..((...((((((((...(((((.......(((.........)))..)))))..))))))))..)).)))))).)))))..)))).....))))).......))))).	631.49	908.25	613.40	178.05	183.00	134.41

	AGT										
hsa-miR-7977_1ss6AG	TTCCCGGCCAACGCACCA	18		gp3	(((((((.(((((..(((.........)))...))))).)))).)))..	601.03	725.99	135.31	426.36	196.26	580.82
rno-miR-122-5p_L+1R+1_2	CTGGAGTGTGACAATGGTGTTTGT	24	JH375727.1	gp2	...(((((((((((..(((((((.(((((((((((...........))))))))))).)))))))..)))))).)))))...	582.92	585.29	541.57	312.62	363.88	361.05
rno-miR-122-5p_L+1R+1_1	CTGGAGTGTGACAATGGTGTTTGA	24		gp3	.((((((((((((....(((((((..(((((((((((((.....))).))))))))))..)))))))....)))).)))))))).	582.92	585.29	541.57	312.62	363.88	361.05
aca-miR-193-5p_R+1	TGGGTCTTTGCGGGCGAGATGA	22		gp3	.......(..((((((..((.(((((.(((((..(((.((.((((..((.....)).)))).)).)))..))))).)))).))..))))))..)...	550.81	544.11	376.97	491.66	352.21	355.17
aca-miR-18a-5p	TAAGGTGCATCTAGTGCAGATAG	23		gp3	....((..((((((((.((((((.(((..(((.((((.(((....((......))..))))))).)))..))).))).))).)))).))))))..)).	546.69	635.24	1060.70	3020.25	4751.61	3696.87
ipu-miR-7550_1ss1AT	TTCCGGCTCGAAGGACCA	18		gp3	.....(((..((((((((((((.......))))....)))))))).))).......	490.70	513.23	135.31	320.37	245.06	392.45
mmu-let-7j_1ss8TG	TGAGGTAGTAGTTTGTGCTGTTAT	24		gp3	....(((((.(((..((((.....(((((((...((((.((((((((.((....((((..........))))).....)).))))))))..))))))))))).....)))))))))))))....	450.36	394.26	459.49	145.89	143.22	63.77
aca-miR-301b-3p	CAGTGCAATAATATTGTCAAAGC	23		gp3	.((..((((((((..(..(((.(((((((((((((((.((.............)).))))))...)))))))))).)))..)..))))))))))	426.48	544.49	1195.37	1726.88	4033.40	2733.40
bbe-miR-375-3p_R+1	TTTGTTCGTTCGGCTCGCGTTATT	24		gp3	.......(((((((.((((((.(((((((..(((..((((((.(((.....)))....))))))..)))..))))))).))))))).)))))))........	421.54	407.23	175.07	147.68	181.41	174.64
ola-miR-126-3p_R+3	TCGTACCGTGAGTAATAATGCA	23		gp3	.........(((((..(((((((.((((((((((..(((((((..................)))))))..)))))))))).)))))))))))))..........	400.96	421.72	698.05	136.96	163.37	86.34

ssa-miR-19a-3p_R+1_1ss11TC	ATGTGCAAATCCATGCAAAACTGT	23		gp3	((((((((((((((((((..(((......)))....))))))..)))))))))))))..	368.58	780.14	3681.55	313.62	466.96	366.61
sha-miR-140_R+2	ACCACAGGGTAGAACCACGGAAA	23		gp3	.........(((((((((((....)))............(((((((.....((((((((((.(((((((((((((.((((((..(((......)))))))))).))))))))))))))).)))))))).)))))))).....))))))))	363.91	486.54	519.13	161.97	171.86	165.81
aca-miR-193-3p_R+2	AACTGGCCTACAAAGTCCCAGT	22		gp3	.......(..((((((..((.(((((.(((((..(((.((.((((..((.....)).)))).)).)))..))))).)))))).))..))))))..)...	306.28	336.69	390.33	340.61	627.68	574.28
efu-miR-9341_L-2R+1	TCGGCCTTTTGGCTAAGATC	20		gp3	.(((..((.(((((((.((((.........((((((..(((((((((((((((((((.....................))))))..)))))))).....))))))))))))))))))))).))))))..)))........	302.98	39.66	25.65	75.03	242.94	129.51
rno-miR-143-3p_R+1	TGAGATGAAGCACTGTAGCTCAT	23		gp3	(((((((..((.((((((..((((((.((((((((((.((((((.((((......)).)).)))))).)))))))))).)))))))..))))))))..)))))))...	278.28	203.61	418.76	211.99	207.93	198.19
aca-miR-144-3p_R+2	TACAGTATAGATGATGTACTCT	22		gp3	.(((......(((..((((((((((.(((((((((.((.(........)))))))))))))))))))).)).))).........))).	269.23	566.10	619.49	678.84	611.59	581.81
tgu-let-7i-3p	CTGCGCAAGCTACTGCCTTGCT	22	1	gp2	((((..(((((.((((((((((((((((((.(((((.(((((.........)))))))........))).)))))))))))))))))))))..))))..........	240.41	212.00	132.11	190.55	204.75	211.92
xtr-miR-132	TAACAGTCTACAGCCATGGTCG	22		gp3	..((((((.((((.(((((((((.(((((((((((((((....)))...)))))))))))).))))))))).))))).))..))))..	233.83	296.52	176.36	165.54	101.31	76.53
ssa-miR-125a-5p_R+2	TCCCTGAGACCCTAACTTGTGAAT	24		gp3	..((((((((.((((((..(((((.............)).)))..)))))).)))))))....	200.07	253.18	281.85	79.79	75.85	57.89
ssa-miR-99-5p_R+2	AACCCGTAGATCCGATCTTGTGAA	24		gp3	((((......)))).((((((...((((..(((..(((.((((((.............)))))).)))..)))..))))...))))))...((((....)))).	182.78	338.59	690.03	433.90	434.42	228.93

tgu-miR-2970-3p_L+1	CAGATCACCTCTTGGCTGTGGGT	23		gp3	((((((((((.(..((((.((...(((...(((..((((((((.(..(..(((((((..((.(((((((((((.....)))....)))))))).))..)))))))..)..).))).)))))..))))))...)).))))..)))))))))))	169.61	173.87	84.65	158.40	101.84	60.83
dre-miR-24_R+2	TGGCTCAGTTCAGCAGGAACAGTT	24		gp3	(((((((..((.(((((((.((((((.((((((..(((((((.........))))))).)))))).)))))).)))))))).)))))))))	151.49	171.58	202.65	70.27	101.84	88.30
xtr-miR-146b_R+2	TGAGAACTGAATTCCATGGACTTT	24		gp3	....((((..((((.((((..(((.(((((((....(((((((.................)))))))....))))))).))).)))).))))))))...	146.83	299.76	494.12	137.75	145.25	123.29
oan-let-7f-1-3p_R+2_1ss8AG	CTATACAGTCTATTGCCTTCCT	22		gp3	...(((..(((((((((((((..(((...((((((((((((((((((((.(((((((((.....))))))))........)).))))))))))))))))))))..))).)))))))....((......))..))))))).)))....	143.26	167.77	128.90	66.69	60.47	49.06
oan-let-7f-5p_R+1_1ss22TA	TGAGGTAGTAGATTGTATAGTAT	23		gp3	...(((..(((((((((((((..(((...((((((((((((((((((((.(((((((((.....))))))))........)).))))))))))))))))))))..))).)))))))....((......))..))))))).)))....	142.85	203.79	319.68	18.46	26.34	16.68
aca-let-7f-5p_R+1_1ss22TA	TGAGGTAGTAGATTGTATAGTAT	23		gp3	...(((((.(((..((((((((((((((((...(((.....))).(((.....))))))))))))))))))..)))))))))..	142.85	203.79	319.68	18.46	26.34	16.68
tgu-miR-125-2-3p_R+1	ACGGGTTAGGCTCTTGGGAGCT	22	24	gp2	...(((..((((..(((.((.((((.((((.((((((..(((..((((((...)))))).)))..)))))).))))))))..)).)))..))))..)))...	141.61	134.98	145.57	179.83	231.27	308.07
ggo-miR-1260b_R+2_1ss9AG	ATCCCACCGCTGCCACCATT	20		gp3	(((((.....(((.((((...((((((((((..(((((((((..(((.....((((....)))).)))..)))))))))..)))..))))))))))).))))))))..	139.97	134.98	40.40	41.68	22.28	17.66
tgu-miR-222-5p_R+1	CGCTCAGTAGTCAGTGTAGATTC	23	1	gp2	...((.((.((..(((((...((((((((((((((((((...(((..........)))...))))).))))))))))))))...))))).)).))))....	133.38	129.64	159.68	192.93	212.17	151.09
hsa-miR-132-5p_1ss12CA	ACCGTGGCTTTAGATTGTTACT	22		gp3	..((((((.((((.(((((((((.((((((((((((((((....)))...))))))))))))).))))))))).)))).))..))))..	121.85	125.83	82.73	69.08	75.32	51.02
aca-miR-144-5p_R+2	GGATATCATCATATACTGTAAG	23		gp3	.(((......(((..((((((((((.(((((((((.((.(........)))))))))))))))))))))).)).)))........))).	117.74	299.70	477.12	330.49	347.96	238.41

	T										
	CGGCTGGGAAG	2		gp	..((((((((.((((((((.((..((.((((.(((((..((((.......)))).)))))))))))).)).)))))	116.	54.9	33.3	61.9	54.1	131.
PC-5p-8271_542	GGCTGCCGGC	1	24	4	))).)))).))))))..	91	1	5	3	0	47
cin-miR-4171-5p_R-	TGACTCTCTTAA	1		gp		110.	16.0		41.6	33.9	92.2
2_1ss15AT	GGTAGC	8		3	.(((((((((((((((........)))))))))))..)))))).	33	1	3.21	8	5	3
ola-miR-199a-3p_R+	ACAGTAGTCTG	2		gp	..((((((((.(((...(((((((.((((((((.(((.((..(......)..)).))))))))))).)))))))...	109.	126.	135.	69.6	58.8	42.1
1_1ss22AG	CACATTGGTTGT	3		3	))).)))))))))..	50	21	95	7	8	9
	TGGTGTATGTG	1		gp		107.	17.5	16.6	35.7	38.1	259.
PC-5p-15509_312	CTTGGCT	8	24	4	...(((((.(((((.(((((((...((..(((......))).)).))))))))..))))))..)))))...	03	4	7	3	9	02
	TAATACTGTCTG	2		gp	.(((...((((((((((..((((((((((..(((((((..((.....)).))))))))..))))))))))...)))))	97.1	150.	135.	35.7	41.3	43.1
xtr-miR-429_R+2	GTAATGCCGTTT	4		3	))))....)))	5	99	31	3	7	7
aca-miR-212-3p_R-2	TAACAGTCTAC	2		gp	(((.(((((((((.((.(((((...(((((.((((((.....((....))......))))))))))))...))))).)	95.5	99.0	82.4	94.0	106.	50.0
_1ss9CT	AGTCATGGCT	1		3	))).....))))..))).))).	1	1	1	9	62	4
	TAACAGTCTAC	2		gp	..((((.(((.((((..((.(((((.(((((((.(((((((....((((...))))..))))))))))))))).))	95.5	99.0	82.4	94.0	106.	50.0
xtr-miR-212	AGTCATGGCT	1		3	))).))...)))).)))))))	1	1	1	9	62	4
tgu-miR-1784-3p_1s	TGACTTAAATA	2		gp	...(((((((((.((((((((((((((((((.((.((((((((....))..)))))).)).)))))))))))))	88.9	67.1	96.8			
s11GA	GGAGCAGAATT	2	3	2	))))).)))))))))...	2	1	4	2.38	0	0
hsa-miR-32-3p_R-1_	CAATTTAGTGT	2		gp	....(((.(((.((.(((((..(((((((((.((((((((((((((..((....))..)).)))))))))))))))	87.2	103.	90.4	58.3	82.7	84.3
2ss15TC20TC	GTGCGATACT	1	2	2	)))))))..)))))...)).))).)))..)	7	71	2	6	5	8
	AGCTACATTGT										
	CTGCTGGGTTTC	2		gp	..(((...((..(((((.(((..(((.(((((((((....((((((((...)))).)))))))))))))).)))..)	84.8	131.	170.	78.6	105.	105.
xtr-miR-221_R+2	TT	5		3	))..))))))..))))).	0	17	26	0	56	96
	CCCAATATTATT	2		gp	..((((.((((((((((((..(((((((((.((..(((........))).)).)))))))))..)))))))))..)).)	83.9	121.	128.	145.	264.	198.
tgu-miR-16b-3p	GTGCTGCTT	1	9	2	)))	8	25	90	30	16	19
hsa-miR-219b-5p_L-	GATGTCCAGAC	2		gp	...((((((((((...(((((((((((.((((((((((...(((.........)))..)))))))))).)))))))).	83.9	93.8	164.	91.7	103.	121.
1_2ss11CA21CT	ACAATTCTTG	1	17	2	)))...)))))))))))...	8	0	17	0	96	66

hsa-miR-4792_R+1_1ss9GT	CGGTGAGCTCTCGCTGGCC	19	gp3	((((...((((.((((..(((.(((((.(..((..((....)).))..))))))..))).)))).)))).))))	80.69	52.62	30.14	114.33	73.20	335.54
oan-miR-16c-5p_R+2	TAGCAGCACGTAAATACTGGAGAT	24	gp3	((.((((.(((....))))))).))...(((((((....((((((((((..(((((...)))))).....)))))))))....)))))))...(((.((((((.....))).)))..)))	73.28	100.66	116.72	64.31	50.92	45.13
hsa-miR-4454_L+1_1ss3GA	CGAATCCGAGTCACGGCACCA	21	gp3	..((.((.....((((((((((.......)).))).)))))....))..))....	72.45	54.14	26.93	48.83	30.77	54.94
hsa-miR-4508_L+2R-1	AAGCGGGGCTGGGCGCGC	18	gp3	.((.(((.((((.(((.((((((.((.((.((((...)))).)).)))))))))))..)))).)))))).	70.81	58.72	21.80	61.93	22.28	94.19
mdo-miR-22-3p_1ss21GC	AAGCTGCCAGTTGAAGAACTCC	22	gp3	..((((..((((((((((((((((((.(((((((.(.((......))))))))))))))).))))))))))))).))))..	67.51	54.14	50.66	65.50	41.37	51.02
bta-miR-2478_1ss2TA	GAATCCCACTTCTGACACCA	20	gp3	..(((..(((((..(((.(((((....((((.....))))..)))))....)))..))))).)))............	67.51	83.89	30.78	53.59	45.62	56.91
mmu-miR-6238_L-1R-3_1ss12TC	TATTAGTCAGCGGAGGAA	18	gp3	....((.(((...((.(((...(((((..(((((...(((((...(((((...(((((........)).)))))))))).)))))).))))).)))))...)))....)).))).))..............	59.28	10.68	5.13	19.06	16.97	92.23
aca-miR-30c-5p_R+4	TGTAAACATCCTACACTCTCAGCGT	25	gp3	(((((...((((....(((((((.(((...(((((((((...............))))))))).))).)))))))....)))).)))))...	55.99	78.55	102.61	116.71	126.24	125.58
mml-let-7a-5p_R+2	TGAGGTAGTAGGTTGTATAGTTTA	24	gp3	(((..(((.(((.(((((((((((((.........(((......))))))))))))))))).))).))).)))	52.83	95.37	145.57	31.36	45.26	36.30
mmu-miR-3968_1ss14AT	CGAATCCCACTCCTGACACCA	21	gp3	...((..((..((((((((.((((.(((..((.....((((((((.........))))))))...))...))).)))).))......))))))..)).))	51.05	49.57	24.37	28.58	19.10	31.40
aca-miR-212-5p_R+1	ACCTTGGCTCTAGACTGCTTACT	23	gp3	(((.(((((((((.((.(((((...(((((.((((((.....((....))......)))))))))))...))))).))))).....)))))..))).))).	47.20	50.20	33.03	51.21	26.52	20.93
ssa-miR-212b-5p	ACCTTGGCTCTA	2	gp	..((((.(((.((((..((.(((((.(((((((.(((((((....((((...))))..)))))))))))))).))	47.2	50.2	33.0	51.2	26.5	20.9

	GACTGCTTACT	3		3	))).))...)))).)))))))	0	0	3	1	2	3
	ACCTTGGCTCTA	2		gp	((((((...((....)).(((.(((.(((..((.(((((..((((((.((((((...(((...))).)))))))))	47.2	50.2	33.0	51.2	26.5	20.9
hsa-miR-212-5p	GACTGCTTACT	3		3	)))..))))).))...))).)))))))))))..	0	0	3	1	2	3
	AACCCGTAGAT										
aca-miR-99b-5p_1ss	CCGAACTTGCG	2		gp		42.8	60.2	66.6	27.1	45.6	28.7
23GT	T	3		3	..((((.((((..(((..(((.(((((((.....)))).......)))).)))..)))..)))).))))..	1	5	9	9	2	8
	AGCAGCATTGT										
	ACAGGGCTATC	2		gp	..........((((..(((.((((((.(((.(((((((((((.((((.((...)).).))))))))))))))).)))	42.8	76.2	106.	42.8	46.6	51.0
ola-miR-103_R+4	ATT	5		3	))..)))).)))..))))..............	1	6	45	7	8	2
	ACATCCTCCTTT	1		gp	.(((((.((((((((...((((((.(((.((..((((.....(((..........)))......))))..)).))))))	41.1					13.7
PC-3p-131359_24	CCCTCCA	9	13	4	))).)))))))))))).....	7	0.76	0.64	3.57	7.43	4
	TATGGCACTGG	2		gp		39.5	39.6	40.4	65.5	181.	51.0
bta-miR-183_R-1	TAGAATTCACT	2		3	((((.((((((..(((((..(((((((((((......)))..))))))))))))....)))))).))))	2	6	0	0	41	2
	TTTGGCACTAG	2		gp	(((..........(((......................)))(((.((((....))))))))).(((((...((((..(((	37.8	73.2	111.	69.0	143.	54.9
hsa-miR-96-5p	CACATTTTTGCT	3	1	2	((((((.(((((..((((((.((.......))...))))))..))))))))))))))..))))....)))))...	7	1	58	8	22	4
	TGGAGTGTGAC										
oha-miR-122-5p_R+	AATGGTGTTTGT	2		gp		37.8	77.7	96.8	14.2	20.1	13.7
1_1ss25TA	AAT	6		3	...((((((....(((((((.(((((((((((.((.......))))))))))))).)))))))....)))).)).	7	8	4	9	6	4
	CCCAGTGTTCA										
ola-miR-199a-5p_R+	GACTACCTGTTC	2		gp	..((((((((.(((...(((((((.((((((((.(((.((..(......)..)).))))))))))).)))))))...	37.0	49.9	74.0	37.5	37.6	20.6
3	TT	5		3	))).)))))))))..	5	5	7	1	6	0
	CCCAGTGTTCA										
	GACTACCTGTTC	2		gp	(((.((.((((.(..(((((((.((((((((.((((((............))))))))))))))).)))))))..).	37.0	49.9	74.0	37.5	37.6	20.6
tni-miR-199_R+2	TT	5		3	)))).)).)))........	5	5	7	1	6	0
hsa-miR-16-5p_1ss2	TAGCAGCACGT	2		gp	((..((((..(((((((((..((((((((.((..(((........))).)).))))))))..)))))))))..)))	36.2	40.4	64.1	71.4	64.7	29.4
2GA	AAATATTGGCA	2		3	)..))	3	2	3	6	1	3

hsa-miR-21-5p_R+2_1ss1TN	NAGCTTATCAGACTGATGTTGACA	24		gp3	.(((((..(((..(((.(((((.(((((.(..(.(.....))..)))))).)))))))..)))..))))).	32.93	40.42	41.68	5.36	22.28	12.75
ggo-miR-342_R+1	TCTCACACAGAAATCGCACCCGT	23		gp3	.........(((((.(((((.((((((((.((((((..((((((.....((((((...((....))..)))))).))))))....)))))).)))))))))))))).)).)))	32.93	17.54	16.67	71.46	60.47	39.24
hsa-miR-3120-3p_R+1	CACAGCAAGTGTAGACAGGCAT	22	8	gp2	...(((((.......(((.(((((((((((((.(((((((((((..(.((((((.((((..........)))).)))))).)..)))))))))))).))))))))))))).))))))))...	31.29	26.69	21.16	10.72	5.30	13.74
oan-miR-15a-3p	CAGGCCATATTGTGCTGCCTCA	22		gp3	((((((((((((..........((((((((..((((..((((..........))))..))))..))))))))..........))))))...)).)))))	27.99	14.49	22.45	16.67	23.34	29.43
oan-miR-1386_L+2	GACTCCTGGCTGGCTCGCCA	20		gp3	(((((.(((((...((((((..(((((......)))))...............((((((.....))))))......).)))))).))))).).)))))	26.35	28.22	6.41	21.44	6.37	17.66
tgu-miR-30a-3p_R+1_1ss22CT	CTTTCAGTCGGATGTTTACAGTA	23		gp3	.....((((((..(((..((((((((((((..((((((((((((..............)))))))))))))))))))))))))..)))))))))	25.52	26.69	46.49	26.80	37.66	36.30
oan-miR-1388-3p_R+2	ATCTCAGGTTCGTCAGCCCATGTA	24		gp3	....((((((((((.((..((.(((...(((((((((....((((........)))).)))))))))...))).))..)).))))))..)))))..	24.70	63.68	46.49	0	0	0
mmu-miR-3971_R-1_1ss6CA	CTCCCAACCCCTGTACCAGTG	21		gp3	......((((..(...(((.(..((.(((((.((((((((.....))))))))..)))))))..).))))..))))........	24.70	28.22	11.22	5.36	4.24	6.87
cin-miR-33_R+4	GTGCATTGTAGTTGCATTGCAAT	23		gp3	((((((...((((...((.(((((.((..((((((.(..(......)..)))))))..)).))))).))...))))...)))).))...............	23.05	22.12	22.45	0	3.18	3.92
ssc-miR-451_L+3R+1	ATCAAACCGTTACCATTACTGAGTTT	26		gp3	.((.(..(((((..((((((((((((((((((....))))))))))))))))))..)))))..).))	23.05	57.19	63.49	16.67	14.85	7.85
aca-miR-18a-3p_L+	TACTGCCCTAA	2		gp	....((..((((((((.((((((.(((..(((.((((.(((....((......))..))))))).)))..))).))).	21.4	28.2	20.5	86.9	107.	98.1

1R-1_1ss12GA	ATGCTCCTTCT	2		3	.))).))))))))..)).	1	2	2	4	15	1
	CAAAGTGCTTA										
dre-miR-17a-5p_R+	CAGTGCAGGTA	2		gp	(((((.(((..((..((((((((.((.(((((.(((...........))))))))).)).))))))))...))..)))	19.7	37.5	69.5	20.5	22.2	31.4
3	TTT	5		3	.)))))	6	6	8	4	8	0
hsa-miR-7704_R-1_	CGGGGTCCGCG	1		gp		19.7	20.5		20.2	14.8	19.6
1ss8GC	GCGACGT	8		3	(((((((((.((((....((..(((((((...)))))))..))))))))))).))))..	6	9	3.21	5	5	2
	AAGCTGAAGGA										
mmu-miR-8094_L+1	CAACGAGAAGA	2		gp	..((..(((((.(((.(((...(((.((((((((((.((((((((...((.....))....)))))))).))))))	19.7			21.4	32.8	45.1
R+3_1ss3AG	ACC	5		3	.............))))).)))..))))))))))).)).	6	0	0	4	9	3
	GTTAGTTTTGCA	2		gp		18.1	23.6	23.0	41.6	62.5	37.2
tgu-miR-19a-5p_L+2	TAGTTGCACT	2	1	2	.(..((((((((((((((((((((((((.......))))...))))))..)))))))))))))..)..	1	4	9	8	9	8
	TCACAGTGAAC										
	CGGTCTCTTTTA	2		gp	..((((..((((..((((((((...((((((..((((............))))))))))...))))))))..))))..))	18.1	25.9	49.3	35.7	22.2	29.4
xtr-miR-128_R+2	T	4		3	))	1	3	8	3	8	3
tgu-miR-1662_L+1_	CTTGACATCATC	2		gp		18.1	15.2	12.8			
1ss23TA	ATACTTGGGAA	3		3	.((((((((((((((...((((((((............))))))))..)).))))))))))))).	1	5	3	9.53	5.30	3.92
	CAGAGCAGCTC	1		gp	...(((.((((((.(((.(((((((..(((((((((.((......)).)))))..))))....))))))).))).))	18.1			14.2		54.9
PC-3p-44198_100	CCTCGCT	8	7	4	)))))))...	1	7.63	3.85	9	9.55	4
	CAAAGTGCTTA										
	CAGTGCAGGTA	2		gp	....((((((((..((((((((.((.(((((.((((((((....)))).)))))))).)).))))))))...))))	16.4	17.7	25.3	15.7		17.6
ccr-miR-17-5p_R+3	GTAT	6		3	))))	7	3	3	8	8.49	6
	TGTCAGTTTGTC										
	AAATACCCCAA	2		gp	...................((((((((...((((((((((((((......(((((((.........)))))))))))))	14.8	28.9	29.5			
dre-miR-223_R+4	AA	5		3	))))))))...))))))))	2	8	0	8.34	7.43	7.85
mmu-miR-2137_L-1	CCGGCGGGAGC	1		gp		14.8					21.5
R-1_1ss16AG	CCCGGGGA	9		3	...(((...((((((((((..(((..(..((....))..)..).))..)))))))..)))...)))	2	5.34	0	9.53	8.49	8

efu-miR-9226_1ss22	TCAAGTCCCTGT	2		gp	...(((...(((..(((.((((..((...(((((....((((((....))))))))))..))..))))))))))..)	14.8	32.0		27.3	28.6	43.1
GA	TCGGGCGCCA	2		3	))..........	2	3	7.70	9	4	7
aca-let-7e-3p_L-3_2s	CTATACAGTCT	2		gp	...(((((.(((..((((((((((((((((...(((.....))).(((.....))))))))))))))))))))..))	14.8	22.1	25.0	15.4	23.3	21.5
s11AG23TC	ACTGTCTTCC	1		3	)))))))..	2	2	1	8	4	8
	GAATTGCATCT				.........((((((((((.(((((((.....((((((..(((((((((((((((..((...........))..))))						
	GGATTAAGAAC	2		gp	.))))))))))).))).(((..........................)))........)))).))......)))))))....))).	14.8					
PC-3p-854650_3	A	3	14	4	)))))))	2	0	0.64	1.19	4.24	3.92
	TCGGATCCGTCT	2		gp	...(((((((.((((..((.((....))....(((..((.(((.(((((((((.((.((((((((.....))..))))	13.1					17.6
efu-miR-127_L-1R-1	GAGCTTGGCT	2		3	)).)).))))))))))))).))..))).))..)))).)))))))(....).....	7	1.53	1.28	3.57	4.24	6
	ATGACCTATGA	2		gp	(..((((((((...((.(((((((..(((((((((.....((.....))...))))))))).)).))))).))...)	13.1			30.3	33.9	
rno-miR-215	TTTGACAGACA	2		3	)))))))..)..............	7	0.76	6.73	7	5	0
aca-miR-425-3p_L-2	TCGGGGATGTC	2		gp	...((((((((((((((..(((((((.((..(((((((.((.......))..))))...)))..)))))))))..))	13.1	12.2				
R+1_1ss3AT	GTGTCTGTCC	1		3	))))))))))))...	7	0	5.77	8.34	7.43	9.81
					...(((.(((((....(((.....)))...)))))...((((((......))))))...)))..((((((.((((((..						
	AATGTGGAAAG	2		gp	.(((((..((.((.((((((((...(((((.......((((((.....))))))...)))))......))))))))...	13.1					
PC-5p-1529983_2	CTGCCTCTTGCC	3	24	4	))))..))))))))))))))))))	7	0	0	0	0	9.81
aca-miR-10c-5p_R-1	TACCCTGTAGA	2		gp	.(((((..(((((..((((.((((..((((((((((((((...))))..))))))))))..))))))))..))))	11.8	33.1	28.4	10.3	14.8	18.9
_1ss12AC	CTCGAATTTGT	2		3	)..)))))......	0	7	3	2	5	7
pma-miR-10a_1ss9G	TACCCTGTAGA	2		gp	...(((((((..(((((...(((..(((.(((((((((((....(.....)....)))))))))))).)))..)))..)	11.8	33.1	28.4	10.3	14.8	18.9
A	CTCGAATTTGT	2		3	)))).)))))))...	0	7	3	2	5	7
ola-miR-126-5p_R+	NATTATTACTTT	2		gp	.........(((((..(((((((.((((((((((..(((((((...................)))))))..))))))))	11.5	16.0	14.1			
1_1ss1CN	TGGTACGCGA	2		3	)).))))))))))))..........	3	1	1	8.93	7.96	4.91
aca-miR-129b-3p_R	AAGCCCTTACC	2		gp	....((((.(((((((((((((((.(((..((((((((((((((.......)))).......)))))))))..))).)	11.5				16.4	
+1	CCAAAAAGCAT	2		3	))))))).)))))))))..))))...(((...))).....	3	9.15	9.62	9.53	4	5.89
aca-miR-194-5p_R+	TGTAACAGCAA	2		gp	..((..((((((((((((((((((..((((((.((((((((......))..))))))))))))..))))))))))))	11.5					
2	CTCCATGTGGA	4		3	)))))))..))..	3	2.67	0.64	0	1.06	0

	AA										
dre-miR-2184_R-1_1ss14TG	AACAGTAAGAGTTGATGTGC	20		gp3	((((..((..((((...(((((...((((((((...((((((((((........))))))))))...))))))))..)))))...))))..))..))))..........	11.53	22.88	8.34	4.76	1.06	1.96
PC-3p-639958_4	CAACTGGAGGAGCTGCTGAGC	21	5	gp4	(((((..........(((((((((.((((((((((...(((..((..((((.....))))..))....)))...))))....)))))).))).)))))))..)))))...	11.53	0	0	0	2.12	0
ssa-miR-29b-3p_R-1_2ss20TC21TC	TAGCACCATTTGAAATCGGCC	21		gp3	((((((((((((((.....))))).((.(((((....)))))..))....))))))))))..	10.70	6.93	5.93	0	1.06	0.65
dre-miR-187_R+4	TCGTGTCTTGTGTTGCAGCCAGTT	24		gp3	............(..(((..((((((((((((((((((((((((.((.....)))))))....))))))))))))))))))..)))..).	10.70	6.86	5.45	1.19	2.12	9.81
mmu-miR-6412_R-2_1ss15AT	TCGAAACCATCCTCTGCTAC	20		gp3	.(((.((((.(((.(((((..((.(((((((..((((....((((.((....)))))).))))...))))))).)).).)))))..))))))))))..	9.88	13.73	10.26	2.38	6.37	5.89
PC-5p-278153_9	ATCAAATGCATGCATGCC	18	10	gp4	.....(((((....((((((.((((...(((((((((........(((((((((((((........))...))))))).)))))....)))))..))))..))))..............(((((...((....))..))))).))))))).....)))))......	9.88	0	0.64	4.76	8.49	3.92
oha-let-7i-5p_R+2	TGAGGTAGTAGTTTGTGCTGTTCT	24		gp3	.....(((((.((((((((((((((((((.(((.(((((((.(.....).))))........)))))).)))))))))))))))))))))))....	9.47	12.20	8.66	7.74	6.37	0.98
tgu-miR-30a-5p_R+2	TGTAAACATCCTTGACTGGAAGCTTG	26		gp3	.....((((((..(((..((((((((((((..(((((((((((..............)))))))))))))))))))))))))..)))))))))	9.06	12.39	19.61	26.50	40.93	37.94
mdo-miR-210-3p_L-1R+3	CTGTGCGTGTGACAGCGGCTACT	23		gp3	((((..(((.((.((((.(((....(((((((.((...........)).)))))))....))).)))).)).)))..)))).	9.06	8.01	2.89	23.22	11.14	3.92
mmu-miR-486a-5p	TCCTGTACTGAGCTGCCCCGAG	22		gp3	..((...(((((...((.(((.((((((...((((((((((((((((((((((........(((....))))))))))))))))))))))))))))...)))))).))).))...))))).....))..	9.06	8.39	43.61	69.67	83.81	13.74

tgu-miR-194-3p	CCAGTGGAGATGCTGTTACTTT	22	3	gp2	...(((((((((.((((((.((((...........)))))))))).)))))))))).....	8.23	0	0.64	0	2.12	1.96
aca-miR-23a-5p_2ss12TC17TC	GGGGTTCCTGGCGATGCGATTT	22		gp3	.(((((......(..((((((((..((((((((((............))))))))))..))))))))..).......))))).((....)).	8.23	5.34	3.85	7.15	2.12	7.85
rno-miR-25-3p	CATTGCACTTGTCTCGGTCTGA	22		gp3	((((.(..(((..((((.((((((.(((((((.(((((.......))..)))))))).)))))))).)))).))).).))))	8.23	3.05	30.14	103.61	75.32	13.74
age-miR-93_L+1	CAAAGTGCTGTTCGTGCAGGTAG	23		gp3	((((((((((..((((((((((((.((((.(((.((.........)))))))))))))).)))))))...)))))))))))	8.23	2.29	25.65	70.27	27.58	13.74
ola-miR-222_R+7	AGCTACATCTGGCTACTGGGTCTCCT	26		gp3	((((...((((.((((((((...((((((((((((((((((...((((.......))))...))))).))))))))))))))...))))).)))))).))...))))	8.23	5.34	8.34	15.48	8.49	13.74
bta-miR-454_R+1_1ss23TA	TAGTGCAATATTGCTTATAGGGAT	24		gp3	.(((((....(((((..((((((((((((((((((((((((.(((((....)))))...)))....))))))))).)))))))))))))..)))))...))))).........	8.23	26.69	76.31	23.82	40.31	27.47
PC-5p-350760_7	TGATTTGTATTTCTTCTTTTCC	22	2	gp4	..(((((...((((((((..((.....))..............................((((((.(((((....((..(((((..((((((..((...(((.....))).)).))))))..))).))..)).......))))).))))))......))))))))....))))))..	8.23	0	0	8.34	13.79	9.81
PC-5p-307705_8	AATTGATTTTTTGCTCGTGCATT	37	7	gp4	....((....)).....((((((.((.(((...((((((......))))))..))).)).))))))..............((((((((((((..(((((...(((.........)))...)))))..))))))))))))((((((((((.((((((.....……)))))))))).)))))	8.23	0.76	1.28	7.15	15.91	9.81
aca-miR-129b-5p_R+1_1ss20TG	CTTTTTGCGGTCTGGGCTTGC	21		gp3	....((((.(((((((((((((((.(((..((((((((((((((.......))))).......)))))))))..))).))))))).))))))))..))))...(((...)))….	7.41	4.58	5.45	10.72	8.49	8.83
pma-miR-181a-5p_R+3	AACATTCAACGCTGTCGGTGAGTTAT	26		gp3	...(((...(((.((((((..(((((((.((((.(((......)))...))))))))))))))))).)))...)))...	7.41	5.72	10.90	20.84	14.32	25.51

hsa-miR-181b-3p_L	GCTCACTGAAC	2		gp	.....((.((((.....))))....(((((((.....(((((((((...((((((((((...((....))...))))))				18.4	17.5	12.7
+1R-1	AATGAATGCA	1		3	)))).))))))))))....))))))).))..	7.41	1.53	7.37	6	0	5
	AACATTCAACG										
	CTGTCGGTGAG	2		gp	..(((((((..(((..((((((((((..(((((((.((((..((...))....))))))))))))))))))))))..			10.9	20.8	14.3	25.5
aca-miR-181a_R+3	TTAT	6		3	))).))))))).....	7.41	5.72	0	4	2	1
	TAACACTGTCT										
dre-miR-141-3p_R+	GGTAACGATGA	2		gp	(((.((((((((.(((((((((.(((((((.((((....((...))....))))))))))).))))).)))).)))			14.7			
1_1ss22CA	A	3		3	)))).)))	6.59	3.05	5	2.38	5.30	0
mdo-miR-186-5p_R-	CAAAGAATTCT	2		gp					13.1	13.7	27.4
1	CCTTTTGGGCT	2		3	...(((((((((((((.(((((.(((((...((((...))))..)))))))))).)))))))))))))	6.59	4.58	3.21	0	9	7
tgu-miR-338-3p_R+	TCCAGCATCAG	2		gp				17.9			
1_1ss22GA	TGATTTTGTTAT	3		3	(((((((..((((((((((((...((((..........))))))))))))).)))..))))))))........	6.59	6.86	6	3.57	1.59	0
dre-miR-204-5p_R+	TTCCCTTTGTCA	2		gp			26.6				18.6
2	TCCTATGCCTGT	4	28	2	.(.(((((((((..((.(((((.((((..((...))....))))))))).))..))))))))).).	5.76	9	9.30	2.98	3.71	4
hsa-miR-374a-5p_R-	TTATAATACAA	2		gp					10.7	11.6	19.6
1	CCTGATAAGT	1		3	((((..(((.((((((((((((.((((((((((((....)))))))))))).)))))))))))).)))..))))	5.76	6.10	2.57	2	7	2
	TCAGGCTCAGT	2		gp							
bta-miR-484	CCCCTCCCGAT	2		3	((((((...(((((((....((......))......(((.....))).))))))).))).)))	5.76	1.91	1.28	4.17	3.18	5.89
xtr-miR-10c_L-1_1s	ACCCTGTAGAC	2		gp							
s12AC	TCGAATTTGT	1		3	(((((((.(((..((((..((((((((((...........))))))))))..))))..))))))))))..	5.21	8.77	9.83	4.96	5.30	8.18
	TGAGAACTGAA										
	TTCCATAGGCT	2		gp	...((......((((((((.(((.((.((((((((.((((.((((..((..((....))..))..)))))))).)))						
chi-miR-146b-5p	GT	4		3	))))))).))).)).)).))))).....))...	5.21	4.51	2.99	3.18	8.93	5.56
mmu-mir-703-p3_1s	AGACTGGTTAA	1		gp							
s16AT	TGATTACA	9	3	2	...((((((((.(((..(((((((.((....)).))))))).)))))))))))...	4.94	0.76	0	5.95	5.30	9.81
pma-miR-1c-3p_R+1	TGGAATGTAAA	2		gp	...(((((((.((((.((((((((.(((..(((.((((.......(((....))))))))))..))).)))))))).	4.94	0.76	8.98	1.19	3.18	5.89

	GAAGTATGTAC	3		3	)))).)))))))...						
	C										
	CTGGCAGTGTC										
dre-miR-34a_L+1R+	TTAGCTGGTTGT	2		gp	.....((((..((((((..(((((((((((.((((((((((..((.........))..))))))))))))).)))))						
2	TT	5		3	)))..)))).))..))))...	4.94	6.10	7.70	4.76	5.30	5.89
	GTCCAGTTTTCC										
pma-miR-145-5p_R	CAGGAATCCCT	2		gp	.(((((.((.(((..(((((((((..((((((((.............)))))))).)))).))))).))).)).))		21.3	42.0			11.7
+2	AT	5		3	))).	4.94	5	0	7.15	8.49	7
	TTATAAAGCAA	2		gp	..((((((..(((((((...((((((.(((...((((.......))))..))).))))))....))))))).))))						
cgr-miR-340-5p	TGAGACTGATT	2		3	)).….	4.94	3.43	1.60	5.36	7.43	2.94
	TTATAAAGCAA	2		gp	..(((((.((((((..(((((((...((((((.((((..((.........))...)))).))))))....))))))).						
hsa-miR-340-5p	TGAGACTGATT	2		3	.)))))).)))))….	4.94	3.43	1.60	5.36	7.43	2.94
	GTGGATCACGA	2		gp	.....((..(((.(((..(((((((((..((.((((((((((.................))))))))))..)))))))				25.2	27.9	34.3
aca-miR-363-5p	TGCAATTTTGA	2		3	))..))).)))..)).….	4.94	2.54	3.95	1	4	4
	ACTGGACTTGG	2		gp	.........(((((((((....((((.((((.((.((((((((((((((...((((((........)))))).)))))			13.4	46.4	21.2	
ggo-miR-378a_R-1	AGTCAGAAGGC	2		3	))))))))).)).)))).)))).))))))))))..	4.94	1.53	7	5	2	5.89
	CAGGCTGGTTA										
pma-miR-456_R+1_	GATGGTTGTCC	2		gp	(((((((..(((((((..(((((..(((((((....(..(..(....)..)..))))))))..)))))..)).))))				13.1	13.7	13.7
1ss22AC	C	3		3	))))))))	4.94	2.29	3.85	0	9	4
	CCTGCATTGTAC										
aca-miR-460a-5p_R	ACACTGTGTGA	2		gp	.(((.((((((...(..(((((((....(((((..(...........)..)))))....))))))))..)...))))))..		11.4				
+2	A	4		3	))).	4.94	4	8.34	4.76	9.55	5.89
rno-miR-551b-3p_L-	GCGACCCATAC	2		gp	..(((((((..(((..((((((((((..((((((...((((........))))....).)))))..))))))))))..					10.6	17.6
1R+1	TTGGTTTCAGTT	3		3	)))..)))))...)).….	4.94	5.34	6.41	3.57	1	6
	ACTGCATTTAA	2		gp	.....(((((....((((((.((((...(((((((((........((((((((((((((........))...)))))))						11.7
PC-3p-1007407_3	ACTCGTATCCT	2	10	4	.)))))....)))))..))))..)))).............(((((...((....))..))))).))))))....))))	4.94	1.53	0	2.38	8.49	7

					)......						
PC-5p-45606_96	TGATGAAATCCCCCTATGGCCTGT	24	2	gp4	...(((..(..((((((((((.(((((..(((((..(((....))).))))))))))).))))))))))..)..)))...	4.94	26.69	28.22	11.91	20.16	9.81
mmu-miR-466i-5p_L-1R-1	GTGTGTGTGTGTGTGTGT	18		gp3	(((..((.((..((((((((((((((((..((((.....))))..))))))))))))))))..)).))..))).	4.47	0	0	0	0.80	0
aca-miR-146a-5p_R+2	TGAGAACTGAATTCCATAGGCTA	23		gp3	(((((((...((((((((((.((((.((((((((.....)))))..)))..)))))))))))))..))))))).	4.39	0.44	1.71	0	0.27	0.98
oha-miR-29b-3p_R+1_1ss23TA	TAGCACCATTTGAAATCAGTGTAT	24		gp3	...(((..((..((((((((((((.((((((..((((((............)))))))))))).)))))))))).)))..))..)))...	4.12	12.77	26.77	1.79	6.37	7.85
cgr-miR-151-5p	TCGAGGAGCTCACAGTCTAGT	21		gp3	((((((.((((((((((((..((((((((((...........)))))))))).)))))))))))).))))))...	4.12	4.96	1.60	5.36	6.90	11.77
aca-miR-221-3p_R+2	AGCTACATTGTCTGCTGGGTTTTA	24		gp3	....((((((((((..(((.((((((((((....(((((.(.....))))))))))))))).)))..)))).))))))....	4.12	8.39	15.07	5.95	7.96	9.81
eca-miR-590-3p	TAATTTTATGTATAAGCTAGT	21		gp3	((((((((.((.((((((((.((((((((.(((((...............))))).))))))))).)))))))).)).)))))))	4.12	0.76	0.64	0.60	1.06	4.91
bta-mir-2406-p3_1ss9GA	AAAGGGAAAAAGGAAGCTG	19	1	gp2	..((((((((((.(((.(((......(((.(((((.((.....(((((...........))))).)).))))))))......))).))).))))))))))...	3.29	0	0	0	0	0
hsa-mir-3120-p5	CTGCCTGTCTGTGCCTGCTGT	21	8	gp2	...(((((.......(((.((((((((((((((.((((((((((((..(.((((((.((((...........)))).)))))).)..))))))))))).))))))))))))).))))))))...	3.29	8.39	2.57	0	0	1.96
odi-miR-7_R+2	TGGAAGACTAGTGATTTTGTTGAA	24		gp3	((((((.((.((((..((.((((((((((.....((((((..(((((....))))....)..))))))...))))))))).))..))))))))))))......	3.29	2.29	3.85	0	0	0

aca-miR-10b-5p_R+4_1ss12AT	TACCCTGTAGATCCGAATTTGTGTGG	26	gp3	((((.((((((((.((((.(((((.((((((((((..((......))...)))))))))).))))))))).)))))))).)))))......	3.29	0	1.28	2.38	0	0
aca-miR-30c-3p_R+2	CTGGGAGAAGGCTGTTTACTCTTT	24	gp3	(((((...((((....(((((((.(((...(((((((((...............))))))))).))).)))))))....)))).)))))...	3.29	0	1.92	1.19	1.06	0
aca-miR-30d-5p_2ss1TN22GA	NGTAAACATCCCCGACTGGAAA	22	gp3	..(((((((.((.(..(((((((((...((((((((((((.......)))))))))))).)))))))))..).)).)))))))..	3.29	10.87	10.31	8.63	9.64	15.37
efu-miR-99b_R-3	CACCCGTAGAACCGACCTTGCG	22	gp3	.((.(((((((((((((((((.....(((((((((((((..((((..(((..(((.((((((((((...)).)))))....)).)))..)))..))))..))))).))))))))........)))))....))))))))))))).	3.29	1.53	2.57	9.53	5.30	19.62
cgr-miR-125a-5p_R-2	TCCCTGAGACCCTTTAACCTGT	22	gp3	(((...(((..((..(((.((((..((((((((((....))....))))))))))))).)))..))..))))))....	3.29	1.53	5.13	8.34	19.10	7.85
hsa-miR-127-5p	CTGAAGCTCAGAGGGCTCTGATAGGGCTCTGAT	22	gp3	...(((((((.((((..((.((....))....(((..((.(((.(((((((((.((.((((((((.....))..)))))).)).)))))))))))).))..))).))..))))..)))))))(....).....	3.29	0	0	0	0	0
cgr-miR-130a-3p_R+2	CAGTGCAATGTTAAAAGGGCATCT	24	gp3	...(((((..((((((((.((((((..((.((((...........)).)).))..)))))).))))))))..)))))....	3.29	12.20	5.77	4.76	7.43	7.85
aca-miR-138-5p_R+4	AGCTGGTGTTGTGAATCAGGCCGTTT	26	gp3	......(((..((.(((((..((((((((((((...(((((((...........)))))))..)))))))))))))).))))).))..)))	3.29	3.81	3.21	25.01	21.22	7.85
bta-miR-320a	AAAAGCTGGGTTGAGAGGGCGA	22	gp3	...((((....))))......(((((((((..(((((((.((((((....)))))).)))))))..)))))))))....	3.29	4.58	44.89	173.88	120.94	5.89
bta-miR-335	TCAAGAGCAATAACGAAAAATGT	23	gp3	...((((.((((((((.(((((((((.((((((((.((......)).)))))))).))))))))).)))))))).))))............	3.29	0.76	2.57	2.38	9.55	3.92
hsa-miR-361-3p_R-1	TCCCCCAGGTG	2	gp	..........((..(((((((((....((((...((((((..(((((((((.((.((((.((((...........)))).	3.29	0	1.28	0	0	0.98

	TGATTCTGATT	2		3	)))).))...)))))))))..))))))...))))........)))))))))..))....						
	TCCCCCAGGTG	2		gp	...((((...((((((..(((((((((.((.((((.((((...........)))).)))).))...)))))))))..)						
chi-miR-361-3p	TGATTCTGATT	2		3	)))))...))))......	3.29	0	1.28	0	0	0.98
aca-miR-460a-3p_R	CACAGCGCATA	2		gp	.(((.((((((...(..(((((((....(((((..(...........)..)))))....)))))))..)...))))))..						
+1_1ss21TA	CAATGTGGAAA	2		3	))).	3.29	4.58	9.62	3.57	2.12	0
hsa-miR-629-5p_R-1	TGGGTTTACCTT	2		gp	..........(((((((((((((((((((((((((((((((((...((....))..)))))))))))))))))))						
_1ss10GC	GGGAGAAC	0		3	))))))))))))))))......	3.29	0	0	0	0	0
	GAGCGGAGCGG										
tgu-miR-2976_L+2R	AGCGGAGCGGA	2		gp	((((((((((..((((..(((..(((..(((..(((..((.(((((..((((((...((((.....))))...)))						
+3_1ss22GA	GCGG	6		3	.)))..)))))))..)))..)))..)))..)))..)))))))....))))))))	3.29	0	0.64	1.19	1.06	5.89
mmu-miR-5124a_L-	GTCAAGTGACT	1		gp							
1R-1_1ss5CA	AAGAGCA	8		3	((((((..(((((((..((..(((((((((..........)))))))))))..))))))).......)))))).	3.29	0	0	0	0	0
	GGCAGATACTG				((((.((....((((..((((....(((..(((.((((((((((((..((..(((((((.(((..((.....)).))						
	GCCGAGCACCT	2		gp	))))))).))..))....))))))))))).))..)))..))).........(((((.........))))).)))))..))))						17.6
PC-3p-1733681_2	TTCA	6	7	4	)))))).......	3.29	0	0	5.95	8.49	6
	TAGCACCATTT										
	GAAATCGGTTA	2		gp	(((((((((....(((((((((.((...(((((((..(((((....)))))).....)))))))...)).)))))))			16.1			
xtr-miR-29a_R+4	AAA	5		3	))..))))))).))	2.47	6.55	9	1.19	0	2.62
	CAGCAGCAATT	2		gp	.........((((((((((.((..(((((..((((((((((((((............))))))))))))))..)))))..						
mmu-miR-322-5p	CATGTTTTGGA	2		3	)).))))))).)))...	2.47	0.38	0.64	5.95	4.77	0
	TAGCAGCACGT										
ccr-miR-457b_L+1R	AAATACTGGAG	2		gp							
+2_1ss10AG	GT	4		3	.(((((((((((.(((..(((((((((................)))))))))..))).)))))))))))...	2.47	0.76	0	3.57	2.12	0
mmu-miR-466i-5p_	TGTGTGTGTATG	2		gp	((((((..((.(((....))).)).))))))..(((((((..(((((..(((((((((((((((((((((((((.						
R+4_1ss10GA	TGTGTGTGTATA	4		3	...)))))))))))))))))))))).)))))..))))))..))))))).	1.98	0	0	0.57	0.80	0
ssa-miR-7a-4-3p_2ss	CAACAAGTCAC	2	10	gp	...((((((.(((.(((..(..(((((..(((.........(((((..(((((((..((.(((((.((((.((((((	1.65	0	0	0	0	0

17AG21CT	AGTCTGCCTT	1		2	((.((..((......)))).))))))))))))))))).))..))))))).))))))))..))))).).))).)))<br>.)))).))						
tni-mir-101b-p5	TCGGTTATCATG<br>GTACCGC	1<br>9	Z	gp<br>2	...(((.((((((.((((((((((((((..((((..(((((............))))).)))).)).)))))))))))<br>)).))))))..)))...	1.65	0	0	0	0	0
hsa-miR-219a-2-3p	AGAATTGTGGC<br>TGGACATCTGT	2<br>2	17	gp<br>2	...((((((((((...(((.(((((.((((((((((((((((....).)))))......)))))))))))).))<br>))))))...)))))))))))...	1.65	0.76	0.64	0	1.06	0
mdo-miR-449c-5p_R<br>+2_1ss1GA	AGGCAGTGTAT<br>TGTTAGTTAGCT	2<br>3	Z	gp<br>2	...(((((.(((((.(((((....(((((((((..(((((((.((((............))))))))))))))))))<br>)).)))))))))).)))))...	1.65	3.05	0	1.19	1.06	1.96
mmu-miR-2137_R-2<br>_1ss16AG	GCCGGCGGGAG<br>CCCCGGGG	1<br>9	6	gp<br>2	...((((((((...((((((((.(((((..((.((.(((.(((..(((((((((........)))))))...)).))<br>)..))).)).)).))))).))).)))))))))))...	1.65	2.29	0	1.19	0	1.96
tgu-miR-2984-5p_R<br>+1	AGCCTGCTGAG<br>AGTGAAATTGA	2<br>2	1	gp<br>2	...(((((((((((.((((((((.(((((((((((.......((.......)).....))))))))))).))))))<br>)).).)))))))))))...	1.65	3.81	1.28	0	0	0
hsa-mir-3618-p5_1ss<br>21GA	TGTGATTTCCAA<br>TAATTGAGAC	2<br>2	15	gp<br>2	...(((((((((.((((((((..(((.((.((((..(((((((..........))))))).)))).)).)))..))<br>)))))).)))))))))...	1.65	0	0	0	1.06	1.96
oha-mir-3618-p3	AGTGATTCTGA<br>AAGCTGT	1<br>8	15	gp<br>2	...(((((((((.((((((((..(((.((.((((..(((((((..........))))))).)))).)).)))..))<br>)))))).)))))))))...	1.65	0.76	0	0	2.12	0
hsa-mir-3665-p3_1ss<br>17AC	GGGCGGCGGCG<br>GCGGCCGC	1<br>9	1	gp<br>2	...((((((((((((((((.(((..........))).)))))))))).))))))))))...	1.65	3.05	0	1.19	1.06	0
hsa-mir-6730-p5_1ss<br>9CG	CAGAGCTGGAG<br>CTGCTCC	1<br>8	15	gp<br>2	...((((((((((((((...((..(......)..))....))))))))))).))))...	1.65	0	0	0	0	0
oan-miR-19b-1-5p_<br>R+4	AGTTTTGCAGG<br>TTTGCATCCCAG<br>C	2<br>4		gp<br>3	((((.((((..((((((((((((((((((((((((((((...(((((.........))))))))))).)).)))))<br>))))))))))..)))...)))).))))	1.65	3.81	2.57	13.1<br>0	19.6<br>3	18.6<br>4
hsa-miR-21-3p	CAACACCAGTC<br>GATGGGCTGT	2<br>1		gp<br>3	.(((((..(((..(((.(((((.(((((.(..(.(.....))..)))))).))))))))..)))..))))).	1.65	0.76	1.28	0	3.18	5.89
cgr-miR-28-3p	CACTAGATTGT	2		gp	.(((.(((((.((((((((((((((((..(((((........)))))......)))))))))))))))).))))).	1.65	0	0.32	1.19	1.06	0.98

	GAGCTCCTGGA	2	3	)))..						
ipu-miR-30d_1ss20T	CTTTCAGTTGGA	2	gp							
A	TGTTTGCAGT	2	3	.((((((((((..(((((((((((((............)).)))))))))))))))))))))...	1.65	0	0	0	0	0
	AGGCAGTGTAG									
cgr-miR-34c-5p_R+	TTAGCTGATTG	2	gp							
1_1ss23CA	AT	4	3	((..((((..(((.((((.(((((.((((((.((....)).))))))))))).)))).))).))))..))...	1.65	0	0	0	0	0
cin-miR-101_L+1R-	GTACAGTACTG	2	gp	.....((.(((((((((.(((((..((.(((...((((.(((((((...........)))))))...))))..))).)						
2_1ss18AC	TGATAACTAT	1	3	)....)))))..))))))))).))	1.65	0.76	7.70	2.38	1.06	1.96
bbe-miR-129a-5p_L-	TTTTTGTAGTTT	1	gp	...(((.....)))..(((((((((((.(((..((((((((.....(((....)))))))))))).))).)))))))						
2R-3_1ss10GA	GGGGCT	8	3	)))))	1.65	0	0	0	0	0
dre-miR-130c-5p_R-	TCCCTTTTTCTG	1	gp	((((((((..((...(((((....(((..(((((((((((.(((((((((..(((((((((.((.............)						
3_1ss1GT	TTGTACT	9	3	).))))))))))..))))))))).)))...))))))))..)))..)))))...)).)))))))))	1.65	0	0	0	0	0
dre-miR-130c-3p_R-	CAGTGCAATAT	2	gp	((((((((..((...(((((....(((..(((((((((((.(((((((((..(((((((((.((.............)						
1_2ss20CG21AC	TAAAAGGGGC	1	3	).))))))))))..))))))))).)))...))))))))..)))..)))))...)).)))))))))	1.65	0	2.57	1.19	1.06	0
mmu-miR-130b-5p_	ACTCTTTCCCTG	2	gp	(((..(..(((..(((((((...((((((((...((...((....))..))))))))))...)))))))..)))..).						
1ss22TA	TTGCACTACA	2	3	.)))	1.65	0	0.64	0	0	0
	TGGCAGTGCAA									
aca-miR-130b-3p_L	TAATGAAAGGG	2	gp	..((((((.(((....(((((((...(((((((((.((((........))))......)))))))))..)))))))...				11.3		
+3R+2	CGT	5	3	))).))))))	1.65	1.14	3.85	1	8.49	0.98
	TTTGGTCCCCTT									
cin-miR-133-3p_L+1	CAACCAGCTGA	2	gp	.........(((((((..(((..((((((.(((.((.((((((((....((.......))..)))))))).))))))..)						
R+2	T	4	3	)))))..)))..))).))))..	1.65	1.53	1.92	0	0	1.96
	ACTCCATTTGTT	2	gp	((((((((((....(..((.....(((((((((((.(((((((.((((((...((((((((((.........)))))						
efu-miR-136	TTGATGATGG	2	3	)))))...))))))))))))))).)))))))).)))...))..))))))))).)).........	1.65	0	0	3.57	1.06	0
aca-miR-137b-3p_1s	TTATTGCTGGA	2	gp	......((((((((((((..(((.(((((((..((((.(((((((.......).))))))))))..))))))).)						
s9TG	GAATACGCGTA	3	3	))..))))))))).))))...	1.65	3.43	6.73	7.74	6.37	0

	G									
tgu-miR-139-3p_R-2<br>_2ss4AG7TC	TGGGGACGCGG<br>CCCTGTTGGA	2<br>1	gp<br>3	...((((((((((.((.((((((((((((...........)))))))))))))).)))).))))))..	1.65	2.67	1.60	5.36	7.43	2.94
eca-miR-139-3p_L+<br>1R-2_1ss4AG	TGGGGACGCGG<br>CCCTGTTGGA	2<br>1	gp<br>3	((((((((((.((.((((((((((((.((....))..)))))))))))))).)))).))))))	1.65	2.67	1.60	5.36	7.43	2.94
xtr-miR-140_L+1R+<br>1_1ss22GC	CAGTGGTTTTAC<br>CCTATGGTACC	2<br>3	gp<br>3	..((((((((((((.((.((((((((((((((.(((((((..(((......))))))))))).))))))))))))<br>)))..))))))))))))))..	1.65	1.53	8.34	0	2.12	2.94
rno-miR-143-5p_R+<br>4	GGTGCAGTGCT<br>GCATCTCTGGT<br>AAG	2<br>5	gp<br>3	(((((((..((.((((((..((((((.(((((((((.((((((.((((......)).)).)))))).)))))))))<br>).)))))))..))))))))..)))))))...	1.65	0	0.64	1.19	0	0
pma-miR-144-3p_R-<br>2_1ss18GA	TACAGTATATA<br>TGATGTACT	2<br>0	gp<br>3	..((((.(((((.((((((((.((((((((((.(((....)))....))))))))))))))))))).))))).))<br>)).	1.65	0.25	0	0	0	0
rno-miR-184_R+2	TGGACGGAGAA<br>CTGATAAGGGT<br>AA	2<br>4	gp<br>3	(((((.((((((((((((((((..(((((((((......)))).)))))..))))))))))))))))).))))).<br>...	1.65	1.53	1.28	0	1.06	0
ola-miR-192-5p	TGACCTATGAA<br>TTGACAGCCA	2<br>1	gp<br>3	........(((.(((((((..((.(((((((((((((((.(((.............))).)))))))))))).))))).)<br>)..)))....)))).)))..	1.65	0	0.32	0.60	1.06	0
bta-miR-200c	TAATACTGCCG<br>GGTAATGATGG<br>A	2<br>3	gp<br>3	((((((((((.((((((((.(((.(((.......)))))).)))))))).)))))).))))....	1.65	0	2.57	2.38	1.06	3.92
aca-miR-203-3p_L-1<br>R+2	TGAAATGTTTA<br>GGACCACTTGT<br>T	2<br>3	gp<br>3	.....((..(((((((((((..(((((((((.((((.((((.(((((.........))))))))).)))).)))))<br>))))..)))))))))))...))	1.65	1.53	1.28	0	0.53	0.98
bta-miR-208b	ATAAGACGAAC<br>AAAAGGTTTGT	2<br>2	gp<br>3	.(((((((..((((((((((.(((..((((((((.......))))))))..))).))))))))))..)))))))..<br>.	1.65	0	0	0	0	0
sha-miR-216b_R+1_	AAATCTCCGCA	2	gp	........((.((((.(((((((((.......((((((((((.((.((((.((..((....))...)))))).)).)	1.65	0	0	0	0	0

1ss8TC	GGCAAATGTGG	2	3	))))))))))))))))).)).)))))).(((.((((...(((........)))......)))).))).....						
	TTATTGAGCAC	2	gp	((((((((((..(((.(((((((((((((..(.((((...))))..)..))))))))))))).)))..)))))))						
cgr-miR-325-3p_L-1	CTCCTATCA	0	3	)))	1.65	0	0	0	0	0
	TTTTTCATTATT	2	gp	...((((.((((((((.((((((((.((((((((.((......)).)))))))).)))))))).)))))))).						
hsa-miR-335-3p	GCTCCTGACC	2	3	)))).............	1.65	0	0	0	0	0
	AACAATATCCT									
aca-miR-338-5p_R+	GGTGCTGAGTT	2	gp	....((..((((((((((.((((((((((((((((((.....)))....))).))))))))).))).))))))))..						
1_1ss22GT	G	3	3	.)))..)).	1.65	0	0	0	0	0
	TCCAGCATCAG									
aca-miR-338-3p_R+	TGATTTTGTTGA	2	gp	....((..((((((((((.((((((((((((((((((.....)))....))).))))))))).))).))))))))..						
4	AT	5	3	.)))..)).	1.65	0	1.28	0	0.53	1.96
	TCCCTGTCCTCC	2	gp	..(((((((((...((..(((((.((.(((.(((((((((....)))..)))))).))).)).))))).))....))						
rno-miR-339-5p_R-2	AGGAGCTCA	1	3	))))))))............	1.65	1.53	1.92	6.15	1.59	0.65
	TCCCTGTCCTCC	2	gp	.(((((((.((((....(((((.((.(((.((((((..((....))..)))))).))).)).)))))....)))).)						
hsa-miR-339-5p_R-2	AGGAGCTCA	1	3	))))))..........	1.65	1.53	1.92	6.15	1.59	0.65
aca-miR-365-3p_2ss	NAATGCCCCTA	2	gp	(((((.((((((((..(((.(((((((((((.(((.....))).))))).....))))))))))..)))))))).)						
1TN22TC	AAAATCCTTAC	2	3	))))	1.65	0.38	1.60	1.19	0.53	0
	AACATAGAGGA	2	gp	(((((((((.((((.(((((..((((.((((((((((.............)))))))))))))..))))).))))						
hsa-miR-376c-3p	AATTCCACGT	1	3	.)))))))))	1.65	0	0	0	0	0
mmu-miR-434-5p_L	AGCTCGACTCA	2	gp	.........(((((((((((.((.((((...((((((..((((((..........))))))..))))))...)))))).						
+1R-1	TGGTTTGAACC	2	3	.)))))))))))...	1.65	0	0	0	0	0
	TTTGAACCATC	2	gp	.........(((((((((((.((.((((...((((((..((((((..........))))))..))))))...)))))).						
mmu-miR-434-3p	ACTCGACTCCT	2	3	.)))))))))))...	1.65	0.76	0	0	0	1.96
	TTTTGCGATGTG	2	gp	........(((((.(((((((...(((((..((((((((((((..(.......)..))))))))))))..)))))...))						
bta-miR-450a	TTCCTAATAT	2	3	))))).))))))...	1.65	0	3.85	7.15	5.30	7.85
tni-miR-455_L+1_2s	GTATGTGCCCTT	2	gp	((.(((...(((.((((((((((.((((((.(((.(((........))).))).)))))).)))))))))).))).	1.65	2.29	1.28	0	1.06	0

s22CT23GT	GGACTACATTT	3	3	..))).))						
	GGAGAAATTAT	2	gp	((((((.((((((((((.((((((.((((((((((.......)).)))).)))))))))).)))))))).))))						
bta-miR-539	CCTTGGTGTGT	2	3	))))	1.65	0	0	0	0.35	0
	GGAGAAATTAT	2	gp	(((((..(..(((((((.((((((.((((((((.((.......)))))).)))))))))).)))))))..)..)))						
hsa-miR-539-5p	CCTTGGTGTGT	2	3	))	1.65	0	0	0	0.35	0
	CAAAACTGGCA	2	gp	.......((((((((....((((((((((((((.(((((..((((((((....))))))))))))))..)))))))						
hsa-miR-548a-3p	ATTACTTTTGC	2	3	))))))))....)))))))).	1.65	0.25	0	0	0	0
hsa-miR-548az-3p_L										
+1_4ss6AC7CT8TG	CAAAACTGGCA	2	gp	.....(((...)))..((..(((((..((((((((((((((((((...(((((.((.((....)).)).)))))...						
14CT	ATTACTTTTGC	2	3	))))))))))))))))))...)))))..))..	1.65	0.25	0	0	0	0
hsa-miR-589-5p_R+	TGAGAACCACG	2	gp	.((((.(((......(((((..(((.(((((.(((.((.((((((((.(((.....)))))))))))).)).))).)..)))						
1	TCTGCTCTGAGC	3	3	)).)))..)))))))))))))..	1.65	0	0.64	0	0	0
	TACCCATTGCAT	2	gp	((.(.((((...((((..(((((.....(((.((((((..((((.(((...........))).))))..)))))))))						
efu-miR-660	ATCGGAGTTGT	3	3	......)))))..)))).))))).)).......	1.65	0	0	1.19	1.06	0
mmu-miR-704_L-1R	GAGATGTGCTC	1	gp							
-2_1ss4CG	TGCTCCT	8	3	.((((((((.((((.(..((.....(((..(((.....)))))).))..).))))))))))))..............	1.65	0	0	0	0	0
dre-miR-732_R-4_1s	CCCAAAGCAGA	1	gp	(((((...((((((((((((((...((((((((..(((...........)))..))))))))...))))))))))))))						
s2TC	GAACTCT	8	3	...)))))	1.65	0	0	0	0	0
cgr-miR-744-5p_R+	TGCGGGGCTAG	2	gp	((((.(((((..((((.((((((.((((((((.((.....)).((((((...))))))).......))))))))))).)			14.1	51.2	24.4	
1	GGCTAACAGCA	2	3	)).))))).))))).))))...	1.65	4.58	1	1	0	0
	AAGGTTACTTG	2	gp	(((..((.((.(((((((.((((((((((((...............))))))))))..)))))))))).)).))..)				15.4		
cgr-miR-872-5p	TTAGTTCAGG	1	3	))	1.65	0	3.21	8	6.37	0
hsa-miR-873-5p_L+	TGCAGGAACTT	1	gp							
1R-3	GTGAGTCT	9	3	...(((....)))((((((..(.(((((((((.((.......)).))))))))).)..)))))).(((....)))....	1.65	0.76	0	0	0	0
bta-miR-2369_L-2R-	GGGTTGTGGGT	1	gp							
1_1ss3AG	TTTTGTT	8	3	((((((.((((((...(((.........))).(((..(((....)))..)))......))))))))))))	1.65	0	0	0	0	0

bta-miR-2374_L-1R-2_1ss15AT	TGGGGCTGGGGAGTGGCG	18	gp3	((((.(((..((....(((((..(..((..((.(((....)))))..)).)..)))))..))..))).)))).....	1.65	0	0	0	0	0
mmu-miR-3059-5p	TTTCCTCTCTGCCCCATAGGGT	22	gp3	((..((.(((((...(((((((..(((.(((((.((((......)))).)))))))).))))).))..))))).))..)).	1.65	0	0	0	0	0
hsa-miR-3184-3p_L-1R-4_1ss9GT	AAGTCTCTCTCTCTGCCC	18	gp3	(((..((.(((((((((...(((.(((((((((.........))))).)))).))).)))))))))).))..)))	1.65	0	0	0	0	0
rno-miR-3549_R+3_1ss5TC	ATCTCGCCCGCAAAGACCCAGC	22	gp3	(((....(((((..(((((((.(((..(((((.(((((..(((.((.(((..........))).)).))).)))))))).)))))..))).)))))))...))))..))..))).	1.65	4.58	1.92	1.19	2.12	0
rno-miR-3589_L-1R-3_1ss12CA	AGGAAACCAGAAAGTGTT	18	gp3	((((..((.((((((.(((.((((...(((..((((.(((((((((.(((.....))).......))))))))).)))).))).))))....)))))))))))).)).....))))	1.65	0	0	0	0	0
hsa-miR-3679-5p_L-2R-3_1ss20GC	AGGATATGGCAGGGAAGC	18	gp3	((((((((((((.(((..((((((((((...........)))))))))).)))...))))))))))))	1.65	0	0	0	0	0
mmu-miR-3964_L+1_1ss6GA	CATAAAGTAGAAAGCACTAAA	21	gp3	.(((((((((((....(((((.((((....((.....))...)))).)))))....)))))))).......))))	1.65	1.53	1.28	0	0	0
hsa-miR-4426_R+1_1ss10CG	GAAGATGGAGGTACTTTG	18	gp3	.....((((((((((...((((..................))))..))))).)))))......	1.65	0	0	0	0	0
hsa-miR-4446-3p_1ss22TG	CAGGGCTGGCAGTGACATGGGG	22	gp3	.((..((((.((.((((((...((((((.............)))))).)))))).)).))))..)).	1.65	0	0	0	0	0
hsa-miR-4448_R-2_1ss6CG	GGCTCGTTGGTCTAGGGG	18	gp3	.((....(((...((((..(((...(((((((((.....((((.....))))....))))))))))))).))))...)))....)).	1.65	4.58	1.28	0	1.06	0
hsa-miR-4538_L-3R-1_1ss21GT	CTTGGATGAGCTGGGCTT	18	gp3	.((((..(((.((((((.((.((((..(((..((........))..)))..)))).)))))).)).)))..))))...	1.65	0	0	0	0	0
hsa-miR-4647_L-3R-2_1ss5AG	GGTGGTGCTGTGCTGAGG	18	gp3	(((((((..((.((.(((((..((((((.((..((((.........)))).))))))))))))).))..))..))))))))	1.65	0	0	0	0	0
hsa-miR-4677-3p_R-3	TCTGTGAGACCAAAGAACT	19	gp3	((.((((((.....((((((((((((..(((.((.((........)))).)))..))))))))))))....)))))).))	1.65	0	0	0	0	0

mmu-miR-5112_L+2R+2	CGTAGCTCAGCGGGAGAGCACCT	23	gp3	(((.(..((((((((....((((((((....))))..)))).))))))))..)....)))	1.65	0	0	0	1.06	0
mmu-miR-5116	TTTGATAGGAACCCCGCCTGA	21	gp3	.(((((((((((((((.((((((..(..........)..)))))).)))).))).))))))))	1.65	0	0	1.19	0	0
mml-miR-6134_R+4_1ss18GT	TGAGGTAGTAGGATGTATAGTTA	23	gp3	.((((((((.......)))))))).(((((((((((((.(((((((.((((((.((((.........)))))))))...))))))).)))))))....))))))...	1.65	0.87	1.28	1.19	2.12	0
hsa-miR-6719-3p_L-1R-2_1ss6CA	CTGAAATCAGTGATTCTCC	19	gp3	..........((((((((.(((((((((....((((((((.....))).)))))....))))))))..)))))))).	1.65	0	0	0	0	0
hsa-miR-6760-5p_L-5_1ss16AG	AGAAGGTGGAGGTGCAGA	18	gp3	.......(((((((((.((....((((.((((((.........)))))).)))))))))))))))...	1.65	0	0	0	0	0
hsa-miR-6862-5p_L-2R-2_1ss10TC	GGCATGCCGGGAGAGACT	18	gp3	...((.((((..((((((.(((.(((((.((......))))))).))).))))))......)))).))..	1.65	0	0	0	0	0
mmu-miR-7118-3p_L-2R-2_1ss9CA	ACTCTAAGTCCTCCTCCA	18	gp3	.....((((((.((((..((((..................)))).)))).))).)))...	1.65	0	0	0	0	0
ssc-miR-7136-5p_L-2_1ss7CG	TGGTGCAGACACTGTGGAGC	20	gp3	((((((.(((((((((.((((.((..........)))))).))))))))).))))))...	1.65	0	0	0	0	1.96
mmu-miR-7222-3p_L-2R-3_1ss12GT	CAGGACAGTTGGCAGGAG	18	gp3	.((.((((((.....(((..(((((....((((((......).)))))....)))))..)))..)))))).))..	1.65	0	0	0	0	0
mdo-miR-7307-3p_L-2R-2_1ss8TC	AATGGCCATTTCTGAGCT	18	gp3	((((((((.((.((..((((((((.....)))).......))))...)).))..))))))))..	1.65	0	0	0	0	0
mdo-miR-7385b-5p_L-3_1ss13CG	TCTGACAGTGTATCTTCC	18	gp3	((((((((.((.((((.((((..(((....)))......)))).)))).)).))))))))..	1.65	0	0	0	0	0
mmu-miR-7661-5p_L-3R-4_1ss15GC	AAAGAAACCTGCAGTTTA	18	gp3	.(((.((((((..((((((.......((....)).......))))))..)))))).)))..	1.65	0	0	0	0	0

eca-miR-9095_L-2R-5_1ss3AC	CAGGACAAGTTGGTTTCT	18		gp3	...(((((((((..(((((((((((......((.(((((((((....((((((((.....(((((((((....))).))).)))...))).)))))....))))).)))).)).))).)))..))).)))))...))).))))))....	1.65	0	0	0	0	0
eca-miR-9104_R-3_1ss4AC	CTGCCCTGAGGCCTCTGCT	19		gp3	...((..(((((((..((.(((....((((((..((.(((((((((((...(((((((.((((.......)).)).))))))))...))).))))))).)).))))))))).)))))..)))...))))..)).......	1.65	0	0	0	0	0
eca-miR-9109_L-1R-3_1ss4CG	AAGTGTGCCCGTGGGGAGAAC	21		gp3	(((((.....(((.....((((((((..(..((((((((...(((.(((((((....(((...((((..((((.....))))...))))))).)))))))......)))...))))))))....)..)))))))))))..)))))	1.65	0	0	0	0	0
efu-miR-9265_L-7_1ss15GT	AGAGGCTTTTAGGAGACA	18		gp3	(((..(((.((((((((((.......((..(((((((((.((((((....(((..((((..((..(.((........)).)..)).)))).)))...))))))))))...)))))..))......)))))))))))..)))....	1.65	0	0	0	0	0
pma-let-7b-5p_R-3_1ss15TG	TGAGGTAGTAGGTTGTGTA	19		gp3	...(((..((((((((((((.((((((((((((((((((...))))))).)))........)))))))).)))))))))))..)))...	1.65	0.11	0.64	0	1.06	0
PC-5p-479025_5	AAGAGCTCATCAGATATC	18	7	gp4	((((.((....((((..((((....(((..(((.((((((((((((..((..(((((((.(((..((.....)).))))))))).))..))....))))))))))).))..)))..)))........(((((.........))))).))))..))))))))).......	1.65	0	0	22.63	20.16	31.40
PC-3p-82903_44	TAGCAGTAAAGTGAGATC	18	5	gp4	...((((((((..((((..(((..((((((((((((((.....(((((((.....(((..((((((.(((((((...))))).)).)))))).)))..)))))))).......)).))))).)))))..))..)))..))))))))).)))...	1.65	0	0	2.38	6.37	23.55
PC-3p-68190_58	CTGATGAGGATCTTAAAA	18	1	gp4	...((((.((((((((((((.((..((((...(((.................))).))))..)).)))))))))))).....))))...	1.65	0	0	13.10	10.61	13.74
rno-mir-122-p3	AAACGCCATCATCACACTAA	20	JH375727.1	gp2	...((((((((((((..(((((((.((((((((((((...........)))))))))))).))))))))..)))))).)))))...	0.82	0	0.64	0	0	1.96
aca-miR-142-5p_L+4R-1	GATCCATAAAGTAGAAAGCACTACT	25		gp3	..(((((..((((((((((((((((((.((((((((((.....((....))......)))))))))).)))))))))))))))))))..))))).	0.82	0.38	0.96	0	0	0
efu-miR-185_R-1	TGGAGAGAAAGGCAGTTCCTGA	22		gp3	...((((.((.((((((((...(..(((((((.(((((((..(((.(((((.(((..((((..((......)).)))))..))).)))))))))..)))))))))))))).....)..)......))).))))))).))))...	0.82	1.14	4.81	13.70	6.90	2.94
cgr-miR-185-5p	TGGAGAGAAAGGCAGTTCCTGA	22		gp3	..((((((((..(((.(((((.(((..((((..((......))))))..))).))))))))..)))))))).....	0.82	1.14	4.81	13.70	6.90	2.94

rno-miR-210-3p_R+1_1ss21GT	CTGTGCGTGTGACAGCGGCTTAT	23	gp3	..((((...)))).(((((..(((..(((((.(((..(((((((((.((.(((......))).)).))))).)))).)))).)))))..)))..)))))............	0.82	0.38	2.24	2.98	4.77	0
hsa-miR-219b-3p_R+1	AGAATTGCGTTTGGACAATCAGTT	24	gp3	((.(((..(((((.((((((((((...((((((((((....)))............)))))))...))))))).)).)))))..)))))	0.82	0	0.96	0	0	0
mmu-miR-330-5p_R+1	TCTCTGGGCCTGTGTCTTAGGCT	23	gp3	........((((....((((((((((((((((((.(((..((((.............)))).))))))).))))..))))))))))))))......	0.82	0	0	0.60	0.53	0
rno-miR-339-3p_R-1	TGAGCGCCTCGACGACAGAGCC	22	gp3	..(((((((((...((..(((((.((.(((.(((((((((....)))..))))))).))).)).))))).))....)))))))))..........	0.82	0	1.28	10.12	2.12	0.98
ptr-miR-339	TGAGCGCCTCGACGACAGAGCC	22	gp3	.(((((((.((((....(((((.((.(((.((((((..((....))..)))))).))).)).)))))....)))).)))))))..........	0.82	0	1.28	10.12	2.12	0.98
hsa-miR-374a-3p	CTTATCAGATTGTATTGTAATT	22	gp3	((((..(((.((((((((((((.(((((((((((....))))))))))).)))))))))))).)))..))))	0.82	0.38	0	0.60	0.53	0
ggo-miR-374a_R+1	CTTATCAGATTGTATTGTAATT	22	gp3	.....((((((.((....))..........((((..(((.((((((((((((.(((((((((((....)))))))))))).)))))))))))).)))..))))..))).))).	0.82	0.38	0	0.60	0.53	0
cgr-miR-409-3p	GAATGTTGCTCGGTGAACCCCT	22	gp3	.((((((((.((((..((((((((((((..((.....)))))).))))))))))..)))).)))))))))....	0.82	0.38	0	0	0	0
hsa-miR-409-3p	GAATGTTGCTCGGTGAACCCCT	22	gp3	.((((((.((..(((.((((..((((((((((((..((.....)))))).))))))))))..)))).)))..))))))).	0.82	0.38	0	0	0	0
bta-miR-423-3p_L-1	AGCTCGGTCTGAGGCCCCTCAGT	23	gp3	....((((((..((((((((((((..(((.((((.(((((.........))))))))).)))...))))))))))))..)))))).........	0.82	1.14	2.89	7.15	5.30	0.98
bta-miR-424-5p	CAGCAGCAATTCATGTTTTGA	21	gp3	...((..((((((((((.(..((((((..(((((((((((((((.......))..))))))))))))))..))))))..).))))))).)))..)).	0.82	0.38	0	1.19	1.59	0
cgr-miR-505-3p	GTCAACACTTG	2	gp	.((((((.(((((((((((.((((.((((((((...........)))))))).)))).))))))))))).)))).	0.82	0	1.60	1.79	1.06	0.98

	CTGGTTTCCTCT	3		3	))..						
	TTACAGTTGTTC	2		gp	........((..(((((((....((((((((((((((((((.............)))))))))))))))))))....						
ggo-miR-582_R+1	AACCAGTTACT	3		3	))))))))..))........(((......)))..	0.82	0.38	0	2.38	0.53	0
	TTACAGTTGTTC	2		gp	..((.((...((((((((((((...(((((((((((((.(((((............))))).))))))))))))).						
hsa-miR-582-5p	AACCAGTTACT	3		3	..))))))))))))...))))...	0.82	0.38	0	2.38	0.53	0
	AATGGCGCCAC	2		gp	......((...((((((((((((((((((.....(((((((((((............))))))))))).))))))))						
bta-miR-652_R+1	TAGGGTTGTGT	2		3	))))))))))...)).....	0.82	0	2.24	7.15	4.77	0.98
cgr-miR-652-3p_R+	AATGGCGCCAC	2		gp	.((((((((((((((((((.....(((((((((((..((.....))..))))))))))).)))))))))))))))						
1	TAGGGTTGTGT	2		3	)))..	0.82	0	2.24	7.15	4.77	0.98
	TGAGACCTCTG	2		gp	..........((((((((.(..(((((((.(((.((..((((.(((........)))))))..)).))).)))))))..						
ggo-miR-769_R+1	GGTTCTGAGCT	2		3	).)))))))))((..((....))..))......	0.82	0	0	0	0.53	0
	TGAGACCTCTG	2		gp	...(((((((((((..(.((.....((((.(((((((.(..(((((((.(((((((.((((..((.......))..))						
hsa-miR-769-5p	GGTTCTGAGCT	2		3	))))))))))..))))))))..).))))))))))))))..)..))))...))).)))))........	0.82	0	0	0	0.53	0
	NGGACTGTCTA										
oan-miR-1388-5p_R	ACCTGAGAATG	2		gp	....((((((((((.((..((.(((...(((((((((....((((........)))).)))))))))...))).))..						
+1_1ss1AN	T	3		3	)).))))))..)))))..	0.82	1.14	2.24	0	0	0
	CAACAAATCAT	2		gp	...((((((...((((((((.((.((.((((.(((((((((((...........))))))))))))))))).)).)						
tgu-miR-7-4-3p	AGCCTGCCATA	2	28	2	))))))))..)))))))...	0	0	0.64	1.19	1.06	3.92
hsa-miR-20b-5p_L-5	TGCTCATAGTG	1		gp							
R+1	CAGGTAGT	9	4	2	...(((((..(((..((((((((...(((((................))))).))))))))..)))..)))))...	0	0	0	0.60	0	0
mmu-mir-30f-p5_1ss	TGTAAACATCC	1		gp	((((((....((.((((((((((((((((((((.((((......(((....)))..)))).)))))..))))))))))						
17AG	GACTGGA	8	23	2	))).))..)).))))...	0	0	0	1.19	1.06	0
	AGGCAAGATGT										
hsa-miR-31-5p_R+2	TGGCATAGCTG	2		gp	...(((((.((((((((((.(((..(..(((((.((((((((((((((.(((........)))..)))))))))))))						
_1ss11CT	A	3	Z	2	.))))))..)..))).))...))))))).))))))...	0	1.53	1.28	1.19	4.24	0
hsa-miR-31-3p_R+1	TGCTATGCCAA	2	Z	gp	...(((((.((((((((((.(((..(..(((((.((((((((((((((.(((........)))..)))))))))))))	0	0	0.64	2.38	1.06	0

	CATATTGCCATC	3		2	.)))))..)..))).))...))))))).)))))...						
bbe-mir-92a-1-p5_1s	GAAGGTAGTCA	1		gp	...((((((((....(((.(((((((..((....((((...(((.(((((..(((((.......))))))))))).)))						
s5TG	TGACAAG	8	5	2	.))))....))......)))))))...)))).....))))))))...	0	0	0.64	0	0	0
hsa-miR-103b_L-2R	ATAGCCCTGTA	2		gp	...((((((.(((((..(((.((.((((.((((((((....((....))...)))))))).)))).))..)))))))						
+1	CAATGCTGCTT	2	4	2	).))))))...	0	0	0	0	0.53	0
	CTTTCTGACTCT	2		gp	.......(((((((((((..(((.((((((((..(..(.(((((((.....)).))))))..)..)))))))).)))						
oan-mir-137a-p5	CTTCGGTG	0	8	2	..)))))))))))...	0	0	0.64	1.19	0	0
tgu-miR-155-3p_1ss	CTCCTACATGTT	2		gp	...(((((((..((.((((.((((..((((((((((((.((((.((((((((((((....)))).))))))))))						
9CT	AGCATTAACA	2	1	2	)))))))))))))))..))))...))))..))..))))))))...	0	0	0.64	0	0	0
	TGAATTACCAT										
ssa-miR-183-3p_R+	AGGGCCATAAA	2		gp	(((..........(((......................)))(((.((((....))))))))))).(((((...((((..(((						
3	T	3	1	2	((((((.(((((..((((((.((.......))...))))))..)))))))))))))))..))))....)))))...	0	1.53	0	0	1.06	0
rno-miR-190b-5p_R-	TGATATGTTTGA	2		gp	...((((((.((((.(((((..(((((((..(((((((.......)))))......))..)))))))..))))).))						
1	TATTAGGT	0	1	2	)).))).))))...	0	1.14	0.64	0	0	0
hsa-miR-196b-5p_R-	TAGGTAGTTTCC	2		gp	..(((((....((..((.((((((((((.((((((.((((...........)))).)))))).)))))))))).)).						
1	TGTTGTTGG	1	2	2	.))..)))))..	0	0	0	0.60	0	0
tgu-miR-203-5p_R+	AGTGGTTCTTA	2		gp	...(((..(((((..((((..(((((((((.((((.((((.(((((.........))))))))).)))).))))))						
1	ACAGTTCAACA	2	5	2	)))..)))))..))))))..)))...	0	0	0.64	0	0	1.96
oan-miR-204-3p_L+	GCAGGGACAGC	2		gp	...(((((.....(((...(((((.(((((((((..((((.(((((.............)))))))))..))))))))						
1_1ss21AC	AAAGGGATGC	1	10	2	).))))))...))))))))...	0	0	0	1.19	1.06	9.81
tgu-miR-218-3p_R+	AAACATGGTTC	2		gp							
1	TGTCAAGCACC	2	13	2	.((...(((((((((..((((((((....................))))))))..)))))))))...))	0	0	0.64	0	0	1.96
			AADN								
	CCTCTGGGCCCT	1	030100	gp	...((((((((.(((((..((....((..((.(((.(((......))).)))))...))..))..))))).)))))))).						
hsa-miR-326_R-1	TCCTCCA	9	64.1	2	..	0	0	0	1.19	0	0
aca-miR-375-5p_R+	GCGTCGAGCCC	2	7	gp	...(((.((((((((..((.((((((..(((..((((((...........))))))..)))..))))))))..)))))	0	0	0	3.57	1.06	0

1_2ss16CT17AG	CACGTGCAAGA	3		2	))).)))...						
	C										
sha-mir-497-p3_1ss1	TGGTTTGTTTAG	1		gp							
0CT	TGTTGTG	9	2	2	...(((((((((.((((..(..(((.....(((......)))......)))..)..)))).))))))))))....	0	0	0	0	1.06	1.96
eca-mir-1271b-p5_1s	AAAATAGCTCT	1		gp	...((.((((((((((..(((((.((((......(((((((((((((((........(((((......))))))))						
s9AT	GATCAGT	8	15	2	))))..))))))))).)))))))))..........))))).))))))).	0	0	0	0	0	1.96
bfl-miR-2056_L-1R-	TTCACTGTAGCT	1		gp							
2_1ss12AC	CTACCTG	9	25	2	.((((((..((((.((.((((....(((.................))).)))).))))))..)))))).	0	0	0	1.19	0	1.96
tgu-mir-2976-3-p5_1	GCGGCGGTGGG	2		gp							
ss5AC	AGCGGAGCG	0	6	2	((.((((.((((((((((..((((((((....))))))))..).))))))))).)))).))	0	0	0	0	0	1.96
tgu-miR-2984-3p_1s	AATTTCACTCAC	2		gp	...((((((((((((.((((((((.(((((((((((.......((.......)).....))))))))))).)))))))						
s16GA	AGCAGGCACTT	3	1	2	)).).)))))))))))...	0	0.76	1.28	0	1.06	0
hsa-miR-3529-3p_L-	ACAAAATCACT	2		gp	...(((((((.(((((..((.(((((((((.(((((((.(((((.....)).))).)))))))..)))).))))).						
4R+2	AGTCTTCCAGA	2	10	2	))..))))))..)))))))...	0	0	0.32	0	0	2.94
rno-miR-3570_L-2R	TACAATCAACG	2		gp	...((.((((((..((((..((.(((((.(((..((...(((.((((((((((.((((((............)))).						
+1	GTCGATGGTT	1	8	2	)).)))))..)))))).)))...))..))))))))))).........))))....))))..))))...	0	0	0.64	0	0	0
	GTTTTGTTTTGT	2		gp							
mmu-mir-5098-p5	TTTGTTTTT	1	1	2	((((((...((((((((.((((((......................)))))).))))))))...))))))......	0	0	0	1.19	1.06	1.96
efu-mir-9206-p3_1ss	GGCAGCCGGCT	1		gp	...((((.((.(((((((((.((..(((.(.(((..(((.......)))..)))).)))..)).))))))))).)).						
6GC	TCTTCAT	8	4	2	))))...	0	0	0	0	0	1.96
xtr-let-7e_R-3_1ss19	TGAGGTAGTAG	1		gp	...(((((((((((((.((((.....((...((.(((......)))...))..))...))))))))..)))))))).						
AT	GTTGTTTT	9	4	2	..	0	1.53	0.64	0	0	0
	TCTTTGGTTATC										
	TAGCTGTATGA	2		gp	.((((((.((((((((((((((((.((((.((........))..)))).))))))))))))))))))))).......						
bfl-miR-9-5p_R+3	TA	5		3	......	0	0.76	1.28	1.19	0	0
aca-miR-10c-3p_R+	GCAAATTCGTC	2		gp	.(((((..(((((..((((.((((..((((((((((((((...))))..))))))))))..))))))))..))))	0	1.53	0	0	0	0

2_1ss1AG	TCTAGGGGAAT	2	3	)..)))))......						
bbe-miR-10b-5p_L-1	ACCCTGTAGAT	2	gp	..((((..(((.(((((((.((.(((((..((((.((((((((.............)))))))).)))).))))))						
R-2_1ss9GA	CCGATCTTGT	1	3	).))))))).)))...))))....	0	2.29	1.92	0	0	0
aca-miR-15a-5p_1ss	TAGCAGCACAT	2	gp	.(((((((((.((..(((((((.((.(((((((((((((........))))))))))))).)).)))))))..))						
12CA	AATGATTTGT	1	3	.)))))))))	0	0.76	1.60	1.19	1.06	0
ola-miR-15a_R+2_1	TAGCAGCACGT	2	gp	((..(((((....)))))..(((((..(((((((((.((((..(((...............)))..)))).)))))))						
ss11GT	AATGGTTTGTG	2	3	))..))))))..))..............	0	0	0.32	0.60	0	0
hsa-miR-16-2-3p_L+	ACCAATATTAC	2	gp	((..((((..(((((((((..((((((((.((..(((........))).)).))))))))..)))))))))..)))						
1R-1	TGTGCTGCTTT	2	3	)..))	0	0	0.64	0	1.06	1.96
	AGGCGGAGACA	2	gp	((((.(..(((..((((.((((((.((((((((.(((((.......))..)))))))).)))))))).)))).)..)))						
rno-miR-25-5p	CGGGCAATTGC	2	3	..).))))	0	0	1.92	2.38	1.06	0
	CCTATTCTTGAT	2	gp	((((((..(..((..(((((((((.((((((((((..............)))))))))).)))))))))..))..)..						
hsa-miR-26a-2-3p	TACTTGTTTC	2	3	))))))	0	0	0	1.19	0	0
	AGGGCTTAGCT	2	gp	((((.(((((.(..((((((..(.((((((((..((.....))......)))))))))..))))))..).))))))..				15.4		
cgr-miR-27a-5p	GCTTGTGAGCA	2	3	))))	0	0	1.28	8	4.24	5.89
	AAGGAGCTCAC	2	gp	.(((.(((((.((((((((((((((((..((((((........)))))......)))))))))))))))).))))).						
cgr-miR-28-5p	AGTCTATTGA	1	3	))))..	0	0.76	0.64	2.38	0	2.94
aca-miR-30d-3p_R+	TTTCAGTCAGAT	2	gp	..(((((((.((.(..(((((((((...((((((((((((.......)))))))))))).)))))))))..).)).						
2	GTTTGCTGCTA	3	3	)))))))..	0	0.76	4.49	2.38	5.30	0
oan-miR-30f_R-1_1s	TGTAAACATCC	2	gp	(((((((((((((((..((...(((((((...((((((((.(((((..(((.....))).)))))).........)))						
s19CA	TCCCCTCAGC	1	3	)..)))))..))))).))))..)).))))))))..))))).))))	0	0	0.64	0	1.06	0
	AGGCAAGATGC									
aca-miR-31-5p_R+2	TGGCATAGCTG	2	gp	..........(((..(..(((((.((((((((((((((.................)))))))))))))).)))))..)..))						
_1ss11TC	A	3	3	)..	0	0	0.32	0	0	0
	TATTGCACATTA	2	gp							
eca-miR-32_R+3	CTAAGTTGCAC	5	3	((((((((..((((.(((((((((((((((...((....))....)))))))))))))))))..))))))))	0	0.76	1.28	0	0	0

	CT									
	CAATTTAGTGT	2	gp							
hsa-miR-32-3p_R-1	GTGTGATATT	1	3	((((((((..((((.((((((((((((((...((....))....))))))))))))))))..))))))))	0	0	0	1.19	0	0
	ATCAGCAAGTA									
mmu-miR-34a-3p_L	TACTGCCGTAG	2	gp	.....((((..((((((..(((((((((((.((((((((((..((.........))..))))))))))))).)))))						
-1R+3_1ss20CG	T	3	3	)))..)))).))..))))...	0	0	0	0	1.06	0
csa-miR-92c_R-1_1s	TATTGCACTTGT	2	gp	.......(((..(((((.((((((.....((((((..(((((..((....))..)))))))))))).....)))))).)						
s9CT	CCCGGCCGA	1	3	))))..))))....	0	0.76	0.64	0.60	0	0
	TATTGCACTTGT									
bbe-miR-92d-3p_R+	CCTGGCCTGTC	2	gp	...((((((((((((((((((((.((((..((((((...((...))..))))))..))))))))))))))))))))						
2_1ss11AG	A	4	3	)))))...	0	0	0.32	0	0	0
cin-miR-92c-3p_1ss	TATTGCACTCGT	2	gp	.................((((...((((.((((((.(((((.(((((.((((..........)))).)))))))).)).						
18TC	CCCGGCCTAT	2	3	)))))).))))..))))....	0	0.76	0	0	0	0
	ACTGCTGAGCT									
	AGCACTTCCCG	2	gp	((((((((((..((((((((((((.((((.(((.((.........))))))))))))))).)))))))...)))))						
hsa-miR-93-3p_R+1	A	3	3	)))))	0	0	0.64	4.76	4.24	0
	TTCAACGGGTA	2	gp	..(((((.(((.((((((((((((..(((((((((.((.....)).)))))))))..)))))))))))).))))..						
bta-miR-95_R-1	TTTATTGAGC	1	3	.)))))	0	0	0.64	0	0	0
rno-miR-101b-3p_R	TACAGTTCTGTG	2	gp	.........((.((((((.((((((((((((((..((......((((((.((.....)).))))))))..))))))))						
+1_1ss7AT	ATAGCTGAAG	2	3	)))))).)))))))..))..	0	0	0	0	1.06	0
	AGCAGCATTGT									
pma-miR-103a_1ss9	ACAGGGCTTGG	2	gp	.((((..((((((((.((.(((((((((((.(((...............)))))))))))))).))))))).)))))						
CT	A	3	3	)))...	0	0	0.64	0	0	0
	TAAAGTGCTGA	2	gp	(((((((((((..((((((..((((((((.....(((((.......))))).))))))))..))))))...)))))))				28.5	18.5	
bta-miR-106b	CAGTGCAGAT	1	3	))))	0	3.05	8.34	8	7	1.96
hsa-miR-106b-3p_R-	CCGCACTGTGG	2	gp	(((((((((((..((((((..((((((((.....(((((.......))))).))))))))..))))))...)))))))	0	0	1.28	1.19	1.59	1.96

2	GTACTTGCT	0	3	))))						
cgr-miR-106b-5p_R-	TAAAGTGCTGA	2	gp	((((((((((..((((((..(((((((((((((.(.....).)))).))...)))))))..)))))) ...))))))				28.5	18.5	
1	CAGTGCAGAT	1	3	)))).	0	3.05	8.34	8	7	1.96
cgr-miR-106b-3p_R-	CCGCACTGTGG	2	gp	((((((((((..((((((..(((((((((((((.(.....).)))).))...)))))))..))))))...))))))						
2	GTACTTGCT	0	3	)))).	0	0	1.28	1.19	1.59	1.96
rno-miR-122-3p_R+	AACGCCATCAT	2	gp	.((((((((((((....(((((((..((((((((((((((.....))).)))))))))))..)))))))....)))).						
3	CACACTAAACA	2	3	)))))))).	0	0	0	0	1.06	0
	TGCCAAACGCC									
oha-miR-122-3p_L+	ATTATCACACT	2	gp							
3R+4	AAAT	6	3	...((((((....(((((((.(((((((((((.((.......))))))))))))).)))))))....)))).)).	0	0	0.64	0	0	0
	TCAAGGTCCGC	2	gp							
ccr-miR-124a_R+2	TGTGAACACGC	2	3	(((((((...(((((.(((..((((.(((..((....))...))).)))).)))..))).)))))..).))))))).	0	0	0.64	0	0	0
	ACAGGTGAGGT	2	gp							
cgr-miR-125a-3p	TCTTGGGAGC	1	3	(((...(((..((..(((.((((..((((((((((....))....))))))))))).)))..))..)))))).....	0	0	0.64	0	0	0
	CAGTGCAATGA	2	gp	(((..(..(((..(((((((...((((((((...((...((....))..))))))))))...)))))))..)))..).				26.8	15.9	
mmu-miR-130b-3p	TGAAAGGGCAT	2	3	.)))	0	1.14	7.70	0	1	0.98
ccr-miR-132b_1ss4A	ACCGTGGCTGT	2	gp	.......(((((((((.((((((.((((..((((.((((((....((((....))))...)))))).)))).))))						
G	AGACTGTTACC	2	3	.)))))).)))).)))))......	0	0	0	0	0	1.96
dre-miR-135a_R+1_	TATGGCTTTTTA	2	gp	...((((.(((((.(((((((((..((((((((..(((.............)))..)))))))).)))))).)))))						
1ss23AT	TTCCTATGTGTA	4	3	)))))))..............	0	1.53	4.49	1.19	1.06	0
	ATCATCGTCTCA	2	gp	((((((((((....(..((.....(((((((((((.(((((((.((((((...((((((((((.........)))))						
hsa-miR-136-3p_L-1	AATGAGTCT	1	3	)))))...))))))))))))).)))))))).)))...))..)))))))))).))..........	0	0	0	2.38	0	3.92
	GCTACTTCACA									
aca-miR-138-2-3p_L	ACACCAGGGTT	2	gp	......(((..((.((((((..((((((((((((((...((((((((...........)))))))..))))))))))))).						
-2R+3	T	3	3	))))).))..)))	0	0	0	2.38	1.06	1.96
aca-miR-142-3p_L+	CTGTAGTGTTTC	2	gp	..(((((..((((((((((((((((((.((((((((((.....((....))......)))))))))).))))))))))))	0	2.16	1.92	0	0	0.98

1R+2	CTACTTTATGGT	4	3	))))))..)))))).						
mmu-miR-145b_R-2	GTCCAGTTTTCC	1	gp							
_1ss18GC	CAGGACA	9	3	.((((((((.....((..((((((((..((((((((....)))).)))).)))))))))).....)))))))).	0	0	0.32	0	0	0
aca-miR-148b-5p_R	GAAGTTCTGTT	2	gp	.......(((((.(((((((..(((((((((.((.((((..((((.............)))))))).)).))))))))						
+1_1ss20TA	ATACACTCAGG	2	3	)..))))))).))).))..	0	0	0	0	0.53	0
	TCTGGCTCCGTG	2	gp	.(((((((((.(((...((((((((..((((.(((((...(((....)))..)))))))))..)).))))))..))						
hsa-miR-149-5p_R-2	TCTTCACTC	1	3	).))))).)))))).	0	0	0.64	0	2.12	0
	CTAGACTGAGG	2	gp							
cgr-miR-151-3p	CTCCTTGAGG	1	3	((((((.((((((((((((..((((((((((...........)))))))))).))))))))))))).))))))...	0	0.76	2.24	4.76	5.30	2.94
aca-miR-153-5p_L+	GTCATTTTTGTG	2	gp	((((((((((((((((((((((((((((.((.......))..))))))).)))))))))))))).)))))).....			16.0			
1	ATTTGCAGCT	2	3	....	0	2.29	3	1.19	4.24	0
	TTGCATAGTCA									
aca-miR-153-3p_R+	CAAAAGTGATC	2	gp	((((((((((((((((((((((((((((.((.......))..))))))).)))))))))))))).)))))).....						
5	GTTT	6	3	....	0	0	1.28	1.19	0	0
pma-miR-181a-3p_R	ACCATCGACCG	2	gp	...(((...(((.((((((..(((((((.((((.(((......)))...)))))))))))))))))).)))...))).						
-1_2ss16CG19TG	TTGAGTGGAC	1	3	..	0	0	0	1.19	0	0
	AACATTCATTG									
hsa-miR-181b-5p_R	CTGTCGGTGGG	2	gp	.....((.((((.....))))....(((((((.....(((((((((...((((((((((...((....))...))))))						
+3	TTAA	6	3	)))).))))))))))....))))))))..))..	0	2.29	2.57	1.79	3.18	7.85
	AACATTCATTG									
	CTGTCGGTGGG	2	gp	.....(((((((.....(((((((((...(((((((((((..((....))..))))))))))).))))))))))....						
aca-miR-181b_R+4	TTAA	6	3	)))))))....	0	2.29	2.57	1.79	3.18	7.85
	CATCCCTTGCAT	2	gp	.((((((..(((..((..(((((((((..((((((................))))))..)))))))))..))..))).)						
ptr-miR-188	GGTGGAGGGT	2	3	).)))).	0	0	0	0	1.06	0
	GCTGCACTTGG	2	gp	((..((.((..((.((((((((((.((..((((((((.................))))))))..)))))))))))).)).						
aca-miR-191-3p_R-1	ATTTCGTTCC	1	3	.)).))..))..	0	0	0	1.79	1.59	0

oha-miR-191-3p_R-1_1ss15CT	GCTGCACTTGGATTTCGTTCC	21	gp3	((..((.((..((.((((((((((.((..(((((((.................)))))))..))))))))))).)).)).)).))..))..	0	0	0	1.79	1.59	0
cgr-miR-196b_R+1	TAGGTAGTTTCCTGTTGTTGGGT	23	gp3	...((((((..(((((((.((.(((((((((((..........))))))))))).)).)))))))..)))))).......	0	0	0.64	0	0	0
bta-miR-197	TTCACCACCTTCTCCACCCAGC	22	gp3	..(((..(((.((((.(((((((..((((..((((((....))).)))..))))..))))))).)))).)))..))))....	0	0	0.64	1.19	1.06	0
tni-miR-205_R+2	TCCTTCATTCCACCGGAGTCTGAA	24	gp3	((..(((((..((((.((((((((..............)))))))).))))..)))))..))....	0	0	0	1.19	2.12	1.96
rno-miR-210-5p	AGCCACTGCCCACAGCACACTG	22	gp3	..((((...)))).(((((..(((..(((((.(((..(((((((((.((.(((......))).)).)))))).)))))).)))).))))))..)))..)))))............	0	0	0.64	1.19	0	0
hsa-miR-212-3p_R+1	TAACAGTCTCCAGTCACGGCCA	22	gp3	((((((...((.....)).(((.(((.(((..((.(((((..((((((.((((((...(((...))).))))))))))))..))))).))...))).)))))))))))))..	0	0	0	0	1.06	0
hsa-miR-215-3p_R-1_2ss10T-13TG	TCTGTCATTCTGTAGGCCAAT	21	gp3	(..((((((((...((.(((((((..(((((((((.....((.....))...))))))))).)).))))).))...))))))))..)..............	0	0	0	0	1.06	0
aca-miR-221-5p_R-1_1ss22TA	AACCTGGCATACAATGTAGAAA	22	gp3	....((((((((((..(((.(((((((((....(((((.(.....)))))))))))))))).)))..)))).))))))....	0	0.38	0.96	0	0	0
hsa-miR-222-5p_L+2R-2_2ss11CT21TC	CGCTCAGTAGTCAGTGTAGACC	22	gp3	((((...((((.((((((((...((((((((((((((((((...((((.......))))...))))).)))))))))))))...))))))..))))).))...)))))	0	0	0	0	1.06	0
dre-miR-222b_R+2_1ss13AT	AGCTACATCTGATTACTGGGTCAGT	25	gp3	(((((..((..(.((((((((((.((((((((((..((((.......))))......))))).)))).))))))))))..)).)))))).........	0	0.76	1.28	0	0	0
rno-miR-224-5p_R+2_1ss1CT	TAAGTCACTAGTGGTTCCGTTTAG	24	gp3	(((((((..((((((((((.((..((((((...(((..((......))..)))..))))))..)))))))))))..)))))))	0	0	0	0	1.06	1.96
cgr-miR-298-5p_R-1	GGCAGAGGAGG	2	gp	((..(((..((((((((((((((((((((((((..((........)).))))))))).))))))).)))))))).))	0	0	0	1.19	0	0

	GCTGTTCTT	0	3	)..)).						
eca-miR-302d_L-1_	AAGTGCTTCCA	2	gp							
1ss18TC	TGTTTCAGTGT	2	3	((..((((..((((((((((((((((.................))))))))))))))))..)))).))	0	0	0	0	0.35	0
hsa-miR-302d-3p_L-	AAGTGCTTCCA	2	gp							
1_1ss18GC	TGTTTCAGTGT	2	3	((..((((..((((((((((((((((.................))))))))))))))))..)))).))..	0	0	0	0	0.35	0
mmu-miR-322-3p_R	AAACATGAAGC	2	gp	.........((((((((((.((..(((((..(((((((((((((............)))))))))))))..)))))..						
-1	GCTGCAACA	0	3	)).))))))).)))...	0	0	1.28	8.34	7.43	0
	CCCAATACACG	2	gp	(((.....((((((((...))))))))((((..(((((((.(((((........(((((((...)))))))......)						
ggo-miR-323b_R+1	GTCGACCTCG	1	3	)))).))))))))..))))....))).....	0	0	0.64	0	0	0
	CGCATCCCCTA									
	GGGCATTGGTG	2	gp							
cfa-miR-324	T	3	3	.(((.((((((((((((((((.(..(...(((....))))..).))).))))).))))))).))))....	0	0.38	0.32	1.19	1.59	0
hsa-miR-324-3p_R+	ACTGCCCCAGG	2	gp							
1	TGCTGCTGGT	1	3	.(((.((((((((((((((((.(..(...(((....))))..).))).))))).))))))).))))....	0	0.38	0	1.19	0	0
	CGCATCCCCTA									
cgr-miR-324-5p_R+	GGGCATTGGTG	2	gp							
1	T	3	3	..((((((..(((...(((.(((((.(((.((..........))))).))))).)))...))).))))))......	0	0.38	0.32	1.19	1.59	0
cgr-miR-324-3p_L-2	ACTGCCCCAGG	2	gp							
R+1	TGCTGCTGGT	1	3	..((((((..(((...(((.(((((.(((.((..........))))).))))).)))...))).))))))......	0	0.38	0	1.19	0	0
	CCTAGTAGGTG	2	gp	((((((((((..(((.((((((((((((((..(.((((...))))..)..)))))))))))))).)))..)))))))						
cgr-miR-325-5p	CTCAGTAAGT	1	3	)))	0	0	0	0	0	1.96
	CCTCTGGGCCCT	2	gp	...((((((((((((.(((((((..((((...((((.....))))..))))....))))))).))))))).)))))						
cgr-miR-326	TCCTCCAGT	1	3	....	0	0.76	1.28	7.15	8.49	3.92
	CTGGCCCTCTCT	2	gp	.......(((((..((..(((..((((((..(((((.(((((...((((....)))).))))))))))..))))))..				40.4	25.4	
bta-miR-328	GCCCTTCCGT	2	3	)))..))..)))))	0	0	8.98	9	6	0
mml-miR-329-3p_R-	AACACACCTGG	2	gp	(((((..(((..((((((..(..(((...((((((.((......)).))))))).)))..)..))))))..)))..)))	0	0	0	1.19	0	0

1	TTAACCTCTT	1	3	))						
	CAAAGCACAGG									
mmu-miR-330-3p_L	GCCTGCAGAGA	2	gp	........((((....((((((((((((((((((.(((..((((.............)))).))))))).))))..))						
-1R+1	G	3	3	))))))))))))......	0	0	0.32	2.98	1.06	0
	TTCTAGGTATG	2	gp	.(((((...))))).((((...((((((((.((((((((.((((.(((((((..(.............)..))))))						
chi-miR-331-5p_L+1	GTCCCAGGGAT	2	3	).))))))))))).)))))))))..)).))..	0	0	0	0	0.35	0
chi-miR-331-3p_L+1	GCCCCTGGGCC	2	gp	.(((((...))))).((((...((((((((.((((((((.((((.(((((((..(.............)..))))))						
_1ss22CT	TATCCTAGAAT	2	3	).))))))))))).)))))))))..)).))..	0	0	0	0	1.06	1.96
	TCCGTCTCAGTT	2	gp	..((((((..(((((((...((((((.(((...((((.......))))..))).))))))....))))))).))))						
cgr-miR-340-3p	ACTTTATAGCC	3	3	)).....	0	0	0	1.19	0	0
	TCCGTCTCAGTT	2	gp	..(((((.((((((..(((((((...((((((.((((..((.........))...)))).))))))....))))))).						
bta-miR-340	ACTTTATAGCC	3	3	.)))))).)))))....	0	0	0	1.19	0	0
	AGGGGTGCTAT	2	gp	........(((((.(((((.((((((((.((((((..((((((.....((((((...((....))..)))))).)))						
hsa-miR-342-5p	CTGTGATTGA	1	3	))).....)))))).))))))))))))).)).)))	0	0	0	1.19	0	0
	GCTGACTCCTA	2	gp	((....(((((((.((((((.(((((((((.((.(((((((((..........))))))))).)).))))))).))						
ggo-miR-345_R-2	GTCCAGGGCT	1	3	))))))))))))....))..(((.....)))..	0	0	0	1.79	0	0.98
hsa-miR-345-3p_L-1	CCCTGAACTAG	2	gp	((....(((((((.((((((.(((((((((.((.(((((((((..........))))))))).)).))))))).))						
R+1_1ss10GT_2	GGGTCTGGAGT	2	3	))))))))))))....))..(((.....)))..	0	0	0	0	0.53	0
	GCTGACTCCTA	2	gp	...(((((((.((((((.(((((((((.((.(((((((((..........))))))))).)).))))))).)))))						
hsa-miR-345-5p_R-1	GTCCAGGGCT	1	3	))))))))))............	0	0	0	1.79	0	0.98
hsa-miR-345-3p_L-1	CCCTGAACTAG	2	gp	...(((((((.((((((.(((((((((.((.(((((((((..........))))))))).)).))))))).)))))						
R+1_1ss10GT_1	GGGTCTGGAGA	2	3	))))))))))............	0	0	0	0	0.53	0
	TTCACAAAGCC	2	gp	...(((.((((((((.(((.(((((((((((.((.......)).))))).)))))).))).)))))).)).)))..						
cgr-miR-350-3p_R-2	CATACACTTTC	2	3	....	0	0	1.28	3.57	3.18	0
	TTATCAGAATCT	2	gp	..........((..(((((((((....((((...((((((..(((((((((.((.((((.((((...........)))).						
efu-miR-361	CCAGGGGTAC	2	3	)))).))...)))))))))..))))))...))))........)))))))))..))....	0	0.76	0.64	3.57	2.12	1.96

chi-miR-361-5p	TTATCAGAATCTCCAGGGGTAC	22	gp3	...((((...((((((..(((((((((.((.((((.((((...........)))).)))).))...)))))))))..))))))...))))......	0	0.76	0.64	3.57	2.12	1.96
cgr-miR-362_R-2	AATCCTTGGAACCTAGGTGTGAGT	24	gp3	..(((((.(((((((((((...((((((((.(..(((...)))..)..))))))))))))))))))))..)))))......	0	0.76	0	0	0.53	0.98
hsa-miR-362-3p_1ss10AG	AACACACCTGTTCAAGGATTCA	22	gp3	..(((((.(((((((((((...((((((((.(..(((...)))..)..))))))))))))))))))))..)))))......	0	0	0.32	2.98	0	0
mmu-miR-362-5p_1ss23AG	AATCCTTGGAACCTAGGTGTGAGT	24	gp3	...(((((((((((...((((((((..(((.((....))))).))))))))))))))))))))...	0	0.76	0	0	0.53	0.98
mmu-miR-362-3p	AACACACCTGTTCAAGGATTCA	22	gp3	...(((((((((((...((((((((..(((.((....))))).))))))))))))))))))))...	0	0	0.32	2.98	0	0
hsa-miR-369-5p	AGATCGACCGTGTTATATTCGC	22	gp3	.((.((..((((((((((((..((((((......(((((.((.((..((((((((((((..((((((...((....)).))))))..))))))))))))).)..)))))))))......))))))..))))))))))))).)..)))).	0	0	0	0	0	1.96
ssc-miR-369	AATAATACATGGTTGATCTTT	21	gp3	.((.((..((((((((((((..((((((......(((((.((.((..((((((((((((..((((((...((....)).))))))..))))))))))))).)..)))))))))......))))))..))))))))))))).)..)))).	0	0	0	0	3.18	1.96
hsa-miR-376a-2-5p_L-1R+3_3ss9TC20GA21TG	GTAGATTCTCCTTCTATGAGTAC	23	gp3	........(((((((((.((((.((.((((.((((.((((((....(((......)))...)))))))))).)))).)).)))).))))))))).....	0	0.38	0	0	0	0
bta-miR-376c_R-1_1ss19TG	GTGGATATTCCTTCTATGGTT	21	gp3	(((((((((.((((.(((((..((((.((((((((((.............)))))))))))))..))))).)))).)))))))))	0	0	0	0	1.06	0
chi-miR-376a_R+2	GTAGATTCTCCTTCTATGAGTAC	23	gp3	(((..(.(((........(((((((((.((((.((.((((.((((.((((((....(((......)))...))))))))))).)))).)).)))).)))))))))..))))..)))	0	0.38	0	0	0	0
hsa-miR-378a-5p	CTCCTGACTCCAGGTCCTGTGT	22	gp3	.........(((((((((....((((.((((.((.((((((((((((((...((((((........)))))).)))))))))))))))).)).)))).)))).))))))))))..	0	0	0	1.19	0	0
cgr-miR-382	GAAGTTGTTCG	2	gp	..(((((((((.(((((((((((..((((((..(((....)))))))))..)))))))))))..)))))))))..	0	0	0	0.60	0.53	0

	TGGTGGATTCG	23		.....						
chi-miR-382-5p	GAAGTTGTTCGTGGTGGATTCG	22	gp3	....((........))............((((((.((((((((.(((((((((((..((((((.((.......))))))))..)))))))))))..)))))))))))))..............	0	0	0	0.60	0.53	0
cgr-miR-384-5p	TGTAAACAATTCCTAGGCAATGT	23	gp3	...((((.(((((.(((((((.(((((.((((..(((.......)))...))))))))).))))))).))))).)))))....	0	0	0	1.19	0	0
cgr-miR-409-5p_R-1	AGGTTACCCGAGCAACTTTGCAT	23	gp3	.((((((((.((((..((((((((((((..((.....)))))).)))))))))..)))).))))))))....	0	0	0	0.60	0	0.98
bta-miR-409a	AGGTTACCCGAGCAACTTTGCAT	23	gp3	.(((((.((..(((.((((..((((((((((((..((.....)))))).)))))))))..)))).)))..))))))).	0	0	0	0.60	0	0.98
bta-miR-410	AATATAACACAGATGGCCTGT	21	gp3	.(((((.(((..(.((((..((((((.((..((((.((.......))))))..)).))))))..)))).)..)))))))).	0	0	0	0	0.53	0
cgr-miR-410-3p	AATATAACACAGATGGCCTGT	21	gp3	.((((..(.((((..((((((.((..((((.((.......))))))..)).))))))..)))).)..))))....	0	0	0	0	0.53	0
cfa-miR-411_L-1	TAGTAGACCGTATAGCGTACG	21	gp3	.((((.((((((...(((((((.((.......)))))))))...)))))).))))....	0	0	0	0	2.12	3.92
bta-miR-423-5p	TGAGGGGCAGAGAGCGAGACTTT	23	gp3	....((((((..((((((((((((..(((.((((.(((((.........)))))))))).)))...)))))))))))))..)))))).........	0	0	10.96	32.16	14.32	0
bta-miR-424-3p_R+1	CAAAACGTGAGGCGCTGCTATA	22	gp3	...((..((((((((((.(..((((((..(((((((((((((((.......))..))))))))))))))..))))))..).))))))).)))..)).	0	0	0	1.19	0	0
eca-miR-425_L-1R-1	CATCGGGAATATCGTGTCCGCC	22	gp3	......(((((.....((((((((.(((((((((((..((...((((((((...(((((.......(((.........)))..)))))..))))))))..)).))))))).))))))..)))......)))))).......))))).	0	0	3.21	5.95	3.18	1.96
pma-miR-429-3p_1s	TAATACTGTCTG	2	gp	((..((((((((((((....((((((((...((((((...((.....))....))))))...))))))))...)))))	0	0	0	0	0	1.96

s15TA	GTAATGTCGC	2	3	)))))))..))						
eca-miR-431_L-2R-2	TCTTGCAGGCCGTCATGCA	19	gp3	.((((((((((..(.(((((.((((........)))).....((((....)))))))))).)..)))))))))).((((....)).))..	0	0.38	0	0	0	0
hsa-miR-431-5p_L-2	TCTTGCAGGCCGTCATGCA	19	gp3	...((.(((((.((((((..((((((((((.((...((((((((........)))....)))))...)).))))))))))..)))))).))))))).................	0	0.38	0	0	0	0
rno-miR-466c-5p_R+1	TGTGATGTGTGTATGTACATGT	22	gp3	......(((..(((.((((((((((((...((..((((((((......))))))))..)).)))))))))))))..)))	0	0	0.64	2.38	1.06	0
rno-miR-466b-4-3p_L-1	TATACATACACACATACACA	20	gp3	..(((((((((.((((((((((((((((..((((((((((......))))))))))))))))))))))))))).)))))))))....	0	0	0.32	0.17	0	0
mmu-miR-466f-5p_L-2R-2_1ss14AT	CGTGTGTGTGCTTGTGCA	18	gp3	(((..((((....(((..((((((((..(((((..(((((..((..(((((.......)).))).)).)..)))))..)))))..)))))....)))..)))....)))).))).......	0	0	0	0	1.06	0
mmu-miR-486b-3p_R+2	CGGGGCAGCTCAGTACAGGATG	22	gp3	..((...(((((...((.(((.((((((...((((((((((((((((((((((........(((....))))))))))))))))))))))))...)))))).))).))...)))))).....))..	0	0	1.28	2.98	0	0
mmu-miR-488-3p_R+1	TTGAAAGGCTGTTTCTTGGTCT	22	gp3	(((((..((((.(((((.((.(((..((.(((.((((((.((.(((((((((.........)))))))).)))))))))))).))..))).)).))))).))))..)))))	0	0	0.64	5.95	0	0
aca-miR-490-3p_L-1R-4_1ss2AC	CACCTGGAGGACTCCATG	18	gp3	.....((..((.(.(((((((.(((((((((((((((.(((................))).)))))))))..))))))).))))))))))..)).....	0	0	0	0	1.06	0
bta-miR-497_R-1	CAGCAGCACACTGTGGTTTGT	21	gp3	...((.(((((((.(((((((((.((((.((((.(((((((((.(((((......)))..)).)))))))))).)))).))))).......))))))))))))....)))).))	0	0	7.05	2.38	8.49	3.92
mmu-miR-500-3p_R-1	AATGCACCTGGGCAAGGGTTC	21	gp3	.......((((.((.((((((((((((((...(..(((((....(((.........)))..)))))..))))))))))).))))).)).))))	0	0.38	0.96	1.19	1.59	1.96
hsa-miR-500b-5p_L+1R+4	TAATCCTTGCTACCTGGGTGAGA	23	gp3	((((((....(((((((((...((((((((....(((.........)))...))))))))))))))))).....)))))).	0	0	0	0	1.06	0
hsa-miR-501-3p	AATGCACCCGGGCAAGGATTCT	22	gp3	((((..((((((.((((((((.(.(((.((((((....(((.........)))...))))))))))))))))..)))))))..))))	0	0.76	0.64	0.60	0	1.96
cgr-miR-500	AATGCACCTGG	2	gp	....((.(((((((((((((((...((((((((...(((.........)))..))))))))))))))))).))))).)	0	0.38	0.96	1.19	1.59	1.96

	GCAAGGGTTC	1	3	)..						
rno-miR-503-5p	TAGCAGCGGGAACAGTACTGCAG	23	gp3	(((((..(((((((((((((((((.(((...((.......)).))).)))))))))))))))))..)))))	0	0	0.64	1.79	0	0.98
rno-miR-503-3p_R+1	GGAGTATTGTTTCCGCTGCCTGGT	24	gp3	(((((..(((((((((((((((((.(((...((.......)).))).)))))))))))))))))..)))))	0	0	0	0.60	1.06	0
mmu-miR-511-3p_R-1	AATGTGTAGCAAAAGACAGGA	21	gp3	(((.((((((.((.((((((((..(((.(((.(((((....)))))))).)))..)))))))).)))).)))).)))..	0	0	0	0	1.06	0
ggo-miR-515-5p_R-5_1ss17AT	TTCTCCAAAAGAAAGCTCT	19	gp3	.....(((......(((((..((((.(..(((((((((((.((((..((((((.......)))))))))).))))))))))..).))))..)))))).....))).........	0	0	0	0	0	1.96
ggo-miR-524-5p_L-4_1ss9GT	AAAGTGAAGCACTTTCTC	18	gp3	.............(((((..(((((((.((.(((((((((((.((((((....(((...)))..)))))).))))))))))).)).)))))))..)))))....	0	0	0	0	0	0.98
bta-miR-532	CATGCCTTGAGTGTAGGACCGT	22	gp3	..........(((((((.((.((((((.((((.(((..((.............))..))).)))).)))))).)).))))))...	0	0	1.28	0.60	0.53	0.98
hsa-miR-532-3p_R-1	CCTCCCACACCCAAGGCTTGC	21	gp3	..........(((((((.((.((((((.((((.(((..((.............))..))).)))).)))))).)).))))))...	0	0	0	1.19	1.06	0
chi-miR-532-5p	CATGCCTTGAGTGTAGGACCGT	22	gp3	.....(((..(((((((.((.((((((.((((.(((..((.............))..))).)))).)))))).)).)))))))..)))..............	0	0	1.28	0.60	0.53	0.98
chi-miR-532-3p	CCTCCCACACCCAAGGCTTGC	21	gp3	.....(((..(((((((.((.((((((.((((.(((..((.............))..))).)))).)))))).)).)))))))..)))..............	0	0	0	1.19	1.06	0
hsa-miR-539-3p	ATCATACAAGGACAATTTCTTT	22	gp3	((((((.((((((((((.((((((.((((((((((.......)).)))).)))))))))).)))))))).))))))))	0	0	0	0	0.53	0
chi-miR-542-3p_R+2	TGTGACAGATTGATAACTGAAA	22	gp3	....((((((.......(((((((...((((..((((...(((((((((.(((....)))..)))))))))...))))..))))...)))))))........))))))...	0	0.38	0.32	0.60	0	0
hsa-miR-548l	AAAAGTATTTG	2	gp	((((.((((((((((.(((((..(((((.(((((((((........))))))))).)))))..))))).))))))	0	0	0	1.19	0	0

	CGGGTTTTGTC	2	3	)))).)))))..						
hsa-miR-548ba_L-2_	AGGTAGCTGTG	2	gp							
1ss8AG	ATTTTTGCT	0	3	.(((((((.(((..((((((((((((.....))))))))))))..))).)))))))	0	0	0	0	1.06	0
mml-miR-548f_L-1	AAAACCAGAGT	1	gp	...((((((((((((((((.(((((.((.(((.(((((((....))))))).))).)).))))).)))))))))						
R-2_1ss9CG	TCCTTTT	8	3	)))))))....	0	0	0	0	1.06	0
hsa-miR-548y_R-3_	AAAAGTCATCA	1	gp	........(((((((((((((((((((((((((.((((((((((((......)))))))))))).)))))))))						
1ss7AC	CTGTTTTT	9	3	))))))))))))))))...((..(.....)..))	0	0	0	0	0.53	0
	GATGAGCTCAT	2	gp	..............(((.(((((((((((((((((((((.((.((((((((((..............)))))))))).)						
hsa-miR-556-5p_R-1	TGTAATATGA	1	3	).)))))))))))))))))))))).)))...	0	0.76	0	0	0	0
	ATATTACCATTA	2	gp	..............(((.(((((((((((((((((((((.((.((((((((((..............)))))))))).)						
ggo-miR-556_R+2	GCTCATCTTT	2	3	).)))))))))))))))))))))).)))...	0	0	0	0	1.06	0
	CACGCTCATGC	2	gp	..........((((((((....((((.(((((((((...(((((((..((((((((..((......))..))))))))						
ggo-miR-574	ACACACCCACA	2	3	..))))))).))))))))).))))))))))))	0	0	0.96	1.79	3.71	0
hsa-miR-576-5p_R+	ATTCTAATTTCT	2	gp	..(((((.((.((((((((((((((.((((((((((((..((......))..)))))))))))).))))))))))						
1	CCACGTCTTTG	3	3	)))).)).)))))..........	0	0	0	0	1.06	0
hsa-miR-576-3p_R-4	AAGATGTGGAA	1	gp	..(((((.((.((((((((((((((.((((((((((((..((......))..)))))))))))).))))))))))						
_1ss18GC	AAATTGC	8	3	)))).)).)))))..........	0	0	0	0	1.06	0
	GTAGATAAAAT	2	gp	((((((((((((((((((((((.(((((..(((((.............)))))..))))).)))))))))))))						
hsa-miR-577_L+1	ATTGGTACCTG	2	3	))))))))))...........	0	0	0.64	0	0	0
	TTATGGTTTGCC	2	gp	....((((((.(((((((((...(((((.((((((((((((...(((......)))...)))))))))))).)))))						
ggo-miR-584_R+1	TGGGACTGAT	2	3	)...))))))))).))))))).((((.....)))).	0	0.76	0	0	0	1.96
	TACGTCATCGTT	2	gp							
eca-miR-598_R-1	GTCATCGTC	1	3	.((((.((((..((((..(((((..(..((((........))).)..)..)))))..)))).)))).)))).	0	0	0	2.38	0	0
	TACGTCATCGTT	2	gp	...((((((((..((....)).(((((((((..(((((..((.(((............)))))..)))))..))))))						
ptr-miR-598_R-1	GTCATCGTC	1	3	)))..))))))))).....	0	0	0	2.38	0	0
cgr-miR-615-3p_R-1	TCCGAGCCTGG	2	gp	((((.((((((((((..(((((.((((((((((.(((..(...)..)))...))))))))))))))..))))))))	0	0	0	0.60	1.06	0

	GTCTCCCTCT	1	3	))))))...						
	TAGTACCAGTA	2	gp	(((((((((((((((((((((((((((((((((...((....)).....))))))))))))))))))))))						
hsa-miR-624-5p_R-1	CCTTGTGTTC	1	3	))))))))))))...........	0	0	0.32	0	0	0
hsa-miR-625-3p_1ss	GACTATAGAAC	2	gp	(((((((((((((((((((((((((((((((((((...........)))))))))))))))))))))))))						
22AT	TTTCCCCCTCT	2	3	))))))))))))	0	0	0	0	0	1.96
hsa-miR-652-5p_L+	ACAACCCTAGG	2	gp	......((...(((((((((((((((((.....(((((((((((.............))))))))))).))))))))						
1R-4_1ss14AG	AGGGGGTGCCA	2	3	))))))))))...)).....	0	0	0	0	0	0.98
cgr-miR-652-5p_L+	ACAACCCTAGG	2	gp	.(((((((((((((((((.....(((((((((((..((.....))..))))))))))).)))))))))))))))						
1R-4_1ss14AG	AGGGGGTGCCA	2	3	)))..	0	0	0	0	0	0.98
	TATGTCTGCTGA	2	gp	(((((.....(((((((((((...(((((.((((((((.........).))))))))))))...)))))))))))...)						
bta-miR-654_R-2	CCATCACC	0	3	))))	0	0	0	0	1.06	0.98
	TATGTCTGCTGA	2	gp	....((((((..((((....))))......((((.((((((...((((.((((((((((....)).).)))))))))						
hsa-miR-654-3p_R-2	CCATCACC	0	3	))...))))))).))))...)))))).......	0	0	0	0	1.06	0.98
cgr-miR-664-3p_1ss	TATTCATTTACT	2	gp							
23CT	CCCCAGCCTAT	3	3	...........((((((((((((((..((((((......))))))...)))))))..))))))))).............	0	0	2.57	0	1.06	0
	ACCAGGAGGCT	2	gp							
hsa-miR-665_R+2	GAGGCCCCTCT	2	3	((.(((.((((((((((.((((((...((.((.....)).))....)))))).))))))))))..))).)).	0	0	0	0	0	1.96
ggo-miR-668_L-1R+	TGTCACTCGGCT	2	gp	..((((.((((((.....)))))).))))..(((.((((.(((((((((((.((((((((.......)))))))))))						
1	CGGCCCACT	1	3	)))))...))).))))))))................	0	0	0	0	1.06	0
mmu-miR-674-5p_R	GCACTGAGATG	2	gp	.......((((((((((..((((((((((((..(((((..((....)).)))))..))))))))))))..))))))						
-1	GGAGTGGTGT	1	3	))))	0	0	0.64	5.95	4.24	0
mmu-miR-690_L-1R	AAGGCTAGGCT	2	gp	((((((((..(((((((.((((((...........))))))..((..((((((((.......))))).)))..)).((						
+1	CACAACCAAAA	2	3	(......)))..))))).))...))))))))	0	0	0	0.30	0	0
	TGCCCACCCTTT									
rno-miR-702-3p_R+	ACCCCACTCCA	2	gp	..((((((((((((.((((((((....)).((..((((((((((.((((.....)))).))..)).)))).))..)						
2	GT	5	3	).))))))).)))....)))))))))......	0	0	0	1.19	0	0

eca-miR-703_L-2R-1	AACCTTCAGAAGGAAAGG	18	gp3	(((((((..((.((..((....(((((.((....)).)))))..))..)).))..)..))))))	0	0.76	0	0	0	0
bta-miR-708	AAGGAGCTTACAATCTAGCTGGG	23	gp3	............(((..((((((((.(((((((((((.(((((.....(((.....)))(((........))).))))).))))))))))).))))))))..)))...	0	0.76	0	0	0	0
hhi-miR-723_L-2R-1_1ss9GT	ATCTGTTCTTATCCTTGCT	19	gp3	.((((.((((((..((((.((((.((((((..((((((.......)))))))))))).)))).)))).)))))).)))).	0	0	0	0	1.06	0
mmu-miR-762_R-3_1ss15GC	GGGGCTGGGGCCGGCACAG	19	gp3	(((((((((...((((((((((...((((((.((((((...)))))).))))))))))))))))).))))).).)))	0	0	0	0	1.06	0
ggo-miR-766	ACTCCAGCCCCACAGCCTCAGC	22	gp3	................(((...((((.((((((((..((((...(((.(((((....(((.........)))..))))).)))...)))).))))))).))))....)))...	0	0	0	0	0	1.96
cfa-miR-802_L+1R-1	TCAGTAACAAAGATTCATCCTTG	23	gp3	..((((((.((..(((.(((.((((((((((.(((((((((.......)).)))))))))))))))))).))).)))..)).))))))...	0	0	0	0	1.06	0
rno-miR-871-3p_R-4_1ss13TC	TGACTGGCACCACACTGG	18	gp3	.(((.(((..(((((((..((((((((((((.(((.....))).))))))))))))..)))))))..))).)))...	0	0	0	0	1.06	1.96
cgr-miR-872-3p_R+1	TGAACTATTGCAGTAGCCTCCT	22	gp3	(((..((.((.(((((((.((((((((((((...............)))))))))..)))))))))).)).))..)))	0	1.53	0	0	4.24	0
cgr-miR-874_R-1	CTGCCCTGGCCCGAGGGACCGA	22	gp3	.....(((((...(((.(((.((..((((((((.(..((.....))..)))))))))..)).))).)))...)))))..	0	0.76	1.28	1.19	1.06	2.94
bta-miR-877_R+3	GTAGAGGAGATGGCGCAGGGGAC	23	gp3	.........((..((((((..((...((((((((((.........((((((.((....)).))))))...))))))))))...))))))))))).........	0	0	0.64	1.19	0	0
mmu-miR-877-3p_1ss22AT	TGTCCTCTTCTCCCTCCTCCCT	22	gp3	.........((..((((((..((...((((((((((.........((((((.((....)).))))))...))))))))))...))))))))))).........	0	0	0	1.19	1.06	0
hsa-miR-939-3p_R+1	CCCTGGGCCTCTGCTCCCCAGT	22	gp3	.((((...((((.(((((((((.(((((.(..(((..(((....)))..)))..)))))).))))))))).)))).)))).	0	0	0	1.19	0	0

mmu-miR-1195_L-2R-3_1ss20CG	AGTTCGAGGCCAGCCTGG	18	gp3	..((((((((....((((((((.((((..(((..(((((((((......)))))....)))).)))..)))).))))))))...))))))))......	0	0	0	1.19	0	0
mmu-miR-1198-5p	TATGTGTTCCTGGCTGGCTTGG	22	gp3	.......................(((.(((((.((.(((..(((.(((...((((((((((((((......)))..))))))))))))...))).)))..))).))..))))).))))....	0	0	0	0	3.18	0
ptr-miR-1244_L+1R-8	AAAGTAGTTGGTTTGTATG	19	gp3	((((((.....(((..((((..(((......((((.((((......))))))))))..))))..))))))))))..........	0	0	0	0	1.06	0
bta-miR-1249	ACGCCCTTCCCCCCCTTCTTCA	22	gp3	.(((((((((.((.((.((((...(((((........))))))))).)).))))))))))))....	0	0	0.64	1.19	0	0
hsa-miR-1252-5p_L-1R-2_1ss11TA	GAAGGAAATAGAATTCATT	19	gp3	((((((((((((((((..((((((.(((.......))).))))))..))))))))))))))))	0	0	0	1.19	0	0
mml-miR-1271-5p	CTTGGCACCTAGCAAGCACTCA	22	gp3	(((((((.(((((..((((((..(((((.(((((.(((((((..((....))..))))))).))))).)))))..))))))..))).)))))....))))..........	0	0	0	1.19	0	1.96
cgr-miR-1271_L+1R+1_1ss11GC	AGTGCCTGCTCTGTGCCAAGGC	22	gp3	.....((..((((((((((((..((((.(((((((............))))))).))))..)))))))))).)))))..))	0	0	0	1.19	1.06	0
cgr-miR-1285_L+2R-4	ACTGCACTCCAGCCTGGGCAAC	22	gp3	((((...((((((..((((...(((..((((((((....((((....))))..)))))))))).))))..)))......)))..))))..	0	0	0.16	0.30	0	0
eca-miR-1298_R+1	TTCATTCGGCTGTCCAGATGTATTTGCAGCTGCCT	23	gp3	...((((..((..(((((((((.((((.((((((((((((((((((((......)))))))......)))))))))))))).))))).)))))))))..))..))))......	0	0	0	0	0	3.92
ggo-miR-1301_R+4	GGGAGTGACTTCAT	26	gp3	...............((((..((((((((((.(..((((((((((((.((.((((.....)))).))..((....)).))).))))))))..).)))))))))))))))...	0	0.76	0	0	0	0
hsa-miR-1307-5p	TCGACCGGACCTCGACCGGCTACTCGGCGTGG	21	gp3	((((.(((((..(((((.((.(((..((((((((...(((.(((((((.((.((.(((..(((..............)))..))))).))..))))))).)))...))))))))....)))..)).))....)))...))))))))))..	0	0	0	1.79	1.06	0
bta-miR-1307_R+1	CGTCGGTCGTGT	23	gp3	((((.(((((..(((((.((.(((..((((((((...(((.(((((((.((.((.(((..(((..............)))..))))).))..))))))).)))...))))))))....)))..)).))....)))...))))))))))..	0	0	0	0	0.53	0

chi-miR-1307-5p_R+4	TCGACCGGACCTCGACCGGCT	21	gp3	..((((((.(((.(((.......((((((..((((((....(((((((.((.((.(((..(((..............)))..))))).))..)))))))))))))...))))))....))).))).)))).))..((..(....)..))	0	0	0	1.79	1.06	0
chi-miR-1307-3p_1ss23GT	ACTCGGCGTGGCGTCGGTCGTGT	23	gp3	..((((((.(((.(((.......((((((..((((((....(((((((.((.((.(((..(((..............)))..))))).))..)))))))))))))...))))))....))).))).)))).))..((..(....)..))	0	0	0	0	0.53	0
oan-miR-1405-5p_L-4R+1	CAATGATTGTGGCTGCAG	18	gp3	..((..(((..(((((...(((((((((..(((.((((((((...........)))))))).)))..)))))))))...)))))..)))..))...........	0	0	0	0	0	1.96
bta-miR-1468_R-2	CTCCGTTTGCCTGTTTTGCT	20	gp3	........((((((((..((.(((((((((.(((((((((((..................))))))))))).))))))))).))..))))))))...	0	0	0	0	2.12	0
hsa-miR-1538_L-5_1ss13CA	CGGGCTGATGCTGTTCCT	18	gp3	(((((((((((((...((((((.((((.......)))).))))))...)))))))))))))	0	0	0	0	1.06	0
hsa-miR-1587_1ss6CT	TTGGGTTGGGCTGGGTTGGG	20	gp3	.((((...(((.(((((..(((((.....)))))..))))))))....)))).	0	0	0	1.19	0	0
aca-miR-1805-3p_R+1_1ss22CA	TGTATTGGAACACTACAGCTCAA	23	gp3	.(((..(((..(((((((((((.((((((((((.....((......))...)))).)))))).)))))))))))..)))..)))	0	0	0.64	0	0	0
cgr-miR-1839-5p_R-1	AAGGTAGATAGAACAGGTCTTG	22	gp3	..........(((((((((...((((((((.((........))..))))))))..)))))))))...............	0	0	1.92	2.38	3.18	0
bbe-miR-2070-5p_R-4_1ss2TC	TCTCCACAGCCTCTACAC	18	gp3	.((((..(((((((((.....(((((((..((........)))))))))).....)))))))))..))))............	0	0.76	0	0	0	0
hsa-miR-2110_R+3	TTGGGGAAACGGCCGCTGAGTGAGT	25	gp3	(((((((..(((((((.((((((.(.((((((...((....))..))))))))))))).))))))..)))))))	0	0	0	0	1.06	0
dre-miR-2187-5p_R+1	TTAATTAGTATAGCCTGTTTTAG	23	gp3	..((.((((.((((((((((....((((((((((((((.....((((...))))........)))))))))))))))....)))))))))).)))).)).....	0	0	0	1.19	0	0
aca-miR-2188-5p_L	TCAAGGTCCAA	2	gp	......(((.((((((((((...(((((..(((.((((..((((...............))))..)))).)))..))))	0	1.53	1.28	0	0.53	0

+2R+2_1ss18GA	CCTCACATGTCCT	4	3	)...)))))))...))))))						
bta-miR-2285aa_L-2R-3_1ss9AT	AACTGGTACGAACTTTTG	18	gp3	..(((((((((((((.((((((..(((....)))..)))))))))).))))))))))....	0	0	0	0	1.06	0
bta-miR-2285a_R-4_1ss14AC	AAAAACTGAATGACATTC	18	gp3	(((((((((((((.(((((((((.((((...((((......))))))))))))))))).)))))))))))))	0	0	0	0	1.06	0
hsa-miR-2355-5p_R+1	ATCCCCAGATACAATGGACAAT	22	gp3	(((..((((.(((.((((((((((.(((((..(((((((.........)))))))))))))).))))))).))).)))))..))))..	0	0.76	0.64	1.19	0	0
bta-miR-2444_R-1_1ss4GT	TTTTTGTTGTTTTTTGTTT	19	gp3	...((((((..((..((........(((((((..(....)..)))))))).........))..))..))))))...	0	0.76	0	0	1.06	0
hsa-miR-2467-5p_R-1	TGAGGCTCTGTTAGCCTTGGCT	22	gp3	((((((((.((((((.((((.((.((((..((((((((......)))..)))))..)))))))))).)))))))))))))))	0	0	0	0	0	1.96
tgu-miR-2991_1ss7CT	ATGTATTGTAGTCTGTTGC	19	gp3	....((..((..((((((..((((.((..((....))..)).))))..)))))..))..)).........	0	0.76	0	0	0	0
mmu-miR-3068-3p_L-2R+1	TGAATTGCAGTACTCCAACAT	21	gp3	((.(((((..(((((((...(((((.(((...((((((........))))))..))))))))..))))))).)))))))	0	0	0	1.19	0	0
mmu-miR-3472_L-7_1ss23AC	CAGAAGCTGGAAGGACCC	18	gp3	(((((((((((((((((((((((((((((((((.....))))))))))))))))))))))))))))))))))..	0	0	0	1.19	0	0
mmu-miR-3473b_L+2R-1	TAGGGCTGGAGAGATGGCTCA	21	gp3	(((((((((((((((((((((((((...)))))..))))))))))))))))))))	0	0	0	2.18	0	0
mmu-miR-3473a_L+3R-1	GGCTGGAGAGATGGCTCAGC	20	gp3	.((((((((((((((.(((((..(((....((((.........))))..)))...))))).))))))))))))))).	0	0	0	0.30	0	0
rno-miR-3473_R-1	TCTAGGGCTGGAGAGATGGCT	21	gp3	.((((((((...((((((((((...(((((.......)))))))))).)))))...))))..)))).	0	0	0	3.37	0	0
rno-miR-3559-5p	TGACAGACTTAGTACTACATGA	22	gp3	(((..(((.....)))...(((.((((..((((((((((((((((((....(((....)))......))))))))))))))))))..)))).)))..............))).	0	0	0.64	2.38	4.24	0

cin-miR-4072-3p_R-2_1ss15CG	AAAATGGATCTTGAGAAA	18	gp3	((((((..((((.((((((((((((....)).))))))))))))))))))))..	0	0	0	1.19	0	0
cin-miR-4092-5p_L-2_1ss16GT	TGTTGTGGAGAACTACAA	18	gp3	..(((((((.(((..(((..(((((.....))))).)))..))).))))))).	0	0	0	1.19	0	0
cin-miR-4123-5p_L-2R-2_1ss13AT	CTTCTTCCTCTGCTCGCT	18	gp3	.((..(((((..((...(((((.....)))))...))..)))))..)).	0	0	0	0	0	1.96
hsa-miR-4286_R+1	ACCCCACTCCTGGTACCA	18	gp3	.......((((((.(((((.((((((((((((..((((((....))).)))..)))))).)))))).))....))).)))).)))........	0	0	0.64	1.19	2.12	1.96
hsa-miR-4433b-5p_R+1	ATGTCCCACCCCCACTCCTGTT	22	gp3	....((((((((((((((((((((((((((((((((((((((.............)))))))))).))))))))))))))))))))))))))))).......	0	1.53	0.64	0	1.06	1.96
hsa-miR-4443_R+1	TTGGAGGCGTGGGTTTTT	18	gp3	.((.(((((((((((..((((((....))))))..))..))))))))))..)).	0	0.76	0	1.19	2.12	1.96
hsa-miR-4459_L-2_1ss9CA	AGGAGGAGGAGGAGGTGGAG	20	gp3	.((((((....((((..((((..((((((.....))...))))....))))..))))..))))))).	0	0.76	0	0	0	0
hsa-miR-4482-5p_L-3R-1_1ss19AT	CCAGTGGGCTATGGATAT	18	gp3	.(((((...((((.((((..((((((((((..........))))))))))..)))).))))...))))).	0	0	0	0	1.06	0
hsa-miR-4492_L+2	CCGGGGCTGGGCGCGCGCC	19	gp3	..((.((((((...(((((.((((..(((.((((..............))))))))..))))))))).))))))..))..	0	0.76	0	0	0	0
hsa-miR-4497_L+1_1ss18CA	CCTCCGGGACGGCTGGGA	18	gp3	(((..((((.(((..((((((((((((((((((..((..........)).)))))))))))......)))))))))..))))))..))).	0	0	0	1.19	1.06	0
hsa-miR-4716-5p_L-4_1ss14CT	TGTTTCCTTTCCCCTTCT	18	gp3	((....)))))....))	0	0	0	0	0	1.96
mmu-miR-5110_L-6R-1	GGTAGAGGGTGGTGGAAT	18	gp3	..(((((..((((((((.((..(........)..)))))))))).)).)))..((((((...((.((....)).))..))))))...	0	0.76	0	0	0	0
aca-miR-5426-5p_L-1R-3_1ss3CT	ATTCTAAGTCTACTTCCA	18	gp3	...(((((..((...(((((((.(((((((.(((....((.....))...))).))))))).)))))))...))..))))))...	0	0	0	0	0	1.96

aca-miR-5445_L-1R-2_1ss2TA	AAAGAGCTCTCTGGAAGA	18	gp3	...((.(((((..((((((.(((.(..(((((((((((((..................)))).)))))))))..).))).))))))..))))).))....	0	0	0	0	0	1.96
hsa-miR-5589-5p_L-3_1ss13TA	TGGGTGCTCATGTGCAGT	18	gp3	(((((((((((..(((((((((..............)))))))).))).)))))))))....	0	0	0	0	1.06	0
cin-miR-5605-5p_R-4_1ss9TC	TCCCATCTCATCCCATCC	18	gp3	((.((((((..(((((((.((((((....)))))).)))))))..)))))).))...	0	0.76	0	0	0	0
hsa-miR-5683_L-4R-2_1ss7TC	GACGCAGATTCTCTGACT	18	gp3	((..((((((.((((((((((((.(.((((((((..((((...)))))))))))))))))))))))))))))))))..))	0	0	0	1.19	0	0
hsa-miR-5690	TCAGCTACTACCTCTATTAGG	21	gp3	((((((((((((((((.((((((((((((...............)))))))))))).))))))))))))))))	0	0	0	1.19	0	0
hsa-miR-6089_L-6_1ss14GC	CGGGGTGCGGCGGGGCGG	18	gp3	((((..((((.((...(((.((((..((..((....))...))..)))).))))).))))))))	0	0.76	0	0	0	0
rno-miR-6216_R-1	GATACACAGAGGCAGGAGGAGA	22	gp3	((((((..((((..(((((((((.(((((((((((........))))))))))).)))))))))..))))..))))))	0	0	0.64	0	0	0
rno-miR-6324_R-2	TCAGTAGGCCAGACAGCAAGC	21	gp3	.((..(((..((((.((((((((.((((((((.(((((.((((((((..((((.(((...)))....))))..)))).))).)))))))))))))).))))))...))).)))))...))).)).	0	0	0	2.38	0	0
mmu-miR-6377_L-5_1ss6TG	GGTCTTCGTGTCTGCATC	18	gp3	...((((.((((((((.(((..((((((..(((.(..(.(((((.....((((.((.....)).))))....))))).)..).))).))))))))).))))).))).))))...	0	0	0	0	1.06	0
hsa-miR-6513-5p	TTTGGGATTGACGCCACATGTCT	23	gp3	((((.(((((((((((....((.(((((........))))).)).))))))...))))).))))	0	0	0	1.19	0	0
hsa-miR-6753-5p_L-1R-3_1ss2AC	CCCAGGGCAGAGCAGGGC	18	gp3	..(((((((((((..(((((..((.((((......))))))((((..((((((((((...((((((.....)))))))...((((((((((......))))))))))....))))).......))))))..)).)).....))))).)))))))))))).......	0	0	0	0	0	1.96
hsa-miR-6814-5p_L-4_1ss6AT	ATGGGTGAGATGCTGCCA	18	gp3	....((.((((((((...(((((.((......))....(((.....)))....)))))...)))))))).))	0	0	0	1.19	0	0

hsa-miR-6875-3p_L-	TTCTTCCTGCCC	1	gp							
1R-3_1ss18TC	TGGCCC	8	3	....((((((((.(((((.((((((((.((..((....))..)).))).))))).))))).))...))))))	0	0	0	0	0	0.98
hsa-miR-6894-3p_R-	TTGCCTGCCCTC	2	gp							
1	TTCCTCCA	0	3	......((((((.((((.((.((((.(((.....))).)))))).))))))))))..	0	0	0	0	1.06	0
mmu-miR-6911-5p_	CTGAGTGTGTCT	1	gp							
L-4R-1_1ss12GT	GTGGCA	8	3	....((((((.((((((((.((.((((................)))).))))).))))).))))))..	0	0	0	0	1.06	0
mmu-miR-6937-5p_	AGCTCTAAGGG	1	gp							
L-1R-5_1ss6GC	CTGGGTC	8	3	(((.((((((((..(..((((((............((....)))))))).).)))))))))))).	0	0	0	0	1.06	0
mmu-miR-6970-5p_	ATGTTCAGGGC	1	gp							
L-2R-5_1ss4AT	TGGGAGC	8	3	...((..(((((.((((.((.((((((..((....))..))))))..))..)))))))))..))...	0	0	0	0	0	1.96
mmu-miR-7057-3p_	TTTGATGTCTTT	1	gp							
L-3R-1_1ss18CG	CTGCAC	8	3	..(((..((((((((((((((((((((....))))))))))).))))).)))))..)))	0	0	0	0	1.06	0
mmu-miR-7115-5p_	TCGGGGGTTGT	1	gp							
R-5_1ss17CT	GGTGCTGA	9	3	((.((((((...((..((((..((.((.((.....)).)).)).))))..))..))))))))..	0	0	0.64	0	0	0
mml-miR-7185-5p_	GATCAGGAGCT	1	gp							
L-4_1ss13TG	GCAGAGC	8	3	..((((((((((((((((((((..........))))))))))))))))))))....	0	0	0	1.19	0	0
mdo-miR-7272-5p_L	ACAGTTCTGGTT	1	gp							
-3_1ss17TC	TCGTCA	8	3	(((.((((((((((.(((((((.................))))))).))))))))))).)))..	0	0	0	0	0	1.96
mdo-miR-7279-5p_L	AGCTGGGCTTT	1	gp							
-2R-5_1ss15GC	GCTGTTGG	9	3	.(((..(((....((.((((((((.(((.......))).)))))))).)).))).)))...	0	0.76	0	0	0	0
mdo-miR-7374c-3p_	CAAAAGGAAAG	1	gp							
L-4_1ss14GA	GATTGCT	8	3	.((((((((.((.(((((.((((..........)))).))))).)))))))).)))..	0	0	0	0	1.06	0
mdo-miR-7394b-5p_	ACATGTATGCC	1	gp							
R-3_1ss12CA	AAGGTTAC	9	3	(((((((((...(((((((.((........)).)))))))...)))))))))..	0	0	0	1.19	0	0
	AGGGCTGGGCC	2	gp							
mmu-miR-7648-3p	CGGGACGCGG	1	3	((((((((((((((((((.((..(((...))))).))))))))))))))))))	0	0	0	1.19	0	1.96

mmu-miR-7687-5p_R-4	AGGCGGGGAACCTGAGGCGCA	21	gp3	.(((((((...(((((.....(((((.(.....).))))).)))))...)))))))...	0	0	0	1.19	0	0
hsa-miR-7844-5p_L-3R-1_1ss10AT	ACTAGGTCTGTGTGGTGT	18	gp3	.......(((((.((((((....(((..((.(((((((..((((...(((..((((...............))))..))))))...))))))).))..)))....)))))).)))))....	0	0	0	0	0	1.96
mmu-miR-8101_L-4_1ss16AG	AGCGGAGCCGGGGGAGCC	18	gp3	.................(((((((((((.((((((((((.(((((.(((.....(((((.....))))).....)))))).)).)))..))).)))))))))))))))	0	0	0	0	0	1.96
mmu-miR-8118_L-2R-2_1ss20AT	CAAACATGACTATGCTGT	18	gp3	....(((.......((((((((((..((((((((((((((((((((((.((...................)).))))))))))))))))))))..))))))......)))))........))).....	0	0	0	0	1.06	0
ssa-miR-8157-3p_L-4_1ss11CG	TGTGCTGTGCTGTGCTGT	18	gp3	.........((((((((((.(((....))).((((........))))...))))))))))	0	0	0	1.19	0	0
cfa-miR-8791a_L-7_1ss19CA	TGTCCTGTGCCATCCCGG	18	gp3	(((.((((((.....(((((.((.(((((.((((....(((((..((.(((((((((..((((((............))))))...)))))))))))).))))).))))))))).)).)))))....)))))).)))..........	0	0	0	1.19	0	0
eca-miR-8964_L-2R-2_1ss15GA	TGTAGAATTTAAAGAGATG	19	gp3	....((((...(((((((..((..((((((((((.((..(.((((((((((((((((((..(((.....((.....)).)))))))))))))))))))))))).)..)).))))))))))..))..)))))))......)).)).	0	0	0	0	1.06	0
eca-miR-9065_L-1R-6_1ss13TC	GGGACTGAGCCCGTAGGG	18	gp3	..((((..((((...(((......(((((.((.((((.((.((((((((.((((....)))))))).((((.(((....((((.....))))))).))))).....)))))).)))))).))))).......)))...))))..)))).	0	0	0	0	1.06	0
eca-miR-9090_R-6_1ss1TG	GGAGATTTCAGAGCCTGAC	19	gp3	..((..((...(((((.((((((.......(((.((((((..(..(((..((.(((((((((.((..((((((....))).)))..)).)))).))))).)).)))..).))))))..)))...)))))).)))))...))..))	0	0	0	1.19	1.06	0
efu-miR-9288_L-3R-4_1ss13GA	CATGTTTTCATGTTGTCT	18	gp3	.(((..((((((((((.....(((((((..(((.(((.((((....)))).)))))))..)).)))))....))))))((((....)))))....)))))...(((((((.((....)).))..))))).)))..	0	0	0	0	1.06	0
efu-miR-9298_L+1R-4_1ss13TC	TGCTGATTGTCACGTTCTGATT	22	gp3	(..(.(..((..(((((((((.(((((..(..((((.....(((((...((((....(((((..((((((((.....)))).)))..))))).))))..)))))..))))..)..))))).))))))))).))..).)..).....	0	0	0	1.19	0	0
efu-miR-9329_L-3R-2_1ss4GT	TCCTCTGGACTGTGAGGC	18	gp3	........((..((..((((.(((((..((((((((((.(((.(((..(((........)))...)))))).)))))))).....))..)))))))))..))..)).....	0	0	0	0	1.06	0
aca-let-7d-5p_R+2	AGAGGTAGTAGGTTGCATAGTT	23	gp3	...(((.((((((.((((((((((((((((.((((((((.((((((.....)))))).)).......)))))).)))))))))))))))))))))))))	0	0.38	0.32	2.98	0.53	0

	A										
aca-let-7d-3p_R+1_1ss7AG	CTATACGACCTGCTGCCTTTCG	22		gp3	...(((.((((((.(((((((((((((((.((((((((.((((((.....)))))).))........)))))).)))))))))))))))))))))))))	0	0	0.32	0.60	1.06	0
hsa-let-7d-5p_R+1	AGAGGTAGTAGGTTGCATAGTTA	23		gp3	....(((.(((((((.((((((((((((((((.((((((...((((((.....))))))..........)))))).))))))))))))))))))))))))))..(((((....))))).........	0	0.38	0.32	2.98	0.53	0
cfa-let-7d_R-3_1ss22TG	CTATACGACCTGCTGCCTTTCG	22		gp3	....(((.(((((((.((((((((((((((((.((((((...((((((.....))))))..........)))))).))))))))))))))))))))))))))..(((((....))))).........	0	0	0.32	0.60	1.06	0
oha-let-7i-3p_R-1_2ss10CA22TC	CTGCGCAAGATACTGCCTTGCC	22		gp3	.....(((((.((((((((((((((((((.(((.(((((((.(.....).))))........)))))).)))))))))))))))))))))))....	0	0.76	0	0	0	0
odi-let-7d_1ss19CA	TGAGGTAGTGGGTTGTATAGCT	22		gp3	((((((..((((....((((((..((((((.((((.(((((((....)))...)))))))).))))))..))))))....))))..).))))).......	0	0.36	0.64	1.59	0.35	0
PC-5p-242466_11	TAGTTCAGCTGTCTGTGAATGAGG	24	13	gp4	..(((..((((((...((((((((.((..(((((.(((.(((.((((((.......)))))))).))))))))..)).))))))))))))))...))).	0	3.05	1.92	5.95	10.61	1.96

Table S5. Target genes of the 191 differentially expressed miRNAs related to postnatal liver maturation which were selected with the criteria of P < 0.05 and fold changes either > 1.5 or < 0.67 and the target genes of the 32 differentially expressed miRNAs which were selected with the criteria of FDR < 0.05 and fold changes either > 1.5 or < 0.67.

creteria	miRNA	genes
FDR <0.05	rno-miR-122-5p_L+1R+1_2	TRIM39\TCF25\TBC1D22B\CCL4\WBSCR16\RABGAP1\CREB3L3\PLLP\ZAP70\PKM2\PRKCD\POLR2D\NCOA4\NR2E3\DTWD2\GK5\WDR7\MLX\MTOR\LOC100859426\CHD9\PAX7\ADAM10\P4HA1\LOC424430\LOC428693\SH3GLB2\MTHFR\SCG3\PRPS1\DUT\RHBDD1\PTGS2\TRIM37\PLAU\CLTC\SLC25A36\DHRS11\TAF1\YTHDF1\GCLM\FARP2\C12H3ORF19\FRA10AC1\AP2A2\FAM174B\BDH1\PGS1\UTS2D\MASP1\TIPIN\LRRC59\TMEM104\C17ORF28\CALML4\UBE2V1\TMEM209\PAK3\GDI2\SDSL\NET1\TRDMT1\ITGA8\SPTBN5\VPS39\LBH\DPF3\CAPZA2\GXYLT1\PROX1\DTL\TRAF5\TMBIM4\FAM177A1\MBNL1\LOC395824\LEF1\PPPDE1\BZW2\SNX10\AGPAT9\DYNC1LI1\CKB\SCYL2\UBA6\C22ORF32\CCR8\RTN1\ANKRD54\ISCA1\FANCC\LOC420864\ESR1\USP18\GALNT1\CNDP2\NPEPPS\DLC1\SLMO2\VPS45\TACR1\PHC1\KLB\LPCAT3\CERS3\SMCHD1\SH3BP2\FABP5\TP53INP1\CSMD3\FBXO32\CALB1\TUBGCP5\UBAC2\IPO5\IRG1\SLC46A3\N6AMT2\ARL6IP5\DPT\GTPBP1\CCDC41\CHAD\GAL7\SUCLG1\SLC20A2\AK6\MCM6\UBAP1\NME2\CLSTN1\LOC419404\FLNB\TGFB3\ARHGAP15\CYP24A1\TMEM120B\DDX19B\RAPGEF6\AP4B1\WRAP73\P4HA1\
FDR <0.05	rno-miR-122-5p_L+1R+1_1	TRIM39\TCF25\TBC1D22B\CCL4\WBSCR16\RABGAP1\CREB3L3\PLLP\ZAP70\PKM2\PRKCD\POLR2D\NCOA4\NR2E3\DTWD2\GK5\WDR7\MLX\MTOR\LOC100859426\CHD9\PAX7\ADAM10\P4HA1\LOC424430\LOC428693\SH3GLB2\MTHFR\SCG3\PRPS1\DUT\RHBDD1\PTGS2\TRIM37\PLAU\CLTC\SLC25A36\DHRS11\TAF1\YTHDF1\GCLM\FARP2\C12H3ORF19\FRA10AC1\AP2A2\FAM174B\BDH1\PGS1\UTS2D\MASP1\TIPIN\LRRC59\TMEM104\C17ORF28\CALML4\UBE2V1\TMEM209\PAK3\GDI2\SDSL\NET1\TRDMT1\ITGA8\SPTBN5\VPS39\LBH\DPF3\CAPZA2\GXYLT1\PROX1\DTL\TRAF5\TMBIM4\FAM177A1\MBNL1\LOC395824\LEF1\PPPDE1\BZW2\SNX10\AGPAT9\DYNC1LI1\CKB\SCYL2\UBA6\C22ORF32\CCR8\RTN1\ANKRD54\ISCA1\FANCC\LOC420864\ESR1\USP18\GALNT1\CNDP2\NPEPPS\DLC1\SLMO2\VPS45\TACR1\PHC1\KLB\LPCAT3\CERS3\SMCHD1\SH3BP2\FABP5\TP53INP1\CSMD3\FBXO32\CALB1\TUBGCP5\UBAC2\IPO5\IRG1\SLC46A3\N6AMT2\ARL6IP5\DPT\GTPBP1\CCDC41\CHAD\GAL7\SUCLG1\SLC20A2\AK6\MCM6\UBAP1\NME2\CLSTN1\LOC419404\FLNB\TG

		FB3\ARHGAP15\CYP24A1\TMEM120B\DDX19B\RAPGEF6\AP4B1\WRAP73\P4HA1\
FDR <0.05	pma-miR-456_R+1_1ss22AC	YTHDC2\STARD4\KCTD9\DPAGT1\HMBS\ADAM28\SPPL2B\PADI3\STRADA\RBBP5\GDPD4\WWP2\FBXW2\CSNK2A2\NUDC\WDTC1\MTMR4\STRBP\SLC25A1\APLP2\FAM18B1\UBL7\ATG16L1\ROGDI\WDR33\HIBCH\GJA4\SELP\TMEM170A\CPS1\MLX\SFRP1\MED27\PAX7\DNMT3A\FNBP1\STK4\RIC8A\DOLPP1\SGPL1\CD74\AGTRAP\GNAI2\CLIC2\MYEF2\C2H3ORF75\INTS2\CDH5\RRP1B\APPBP2\AATF\HPS4\COL9A3\SBF2\XBP1\BTBD1\SMTNL2\GSTT1\OSBPL5\PDPK1\VIPR2\ZMYND11\CTNNBL1\BDH1\RNF185\KIFAP3\MAFG\RAB35\LPP\RAB22A\RRP12\PCK1\TMEM104\ATP7A\SF3A1\SLC26A4\UBE2V1\CD82\ERLEC1\ORC5\NT5C2\NR1H3\DDB2\ADAM23\ITPKA\C3H20ORF3\KNG1\LRSAM1\DNAJC10\C6H10ORF118\EDF1\ETFDH\C2H7ORF64\HCLS1\BRE\FAM177A1\NFKBIE\TMED10\LOC395824\MEMO1\SCIN\INADL\KANK4\SNX10\AGPAT9\KAT2B\GADL1\SEMA5B\MYLK\SEPT7\TAB1\ATP5S\MMADHC\MPST\PWP1\LOC100857286\MYO10\WNK1\BID\DERA\GALNT1\CCDC127\MBP\MVK\ARFGAP3\FOXJ2\ZCCHC4\MYH10\GPR98\RABL3\PLIN2\HJURP\PPP2R2C\ABLIM2\HSDL2\FABP5\PRADC1\NOV\COL22A1\NT5C1B\KRTCAP3\ASXL2\REPS2\MSL3\GAL10\N4BP2L2\SLC46A3\WASH1\PICK1\HIGD1A\CHAD\XRCC3\AK6\SUMO3\FSCN1\SLC9A4\EGFR\RAD17\ACSF2\HNF1A\MON2\XIRP1\LOC422308\WDR43\LOC427826\PYGB\CYP2H1\PNPLA7\EPS8L2\
FDR <0.05	PC-3p-68190_58	NFU1\TLK2\RANBP3\CHD6\NCAPD3\TNFRSF18\PMPCA\TRIM33\TMEM194B\ENPP3\TMED2\IFT81\CEP112\ITM2A\NADSYN1\RNF111\LOC100859686\ATXN2\C5H15ORF57\FLCN\DKC1\PLCG2\TNFAIP1\TMEM41B\HPRT1\MOSPD1\OSBPL5\HTATSF1\SEMA3D\APOD\TLR4\ALDH1A3\RAB3IL1\SSRP1\KIAA0226\MTMR8\CALML4\C15H12ORF49\SHOC2\UGP2\WDR31\ACBD3\LOC431656\SLC39A9\MARC2\PPP2R5A\GEMIN2\INTU\ALKBH1\PAPSS1\GPX7\EXO1\PPAP2B\ITPK1\UBR7\SP4\WLS\RFTN1\ERCC3\UHRF1BP1L\MRPL3\CLEC3B\CLASP2\SFRP4\C5H14ORF101\METAP1\EPC2\MMADHC\ISCA1\ATP9B\LOC101750080\BLOC1S5\CDH20\RECQL\CLEC3A\CYP4V2\AGMAT\FUCA2\KIF2A\MTX3\PARP8\HEXB\MLLT3\ZDHHC23\PPP2R2C\MXD4\PHF20L1\DMD\KLHL15\PRDX4\LPIN1\RGN\CHST10\PDCL3\ARGLU1\SLC25A15\HMGB1\UBL3\COTL1\DPT\INVS\LOC776051\LOC422002\SRD5A3\NSDHL\AK6\PLA2R1\RNF7\FYB\RBM6\POU1F1\CHRNA6\SLC10A7\OAT\CD151\TBC1D22A\COG5\MAGI2\ANG\CD320\
FDR <0.05	oan-miR-363-3p_R+1	ARMC1\STARD4\NEFM\SPPL2B\CDC27\CNTN2\WWP2\SLC41A1\LOC100857226\TPRG1L\SRPR\C20H20ORF24\TMEM57\KBP\C4BPA\PPP2R3A\PIK3R5\SERPIND1\GSN\RAB14\CCNL2\ATP6V1B2\ACSBG2\C15H22ORF25\ACOX1\PLEKHB2\HSD17B4\HIBCH\C10ORF58\KCTD7\BMS1\TRIP12\MMD\EIF2B1\KIAA1191\BSDC1\BARD1\MYPN\AP1M1\KDM4B\ITM2A\RIC8A\ADAM10\FAM20B\PLG\SHISA5\BUD31\RGL1\PPIF\MPP1\C8H1ORF27\CLTC\INTS2\BCAS3\PPIC\GATA5\PIK3CB\SYNRG\PNAT10\ST6GAL1\DKK3\RNF141\XBP1\PAFAH1B1\ABTB1\HPRT1\OSBPL5\ATP12A\KIAA1024\TOLLIP\CHKA\BRWD3\ERGIC2\UTS2D\SYNPR\ZDHHC5\PRKAB1\CLCN7\MTMR8\RAB11A\CHIC1\ZWILCH\CSTF1\CBLN4\BCL11A\GTF3C3\SF3B1\COQ10B\RTN4\WSB2\C15H12ORF49\NEURL\FGL2\PHTF2\GRIA3\DUSP5\ADAM23\G

		TF2H4\CSRP2BP\SHOC2\WASL\VPS54\FXR1\PIGK\TMEM87A\TASP1\NVL\CRAMP1L\GUCY1B3\PRKCI\KRIT1\CAV2\MIA3\DUSP10\TMEM229B\SLC25A12\MSMO1\METTL8\COL1A2\GPR26\LPGAT1\GNS\ATP6V0B\GPR149\LOC424620\AGXT2L1\SGMS2\HEATR5B\ACSL1\PODN\GOLGA5\PPAP2B\NUDT9\ROR1\LEPROT\HACL1\AGPAT9\PPP1R13B\HIPK3\KIAA1109\ACO2\CCR8\TOB2\PCDP1\HERPUD2\ZRANB3\LRRFIP2\TRIP13\BAZ2B\ISCA1\SLC37A3\CCDC127\PYROXD1\C3AR1\TMF1\UBA3\FAM149A\MTHFD2\CTRC\HBS1L\NUP50\CSTB\FBXO4\RABL3\FAM26E\NSG1\C9ORF93\GNE\FNTA\PDZK1\TCEB1\FABP5\SLC17A5\OTUD6B\THNSL2\FAM135A\RAD21\PSCA\SIK1\RAB23\ASXL2\PIGA\MTMR9\RP2\TNFSF13B\LIG4\DNAJC3\MZT1\UFM1\CRYL1\NOX4\LOC426128\ITM2B\PDGFRL\AK6\C7H2ORF76\LOC419830\OVAL\PSMC6\GOLGA4\HOXA11\CDCA7L\DGKZ\CNTN1\PGRMC1\ATP2A2\CYP2H1\SUPV3L1\
FDR <0.05	mmu-let-7j_1ss8TG	RDM1\KCTD9\GALK2\CYB5B\HBEGF\STRBP\LOC428335\ST14\CNTRL\NFS1\HEXDC\LRIG2\TMEM194B\GMPPB\PCTP\UBIAD1\OSMR\TARSL2\TNFRSF1B\P2RX5\PDCD5\MAPK6\PTGS2\DEAF1\EPHA4\SLC25A36\USP32\PNAT10\ABCD3\TMED5\KLHL13\KIAA0753\ZNF326\SRPRB\IL13\MAP3K8\ITGB2\MAGT1\EIF4E2\TMEM164\C6H10ORF32\TYRO3\SNAP23\ETFDH\C5H15ORF29\COL1A2\ACADSB\C5H15ORF41\WDR65\EAPP\BRE\BTBD9\ABCD4\MRPS18A\SMARCAD1\ICA1\BZW2\IGF2BP3\GLRX5\BCS1L\EXOC3L4\TNFAIP2\TSN\IL8L1\ATF7IP\SERHL2\C22ORF32\SLC12A8\CEP70\ADH1C\STK17A\TGFBR1\RBFA\LOC420849\GNPTAB\AKR1D1\SERPINB10\WNK1\PBX4\SLMO2\C2H18ORF19\MAP3K5\CHD4\POC5\GTF3C6\UHRF2\LNP1\NANS\RARS2\SYNCRIP\CCNE2\ST3GAL1\DMD\PIGA\GAL10\MGAT4A\COL4A2\TGDS\WDFY2\DDI2\HSD3B2\LOC420301\E2F5\TERF1\CZH9ORF80\HABP2\DNTT\BZW1\GLT1D1\CRTAP\GATM\GCSH\MYO19\DDX19B\EIF4G2\C5H11ORF46\YOD1\PDZD11\ARHGEF10L\STK40\TMEM130\
FDR <0.05	gga-miR-6710-3p_R+1	RFK\ARMC1\STARD4\SPPL2B\FAM125B\ARMC8\RAB14\ZZEF1\CAMSAP1\EIF6\VAV3\NCBP1\NR5A2\CSNK1G1\MAPKAPK3\PAIP2\C19H17ORF85\BRD3\NCKAP1\SARDH\CPPED1\PEF1\SUB1\KHDRBS1\MFAP3\CSRP3\SRSF10\YWHAB\NADSYN1\RNF111\URI1\PLOD1\CELA2A\ATXN2\PDCD5\RGS9BP\CEP89\DEAF1\TAF4\AP1S3\CDH11\APPBP2\ARHGEF3\ZNF598\RNF141\ABCD3\TNFAIP1\YTHDF1\DCAF12\PAFAH1B1\TNRC6A\DDT\ANKRD1\TOLLIP\GTPBP4\ARL6IP1\KIF11\FAM174B\CHM\SPPL3\LOC100858827\ALDH1A3\PGS1\FTH1\NPEPL1\RAB22A\CHIC1\KCTD2\GTF3C3\ACTR1A\FGL2\F2\LOC417741\TRDMT1\PRKCI\PRDX3\CAPZA2\FNIP2\EIF2S1\C5H15ORF29\GFM1\C1GALT1\GNS\ARHGAP10\GEMIN2\ZDHHC17\TXNDC12\GLCCI1\DHCR24\TRA2A\KITLG\PPP2R5C\KERA\MSH4\AMPH\LUC7L2\CEP70\PAX6\CASP6\AVL9\WDSUB1\TXNRD1\SSR1\DGKI\C6ORF97\LOC418170\DERA\SOGA2\FAM114A1\NPEPPS\GTF2H5\C2H18ORF19\CYB5R3\TEC\KIAA0930\RELL1\ERAP1\FAM26E\F2RL1\GTF3C6\QRSL1\NFKBIZ\CD200\FBXL4\TMEM129\SYNCRIP\GRIK1\MMP16\OTUD6B\CCNE2\MORC3\HMGN1\U2AF1\ADCY8\SYAP1\PIGA\INPP4A\ELF1\CCNA1\NUPL1\CRYL1\DYNC2H1\MMP1\SUV39H2\B4GALT1\TPD52L2\PTGR2\UNG\LOC415708\GCOM1\ALDH1A2\NCF1\BIRC2\AZIN1\HPCAL1\EGFR\CRTAP\RAD17\RNASET2\NSA2\GABPB1\EMB\ADAL\WDR43\EIF4G2\LIN7B\GNB1\GFOD2\LOC770653\

FDR<0.05	gga-miR-489-3p_L-2R+1	RASSF2\WDR36\RPS6KA1\SPPL2B\PM20D1\C11ORF30\ARHGEF16\UBE2L3\VAV3\PEX12\NR5A2\LOC427001\DTWD2\TRAPPC3\MCCC2\RGS4\RAN\INPP5K\RFNG\ARID5B\GBGT1\TMEM38A\IQCA1\MFAP3\CHMP1B\RIC8A\TTPAL\GOSR1\PSMD9\ASB6\DIABLO\NPFFR1\URI1\SGPL1\NCOA3\P2RX5\AKAP10\C14H17ORF103\ADK\FAM120A\RRP1B\PTH\DHRS11\SNAP47\CCDC12\RNF141\C20H20ORF11\TMEM97\GCLM\SLC13A5\NDUFAB1\OSBPL5\CAMLG\UBE2I\PM5\IL13\MARCH5\CHM\DLG1\IGF1R\KDELR2\KIF21A\ACOX2\OPA1\PGS1\RAB35\LOC416354\CHIC1\EGR1\CD44\CHL1\EIF4E2\RNF114\UQCR10\CHRDL1\ACP2\ORC2\GSTO1\MRPS25\GTF2H4\B3GNT2\PIGK\YPEL5\SP3\PRKCI\LOC423256\TMEM229B\CCDC132\TLK1\MYO3B\C5H15ORF29\CHST15\SHFM1\RAB33B\PPP2R5A\NKTR\TMEM184C\GEMIN2\TBC1D15\YIPF3\SNW1\LEF1\ACSL1\SCIN\ALG6\PGM1\MIER1\HHIPL1\AGPAT9\TMEM66\TNFAIP2\SH3BP5\SEC24B\IL8L1\LZTFL1\JOSD1\SOX10\HECW1\MMADHC\PKP4\C1H12ORF48\WRNIP1\ANKRA2\FAM76A\UCHL1\PHYH\PRRC1\KIF2A\SERINC1\ZBTB49\NSG1\CYP7A1\GJA5\CASP8AP2\PEX2\WHSC2\LYRM2\ORC3\SLC17A5\PTDSS1\LY6E\DDX3X\DMD\GCM1\APOO\GRHL1\LOC421978\DNAJC3\WDFY2\ELMOD1\FAM76B\CASP2\POT1\HIGD1A\LOC101751006\PPP1CB\SUCLG1\INCENP\ZP4\CDC42\ROS1\ZC3H14\ARHGAP15\HOPX\PON2\OAT\LOC100859627\GATM\GLT25D2\GRIN1\TUBGCP2\C18H17ORF67\NMB\
FDR<0.05	gga-miR-458a-3p	TCF25\PRNP\STRADA\EYA3\SLC7A6\C20H20ORF24\SAMHD1\NT5DC2\NFS1\ORAI2\TEK\OPN4\RERE\HIBCH\SORD\DCXR\UPF1\AIMP2\TAF8\SKP2\CNOT8\DHRS3\DCTN4\ECM2\SCG3\PRPSAP2\DPM2\PLAU\SERPINE2\PPP3CB\USP32\JMJD4\LOC768701\TCOF1\ADIPOQ\SYNGR3\WSB1\HIF1AN\ARFGAP1\DCTN5\SKP1\PDE6C\HSPA14\MAPRE1\RNF185\APIP\UACA\DDB2\DSTN\TRDMT1\B3GNT2\NHLRC2\LEFTY2\KRIT1\GAD1\SSB\MCFD2\FAM177A1\GTF2A1\DHCR24\NUDT9\INADL\WDFY3\BAG5\PTPLB\FSHB\SHPRH\ISCA1\EEF1E1\DERA\ANP32E\MTUS1\NPEPPS\PPAT\FAM38B\FGFR1OP2\RELL1\SEPSECS\ZYX\EPHB6\ST3GAL6\EPHA7\UBE2J1\ELOVL4\RAD21\MED14\FBXO9\MZT1\RNASEH2B\PAN3\GZMA\SLC25A14\PTGR2\CAPN3\SYK\RGS1\FYB\GLT1D1\NFYC\TRIB2\PDLIM5\LPL\GYPC\CUL2\PARP1\ATP2A2\ACE\HSF1\
FDR<0.05	gga-miR-456-3p_R-1	YTHDC2\STARD4\KCTD9\DPAGT1\HMBS\ADAM28\SPPL2B\PADI3\STRADA\RBBP5\GDPD4\WWP2\FBXW2\CSNK2A2\NUDC\WDTC1\MTMR4\STRBP\SLC25A1\APLP2\FAM18B1\UBL7\ATG16L1\ROGDI\WDR33\HIBCH\GJA4\SELP\TMEM170A\CPS1\MLX\SFRP1\MED27\PAX7\DNMT3A\FNBP1\STK4\RIC8A\DOLPP1\SGPL1\CD74\AGTRAP\GNAI2\CLIC2\MYEF2\C2H3ORF75\INTS2\CDH5\RRP1B\APPBP2\AATF\HPS4\COL9A3\SBF2\XBP1\SMTNL2\GSTT1\OSBPL5\PDPK1\VIPR2\ZMYND11\CTNNBL1\BDH1\RNF185\KIFAP3\MAFG\RAB35\LPP\RAB22A\RRP12\PCK1\TMEM104\ATP7A\SF3A1\SLC26A4\UBE2V1\CD82\ERLEC1\ORC5\NT5C2\NR1H3\DDB2\ADAM23\ITPKA\C3H20ORF3\KNG1\LRSAM1\DNAJC10\C6H10ORF118\EDF1\ETFDH\C2H7ORF64\HCLS1\BRE\FAM177A1\NFKBIE\TMED10\LOC395824\MEMO1\SCIN\INADL\KANK4\SNX10\AGPAT9\KAT2B\GADL1\SEMA5B\MYLK\SEPT7\TAB1\ATP5S\MMADHC\MPST\PWP1\LOC100857286\MYO10\WNK1\BID\DERA\GALNT1\CCDC127\MBP\MVK\ARFGAP3\FOXJ2\ZCCHC4\MYH10\GPR98\RABL3\PLIN2\HJURP\PPP2R2C\ABLIM2\HSDL2\FABP5\P

		RADC1\COL22A1\NT5C1B\KRTCAP3\ASXL2\REPS2\MSL3\GAL10\N4BP2L2\SLC46A3\WASH1\PICK1\HIGD1A\CHAD\XRCC3\AK6\SUMO3\FSCN1\SLC9A4\EGFR\RAD17\ACSF2\HNF1A\MON2\XIRP1\LOC422308\WDR43\LOC427826\PYGB\CYP2H1\PNPLA7\EPS8L2\
FDR <0.05	gga-miR-456-3p_1ss22AT	YTHDC2\STARD4\KCTD9\DPAGT1\HMBS\ADAM28\SPPL2B\PADI3\STRADA\RBBP5\GDPD4\WWP2\FBXW2\CSNK2A2\NUDC\WDTC1\MTMR4\STRBP\SLC25A1\APLP2\FAM18B1\UBL7\ATG16L1\ROGDI\WDR33\HIBCH\GJA4\SELP\TMEM170A\CPS1\MLX\SFRP1\MED27\PAX7\DNMT3A\FNBP1\STK4\RIC8A\DOLPP1\SGPL1\CD74\AGTRAP\GNAI2\CLIC2\MYEF2\C2H3ORF75\INTS2\CDH5\RRP1B\APPBP2\AATF\HPS4\COL9A3\SBF2\XBP1\SMTNL2\GSTT1\OSBPL5\PDPK1\VIPR2\ZMYND11\CTNNBL1\BDH1\RNF185\KIFAP3\MAFG\RAB35\LPP\RAB22A\RRP12\PCK1\TMEM104\ATP7A\SF3A1\SLC26A4\UBE2V1\CD82\ERLEC1\ORC5\NT5C2\NR1H3\DDB2\ADAM23\ITPKA\C3H20ORF3\KNG1\LRSAM1\DNAJC10\C6H10ORF118\EDF1\ETFDH\C2H7ORF64\HCLS1\BRE\FAM177A1\NFKBIE\TMED10\LOC395824\MEMO1\SCIN\INADL\KANK4\SNX10\AGPAT9\KAT2B\GADL1\SEMA5B\MYLK\SEPT7\TAB1\ATP5S\MMADHC\MPST\PWP1\LOC100857286\MYO10\WNK1\BID\DERA\GALNT1\CCDC127\MBP\MVK\ARFGAP3\FOXJ2\ZCCHC4\MYH10\GPR98\RABL3\PLIN2\HJURP\PPP2R2C\ABLIM2\HSDL2\FABP5\PRADC1\NOV\COL22A1\NT5C1B\KRTCAP3\ASXL2\REPS2\MSL3\GAL10\N4BP2L2\SLC46A3\WASH1\PICK1\HIGD1A\CHAD\XRCC3\AK6\SUMO3\FSCN1\SLC9A4\EGFR\RAD17\ACSF2\HNF1A\MON2\XIRP1\LOC422308\WDR43\LOC427826\PYGB\CYP2H1\PNPLA7\EPS8L2\
FDR <0.05	gga-miR-3529	ARMC1\ELF3\TBC1D22B\HBEGF\POC1A\RRAGC\ATAD3A\MAN2C1\PSMD5\STYXL1\ABCA3\PLEKHB2\TRAPPC3\TSPAN14\DNAJC18\C19H17ORF85\SARDH\TRIP12\ARMC6\PINK1\DDX49\ACSS2\TAF8\ZBTB8OS\POLB\C17H9ORF78\TOR1A\BET1L\DPP9\MCU\ANAPC16\SH3GLB2\PPTC7\RGS9BP\TSEN15\MTFR1\DUT\NDST2\SLC25A36\TNFAIP1\TRAF7\MCM2\KIF1A\CAMLG\OAF\KIF3A\IDE\LIMK2\VIMP\ATP13A4\RAB3IL1\RAB35\TMLHE\CTSZ\MTMR8\LOC416354\KDM3B\SNAPC5\SALL4\CD44\GGA3\CELF1\RINT1\ABHD12\SLC39A12\PPP3R1\PIF1\GANC\LBH\TASP1\ENTPD2\ZFP106\SLC39A9\ETFDH\GXYLT1\MPP5\IRAK4\SRP14\THBS1\ACADSB\CLCN3\MYST2\NAP1L1\ABCC10\PDZK1IP1\RAVER2\SERPINH1\FRMD1\CRADD\RPS6KA2\NEDD1\SCYL2\COX18\DDX47\ATF7IP\LOC425756\METTL14\MAP4K5\RBFA\TDG\ECI2\BID\PPIE\STX17\PI4K2B\WBSCR22\GDAP2\CZH5ORF28\METTL16\ZUFSP\MLLT3\ZFAND5\XCL1\PCNP\GPR89B\RNF20\CHMP3\DSCR3\KLHL15\APOO\FBXO32\MXRA5\CD2AP\CYFIP1\CDC16\HMGB1\MTMR2\SUV39H2\YWHAZ\FAM125A\POPDC2\ETV1\SLC20A2\SLC25A14\ITGA1\KCNAB1\ATP6V0E2\DNTT\PLEKHA3\ZP4\TCEB3\WDR82\LLPH\ATP1B4\TBC1D22A\NUCB2\NUP188\YOD1\GRIN1\GFOD2\KCNMA1\MAP3K3\PDZD11\PCSK7\
FDR <0.05	gga-miR-301a-5p_L+2	RFK\TRIM39\BNIP3L\ZNF593\SLC7A6\FBXW2\NUDC\HES5\WBSCR16\SYF2\SNAP29\LOC428335\MYBBP1A\ADRA1B\IRF1\ZZEF1\ATP6V1B2\APTX\PRKCD\RFFL\DTWD2\MSTN\C10ORF58\KTN1\TRPC1\PLS1\LRRC45\NRG4\TRPC4AP\ITPR3\NFYA\DNAJC7\SFRP1\GOLGA7\BARD1\SKP2\FEN1\KRT15\TRIT1\CSRP3\FAM114A2\FNBP1\SRSF10\ADA\C28H19ORF10\XPO1\ASB6\SH3GLB2\CPSF4\GLE1\SLC12A1\PLAU\HMCN1\FAM120A\CDH11\AATF\PDLIM1\SLC44A3\YTHDF1\SPPL2A\DCAF12\ALOX5\LOC420419\MYD88\RRAS2\DDX26B\CR

		TAM\PDE6C\ABCC1\VPS8\IL13\CHM\PPP1R2\ALDH1A3\ZFYVE27\STAT1\APIP\ATRX\MAGT1\EIF4E2\PIAS1\WSB2\ORC2\MED13L\EHMT1\MAL\EEF1B2\SHOC2\COMMD5\TTC14\JAG1\PIF1\CDC42BPA\SEC23IP\CCDC132\SLC25A12\LOC417800\GPATCH2\C5H15ORF29\ACADSB\SHFM1\RASSF3\DIEXF\FAM177A1\AKR1A1\GPR149\CMPK1\YIPF4\SLC30A6\DPY30\SCIN\ITPK1\UBR7\OMA1\HOOK1\GALNT3\SNX10\LEPROT\ABCB10\PKDCC\BCS1L\BIN1\AMPH\PTPLB\TTC26\MAD2L1\ANK2\PLA2G12A\LRRFIP2\SLC9A3\PWP1\EEF1E1\TXNDC5\MED24\RSPH3\MVK\NUP50\FBLN1\PDS5A\RELL1\SEPSECS\GOLPH3L\FAM172A\CRHBP\BRP44\TRIM55\SOHO-1\WHSC2\FILIP1\PHF20L1\KLHL15\CALB1\PIGA\RP2\C1H2ORF49\ARHGEF7\MZT1\MAB21L1\B4GALT1\CLN8\KLHDC2\CAPN3\SUMO3\CIB3\KPNA1\ERICH6B\RSPO3\TGFB3\HNF1A\SLC1A2\XIRP1\ATP1B4\DDX19B\ERBB4\LIMD2\SLC9A9\KIAA0319L\
FDR <0.05	gga-miR-20b-5p	BNIP3L\IPO9\SPPL2B\CAMTA1\C28H19ORF28\C11ORF30\PPP6C\SAMHD1\WDR16\CAMK1G\RALGAPB\CYB5D2\IRF1\NFS1\ATG16L1\PSMD5\MAK16\RPAIN\SCAMP2\CAMSAP1\MAT2B\PRPSAP1\SDF4\SLC25A24\ARVCF\FAM86A\ACOX1\FBXW11\NUP35\DUSP19\MYO1C\LOC100859747\WDR59\RASA2\BECN1\SCARB1\C26H6ORF130\VAMP4\DDX5\LPCAT2\KPNA2\AKTIP\SALL1\GPR107\INTS1\TTPAL\C28H19ORF10\RCAN3\SEC16B\C11H19ORF12\SH3GLB2\ZMYND8\SGPL1\LOC100859686\MAPK6\SET\ULK2\TMEM116\AIFM2\RASD1\C17ORF39\GPI\SHC4\PTGS2\C8H1ORF27\TRIM37\MYO1H\FGD3\BICD2\EPHA4\SNX2\PTH\BTBD10\ARHGEF3\ST6GAL1\C20H20ORF11\PAFAH1B1\TMED5\WNT8A\MOSPD1\TSG101\CXCL14\OSBPL5\ANKRD1\GRM3\SORL1\MAPRE1\YWHAH\PFKP\USP28\CREM\ALDH1A3\ARHGAP12\RNF166\SYNPR\CDA\ACBD5\RAB22A\KIAA0226\CHIC1\USP12P1\STK17B\SLC26A4\SPATA2\KIF23\CELF1\ATG4A\NAPEPLD\FAM185A\PHTF2\STRADB\CRY2\BMPR2\ABHD12\TUBGCP4\EEF1B2\CXORF56\PPP3R1\CTBS\PPP1R21\NRK\C4HXORF57\VMA21\KIAA0922\C3H1ORF55\C6H10ORF46\AKAP9\GUCY1A3\KRIT1\CAV2\SMOC1\BAG3\SLC39A9\TMEM229B\BUB3\PTPN14\RAB33B\PROX1\LPGAT1\GAB1\IFNG\CFL2\BRMS1L\VLDLR\NPC2\KIAA0494\VIPAR\CMPK1\ALKBH1\EPS15\SLC25A4\ORC1\C4H4ORF41\AGA\TMEM59\SLC24A4\RYR2\AHR\SPC25\RAPGEF5\SERPINA5\LYST\DFNA5\PGM1\AFF1\COBLL1\SMOC2\KAT2B\CRADD\LHX8\TECPR2\ELK3\ERCC3\ANKS1B\BCL2L14\IL8L1\MYLK\DDX47\SIDT2\RPL7L1\PRRG4\SLC12A8\OSBPL11\SFRP4\DTX3L\NAA30\NCKAP5\ZRANB3\UBXN4\SHPRH\TDRD7\DARS\C3H6ORF72\GTDC1\DDHD1\SOUL\FBXO7\LOC101750080\MKRN1\SERPINB2\CLUAP1\STX17\CNOT7\NPEPPS\CYB5A\CEP192\C2H18ORF19\SGCB\FAM76A\SYTL2\OTUD3\SCNN1A\ANKRD52\KLB\ITFG2\RELL1\SEPSECS\CSTB\FBXL5\PHYH\TNKS2\LYSMD3\ARSK\KIF2A\MTX3\PAPD4\WASF1\B4GALT4\PCMTD1\LACE1\KIAA1524\TMEM175\IDUA\ZFP92\USP45\FABP5\CHMP3\LRRCC1\OTUD6B\TMEM67\IFNAR1\IFNGR2\TP53INP1\SIK1\MED14\PRIM2\RAB23\PXDN\HAS2\MSGN1\NT5C1B\LAPTM4A\MCM3\UXS1\RASA3\COL4A1\LIG4\TPP2\POSTN\CCNA1\SLC46A3\NPAT\KLHL5\CCDC41\E2F5\PTPN3\NR1D2\ETV1\CLN8\PPP6R3\ATE1\PLEKHA3\RABGAP1L\ALDH1A2\NOS2\GLT1D1\NADK\TMEM123\POU1F1\ROS1\PAPOLA\PDLIM5\ACSF2\SLC1A2\DOCK4\COL17A1\ATP2A2\SLC47A1\LDHA\NUCB2\PDE3B\NBL1\PTPN22\LIN7B\C5ORF5\CR1L\YOD1\PDZD11\MAP3K14\MBL2\ATCAY\LEPROTL1\

FDR <0.05	gga-miR-202-5p_L-1	RFK\C22H20ORF30\KCTD9\ERMP1\SPIRE2\HNRNPR\KBP\SCLY\SNAP29\LOC428335\SF3A3\ZZEF1\PPIL2\UBAC1\JMJD6\CRYM\RGS4\PIK3CD\ANXA11\CRIP1\PINK1\TFEB\BARD1\ADC\ADA\RCAN3\COL13A1\RGS9BP\SET\OPNP\ALKBH5\FGD3\ABCD3\PLS3\ARHGAP17\FAM122A\ATP12A\CLMP\KIF3A\PFKP\BLNK\CLPX\FYTTD1\EIF4E3\MRPS7\ZC3HC1\NEURL\ATG4A\AP2M1\IDH1\SAMD13\PIK3CA\GXYLT1\GPATCH2\C1GALT1\USP15\PREPL\CRIPT\CNOT2\GPR149\CMPK1\ACSL1\C4H4ORF41\DHCR24\PLCL2\EPYC\KERA\NGLY1\CDC42BPB\PECR\NR1H4\LZTFL1\HIF1A\TRPC3\KIF15\TMC2\CCDC93\METAP1\KLHDC1\ATL1\MTHFD1L\SOUL\HMGXB4\ISCA1\TGFBR1\RBFA\PPFIBP1\PRR5\SLC34A2\ARSK\KIF2A\SERINC1\CRHBP\TPPP2\GPR161\NANS\RNF4\WHSC2\CA2\MMP16\NOV\PTP4A1\FBXO9\HAS2\FAM49A\C3H8ORF80\C1H2ORF49\DNAJC3\IRG1\WBP4\HMGB1\LOC421975\MCPH1\CAPN3\UBASH3A\WDR35\TMED8\TMPO\DGKZ\CUL2\HOXD4\ADAL\RAD50\LOC419851\
FDR <0.05	gga-miR-181b-5p_R+1	LOH11CR2A\RASSF2\LSM7\STRADA\ACOT7\PAK1\FAM125B\CCDC43\C20H20ORF24\C4BPA\ARMC8\SAMHD1\CLTCL1\CNTRL\WDR45L\NFS1\PSMD5\ATP6V1B2\ACSBG2\WWC1\GNB1L\TRIM33\STXBP3\PRKCD\C15H22ORF25\LOC415324\AGO3\DTWD2\GOT2\GBAS\C10ORF58\UCHL5\PIK3CD\LZIC\C19H17ORF85\ATR\ZC3H15\GK5\UHMK1\DNAJC12\C26H6ORF106\RASA2\PHACTR4\TARDBP\SUGP2\MMD\ACSS2\BARD1\IFT52\LPCAT2\GAS2\TOP1\LIFR\SUN1\SLC35E1\P2RX4\MYPN\FAM189A1\IQCA1\STK4\PNRC2\SRSF10\RNF111\GOSR1\SGPL1\P2RX5\MAD2L2\C5H15ORF57\PDAP1\RGL1\NOL8\COPS3\DUT\MKRN2\TRIM37\VCAM1\SLC25A36\GNG13\SASS6\APPBP2\ADAMTS18\JMJD4\LPPR5\ARHGEF3\LOC768701\SYNGR3\RNF141\TSC2\KLHL36\PITPNB\EMC8\SPPL2A\GCLM\RAP1GAP2\MTF2\KLHL13\ABTB1\KIAA0753\MOSPD1\PRKAG2\MLL3\CXCL14\PDPK1\SRPRB\SEMA3A\KIF3B\FRA10AC1\BRSK2\TOLLIP\TAF3\PLOD2\IL13\CHKA\RPTOR\ARRDC4\ALDH1A3\RAB35\MTPAP\ABCC2\ARCN1\TMLHE\ABCC3\STX16\MTMR8\LOC416354\EME1\ZWILCH\SALL4\GGA3\ACSL4\CELF1\MTAP\PDCD11\LRRC17\PHTF2\SMARCA1\SENP5\LAMP2\GTF2H4\LOC424109\PPP3R1\C1D\IDH1\ATP6AP1\LCLAT1\CDC42BPA\C3H1ORF55\LEFTY2\GUCY1B3\PRDX3\SEC23IP\C2H7ORF64\MARK1\GXYLT1\SLC25A13\PUS7L\RARRES1\RRP15\C1GALT1\RAB33B\PROX1\SRGAP1\STXBP6\SCFD1\TMEM184C\CFL2\KIF6\PSMA6\LRBA\MBIP\SCLT1\KANK1\TBC1D15\NAP1L1\TTLL5\MBNL1\MED12L\OSTC\ELAVL4\CC2D1B\SCIN\ANKMY2\ITPK1\RAPGEF5\INADL\DOCK7\LYST\PGM1\CBX3\COBLL1\C5H14ORF129\CCNK\C3H6ORF120\RFTN1\DCN\KAT2B\GPD1L\BAG5\KIF26A\CLASP1\UBP1\CAMK2D\SLC12A8\EIF3M\ITGA9\STK17A\HNMT\NTRK2\CTSL2\TGFBR1\LOC101750080\DEK\TXNRD1\EEF1E1\BLOC1S5\STRAP\MGST1\PRMT8\LOC420992\LRRC16A\TFB1M\NPEPPS\PPAT\CEP76\BCLAF1\IGF2BP2\OCIAD1\M6PR\EDARADD\CCDC58\MBLAC2\HDDC2\TNFRSF1A\FAM26E\FRK\LYAR\MLLT3\HAUS6\MAMDC2\GPR161\TCEA1\TMEM68\KIAA1524\CHMP2B\ELOVL4\DECR1\PTDSS1\DOPEY2\ATP6V1C1\NOV\RAD21\DDX3X\OC90\DST\APOO\MBOAT2\DSCC1\SH3KBP1\PDHA1\TAF1B\LAPTM4A\PIGA\RAB9A\STMN4\RP2\SLC9A7\GAS6\ATP4B\TNFSF13B\LIG4\IPO5\RBM26\BORA\MZT1\RNASEH2B\GDPD5\TXNL4A\SUV39H2\GZMK\CASP2\PACSIN2\LOC101750282\CERK\E2F5\C2H6ORF62\IFNGR1\SLC25A14\PDDC1\EAF2\PLEKHA3\C2OR

		F47\SPECC1L\PRR11\SLC13A2\C26H6ORF89\C19ORF44\RANBP2\TMEM131\WWP1\APP\MTO1\KDM3A\DNAJA1\HOPX\ATP2B1\FAM169B\SLC10A7\ZBTB34\GATM\PRKAG2\NELL2\GRIA2\AMACR\TBC1D22A\ATP2A2\NAALADL2\NUP188\EIF4A2\LSG1\EPS8L2\LOC422654\
FDR <0.05	gga-miR-1729-5p_R-1	ARMC1\TIMM17A\BMP10\BNIP3L\SLC25A46\IPO9\LAPTM5\CCDC47\CCDC43\TMEM57\KBP\APLP2\SNAP29\WDR45L\NACC2\RFFL\MPP3\ZC3H15\CTGF\EIF4EBP1\ELL\LPCAT2\SALL1\ANAPC7\SMARCE1\TARSL2\ITM2A\BET1L\DPP9\AGTRAP\ULK2\C8H1ORF27\CDH11\ADAMTS18\MSRB1\WSB1\TMEM97\SQRDL\RRAS2\MCM2\TPRA1\ABHD17C\GRM3\RPRD1B\AP2A2\MARCH5\DLG1\ALDH1A3\PEBP1\TMEM104\PFDN4\C17ORF28\SLC9A8\HARBI1\VBP1\CRY2\EEF1B2\LOC424109\SHOC2\WASL\B3GNT2\ENTPD2\LIN9\SLC39A9\EXD2\DYNC1I2\CCNL1\RASSF3\INTS7\COCH\RAP1B\SNX6\PPP2CB\LOC421447\TMED10\BIRC6\GTF2A1\PODN\PPAP2B\MACC1\NUDT9\IGF2BP3\DFNA5\WDFY3\ANKRD13C\MYLK\ESR2\RANGAP1\RTN1\FSHB\ITGA9\ATL1\SOUL\RBM24\TDG\C1H12ORF48\SLC37A3\WRNIP1\ADCK2\RHBG\LOH12CR1\SRSF7\SLMO1\FAM76A\TAPBPL\NAT10\EPHA1\SPICE1\GREB1L\NSG1\ST3GAL6\ARMC2\ALCAM\CHMP2B\MYBL1\SH3BP2\TMEM129\GABPA\UBE2J1\CCNE2\TTC3\ATP6V1C1\DDX3X\CALB1\GRHL1\FAM49A\LAPTM4A\PHKA2\MTMR9\FDFT1\MUT\SEPT10\TBC1D4\SETDB2\N4BP2L2\PAN3\GDPD5\VAPA\SYT4\NCOA7\GCG\MCCC1\TGFBR3\STOM\TCEB3\C26H6ORF89\OVAL\EGFR\ARPP19\GCSH\TRIM59\ATP2A2\GTF3C5\PTPN22\SMAD5\REL\LSG1\TACC1\PLEKHJ1\
FDR <0.05	gga-miR-1559-3p	RFK\BMP10\CHMP7\SDHB\STX12\NMT1\CSNK2A2\HSPA5\SRPR\DBR1\SAMHD1\SRRM1\SNAP29\EDC3\RAB14\STXBP3\GLUD1\COMT\C15H22ORF25\DMXL1\SEC16A\ATR\DNAJC12\RASA2\SPHKAP\PCTP\NFYA\DNAJA4\RAB5C\ZNF207\SFRP1\BARD1\SKP2\FEN1\KRT15\RBPJL\TARSL2\MFAP3\KIAA1279\RNF34\SLTM\P2RX5\ATXN2\ENDOG\RGS9BP\STAU1\TMEM116\VPS53\ATPAF2\CENPI\PLAU\CLTC\SLC25A36\ANXA7\SASS6\PTH\JMJD4\PIK3CB\LPPR5\EIF4G2\SYNGR3\C20H20ORF11\TMEM97\TMEM41B\PAFAH1B1\RUVBL1\GPC4\MKLN1\HTATSF1\NAP1L4\DNAJB6\TM9SF4\GRM3\ZMYND11\PDE6C\P4HA2\TOLLIP\AP2A2\ITIH2\ALDH1A3\ERGIC2\MASP1\STX16\VAPB\SNAPC5\SF3A1\NXT2\ACP2\PACSIN3\IFT122\XIAP\PDCD4\TRABD\PPP3R1\DCUN1D1\DNAJC10\FXR1\PPP1R21\FAM3C\SRI\ING3\TSPAN12\DLX5\ATP6V0C\GUCY1A3\PRDX3\KRIT1\BAG3\ETFDH\EXD2\PDCD10\TAC1\XPOT\AQR\RASSF3\SMAD1\NUBPL\BRMS1L\GPR149\YIPF3\YIPF4\C4H4ORF41\MAP1LC3C\LRRC72\ITPK1\ABCB10\COG2\C3H6ORF120\DAZL\RAB5A\LOC421560\DEPDC1\NGLY1\METAP2\SLC44A5\NEDD1\BAG5\GRSF1\ARL1\ESR2\HIF1A\ACO2\MAD2L1\CAMK2D\DTX3L\WDSUB1\RBM24\TDG\ATP6V1E1\CCDC83\ERP44\ANP32E\MTUS1\CNOT7\OPTN\IMPA2\FGFBP2\RFESD\PRRC1\DEPDC1B\LRRC70\SGTB\ZUFSP\F2RL1\CYTL1\NPC1\ZFAND5\PCMTD1\SEC63\CD200\CYP7A1\CPA6\FABP5\IBTK\MMP16\RIPK2\TMEM30A\DSCR3\EIF3E\DDX3X\PDIA6\RP2\UBE3A\DOCK9\MYCBP2\LMO7\MZT1\RCBTB1\POLD3\ARL6IP5\VMO1\ITM2B\SLC7A13\INVS\PTPN3\TMEM196\HIGD1A\CLN8\HSF2\IFNGR1\PTGR2\INCENP\EAF2\MCCC1\MFI2\NADK\IRAK2\IMPG2\EGFR\ARHGAP15\REEP5\TMPO\NEK1\PGRMC1\GCSH\TBC1D22A\TLN2\SPTAN1\PSME3\EIF4G2\FAS\ACTG1\OAZ1\

FDR <0.05	gga-miR-146a-3p_R-1	PRNP\IPO9\CDC27\CHTF8\CTSS\EYA3\AP1G1\LOC100857226\NPHP4\ELAC2\TIRAP\PIK3R5\SLC25A1\NFS1\SDF4\FHOD1\SLC7A4\TMEM194B\ADPRHL2\LOC770371\LZIC\HDAC3\RPL10A\SARDH\DDX49\VAMP4\ESRP2\LOC101749631\CLINT1\PPT1\EMC1\RIC8A\RCAN3\ABCA8\AR\IVD\RGL1\PPIF\IARS\HDAC8\CCNDBP1\RPS6KB1\SLC25A36\GATA5\TEAD1\WFDC1\LOC768701\TFIP11\TNFAIP1\IRF8\BOK\K123\SKP1\PDE6C\HINFP\PFKP\RNF185\PCSK6\WAC\DPP8\NPM3\AMOT\RNF114\TMEM180\CHRDL1\SLC39A13\MTAP\ARFGAP2\NEURL\ABHD12\POLR2H\ANAPC2\PPP3R1\PPP1R21\NOXA1\RGS7\GORASP2\ARID2\GPR26\RASSF3\PRPH2\BRMS1L\PLK3\VASH1\NUDT9\HNRNPA2B1\CRADD\MTHFD1\SULT1B1\PSMA3\DNAL4\DENND5B\ATP6V1E1\PIK3C2G\ANKS6\CYB5A\TAPBPL\MRPL51\PRRC1\C7\BLZF1\CHRNB3\USP45\HSPA13\FILIP1\MORC3\DDX3X\TG\FBXO9\UBAC2\RNASEH2B\SPG20\GDPD5\RNASEL\HIGD1A\NCOA7\H3F3C\SLC20A2\LOC101750010\POPDC3\PDLIM5\LOC100859142\SSTR2\CYP24A1\XIRP1\PGRMC1\EXOSC2\CYP2H1\PSMD13\PLEKHJ1\
FDR <0.05	gga-miR-1451-3p_R-1	RFK\C6H10ORF88\HMBS\PAK1\PM20D1\SLC26A8\NR0B2\WBSCR16\PIK3R5\SNAP29\LOC428335\RAP1A\CALCOCO2\ARVCF\TMEM194B\ADPRHL2\MSTN\AP3S1\LZIC\REEP3\NRG4\BECN1\SCPEP1\TSR1\KIF20A\SCYL3\UBIAD1\RHOT1\GAS2\SUN1\PABPC4\FAN1\ANGPTL1\MAPK6\PSAP\AP4E1\SLC47A2\H2AFY2\DUT\C8H1ORF27\RPS6KB1\CDH5\NUDT7\C20H20ORF11\ITGB1BP3\DCAF12\RTEL1\GGT1\HSPA14\ARPC4\PLOD2\ATP6V1G1\MTMR8\APIP\CD44\GGA3\SRPK2\DDB2\VBP1\ALS2\ZFYVE19\LCLAT1\C2H7ORF64\SRP14\COL1A2\XRCC6BP1\EDNRA\BRMS1L\RAB3B\ACSL1\HDAC9\EPGN\ROR1\RFTN1\DCN\SCARB2\IGF2R\HSPBAP1\LOC425756\SNX4\DNAJC24\UNC5C\ZRANB3\GTDC1\MKRN1\SERPINB6\BCL2\LOH12CR1\CNOT7\APBB2\ELL2\TCEA1\NFKBIZ\CHRNB3\ATG5\PEX2\SLBP\RIPK2\PRIM2\FBXO9\PRDX4\LPIN1\TM9SF2\RBM26\ARHGDIB\C3H2ORF43\SUCLG1\INCENP\NFE2L2\RABGAP1L\SKA2\ATP6AP2\EGFR\TBP\MYO5A\ADAL\PELI1\TMEM130\NGEF\
FDR <0.05	gga-miR-130b-5p_R-1	RFK\ARHGAP25\RASSF2\ADAM28\LSM7\DCAF7\LOC426699\C11H16ORF80\LDLRAP1\SNAP29\MYBBP1A\PI4KA\B3GNTL1\MIB2\PPIL2\UBE2R2\EIF6\CHMP4B\TSPAN14\C10ORF58\CHCHD2\RAN\POLR2E\C19H17ORF85\TFAM\MED23\LRRC45\BNIP1\BRI3BP\FBXL8\GNA11\HDAC1\TM2D2\KPNA6\LOC425137\SLC6A2\BPTF\USE1\CHD9\PPT1\IQCA1\CAPZB\TAF9\SIRT3\ADAM10\FAM20B\TBCA\TNR\C10ORF54\NOL8\H2AFY2\RHBDD1\ALKBH2\CES1\AP1S3\FAM120A\CYB5R2\USP32\JMJD4\PIK3CB\ARHGEF3\TNFAIP1\TPRA1\ZNF326\FAM122A\MLL3\K123\ANKRD1\PDE6C\PISD\HRAS\BDH1\RBM7\METTL9\CREM\CLCN5\NIT1\MTMR8\KDM3B\KIAA1841\APIP\VRK2\TTC17\TMEM209\CHRDL1\SLC39A13\CTDSPL2\CFLAR\SLC24A6\KIF16B\PPP3R1\COMMD5\TTC14\NHLRC2\RABL6\LCLAT1\TRMT6\WDR26\SMOC1\ETFDH\COL1A2\FMN1\C1GALT1\USP15\CENPF\C5H15ORF41\FASTKD1\LPGAT1\RAP1B\TMEM184C\MDGA1\SEC23A\PRDX1\ADCK1\AGA\SCIN\DHCR24\ITPK1\EREG\SIPA1L2\CBX3\RFTN1\UBE2E1\SLC44A5\HSPBAP1\SERHL2\HIF1A\LARS2\EXOSC7\SFRP4\MDGA2\ITGA9\VPS41\C3H6ORF103\PWP1\RBFA\KIF13A\HIPK2\GALNT1\CLPTM1L\LOC426037\SPTLC1\MBP\NPEPPS\PPAT\TMX3\C22H2ORF42\ARNTL2\PDS5A\CSTB\WBSCR22\MBLAC2\GFM2\RABL3\OTOP1\NSG1\B4GALT4\FIG4\HAUS6\IMPACT\TIPRL\PCMTD1\TCEA1\

		KCMF1\FNTA\BRP44\ZFP92\GPR89B\GBE1\GRIN3A\CPA6\ACOX3\WHSC2\RIPK2\CCNE2\PTDSS1\SIK1\DMD\FBXO9\MTSS1\FAM49A\PHKA2\ASXL2\OTOF\CD2AP\HERC2\TNFSF13B\MZT1\GPALPP1\LCP1\POSTN\N6AMT2\FZD4\ARNT\SUV39H2\FAM125A\CBX1\LYN\WASH1\GATA2\C2H6ORF62\PTGR2\SFI1\MPZL3\GLT1D1\NEO1\STOM\NFYC\MXRA8\NADK\CRMP1\RSPO3\RNASET2\DLL1\TGFB3\IGFBP5\WIPF1\UBE3C\FASN\CD151\CACNA1B\REL\MAP3K14\
FDR <0.05	gga-miR-128-1-5p	ITGB3\LIN28A\ANKRD24\MOV10\EXOC3L1\NOC2L\GMPPB\GJA4\VAV2\NKX2-5\ETFA\KPNA6\SDF2\MPRIP\RASD1\PEMT\HAGHL\TADA2A\PITPNB\RAP1GAP2\MYLK2\IL13\GATSL3\UBE2H\LEFTY2\LOC424620\CHGA\CAMK2D\IGF2BP2\KIAA0907\MSX1\ZDHHC23\HMX1\B3GAT2\GNOT1\CALB1\HERC2\UBAC2\NUPL1\PSMD3\ARHGAP19\C20ORF96\RALBP1\WNT6\EPHB3\GFOD2\LIMK1\MAP3K14\STK40\PFKL\
FDR <0.05	gga-miR-125b-5p	ARMC1\BF1\STARD4\CD79B\RPS6KA1\LIN28A\ENTPD4\TIRAP\WBSCR16\CIAPIN1\ABI3\ANP32B\ACSBG2\PRPSAP1\APTX\HEMK1\MPP3\RNF123\CREB3\INPP5K\ENPP1\TRPC4AP\SURF1\CRLF3\HDAC1\EIF3I\SBNO1\LACTB\ACTR5\AKTIP\USE1\LIFR\TMEM38A\TRIT1\MINA\TOMM34\ADA\TTPAL\SGPL1\YIPF6\CTNS\PHKA1\NONO\GBF1\DENND6A\SNX12\CDX1\ABHD6\DCAF12\PAFAH1B1\PLS3\MMP11\GORASP1\MCM2\GALNS\AQP12\SMG1\RNF213\RBM7\HRASLS\CLCN5\YME1L1\ZFYVE27\RAB11A\CDX4\C6H10ORF76\FBXW4\BCO2\KCTD2\PTPN1\UBE2V1\FBXO18\NR1H3\PCGF6\SUMF1\CIB1\ITPKA\NDUFA5\NRK\LBH\ENTPD2\SNAP23\ZFYVE1\SLC39A9\ASNS\PROX1\LOC422448\PREPL\LOC417848\MMACHC\ANGEL1\NKX6-2\SNW1\ADCK1\LOC100859230\SMEK1\SCIN\DFNA5\TARBP1\KERA\TMEM66\ANKS1B\MGP\SERHL2\NAGA\TRANK1\ENPEP\CASP6\ITGA9\SOUL\CCDC148\CDC14B\BLOC1S5\IRF4\PLEKHO1\STX17\MTUS1\LOC426218\PPAT\ENPP7\GPR155\EDARADD\MRPL10\MRPL51\MRPS36\GREB1L\F2RL1\TRAF3IP2\MAMDC2\MDN1\NT5E\RIPK2\TSTA3\SEPT6\CLN8\C26H6ORF89\DGKZ\XIRP1\ZNF335\MYO19\GTF3C5\FLOT2\FAS\TNFRSF10B\HSBP1L1\
FDR <0.05	gga-miR-107-3p_R-1	ARMC1\ARHGAP25\PRNP\MGAT4C\TMEM9\STARD4\CD79B\KCTD9\DHDDS\SLC16A4\HINT1\RAB11B\TBC1D22B\C28H19ORF28\RASSF5\AP1G1\NR0B2\GJC1\PPP6C\WBSCR16\MYBBP1A\MECR\PTPRU\ZAP70\VWA1\PPIL2\MYO1F\ANP32B\CDC45\PSMD5\SCAMP2\VAV3\GNB1L\SLC25A24\PMPCA\LOC419429\ACOX1\PLEKHB2\FAM168B\MRPL38\ADPRHL2\AP3S1\GMPPB\MCCC2\RGS2\PIK3CD\DEF6\SBNO2\SLC15A4\TFAM\TAF11\INPP5K\RFWD3\NTAN1\PEX14\TRAIP\TARDBP\SUGP1\SERPINF1\AGFG1\TADA1L\FBXL8\TSR1\PEF1\ITPA\CRLF3\MTMR9LP\KIAA1191\RHOT1\BSDC1\LOC425137\SFRP1\BARD1\DDX31\MINPP1\WDR70\C21H1ORF144\SALL1\OSMR\UBE2F\TIMD4\SYS1\MFAP3\STK4\FAM81A\TOMM34\PNRC2\VPS26A\SIRT3\SIGIRR\XPO1\ASB6\CCNE1\WIPI2\HVCN1\MTHFR\TAX1BP3\IVD\SET\C14H17ORF103\DHRS7B\SCG3\IARS\VPS53\MTFR1\PEPD\DUT\DLG5\TOP3A\C2H3ORF75\USP54\BICD2\VIPR1\SLC25A36\IBA57\PTH\BRCC3\FREM1\C17ORF78\SYNGR3\COL9A3\C20H20ORF11\DCAF12\TMED5\MMP11\RRAS2\NDUFAB1\MAPK8\RTEL1\LSS\K123\TMEM43\PANK1\SRPRB\C13H5ORF15\KIAA1024\TSSC4\ABCC1\TOLLIP\YWHAH\CSTF2\TSPAN6\POU2AF1\NCBP2\PFKP\RNF185\LOC1008

		58827\TK1\CLPX\ANKRD40\PEBP1\CUTC\TUBB1\KMO\CLCN7\MTMR8\CHIC1\TRAP1\USP12P1\CASS4\TMEM104\EIF4E3\TTC12\BCL11A\DIXDC1\KCTD2\HN1\GGA3\MRPS7\BCAP29\SPATA2\TMEM180\LOC417013\CHRDL1\NDUFS3\KIF23\NT5C2\C15H12ORF49\F2\RAD18\RPL32\SMARCA1\AP2M1\ABHD12\TUBGCP4\GTF2H4\TYRO3\NKRF\LOC431656\C6H10ORF46\DCAF4\LOC423256\CAV2\SERPINI1\TMEM229B\RAB3GAP2\SRP14\MYO3B\ZNF650\GFM1\LXN\SSB\CHST15\LOC422442\MCFD2\GEMIN2\HSPA4L\MMACHC\TMED10\GPR149\MBNL1\ANGEL1\LOC395824\ADCK1\GTF2A1\CC2D1B\NEIL3\GALNTL6\SCIN\MACC1\IGF2BP3\LYST\COBLL1\URB2\PKDCC\WLS\PPP2R5C\WDR20\LTA4H\SDAD1\TCP1\PTPLB\MYLK\GPRC5A\HEBP1\ESR2\SYNE2\ATF7IP\XPNPEP3\QSER1\HERPUD2\LOC420748\PLA2G6\ATP5S\RAB32\UBE2D3\C3H6ORF103\NHEDC2\PLEKHC1\SOUL\FBXO7\WDSUB1\C22ORF28\CTDP1\RBM24\TDG\WRNIP1\MCM4\BPGM\SLC15A5\RHBG\EXOC3\SORBS2\FAM149A\MTUS1\GMNN\TDP2\DNAJC16\AGMAT\EZR\CEP192\TACR1\FAM38B\SGCB\DCUN1D4\FGFR1OP2\TEC\PRR5\SCNN1A\PDS5A\FOXJ2\CD38\PHYH\PNPLA2\HORMAD1\CEP85L\MOCS2\FAM26E\F2RL1\NSG1\TJP2\FAM189A2\PCMTD1\ST18\TBC1D23\TXNL4B\LOC396120\TMEM175\FNTA\HACE1\UBXN2B\CYP7A1\USP45\GJA5\GRPEL1\TCEB1\TPD52\PTCD3\RIPK2\OTUD6B\PLEKHF2\SLC25A32\PRADC1\COL22A1\U2AF1\ST3GAL1\TINAG\FBXO9\FBXO32\DERL1\KLF11\UBXN2A\MOSPD2\MTMR9\DEFB1\HERC2\TNFSF13B\STK24\IRG1\TBC1D4\WBP4\UBL3\SLC7A1\SLC46A3\CDK8\MTMR2\TYR\FZD4\LCMT2\SUV39H2\CLDN10\SEPT6\LOC418472\POT1\CERK\MICAL1\CLN8\PLAC8\SLC20A2\LOC422426\NKAP\KLHDC2\DAB1\MCCC1\ARHGAP19\TGFBR3\RSFR\RRN3\NOS2\LOC419404\NADK\RB1\WWP1\TRIB2\DLL1\TEF\PSMC3\LOC416916\SLC10A7\ZRANB1\WIPF1\GABPB1\MYO5A\ATP1B4\LSP1\TGM2\LOC769242\PELI1\PDE3B\HEATR3\PSME3\PNPLA7\ZIC1\MAP3K3\BUB1B\MYBPH\P4HA1\EFTUD2\ANG\PLEKHJ1\CD320\
FDR <0.05	gga-miR-106-3p_R+1	ARMC1\B-G\TRIM39\BNIP3L\STARD4\ADAM28\HMGN2\CCDC15\SPPL2B\DCAF7\CAMTA1\KIAA0556\STX12\PPP1R8\CTSS\RASSF5\NUDC\WDTC1\NPHP4\NEK6\SAMHD1\GTF2I\SRRM1\LOC100857709\SNAP29\FAM18B1\CASP9\SERPIND1\RAB14\RAP1A\RRAGC\NFS1\MYO1F\MAN2C1\SLC16A1\TNRC6C\ACSBG2\WWC1\PHTF1\GNB1L\LOC419429\ARVCF\FAM86A\NCOA4\KCNMB1\PLEKHB2\RFFL\RERE\AGO3\ETF1\TMEM194B\SPSB3\SORD\OSGEPL1\NOTCH1\GBAS\EXOSC3\CCT6A\MMP17\CDC73\PIK3CD\LZIC\HDAC3\C19H17ORF85\ZC3H15\INPP5K\DNAJC12\MAP2\RFNG\CTNNA3\TMEM170A\TXNL1\DDX49\SURF1\MLX\GABARAPL2\NFYA\SUB1\EIF2B1\COMP\CRLF3\MBTPS1\DDX55\KIAA1191\RHOT1\BYSL\SFRP1\DDX5\PPP1R16B\AKTIP\FEN1\CHD9\UCK1\ANAPC7\KRT20\MINA\COPS8\CNOT8\PPM1M\GALE\GPR107\MICALL2\ITM2A\TOMM34\PNRC2\TOR1A\RNF111\BET1L\BLMH\VPS13D\CYP3A7\EYA2\SH3GLB2\PLOD1\YIPF6\AR\P2RX5\SHISA5\IVD\SET\C14H17ORF103\TSEN15\PPIF\PEMT\PRPS1\OPNP\VGLL4\FAM102A\PLAU\FUT11\MED13\PPP3CB\CDH5\SLC25A36\SASS6\IBA57\DHRS11\PNAT10\MPHOSPH6\NONO\DENND6A\GARS\C20H20ORF11\TMEM97\TNFAIP1\SPPL2A\GCLM\COL7A1\BCAR3\LOC420419\ZFAND4\ARHGAP17\KLHL13\DCTN5\KLHL3\GTF2H1\TBX6\CEP19\HTATSF1\PPP2CA\KIAA1024\MYH11\PDE6C\CEP63\KIF11\AP2A2\POU2AF1\CTSA\RNH1\SPPL3\TLR4\ACAP2\CABP1\ALDH1A3\ITGB1\EPC1\ATP13A4\PGS1\ANKDD1A\SYNPR\CLPX\RAB

		35\ANKRD40\SUDS3\TMLHE\CNNM1\YME1L1\C7ORF26\HMOX2\TIPIN\STAT1\KDM3B\ECI1\TMEM104\TMEFF2\FOXP1\FANCL\VRK2\MLLT10\SUMO2\GGA3\GRB2\UQCR10\CHRDL1\CELF1\FBXW8\GRAMD3\CASP8\STRADB\ALS2\TRH\SLC38A2\MCTS1\COBRA1\DYTN\CLNS1A\EEF1B2\CXORF56\TRDMT1\TRABD\PPP3R1\WASL\DCUN1D1\IDH1\SAMD13\UGP2\B3GNT2\SESTD1\PIF1\TASP1\FSHR\CRDS2\ACBD3\ATF2\NVL\SP3\ETFDH\PPID\SERPINI1\PDCD10\TMEM229B\SLC25A13\MSMO1\FMN1\RPAP3\NKTR\RASSF3\LPGAT1\IL22\SCFD1\SLC35E3\NUBPL\TMEM184C\CNOT2\GLO1\SEC23A\GEMIN2\FAM161B\NAP1L1\E2F7\TMED10\HERC3\TMEM63C\LOC425049\CMPK1\LEF1\YIPF4\SLC25A4\MAGOH2\ASB5\RAPGEF5\FAM126A\HSD17B11\STK31\DFNA5\SNX10\GLRX5\HIBADH\PKDCC\RFTN1\LOC421560\CRADD\TECRL\ZRANB2\TECPR2\T\AMPH\SCYL2\GC\RALB\TSN\C5H14ORF79\PTPLB\MYLK\LZTFL1\POLDIP3\HIF1A\CYP2D6\RPL7L1\ST13\CFI\DLGAP5\CASP6\METAP1\ANKRD54\MAP4K5\LOC420770\MRPL32\SAV1\NUP43\FBXO7\MARCH7\CFC1B\PWP1\TMEM263\SLC41A2\RBM24\TXNRD1\TDG\TXNDC5\DENND2A\ADCK2\CREB3L2\DNM1L\CCDC127\C3AR1\PRMT8\PPIE\UBA3\ANP32E\SYNE1\FAM69C\SERAC1\SLMO1\KDR\TBCE\HBS1L\GRK6\APBB2\KLB\ITFG2\EDARADD\NAT10\LYSMD3\CAST\FBN2\EPHA1\SERINC1\ABHD3\WDR1\FAM26E\ZBTB49\HAUS6\ABHD17B\B4GALT6\DAZAP1\CAMK4L\F5\GNE\NFKBIZ\TMEM175\IDUA\PREP\SDR16C5\MAEL\GJA5\PDZK1\SH3TC1\ZCCHC9\TCEB1\HSPA13\ADD1\MXD4\WHSC2\TPD52\ORC3\SYNCRIP\KIAA1009\OTUD6B\TMEM67\IFNAR1\CDH17\IFNGR2\PTDSS1\RAD21\PDE9A\PHF20L1\OC90\PRDX4\DERL1\IAH1\ENPP2\TAF1B\KLF11\PDIA6\MSGN1\LAPTM4A\ASXL2\SYAP1\PIGA\INTS9\RP2\EIF5B\CHST10\PDCL3\C1H2ORF49\UBAC2\DNAJC3\DZIP1\KLF5\KIAA0564\SUCLA2\CKAP2\SLC25A15\B3GALTL\SLC7A1\CRYL1\CWF19L2\MMP13\MTMR2\NAALAD2\FZD4\ANKRD42\PPME1\GDPD5\SPDYA\KIAA1609\DDI2\FAM125A\LYN\SGK223\CD47\POT1\TMEM196\ETV1\LOC421975\KLHDC2\PTGR2\CHP1\INCENP\EAF2\ATE1\ARHGAP19\RPE\RNF7\ALDH1A2\WDR61\GLT1D1\KPNA1\IFI27L2\DSTYK\CCDC39\TTF2\C4H4ORF52\NT5C3A\RNASET2\ACSF2\GLDC\SSTR2\GARNL3\WIPF1\NELL2\PGRMC1\DDX52\RPL39\TRIM59\LDHA\PELI1\KIAA1328\NBL1\IREB2\CD36\PSME3\USP7\HSPB7\LIN7B\REL\GRIN1\RER1\SLC9A9\OCRL\PDZD11\
FDR<0.05	gga-let-7j-3p_L-1R+2	GALK2\PM20D1\RASSF5\AP1G1\IL10\C20H20ORF24\TMEM57\GTF2I\SNAP29\STMN1\RBM39\ACSBG2\MRPS34\TEK\TRIM33\STXBP3\C15H22ORF25\CHMP4B\FBXW11\LOC427001\AP3S1\GBAS\MATR3\MAT1A\INPP5K\BRCA1\RCN2\CTNNA3\NEDD4L\ASH2L\SUB1\COMP\DDX55\MTOR\DNAJC7\ZNF207\SLC12A4\SLC6A2\BPTF\TOP1\PSMD12\SUN1\FAN1\ITM2A\DDX50\RNF111\SLTM\TTPAL\TSPAN15\VPS13D\COL18A1\FOXK1\HVCN1\NCOA3\NPPC\ZER1\MAPK6\SCG3\COPS3\RPS24\LSM14A\CENPI\PRPSAP2\PTGS2\CUL3\WDFY1\GHRHR\SASS6\DYNLL2\TBL3\EVI2A\RNF141\DENND6A\C20H20ORF11\TMED5\ARHGAP17\RRAS2\MST4\PRKAG2\NSMCE1\H2AFY\IL16\PDPK1\TCF7\KIAA1024\FOPNL\PDE6C\BRSK2\KIF11\KIF3A\MARCH5\CHKA\PFKP\ACADS\KDELR2\KIF21A\CADPS\SPAG9\RAB18\STX16\EGR1\MAP2K1\TRAPPC4\KIAA2022\CBLN4\BCL11A\EFEMP1\MRPS7\NXT2\MOB4\GDI2\RELN\CTDSPL2\PHTF2\NET1\TTL\NKRF\TUBGCP6\TRABD\SAMD13\FBXO11\NRK\ING3\TSPAN12\TASP1\DLX5\HOXD12\CTSO\SMOC1\IFRD1\PDCD10\CCDC132\ATP6V1D\ZNF650\C

		5H15ORF29\C1GALT1\ELMOD2\RASSF3\IFNG\SMARCA5\RAP1B\STRN3\HSPA4L\FCF1\MBNL1\OSTC\GTF2A1\LRRC42\TMEM59\HNRNPA2B1\CBX3\SLC4A10\EML1\SMOC2\WARS\RFTN1\DCN\NGLY1\NEDD1\GRSF1\MAP3K4\PTPLB\SLC38A6\KIAA1143\MAD2L1\METTL14\ARPP21\FBXO34\GMFB\LRRFIP2\SHPRH\ATL1\ISCA1\RBM24\RPP40\ECI2\IRF4\SLC37A3\VPS4B\CLPTM1L\GABBR2\FUCA2\C6ORF115\CEP76\ARNTL2\TEC\ATP10D\SCNN1A\KLB\EDARADD\GPR98\FBN2\PRRC1\SGTB\ERBB2IP\FAM184A\WDR1\ZBTB49\MSX1\SS18\ABHD17B\TMEM68\CD200\UBXN2B\FBXL4\PREX2\UFL1\LRPAP1\ACOT12\TCEB1\SAMSN1\USP25\TPD52\JAM2\USP16\HMGN3\IMPG1\OTUD6B\FAM135A\IFNGR2\FZD6\GNOT1\NOV\WDR4\U2AF1\PHF20L1\TINAG\REPS2\TXLNG\EGFL6\GTPBP6\CYFIP1\REV1\KLF5\BORA\C1H13ORF15\SETDB2\N4BP2L2\FRY\CRYL1\MMP13\MTMR2\LOC426128\YWHAZ\UBE2N\EBAG9\TERF1\PPP4R2\RNF7\STOM\KPNA1\NADK\SLMAP\SUGT1\LOC423536\GOLGA4\SNX13\TP53BP1\TMPO\PSMC3\WIPF1\PRKAG2\CNTN1\GRIA2\XIRP1\MYO5A\TBC1D22A\GHRHR\EIF5A2\PELI1\C5H11ORF46\
FDR <0.05	gga-let-7b_R+2	RDM1\KCTD9\GALK2\CYB5B\HBEGF\LOC428335\ST14\CNTRL\NFS1\HEXDC\LRIG2\VAV3\TMEM194B\GMPPB\PCTP\UBIAD1\GOLGA7\OSMR\TARSL2\TNFRSF1B\P2RX5\PDCD5\MAPK6\PTGS2\DEAF1\SLC25A36\USP32\PNAT10\ABCD3\TMED5\KLHL13\KIAA0753\ZNF326\SRPRB\CBL\IL13\MAP3K8\ITGB2\KIAA2022\MAGT1\EIF4E2\TMEM164\C6H10ORF32\PHTF2\TYRO3\SNAP23\ETFDH\YAF2\C5H15ORF29\COL1A2\ACADSB\C5H15ORF41\WDR65\EAPP\BTBD9\ABCD4\SMARCAD1\ICA1\BZW2\IGF2BP3\GLRX5\BCS1L\EXOC3L4\TNFAIP2\IL8L1\ATF7IP\SERHL2\C22ORF32\SLC12A8\CEP70\ADH1C\TGFBR1\RBFA\LOC420849\GNPTAB\AKR1D1\SERPINB10\WNK1\PBX4\SLMO2\C2H18ORF19\MAP3K5\CHD4\POC5\GTF3C6\UHRF2\LNP1\RARS2\SYNCRIP\CCNE2\U2AF1\ST3GAL1\DMD\PIGA\GAL10\MGAT4A\COL4A2\TGDS\WDFY2\DDI2\HSD3B2\LOC420301\E2F5\TERF1\HCRTR2\CZH9ORF80\HABP2\DNTT\BZW1\GLT1D1\GATM\GCSH\MYO19\TMEM120B\DDX19B\EIF4G2\C5H11ORF46\YOD1\PDZD11\ARHGEF10L\STK40\TMEM130\
FDR <0.05	cin-miR-33_R+4	ARHGAP25\ADORA1\STARD4\CAMK4\PCNX\HMGN2\HNRNPM\ITGB3\HBXIP\DDX42\CDC27\ZCCHC17\FBXW2\NR0B2\SRPR\TMEM57\SAMHD1\RSPRY1\TTC1\STOML1\GLB1L2\ATP6V1B2\ACSBG2\SFMBT1\GFRA2\IWS1\ST6GALNAC2\PLEKHB2\NR5A2\RFFL\NUBP2\HSPA9\ENO1\PSPH\ZC3H15\DUSP19\ACAA2\RFNG\ARID5B\SCPEP1\KIF20A\DNAJA4\LMX1A\SFRP1\ALDH9A1\SARM1\SALL1\KRT20\RNF111\KNTC1\MAPK6\SET\NOL8\SLC12A1\AP3M1\LRRC39\LOC416530\DYNLRB2\BCMO1\TEAD1\ARHGEF3\LOC768701\RNF141\STK25\XBP1\DCAF12\BCAR3\DR1\TMED5\MTF2\MYD88\LOC100858792\RUFY1\ABTB1\ZNF326\GALNS\NAP1L4\C12H3ORF19\DNAJB6\ANKRD1\OAF\YWHAH\POU2AF1\RNF185\METTL9\ACOX2\SYNPR\SVIL\LPP\FYTTD1\MTMR8\HMOX2\LOC416354\KCTD2\SRPK2\ORC5\ALDH7A1\CNTN4\FGL2\SDSL\CRY2\SLC39A12\TYRO3\CXORF56\WASL\CROT\ATP6AP1\SRI\LCLAT1\DEGS1\CAPZA2\SLC39A9\LOC424998\CCDC132\RAB3GAP2\SLC25A12\GPATCH2\LXN\XRCC6BP1\TRAF5\IFNG\G2E3\PRDX1\TSPAN1\MED12L\AHCTF1\PPPDE1\C5H14ORF159\MEOX2\RAPGEF5\VRK1\EML1\EPYC\TECRL\TMEM66\ELK3\UHRF1BP1L\CCK\NAA30\SEPT7\FBXO34\ITGA9\METAP1\UBE2D3\HECW1\STK17

		A\NOL12\WDSUB1\GKAP1\ALDH1L2\TXNRD1\HIPK2\AGK\DAP\BPGM\PIK3C2G\EXOC3\TBC1D1\CYP4V2\FRG1\PPAT\KDR\FGFR1OP2\NUP50\LIMCH1\SLC34A2\EMG1\ARSK\PTPRK\MTX3\GREB1L\ESCO1\RABL3\NPC1\HAUS6\PCMTD1\CLTA\MAEL\CHMP2B\MYBL1\GRK4\TCEB1\HSPA13\SH3BP2\RNF4\SAMSN1\WHSC2\MMP16\IMPG1\TMEM30A\EEF1A1\MORC3\UTP23\SIK1\DDX3X\NR0B1\PXDN\DERL1\NBAS\LAPTM4A\HADHB\PIGA\RAB9A\C1H2ORF49\ATP4B\COL4A1\TNFSF13B\STK24\LMO7\RCBTB1\SLC25A15\POSTN\MAB21L1\N4BP2L2\N6AMT2\VAPA\KLHL5\KIAA1609\LYN\VMO1\C1H21ORF33\PACSIN2\CERK\GATA2\EBAG9\PTPN3\LOC422426\IL13RA2\PTGR2\KCNAB1\TGFBR3\CCDC39\EEF1A2\TAPT1\EGFR\PDLIM5\ARHGAP15\REEP5\LUC7L3\FAM169B\CUL2\SLC10A7\LLPH\WIPF1\CYP24A1\ARPP19\LSP1\TRIM59\TMEM120B\NAALADL2\AP4B1\LIN7B\PDZD11\SLU7\YEATS2\ATCAY\
FDR <0.05	aca-miR-363-5p	BNIP3L\STARD4\SPPL2B\DCAF7\ZBTB48\PPP1R8\TBC1D22B\CCL4\FAM168B\ETF1\TMEM194B\H3F3B\RGP1\CDC73\CREB3\ENPP1\ERLIN1\GBGT1\LRRFIP1\TRMU\EDA\LOC423008\CEBPG\LSM14A\WSB1\NLK\DR1\ARFRP1\CTU2\PDPK1\P4HA2\UPF2\MARCH5\USP28\RMI2\PTPRG\MTMR8\STAT1\NMRAL1\STK17B\MRPS7\NXT2\ARFGAP2\NAPEPLD\NET1\TTL\AK5\PRKCI\LOC423256\BAG3\ELMOD2\ELOVL1\BRMS1L\GEMIN2\XPO5\LOC100859230\EREG\NTPCR\C5H14ORF129\LRRIQ1\SMOC2\DCN\NGLY1\LUC7L2\HIF1A\RPL7L1\LATS1\ALDH1L2\LOC418170\FRG1\FGFR1OP2\LOC421712\C7\USP14\CDH2\TMEM68\PREP\MAP3K7\PEX2\IMMT\GEM\MAOA\FBXO32\PIGA\MGAT4A\C1H2ORF49\LIG4\RNASEH2B\POLD3\FOLR1\KIAA1609\VDAC3\CECR1\RPRD1A\GATA4\C3H2ORF43\SUCLG1\PLEKHA3\ADAP1\STOM\FLNB\PDLIM5\CRB2\AMACR\
FDR <0.05	aca-miR-363-3p_R+1	ARMC1\STARD4\NEFM\SPPL2B\CDC27\CNTN2\WWP2\SLC41A1\LOC100857226\TPRG1L\SRPR\C20H20ORF24\TMEM57\KBP\C4BPA\PPP2R3A\PIK3R5\SERPIND1\GSN\RAB14\CCNL2\ATP6V1B2\ACSBG2\C15H22ORF25\ACOX1\PLEKHB2\HSD17B4\HIBCH\C10ORF58\KCTD7\BMS1\TRIP12\MMD\EIF2B1\KIAA1191\BSDC1\BARD1\MYPN\AP1M1\KDM4B\ITM2A\RIC8A\ADAM10\FAM20B\PLG\SHISA5\BUD31\RGL1\PPIF\MPP1\C8H1ORF27\CLTC\INTS2\BCAS3\PPIC\GATA5\PIK3CB\SYNRG\PNAT10\ST6GAL1\DKK3\RNF141\XBP1\PAFAH1B1\ABTB1\HPRT1\OSBPL5\ATP12A\KIAA1024\TOLLIP\CHKA\BRWD3\ERGIC2\UTS2D\SYNPR\ZDHHC5\PRKAB1\CLCN7\MTMR8\RAB11A\CHIC1\ZWILCH\CSTF1\CBLN4\BCL11A\GTF3C3\SF3B1\COQ10B\RTN4\WSB2\C15H12ORF49\NEURL\FGL2\PHTF2\GRIA3\DUSP5\ADAM23\GTF2H4\CSRP2BP\SHOC2\WASL\VPS54\FXR1\PIGK\TMEM87A\TASP1\NVL\CRAMP1L\GUCY1B3\PRKCI\KRIT1\CAV2\MIA3\DUSP10\TMEM229B\SLC25A12\MSMO1\METTL8\COL1A2\GPR26\LPGAT1\GNS\ATP6V0B\GPR149\LOC424620\AGXT2L1\SGMS2\HEATR5B\ACSL1\PODN\GOLGA5\PPAP2B\NUDT9\ROR1\LEPROT\HACL1\AGPAT9\PPP1R13B\HIPK3\KIAA1109\ACO2\CCR8\TOB2\PCDP1\HERPUD2\ZRANB3\LRRFIP2\TRIP13\BAZ2B\ISCA1\SLC37A3\CCDC127\PYROXD1\C3AR1\TMF1\UBA3\FAM149A\MTHFD2\CTRC\HBS1L\NUP50\CSTB\FBXO4\RABL3\FAM26E\NSG1\C9ORF93\GNE\FNTA\PDZK1\TCEB1\FABP5\SLC17A5\OTUD6B\THNSL2\FAM135A\RAD21\PSCA\SIK1\RAB23\ASXL2\PIGA\MTMR9\RP2\TNFSF13B\LIG4\DNAJC3\MZT1\UFM1\CRYL1\NOX4\LOC426128\ITM2B\PDGFRL\AK6\C7H2ORF76\LOC41

		9830\OVAL\PSMC6\GOLGA4\HOXA11\CDCA7L\DGKZ\CNTN1\PGRMC1\ATP2A2\CYP2H1\SUPV3L1\
FDR <0.05	aca-miR-191-5p_R-1	IRF1\NCAPD3\KCTD7\CRLF3\GLUL\SALL1\LOC101749801\SRSF10\PAFAH1B1\KLHL13\BCL2L1\SRPRB\SORL1\NRP1\CD3E\UBE2V1\MADD\TPCN1\LCLAT1\CRIPT\SNX10\ATG2B\BAG5\KIAA1109\LRRC16A\STIM2\DAZAP1\SLC17A5\MORC3\CKAP2\RNF220\MFI2\YOD1\
FDR <0.05	aca-miR-18a-3p_L+1R-1_1ss12GA	C6H10ORF88\GFPT1\AFMID\SNRPE\BRD2\WDR36\RPS6KA1\TAT\CIAPIN1\SRRM1\RRAGC\SEPT9\PTBP1\AMPD1\DTWD2\SUMF2\SPSB1\KCTD7\ATR\DNAJC9\LRRC45\TSR1\EIF2B1\CRLF3\DNAJC21\LOC100859426\CLINT1\KRT15\COPS8\LOC101749801\RAB33A\TRMU\C28H19ORF10\CCDC69\CYP3A7\SLC2A10\PLOD1\SHISA5\FAM101B\RHBDD1\URM1\TAF7\NAE1\ADAMTS18\NARFL\SLC44A3\GCLM\GORASP1\MCM2\COL6A2\MORN4\ZXDC\PDPK1\KIF3B\DEPDC5\BDH1\PCNT\LRRC28\ITGB1\ATP13A4\CLCN5\LOC422305\TRAP1\MAP2K1\USP12P1\CSTF1\SLC39A13\STAG2\PEX6\TYRO3\DSTN\B3GNT2\SKIL\PRKCI\KRIT1\SLC39A9\DUSP10\SRP14\ACADSB\RSRC1\XRCC6BP1\HCLS1\MDM1\ELOVL1\EDNRA\DPH2\ZNF410\TMED10\ATPAF1\DHCR24\INADL\STK31\SLC4A3\KERA\SCYL2\LOC421584\SEC24B\NR1H4\PTPLB\DDX47\XPNPEP3\SAMD4A\STK17A\TOM1\BPGM\STRAP\UBA3\MBP\FUCA2\CYB5R3\RFC1\COPS7A\WBSCR22\C12ORF57\MBLAC2\LYSMD3\SPAG17\CZH5ORF34\ABHD3\HJURP\GJA5\RNF20\SOHO-1\TP53INP1\MORC3\WRB\ADCY8\FBXO9\FDFT1\CHST10\TGDS\SLC25A15\UFM1\FAM168A\FAM125A\TPD52L2\TRIM3\WASH1\GTPBP1\POT1\ETV1\WDR48\INCENP\LOC431660\CRMP1\TGFB3\RAD54L\SLC1A2\DDX19B\AP4B1\GNB1\PCSK7\
P<0.05	xtr-miR-429_R+2	NFU1\ARMC1\BNIP3L\NEFM\CHMP7\GALK2\TERF2\STX12\FAM72A\C11ORF30\ZMPSTE24\NMT1\FBXW2\NR0B2\TAF12\GJC1\FAM118B\GATSL2\APLP2\SNAP29\GIGYF2\NFS1\INPP5B\LOC416777\UBAC1\SLC16A1\E2F4\TEK\LRWD1\PTBP1\TRIM33\GPRIN2\NR5A2\SEC11A\PAIP2\RGS2\ZNF76\NUP35\DNAJC12\RASA2\RSL1D1\TARDBP\NRG4\AGFG1\MMD\UBIAD1\RHOT1\SLC12A4\FN1\HEATR2\MPHOSPH10\PPT1\COPS8\AP1M1\ITM2A\PNRC2\PABPC1L\SRSF10\YWHAB\DDX50\ADA\RNF111\OIT3\RSRC2\HVCN1\RGS9BP\SET\RGL1\MPRIP\FAM129A\RNF2\PHACTR3\COL4A4\PPARG\GIT2\RPS6KB1\VCAM1\INTS2\SASS6\JMJD4\PNAT10\MPHOSPH6\ARHGEF3\COL9A3\SNX12\CHEK2\C20H20ORF11\SPPL2A\SEPT2\TMEM41B\MTF2\LOC416931\KLHL13\PDE8A\MST4\DDX26B\MKLN1\HTATSF1\UBE2I\ANKRD1\GRM3\PDE6C\AP2A2\IL13\TSPAN6\NCBP2\KIF3A\LOC395991\PPP1R2\ANKDD1A\ZEB1\SPG21\GCN1L1\TOB1\CTSZ\MTMR8\CHIC1\TMEFF2\PUS10\FANCL\EIF4E2\SF3A1\GTF3C3\MRPS7\UBE2V1\SNAI1\CELF1\PLCL1\CTDSPL2\PCGF6\FGL2\ALS2\SMARCA1\STAG2\SLC38A2\TTL\LOC417741\C1D\LRSAM1\NDUFA5\UGP2\FXR1\FAM3C\AK5\C6H10ORF46\IFT140\PRKCI\KRIT1\SLC39A9\SERPINI1\IRAK4\GAD1\GPATCH2\C1GALT1\DTL\NEK2\ZNF330\DIEXF\RAP1B\ABCE1\CDC20\CFL2\FAM177A1\GLO1\FBXO33\LARP1B\VSX2\ZDHHC17\GTF2A1\DCTD\PPPDE1\MEOX2\MTR\SPC25\RAPGEF5\GALNT3\SNX10\GLRX5\ABCB10\SLC4A10\CHN2\C3H6ORF120\EPYC\KERA\AAMP\T\PPP1R13B\IGF2R\CLASP1\MYLK\ESR2\MAD2L1\CLASP2\PCDP1\KIF18A\ITGA9\TAB2\MMADHC\NTRK2\FANCC\DEK\

		SLC41A2\C1H12ORF48\NUP37\BLOC1S5\TXNDC5\RIPK1\BID\ADCY2\SLCO1B1\SDHA\KIAA0528\SORBS2\SYNE1\SGCB\ARHGAP18\CYB5R3\TEC\CORO6\PI4K2B\MYH10\MEF2BNB\ELL2\DEPDC1B\GDAP2\SERINC1\C6ORF170\METTL16\ZUFSP\NPC1\EVC\MAMDC2\ABHD17B\MEP1B\GPR161\PCMTD1\OSTM1\PCNP\HJURP\NRG1\CHMP2B\LOC427306\SH3BP2\MXD4\USP25\LYRM2\LRRCC1\CCNE2\PTDSS1\PRADC1\U2AF1\ASAP1\GCM1\FAM49A\NRBP1\PIGA\EGFL6\EIF5B\REV1\C1H2ORF49\ARGLU1\DNAJC3\LOC418811\BORA\LECT1\GPALPP1\COG6\N4BP2L2\FLT3\MTMR2\ARL6IP5\SUV39H2\PACSIN2\GTPBP1\TMEM196\CLN8\EPM2A\LOC395933\CENPU\PTGR2\INCENP\PDDC1\MCM6\C7H2ORF76\PPP4R2\MFI2\RABGAP1L\RRN3\TEX2\PRR11\TCEB3\WDR82\TMEM131\CRISPLD1\POPDC3\SLIT2\ASF1A\ANLN\TBP\GLDC\SLC10A7\WIPF1\FASN\XIRP1\CNN3\ATP1B4\TBC1D22A\TLN2\HEATR3\LMAN1\C5H11ORF46\NAV2\HNRNPH1\RABEP1\YOD1\
P<0.05	tni-miR-455_L+1_2ss22CT23GT	ADAM28\CHMP7\RHD\B3GNTL1\IRF1\ZZEF1\NFS1\EIF6\ORAI2\DTWD2\ITGAV\ETFA\PINK1\DDX49\MLX\KIF20A\ESRP2\NELL1\CNOT8\AKR7A2\TRMU\ABCA8\KCNJ2\RSRC2\MTFR1\COL4A4\GIT2\TADA2A\PNAT10\TNFAIP1\BCAR3\MAPK8\ABCC1\KIF3A\RAB18\TMLHE\PCK1\CASP8\SLC38A2\SMNDC1\CDC42BPA\UBR1\PLEKHH1\LOC417800\C5H15ORF29\SSB\CCNL1\SLC38A4\SNX6\HEATR5B\PPPDE1\WLS\CRADD\OSBPL10\BAG5\POLDIP3\C22ORF32\NAGA\MYOZ2\NAA30\FBXO34\CASP6\PPP1R17\MTHFD1L\MBD5\RBFA\ALDH1L2\TXNRD1\VPS4B\PLEKHA5\FAM69C\SGCB\ARNTL2\TTLL12\FBLN1\ENO2\SGTB\MTX3\CRHBP\IMPACT\DTNA\SOBP\GPR89B\MYBL1\RARS2\TMEM30A\OTUD6B\NOV\PDE9A\AGPAT3\PRIM2\KLHL15\PRDX4\TAF1B\ASB9\CHST10\ARHGEF7\UBAC2\STK24\ENOX1\ATP7B\N4BP2L2\MED17\VAPA\VMO1\FZD7\RNF7\ALDH1A2\XKR9\CDKN1B\TAPT1\RIF1\POLG\SLC9A9\
P<0.05	tni-miR-205_R+2	C22H20ORF30\PCNX\HMGN2\LOC426699\BTN1A1\PAK1\PPP1R8\SRPR\ARHGEF16\PRKCZ\EDC3\NCAPD3\ACER1\ACSBG2\ORAI2\VAV3\SRP68\NCOA4\LOC415324\NR5A2\TMEM194B\HIBCH\ATG12\ULK1\CALCRL\HSD17B7\RCN2\BNIP1\TARDBP\SLC16A9\LITAF\C26H6ORF130\KIF20A\NFYA\SUB1\UBIAD1\ACSS2\HERC1\CLINT1\KRT222\ITM2A\TRMU\RALGPS2\TNFRSF1B\TNFRSF8\PLOD1\ZMYND8\PPP1CC\ZER1\TMOD3\ULK2\MYEF2\FBN1\DLG5\AP3M1\NUP210\DHX40\CLTC\SOX8\CDH11\IBA57\JMJD4\TADA2A\HPS4\XBP1\IPO7\TMED5\GORASP1\K123\C9H21ORF2\VIPR2\PDE6C\BRSK2\MARCH5\ALDH1A3\VIMP\RAB3IL1\CLCN5\ZEB1\TMLHE\ACBD5\VAPB\RAB22A\SNAPC5\EIF4E3\MAGT1\MRPS7\TMEM180\CHRDL1\NDUFS3\CELF1\RINT1\PUS7\ORC5\COL4A6\ATG4A\EDEM1\CRY2\ZFYVE19\TUBGCP4\PTK7\C3H20ORF3\KIF16B\LIN9\C3H1ORF55\TP53BP2\CTSO\GLRB\CAV2\DUSP10\SEC23IP\TMEM229B\SLC25A12\RAB33B\XPOT\IFNG\DYNC2LI1\CRIPT\AARS2\MMACHC\HERC3\LOC395824\SNW1\RAB3B\BTF3L4\ACSL1\PPPDE1\GALNT7\SCIN\TTC4\MEOX2\ANKMY2\STK31\CHN2\BRP44L\SHROOM3\PPP1R13B\SCYL2\ARL1\HSPBAP1\ATF7IP\L3MBTL2\CAMK2D\ANK2\TRANK1\TXNDC16\CYTH4\WDSUB1\MARCH7\RBFA\LOC100857286\KIF13A\IRF4\CDH20\MCM4\DAP\ERP44\CYP4V2\FRG1\GMNN\MAP7\LOC421049\FGFR1OP2\C2H8orf46\PPFIBP1\OTUD3\ATP10D\UPK3A\PDS5A\ING4\PPWD1\MRPS36\HDDC2\TNFRSF1A\EVC\WASF1\SS18\TMEM68\GNE\TMEM

		175\IDUA\USP45\GPR89B\SNX30\ABCC13\WHSC2\SYNCRIP\LCA5\TP53INP1\SLC25A32\PHF20L1\MYOM2\PXDN\DERL1\ROCK2\ITSN2\MTMR9\RP2\MGAT4A\ADPRHL1\TNFSF13B\UBAC2\STK24\DNAJC3\GPALPP1\SLC46A3\ODZ4\ARL6IP5\VDAC3\B4GALT1\GTPBP1\UBE2N\MRPL13\E2F5\C2H6ORF62\RPRD1A\LOC421975\EPM2A\AKAP14\CHP1\AK6\HABP2\PLA2R1\TANK\MCCC1\NDUFA10\GLT1D1\CDC42\TCEB3\NADK\TRIB2\OVAL\CRMP1\TAPT1\ACSF2\SLC1A2\PGRMC1\TBC1D22A\TBCD\LRRC31\GTF3C5\LMAN1\USP7\RAD50\YOD1\PDZD11\
P<0.05	tgu-miR-30a-5p_R+2	ARID4A\BNIP3L\NEFM\DCAF7\CSNK2A2\IL10\ANKHD1\SNAP29\UBE2L3\PSMD5\CPSF3L\ATP6V1B2\UBAC1\JMJD6\ORAI2\SDF4\NR5A2\AGO3\SORD\AP3S1\CFH\KCTD7\SLC15A4\GK5\INPP5K\RASA2\BECN1\MMD\PARN\NFYA\KIAA1191\ESRP2\DNAJC7\TRRAP\RAPGEF1\XPR1\AP1M1\DNA2\CSRP3\FAM81A\PNRC2\RNF111\RNF34\KIF4A\CACYBP\HVCN1\FAM214A\PDCD5\RGS9BP\NOL8\RASD1\PEMT\AP3M1\SLC25A36\SLC35A3\BTBD10\SELK\RHOG\ARHGEF3\ADRBK2\ABCD3\SPPL2A\TMEM41B\RAB7A\MYD88\GORASP1\WNT8A\HPRT1\GALNS\DDX46\HTATSF1\CARS\UBE2I\LOC422249\SEMA3A\KIF3B\UPB1\NCBP2\CHKA\BDH1\PPP1R2\POLR3E\CABP1\NRP1\RNF10\SYNPR\THOC7\NME1\STX16\RAB22A\MTMR8\SUCLG2\NPM3\PUS10\ATP7A\STK17B\GGA3\RNF114\TMEM180\ACTR1A\HSPD1\MOB4\GDI2\PGGT1B\NAPEPLD\LRRC17\PHTF2\MCTS1\SENP5\TRABD\PPP3R1\SHOC2\ADAM22\TBC1D8B\ZFP106\EHD3\GFRA1\CRDS2\DCAF4\PPID\SERPINI1\PLEKHH1\GXYLT1\YAF2\SRP14\C5H15ORF29\DOCK1\ELMOD2\SMARCA5\RAP1B\SRP54\HP1BP3\ZFC3H1\TBC1D15\LARP1B\NAP1L1\EIF2B2\PPP1R12A\FAAH\BIRC6\CCDC75\SCCPDH\PPPDE1\GOLGA5\AGR2\ITPK1\MACC1\GALNT3\DOCK7\CBX3\C5H14ORF129\CCNK\C3H6ORF120\WDFY3\DEPDC1\LRRC40\LHX8\GGCT\IGF2R\IL2\UBN2\SLC38A6\CYP2D6\POLR3H\XPNPEP3\STARD3NL\SLC35F4\FBXO34\FKBP9\UBE2D3\HECW1\TDRD7\THSD7B\IYD\NOL12\SOUL\KIF13A\TDG\TXNDC5\MKRN1\PRKDC\DDX11\ROPN1L\GALNT1\PRMT8\TDP2\SNX9\MAPK9\SRSF7\C6ORF115\SLMO1\PTPN2\LOC426296\HBS1L\SGCB\GRK6\EED\KIAA0930\SMC1B\MAN1A2\MLF2\SERINC1\NUS1\ESCO1\ABHD3\RABL3\WDR1\B4GALT6\ATG5\SDCBP\GJA5\CPA6\PTGR1\TMEM129\UBE2J1\SYNCRIP\NT5E\C1H21ORF59\OTUD6B\GCFC1\TP53INP1\ATP6V1C1\PHF20L1\PTP4A1\MYOM2\FER1L6\CALB1\DSCC1\SH3KBP1\TAF1B\GRHL1\FAM49A\NT5C1B\RHOB\MTMR9\REV1\CHST10\C1H2ORF49\TNFSF13B\LIG4\DZIP1\BORA\WDFY2\NUPL1\N6AMT2\TYR\ARL6IP5\LCORL\YWHAZ\KIAA1609\C3H2ORF43\CLN8\IMMP1L\C5H11orf24\MCM6\EAF2\DAB1\DCLRE1A\ADAP1\STX2\CDC42\NADK\C26H6ORF89\WDR82\WWP1\COL9A1\KDM3A\RSPO3\BMPR1B\CDCA7L\LOC100859142\CUL2\SLC1A2\WIPF1\GATM\EMB\DDX52\TRIM59\TBC1D22A\TBCD\ATP2A2\GTF3C5\COG5\NAALADL2\NUCB2\DDX19B\SMAD2\COL6A1\ZNF488\TNFSF15\CACNA1B\OCRL\SLU7\YEATS2\HSBP1L1\
P<0.05	tgu-miR-1784-3p_1ss11GA	RFK\SLC23A2\STARD4\SLC25A46\MFSD4\FBXW2\TERF2IP\DLGAP4\DCPS\DBR1\FAM18B1\ZZEF1\PSMD5\CHMP4B\H3F3B\PAIP2\UCHL5\DEF6\MED23\ENPP3\UBIAD1\RAB5C\ZNF207\POMT1\FAM114A2\WIPI2\HVCN1\FAM214A\PRPS1\LSM14A\TRIM37\CLTC\AATF\RHOG\HEATR6\SPPL2A\PAFAH1B1\CLK4\MYH11\SORL1\POU2AF1\CTSZ\ACBD5\FYTTD1\CDX4\APIP\CHRDL1\COPG2\BMPR2\ARRDC1\CT

		LA4\C3H20ORF72\GRM8\ATP6AP1\ITGA4\LCLAT1\SCFD1\CMPK1\ELAVL4\CASP3\SECISBP2\IGF2BP3\PCNXL2\LIN54\WARS\BRP44L\XRCC5\PECR\SLC4A4\IL8L1\CTNNB1\CASP6\UBXN4\MAP4K5\EPC2\ISCA1\RBFA\LOC100857286\TXNDC5\VPS4B\CDH20\DDX11\FUCA2\SGCB\PPFIBP1\UPK3A\FBLN1\ERAP1\WDR1\TMEM68\PCNP\SH3BP2\CYB5R4\HMGN3\TP53INP1\PTDSS1\SLC25A32\DDX3X\FAM49A\UBXN2A\EGFL6\TUBGCP3\MTMR2\FZD4\SUV39H2\LOC424098\LOC415708\MUSTN1\FAM48A\LUC7L3\CNN3\TRIM59\NUP188\TNFSF15\
P<0.05	tgu-miR-1662_L+1_1ss23TA	RASSF2\WDR36\RPS6KA1\NPHP4\HES5\DTWD2\F13B\CDC73\NBR1\BECN1\PROSC\SBNO1\CCZ1\YARS\EGFLAM\TEX264\RNF111\ORC6\VPS35\TNFRSF1B\SGPL1\HVCN1\SHISA5\MYEF2\TSEN2\SNAP47\WFDC1\EIF4G2\PITPNB\ARFGAP1\MTF2\TRIM66\PRKAG2\TBCCD1\AP2A2\MARCH5\HDX\KDELR2\TIPIN\USP12P1\APIP\CD44\KCTD2\UQCR10\CTDSPL2\FGL2\MAL\SHOC2\DCUN1D1\LBH\CDC42BPA\KIAA1715\WDR26\AKAP9\KRIT1\CCDC132\C5H15ORF29\COL1A2\FMN1\CLRN3\RAB33B\NKTR\SCFD1\ABCE1\TMEM184C\MME\KIAA0494\GLRX3\BIRC6\C4H4ORF41\COBLL1\PPP2R5C\SEC24B\NPFFR2\CYP2D6\RAP1GDS1\METAP1\UBE2D3\MAP4K5\MTHFD1L\DDHD1\LOC420864\SLC37A3\MKRN1\BCL2\ESR1\DAP\PYROXD1\TBC1D1\LRRC16A\LNX1\DCUN1D4\OCIAD1\USP48\TAPBPL\SPAG17\RABL3\WDR1\F2RL1\PGM5\ST3GAL6\GNE\HJURP\CYP7A1\USP45\VCPIP1\CASP8AP2\MXD4\WHSC2\SLBP\SYNCRIP\ADCY8\FBXO9\FAM49A\TGDS\MED4\ST3GAL5\LOC422426\INCENP\RGS1\ASIP\GLT1D1\TCEB3\RANBP2\EGFR\GYPC\LOC100859627\PRKAG2\CNTN1\FASN\TRIM59\SETD1B\TLN2\PSME3\CHRNB4\EIF4G2\
P<0.05	ssa-miR-29b-3p_R-1_2ss20TC21TC	TIMM17A\KDM5B\SPPL2B\CNTN2\XKR8\AP1G1\SLC2A8\GJC1\PIK3R5\SAMHD1\SNX19\CCNL2\MAN2C1\DGKD\WWC1\NCOA4\PEX12\ACOX1\PLEKHB2\FAM168B\CNOT1\PAIP2\LZIC\HSD17B7\DUSP19\INPP5K\MMD\DDX49\LOC100857289\SUB1\HSDL1\CRLF3\ERLIN1\SCYL3\UBIAD1\GOLGA7\TAF8\ARRDC2\SKP2\NECAP2\ARPC3\COL6A3\APBA2\PPM1M\CRYBA1\DNA2\MFAP3\PABPC1L\SRSF10\KIF4A\TRMU\FAM20B\BLMH\ANGPTL1\CYP3A4\RGS9BP\GLE1\USP22\FLCN\C17ORF39\LOC769755\ISY1\DPM2\COL11A1\KBTBD5\CHTF18\MRM1\BCMO1\NDUFB10\LPPR5\PNAT10\RHOG\WFDC1\CDX1\C20H20ORF11\YTHDF1\COL7A1\PLS3\MMP11\SMTNL2\COL6A2\HPS5\H2AFY\HTATSF1\C12H3ORF19\TRA2B\MAPRE1\CDK5RAP1\ATP5C1\NCBP2\PFKP\TLR4\APOA4\RMI2\PCSK6\SYNPR\ARCN1\ITGB2\LOC424919\STX16\RAB22A\DPYSL3\BTG4\STK17B\HN1\DCX\UBE2V1\HSPD1\MOB4\PUS7\ADAMTS7\FGL2\CROT\LRSAM1\PPP1R21\SESTD1\AMDHD2\CHRNA1\C2H7ORF64\GAD1\GPATCH2\C5H15ORF29\COL1A2\INTS7\CAMK1D\DIEXF\MCFD2\GTF2E2\MED12L\ANGEL1\LOC100859230\CASP3\C4H4ORF41\SCIN\AREGB\TARBP1\PGM1\LIN54\SEC31A\PHF10\SERPINH1\SLC44A5\GADL1\PPP1R13B\KIF26A\SEC24B\SULT1B1\SNRPD1\RRP7A\NAGA\C5H14ORF135\PDGFB\CASP6\LOC420748\WDHD1\RAP1GDS1\PPP1R17\KDELR3\MMADHC\MPST\JARID2\TDG\ADAM12\AKR1D1\C1H12ORF35\BPGM\PPIE\LOH12CR1\FAM69C\CYB5A\CEP192\SSPN\PHC1\ANKRD52\RFC1\ZNF384\DEPDC1B\TNFRSF1A\REV3L\WASF1\HAUS6\ZDHHC23\TMEM68\ZFP92\CPA6\ADD1\RNF4\HMGN3\RIPK2\TMEM30A\IFNGR2\CCNE2\PLEKHF

		2\DSCR3\NOV\TOP1MT\DDX3X\PRIM2\COL21A1\PXDN\ENPP2\KRTCAP3\REPS2\STMN4\COL4A1\COL4A2\SETDB2\SLC25A15\FLT3\CRYL1\FAM76B\CWC15\PPME1\VAPA\VDAC3\MID1\UBE2N\PDGFRL\C5H11orf24\CZH9ORF80\PLA2R1\DNTT\BIRC2\XKR9\TRIB2\COL9A1\CRMP1\CRTAP\NFIA\OAT\ZBTB34\LOC100859627\MYO5A\TRIM59\COL6A1\IREB2\CACNA1B\PSMD13\
P<0.05	ssa-miR-125a-5p_R+2	ARMC1\BF1\STARD4\CD79B\RPS6KA1\LIN28A\ENTPD4\TIRAP\WBSCR16\CIAPIN1\ABI3\ANP32B\ACSBG2\PRPSAP1\APTX\HEMK1\SEC16A\MPP3\RNF123\GMPPB\CREB3\INPP5K\ENPP1\TRPC4AP\SURF1\CRLF3\HDAC1\EIF3I\SBNO1\LACTB\ACTR5\AKTIP\USE1\LIFR\TMEM38A\TRIT1\MINA\TOMM34\ADA\TTPAL\SGPL1\YIPF6\CTNS\PHKA1\JMJD4\RHOG\NONO\GBF1\DENND6A\SNX12\CDX1\ABHD6\DCAF12\PAFAH1B1\PLS3\MMP11\GORASP1\MCM2\GALNS\AQP12\SMG1\RNF213\RBM7\HRASLS\CLCN5\YME1L1\ZFYVE27\RAB11A\CDX4\C6H10ORF76\FBXW4\BCO2\KCTD2\PTPN1\UBE2V1\FBXO18\NR1H3\PCGF6\SUMF1\CIB1\ITPKA\NDUFA5\NRK\LBH\ENTPD2\SNAP23\ZFYVE1\SLC39A9\ASNS\PROX1\LOC422448\PREPL\LOC417848\MMACHC\ANGEL1\NKX6-2\SNW1\ADCK1\LOC100859230\SMEK1\SCIN\DFNA5\TARBP1\KERA\TMEM66\ANKS1B\MGP\SERHL2\NAGA\TRANK1\ENPEP\CASP6\ITGA9\SOUL\CCDC148\CDC14B\BLOC1S5\IRF4\PLEKHO1\STX17\MTUS1\LOC426218\PPAT\ENPP7\GPR155\EDARADD\MRPL10\MRPL51\MRPS36\GREB1L\F2RL1\TRAF3IP2\MAMDC2\MDN1\NT5E\RIPK2\TSTA3\SEPT6\CLN8\C26H6ORF89\DGKZ\XIRP1\ZNF335\MYO19\GTF3C5\FLOT2\FAS\TNFRSF10B\HSBP1L1\
P<0.05	sha-miR-140_R+2	C22H20ORF30\LOH11CR2A\PRNP\SLC25A46\ITGB3\CHMP7\SPPL2B\LONP1\CYB5B\RBBP5\CACNA1S\DHODH\KDM1A\CSNK2A2\TAT\NUDC\FAM125B\APLP2\EDC3\CASP9\COX10\PLLP\TTC1\CCNL2\WDR45L\CWC25\SEPT9\DGKD\UQCC\C19ORF6\NR5A2\SORD\HSPA9\SUMF2\CRYM\ZMYM4\GJA4\ASL2\C19H17ORF85\WDR5\HSD17B7\DNAJC9\KIF1B\INPP5K\NUDT3\RFNG\BNIP1\TARDBP\MLX\PEF1\C26H6ORF130\EIF2B1\ERLIN1\EIF3I\GBGT1\KIAA1191\BSDC1\GFI1B\LOC101749631\TMEM38A\TARSL2\ASB18\SRSF10\TOR1A\ADA\VPS26A\TAF9\ADAM10\MORN3\TSPAN15\TNFAIP8L1\RALGPS2\P4HA1\COL18A1\ASB6\PPP1CC\AR\P2RX5\CTNS\ENDOG\CPSF4\CLIC2\DHRS7B\PSAP\C17H9ORF16\IARS\RASD1\TAF4\WDFY1\FUT11\SLC25A36\HHATL\IBA57\BRCC3\HELLS\ARHGEF3\TPST2\EMC8\CHRNA4\IPO7\RAP1GAP2\MLST8\LOC420419\MYD88\LTC4S\RRAS2\BTBD1\TNRC6A\WNT8A\ARFRP1\ZNF326\PIK3C2A\EARS2\COL6A2\PAXIP1\PDPK1\C9H21ORF2\TNNT3\RHCG\TMEM41A\OAF\TAF3\CBL\POU2AF1\LIMK2\SPPL3\RBM7\LOC100858827\RMI2\HRASLS\CADPS\LPP\PRKAB1\ANKRD40\C7ORF26\RRP12\SLC9A3R1\KIAA2022\APIP\STK17B\PTPN1\TSEN54\NDUFS3\TYW5\MADD\GRAMD3\ARFGAP2\ATG4A\SUMO1\CRY2\NDUFS1\GTF2H4\ACSS1\SHOC2\UGP2\EPCAM\VMA21\GUCY1A3\RXFP1\BAG3\MIA3\PIGH\SCHIP1\MYO3B\ATPBD4\RAP1B\CRIPT\KIAA0391\GEMIN2\CAPN1\FBXO33\TSPAN1\HERC3\GLRX3\EIF2AK2\PPPDE1\SCIN\ITPK1\HDAC9\STK39\SIPA1L2\ACTA1\SERPINH1\NEDD1\METTL6\MRPL3\MTHFD1\CCNA2\NAGA\CCK\TMC2\METAP1\L2HGDH\EIF3L\TNFAIP6\FANCC\RIC8B\C1H12ORF48\IRF4\MKRN1\DNM1L\USP18\LOC100859438\PCM1\GTF2H5\SLMO2\LOC421049\RELL1\CC2D2A\LYSMD3\KIAA0907\FAM172A\CAST\MSX1\HAUS6\BOC\C3H6ORF203\CD200\CD247\GPR89B\HSPA13\PEX2\TMEM30A\IFNGR2\RUNX1\CHAF1B\U2AF1\LOC39638

		0\GSTA\KLHL15\PDIA6\ACE2\MTMR9\MYO16\LIG4\ELF1\WBP4\C1H13ORF15\MED4\HSPH1\DDX10\MED17\UQCRH\COTL1\KLHL5\PSMD3\C1H21ORF33\INVS\NKAP\SLC25A14\PTGR2\INCENP\C7H2ORF76\MCCC1\NDUFA10\RPE\RNF7\FYB\EXOC7\KPNA1\LOC419404\TAPT1\PDLIM5\TMED8\HMOX1\HOXA11\IRF2\ATP2B1\CUL2\ARSB\ZBTB34\CNTN1\IRF7\TRIM59\EIF5A2\COG5\P4HA1\KIAA0319L\BET1\
P<0.05	sha-miR-125a_R+2	ARMC1\BF1\STARD4\CD79B\RPS6KA1\LIN28A\ENTPD4\TIRAP\WBSCR16\CIAPIN1\ABI3\ANP32B\ACSBG2\PRPSAP1\APTX\HEMK1\SEC16A\MPP3\RNF123\GMPPB\CREB3\INPP5K\ENPP1\TRPC4AP\SURF1\CRLF3\HDAC1\EIF3I\SBNO1\LACTB\ACTR5\AKTIP\USE1\LIFR\TMEM38A\TRIT1\MINA\TOMM34\ADA\TTPAL\SGPL1\YIPF6\CTNS\PHKA1\JMJD4\RHOG\NONO\GBF1\DENND6A\SNX12\CDX1\ABHD6\DCAF12\PAFAH1B1\PLS3\MMP11\GORASP1\MCM2\GALNS\AQP12\SMG1\RNF213\RBM7\HRASLS\CLCN5\YME1L1\ZFYVE27\RAB11A\CDX4\C6H10ORF76\FBXW4\BCO2\KCTD2\PTPN1\UBE2V1\FBXO18\NR1H3\PCGF6\SUMF1\CIB1\ITPKA\NDUFA5\NRK\LBH\ENTPD2\SNAP23\ZFYVE1\SLC39A9\ASNS\PROX1\LOC422448\PREPL\LOC417848\MMACHC\ANGEL1\NKX6-2\SNW1\ADCK1\LOC100859230\SMEK1\SCIN\DFNA5\TARBP1\KERA\TMEM66\ANKS1B\MGP\SERHL2\NAGA\TRANK1\ENPEP\CASP6\ITGA9\SOUL\CCDC148\CDC14B\BLOC1S5\IRF4\PLEKHO1\STX17\MTUS1\LOC426218\PPAT\ENPP7\GPR155\EDARADD\MRPL10\MRPL51\MRPS36\GREB1L\F2RL1\TRAF3IP2\MAMDC2\MDN1\NT5E\RIPK2\TSTA3\SEPT6\CLN8\C26H6ORF89\DGKZ\XIRP1\ZNF335\MYO19\GTF3C5\FLOT2\FAS\TNFRSF10B\HSBP1L1\
P<0.05	pma-miR-181a-5p_R+3	LOH11CR2A\RASSF2\LSM7\STRADA\ACOT7\PAK1\FAM125B\HSPA5\CCDC43\C20H20ORF24\ARMC8\SAMHD1\CLTCL1\PI4KA\CNTRL\WDR45L\NFS1\PSMD5\ATP6V1B2\WWC1\GNB1L\TRIM33\STXBP3\PRKCD\C15H22ORF25\LOC415324\AGO3\DTWD2\GOT2\GBAS\C10ORF58\UCHL5\PIK3CD\LZIC\C19H17ORF85\ATR\ZC3H15\GK5\UHMK1\DNAJC12\C26H6ORF106\RASA2\PHACTR4\TARDBP\SUGP2\MMD\MLX\UBIAD1\ACSS2\BARD1\IFT52\LPCAT2\TOP1\LIFR\SLC35E1\P2RX4\MYPN\FAM189A1\IQCA1\STK4\PNRC2\SRSF10\RNF111\DHRS3\GOSR1\SGPL1\P2RX5\MAD2L2\PDAP1\RGL1\NOL8\COPS3\DUT\MKRN2\TRIM37\GTPBP5\TUBD1\VCAM1\SLC25A36\GNG13\SASS6\APPBP2\ADAMTS18\JMJD4\LPPR5\ARHGEF3\SYNGR3\RNF141\TSC2\KLHL36\PITPNB\EMC8\SPPL2A\GCLM\PAFAH1B1\RAP1GAP2\MTF2\LOC100858792\KLHL13\ABTB1\KIAA0753\MOSPD1\PRKAG2\MLL3\CXCL14\PDPK1\SRPRB\KIAA1024\SEMA3A\KIF3B\FRA10AC1\BRSK2\TOLLIP\TAF3\PLOD2\IL13\CHKA\RPTOR\ARRDC4\ALDH1A3\RAB35\MTPAP\ABCC2\ARCN1\TMLHE\ABCC3\STX16\MTMR8\LOC416354\CHIC1\EME1\SALL4\GGA3\ACSL4\CELF1\MTAP\CASC4\PDCD11\LRRC17\PHTF2\SMARCA1\MCTS1\SENP5\GTF2H4\LOC424109\PPP3R1\C1D\IDH1\ATP6AP1\LCLAT1\CDC42BPA\C3H1ORF55\LEFTY2\GUCY1B3\PRDX3\SEC23IP\C2H7ORF64\MARK1\GXYLT1\PUS7L\RARRES1\RRP15\C1GALT1\RAB33B\PROX1\SRGAP1\STXBP6\SCFD1\TMEM184C\CFL2\KIF6\PSMA6\LRBA\MBIP\SCLT1\KANK1\TBC1D15\NAP1L1\TTLL5\MBNL1\MED12L\OSTC\ELAVL4\BIRC6\CC2D1B\SCIN\ANKMY2\ITPK1\RAPGEF5\INADL\DOCK7\LYST\PGM1\CBX3\COBLL1\C5H14ORF129\CCNK\C3H6ORF120\RFTN1\DCN\KAT2B\GPD1L\BAG5\KIF26A\CLASP1\ZDHHC3\UBP1\CAMK2D\SLC12A8\EIF3M\

		CASP6\ITGA9\STK17A\HNMT\NTRK2\CTSL2\TGFBR1\LOC101750080\DEK\TXNRD1\HSP90B1\EEF1E1\BLOC1S5\SLC37A3\STRAP\MGST1\PRMT8\LOC420992\LRRC16A\TFB1M\NPEPPS\PPAT\CEP76\BCLAF1\IGF2BP2\OCIAD1\TEC\M6PR\EDARADD\PI4K2B\CCDC58\MBLAC2\HDDC2\TNFRSF1A\FAM26E\LYAR\MLLT3\HAUS6\MAMDC2\GPR161\TCEA1\TMEM68\KIAA1524\CHMP2B\LRRCC1\ELOVL4\DECR1\PTDSS1\DOPEY2\ATP6V1C1\NOV\RAD21\DDX3X\OC90\DST\APOO\DSCC1\SH3KBP1\PDHA1\TAF1B\LAPTM4A\PIGA\RAB9A\STMN4\RP2\SLC9A7\GAS6\ATP4B\TNFSF13B\LIG4\IPO5\RBM26\BORA\MZT1\RNASEH2B\GDPD5\TXNL4A\SUV39H2\GZMK\CASP2\PACSIN2\LOC101750282\CERK\E2F5\C2H6ORF62\IFNGR1\SLC25A14\PDDC1\EAF2\PLEKHA3\C2ORF47\SPECC1L\RNF7\PRR11\SLC13A2\C26H6ORF89\C19ORF44\RANBP2\TMEM131\WWP1\APP\MTO1\KDM3A\DNAJA1\HOPX\ATP2B1\SLC10A7\ZBTB34\GATM\PRKAG2\NELL2\GRIA2\AMACR\TBC1D22A\ATP2A2\NAALADL2\NUP188\EIF4A2\CD36\REL\LSG1\EPS8L2\LOC422654\
P<0.05	PC-5p-479025_5	RFK\GFPT1\TCF25\ARHGAP25\DPAGT1\DDX42\ZBTB48\PM20D1\TBC1D22B\MAN1C1\DBR1\RAB14\PHF20\EDC4\PUSL1\PRKRIP1\SDF4\DYNLRB1\ACOX1\UNC45B\NUBP2\ITGB4\DNAJC18\PIK3CD\LZIC\ZC3H15\MAP2\DNER\SUGP2\SURF6\C26H6ORF130\LOC425137\ALDH9A1\SGK2\LOC100859426\PLAT\TRIT1\PPM1M\PNRC2\KIAA1279\TSPAN15\DIABLO\FOXK1\RPS14\PSAP\MTFR1\ACACB\DPM2\PTGS2\NUP210\ANKRD13A\SOX8\TCOF1\ST6GAL1\CDX1\C20H20ORF11\COL7A1\PAFAH1B1\RUVBL1\MST4\XYLB\IL2RA\K123\DDT\SRPRB\DNAJB6\FOPNL\ABCC1\RPRD1B\IL13\CHM\LIMK2\RNF185\TLR4\ACOX2\SYNPR\CLPX\CTSZ\ZFYVE27\STX16\RRP12\TRAPPC4\NMRAL1\CD82\WSB2\GRAMD3\CFLAR\RALGAPA2\PEX16\CDK15\CRY2\STAG2\APLF\NOXA1\AK5\VMA21\MTMR1\C6H10ORF46\SP3\CTSO\PDGFC\BAG3\METTL8\MPP5\IRAK4\TMEM206\SNX6\CCDC167\VLDLR\ABCD4\FAM98A\TXNDC12\PPPDE1\SMEK1\MAP1LC3C\CHGA\NUDT9\TSNAX\COBLL1\SEC31A\DAZL\RAB5A\ACTR6\PTPLB\DDX47\ATF7IP\LOC425756\TOB2\MAD2L1\SNX4\FKBP9\NOL12\TNS3\MPST\BAZ2B\TDG\SLC37A3\CDH20\LOC100858230\CLPTM1L\ANP32E\MTHFD2\TMEM106C\MAP7\KDR\PTPN6\CCLI10\RFESD\GRAMD1C\NANS\CHMP2B\AFAP1\EPHA7\RNF4\RIPK2\IFNGR2\LY6E\COL22A1\ICOSLG\NDRG1\MAOA\GCM1\PDK3\FBXO32\DERL1\SNTB1\MBOAT2\REPS2\REV1\STK24\WBP4\SPRYD7\MTMR2\NAALAD2\MRPL48\VAPA\CD47\POPDC2\GATA4\XRCC3\CHP1\SYK\DCLRE1A\NPPB\LOC419404\LOC419830\BIRC2\AFAP1\ANLN\HOXA11\CRIM1\TMEM169\GABPB1\NELL2\CYP24A1\XIRP1\RPL39\TRIM59\EXOSC2\DDX19B\COL6A1\EIF4A2\PSME3\HSPB7\POLE3\ZIC1\EFTUD2\GSPT1\ATCAY\
P<0.05	ola-miR-199a-3p_R+1_1ss22AG	C22H20ORF30\STARD4\RPS6KA1\LAPTM5\CXXC5\FAM125B\WBSCR16\RABGAP1\APLP2\SNAP29\RRAGC\CNTRL\INPP5B\ACOX1\PLEKHB2\FBXW11\TRAPPC3\ATG12\KTN1\INPP5K\PDXDC1\RFNG\BECN1\TADA1L\ASH2L\CRLF3\TFEB\LPCAT2\PLAT\TMEM38A\ALDH4A1\SMARCE1\PIGT\GEMIN5\CNOT8\AP1M1\MFAP3\ITM2A\GPT2\CCNB2\BET1L\ADAM10\TTPAL\RCAN3\TBCA\PPA1\TGM3\COL4A4\URM1\SHC4\CDH11\SLC25A36\CYB5R2\PIK3CB\PNAT10\ARHGEF3\LOC768701\ADRBK2\NLK\SQRDL\SCD\SPPL2A\BCAR3\ZFAND4\MYD88\KIAA0753\KLHL3\ACTR3B\OSBPL5\ABHD17C\NDE1\SEMA3A\TOLLIP\ARL6IP1\ZEB1\ABCC2\RHBDF1\STX16\MTMR8\BTG4\C20H20ORF4

		3\KIAA1841\APIP\AHCYL2\SPATA2\CELF1\AOX1\AOX2P\CUBN\ITGA8\FXR1\LCLAT1\CRDS2\PRDX3\CTSO\YAF2\DCAF17\IL22\CRIPT\CFL2\PPP2R3C\GEMIN2\HSPA4L\FCF1\CMPK1\PAPSS1\BTF3L4\CC2D1B\PPPDE1\AGA\TMEM48\ITPK1\STK39\IGF2BP3\JAK1\COBLL1\ARHGAP24\MYO10L\CRADD\TECPR2\EXOC3L4\BIN1\BCL2L14\MAP3K4\HIF1A\ZDHHC3\ANK2\FSHB\AVL9\TDRD7\TAB2\ERO1L\NTRK2\RBFA\TDG\HSP90B1\TXNDC5\ECI2\MKRN1\LOC418170\BPGM\DERA\GALNT1\FGL1\MBP\CNOT7\PPAT\OPTN\EZR\SGCB\PPFIBP1\ZNF384\ARSK\ERAP1\SGTB\GFM2\ZUFSP\HAUS6\RORB\B4GALT6\CD200\FNTA\HACE1\PDZK1\HSPA13\CTBP1\TPD52\PGM3\MMP16\CCNE2\ITSN1\TOP1MT\KDM6A\KLHL15\TAF1B\GRHL1\TXLNG\MOSPD2\EGFL6\FDFT1\CD2AP\CHST10\PDCL3\UXS1\SULT1C3\FRY\SLC46A3\ELMOD1\CEP57\PTPN3\CHAD\C3H2ORF43\SPACA1\NOA1\INCENP\DCLRE1A\PPP4R2\AURKA\RABGAP1L\NDUFA8\C19ORF44\FLNB\RB1\POU1F1\RAD17\NOXRED1\CEBPZ\GLDC\GABPB1\PGRMC1\DDX52\ATP1B4\LIN7B\LOC771069\LEPROTL1\
P<0.05	ola-miR-126-5p_R+1_1ss1CN	ARMC1\DCAF7\AP3S1\MAP2\FEN1\HK1\TTPAL\RCAN3\RSRC2\ALDH3A2\MTFR1\KLHL13\RAB22A\MLLT10\MTMR3\CELF1\GRM8\C2H7ORF64\C5H15ORF29\DYRK2\ITPK1\GLRX5\HELQ\FRMD1\DDX47\MTHFD1\CTNNB1\KDELR3\SHPRH\PDK1\FAM114A1\ACTBL2\GPR161\ALDOB\TMEM129\MMP16\IMPG1\OTUD6B\ACP1\CALB1\ZC3H13\ARL6IP5\KLHDC2\REEP5\NBL1\
P<0.05	oha-miR-7-1-3p_1ss10GA	ARID4A\GFPT1\TIMM17A\PCNX\RPS6KA1\DCAF7\TERF2IP\TMEM57\SAMHD1\FAM18B1\PLAA\PRKRIP1\LRWD1\VAV3\STXBP3\POLR2D\PLEKHB2\FBXW11\ENO1\C26H6ORF106\RASA2\TADA1L\C26H6ORF130\TMED2\KIAA1191\HAUS8\TMEM38A\UBR4\GPT2\ADAM10\XPO1\CCPG1\URI1\TNR\SGPL1\HVCN1\PPP1CC\GLE1\PEPD\DUT\PTGS2\TUBD1\TK2\INTS2\SASS6\PIK3CB\LPPR5\RHOG\ST6GAL1\SPPL2A\ARFGAP1\DCAF12\RAP1GAP2\DGCR2\GORASP1\TGFBI\OSBPL5\HTATSF1\PANK1\C16ORF45\VIPR2\ZMYND11\BDH1\USP28\BLNK\PITRM1\ACOX2\RMI2\LARS\RBM27\MTMR8\NAT9\STK17B\SF3A1\DCX\TMEM164\TRIM8\PHTF2\SLC24A6\ALS2\CDK15\CRY2\AFTPH\SAMD13\EPCAM\PIGK\TRMT6\SP3\ETFDH\LOC424998\GXYLT1\GPATCH2\ACADSB\C1GALT1\C5H15ORF41\FASTKD1\ZNF330\CRIPT\NAP1L1\TMEM69\POLR1C\SMARCAD1\CCDC75\UFSP2\DPY30\ECHDC2\AGA\FBXO8\CPSF2\CBX3\COBLL1\PSMD14\WLS\KERA\ANKRD13C\BAG5\UHRF1BP1L\MAP3K4\CENPC1\PTPLB\UBA6\TTC26\POLDIP3\CCK\ITGA9\METAP1\ADCY1\GKAP1\PRKDC\DROSHA\DDX11\TMF1\VIP\CNOT7\SGCB\ARHGAP8\LIMCH1\AK3\ERAP1\FAM184A\REV3L\UHRF2\FIG4\SEC63\TMEM68\IDUA\HACE1\USP45\GPR89B\MAP3K7\PEX2\SCAF4\OTUD6B\TP53INP1\CHAF1B\SLC25A32\COL22A1\U2AF1\DDX3X\DERL1\MBOAT2\REPS2\COL4A1\ABHD13\ARGLU1\DNAJC3\ZAR1L\HMGB1\MTMR2\SUV39H2\UBE2N\CERK\LOC776051\C3H2ORF43\ST3GAL5\NKAP\PPP6R3\MCM6\TM4SF1\RNF7\NOS2\FAM48A\CGA\TMPO\LUC7L3\RAD54L\GATM\NELL2\FASN\EMB\MYO5A\COL17A1\TBC1D22A\ATP2A2\PSME3\SF3B3\LIN7B\BUB1B\APITD1\
P<0.05	odi-miR-7_R+2	ARID4A\C20H20ORF24\SNAP29\CASP9\RALGAPB\MIB2\CCNL2\SMU1\SYT15\ACOX1\TMEM194B\EP400\SMN\PPARD\RASA2\BKJ\TSR1\TARS\ESRP2\SKP2\LPCAT2\FEN1\UBLCP1\DHTKD1\HEATR2\SMARCE1\FAM189A1\PNRC2\PABPC1L\BET1L\VPS26A\RIC8A\KIF4A\H

		NF4A\SH3GLB1\BRAP\RGS9BP\C14H17ORF103\PTGES2\CENPI\CDK9\WDFY1\TK2\BCAS3\SASS6\TM9SF3\PITPNB\USP10\KSR1\ARFGAP1\GCLM\RRAS2\PITPNM3\SRPRB\DNAJB6\VDAC1\TSSC4\ARHGEF12\RPRD1B\POU2AF1\KLHL4\RNF185\PDHB\SPG21\LPP\CTSZ\LOC420491\SUCLG2\EME1\NMRAL1\CHL1\NT5C2\F2\ALS2\RPL32\CCNYL1\SLC38A2\CROT\IDH1\SAMD13\TSPAN12\LCLAT1\VMA21\CTSO\PEX1\MECOM\C2H7ORF64\TMEM229B\VTI1B\GAD1\GPATCH2\C5H15ORF29\HHAT\TRERF1\DYRK2\RAP1B\HSP90AB1\LOC422497\NPC2\SNCA\EIF2AK2\RYR2\ITPK1\COG2\AGT\CRADD\CRYZ\SHROOM3\CKB\RALB\IL8L1\DDX47\CLASP2\ST13\HERPUD2\UBE2D3\ISCA1\FANCC\RIPK1\DERA\CCDC127\CLPTM1L\KIAA0528\EFHA2\CNDP2\KRAS\ADHFE1\ATP10D\ZNF384\EPHA1\ZYX\TRIM23\SGTB\SPICE1\CZH5ORF28\TNFRSF1A\FAM26E\RCL1\ZDHHC23\CAMK4L\SDR16C5\UBXN2B\PREX2\C6ORF162\TP53INP1\CHAF1B\LY6E\WDR4\PRIM2\FBXO32\CALB1\RAB9A\MTMR9\CYP39A1\CHST10\TNFSF13B\COG3\POLD3\FOLR1\PSMG1\DPT\MCM6\PLEKHA3\RNF7\BIRC2\ATP6AP2\KDM3A\PDLIM5\RIF1\NELL2\PGRMC1\TBC1D22A\ACTL6A\PSME3\PYGB\LOC425470\PCSK7\P4HA1\MAP3K14\TNFRSF10B\NGEF\
P<0.05	oan-miR-19b-1-5p_R+4	ARMC1\RASSF2\CHMP7\DCAF7\NR0B2\CSNK2A2\MAN1C1\SRPR\POLRMT\ADRA1B\MECR\CAMKK1\SLC22A7\ELMO3\LOC415664\PMPCA\C15H22ORF25\DTWD2\ETF1\HSPA9\C10ORF58\VKORC1L1\ERGIC1\ARID5B\AGFG1\MMD\SCYL3\ESRP2\CEL\LPCAT2\FN1\CHD9\SALL1\KRT20\LRRFIP1\STX6\SYS1\TRMU\BLMH\XPO1\SGPL1\CD74\P2RX5\MAPK6\ECM2\SET\BUD31\LOC768735\PTPN11\RASD1\URM1\DLG5\DPM2\DHX40\COL11A1\USP32\PTH\RNF141\HIF1AN\SPPL2A\JPH3\TMEM41B\RAP1GAP2\USP8\MGAT4B\MYD88\ABTB1\COPB1\LRRC8B\GTF2H1\PCBD2\CARS\TTLL9\KIAA0430\ABCC1\NOX1\PM5\AP2A2\NCBP2\IDE\PFKP\PCNT\BUD13\NME1\SVIL\TAOK3\CTSZ\FYTTD1\MTMR8\RAB11A\TMEFF2\MAGT1\RNF114\RTN4\ADAMTS7\CASC4\CFLAR\NEURL\STRADB\MCTS1\COBRA1\NDUFS1\TYRO3\ACSS1\NELF\NHLRC2\PIGK\LIN9\LOC431656\LOC100857197\PRKCI\PDGFC\ETFDH\VEPH1\C5H15ORF41\DYNC2LI1\NUBPL\TMEM184C\SNX6\CNOT2\KIF6\GLO1\AARS2\LOC422497\TMED10\CMPK1\ELAVL4\YIPF4\TBCK\GTF2A1\ITPK1\RAPGEF5\SNX10\FRMD1\CRADD\LRRC40\BRP44L\AMPH\ATP2C1\MYLK\KIAA1143\METTL14\CCDC93\DLGAP5\ADH1C\UBXN4\FBXO7\RMI1\C22ORF28\CDC14B\GPLD1\RBM24\NUP153\CREB3L2\DAP\LOC418170\BPGM\FH\CCDC127\C12ORF32\LOC420992\CNOT7\FAM69C\SERAC1\SRSF7\C1H12ORF11\KIAA0930\RELL1\NAT10\EPHA1\ZUFSP\B4GALT6\PCMTD1\OSTM1\QRSL1\ALCAM\TMEM175\MPZL1\VCPIP1\UFL1\MAP3K7\TCEB1\USP25\CTBP1\CHMP3\IMPG1\TMEM30A\MAOA\DDX3X\ADCY8\GRHL1\LAPTM4A\PIGA\RAB9A\INTS9\MTMR9\UBE3A\C1H2ORF49\RASA3\TNFSF13B\IPO5\DNAJC3\MYCBP2\C1H13ORF15\SLC46A3\SPDYA\VAPA\LOC426128\VMO1\CD47\EMP1\WDR48\HCRTR2\ZP4\ASIP\NOS2\NPPB\NADK\RBM6\AZIN1\SMS\WWP1\ATP6AP2\SOD1\CASQ2\NCF4\CDCA7L\LUC7L3\SLC10A7\CD151\DDX52\BIRC5\EIF5A2\NBL1\SLCO4A1\RCC2\LEPROTL1\
P<0	oan-let-7f-1-	RFK\DCAF7\GALK2\RASSF5\AP1G1\LOC100857226\C20H20ORF24\TMEM57\GTF2I\SSU72\RBM39\MAK16\GABRA1\MRPS34\TEK\ARVC

.05	3p_R+2_1ss8AG	F\C15H22ORF25\KLHL6\AP3S1\CDC73\PLS1\C19H17ORF85\ZC3H15\INPP5K\MED23\ARMC6\COMP\MTOR\SLC6A2\TOP1\SUN1\RNF111\SLTM\WIPI1\HVCN1\NCOA3\ZER1\MAPKAPK5\COPS3\LSM14A\PRPSAP2\WDFY1\GHRHR\VCAM1\SASS6\SNAP47\PLCG2\DYNLL2\TBL3\DENND6A\C20H20ORF11\TMED5\RPN1\BTBD1\MST4\ZNF326\IL16\HTATSF1\ATP12A\FOPNL\BRSK2\RASSF7\MARCH5\CHKA\CADPS\RNF214\SYNPR\LEPREL1\BCL6\RAB18\STX16\TRAPPC4\CBLN4\UPRT\CHL1\MRPS7\NXT2\ALS2\NET1\SLC38A2\GTF2H4\TUBGCP6\TRABD\WASL\SAMD13\LBH\DLX5\DCAF4\PRKCI\KRIT1\CAV2\SMOC1\IFRD1\PDCD10\DCAF17\C5H15ORF29\C5H15ORF24\BUB3\LOC422448\ELMOD2\RASSF3\DIEXF\G2E3\SMARCA5\RAP1B\GTF2E2\MBNL1\YIPF4\PPPDE1\LRRC42\TMEM59\UBR7\GSC\HNRNPA2B1\CBX3\NGLY1\GRSF1\MAP3K4\MRPL3\IGF2R\MAD2L1\METTL14\FBXO34\LRRFIP2\HECW1\TNFAIP6\C1H12ORF48\RPP40\SLC15A5\SPTLC1\TMX3\FUCA2\CWH43\EDARADD\MYH10\C1S\PRRC1\DEPDC1B\ERBB2IP\MTX3\FABP7\SERINC1\WDR1\ZBTB49\MSX1\SS18\TMEM175\UBXN2B\BRP44\GPR89B\CHMP2B\UFL1\TCEB1\NOP14\WHSC2\RIPK2\PTDSS1\GNOT1\NOV\RAD21\U2AF1\PHF20L1\CALB1\SYAP1\GTPBP6\CD2AP\CYFIP1\BORA\N4BP2L2\YWHAZ\TATDN3\NKAP\PPP6R3\CKMT2\MCM6\MCCC1\PPP4R2\RNF7\DENR\KPNA1\SLMAP\SNX13\TEF\TP53BP1\GABPB1\XIRP1\TMEM120B\TBC1D22A\GHRHR\PELI1\EIF4A2\
P<0 .05	mmu-miR-8094_ L+1R+3_1ss3AG	TIMM17A\ARHGAP25\SLC25A46\ENTPD4\GALK2\ZNF593\RBBP5\KIAA0556\PPP1R8\EYA3\LHFPL5\PIGR\C20H20ORF24\UBXN6\LOC428335\CAMK1G\ZZEF1\PPIL2\ATG16L1\EIF6\GNB1L\MED16\C15H22ORF25\NCOA4\CHMP4B\UNC45B\NOC2L\KLHL6\EP400\HIBCH\C10ORF58\PIK3CD\LZIC\C19H17ORF85\ANXA11\SCUBE3\TCP11\DNAJC9\ACAA2\RFNG\RCN2\SUGP1\PRDX6\AMFR\KIF20A\SUB1\EIF2B1\CRLF3\ELL\ESRP2\C18H17ORF75\TAF8\SLC12A4\LPCAT2\ANO5\GPN3\UBR4\ASB18\ITM2A\BET1L\TAF9\TRMU\DHRS3\ORC6\TNFRSF8\PPP1CC\RPS14\RGL1\GLE1\NOL8\ALDH3A2\CCNDBP1\PRPS1\DUT\FUT11\RPS6KB1\SEC22C\CDH11\SASS6\FRRS1\PDLIM1\MPHOSPH6\NOLC1\KLHL18\LYVE1\CTDSPL\SBF2\YTHDF1\RRAS2\TPRA1\GINS1\KDM8\IL2RA\DDT\DUSP15\SEMA3A\ZMYND11\EHHADH\ITIH2\RNF185\BLNK\PPP1R2\PXK\PCSK6\FTH1\SUDS3\ABCC2\CD3E\RAB18\ZFYVE27\NPEPL1\RAB22A\KDM3B\EGR1\COPS7B\GGA3\COQ10B\KIF23\MOB4\RTN4\RELN\ALDH7A1\ARFGAP2\EDEM1\CKAP5\MPP4\SUMO1\CRY2\BMPR2\SLC38A2\SMNDC1\ACSS1\STAM\TRDMT1\TRABD\APLF\SHOC2\NDUFA5\SAMD13\PPP1R21\LBH\LCLAT1\VMA21\TBC1D24\CRAMP1L\LOC424998\FNIP2\CCDC132\PLEKHH1\IRAK4\C5H15ORF29\PALLD\METTL5\ATPBD4\TRAF5\NUBPL\CRIPT\TMEM184C\C4H8ORF40\SEC23A\TRAPPC6B\VLDLR\MMACHC\PPP1R12A\MBNL1\CMPK1\LOC100859230\SLC30A6\SCCPDH\PPPDE1\CCDC88C\TMEM48\ANKMY2\MTR\EPGN\FAM126A\SGIP1\VRK1\TAX1BP1\PPP2R5C\DCN\CRADD\NEDD1\XRCC5\PTPLB\TMEM108\UBA6\DDX47\CLASP2\DTX3L\CFI\JOSD1\NIN\NUP37\IRF4\SERPINB10\ADIPOR2\KIAA0528\ANP32E\ANKRA2\TFB1M\HBS1L\FAM76A\UPK3A\FBLN1\PDS5A\MAN1A2\CSTB\FAM162A\TPI1\PTPN6\MEGF10\WDR3\THBS4\LPIN2\CLUL1\GFM2\FAM26E\CYTL1\FIG4\FOCAD\MLLT3\QTRTD1\DAZAP1\PCMTD1\ST3GAL6\IDUA\CHRNB3\PROS1\HACE1\HTRA3\TCEB1\HSPA13\ADD1\MXD4\WHSC2\TMEM30A\TP53INP1\DENND3\U2AF1\OC90\DMD\GSTA\KLHL15\LPIN1\REPS2\UBE

		3A\STK24\TGDS\IRG1\LMO7\ELF1\KIAA0226L\N4BP2L2\SLC46A3\CRYL1\MPHOSPH8\MTMR2\VAPA\RNASEL\GZMA\KIAA1609\TPD52L2\C1H21ORF33\RPRD1A\NR1D2\HIGD1A\C3H2ORF43\MCPH1\CD8B\KLHDC2\INCENP\AK6\CENPH\ATE1\TGFBR3\SPECC1L\RHOA\LOC396531\TCF12\ALDH1A2\GLT1D1\HSF5\GRIA4\CGA\OVST\TAPT1\PAPOLA\PCMT1\NUB1\MYO5A\MYO19\TBC1D22A\TBCD\EIF5A2\WDR43\PTPN22\RABEP1\WRAP73\CD320\
P<0.05	mmu-miR-6412_R-2_1ss15AT	ANP32B\WDR59\ERGIC1\SFRP1\TARSL2\CAMKK2\BCL6\CBLN4\CASP3\ZRANB2\CLEC3B\ZBTB2\SDHA\MSL1\EVC\TIPRL\UBXN2B\COTL1\RABEP1\
P<0.05	mmu-mir-5098-p5	FAM195A\ARMC1\GKN2\CEPT1\RPL9\YTHDC2\SLC25A46\NEFM\GALK2\LOC426699\RANBP3\CAMTA1\HNRNPR\LMX1B\EI24\SRPR\FAM192A\DBR1\CHD6\EDC3\POC1A\LOC416777\SEPT9\CPSF3L\NACC2\WWC1\LRWD1\NTNG1\DYNLRB1\ARVCF\SAP130\NR5A2\AGO3\DTWD2\OGDHL\CSNK1G1\RAN\U2SURP\LZIC\BMS1\SLC16A3\MED23\ERGIC1\BOD1\TARDBP\BRI3BP\CPPED1\SUGP2\REXO4\SLC16A9\STAR\SUB1\EIF2B1\VAMP4\COMP\WHSC1L1\ELL\HERC1\CLINT1\USE1\NARG2\MINA\LOC101749801\UHRF1\BET1L\KDM2B\FAM20B\DHRS3\RCAN3\MAP2K6\TNFRSF1B\NCOR1\SGPL1\LOC100858951\SET\DRG2\DUT\RHBDD1\FAM120A\TK2\CDH11\SLC25A36\CYB5R2\RBP2\GUK1\PTH\TADA2A\PIK3CB\RHOG\ARHGEF3\TM9SF3\TCOF1\KLHL18\ABCD3\C20H20ORF11\GCLM\JPH3\DCAF12\PAFAH1B1\DR1\MTF2\PLS3\LRRC8B\XYLB\IL16\CRKL\SEMA3A\GRM3\ABCC1\BRSK2\UPF2\POU2AF1\KLHL4\LIMK2\TLR4\ALDH1A3\OPA1\FGF12\CLCN5\RNF166\MAP3K8\TAOK3\TH1L\YME1L1\CCND1\EGR1\MAGT1\BCL11A\FANCL\ATP7A\TTC17\PIAS1\SUMO2\ANKRD44\CFLAR\TBX5\NEURL\GHRL\TRH\LAMP2\NKRF\DSTN\NELF\CSRP2BP\SAMD13\SSFA2\PPP1R21\ITGA4\PIGK\JAG1\ING3\YPEL5\NRXN1\DLX5\GFRA1\CRDS2\SFRP2\OLA1\PRDX3\CTSO\SLC39A9\ETFDH\THAP5\DCAF17\GORASP2\GPATCH2\C5H15ORF29\ACADSB\SSB\BUB3\C5H15ORF41\TMEM206\NKTR\PRPH2\SMAD1\TMEM184C\CNOT2\FAM177A1\KIF6\MDGA1\MMACHC\SNCA\ELAVL4\YIPF4\BTF3L4\GTF2A1\ACSL1\AGA\SMEK1\ITPK1\G3BP2\IGF2BP3\CBX3\ARHGAP24\CHN2\COQ2\KITLG\AGPAT9\ZRANB2\LTA4H\AMPH\GRSF1\MYLK\WDR91\ITGB5\TOB2\RTN1\ST13\CAMK2D\FSHB\FKBP9\ADH1C\PPP3CA\VPS41\RBFOX2\FBXO7\DEK\PHACTR1\ELOVL2\GNPTAB\BLOC1S5\SERPINB6\ZBTB2\RMND1\ATP6V1E1\FH\FBXO5\CNOT7\NPEPPS\CDH7\GEMIN6\MED6\C2H18ORF19\VPS45\KIT\C1H12ORF11\FRYL\UPK3A\ANKRD52\MAN1A2\MYH10\MBLAC2\ARSK\SGTB\RABL3\LAMA4\NSG1\RPF2\MLLT3\B4GALT6\F5\OSTM1\ALCAM\TMEM175\ATG5\SDR16C5\SLC44A1\CHMP2B\EPHA7\MAP3K7\SAMSN1\SLBP\CTBP1\CHODL\PNRC1\SYNCRIP\GRIK1\CA2\IMPG1\SMOX\CHAF1B\WRB\KHDRBS3\AGPAT3\DDX3X\PHF20L1\PDK3\KLHL15\SH3KBP1\NBAS\UBXN2A\WWC3\LYG2\RPL31\TNFSF13B\ARGLU1\IRG1\DIS3\BORA\GPALPP1\UBL3\N6AMT2\MED17\ANKRD42\PPME1\SPDYA\VAPA\SUV39H2\YWHAZ\TPD52L2\POU2F1\LSAMP\PHF21A\EMP1\UBE2N\GATA2\COLEC12\RPRD1A\WDR48\C3H2ORF43\FZD7\UBE2CBP\SLC20A2\PPP6R3\MCCC1\RNF220\RNF220\TGFBR3\NDUFA10\RSFR\MARCKSL1\NADK\NUFIP1\SMS\POU1F1\MYH15\TRIB2\HPCAL1\POPDC3\DICER1\PDLIM5\CDCA7L\GYPC\SLC1A2\ZRANB1\DHX38\GATM\GABPB1

		\NELL2\EMB\SLC24A5\ATP2A2\DDX19B\COL6A1\HNRNPH1\ZIC1\EPS8L2\ANG\
P<0.05	mmu-miR-500-3p_R-1	STARD4\KCTD9\SPPL2B\SRSF3\FAM72A\SAMHD1\PSMD5\SEPT9\LRIG2\ODZ2\UBE2J2\TNFRSF18\PMPCA\PLEKHB2\RFFL\ADPRHL2\GBAS\AQP3\ZMYM4\INPP5K\TARDBP\NARS\C26H6ORF130\NFYA\HERC1\G3BP1\ADA\GPX3\SGPL1\SET\RPS24\MTFR1\FUT11\RPS6KB1\TK2\TADA2A\NONO\LOC768701\ADRBK2\IDI1\ACADS\RMI2\MED19\MAP3K8\CD3E\NPEPL1\SUCLG2\CD44\UBE2H\SLC26A4\UBE2V1\NDUFS3\ACTR1A\SUMF1\CCNYL1\SHOC2\JAG1\KIAA0922\BAG3\IFRD1\DYNC1I2\PLEK2\PDK4\ANGEL2\FASTKD1\ZNF330\E2F7\GPR149\UFSP2\IGF2BP3\KCNK1\COG2\VRK1\CHN2\C3H6ORF120\OXNAD1\CRADD\ZRANB2\PECR\C5H14ORF135\ARPP21\BDH2\ATL1\IYD\CYTH4\LOC420807\FARS2\MKRN1\ADCY2\SRSF7\VPS45\SGCB\KRAS\M6PR\MBLAC2\ZDHHC23\ZFAND5\DAZAP1\SEC63\TMEM68\JAKMIP1\AFAP1\TMEM129\IMPG1\RAD21\HADH\U2AF1\FBXO9\PXDN\UBXN2A\LIG4\ABCC4\ZC3H13\KIAA0226L\SUCLA2\PAN3\POLD3\GDPD5\VAPA\C6ORF192\PDGFRL\SLC20A2\CHP1\INCENP\TM4SF1\MCCC1\NEO1\SMS\AFAP1\PDLIM5\TMED8\SLC10A7\BIRC5\TBC1D22A\DDX19B\CSDE1\WRAP73\
P<0.05	mmu-miR-3964_L+1_1ss6GA	RFK\NEK6\DBR1\PBRM1\ATP6V1B2\NCKAP1\SLC16A9\ACSS2\ESRP2\RHOT1\SUN1\GEMIN5\RCAN3\H2AFY2\ISY1\SLC25A36\PIK3CB\RHOG\RNF141\YTHDF1\GORASP1\PIK3C2A\PAXIP1\PDPK1\LOC422249\FOPNL\PDE6C\YWHAH\RBM7\APIP\SPAG6\STK17B\NEBL\PIAS1\ANKRD44\CELF1\CTDSPL2\DDB2\VBP1\SHOC2\ATP6AP1\ATP6V0C\PRKCI\GATAD1\SEC23IP\C2H7ORF64\DYNC1I1\ANGEL2\NENF\RASSF3\RAP1B\GEMIN2\FBXO8\IGF2BP3\CBX3\HIBADH\VRK1\PPP2R5C\KAT2B\CRYZ\PTPLB\UBN2\TRPC3\ST13\LRRFIP2\TDRD7\SOUL\ISCA1\LOC100857286\JARID2\TXNDC5\CLPTM1L\ANKS6\KIT\KRAS\KIAA0930\FBXL5\MBLAC2\LYSMD3\ZUFSP\CRHBP\C1H3ORF26\SNX3\ATP5I\HACE1\MAEL\RNF4\MORC3\DCAF13\NOV\PTP4A1\PIGA\RASA3\TNFSF13B\LIG4\ARGLU1\RNASEH2B\TPTE2\UFM1\ZAR1L\CERK\EAF2\SPECC1L\CRABP1\NADK\RREB1\SLC1A2\ATP2A2\NUCB2\SPTAN1\
P<0.05	hsa-miR-4443_R+1	ARMC1\TIMM17A\LOH11CR2A\STARD4\KCTD9\SPPL2B\DDX42\MYL4\NFASC\RBBP5\HBEGF\TAF12\MTMR4\C4BPA\EDC3\ADRA1B\EIF6\ST6GALNAC1\SAP130\FAM168B\RFFL\DTWD2\SMN\SEC11A\CHCHD2\TMEM170A\SRM\ARL2\GSS\AGPAT6\GRHL3\GOSR1\LOC100859686\C8H1ORF27\WDFY1\RHOG\KLHL18\SNX12\STK25\DCAF12\SPTY2D1\VIPR2\IL13\TNMD\LOC100858827\ALDH1A3\PEBP1\NPEPL1\CD82\CHRDL1\TMEM164\ATG4A\XRN2\MCTS1\TUBGCP4\MAL\DSTN\TRABD\SPTBN5\NCSTN\BAG3\SLC25A12\SHFM1\C1GALT1\TRAF5\IL22\ZFC3H1\VSX2\CYP4B1\QPCT\SCIN\DHCR24\UBR7\COBLL1\ITGB6\NDUFA12\MRAS\ESR2\XPNPEP3\DACT1\ANK2\CCDC93\AVL9\RAB3GAP1\CUL1\TOM1\C22ORF28\MKRN1\TERT\LOH12CR1\ANP32E\LOC426218\CNDP2\PCYOX1\VPS45\IGF2BP2\LIMCH1\MBLAC2\MLLT3\SH3BP2\TMEM129\TP53INP1\SLC25A32\KLHL15\SF3B14\PNOC\BLK\N4BP2L2\PPME1\MRPL48\B4GALT1\DPT\ARHGDIB\E2F5\HIGD1A\HABP2\PAFAH2\ZFAND1\CENPQ\HNF1A\CUL2\XIRP1\EMB\CD151\GTF3C5\PYGB\MYL1\TPCN3\LIN7B\REL\LOC419851\NFASC\NFASC\MYBPH\

P<0.05	hsa-miR-4286_R+1	WDR36\DDX42\CDC27\WWP2\C23H1ORF38\EYA3\SRPR\SH3GL1\CREB3L3\ATP5G1\WDR45L\SEPT9\SLC22A7\PRPSAP1\KIAA1045\LOC419144\PTBP1\POLR2D\AP3B2\PLEKHB2\UNC45B\AGO3\ADPRHL2\PUS1\ENO1\MMP17\PIK3CD\RXRA\VAV2\LRRC45\ENPP1\BOD1\ETFA\HMGCS2\COPE\SUB1\EIF2B1\ERLIN1\ARL10\TFEB\ARRDC2\ATAD1\GLUL\NECAP2\LOC101749801\GPR107\USP20\TRMU\TTPAL\GOSR1\COL18A1\ASB6\SPECC1\RPS14\FBXL18\C5H15ORF57\ALDH2\CDK9\VIPR1\HAGHL\HAGH\HS3ST6\JMJD4\SYNGR3\C20H20ORF11\PAFAH1B1\TXNRD3\DDT\PDPK1\ABHD17C\SRPRB\GGT1\CTSD\RPRD1B\KLHL4\MARCH5\CHM\PDHB\NRP1\BRWD3\PRKAB1\YIPF5\ARCN1\MYO1B\GGA3\SLC39A13\FBXO18\NT5C2\C15H12ORF49\SLC26A5\ZFYVE19\SORCS1\LAMP2\TRABD\ZFP106\AMDHD2\SKIL\CTSO\SLC39A9\ATP6V1D\ZNF650\HCLS1\RAP1B\STRN3\RAB3IP\ELOVL1\CRIPT\GTF2A1\DNAJC6\RFTN1\CRADD\AAMP\PTPLB\MTHFD1\ESR2\ATF7IP\ACTR3\NCKAP5\ZRANB3\UBXN4\TGFBR1\PWP1\ALDH5A1\MKRN1\C6ORF97\ADIPOR2\BPGM\GALNT1\SLCO1B1\NUP50\ING4\CLSTN3\ZUFSP\GTF3C6\FOCAD\RORB\TMEM175\TPD52\PLEKHF2\UBOX5\FBXO32\KRTCAP3\ELP3\SLC9A7\STK24\DNAJC3\MYCBP2\SLC46A3\N6AMT2\PPME1\CD1C\C1H21ORF33\POU2F1\TERF1\C3H2ORF43\FZD7\SLC20A2\AK6\RNF7\UNG\LOC419500\C26H6ORF89\FLNB\CRYAA\USP37\ZBTB34\ISL1\IRF7\DDX27\DDX19B\SLCO4A1\NPRL3\ACTG1\AP4B1\PNPLA7\LIMD2\SASH3\PDZD11\MAP3K14\THY1\TNFRSF10B\NGEF\
P<0.05	hsa-miR-425-5p_R+2_1ss20TC	GFPT1\CAMK4\PSMF1\RPS6KA1\SLC16A4\HBXIP\MYL4\HARS\FAM125B\SYF2\SNAP29\RRAGC\WDR45L\NFS1\LOC417345\DGKD\UPK3BL\DYNLRB1\POLR2D\SORD\RECQL5\RGS2\PIK3CD\SRM\SCPEP1\EIF2B1\GBGT1\KIAA1191\OSMR\PBLD\CCNB2\SLTM\KDM2B\FAM20B\BLMH\SLC47A2\URM1\WDFY1\GTPBP5\SEC22C\TPST2\MYD88\CEP19\NOM1\NAP1L4\ABCC1\UPF2\CTSA\ACADS\CREM\PGS1\RPS25\UPRT\C17ORF28\TMEM209\CHRDL1\RINT1\RELN\NEURL\CUL4B\LOC417741\ITGA8\PIGK\SNAP23\ATP6V0C\C6H10ORF46\KRIT1\SEC23IP\PDCD10\CFL2\MMACHC\CMPK1\LEF1\GTF2A1\TTC7B\SLC24A4\BZW2\AHR\RFTN1\PPP2R5C\SRSF11\ANKRD13C\ELK3\PECR\SLC25A3\BAG5\AMPH\PPP1R13B\IL8L1\PTPLB\HIF1A\FKBP9\LOC420849\KIAA1549\C1H12ORF35\ATP6V1E1\BID\GALNT1\PDK1\NPEPPS\SNX9\NETO1\LTV1\GPR98\ZBTB49\KIAA1524\GPR89B\TCEB1\HSPA13\SLBP\SYNCRIP\IMPG1\PTDSS1\ZNF451\PDK3\KLHL15\ENPP2\EFR3B\MTMR9\DOCK9\TGDS\SPRY2\NAA16\GPALPP1\SLC25A15\N4BP2L2\UBL3\SLC46A3\N6AMT2\PCF11\VAPA\SUV39H2\SLC25A14\PTGR2\AK6\DAB1\GCG\BZW1\ASIP\GLT1D1\GRIA4\SAT1\AKT1\NT5C3A\PCMT1\RPL35\PCSK7\HSBP1L1\
P<0.05	hsa-miR-3120-3p_R+1	ARMC1\TIMM17A\ADAMTS19\C22H20ORF30\STARD4\DPAGT1\CHMP7\CDC27\RBBP5\PM20D1\TBC1D22B\SLC7A6\NR0B2\NUDC\TERF2IP\FAM125B\MPO\SRPR\NEK6\ARMC8\RHD\RAB14\CCNL2\PPIL2\PPM1J\E2F4\TNFRSF18\DYNLRB1\TRIM33\SMU1\HSD17B4\SNX22\FBXW11\DTWD2\HEMK1\EP400\TRAPPC3\ADPRHL2\AP3S1\PUS1\SEC16A\GBA2\C10ORF58\CCT6A\DNAJC18\PIK3CD\POLR2E\LZIC\KCTD7\XRN1\SLC16A3\INPP5K\ACAA2\NTAN1\RASA2\PHACTR4\LRRC45\NKX2-5\ACADL\BOD1\MOXD1\TRIP12\SLC2A6\ARID5B\MMD\PARN\DDX49\REXO4\SLC16A9\CRLF3\GBGT1\DUS2L\GOLGA7\TAF8\RBBP4\SGK2\PPP1R16B\ATAD1\SALL1\ASAH2\SMARCE1\AP1M1\T

		ARSL2\CHRNA7\DNAJA2\PNRC2\SRSF10\ADA\PSMG3\FAM20B\TTPAL\RCAN3\ORC6\VPS35\CCPG1\SAR1A\PLOD1\SGPL1\YIPF6\RPS14\RGS9BP\AKAP10\PDAP1\SET\C14H17ORF103\NOL8\FLCN\COPS3\VPS53\LSM14A\DUT\MKRN2\ALKBH2\TAF7\AP3M1\DEAF1\CLTC\COL11A1\FGD3\EPHA4\VCAM1\CDH5\CDH11\RRP1B\IBA57\HAGHL\BCMO1\MPHOSPH6\TSC2\TNFAIP1\YTHDF1\ARFGAP1\DCAF12\TMEM41B\PAFAH1B1\TMED5\PLS3\GLMN\BTBD1\PSMA1\GORASP1\MCM2\MST4\HPRT1\MAPK8\FAM122A\KLHL3\TPX2\DDX46\CARS\UBE2I\SRPRB\LOC422249\TNNT3\KIF3B\P4HA2\ARPC4\TOLLIP\YWHAH\ARL6IP1\KIF3A\PISD\CTSA\MARCH5\LIMK2\RNF185\ACAP2\CABP1\TK1\P4HB\FGF1\SPAG9\SUDS3\TMLHE\SLC24A1\ITGB2\RAB22A\MTMR8\RAB11A\LOC416354\CHIC1\LRRC59\TRAP1\SNAPC5\NCL\C17ORF28\APIP\TTC12\STK17B\SF3A1\GGA3\TSEN54\CD82\ANP32A\TMEM164\ACTR1A\CELF1\RINT1\MOB4\ANKRD16\EIF3J\ARFGAP2\FGL2\TWSG1\AP2M1\SUMO1\CRY2\TRH\ADAM23\SMC3\NDUFS1\C3H20ORF3\TRABD\C1D\WASL\SAMD13\COMMD5\CERKL\FAM3C\SESTD1\EBNA1BP2\YPEL5\LBH\VMA21\NCSTN\ATP6V0C\DCAF4\CYP51A1\KRIT1\CAV2\SLC39A9\SERPINI1\TMEM229B\PLEKHH1\SI\NMD3\YAF2\MSMO1\GAD1\RRP15\ACADSB\SHFM1\C5H15ORF41\RAP1B\SLC35E3\MYST2\SMAD1\EAPP\CFL2\ZFC3H1\ZDHHC17\TTLL5\FAM98A\LOC425049\GTF2A1\CASP3\SCCPDH\PPPDE1\C5H14ORF159\SCIN\ACOT11\TTC4\ITPK1\SPP1\TRA2A\PGM1\C5H14ORF129\ABCB10\CAPN9\PHF10\SERPINH1\AGPAT9\CRADD\LRRC40\ZRANB2\TMEM66\TNKS\AAMP\NEDD1\BRP44L\T\BAG5\ATP2C1\NR1H4\IGF2R\IL8L1\CENPC1\MYLK\CCNA2\KIF15\RPL7L1\METTL14\CAMK2D\SNX4\C5H14ORF101\PDGFB\METAP1\TARP\UBE2D3\MAP4K5\STK17A\NOL12\DDHD1\SOUL\MMADHC\WDSUB1\HNRNPK\TMEM263\JARID2\TDG\EDN1\IGF1\TBXAS1\MKRN1\SERPINB6\VPS4B\MCM4\CCDC77\MARCH6\BPGM\LDHB\PLEKHO1\ANP32E\MTUS1\CNDP2\NPEPPS\CYB5A\LONRF1\NETO1\OPTN\KIT\FAM38B\HBS1L\SGCB\DCUN1D4\CWH43\NOL6\PARVB\ANKRD52\MRPL51\PHYH\LPCAT3\CADM1\DEPDC1B\SREK1\FABP7\SERINC1\GJA1\METTL16\MOCS2\ABHD3\F2RL1\NSG1\AMD1\FIG4\PLIN2\C9ORF93\XCL1\TBC1D23\MRPL15\NANS\CLTA\ALCAM\TMEM175\IDUA\NRG1\CHMP2B\AFAP1\CPA6\SH3BP2\MXD4\SLBP\GRIK1\SCAF4\IRAK1BP1\MMP16\FABP1\B3GAT2\PLEKHF2\PTDSS1\MORC3\UBR5\DENND3\U2AF1\SIK1\MAOA\DDX3X\USP9X\PTP4A1\PRIM2\GSTA\CALB1\PDHA1\KLF11\LPIN1\FAM49A\MTMR9\MUT\RP2\TUBGCP5\INPP4A\LYG2\C1H2ORF49\UXS1\SPRY2\MYCBP2\LMO7\KIAA0226L\ZAR1L\HSPH1\SLC46A3\MTMR6\SAP18\C1H11ORF70\MED17\PPME1\MRPL48\RRM1\KIAA1609\ITM2B\SEPT6\GSK3B\UBE2N\TERF1\C3H2ORF43\CLN8\EPM2A\SLC25A14\CAPN3\INCENP\CZH9ORF80\MCM6\PLEKHA3\CHMP1A\NDUFA10\RNF7\PRKAR1A\KPNA1\CCDC39\IRAK2\PSPC1\SOD1\TRIB2\HPCAL1\MTO1\AFAP1\CRMP1\POPDC3\TAPT1\PCMT1\RAD17\PDLIM5\TMED8\TGFB3\ACYP1\RARB\CCDC101\REEP5\IRF2\WIPF1\PGRMC1\YBX1\TBCD\BFSP1\EXOSC2\KIAA1328\HEATR3\FLOT2\PSME3\PYGB\C5H11ORF46\NAV2\PSMD13\POLE3\GNB1\DDX6\RCC2\UBE4A\CD320\
P<0.05	hsa-miR-21-5p_R+2_1ss1TN	KCTD9\HMBS\DCAF7\FAM125B\WBSCR17\SYF2\EDC3\INPP5B\ARVCF\C15H22ORF25\RFFL\C10ORF58\INPP5K\RFNG\LRRC45\SCPEP1\KIF20A\KIAA1191\ESRP2\LIFR\CALR3\PPM1M\NADSYN1\RNF111\CCNE1\MPRIP\PEMT\USP32\KLHL18\DCAF12\GLMN\CXCL14\ALD

		H1A3\SPG21\LOC422305\BTG4\BCL11A\PTPN1\TYW5\DDB2\STAG2\MCTS1\CLNS1A\PDCD4\DTD1\UGP2\FXR1\RBM25\KRIT1\DYNC1I2\C5H15ORF24\ACADSB\LOC426599\C1GALT1\PROX1\TMEM206\BRE\MBIP\MBNL1\GLCCI1\DHCR24\GLRX5\LRRC40\LHX8\TNKS\GRSF1\CLEC3B\ITGA9\TARP\RAB32\UBE2D3\MMADHC\RBFA\PIK3C2G\CYP4V2\EXOC1\MBLAC2\PCMTD1\GPR89B\UFL1\ZFYVE28\TP53INP1\RAD21\GCM1\MSGN1\PIGA\COL4A1\LIG4\SPRY2\LECT1\SLC46A3\ARL6IP5\PSMG1\GTPBP1\INCENP\POU1F1\TAPT1\SLC1A2\NELL2\XIRP1\EMB\EIF5A2\PELI1\KIAA1328\YOD1\STK40\MBL2\
P<0.05	hsa-miR-181b-3p_L+1R-1	SPR\PRNP\RDM1\GALK2\RAB11B\TMEM57\CIAPIN1\SLC25A1\SNAP29\MAN2C1\PSMD5\TNFRSF18\PMPCA\FBXW11\TMEM194B\GBAS\GTF2H2\SMN\MATR3\PAIP2\KCTD7\ZNF76\PDXDC1\FECH\C17H9ORF96\SCPEP1\KIF20A\GBGT1\GOLGA7\TSC1\SKP2\YARS\DHTKD1\GLUL\HEATR2\POLB\AP1M1\TAF9\FAM20B\EIF3B\VPS35\DYX1C1\NPPC\C21H1ORF187\RGS9BP\MAPK6\CPSF4\CLIC2\PSAP\LSM14A\DUT\TAF4\ACSL3\INTS2\RRP1B\PTH\JMJD4\SYNGR3\SNX12\C20H20ORF11\LOC422214\IRF8\WHDC1\C5H11ORF58\GALNS\GSTT1\UNKL\FANCI\ARHGEF12\LOC419755\KIF3A\APOOL\METTL9\BUD13\PGS1\ERGIC2\ATP6V1G1\SPG21\CLPX\RAB35\FGF1\PRKAB1\STX16\RAB22A\KIAA0226\SNAPC5\EIF4E3\KAZALD1\BCO2\AAGAB\STK17B\SLC26A4\TMEM180\HSPD1\CEP41\PUS7\NR1H3\CKAP5\TRH\GTF2H4\TYRO3\CD28\SAMD13\ADAM22\C4HXORF57\YPEL5\LIN9\C3H1ORF55\OLA1\SKIL\PRKCI\PDGFC\YAF2\C5H15ORF29\C1GALT1\RAB33B\LOC422442\LPGAT1\TRAF5\PRPH2\BRMS1L\TRAPPC6B\VSX2\GLIPR1L\GPR149\POLR1C\HERC3\ALKBH1\LEF1\DCTD\DHCR24\PPAP2B\SNX10\ELK3\BRP44L\XRCC5\AMPH\MTHFD1\ESR2\LZTFL1\SPATA5\TTC26\LUC7L2\HIF1A\CLEC3B\C22ORF32\PCDP1\METAP1\STK17A\TXNDC16\SOUL\MMADHC\MB\KCNG2\DEK\NUP153\WRNIP1\BPGM\VWC2\CCDC127\CLUAP1\UBA3\CTRC\SGCB\PLA2G2E\NUP50\APBB2\GAPDH\MYH10\GPR98\ZYX\SERINC1\FAM26E\SS18\TIPRL\CPA6\HAPLN1\SYNCRIP\ELOVL4\CPNE3\IMPG1\IFNAR1\IFNGR2\U2AF1\TG\POLA1\SNTB1\SH3KBP1\LOC421978\ASXL2\REPS2\PIGA\MUT\C1H2ORF49\KIAA0564\FLT3\MTMR2\NAALAD2\PPME1\GDPD5\LOC426128\ALG8\LOC101751832\UBE2N\PTGR2\CHP1\AK6\PLA2R1\ARHGAP19\CHMP1A\ALDH1A2\FAM48A\POU1F1\RALBP1\EGFR\AKT1\RIF1\RAC2\RAD54L\TMEM154\CUL2\SLC1A2\ARSB\RPL35\FASN\HNF4beta\TBC1D22A\LRRC31\ATP2A2\GTF3C5\COG5\DDX19B\MYOF\LIN7B\GRIN1\THY1\SNCB\ATCAY\
P<0.05	hsa-miR-143-5p	ARMC1\B-G\GFPT1\TIMM17A\TRIM27\C22H20ORF30\KCTD9\HMBS\PCNX\CHMP7\GALK2\WWP2\AP1G1\LOC100857226\WDTC1\WBSCR16\CREB3L3\PLXNA2\PRKCZ\CAMK1G\IRF1\GIGYF2\ANP32B\SLC22A7\SEC14L1\DGKD\MYL10\RASA4\TNFRSF18\GNB1L\NCOA4\ACOX1\EP400\HIBCH\OLFM1\TLN1\TAF11\DNAJC12\PACS2\TARDBP\ETFA\SPHKAP\MMD\VAMP4\COMP\UBIAD1\KIAA1191\TFEB\SFRP1\TAF8\NECAP2\LRRFIP1\P2RX4\PIGT\CAP1\FAM81A\VPS26A\DHRS3\SIGIRR\RCAN3\ABCA8\BRAP\ULK2\NAA25\LOC768735\CDC25A\URM1\SHC4\PLAU\GIT2\EPHA4\SLC25A36\MSRB1\AATF\TADA2A\RHOG\ASB14\ST6GAL1\ADIPOQ\COL9A3\C20H20ORF11\YTHDF1\BCAR3\ZFAND4\MTF2\MYD88\LSS\NSMCE1\SLCO2A1\VDAC1\TBCCD1\PDE6C\FANCI\TOLLIP\SRPX2\IL13\PFKP\LIMK2\RNF185\LPP\S

		PAG9\ABCC3\FYTTD1\PCK1\LOC416354\STAT1\C6H10ORF76\PRTFDC1\SNAPC5\APIP\BCO2\STK17B\MLLT10\HN1\RNF114\SLC9A8\SLC39A13\RINT1\FBXO18\GDI2\LOC424943\NR1H3\CFLAR\STRADB\ARRDC1\CRELD2\CUL4B\GIPC2\PRKCI\ETFDH\C2H7ORF64\RAB3GAP2\PLEKHH1\GXYLT1\THBS1\LXN\LOC422448\EDNRA\GEMIN2\AARS2\NFKBIE\FCF1\MBNL1\MBNL1\FAM98A\KIAA0494\ADCK1\ELAVL4\HEATR5B\ECHDC2\C5H14ORF102\THSD7A\NEIL3\MAP1LC3C\ANKMY2\RYR2\HSD17B11\DFNA5\KLHL8\PHF10\EVL\RFTN1\NGLY1\CRYZ\AAMP\STT3B\MAR-04\SH3BP5\MAP3K4\HSPBAP1\DDX47\ATF7IP\HIF1A\CCNA2\SLC35F4\FBXO34\PPP3CA\SOX10\MTHFD1L\MYLIP\BLOC1S5\PRPF4B\C1H12ORF35\VWC2\SLC6A19\LOC426037\PLEKHO1\C2H18ORF19\NUP50\MAN1A2\CSTB\CHD4\MYH10\LYSMD3\FAM172A\RBP5\ZYX\EPHB6\METTL4\SPICE1\WDR1\PLIN2\C9ORF93\MAMDC2\ABHD17B\BLZF1\TMEM68\GJA5\GPR89B\MMS22L\PREX2\WHSC2\MMP16\IMPG1\CDH17\TP53INP1\SFXN5\DENND3\U2AF1\DDX3X\OC90\GCLC\KIAA0196\SNTB1\IAH1\MELK\HADHB\C1H2ORF49\TUBGCP3\MYO16\DNAJC3\RBM26\IRG1\BORA\MZT1\WBP4\SLC25A15\PPME1\C11H16ORF87\ARL6IP5\VAPA\KIAA1609\CCDC41\CERK\MYL12A\WDR48\SUCLG1\CD8B\INCENP\AK6\CENPH\ATE1\MCCC1\PHPT1\CDC42\SLC13A2\TCEB3\KPNA1\C26H6ORF89\WDR35\TRIB2\HPCAL1\NT5C3A\PDLIM5\LPL\RPS19BP1\RPL35\NELL2\TRIM59\MYOD1\ATP8B1\NAV2\REL\CR1L\RER1\PDZD11\
P<0.05	ggo-miR-342_R+1	ARMC1\RASSF2\STARD4\ADAM28\ITGB3\LSM7\CDC27\ELF3\CYB5B\RBBP5\PM20D1\RASSF5\TAT\POM121\CCDC43\TMEM57\HES5\TMEM45B\SAMHD1\SRRM1\LOC100857709\FAM18B1\SSU72\RBM39\PIP4K2B\CWC25\PSMD5\ACSBG2\TNFRSF18\HEXA\ZDHHC8\UNC45B\RFFL\MAPKAPK3\RGS4\TRPC1\DNAJC12\C26H6ORF106\RAB24\GBGT1\UBIAD1\ELL\SFRP1\OPTC\TRRAP\MED27\TRIT1\SMARCE1\SYS1\TWF2\MICALL2\SEC16B\CYP3A7\ZER1\ULK2\ALDH3A2\LOC768735\MTFR1\SHC4\DHX30\CDK9\ADK\PRPSAP2\DPM2\PTGS2\TAF4\CKLF\JMJD4\TSPEAR\FLT4\GCLM\DNAI1\DR1\LOC420419\TMED5\MTF2\TRIM66\TPRA1\DDX46\SRPRB\PLXNB1\TBCCD1\KIF3B\RPRD1B\HINFP\SPPL3\HDX\ACADS\ITGB1\ABCC2\CD3E\C10H15ORF44\RAB22A\MMS19\MTMR8\RRP12\USP12P1\ECI1\ACTR1A\SLC39A13\ATG13\PDIA3\PEX6\LAMP2\CLNS1A\NDUFAF1\TYRO3\KIF16B\POLR3F\DCUN1D1\B3GNT2\ENTPD8L\FBXO11\NRK\SLC39A9\TMEM229B\CCDC132\ZNF650\GREM1\RASSF3\TMBIM4\BRE\HP1BP3\TBC1D15\PHF17\BTF3L4\GLRA3\AREGB\DFNA5\KCNK1\CBX3\WLS\RFTN1\KERA\GPD1L\ESR2\SNAPC1\TRIM27\ELMO1\TAB1\DNAL4\DARS\MPST\RBFA\C1H12ORF48\GCM2\TXNDC5\PIK3C2G\GABBR2\CLUAP1\LOC100859438\SYNE1\AGMAT\KDR\SGCB\BIK\OTUD3\EDARADD\TAPBPL\TPI1\HMGCR\RABL3\C3H6ORF186\MAMDC2\ZDHHC23\USP45\TMEM129\UBE2J1\TMEM30A\B3GAT2\TP53INP1\U2AF1\DDX3X\OC90\LOC396380\PDK3\SNTB1\IAH1\PHKA2\RP2\ABHD13\IPO5\DNAJC3\PPME1\LCORL\PSMG1\CD47\WDR48\C3H2ORF43\CLN8\NOA1\PPP6R3\SYK\HABP2\C2ORF47\RHOA\LOC415708\PRR11\CDC42\NDUFA8\KPNA1\PAFAH2\NCF1\TMEM131\POU1F1\PSMC6\RREB1\RPL15\TEF\NFIA\ACSF2\CUL2\WIPF1\ZNF335\CASP18\GTF3C5\HEATR3\AP4B1\REL\RBPMS2\COPA\TIMMDC1\
P<0	gga-miR-9-5p	RFK\LAMTOR3\TRIM39\WDR36\ZNF276\GALK2\ACOT7\PM20D1\FBXW2\CCDC43\LDLRAP1\SRPR\CAMK1G\NISCH\CALCOCO2\LOC41734

.05		5\ATP6V1B2\ACSBG2\MAP1LC3A\VAV3\CDC73\MAT1A\MYO1C\TAF11\TMEM170A\NRG4\KIF20A\MBTPS1\FN1\NELL1\TEX264\MANBAL\TIMD4\P2RX4\RNF111\TTPAL\AP3B1\DIABLO\AR\P2RX5\ULK2\CEP89\AP4E1\AIFM2\VGLL4\FBN1\SLC27A4\AP3M1\BCMO1\JMJD4\FREM1\TADA2A\SNX7\EVI2A\SLC9A3R2\TMEM97\SPPL2A\DCAF12\COL7A1\DR1\TPRA1\RTEL1\NOM1\SRPRB\C12H3ORF19\SKP1\TOLLIP\AP2A2\POU2AF1\CHID1\ITIH2\RNF185\TNC\SPG21\SVIL\FGF1\SUDS3\TUBB1\CTSZ\GOT1\FYTTD1\LOC416354\EIF4E3\MAGT1\VRK2\LAMB1\KCTD2\AHCYL2\RNF114\NPHP1\PCGF6\CNTN4\PHTF2\ARHGDIA\CNRIP1\SAMD13\NRK\ISM1\AMDHD2\BAG3\SLC39A9\GAD1\ASNS\RASSF3\SCFD1\ELOVL1\LOC417848\MDGA1\AARS2\INTU\NAP1L1\SPTLC2\SGMS2\CC2D1B\SLC24A4\LGMN\HOOK1\FAM126A\GALNT3\IGF2BP3\NTPCR\C5H14ORF129\LOC421560\LRRC40\FGFR1OP\STT3B\PPP1R13B\COX18\HEBP1\SPATA5\DNAJC24\PLA2G12A\LOC420748\MDGA2\SOX10\ADCY1\SOUL\ISCA1\ADAM12\C1H12ORF48\TRIM24\IRF4\CREB3L2\TDP2\PPAT\RTTN\MPPE1\MAP7\KIT\IGF2BP2\SGCB\ARHGAP8\PI4K2B\SEPSECS\MBLAC2\HORMAD1\EVC\BLZF1\TBC1D23\CD200\SDR16C5\CD247\ABLIM2\HSPA13\PMP2\LOC100857673\SLC17A5\U2AF1\SIK1\NDRG1\PXDN\ELP3\MCM3\TPP2\IRG1\KLF5\WBP4\LCP1\FRY\RNF17\MTMR2\NOX4\FZD4\VAPA\KLHL5\C3H2ORF43\CLN8\SUCLG1\SLC20A2\PPP6R3\SYK\ATE1\TANK\SOD1\GOLGA4\HOXA11\HOPX\CRIM1\LLPH\FASN\EMB\TBC1D22A\EIF5A2\LDHA\COL6A1\MYOF\AP4B1\BUB1B\SLC9A9\ARPC1A\BET1\
P<0.05	gga-miR-92-5p	HEP21\BMP10\PRNP\KCTD9\TNNI1\ZNF276\RAB11B\ZBTB48\CNTN2\PIK3R5\CASP9\COX10\SCAMP2\PTBP1\SYT15\NR2E3\ACOX1\EP400\VPRBP\KCTD7\C26H6ORF106\LOC100857356\EIF3I\PLEKHA2\ELL\ESRP2\SFRP1\FNDC5\LPCAT2\C21H1ORF144\KRT14\BET1L\LOC417192\ASB6\TNFRSF1B\IVD\EPB42\FAM101B\PTGS2\VIPR1\PITPNB\TMEM97\VILL\PLS3\COL6A2\MORN4\XYLB\TTLL9\PPP2CA\KIF3B\RLBP1\LIMK2\GCN1L1\CLPX\CDA\CTSZ\YME1L1\RAB11A\BCL11A\ANP32A\ZFYVE19\CTBS\LPHN2\SFRP2\CTSO\TMEM229B\CPXM2\LPGAT1\HELB\PPP2R5C\TECRL\ILF2\MRAS\CCR8\CDKN3\KDELR3\SLC16A8\C3H6ORF72\ADCY1\FBXO7\PWP1\SERPINB10\OVALY\MBP\AGMAT\S100A9\PRR5\RELL1\FIG4\TBC1D23\ALCAM\ADD1\MXD4\CD8A\KIAA1429\C1HXORF36\UBXN2A\IRG1\NAALAD2\CHRDL2\UCP3\GDPD5\CD1C\KIAA1609\COLEC12\TMEM196\DAB1\SKA2\LOC419404\C26H6ORF89\RB1\EGFR\REEP5\HNF1A\TGM2\SFXN3\TPM1\CACNA1B\
P<0.05	gga-miR-7460-3p	BNIP3L\HMBS\PGM2\KLHL21\RBBP5\FBXW2\NUDC\ADRA1B\TTC1\GUCA1A\RAB14\CYB5D2\MAN2C1\MRPS34\BBS4\UBE2J2\SDF4\HEXA\VCP\GPSM2\PLEKHB2\AGO3\C10ORF58\MCCC2\PLS1\C19H17ORF85\HSD17B7\PINK1\NFYA\WHSC1L1\MED20\TAF8\RBBP4\HPCA\FN1\DHTKD1\LIFR\TMEM38A\SRSF10\G3BP1\RNF111\FAM20B\DHRS3\CCDC69\MAP2K6\SPECC1\ASPA\USP22\DMXL2\LSM14A\DRG2\MPP1\TAF4\RRP1B\MPHOSPH6\ARFGAP1\GCLM\DGCR2\MYD88\TXNRD3\CARS\C9H21ORF2\RNH1\PFKP\RNF185\TLR4\PPP1R2\ACOX2\COQ5\C10H15ORF44\STAT1\EIF4E3\DNAJA3\CD44\ACTR1A\ACP2\ORC2\SUMF1\NET1\TYRO3\ACSS1\KIF16B\SHOC2\NDUFA5\LCLAT1\LIN9\AMDHD2\KRIT1\IRAK4\CHST15\FMN1\C1GALT1\TRERF1\MYST2\ELOVL1\VLDLR\PPP2CB\LOC100857182\CMPK1\LEF1\MEOX2

		\OMA1\CBX3\GLRX5\C5H14ORF129\LRRIQ1\CAPN9\CRADD\ZRANB2\ELK3\PECR\AMPH\RALB\HEBP1\CLEC3B\CYP2D6\MAD2L1\RPL7L1\QSER1\ANK2\FSHB\SOX10\CYTH4\PWP1\VPS4B\BCL2\DAP\MARCH6\SLCO1B1\CLPTM1L\PBX1\RSPH3\SLMO2\MAP7\KIT\SCNN1A\EDARADD\SLC34A2\SEPSECS\TAPBPL\GJA1\FAM26E\CRHBP\NFKBIZ\ALCAM\ACOT12\RNF4\RIPK2\FILIP1\ASRGL1\MORC3\DSCR3\RAB9A\MXRA5\C1H2ORF49\N4BP2L2\PAN3\CASP2\E2F5\TERF1\RPRD1A\LOC770433\KCNAB1\STARD5\CIB3\IRAK2\CNBP\KDM3A\STX7\PKD2\GARNL3\MON2\ARF1\NUP188\LOC419851\
P<0.05	gga-miR-6604-5p_1ss7GA	TMEM9\BNIP3L\PSMF1\KRTAP10-4\PSMC5\LOC426699\CDC27\RAB11B\ZCCHC17\CHTF8\RBBP5\MFSD4\WWP2\CACNA1S\SLC7A6\LOC100857226\LHFPL5\EI24\TPRG1L\CIAPIN1\SLC25A1\SIRT6\SNAP29\COX10\CNTRL\ACAD8\MYO1F\LOC416777\SEPT9\UBAC1\ORAI2\ST6GALNAC1\DYNLRB1\PTBP1\TMEM120A\ZDHHC8\AP2B1\RFFL\HIBCH\RGP1\SEC11A\CRYM\DEF6\ANXA11\C26H6ORF106\BNIP1\ACADL\TRPC4AP\MLX\FBXL8\TSR1\PEF1\FGFR1\FAF2\GBGT1\SCYL3\PLEKHA2\UBIAD1\ESRP2\RYR3\ZBTB8A\SGK2\SLC6A2\YARS\LPCAT2\DHTKD1\HEATR2\RAPGEF1\C21H1ORF144\NECAP2\TMEM38A\SDC4\LOC101749801\TWF2\DNMT3A\PPM1M\INTS1\TOMM34\GPR174\PABPC1L\BET1L\FAM20B\DHRS3\TSPAN15\TNFAIP8L1\RALGPS2\ORC6\CCDC69\HVCN1\AR\P2RX5\IVD\RGL1\LOC422179\C14H17ORF103\CEP89\HDAC8\URM1\CENPI\NARFL\ZMYM3\ABHD6\SPPL2A\TMEM41B\LOC420419\PXDNL\MGAT4B\AQP8\TPRA1\PLK1\EARS2\MORN4\IL21R\PDPK1\DNAJB6\MAPRE1\TOLLIP\OAF\LOC395381\CDK5RAP1\COQ7\PISD\RNF185\APOOL\TLR4\PPP1R2\PGS1\KIF5B\UTS2D\GCN1L1\PRKAB1\SUDS3\TMPRSS4\KMO\ACBD5\RAB22A\MTMR8\RRP12\RAB11A\SLC9A3R1\TRAP1\USP12P1\TMEM104\BCO2\AAGAB\COMMD9\KCTD2\GATSL3\HN1\TMEM209\ACTR1A\SFXN2\ADAMTS7\GDI2\CASC4\ALDH7A1\EHMT1\SLC38A2\MCTS1\ABHD12\DSTN\NELF\CUBN\SESTD1\PIF1\LBH\LCLAT1\ENTPD2\AMDHD2\SFRP2\C6H10ORF46\NVL\FAM45A\PRKCI\SEC62\ST7\PLEKHH1\SRP14\C5H15ORF29\AVEN\MUM1\HCLS1\C5H15ORF41\ANGEL2\XPOT\RASSF3\HAAO\TIE1\EDNRA\FAM177A1\HP1BP3\KANK1\CAPN1\ABCD4\NAP1L1\CMPK1\YIPF4\CCDC75\C4H4ORF41\TTC7B\URB2\WLS\RFTN1\ZRANB2\CRYZ\ELK3\TNFAIP2\SH3BP5\MRPL3\COX18\HEBP1\RRP7A\CLEC3B\SIDT2\TOB2\UBP1\SLC12A8\EEPD1\PAX6\ACTR3\NCKAP5\CBY1\CIRBP\METTL21D\MPST\MYH9\C22ORF28\ALDH5A1\JARID2\NCS1\RBM24\EEF1E1\BLOC1S5\MKRN1\SERPINB6\USP18\RHBG\LOH12CR1\MR1\FBXO5\NPEPPS\ENPP7\EXOC1\HBS1L\PPFIBP1\USP48\PARVB\NUP50\EDARADD\CSTB\C15H12orf51\CADM1\LYSMD3\RFESD\MAP3K1\KIF2A\RABL3\MSX1\ZDHHC23\MEP1B\TAF13\NANS\IDUA\PDZK1\CHMP2B\CPA6\UFL1\SH3BP2\SLC17A5\CCNE2\TP53INP1\ITSN1\DSCR3\FZD6\GNOT1\TOP1MT\LY6E\DDX3X\LOC396380\GSTA\LAPTM4A\PPM1G\HADHB\EGFL6\ELP3\INTS9\CYFIP1\REV1\F7\ABHD13\SLAIN1\SLAIN1\IRG1\WBP4\SETDB2\SPG20\N4BP2L1\SLC7A1\KIAA1609\TPD52L2\CECR1\ARHGDIB\FAM49B\LOC100857925\SUCLG1\C5H11orf24\MCCC1\C20ORF96\PRKAR1A\IRAK2\WDR82\POU1F1\MYOM1\CRTAP\SMC2\CDCA7L\MORC2\ACSF2\DRG1\SSTR2\SLC10A7\CYP24A1\DDX52\GTF3C5\SLC47A1\LOC422308\TPM1\PYGB\PNPLA7\RPUSD4\PFKL\CD320\LOC100859100\

P<0.05	gga-miR-6548-5p	CEPT1\C22H20ORF30\STARD4\DCAF7\CDC27\ZCCHC17\KLHL21\WWP2\AP1G1\IL10\SRPR\ARMC8\SAMHD1\APLP2\CLTCL1\RAB14\GLB1L2\MAK16\UBE2R2\PUSL1\E2F4\MYL10\IWS1\EIF6\PHYHIPL\ORAI2\UBE2J2\VAV3\FAM86A\C15H22ORF25\KCNMB1\FAM168B\NR5A2\RFFL\AP3S1\GTF2H2\CDC73\MAT1A\CHCHD2\PIK3CD\DNAJC9\C26H6ORF106\RASA2\ENPP1\MOXD1\TXNL1\CCL20\BBS2\AGFG1\NARS\DDX49\MLX\TMED2\EIF3I\GBGT1\KIAA1191\ESRP2\SFRP1\GOLGA7\SLC6A2\FEN1\DHX35\CHD9\NELL1\TMEM38A\GTF2A2\KRT222\MPHOSPH10\UBR4\HMGCL\STK4\YWHAB\BET1L\TRMU\RCAN3\ORC6\TNFRSF1B\AP5Z1\DCTN4\HVCN1\AKAP10\CLIC2\SCG3\COPS3\GPI\TSEN2\PRPSAP2\DEAF1\WDFY1\PPP3CB\TK2\SEC22C\IBA57\USP32\HAGHL\DYNLRB2\JMJD4\PDLIM1\COL9A3\ABCD3\GCLM\TMEM41B\RAP1GAP2\DGCR2\MYD88\SLC13A5\TPRA1\FAM122A\CHCHD4\TMEM43\DDX46\VDAC1\GRM3\MAPRE1\TOLLIP\PM5\TAF3\CSTF2\RPRD1B\AP2A2\IL13\POU2AF1\KIF3A\CHM\BDH1\RNF185\GCN1L1\SYNPR\TUBB1\GOT1\SLC24A1\DPYSL3\MTMR8\PCK1\KIAA2022\CBLN4\PFDN4\MLLT10\KCTD2\PGK1\SF3A1\TMEM164\SLC39A13\NT5C2\RELN\NR1H3\CFLAR\NAPEPLD\CASP8\ALS2\CRNKL1\NET1\NOP58\ARRDC1\SLC38A2\SENP5\LAMP2\EEF1B2\AHSG\MGA\TUBGCP6\PDCD4\TRABD\GRM8\PPP3R1\SEC23B\DCUN1D1\CROT\IDH1\CERKL\PIGK\LBH\LCLAT1\EHD3\IDS\GFRA1\KIAA1715\NVL\DPF3\SLC39A9\DUSP10\GXYLT1\SLC25A12\C5H15ORF24\ACADSB\CLCN3\C1GALT1\IFNG\G2E3\RAP1B\SPOP\MYST2\SMAD1\ELOVL1\CRIPT\PPP2R3C\HSP90AB1\MMACHC\HERC3\FAM98A\GYG1\OSTC\GTF2A1\DPY30\GPX7\SCCPDH\TTC7B\MIOS\TMEM59\ANKMY2\MTR\IL6\HNRNPA2B1\C5H14ORF129\SEC31A\KITLG\ANKRD28\RFTN1\PPP2R5C\KERA\DCN\USP44\ELK3\CKB\PPP1R13B\SCYL2\SOD2\ESR2\KIF15\TMC2\ST13\SLC12A8\TRANK1\CCDC93\SEPT7\FSHB\FKBP9\CCNT2\CIRBP\STK17A\GTDC1\PRPF39\HNRNPK\ISCA1\RBFA\LOC101750080\DEK\HSP90B1\MKRN1\MARCH6\GALNT1\STX17\ANP32E\ANKRA2\GMNN\MBP\CNOT7\DNAJC16\CYB5A\EXOC1\PTPN2\FAM76A\FGFR1OP2\OTUD3\SCNN1A\RELL1\EMG1\FNBP4\ARSK\RFESD\ELL2\CAST\DEPDC1B\MTX3\PAPD4\TNFRSF1A\GFM2\WDR1\F2RL1\CRHBP\NSG1\TMEM39A\ZFAND5\PCMTD1\PAM\TBC1D23\GNE\ALCAM\KIAA1524\ATG5\FNTA\PREP\CHMP2B\MXD4\WHSC2\CTBP1\IMMT\MMP16\C4H20ORF194\GNOT1\NOV\LY6E\MED14\PRIM2\ADCY8\TINAG\PDK3\KLHL15\FER1L6\FBXO25\C2H8ORF76\KRTCAP3\PPM1G\LOC421988\PIGA\FDFT1\MCM3\LIG4\KDELC1\SPRYD7\RNASEH2B\WDFY2\CKAP2\TPTE2\SAP18\MPHOSPH8\RNF17\MED17\TYR\PPME1\VDAC3\PSMG1\GSK3B\PACSIN2\TERF1\UPP1\NR1D2\WDR48\C3H2ORF43\C6ORF192\LOC395933\SUCLG1\KLHDC2\INCENP\RAD51\TM4SF1\DAB1\PLA2R1\UBE2E3\TGFBR3\C2ORF47\SUMO3\UNG\TCF12\PRR11\HMGCL\PUM1\C26H6ORF89\IRAK2\BIRC2\TMEM123\CENPQ\RALBP1\TTF2\TAPT1\EGFR\PAPOLA\ACYP1\RAC2\REEP5\XRCC6\NFIA\LUC7L3\OIP5\ATP2B1\CUL2\SLC10A7\ARSB\NELL2\NUB1\CYP24A1\KIAA1328\NBL1\PSME3\MYL1\LIN7B\DDX6\ADAR\ARL8A\NUDT1\PLEKHJ1\PLAC9\LEPROTL1\
P<0.05	gga-miR-551-3p_R+1	NEFM\SLC16A1\EIF2B1\FAM101B\KIF3B\APOD\PPP1R21\CDAN1\GALNTL6\APOO\N6AMT2\TRIM3\CCDC101\

P<0.05	gga-miR-490-3p_R+1	TIMM17A\BNIP3L\CSRP1\RBBP5\ZMAT2\NR0B2\SNAP29\PI4KA\JMJD6\SDF4\PMPCA\LOC415324\UNC45B\DTWD2\RASA2\TMEM170A\DDX49\MLX\TMED2\USE1\P2RX4\CSRP3\GALE\BET1L\KIF4A\HKDC1\EIF3B\GOSR1\TK2\EVI2A\DCAF12\DR1\E4F1\COPB1\TPX2\HTATSF1\SLC6A6\SKP1\CRTAM\POLR3E\REXO2\ITGB1\SYNPR\YIPF5\SNAPC5\APIP\FANCL\CEP41\FBXO18\NAPEPLD\ABHD12\B3GNT2\UBR1\C6H10ORF46\FMN1\PROX1\TRERF1\IL22\EDNRA\KIF6\HECTD3\KIAA0317\PPP1R12A\GYG1\NID1\TSNAX\C3H6ORF120\DAZL\ELK3\MYLK\PRKCH\CYP2D6\C5H14ORF135\METTL14\LARP7\GTDC1\MYH9\SLC37A3\BPGM\CTNNAL1\CNDP2\GPR126\MAP7\BIK\ATP10D\UPK3A\SEPSECS\MRPL51\MBLAC2\LMNB1\GSTK1\PAPD4\TRAF3IP2\TMEM68\GNE\USP45\MMS22L\VCPIP1\SYNCRIP\ESRP1\WRB\WDR4\ST3GAL1\DERL1\CALB1\GAL10\NAA16\SPRYD7\IFT88\NOX4\PLEKHB1\ALG8\PICK1\PPP6R3\AK6\DAB1\DCLRE1A\STX2\PAPOLA\PCMT1\SLC1A2\COL17A1\ARF1\TMEM120B\GTF3C5\EPHB3\EPS8L2\SLU7\
P<0.05	gga-miR-460a-5p	ARID4A\ARMC1\GFPT1\ARHGAP25\BNIP3L\HMGN2\HBXIP\SPPL2B\DCAF7\LOC426699\PPP1R8\LDLRAP1\C20H20ORF24\TIRAP\PIK3R5\SNAP29\COX10\UBL7\RBM39\INPP5B\ACSBG2\PTBP1\GPRIN2\CLCC1\CPEB1\NR5A2\SLC7A4\RFFL\DTWD2\EP400\PUS1\ENO1\RECQL5\RNF123\LOC423629\SFPQ\U2SURP\PLS1\DUSP19\RFNG\TMEM170A\BNIP1\BOD1\NEDD4L\SPHKAP\MKL2\TRPC4AP\KIF20A\ASH2L\DNAJA4\CDK2AP1\GFI1B\UCK2\DDX5\PPP1R16B\DHTKD1\KRT19\MINA\AP1M1\PNRC2\SRSF10\YWHAB\RNF111\TTPAL\ANGPTL1\PSMD9\ANAPC16\TNFRSF1B\LOC100858951\SHISA5\MAPK6\PLCB2\DMXL2\CEBPG\LSM14A\CENPI\MAP4K4\PLAU\CES1\GHRHR\SLC25A36\BTBD10\DHRS11\AATF\PNAT10\MPHOSPH6\ARHGEF3\ABCD3\C20H20ORF11\LOC422214\IRF8\TRAF7\GCLM\DR1\BANP\DGCR2\MTF2\MYD88\KLHL13\GORASP1\ARFRP1\ZNF326\DDX26B\GINS1\CXCL14\SKP1\RPP30\PDE6C\YWHAH\REXO2\OPA1\PTPRG\RAB3IL1\ARHGAP12\GCN1L1\MPZL2\DPP8\TH1L\LARS\MTMR8\ANO1\LOC416354\NCL\C20H20ORF43\MYO1B\CASS4\PUS10\STK17B\KCTD2\SLC9A8\CHRDL1\NDUFS3\NXT2\PLCL1\RTN4\ORC5\CASP8\STRADB\ALS2\SLC38A2\NKRF\CXORF56\LMOD2\IDH1\SAMD13\ATP6AP1\FXR1\TASP1\TMEM185A\TBC1D24\FAM45A\SKIL\PRDX3\CAV2\PEX1\PDCD10\TMEM229B\YAF2\THBS1\GPATCH2\ACADSB\SHFM1\AQR\WDR65\HAAO\RAP1B\EDNRA\LOC417848\FAM177A1\PPP2R3C\CCDC167\VLDLR\PRDX1\PPP1R12A\HERC3\LOC425049\EPS15\AHCTF1\GPX7\LRRC72\ANKMY2\GOLGA5\AREGB\RAPGEF5\KLHL7\GALNT3\DFNA5\TSNAX\GLRX5\ATG2B\C5H14ORF129\CHN2\EML1\SERPINH1\KAT2B\ELK3\ERI1\BAG5\MYLK\SERHL2\CCNA2\SLC38A6\CTNNB1\METTL14\RTN1\ST13\OSBPL11\DTX3L\CCDC93\KIF18A\METAP1\BDH2\EPC2\NAA20\MYLIP\TDG\SERPINB6\BCL2\C5ORF22\ATP6V1E1\LOC418170\BPGM\SDHA\PYROXD1\CLPTM1L\C3AR1\ABCC9\SOGA2\CLUAP1\ANP32E\TLR3\LONRF1\SLMO2\LOC421049\KDR\FGFR1OP2\NUP50\M6PR\UCHL1\MAN1A2\EDARADD\PI4K2B\CSTA\ACTN2\LYSMD3\ARSK\IL6ST\KIF2A\EPHB6\SGTB\PAPD4\METTL16\GREB1L\ESCO1\WDR1\NPC1\EVC\RPF2\C3H6ORF186\TBC1D23\PCNP\QRSL1\CD247\MAEL\PDZK1\CHMP2B\SOHO-1\CASP8AP2\TCEB1\WHSC2\TXN\SLBP\UBE2J1\LCA5\MMP16\RIPK2\TMEM30A\OTUD6B\PLEKHF2\DDRGK1\TTC3\FZD6\UTP23\U2AF1\DDX3X\FBXO9\KLHL15\FAM49A\UBXN2A\REPS2\C1H2ORF49\TFDP1\DNAJC3\SLAIN

		1\SLAIN1\LOC418811\WBP4\LCP1\POSTN\N6AMT2\MPHOSPH8\ALKBH8\NOX4\LYN\PSMG1\INVS\ABHD5\NR1D2\EPM2A\NFE2L2\RGS1\GLT1D1\NADK\C26H6ORF89\SUGT1\TMEM131\CENPQ\COL9A1\OVAL\SLIT2\LOC423536\ROS1\ARHGAP15\TMPO\LUC7L3\TMEM154\FAM169B\LOC416916\WIPF1\CYP24A1\UPF3B\GHRHR\ADAL\KIAA1328\EIF4A2\AP4B1\REL\YOD1\SLC9A9\HSF1\SLU7\TIMMDC1\BET1\
P<0.05	gga-miR-460a-3p	TCF25\FANCA\BTN1A1\SSR2\SCLY\PLLP\RAB14\PHF20\ORAI2\TNFRSF18\BCAS2\NR2E3\GJA4\SRM\WHSC1L1\KIAA1191\RHOT1\PIGS\G3BP1\NCOA3\DHRS11\C20H20ORF11\ITGB1BP3\ST5\DNAJB6\CLMP\TBCCD1\POFUT1\ARL6IP1\POU2AF1\MTMR8\BCL11A\XRN2\COBRA1\TRABD\LCLAT1\FAM45A\THBS1\CAPN1\LGMN\BAG5\TOB2\RANGAP1\CAMK2D\SOUL\WDSUB1\CCDC83\ANP32E\TMEM106C\VPS45\ACTBL2\GNOT1\MYOM2\FAM49A\DNAJC3\RCBTB1\GZMK\ALG8\PPP6R3\CENPH\LOC101751517\LOC101749395\EGFR\CRB2\CACNA1B\PRPF3\ARL8A\LOC100859100\
P<0.05	gga-miR-455-5p	RFK\CHMP7\RANBP3\RBBP5\SLC7A6OS\FBXW2\PANK4\WBSCR16\FHL3\WDR45L\PRKRIP1\COMT\CDC73\SLC16A3\PROSC\KIF20A\TMED2\GBGT1\ESRP2\ALDH9A1\NARG2\ANAPC7\RAB33A\TTPAL\KCNJ2\RSRC2\SGPL1\PLCB2\PTGS2\RPS6KB1\HHATL\CDX1\GORASP1\CRKL\ANKRD1\HSPA8\PGS1\TK1\HRASLS\TMLHE\USP12P1\UBE2V1\CELF1\NT5C2\ACP2\SEMA4B\EIF2B5\ARRDC1\SMNDC1\TYRO3\DNAJC10\NHLRC2\FSHR\CDC42BPA\UBR1\DPF3\SLC25A12\RPAP3\RASSF3\EGLN3\TMEM184C\AARS2\ADCK1\EIF2AK2\ASB5\LEPROT\C5H14ORF129\HIBADH\URB2\STT3B\MAP3K4\ATF7IP\SLC38A6\MYOZ2\CASP6\LRRFIP2\SOUL\ISCA1\RBFA\C1H12ORF48\ZBTB2\AGMAT\EDARADD\EPHA1\CLUL1\ABHD3\C3H6ORF186\TMEM175\KIAA0232\CCNH\U2AF1\DDX3X\CALB1\TAF1B\CDC16\ENOX1\RFX8\PACSIN2\E2F5\MICAL1\TATDN3\CHP1\C2ORF47\RNF7\ALDH1A2\PRR11\KPNA1\TMEM123\RB1\POU1F1\TAPT1\IRF2\LUC7L3\TMEM120B\ATP2A2\BFSP1\RCC2\
P<0.05	gga-miR-454-3p	RFK\ARMC1\RASSF2\TMEM9\LSM7\LAPTM5\CAMTA1\PM20D1\FAM72A\IL10\PANK4\RSPRY1\UBE2L3\PTPRU\MIB2\GLB1L2\ATG16L1\MAT2B\GNB1L\SLC25A24\CHMP4B\LOC415325\CAMSAP2\FBXW11\GJA4\KCTD7\NUP35\SLC15A4\DNAJC9\INPP5K\FRZB\MED23\SCAPER\NKX2-5\BOD1\MOXD1\MTOR\SBNO1\SFRP1\LPCAT2\MED27\MINA\COL6A3\PPT1\SLC25A16\RCAN3\DIABLO\SH3GLB2\SGPL1\HVCN1\MTHFR\P2RX5\KPNA7\CPSF4\RGL1\CLIC2\PSAP\PPIF\RPS24\MTFR1\VGLL4\PPARG\PRPSAP2\NUP210\FAM96B\CLTC\VIPR1\CDH5\RRP1B\ANXA7\SNX2\ADAMTS18\CA4\MSRB1\RHOG\DYNLL2\SGSM2\ARFGAP1\KLHL13\DCTN5\HPRT1\ZNF326\GTF2H1\IL2RA\CXCL14\SLC6A6\ATP12A\SKP1\DNAJB6\MRPL23\YWHAH\IL13\POU2AF1\PFKP\USP28\ALDH1A3\PTPRG\PGS1\CLCN5\ARHGAP12\NPEPL1\LUC7L\RAB22A\C17ORF28\STK17B\MLLT10\SFXN2\MOB4\NR1H3\ITPR1\CIB1\FGL2\NAPB\PHTF2\BMPR2\ABHD12\CRELD2\STAM\FXR1\NRK\C4HXORF57\SESTD1\YPEL5\LCLAT1\SNAP23\ACBD3\C6H10ORF46\AKAP9\RBM25\PRDX3\SMOC1\SEC23IP\MSMO1\ZNF650\C5H15ORF29\ACADSB\PTPN14\RAB33B\RASSF3\HHAT\IFNG\RAB3IP\HSPA4L\CAPN1\NPC2\NAP1L1\GYG1\VIPAR\CMPK1\ALKBH1\EP

		S15\BTF3L4\SLC25A4\MEMO1\ACSL1\C4H4ORF41\PPPDE1\GOLGA5\AHR\NUDT9\INADL\SERPINA5\AFF1\COBLL1\SEC31A\SMOC2\PLCL2\RAB5A\BTG1\TMEM66\FGFR1OP\ELK3\GPD1L\PECR\BCL2L14\UHRF1BP1L\SOD2\ACAD11\ESR2\ATF7IP\KIAA1143\RPL7L1\METTL14\RTN1\PSMA3\SFRP4\NCKAP5\GMFB\SHPRH\TDRD7\EPC2\GKAP1\LOC100857286\JARID2\TDG\ADAM12\GCM2\BLOC1S5\SERPINB2\SERPINB6\KIAA1468\C5ORF22\ADCY2\BPGM\DERA\TBL1X\ERP44\MBP\CNOT7\LOC426218\LONRF1\KIT\SYTL2\USP48\ITFG2\FGFBP2\PHYH\LYSMD3\CAST\ABHD3\RBBP8\CHD1L\TAF4B\DAZAP1\RB1CC1\CLTA\UBXN2B\LOC421792\USP45\GPR89B\CHMP2B\PREX2\LRPAP1\ABCC13\SAMSN1\PNRC1\CYB5R4\C1H21ORF59\MMP16\MORC3\FZD6\SIK1\PDK3\GRHL1\ASXL2\RP2\UXS1\SLC15A1\SLAIN1\SLAIN1\LCP1\RNASEH2B\ZAR1L\UBL3\SLC46A3\PAN3\NPAT\MTMR2\KLHL5\KIAA1609\SEPT6\CLN8\PPP6R3\MCM6\EAF2\FAM175B\ATE1\GCG\RABGAP1L\IRAK2\MUSTN1\UVRAG\POU1F1\RALBP1\HPCAL1\DLL1\SLC1A2\EMB\MYO5A\ATP2A2\GTF3C5\SLC47A1\LDHA\NAALADL2\ZSWIM7\TLN2\NUCB2\TNFSF15\C5H11ORF46\CR1L\TIMMDC1\LOC422654\
P<0.05	gga-miR-429-5p_L-1	AP1G1\SLC7A6\C4BPA\RBM39\PSMD5\PRKRIP1\PTBP1\DTWD2\ADPRHL2\ETFA\ARID5B\KPNA6\RYR3\CLINT1\SYS1\AP1M1\ZMYND8\FBXL18\VGLL4\PTGS2\CUL3\RPS6KB1\INTS2\PNAT10\DYNLL2\LOC422214\GLMN\MST4\HMOX2\SNAPC5\EIF4E3\TMEM164\HSPD1\COPG2\ARFGAP2\UGP2\IFRD1\RPAP3\DOCK1\PRPH2\KIAA0020\MED12L\ANGEL1\SNW1\GTF2A1\UFSP2\TMEM48\ITPK1\C5H14ORF129\RFTN1\C5H14ORF79\ATF7IP\CYP2D6\ARPP21\LOC420748\RAB3GAP1\ISCA1\C1H12ORF48\DROSHA\ZBTB2\PBX4\M6PR\GPR162\EVC\TMEM68\UBXN2B\UFL1\HAPLN1\MXD4\WHSC2\SYNCRIP\IMPG1\CCNE2\MORC3\DCAF13\MTMR9\CYFIP1\C1H2ORF49\NCK2\LIG4\IRG1\LCP1\HMGB1\SUV39H2\GTPBP1\C5H11orf24\TANK\C20ORF96\EGFR\ZC3H14\PSMC3\FAM169B\POLG\ZBTB34\ZSWIM7\ATP11B\YOD1\
P<0.05	gga-miR-429-3p	NFU1\ARMC1\BNIP3L\NEFM\CHMP7\GALK2\TERF2\STX12\FAM72A\C11ORF30\ZMPSTE24\NMT1\FBXW2\NR0B2\TAF12\GJC1\FAM118B\GATSL2\SNAP29\GIGYF2\NFS1\INPP5B\LOC416777\UBAC1\SLC16A1\E2F4\TEK\LRWD1\PTBP1\TRIM33\GPRIN2\NR5A2\SEC11A\PAIP2\RGS2\NUP35\DNAJC12\RASA2\RSL1D1\TARDBP\NRG4\AGFG1\MMD\UBIAD1\RHOT1\SLC12A4\FN1\HEATR2\MPHOSPH10\PPT1\COPS8\AP1M1\PNRC2\PABPC1L\SRSF10\YWHAB\DDX50\ADA\RNF111\OIT3\RSRC2\HVCN1\RGS9BP\SET\RGL1\MPRIP\FAM129A\RNF2\PHACTR3\COL4A4\PPARG\GIT2\RPS6KB1\VCAM1\INTS2\SASS6\JMJD4\PNAT10\MPHOSPH6\ARHGEF3\SNX12\CHEK2\C20H20ORF11\SPPL2A\SEPT2\TMEM41B\MTF2\LOC416931\KLHL13\PDE8A\MST4\DDX26B\MKLN1\HTATSF1\UBE2I\ANKRD1\GRM3\PDE6C\AP2A2\IL13\TSPAN6\NCBP2\KIF3A\LOC395991\PPP1R2\ANKDD1A\ZEB1\SPG21\GCN1L1\TOB1\CTSZ\MTMR8\CHIC1\TMEFF2\PUS10\EIF4E2\SF3A1\GTF3C3\MRPS7\UBE2V1\SNAI1\CELF1\CTDSPL2\PCGF6\FGL2\ALS2\SMARCA1\STAG2\SLC38A2\TTL\LOC417741\C1D\LRSAM1\NDUFA5\UGP2\FXR1\FAM3C\AK5\C6H10ORF46\IFT140\PRKCI\KRIT1\SLC39A9\SERPINI1\IRAK4\GAD1\GPATCH2\C1GALT1\DTL\NEK2\ZNF330\DIEXF\RAP1B\ABCE1\CDC20\CFL2\FAM177A1\GLO1\FBXO33\LARP1B\VSX2\ZDHHC17\GTF2A1\DCTD\PPPDE1\MEOX2\M

		TR\SPC25\RAPGEF5\GALNT3\SNX10\GLRX5\ABCB10\SLC4A10\CHN2\C3H6ORF120\EPYC\KERA\AAMP\T\PPP1R13B\IGF2R\CLASP1\MYLK\ESR2\MAD2L1\CLASP2\PCDP1\KIF18A\ITGA9\TAB2\MMADHC\NTRK2\FANCC\DEK\SLC41A2\C1H12ORF48\NUP37\BLOC1S5\TXNDC5\RIPK1\BID\ADCY2\KIAA0528\SORBS2\SYNE1\SGCB\ARHGAP18\CYB5R3\TEC\CORO6\MYH10\MEF2BNB\ELL2\DEPDC1B\GDAP2\SERINC1\C6ORF170\METTL16\ZUFSP\NPC1\EVC\MAMDC2\ABHD17B\MEP1B\GPR161\PCMTD1\OSTM1\HJURP\NRG1\CHMP2B\LOC427306\SH3BP2\MXD4\USP25\LYRM2\LRRCC1\CCNE2\PTDSS1\PRADC1\U2AF1\ASAP1\GCM1\FAM49A\NRBP1\PIGA\EGFL6\EIF5B\REV1\C1H2ORF49\ARGLU1\DNAJC3\LOC418811\BORA\GPALPP1\COG6\N4BP2L2\FLT3\MTMR2\ARL6IP5\SUV39H2\PACSIN2\GTPBP1\TMEM196\CLN8\EPM2A\LOC395933\PTGR2\INCENP\PDDC1\MCM6\C7H2ORF76\PPP4R2\MFI2\RABGAP1L\RRN3\TEX2\PRR11\TCEB3\WDR82\TMEM131\CRISPLD1\POPDC3\SLIT2\ASF1A\ANLN\TBP\GLDC\SLC10A7\WIPF1\FASN\XIRP1\CNN3\ATP1B4\TBC1D22A\TLN2\HEATR3\LMAN1\C5H11ORF46\NAV2\HNRNPH1\RABEP1\YOD1\
P<0.05	gga-miR-383-3p	TRIM27\YTHDC2\ADAM28\CHMP7\DCAF7\ZNF276\ZNF593\DDX42\CHTF8\CNTN2\CYB5B\KLHDC8A\PM20D1\CACNA1S\TBC1D22B\HYAL1\SLC7A6\NUDC\LHFPL5\PFDN1\NEK6\CIAPIN1\PRKCZ\SCLY\SNAP29\CASP9\RALGAPB\GUCA1A\PTPRU\RAB14\IRF1\GIGYF2\CCNL2\MOV10\PPM1J\RPAIN\SLC16A1\EIF6\PRKRIP1\ORAI2\TEK\ROGDI\ST6GALNAC1\TRIM33\SMU1\NCOA4\HSD17B4\WBP2\FBXW11\HEMK1\KLHL6\ADPRHL2\AP3S1\SEC16A\GBAS\RGP1\VPRBP\SMN\GPR157\DNAJC18\LZIC\ZC3H15\DNAJC9\XRN1\SLC16A3\INPP5K\C26H6ORF106\TMEM170A\PHACTR4\LRRC45\TARDBP\TRIP12\SRM\TADA1L\CERS1\GABARAPL2\KIF20A\FAF2\GBGT1\SCYL3\UBIAD1\RAB5C\ARL10\KIAA1191\ESRP2\DNAJC7\RBBP4\SGK2\SKP2\VTN\NT5C3L\TOP1\USE1\WDR70\ANO5\ASAH2\TRIT1\SMARCE1\P2RX4\PPM1M\CAMKK2\RAB33A\KDM4B\CHRNA7\STK4\SRSF10\RNF111\KIF4A\PSMG3\FAM20B\LOC420160\LFNG\GOSR1\OIT3\CCDC69\DYX1C1\CCPG1\SH3GLB2\SGPL1\PPP1CC\RPS14\C14H17ORF103\NOL8\PPIF\FLCN\COPS3\CCNDBP1\DUT\TSEN2\DKC1\DPM2\TRIM37\MYO1H\DHX40\WDFY1\FUT11\COL11A1\RPS6KB1\EPHA4\VCAM1\TK2\SLC25A36\IBA57\HAGHL\AATF\C17ORF78\TAF1\WFDC1\ASB14\NTHL1\TRPM2\SNX12\USP10\CHEK2\SGSM2\BOK\ARFGAP1\DCAF12\TMEM41B\ACAA1\TMED5\MYD88\TRIM66\GLMN\TPRA1\ARFRP1\EARS2\RTEL1\MORN4\DDX26B\HTATSF1\LOC422249\NDE1\TNNT3\TOLLIP\OAF\CBL\KLHDC8B\ARL6IP1\IL13\KIF3A\PISD\CTSA\FAM174B\RNH1\CHM\LIMK2\BLNK\POLR3E\PYROXD2\ACAP2\ITGB1\RMI2\COQ5\UTS2D\RNF166\RNF214\CLPX\SUDS3\TMPRSS4\ARCN1\MTMR8\STAT1\SUCLG2\CHIC1\LRRC59\NPM3\C17ORF28\PTS\MLLT10\KCTD2\SF3A1\HN1\GGA3\UBE2V1\TMEM180\LOC417013\SLC9A8\ANP32A\TMEM164\CELF1\MOB4\UACA\ADAMTS7\ANKRD16\CTDSPL2\FBXO21\CASC4\DDB2\ORC2\MED13L\ARFGAP2\NEURL\SDSL\PDIA3\NDUFS1\PDCD4\TRABD\SHOC2\DCUN1D1\UGP2\FAM3C\TMEM87A\PIF1\EBNA1BP2\LCLAT1\NCSTN\ACBD3\LEFTY2\DEGS1\DPF3\PRKCI\CYP51A1\ST7\CAPZA2\CAV2\SLC39A9\SERPINI1\TMEM229B\SCHIP1\ACADSB\MUM1\C5H15ORF41\IFNG\HAAO\SLC35E3\DYNC2LI1\SMAD1\EDNRA\BRE\C4H8ORF40\SEC23A\AARS2\GLIS3\FBXO33\NPC2\GPR149\MED12L\SNCA\EIF2A\FAM98A\LOC42504

		9\CMPK1\SNW1\EPS15\YIPF4\CCDC75\GTF2A1\UFSP2\ACSL1\C4H4ORF41\PPPDE1\C5H14ORF159\AGA\MAP1LC3C\GOLGA5\UBR7\AREGB\MACC1\SPC25\SPP1\STK31\PGM1\ROR1\TSNAX\ATG2B\C5H14ORF129\CDS1\RFTN1\KERA\ZRANB2\CRYZ\BCS1L\TMEM66\AAMP\TNFAIP2\NEDD1\BRP44L\T\NUP54\BAG5\BIN1\AMPH\GRSF1\NR1H4\IL8L1\MTHFD1\HEBP1\ESR2\SYNE2\POLDIP3\CCNA2\KIAA1143\CTNNB1\RANGAP1\FBXL2\SEC24D\METTL14\CAMK2D\TAB1\LOC420748\ITGA9\TARP\TOM1\WDSUB1\PRDM4\RBM24\HSP90B1\BLOC1S5\TBXAS1\ECI2\VPS4B\MYO10\CCDC77\DAP\ATP6V1E1\CLPTM1L\RHBG\LOH12CR1\ANP32E\FAM149A\MTUS1\LOC426218\DNAJC16\NPEPPS\CYB5A\EZR\MVK\MAP7\HBS1L\SGCB\FAM76A\DCUN1D4\CWH43\OCIAD1\PPFIBP1\PARVB\SCNN1A\RFC1\ITFG2\EDARADD\ZCCHC4\MRPL51\LOC431003\FGFBP2\CD38\PHYH\LPCAT3\CADM1\GPR98\MTX3\C7\CEP85L\METTL16\JAK2\AMD1\MEP1B\DTNA\C1H3ORF26\TBC1D23\C3H6ORF203\NANS\KCMF1\ARL13B\SDR16C5\UBXN2B\SDCBP\CHMP2B\AFAP1\SH3TC1\ADD1\SH3BP2\USP25\TMEM129\LPAR1\SNX14\CA2\ATP6V0D2\LOC100857673\IRAK1BP1\TMEM30A\RAD54B\DONSON\PLEKHF2\MORC3\CHAF1B\UBR5\DDX3X\FBXO9\LOC396380\DERL1\CALB1\FAM49A\MSGN1\UBXN2A\SF3B14\KRTCAP3\C1H2ORF49\UXS1\COL4A2\CARS2\TNFSF13B\ABHD13\DNAJC3\MYCBP2\BORA\LCP1\SLC25A15\ZAR1L\SLC46A3\NUPL1\FZD4\LCMT2\RRM1\ARL6IP5\PSMB4\CD1C\KLHL5\ALG8\SEPT6\GTPBP1\UBE2N\CERK\TERF1\RPRD1A\UPP1\ETV1\WDR48\CLN8\HCRTR2\C6ORF192\LOC422284\F8\SLC25A14\KLHDC2\KCNAB1\EAF2\HABP2\KLF1\ARHGAP19\C20ORF96\GRAP\RPE\RNF7\RRN3\NCF1\PUF60\TRIB2\LGSN\MTO1\AFAP1\C4H4ORF52\CRTAP\PCMT1\PDLIM5\TGFB3\ACYP1\CCDC101\IRF2\RAD54L\RPL35\DHX38\GABPB1\CYP24A1\FASN\EMB\BIRC5\COL17A1\GTF3C5\SLC47A1\C5H11ORF46\LIN7B\C5ORF5\PSMD13\POLE3\WRAP73\B4GALT7\PLEKHJ1\YEATS2\CD320\LOC100859100\
P<0.05	gga-miR-365-2-5p_L-1R+1	GFPT1\SPPL2B\RPA2\TERF2IP\TIRAP\HES5\SAP130\VAV2\RFNG\AARSD1\DNAJA4\FGFR1\SBNO1\DNAJC7\SFRP1\FEN1\PRRC2B\NT5C1A\NDST1\MAPKAPK5\ADK\CDC14A\CER1\WFDC1\ABHD6\C20H20ORF11\IDE\CDA\MASP1\ITGB2\LOC424919\MAGT1\SFXN2\ARFGAP2\YPEL5\EHD3\CDAN1\YAF2\MRPS10\MME\DAZL\CRADD\GPD1L\BIN1\ATP2C1\DEK\BLOC1S5\CREB3L2\KIAA1468\SOGA2\PDLIM3\GFPT2\MED6\MYB\TTC37\SPAG17\C9ORF93\NRG1\PSD3\TCEB1\CDH17\RAD21\GSTA3\GSTA3\FDFT1\N4BP2L2\MMP13\FAM125A\C2H6ORF62\LOC776051\WDR48\LOC100859645\LOC424098\NDUFA10\NADK\LOC101750010\OVST\PDLIM5\TBC1D22A\PSME3\ANG\
P<0.05	gga-miR-3607-5p_R+1	GFPT1\PRNP\LOC426699\CAPN5\ATP6V1B2\JMJD6\PMPCA\PARG\NR5A2\AGO3\KCTD7\CALCRL\MLX\KIF20A\ERLIN1\TM2D2\LPCAT2\KPNA2\CLINT1\CHRNA7\PNRC2\KCNJ2\TMEM116\RNF2\COL4A4\ALKBH5\TSEN2\CLTC\FAM120A\PTH\LPPR5\C20H20ORF11\TNFAIP1\MTF2\GLMN\WNT8A\MAPK8\MOSPD1\TGFBI\PCBD2\PDPK1\ANKRD1\C13H5ORF15\BCCIP\ITGB1\ZEB1\CUTC\KMO\MTMR8\KIAA1841\APIP\RTN4\NR1H3\CASC4\CNTN4\PSMD10\EDEM1\WDR96\AP2M1\SLC38A2\MCTS1\PDCD4\RSU1\PPP3R1\SHOC2\FXR1\COMMD5\PIGK\FMR1\LEFTY2\KRIT1\IFRD1\VTI1B\YAF2\DCAF17\GPATCH2\SHFM1\PROX1\NEK2\MBIP\VLDLR\GPR149\ANGEL1\EPS15\YIPF4\AHR\ERO1LB\NTPCR\PGM1\LEPROT\GLRX5\IL8L1\ATF7IP\STARD3NL\BMPER\AVL9\ITGA9\LRRFIP2\SLC16A8\STK17A\SLC9A3\HMGX

		B4\SLC41A2\IRF4\ZBTB2\ESR1\ATP6V1E1\PLEKHA5\CLEC3A\ABCC9\TMF1\PLEKHO1\SPTLC1\PCM1\MED6\LOC421049\CWH43\ARFGAP3\FBLN1\SCNN1A\RELL1\PI4K2B\FAM162A\CD38\SGTB\ANKDD1B\OTOP1\JAK2\ZFAND5\NRG1\ACOT12\LOC418465\PEX2\CTBP1\GABPA\SYNCRIP\MMP16\GCFC1\NOV\HADH\SIK1\PRIM2\FBXO9\SNTB1\LAPTM4A\RAB9A\COL4A1\TNFSF13B\DOCK9\SPRY2\SLAIN1\SLAIN1\IRG1\UCHL3\KIAA0226L\MRPL48\C11H16ORF87\TXNL4A\UBE2N\POT1\SUCLG1\PPP6R3\ATE1\ALDH1A2\SLC13A2\CENPQ\GAL8\POU1F1\KDM3A\CRTAP\ARHGAP15\HNF1A\EIF4A3\PELI1\TNFSF15\LIN7B\SMAD5\
P<0.05	gga-miR-3523	NFU1\MAPKAP1\NKX2-5\HIGD2A\CDH5\HNF1B\TADA3\TTL\SPP1\ALCAM\MORC3\HOXA11\KIAA1328\SNCB\
P<0.05	gga-miR-34a-3p_R-1	SLC23A2\SPPL2B\GALK2\LAPTM5\RBBP5\PHF20\ANP32B\ITIH3\MYL10\PRKCD\ARVCF\TAF15\HIBCH\PCTP\ACSBG1\UBIAD1\GOLGA7\NECAP2\LOC101749801\PNRC2\KIAA1279\ASB6\HVCN1\OPHN1\TSEN15\MYEF2\PLAU\CDC14A\ARHGEF3\LOC768701\HEATR6\ABCD3\HSCB\LSS\GGT1\ARL6IP1\MARCH5\PPP1R2\METTL9\COQ5\ZEB1\C10H15ORF44\LOC420491\MAP2K1\USP12P1\APIP\EIF4E2\PGK1\B3GNT2\TMEM87A\PTTG1IP\LCLAT1\TASP1\ZFP106\PNLIP\PDGFC\RARRES1\C5H15ORF29\CHST15\RAP1B\MYST2\KIF6\MMACHC\GSTZ1\AGXT2L1\GTF2A1\SECISBP2\TTC4\ITPK1\ERO1LB\IGF2BP3\CBX3\LEPROT\GLRX5\ELK3\SCYL2\C5H14ORF79\LZTFL1\RANGAP1\SNX4\METAP1\TST\NAA35\TXNDC5\C6ORF97\CYP4V2\CNOT7\SLMO2\MAP7\FAM76A\ARFGAP3\FBLN1\ACTN2\CC2D2A\SGTB\LOC421712\HDAC2\GTF3C6\PCMTD1\TMEM175\PREP\FBXL4\SH3BP2\PEX2\UBE2J1\PNRC1\GRIK1\THNSL2\MORC3\TSTA3\U2AF1\PHF20L1\GSTA\FER1L6\DERL1\TAF2\FAM49A\INTS9\TUBGCP5\TNFSF13B\IRG1\LECT1\ELF1\COG3\RNASEH2B\SLC46A3\C1H11ORF54\C1H21ORF33\CLN8\AK6\ZNHIT6\SUMO3\MPZL3\NADK\WDR82\SNX13\POLE3\GRIN1\SLU7\PLEKHJ1\
P<0.05	gga-miR-33-5p	ARHGAP25\CAMK4\PCNX\HMGN2\ITGB3\HBXIP\SPPL2B\DDX42\CDC27\ZCCHC17\FBXW2\NR0B2\SRPR\SAMHD1\RSPRY1\TTC1\STOML1\GLB1L2\ATP6V1B2\ACSBG2\SFMBT1\GFRA2\IWS1\ST6GALNAC2\PLEKHB2\NR5A2\RFFL\NUBP2\HSPA9\ENO1\ZC3H15\DUSP19\ACAA2\RFNG\ARID5B\SCPEP1\KIF20A\DNAJA4\LMX1A\SFRP1\ALDH9A1\SARM1\KRT20\RNF111\KNTC1\MAPK6\SET\SLC12A1\AP3M1\LRRC39\LOC416530\DYNLRB2\BCMO1\TEAD1\ARHGEF3\LOC768701\RNF141\STK25\XBP1\DCAF12\BCAR3\DR1\MTF2\MYD88\LOC100858792\RUFY1\ABTB1\GALNS\NAP1L4\C12H3ORF19\DNAJB6\ANKRD1\OAF\YWHAH\POU2AF1\RNF185\METTL9\ACOX2\SYNPR\SVIL\LPP\FYTTD1\MTMR8\HMOX2\LOC416354\KCTD2\SRPK2\ORC5\ALDH7A1\CNTN4\FGL2\SDSL\CRY2\SLC39A12\TYRO3\CXORF56\WASL\CROT\SRI\LCLAT1\DEGS1\SLC39A9\LOC424998\CCDC132\RAB3GAP2\GPATCH2\LXN\XRCC6BP1\TRAF5\IFNG\G2E3\PRDX1\TSPAN1\MED12L\AHCTF1\PPPDE1\C5H14ORF159\MEOX2\RAPGEF5\VRK1\EML1\TECRL\TMEM66\ELK3\UHRF1BP1L\CCK\NAA30\FBXO34\ITGA9\METAP1\UBE2D3\STK17A\NOL12\WDSUB1\GKAP1\TXNRD1\HIPK2\AGK\DAP\BPGM\EXOC3\TBC1D1\CYP4V2\FRG1\PPAT\KDR\FGFR1OP2\NUP50\LIMCH1\SLC34A2\EMG1\ARSK\PTPRK\MTX3\GREB1L\ESCO1\RABL3\NPC1\CLTA\MAEL\CHMP2B\MYBL1\GRK4\TCEB1\HSPA13\S

		H3BP2\RNF4\WHSC2\MMP16\IMPG1\TMEM30A\EEF1A1\MORC3\UTP23\SIK1\DDX3X\PXDN\DERL1\NBAS\LAPTM4A\HADHB\PIGA\RAB9A\C1H2ORF49\ATP4B\COL4A1\TNFSF13B\STK24\LMO7\RCBTB1\SLC25A15\POSTN\MAB21L1\N6AMT2\VAPA\KLHL5\LYN\VMO1\C1H21ORF33\PACSIN2\CERK\GATA2\EBAG9\PTPN3\LOC422426\IL13RA2\PTGR2\KCNAB1\TGFBR3\CCDC39\EEF1A2\TAPT1\EGFR\PDLIM5\ARHGAP15\REEP5\LUC7L3\FAM169B\CUL2\SLC10A7\LLPH\WIPF1\CYP24A1\ARPP19\LSP1\TRIM59\TMEM120B\AP4B1\LIN7B\PDZD11\SLU7\YEATS2\ATCAY\
P<0.05	gga-miR-30e-5p_R+5	ARID4A\BNIP3L\NEFM\DCAF7\CSNK2A2\IL10\ANKHD1\SNAP29\UBE2L3\PSMD5\CPSF3L\ATP6V1B2\UBAC1\JMJD6\ORAI2\SDF4\NR5A2\AGO3\SORD\AP3S1\CFH\KCTD7\SLC15A4\GK5\INPP5K\RASA2\NEDD4L\BECN1\MMD\PARN\NFYA\KIAA1191\ESRP2\DNAJC7\TRRAP\RAPGEF1\XPR1\AP1M1\DNA2\CSRP3\FAM81A\PNRC2\RNF111\RNF34\KIF4A\CACYBP\HVCN1\FAM214A\PDCD5\RGS9BP\NOL8\RASD1\PEMT\AP3M1\SLC25A36\SLC35A3\BTBD10\RHOG\ARHGEF3\ADRBK2\ABCD3\SPPL2A\TMEM41B\RAB7A\MYD88\GORASP1\WNT8A\HPRT1\GALNS\DDX46\HTATSF1\CARS\UBE2I\LOC422249\SEMA3A\KIF3B\UPB1\NCBP2\BDH1\PPP1R2\POLR3E\CABP1\NRP1\RNF10\SYNPR\THOC7\NME1\STX16\RAB22A\MTMR8\SUCLG2\NPM3\PUS10\ATP7A\STK17B\GGA3\RNF114\TMEM180\ACTR1A\HSPD1\MOB4\GDI2\PGGT1B\NAPEPLD\LRRC17\PHTF2\MCTS1\SENP5\TRABD\PPP3R1\SHOC2\ADAM22\TBC1D8B\ZFP106\EHD3\GFRA1\CRDS2\DCAF4\PPID\SERPINI1\PLEKHH1\GXYLT1\YAF2\SRP14\C5H15ORF29\DOCK1\ELMOD2\SMARCA5\RAP1B\SRP54\HP1BP3\ZFC3H1\TBC1D15\LARP1B\NAP1L1\EIF2B2\FAAH\BIRC6\CCDC75\SCCPDH\PPPDE1\GOLGA5\AGR2\ITPK1\MACC1\GALNT3\CBX3\C5H14ORF129\CCNK\C3H6ORF120\WDFY3\DEPDC1\LRRC40\LHX8\GGCT\IGF2R\IL2\UBN2\SLC38A6\CYP2D6\POLR3H\XPNPEP3\STARD3NL\SLC35F4\FBXO34\FKBP9\UBE2D3\HECW1\TDRD7\THSD7B\IYD\NOL12\SOUL\KIF13A\TDG\TXNDC5\MKRN1\PRKDC\DDX11\ROPN1L\GALNT1\PRMT8\TDP2\MAPK9\C6ORF115\SLMO1\PTPN2\LOC426296\HBS1L\SGCB\GRK6\EED\KIAA0930\SMC1B\MAN1A2\MLF2\SERINC1\NUS1\ESCO1\ABHD3\RABL3\WDR1\B4GALT6\ATG5\SDCBP\GJA5\CPA6\PTGR1\TMEM129\UBE2J1\SYNCRIP\NT5E\C1H21ORF59\OTUD6B\GCFC1\TP53INP1\ATP6V1C1\PHF20L1\PTP4A1\MYOM2\FER1L6\CALB1\DSCC1\SH3KBP1\TAF1B\GRHL1\FAM49A\NT5C1B\RHOB\MTMR9\REV1\CHST10\C1H2ORF49\TNFSF13B\LIG4\DZIP1\BORA\WDFY2\NUPL1\N6AMT2\TYR\ARL6IP5\LCORL\YWHAZ\KIAA1609\C3H2ORF43\CLN8\IMMP1L\C5H11orf24\MCM6\EAF2\DAB1\DCLRE1A\ADAP1\STX2\CDC42\NADK\C26H6ORF89\WDR82\WWP1\COL9A1\KDM3A\RSPO3\BMPR1B\CDCA7L\LOC100859142\CUL2\SLC1A2\WIPF1\GATM\EMB\DDX52\TRIM59\TBC1D22A\TBCD\ATP2A2\GTF3C5\COG5\NAALADL2\NUCB2\DDX19B\SMAD2\COL6A1\ZNF488\TNFSF15\CACNA1B\OCRL\SLU7\YEATS2\HSBP1L1\
P<0.05	gga-miR-30e-3p_L+1	RFK\ARID4A\ARHGAP25\LOH11CR2A\SPPL2B\LMNB2\PGM2\ACOT7\ZMAT2\AP1G1\CCL4\GAPVD1\LDLRAP1\C4BPA\APLP2\SNAP29\SSU72\RBM39\WDR45L\UBE2R2\ODZ2\EIF6\SDF4\AMY2A\DYNLRB1\COMT\NCOA4\ACO1\AGO3\MAPKAPK3\CDC73\CALCRL\C19H17ORF85\ZC3H15\MED23\NUDT3\ETFA\SUGP2\FECH\MMD\ERLIN2\REXO4\ASH2L\SUB1\ACSBG1\KIAA1191\SBNO1\SLC12A4\KPNA2\UBLCP1\

		CLINT1\LIFR\RICTOR\KRT19\MFAP3\FAM114A2\PNRC2\NADSYN1\RNF111\FAM20B\RALGPS2\RCAN3\GOSR1\TBCA\SH3GLB1\SGPL1\HVCN1\RGS9BP\MAPK6\CPSF4\LOC768735\LSM14A\DRG2\TSEN2\ALKBH2\PTGS2\PLAU\INTS2\RRP1B\TRMT13\IBA57\NARFL\FRRS1\TADA2A\NONO\LOC768701\ADIPOQ\RNF141\C20H20ORF11\TNFAIP1\SQRDL\SPPL2A\TRAF7\SQSTM1\MTF2\KLHL13\MED31\SH3GL3\MST4\RPS13\FAM122A\CCBL2\ACTR3B\DDT\SPTY2D1\TBX6\PDPK1\ABHD17C\ANKRD1\C9H21ORF2\ABCC1\TMEM41A\NCBP2\CHM\PFKP\BDH1\ARRDC4\ALDH1A3\RNF10\ITGB1\CLCN5\RAB35\TOB1\RBM27\ACBD5\RAB22A\CDC23\BTG4\SUCLG2\USP12P1\MAGT1\STK17B\DCX\TSEN54\UQCR10\LOC424943\AOX2P\RELN\FBXO21\BUB1\ORC2\CFLAR\TBX3\CNTN4\CRNKL1\SLC38A2\ABHD12\COBRA1\CRELD2\LOC417741\NKRF\KNG1\TRDMT1\CUBN\POLR3F\LOC769134\SHOC2\DCUN1D1\SAMD13\FXR1\SRI\TSPAN12\SESTD1\SPTLC3\TRMT6\SP3\SEC62\ST7\CAPZA2\GLRB\RXFP1\BAG3\EXD2\YAF2\SLC25A12\TLK1\RARRES1\EPRS\RRP15\METTL5\SSB\RAB33B\LPGAT1\NEK2\HHAT\G2E3\BRE\GEMIN2\VLDLR\MMACHC\ZDHHC17\GPR149\VASH1\MED12L\FAM98A\FAAH\AHSA1\ALKBH1\RNF11\BIRC6\BTF3L4\GTF2A1\DPY30\MEMO1\GALNT7\FMN2\MEOX2\LGMN\GOLGA5\ITPK1\ASB5\UNC79\TRA2A\HSD17B11\C3H6ORF120\SERPINH1\DAZL\WDFY3\CRADD\PLXNC1\LTA4H\ELK3\EIF5\DYNC1LI1\BIN1\UHRF1BP1L\MKI67IP\C5H14ORF79\PTPLB\IL2\CCR8\SNX4\FSHB\TARP\PPP3CA\VPS41\DARS\MMADHC\TRIP13\WDSUB1\TGFBR1\PWP1\RBFA\TMEM263\CTDP1\PRL\ALDH1L2\SLC41A2\MYLIP\C1H12ORF48\SSR1\SERPINB10\KIAA1468\ZBTB2\BPGM\STRAP\TERT\GABBR2\CNOT7\CYB5A\MPPE1\KIT\SGCB\SYTL2\NFXL1\ADHFE1\PDS5A\RFC1\RELL1\EDARADD\ANAPC4\SEPSECS\ING4\CD38\ERAP1\EPHA1\KIF2A\LRRC70\SGTB\SREK1\PAPD4\C6ORF170\METTL16\RABL3\FOCAD\HAUS6\CDH2\PCMTD1\TBC1D23\HJURP\SDR16C5\SDCBP\BRP44\GPR89B\ABLIM2\ACOX3\TCEB1\FAM193A\PEX2\WHSC2\JAM2\CYB5R4\CA2\LCA5\SLC17A5\ITSN1\TTC3\OXR1\EMC2\NOV\RAD21\DENND3\SIK1\DDX3X\GSTA3\FBXO32\CALB1\MBOAT2\MELK\GRHL1\ROCK2\LPIN1\FAM49A\CYP39A1\CHST10\TNFSF13B\STK24\SLAIN1\SLAIN1\IRG1\LMO7\BORA\C1H13ORF15\UFM1\N4BP2L2\UBL3\MTMR2\MID1\POU2F1\PACSIN2\UBE2N\COLEC12\C2H6ORF62\PTPN3\MLANA\LOC100859645\SGK1\IMMP1L\LOC395933\LOC422426\PTGR2\C5H11orf24\MCM6\TM4SF1\DAB1\TANK\GCG\PLEKHA3\SPECC1L\LOC427470\FYB\TJP1\NADK\IRAK2\FLNB\RBM6\FAM48A\ATP6AP2\IL7\SOD1\POU1F1\PIGN\OVAL\ROS1\EGFR\PDLIM5\HOXA11\ARHGAP15\NPVF\CDCA7L\MORC2\CUL2\SLC10A7\ARSB\MON2\NELL2\EMB\DDX27\MYO5A\TMEM120B\POP4\PELI1\KIAA1328\PTPN22\C5H11ORF46\RABEP1\WRAP73\SLC9A9\PDZD11\TIMMDC1\
P<0.05	gga-miR-30d_R+2	ARID4A\BNIP3L\NEFM\DCAF7\CDC27\CSNK2A2\IL10\ANKHD1\UBE2L3\PSMD5\CPSF3L\ATP6V1B2\UBAC1\JMJD6\ORAI2\SDF4\NR5A2\AGO3\SORD\AP3S1\GTF2H2\CFH\KCTD7\SLC15A4\GK5\INPP5K\RASA2\BECN1\MMD\PARN\NFYA\KIAA1191\DNAJC7\TRRAP\RAPGEF1\XPR1\AP1M1\DNA2\CSRP3\FAM81A\PNRC2\RNF111\RNF34\KIF4A\CACYBP\HVCN1\FAM214A\PDCD5\RGS9BP\MAPK6\NOL8\RASD1\PEMT\AP3M1\SLC25A36\SLC35A3\SELK\RHOG\ARHGEF3\ADRBK2\ABCD3\SPPL2A\TMEM41B\RAB7A\MYD88\GORASP1\WNT8A\HPRT1\

		GALNS\DDX46\HTATSF1\CARS\UBE2I\LOC422249\SEMA3A\KIF3B\UPB1\NCBP2\BDH1\PPP1R2\POLR3E\CABP1\NRP1\RNF10\SYNPR\THOC7\NME1\STX16\MTMR8\NPM3\PUS10\ATP7A\STK17B\GGA3\RNF114\TMEM180\ACTR1A\HSPD1\MOB4\GDI2\PGGT1B\LRRC17\PHTF2\MCTS1\SENP5\TRABD\PPP3R1\SHOC2\SEC23B\TBC1D8B\ZFP106\EHD3\GFRA1\CRDS2\DCAF4\PPID\SERPINI1\PLEKHH1\GXYLT1\YAF2\SRP14\C5H15ORF29\DOCK1\ELMOD2\SMARCA5\RAP1B\SRP54\HP1BP3\ZFC3H1\TBC1D15\LARP1B\EIF2B2\FAAH\VIPAR\BIRC6\CCDC75\SCCPDH\PPPDE1\GOLGA5\AGR2\ITPK1\MACC1\GALNT3\DOCK7\CBX3\C5H14ORF129\CCNK\C3H6ORF120\WDFY3\DEPDC1\LRRC40\LHX8\GGCT\IGF2R\IL2\UBN2\SLC38A6\POLR3H\XPNPEP3\STARD3NL\SLC35F4\FBXO34\FKBP9\HECW1\THSD7B\IYD\SOUL\KIF13A\TDG\TXNDC5\MKRN1\PRKDC\DDX11\ROPN1L\GALNT1\TDP2\SNX9\MAPK9\C6ORF115\PTPN2\LOC426296\HBS1L\SGCB\GRK6\EED\KIAA0930\SMC1B\MAN1A2\MLF2\SERINC1\NUS1\ESCO1\ABHD3\WDR1\B4GALT6\ATG5\UBXN2B\SDCBP\GJA5\GPR89B\CPA6\PTGR1\SYNCRIP\NT5E\C1H21ORF59\OTUD6B\GCFC1\TP53INP1\ATP6V1C1\PHF20L1\MYOM2\FER1L6\CALB1\DSCC1\SH3KBP1\TAF1B\GRHL1\FAM49A\RHOB\MTMR9\REV1\CHST10\C1H2ORF49\TNFSF13B\BORA\WDFY2\TYR\ARL6IP5\LCORL\YWHAZ\KIAA1609\FAM49B\C3H2ORF43\CLN8\IMMP1L\C5H11orf24\MCM6\EAF2\DAB1\ADAP1\STX2\CDC42\NADK\C26H6ORF89\WDR82\WWP1\COL9A1\KDM3A\RSPO3\BMPR1B\CDCA7L\LOC100859142\CUL2\SLC1A2\WIPF1\GATM\EMB\PGRMC1\DDX52\TRIM59\TBC1D22A\TBCD\ATP2A2\GTF3C5\COG5\NAALADL2\NUCB2\DDX19B\SMAD2\COL6A1\ZNF488\TNFSF15\CACNA1B\YOD1\OCRL\SLU7\YEATS2\HSBP1L1\
P<0.05	gga-miR-30c-5p	ARID4A\BNIP3L\DCAF7\CSNK2A2\IL10\ANKHD1\UBE2L3\PSMD5\ATP6V1B2\UBAC1\JMJD6\ORAI2\SDF4\AGO3\SORD\AP3S1\GTF2H2\CFH\KCTD7\SLC15A4\GK5\NEDD4L\BECN1\MMD\PARN\NFYA\KIAA1191\DNAJC7\RAPGEF1\XPR1\AP1M1\DNA2\CSRP3\FAM81A\PNRC2\RNF111\RNF34\KIF4A\CACYBP\HVCN1\FAM214A\PDCD5\RGS9BP\NOL8\RASD1\SLC35A3\RHOG\ARHGEF3\ADRBK2\SPPL2A\RAB7A\MYD88\GORASP1\WNT8A\GALNS\DDX46\HTATSF1\CARS\LOC422249\SEMA3A\UPB1\NCBP2\CHKA\BDH1\PPP1R2\CABP1\NRP1\RNF10\SYNPR\THOC7\NME1\STX16\MTMR8\SLC9A3R1\NPM3\PUS10\ATP7A\STK17B\GGA3\TMEM180\HSPD1\LRRC17\PHTF2\MCTS1\SENP5\TRABD\PPP3R1\SHOC2\ADAM22\TBC1D8B\ZFP106\EHD3\CRDS2\DCAF4\PPID\SERPINI1\GXYLT1\SRP14\C5H15ORF29\RAP1B\SRP54\ZFC3H1\EIF2B2\PPP1R12A\FAAH\BIRC6\SCCPDH\PPPDE1\GOLGA5\ITPK1\MACC1\DOCK7\CBX3\C5H14ORF129\CCNK\WDFY3\DEPDC1\LRRC40\LHX8\IGF2R\IL2\UBN2\KIAA1109\SLC38A6\CAMK2D\FBXO34\FKBP9\HECW1\THSD7B\IYD\SOUL\KIF13A\TDG\TXNDC5\IRF4\MKRN1\PRKDC\DDX11\GALNT1\TDP2\C6ORF115\LOC426296\HBS1L\SGCB\GRK6\EED\KIAA0930\SMC1B\MAN1A2\EDARADD\MLF2\SERINC1\NUS1\ESCO1\ABHD3\WDR1\B4GALT6\ATG5\UBXN2B\GJA5\CPA6\UBE2J1\OTUD6B\GCFC1\TP53INP1\ATP6V1C1\PHF20L1\KIAA0196\MYOM2\FER1L6\CALB1\TAF1B\FAM49A\NT5C1B\RHOB\MTMR9\REV1\C1H2ORF49\TNFSF13B\LIG4\UBAC2\WDFY2\NUPL1\N6AMT2\MTMR2\TYR\ARL6IP5\LCORL\KIAA1609\C3H2ORF43\CLN8\IMMP1L\CHP1\C5H11orf24\PDDC1\MCM6\EAF2\DAB1\ADAP1\CDC42\NADK\C26H6ORF89\WDR82\WWP1\COL9A1\KDM3A\BMPR1B\CDCA7L\LOC100859142\CUL2\SLC1A2\WIPF1\GATM\EMB\DDX52\DDOST

		\TRIM59\TBC1D22A\TBCD\ATP2A2\GTF3C5\COG5\NUCB2\DDX19B\SMAD2\COL6A1\TNFSF15\CACNA1B\OCRL\HSF1\YEATS2\HSBP1L1\
P<0.05	gga-miR-30c-1-3p_R+1	RASSF2\LIN28A\KCNC4\RAB11B\TERF2\PM20D1\PPP1R8\SLC26A9\TBC1D22B\FBXW2\KIF18B\NUDC\GFAP\NR5A1\NEK6\LOC419722\CHD6\MRRF\WDR45L\MAN2C1\SEC14L1\SCAMP2\JMJD6\MAP1LC3A\PKM2\BCAS2\C15H22ORF25\AP3B2\OGDHL\HIBCH\SORD\ENO1\SEC11A\RGS4\COG4\LZIC\XPO6\PEX14\GRM4\RHO\AARSD1\TRAIP\TARDBP\PRPF8\ERLIN2\DDX49\TSR1\KIF20A\UBIAD1\KPNA6\C18H17ORF75\LOC425137\PPP1R16B\GLUL\TEX264\KRT15\DBNDD2\AP1M1\ZDHHC9\LYPLA2\USP20\TOR1A\TAF9\RIC8A\IFITM5\TTPAL\TMIGD1\CYP3A7\HVCN1\CD74\C21H1ORF187\SMG7\MPRIP\VGLL4\ACACB\PTGS2\WNT7A\VIPR1\TK2\CDH5\DHRS11\KLHL18\SNX12\SLC30A4\BOK\MMP11\RRAS2\HELZ2\SLC41A3\CXCL14\MNX1\NAP1L4\TTLL9\POFUT1\FANCI\TOLLIP\CDK5RAP1\AZI1\IGF1R\NRP1\ATP13A4\KIF5B\ITGB2\ZFYVE27\NPEPL1\MTMR8\C7ORF26\TRAPPC4\PAM16\MRPS7\SLC39A13\C6H10ORF32\GDI2\ORC5\MDK\CLNS1A\CYR61\SNAP23\LOC431656\RNPS1\GUCY1B3\SLC39A9\C2H7ORF64\C5H15ORF24\SLC38A4\TMEM206\INTS10\VSX2\MMACHC\AHSA1\LOC424620\ADCK1\PPPDE1\BCL2L14\HEBP1\CLEC3B\LOC425756\CCR8\MAD2L1\DLGAP5\PDGFB\ITGA9\KDELR3\UBE2D3\TIMP3\NTRK2\FANCC\CDH20\ZBTB2\LOC100858230\CHUNK-1\MARCH6\SDHA\CLPTM1L\TBL1X\ISG12-2\DNAJC16\SNX9\VPS45\HBS1L\S100A9\SCNN1A\PHC1\MRPL51\COPS7A\ZYX\TNFRSF1A\RABL3\TRAF3IP2\FOCAD\PLIN2\GRAMD1C\ALDOB\IMPG1\CALB1\KLF11\UBE3A\C1H2ORF49\UBAC2\WBP4\KIAA0226L\N4BP2L2\SLC7A1\CWC15\FZD4\PPME1\SEPT6\GSK3B\CHAD\LOC422002\XRCC3\PTGR2\AK6\NDUFA10\TOM1L1\KPNA1\LOC419830\C26H6ORF89\LOC101750010\TMPO\ACSF2\FASN\XIRP1\IRF7\ATP1B4\CORO7\DDX19B\CHRNB4\CACNA1B\C5H11ORF46\ADAR\THY1\GSPT1\TIMMDC1\
P<0.05	gga-miR-30b-5p	ARID4A\BNIP3L\DCAF7\CSNK2A2\IL10\ANKHD1\UBE2L3\PSMD5\ATP6V1B2\UBAC1\JMJD6\SDF4\AGO3\SORD\AP3S1\GTF2H2\CFH\KCTD7\SLC15A4\GK5\NEDD4L\BECN1\MMD\PARN\KIAA1191\TAF8\RAPGEF1\XPR1\AP1M1\DNA2\CSRP3\FAM81A\PNRC2\RNF111\RNF34\KIF4A\CACYBP\FAM214A\PDCD5\RGS9BP\NOL8\RASD1\PEMT\SLC35A3\RHOG\ARHGEF3\ADRBK2\SPPL2A\RAB7A\MYD88\GORASP1\WNT8A\GALNS\DDX46\HTATSF1\UBE2I\LOC422249\SEMA3A\UPB1\NCBP2\BDH1\CABP1\NRP1\RNF10\SYNPR\THOC7\NME1\STX16\MTMR8\SLC9A3R1\NPM3\ATP7A\STK17B\GGA3\TMEM180\HSPD1\GDI2\PHTF2\MCTS1\SENP5\TRABD\PPP3R1\SHOC2\TBC1D8B\ZFP106\EHD3\CRDS2\DCAF4\PPID\SERPINI1\YAF2\SRP14\C5H15ORF29\ZFC3H1\EIF2B2\PPP1R12A\FAAH\BIRC6\SCCPDH\PPPDE1\GOLGA5\ITPK1\MACC1\DOCK7\CBX3\C5H14ORF129\CCNK\DEPDC1\LHX8\IGF2R\IL2\UBN2\KIAA1109\SLC38A6\CAMK2D\FBXO34\THSD7B\IYD\SOUL\KIF13A\TDG\TXNDC5\PARP12\IRF4\MKRN1\PRKDC\DDX11\GALNT1\TDP2\C6ORF115\PTPN2\LOC426296\SGCB\GRK6\EED\KIAA0930\SMC1B\MAN1A2\EDARADD\MLF2\SERINC1\NUS1\ESCO1\ABHD3\WDR1\B4GALT6\ATG5\UBXN2B\GJA5\CPA6\UBE2J1\OTUD6B\ATP6V1C1\PHF20L1\KIAA0196\MYOM2\FER1L6\CALB1\TAF1B\FAM49A\NT5C1B\RHOB\MTMR9\REV1\C1H2ORF49\TNFSF13B\LIG4\

		UBAC2\NUPL1\N6AMT2\MTMR2\TYR\ARL6IP5\LCORL\KIAA1609\C3H2ORF43\CLN8\IMMP1L\CHP1\C5H11orf24\PDDC1\MCM6\EAF2\ADAP1\NADK\WDR82\COL9A1\KDM3A\BMPR1B\LOC100859142\SLC1A2\WIPF1\GATM\EMB\DDX52\DDOST\TRIM59\TBC1D22A\TBCD\ATP2A2\GTF3C5\COG5\DDX19B\COL6A1\TNFSF15\CACNA1B\OCRL\HSF1\HSBP1L1\
P<0.05	gga-miR-30a-5p_R+2_1ss13CT	ARID4A\BNIP3L\NEFM\DCAF7\CSNK2A2\IL10\ANKHD1\SNAP29\UBE2L3\PSMD5\CPSF3L\ATP6V1B2\UBAC1\JMJD6\ORAI2\SDF4\NR5A2\AGO3\SORD\AP3S1\CFH\KCTD7\SLC15A4\GK5\INPP5K\RASA2\NEDD4L\BECN1\MMD\PARN\NFYA\KIAA1191\ESRP2\DNAJC7\TRRAP\RAPGEF1\XPR1\AP1M1\DNA2\CSRP3\FAM81A\PNRC2\RNF111\RNF34\KIF4A\CACYBP\HVCN1\FAM214A\PDCD5\RGS9BP\NOL8\RASD1\PEMT\AP3M1\SLC25A36\SLC35A3\BTBD10\RHOG\ARHGEF3\ADRBK2\ABCD3\SPPL2A\TMEM41B\RAB7A\MYD88\GORASP1\WNT8A\HPRT1\GALNS\DDX46\HTATSF1\CARS\UBE2I\LOC422249\SEMA3A\KIF3B\UPB1\NCBP2\BDH1\PPP1R2\POLR3E\CABP1\NRP1\RNF10\SYNPR\THOC7\NME1\STX16\RAB22A\MTMR8\SUCLG2\NPM3\PUS10\ATP7A\STK17B\GGA3\RNF114\TMEM180\ACTR1A\HSPD1\MOB4\GDI2\PGGT1B\NAPEPLD\LRRC17\PHTF2\MCTS1\SENP5\TRABD\PPP3R1\SHOC2\ADAM22\TBC1D8B\ZFP106\EHD3\GFRA1\CRDS2\DCAF4\PPID\SERPINI1\PLEKHH1\GXYLT1\YAF2\SRP14\C5H15ORF29\DOCK1\ELMOD2\SMARCA5\RAP1B\SRP54\HP1BP3\ZFC3H1\TBC1D15\LARP1B\NAP1L1\EIF2B2\FAAH\BIRC6\CCDC75\SCCPDH\PPPDE1\GOLGA5\AGR2\ITPK1\MACC1\GALNT3\CBX3\C5H14ORF129\CCNK\C3H6ORF120\WDFY3\DEPDC1\LRRC40\LHX8\GGCT\IGF2R\IL2\UBN2\SLC38A6\CYP2D6\POLR3H\XPNPEP3\STARD3NL\SLC35F4\FBXO34\FKBP9\UBE2D3\HECW1\TDRD7\THSD7B\IYD\NOL12\SOUL\KIF13A\TDG\TXNDC5\MKRN1\PRKDC\DDX11\ROPN1L\GALNT1\PRMT8\TDP2\MAPK9\C6ORF115\SLMO1\PTPN2\LOC426296\HBS1L\SGCB\GRK6\EED\KIAA0930\SMC1B\MAN1A2\MLF2\SERINC1\NUS1\ESCO1\ABHD3\RABL3\WDR1\B4GALT6\ATG5\SDCBP\GJA5\CPA6\PTGR1\TMEM129\UBE2J1\SYNCRIP\NT5E\C1H21ORF59\OTUD6B\GCFC1\TP53INP1\ATP6V1C1\PHF20L1\PTP4A1\MYOM2\FER1L6\CALB1\DSCC1\SH3KBP1\TAF1B\GRHL1\FAM49A\NT5C1B\RHOB\MTMR9\REV1\CHST10\C1H2ORF49\TNFSF13B\LIG4\DZIP1\BORA\WDFY2\NUPL1\N6AMT2\TYR\ARL6IP5\LCORL\YWHAZ\KIAA1609\C3H2ORF43\CLN8\IMMP1L\C5H11orf24\MCM6\EAF2\DAB1\DCLRE1A\ADAP1\STX2\CDC42\NADK\C26H6ORF89\WDR82\WWP1\COL9A1\KDM3A\RSPO3\BMPR1B\CDCA7L\LOC100859142\CUL2\SLC1A2\WIPF1\GATM\EMB\DDX52\TRIM59\TBC1D22A\TBCD\ATP2A2\GTF3C5\COG5\NAALADL2\NUCB2\DDX19B\SMAD2\COL6A1\ZNF488\TNFSF15\CACNA1B\OCRL\SLU7\YEATS2\HSBP1L1\
P<0.05	gga-miR-30a-3p_2ss10GA20AT	RFK\ARID4A\ARMC1\ARHGAP25\LOH11CR2A\SPPL2B\PGM2\ACOT7\AP1G1\TERF2IP\CCL4\GAPVD1\EI24\LDLRAP1\C4BPA\APLP2\PRKCZ\WDR45L\CAMKK1\UBE2R2\GABRA1\DGKD\ODZ2\EIF6\SDF4\AMY2A\DYNLRB1\COMT\AGO3\DTWD2\MAPKAPK3\AP3S1\C10ORF58\CDC73\CALCRL\C19H17ORF85\ZC3H15\INPP5K\MED23\NUDT3\ETFA\SUGP2\FECH\MMD\ERLIN2\ASH2L\KIAA1191\SBNO1\KPNA2\PTEN\UBLCP1\CLINT1\LIFR\KRT19\MYPN\PPT1\PNRC2\NADSYN1\RNF111\FAM20B\TWIST2\RCAN3\GOSR1\TBCA\BCL7A\SH3GLB1\ZMYND8\SGPL1\HVCN1\ATXN2\RGS9BP\MAPK6\CPSF4\LOC768735\LSM14A\DRG2\TSEN2\ALKBH2\PLAU\CLTC\INTS2\RRP1B\TRMT13\IBA

		57\NARFL\FRRS1\TADA2A\RHOG\NONO\LOC768701\ADIPOQ\SYNGR3\RNF141\C20H20ORF11\TNFAIP1\SQRDL\SPPL2A\TRAF7\SQSTM1\MTF2\KLHL13\MED31\SH3GL3\MST4\RPS13\FAM122A\CCBL2\ACTR3B\DDT\TBX6\SEC24A\PDPK1\NAP1L4\ANKRD1\KIAA1024\C9H21ORF2\ABCC1\TMEM41A\NCBP2\MARCH5\CHM\PFKP\BDH1\RNF185\RNF10\ITGB1\CLCN5\RAB35\RBM27\ACBD5\RAB22A\CDC23\BTG4\SUCLG2\CDX4\LRRC59\USP12P1\CSTF1\EIF4E3\MAGT1\DCX\TSEN54\UQCR10\LOC424943\AOX2P\RELN\BUB1\ORC2\CFLAR\TBX3\CNTN4\CRNKL1\SLC38A2\ABHD12\COBRA1\CRELD2\NKRF\KNG1\TRDMT1\CUBN\ITGA8\POLR3F\LOC769134\SHOC2\DCUN1D1\SAMD13\FXR1\SRI\SESTD1\SPTLC3\TRMT6\SEC62\ST7\CAPZA2\GLRB\RXFP1\BAG3\ETFDH\EXD2\YAF2\TLK1\RRP15\METTL5\SSB\RAB33B\LPGAT1\NEK2\HHAT\G2E3\BRE\GEMIN2\VLDLR\MMACHC\GPR149\MED12L\FAM98A\FAAH\AHSA1\ALKBH1\RNF11\BIRC6\GTF2A1\DPY30\SCCPDH\GALNT7\MEOX2\LGMN\ITPK1\UNC79\TRA2A\HSD17B11\GLRX5\C3H6ORF120\SERPINH1\CRADD\PLXNC1\LTA4H\ELK3\BIN1\UHRF1BP1L\MKI67IP\C5H14ORF79\PTPLB\UMPS\IL2\CCR8\SNX4\FSHB\TARP\PPP3CA\VPS41\DARS\MMADHC\WDSUB1\TGFBR1\PWP1\RBFA\TMEM263\CTDP1\PRL\ALDH1L2\SLC41A2\C1H12ORF48\SSR1\SERPINB10\KIAA1468\ZBTB2\BPGM\STRAP\TERT\GABBR2\PDK1\VIP\CNOT7\CYB5A\TMX3\MPPE1\SLMO2\KIT\SGCB\NFXL1\ADHFE1\PDS5A\UBE2K\RELL1\EDARADD\ANAPC4\SEPSECS\ING4\ERAP1\EPHA1\KIF2A\LRRC70\SGTB\PAPD4\C6ORF170\METTL16\RABL3\FOCAD\HAUS6\CDH2\PCMTD1\TBC1D23\GNE\HJURP\SDR16C5\SDCBP\BRP44\ABLIM2\ACOX3\FAM193A\PEX2\WHSC2\CTBP1\CYB5R4\CA2\LCA5\SLC17A5\TTC3\FZD6\OXR1\DENND3\SIK1\DDX3X\OC90\FBXO32\CALB1\MELK\GRHL1\ROCK2\FAM49A\CHST10\TNFSF13B\ABHD13\LMO7\BORA\C1H13ORF15\N4BP2L2\UBL3\MTMR2\MID1\POU2F1\PACSIN2\ARHGDIB\COLEC12\PTPN3\MLANA\LOC422426\PTGR2\C5H11orf24\MCM6\DAB1\TANK\GCG\PLEKHA3\SPECC1L\LOC427470\MPZL3\FYB\TJP1\FLNB\RBM6\FAM48A\ERICH6B\ATP6AP2\IL7\GART\SOD1\POU1F1\PIGN\OVAL\ROS1\EGFR\PDLIM5\SGOL1\CHRNA6\HOXA11\ARHGAP15\NPVF\MORC2\CUL2\ARSB\MON2\NELL2\EMB\DDX27\MYO5A\DDX52\TRIM59\TMEM120B\POP4\PELI1\KIAA1328\DDX19B\PTPN22\C5H11ORF46\RABEP1\WRAP73\PDZD11\TIMMDC1\
P<0.05	gga-miR-30a-3p_1ss18GA	RFK\ARID4A\ARHGAP25\LOH11CR2A\SPPL2B\LMNB2\PGM2\ACOT7\ZMAT2\AP1G1\CCL4\GAPVD1\LDLRAP1\C4BPA\APLP2\SNAP29\SSU72\RBM39\WDR45L\UBE2R2\ODZ2\EIF6\SDF4\AMY2A\DYNLRB1\COMT\NCOA4\ACO1\AGO3\MAPKAPK3\CDC73\CALCRL\C19H17ORF85\ZC3H15\MED23\NUDT3\ETFA\SUGP2\FECH\MMD\ERLIN2\REXO4\ASH2L\SUB1\ACSBG1\KIAA1191\SBNO1\SLC12A4\KPNA2\UBLCP1\CLINT1\LIFR\RICTOR\KRT19\MFAP3\FAM114A2\PNRC2\NADSYN1\RNF111\FAM20B\RALGPS2\RCAN3\GOSR1\TBCA\SH3GLB1\SGPL1\HVCN1\RGS9BP\MAPK6\CPSF4\LOC768735\LSM14A\DRG2\TSEN2\ALKBH2\PTGS2\PLAU\INTS2\RRP1B\TRMT13\IBA57\NARFL\FRRS1\TADA2A\NONO\LOC768701\ADIPOQ\RNF141\C20H20ORF11\TNFAIP1\SQRDL\SPPL2A\TRAF7\SQSTM1\MTF2\KLHL13\MED31\SH3GL3\MST4\RPS13\FAM122A\CCBL2\ACTR3B\DDT\SPTY2D1\TBX6\PDPK1\ABHD17C\ANKRD1\C9H21ORF2\ABCC1\TMEM41A\NCBP2\CHM\PFKP\BDH1\ARRDC4\ALDH1A3\RNF10\ITGB1\CLCN5\RAB35\TOB1\RBM27\ACBD5\RAB22A\CDC23\BTG4\SUCLG2\USP12P1\MAGT1\STK1

		7B\DCX\TSEN54\UQCR10\LOC424943\AOX2P\RELN\FBXO21\BUB1\ORC2\CFLAR\TBX3\CNTN4\CRNKL1\SLC38A2\ABHD12\COBRA1\CRELD2\LOC417741\NKRF\KNG1\TRDMT1\CUBN\POLR3F\LOC769134\SHOC2\DCUN1D1\SAMD13\FXR1\SRI\TSPAN12\SESTD1\SPTLC3\TRMT6\SP3\SEC62\ST7\CAPZA2\GLRB\RXFP1\BAG3\EXD2\YAF2\SLC25A12\TLK1\RARRES1\EPRS\RRP15\METTL5\SSB\RAB33B\LPGAT1\NEK2\HHAT\G2E3\BRE\GEMIN2\VLDLR\MMACHC\ZDHHC17\GPR149\VASH1\MED12L\FAM98A\FAAH\AHSA1\ALKBH1\RNF11\BIRC6\BTF3L4\GTF2A1\DPY30\MEMO1\GALNT7\FMN2\MEOX2\LGMN\GOLGA5\ITPK1\ASB5\UNC79\TRA2A\HSD17B11\C3H6ORF120\SERPINH1\DAZL\WDFY3\CRADD\PLXNC1\LTA4H\ELK3\EIF5\DYNC1LI1\BIN1\UHRF1BP1L\MKI67IP\C5H14ORF79\PTPLB\IL2\CCR8\SNX4\FSHB\TARP\PPP3CA\VPS41\DARS\MMADHC\TRIP13\WDSUB1\TGFBR1\PWP1\RBFA\TMEM263\CTDP1\PRL\ALDH1L2\SLC41A2\MYLIP\C1H12ORF48\SSR1\SERPINB10\KIAA1468\ZBTB2\BPGM\STRAP\TERT\GABBR2\CNOT7\CYB5A\MPPE1\KIT\SGCB\SYTL2\NFXL1\ADHFE1\PDS5A\RFC1\RELL1\EDARADD\ANAPC4\SEPSECS\ING4\CD38\ERAP1\EPHA1\KIF2A\LRRC70\SGTB\SREK1\PAPD4\C6ORF170\METTL16\RABL3\FOCAD\HAUS6\CDH2\PCMTD1\TBC1D23\HJURP\SDR16C5\SDCBP\BRP44\GPR89B\ABLIM2\ACOX3\TCEB1\FAM193A\PEX2\WHSC2\JAM2\CYB5R4\CA2\LCA5\SLC17A5\ITSN1\TTC3\OXR1\EMC2\NOV\RAD21\DENND3\SIK1\DDX3X\GSTA3\FBXO32\CALB1\MBOAT2\MELK\GRHL1\ROCK2\LPIN1\FAM49A\CYP39A1\CHST10\TNFSF13B\STK24\SLAIN1\SLAIN1\IRG1\LMO7\BORA\C1H13ORF15\UFM1\N4BP2L2\UBL3\MTMR2\MID1\POU2F1\PACSIN2\UBE2N\COLEC12\C2H6ORF62\PTPN3\MLANA\LOC100859645\SGK1\IMMP1L\LOC395933\LOC422426\PTGR2\C5H11orf24\MCM6\TM4SF1\DAB1\TANK\GCG\PLEKHA3\SPECC1L\LOC427470\FYB\TJP1\NADK\IRAK2\FLNB\RBM6\FAM48A\ATP6AP2\IL7\SOD1\POU1F1\PIGN\OVAL\ROS1\EGFR\PDLIM5\HOXA11\ARHGAP15\NPVF\CDCA7L\MORC2\CUL2\SLC10A7\ARSB\MON2\NELL2\EMB\DDX27\MYO5A\TMEM120B\POP4\PELI1\KIAA1328\PTPN22\C5H11ORF46\RABEP1\WRAP73\SLC9A9\PDZD11\TIMMDC1\
P<0.05	gga-miR-29c-3p_R+1_1ss18GA	TIMM17A\SLC23A2\KDM5B\SPPL2B\STRADA\CNTN2\XKR8\AP1G1\SLC2A8\GJC1\PIK3R5\SAMHD1\SNX19\CCNL2\MAN2C1\DGKD\WWC1\NCOA4\PEX12\ACOX1\PLEKHB2\FAM168B\CNOT1\PAIP2\LZIC\HSD17B7\DUSP19\INPP5K\MMD\DDX49\LOC100857289\SUB1\HSDL1\CRLF3\ERLIN1\SCYL3\UBIAD1\GOLGA7\TAF8\ARRDC2\SKP2\NECAP2\ARPC3\COL6A3\APBA2\CRYBA1\DNA2\MFAP3\PABPC1L\SRSF10\KIF4A\TRMU\FAM20B\BLMH\ANGPTL1\CYP3A4\RGS9BP\GLE1\USP22\FLCN\C17ORF39\LOC769755\ISY1\DPM2\COL11A1\VIPR1\KBTBD5\CHTF18\MRM1\BCMO1\NDUFB10\LPPR5\PNAT10\RHOG\WFDC1\CDX1\C20H20ORF11\YTHDF1\COL7A1\PLS3\MMP11\SMTNL2\PLK1\COL6A2\HPS5\H2AFY\HTATSF1\C12H3ORF19\TRA2B\MAPRE1\CDK5RAP1\ATP5C1\HINFP\NCBP2\PFKP\TLR4\APOA4\RMI2\PCSK6\SYNPR\ARCN1\ITGB2\LOC424919\STX16\RAB22A\DPYSL3\BTG4\STK17B\HN1\DCX\UBE2V1\HSPD1\MOB4\PUS7\ADAMTS7\FGL2\CROT\LRSAM1\PPP1R21\SESTD1\AMDHD2\CHRNA1\C2H7ORF64\GAD1\GPATCH2\C5H15ORF29\COL1A2\INTS7\CAMK1D\DIEXF\MCFD2\GTF2E2\MED12L\ANGEL1\LOC100859230\CASP3\C4H4ORF41\SCIN\AREGB\TARBP1\PGM1\LIN54\SEC31A\PHF10\SERPINH1\SLC44A5

		\GADL1\PPP1R13B\KIF26A\SEC24B\SULT1B1\SNRPD1\RRP7A\NAGA\KIF15\C5H14ORF135\PDGFB\CASP6\LOC420748\WDHD1\RAP1GDS1\PPP1R17\KDELR3\RAB32\MMADHC\MPST\JARID2\TDG\ADAM12\AKR1D1\C1H12ORF35\BPGM\PPIE\LOH12CR1\FAM69C\CYB5A\CEP192\SSPN\PHC1\ANKRD52\RFC1\ZNF384\DEPDC1B\TNFRSF1A\REV3L\WASF1\HAUS6\ZDHHC23\TMEM68\CPA6\ADD1\RNF4\HMGN3\RIPK2\TMEM30A\IFNGR2\CCNE2\PLEKHF2\DSCR3\NOV\TOP1MT\DDX3X\PRIM2\COL21A1\PXDN\ENPP2\KRTCAP3\REPS2\STMN4\COL4A1\COL4A2\SETDB2\SLC25A15\FLT3\CRYL1\FAM76B\CWC15\PPME1\VAPA\VDAC3\MID1\UBE2N\PDGFRL\C5H11orf24\CZH9ORF80\PLA2R1\DNTT\BIRC2\XKR9\TRIB2\COL9A1\CRMP1\CRTAP\NFIA\OAT\ZBTB34\LOC100859627\MYO5A\TRIM59\COL6A1\IREB2\CACNA1B\PSMD13\MAP3K3\
P<0.05	gga-miR-29c-3p_R+1	TIMM17A\KDM5B\SPPL2B\STRADA\CNTN2\XKR8\AP1G1\SLC2A8\GJC1\PIK3R5\SAMHD1\SNX19\CCNL2\MAN2C1\DGKD\WWC1\NCOA4\PEX12\ACOX1\PLEKHB2\FAM168B\CNOT1\PAIP2\LZIC\HSD17B7\DUSP19\INPP5K\MMD\DDX49\LOC100857289\SUB1\HSDL1\CRLF3\ERLIN1\SCYL3\UBIAD1\GOLGA7\TAF8\ARRDC2\SKP2\NECAP2\ARPC3\COL6A3\APBA2\CRYBA1\DNA2\MFAP3\PABPC1L\SRSF10\KIF4A\TRMU\FAM20B\BLMH\ANGPTL1\CYP3A4\RGS9BP\GLE1\USP22\FLCN\C17ORF39\LOC769755\ISY1\DPM2\COL11A1\KBTBD5\CHTF18\MRM1\BCMO1\NDUFB10\LPPR5\PNAT10\RHOG\WFDC1\CDX1\C20H20ORF11\YTHDF1\COL7A1\PLS3\MMP11\SMTNL2\COL6A2\HPS5\H2AFY\HTATSF1\C12H3ORF19\TRA2B\MAPRE1\CDK5RAP1\ATP5C1\NCBP2\PFKP\TLR4\APOA4\RMI2\PCSK6\SYNPR\ARCN1\ITGB2\LOC424919\STX16\RAB22A\DPYSL3\BTG4\STK17B\HN1\DCX\UBE2V1\HSPD1\MOB4\PUS7\ADAMTS7\FGL2\CROT\PPP1R21\SESTD1\AMDHD2\CHRNA1\C2H7ORF64\GAD1\GPATCH2\C5H15ORF29\COL1A2\INTS7\CAMK1D\DIEXF\MCFD2\GTF2E2\MED12L\ANGEL1\LOC100859230\CASP3\C4H4ORF41\SCIN\AREGB\TARBP1\PGM1\LIN54\SEC31A\PHF10\SERPINH1\SLC44A5\GADL1\PPP1R13B\KIF26A\SEC24B\SULT1B1\SNRPD1\RRP7A\NAGA\C5H14ORF135\PDGFB\CASP6\LOC420748\WDHD1\RAP1GDS1\PPP1R17\KDELR3\RAB32\MMADHC\MPST\JARID2\TDG\ADAM12\AKR1D1\C1H12ORF35\BPGM\PPIE\LOH12CR1\FAM69C\CYB5A\CEP192\SSPN\PHC1\ANKRD52\RFC1\ZNF384\DEPDC1B\TNFRSF1A\REV3L\WASF1\HAUS6\ZDHHC23\TMEM68\ZFP92\CPA6\ADD1\RNF4\NT5E\HMGN3\RIPK2\TMEM30A\IFNGR2\CCNE2\PLEKHF2\DSCR3\NOV\TOP1MT\DDX3X\PRIM2\COL21A1\PXDN\ENPP2\KRTCAP3\REPS2\STMN4\COL4A1\COL4A2\SETDB2\SLC25A15\FLT3\CRYL1\FAM76B\CWC15\PPME1\VAPA\VDAC3\MID1\UBE2N\PDGFRL\C5H11orf24\CZH9ORF80\PLA2R1\DNTT\BIRC2\XKR9\TRIB2\COL9A1\CRMP1\CRTAP\NFIA\OAT\ZBTB34\LOC100859627\MYO5A\TRIM59\COL6A1\IREB2\CACNA1B\PSMD13\
P<0.05	gga-miR-29c-3p_L+1R+1_1ss19GA	SLC23A2\KDM5B\SPPL2B\ZNF593\SLC2A8\CCNL2\MAN2C1\ATP6V1B2\DGKD\RERE\DTWD2\EP400\PAIP2\LZIC\HSD17B7\SUB1\HSDL1\SCYL3\UBIAD1\SKP2\SMARCE1\MPHOSPH10\COL6A3\AP1M1\CRYBA1\KDM4B\SRSF10\BLMH\COL18A1\ORC6\MAP2K6\RGS9BP\PSAP\LOC769755\MAP4K4\TRIM37\COL11A1\KBTBD5\CHTF18\BCMO1\WFDC1\TNFAIP1\COL7A1\SLC13A5\RRAS2\CNOT6\SMTNL2\COL6A2\TBX6\HTATSF1\ZMYND11\MAPRE1\EPC1\ATP6V1G1\SVIL\ITGB2\BTG4\LRRC59\SNAPC5\TMEM104\UBE2H\RNF114\SLC39A13\ADAM

		TS7\ORC5\FBXO21\TUBGCP4\OTOR\C6H10ORF118\SESTD1\LCLAT1\SLC39A9\GPATCH2\COL1A2\SCLT1\CASP3\ASB5\TARBP1\PHF10\SERPINH1\TOP2B\SLC44A5\ELK3\GADL1\KIF26A\PTPLB\KIF15\C5H14ORF135\CASP6\WDHD1\DNAL4\PPP1R17\KDELR3\TBC1D7\PLEKHO1\CYP4V2\SNX9\PHC1\RFC1\MRPL51\ZNF384\EPHA1\FAM26E\REV3L\GPR161\GJA5\GPR89B\CPA6\ADD1\SAMSN1\RIPK2\PLEKHF2\PTDSS1\DSCR3\UTP23\DDX3X\MYOM2\PXDN\REPS2\COL4A1\LIG4\SETDB2\RNASEH2B\SLC25A15\POSTN\TYR\PLA2R1\TRIB2\LOC100859627\TRIM59\GTF3C5\COL6A1\IREB2\C5H11ORF46\LIN7B\RCC2\MBD3\
P<0.05	gga-miR-29b-3p_R-1	TIMM17A\SLC23A2\KDM5B\SPPL2B\STRADA\CNTN2\XKR8\AP1G1\SLC2A8\GJC1\PIK3R5\SAMHD1\SNX19\CCNL2\MAN2C1\DGKD\WWC1\NCOA4\PEX12\ACOX1\PLEKHB2\FAM168B\CNOT1\PAIP2\LZIC\HSD17B7\DUSP19\INPP5K\MMD\DDX49\LOC100857289\SUB1\HSDL1\CRLF3\ERLIN1\SCYL3\UBIAD1\GOLGA7\TAF8\ARRDC2\SKP2\NECAP2\ARPC3\COL6A3\APBA2\CRYBA1\DNA2\MFAP3\PABPC1L\SRSF10\KIF4A\TRMU\FAM20B\BLMH\ANGPTL1\CYP3A4\RGS9BP\GLE1\USP22\FLCN\C17ORF39\LOC769755\ISY1\DPM2\COL11A1\VIPR1\KBTBD5\CHTF18\MRM1\BCMO1\NDUFB10\LPPR5\PNAT10\RHOG\WFDC1\CDX1\C20H20ORF11\YTHDF1\COL7A1\PLS3\MMP11\SMTNL2\PLK1\COL6A2\HPS5\H2AFY\HTATSF1\C12H3ORF19\TRA2B\MAPRE1\CDK5RAP1\ATP5C1\HINFP\NCBP2\PFKP\TLR4\APOA4\RMI2\PCSK6\SYNPR\ARCN1\ITGB2\LOC424919\STX16\RAB22A\DPYSL3\BTG4\STK17B\HN1\DCX\UBE2V1\HSPD1\MOB4\PUS7\ADAMTS7\FGL2\CROT\LRSAM1\PPP1R21\SESTD1\AMDHD2\CHRNA1\C2H7ORF64\GAD1\GPATCH2\C5H15ORF29\COL1A2\INTS7\CAMK1D\DIEXF\MCFD2\GTF2E2\MED12L\ANGEL1\LOC100859230\CASP3\C4H4ORF41\SCIN\AREGB\TARBP1\PGM1\LIN54\SEC31A\PHF10\SERPINH1\SLC44A5\GADL1\PPP1R13B\KIF26A\SEC24B\SULT1B1\SNRPD1\RRP7A\NAGA\KIF15\C5H14ORF135\PDGFB\CASP6\LOC420748\WDHD1\RAP1GDS1\PPP1R17\KDELR3\RAB32\MMADHC\MPST\JARID2\TDG\ADAM12\AKR1D1\C1H12ORF35\BPGM\PPIE\LOH12CR1\FAM69C\CYB5A\CEP192\SSPN\PHC1\ANKRD52\RFC1\ZNF384\DEPDC1B\TNFRSF1A\REV3L\WASF1\HAUS6\ZDHHC23\TMEM68\CPA6\ADD1\RNF4\HMGN3\RIPK2\TMEM30A\IFNGR2\CCNE2\PLEKHF2\DSCR3\NOV\TOP1MT\DDX3X\PRIM2\COL21A1\PXDN\ENPP2\KRTCAP3\REPS2\STMN4\COL4A1\COL4A2\SETDB2\SLC25A15\FLT3\CRYL1\FAM76B\CWC15\PPME1\VAPA\VDAC3\MID1\UBE2N\PDGFRL\C5H11orf24\CZH9ORF80\PLA2R1\DNTT\BIRC2\XKR9\TRIB2\COL9A1\CRMP1\CRTAP\NFIA\OAT\ZBTB34\LOC100859627\MYO5A\TRIM59\COL6A1\IREB2\CACNA1B\PSMD13\
P<0.05	gga-miR-29a-5p_L-1R-2	C22H20ORF30\DDX42\C11ORF30\LOC100857165\SEC14L1\CAMSAP1\ODZ2\PRKRIP1\ORAI2\VAV3\PMPCA\PTBP1\STXBP3\C15H22ORF25\TSTD2\KCNMB1\DTWD2\HEMK1\OGDHL\SORD\C10ORF58\MCCC2\RASA2\TRPC4AP\ZFR\SUB1\EIF3I\ZBTB80S\SKP2\TRRAP\C21H1ORF144\COPS8\PNRC2\USP20\FAM20B\TWIST2\RCAN3\TRPV2\TNR\HVCN1\NCOA3\RGS9BP\SEC24C\RRP1B\PTH\ARHGEF3\LOC768701\PIK3AP1\EVI2A\DENND6A\KSR1\CHEK2\GCLM\DCAF12\TMED5\RTEL1\KIF1A\MLL3\GTF2H1\PCBD2\POFUT1\KIF3B\TOLLIP\SMG1\APOOL\PPP1R2\RBM7\REXO2\PTPRG\ATP13A4\ZEB1\GCN1L1\CD3E\FYTTD1\MTMR8\SUCLG2\CDX4\RNF114\HSPD1\KIF23\CELF1\

		SUMF1\NAPB\XRN2\THUMPD3\SUMO1\NDUFS1\C3H20ORF3\IDH1\FXR1\GIPC2\ITGA4\GANC\YPEL5\EHD3\TMEM185A\CRDS2\ATF2\SP3\DPF3\PRDX3\CTSO\MSMO1\C5H15ORF29\ACADSB\SSB\DIEXF\PRPH2\SCFD1\KIF6\BRMS1L\GLIS3\E2F7\PPP2CB\BTF3L4\SCCPDH\THSD7A\TMEM59\TTC4\LRRC72\IGF2BP3\TSNAX\GLRX5\CCNK\EML1\IL12RB2\KERA\NGLY1\UHRF1BP1L\SCYL2\COX18\HEBP1\ATF7IP\POLDIP3\NAGA\CCK\TARP\HNMT\C22ORF28\BLOC1S5\MKRN1\CCDC77\DAP\BID\USP18\ATP13A1\MAP7\STIM2\EDARADD\RFESD\PRRC1\EPHB6\SMCHD1\LPIN2\ZBTB49\EVC\TJP2\SS18\LOC427250\DAZAP1\CLTA\NFKBIZ\SDR16C5\PDZK1\TXN\GCFC1\CHAF1B\FBXO25\PXDN\CALB1\NOL10\HADHB\ASB9\CYFIP1\CHST10\ATP4B\ARHGEF7\KLF5\WBP4\SETDB2\RNASEH2B\SLC46A3\SPDYA\ARL6IP5\KLHL5\RFX8\INVS\PTPN3\EPM2A\DAB1\PPP4R2\ALDH1A2\NADK\C19ORF44\CNBP\GAL9\APP\GOLGA4\RNASET2\NFIA\CD151\DDX27\CNN3\TMEM120B\LOC422308\KIAA1328\TLN2\HEATR3\MYOF\
P<0.05	gga-miR-2954_R+2	RFK\CAMK4\SLC25A46\C11ORF30\ANKHD1\PIK3R5\COX10\ATP2A3\PRKRIP1\ROGDI\SNIP1\SMU1\POR\SRP68\NCBP1\ACO1\LIG3\ATG12\GBA2\INPP5K\APEH\WDR59\SEC11C\RHO\TRAIP\SUB1\UBIAD1\LOC100859426\RICTOR\FAM20B\TTPAL\RALGPS2\RBM22\VPS53\CDH5\CHEK2\HSCB\DCAF12\RTEL1\KIF1A\ABHD17C\KIF3B\RPRD1B\CHM\LOC395991\BDH1\LPP\ZFYVE27\TMEM104\DNAJA3\HN1\UBE2V1\SLC39A13\ANKRD16\NT5C2\NEURL\SDSL\CRY2\FAM188A\TSPAN12\PTTG1IP\DOCK1\NENF\TRAF5\CPM\CRIPT\PTPRF\KANK1\TMED10\LOC100859230\ITPK1\KLHL7\NTPCR\CBX3\CRADD\SRSF11\BRP44L\CNOT10\CCR8\STARD3NL\SLC16A8\TLE1\UBQLN1\ISCA1\MYLIP\TXNRD1\PYROXD1\MED6\SCNN1A\SEPSECS\LOC426626\LYSMD3\RFESD\PRRC1\DEPDC1B\KIF2A\SGTB\NLN\MTX3\PAPD4\SPICE1\ZUFSP\F2RL1\PGM5\UHRF2\TJP2\RORB\PAM\GNE\LOC396454\CHRNB3\TCEB1\SNX30\DNAJC25\PTGR1\PRIM2\MELK\KLF11\MXRA5\HMGB1\MMP13\SPCS2\SYK\FAM175B\FYB\SUGT1\GOLGA4\DNAJA1\LPL\CCDC101\REEP5\SLC1A2\XIRP1\AMACR\GLT25D2\EIF4A2\LIN7B\TUBAL3\GRIN1\PDZD11\RCC2\
P<0.05	gga-miR-22-3p	TIMM17A\C22H20ORF30\KDM5B\NR0B2\IL10\NUDC\C20H20ORF24\WBSCR16\C4BPA\SIRT6\CLTCL1\CAMK1G\PSMD5\SEC14L1\WAPAL\LOC419144\GNB1L\GPSM2\PEX12\MRPL38\H3F3B\LIG3\AP3S1\ENO1\GBAS\SMN\OLFM1\CREB3\ANXA11\ENPP1\ETFA\SERPINF2\ARMC6\SURF6\C26H6ORF130\KIF20A\CRLF3\RILPL1\FAF2\DNAJC21\OPTC\RAPGEF1\NECAP2\OSMR\PLCG1\PRRC2B\TMEM38A\SLC35E1\LRRFIP1\CNOT8\GPR107\MICALL2\PNRC2\SRSF10\TOR1A\BET1L\RIC8A\IFITM5\TTPAL\CPD\OIT3\ASB6\SGPL1\C10ORF54\EPHB2\C8H1ORF27\KCTD10\JMJD4\TAF1\RHOG\ARHGEF3\ST6GAL1\TRPM2\SNX12\TMEM97\BOK\IPO7\C9ORF24\FNBP1L\MLST8\TMED5\MMP11\PITPNM3\DNAJC5\MST4\HPS5\TBX6\C9H21ORF2\KIF3B\OAF\DEPDC5\KIF3A\ARIH2\TLR4\EPC1\ATP13A4\ANKDD1A\PRKAB1\C10H15ORF44\ACBD5\MTMR8\LOC416354\TRAPPC4\NMRAL1\SLC26A4\SPATA2\CEP41\NR1H3\PPIL3\MED13L\THUMPD3\CRNKL1\NET1\SLC38A2\GTF2H4\TYRO3\SHOC2\TASP1\SKIL\PRKCI\MECOM\DUSP10\C2H7ORF64\TMEM229B\ATP6V1D\LXN\TRAF5\IFNG\MDM1\MYST2\KIAA0391\SCLT1\AARS2\EIF2B2\CP\ATPAF1\VIPAR\PDZK1IP1\AREGB\FAM126A\ATG4C\KLHL8\BTG1\AAMP\T\IL8L1\WDR

		91\SPATA5\POLDIP3\KIF15\CCR8\MYOZ2\CAMK2D\FSHB\CDKN3\LRR1\SAV1\DARS\CUL1\MMADHC\ISX\LOC100857286\JARID2\C1H12ORF48\BLOC1S5\AKR1D1\CDH20\YARS2\USP18\BPGM\SYNE1\GALM\VPS45\KDR\BIK\NUP50\PHC1\ANKRD52\TAPBPL\TPI1\CERS3\HORMAD1\F2RL1\C3H6ORF186\WASF1\PLIN2\QTRTD1\ZDHHC23\TBX19\TBC1D23\GJA5\UFL1\RNF4\PTCD3\PGM3\CDH17\TP53INP1\CHAF1B\SLC25A32\RAD21\TSTA3\ICOSLG\PHF20L1\GCM1\PRDX4\MTMR9\MEP1A\INPP4A\SLC7A1\RNF17\DDX10\NAALAD2\POLD3\UCP3\COTL1\VMO1\MID1\CASP2\GTPBP1\UBE2N\FAM49B\MICAL1\SGK1\SLC20A2\PTGR2\INCENP\GCG\C26H6ORF89\HPCAL1\KDM3A\CRMP1\EGFR\TMED8\TMPO\OIP5\GATM\XIRP1\UPF3B\TMEM120B\EXOSC2\DDX19B\FLOT2\FAS\ACLY\TPCN3\LIN7B\PSMD13\YOD1\PDZD11\
P<0.05	gga-miR-223_R+1	SRSF3\ELAVL1\CALN1\SNAP29\ADRA1B\PPIL2\LOC415664\JMJD6\C15H22ORF25\ACO1\NR5A2\AGO3\EP400\ADPRHL2\CDC73\MAT1A\ZC3H15\C26H6ORF106\PHACTR4\SRM\C26H6ORF130\NFYA\SUB1\BSDC1\PPP1R16B\THG1L\DBNDD2\PBLD\BET1L\RNF34\DHRS3\TNFRSF1B\PPP1CC\MAD2L2\PTGES2\RASD1\MTFR1\USP30\C2H3ORF75\RPS6KB1\SASS6\TEAD1\RNF141\TNFAIP1\SPPL2A\ABCG2\ARHGAP17\RRAS2\BTBD1\SMTNL2\ZNF326\MKLN1\POFUT1\KIF3B\ITIH2\RNF185\LRRC28\PDHB\ALDH1A3\VIMP\TK1\YME1L1\RAB22A\LOC416354\STK17B\HN1\HSPD1\CELF1\MOB4\ATG13\CDK15\AHSG\KNG1\ITGA8\TTC14\B3GNT2\LBR\SP3\GUCY1A3\PRKCI\CTSO\BAG3\YAF2\MSMO1\THBS1\C5H15ORF29\ACADSB\DOCK1\LOC422448\ZNF330\HELB\RAB3IP\TMEM184C\CFL2\GLO1\FAM98A\CMPK1\SCCPDH\PPPDE1\FBXO8\STK39\GLRX5\C5H14ORF129\C3H6ORF120\EPYC\KERA\DCN\CINP\TECPR2\BAG5\COX18\SLC38A6\TRANK1\DTX3L\ADH1C\WDSUB1\MARCH7\RBFA\KIF13A\SLC41A2\TXNRD1\HSP90B1\IRF4\SLC37A3\ADCK2\RDX\ZBTB2\DAP\ANP32E\EZR\RSPH3\SLMO1\HBS1L\SGCB\TEC\NUP50\M6PR\STIM2\CHD4\GPR162\MYH10\FGFBP2\ASH1L\SREK1\MTX3\F2RL1\GTF3C6\HAUS6\TJP2\RORB\TBC1D23\GNE\IDUA\ADD1\SYNCRIP\CA2\B3GAT2\PLEKHF2\WRB\SFXN5\SIK1\OC90\ADCY8\WDR67\KLF11\FAM49A\MSGN1\RHOB\PIGA\RAB9A\RGN\C1H2ORF49\ABHD13\BIVM\SPRYD7\TPTE2\MTMR2\NPTX1\RNASEL\KIAA1609\GTPBP1\POT1\SLC7A13\C6ORF192\EPM2A\IL13RA2\RABGAP1L\WDR82\POU1F1\LOC423536\EGFR\CRTAP\DNAJA1\CRIM1\CUL2\EIF5A2\PELI1\LIN7B\LSG1\PLEKHJ1\LEPROTL1\
P<0.05	gga-miR-223_1ss19CT	SRSF3\ELAVL1\CALN1\SNAP29\ADRA1B\PPIL2\LOC415664\JMJD6\C15H22ORF25\ACO1\NR5A2\AGO3\EP400\ADPRHL2\CDC73\MAT1A\ZC3H15\C26H6ORF106\PHACTR4\SRM\C26H6ORF130\NFYA\SUB1\BSDC1\PPP1R16B\THG1L\DBNDD2\PBLD\BET1L\RNF34\DHRS3\TNFRSF1B\MAD2L2\PTGES2\RASD1\MTFR1\USP30\C2H3ORF75\RPS6KB1\SASS6\TEAD1\RNF141\TNFAIP1\SPPL2A\ABCG2\ARHGAP17\RRAS2\BTBD1\SMTNL2\ZNF326\MKLN1\POFUT1\KIF3B\ITIH2\RNF185\LRRC28\PDHB\ALDH1A3\VIMP\TK1\YME1L1\RAB22A\LOC416354\STK17B\HN1\HSPD1\CELF1\MOB4\ATG13\CDK15\AHSG\KNG1\ITGA8\TTC14\B3GNT2\LBR\SP3\GUCY1A3\PRKCI\CTSO\BAG3\YAF2\MSMO1\C5H15ORF29\ACADSB\DOCK1\LOC422448\ZNF330\HELB\RAB3IP\TMEM184C\CFL2\GLO1\FAM98A\CMPK1\SCCPDH\PPPDE1

		\FBXO8\STK39\GLRX5\C5H14ORF129\C3H6ORF120\EPYC\KERA\DCN\CINP\TECPR2\BAG5\COX18\SLC38A6\TRANK1\DTX3L\ADH1C\WDSUB1\MARCH7\RBFA\KIF13A\SLC41A2\TXNRD1\HSP90B1\IRF4\SLC37A3\ADCK2\RDX\ZBTB2\DAP\ANP32E\EZR\RSPH3\SLMO1\HBS1L\SGCB\TEC\NUP50\M6PR\CHD4\GPR162\MYH10\FGFBP2\ASH1L\SREK1\MTX3\F2RL1\GTF3C6\HAUS6\TJP2\RORB\TBC1D23\GNE\IDUA\ADD1\SYNCRIP\CA2\B3GAT2\PLEKHF2\WRB\SFXN5\OC90\ADCY8\WDR67\KLF11\FAM49A\MSGN1\RHOB\PIGA\RAB9A\RGN\C1H2ORF49\BIVM\CLN5\SPRYD7\TPTE2\MTMR2\NPTX1\RNASEL\KIAA1609\GTPBP1\POT1\SLC7A13\C6ORF192\EPM2A\IL13RA2\RABGAP1L\WDR82\POU1F1\LOC423536\EGFR\CRTAP\DNAJA1\CRIM1\CUL2\EIF5A2\PELI1\LIN7B\LSG1\PLEKHJ1\LEPROTL1\
$P<0.05$	gga-miR-223_1ss15AG	SRSF3\ELAVL1\CALN1\SNAP29\ADRA1B\PPIL2\LOC415664\JMJD6\C15H22ORF25\NCOA4\ACO1\NR5A2\AGO3\DTWD2\EP400\ADPRHL2\CDC73\MAT1A\ZC3H15\TFAM\C26H6ORF106\PHACTR4\SRM\CDK1\C26H6ORF130\NFYA\SUB1\BSDC1\PPP1R16B\THG1L\DBNDD2\PBLD\BET1L\RNF34\DHRS3\TNFRSF1B\PPP1CC\MAD2L2\PTGES2\RASD1\MTFR1\USP30\C2H3ORF75\AP3M1\RPS6KB1\SLC25A36\SASS6\TEAD1\RNF141\TNFAIP1\ABCG2\ARHGAP17\RRAS2\BTBD1\SMTNL2\ZNF326\MKLN1\POFUT1\KIF3B\ITIH2\RNF185\LRRC28\CREM\PDHB\ALDH1A3\VIMP\TK1\YME1L1\RAB22A\LOC416354\STK17B\HN1\HSPD1\CELF1\MOB4\PHTF2\ATG13\CDK15\COBRA1\AHSG\KNG1\ITGA8\TTC14\B3GNT2\SP3\GUCY1A3\PRKCI\CTSO\BAG3\YAF2\MSMO1\THBS1\C5H15ORF29\ACADSB\DOCK1\LOC422448\ZNF330\HELB\RAB3IP\TMEM184C\CFL2\GLO1\FAM98A\CMPK1\SCCPDH\PPPDE1\FBXO8\STK39\GLRX5\C5H14ORF129\C3H6ORF120\EPYC\KERA\DCN\CINP\TECPR2\BAG5\COX18\CCNA2\SLC38A6\TRANK1\DTX3L\ADH1C\WDSUB1\MARCH7\RBFA\KIF13A\SLC41A2\TXNRD1\HSP90B1\IRF4\SLC37A3\ADCK2\RDX\ZBTB2\DAP\ANP32E\EZR\RSPH3\SLMO1\HBS1L\SGCB\FAM76A\TEC\NUP50\STIM2\CHD4\GPR162\MYH10\FGFBP2\ASH1L\SREK1\MTX3\F2RL1\GTF3C6\HAUS6\TJP2\RORB\DTNA\TBC1D23\GNE\IDUA\MYBL1\ADD1\SYNCRIP\CA2\B3GAT2\WRB\SFXN5\OC90\ADCY8\WDR67\KLF11\MSGN1\RHOB\PIGA\RAB9A\CD2AP\RGN\C1H2ORF49\BIVM\SPRYD7\TPTE2\MTMR2\NPTX1\RNASEL\KIAA1609\GTPBP1\POT1\SLC7A13\C6ORF192\EPM2A\IL13RA2\RABGAP1L\WDR82\SOD1\POU1F1\LOC423536\EGFR\CRTAP\DNAJA1\CRIM1\CUL2\EIF5A2\PELI1\LIN7B\LSG1\PLEKHJ1\LEPROTL1\
$P<0.05$	gga-miR-222b-5p_R-4	C22H20ORF30\PRNP\BNIP3L\STARD4\NEFM\STRADA\CDC27\CHTF8\TERF2\TBC1D22B\C28H19ORF28\NR0B2\IL10\HES5\WBSCR16\FAM192A\STX8\ADRA1B\RAB14\LOC419409\MAN2C1\SLC22A7\SLC16A1\JMJD6\ST6GALNAC2\PMPCA\NCOA4\PARG\GOLGA3\RAD51D\DTWD2\AP3S1\MATR3\SPSB1\GJA4\LZIC\HSD17B7\TMEM170A\SPHKAP\BKJ\C26H6ORF130\KIF20A\MTMR9LP\KIAA1191\ELL\SFRP1\ANO5\MINA\STX6\CNOT8\GALE\BLMH\TWIST2\ABCA8\ASB6\VPS33A\SH3GLB2\P2RX5\AGTRAP\RGS9BP\USP22\PTGES2\WNT4\COPS3\CEBPG\PEMT\MYEF2\CDC25A\VGLL4\KIAA0355\URM1\DLG5\TOP3A\PTGS2\CDC14A\VIPR1\INTS2\RRP1B\RPUSD1\BOK\AQP8\ABTB1\MCM2\SMTNL2\PLK1\COL6A2\CLMP\HSPA8\KIF3B\OAF\KIF11\TNMD\RNH1\CHM\LIMK2\PITRM1\KDELR2\NRP1\PTPRG\COQ5\CLPX\SPAG9\ABCC3\FYTTD1\BTG4\LOC422319\FOXP1\BCL11A\CHL1\MLLT10\CD82\ACTR1A\SFXN2\COPG2\ADAMTS7\ANKRD16\GDI2\PC

		GF6\ANAPC1\FGL2\PEX16\SLC39A12\NELF\RBM20\IDH1\FKBP15\SPTBN5\AMBP\JAG1\FMR1\MND1\KIAA1715\RGS7\LOC423256\MECOM\CCDC132\METTL5\SLC38A4\NAA15\TRERF1\STRN3\CPSF6\SMAD1\CNOT2\TBC1D15\VSX2\GLIPR1L\MMACHC\NPC2\ZDHHC17\XPO5\TSPAN1\SNCA\GYG1\CMPK1\TMEM48\SCIN\DHCR24\ERO1LB\CYP2J2\DPP4\ITGB6\DAZL\ILF2\MYLK\DDX47\HEBP1\EXOSC7\KIAA1143\SFRP4\AGTPBP1\CZH9ORF3\TMEM263\LOC420849\CDH20\C6ORF97\KDM5A\GABBR2\LDHB\NCLN\TULP3\MTHFD2\MBP\MRPL47\FUCA2\PCYOX1\MAP7\MYB\OTUD3\M6PR\ITFG2\MRPL10\TAPBPL\GPR98\RFESD\ZYX\PHAX\EVC\RPF2\B4GALT3\NFKBIZ\ALCAM\IDUA\GJA5\GPR89B\VCPIP1\JAKMIP1\ADD1\RARS2\PTDSS1\TTC3\WRB\SLC25A32\PRADC1\DENND3\FBXO32\DERL1\LAPTM4A\RHOB\MTMR9\MGAT4A\ARHGEF7\TNFSF13B\LECT1\SETDB2\FRY\SLC46A3\NUPL1\CWF19L2\NAALAD2\LCMT2\UQCRH\SUV39H2\C1H21ORF33\CERK\E2F5\LOC421975\CLN8\IMMP1L\NOA1\INCENP\MCM6\KCNAB1\LOC427470\UBAP1\LOC101749395\POU1F1\WDR35\TTF2\CD4\TAPT1\CHRNA6\HOPX\PSMC3\BPNT1\SLC10A7\GABPB1\EMB\CD151\COL17A1\GTF3C5\PYGB\ZNF488\CYP2H1\LIN7B\PNPLA7\LIMD2\NGEF\APITD1\
P<0.05	gga-miR-222a	RASSF2\BNIP3L\ITGB3\ETS1\DBR1\RABGAP1\SNAP29\RFFL\RUSC2\PAIP2\NUP35\MED23\PHACTR4\ACADL\ETFA\CHRNA7\C28H19ORF10\SIGIRR\RALGPS2\RCAN3\ORC6\TNFRSF1B\CYP3A7\C11H19ORF12\HVCN1\FAM214A\MAD2L2\MAPK6\VGLL4\GPI\PTGS2\BCMO1\SYNGR3\NLK\ARFGAP1\RAP1GAP2\MST4\PIK3C2A\OSBPL5\C14H16ORF42\NOM1\SKP1\TCF7\KIF3B\CDK5RAP1\KLHDC8B\ARL6IP1\ACAP2\ALDH1A3\ITGB1\TMLHE\FYTTD1\MTMR8\SNAPC5\NPM3\MYO1B\SF3A1\TRPC5\CELF1\MOB4\EIF3J\CTDSPL2\C15H12ORF49\SLC38A2\SENP5\ITPKA\DSTN\RSU1\PPP3R1\COMMD5\C4HXORF57\PTTG1IP\ACBD3\SMOC1\MIA3\SLC39A9\MARC2\GXYLT1\SLC25A12\GPATCH2\ANGEL2\TMEM206\PTPRA\LOC422448\RASSF3\MDM1\RAP1B\BRMS1L\NAP1L1\FAM98A\SNW1\BTF3L4\LGMN\GOLGA5\DFNA5\SIPA1L2\COBLL1\MYO10L\SEC24B\ATF7IP\CCNA2\KIF18A\ITGA9\STK17A\SLC41A2\MYLIP\RBM24\GNPTAB\ADCK2\KDM5A\OPTN\RTTN\LOC421049\SGCB\ARFGAP3\RFC1\EDARADD\CD38\MBLAC2\ARSK\GJA1\ZBTB49\NSG1\ABHD17B\PCMTD1\NRG1\CHMP2B\LOC427306\MXD4\UBE2J1\SLC17A5\CHAF1B\SLC25A32\KLHL15\RNASEH1\REPS2\MSL3\ASMT\RASA3\TNFSF13B\RNASEH2B\UFM1\POSTN\ZAR1L\FLT3\CRYL1\DPT\TERF1\DAB1\C2ORF47\SPECC1L\RB1\WWP1\TRIB2\PSMC6\EGFR\IRF2\DGKZ\CUL2\NEK1\FASN\STK40\
P<0.05	gga-miR-216b	PPP6C\C4BPA\TMEM45B\APLP2\CASP9\PRKRIP1\PTBP1\RFFL\OSGEPL1\RIMBP2\TFAM\BRCA1\DDX49\ACSBG1\NECAP2\SMARCE1\MPHOSPH10\CNOT8\YWHAB\HKDC1\TNFRSF1B\SET\GPI\RHBDD1\SLC27A4\SMG8\FAM120A\ADAMTS18\AATF\RPS2\NONO\TPST2\NOM1\ATP12A\POFUT1\ZMYND11\IL13\METTL9\ACOX2\ZEB1\TMPRSS4\SUCLG2\FBXW4\SALL4\PGK1\TRIM8\MOB4\PUS7\CTDSPL2\EDEM1\PEX16\SLC38A2\ABHD12\CYR61\SAMD13\UGP2\TMEM185A\GLRB\CCDC132\MSMO1\GAD1\LOC417800\ACADSB\C5H15ORF41\HHAT\IFNG\RAP1B\EIF2AK2\BTF3L4\ZYG11B\AGA\FBXO8\ANKMY2\ITPK1\C8B\LRRIQ1\HACL1\RPS6KA2\NEDD1\IGF2R\CENPC1\TMC2\TOB2\ITGA9\METAP1\C3H6ORF103\TDRD7\SOUL\MMADHC\FBXO7\RECK\BPGM\SLCO1A2\ST8SIA1\PRMT8\UBA3\ENPP7\EXOC1\IMPA2\NUP5

		0\RELL1\ZCCHC4\FAM172A\MAP3K1\KIF2A\EPHB6\MRPS36\MTX3\F2RL1\TRIM14\ATG5\ARL13B\ACOX3\EPHA7\CTBP1\CHMP3\MMP16\TMEM30A\MAOA\ROCK2\INTS9\TNFSF13B\RBM26\SETDB2\N4BP2L2\NAALAD2\CD47\GTPBP1\TMEM196\IFNGR1\PTGR2\PPP6R3\GCG\ZNHIT6\IRAK2\TMEM123\PIGN\FAM169B\NSA2\EMB\TMEM120B\TBC1D22A\NUCB2\HSPB7\WRAP73\THY1\LOC422654\
P<0.05	gga-miR-21-5p	KCTD9\HMBS\SPPL2B\FAM125B\SYF2\EDC3\INPP5B\ARVCF\RFFL\C10ORF58\INPP5K\LRRC45\SCPEP1\KIAA1191\ESRP2\NFATC3\UBLCP1\LIFR\CALR3\PPM1M\NADSYN1\RNF111\VPS26A\CCNE1\MPRIP\PEMT\USP32\KLHL18\DCAF12\CXCL14\ALDH1A3\SPG21\LOC422305\BCL11A\PTPN1\SNAI1\DDB2\STAG2\MCTS1\CLNS1A\CXORF56\PDCD4\DTD1\UGP2\FXR1\EHD3\RBM25\KRIT1\DYNC1I2\C5H15ORF24\ACADSB\LOC426599\C1GALT1\PROX1\TMEM206\LOC422448\BRE\MBIP\MBNL1\GLCCI1\DHCR24\HOOK1\FAM126A\GLRX5\ARHGAP24\LRRC40\LHX8\TNKS\GRSF1\CLEC3B\RAB32\UBE2D3\MMADHC\RECK\RBFA\SLC15A5\PIK3C2G\PDK1\CYP4V2\EXOC1\MBLAC2\PCMTD1\LOC396120\GPR89B\UFL1\SH3BP2\ZFYVE28\TP53INP1\RAD21\TG\GCM1\GSTA\DERL1\MSGN1\PIGA\COL4A1\LIG4\SPRY2\LECT1\SLC46A3\NUPL1\FZD4\ARL6IP5\GTPBP1\INCENP\ATE1\DCLRE1A\WWP1\POU1F1\TRIB2\TAPT1\NCF4\SLC1A2\NELL2\XIRP1\EIF5A2\PELI1\KIAA1328\CD36\YOD1\STK40\MBL2\
P<0.05	gga-miR-214_L+1R-3_1ss19GA	RFK\EBF2\KRTAP10-4\DHDDS\STRADA\DDX42\PM20D1\FAM72A\DHODH\RASSF5\AP1G1\NUDC\WDTC1\FAM125B\EI24\MTMR4\C4BPA\MAP2K2\MRRF\COX10\UBL7\MECR\RAB14\STMN1\NCAPD3\PPIL2\CAMKK1\RPAIN\UBE2R2\SLC16A1\UQCC\JMJD6\EIF6\PRKRIP1\TEK\AMY2A\TNFRSF18\WAPAL\DYNLRB1\HEXA\PMPCA\GPRIN2\TRMT2A\LOC415325\ZDHHC8\ACOX1\PLEKHB2\RFFL\AGO3\ETF1\TRAPPC3\SPSB3\LIG3\HIBCH\VPRBP\CCT6A\GPR157\LOC423629\UCHL5\DNAJC18\GJA4\PIK3CD\ZNF76\C19H17ORF85\ZC3H15\DNAJC9\TFAM\INPP5K\WDR59\PEX14\TMEM170A\REEP3\TARDBP\JMJD1C\NRG4\RAB24\TADA1L\PARN\DDX49\LOC100857289\MLX\GABARAPL2\C26H6ORF130\NFYA\VAMP4\COMP\CRLF3\UBIAD1\KIAA1191\RHOT1\GOLGA7\ARRDC2\TRRAP\UBLCP1\THG1L\EGFLAM\ANO5\POLB\TMEM38A\SLC35E1\SMARCE1\P2RX4\RBPJL\AP1M1\ITFG1\SLC25A16\FAM114A2\MICALL2\KDM4B\STK4\FAM81A\SRSF10\G3BP1\RNF111\BET1L\RIC8A\SIRT3\HK1\DHRS3\TMIGD1\TNFAIP8L1\CPD\DYX1C1\AP5Z1\SH3GLB2\SGPL1\WIPI2\P2RX5\NPL\TAX1BP3\MAD2L2\CPSF4\BMP15\C14H17ORF103\PSAP\MPRIP\FLCN\MYEF2\OPNP\SHC4\DHX30\CENPI\DKC1\AP3M1\DHX40\FAM96B\WDFY1\AP1S3\MED13\TK2\SLC25A36\HHATL\IBA57\BRCC3\AATF\TADA2A\ARHGEF3\LOC768701\GALNTL4\SLC44A3\CTR9\RNF141\TSC2\ABCD3\SESN2\CHEK2\TNFAIP1\SGSM2\STK25\GCLM\TMEM41B\PAFAH1B1\DR1\ACAA1\MLST8\GDF2\TPRA1\WNT8A\ARFRP1\CDK10\TPX2\ZXDC\DDT\H2AFY\PCBD2\UBE2I\DUSP15\ATP12A\NDE1\HSPA8\SNRPD3\DEPDC5\CBL\ARL6IP1\FAM174B\BDH1\BLNK\LOC100858827\REXO2\KIF21A\COQ5\ATP6V1G1\TK1\TMPRSS4\RAB18\DPP8\STK32A\NIT1\MTMR8\ASB12\EGR1\NPM3\MYH7B\USP12P1\DIXDC1\UBE2H\GGA3\SPATA2\TMEM180\UQCR10\TMEM164\CELF1\COPG2\NR1H3\DDB2\BUB1\MAN2A2\ATG13\STRADB\SUMO1\MAL\PEX6\SMC3\NDUFAF1\GTF2H4\PDCD4\TRABD\RPF1\UGP2\COMMD5\EDF1\JAG1\EBNA1BP2\LCLAT1\TASP1\NDUFB5\EHD3\THUMPD2\DEGS1\CA

		V2\SLC39A9\SERPINI1\CCDC132\SLC25A12\SRP14\LOC417800\ZNF650\GPATCH2\C5H15ORF29\ACADSB\RSRC1\DYNC1I1\ASNS\HCLS1\IFNG\G2E3\PRPH2\SLC35B1\DYNC2LI1\SMAD1\BAZ1A\CNOT2\FAM177A1\C4H8ORF40\CCDC167\MIA2\TBC1D15\FAM161B\VSX2\NPC2\PPP2CB\MME\POLR1C\FAM98A\ALKBH1\PAPSS1\EPS15\GTF2A1\UFSP2\ZYG11B\PPPDE1\TMEM59\CPSF2\ITPK1\EPGN\CBX3\SNX10\VRK1\ITGB6\PKDCC\WARS\KERA\RAB5A\NKIRAS1\NDUFA12\CTH\CMTM7\BAG5\BIN1\SDAD1\ILF2\COX18\PTPLB\MTHFD1\ESR2\IL2\UBN2\RRP7A\SNAPC1\CLEC3B\SLC38A6\C22ORF32\CTNNB1\DHRS7\TOB2\DTX3L\RAP1GDS1\KLHDC1\MAFF\STK17A\HNMT\ERO1L\CYTH4\NAA35\RBFA\TDG\C1H12ORF48\PMCH\BLOC1S5\TXNDC5\SSR1\WRNIP1\VPS4B\YARS2\MYO10\BPGM\CLPTM1L\EXOC3\PLEKHO1\LOH12CR1\STX17\SYNE1\TDP2\MBP\CNOT7\CYB5A\PPAT\SLMO1\MVK\DCUN1D4\FGFR1OP2\PPFIBP1\BIK\PRR5\KIAA0930\EDARADD\PI4K2B\TAPBPL\MRPL51\LOC431003\CHD5\FGFBP2\CADM1\EPHA1\DEPDC1B\KIF2A\TRIM23\CZH5ORF44\SGTB\SREK1\MTX3\C3H6ORF174\TRMT11\CEP85L\FAM26E\NT5DC1\HDAC2\LYAR\JAK2\FAM189A2\QTRTD1\DTNA\C3H6ORF203\CLTA\ATP5I\ATG5\KCMF1\HACE1\UBXN2B\USP45\VCPIP1\CCNH\NOP14\HSPA13\SH3BP2\RNF4\RWDD2B\SNX14\SCAF4\CA2\ATP6V0D2\MMP16\TMEM30A\CRYZL1\SLC25A32\TSTA3\PSCA\PDE9A\SIK1\ST3GAL1\PHF20L1\DMD\ASAP1\FBXO9\LOC396380\KLHL15\FER1L6\PXDN\RNASEH1\SNTB1\FAM49A\UBXN2A\SF3B14\ACE2\INTS9\MTMR9\CYP39A1\CD2AP\UBE3A\EIF5B\CARS2\ABHD13\DNAJC3\WBP4\LCP1\RCBTB1\ZAR1L\SLC46A3\CEP57\PPME1\LCMT2\C11H16ORF87\VAPA\SUV39H2\RNASEL\LOC426128\YWHAZ\LYN\CCDC82\ITM2B\WASH1\UBE2N\FAM49B\E2F5\INVS\ETV1\HSF2\C6ORF192\IMMP1L\LOC395933\ST3GAL5\SLC20A2\KCNAB1\EAF2\DAB1\HABP2\KLF1\PLEKHA3\MFI2\RABGAP1L\SUMO3\RPE\RNF7\LOC415708\CDC42\LOC419500\TCEB3\PUM1\VAMP3\C26H6ORF89\C19ORF44\IRAK2\CDC5L\SOD1\APP\POU1F1\WDR35\TTF2\MRPS26\EGFR\NT5C3A\PCMT1\PDLIM5\TGFB3\RIF1\RAD54L\SSTR2\CUL2\LOC416916\DHX38\DDX52\ARF1\GTF3C5\COG5\POP4\PELI1\HSPB7\ACLY\TPCN3\LIN7B\C5ORF5\TUBAL3\CR1L\OCRL\TACC1\SUPV3L1\SNCB\SNRNP40\LOC422654\
P<0.05	gga-miR-2131-3p_R+1	BNIP3L\WDR36\HMBS\RAB14\PSMD5\SLC16A1\PTBP1\COMT\POLR2D\GPSM2\PEX12\COMMD10\MPP3\BRCA1\RASA2\RILPL1\UBIAD1\DNAJC7\DDX5\SLC6A2\LPCAT2\COPS8\FAM189A1\PHKB\FAM114A2\YWHAB\DHRS3\SH3GLB2\ATXN2\PPIF\OPNP\VGLL4\DKC1\RRP1B\PTH\WFDC1\COL9A3\DENND6A\MYD88\KLHL13\GINS1\NAP1L4\SORL1\ABCC1\PLOD2\RPRD1B\AP2A2\RMI2\KMO\KDM3B\EME1\USP12P1\CD44\IL18\AAGAB\EIF3J\CASC4\NEURL\F2\ANKRD12\ITPKA\CROT\GANC\SP3\KRIT1\GLRB\CAV2\MECOM\LOC424998\FNIP2\YAF2\IRAK4\C1GALT1\RASSF3\HAAO\SMAD1\TCTE1\MRPS18A\YIPF4\SLC25A4\GPX7\SCIN\IL6\FAM126A\SERPINA5\PGM1\LIN54\MYO10L\KERA\BTG1\ELK3\ATP2C1\ATF7IP\SEPT7\FSHB\GMFB\UBE2D3\IYD\ISCA1\FANCC\TXNRD1\SERPINB10\SLCO1B1\PDLIM3\SYNE1\FBXO5\MAP7\FAM76A\ARNTL2\STIM2\MYH10\MEF2BNB\THBS4\RPL37\NSG1\PCMTD1\TBC1D23\CD247\USP45\LRPAP1\ADD1\WHSC2\CHODL\LTN1\MMP16\ASRGL1\DDX3X\DSCC1\MSGN1\RAB9A\INPP4A\UFM1\SUV39H2\EPM2A\NKAP\MCM6\ATE1\GCOM1\NPPB\N

		ADK\RBM6\KDM3A\TBP\REEP5\MYSM1\TMPO\CUL2\MYO5A\TRIM59\ATP2A2\ACTG1\RABEP1\PLAC9\
P<0.05	gga-miR-20b-3p_R-1	CEPT1\ADAMTS19\KCTD9\ADAM28\HNRNPM\GALK2\CCDC47\C11H16ORF80\TPRG1L\TMEM57\SNAP29\RAB14\PLAA\PMPCA\PTBP1\AP3B2\FBXW11\AP3S1\RUSC2\C19H17ORF85\INPP5K\NCKAP1\PACS2\MMD\NFYA\CRLF3\TMEM39B\AIMP2\CDK2AP1\ATAD1\PSMD12\SALL1\MINA\MFAP3\MICALL2\ADA\TRMU\C28H19ORF10\ANGPTL1\P4HA1\TNFRSF1B\RBM22\RGL1\STAU1\VPS53\LSM14A\TSEN2\AP3M1\WDFY1\CKLF\VCAM1\RRP1B\LOC416530\BTBD10\ARHGEF3\RNF141\C20H20ORF11\SPPL2A\E4F1\MTF2\PDE8A\COL6A2\CEP19\SLC6A6\C12H3ORF19\DNAJB6\TSSC4\GRM3\PDE6C\MARCH5\APOOL\KDELR2\GDF9\PGS1\ARHGAP12\MPZL2\RAB18\TUBB1\NPEPL1\GLS\FYTTD1\MTMR8\CDC23\PPFIA1\HN1\SF3B1\TMEM164\MOB4\PUS7\GDI2\DDB2\FGL2\F2\MPP4\TRH\MCTS1\ABHD12\TUBGCP4\CYR61\PPP3R1\SESTD1\YPEL5\ATP6V0C\LEFTY2\TP53BP2\CRAMP1L\CTSO\TMEM229B\NMD3\YAF2\KLHL2\RARRES1\LXN\LOC422448\STXBP6\RAP1B\CPSF6\CFL2\MMACHC\TIPARP\ABCC10\PAPSS1\GTF2A1\SLC30A6\FBXO8\SCIN\LGMN\MTR\MACC1\RFTN1\LUM\RAB5A\DCN\CRADD\AAMP\STT3B\T\PTPLB\MYLK\TMEM108\KIAA1143\FSHB\CASP6\ADH1C\ATL1\WDSUB1\LOC101750080\TDG\ADAM12\ELOVL2\STRAP\DERA\PIK3C2G\C18ORF21\CLPTM1L\SORBS2\STX17\ANP32E\FAM76A\PPFIBP1\TTLL12\UPK3A\AK3\MYH10\PRRC1\PTPRK\PAPD4\RABL3\RIOK3\B4GALT3\ZDHHC23\TIPRL\TXNL4B\IDUA\CADM2\ACOX3\SH3BP2\MXD4\WHSC2\MMP16\IMPG1\CCNE2\PDE9A\SIK1\MAOA\NR0B1\GSTA\FER1L6\MBOAT2\TAF2\PIGA\MTMR9\RP2\ATP4B\LMO7\BORA\ELF1\ATP7B\N4BP2L2\HMGB1\LCORL\KLHL5\FAM125A\C1H21ORF33\PACSIN2\UBE2N\RPRD1A\LOC395933\PPP1CB\PPP6R3\VTG1\PPP4R2\RABGAP1L\NDUFA10\KPNA1\LOC419404\TMEM131\WDR35\TRIB2\DYNC1H1\RPS19BP1\IRF2\CUL2\GARNL3\SLC24A5\ARPP19\CD72\BFSP1\COG5\CYP2H1\P4HA1\OAZ1\LOC100859100\LOC422654\
P<0.05	gga-miR-205b	C22H20ORF30\PCNX\HMGN2\LOC426699\BTN1A1\PAK1\PPP1R8\SRPR\ARHGEF16\PRKCZ\EDC3\NCAPD3\ACER1\ACSBG2\ORAI2\VAV3\SRP68\NCOA4\LOC415324\NR5A2\TMEM194B\HIBCH\ATG12\ULK1\CALCRL\RCN2\BNIP1\TARDBP\LITAF\KIF20A\NFYA\SUB1\UBIAD1\HERC1\CLINT1\KRT222\ITM2A\RALGPS2\TNFRSF1B\TNFRSF8\PLOD1\ZMYND8\PPP1CC\ZER1\TMOD3\ULK2\MYEF2\DLG5\NUP210\DHX40\SOX8\CDH11\IBA57\JMJD4\TADA2A\HPS4\XBP1\IPO7\TMED5\GORASP1\K123\C9H21ORF2\VIPR2\PDE6C\BRSK2\MARCH5\PIK3IP1\ALDH1A3\VIMP\RAB3IL1\CLCN5\TMLHE\ACBD5\VAPB\RAB22A\SNAPC5\EIF4E3\MAGT1\MRPS7\TMEM180\CHRDL1\NDUFS3\CELF1\RINT1\PUS7\ORC5\ATG4A\EDEM1\CRY2\ZFYVE19\TUBGCP4\PTK7\C3H20ORF3\KIF16B\LIN9\C3H1ORF55\CTSO\GLRB\CAV2\DUSP10\SEC23IP\TMEM229B\SLC25A12\RAB33B\XPOT\IFNG\DYNC2LI1\CRIPT\AARS2\MMACHC\HERC3\LOC395824\CYP4B1\SNW1\RAB3B\BTF3L4\ACSL1\PPPDE1\GALNT7\TTC4\MEOX2\ANKMY2\STK31\BRP44L\PPP1R13B\SCYL2\HSPBAP1\L3MBTL2\CAMK2D\ANK2\TXNDC16\CYTH4\WDSUB1\RBFA\LOC100857286\KIF13A\IRF4\CDH20\MCM4\DAP\ERP44\CYP4V2\FRG1\MAP7\FGFR1OP2\C2H8orf46\PPFIBP1\OTUD3\ATP10D\UPK3A\PDS5A\ING4\PPWD1\MRPS36\HDDC2\TNFRSF1A\EVC\TMEM68\GNE\TMEM175\IDUA\USP45\GPR89B\S

		NX30\ABCC13\WHSC2\SYNCRIP\LCA5\TP53INP1\SLC25A32\PHF20L1\MYOM2\PXDN\DERL1\ROCK2\MTMR9\RP2\MGAT4A\ADPRHL1\TNFSF13B\UBAC2\STK24\DNAJC3\GPALPP1\SLC46A3\ARL6IP5\VDAC3\PSMG1\GTPBP1\UBE2N\E2F5\C2H6ORF62\RPRD1A\LOC421975\EPM2A\AKAP14\AK6\TANK\MCCC1\NDUFA10\GLT1D1\CDC42\TCEB3\NADK\TRIB2\OVAL\ACSF2\SLC1A2\PGRMC1\TBC1D22A\GTF3C5\LMAN1\USP7\RAD50\YOD1\PDZD11\
P<0.05	gga-miR-205a_R+1	C22H20ORF30\PCNX\HMGN2\LOC426699\BTN1A1\PAK1\PPP1R8\SRPR\ARHGEF16\PRKCZ\EDC3\NCAPD3\ACER1\ACSBG2\ORAI2\VAV3\SRP68\NCOA4\LOC415324\NR5A2\TMEM194B\HIBCH\ATG12\ULK1\CALCRL\RCN2\BNIP1\TARDBP\SLC16A9\LITAF\C26H6ORF130\KIF20A\NFYA\SUB1\UBIAD1\ACSS2\HERC1\CLINT1\KRT222\ITM2A\RALGPS2\TNFRSF1B\TNFRSF8\PLOD1\ZMYND8\PPP1CC\ZER1\TMOD3\ULK2\MYEF2\FBN1\DLG5\AP3M1\NUP210\DHX40\CLTC\SOX8\CDH11\IBA57\JMJD4\TADA2A\HPS4\XBP1\IPO7\TMED5\GORASP1\K123\C9H21ORF2\VIPR2\PDE6C\BRSK2\MARCH5\ALDH1A3\VIMP\RAB3IL1\CLCN5\ZEB1\TMLHE\ACBD5\VAPB\RAB22A\SNAPC5\EIF4E3\MAGT1\MRPS7\TMEM180\CHRDL1\NDUFS3\CELF1\RINT1\PUS7\ORC5\COL4A6\ATG4A\EDEM1\CRY2\ZFYVE19\TUBGCP4\PTK7\C3H20ORF3\KIF16B\LIN9\C3H1ORF55\TP53BP2\CTSO\GLRB\CAV2\DUSP10\SEC23IP\TMEM229B\SLC25A12\RAB33B\XPOT\IFNG\DYNC2LI1\CRIPT\AARS2\MMACHC\HERC3\LOC395824\SNW1\RAB3B\BTF3L4\ACSL1\PPPDE1\GALNT7\SCIN\TTC4\MEOX2\ANKMY2\STK31\CHN2\BRP44L\SHROOM3\PPP1R13B\SCYL2\ARL1\HSPBAP1\ATF7IP\L3MBTL2\CAMK2D\ANK2\TXNDC16\CYTH4\WDSUB1\MARCH7\RBFA\LOC100857286\KIF13A\IRF4\CDH20\MCM4\DAP\ERP44\CYP4V2\FRG1\MAP7\LOC421049\FGFR1OP2\C2H8orf46\PPFIBP1\OTUD3\ATP10D\UPK3A\PDS5A\ING4\PPWD1\MRPS36\HDDC2\TNFRSF1A\EVC\WASF1\SS18\TMEM68\GNE\TMEM175\IDUA\USP45\GPR89B\SNX30\ABCC13\WHSC2\SYNCRIP\LCA5\TP53INP1\SLC25A32\PHF20L1\MYOM2\PXDN\DERL1\ROCK2\ITSN2\MTMR9\RP2\MGAT4A\ADPRHL1\TNFSF13B\UBAC2\STK24\DNAJC3\GPALPP1\SLC46A3\ODZ4\ARL6IP5\VDAC3\B4GALT1\GTPBP1\UBE2N\MRPL13\E2F5\C2H6ORF62\RPRD1A\LOC421975\EPM2A\AKAP14\CHP1\AK6\TANK\MCCC1\NDUFA10\GLT1D1\CDC42\TCEB3\NADK\TRIB2\OVAL\CRMP1\TAPT1\ACSF2\SLC1A2\PGRMC1\TBC1D22A\TBCD\LRRC31\GTF3C5\LMAN1\USP7\RAD50\YOD1\PDZD11\
P<0.05	gga-miR-200b-5p_L+2	HNRNPR\NDUFA2\SYF2\PRKCZ\SNAP29\MECR\RASA2\TARDBP\SRM\KIF20A\SCYL3\UBIAD1\SBNO1\GOLGA7\HMGCL\STK4\GOSR1\TAX1BP3\DRG2\AP3M1\RRP1B\LPPR5\PNAT10\SBF2\ARFGAP1\PAFAH1B1\TMED5\MST4\MAPK8\DNAJB6\HSPA14\MARCH5\ITGB1\TOB1\YIPF5\YME1L1\CDX4\SALL4\CD82\TMEM164\PLCL1\C6H10ORF32\CUL4B\SRI\FZD1\ACBD3\SP3\PRKCI\IFRD1\YAF2\GPATCH2\C1GALT1\RASSF3\PRPH2\RAB3IP\MCFD2\PPP2R3C\KIAA0020\MBNL1\RNF11\UFSP2\NUDT9\CAPN9\DAZL\ZRANB2\ERI1\XRCC5\IGF2R\SEC22A\DDX47\HEBP1\ARPP21\SLC35F4\NHEDC2\RBFA\C1H12ORF48\BLOC1S5\DROSHA\PCYOX1\ATN1\ARSK\RFESD\LRRC70\FAM184A\EVC\SS18\QTRTD1\ZFAND5\ARMC2\TMEM175\USP45\MAP3K7\CCNH\SYNCRIP\RAD54B\IFNGR2\MORC3\TTC3\EEF1D\DST\FBXO32\ITSN2\PIGA\INPP4A\C1H2ORF49\TNFSF13B\UFM1\HMGB1\POLD3\SUV39H2\CD9\CERK\TERF1\RPRD1A\HIGD1A\NKAP\HABP2\FYB\R

		RN3\GLT1D1\HMGCL\LOC419404\CNBP\ATP6AP2\GAL8\FAM169B\AMACR\LIN7B\C5ORF5\
P<0.05	gga-miR-196-5p_R+2	CAMK4\RANBP3\FAM125B\LRIG2\VAV3\NCOA4\TMEM194B\ERGIC1\KPNA2\C21H1ORF144\OSMR\COL6A3\SRSF10\RALGPS2\BUD31\LOC769755\PTGS2\FAM96B\BICD2\C17ORF78\RNF141\RPAP2\KIAA0753\SLC9A6\CXCL14\C14H16ORF42\ZMYND11\SVIL\CDX4\NAT9\PEX13\CNTN6\CD82\LOC416735\LBH\ADCK3\UBR1\KRIT1\COL1A2\TRERF1\MCFD2\GLO1\FAM98A\ADCK1\ELAVL4\BIRC6\GTF2A1\ICA1\FBXO8\PPAP2B\IGF2BP3\LEPROT\DAZL\PPP1R13B\ANXA5\PRRG4\AVL9\ADH1C\EPC2\NMI\PHACTR1\ESR1\LOC418170\SLC34A2\UHRF2\LNP1\TFG\EPHA7\RARS2\SNAP91\IMPG1\OTUD6B\FZD6\U2AF1\CASK\PRIM2\PIGA\ELF1\MMP13\DDI2\MBNL2\GATA4\UBE2CBP\DCLRE1A\LOC415708\GLT1D1\TMEM123\GYPC\PGRMC1\GCSH\YOD1\UBE4A\TMEM130\
P<0.05	gga-miR-181b-1-3p_L+2R-2	SPR\PRNP\RDM1\GALK2\RAB11B\TMEM57\CIAPIN1\SLC25A1\SNAP29\MAN2C1\PSMD5\TNFRSF18\PMPCA\FBXW11\TMEM194B\GBAS\GTF2H2\SMN\MATR3\PAIP2\KCTD7\ZNF76\PDXDC1\FECH\C17H9ORF96\SCPEP1\KIF20A\GBGT1\GOLGA7\TSC1\SKP2\YARS\DHTKD1\GLUL\HEATR2\POLB\AP1M1\TAF9\FAM20B\EIF3B\VPS35\DYX1C1\NPPC\C21H1ORF187\RGS9BP\MAPK6\CPSF4\CLIC2\PSAP\LSM14A\DUT\TAF4\ACSL3\INTS2\RRP1B\PTH\JMJD4\SYNGR3\SNX12\C20H20ORF11\LOC422214\IRF8\WHDC1\C5H11ORF58\GALNS\GSTT1\UNKL\FANCI\ARHGEF12\LOC419755\KIF3A\APOOL\METTL9\BUD13\PGS1\ERGIC2\ATP6V1G1\SPG21\CLPX\RAB35\FGF1\PRKAB1\STX16\RAB22A\KIAA0226\SNAPC5\EIF4E3\KAZALD1\BCO2\AAGAB\STK17B\SLC26A4\TMEM180\HSPD1\CEP41\PUS7\NR1H3\CKAP5\TRH\GTF2H4\TYRO3\CD28\SAMD13\ADAM22\C4HXORF57\YPEL5\LIN9\C3H1ORF55\OLA1\SKIL\PRKCI\PDGFC\YAF2\C5H15ORF29\C1GALT1\RAB33B\LOC422442\LPGAT1\TRAF5\PRPH2\BRMS1L\TRAPPC6B\VSX2\GLIPR1L\GPR149\POLR1C\HERC3\ALKBH1\LEF1\DCTD\DHCR24\PPAP2B\SNX10\ELK3\BRP44L\XRCC5\AMPH\MTHFD1\ESR2\LZTFL1\SPATA5\TTC26\LUC7L2\HIF1A\CLEC3B\C22ORF32\PCDP1\METAP1\STK17A\TXNDC16\SOUL\MMADHC\MB\KCNG2\DEK\NUP153\WRNIP1\BPGM\VWC2\CCDC127\CLUAP1\UBA3\CTRC\SGCB\PLA2G2E\NUP50\APBB2\GAPDH\MYH10\GPR98\ZYX\SERINC1\FAM26E\SS18\TIPRL\CPA6\HAPLN1\SYNCRIP\ELOVL4\CPNE3\IMPG1\IFNAR1\IFNGR2\U2AF1\TG\POLA1\SNTB1\SH3KBP1\LOC421978\ASXL2\REPS2\PIGA\MUT\C1H2ORF49\KIAA0564\FLT3\MTMR2\NAALAD2\PPME1\GDPD5\LOC426128\ALG8\LOC101751832\UBE2N\PTGR2\CHP1\AK6\PLA2R1\ARHGAP19\CHMP1A\ALDH1A2\FAM48A\POU1F1\RALBP1\EGFR\AKT1\RIF1\RAC2\RAD54L\TMEM154\CUL2\SLC1A2\ARSB\RPL35\FASN\HNF4beta\TBC1D22A\LRRC31\ATP2A2\GTF3C5\COG5\DDX19B\MYOF\LIN7B\GRIN1\THY1\SNCB\ATCAY\
P<0.05	gga-miR-181b-1-3p_L+1R-1	C22H20ORF30\BNIP3L\SPPL2B\GALK2\PER3\RAB11B\LOC100857226\NEK6\RABGAP1\CLTCL1\LOC428335\NCAPD3\MAT2B\BBS4\POLR2D\SAP130\EP400\GBAS\GTF2H2\UCHL5\PIK3CD\ZC3H15\HSD17B7\NUP35\SLC16A3\RFNG\RASA2\ERGIC1\FECH\PTP4A2\DDX55\ADAP2\TAF8\LPCAT2\AKTIP\FEN1\BPTF\HEATR2\PPM1M\MYOM3\ADA\DHRS3\RCAN3\VPS35\RSRC2\DCTN4\NPPC\SHISA5\TMOD3\CPSF4\PEPD\VIPR1\RRP1B\CYB5R2\SASS6\HELLS\ARHGEF3\TM9SF3\SYNGR3\RNF141\ABCD3\DENND6A\SNX12\C20H20ORF11\TNFAI

P<0.05	gga-miR-181a-5p	P1\TMED5\MYD88\KLHL13\MOSPD1\GALNS\ACTR3B\GSTT1\UNKL\MYH11\TNNT3\TOLLIP\LOC419755\RPRD1B\AP2A2\KIF3A\RNF213\TLR4\KIFAP3\ATP6V1G1\SPG21\CLPX\EDNRB2\NPEPL1\VAPB\RAB22A\RAB11A\LOC416354\USP12P1\NMRAL1\MAGT1\BCO2\ANKRD44\MRPS7\SPATA2\HSPD1\PUS7\FBXO18\SUMO1\TRH\MCTS1\POLR2H\RPF1\SAMD13\MDH1\COMMD5\SNAP23\C3H1ORF55\C6H10ORF46\SKIL\PRKCI\ETFDH\SEC23IP\C2H7ORF64\YAF2\GAD1\C5H15ORF29\RAB33B\FAM177A1\SRP54\BRMS1L\LOC422497\GPR149\HERC3\VIPAR\ALKBH1\SCCPDH\DCTD\MIOS\HMGB2\ROR1\MIER1\LRRIQ1\BTG1\EIF5\DYNC1LI1\BIN1\MAP3K4\DDX47\MTHFD1\ESR2\POLDIP3\CLEC3B\ST13\SLC12A8\STARD3NL\PCDP1\MRPL32\TAB2\UBQLN1\ADAM12\ADCK2\SERPINB6\AGK\OVALY\CREB3L2\C6ORF97\CCDC77\BPGM\DDC\SDHA\CLPTM1L\ABCC9\CLUAP1\CTRC\SRSF7\C2H18ORF19\SGCB\NUP50\APBB2\MYH10\ACTBL2\MTX3\RPL37\FAM26E\F2RL1\OTOP1\C3H6ORF186\MAMDC2\NANS\TMEM68\PCNP\QRSL1\ALCAM\PLCXD2\PROS1\SDCBP\GJA5\GRPEL1\CPA6\ATP6AP1L\WHSC2\TMEM129\PMP2\RWDD2B\MMP16\CCNE2\DSCR3\U2AF1\ST3GAL1\MAOA\DDX3X\KLHL15\FER1L6\MCM3\MUT\BORA\CKAP2\N4BP2L2\N6AMT2\SUV39H2\KIAA1609\LYN\SEPT6\PSMG1\CCDC41\UBE2N\E2F5\LOC422106\LOC421975\CENPU\XRCC3\HABP2\MCCC1\CHMP1A\CNBP\PUF60\CRYAA\POU1F1\AKT1\PDLIM5\SLC1A2\GABPB1\EMB\CD36\LOC419851\GRIN1\HSF1\MBD3\HSBP1L1\LOH11CR2A\RASSF2\LSM7\STRADA\ACOT7\PAK1\FAM125B\CCDC43\ARMC8\SAMHD1\CLTCL1\PI4KA\CNTRL\WDR45L\PSMD5\ATP6V1B2\WWC1\GNB1L\TRIM33\STXBP3\PRKCD\C15H22ORF25\LOC415324\AGO3\GOT2\GBAS\C10ORF58\UCHL5\PIK3CD\LZIC\C19H17ORF85\ATR\ZC3H15\GK5\UHMK1\DNAJC12\C26H6ORF106\RASA2\PHACTR4\TARDBP\SUGP2\MMD\UBIAD1\ACSS2\BARD1\IFT52\LPCAT2\TOP1\LIFR\P2RX4\MYPN\FAM189A1\IQCA1\STK4\PNRC2\SRSF10\RNF111\GOSR1\SGPL1\P2RX5\MAD2L2\PDAP1\RGL1\NOL8\COPS3\DUT\MKRN2\TRIM37\TUBD1\VCAM1\SLC25A36\GNG13\SASS6\APPBP2\ADAMTS18\JMJD4\LPPR5\ARHGEF3\SYNGR3\TSC2\KLHL36\PITPNB\EMC8\SPPL2A\GCLM\RAP1GAP2\MTF2\KLHL13\ABTB1\KIAA0753\MOSPD1\PRKAG2\MLL3\CXCL14\PDPK1\SRPRB\KIAA1024\SEMA3A\KIF3B\FRA10AC1\BRSK2\TOLLIP\TAF3\PLOD2\IL13\CHKA\RPTOR\ARRDC4\ALDH1A3\RAB35\MTPAP\ABCC2\ARCN1\TMLHE\ABCC3\STX16\MTMR8\LOC416354\CHIC1\EME1\SALL4\GGA3\ACSL4\CELF1\MTAP\CASC4\PDCD11\LRRC17\PHTF2\SMARCA1\MCTS1\SENP5\LOC424109\PPP3R1\C1D\IDH1\ATP6AP1\LCLAT1\CDC42BPA\C3H1ORF55\LEFTY2\GUCY1B3\PRDX3\SEC23IP\C2H7ORF64\MARK1\GXYLT1\PUS7L\RARRES1\RRP15\C1GALT1\RAB33B\PROX1\SRGAP1\STXBP6\SCFD1\TMEM184C\CFL2\KIF6\PSMA6\MBIP\KANK1\TBC1D15\NAP1L1\TTLL5\MBNL1\OSTC\ELAVL4\BIRC6\CC2D1B\SCIN\ANKMY2\ITPK1\RAPGEF5\INADL\DOCK7\LYST\PGM1\CBX3\COBLL1\C5H14ORF129\CCNK\C3H6ORF120\RFTN1\DCN\KAT2B\GPD1L\BAG5\KIF26A\CLASP1\UBP1\CAMK2D\SLC12A8\EIF3M\CASP6\ITGA9\STK17A\CTSL2\TGFBR1\LOC101750080\DEK\TXNRD1\EEF1E1\BLOC1S5\STRAP\MGST1\PRMT8\LOC420992\LRRC16A\TFB1M\NPEPPS\PPAT\CEP76\BCLAF1\IGF2BP2\OCIAD1\M6PR\EDARADD\PI4K2B\CCDC58\MBLAC2\HDDC2\TNFRSF1A\FAM26E\LYAR\HAUS6\MAMDC2\GPR161\TCEA1\TMEM68\KIAA1524\CHMP2B\ELOVL4\DECR1\PTDSS1\DOPEY2\ATP6V1C1\NOV\RAD21\DDX3X\OC90\DST\APOO\

		DSCC1\SH3KBP1\PDHA1\TAF1B\LAPTM4A\PIGA\RAB9A\STMN4\RP2\SLC9A7\GAS6\ATP4B\TNFSF13B\IPO5\RBM26\BORA\MZT1\RNASEH2B\GDPD5\TXNL4A\SUV39H2\GZMK\CASP2\PACSIN2\LOC101750282\CERK\E2F5\C2H6ORF62\IFNGR1\SLC25A14\PDDC1\EAF2\PLEKHA3\C2ORF47\SPECC1L\RNF7\PRR11\SLC13A2\C26H6ORF89\C19ORF44\RANBP2\TMEM131\WWP1\APP\MTO1\KDM3A\DNAJA1\HOPX\ATP2B1\SLC10A7\ZBTB34\GATM\PRKAG2\NELL2\GRIA2\AMACR\TBC1D22A\ATP2A2\NAALADL2\NUP188\EIF4A2\CD36\LSG1\EPS8L2\LOC422654\
P<0.05	gga-miR-181a-3p	MGAT4C\RBM39\RPA1\GHDC\DKK1\TLCD1\TOR1A\GOSR1\SLC39A11\LSM14A\ISY1\MPP1\HEATR6\EIF4G2\KLHL3\C9H21ORF2\KIF3A\CLPX\SP3\ARHGAP10\YIPF4\FBXO7\VPS4B\FAM76A\RBP5\HAUS6\PTDSS1\ICOSLG\LYN\NUFIP1\MORC2\XIRP1\EIF4G2\
P<0.05	gga-miR-1796	RFK\LOC420050\NEFM\STRADA\DDX42\BTN1A1\SLC41A1\STX12\XKR8\HNRNPR\HSPA5\FAM118B\SLC25A1\GTF2I\FAM18B1\TTC1\RAP1A\CALCOCO2\ZZEF1\MOV10\WDR45L\NFS1\ANP32B\CCNG1\VAV3\SMU1\NCOA4\PLEKHB2\FAM168B\DTWD2\TSPAN14\MATR3\TAF11\INPP5K\DNAJC12\DFFA\LRRC45\SUGP1\BBS2\EXOSC10\ERLIN2\KIF20A\KHDRBS1\TMED2\UBIAD1\KIAA1191\DNAJC7\DDX5\UBLCP1\HEATR2\USE1\PRRC2B\SYS1\LOC101749801\MFAP3\SRSF10\BET1L\RNF34\P4HA1\CCNE1\HVCN1\NAA25\CEBPG\MTFR1\USP30\TOP3A\NUP210\TAF4\SERPINE2\FUT11\CDC14A\INTS2\TRMT13\APPBP2\USP32\LOC768701\TFIP11\KSR1\SQRDL\LOC422214\SEPT2\TMED5\MYD88\LOC100858792\KLHL13\CLK4\LRRC8B\ILKAP\CRKL\CEP19\DNAJB6\HSPA8\MAPRE1\OAF\YWHAH\CHM\BDH1\HDX\RNF10\VIMP\TMLHE\TUBB1\C7ORF26\SUCLG2\NAT9\TMEM104\APIP\PUS10\PTS\TTC17\UBE2H\CASC4\MED13L\CNTN4\SUMF1\PION\ATG13\NUSAP1\AHSG\TRABD\SHOC2\DCUN1D1\TSPAN12\KIAA1715\ST7\SLC39A9\DUSP10\RAB3GAP2\C5H15ORF29\COL1A2\RSRC1\GPR26\C1GALT1\TMEM206\STXBP6\G2E3\SMAD1\MED8\GLO1\GEMIN2\COQ6\TIPARP\MED12L\CMPK1\SNW1\LOC100859230\EPS15\NRD1\GTF2A1\DCTD\MIOS\CPSF2\ITPK1\AREGB\STK39\GALNT3\HSD17B11\NID1\C5H14ORF129\URB2\COQ2\SERPINH1\CRYZ\SLC44A5\GRSF1\IGF2R\CSTF3\PTPLB\MYLK\ATF7IP\LUC7L2\HIF1A\CTNNB1\CCR8\UGT8\CAMK2D\DTX3L\CCDC93\SAV1\STK17A\LATS1\RMI1\FANCC\RBFA\TDG\ADAM12\SSR1\PRPF4B\RIPK1\AGK\ADIPOR2\LOC418169\CLUAP1\FRG1\PPAT\MRPL47\SGCB\ARHGAP8\KIAA0930\FKBP4\ZNF384\FNBP4\MBLAC2\SMCHD1\LOC421712\GREB1L\CYTL1\TRAF3IP2\WASF1\ABHD17B\ZFAND5\PCMTD1\TBC1D23\TMEM68\QRSL1\KIAA1524\CHRNB3\MAEL\GPR89B\TCEB1\SLBP\LOC100857673\NBN\IFNGR2\PLEKHF2\WRB\DYSF\DERL1\PDHA1\NT5C1B\ASB9\RAB9A\INTS9\C1H2ORF49\TFDP1\MYCBP2\WBP4\DNAJC15\ZC3H13\MPHOSPH8\DDX10\CD9\VMO1\PSMG1\GTPBP1\CERK\SYT4\CENPU\PTGR2\INCENP\PPP6R3\AK6\SYK\FAM175B\DNTT\PLEKHA3\PPP4R2\STOM\NADK\IRAK2\CRISPLD1\HPCAL1\TAPT1\PPARA\SMC2\PDLIM5\LPL\IRF2\SLC1A2\WIPF1\GATM\NELL2\XIRP1\EMB\AMACR\ACTG1\LIN7B\SMAD5\REL\GNB1\DNAJC17\RER1\BUB1B\P4HA1\PLEKHJ1\TIMMDC1\
P<0	gga-miR-1788-	PRNP\RDM1\ERMP1\LAPTM5\WWP2\PPP1R8\RPA2\SLC7A6OS\PIK3R5\PLXNA2\CHD6\THYN1\WDR45L\OPN4\ACO1\RERE\TMEM194B\CO

.05	5p	MMD10\OAZ2\SMN\TAF11\RHO\RASA2\BBS2\KIF20A\TMED2\GOLGA7\PPP1R16B\AP1M1\SLC25A16\FAM114A2\BET1L\MORN3\AGTRAP\SET\FAM129A\LOC768735\COL4A4\TAF4\BICD2\SNX2\BCMO1\SYNRG\MPHOSPH6\TFIP11\ABCD3\C20H20ORF11\HSCB\TMED5\PLS3\GLMN\GPC3\XYLB\SLC9A6\GTF2B\MLL3\FOPNL\OAF\USP28\PTPRG\SUDS3\RAB22A\RAB11A\APIP\PEX13\MAGT1\BCL11A\FANCL\ACTR1A\SLC39A13\C15H12ORF49\BUB1\ORC2\NEURL\FBXL13\F2\PHTF2\XRN2\SUMO1\COBRA1\ADD3\MAL\NDUFS1\ATP6AP1\NHLRC2\NRK\ENTPD2\NRXN1\EIF3A\PRDX3\CAV2\ACADSB\SSB\CENPF\NEK2\VLDLR\CMPK1\QPCT\SCCPDH\ICA1\AREGB\C5H14ORF129\SLC4A10\C3H6ORF120\COQ2\SERPINH1\BRP44L\PECR\BIN1\CAPN7\KIF26A\RALB\HSPBAP1\SYNE2\ITGA9\LATS1\CYTH4\BLOC1S5\SERPINB2\AGK\PIK3C2G\STX17\FRG1\HBS1L\DCUN1D4\FGFR1OP2\PPFIBP1\FBLN1\ATP8A1\APBB2\RFC1\ZNF384\MYH10\MBLAC2\LYSMD3\SPICE1\FAM184A\FAM26E\IDUA\CHRNB3\CD247\DNAJC25\TPD52\OTUD6B\PTDSS1\ITSN1\SIK1\ZFAT\DDX3X\CALB1\PIGA\MCM3\MYO16\NPAT\MMP13\VMO1\TERF1\MCPH1\INCENP\KCNAB1\SPECC1L\LOC396531\CDC42\KPNA1\NADK\AZIN1\TRIB2\POPDC3\PCMT1\TMPO\SLC1A2\ZBTB34\NELL2\ARF1\TMEM120B\GTF3C5\MYOF\LIN7B\LOC419851\GNB1\SLC9A9\PDZD11\MYBPH\
P<0.05	gga-miR-1788-3p_R+1	B-G\GFPT1\CD79B\ITGB3\CHMP7\DCAF7\ZNF276\STRADA\ZCCHC17\CNTN2\LOC415661\LMX1B\FAM54B\PPP6C\SRPR\C20H20ORF24\WBSCR16\CIAPIN1\SNAP29\FAM18B1\COX10\POLRMT\RAB14\LOC419409\PPIL2\BBS4\UQCC\JMJD6\ROGDI\SRP68\PGAM5\SLC7A4\RAD51D\AGO3\NUBP2\GOT2\AP3S1\ULK1\RECQL5\KTN1\PIK3CD\LZIC\C19H17ORF85\DNAJC9\RFWD3\RFNG\SARDH\ETFA\SCPEP1\TSR1\ALDH9A1\TAF8\RBBP4\FNDC5\NECAP2\ANXA2\SMARCE1\APBA2\EMC1\TLCD1\MFAP3\HMGCL\STK4\GPR174\BET1L\RNF34\KIF4A\TRMU\TSPAN15\C28H19ORF10\EIF3B\ANO9\HNF4A\COL13A1\AP3B1\PLOD1\HVCN1\CTNS\C21H1ORF187\MAD2L2\C5H15ORF57\BRAP\GLE1\MAPKAPK5\C14H17ORF103\FAM129A\FLCN\CCNDBP1\CENPI\ADK\AP3M1\SLC25A36\APPBP2\BCMO1\TADA2A\WFDC1\COL9A3\SNX12\GARS\SLC30A4\LOC422214\GDPD2\LOC420419\PASK\CALCA\HPRT1\COL6A2\MORN4\CSNK2A1\KIF1A\CAMLG\ANKRD1\CRTAM\TH\VPS8\NOX1\IDI1\AEN\KIF3A\FAM174B\POLR3E\SUDS3\MASP1\CTSZ\GOT1\LOC424919\STX16\CLCN7\RAB11A\CHIC1\KDM3B\EGR1\NPM3\TMEM104\CBLN4\HN1\ACTR1A\SFXN2\ORC5\TBX5\TPCN1\ABHD12\ITPKA\TYRO3\LOC424109\CTBS\CROT\SAMD13\ATP6AP1\GANC\YPEL5\LCLAT1\MTMR1\SFRP2\LEFTY2\EIF3A\SLC39A9\YAF2\C5H15ORF29\ACADSB\TMEM206\DIEXF\IL22\STRN3\PREPL\ELOVL1\MCFD2\SNX6\SEC23A\GEMIN2\GPR149\ALDH18A1\CC2D1B\SCCPDH\PPPDE1\TTC4\STK31\RAVER2\COBLL1\EVL\WLS\LRRC40\PLXNC1\SLC44A5\TNFAIP2\STT3B\GPD1L\SHROOM3\BAG5\L3MBTL2\KBTBD2\DDX17\MICALL1\ANKRD54\UBE2D3\STK17A\TAB2\C3H6ORF72\CUL1\MMADHC\TGFBR1\TBXAS1\FARS2\MKRN1\SERPINB6\BID\MICAL3\LOH12CR1\MTUS1\DNAJC16\LONRF1\GTF2H5\ENPP7\MED6\VPS45\FAM38B\ITPR2\PPFIBP1\PHC1\GAPDH\CHD4\C15H12orf51\HORMAD1\SGTB\SPAG17\GREB1L\RABL3\ZBTB49\RPF2\ALCAM\TAGLN3\RAB2A\ZFP92\GJA5\UFL1\FAM193A\LOC422887\SLBP\GRIK1\CPNE3\ESRP1\UBR5\TTC3\SLC25A32\LY6E\LOC100858102\ASAP1\KLHL15\DERL1\MSGN1\PHKA2\REPS2\MOSPD2\C2ORF18\MTMR9\MEP1A\LYG2\IL1RL1\DNAJC3\C1H13ORF15\F

		LT3\NUPL1\ACAT1\MED17\TPD52L2\DNASE1\C1H21ORF33\DPT\ARHGDIB\E2F5\DPYSL2\NCOA7\C6ORF192\SUCLG1\PLAC8\AK6\C14H16ORF73\LOC415708\ALDH1A2\HMGCL\POU1F1\LOC416916\GABPB1\CYP24A1\LRRC31\GTF3C5\BFSP1\PDE3B\USP7\PYGB\ACLY\ERBB4\TUBAL3\PNPLA7\TACC1\HSF1\ADAR\TNFRSF10B\
P<0.05	gga-miR-1784-5p	GFPT1\RPS6KA1\LSM7\CCDC15\CAMTA1\SSR2\RASSF5\NUDC\PIGR\FAM125B\CCDC43\TIRAP\GATSL2\FAM192A\MYBBP1A\MECR\GUCA1A\POC1A\NISCH\CALCOCO2\CWC25\DGKD\PRKRIP1\ORAI2\RASA4\FAM86A\POLR2D\CLCC1\ZDHHC8\ACOX1\WBP2\HSPA9\VAC14\RGS4\RAN\DEF6\ZNF76\WDR59\SCAPER\SPHKAP\MLX\DNAJA4\HSDL1\CRLF3\ERLIN1\UBIAD1\KIAA1191\GOLGA7\RBBP4\SGK2\FNDC5\TOP1\PAPSS2\USE1\PABPC4\DKK1\MINA\PIGT\LOC101749801\TARSL2\AKR7A2\MFAP3\INTS1\PABPC1L\TOR1A\ADA\SIRT3\ORC6\ASB6\ANAPC16\DOLPP1\DMGDH\SHISA5\SET\RGL1\PTGES2\COPS3\MED9\CEBPG\SLC12A1\DUT\LOC423740\CDH5\SLC25A36\RRP1B\PTH\RHOG\EIF4G2\SLC44A3\TFIP11\CHEK2\ABHD6\ARFGAP1\CHRNA4\TMEM41B\DR1\MYD88\ABTB1\DOCK11\TPRA1\KLHL3\ERCC6\C12H3ORF19\LOC422249\CRTAM\ZMYND11\KIF3B\EHHADH\DEPDC5\AP2A2\KIF3A\LOC395991\RNF185\SDR42E2\CPN1\PTPRG\LPP\FGF1\MASP1\TMLHE\RAB22A\LOC416354\STAT1\TMEM104\TMEM209\ARL3\CHRDL1\CEP41\SLC39A13\ORC5\TRIM36\FBXW8\RNFT2\ATG4A\SUMF1\MDK\THUMPD3\WDR12\TRH\MCTS1\GTF2H4\TRDMT1\RBM20\NRK\GANC\LBH\EHD3\FSHR\IFT140\CRAMP1L\MECOM\DUSP10\YAF2\IARS2\SCHIP1\IRAK4\C5H15ORF29\ARID2\GPR26\TMEM206\MED8\FAM177A1\BTBD9\CCDC167\TRAPPC6B\AARS2\TBC1D15\MMACHC\ZDHHC17\GTF2E2\MME\FAF1\YIPF4\CASP3\SCP2\TTC7B\SMEK1\SCIN\CHGA\ITPK1\ERO1LB\HSD17B11\DFNA5\ROR1\HNRNPA2B1\SERPINH1\WLS\PPP2R5C\MLLT4\ZRANB2\CRYZ\T\XRCC5\BIN1\ILF2\HEBP1\SPATA5\CLEC3B\CCR8\L3MBTL2\EEPD1\C5H14ORF101\HERPUD2\FBXO34\TARP\EIF3L\CYTH4\CCDC148\RMI1\PWP1\C12ORF45\RANBP9\JARID2\LOC420864\TBXAS1\SLC22A23\RIPK1\IRF4\MKRN1\BCL2\LOH12CR1\TBC1D1\C6H10ORF137\LTV1\MAP7\KIT\PPFIBP1\ARFGAP3\PRR5\FKBP4\ZNF384\MLF2\ATN1\HORMAD1\PAPD4\RABL3\FAM26E\HDAC2\ZBTB49\TRAF3IP2\C3H6ORF186\ST3GAL6\GNE\USP45\GRIN3A\ADD1\SH3BP2\SAMSN1\PNRC1\CD8A\PGM3\CPNE3\OTUD6B\TMEM67\DONSON\CHAF1B\SLC25A32\RAD21\U2AF1\SIK1\PHF20L1\PTP4A1\NR0B1\POLA1\DERL1\IAH1\PIGA\INTS9\INPP4A\C1H2ORF49\NCK2\BIVM\WBP4\UBL3\RNF17\LYN\LOC418472\UBE2N\FZD7\CLN8\IFNGR1\SLC25A14\XRCC3\C5H11orf24\ITGA1\NDUFA10\KPNA1\NADK\LOC101749395\WDR82\TMEM123\PSPC1\PUF60\GAL8\GART\TTF2\COL9A1\CD4\RSPO3\PDLIM5\NEK1\EMB\CD151\GTF3C5\EIF4G2\SF3B3\CYP2H1\TPCN3\REL\RABEP1\THY1\B4GALT7\NUDT1\RPUSD4\PLEKHJ1\
P<0.05	gga-miR-1783_R+1	ARID4A\TRIM39\C22H20ORF30\PSMF1\DPAGT1\PCNX\CSRP1\ADAM28\SPPL2B\LOC395926\RBBP5\KLHDC8A\PPP1R8\FAM72A\FBXW2\KIF18B\IL10\NUDC\ARHGEF16\TIRAP\HES5\WDR16\ATP5G1\UBL7\RALGAPB\GUCA1A\PPIL2\ANP32B\CAMSAP1\ATP6V1B2\DGKD\ACSBG2\ROGDI\SDF4\GNB1L\APTX\SAP130\MRPL38\FHOD1\FBXW11\SEC16A\LOC423629\PIK3CD\COG4\LZIC\ZNF76\TRPC1\PLS1\D

		NAJC9\INPP5K\C26H6ORF106\MAP2\WDR59\DCXR\PINK1\COPE\C26H6ORF130\DNAJA4\TMEM39B\SFRP1\BARD1\SKP2\SLC6A2\FNDC5\TRRAP\LOC100859426\NECAP2\SLC35E1\PABPC4\TRIT1\SMARCE1\PPT1\SRSF10\FAM20B\ASB6\PPTC7\WIPI2\HVCN1\MTHFR\P2RX5\BRAP\GLE1\PTGES2\MPRIP\MTFR1\SLC12A1\URM1\MPP1\NUP210\HMCN1\ANKRD13A\INTS2\SLC25A36\KBTBD5\HAGHL\RHOG\SLC44A3\SPPL2A\STK25\DCAF12\TMEM41B\ACAA1\LOC420419\RAB7A\MYD88\KLHL13\ABTB1\PHF6\MYLK2\GINS1\IL2RA\K123\GSTT1\NAP1L4\ABCC1\VPS8\AEN\CHM\RNF185\PPP1R2\APOA4\ALDH1A3\PTPRG\SYNPR\CLPX\PRKAB1\WAC\CD3E\MMS19\CHIC1\TMEM104\EIF4E3\EIF4E2\TRPC5\UBE2H\NXT2\WSB2\AOX2P\FBXW8\ORC2\NEURL\CASP8\SUMO1\CRY2\EHMT1\DUSP5\ACSS1\TRDMT1\SHOC2\DCUN1D1\CDAN1\CTSO\BAG3\DUSP10\EXD2\CCDC132\DYNC1I1\NAA15\PTPN14\DTL\RAP1B\BRE\GEMIN2\INTU\MMACHC\TMED10\GSTZ1\UFSP2\SLC30A6\SCCPDH\TTC7B\DHCR24\UBR7\EPGN\OMA1\MACC1\HSD17B11\HIBADH\NGLY1\AMPH\PTPLB\ESR2\ATF7IP\RANGAP1\NEMF\LRRFIP2\TAB2\NOL12\FBXO7\WDSUB1\RIC8B\TXNDC5\IRF4\BID\MICAL3\BPGM\CLPTM1L\ANKS6\ABCC9\VPS45\MAP7\KIT\PPFIBP1\USP5\PTPN6\MBLAC2\HORMAD1\F2RL1\CRHBP\C3H6ORF186\MAMDC2\BOC\PCMTD1\IDUA\HTRA3\LRPAP1\ADD1\WHSC2\SYNCRIP\SLC17A5\PLEKHF2\GNOT1\PRDX4\SNTB1\MBOAT2\KLF11\UBXN2A\INTS9\RP2\ABHD13\SLC25A30\KIAA0226L\SLC46A3\MPHOSPH8\MRPL48\B4GALT1\POT1\E2F5\NCOA7\SLC25A14\XRCC3\ATE1\NDUFA10\LOC415708\KPNA1\MARCKSL1\LRIG1\PUF60\GART\ANLN\PDLIM5\HNF1A\DGKZ\SLC1A2\GABPB1\HNF4beta\CD151\LRRC31\SLC47A1\LIMD2\SLC9A9\MAP3K14\EFTUD2\KIAA0319L\
P<0.05	gga-miR-1759-3p_L-3	PCNX\TNNI1\LIN28A\LSM7\CHMP7\LONP1\CCDC47\TERF2\RASSF5\NUDC\TMEM222\NPHP4\GAPVD1\TPRG1L\NEK6\WBSCR17\CIAPIN1\ENKD1\APLP2\LOC100857709\RALGAPB\NFS1\PRPSAP1\ABCA3\TMEM120A\PEX12\TMEM194B\ADPRHL2\SMN\MMP17\CREB3\FANCE\PIK3CD\KCTD7\CRIP1\ENPP1\DDX49\SCPEP1\MLX\NFYA\UBIAD1\ACSS2\KIAA1191\ESRP2\SBNO1\CCND3\ZNF511\PPP1R16B\AP3M2\PRRC2B\TMEM38A\TRIT1\LOC101749801\ZDHHC9\PPM1M\MFAP3\HMGCL\CHRNA7\NADSYN1\ATOX1\FAM20B\TMIGD1\C28H19ORF10\SIGIRR\DOLPP1\SGPL1\MPRIP\KIAA0355\CDK9\VIPR1\RRP1B\KBTBD5\RPUSD1\POLR1E\RHOG\DENND6A\WSB1\ARFGAP1\RAP1GAP2\MLST8\DGCR2\RPN1\PLS3\SLC22A13\ARHGAP17\TPRA1\EARS2\RTEL1\KIF1A\HPS5\TGFBI\TMEM43\CXCL14\PDPK1\FAH\PROCR\CSTF2\CHID1\CTSA\RNF185\ARRDC4\ACADS\BRWD3\PGS1\TK1\HSPA4\SUDS3\KMO\RAB22A\CLCN7\NIT1\SLC9A3R1\STAT1\FBXW4\EIF4E3\DNAJA3\NMRAL1\APIP\TTC17\SLC9A8\CELF1\ORC5\ORC2\TBX3\NEURL\CIB1\SORCS1\CCNYL1\SLC38A2\TYRO3\SHOC2\EXD3\FAM3C\ING3\YPEL5\SNAP23\NCSTN\C2H7ORF64\SLC25A12\EIF2S1\CLCN3\CPXM2\RPAP3\ASNS\SRGAP1\TMEM184C\GLO1\INTS10\C4H8ORF40\GLIS3\MMACHC\ADCK1\OSTC\GTF2A1\DHCR24\TSNAX\HNRNPA2B1\HHIPL1\CRADD\FGFR1OP\BAG5\KIF26A\ATF7IP\POLDIP3\SREBF2\SCRN2\ST13\RAB3GAP1\MICALL1\NHEDC2\TBXAS1\KIAA1549\FARS2\SLC37A3\CREB3L2\GALNT1\SORBS2\AGMAT\NPEPPS\SLMO2\MVK\SCNN1A\ANKRD52\FOXJ2\MAN1A2\GAPDH\CHD4\EMG1\KIAA0907\HORMAD1\ARSK\SERINC1\TNFRSF1A\NPC1\

		C3H6ORF186\B4GALT4\ZDHHC23\NFKBIZ\UFL1\CASP8AP2\WHSC2\PLEKHF2\UBOX5\MORC3\HMGN1\SFXN5\TSTA3\SIK1\PHF20L1\DMD\FBXO9\FBXO25\TAF2\LAPTM4A\RHOB\TMEM214\ASXL2\PTK2B\TLR7\CENPO\ELP3\MCM3\CD2AP\WBP4\SLC25A15\XPO4\MED17\UCP3\NTM\RNASEL\UTP15\LSAMP\GTPBP1\EMP1\TERF1\FAM175B\TANK\MCCC1\ARHGAP19\CDC42\HMGCL\CD4\RREB1\PCMT1\BMPR1B\PDLIM5\HOXA11\PON2\IRF2\RTBDN\PSMC3\SLC6A9\SLC10A7\DDX27\GTF3C5\NUCB2\TNFSF15\CACNA1B\PTPN22\PSMD13\PCSK7\THY1\HSBP1L1\
P<0.05	gga-miR-1754-5p	TIMM17A\RPL9\CCDC15\ELF3\TIRAP\NEK6\UBL7\MOV10\ANP32B\SLC16A1\AANAT\R3HDM4\FBXW11\SEC16A\MMP17\PIK3CD\POLR2E\DEF6\TAF11\RHO\LOC100857356\NRG4\SPHKAP\RPA1\FBXL8\GBGT1\UCK2\GLUL\CAMKK2\DCTN4\C21H1ORF187\TMEM116\PSAP\FLCN\PEMT\URM1\IBA57\HS3ST6\MLYCD\DCAF12\TNFRSF6B\AQP12\C9H21ORF2\KIF3B\POU2AF1\ANKDD1A\CHIC1\NMRAL1\TBX3\PDCD4\FAM45A\RGS7\PLEKHH1\VASH1\LOC424620\SNW1\LY75\WLS\TSN\HIF1A\TNFAIP6\PRKDC\ACTN2\EPHA1\ZYX\TAGLN3\CD247\PGM3\DYSF\KLHL15\IAH1\RHOB\RAB9A\INTS9\ABHD13\BORA\CHRDL2\HSD3B2\RPRD1A\LOC770433\FAM175B\RB1\EGFR\MYSM1\TMEM154\SSTR2\ZRANB1\HDC\TBC1D22A\CACNA1B\LIMD2\SASH3\
P<0.05	gga-miR-1747-3p	ARMC1\C22H20ORF30\BNIP3L\ZNF276\RBBP5\AP1G1\PIGR\CCDC43\PHB\APLP2\PLXNA2\CASP9\TTC1\ITIH3\E2F4\ODZ2\VAV3\CCDC99\PLEKHB2\FBXW11\COMMD10\LOC770371\UQCRC2\LZIC\INPP5K\ACAA2\NKX2-5\SMYD4\STAR\NFYA\CRLF3\DNAJC21\HEATR2\ANO5\TIMD4\FNBP1\ITM2A\FAM20B\RCAN3\P4HA1\MCU\SLC39A11\DUT\ANXA7\SASS6\PTH\NONO\TFIP11\DENND6A\XBP1\PAFAH1B1\ACAA1\ANKRD1\TOLLIP\FAM174B\RNF185\BRWD3\YME1L1\TMEM104\SLC26A4\PTPN1\SPATA2\UQCR10\LOC424943\FBXW8\F2\BRD1\MGA\DSTN\PPP3R1\NDUFA5\EPCAM\LBH\TMEM185A\CTSO\CAV2\ETFDH\YAF2\RASSF3\GLIPR1L\ABCD4\TMED10\PDZK1IP1\ALKBH1\EIF2AK2\BTF3L4\GTF2A1\CASP3\C5H14ORF159\TMEM59\MEOX2\HSD17B11\OSBPL10\SCYL2\UBE2D3\TGFBR1\VPS4B\PIK3C2G\CCDC127\RHBG\CNDP2\SGCB\FGFR1OP2\ATP10D\SCNN1A\PDS5A\SEPSECS\CCLI10\LYSMD3\EPHA1\EPHB6\TNFRSF1A\POLK\F2RL1\RPF2\AMD1\MLLT3\SDCBP\GPR89B\KIAA0232\MXD4\KIAA1009\PLEKHF2\PRIM2\ACP1\FAM49A\C1H2ORF49\TNFSF13B\ABHD13\STK24\LCP1\LCORL\VAPA\GZMK\CASP2\TFEC\C2H6ORF62\C3H2ORF43\PTGR2\DAB1\SUMO3\CDC42\NFYC\RBM6\PDLIM5\PON2\GLDC\RAD54L\TMEM154\FAM169B\P4HA1\
P<0.05	gga-miR-17-3p_L-1R+3	B-G\GFPT1\TRIM27\BNIP3L\KCTD9\HMBS\RNPEP\DCAF7\GALK2\CAMTA1\CNTN2\STX12\RASSF5\SLC2A8\LOC100857226\TAF12\GAPVD1\NEK6\PIK3R5\CREB3L3\SRRM1\CAMK1G\PI4KA\B3GNTL1\RAP1A\GLB1L2\SLC22A7\MYL10\PHTF1\TNFRSF18\ST6GALNAC2\VAV3\GNB1L\SMU1\NCOA4\SAP130\KCNMB1\ACOX1\DTWD2\NUBP2\MMP17\CDC73\TLN1\PIK3CD\INPP5K\MED23\RHO\JMJD1C\TRPC4AP\SURF1\FBXL8\SUB1\COMP\OCM\LOC425137\SFRP1\PPP1R16B\TRRAP\AKTIP\CHD9\UCK1\NECAP2\OSMR\KRT20\TIMD4\SMARCE1\COPS8\CNOT8\RBPJL\TOR1A\ADAM10\KDM2B\TSPAN15\GOSR1\CYP3A7\PLOD1\YIPF6\P2RX5\BRAP\IVD\ECM2\TSEN15\PPIF\HDAC8\

		VPS53\OPNP\VGLL4\ADK\FAM102A\SMG8\MED13\RRP1B\SNX2\SLC35A3\BTBD10\JMJD4\ADIPOQ\DENND6A\C20H20ORF11\TNFAIP1\SPPL2A\BANP\MTF2\LOC100858792\KLHL13\SLC13A5\PI4K2A\KLHL3\GSTT1\BCL2A1\MYH11\COMMD7\MRPS11\KIF11\SRPX2\MARCH5\RNH1\PFKP\LOC395991\SDR42E2\BLNK\TNFSF8\PGS1\RNF166\SYNPR\MAP3K8\CUTC\RAB11A\STAT1\ECI1\NMRAL1\PTS\GGA3\SLC26A4\UQCR10\CHRDL1\ACTR1A\SFXN2\FBXO18\MADD\RELN\ARFGAP2\CIB1\CASP8\STRADB\TRH\COBRA1\CLNS1A\TYRO3\C3H20ORF3\WASL\TASP1\LIN9\LEFTY2\GLRB\CAV2\ETFDH\LOC424998\PLEKHH1\KCTD3\SHFM1\LPGAT1\GLO1\AARS2\CAPN1\FAM161B\GTF2E2\MBNL1\MBNL1\FAM98A\AGA\NEIL3\ITPK1\FAM126A\DFNA5\KLHL8\SNX10\EVL\WLS\TECRL\AAMP\ERI1\GPD1L\AMPH\GC\HSPBAP1\DDX47\SNRPD1\SERHL2\HIF1A\SFRP4\NCKAP5\CASP6\BBS9\MAP4K5\LOC420770\ATL1\SAV1\NOL12\GTDC1\ISX\RMI1\TXNRD1\TXNDC5\ADCK2\VPS4B\MICAL3\LOC418169\VWC2\PIK3C2G\SLCO1B1\CCDC127\SLC6A19\CLUAP1\ANP32E\VPS45\HBS1L\GRK6\MAN1A2\ATN1\MBLAC2\ZYX\ERCC8\F2RL1\ZBTB49\HAUS6\ABHD17B\F5\SEC63\TMEM68\NFKBIZ\FNTA\UBXN2B\USP45\GJA5\PDZK1\PPP2R2C\HAPLN1\TCEB1\ADD1\LOC422887\MXD4\RARS2\CDH17\TP53INP1\ITSN1\SFXN5\RAD21\PDE9A\DDX3X\OC90\GSTA\DERL1\MELK\FAM49A\SDC1\SYAP1\RAB9A\PRPS2\CENPO\INTS9\CHST10\PROZ\COL4A2\TNFSF13B\DNAJC3\LECT1\WBP4\SLC25A15\MPHOSPH8\MMP1\ANKRD42\POLD3\PPME1\PLEKHB1\SPDYA\VAPA\KIAA1609\LOC420301\MICAL1\CLN8\LOC395933\SUCLG1\SLC20A2\PTGR2\ATE1\RNF7\PHPT1\WDR82\SMS\GAL8\POU1F1\C4H4ORF52\ROS1\NT5C3A\PCMT1\NFKB1\DLL1\NCF4\SLC10A7\SLC1A2\GABPB1\CRB2\NELL2\PGRMC1\LDHA\USP7\PTPN22\TUBAL3\REL\YOD1\PDZD11\TACC1\SLU7\
P<0.05	gga-miR-1731-5p	CCDC15\DCAF7\KDM1A\PIK3R6\PBRM1\EIF6\DTWD2\RASA2\ENPP3\TRIP12\SURF1\KIF20A\SUB1\SLC6A2\BPTF\CLINT1\USP4\MYPN\SYS1\HMGCL\CHRNA7\SRSF10\ADAM10\PSMD9\C21H1ORF187\FAM129A\EPHA4\JMJD4\DR1\XPC\KIAA0430\FOPNL\TH\RPRD1B\ACOX2\COX6A1\EDNRB2\UPRT\ATP7A\TRPC5\SF3B1\ACSL4\SLC38A2\ITPKA\LOC424109\AK5\LEFTY2\WDR26\SKIL\SEC62\CAV2\BAG3\THAP5\CCDC132\CPSF6\CCDC167\PRDX1\CC2D1B\SECISBP2\SMEK1\STK39\SLC4A3\RAB5A\KIAA1143\UBXN4\PARP12\KIAA1549\DAP\ISG12-2\FRG1\FGFR1OP2\FKBP4\MAN1A2\REV3L\PLIN2\OSTM1\HJURP\USP45\GRPEL1\CASP8AP2\PEX2\PTDSS1\WRB\PRIM2\FBXO32\DSCC1\CHST10\TGDS\UBL3\TYR\LCMT2\TERF1\LOC770433\SRD5A3\PPP6R3\RGS1\EXOC7\TMEM154\SLC24A5\TBCD\TLN2\CD36\PSMD13\
P<0.05	gga-mir-1724-p5_1ss21CT	RAB11B\PRKCD\TFEB\ARRDC2\OPTC\RAPGEF1\AR\WFDC1\SLC9A3R1\CD82\TYRO3\TMEM229B\RANGAP1\MXD4\KLF11\CD2AP\ODZ4\CHRDL2\
P<0.05	gga-miR-1720-5p	A2ML4\HMGN2\RPA2\KBP\FAM192A\RBM39\NFS1\PSMD5\COMT\H3F3B\SERPINF2\SRM\MBTPS1\KPNA6\CLINT1\SALL1\MINA\MFAP3\FTSJ2\TNFAIP8L1\PSMD9\MCU\P2RX5\PEPD\LOC769755\ADK\TK2\TFIP11\USP10\DCAF12\RPAP2\GORASP1\MCM2\XYLB\ANKRD1\KDELR2\SUDS3\TMLHE\CBLN4\GRB2\TMEM164\PCGF6\NAPEPLD\FAM185A\ABHD12\SKIL\SSB\HHAT\MIA2\TBC1D15\MBNL1\MBNL1\TB

		CK\C5H14ORF159\PPAP2B\EREG\GADL1\PTPLB\SYNE2\METTL14\METAP1\TMEM263\SLC37A3\SYNE1\TRMT11\CLUL1\PEX2\PTDSS1\HMGN1\DMD\ASAP1\FBXO32\UBXN2A\SYAP1\CHRNA2\CD2AP\CCNA1\N4BP2L2\RRM1\ARL6IP5\VDAC3\C2H6ORF62\SLC25A14\MARCKSL1\IMPG2\EGFR\DNAJA1\OAT\GTF3C5\CHRNB4\TPCN3\TNFRSF10B\AFAP1L1\
P<0.05	gga-miR-1716_L-2	TCF25\DDX42\FABP3\IL10\CGN\NR5A1\MOV10\PTBP1\WDR33\POR\ABCA7\C26H6ORF106\NKX2-5\BSDC1\ARRDC2\TOP1\CLIC2\BCMO1\RAP1GAP2\PROCR\KIF7\LOC770215\KIFAP3\PLA2G1B\PPRC1\CNNM1\SLC9A3R1\FBXW4\BCL11A\UBE2H\MRPS7\C5H14ORF102\TOB2\FBXO7\WNK1\SOHO-1\UNC5A\CD5\NF2\MARCKSL1\IGFBP5\HOXD8\EFTUD2\THY1\CD320\
P<0.05	gga-miR-16c-5p	RFK\ARHGAP25\C22H20ORF30\LOH11CR2A\DAD1\PRNP\TMEM9\PSMF1\KCTD9\PSMC5\DHDDS\SPPL2B\ZNF593\CDC27\RAB11B\RBBP5\KIAA0556\PM20D1\RASSF5\KIF18B\NUDC\PPP6C\CCDC43\C4BPA\SAMHD1\WDR16\PLXNA2\MAP2K2\PRKCZ\CAMK1G\POLRMT\ADRA1B\MECR\MIB2\STMN1\IRF1\LOC419409\NCAPD3\PPIL2\NFS1\MYO1F\ATP6V1B2\SLC16A1\GLYR1\SDF4\WAPAL\GNB1L\PMPCA\STXBP3\LOC100859653\ACOX1\PLEKHB2\RFFL\EP400\HIBCH\NUBP2\ENO1\MAT1A\CRYM\SLC25A33\RGS4\APH1A\KCTD7\TRPC1\TFAM\VAT1\NTAN1\TADA1L\EXOSC10\PARN\ERLIN2\ITPA\VAMP4\CRLF3\ERLIN1\KPNA6\RHOT1\BSDC1\DNAJC7\SFRP1\CCND3\TAF8\ZNF511\YARS\TRRAP\NT5C3L\MINPP1\C21H1ORF144\SALL1\TMEM38A\SMARCE1\P2RX4\N4BP1\DBNDD2\MYPN\AP1M1\ASB18\PPM1M\DNA2\MICALL2\FAM81A\TOMM34\TDRD5\RNF111\ATOX1\RNF34\SIRT3\HK1\TMIGD1\SIGIRR\KCNJ2\PPA1\CCNE1\WIPI2\HVCN1\MTHFR\P2RX5\TAX1BP3\ATXN2\LOC423008\PDCD5\RGS9BP\SET\CPSF4\PSAP\PHKA1\IARS\FLCN\WSCD2\PHACTR3\VPS53\MTFR1\PEPD\MYEF2\DRG2\DUT\TAF7\TOP3A\PLA2G4A\NUP210\WDFY1\RPS6KB1\CDC14A\BICD2\CDH5\SLC25A36\IBA57\PTH\CA4\MRM1\PDLIM1\TADA2A\MPHOSPH6\LOC768701\SYNGR3\KLHL18\NTHL1\COL9A3\RNF141\TSC2\DENND6A\STK25\ARFGAP1\DCAF12\PLS3\GDF2\ABTB1\PRPF6\DOCK11\GORASP1\MCM2\WNT8A\MAPK8\RTEL1\COL6A2\FAM122A\HPS5\MIF\K123\TMEM43\DDT\CRKL\PDPK1\PANK1\TTLL9\ANKRD1\FOPNL\DNAJB11\TBCCD1\TNNT3\BCCIP\ABCC1\FANCI\TOLLIP\YWHAH\CBL\TSPAN6\CTNNBL1\NCBP2\PISD\MARCH5\PFKP\BDH1\RNF185\LOC100858827\PITRM1\RNF10\RMI2\ANKDD1A\GCN1L1\SVIL\ANKRD40\ABCC2\TUBB1\ACBD5\ZFYVE27\RRP12\CDX4\C6H10ORF76\CHIC1\TRAP1\MAP2K1\EIF4E3\SALL4\VRK2\KCTD2\SF3A1\ZC3HC1\BCAP29\UBE2V1\RNF114\SPATA2\LOC417013\CD82\ARL3\UQCR10\CHRDL1\NDUFS3\MOB4\NT5C2\RELN\C15H12ORF49\CKMT1A\RAD18\STRADB\SMARCA1\AP2M1\ARHGDIA\ARRDC1\ABHD12\FASTKD2\TUBGCP4\SMNDC1\PTK7\GTF2H4\PDCD4\RSU1\ITGA8\RPF1\LMOD2\DCUN1D1\PPP1R21\EPCAM\ENTPD2\ACBD3\C3H1ORF55\C6H10ORF46\OLA1\DCAF4\DPF3\CAPZA2\LOC423256\CAV2\RXFP1\SERPINI1\RAB3GAP2\SLC25A12\MYO3B\ZNF650\GFM1\CLCN3\DYNC1I1\ASNS\C1GALT1\LOC422442\G2E3\SPOP\MYST2\EAPP\CRIPT\MCFD2\GEMIN2\HSPA4L\EIF2B2\POLR1C\LOC395824\CMPK1\ALKBH1\ADCK1\EPS15\BTF3L4\CC2D1B\SCP2\TFB2M\HMGB2\SLC24A4\MEOX2\AREGB\MACC1\CYP2J2\STK39\PGM1\DNAJC6\SNX10\COBLL1\CAPN9\PKDCC\C3H6ORF120\EVL\AGPAT9\WLS\OXNAD1\PPP2R5C\CRADD\TECRL\CRYZ\LTA4H\T\CAPN7\GRSF1

		\MAP3K4\C5H14ORF79\PTPLB\MYLK\MGP\ITGB5\ESR2\SYNE2\SERHL2\LARS2\CLEC3B\C5H14ORF135\XPNPEP3\RTN1\ARPP21\AVL9\RAB3GAP1\RAP1GDS1\ZRANB3\ADH1C\ATP5S\RAB32\NHEDC2\STK17A\IYD\TXNDC16\PLEKHC1\FBXO7\WDSUB1\PWP1\RBM24\TXNDC5\SSR1\MKRN1\WRNIP1\CREB3L2\BPGM\PLEKHA5\GALNT1\EXOC3\TULP3\SORBS2\FAM149A\GMNN\TDP2\VPS37A\LOC426218\DNAJC16\AGMAT\NPEPPS\CEP192\MAP7\SGCB\EED\DCUN1D4\FGFR1OP2\PPFIBP1\TEC\GPR155\PRR5\NUP50\SCNN1A\APBB2\EDARADD\CD38\TPI1\C12ORF57\ACTBL2\MBLAC2\HORMAD1\ERAP1\EPHA1\SGTB\WDR3\SMCHD1\SERINC1\CEP85L\MOCS2\RABL3\WDR1\FAM26E\ZBTB49\QTRTD1\TAF13\TBC1D23\OSTM1\TXNL4B\TMEM175\IDUA\ATG5\CD200\SDR16C5\CYP7A1\MAEL\USP45\GJA5\GPR89B\CHMP2B\RNF20\ZCCHC9\TCEB1\HSPA13\SH3BP2\USP25\MDN1\PTCD3\CD8A\TTK\RIPK2\TMEM30A\OTUD6B\IFNGR2\CRYZL1\PTDSS1\EMC2\TOP1MT\U2AF1\SIK1\NDRG1\MAOA\DDX3X\TINAG\POLA1\FBXO9\WDYHV1\RNASEH1\ITSN2\PHKA2\ASXL2\REPS2\HADHB\RAB9A\MTMR9\FDFT1\DEFB1\ASMT\CHST10\IL1RL1\ADPRHL1\STK24\DNAJC3\IRG1\LMO7\TBC1D4\WBP4\CPB2\WDFY2\UFM1\UBL3\SLC7A1\SLC46A3\FLT3\NUPL1\MED17\PPME1\C11H16ORF87\VAPA\TPD52L2\LYN\CLDN10\MBNL2\GTPBP1\ARHGDIB\POT1\CERK\UPP1\ETV1\CLN8\SGK1\SLC20A2\PPP6R3\DAB1\ARHGAP19\ATP6V0E2\TGFBR3\RPE\RSFR\LOC427470\RRN3\PRR11\TCEB3\KPNA1\LOC419404\NADK\LOC101750010\IRAK2\RBM6\BIRC2\TMEM123\RB1\WWP1\CENPQ\SLIT2\LOC423536\EGFR\GOLGA4\PDLIM5\DLL1\RARB\LPL\ATF4\CEBPZ\RAD54L\LOC100859142\CUL2\HOXD4\LOC100859627\GABPB1\CRB2\PGRMC1\MYO5A\ARPP19\MYO19\TMEM120B\PELI1\KIAA1328\ACTL6A\HEATR3\ATP11B\WDR43\PSME3\USP7\CYP1A4\RAPGEF6\CACNA1B\LIN7B\PDZD11\P4HA1\ANG\PFKL\CD320\TIMMDC1\
P<0.05	gga-miR-16c-3p_L+1R-1	WDR36\ERMP1\GALK2\RBBP5\MFSD4\FBXW2\SRPR\POC1A\SLC16A1\GLYR1\PHTF1\ACO1\ENO1\GBAS\TRIP12\EXOSC10\HSDL1\ERLIN1\CCZ1\GOLGA7\USE1\CHD9\OSMR\RICTOR\TMEM38A\NADSYN1\BET1L\EIF3B\SLC13A3\RSRC2\OPHN1\MAPK6\DHX30\CDC14A\ARHGEF3\EVI2A\PITPNB\DENND6A\CDX1\IPO7\DR1\KIAA0753\BTBD1\GPC4\POFUT2\IL2RA\MAP3K13\TOLLIP\SPPL3\MTMR8\CBLN4\KIAA1841\LAMB1\HN1\MRPS7\UBE2V1\TMEM164\NDUFS3\SLC39A13\AOX2P\DDB2\ANKRD12\XPNPEP1\NELF\LOC431656\CRAMP1L\PRKCI\MSMO1\SRP14\C5H15ORF29\PTPN14\RAB33B\LPGAT1\TMEM184C\BRE\KIF6\GEMIN2\FCF1\SLC30A6\RYR2\GOLGA5\CBX3\C5H14ORF129\WARS\KERA\TECPR2\SEMA5B\ATF7IP\SNAPC1\HIF1A\ELMO1\HERPUD2\CASP6\FKBP9\LOC420748\GMFB\HNRNPK\MYLIP\C1H12ORF48\EEF1E1\SERPINB10\ZBTB2\CECR2\GRB10\CNDP2\SGCB\EDARADD\SEPSECS\DHX15\PRRC1\DEPDC1B\KIF2A\LPIN2\ANKDD1B\ZBTB49\HAUS6\GNE\IDUA\HACE1\ACOT12\TCEB1\SNX30\WHSC2\KIAA1009\IMPG1\CALB1\KLF11\UBXN2A\EGFL6\C1H2ORF49\ATP4B\ARGLU1\SLC25A15\UFM1\H2AFZ\ALG8\VMO1\E2F5\SLC20A2\MFI2\ZP4\MPZL3\FLNB\OVAL\ROS1\PDLIM5\TBC1D22A\ATP2A2\REL\CR1L\SUPV3L1\
P<0	gga-miR-1699_	SLC25A46\HMGN2\LSM7\HBXIP\PADI3\RAB11B\KIF18B\C11H16ORF80\TMEM222\NCAPD3\ACAD8\JMJD6\PHYHIPL\PHTF1\PTBP1\ME

.05	L-1R-2	D16\GLUD1\POR\GPSM2\NR5A2\WBP2\GBA2\AQP3\RGS4\INPP5K\FRZB\WDR59\LRRC45\TARDBP\REG4\ETFA\RAB5C\TAF8\RBBP4\DDX31\PLAT\EGFLAM\OSMR\TMEM38A\PAX7\PIGT\RNF111\LOC771090\IFITM1\MAP2K6\VPS33A\KNTC1\PLOD1\NDST1\C21H1ORF187\CLIC2\BMP15\C14H17ORF103\FAM129A\LOC768735\CCNDBP1\ATPAF2\VGLL4\USP30\FAM120A\USP54\VIPR1\SLC25A36\PTH\HAGH\CCDC12\NOLC1\SYNGR3\XBP1\TMED5\DPP6\NAP1L4\SRPRB\C9H21ORF2\TOLLIP\NOX1\CBL\ITIH2\ARIH2\TLR4\PDHB\NRP1\SYNPR\ITGB2\RAB22A\CDX4\CHIC1\TRAP1\BCL11A\PGK1\SNAI1\ANP32A\ITPR1\CIB1\FGL2\STRADB\TWSG1\RALGAPA2\AP2M1\STAG2\TUBGCP4\EEF1B2\TUBGCP6\TRDMT1\SHOC2\AFTPH\UGP2\THUMPD2\AMDHD2\SFRP2\ZFYVE1\ETFDH\GPATCH2\ACADSB\LOC426599\COCH\TMEM184C\FAM177A1\KIAA0391\AARS2\FCF1\VIPAR\LOC424620\ALKBH1\ALDH18A1\BTF3L4\CHN2\EPYC\ZRANB2\BCS1L\BRP44L\ARL1\IL8L1\SNRPD1\CLEC3B\C22ORF32\KDELR3\SOX10\MMADHC\TIMP3\NTRK2\DEK\RBM24\TXNRD1\TDG\EEF1E1\TXNDC5\BID\DDC\LOH12CR1\PCYOX1\FAM76A\ENO2\C1S\LYSMD3\SPICE1\SS18\GRAMD1C\SNX3\ALDOB\AFAP1\C6ORF162\RIPK2\TOP1MT\LY6E\NRBP1\REPS2\F7\CAB39L\POSTN\FLT3\DPT\ARHGDIB\CERK\LOC100857925\MLANA\EPM2A\PPP1CB\XRCC3\AK6\LEPR\KCNAB1\ARHGAP19\ATP6V0E2\C2ORF47\LOC101750010\AFAP1\ARF1\ATP2A2\PELI1\NBL1\MYOF\PSME3\EIF4G2\PYGB\GNB1\P4HA1\UBE4A\
P<0.05	gga-miR-1668-3p	B-G\SPR\GFPT1\KCTD9\IPO9\LSM7\SRSF3\EYA3\RASSF5\POM121\CIAPIN1\SAMHD1\COX10\GUCA1A\ACAD8\NFS1\MYO1F\SLC16A1\DGKD\C1H11ORF73\PMPCA\R3HDM4\UNC45B\AGO3\DTWD2\COMMD10\HIBCH\SORD\RUSC2\CRYM\LZIC\ITGAV\SLC16A3\LOC100857356\CTGF\SCPEP1\CHUK\TMED2\ARL10\ALDH9A1\ARRDC2\BAIAP2L1\FEN1\MED27\PLAT\SALL1\KRT20\MINA\SMARCE1\COPS8\ZDHHC9\GPT2\BET1L\SLTM\ADAM10\TMIGD1\AP5Z1\YIPF6\CD74\AKAP10\STAU1\PHACTR3\DUT\C2H3ORF75\AP3M1\MPP1\GTPBP5\GHRHR\SLC25A36\DYNLRB2\CCDC12\WFDC1\TFIP11\BANP\RPN1\MTF2\MGAT4B\MYD88\COL6A2\XPC\OSBPL5\C13H5ORF15\CRTAM\ZMYND11\RLBP1\PLOD2\RPTOR\RNF185\KDELR2\ACOX2\RMI2\COQ5\SUDS3\ARCN1\TUBB1\EDNRB2\C10H15ORF44\YME1L1\SNAPC5\TRAPPC4\BCL11A\BCO2\ZC3HC1\TMEM180\CELF1\RINT1\MOB4\CASC4\EDEM1\XPNPEP1\AHSG\VPS54\PRPF4\JAG1\YPEL5\SFRP2\DEGS1\CTSO\KRIT1\CAV2\ACTN1\TMEM229B\GPATCH2\CCNL1\PTPN14\RAB33B\C5H15ORF41\TMEM206\GNS\WDR65\CPSF6\ABCE1\CAPN1\VSX2\FAM98A\CMPK1\EPS15\RAB3B\GTF2A1\UFSP2\PPPDE1\GALNTL6\SCIN\NID1\PGM1\LEPROT\RFTN1\NDUFA12\TNFAIP2\BAG5\NR1H4\SEMA5B\SYNE2\LOC425756\TMC2\FBXL2\PCDP1\CCDC93\LRRFIP2\C3H6ORF103\SOUL\MMADHC\C22ORF28\TMEM263\NFATC1\TXNRD1\SSR1\SERPINB6\PRKDC\CHUNK-1\BID\EXOC3\PBX4\LOC420992\PLEKHO1\LOH12CR1\PPP6R2\CTRC\KDR\SGCB\RPS12\ARNTL2\C2H8orf46\NUP50\UCHL1\MBLAC2\SGTB\MTX3\RPL37\HAUS6\XCL1\GPR89B\MMS22L\KIAA0232\GRPEL1\ABLIM2\RNF4\WHSC2\CD8A\TP53INP1\PLEKHF2\MORC3\SIK1\LOC396380\RHOB\PIGA\CENPO\DNAJC15\HMGB1\FLT3\MMP27\PLEKHB1\COTL1\GZMA\UBE2N\ETV1\CLN8\NCOA7\CAPN3\RPL22L1\FAM183A\NDUFA10\KPNA1\NCF1\IRAK2\TMEM123\GRIA4\TMEM131\POPDC3\TAPT1\DNAJA1\PCMT1\NFKB1\HMOX1\GYPC\USP37\HNF1A\CUL2\PGRMC1\CD151\TBC1D22A\GHRHR\GTF3C5\KIAA1328\NPRL3\CYP2H1\REL\RER1\

		SLC9A9\MAP3K14\EFTUD2\TMEM130\CD320\NGEF\LEPROTL1\
P<0.05	gga-miR-1663-5p_R+2	TIMM17A\STARD4\PSMF1\HMBS\RBBP5\MFSD4\PM20D1\PPP1R8\ZMPSTE24\NR0B2\TPRG1L\DBR1\ATP5G1\MIB2\RAB14\STMN1\CYB5D2\ZAP70\RRAGC\SSU72\NISCH\PPIL2\HAUS1\MYL10\GFRA2\GNB1L\AANAT\GOLGA3\ACO1\FBXW11\GALNT9\RAN\NUP35\NCKAP1\CTGF\SPHKAP\SERPINF1\PRDX6\C26H6ORF130\DDX55\WHSC1L1\UBIAD1\GOLGA7\RBBP4\SLC6A2\USE1\EGFLAM\TAF9\CCPG1\CYP3A4\C21H1ORF187\ASPA\CEBPG\MKRN2\ACACB\PTGS2\FGD3\TK2\SELK\HELLS\DYNLL2\SYNGR3\TFIP11\DENND6A\C20H20ORF11\PAFAH1B1\TRIM66\PSMA1\HPRT1\TBX6\C12H3ORF19\POFUT1\PM5\AP2A2\MARCH5\PFKP\BDH1\BLNK\DUSP12\PTPRG\NME1\FGF1\C10H15ORF44\NPEPL1\FYTTD1\RAB11A\EIF4E2\STK17B\CD82\TMEM164\NEURL\CDK15\CRNKL1\SUMO1\STAG2\TRH\MRPS25\CUL4B\ANAPC2\DSTN\KIF16B\PPP3R1\SAMD13\SESTD1\TASP1\AMDHD2\BAG3\ETFDH\YAF2\DYNC1I2\BUB3\HCLS1\HHAT\PRPH2\PHF17\HECTD3\E2F7\MRPS18A\SLC33A1\HERC3\ADCK1\C2ORF56\PPPDE1\SCIN\EREG\G3BP2\HOOK1\LYST\ALG6\HNRNPA2B1\CBX3\EML1\FRMD1\ERI1\BAG5\PTPLB\LZTFL1\CCNA2\LOC425756\POLR3H\CCR8\DACT1\CAMK2D\LRRFIP2\NMI\NAA35\ALDH1L2\SLC41A2\ADAM12\BLOC1S5\ECI2\RDX\LOC418169\MGST1\CLEC3A\PLEKHO1\C6H10ORF137\C2H18ORF19\VPS45\ARHGAP18\CYB5R3\FKBP4\EDARADD\LYSMD3\FAM172A\SGTB\RABL3\C1H3ORF26\ARL13B\GRPEL1\ADD1\MDN1\LYRM2\RARS2\SYNCRIP\HMGN3\EEF1A1\B3GAT2\ATP6V1C1\RHOB\PIGA\TUBGCP5\EIF5B\TNFSF13B\LMO7\RNF17\FAM76B\ARL6IP5\VAPA\SUV39H2\GTPBP1\UBE2N\INVS\RPRD1A\WDR48\PPP4R2\NEO1\NFYC\NADK\C26H6ORF89\TMEM123\GAL8\WDR35\COL9A1\EEF1A2\MRPS26\VWF\DNAJA1\TMED8\NPVF\TMPO\DGKZ\PSMC3\RPL35\GATM\FASN\EMB\CSDE1\LIN7B\MBD3\
P<0.05	gga-miR-1662_R-3	RASSF2\RDM1\WDR36\RPS6KA1\LSM7\PM20D1\C23H1ORF38\NR0B2\NPHP4\MYBBP1A\WDR45L\TEK\VAV3\DTWD2\OSGEPL1\RGS4\RAN\DEF6\ACAA2\TMEM231\GBGT1\SBNO1\CCZ1\CCND3\SYS1\CHMP1B\TTPAL\NPFFR1\ZMYND8\SGPL1\VPS53\FAM120A\SNAP47\MPHOSPH6\CCDC12\RNF141\TMEM97\GCLM\IPO7\TMED5\MYD88\UBE2I\TBCCD1\AP2A2\IL13\MARCH5\KDELR2\ACOX2\CD3E\MTMR8\LOC416354\TIPIN\EGR1\DNAJA3\CD44\CHL1\KCTD2\UQCR10\HSPD1\SRPK2\PSMD10\FGL2\F2\ATG13\PIGK\AKAP9\PRKCI\KRIT1\C5H15ORF29\FMN1\SHFM1\RAB33B\PPP2R5A\NKTR\TMEM184C\CFL2\GEMIN2\KIAA0494\SNW1\LEF1\ALG6\AGPAT9\TNFAIP2\SEC24B\IGF2R\BFSP2\TTC26\SOX10\HECW1\DDHD1\MMADHC\MKRN1\CLPTM1L\STX17\FAM149A\ANKRA2\TBC1D1\MAP7\FAM76A\PPFIBP1\SCNN1A\ACTBL2\ZYX\PRRC1\SERINC1\F2RL1\ZBTB49\NSG1\ST3GAL6\ALCAM\HJURP\IDUA\CYP7A1\CASP8AP2\TMEM129\SLBP\ORC3\EEF1D\FAM49A\UBXN2A\LOC421978\EFR3A\TGDS\SPRY2\C1H13ORF15\MED4\CAB39L\WDFY2\FAM76B\KIAA1609\UBE2CBP\LOC101751006\PPP1CB\ZP4\CDC42\TCEB3\RSPO3\PELI1\CHRNB4\
P<0.05	gga-miR-16-1-3p_R-1	C22H20ORF30\DAD1\LSM7\CHMP7\GALK2\ZBTB48\MFSD4\EYA3\SRPR\SNAP29\SLC16A1\CCNG1\JMJD6\PHTF1\PMPCA\ACO1\FBXW11\TMEM194B\ENO1\MATR3\KTN1\PIK3CD\TARDBP\TRIP12\NRG4\MMD\REXO4\MLX\EIF4EBP1\ARL10\ESRP2\SBNO1\PPP1R16B\PHKB\

		NADSYN1\LOC422151\SLC13A3\MAPK6\CLIC2\NOL8\LSM14A\C8H1ORF27\PLAU\CUL3\WDFY1\COL11A1\CDH11\TADA2A\EIF4G2\DENND6A\DCAF12\IPO7\PAFAH1B1\MTF2\RRAS2\KIAA0753\GORASP1\MST4\PLK1\ACTR3B\CHCHD4\IL2RA\SEC24A\PANK1\MNX1\PPP2CA\BRPF1\MARCH5\CHKA\PGS1\ANKDD1A\ZEB1\CADPS\CDX4\USP12P1\CSTF1\KIAA1841\APIP\ATP7A\LAMB1\UBE2V1\TMEM164\CTDSPL2\NAPEPLD\ANKRD12\TWSG1\MCTS1\NUSAP1\TTL\TRDMT1\WASL\SAMD13\VPS54\ZZZ3\JAG1\PIF1\C6H10ORF46\CTSO\C2H7ORF64\CCDC132\SLC25A12\DCAF17\MSMO1\C5H15ORF29\ARID2\METTL5\PTPN14\XPOT\STXBP6\IFNG\PREPL\BRE\GEMIN2\GTF2E2\PRPF38A\PPPDE1\MEOX2\DHCR24\ANKMY2\RYR2\UBR7\INADL\IGF2BP3\NTPCR\PGM1\TSNAX\CBX3\GLRX5\C5H14ORF129\ITGB6\WARS\KERA\SLC44A5\AAMP\BIN1\UMPS\SYNE2\ATF7IP\FAIM\ELMO1\MUDENG\CCDC93\CASP6\FKBP9\ZRANB3\MYLIP\TXNRD1\PMCH\EEF1E1\TRIM24\VPS4B\DNM1L\LOC418169\CLPTM1L\FBXO5\GMNN\DNAJC16\EXOC1\MPPE1\CEP76\SGCB\ARNTL2\M6PR\FKBP4\SEPSECS\PRRC1\DEPDC1B\SGTB\TRMT11\ANKDD1B\ZBTB49\PCMTD1\TMEM68\SDCBP\ACOT12\TCEB1\HSPA13\SAMSN1\WHSC2\FABP5\SCAF4\CA2\B3GAT2\FAM135A\TP53INP1\PTDSS1\MORC3\EIF3H\ZNF451\ENPP2\PIGA\RP2\C1H2ORF49\ATP4B\CARS2\DZIP1\WBP4\COG6\UFM1\N6AMT2\CRYL1\RNF17\LOC419074\VAPA\SUV39H2\LYN\PACSIN2\POT1\CERK\PTPN3\RPRD1A\WDR48\HCRTR2\CZH9ORF80\FAM175B\MCCC1\ZP4\KPNA1\SMS\SOD1\PIGN\OVAL\PPARA\NT5C3A\RNASET2\CHRNA6\DGKZ\TTC21B\BPNT1\SLC1A2\WIPF1\GABPB1\RNF13\DDX52\CNN3\ARF1\TBC1D22A\NUCB2\EIF4G2\ACLY\REL\CR1L\YOD1\GSPT1\
$P<0.05$	gga-mir-1609-p3	WWP2\LOC428335\RALGAPB\STOML1\RRAGC\EIF6\ADPRHL2\MTMR9LP\SKP2\FAM189A1\TWF2\SRSF10\ANXA6\SAR1A\NUP62\MYEF2\MKRN2\ASB14\COL9A3\DCAF12\CDK10\KIAA0430\NCOA5\ABCC2\ARCN1\EME1\TMEM104\NMRAL1\NT5C2\CASC4\PHTF2\CFTR\C4H4ORF27\NUP107\LRRC40\USP18\FBXO40\C6ORF170\ORC3\DDX3X\CLN5\MED17\YWHAZ\FAM125A\TPD52L2\LOC422075\PPP6R3\LOC101750010\DNASE1L3\MATN3\NBL1\ARPC1A\
$P<0.05$	gga-miR-1602	GFPT1\SPPL2B\ELF3\KLHDC8A\PPP1R8\DHODH\LHFPL5\NEK6\PIK3R6\PIK3R5\SIRT6\PPIL2\MOV10\ATP6V1B2\EIF6\TNFRSF18\AANAT\PLEKHB2\LOC427001\TRAPPC3\RUSC2\GBAS\GMPPB\TLN1\SBNO2\DNAJC12\GRM4\GBGT1\PGC\CCND3\PIGS\SMARCE1\LOC101749801\ZDHHC9\EMC1\CAMKK2\GPR174\SIRT3\GOSR1\TBCA\BAP1\ASB6\TMEM116\PTGES2\LOC768735\FAM101B\OPNP\MPP1\TRIM37\NDST2\INTS2\CDH5\RPUSD1\TNFAIP1\PAFAH1B1\GDPD2\RAB7A\CDK10\XYLB\CRKL\DNAJB6\DEPDC5\PPP1R2\TNC\RAB35\FGF1\ACBD5\EIF4E3\SF3A1\TRPC5\GGA3\TMEM180\CD82\RINT1\WSB2\SUMF1\AP2M1\SUMO1\EHMT1\DUSP5\MGA\YPEL5\SFRP2\TMEM229B\POLR1C\CYP4B1\LOC100859230\TFB2M\TTC4\LGMN\DFNA5\HNRNPA2B1\LUM\EXOC3L4\CKB\IGF2R\HSPBAP1\CLASP2\TAB1\C3H6ORF103\TNFAIP6\MKRN1\MBP\SLMO2\C2H18ORF1\LOC421049\ITPR2\FOXJ2\ZNF384\TPI1\FAM26E\SDR16C5\TRIM55\WHSC2\THNSL2\ADRA1D\MORC3\DSCR3\GNOT1\LOC421988\ZNF395\INTS9\WBP4\RCBTB1\DYNC2H1\ANKRD42\LSAMP\NDUFA10\FSCN1\DENR\LAMB2\FLNB\ALAS1\GAL9\CYP24A1\XIRP1\NBL1\

P<0.05	gga-miR-15b-5p_R-1	RFK\ARHGAP25\LOH11CR2A\DAD1\PRNP\TMEM9\PSMF1\KCTD9\PSMC5\DHDDS\SPPL2B\ZNF593\RAB11B\RBBP5\KIAA0556\RASSF5\KIF18B\NUDC\PPP6C\CCDC43\C4BPA\WDR16\PLXNA2\MAP2K2\PRKCZ\CAMK1G\POLRMT\ADRA1B\MECR\MIB2\STMN1\IRF1\LOC419409\NCAPD3\PPIL2\NFS1\MYO1F\ATP6V1B2\SLC16A1\GLYR1\SDF4\WAPAL\GNB1L\PMPCA\PKM2\STXBP3\C19ORF6\LOC100859653\ACOX1\PLEKHB2\RFFL\EP400\NUBP2\ENO1\MAT1A\CRYM\SLC25A33\GJA4\RGS4\APH1A\KCTD7\TRPC1\TFAM\RFWD3\VAT1\NTAN1\TADA1L\EXOSC10\PARN\ERLIN2\DDX49\TSR1\ITPA\VAMP4\CRLF3\ERLIN1\KPNA6\RHOT1\BSDC1\DNAJC7\SFRP1\CCND3\TAF8\ZNF511\YARS\FNDC5\TRRAP\NT5C3L\MINPP1\CHD9\C21H1ORF144\SALL1\TMEM38A\SMARCE1\P2RX4\N4BP1\DBNDD2\MYPN\AP1M1\ASB18\PPM1M\DNA2\MFAP3\MICALL2\FAM81A\TOMM34\TDRD5\RNF111\ATOX1\RNF34\SIRT3\HK1\FAM20B\TMIGD1\SIGIRR\RCAN3\KCNJ2\PPA1\CCNE1\WIPI2\HVCN1\MTHFR\P2RX5\TAX1BP3\ATXN2\LOC423008\PDCD5\RGS9BP\SET\CPSF4\PHKA1\IARS\FLCN\WSCD2\PHACTR3\MTFR1\PEPD\MYEF2\DRG2\DUT\TOP3A\PLA2G4A\AP3M1\NUP210\WDFY1\RPS6KB1\CDC14A\BICD2\CDH5\SLC25A36\IBA57\PTH\CA4\MRM1\TADA2A\MPHOSPH6\WFDC1\LOC768701\SYNGR3\KLHL18\NTHL1\COL9A3\DENND6A\ARFGAP1\DCAF12\TMEM41B\PLS3\GDF2\PRPF6\DOCK11\GORASP1\MCM2\NDUFAB1\WNT8A\MAPK8\RTEL1\COL6A2\FAM122A\MIF\K123\TMEM43\CRKL\PDPK1\PANK1\TTLL9\ATP12A\ANKRD1\FOPNL\DNAJB11\TBCCD1\TNNT3\BCCIP\ABCC1\FANCI\TOLLIP\YWHAH\ATP5C1\CBL\TSPAN6\CTNNBL1\NCBP2\PISD\MARCH5\PFKP\RNF185\LOC100858827\PITRM1\RNF10\RMI2\ANKDD1A\GCN1L1\SVIL\ANKRD40\ABCC2\CD3D\TUBB1\YME1L1\ACBD5\ZFYVE27\RRP12\CDX4\CHIC1\TRAP1\MAP2K1\EIF4E3\SALL4\VRK2\KCTD2\SF3A1\ZC3HC1\BCAP29\RNF114\SPATA2\LOC417013\CD82\ARL3\UQCR10\CHRDL1\NDUFS3\MOB4\NT5C2\RELN\C15H12ORF49\CKMT1A\RAD18\STRADB\SMARCA1\AP2M1\ARHGDIA\ARRDC1\ABHD12\FASTKD2\TUBGCP4\SMNDC1\PTK7\GTF2H4\PDCD4\RSU1\ITGA8\RPF1\LMOD2\DCUN1D1\FKBP15\PPP1R21\EPCAM\TASP1\C3H1ORF55\C6H10ORF46\OLA1\DCAF4\DPF3\CAPZA2\LOC423256\CAV2\RXFP1\SERPINI1\RAB3GAP2\SLC25A12\IARS2\MYO3B\ZNF650\GFM1\DYNC1I1\C1GALT1\LOC422442\G2E3\SPOP\MYST2\EAPP\CRIPT\MCFD2\GEMIN2\HSPA4L\EIF2B2\POLR1C\LOC395824\PDZK1IP1\ALKBH1\ADCK1\EPS15\BTF3L4\CC2D1B\SCP2\TFB2M\GALNTL6\HMGB2\SLC24A4\MEOX2\PPAP2B\AREGB\MACC1\CYP2J2\STK39\PGM1\CBX3\DNAJC6\SNX10\COBLL1\CAPN9\PKDCC\C3H6ORF120\EVL\AGPAT9\OXNAD1\PPP2R5C\CRADD\TECRL\CRYZ\LTA4H\T\CAPN7\GRSF1\C5H14ORF79\PTPLB\MYLK\ITGB5\ESR2\SYNE2\SERHL2\LARS2\CLEC3B\C5H14ORF135\XPNPEP3\ARPP21\AVL9\RAB3GAP1\RAP1GDS1\ZRANB3\ADH1C\ATP5S\RAB32\NHEDC2\IYD\TXNDC16\PLEKHC1\FBXO7\WDSUB1\RBM24\TXNDC5\SSR1\SLC37A3\WRNIP1\CREB3L2\MYO10\MICAL3\BPGM\PLEKHA5\GALNT1\EXOC3\TULP3\SORBS2\FAM149A\GMNN\TDP2\VPS37A\LOC426218\DNAJC16\AGMAT\NPEPPS\RSPH3\CEP192\MAP7\SGCB\EED\DCUN1D4\FGFR1OP2\PPFIBP1\TEC\GPR155\PRR5\NUP50\SCNN1A\APBB2\EDARADD\PI4K2B\MLF2\CD38\TPI1\C12ORF57\PTPN6\ACTBL2\MBLAC2\HORMAD1\ERAP1\EPHA1\WDR3\SMCHD1\SERINC1\CEP85L\MOCS2\GFM2\RABL3\WDR1\ZBTB49\HAUS6\ZDHHC23\TAF13\TBC1D23\OSTM1\TXNL4B\TMEM175\IDUA\ATG5\CD200\SDR16C5\CYP7A1\

		MAEL\USP45\GJA5\GPR89B\CHMP2B\RNF20\ZCCHC9\TCEB1\HSPA13\SH3BP2\USP25\MDN1\PTCD3\CD8A\TTK\RIPK2\TMEM30A\OTUD6B\IFNGR2\CRYZL1\PTDSS1\UBOX5\EMC2\TOP1MT\U2AF1\NDRG1\MAOA\DDX3X\TINAG\POLA1\FBXO9\WDYHV1\RNASEH1\ITSN2\PHKA2\ASXL2\RAB9A\MTMR9\DEFB1\ASMT\IL1RL1\ADPRHL1\STK24\DNAJC3\IRG1\LMO7\TBC1D4\WBP4\CPB2\WDFY2\UFM1\UBL3\SLC7A1\SLC46A3\FLT3\NUPL1\MED17\PPME1\C11H16ORF87\VAPA\TPD52L2\LYN\CLDN10\MBNL2\C1H21ORF33\GTPBP1\ARHGDIB\POT1\CERK\UPP1\ETV1\CLN8\SGK1\SLC20A2\PPP6R3\DAB1\ARHGAP19\ATP6V0E2\TGFBR3\RPE\RSFR\LOC427470\RRN3\PRR11\LOC419404\LOC101750010\RBM6\TMEM123\RB1\WWP1\CENPQ\SLIT2\LOC423536\EGFR\GOLGA4\PDLIM5\DLL1\RARB\LPL\ATF4\CEBPZ\MRPL37\LOC100859142\CUL2\HOXD4\LOC100859627\GABPB1\PGRMC1\MYO5A\ARPP19\MYO19\TMEM120B\PELI1\KIAA1328\ACTL6A\DDX19B\HEATR3\ATP11B\WDR43\PSME3\USP7\CYP1A4\RAPGEF6\CACNA1B\LIN7B\PDZD11\P4HA1\ANG\PFKL\PLEKHJ1\CD320\TIMMDC1\
P<0.05	gga-miR-15b-3p_L+1_1ss22AC	BMP10\RASSF2\CDC27\PANK4\FAM192A\RRAGC\E2F4\TEK\PLEKHB2\NR5A2\HSD17B4\DTWD2\INPP5K\BECN1\MMD\CRLF3\CLINT1\USE1\TMEM38A\KRT20\APBA2\ZDHHC9\LRRC49\ADAM10\TTPAL\IVD\MYEF2\DUT\CLTC\CDH5\BRCC3\MST4\ANKRD1\RBM7\SYNPR\SPATA2\MGA\POLR3F\NDUFA5\COMMD5\TSPAN12\CAV2\ETFDH\CENPF\ATPBD4\INTS7\LPGAT1\HP1BP3\INTU\ZFC3H1\NPC2\TMED10\C5H14ORF159\LOC421560\ZRANB2\SEC24B\KIAA1109\TGM4\C5H14ORF135\CCR8\PPP1R17\GTDC1\FANCC\NUP153\MGST1\PIK3C2G\CSTB\CCLI10\AMD1\PAM\OTC\MYOM2\MOSPD2\KIAA0226L\KIAA1609\INCENP\LEPR\DAB1\TANK\NFE2L2\STOM\SLMAP\UBASH3A\MYH15\FAM169B\UBE3C\KIAA1328\ACLY\
P<0.05	gga-miR-1559-5p	ADAM28\MAPK8IP3\SLC13A3\PDAP1\MKLN1\USP12P1\CLCN3\RAB33B\DFNA5\ANKRD13C\NEDD1\KIF15\PRSS12\RBM24\TDG\VPS37A\KIT\FBLN1\SOHO-1\MICAL1\USP37\
P<0.05	gga-miR-1555-5p	TRIM39\ARHGAP25\KDM5B\CDC27\PTPN7\CNTN2\LOC419726\EDC3\PTPRU\PSMD5\UBE2R2\ATP6V1B2\PUSL1\BBS4\RHBDF2\STXBP3\EIF2S2\PARG\WBP2\PHKG1\CHCHD2\RGS2\RGS4\GULP1\TARDBP\PINK1\SCPEP1\KIF20A\ELL\PPP1R16B\GLUL\HAUS8\GPR174\ADAM10\PLG\LOC428693\LOC100859686\TAX1BP3\SHISA5\SMG7\LOC422179\TSEN15\MTFR1\VGLL4\DUT\ALKBH2\SLC25A36\JMJD4\STK25\PAFAH1B1\ERCC6\NOM1\TSSC4\POFUT1\MAPRE1\OAF\AP2A2\POU2AF1\KIF3A\CHM\DLG1\VIMP\RAB35\PRKAB1\RHBDF1\TMLHE\TUBB1\RAB22A\CCND1\MTMR8\SLC9A3R1\EIF4E3\SF3A1\PSMD10\DSTN\PRPF4\LEFTY2\SERPINI1\TMEM229B\PLEKHH1\LOC422448\LOC417848\CNOT2\BRMS1L\FAM98A\ACSL1\SCCPDH\C4H4ORF41\IL6\LOC417058\RAVER2\GLRX5\DCN\SCYL2\IGF2R\ARL1\ATF7IP\ACO2\CCR8\ARPP21\SFRP4\TARP\PWP1\MRPL24\TBL1X\LOH12CR1\LOC426218\MED6\KIT\SGCB\TNFRSF1A\TMEM68\MXD4\USP25\MMP16\C4H20ORF194\DPYS\LOC100858102\WDYHV1\DERL1\ASXL2\ZNF395\MTMR9\MUT\CD2AP\NCK2\SLC25A30\VAPA\CDK3\TPD52L2\SEPT6\ETV1\CENPH\ZNHIT6\PUF60\SOD1\STX7\NT5C3A\CLOCK\DNAJA1\WIPF1\TPCN3\CD320\TIMMDC1\

P<0.05	gga-miR-1552-5p_R+1	RASSF2\PM20D1\LOC100857226\WDTC1\CDC73\NFYA\SUB1\SFRP1\RGS9BP\SUCLG2\MMACHC\RAB5A\THRB\PTPLB\HIF1A\NUP153\BPGM\MMP16\DOPEY2\PTP4A1\HADHB\PPP6R3\EAF2\IREB2\TNFSF15\
P<0.05	gga-miR-1552-3p_R+1	YTHDC2\BNIP3L\C20H20ORF24\SNAP29\MAN2C1\SMU1\GPSM2\OGDHL\H3F3B\LIG3\NUBP2\HSPA9\SLC25A33\BOD1\TSR1\C26H6ORF130\DNAJC7\SLC12A4\SKP2\SLC35E1\CNOT8\DOLPP1\OPHN1\MTHFR\IVD\TSEN15\URM1\TAF1\C20H20ORF11\ARFGAP1\GCLM\STMN3\ZXDC\NOM1\KIAA0430\DEPDC5\PFKP\ARRDC4\KDELR2\ANKDD1A\TUBB1\C10H15ORF44\YME1L1\TRAP1\SNAPC5\RLIM\KIAA2022\DOK5\COMMD9\UBE2V1\RNF114\RINT1\CTDSPL2\NET1\SLC38A2\ITPKA\TYRO3\PPP1R21\EBNA1BP2\LCLAT1\RBM25\PRKCI\ETFDH\LXN\C4H4ORF27\KRR1\EIF2B2\ATPAF1\GTF2A1\LRRC72\FAM126A\SCARB2\MYLK\ACO2\MAD2L1\RPL7L1\SFRP4\FBXO34\WDHD1\ISCA1\ATP9B\C12ORF45\SERPINB6\TULP3\LRRC16A\IMPA2\PTPN2\VPS45\PHC1\FGFBP2\NAT10\CERS3\SGTB\C6ORF170\WDR1\FAM26E\EVC\ZFAND5\TAF13\TCEA1\GNE\TAGLN3\FNTA\SDR16C5\ADD1\PHF20L1\KLHL15\PRDX4\FBXO32\SNTB1\NCK2\SPRY2\CLN5\SPRYD7\ACAT1\ANKRD42\UQCRH\SPDYA\LYN\VMO1\SEPT6\UBE2N\GATA2\FAM49B\EBAG9\E2F5\C3H2ORF43\SLC2A9\NOA1\PTGR2\DNTT\DNASE1L3\TRIB2\CASQ2\SLC10A7\NELL2\EMB\PGRMC1\UPF3B\CD72\NUCB2\USP7\TIMMDC1\
P<0.05	gga-miR-155_R+1	C22H20ORF30\SLC23A2\KCTD9\SPPL2B\PGM2\FBXW2\NR0B2\CSNK2A2\C20H20ORF24\HERPUD1\SNAP29\SEPT9\LRIG2\WWC1\ORAI2\UBE2J2\PRKCD\C15H22ORF25\RFFL\TSPAN14\RNF123\PAIP2\DNAJC12\ETFA\CCL20\TRPC4AP\ERLIN1\UBIAD1\RAB5C\KIAA1191\BARD1\CLINT1\KRT20\IQCA1\MFAP3\SLC25A16\ITM2A\PNRC2\DHRS3\COL13A1\VPS13D\GOSR1\TNFRSF1B\RAB27A\CYP3A4\ZMYND8\FAM214A\SET\RGL1\C17H9ORF16\H2AFY2\FLCN\LSM14A\DUT\PPARG\FGD3\RRP1B\SNX2\ADIPOQ\RNF141\DENND6A\C20H20ORF11\PLS3\PITPNM3\PIK3C2A\PAXIP1\DNAJB6\ANKRD1\LOC419755\LRRC28\PGS1\MTPAP\SUDS3\LRRC59\IQCD\KCTD2\UBE2V1\RNF114\MOB4\FBXO18\ATG4A\F2\MCTS1\LOC424109\UGP2\FXR1\SPTBN5\TSPAN12\VMA21\PNLIP\LIN9\C6H10ORF46\DEGS1\PRKCI\CAV2\C2H7ORF64\THAP5\TLK1\GPATCH2\C5H15ORF29\GPR26\DYNC1I1\PRPH2\SCFD1\RAP1B\EDNRA\PPP1R12A\ADCK1\NRD1\HEATR5B\LRRC72\ACTA1\C5H14ORF129\CHN2\WLS\MARK3\ERCC3\LZTFL1\PPP1R17\ITGA9\GCAT\BDH2\IYD\JARID2\ADAM12\AKR1D1\TRIO\DERA\EXOC1\PTPN2\PCYOX1\KRAS\OCIAD1\PPFIBP1\SEPSECS\CC2D2A\CAST\PRRC1\ERBB2IP\METTL4\TNFRSF1A\ABHD3\WDR1\CYTL1\TRAF3IP2\HAUS6\SNX3\RNF4\MMP16\TMEM30A\IFNGR2\CCNE2\DDRGK1\DOPEY2\MORC3\CHAF1B\SLC25A32\MX1\LPIN1\REPS2\CYFIP1\C1H2ORF49\SULT1C3\SLC15A1\LMO7\POSTN\CRYL1\MMP13\VAPA\GZMA\GTPBP1\EBAG9\ETV1\CLN8\XRCC3\CHP1\HABP2\PLA2R1\TANK\TMEM141\LOC431660\RB1\COL9A1\RREB1\TAPT1\EGFR\AKT1\RNASET2\PSMC3\CYP24A1\EMB\TBC1D22A\GTF3C5\PELI1\EIF4A2\LOC419851\GRIN1\PLEKHJ1\LEPROTL1\
P<0.05	gga-mir-147-p5	ARID4A\RASSF2\ADAM28\SLC7A6\NCOA6\SNAP29\FAM18B1\RBM39\GLB1L2\ATP6V1B2\PTBP1\LOC415325\ACOX1\TMEM194B\MMD\MLX\GBGT1\SCYL3\RYR3\LOC100859426\MTHFR\PPIF\AP3M1\CES1\CDH5\KBTBD5\GATA5\WFDC1\RNF141\GCLM\HDLBP\TPRA1\TNRC

		6A\DDX46\PPP2CA\ANKRD1\HSPA8\CRTAM\MAP3K13\KDELR2\PSMD6\SALL4\NEBL\HN1\GGA3\SLC26A4\MRPS7\BCAP29\CEP41\F2\XRN2\BRD1\NUSAP1\C3H20ORF3\DSTN\SAMD13\PRPF4\FSHR\SFRP2\PRDX3\ETFDH\GORASP2\GAD1\GPATCH2\C1GALT1\LOC422442\TRAF5\TMBIM4\CAND1\TMEM184C\HSPA4L\GLRX3\RYR2\TRA2A\TSNAX\LEPROT\SERPINH1\RFTN1\DCN\NEDD1\NUP54\UMPS\DDX47\EXOSC7\ZDHHC3\PRDM5\FSHB\RAP1GDS1\NOL12\RECK\SLC41A2\RBM24\HSP90B1\ADAM12\TXNDC5\WRNIP1\ADCK2\SERPINB6\PIK3C2G\SPTLC1\EFHA2\CYB5A\LOC421049\PPFIBP1\SCNN1A\RELL1\ING4\LYSMD3\EPHB6\WASF1\HAUS6\ZDHHC23\BLZF1\SNAI2\IDUA\LOC421792\GPR89B\VCPIP1\UFL1\ACOX3\HMX1\ADD1\ADRA1D\WRB\MAOA\TINAG\PDK3\TAF2\HADHB\MOSPD2\GAL10\MGAT4A\TM9SF2\STK24\WDFY2\POSTN\MAB21L1\PAN3\N6AMT2\NOX4\MRPL48\GZMK\LOC418472\E2F5\CLN8\LOC395933\NSDHL\PTGR2\CAPN3\CHP1\AK6\KCNAB1\DCLRE1A\SMS\PIGN\EGFR\NT5C3A\SGOL1\CHRNA6\SLC10A7\WIPF1\PGRMC1\ARF1\NUF2\PLK1S1\C5H11ORF46\WRAP73\PDZD11\P4HA1\UBE4A\SNRNP40\
P<0.05	gga-mir-147-p3_2	GFPT1\RASSF2\STARD4\LSM7\PM20D1\ANKHD1\GATSL2\HERPUD1\SNAP29\CAMSAP1\E2F4\LOC415664\SDF4\DTWD2\EP400\GPR157\MMD\ERLIN2\BFAR\HDAC1\DNAJC7\ARPC3\P2RX4\APBA2\LOC101749801\FAM189A1\ZDHHC9\FAM114A2\TTPAL\TBCA\DIABLO\HVCN1\MAPK6\MYEF2\DUT\CLTC\RNF141\COX4I1\STK25\STMN3\COG7\CRTAM\TRA2B\KNDC1\RBM7\SPG21\TUBB1\NAT9\CD82\TRABD\NHLRC2\CFTR\DUSP10\EIF2S1\C4H4ORF27\LOC426599\AQR\GLIS3\TMEM59\MLLT4\CDC42BPB\ACAD11\PTPLB\DDX47\SPATA5\KIAA1109\SNAPC1\SLC38A6\STARD3NL\TAB1\GTDC1\ISX\DAP\ANKS6\ARNTL2\RELL1\CSTB\KIAA0907\PRRC1\SERINC1\TNFRSF1A\ALCAM\CD200\GRIN3A\TCEB1\PEX2\TMEM129\SLBP\TPD52\NT5E\MMP16\RAD54B\WRB\ST3GAL1\MYOM2\MOSPD2\DNAJC3\SLC25A15\ANKRD42\H2AFZ\SEPT6\POPDC2\EPM2A\NKAP\EFCAB11\DAB1\GLT1D1\POU1F1\CASQ2\NT5C3A\SGOL1\SLC10A7\KIAA1328\PFKL\TIMMDC1\
P<0.05	gga-mir-147-p3_1	ARID4A\RASSF2\ADAM28\SLC7A6\NCOA6\SNAP29\FAM18B1\RBM39\GLB1L2\ATP6V1B2\PTBP1\LOC415325\ACOX1\TMEM194B\MMD\MLX\GBGT1\SCYL3\RYR3\LOC100859426\MTHFR\PPIF\AP3M1\CES1\CDH5\KBTBD5\GATA5\WFDC1\RNF141\GCLM\HDLBP\TPRA1\TNRC6A\DDX46\PPP2CA\ANKRD1\HSPA8\CRTAM\MAP3K13\KDELR2\PSMD6\SALL4\NEBL\HN1\GGA3\SLC26A4\MRPS7\BCAP29\CEP41\F2\XRN2\BRD1\NUSAP1\C3H20ORF3\DSTN\SAMD13\PRPF4\FSHR\SFRP2\PRDX3\ETFDH\GORASP2\GAD1\GPATCH2\C1GALT1\LOC422442\TRAF5\TMBIM4\CAND1\TMEM184C\HSPA4L\GLRX3\RYR2\TRA2A\TSNAX\LEPROT\SERPINH1\RFTN1\DCN\NEDD1\NUP54\UMPS\DDX47\EXOSC7\ZDHHC3\PRDM5\FSHB\RAP1GDS1\NOL12\RECK\SLC41A2\RBM24\HSP90B1\ADAM12\TXNDC5\WRNIP1\ADCK2\SERPINB6\PIK3C2G\SPTLC1\EFHA2\CYB5A\LOC421049\PPFIBP1\SCNN1A\RELL1\ING4\LYSMD3\EPHB6\WASF1\HAUS6\ZDHHC23\BLZF1\SNAI2\IDUA\LOC421792\GPR89B\VCPIP1\UFL1\ACOX3\HMX1\ADD1\ADRA1D\WRB\MAOA\TINAG\PDK3\TAF2\HADHB\MOSPD2\GAL10\MGAT4A\TM9SF2\STK24\WDFY2\POSTN\MAB21L1\PAN3\N6AMT2\NOX4\MRPL48\GZMK\LOC418472\E2F5\CLN8\LOC395933\NSDHL\PTGR2\CAPN3\

		CHP1\AK6\KCNAB1\DCLRE1A\SMS\PIGN\EGFR\NT5C3A\SGOL1\CHRNA6\SLC10A7\WIPF1\PGRMC1\ARF1\NUF2\PLK1S1\C5H11ORF46\WRAP73\PDZD11\P4HA1\UBE4A\SNRNP40\
P<0.05	gga-miR-147_R+1	E2F4\MSTN\SCPEP1\POLR1E\RBM7\EPC1\GNAI1\DYTN\DUSP10\TCERG1L\NAGA\NUP153\C1H12ORF11\FKBP4\SLC17A5\DMD\FAM175B\LIMK1\RCC2\
P<0.05	gga-miR-146c-5p_4ss18GA20AG22TG23GT	RFK\LAMTOR3\STARD4\KCTD9\LAPTM5\FBXW2\TERF2IP\STRBP\SAMHD1\RRAGC\SLC16A1\PRKRIP1\PRPSAP1\TNFRSF18\VAV3\NCBP1\PLEKHB2\DTWD2\ADPRHL2\PMS1\SEC16A\ACAA2\PHACTR4\AARSD1\SUGP2\MMD\MLX\FBXL8\KIF20A\SUB1\ACSBG1\UBIAD1\CDK2AP1\UCK1\XPR1\MFAP3\STK4\ITM2A\TTPAL\RALGPS2\MCU\SGPL1\IVD\AIFM2\PLCB2\PPIF\PEMT\VPS53\NUP210\RNPC3\SASS6\APPBP2\USP32\LOC768701\EIF4G2\ABCD3\ABHD6\TNFAIP1\TRAF7\GCLM\COL20A1\DCAF12\CHRNA4\MTF2\LOC416931\PRKAG2\TGFBI\CHCHD4\LOC422249\SEMA3A\PDE6C\MAPRE1\MRPL46\KIF11\AP2A2\MARCH5\RAC1\SVIL\BCL6\RAB22A\TMEM104\MAGT1\SUMO2\UBE2H\GGA3\MOB4\DDB2\NAPEPLD\LRRC17\F2\SUMO1\SAMD13\MDH1\DEGS1\SLC25A13\ATP6V1D\LOC426599\HCLS1\C5H15ORF41\LOC422448\MYST2\EAPP\MCFD2\HP1BP3\MBNL1\CMPK1\UFSP2\QPCT\SCIN\CHGA\ERO1LB\HSD17B11\DFNA5\RFTN1\BAG5\PPP1R13B\SCYL2\HEBP1\ATF7IP\RAB32\STK17A\LOC101750080\ALDH1L2\C12ORF45\SLC41A2\TXNRD1\NUP153\SLC37A3\ZBTB2\WNK1\STRAP\SLCO1B1\ANKRA2\VIP\NPEPPS\MED6\SGCB\NUP50\SCNN1A\PRRC1\LOC427201\LAMA4\ZBTB49\NSG1\GNE\TMEM175\TAGLN3\GJA5\PDZK1\CHMP2B\ZCCHC9\HSPA13\RNF4\RARS2\MMP16\PLEKHF2\LOC396380\EFR3A\FDFT1\CYP39A1\C1H2ORF49\ARGLU1\BORA\ZC3H13\SPRYD7\N4BP2L2\PPME1\MRPL48\GDPD5\TPD52L2\LYN\RFX8\INVS\PSEN1\PLEKHA3\HDAC4\ALDH1A2\IRAK2\POU1F1\DNAJA1\LOC769743\TBP\MYSM1\PRKAG2\FASN\DDOST\EXOSC2\EIF4G2\REL\YOD1\P4HA1\HSF1\LOC422654\
P<0.05	gga-miR-146b-3p_L+2	RASSF2\RPS6KA1\CAMTA1\GUCA1A\PSMD5\C15H22ORF25\GBAS\MMD\DDX5\LOC101749801\PLOD1\COPS3\PPIC\PAFAH1B1\ABTB1\MAPK8\RTEL1\ABCC1\SPG21\CHIC1\MAGT1\COMMD5\NOXA1\YPEL5\ACBD3\LOC424620\BRP44L\RALB\ARL1\ENPEP\FANCC\TDP2\SRSF7\FGFR1OP2\USP48\ANKRD52\EDARADD\FGFBP2\C3H6ORF186\KIAA1524\IDUA\USP45\CCNH\RAD21\PRIM2\NRBP1\BORA\NOX4\FAM168A\E2F5\TERF1\INCENP\RNF7\ANLN\ACSF2\ATP2A2\ADAL\LMAN1\REL\PLEKHJ1\
P<0.05	gga-miR-1467-3p_L+1	ARMC1\DHDDS\LSM7\GALK2\SRSF3\CDC27\RBBP5\ZMPSTE24\ARMC8\SRRM1\LOC428335\FAM18B1\SEPT9\JMJD6\ARVCF\COMMD10\ATG12\SMN\HSD17B7\WDR59\CTGF\RGS14\EIF4EBP1\VAMP4\CRLF3\GOLGA7\ZBTB8OS\MTG1\TRRAP\USE1\POLB\SMARCE1\APBA2\AP1M1\TWF2\CAPZB\STK4\FAM20B\ASB6\CYP3A7\SGPL1\LOC100858951\P2RX5\ULK2\AP4E1\MTFR1\BCMO1\TADA2A\SNX12\RPN1\LOC100858792\KLHL13\GALNS\HTATSF1\SRPRB\NDE1\KIAA1024\PROCR\OAF\KIF3A\LIMK2\SPPL3\ITGB1\FADS2\EDNRB2\ACBD5\STX16\MTMR8\USP12P1\TMEM104\NMRAL1\KCTD2\CALML4\TMEM180\LOC424943\MERTK\NEURL\ATG13\PDIA3\GRIA3\GTF2H4\KIF16B\APLF\DCUN1D1\TSPAN12\VMA21\AMDHD2\CRAMP1L\PRKCI\GXYLT1\ZNF650\GPATCH2\ARID2\HCLS1\TMEM206\TRAF5\MYST2\SRP5

		4\PHF17\HECTD3\NAP1L1\DHCR24\ITPK1\NID1\DEPDC1\ZRANB2\SPATA5\CCNA2\PCDP1\ALDH1L2\TXNRD1\C1H12ORF48\GCM2\TXNDC5\MKRN1\SLCO1B1\EXOC3\LOH12CR1\STX17\MAP3K5\VPS45\SCNN1A\M6PR\ATN1\PTPN6\KIF2A\USP14\HEXB\RABL3\C3H6ORF186\ZDHHC23\TIPRL\SDR16C5\ZFP92\MAP3K7\PMP2\CA2\B3GAT2\PRADC1\ST3GAL1\DDX3X\GCLC\PDK3\FBXO32\PHKA2\TUBGCP5\CARS2\MZT1\HMGB1\MPHOSPH8\PPME1\LCMT2\CD47\DPT\GTPBP1\LOC420220\TERF1\CLN8\C2ORF47\NEO1\PAFAH2\RIF1\NFIA\GLDC\PGRMC1\CD151\ATP2A2\LDHA\DDX19B\EIF4G2\PTPN22\PCSK7\HSF1\COPA\SLU7\RPUSD4\
P<0.05	gga-miR-144-3p_L-1R-2	C22H20ORF30\RASSF2\STARD4\KCTD9\CHMP7\ERMP1\PGM2\GALK2\ZBTB48\PPP1R8\TPRG1L\MAN1C1\NCOA6\DBR1\LOC428335\CAMK1G\FAM18B1\COX10\STMN1\SSU72\SLC16A1\PTBP1\STXBP3\WDR33\EIF2S2\C15H22ORF25\GPSM2\HS6ST1\FBXW11\TMEM194B\MATR3\DNAJC18\LZIC\WDR5\ATR\DNAJC12\TARDBP\PRDX6\MMD\EIF4EBP1\SUB1\COMP\RAI14\ELL\ESRP2\ZNF207\SKP2\FN1\UBLCP1\SALL1\RICTOR\GTF2A2\ARPC3\PBLD\CNOT8\PHKB\AKR7A2\CAPZB\GPR174\NADSYN1\TTPAL\RCAN3\HVCN1\LOC100858951\PDCD5\MAPK6\CLIC2\STAU1\MAPKAPK5\NOL8\SCG3\COPS3\MYEF2\LSM14A\PLAU\CUL3\COL11A1\TEKT4\CDH11\GNG13\LPPR5\NONO\EIF4G2\DENND6A\SPATA5L1\NLK\ARFGAP1\DCAF12\PAFAH1B1\CNOT6\GORASP1\DDX46\CAMLG\PANK1\MNX1\PDE6C\KIF3A\MARCH5\CREM\KDELR2\ACOX2\ITGB1\PTPRG\ATP6V1G1\ZEB1\CADPS\PSMD6\ANKRD40\CTSZ\STX16\SUCLG2\CHIC1\TRAP1\APIP\FANCL\ATP7A\UBE2V1\SPATA2\MOB4\ORC5\AOX2P\PGGT1B\CTDSPL2\NAPEPLD\ITPR1\PHTF2\SLC38A2\TTL\GTF2H4\TRABD\APLF\SEC23B\SAMD13\UGP2\ZZZ3\JAG1\PIF1\GFRA1\CYP51A1\SMOC1\ETFDH\C2H7ORF64\CCDC132\ARID2\C5H15ORF41\IFNG\SMARCA5\SMAD1\FAM177A1\HSPA4L\ZDHHC17\ALDH18A1\GTF2A1\PRPF38A\THSD7A\NEIL3\TMEM59\ANKMY2\UBR7\UNC79\CBX3\LEPROT\C5H14ORF129\SEC31A\WDFY3\NGLY1\TECPR2\SLC44A5\OSBPL10\NEDD1\BRP44L\PECR\NUP54\POLDIP3\MYOZ2\ST13\ARPP21\MUDENG\BMPER\CCNT2\STK17A\ISCA1\C22ORF28\RBM24\IRF4\SLC37A3\VPS4B\YARS2\MARCH6\LOC418169\CLPTM1L\GABBR2\PLEKHO1\GMNN\DNAJC16\EXOC1\MPPE1\CEP76\C2H18ORF19\KIT\ARNTL2\PPFIBP1\TTLL12\ADHFE1\CORO6\M6PR\EDARADD\PI4K2B\CSTB\MYH10\HORMAD1\MEGF10\PRRC1\DEPDC1B\SERINC1\C6ORF170\CLUL1\ANKDD1B\F2RL1\MSX1\RLN3\REV3L\TMEM39A\C3H6ORF203\GNE\TMEM175\TPD52\SYNCRIP\CYB5R4\TP53INP1\EIF3H\EEF1D\WDR4\KHDRBS3\PHF20L1\PTP4A1\DMD\ZNF451\TINAG\ASAP1\CALB1\RBBP7\ATP4B\TNFSF13B\SLC15A1\DZIP1\SUCLA2\FRY\CRYL1\MMP13\PPME1\LOC419074\VAPA\CD9\UBE2N\CERK\TERF1\PTPN3\WDR48\HCRTR2\EPM2A\CZH9ORF80\LEPR\NFE2L2\STOM\NADK\STX7\DYNC1H1\LUC7L3\GLDC\SLC10A7\WIPF1\GABPB1\PARP1\CNTN1\TGM2\PELI1\MYOF\EIF4G2\ACLY\C5ORF5\REL\CR1L\YOD1\PDZD11\GSPT1\
P<0.05	gga-miR-142-5p_L-2R+1	RFK\SPPL2B\NEK6\DBR1\PBRM1\MAPKAPK3\NCKAP1\SLC16A9\ACSS2\ESRP2\RHOT1\SUN1\GEMIN5\ITM2A\RCAN3\H2AFY2\ISY1\HDAC11\SLC25A36\PIK3CB\RHOG\MED12\RNF141\YTHDF1\GORASP1\HPRT1\PIK3C2A\PAXIP1\DDT\PDPK1\LOC422249\FOPNL\PDE6C\YWHAH\RBM7\SYNPR\APIP\SPAG6\STK17B\NEBL\PIAS1\ANKRD44\CELF1\CTDSPL2\DDB2\VBP1\SHOC2\ATP6AP1\MDH1\ATP6V0C\PR

		KCI\GATAD1\SEC23IP\C2H7ORF64\DYNC1I1\ANGEL2\NENF\RASSF3\RAP1B\INTS10\GEMIN2\FBXO8\IGF2BP3\CBX3\HIBADH\VRK1\CCNK\PPP2R5C\KAT2B\CRYZ\PTPLB\MYLK\BFSP2\UBN2\TRPC3\ST13\LRRFIP2\TDRD7\SOUL\ISCA1\LOC100857286\JARID2\TXNDC5\CLPTM1L\ANKS6\KIT\KRAS\KIAA0930\FBXL5\MBLAC2\LYSMD3\ZUFSP\CRHBP\C1H3ORF26\ATP5I\HACE1\MAEL\RNF4\MORC3\DCAF13\PTP4A1\PIGA\RASA3\TNFSF13B\LIG4\ARGLU1\RNASEH2B\TPTE2\UFM1\ZAR1L\CERK\EAF2\SPECC1L\CRABP1\NADK\RREB1\PDLIM5\SLC1A2\ATP2A2\NUCB2\SPTAN1\
P<0.05	gga-miR-142-3p	FANCA\CCDC47\COX10\GUCA1A\RAB14\BMPR1A\FAM168B\NBR1\RHO\TARDBP\SLC16A9\KIF20A\SUB1\COMP\LPCAT2\OSMR\ITM2A\ZMYND8\PTGS2\AP3M1\USP54\KLHL18\DENND6A\TNFAIP1\ZFAND4\C14H16ORF42\VDAC1\TH\TSPAN6\CHID1\TLR4\VAPB\TMEM104\EIF4E3\TRPC5\ACTR1A\C15H12ORF49\ABHD12\NELF\WASL\IDH1\C6H10ORF118\GANC\LCLAT1\CDC42BPA\IFT140\SLC39A9\ACADSB\HECTD1\EDNRA\CFL2\FAM177A1\ABCD4\GTF2A1\GPX7\THSD7A\ATXN3\LGMN\ASB5\EPYC\DEPDC1\OSBPL10\NEDD1\MAP3K4\COX18\ATF7IP\QSER1\HECW1\TAB2\NTRK2\PWP1\ATP9B\LOC418170\CLPTM1L\PDK1\EZR\SLC2A12\DCUN1D4\KIAA0930\RELL1\MYH10\TNFRSF1A\SS18\LNP1\CLTA\CD200\CHMP2B\MMP16\WRB\SLC25A32\TSTA3\SIK1\EGFL6\KDELC1\HMGB1\KIAA1609\LOC770433\C3H2ORF43\INCENP\TJP1\APP\PCMT1\LUC7L3\KIAA1328\GFOD2\PDZD11\
P<0.05	gga-miR-140-5p_L+1	CHMP7\GALK2\LAPTM5\MYO1D\STX12\HYAL1\LOC100857226\GAPVD1\LDLRAP1\NR5A1\SRRM1\MECR\CCNL2\LOC416777\SLC22A7\CAMSAP1\TNFRSF18\PTBP1\AP2B1\SNX22\TMEM194B\CDC73\RFNG\MMD\CRLF3\RAB5C\LOC425137\GLUL\LOC101749631\WDR70\APBA2\PBLD\RPL23A\ADA\ADAM10\COL18A1\FAM214A\PPP1CC\CTNS\PEMT\FBN1\PLAU\SERPINE2\WDFY1\RBP2\SNX2\ADAMTS18\JMJD4\HCCS\EIF4G2\TFIP11\SNX12\NLK\C20H20ORF11\SBF2\ACAA1\LOC420419\MYD88\OXSR1\AQP8\SLC13A5\GSTT1\C12H3ORF19\TCF7\TBC1D12\ITIH2\COQ7\FAM174B\PLTP\PPP1R2\ACADS\ATP13A4\PEBP1\RAB22A\SUCLG2\SNAI1\SLC39A13\SRPK2\ERLEC1\AOX2P\ACP2\ORC2\ATG4A\FAM185A\PHTF2\CASP8\SLC38A2\LOC417253\DSTN\MDH1\FXR1\ETFDH\TMEM229B\C5H15ORF29\ACADSB\CAND1\MYST2\ARHGAP10\VLDLR\MFSD8\KRR1\TM4SF18\BIRC6\EPS15\BTF3L4\TFB2M\AGA\SCIN\DFNA5\ROR1\GLRX5\ARHGAP24\CHN2\PHF10\SCARB2\ACAT2\IL8L1\TMC2\THSD7B\WDSUB1\ISCA1\C22ORF28\TGFBR1\EEF1E1\IRF4\DENND2A\PRMT8\UBA3\PPAT\PARVB\RELL1\LYSMD3\ERBB2IP\WDR3\DCBLD1\HGD\B4GALT6\GPR161\OSTM1\TRIM14\TMEM68\IDUA\GPR89B\CASP8AP2\TCEB1\HSPA13\ADD1\NT5E\IMPG1\IFNGR2\SLC25A32\DENND3\PDE9A\PHF20L1\MELK\FAM49A\ASMT\LMO7\NUPL1\KIAA1609\DNASE1\TFEC\POT1\ABHD5\ETV1\FAM175B\ARHGAP19\TJP1\CRMP1\RAD17\NOXRED1\TBP\CDCA7L\SNX13\MYO5A\KIAA1328\TLN2\NUCB2\SLCO4A1\EIF4G2\PYGB\YOD1\GFOD2\ZIC1\LAS1L\
P<0.05	gga-miR-140-3p_L+1R+1_1ss2	C22H20ORF30\LOH11CR2A\PRNP\SLC25A46\ITGB3\CHMP7\SPPL2B\LONP1\CYB5B\RBBP5\CACNA1S\DHODH\KDM1A\CSNK2A2\TAT\NUDC\FAM125B\APLP2\EDC3\CASP9\COX10\PLLP\TTC1\CCNL2\WDR45L\CWC25\SEPT9\DGKD\UQCC\C19ORF6\NR5A2\SORD\HSPA9\SUM

	2CA	F2\CRYM\ZMYM4\GJA4\ASL2\C19H17ORF85\WDR5\HSD17B7\DNAJC9\KIF1B\INPP5K\NUDT3\RFNG\BNIP1\TARDBP\MLX\PEF1\C26H6ORF130\EIF2B1\ERLIN1\EIF3I\GBGT1\KIAA1191\BSDC1\GFI1B\LOC101749631\TMEM38A\TARSL2\ASB18\SRSF10\TOR1A\ADA\VPS26A\TAF9\ADAM10\MORN3\TSPAN15\TNFAIP8L1\RALGPS2\P4HA1\COL18A1\ASB6\PPP1CC\AR\P2RX5\CTNS\ENDOG\CPSF4\CLIC2\DHRS7B\PSAP\C17H9ORF16\IARS\RASD1\TAF4\WDFY1\FUT11\SLC25A36\HHATL\IBA57\BRCC3\HELLS\ARHGEF3\TPST2\EMC8\CHRNA4\IPO7\RAP1GAP2\MLST8\LOC420419\MYD88\LTC4S\RRAS2\BTBD1\TNRC6A\WNT8A\ARFRP1\ZNF326\PIK3C2A\EARS2\COL6A2\PAXIP1\PDPK1\C9H21ORF2\TNNT3\RHCG\TMEM41A\OAF\TAF3\CBL\POU2AF1\LIMK2\SPPL3\RBM7\LOC100858827\RMI2\HRASLS\CADPS\LPP\PRKAB1\ANKRD40\C7ORF26\RRP12\SLC9A3R1\KIAA2022\APIP\STK17B\PTPN1\TSEN54\NDUFS3\TYW5\MADD\GRAMD3\ARFGAP2\ATG4A\SUMO1\CRY2\NDUFS1\GTF2H4\ACSS1\SHOC2\UGP2\EPCAM\VMA21\GUCY1A3\RXFP1\BAG3\MIA3\PIGH\SCHIP1\MYO3B\ATPBD4\RAP1B\CRIPT\KIAA0391\GEMIN2\CAPN1\FBXO33\TSPAN1\HERC3\GLRX3\EIF2AK2\PPPDE1\SCIN\ITPK1\HDAC9\STK39\SIPA1L2\ACTA1\SERPINH1\NEDD1\METTL6\MRPL3\MTHFD1\CCNA2\NAGA\CCK\TMC2\METAP1\L2HGDH\EIF3L\TNFAIP6\FANCC\RIC8B\C1H12ORF48\IRF4\MKRN1\DNM1L\USP18\LOC100859438\PCM1\GTF2H5\SLMO2\LOC421049\RELL1\CC2D2A\LYSMD3\KIAA0907\FAM172A\CAST\MSX1\HAUS6\BOC\C3H6ORF203\CD200\CD247\GPR89B\HSPA13\PEX2\TMEM30A\IFNGR2\RUNX1\CHAF1B\U2AF1\LOC396380\GSTA\KLHL15\PDIA6\ACE2\MTMR9\MYO16\LIG4\ELF1\WBP4\C1H13ORF15\MED4\HSPH1\DDX10\MED17\UQCRH\COTL1\KLHL5\PSMD3\C1H21ORF33\INVS\NKAP\SLC25A14\PTGR2\INCENP\C7H2ORF76\MCCC1\NDUFA10\RPE\RNF7\FYB\EXOC7\KPNA1\LOC419404\TAPT1\PDLIM5\TMED8\HMOX1\HOXA11\IRF2\ATP2B1\CUL2\ARSB\ZBTB34\CNTN1\IRF7\TRIM59\EIF5A2\COG5\P4HA1\KIAA0319L\BET1\
P<0.05	gga-miR-138-5p_R+7	RFK\SPR\TIMM17A\BMP10\ARHGAP25\BF1\YTHDC2\BNIP3L\RDM1\CAMK4\HMBS\ITGB3\LIN28A\CHTF8\FBXW2\KIF18B\LHFPL5\POM121\GAPVD1\GJC1\MAP2K2\SF3A3\ATG16L1\LOC417345\E2F4\GFRA2\JMJD6\VAV3\TMEM194B\ENO1\COG4\SBNO2\TAF11\RSL1D1\BRI3BP\JMJD1C\PRDX6\AMFR\EIF4EBP1\TSR1\LGALS2\TAF8\ZNF511\ATIC\VTN\TMEM38A\KRT15\SYS1\TLCD1\GPR174\BET1L\SLTM\SIRT3\MORN3\MCU\C15ORF23\C14H17ORF103\MPRIP\MYEF2\C17ORF39\LSM14A\CENPI\GHRHR\CYB5R2\MRM1\GRHPR\LOC768701\KLHL18\SLC9A3R2\KSR1\MTF2\PASK\MST4\HPRT1\COL6A2\GALNT11\MLL3\CTU2\K123\SLC25A48\OSBPL5\SLCO2A1\DNAJB6\CCM2L\ANKRD1\CLMP\TNNT3\ZMYND11\IDE\PFKP\SDR42E2\NCOA5\PXK\KDELR2\CABP1\LPP\CD3E\TMLHE\STK32A\NAT9\CD44\BCL11A\KCTD2\UBE2V1\COPG2\ERLEC1\CTDSPL2\FBXW8\STRADB\ITPKA\TRABD\NEUROD1\JAG1\ADCK3\LIN9\BAG3\EXD2\C2H7ORF64\TMEM229B\GPATCH2\KANK1\AARS2\MMACHC\GTPBP2\BTF3L4\PPPDE1\ITPK1\HSD17B11\ACTA1\SERPINH1\PPP2R5C\BRP44L\BAG5\ILF2\ESR2\HIF1A\SCRN2\PCDP1\SOX10\BDH2\TMEM263\DEK\TXNDC5\MKRN1\DAP\PLEKHA5\RHBG\TBL1X\AGMAT\PPAT\NETO1\PTPN2\KDR\SSPN\FAM76A\FGFR1OP2\PPFIBP1\MAN1A2\MRPL10\CSTB\ASH1L\EPHB6\TNFRSF1A\DAZAP1\ARL13B\GRIN3A\CPA6\PHF20L1

		\PTP4A1\GCM1\PDK3\FBXO32\TAF2\KLF11\NOL10\PPM1G\ASXL2\MEP1A\DNAJC3\WBP4\NBEA\N4BP2L2\C1H11ORF54\PPME1\SUV39H2\B4GALT1\GTPBP1\CERK\E2F5\TMEM70\SUCLG1\XRCC3\STARD5\ALDH1A2\GLT1D1\P2RX1\NCF1\LRIG1\RBM6\TMEM123\ERICH6B\EGFR\RIF1\SPG11\USP37\BPNT1\GHRHR\LOC769242\SLC47A1\ENTPD7\PNPLA7\LCN8\SNCB\PFKL\ATCAY\
P<0.05	gga-miR-137-3p_L+1	KDM5B\DCAF7\MFSD4\WNT11\FABP6\SSU72\NCAPD3\CNTRL\SLC22A7\EIF6\VTG2\DTX2\SMU1\C15H22ORF25\PLEKHB2\SNX22\XRN1\RFWD3\SARDH\BECN1\REXO4\C17H9ORF96\DNAJA4\CDHR2\FN1\FEN1\HEATR2\C21H1ORF144\SDC4\SLC25A16\YWHAB\NADSYN1\PALD1\HVCN1\P2RX5\PPIF\CDK9\DPM2\FAM96B\WDFY1\FUT11\SYNGR3\HPS4\SLC30A4\DR1\TMED5\SLC22A13\MYD88\ABTB1\GORASP1\MST4\TGFBI\OSBPL5\SLC6A6\TNMD\NCBP2\USP28\BLNK\PCSK6\PTPRG\CADPS\TUBB1\RBM27\LOC416354\SUCLG2\TRAP1\SNAPC5\GGA3\SLC39A13\NR1H3\NAPEPLD\CIB1\FGL2\F2\RALGAPA2\ITGA8\NEUROD1\MND1\TP53BP2\TMEM229B\CCDC132\RAB3GAP2\GXYLT1\YAF2\LXN\LOC426599\FMN1\HCLS1\ELOVL1\BAZ1A\GLO1\ANGEL1\FAM98A\GLRX3\PDZK1IP1\CMPK1\LEF1\PRPF38A\PPPDE1\UNC79\RAVER2\HIBADH\VRK1\RFTN1\EPYC\KERA\NKIRAS1\MARK3\ACAD11\MGP\ENPEP\NCKAP5\SAV1\TDRD7\SLC9A3\TMEM263\C1H12ORF48\STRAP\CNOT7\LTV1\VPS45\FBXO40\ITPR2\TEC\BIK\TTLL12\LIMCH1\PI4K2B\GPR98\MAP3K1\NLN\SPAG17\MOCS2\LYAR\SS18\TMEM175\ACOT12\TCEB1\CTBP1\CYB5R4\HMGN3\RAD54B\IFNGR2\DONSON\SETD4\OXR1\SIK1\PRIM2\ANXA13\DDX1\CENPO\GAL10\GTPBP6\RP2\C1H2ORF49\TBC1D4\LCP1\FRY\IFT88\CRYL1\FZD4\ARL6IP5\C3H2ORF43\PTGR2\INCENP\ATE1\DNTT\PPP4R2\ALDH1A2\WDR82\C4H4ORF52\RNASET2\SLC12A2\LPL\CDCA7L\USP37\CNTN1\YBX1\TMEM120B\ACTL6A\WDR43\
P<0.05	gga-miR-135a-2-3p_L-1R+1	C22H20ORF30\BTN1A1\SLC25A1\ZAP70\SDF4\AQP3\GPR157\CREB3\LZIC\INPP5K\MED23\SERPINF2\DDX49\COMP\GOLGA7\SLC6A2\LOC101749631\PRRC2B\FNBP1\OIT3\TNFRSF1B\CLIC2\TGM3\SLC25A36\BRCC3\TNFAIP1\BOK\C12H3ORF19\PDE6C\TOLLIP\KIF3A\CREM\FGF1\LARS\KMO\ITGB2\ACBD5\STK32A\TIPIN\SNAPC5\SALL4\TMEM164\C15H12ORF49\ENTPD2\CTSO\LOC417800\VEPH1\C1GALT1\XPOT\FAM177A1\DPH2\CAPN1\MMACHC\XPO5\GYG1\TRA2A\EPYC\ACAT2\IRF4\BCL2\SORBS2\ANP32E\TDP2\CLUL1\F2RL1\C3H6ORF186\SNAP91\RAD54B\TAF2\MSL3\C1H2ORF49\LECT1\SEPT6\SLC25A14\KPNA1\CNTN1\LIN7B\GNB1\
P<0.05	gga-miR-1329-5p	RFK\FAM195A\ARMC1\GFPT1\ADORA1\BNIP3L\KCTD9\RPS6KA1\ITGB3\C20H20ORF24\KBP\PPP2R3A\SAMHD1\SRRM1\CLTCL1\GIGYF2\RRAGC\NCAPD3\ANP32B\INPP5B\UBE2R2\ODZ2\MRPS34\VAV3\ARVCF\GOLGA3\RFFL\H3F3B\HIBCH\AP3S1\PAIP2\PCDH12\PIK3CD\XPO6\FRZB\TARDBP\CCL20\PARN\TMED2\ELL\ESRP2\C18H17ORF75\GOLGA7\BARD1\SGK2\SKP2\LPCAT2\FN1\GLUL\LOC101749631\C21H1ORF144\TMEM38A\LRRFIP1\TIMD4\SMARCE1\PBLD\PPT1\MFAP3\FUCA1\DDX50\GRHL3\TNFAIP8L1\SGPL1\HVCN1\LOC100859686\ZER1\RGS9BP\MAPK6\PPIF\FAM129A\FLCN\LOC768735\CEBPG\CLTC\CKLF\VCAM1\TK2\SLC25A36\PPM1D\RBP2\BCMO1\PDLIM1\PIK3CB\CCDC12\ST6GAL1\ABCD3\DENND6A\C20H20ORF11\LOC422214\GCLM\PAFAH1B1\MYD88\GDF2\ABTB1\BTBD1\GORASP1\XYLB\PRKAG2\KLHL3\EHHADH\KIF11\AP2A2\KIF3A\MARCH5\FAM174B\LIMK2\RNF185\POLR3E\ALDH1A3\PCSK6\ZEB1\SPG21\THO

		C7\SPAG9\RBFOX1\C10H15ORF44\STAT1\IQCD\CBLN4\VRK2\LAMB1\CHRDL1\NXT2\HSPD1\CELF1\SLC39A13\FBXO18\RELN\DDB2\ALDH7A1\ORC2\F2\PHTF2\CCDC147\XIAP\MCTS1\XPNPEP1\TYRO3\PLEK\IDH1\NDUFA5\SAMD13\DNAJC10\AK5\LBH\EHD3\C3H1ORF55\LOC100857197\DPF3\GUCY1A3\CAPZA2\ETFDH\IFRD1\SLC25A13\GAD1\SRP14\GPATCH2\C5H15ORF29\DYNC1I1\TMBIM4\IL22\ELOVL1\CRIPT\MCFD2\FAM177A1\BTBD9\INTU\NFKBIE\GTF2E2\GRID2\ALKBH1\ALDH18A1\BTF3L4\QPCT\PODN\PPPDE1\AGA\TMEM48\EXO1\ITPK1\UBR7\SP4\DFNA5\NTPCR\SIPA1L2\HNRNPA2B1\CBX3\GLRX5\LY75\GNPAT\EML1\SMOC2\PPP2R5C\DCN\CRADD\NEDD1\SEC24B\RALB\GUCY2C\TTC26\HIF1A\FSHB\FKBP9\VPS41\ATL1\PRPF39\TNFAIP6\WDSUB1\HNRNPK\ISCA1\C22ORF28\TGFBR1\JARID2\LOC420849\PRPF4B\TRIM24\IRF4\SLC37A3\PYROXD1\CLPTM1L\LDHB\TMEM106C\TDP2\CNDP2\LONRF1\EZR\SLMO1\PCYOX1\C2H18ORF19\DCUN1D4\TEC\PARVB\GUF1\MRPL51\ZNF384\C1S\LYSMD3\ARSK\RFESD\MAP3K1\EPHB6\MTX3\CZH5ORF28\CZH5ORF34\C6ORF170\GREB1L\F2RL1\CRHBP\B4GALT3\RORB\C3H6ORF203\TMEM68\NFKBIZ\IDUA\GRPEL1\UFL1\RNF4\TMEM129\CTBP1\SYNCRIP\FILIP1\PTDSS1\DCAF13\U2AF1\DDX3X\USP9X\PTP4A1\EIF2S3\KLHL15\PXDN\DERL1\SNTB1\FAM49A\RHOB\PPM1G\SYAP1\ATP4B\ABHD13\STK24\WBP4\RNASEH2B\ELMOD1\MTMR2\ANKRD42\RRM1\LCORL\UTP15\TPD52L2\PACSIN2\CERK\TERF1\PTPN3\RPRD1A\NR1D2\WDR48\HSF2\C6ORF192\IFNGR1\SUCLG1\CHP1\PPP6R3\PLA2R1\TANK\MCCC1\PPP4R2\LOC415708\DENR\NCF1\NADK\PUM1\NUFIP1\ZFAND1\CDC5L\CASQ2\EGFR\CRTAP\PCMT1\TBP\CHRNA6\REEP5\TEF\TMPO\GABPB1\PRKAG2\CYP24A1\PGRMC1\ARF1\LDHA\NUCB2\PDE3B\ZNF488\CYP2H1\CACNA1B\IRF6\GNB1\PDZD11\COPA\SLU7\
P<0.05	gga-miR-1329-3p	SPPL2B\FAM125B\AP3D1\FAM86A\RALGPS2\GOSR1\SLC13A3\TMEM116\WSCD2\FAM96B\GORASP1\RALGAPA2\WDR65\XPO5\PAPSS1\URB2\MAP7\USP14\SETD4\LECT1\SPRYD7\UNC5A\SYT4\TBCD\
P<0.05	gga-miR-130b-5p_R-3	RFK\ARHGAP25\RASSF2\ADAM28\DCAF7\LOC426699\C11H16ORF80\LDLRAP1\SNAP29\MYBBP1A\PI4KA\B3GNTL1\MIB2\PPIL2\UBE2R2\EIF6\CHMP4B\TSPAN14\C10ORF58\CHCHD2\RAN\POLR2E\C19H17ORF85\TFAM\MED23\LRRC45\BNIP1\BRI3BP\FBXL8\GNA11\HDAC1\TM2D2\KPNA6\LOC425137\SLC6A2\BPTF\USE1\CHD9\PPT1\IQCA1\CAPZB\TAF9\SIRT3\ADAM10\FAM20B\TBCA\TNR\C10ORF54\H2AFY2\ALKBH2\CES1\AP1S3\FAM120A\CYB5R2\USP32\JMJD4\PIK3CB\ARHGEF3\TNFAIP1\TPRA1\ZNF326\FAM122A\MLL3\K123\ANKRD1\PDE6C\PISD\HRAS\BDH1\RBM7\METTL9\CREM\CLCN5\NIT1\MTMR8\KDM3B\KIAA1841\APIP\VRK2\TTC17\TMEM209\CHRDL1\SLC39A13\CTDSPL2\CFLAR\SLC24A6\KIF16B\PPP3R1\COMMD5\TTC14\RABL6\LCLAT1\TRMT6\WDR26\SMOC1\COL1A2\FMN1\C1GALT1\C5H15ORF41\FASTKD1\LPGAT1\RAP1B\MDGA1\SEC23A\PRDX1\ADCK1\AGA\DHCR24\ITPK1\EREG\SIPA1L2\CBX3\RFTN1\UBE2E1\HSPBAP1\SERHL2\HIF1A\LARS2\EXOSC7\SFRP4\MDGA2\ITGA9\VPS41\C3H6ORF103\PWP1\RBFA\KIF13A\HIPK2\GALNT1\CLPTM1L\LOC426037\SPTLC1\MBP\NPEPPS\PPAT\TMX3\C22H2ORF42\PDS5A\CSTB\WBSCR22\MBLAC2\GFM2\RABL3\OTOP1\FIG4\HAUS6\TIPRL\PCMTD1\TCEA1\FNTA\BRP44\ZFP92\GPR89B\GBE1\GRIN3A\CPA6\ACOX3\WHSC2\RIPK2\CCNE2\PTDSS1\SIK1\DMD\FBXO9\MTSS

		1\FAM49A\PHKA2\ASXL2\CD2AP\HERC2\TNFSF13B\GPALPP1\LCP1\N6AMT2\FZD4\ARNT\FAM125A\CBX1\LYN\WASH1\GATA2\C2H6ORF62\PTGR2\SFI1\MPZL3\GLT1D1\NEO1\STOM\NFYC\MXRA8\NADK\CRMP1\RSPO3\RNASET2\DLL1\TGFB3\WIPF1\UBE3C\FASN\CD151\CACNA1B\REL\MAP3K14\
P<0.05	gga-miR-130b-5p	RFK\ARHGAP25\RASSF2\ADAM28\LSM7\DCAF7\LOC426699\C11H16ORF80\LDLRAP1\SNAP29\MYBBP1A\PI4KA\B3GNTL1\MIB2\PPIL2\UBE2R2\EIF6\CHMP4B\MRPL38\TSPAN14\C10ORF58\CHCHD2\RAN\POLR2E\C19H17ORF85\TFAM\MED23\LRRC45\BNIP1\BRI3BP\FBXL8\GNA11\HDAC1\TM2D2\KPNA6\LOC425137\SLC6A2\BPTF\USE1\CHD9\PPT1\IQCA1\CAPZB\TAF9\SIRT3\ADAM10\FAM20B\TBCA\TNR\C10ORF54\NOL8\H2AFY2\PTPN11\RHBDD1\ALKBH2\CES1\AP1S3\FAM120A\CYB5R2\USP32\JMJD4\PIK3CB\ARHGEF3\TNFAIP1\TPRA1\ZNF326\FAM122A\MLL3\K123\ANKRD1\PDE6C\PISD\HRAS\BDH1\RBM7\METTL9\CREM\ACOX2\CLCN5\NIT1\MTMR8\KDM3B\KIAA1841\APIP\VRK2\TTC17\TMEM209\CHRDL1\SLC39A13\CTDSPL2\CFLAR\SLC24A6\KIF16B\PPP3R1\COMMD5\TTC14\NHLRC2\RABL6\LCLAT1\TRMT6\WDR26\SKIL\SMOC1\ETFDH\COL1A2\FMN1\C1GALT1\USP15\CENPF\C5H15ORF41\FASTKD1\LPGAT1\RAP1B\TMEM184C\MDGA1\SEC23A\PRDX1\ADCK1\AGA\SCIN\DHCR24\ITPK1\EREG\SIPA1L2\CBX3\RFTN1\UBE2E1\SLC44A5\HSPBAP1\SERHL2\HIF1A\LARS2\EXOSC7\LARP7\SFRP4\MDGA2\ITGA9\VPS41\C3H6ORF103\PWP1\RBFA\KIF13A\HIPK2\GALNT1\CLPTM1L\LOC426037\SPTLC1\MBP\NPEPPS\PPAT\TMX3\C22H20ORF42\ARNTL2\PDS5A\CSTB\WBSCR22\MBLAC2\GFM2\RABL3\OTOP1\NSG1\B4GALT4\FIG4\HAUS6\IMPACT\TIPRL\PCMTD1\TCEA1\KCMF1\FNTA\BRP44\ZFP92\GPR89B\GBE1\GRIN3A\CPA6\ACOX3\WHSC2\RIPK2\CCNE2\PTDSS1\SIK1\DMD\FBXO9\MTSS1\FAM49A\PHKA2\ASXL2\OTOF\CD2AP\HERC2\TNFSF13B\MZT1\GPALPP1\LCP1\POSTN\N6AMT2\FZD4\ARNT\SUV39H2\FAM125A\CBX1\LYN\WASH1\GATA2\C2H6ORF62\PTGR2\SFI1\MPZL3\GLT1D1\NEO1\STOM\NFYC\MXRA8\NADK\SMS\CRMP1\RSPO3\RNASET2\DLL1\TGFB3\IGFBP5\WIPF1\UBE3C\FASN\CD151\DDX19B\CACNA1B\REL\MAP3K14\
P<0.05	gga-miR-130b-3p	RFK\ARMC1\RASSF2\TMEM9\LSM7\CAMTA1\PM20D1\FAM72A\IL10\RSPRY1\UBE2L3\PTPRU\RAB14\NFS1\GLB1L2\ATG16L1\MAT2B\GNB1L\SLC25A24\CHMP4B\LOC415325\CPEB1\FBXW11\GJA4\SLC15A4\DNAJC9\FRZB\MED23\SCAPER\NKX2-5\BOD1\MOXD1\SBNO1\MED27\MINA\PPT1\SLC25A16\DIABLO\SH3GLB2\SGPL1\HVCN1\MTHFR\P2RX5\KPNA7\CPSF4\RGL1\CLIC2\TSEN15\PPIF\RPS24\MTFR1\VGLL4\PPARG\PRPSAP2\NUP210\FAM96B\CLTC\VIPR1\CDH5\SNX2\ADAMTS18\CA4\PIK3CB\RHOG\DYNLL2\C20H20ORF11\SGSM2\ARFGAP1\DCTN5\HPRT1\ZNF326\IL2RA\DDT\SLC6A6\ATP12A\DNAJB6\VDAC1\YWHAH\ARL6IP1\IL13\PFKP\USP28\CREM\ALDH1A3\PTPRG\PGS1\CLCN5\ARHGAP12\NPEPL1\LUC7L\RAB22A\CHIC1\C17ORF28\STK17B\MLLT10\MOB4\NR1H3\ITPR1\FGL2\PHTF2\BMPR2\ABHD12\CRELD2\STAM\FXR1\C4HXORF57\SESTD1\YPEL5\LCLAT1\ACBD3\C6H10ORF46\RBM25\SMOC1\SEC23IP\NRCAM\MSMO1\ZNF650\C5H15ORF29\ACADSB\PTPN14\RAB33B\HHAT\IFNG\RAB3IP\CRIPT\HSPA4L\ALDH6A1\MMACHC\NPC2\NAP1L1\MBNL1\GYG1\VIPAR\CMPK1\ALKBH1\EPS15\BTF3L4\MEMO1\C4H4ORF41\PPPDE1\TMEM59\MEOX2\GOLGA5\AHR\NUDT9\INADL\SERPINA5\AFF1\COB

		LL1\ARHGAP24\SEC31A\PLCL2\RAB5A\BTG1\TMEM66\FGFR1OP\ELK3\GPD1L\PECR\MARK3\BCL2L14\PPP1R13B\MKI67IP\SOD2\ACAD11\RPL7L1\METTL14\RTN1\SFRP4\NAA30\NCKAP5\GMFB\TDRD7\EPC2\GKAP1\LOC100857286\JARID2\GCM2\BLOC1S5\SERPINB2\SERPINB6\KIAA1468\C5ORF22\ADCY2\BPGM\DERA\ERP44\MBP\CNOT7\LOC426218\NPEPPS\LONRF1\SLMO2\KIT\ITFG2\FGFBP2\PHYH\LYSMD3\CAST\DEPDC1B\ABHD3\RBBP8\CHD1L\TAF4B\DAZAP1\RB1CC1\CLTA\LOC421792\GPR89B\PREX2\LRPAP1\ABCC13\SAMSN1\TACC3\PNRC1\IMMT\CYB5R4\MMP16\MORC3\FZD6\SIK1\PDK3\GRHL1\LAPTM4A\UBXN2A\RP2\UXS1\SLC15A1\ABCC4\SLAIN1\SLAIN1\RNASEH2B\ZAR1L\SLC46A3\PAN3\MTMR2\ODZ4\KLHL5\KIAA1609\SEPT6\PSMG1\CLN8\INCENP\PPP6R3\MCM6\EAF2\ATE1\GCG\RABGAP1L\IRAK2\MUSTN1\UVRAG\POU1F1\RALBP1\DLL1\SLC1A2\EMB\MYO5A\SLC47A1\LDHA\NAALADL2\ZSWIM7\TLN2\NUCB2\TNFSF15\C5H11ORF46\TIMMDC1\
P<0.05	gga-miR-1306-3p_R-1	CD79B\CHFR\HEMK1\ZMYM4\SFPQ\DDHD2\TBC1D2B\ZDHHC9\CEP112\KIAA1279\TRMU\DHRS7B\PEMT\DKC1\GCLM\OAF\RNH1\ERLEC1\AP2M1\NDUFA5\CERKL\NCSTN\TRAF5\MRPS18A\PPP2R5C\FGFR1OP\PRSS12\DNAL4\BRAF\EXOC3\TAPBPL\ST3GAL6\HJURP\TP53INP1\ANXA13\SEPT6\RHOA\TCEB3\LOC100859142\
P<0.05	gga-miR-125b-3p_R-1_1ss21CT	C6H10ORF88\BNIP3L\DCAF7\TAF12\C20H20ORF24\CIAPIN1\GTF2I\CASP9\ANP32B\LRIG2\UQCC\UBE2J2\PMPCA\PTBP1\HSD17B4\H3F3B\CDC73\RGS2\RGS4\WDR5\NKX2-5\JMJD1C\SUB1\RILPL1\ANO5\COPS8\STK4\DNAJA2\PABPC1L\SRSF10\KIAA1279\ADA\ADAM10\HKDC1\FAM20B\CCPG1\NCOR1\HVCN1\PPP1CC\NPPC\P2RX5\MAD2L2\RGS9BP\ULK2\NOL8\C17H9ORF16\H2AFY2\DUT\ISY1\MYO1H\MED13\RRP1B\LPPR5\LOC768701\ADIPOQ\NOLC1\KLHL18\SLC44A3\DCAF12\BTBD1\PIK3C2A\HELZ2\C12H3ORF19\TBCCD1\MAPRE1\TSPAN6\MARCH5\CHKA\ACAP2\KIFAP3\FTH1\TH1L\ABCC3\LOC422305\RAB22A\RAB11A\KIAA1841\APIP\CD44\SF3A1\TTC17\TMEM209\MOB4\F2\XRN2\SUMO1\IFT122\EEF1B2\ACSS1\POLR3F\YPEL5\LCLAT1\SNAP23\ATP6V0C\IFT140\DCAF4\PRKCI\CAV2\EXD2\THAP5\CCDC132\RAB3GAP2\MSMO1\XRCC6BP1\PPP2R5A\KIF6\BTBD9\NAP1L1\UFSP2\CASP3\C5H14ORF102\FMN2\MACC1\G3BP2\STK39\NID1\TSNAX\LIN54\SMOC2\NGLY1\CINP\EIF5\STT3B\PECR\ERCC3\SNAPC1\TOB2\RPL7L1\ACTR3\C3H6ORF72\TNFAIP6\RECK\PWP1\RANBP9\RBM24\HSP90B1\LOC420864\SLC37A3\MKRN1\KDM5A\LOC418170\SLC6A19\LOC100859438\PCM1\TMX3\LTV1\IGF2BP2\SGCB\PPFIBP1\KLB\FAM162A\TNKS2\DEPDC1B\MTX3\RABL3\WDR1\FAM26E\ZBTB49\NSG1\NPC1\RPF2\PCMTD1\RB1CC1\SNX3\OSTM1\RPL24\TMEM175\MYBL1\MAP3K7\TCEB1\SH3BP2\PNRC1\CYB5R4\TTK\GEM\MORC3\TOP1MT\DENND3\U2AF1\KDM6A\DDX3X\LRRC6\PXDN\MBOAT2\ASXL2\ASB9\UFM1\MRPL48\LCMT2\LOC426128\ITM2B\FAM49B\ETV1\WDR48\C3H2ORF43\FZD7\EPM2A\SUCLG1\MCM6\RABGAP1L\UNG\KPNA1\WWP1\MTO1\TAPT1\SLC12A2\XRCC6\SLC10A7\SLC1A2\PARP1\EMB\ATP2A2\GTF3C5\TLN2\ACTG1\RABEP1\DDX6\SNRNP40\
P<0	gga-miR-122-5	TRIM39\CEPT1\MAN1C1\WBSCR16\RABGAP1\MRRF\COX10\PLLP\GLB1L2\JMJD6\PKM2\SYT15\POLR2D\PLEKHB2\COMMD10\VPRBP\RG

.05	p	S2\KTN1\ETFA\TRPC4AP\SPINK7\OPTC\DBNDD2\AKR7A2\RIC8A\P4HA1\PPP1CC\MTHFR\C21H1ORF187\PRPS1\DUT\CLTC\SLC25A36\PPIC\GCLM\ABCC1\AP2A2\ITGB1\PCSK6\MASP1\ARCN1\TMLHE\UBE2V1\TMEM209\UACA\ITGA8\SPTBN5\NHLRC2\LBH\ATP6V0C\CAPZA2\DUSP10\YAF2\SHFM1\TRAF5\VSX2\GLIPR1L\NASP\BTF3L4\PPPDE1\BZW2\UMPS\C22ORF32\CCK\UGT8\CASP6\RAB32\ESR1\USP18\MTHFD2\TDP2\CNDP2\NPEPPS\DLC1\TACR1\MYB\LPCAT3\SMCHD1\BOC\TMEM68\GJA5\GPR89B\PDZK1\PREX2\FABP5\CD8A\CSMD3\RAD21\TSTA3\FBXO32\CALB1\TUBGCP5\SPRY2\IRG1\ELF1\WBP4\TYR\ARL6IP5\CCDC41\CLSTN1\LOC419404\VAMP3\NT5C3A\TGFB3\ARHGAP15\SLC10A7\MYO5A\TMEM120B\P4HA1\LEPROTL1\
P<0.05	gga-miR-107-5p_R+1	ARMC1\SLC23A2\RASSF2\BNIP3L\ADAM28\ZNF593\STRADA\DDX42\CAMTA1\KIAA0556\TAT\TAF12\HERPUD1\PIK3R6\ATP5G1\MYBBP1A\IRF1\LOC419409\ZAP70\SSU72\NFS1\EIF6\VAV3\RHBDF2\TRIM33\VCP\LOC419429\C15H22ORF25\PPIB\AGO3\EP400\OSGEPL1\GMPPB\HSD17B7\RFNG\RASA2\LOC100857356\TARDBP\FBXL8\ASH2L\VAMP4\RILPL1\SKP2\BPTF\TMEM38A\PABPC4\LRRFIP1\P2RX7\TIMD4\XPR1\SYS1\PPT1\KLF2\LOC101749801\FAM189A1\CAPZB\CSRP3\FAM81A\DDX50\TOR1A\TAF9\COL13A1\MAP2K6\AP3B1\PALD1\CCNE1\PPTC7\HVCN1\MTHFR\AKAP10\SET\ULK2\RGL1\C14H17ORF103\PTGES2\CENPI\PRPSAP2\CDH11\CYB5R2\SELK\GGNBP2\ARHGEF3\SYNGR3\COL9A3\VILL\C9ORF24\TMED5\MYD88\ARHGAP17\ABTB1\TPRA1\HPRT1\MORN4\DDX26B\PTPRJ\HTATSF1\ZMYND11\RLBP1\TAF3\YWHAH\TLR4\PPP1R2\ACOX2\ATP13A4\ARHGAP12\NME1\ANKRD40\SUDS3\PEBP1\CHIC1\LRRC59\EIF4E3\NMRAL1\HN1\UBE2H\GGA3\MRPS7\BCAP29\RNF114\CD82\CHRDL1\NDUFS3\AOX2P\PCGF6\ORC2\FAM185A\F2\MPP4\MCTS1\SMC3\MGA\CNRIP1\DCUN1D1\SAMD13\VPS54\COMMD5\USP13\EDF1\TSPAN12\ZFP106\LIN9\KIAA1715\NVL\DEGS1\CRAMP1L\CTSO\CAPZA2\RXFP1\SLC39A9\ETFDH\C2H7ORF64\RAB3GAP2\ZNF650\C5H15ORF29\LXN\ARID2\SSB\FMN1\C5H15ORF41\TRAF5\WDR65\RAP1B\PREPL\EDNRA\BRE\GLO1\GEMIN2\MMACHC\MME\TTLL5\TSPAN1\MBNL1\MBNL1\HERC3\ALKBH1\SNW1\OSTC\BIRC6\UFSP2\PODN\MAP1LC3C\TMEM59\TTC4\LGMN\MTR\PGM1\HNRNPA2B1\CBX3\LEPROT\ACTA1\CHN2\SEC31A\SMOC2\ANKRD28\TRAF3\T\BAG5\SDAD1\ILF2\MAP3K4\NPFFR2\HEBP1\KIF15\KIAA1143\METTL14\SLC12A8\TRANK1\ELMO1\SOX10\STK17A\PLEKHC1\WDSUB1\TMEM263\KCNG2\MYLIP\PMCH\ECI2\SLC37A3\MKRN1\DROSHA\DERA\PDLIM3\LOC420992\LOH12CR1\STX17\PPP6R2\CYB5A\ENPP7\RSPH3\LOC426296\TBCE\ARNTL2\APBB2\RELL1\FGFBP2\RBP5\RFESD\PRRC1\SGTB\ERBB2IP\SMCHD1\SERINC1\ABHD3\GFM2\TRAF3IP2\TJP2\ZDHHC23\GRAMD1C\F5\TCEA1\GNE\NFKBIZ\USP45\GPR89B\GBE1\ACOX3\EPHA7\WHSC2\LTN1\NT5E\TP53INP1\TSTA3\SIK1\NDRG1\PHF20L1\PRIM2\TAF2\GAL10\MCM3\STAG3\PLCXD1\TUBGCP5\CHST10\C1H2ORF49\UXS1\TM9SF2\IPO5\RBM26\WDFY2\SLC25A15\N6AMT2\MTMR2\LCMT2\H2AFZ\SUV39H2\TERF1\ABHD5\LOC100857925\CLN8\SLC20A2\PPP4R2\LMO4\HDAC4\C20ORF96\NADK\IRAK2\ATXN7\WDR82\PI15\CENPQ\MYOM1\CDKN1B\SLIT2\EGFR\DLL1\NPVF\DGKZ\TMEM154\ZBTB34\MYO5A\ATP1B4\ARF1\TMEM120B\TBC1D22A\ATP2A2\KIAA1328\ZSWIM7\NUCB2\LOC427826\CSDE1\REL\CR1L\WRAP73\SLC9A9\HSF1\NMB\

P<0.05	gga-let-7k-5p_R+1_1ss16AT	RDM1\KCTD9\GALK2\CYB5B\HBEGF\STRBP\LOC428335\ST14\CNTRL\NFS1\HEXDC\LRIG2\VAV3\TMEM194B\GMPPB\PCTP\UBIAD1\GOLGA7\OSMR\TARSL2\TNFRSF1B\P2RX5\PDCD5\MAPK6\PTGS2\DEAF1\EPHA4\SLC25A36\USP32\PNAT10\ABCD3\TMED5\KLHL13\KIAA0753\SRPRB\IL13\MAP3K8\ITGB2\KIAA2022\MAGT1\EIF4E2\TMEM164\C6H10ORF32\TYRO3\ETFDH\C5H15ORF29\COL1A2\ACADSB\C5H15ORF41\WDR65\EAPP\BTBD9\ABCD4\SMARCAD1\ICA1\BZW2\IGF2BP3\GLRX5\BCS1L\EXOC3L4\TNFAIP2\IL8L1\SERHL2\C22ORF32\SLC12A8\CEP70\ADH1C\TGFBR1\RBFA\LOC420849\GNPTAB\AKR1D1\SERPINB10\WNK1\PBX4\SLMO2\C2H18ORF19\MAP3K5\CHD4\POC5\GTF3C6\UHRF2\LNP1\NANS\RARS2\SYNCRIP\CCNE2\DMD\PIGA\GAL10\MGAT4A\COL4A2\ARHGEF7\TGDS\WDFY2\DDI2\HSD3B2\LOC420301\E2F5\TERF1\CZH9ORF80\HABP2\DNTT\BZW1\GLT1D1\CRTAP\SLC10A7\GATM\PGRMC1\GCSH\MYO19\DDX19B\EIF4G2\C5H11ORF46\YOD1\PDZD11\ARHGEF10L\STK40\TMEM130\
P<0.05	gga-let-7g-5p_R+1_2ss14TG18CA	RDM1\KCTD9\GALK2\CYB5B\HBEGF\STRBP\LOC428335\ST14\CNTRL\NFS1\HEXDC\LRIG2\VAV3\TMEM194B\GMPPB\PCTP\UBIAD1\GOLGA7\OSMR\TARSL2\PNRC2\HNF4A\TNFRSF1B\P2RX5\PDCD5\MAPK6\PTGS2\DEAF1\SLC25A36\USP32\PNAT10\ABCD3\TMED5\KLHL13\KIAA0753\SRPRB\CBL\IL13\MAP3K8\ITGB2\KIAA2022\MAGT1\EIF4E2\TMEM164\C6H10ORF32\PHTF2\TYRO3\SNAP23\KRIT1\ETFDH\CCDC132\C5H15ORF29\COL1A2\ACADSB\RAB33B\C5H15ORF41\WDR65\EAPP\BTBD9\ABCD4\SMARCAD1\ICA1\BZW2\IGF2BP3\GLRX5\BCS1L\EXOC3L4\TNFAIP2\IL8L1\ATF7IP\SERHL2\C22ORF32\ST13\SLC12A8\CEP70\ADH1C\TGFBR1\RBFA\LOC420849\GNPTAB\AKR1D1\SERPINB10\WNK1\PBX4\SLMO2\C2H18ORF19\MAP3K5\CHD4\POC5\GTF3C6\UHRF2\LNP1\NANS\RARS2\SYNCRIP\CCNE2\ST3GAL1\DMD\PIGA\GAL10\CYP39A1\MGAT4A\COL4A2\ARHGEF7\TGDS\WDFY2\DDI2\HSD3B2\LOC420301\E2F5\TERF1\CZH9ORF80\HABP2\DNTT\BZW1\GLT1D1\CRTAP\SLC10A7\GATM\PGRMC1\GCSH\MYO19\DDX19B\EIF4G2\C5H11ORF46\YOD1\PDZD11\ARHGEF10L\STK40\TMEM130\
P<0.05	gga-let-7f-3p_1ss22CT	KCTD9\SPPL2B\SERPIND1\SEC14L1\WWC1\VAV3\NR5A2\CDC73\NUP35\C26H6ORF106\DNER\NFYA\SUB1\SBNO1\C18H17ORF75\UBLCP1\GTF2A2\COL6A3\DNAJA2\C17H9ORF78\SLTM\RCAN3\VPS13D\RGS9BP\SET\TMOD3\LSM14A\DHX30\NUP210\RRP1B\TRMT13\NONO\HIF1AN\DGCR2\MMP11\BTBD1\ARL6IP1\RMI2\VAPB\MTMR8\IL18\EEF1B2\NDUFS1\C5H15ORF29\RPAP3\G2E3\MDM1\FAM177A1\HSPA4L\NASP\FAM98A\BTF3L4\GTF2A1\SMEK1\ASB5\KLHL7\GLRX5\CCNK\CNOT10\BAG5\PTPLB\POLDIP3\KIF15\RTN1\CAMK2D\FBXO34\NCKAP5\ITGA9\CUL1\MMADHC\C1H12ORF48\TRIM24\USP18\TMF1\LRRC16A\CEP76\C2H18ORF19\SMCHD1\CZH5ORF28\HDAC2\SS18\ABHD17B\TMEM68\PCNP\CD247\GRPEL1\WHSC2\TMEM129\MORC3\RAD21\MED14\PRIM2\FER1L6\TRAPPC12\RBBP7\SYAP1\SPRY2\SUCLA2\POSTN\UBL3\RRM1\VAPA\HSD3B2\FAM206A\PPP4R2\C2ORF47\GLT1D1\NPPB\KPNA1\IMPG2\KDM3A\PCMT1\CUL2\CNTN1\
P<0.05	gga-let-7c-5p_1ss17AG	RDM1\KCTD9\GALK2\CYB5B\HBEGF\LOC428335\ST14\CNTRL\NFS1\HEXDC\LRIG2\VAV3\TMEM194B\GMPPB\PCTP\UBIAD1\GOLGA7\OSMR\TARSL2\TNFRSF1B\P2RX5\PDCD5\MAPK6\PTGS2\DEAF1\SLC25A36\USP32\PNAT10\ABCD3\TMED5\KLHL13\KIAA0753\SRPRB\C

		BL\IL13\MAP3K8\ITGB2\MAGT1\EIF4E2\TMEM164\C6H10ORF32\TYRO3\ETFDH\C5H15ORF29\COL1A2\ACADSB\C5H15ORF41\WDR65\EAPP\BTBD9\ABCD4\SMARCAD1\ICA1\BZW2\IGF2BP3\GLRX5\BCS1L\EXOC3L4\TNFAIP2\IL8L1\ATF7IP\SERHL2\C22ORF32\SLC12A8\CEP70\ADH1C\TGFBR1\RBFA\LOC420849\GNPTAB\AKR1D1\SERPINB10\WNK1\PBX4\SLMO2\C2H18ORF19\MAP3K5\CHD4\POC5\GTF3C6\UHRF2\LNP1\RARS2\SYNCRIP\CCNE2\DMD\PIGA\GAL10\MGAT4A\COL4A2\TGDS\WDFY2\DDI2\HSD3B2\LOC420301\E2F5\TERF1\HCRTR2\CZH9ORF80\HABP2\DNTT\BZW1\GLT1D1\GATM\GCSH\MYO19\TMEM120B\DDX19B\EIF4G2\C5H11ORF46\YOD1\PDZD11\ARHGEF10L\STK40\TMEM130\
P<0.05	gga-let-7c-3p_1ss12TC	ARMC1\ADORA1\RASSF2\LOC426699\RAB11B\SLC41A1\PPP1R8\EYA3\GAPVD1\FAM118B\HES5\CLK3\RAB14\CYB5D2\NCAPD3\RBM39\TTI2\SLC22A7\E2F4\IWS1\FBXW11\PMS1\KTN1\PPARD\RFNG\JMJD1C\SPHKAP\CCL20\MBTPS1\SBNO1\POMT1\NELL1\SUN1\MPHOSPH10\YWHAB\LOC422151\BET1L\ADAM10\WIPI1\COL13A1\BAP1\RSRC2\PPP1CC\MAD2L2\TGM3\PTGS2\WDFY1\VCAM1\APPBP2\BCMO1\PIK3AP1\MED12\ABHD6\NLK\DR1\TMED5\KLHL13\CLK4\PAXIP1\HTATSF1\PDPK1\GRM3\P4HA2\YWHAH\AP2A2\QRICH1\CHM\BDH1\ARIH2\RNF185\LRP5\PPP1R2\PDHB\ALDH1A3\KIFAP3\LEPREL1\YME1L1\DPYSL3\CHIC1\KIAA2022\CBLN4\MAGT1\BCL11A\CNTN6\PRORSD1P\SRPK2\NET1\FERMT1\ATP6AP1\COMMD5\NEUROD1\MKKS\TASP1\MND1\SP3\KRIT1\BAG3\IFRD1\THAP5\GAD1\SSB\C1GALT1\TBK1\EDNRA\BAZ1A\MIA2\PPP1R12A\FAM98A\NKX6-2\LOC100859230\GTF2A1\ECHDC2\SCIN\DHCR24\HSD17B11\ROR1\HNRNPA2B1\MIER1\PPP2R5C\BTG1\USP44\CDC42BPB\NEDD1\SEMA5B\SNRPD1\QSER1\FSHB\GMFB\CXCR4\ERO1L\GTDC1\DEK\NUP153\DAP\MARCH6\PLEKHA5\IAPP\PDLIM3\ANP32E\MTHFD2\CNOT7\TOMM20\KIF2A\ERBB2IP\FIG4\ZDHHC23\ARMC2\NANS\KIAA1524\NRG1\UBXN2B\LOC427306\GRK4\SAMSN1\WHSC2\LRRCC1\SCAF4\NOV\PTP4A1\DST\ADCY8\DERL1\FAM49A\NRBP1\ACE2\MGAT4A\UXS1\ATP4B\COL4A2\DZIP1\SPRY2\LECT1\NAA16\MED4\SPRYD7\NUPL1\MPHOSPH8\LOC419074\H2AFZ\GZMK\CD47\PACSIN2\UBE2N\SLC25A14\INCENP\PPP6R3\PPP4R2\NEO1\CRISPLD1\APP\COL9A1\RAD17\SGOL1\NCF4\SLC10A7\GABPB1\CYP24A1\ANAPC5\LDHA\CYP2H1\ARL8A\SLU7\
P<0.05	gga-let-7b_L-1R-1	CAMK4\RANBP3\FAM125B\LRIG2\VAV3\NCOA4\TMEM194B\ERGIC1\KPNA2\C21H1ORF144\OSMR\SRSF10\RALGPS2\BUD31\ALDH3A2\CEBPG\LOC769755\PTGS2\FAM96B\BICD2\C17ORF78\RNF141\KIAA0753\SLC9A6\CXCL14\C14H16ORF42\ZMYND11\AP2A2\SVIL\CDX4\NAT9\PEX13\CNTN6\CD82\LOC416735\LBH\ADCK3\UBR1\KRIT1\COL1A2\TRERF1\MCFD2\GLO1\ADCK1\ELAVL4\BIRC6\GTF2A1\FBXO8\PPAP2B\ITPK1\IGF2BP3\LEPROT\DAZL\PPP1R13B\ANXA5\PRRG4\AVL9\ADH1C\EPC2\NMI\PHACTR1\ESR1\LOC418170\SLC34A2\UHRF2\LNP1\TFG\EPHA7\RARS2\SNAP91\IMPG1\OTUD6B\FZD6\U2AF1\CASK\PRIM2\PIGA\MMP13\DDI2\MBNL2\UBE2CBP\DCLRE1A\LOC415708\GLT1D1\IMPG2\GYPC\PGRMC1\GCSH\YOD1\RCC2\UBE4A\TMEM130\
P<0	gga-let-7a-5p	RDM1\KCTD9\GALK2\CYB5B\HBEGF\STRBP\LOC428335\ST14\CNTRL\NFS1\HEXDC\LRIG2\VAV3\TMEM194B\GMPPB\PCTP\UBIAD1\GO

.05	_2ss12GT18TC	LGA7\OSMR\TARSL2\TNFRSF1B\P2RX5\PDCD5\MAPK6\PTGS2\DEAF1\EPHA4\SLC25A36\USP32\PNAT10\ABCD3\TMED5\KLHL13\KIAA0753\SRPRB\IL13\MAP3K8\ITGB2\KIAA2022\MAGT1\EIF4E2\TMEM164\C6H10ORF32\TYRO3\KRIT1\ETFDH\CCDC132\C5H15ORF29\COL1A2\ACADSB\C5H15ORF41\WDR65\EAPP\BTBD9\ABCD4\SMARCAD1\ICA1\BZW2\IGF2BP3\GLRX5\BCS1L\EXOC3L4\TNFAIP2\IL8L1\SERHL2\C22ORF32\SLC12A8\CEP70\ADH1C\TGFBR1\RBFA\LOC420849\GNPTAB\AKR1D1\SERPINB10\WNK1\PBX4\SLMO2\C2H18ORF19\MAP3K5\CHD4\POC5\GTF3C6\UHRF2\B4GALT3\LNP1\NANS\CD200\RARS2\SYNCRIP\CCNE2\DMD\PIGA\GAL10\MGAT4A\COL4A2\TGDS\WDFY2\DDI2\HSD3B2\LOC420301\E2F5\TERF1\CZH9ORF80\HABP2\DNTT\BZW1\GLT1D1\CRTAP\SLC10A7\GATM\GCSH\MYO19\DDX19B\EIF4G2\C5H11ORF46\YOD1\PDZD11\ARHGEF10L\STK40\TMEM130\
P<0.05	gga-let-7a-5p	RDM1\KCTD9\GALK2\CYB5B\HBEGF\STRBP\LOC428335\ST14\CNTRL\NFS1\HEXDC\LRIG2\VAV3\TMEM194B\GMPPB\PCTP\UBIAD1\GOLGA7\OSMR\TARSL2\TNFRSF1B\P2RX5\PDCD5\MAPK6\PTGS2\DEAF1\SLC25A36\USP32\PNAT10\ABCD3\TMED5\KLHL13\KIAA0753\SRPRB\CBL\IL13\MAP3K8\ITGB2\KIAA2022\MAGT1\EIF4E2\TMEM164\C6H10ORF32\TYRO3\ETFDH\C5H15ORF29\COL1A2\ACADSB\C5H15ORF41\WDR65\EAPP\BTBD9\ABCD4\SMARCAD1\ICA1\BZW2\IGF2BP3\GLRX5\BCS1L\EXOC3L4\TNFAIP2\IL8L1\SERHL2\C22ORF32\SLC12A8\CEP70\ADH1C\TGFBR1\RBFA\LOC420849\GNPTAB\AKR1D1\SERPINB10\WNK1\PBX4\SLMO2\C2H18ORF19\MAP3K5\CHD4\POC5\GTF3C6\UHRF2\LNP1\NANS\RARS2\SYNCRIP\CCNE2\DMD\PIGA\GAL10\MGAT4A\COL4A2\TGDS\WDFY2\DDI2\HSD3B2\LOC420301\E2F5\TERF1\HCRTR2\CZH9ORF80\HABP2\DNTT\BZW1\GLT1D1\KDM3A\CRTAP\SLC10A7\GATM\GCSH\MYO19\DDX19B\EIF4G2\C5H11ORF46\YOD1\PDZD11\ARHGEF10L\STK40\TMEM130\
P<0.05	efu-miR-361	TRIM27\C22H20ORF30\BNIP3L\RAB11B\C4BPA\DBR1\APLP2\CAMK1G\MECR\CCNL2\NFS1\ITIH3\ROGDI\ACO1\WBP2\AP3S1\HSPA9\CHCHD2\PLS1\ZC3H15\KIF1B\SPHKAP\RPA1\MMD\SUB1\TMED2\RYR3\SFRP1\GFI1B\TSC1\LIFR\SUN1\HVCN1\MTHFR\LOC425531\CCNDBP1\TSEN2\CES1\ACSL3\TK2\SASS6\TEAD1\JMJD4\PIK3CB\PNAT10\RHOG\NONO\TNFAIP1\TMEM41B\TMED5\MTF2\MYD88\MST4\MAP3K13\MAP3K8\NPEPL1\RAB22A\ANO1\CD82\CHRDL1\CELF1\EIF3J\DDB2\SLC26A5\TRH\ACSS1\TRDMT1\DCUN1D1\CROT\MDH1\ING3\MTMR1\PRKCI\CAPZA2\GLRB\LOC424998\DCAF17\ARID2\PROX1\MYST2\SLC33A1\ADCK1\GTF2A1\SLC30A6\NEIL3\ITPK1\DFNA5\TSNAX\EML1\SERPINH1\T\PECR\CAMK2D\OSBPL11\BBS9\PPP3CA\MRPL32\STK17A\FBXO7\ISCA1\NUP153\SYCP3\ADCY2\C3AR1\LOC420992\ANP32E\LOC100859438\CYP4V2\C2H18ORF19\ARHGAP8\NUP50\FKBP4\EDARADD\TAPBPL\LYSMD3\IL6ST\FBXO4\MEP1B\PCMTD1\TMEM175\EPHA7\CASP8AP2\EEF1D\DDX3X\PTP4A1\INTS9\TFDP1\ARGLU1\BIVM\C1H13ORF15\SETDB2\CRYL1\PLEKHB1\SPDYA\LCORL\C1H21ORF33\ARHGDIB\LOC101750282\C7ORF53\POT1\CERK\RPRD1A\CLN8\HSF2\IFNGR1\PSEN1\EAF2\PLEKHA3\STX2\MXRA8\LOC419404\GART\TRIB2\RREB1\PCMT1\RNASET2\CUL2\ZBTB34\NEK1\ATP2A2\C5H11ORF46\HSF1\
P<0	dre-miR-24_R+	SPR\GFPT1\PRNP\BNIP3L\NEFM\MYL4\CDC27\TBC1D22B\C11H16ORF80\HBEGF\FAM125B\FAM118B\POLR2C\WBSCR16\FAM192A\LOC

.05	2	419726\SAMHD1\CREB3L3\ADRA1B\RAP1A\MOV10\MAN2C1\MAK16\GOLGA3\NUBP2\AP3S1\HSPA9\DNAJC18\PIK3CD\SBNO2\GK5\MTA1\LRRC45\NEDD4L\ETFA\SRM\DDX49\ARL2\TUBG1\CRLF3\TMED2\UBIAD1\ELL\BSDC1\FNDC5\PPP1R16B\TOP1\CNOT8\PPM1M\GALE\USP20\ANXA6\BAP1\ASB6\C14H17ORF103\INTS2\SLC25A36\GUK1\LOC768701\TRPM2\NLK\BOK\CNOT6\GORASP1\MCM2\TPRA1\MOSPD1\MYLK2\SLC25A48\CEP19\KIF3B\TOLLIP\TAF3\PLOD2\RPRD1B\KIF11\TNMD\CTSA\DALRD3\PPP1R2\NRP1\RMI2\UTS2D\TRAIL-LIKE\CLPX\PPRC1\GOT1\FYTTD1\STAT1\KCTD2\C6H10ORF32\COPG2\ANKRD16\FBXW8\CASC4\TBX3\EDEM1\NET1\ARRDC1\GRIA3\SPTBN5\NRK\EHD3\PRKCI\ARID2\CLCN3\DYRK2\RAP1B\CPSF6\SMAD1\CRIPT\LOC417848\TMEM184C\PTPRF\C4H8ORF40\CAPN1\VSX2\ABCD4\FCF1\TMED10\XPO5\TMEM48\SCIN\TTC4\CYP2J2\INADL\TSNAX\DPP4\CRADD\SEPT11\MKI67IP\PTPLB\ITGB5\HEBP1\ANK2\LARP7\AVL9\ERO1L\NOL12\TMEM263\SLC41A2\TXNDC5\IRF4\C6ORF97\ATP6V1E1\PDK1\NCLN\PPIE\ANP32E\LOC426218\FUCA2\TEC\CORO6\ZNF384\WBSCR22\RFESD\ZYX\B4GALT3\CDON\DAZAP1\IDUA\CD200\TRIM55\VCPIP1\AFAP1\HTRA3\CHMP3\IFNGR2\LAPTM4A\RAB9A\CENPO\DNAJC3\CWF19L2\MTMR2\E2F5\KLHDC2\EAF2\NDUFA10\MATN3\WDR35\CASQ2\CD4\AFAP1\RAD17\HOPX\PRKAG3\HDC\KCNT1\EMB\CD151\TGM2\TMEM120B\EXOSC2\COL6A1\CR1L\LIMD2\PCSK7\P4HA1\TNFRSF10B\BET1\
P<0.05	csa-let-7d_1ss10TC	RDM1\KCTD9\GALK2\CYB5B\HBEGF\LOC428335\ST14\CNTRL\NFS1\HEXDC\LRIG2\VAV3\TMEM194B\GMPPB\PCTP\UBIAD1\GOLGA7\OSMR\TARSL2\TNFRSF1B\P2RX5\PDCD5\MAPK6\GLE1\PTGS2\DEAF1\SLC25A36\USP32\PNAT10\ABCD3\TMED5\KLHL13\KIAA0753\SRPRB\CBL\IL13\MAP3K8\ITGB2\MAGT1\EIF4E2\TMEM164\C6H10ORF32\TYRO3\ETFDH\C5H15ORF29\COL1A2\ACADSB\C5H15ORF41\WDR65\EAPP\BRE\BTBD9\ABCD4\SMARCAD1\ICA1\BZW2\IGF2BP3\GLRX5\BCS1L\EXOC3L4\TNFAIP2\IL8L1\SERHL2\C22ORF32\SLC12A8\CEP70\ADH1C\TGFBR1\RBFA\LOC420849\GNPTAB\AKR1D1\SERPINB10\WNK1\PBX4\SLMO2\C2H18ORF19\MAP3K5\LIMCH1\CHD4\POC5\GTF3C6\UHRF2\LNP1\NANS\RARS2\SYNCRIP\CCNE2\ST3GAL1\DMD\PIGA\GAL10\MGAT4A\COL4A2\TGDS\WDFY2\DDI2\HSD3B2\LOC420301\E2F5\TERF1\CZH9ORF80\DNTT\BZW1\GLT1D1\SLC10A7\GATM\GCSH\MYO19\DDX19B\EIF4G2\C5H11ORF46\YOD1\PDZD11\ARHGEF10L\STK40\TMEM130\
P<0.05	chi-miR-361-5p	TRIM27\C22H20ORF30\BNIP3L\RAB11B\C4BPA\DBR1\APLP2\CAMK1G\MECR\CCNL2\NFS1\ITIH3\ROGDI\ACO1\WBP2\AP3S1\HSPA9\CHCHD2\PLS1\ZC3H15\KIF1B\SPHKAP\RPA1\MMD\SUB1\TMED2\RYR3\SFRP1\GFI1B\TSC1\LIFR\SUN1\HVCN1\MTHFR\LOC425531\CCNDBP1\TSEN2\CES1\ACSL3\TK2\SASS6\TEAD1\JMJD4\PIK3CB\PNAT10\RHOG\NONO\TNFAIP1\TMEM41B\TMED5\MTF2\MYD88\MST4\MAP3K13\MAP3K8\NPEPL1\RAB22A\ANO1\CD82\CHRDL1\CELF1\EIF3J\DDB2\SLC26A5\TRH\ACSS1\TRDMT1\DCUN1D1\CROT\MDH1\ING3\MTMR1\PRKCI\CAPZA2\GLRB\LOC424998\DCAF17\ARID2\PROX1\MYST2\SLC33A1\ADCK1\GTF2A1\SLC30A6\NEIL3\ITPK1\DFNA5\TSNAX\EML1\SERPINH1\T\PECR\CAMK2D\OSBPL11\BBS9\PPP3CA\MRPL32\STK17A\FBXO7\ISCA1\NUP153\SYCP3\ADCY2\C3AR1\LOC420992\ANP32E\LOC100859438\CYP4V2\C2H18ORF19\ARHGAP8\NUP50\FKBP4\EDARADD\TAPBPL\LYSMD3\IL6ST\FBXO4\MEP

		1B\PCMTD1\TMEM175\EPHA7\CASP8AP2\EEF1D\DDX3X\PTP4A1\INTS9\TFDP1\ARGLU1\BIVM\C1H13ORF15\SETDB2\CRYL1\PLEKHB1\SPDYA\LCORL\C1H21ORF33\ARHGDIB\LOC101750282\C7ORF53\POT1\CERK\RPRD1A\CLN8\HSF2\IFNGR1\PSEN1\EAF2\PLEKHA3\STX2\MXRA8\LOC419404\GART\TRIB2\RREB1\PCMT1\RNASET2\CUL2\ZBTB34\NEK1\ATP2A2\C5H11ORF46\HSF1\
P<0.05	cgr-miR-500	STARD4\KCTD9\SPPL2B\SRSF3\FAM72A\SAMHD1\PSMD5\SEPT9\LRIG2\ODZ2\UBE2J2\TNFRSF18\PMPCA\PLEKHB2\RFFL\ADPRHL2\GBAS\AQP3\ZMYM4\INPP5K\TARDBP\NARS\C26H6ORF130\NFYA\HERC1\G3BP1\ADA\GPX3\SGPL1\SET\RPS24\MTFR1\FUT11\RPS6KB1\TK2\TADA2A\NONO\LOC768701\ADRBK2\IDI1\ACADS\RMI2\MED19\MAP3K8\CD3E\NPEPL1\SUCLG2\CD44\UBE2H\SLC26A4\UBE2V1\NDUFS3\ACTR1A\SUMF1\CCNYL1\SHOC2\JAG1\KIAA0922\BAG3\IFRD1\DYNC1I2\PLEK2\PDK4\ANGEL2\FASTKD1\ZNF330\E2F7\GPR149\UFSP2\IGF2BP3\KCNK1\COG2\VRK1\CHN2\C3H6ORF120\OXNAD1\CRADD\ZRANB2\PECR\C5H14ORF135\ARPP21\BDH2\ATL1\IYD\CYTH4\LOC420807\FARS2\MKRN1\ADCY2\SRSF7\VPS45\SGCB\KRAS\M6PR\MBLAC2\ZDHHC23\ZFAND5\DAZAP1\SEC63\TMEM68\JAKMIP1\AFAP1\TMEM129\IMPG1\RAD21\HADH\U2AF1\FBXO9\PXDN\UBXN2A\LIG4\ABCC4\ZC3H13\KIAA0226L\SUCLA2\PAN3\POLD3\GDPD5\VAPA\C6ORF192\PDGFRL\SLC20A2\CHP1\INCENP\TM4SF1\MCCC1\NEO1\SMS\AFAP1\PDLIM5\TMED8\SLC10A7\BIRC5\TBC1D22A\DDX19B\CSDE1\WRAP73\
P<0.05	cgr-miR-326	TRIM39\CD79B\DOCK5\DHDDS\ENTPD4\KLHDC8A\PPP1R8\TJP3\RASSF5\EI24\STUB1\SRPR\POLR2C\WBSCR16\TMEM45B\PIK3R5\STX8\GTF2I\RAB14\JMJD6\PRKRIP1\CCDC99\ARVCF\LOC100859653\MSTN\RUSC2\OSGEPL1\GBA2\PIK3CD\LZIC\INPP5K\AARSD1\SRR\FBXL8\NFYA\DDHD2\GBGT1\SBNO1\SFRP1\CCND3\TAF8\ANXA2\NT5C1A\LOC101749801\CAMKK2\STK4\DPP9\TNFRSF8\SLC13A3\NCOR1\TNR\LOC100859686\TAX1BP3\FAM101B\GPI\CDH5\SLC25A36\GNG13\GATA5\RHOG\TFIP11\GBF1\TSPEAR\NLK\ARFGAP1\RTEL1\LSS\MIF\BPI\CBL\RNH1\RNF10\PCSK6\PXN\RNF166\GCN1L1\ARCN1\C10H15ORF44\STK32A\TRAPPC4\UPRT\TTC12\SALL4\KCTD2\CHRDL1\SLC39A13\FBXO18\ACP2\CRY2\ARHGDIA\CCNYL1\TRH\POLR2H\MGA\LPHN2\GIPC2\YPEL5\GFRA1\LOC431656\TMEM229B\LOC417800\ARSA\RASSF3\SMAD1\ELOVL1\TMEM184C\HSP90AB1\CYP4B1\NKX6-3\ALDH18A1\RAVER2\HNRNPA2B1\DLK1\TECRL\T\ATF7IP\SLC38A6\BBS9\MAFF\SLC9A3\HMGXB4\FBXO7\PRDM4\KIF13A\ZBTB2\TRIO\SDHA\ANKS6\LOH12CR1\EZR\KDR\KIT\S100A9\SCNN1A\PDS5A\DCBLD1\USP14\HJURP\MPZL1\ALDOB\LYRM2\PGM3\THNSL2\TP53INP1\RUNX1\SFXN5\TSTA3\EEF1D\HAS2\FAM49A\MCM3\DNAJC3\RCBTB1\PLEKHB1\CD1C\DDI2\UNC5A\LOC418424\POT1\GATA2\CHAD\LOC101751006\SUCLG1\INCENP\ARHGAP19\ATP6V0E2\RBM6\CRYAA\OVAL\POPDC3\NOXRED1\REEP5\LUC7L3\TMEM120B\CORO7\PSME3\CYP2H1\LIN7B\GNB1\SASH3\B4GALT7\AFAP1L1\ATCAY\
P<0.05	cgr-miR-151-3p	RDM1\KCTD9\RBBP5\ELAVL1\DLGAP4\SYF2\GLYR1\DTWD2\COMMD10\LZIC\SCPEP1\UBIAD1\CCND3\ANAPC7\KRT20\BET1L\ADAM10\RALGPS2\COL18A1\CENPP\PHACTR3\DBT\ARHGEF3\RAB7A\RUVBL1\KLHL13\TRAPPC2L\SNRPD3\KIF21A\FADS2\ANKDD1A\CD3E\YME

		1L1\LOC416354\APIP\DCX\TMEM180\CRELD2\ITGA8\FXR1\CRDS2\SP3\PLEK2\XRCC6BP1\TMBIM4\RAP1B\FAM177A1\LEF1\TFB2M\PPPDE1\FBXO8\IGF2BP3\ROR1\FGFR1OP\BIN1\MYLK\DDX47\ATF7IP\HIF1A\CCDC93\SOX10\KIAA1549\M6PR\UCHL1\FBXL5\MBLAC2\RLN3\AMD1\C9ORF93\ZFAND5\TBC1D23\ACOT12\SNX30\JAM2\DECR1\IFNGR2\TP53INP1\OXR1\FAM49A\CYP39A1\ATP4B\LECT1\UBE2N\CERK\WDR48\SPECC1L\GLT1D1\CCDC39\SUGT1\SOD1\RPL15\NCF4\FAM169B\PGRMC1\TNFRSF10B\SLU7\
P<0.05	bta-miR-450a	TMOD3\SCG3\RASD1\HELLS\RRAS2\PRKAG2\TTC36\RNF185\CCDC40\RMI2\YIPF5\NR1H4\FARS2\MBP\LIMCH1\MYH10\GPR89B\PRKAG2\CYP24A1\
P<0.05	aca-miR-425-5p_R+4	GFPT1\CAMK4\PSMF1\RPS6KA1\SLC16A4\HBXIP\MYL4\HARS\FAM125B\SYF2\SNAP29\RRAGC\WDR45L\NFS1\LOC417345\DGKD\UPK3BL\DYNLRB1\POLR2D\SORD\RECQL5\RGS2\PIK3CD\SRM\SCPEP1\EIF2B1\GBGT1\KIAA1191\OSMR\PBLD\CCNB2\SLTM\KDM2B\FAM20B\BLMH\SLC47A2\URM1\WDFY1\GTPBP5\SEC22C\TPST2\MYD88\CEP19\NOM1\NAP1L4\ABCC1\UPF2\CTSA\ACADS\CREM\PGS1\RPS25\UPRT\C17ORF28\TMEM209\CHRDL1\RINT1\RELN\NEURL\CUL4B\LOC417741\ITGA8\PIGK\SNAP23\ATP6V0C\C6H10ORF46\KRIT1\SEC23IP\PDCD10\CFL2\MMACHC\CMPK1\LEF1\GTF2A1\TTC7B\SLC24A4\BZW2\AHR\RFTN1\PPP2R5C\SRSF11\ANKRD13C\ELK3\PECR\SLC25A3\BAG5\AMPH\PPP1R13B\IL8L1\PTPLB\HIF1A\FKBP9\LOC420849\KIAA1549\C1H12ORF35\ATP6V1E1\BID\GALNT1\PDK1\NPEPPS\SNX9\NETO1\LTV1\GPR98\ZBTB49\KIAA1524\GPR89B\TCEB1\HSPA13\SLBP\SYNCRIP\IMPG1\PTDSS1\ZNF451\PDK3\KLHL15\ENPP2\EFR3B\MTMR9\DOCK9\TGDS\SPRY2\NAA16\GPALPP1\SLC25A15\N4BP2L2\UBL3\SLC46A3\N6AMT2\PCF11\VAPA\SUV39H2\SLC25A14\PTGR2\AK6\DAB1\GCG\BZW1\ASIP\GLT1D1\GRIA4\SAT1\AKT1\NT5C3A\PCMT1\RPL35\PCSK7\HSBP1L1\
P<0.05	aca-miR-18a-5p	BNIP3L\MYL4\SLC7A6\MAN1C1\PIK3R5\NFS1\SLC22A7\DGKD\JMJD6\CCDC99\RFFL\POLR2E\KCTD7\C19H17ORF85\INPP5K\RFWD3\ERLIN1\RAB5C\ARL10\KIAA1191\STK4\GPR174\BET1L\TMIGD1\RCAN3\URI1\AGTRAP\SHISA5\SCG3\WDFY1\LOC416530\CYB5R2\HIF1AN\GCLM\MGAT4B\SLC13A5\RRAS2\CNOT6\GORASP1\MCM2\UCKL1\HPRT1\MAPK8\GINS1\DDT\SEC24A\INS\PDE6C\FANCI\CABP1\SYNPR\RAB35\SPAG9\ACBD5\DLD\CD82\NR1H3\MPP4\SENP5\CYR61\TMEM87A\CDAN1\FNDC3B\C6H10ORF46\KLHL2\GAD1\ASNS\BTBD9\C4H8ORF40\MMACHC\MBNL1\MBNL1\HERC3\GYG1\OSTC\EPS15\NID1\LOC429115\CRADD\NGLY1\NR2C1\CRYZ\METAP2\ERI1\MYLK\ESR2\POLDIP3\HIF1A\CCR8\ELMO1\ENPEP\ITGA9\METAP1\SAV1\IYD\PMCH\LOC100858230\ADCY2\GRB10\CLPTM1L\FBXO15\PPAT\MAP7\KIT\FRYL\LIMCH1\PHC1\SS18\ZDHHC23\LOC427250\CDON\TBC1D23\ALCAM\ABLIM2\SH3BP2\TMEM129\SYNCRIP\PDK3\FBXO32\MBOAT2\KLF11\ASXL2\HMBOX1\MTMR9\GAL10\MGAT4A\NUPL1\MTMR2\FCHSD2\C1H21ORF33\SUCLG1\PLEKHA3\LMO4\MFI2\CDC42\NADK\IL7\TRIB2\IMPG2\LOC423536\PCMT1\PDLIM5\IRF2\LUC7L3\LOC422308\USP7\TUBAL3\YEATS2\APITD1\
P<0.05	aca-miR-181a_R+3	LOH11CR2A\RASSF2\LSM7\STRADA\ACOT7\PAK1\FAM125B\HSPA5\CCDC43\C20H20ORF24\ARMC8\SAMHD1\CLTCL1\PI4KA\CNTRL\WDR45L\NFS1\PSMD5\ATP6V1B2\WWC1\GNB1L\TRIM33\STXBP3\PRKCD\C15H22ORF25\LOC415324\AGO3\DTWD2\GOT2\GBAS\C10ORF58

		\UCHL5\PIK3CD\LZIC\C19H17ORF85\ATR\ZC3H15\GK5\UHMK1\DNAJC12\C26H6ORF106\RASA2\PHACTR4\TARDBP\SUGP2\MMD\MLX\UBIAD1\ACSS2\BARD1\IFT52\LPCAT2\TOP1\LIFR\SLC35E1\P2RX4\MYPN\FAM189A1\IQCA1\STK4\PNRC2\SRSF10\RNF111\DHRS3\GOSR1\SGPL1\P2RX5\MAD2L2\PDAP1\RGL1\NOL8\COPS3\DUT\MKRN2\TRIM37\GTPBP5\TUBD1\VCAM1\SLC25A36\GNG13\SASS6\APPBP2\ADAMTS18\JMJD4\LPPR5\ARHGEF3\SYNGR3\RNF141\TSC2\KLHL36\PITPNB\EMC8\SPPL2A\GCLM\PAFAH1B1\RAP1GAP2\MTF2\LOC100858792\KLHL13\ABTB1\KIAA0753\MOSPD1\PRKAG2\MLL3\CXCL14\PDPK1\SRPRB\KIAA1024\SEMA3A\KIF3B\FRA10AC1\BRSK2\TOLLIP\TAF3\PLOD2\IL13\CHKA\RPTOR\ARRDC4\ALDH1A3\RAB35\MTPAP\ABCC2\ARCN1\TMLHE\ABCC3\STX16\MTMR8\LOC416354\CHIC1\EME1\SALL4\GGA3\ACSL4\CELF1\MTAP\CASC4\PDCD11\LRRC17\PHTF2\SMARCA1\MCTS1\SENP5\GTF2H4\LOC424109\PPP3R1\C1D\IDH1\ATP6AP1\LCLAT1\CDC42BPA\C3H1ORF55\LEFTY2\GUCY1B3\PRDX3\SEC23IP\C2H7ORF64\MARK1\GXYLT1\PUS7L\RARRES1\RRP15\C1GALT1\RAB33B\PROX1\SRGAP1\STXBP6\SCFD1\TMEM184C\CFL2\KIF6\PSMA6\LRBA\MBIP\SCLT1\KANK1\TBC1D15\NAP1L1\TTLL5\MBNL1\MED12L\OSTC\ELAVL4\BIRC6\CC2D1B\SCIN\ANKMY2\ITPK1\RAPGEF5\INADL\DOCK7\LYST\PGM1\CBX3\COBLL1\C5H14ORF129\CCNK\C3H6ORF120\RFTN1\DCN\KAT2B\GPD1L\BAG5\KIF26A\CLASP1\ZDHHC3\UBP1\CAMK2D\SLC12A8\EIF3M\CASP6\ITGA9\STK17A\HNMT\NTRK2\CTSL2\TGFBR1\LOC101750080\DEK\TXNRD1\HSP90B1\EEF1E1\BLOC1S5\SLC37A3\STRAP\MGST1\PRMT8\LOC420992\LRRC16A\TFB1M\NPEPPS\PPAT\CEP76\BCLAF1\IGF2BP2\OCIAD1\TEC\M6PR\EDARADD\PI4K2B\CCDC58\MBLAC2\HDDC2\TNFRSF1A\FAM26E\LYAR\MLLT3\HAUS6\MAMDC2\GPR161\TCEA1\TMEM68\KIAA1524\CHMP2B\LRRCC1\ELOVL4\DECR1\PTDSS1\DOPEY2\ATP6V1C1\NOV\RAD21\DDX3X\OC90\DST\APOO\DSCC1\SH3KBP1\PDHA1\TAF1B\LAPTM4A\PIGA\RAB9A\STMN4\RP2\SLC9A7\GAS6\ATP4B\TNFSF13B\LIG4\IPO5\RBM26\BORA\MZT1\RNASEH2B\GDPD5\TXNL4A\SUV39H2\GZMK\CASP2\PACSIN2\LOC101750282\CERK\E2F5\C2H6ORF62\IFNGR1\SLC25A14\PDDC1\EAF2\PLEKHA3\C2ORF47\SPECC1L\RNF7\PRR11\SLC13A2\C26H6ORF89\C19ORF44\RANBP2\TMEM131\WWP1\APP\MTO1\KDM3A\DNAJA1\HOPX\ATP2B1\SLC10A7\ZBTB34\GATM\PRKAG2\NELL2\GRIA2\AMACR\TBC1D22A\ATP2A2\NAALADL2\NUP188\EIF4A2\CD36\REL\LSG1\EPS8L2\LOC422654\
P<0.05	aca-miR-150-5p_1ss12TC	TRIM39\WDR36\SPPL2B\RPA2\GPATCH3\C20H20ORF24\NR5A1\WBSCR17\ETS1\PIK3R5\SLC25A1\FAM18B1\HSD11B1\SNX19\RAB14\WDR45L\UBE2R2\UBAC1\SLC16A1\MRPS34\SDF4\C15H22ORF25\POLR2D\TAF15\RAD51D\AGO3\HSPA9\OLFM1\CALCRL\GK5\RCN2\PHACTR4\LRRC45\BOD1\DHX37\GSS\SKP2\ATIC\FEN1\NELL1\PIGT\LOC101749801\CHRNA7\ADA\SOAT1\AR\RGS9BP\LOC768735\VPS53\PTGS2\WDFY1\RPS6KB1\CDH5\ADAMTS18\RNF141\TMEM41B\TMED5\KLHL13\TXNRD3\GSTT1\SRPRB\CRTAM\C9H21ORF2\VIPR2\POFUT1\AP2A2\IGF1R\CREM\PCSK6\ZEB1\CLPX\RAB35\TMLHE\RRP12\SLC9A3R1\FOXP1\CD44\PCGF6\ORC2\ATG4A\NAPEPLD\GTF2H4\PDCD4\SAMD13\AK5\PRKCI\EXD2\HAT1\YAF2\C5H15ORF29\LOC422448\RAP1B\FAM177A1\ABCD4\RSPH9\CMPK1\ANKMY2\ITPK1\HDAC9\SNX10\C5H14ORF129\EML1\C3H6ORF120\KAT2B\TECPR2\SLC44A5\EXOC3L4\ANKS1B\ACTR6\ATP2C1\ESR2\ATF7IP\CLEC3B

		\CTNNB1\PRRG4\DNAL4\CBY1\STK17A\MARCH7\C22ORF28\PWP1\RIC8B\JARID2\MYLIP\TXNRD1\EEF1E1\CREB3L2\ADIPOR2\ROPN1L\ST8SIA1\ERP44\MTHFD2\LNX1\MYB\SYTL2\PPFIBP1\ARFGAP3\PARVB\M6PR\PHC1\CADM1\MEGF10\HDDC2\SERINC1\METTL16\RORB\ST3GAL6\CA2\TP53INP1\HMGN1\PTP4A1\PDK3\PDIA6\MTMR9\EIF5B\C1H2ORF49\DZIP1\CEP57\FZD4\LCORL\VAPA\RNASEL\LOC426128\TPD52L2\MBNL2\SLC26A3\SYT4\INCENP\FAM175B\DAB1\PPP4R2\C20ORF96\GLT1D1\C26H6ORF89\BMPR1B\SGOL1\CRIM1\SSTR2\ZBTB34\CNTN1\MYO5A\MYO19\TMEM120B\LSG1\YOD1\RPUSD4\
P<0.05	aca-miR-138-2-3p_L-2R+3	METTL2A\LAPTM5\HNRNPR\C20H20ORF24\CLTCL1\IRF1\RBM39\PRKRIP1\GNB1L\STXBP3\NCBP1\CHMP4B\LIG3\PUS1\FRZB\STAR\HDAC1\GOLGA7\GLUL\POLB\TMEM38A\MINA\MFAP3\RCAN3\BAP1\RGS9BP\TRIM37\USP32\GRHPR\PALMD\WSB1\TNFAIP1\GCLM\TMED5\ABTB1\DNAJC5\STMN3\KDM8\HTATSF1\PDPK1\POFUT1\KIF11\CHKA\HDX\ATP13A4\MTPAP\ARCN1\KMO\CDC23\PCK1\CHIC1\SALL4\MLLT10\ANKRD44\TMEM180\CHRDL1\TYW5\CFLAR\PDCD11\APLF\CROT\DEGS1\CTSO\IFRD1\GXYLT1\IRAK4\AQR\CAND1\IFNG\G2E3\CFL2\BRE\GPR149\AGA\MACC1\CBX3\RFTN1\NDUFA12\LHX8\SEC22A\SERHL2\L3MBTL2\HNMT\C5H14ORF166\BLOC1S5\ZBTB2\DAP\CLPTM1L\CNDP2\GPR126\BCLAF1\ARFGAP3\PI4K2B\SGTB\SERINC1\TNFRSF1A\RIOK3\B4GALT6\LOC396120\KIAA1524\CD200\SDR16C5\CHMP2B\UFL1\JAM2\NT5E\SCAF4\PRIM2\GSTA3\CALB1\REPS2\RP2\TNFSF13B\DNAJC3\IRG1\DNAJC15\UFM1\N4BP2L2\MTMR2\FZD4\GDPD5\VAPA\MLANA\CLN8\SYK\MCM6\MCCC1\PLEKHA3\SPECC1L\TOM1L1\GYPC\LUC7L3\GLDC\CRB2\XIRP1\DDX52\TBCD\COG5\LMAN1\RAD50\ZNF488\YOD1\LAS1L\MAP3K14\SNRNP40\LEPROTL1\
P<0.05	aca-miR-129b-5p_R+1_1ss20TG	ARMC1\RASSF2\BNIP3L\KLHL21\KIAA0556\WNT11\FAM192A\SNAP29\LOC428335\ANP32B\CAMKK1\UBE2R2\ODZ2\EIF6\MAP1LC3A\DYNLRB1\GNB1L\PTBP1\ACOX1\FAM168B\RAD51D\AGO3\DTWD2\GBAS\TSPAN14\MATR3\RGS4\CZH9ORF100\C19H17ORF85\LIPG\INPP5K\NTAN1\GRM4\RFNG\TRPC4AP\CDK2AP1\DNAJC7\FNDC5\RNLS\LOC101749631\POLB\MPHOSPH10\CHRNA7\PNRC2\YWHAB\MCU\BCL7A\RSRC2\HVCN1\MAPK6\WNT4\PEPD\CENPI\APPBP2\HAGHL\HCCS\SYNGR3\SLC44A3\RNF141\SNX12\GCLM\DCAF12\TMED5\COL6A2\PRKAG2\PAXIP1\CXCL14\FOPNL\VIPR2\SNRPD3\MAP3K13\CHID1\KLHL4\BDH1\ACBD5\CDC23\STAT1\EGR1\MAP2K1\USP12P1\COPS7B\APIP\CD44\VRK2\RNF114\WSB2\CTDSPL2\CASC4\PCGF6\FBXL13\CCNYL1\FASTKD2\NUSAP1\EEF1B2\LOC417741\TUBGCP6\ITGA8\NDUFA5\MDH1\PPP1R21\YPEL5\ZFP106\PNLIP\LIN9\KRIT1\CAV2\SLC39A9\HAT1\MSMO1\CPXM2\NAA15\ANGEL2\AQR\SNX6\CNOT2\CCDC167\MME\VASH1\ANGEL1\ELAVL4\YIPF4\GTF2A1\PPPDE1\CPSF2\MEOX2\ITPK1\INADL\CBX3\LEPROT\CHN2\SERPINH1\ANKRD28\NDUFA12\LTA4H\BRP44L\DYNC1LI1\BAG5\MYLK\ATF7IP\CYP2D6\FSHB\WDHD1\SAMD4A\L2HGDH\NUP43\MMADHC\FBXO7\GKAP1\CZH9ORF3\DEK\MYLIP\FARS2\SERPINB10\ZBTB2\CCDC127\RECQL\ABCC9\STX17\NPEPPS\MAPK9\FUCA2\SLMO1\C2H18ORF19\KIT\LNX1\FAM38B\TEC\PLA2G2A\PARVB\NUP50\PDS5A\LOC431003\MBLAC2\EPHB6\CZH5ORF34\PARP8\TNFRSF1A\ABHD3\FAM26E\NSG1\AMD1\NFKBIZ\VCPIP1\JAKMIP1\ABLIM2\HAPLN1\TCEB1\PTGR1\RIPK2\PLEKHF2\UBOX5\MORC3\CHAF1B\WRB\DMD\ADCY8\

	C2H8ORF76\REPS2\ACE2\PIGA\C1H2ORF49\TUBGCP3\RCBTB1\HMGB1\UBL3\MTMR2\CTSC\VAPA\PSMB4\SEPT6\GATA2\E2F5\TERF1\GAL7\C3H2ORF43\MCPH1\NCOA7\SUCLG1\LOC427470\GLT1D1\POU1F1\IMPG2\SLIT2\ANLN\PDLIM5\SLC12A2\HMOX1\CDCA7L\USP37\SLC10A7\GARNL3\NEK1\GABPB1\PRKAG2\NUB1\CNTN1\XIRP1\CD151\BIRC5\COL17A1\MYO19\NUF2\ZNF488\PODXL\

Table S6. The significantly enriched GO terms (P < 0.05) of predicted targets of differentially expressed miRNAs which selected with the criteria of P < 0.05 and fold changes either > 1.5 or < 0.67.

GO Id	Function	GO term	S gene number	TS gene number	B gene number	TB gene number	P Value
GO:0000910	Biological process	cytokinesis	7	1,483	7	2,717	0.014
GO:0045860		positive regulation of protein kinase activity	7	1,483	7	2,717	0.014
GO:0007032		endosome organization	7	1,483	7	2,717	0.014
GO:0009117		nucleotide metabolic process	6	1,483	6	2,717	0.026
GO:0016575		histone deacetylation	6	1,483	6	2,717	0.026
GO:0000209		protein polyubiquitination	8	1,483	9	2,717	0.036
GO:0030307		positive regulation of cell growth	8	1,483	9	2,717	0.036
GO:0006397		mRNA processing	26	1,483	37	2,717	0.037
GO:0008152		metabolic process	21	1,483	29	2,717	0.038
GO:0006511		ubiquitin-dependent protein catabolic process	17	1,483	23	2,717	0.046
GO:0010951		negative regulation of endopeptidase activity	5	1,483	5	2,717	0.048
GO:0009058		biosynthetic process	5	1,483	5	2,717	0.048

GO:0060766		negative regulation of androgen receptor signaling pathway	5	1,483	5	2,717	0.048
GO:0016180		snRNA processing	5	1,483	5	2,717	0.048
GO:0060740		prostate gland epithelium morphogenesis	5	1,483	5	2,717	0.048
GO:0043388		positive regulation of DNA binding	5	1,483	5	2,717	0.048
GO:0030030		cell projection organization	5	1,483	5	2,717	0.048
GO:0005739		mitochondrion	135	1,483	190	2,717	0.000
GO:0005737		cytoplasm	476	1,483	780	2,717	0.000
GO:0016020		membrane	268	1,483	448	2,717	0.008
GO:0005769		early endosome	13	1,483	15	2,717	0.010
GO:0005829	Cellular	cytosol	68	1,483	103	2,717	0.011
GO:0005635	component	nuclear envelope	7	1,483	7	2,717	0.014
GO:0005759		mitochondrial matrix	14	1,483	17	2,717	0.017
GO:0005669		transcription factor TFIID complex	6	1,483	6	2,717	0.026
GO:0016581		NuRD complex	6	1,483	6	2,717	0.026
GO:0032039		integrator complex	6	1,483	6	2,717	0.026

GO:0005730		nucleolus	48	1,483	74	2,717	0.045
GO:0000793		condensed chromosome	5	1,483	5	2,717	0.048
GO:0042382		paraspeckles	5	1,483	5	2,717	0.048
GO:0035098		ESC/E(Z) complex	5	1,483	5	2,717	0.048
GO:0005852		eukaryotic translation initiation factor 3 complex	5	1,483	5	2,717	0.048
GO:0000166	Molecular function	nucleotide binding	231	1,483	372	2,717	0.001
GO:0016787		hydrolase activity	93	1,483	139	2,717	0.002
GO:0008026		ATP-dependent helicase activity	10	1,483	10	2,717	0.002
GO:0019003		GDP binding	10	1,483	10	2,717	0.002
GO:0005524		ATP binding	168	1,483	271	2,717	0.006
GO:0004721		phosphoprotein phosphatase activity	8	1,483	8	2,717	0.008
GO:0003723		RNA binding	55	1,483	81	2,717	0.009
GO:0003743		translation initiation factor activity	13	1,483	15	2,717	0.010
GO:0000287		magnesium ion binding	23	1,483	30	2,717	0.010
GO:0019899		enzyme binding	15	1,483	18	2,717	0.011

GO:0004386	helicase activity	17	1,483	21	2,717	0.011
GO:0004674	protein serine/threonine kinase activity	26	1,483	35	2,717	0.013
GO:0016301	kinase activity	18	1,483	23	2,717	0.017
GO:0016772	transferase activity, transferring phosphorus-containing groups	16	1,483	20	2,717	0.017
GO:0042802	identical protein binding	38	1,483	55	2,717	0.019
GO:0016491	oxidoreductase activity	39	1,483	57	2,717	0.022
GO:0000975	regulatory region DNA binding	6	1,483	6	2,717	0.026
GO:0003777	microtubule motor activity	6	1,483	6	2,717	0.026
GO:0016740	transferase activity	55	1,483	85	2,717	0.036
GO:0005488	binding	66	1,483	104	2,717	0.039
GO:0016702	oxidoreductase activity, acting on single donors with incorporation of molecular oxygen, incorporation of two atoms of oxygen	10	1,483	12	2,717	0.039
GO:0032041	NAD-dependent histone deacetylase activity (H3-K14 specific)	5	1,483	5	2,717	0.048

GO:0016791	phosphatase activity	5	1,483	5	2,717	0.048
GO:0046969	NAD-dependent histone deacetylase activity (H3-K9 specific)	5	1,483	5	2,717	0.048
GO:0034739	histone deacetylase activity (H4-K16 specific)	5	1,483	5	2,717	0.048
GO:0046970	NAD-dependent histone deacetylase activity (H4-K16 specific)	5	1,483	5	2,717	0.048

Table S7. The significantly enriched KEGG pathways (P < 0.05) of predicted targets of differentially expressed miRNAs which selected with the criteria of *P* < 0.05 and fold changes either > 1.5 or < 0.67.

Pathway Id	pathway description	S gene number	TS gene number	B gene number	TB gene number	P value
ko00071	Fatty acid metabolism	23	1,576	30	3,670	0.000
ko00330	Arginine and proline metabolism	27	1,576	37	3,670	0.000
ko03013	RNA transport	67	1,576	114	3,670	0.000
ko00280	Valine, leucine and isoleucine degradation	27	1,576	38	3,670	0.000
ko00620	Pyruvate metabolism	23	1,576	32	3,670	0.001
ko04114	Oocyte meiosis	53	1,576	89	3,670	0.001
ko04110	Cell cycle	60	1,576	103	3,670	0.001
ko00010	Glycolysis / Gluconeogenesis	28	1,576	44	3,670	0.004
ko04510	Focal adhesion	89	1,576	169	3,670	0.006
ko04141	Protein processing in endoplasmic reticulum	71	1,576	132	3,670	0.007
ko03022	Basal transcription factors	21	1,576	32	3,670	0.008
ko03040	Spliceosome	54	1,576	98	3,670	0.009

ko00640	Propanoate metabolism	17	1,576	25	3,670	0.010
ko03320	PPAR signaling pathway	32	1,576	54	3,670	0.011
ko00480	Glutathione metabolism	21	1,576	33	3,670	0.013
ko04914	Progesterone-mediated oocyte maturation	40	1,576	71	3,670	0.015
ko03008	Ribosome biogenesis in eukaryotes	36	1,576	63	3,670	0.015
ko03015	mRNA surveillance pathway	35	1,576	61	3,670	0.016
ko04650	Natural killer cell mediated cytotoxicity	37	1,576	66	3,670	0.021
ko04146	Peroxisome	37	1,576	66	3,670	0.021
ko03018	RNA degradation	32	1,576	56	3,670	0.022
ko04120	Ubiquitin mediated proteolysis	60	1,576	115	3,670	0.027
ko00270	Cysteine and methionine metabolism	19	1,576	31	3,670	0.030

Table S8. The significantly enriched GO terms (P < 0.05) of predicted targets of differentially expressed miRNAs which selected with the criteria of FDR < 0.05 and fold changes either > 1.5 or < 0.67.

GO Id	Function	GO term	S gene number	TS gene number	B gene number	TB gene number	P Value
GO:0000910	Biological process	cytokinesis	6	934	7	2,717	0.008
GO:0007032		endosome organization	6	934	7	2,717	0.008
GO:0000209		protein polyubiquitination	7	934	9	2,717	0.010
GO:0007512		adult heart development	4	934	4	2,717	0.014
GO:0018105		peptidyl-serine phosphorylation	4	934	4	2,717	0.014
GO:0009117		nucleotide metabolic process	5	934	6	2,717	0.020
GO:0007179		transforming growth factor beta receptor signaling pathway	8	934	12	2,717	0.023
GO:0030509		BMP signaling pathway	10	934	17	2,717	0.033
GO:0006886		intracellular protein transport	13	934	24	2,717	0.036
GO:0001952		regulation of cell-matrix adhesion	3	934	3	2,717	0.041
GO:0051225		spindle assembly	3	934	3	2,717	0.041

GO:0034145		positive regulation of toll-like receptor 4 signaling pathway	3	934	3	2,717	0.041
GO:0045671		negative regulation of osteoclast differentiation	3	934	3	2,717	0.041
GO:0055015		ventricular cardiac muscle cell development	3	934	3	2,717	0.041
GO:0006807		nitrogen compound metabolic process	3	934	3	2,717	0.041
GO:0048265		response to pain	3	934	3	2,717	0.041
GO:0031647		regulation of protein stability	3	934	3	2,717	0.041
GO:0030307		positive regulation of cell growth	6	934	9	2,717	0.049
GO:0006874		cellular calcium ion homeostasis	6	934	9	2,717	0.049
GO:0016020	Cellular component	membrane	186	934	448	2,717	0.000
GO:0005764		lysosome	10	934	16	2,717	0.020
GO:0005739		mitochondrion	78	934	190	2,717	0.028
GO:0000139		Golgi membrane	24	934	51	2,717	0.040
GO:0019005		SCF ubiquitin ligase complex	3	934	3	2,717	0.041
GO:0055038		recycling endosome membrane	3	934	3	2,717	0.041
GO:0030132		clathrin coat of coated pit	3	934	3	2,717	0.041

GO:0030131		clathrin adaptor complex	3	934	3	2,717	0.041
GO:0030897		HOPS complex	3	934	3	2,717	0.041
GO:0005829		cytosol	44	934	103	2,717	0.045
GO:0016363		nuclear matrix	6	934	9	2,717	0.049
GO:0016301		kinase activity	16	934	23	2,717	0.001
GO:0000166		nucleotide binding	151	934	372	2,717	0.004
GO:0004842		ubiquitin-protein transferase activity	15	934	25	2,717	0.007
GO:0000287		magnesium ion binding	17	934	30	2,717	0.010
GO:0042802		identical protein binding	27	934	55	2,717	0.016
GO:0005096	Molecular function	GTPase activator activity	11	934	18	2,717	0.018
GO:0019003		GDP binding	7	934	10	2,717	0.023
GO:0016491		oxidoreductase activity	27	934	57	2,717	0.028
GO:0005524		ATP binding	107	934	271	2,717	0.037
GO:0016787		hydrolase activity	58	934	139	2,717	0.039
GO:0005114		type II transforming growth factor beta receptor binding	3	934	3	2,717	0.041

GO:0016772	transferase activity, transferring phosphorus-containing groups	11	934	20	2,717	0.046
GO:0004714	transmembrane receptor protein tyrosine kinase activity	6	934	9	2,717	0.049

Table S9. The significantly enriched KEGG pathways (P < 0.05) of predicted targets of differentially expressed miRNAs which selected with the criteria of FDR < 0.05 and fold changes either > 1.5 or < 0.67.

Pathway Id	Pathway description	S gene number	TS gene number	B gene number	TB gene number	P value
ko04142	Lysosome	37	860	92	3,670	0.000
ko04120	Ubiquitin mediated proteolysis	43	860	115	3,670	0.000
ko04650	Natural killer cell mediated cytotoxicity	25	860	66	3,670	0.006
ko00071	Fatty acid metabolism	13	860	30	3,670	0.012
ko04122	Sulfur relay system	6	860	10	3,670	0.014
ko03013	RNA transport	37	860	114	3,670	0.016
ko03015	mRNA surveillance pathway	22	860	61	3,670	0.017
ko03022	Basal transcription factors	13	860	32	3,670	0.022
ko04146	Peroxisome	23	860	66	3,670	0.023
ko04114	Oocyte meiosis	29	860	89	3,670	0.029
ko00062	Fatty acid elongation	4	860	6	3,670	0.030
ko00330	Arginine and proline metabolism	14	860	37	3,670	0.034

ko04150	mTOR signaling pathway	15	860	41	3,670	0.039
ko04110	Cell cycle	32	860	103	3,670	0.044
ko03008	Ribosome biogenesis in eukaryotes	21	860	63	3,670	0.046

Table S10. Summary of differentially expressed mRNAs between young chicks group (immature livers) and adult chickens group (mature livers).

gene	Description	YC_fpkm	AC_fpkm	log2(fold_change)	regulation	FDR_value
SMO	smoothened, frizzled class receptor [Source:HGNC Symbol;Acc:HGNC:11119]	13.6243	4.5794	1.57296	up	0.00057
DHTKD1	dehydrogenase E1 and transketolase domain containing 1 [Source:HGNC Symbol;Acc:HGNC:23537]	61.4474	26.945	1.18933	up	0.00057
CAMK1D	calcium/calmodulin dependent protein kinase ID [Source:HGNC Symbol;Acc:HGNC:19341]	0.5093	3.7228	-2.86971	down	0.00057

		3				
FGL2	fibroleukin precursor [Source:RefSeq peptide;Acc:NP_001072954]	1.2<br>242<br>5	16.<br>396<br>4	-3.7434<br>2	do<br>wn	0.00<br>057
GTSE1	G2 and S phase-expressed protein 1 [Source:RefSeq peptide;Acc:NP_001026503]	3.6<br>748<br>3	0.5<br>303<br>9	2.79254	up	0.00<br>057
DOCK4	dedicator of cytokinesis 4 [Source:HGNC Symbol;Acc:HGNC:19192]	2.2<br>879<br>1	5.1<br>114<br>7	-1.1597<br>1	do<br>wn	0.00<br>057
SLC16A7	monocarboxylate transporter 2 [Source:RefSeq peptide;Acc:NP_001186533]	0.6<br>131<br>4	1.6<br>286<br>8	-1.4094<br>2	do<br>wn	0.00<br>057

HMGI-C	Gallus gallus high mobility group AT-hook 2 (HMGA2), mRNA. [Source:RefSeq mRNA;Acc:NM_205001]	1.6 612 4	0.2 619 9	2.66468	up	0.00 057
CCT2	T-complex protein 1 subunit beta [Source:RefSeq peptide;Acc:NP_001012551]	9.9 729 8	4.9 549 3	1.00916	up	0.00 057
GLIPR1L	glioma pathogenesis-related protein 1-like precursor [Source:RefSeq peptide;Acc:NP_001025914]	1.8 548 2	12. 303 6	-2.7297 3	do wn	0.00 057
SOCS2	suppressor of cytokine signaling 2 [Source:RefSeq peptide;Acc:NP_989871]	2.1 212 8	6.6 907 5	-1.6572 3	do wn	0.00 057
SNRPF	small nuclear ribonucleoprotein F [Source:RefSeq peptide;Acc:NP_001264291]	58.	28.	1.03332	up	0.00

		812<br>6	734<br>9			057
SLC25A3	phosphate carrier protein, mitochondrial [Source:RefSeq peptide;Acc:NP_001006236]	452<br>.23<br>7	203<br>.19<br>1	1.15424	up	0.00<br>057
CENPM	Centromere protein M [Source:UniProtKB/Swiss-Prot;Acc:Q1T7B9]	3.8<br>316<br>5	0.2<br>624<br>5	3.86786	up	0.00<br>057
MFNG	MFNG O-fucosylpeptide 3-beta-N-acetylglucosaminyltransferase [Source:HGNC Symbol;Acc:HGNC:7038]	0.2<br>267<br>6	1.1<br>993<br>4	-2.4029<br>8	do<br>wn	0.00<br>057
RAC2	ras-related C3 botulinum toxin substrate 2 [Source:RefSeq peptide;Acc:NP_001188381]	7.9<br>071	46.<br>008	-2.5406<br>7	do<br>wn	0.00<br>057

		2	5			
NUAK1	NUAK family, SNF1-like kinase, 1 [Source:HGNC Symbol;Acc:HGNC:14311]	11.1996	23.4731	-1.06756	down	0.00057
AKR1D1	3-oxo-5-beta-steroid 4-dehydrogenase [Source:RefSeq peptide;Acc:NP_001264322]	199.046	69.7546	1.51274	up	0.00057
PKP2	plakophilin 2 [Source:HGNC Symbol;Acc:HGNC:9024]	2.60388	0.17193	3.92078	up	0.00057
DENND5B	DENN/MADD domain containing 5B [Source:HGNC Symbol;Acc:HGNC:28338]	16.9115	6.62393	1.35224	up	0.00057

BPGM	bisphosphoglycerate mutase [Source:RefSeq peptide;Acc:NP_001025939]	3.1432	1.35086	1.21835	up	0.00057
BCAT1	branched chain amino-acid transaminase 1, cytosolic [Source:HGNC Symbol;Acc:HGNC:976]	5.48561	12.6133	-1.20122	down	0.00057
GYS2	glycogen synthase 2 [Source:HGNC Symbol;Acc:HGNC:4707]	26.9206	65.2603	-1.2775	down	0.00057
PARVG	parvin gamma [Source:HGNC Symbol;Acc:HGNC:14654]	0.44918	2.22679	-2.30961	down	0.00057
FOXM1	forkhead box protein M1 [Source:RefSeq peptide;Acc:NP_001012973]	4.3	1.0	2.11444	up	0.00

		950 2	149 7			057
SLC2A3	solute carrier family 2, facilitated glucose transporter member 3 [Source:RefSeq peptide;Acc:NP_990842]	1.0 676 1	4.7 960 7	-2.1674 7	do wn	0.00 057
CASR	calcium-sensing receptor [Source:HGNC Symbol;Acc:HGNC:1514]	0.0 414 6	1.5 589	-5.2325 9	do wn	0.00 057
FAM162 A	family with sequence similarity 162 member A [Source:HGNC Symbol;Acc:HGNC:17865]	45. 220 9	11. 484 8	1.97727	up	0.00 057
TAPBPL	tapasin-related protein [Source:RefSeq peptide;Acc:NP_001026543]	1.4 203	3.4 073	-1.2624 2	do wn	0.00 057

		4	6			
GAPDH	Glyceraldehyde-3-phosphate dehydrogenase [Source:UniProtKB/Swiss-Prot;Acc:P00356]	253<br>5.9<br>1	125<br>6.8<br>3	1.01272	up	0.00<br>057
TPI1	Triosephosphate isomerase [Source:UniProtKB/Swiss-Prot;Acc:P00940]	445<br>.14<br>6	162<br>.25<br>7	1.456	up	0.00<br>057
ATN1	atrophin 1 [Source:HGNC Symbol;Acc:HGNC:3033]	3.3<br>058<br>7	0.9<br>743<br>6	1.76251	up	0.00<br>057
PTPN6	tyrosine-protein phosphatase non-receptor type 6 [Source:RefSeq peptide;Acc:NP_001026655]	4.2<br>243<br>3	25.<br>662<br>9	-2.6028<br>9	do<br>wn	0.00<br>057

C1S	complement C1s subcomponent precursor [Source:RefSeq peptide;Acc:NP_001025948]	260.113	575.055	-1.14456	down	0.00057
DPT	dermatopontin [Source:HGNC Symbol;Acc:HGNC:3011]	1.65082	3.69882	-1.16388	down	0.00057
ALCAM	CD166 antigen [Source:UniProtKB/Swiss-Prot;Acc:P42292]	1.19282	2.5906	-1.11892	down	0.00057
CREG1	cellular repressor of E1A-stimulated genes 1 [Source:HGNC Symbol;Acc:HGNC:2351]	15.9238	72.1066	-2.17894	down	0.00057
PTGFRN	prostaglandin F2 receptor inhibitor [Source:HGNC Symbol;Acc:HGNC:9601]	6.4	2.5	1.34947	up	0.00

		424	282			057
		1	3			
IFNAR1	interferon alpha/beta receptor 1 precursor [Source:RefSeq peptide;Acc:NP_990190]	6.9	21.	-1.6271	do	0.00
		644	513	6	wn	057
		5	5			
ABCG1	ATP binding cassette subfamily G member 1 [Source:HGNC Symbol;Acc:HGNC:73]	0.1	1.0	-2.4885	do	0.00
		846	363	3	wn	057
		7	6			
MAOB	monoamine oxidase B [Source:HGNC Symbol;Acc:HGNC:6834]	3.5	9.0	-1.3746	do	0.00
		031	839	6	wn	057
		7	8			
TMEM47	transmembrane protein 47 [Source:HGNC Symbol;Acc:HGNC:18515]	0.5	1.8	-1.7532	do	0.00
		464	419		wn	057

			4			
TMEM27	transmembrane protein 27 [Source:HGNC Symbol;Acc:HGNC:29437]	0.1<br>910<br>8	0.6<br>738<br>4	-1.8182<br>4	do<br>wn	0.00<br>057
FIGF	vascular endothelial growth factor D precursor [Source:RefSeq peptide;Acc:NP_989899]	0.0<br>638<br>2	0.7<br>674<br>5	-3.5880<br>3	do<br>wn	0.00<br>057
ENSGAL<br>G000000<br>16691	NA	370<br>.72<br>8	155<br>.19<br>7	1.25627	up	0.00<br>057
SLC9A7	solute carrier family 9, subfamily A (NHE7, cation proton antiporter 7), member 7 [Source:HGNC Symbol;Acc:HGNC:17123]	2.5<br>225<br>3	5.5<br>874<br>5	-1.1473<br>2	do<br>wn	0.00<br>057

ATP10A	ATPase, class V, type 10A [Source:HGNC Symbol;Acc:HGNC:13542]	0.5 085 7	1.5 311 6	-1.5901 2	do wn	0.00 057
IL1RL1	interleukin-1 receptor-like 1 isoform L precursor [Source:RefSeq peptide;Acc:NP_989606]	11. 860 5	73. 553 6	-2.6326 3	do wn	0.00 057
RASA3	ras GTPase-activating protein 3 [Source:RefSeq peptide;Acc:NP_001012843]	1.1 784 9	3.1 362 8	-1.4121 2	do wn	0.00 057
GAS6	growth arrest specific 6 [Source:HGNC Symbol;Acc:HGNC:4168]	1.2 938 2	5.8 966 1	-2.1882 5	do wn	0.00 057
DIS3	DIS3 homolog, exosome endoribonuclease and 3'-5' exoribonuclease [Source:HGNC	5.0	2.1	1.23138	up	0.00

	Symbol;Acc:HGNC:20604]	112<br>1	343<br>3			057
ENSGAL<br>G000000<br>16934	Uncharacterized protein [Source:UniProtKB/TrEMBL;Acc:F1NY34]	0.2<br>894<br>6	1.8<br>920<br>3	-2.7085<br>1	do<br>wn	0.00<br>057
DIAPH3	diaphanous related formin 3 [Source:HGNC Symbol;Acc:HGNC:15480]	1.1<br>687<br>3	0.1<br>167<br>4	3.32353	up	0.00<br>057
FNDC3A	fibronectin type-III domain-containing protein 3a [Source:RefSeq peptide;Acc:NP_001012844]	13.<br>708<br>3	29.<br>679<br>5	-1.1144<br>2	do<br>wn	0.00<br>057
SLC46A3	solute carrier family 46 member 3 precursor [Source:RefSeq peptide;Acc:NP_001025999]	0.7<br>894	4.1<br>399	-2.3906<br>7	do<br>wn	0.00<br>057

		6	6			
CENPJ	centromere protein J [Source:HGNC Symbol;Acc:HGNC:17272]	1.0	0.1	2.585	up	0.00
		259	709			057
			8			
ENSGAL	NA	22.	48.	-1.1203	do	0.00
G000000		367	628	8	wn	057
17239		7	4			
WNT11	Protein Wnt-11 [Source:UniProtKB/Swiss-Prot;Acc:P49339]	2.2	5.3	-1.2775	do	0.00
		124	634	1	wn	057
		5	4			
ENSGAL	NAD(P)(+)--arginine ADP-ribosyltransferase [Source:UniProtKB/TrEMBL;Acc:R4GGY0]	6.0	18.	-1.5835	do	0.00
G000000		156	029	3	wn	057
28988		7	1			

HPX	hemopexin [Source:HGNC Symbol;Acc:HGNC:5171]	252<br>1.3<br>3	714<br>4.2<br>1	-1.5025<br>9	do<br>wn	0.00<br>057
SBF1	SET binding factor 1 [Source:HGNC Symbol;Acc:HGNC:10542]	5.2<br>259<br>2	1.2<br>584	2.0541	up	0.00<br>057
NET1	neuroepithelial cell-transforming gene 1 protein [Source:RefSeq peptide;Acc:NP_001025819]	6.6<br>622	17.<br>241<br>2	-1.3717<br>9	do<br>wn	0.00<br>057
FAM107<br>B	family with sequence similarity 107 member B [Source:HGNC Symbol;Acc:HGNC:23726]	3.1<br>052<br>1	7.0<br>209<br>7	-1.1769<br>8	do<br>wn	0.00<br>057
NAPEPL	N-acyl-phosphatidylethanolamine-hydrolyzing phospholipase D precursor [Source:RefSeq	3.2	8.5	-1.3730	do	0.00

D	peptide;Acc:NP_001025901]	917<br>6	264<br>2	8	wn	057
IFRD1	interferon-related developmental regulator 1 [Source:RefSeq peptide;Acc:NP_001001468]	41.<br>622<br>3	16.<br>441<br>5	1.34002	up	0.00<br>057
PPM1H	protein phosphatase, Mg2+/Mn2+ dependent 1H [Source:HGNC Symbol;Acc:HGNC:18583]	0.9<br>853<br>2	2.0<br>336<br>2	-1.0453<br>9	do<br>wn	0.00<br>057
CSRP2	Cysteine and glycine-rich protein 2 [Source:UniProtKB/Swiss-Prot;Acc:P50460]	1.1<br>263<br>2	4.5<br>598<br>5	-2.0173<br>6	do<br>wn	0.00<br>057
BTG1	Protein BTG1 [Source:UniProtKB/Swiss-Prot;Acc:P34743]	59.<br>645	205<br>.73	-1.7862<br>8	do<br>wn	0.00<br>057

		7	3			
UHRF1B	UHRF1 binding protein 1-like [Source:HGNC Symbol;Acc:HGNC:29102]	8.8	4.1	1.09022	up	0.00
P1L		572	601			057
		9	7			
ACO2	aconitate hydratase, mitochondrial [Source:RefSeq peptide;Acc:NP_989519]	167	55.	1.58901	up	0.00
		.18	572			057
		6	8			
ADSL	adenylosuccinate lyase [Source:RefSeq peptide;Acc:NP_990860]	567	220	1.36099	up	0.00
		.33	.87			057
		9	3			
ENSGAL	Uncharacterized protein [Source:UniProtKB/TrEMBL;Acc:R4GJW7]	0.1	0.8	-2.6114	do	0.00
G000000		322	079	3	wn	057
25881						

NCF4	neutrophil cytosol factor 4 [Source:RefSeq peptide;Acc:NP_001034361]	0.4<br>549	3.7<br>504<br>9	-3.0434<br>6	do<br>wn	0.00<br>057
MCM5	DNA replication licensing factor MCM5 [Source:RefSeq peptide;Acc:NP_001006243]	18.<br>477<br>6	1.6<br>164	3.51492	up	0.00<br>057
HMOX1	heme oxygenase 1 [Source:RefSeq peptide;Acc:NP_990675]	100<br>.84<br>4	495<br>.83<br>6	-2.2977<br>3	do<br>wn	0.00<br>057
ENSGAL<br>G000000<br>28627	Uncharacterized protein [Source:UniProtKB/TrEMBL;Acc:R4GFR4]	21.<br>465<br>1	52.<br>466<br>2	-1.2894	do<br>wn	0.00<br>057
TIMP3	Metalloproteinase inhibitor 3 [Source:UniProtKB/Swiss-Prot;Acc:P26652]	35.	88.	-1.3381	do	0.00

		066<br>2	654<br>5	1	wn	057
PWP1	periodic tryptophan protein 1 homolog [Source:RefSeq peptide;Acc:NP_001025932]	14.<br>354<br>5	6.5<br>450<br>5	1.13303	up	0.00<br>057
OCC-1	Overexpressed in colon carcinoma 1 protein homolog [Source:UniProtKB/Swiss-Prot;Acc:P0C915]	0.8<br>389	0.1<br>345<br>7	2.64009	up	0.00<br>057
DRAM1	DNA damage regulated autophagy modulator 1 [Source:HGNC Symbol;Acc:HGNC:25645]	0.5<br>059<br>4	4.3<br>749<br>3	-3.1122<br>3	do<br>wn	0.00<br>057
TRIM24	tripartite motif containing 24 [Source:HGNC Symbol;Acc:HGNC:11812]	1.9<br>320	5.3<br>400	-1.4667<br>5	do<br>wn	0.00<br>057

		1	6			
FGD4	FYVE, RhoGEF and PH domain containing 4 [Source:HGNC Symbol;Acc:HGNC:19125]	0.7697	4.32949	-2.49182	down	0.00057
CECR5	cat eye syndrome chromosome region, candidate 5 [Source:HGNC Symbol;Acc:HGNC:1843]	22.855	7.79783	1.55136	up	0.00057
ENSGALG00000013067	aldo-keto reductase family 1, member B10 (aldose reductase) [Source:RefSeq peptide;Acc:NP_001264828]	7.42096	26.0303	-1.81051	down	0.00057
PACSIN2	protein kinase C and casein kinase substrate in neurons protein 2 isoform 1 [Source:RefSeq peptide;Acc:NP_001152983]	3.83303	15.3844	-2.00491	down	0.00057

VWF	von Willebrand factor [Source:HGNC Symbol;Acc:HGNC:12726]	16.<br>304	38.<br>829	-1.2519<br>1	do<br>wn	0.00<br>057
ENSGAL<br>G000000<br>11930	Uncharacterized protein [Source:UniProtKB/TrEMBL;Acc:F1NEW8]	0.5<br>745<br>2	1.9<br>652<br>4	-1.7742<br>7	do<br>wn	0.00<br>057
LPAR5	lysophosphatidic acid receptor 5 [Source:HGNC Symbol;Acc:HGNC:13307]	0.2<br>766<br>5	1.4<br>283<br>7	-2.3682<br>6	do<br>wn	0.00<br>057
LPCAT3	lysophosphatidylcholine acyltransferase 3 [Source:HGNC Symbol;Acc:HGNC:30244]	41.<br>959	19.<br>739<br>8	1.08787	up	0.00<br>057
ENSGAL<br>G000000	Uncharacterized protein [Source:UniProtKB/TrEMBL;Acc:F1NAB7]	61.<br>458	132<br>.24	-1.1055<br>4	do<br>wn	0.00<br>057

14659		5	6			
FAM46C	Protein FAM46C [Source:UniProtKB/Swiss-Prot;Acc:Q5ZL95]	0.1	1.0	-2.4354	do	0.00
		961	612	3	wn	057
		9	4			
COL8A1	collagen, type VIII, alpha 1 [Source:HGNC Symbol;Acc:HGNC:2215]	1.1	3.3	-1.567	do	0.00
		317	532		wn	057
		5	3			
KIAA152	protein CIP2A homolog [Source:RefSeq peptide;Acc:NP_001025956]	1.8	0.3	2.21078	up	0.00
4		474	990			057
		4	8			
RCSD1	capZ-interacting protein [Source:RefSeq peptide;Acc:NP_001025960]	4.1	9.7	-1.226	do	0.00
		617	349		wn	057
		1	7			

ENSGAL G000000 16007	Uncharacterized protein [Source:UniProtKB/TrEMBL;Acc:F1P304]	488 .54	237 .33 6	1.04154	up	0.00 057
SIK1	serine/threonine-protein kinase SIK2 [Source:RefSeq peptide;Acc:NP_990013]	1.8 520 5	80. 808 7	-5.4473 2	do wn	0.00 057
ENSGAL G000000 16211	Uncharacterized protein [Source:UniProtKB/TrEMBL;Acc:E1C6Q2]	0.1 700 6	1.5 414	-3.1801 4	do wn	0.00 057
MAOA	monoamine oxidase A [Source:RefSeq peptide;Acc:NP_001025970]	4.5 025 4	12. 894	-1.5178 9	do wn	0.00 057
Nox2	cytochrome b-245, beta polypeptide [Source:RefSeq peptide;Acc:NP_001093756]	0.6	8.8	-3.6934	do	0.00

		877 8	978	3	wn	057
POLA1	polymerase (DNA directed), alpha 1, catalytic subunit [Source:HGNC Symbol;Acc:HGNC:9173]	4.1 689 4	1.1 757 2	1.82614	up	0.00 057
ENSGAL G000000 16326	Uncharacterized protein [Source:UniProtKB/TrEMBL;Acc:E1BTD2]	0.2 251 4	1.1 981 2	-2.4118 9	do wn	0.00 057
SAT1	Diamine acetyltransferase 1 [Source:UniProtKB/Swiss-Prot;Acc:Q8AXL1]	53. 381 4	128 .31 1	-1.2652 3	do wn	0.00 057
PDHA1	pyruvate dehydrogenase E1 component subunit alpha, somatic form, mitochondrial precursor [Source:RefSeq peptide;Acc:NP_001012562]	40. 807	13. 329	1.61418	up	0.00 057

		6	9			
REPS2	ralBP1-associated Eps domain-containing protein 2 [Source:RefSeq peptide;Acc:NP_001025977]	5.46205	0.91797	2.57293	up	0.00057
TMSB4X	thymosin, beta 4 [Source:RefSeq peptide;Acc:NP_001001315]	190.015	655.702	-1.78693	down	0.00057
ENSGALG00000019147	Uncharacterized protein [Source:UniProtKB/TrEMBL;Acc:E1C777]	0.19973	3.16319	-3.98524	down	0.00057
RGN	regucalcin [Source:RefSeq peptide;Acc:NP_990060]	86.5976	307.222	-1.82688	down	0.00057

LYG2	lysozyme g precursor [Source:RefSeq peptide;Acc:NP_001001470]	4.8035 5	64. 962	-3.7574 2	do wn	0.00 057
CHST10	Carbohydrate sulfotransferase 10 [Source:UniProtKB/Swiss-Prot;Acc:Q5ZIE4]	1.4 167 2	3.5 615 1	-1.3299 4	do wn	0.00 057
BAFF	tumor necrosis factor ligand superfamily member 13B [Source:RefSeq peptide;Acc:NP_989658]	0.2 790 8	1.5 613 3	-2.4840 2	do wn	0.00 057
IRG1	immunoresponsive 1 homolog [Source:RefSeq peptide;Acc:NP_001025992]	0.0 948 8	6.4 302 3	-6.0825 5	do wn	0.00 057
UCHL3	ubiquitin carboxyl-terminal hydrolase isozyme L3 [Source:RefSeq peptide;Acc:NP_990156]	41.	20.	1.00715	up	0.00

		2305	5134			057
LCP1	plastin-2 [Source:RefSeq peptide;Acc:NP_001008440]	19.1775	69.4855	-1.8573	down	0.00057
SUCLA2	succinate-CoA ligase, ADP-forming, beta subunit [Source:RefSeq peptide;Acc:NP_001006271]	3.43384	6.93278	-1.01361	down	0.00057
DHRS12	dehydrogenase/reductase (SDR family) member 12 [Source:HGNC Symbol;Acc:HGNC:25832]	0.88504	2.42147	-1.45207	down	0.00057
CKAP2	cytoskeleton-associated protein 2 [Source:RefSeq peptide;Acc:NP_001006274]	11.635	0.3405	5.09462	up	0.00057

		7	3			
RFC3	replication factor C subunit 3 [Source:RefSeq peptide;Acc:NP_001006276]	1.6745 1	0.5457 6	1.6174	up	0.00057
ENSGALG00000017075	NA	4.1263 9	1.0327 7	1.99836	up	0.00057
ALOX5AP	arachidonate 5-lipoxygenase-activating protein [Source:RefSeq peptide;Acc:NP_001265073]	0.0699 5	2.6146 3	-5.2242 1	down	0.00057
[2Fe-2S]	Cytochrome b-c1 complex subunit Rieske, mitochondrial [Source:UniProtKB/Swiss-Prot;Acc:Q5ZLR5]	36.977 8	16.220 5	1.18884	up	0.00057

BIRC2	Inhibitor of apoptosis protein [Source:UniProtKB/Swiss-Prot;Acc:Q90660]	17.5749	40.12	-1.19081	down	0.00057
CEP57	centrosomal protein 57kDa [Source:HGNC Symbol;Acc:HGNC:30794]	0.85063	1.97432	-1.21475	down	0.00057
FUT4	Gallus gallus fucosyltransferase 4 (alpha (1,3) fucosyltransferase, myeloid-specific) (FUT4), mRNA. [Source:RefSeq mRNA;Acc:NM_001031487]	0.09428	0.74523	-2.98261	down	0.00057
SMPD1	sphingomyelin phosphodiesterase 1 [Source:HGNC Symbol;Acc:HGNC:11120]	198.8	77.9372	1.35094	up	0.00057
RRM1	ribonucleoside-diphosphate reductase large subunit [Source:RefSeq	4.3	0.9	2.24985	up	0.00

	peptide;Acc:NP_001026008]	776	203<br>8			057
OAZ2	Gallus gallus ornithine decarboxylase antizyme 2 (OAZ2), mRNA. [Source:RefSeq mRNA;Acc:NM_001142862]	16.<br>357<br>2	38.<br>623<br>5	-1.2395<br>6	do<br>wn	0.00<br>057
PPIB	peptidyl-prolyl cis-trans isomerase B precursor [Source:RefSeq peptide;Acc:NP_990792]	686<br>.63<br>3	284<br>.71<br>3	1.27003	up	0.00<br>057
CYP1A4	cytochrome P450 1A4 [Source:RefSeq peptide;Acc:NP_990478]	41.<br>561<br>4	6.1<br>924<br>1	2.74667	up	0.00<br>057
PYGO1	pygopus homolog 1 [Source:RefSeq peptide;Acc:NP_001019862]	0.3<br>545	1.3<br>664	-1.9465<br>6	do<br>wn	0.00<br>057

		1	8			
ARPP19	cAMP-regulated phosphoprotein 19 [Source:UniProtKB/Swiss-Prot;Acc:Q5ZLY8]	27.0943	11.7122	1.20998	up	0.00057
C15orf48	chromosome 15 open reading frame 48 [Source:HGNC Symbol;Acc:HGNC:29898]	0.41959	9.38483	-4.48328	down	0.00057
GATM	glycine amidinotransferase, mitochondrial [Source:RefSeq peptide;Acc:NP_990076]	196.149	6.85167	4.83935	up	0.00057
TM6SF1	transmembrane 6 superfamily member 1 [Source:RefSeq peptide;Acc:NP_001264390]	3.29626	14.2645	-2.11353	down	0.00057

STARD5	StAR related lipid transfer domain containing 5 [Source:HGNC Symbol;Acc:HGNC:18065]	3.0 493 8	7.8 767 3	-1.3690 8	do wn	0.00 057
ARRDC4	arrestin domain containing 4 [Source:HGNC Symbol;Acc:HGNC:28087]	1.9 075	9.4 963 9	-2.3157	do wn	0.00 057
ADAMT S7	ADAM metallopeptidase with thrombospondin type 1 motif 7 [Source:HGNC Symbol;Acc:HGNC:223]	4.8 522 5	1.5 655 1	1.63202	up	0.00 057
VPS33B	vacuolar protein sorting-associated protein 33B [Source:RefSeq peptide;Acc:NP_001026519]	15. 447 4	5.4 159 7	1.51207	up	0.00 057
PKM	Pyruvate kinase PKM [Source:UniProtKB/Swiss-Prot;Acc:P00548]	7.6	20.	-1.4163	do	0.00

		367	382	2	wn	057
		7	9			
HEXA	beta-hexosaminidase subunit alpha precursor [Source:RefSeq peptide;Acc:NP_001025561]	4.2	12.	-1.5486	do	0.00
		382	398	4	wn	057
		3	6			
KLF13	Kruppel-like factor 13 [Source:HGNC Symbol;Acc:HGNC:13672]	4.9	1.4	1.7502	up	0.00
		881	827			057
		7	9			
ITGB1BP	integrin beta 1 binding protein 3 [Source:RefSeq peptide;Acc:NP_001025721]	210	45.	2.2136	up	0.00
3		.60	405			057
		5	6			
ENSGAL	Uncharacterized protein [Source:UniProtKB/TrEMBL;Acc:F1NB21]	2.1	4.9	-1.2225	do	0.00
G000000		425	997	1	wn	057

06036		8	7			
IL16	interleukin 16 [Source:RefSeq peptide;Acc:NP_001264925]	0.1<br>321<br>2	2.8<br>132<br>5	-4.4122<br>8	do<br>wn	0.00<br>057
FAH	fumarylacetoacetase [Source:RefSeq peptide;Acc:NP_001264402]	62.<br>749<br>1	24.<br>137<br>6	1.37831	up	0.00<br>057
TICRR	TOPBP1-interacting checkpoint and replication regulator [Source:HGNC Symbol;Acc:HGNC:28704]	2.1<br>588<br>9	0.5<br>087	2.08539	up	0.00<br>057
FANCI	Fanconi anemia, complementation group I [Source:RefSeq peptide;Acc:NP_001108323]	2.6<br>242<br>1	0.4<br>641<br>2	2.49932	up	0.00<br>057

CLN6	ceroid-lipofuscinosis, neuronal 6, late infantile, variant [Source:RefSeq peptide;Acc:NP_001264557]	13.<br>955<br>3	6.6<br>477<br>6	1.06987	up	0.00<br>057
IDH2	isocitrate dehydrogenase [Source:RefSeq peptide;Acc:NP_001026770]	343<br>.28<br>2	139<br>.39	1.30027	up	0.00<br>057
MAN2A2	mannosidase, alpha, class 2A, member 2 [Source:HGNC Symbol;Acc:HGNC:6825]	8.1<br>913<br>3	4.0<br>076<br>7	1.03133	up	0.00<br>057
HYPK	huntingtin interacting protein K [Source:RefSeq peptide;Acc:NP_001264583]	55.<br>686<br>5	20.<br>328<br>7	1.45381	up	0.00<br>057
ENSGAL	Uncharacterized protein [Source:UniProtKB/TrEMBL;Acc:F1NFS8]	60.	23.	1.38469	up	0.00

G00000001824		7361	2602			057
RGS9BP	Regulator of G-protein signaling 9-binding protein [Source:UniProtKB/Swiss-Prot;Acc:Q6XK22]	0.44817	1.57203	-1.81051	down	0.00057
RRAD	GTP-binding protein RAD [Source:RefSeq peptide;Acc:NP_001264535]	2.47059	0.65295	1.91981	up	0.00057
ENSGALG00000005720	Uncharacterized protein [Source:UniProtKB/TrEMBL;Acc:E1BS53]	1.85837	6.54241	-1.81578	down	0.00057
TUBB3	tubulin beta-4 chain [Source:RefSeq peptide;Acc:NP_001026769]	9.9991	1.5797	2.6621	up	0.00057

		7	6			
CDH3	cadherin 3, type 1, P-cadherin (placental) [Source:HGNC Symbol;Acc:HGNC:1762]	4.76354	1.53077	1.63777	up	0.00057
CDH1	cadherin-1 precursor [Source:RefSeq peptide;Acc:NP_001034347]	84.0946	28.7493	1.54849	up	0.00057
UTP4	UTP4 small subunit processome component [Source:HGNC Symbol;Acc:HGNC:1983]	9.51992	1.7371	2.45427	up	0.00057
TAT	tyrosine aminotransferase [Source:HGNC Symbol;Acc:HGNC:11573]	385.035	941.036	-1.28926	down	0.00057

ENSGAL<br>G000000<br>21512	Uncharacterized protein [Source:UniProtKB/TrEMBL;Acc:E1C8U0]	1.1<br>320<br>6	6.6<br>558<br>2	-2.5556<br>7	do<br>wn	0.00<br>057
MMP15	matrix metallopeptidase 15 [Source:HGNC Symbol;Acc:HGNC:7161]	73.<br>419<br>2	27.<br>800<br>8	1.40103	up	0.00<br>057
IL34	interleukin 34 [Source:HGNC Symbol;Acc:HGNC:28529]	1.8<br>049<br>7	4.9<br>856<br>8	-1.4658<br>2	do<br>wn	0.00<br>057
CFDP1	craniofacial development protein 1 [Source:RefSeq peptide;Acc:NP_001001189]	4.3<br>484<br>8	1.7<br>705<br>7	1.29629	up	0.00<br>057
MT4	Metallothionein [Source:UniProtKB/Swiss-Prot;Acc:P68497]	753	407	-2.4368	do	0.00

		.32<br>5	9.0<br>6	9	wn	057
MT1	metallothionein-3 [Source:RefSeq peptide;Acc:NP_001091007]	280<br>.08<br>3	262<br>0.0<br>3	-3.2256<br>6	do<br>wn	0.00<br>057
ENSGAL<br>G000000<br>21225	Uncharacterized protein [Source:UniProtKB/TrEMBL;Acc:E1BZF2]	1.8<br>438<br>4	10.<br>284<br>5	-2.4796<br>9	do<br>wn	0.00<br>057
SDR42E1	short chain dehydrogenase/reductase family 42E, member 1 [Source:HGNC Symbol;Acc:HGNC:29834]	3.2<br>715<br>2	8.5<br>971<br>8	-1.3939	do<br>wn	0.00<br>057
COX4I1	cytochrome c oxidase subunit 4 isoform 1, mitochondrial [Source:RefSeq peptide;Acc:NP_001025748]	500<br>.97	250<br>.33	1.00088	up	0.00<br>057

			1			
CYBA	cytochrome b-245, alpha polypeptide [Source:HGNC Symbol;Acc:HGNC:2577]	11.8729	26.8738	-1.17853	down	0.00057
CHTF8	CTF8, chromosome transmission fidelity factor 8 homolog [Source:RefSeq peptide;Acc:NP_001264567]	3.13526	0.53472	2.55174	up	0.00057
NQO1	NAD(P)H dehydrogenase [Source:RefSeq peptide;Acc:NP_001264548]	209.603	82.7839	1.34023	up	0.00057
RRP9	ribosomal RNA processing 9, small subunit (SSU) processome component, homolog (yeast) [Source:HGNC Symbol;Acc:HGNC:16829]	10.4735	2.01216	2.37993	up	0.00057

MANF	mesencephalic astrocyte-derived neurotrophic factor [Source:HGNC Symbol;Acc:HGNC:15461]	149.748	57.0352	1.39261	up	0.00057
PDE12	phosphodiesterase 12 [Source:HGNC Symbol;Acc:HGNC:25386]	4.83623	1.74028	1.47457	up	0.00057
IP6K2	inositol hexakisphosphate kinase 2 [Source:RefSeq peptide;Acc:NP_001025767]	21.0058	7.79819	1.42957	up	0.00057
UQCRC1	ubiquinol-cytochrome c reductase core protein I [Source:HGNC Symbol;Acc:HGNC:12585]	264.724	106.663	1.31142	up	0.00057
QARS	glutaminyl-tRNA synthetase [Source:RefSeq peptide;Acc:NP_001012800]	20.	8.1	1.35806	up	0.00

		872	424			057
		4	6			
C3orf14	chromosome 3 open reading frame 14 [Source:HGNC Symbol;Acc:HGNC:25024]	0.8	0.3	1.35249	up	0.00
		431	302			057
		8				
KBTBD8	kelch repeat and BTB (POZ) domain containing 8 [Source:HGNC Symbol;Acc:HGNC:30691]	2.4	6.3	-1.3584	do	0.00
		740	437	7	wn	057
		4	4			
ARL6IP5	PRA1 family protein 3 [Source:UniProtKB/Swiss-Prot;Acc:Q5F433]	6.4	13.	-1.0573	do	0.00
		166	353	1	wn	057
		2	3			
TNNC1	Troponin C, slow skeletal and cardiac muscles [Source:UniProtKB/Swiss-Prot;Acc:P09860]	7.4	30.	-2.0111	do	0.00
		780	144	5	wn	057

		5	3			
NT5DC2	5'-nucleotidase domain containing 2 [Source:HGNC Symbol;Acc:HGNC:25717]	10.	43.	-2.0874	do	0.00
		231	484	9	wn	057
		4	4			
CACNA2	calcium channel, voltage-dependent, alpha 2/delta subunit 2 [Source:HGNC	1.9	0.4	2.163	up	0.00
D2	Symbol;Acc:HGNC:1400]	051	254			057
		8	1			
UBA7	ubiquitin-like modifier activating enzyme 7 [Source:HGNC Symbol;Acc:HGNC:12471]	7.8	3.6	1.12713	up	0.00
		633	000			057
		4	3			
AMT	aminomethyltransferase, mitochondrial precursor [Source:RefSeq peptide;Acc:NP_990119]	197	48.	2.02944	up	0.00
		.44	363			057
		4	9			

ENSGALG00000027588	Aminoacylase-1 [Source:UniProtKB/TrEMBL;Acc:R4GL04]	74.0192	17.1119	2.1129	up	0.00057
OAS*A	59 kDa 2'-5'-oligoadenylate synthase-like protein [Source:RefSeq peptide;Acc:NP_990372]	0.61696	8.84421	-3.84148	down	0.00057
TKT	transketolase [Source:HGNC Symbol;Acc:HGNC:11834]	90.4829	23.3336	1.95523	up	0.00057
ARHGEF3	rho guanine nucleotide exchange factor 3 [Source:RefSeq peptide;Acc:NP_001025766]	4.01104	9.67506	-1.27029	down	0.00057
MCM2	DNA replication licensing factor MCM2 [Source:RefSeq peptide;Acc:NP_001006139]	13.	2.3	2.58279	up	0.00

		985<br>7	344<br>6			057
FGD5	FYVE, RhoGEF and PH domain containing 5 [Source:HGNC Symbol;Acc:HGNC:19117]	1.9<br>261<br>4	4.4<br>405<br>3	-1.2050<br>2	do<br>wn	0.00<br>057
PCDH1	Gallus gallus protocadherin 1 (PCDH1), mRNA. [Source:RefSeq mRNA;Acc:NM_001045827]	14.<br>568<br>9	4.7<br>037<br>4	1.63101	up	0.00<br>057
HSPA9	stress-70 protein, mitochondrial precursor [Source:RefSeq peptide;Acc:NP_001006147]	126<br>.34	62.<br>608<br>9	1.01288	up	0.00<br>057
SLP76	lymphocyte cytosolic protein 2 [Source:RefSeq peptide;Acc:NP_990032]	0.7<br>301	5.7<br>349	-2.9736<br>3	do<br>wn	0.00<br>057

			8			
SH3PXD2B	SH3 and PX domains 2B [Source:HGNC Symbol;Acc:HGNC:29242]	9.62944	4.03223	1.25587	up	0.00057
C13H5ORF41	Uncharacterized protein [Source:UniProtKB/TrEMBL;Acc:Q5ZMD5]	1.61539	5.95621	-1.88251	down	0.00057
ENSGALG00000003691	Uncharacterized protein [Source:UniProtKB/TrEMBL;Acc:E1C258]	5.6351	11.3427	-1.00925	down	0.00057
TIMD4	T-cell immunoglobulin and mucin domain-containing protein 4 precursor [Source:RefSeq peptide;Acc:NP_001006149]	10.8855	73.2284	-2.75	down	0.00057

ENSGALG00000004078	Uncharacterized protein [Source:UniProtKB/TrEMBL;Acc:F1NWA3]	5.07091	22.9621	-2.17894	down	0.00057
CSF1R	colony stimulating factor 1 receptor [Source:HGNC Symbol;Acc:HGNC:2433]	13.1579	51.5485	-1.97001	down	0.00057
ADAMTS2	ADAM metallopeptidase with thrombospondin type 1 motif 2 [Source:HGNC Symbol;Acc:HGNC:218]	7.0048	2.84023	1.30234	up	0.00057
C13H5ORF15	keratinocyte-associated transmembrane protein 2 precursor [Source:RefSeq peptide;Acc:NP_001006154]	26.1074	66.7549	-1.35442	down	0.00057
ENSGAL	Uncharacterized protein [Source:UniProtKB/TrEMBL;Acc:F1N828]	87.	27.	1.68812	up	0.00

G000000		509	156			057
07096		8	9			
KIF20A	kinesin-like protein KIF20A [Source:RefSeq peptide;Acc:NP_001012801]	9.6	2.2	2.12607	up	0.00
		790	172			057
		2	9			
B4GAL-	beta-1,4-galactosyltransferase 7 [Source:RefSeq peptide;Acc:NP_001035000]	20.	9.4	1.10632	up	0.00
T7		332	441			057
		9	7			
ENSGAL	Uncharacterized protein [Source:UniProtKB/TrEMBL;Acc:F1NUW0]	192	85.	1.16303	up	0.00
G000000		.01	747			057
03348		1	2			
LSM11	LSM11, U7 small nuclear RNA associated [Source:HGNC Symbol;Acc:HGNC:30860]	6.6	2.7	1.24112	up	0.00
		141	980			057

		7	9			
GM2A	GM2 ganglioside activator [Source:HGNC Symbol;Acc:HGNC:4367]	11.376	69.7758	-2.61674	down	0.00057
TGFBI	transforming growth factor-beta-induced protein ig-h3 precursor [Source:RefSeq peptide;Acc:NP_990367]	11.9311	28.604	-1.26149	down	0.00057
NUDT1	nudix (nucleoside diphosphate linked moiety X)-type motif 1 [Source:RefSeq peptide;Acc:NP_001006160]	74.8799	17.862	2.06768	up	0.00057
ARPC1B	actin-related protein 2/3 complex subunit 1B [Source:RefSeq peptide;Acc:NP_001025803]	9.02422	41.9247	-2.21593	down	0.00057

FAHD1	fumarylacetoacetate hydrolase domain containing 1 [Source:RefSeq peptide;Acc:NP_001264681]	28.<br>519<br>1	7.1<br>793<br>5	1.99	up	0.00<br>057
TBL3	transducin beta-like protein 3 [Source:RefSeq peptide;Acc:NP_001006166]	22.<br>236	10.<br>615<br>5	1.06672	up	0.00<br>057
PLK1	serine/threonine-protein kinase PLK1 [Source:RefSeq peptide;Acc:NP_001025810]	21.<br>216<br>1	0.8<br>690<br>1	4.60965	up	0.00<br>057
IL4RA	Uncharacterized protein [Source:UniProtKB/TrEMBL;Acc:F1P598]	3.5<br>685<br>4	9.6<br>478<br>5	-1.4348<br>7	do<br>wn	0.00<br>057
ABCC1	multidrug resistance-associated protein 1 [Source:RefSeq peptide;Acc:NP_001012540]	1.1	3.7	-1.7499	do	0.00

		119<br>9	400<br>8	2	wn	057
NDUFB6	NADH dehydrogenase [Source:RefSeq peptide;Acc:NP_001264454]	279<br>.34<br>8	123<br>.85<br>1	1.17345	up	0.00<br>057
FAM195<br>A	family with sequence similarity 195 member A [Source:HGNC Symbol;Acc:HGNC:14142]	135<br>.62<br>8	39.<br>753<br>9	1.77049	up	0.00<br>057
AIMP2	aminoacyl tRNA synthetase complex-interacting multifunctional protein 2 [Source:HGNC Symbol;Acc:HGNC:20609]	21.<br>450<br>3	7.8<br>157	1.45655	up	0.00<br>057
ZFAND2<br>A	zinc finger, AN1-type domain 2A [Source:HGNC Symbol;Acc:HGNC:28073]	4.3<br>051	11.<br>264	-1.3876	do<br>wn	0.00<br>057

		1				
FLII	protein flightless-1 homolog [Source:RefSeq peptide;Acc:NP_001025806]	11.1713	5.25161	1.08897	up	0.00057
SOX8	transcription factor SOX-8 [Source:RefSeq peptide;Acc:NP_990062]	1.25535	0.13081	3.26255	up	0.00057
NDUFAB1	NADH:ubiquinone oxidoreductase subunit AB1 [Source:HGNC Symbol;Acc:HGNC:7694]	78.9595	26.9604	1.55027	up	0.00057
ERN2	endoplasmic reticulum to nucleus signaling 2 [Source:HGNC Symbol;Acc:HGNC:16942]	1.43998	4.46537	-1.63273	down	0.00057

NUDT16L1	Protein syndesmos [Source:UniProtKB/Swiss-Prot;Acc:Q9IAY5]	89.6879	38.5194	1.21933	up	0.00057
TRAP1	heat shock protein 75 kDa, mitochondrial [Source:RefSeq peptide;Acc:NP_001006175]	53.2166	25.6011	1.05567	up	0.00057
PGAM5	PGAM family member 5, serine/threonine protein phosphatase, mitochondrial [Source:HGNC Symbol;Acc:HGNC:28763]	19.2333	9.20493	1.06313	up	0.00057
SLC15A4	solute carrier family 15 (oligopeptide transporter), member 4 [Source:HGNC Symbol;Acc:HGNC:23090]	5.06225	15.9941	-1.65969	down	0.00057
RILPL2	Rab interacting lysosomal protein-like 2 [Source:HGNC Symbol;Acc:HGNC:28787]	29.	12.	1.27877	up	0.00

		503<br>9	159<br>9			057
ENSGAL<br>G000000<br>04725	Uncharacterized protein [Source:UniProtKB/TrEMBL;Acc:E1BT93]	236<br>.67<br>5	589<br>.67<br>9	-1.3170<br>2	do<br>wn	0.00<br>057
KIAA167<br>1	KIAA1671 [Source:HGNC Symbol;Acc:HGNC:29345]	4.9<br>724<br>6	24.<br>194<br>9	-2.2826<br>7	do<br>wn	0.00<br>057
HSCB	HscB mitochondrial iron-sulfur cluster co-chaperone [Source:HGNC Symbol;Acc:HGNC:28913]	21.<br>595<br>9	10.<br>021	1.10773	up	0.00<br>057
MMP11	SWI/SNF-related matrix-associated actin-dependent regulator of chromatin subfamily B member 1 [Source:UniProtKB/Swiss-Prot;Acc:Q5ZK40]	18.<br>035	8.8<br>110	1.03343	up	0.00<br>057

		3	5			
DDT	D-dopachrome decarboxylase [Source:RefSeq peptide;Acc:NP_001025838]	293.535	134.223	1.1289	up	0.00057
PIK3IP1	phosphoinositide-3-kinase interacting protein 1 [Source:HGNC Symbol;Acc:HGNC:24942]	7.21174	23.9103	-1.72921	down	0.00057
PES1	pescadillo ribosomal biogenesis factor 1 [Source:HGNC Symbol;Acc:HGNC:8848]	15.2684	4.75033	1.68445	up	0.00057
SLC25A1	solute carrier family 25 (mitochondrial carrier; citrate transporter), member 1 [Source:HGNC Symbol;Acc:HGNC:10979]	289.693	140.537	1.04357	up	0.00057

10	Sarcoplasmic/endoplasmic reticulum calcium ATPase 2 [Source:UniProtKB/Swiss-Prot;Acc:Q03669]	18.521	7.3564 9	1.33207	up	0.00 057
XBP1	X-box binding protein 1 [Source:RefSeq peptide;Acc:NP_001006192]	85.42	263 .87 5	-1.6272 1	do wn	0.00 057
ENSGAL G000000 06728	NA	0.2 667 3	0.9 131 5	-1.7754 7	do wn	0.00 057
COX6A1	cytochrome c oxidase subunit VIa polypeptide 1 isoform 1 [Source:RefSeq peptide;Acc:NP_001264438]	878 .83 9	436 .68 5	1.009	up	0.00 057
RFC5	replication factor C subunit 5 [Source:HGNC Symbol;Acc:HGNC:9973]	28.	8.8	1.6796	up	0.00

		285	299			057
		9	8			
CD1.2	T-cell surface glycoprotein CD1b precursor [Source:RefSeq peptide;Acc:NP_001019753]	3.2	6.8	-1.0828	do	0.00
		427	689	7	wn	057
		4	2			
BLB1	Uncharacterized protein [Source:UniProtKB/TrEMBL;Acc:F1NSZ6]	17.	355	-4.3130	do	0.00
		890	.61	4	wn	057
		4	1			
BF1	MHC class I alpha chain 1 [Source:UniProtKB/TrEMBL;Acc:A0ZXL5]	11.	119	-3.3585	do	0.00
		697	.97	1	wn	057
		2	6			
ENSGAL	Uncharacterized protein [Source:UniProtKB/TrEMBL;Acc:E1BZZ5]	0.7	7.5	-3.4336	do	0.00
G000000		032	985	4	wn	057

04772		4	6			
ENTPD2	ectonucleoside triphosphate diphosphohydrolase 2 precursor [Source:RefSeq peptide;Acc:NP_990285]	1.6 707	7.9 272 1	-2.2463 6	do wn	0.00 057
AK1	Adenylate kinase isoenzyme 1 [Source:UniProtKB/Swiss-Prot;Acc:P05081]	21. 315	2.7 616 2	2.94829	up	0.00 057
ASS1	argininosuccinate synthase [Source:RefSeq peptide;Acc:NP_001013413]	9.7 794	49. 088 2	-2.3275 6	do wn	0.00 057
PLPP7	phospholipid phosphatase 7 (inactive) [Source:HGNC Symbol;Acc:HGNC:28174]	1.1 446 1	5.8 864 5	-2.3625 4	do wn	0.00 057

Ex-FABP	extracellular fatty acid-binding protein precursor [Source:RefSeq peptide;Acc:NP_990753]	4.7 202 7	64. 184 3	-3.7652 8	do wn	0.00 057
PTGDS	prostaglandin-H2 D-isomerase precursor [Source:RefSeq peptide;Acc:NP_989590]	237 .58 1	43. 777 7	2.44015	up	0.00 057
SLC31A1	solute carrier family 31 (copper transporter), member 1 [Source:HGNC Symbol;Acc:HGNC:11016]	5.4 646	28. 872 7	-2.4015 2	do wn	0.00 057
TUBB4B	tubulin beta-3 chain [Source:RefSeq peptide;Acc:NP_001074329]	206 .75	70. 044 4	1.56154	up	0.00 057
CERCA	cerebral endothelial cell adhesion molecule [Source:HGNC Symbol;Acc:HGNC:23723]	2.4	0.6	1.86748	up	0.00

M		745	781			057
			4			
FAM78A	family with sequence similarity 78 member A [Source:HGNC Symbol;Acc:HGNC:25465]	0.6	1.9	-1.6488	do	0.00
		232	543	5	wn	057
		3	5			
GBGT1	globoside alpha-1,3-N-acetylgalactosaminyltransferase 1 [Source:RefSeq	0.9	2.5	-1.3773	do	0.00
	peptide;Acc:NP_001025854]	803	469	8	wn	057
		6	4			
PMPCA	mitochondrial-processing peptidase subunit alpha [Source:RefSeq	45.	19.	1.24378	up	0.00
	peptide;Acc:NP_001006197]	047	022			057
		8				
NDUFA8	NADH dehydrogenase [Source:RefSeq peptide;Acc:NP_001264584]	90.	41.	1.12478	up	0.00
		528	514			057

		9				
FAM18B1	family with sequence similarity 18, member B1 [Source:RefSeq peptide;Acc:NP_001025870]	26.0049	58.5343	-1.1705	down	0.00057
RAB40B	ras-related protein Rab-40B [Source:RefSeq peptide;Acc:NP_001264979]	1.61973	4.20739	-1.37717	down	0.00057
ENSGALG00000001629	Uncharacterized protein [Source:UniProtKB/TrEMBL;Acc:E1BYA7]	1.09133	24.4671	-4.48668	down	0.00057
HLF	hepatic leukemia factor [Source:HGNC Symbol;Acc:HGNC:4977]	8.56247	2.85497	1.58455	up	0.00057

ATAD5	ATPase family AAA domain-containing protein 5 [Source:RefSeq peptide;Acc:NP_001257654]	2.3 332 1	0.8 736 4	1.41721	up	0.00 057
ADAP2	ArfGAP with dual PH domains 2 [Source:HGNC Symbol;Acc:HGNC:16487]	2.4 394 5	6.6 664 6	-1.4503 6	do wn	0.00 057
MAP2K6	mitogen-activated protein kinase kinase 6 [Source:HGNC Symbol;Acc:HGNC:6846]	4.5 186 9	1.1 712 3	1.94788	up	0.00 057
GPRC5C	G protein-coupled receptor, class C, group 5, member C [Source:HGNC Symbol;Acc:HGNC:13309]	8.5 093 7	1.7 281	2.29987	up	0.00 057
ENSGAL	Uncharacterized protein [Source:UniProtKB/TrEMBL;Acc:E1C1N0]	5.7	11.	-1.0192	do	0.00

G000000 06900		092 9	571 7	2	wn	057
TK1	Thymidine kinase, cytosolic [Source:UniProtKB/Swiss-Prot;Acc:P04047]	25. 417 8	1.7 285 8	3.87818	up	0.00 057
SLC9A3 R1	Na(+)/H(+) exchange regulatory cofactor NHE-RF1 [Source:RefSeq peptide;Acc:NP_001006424]	78. 297 5	34. 173 6	1.19608	up	0.00 057
COX10	protoheme IX farnesyltransferase, mitochondrial [Source:RefSeq peptide;Acc:NP_001025871]	13. 468	5.8 462 8	1.20394	up	0.00 057
METRN L	meteorin, glial cell differentiation regulator-like [Source:HGNC Symbol;Acc:HGNC:27584]	1.1 174	3.8 840	-1.7973 4	do wn	0.00 057

		5	5			
FN3K	fructosamine 3 kinase [Source:HGNC Symbol;Acc:HGNC:24822]	10.777	2.37524	2.18182	up	0.00057
NARF	nuclear prelamin A recognition factor [Source:HGNC Symbol;Acc:HGNC:29916]	3.35952	15.9729	-2.2493	down	0.00057
SCARNA16	Small Cajal body specific RNA 16 [Source:RFAM;Acc:RF00424]	2971.9	12834.2	-2.11054	down	0.00057
KPNA2	importin subunit alpha-2 [Source:RefSeq peptide;Acc:NP_001006209]	35.3553	1.40654	4.65171	up	0.00057

SLC16A6	solute carrier family 16 member 6 [Source:HGNC Symbol;Acc:HGNC:10927]	0.34421	4.53575	-3.71997	down	0.00057
ENSGALG00000007300	Pyrroline-5-carboxylate reductase [Source:UniProtKB/TrEMBL;Acc:F1N914]	10.837	4.6076	1.23389	up	0.00057
HID1	HID1 domain containing [Source:HGNC Symbol;Acc:HGNC:15736]	3.9932	19.0268	-2.25241	down	0.00057
ENSGALG00000000865	NA	0.78302	3.0289	-1.95168	down	0.00057
LIMK1	LIM domain kinase 1 [Source:UniProtKB/Swiss-Prot;Acc:Q8QFP8]	2.8	0.7	2.00082	up	0.00

		297<br>2	070<br>3			057
C1QBP	complement component 1, q subcomponent binding protein [Source:HGNC Symbol;Acc:HGNC:1243]	154<br>.71<br>4	66.<br>113<br>7	1.22658	up	0.00<br>057
RFFL	E3 ubiquitin-protein ligase rififylin [Source:RefSeq peptide;Acc:NP_001025882]	5.3<br>878<br>4	14.<br>569<br>3	-1.4351<br>5	do<br>wn	0.00<br>057
CCLI10	Chemokine [Source:UniProtKB/TrEMBL;Acc:E1C733]	15.<br>803<br>9	154<br>.32<br>3	-3.2876	do<br>wn	0.00<br>057
PIPOX	pipecolic acid oxidase [Source:HGNC Symbol;Acc:HGNC:17804]	11.<br>428	2.2<br>722	2.33041	up	0.00<br>057

		5	9			
ALDH3A	fatty aldehyde dehydrogenase [Source:RefSeq peptide;Acc:NP_001006223]	173	85.	1.02446	up	0.00
2		.79	438			057
		8	4			
WBSCR2	Williams Beuren syndrome chromosome region 27 [Source:HGNC Symbol;Acc:HGNC:19068]	60.	14.	2.05991	up	0.00
7		986	626			057
		6	4			
MDH2	malate dehydrogenase 2 [Source:HGNC Symbol;Acc:HGNC:6971]	205	54.	1.92322	up	0.00
		.33	140			057
		7	1			
PSPH	phosphoserine phosphatase [Source:RefSeq peptide;Acc:NP_001239201]	159	750	-2.2311	do	0.00
		8.6	5.6	4	wn	057
		4	8			

CHCHD2	coiled-coil-helix-coiled-coil-helix domain-containing protein 2, mitochondrial [Source:RefSeq peptide;Acc:NP_001006218]	101 7.0 2	463 .24 1	1.13451	up	0.00 057
PITPNA	phosphatidylinositol transfer protein alpha [Source:HGNC Symbol;Acc:HGNC:9001]	4.9 621 3	11. 146 4	-1.1675 5	do wn	0.00 057
PIGS	phosphatidylinositol glycan anchor biosynthesis class S [Source:HGNC Symbol;Acc:HGNC:14937]	15. 194 5	7.1 420 6	1.08913	up	0.00 057
ENSGAL G000000 08915	NA	1.0 673 1	2.5 869	-1.2772 5	do wn	0.00 057
SCAP	SREBF chaperone [Source:HGNC Symbol;Acc:HGNC:30634]	23.	6.6	1.82805	up	0.00

		510	216			057
		6	7			
ENSGAL	Uncharacterized protein [Source:UniProtKB/TrEMBL;Acc:F1NW48]	8.5	3.7	1.17571	up	0.00
G000000		61	896			057
06292			7			
CUBN	cubilin [Source:HGNC Symbol;Acc:HGNC:2548]	0.7	0.0	5.90982	up	0.00
		012	116			057
		9	6			
COL1A2	collagen alpha-2(I) chain precursor [Source:RefSeq peptide;Acc:NP_001073182]	52.	8.8	2.57527	up	0.00
		536	15			057
		1				
GLCCI1	glucocorticoid-induced transcript 1 protein [Source:RefSeq peptide;Acc:NP_001012873]	1.4	12.	-3.0958	do	0.00
		865	709	5	wn	057

		8	6			
SCIN	Adseverin [Source:UniProtKB/Swiss-Prot;Acc:Q5ZIV9]	0.57301	9.18478	-4.0026	down	0.00057
ARL4A	ADP ribosylation factor like GTPase 4A [Source:HGNC Symbol;Acc:HGNC:695]	13.1727	26.8311	-1.02635	down	0.00057
TAX1BP1	Tax1 (human T-cell leukemia virus type I) binding protein 1 [Source:RefSeq peptide;Acc:NP_990069]	21.6684	43.8164	-1.01588	down	0.00057
CMTM8	CKLF-like MARVEL transmembrane domain-containing protein 8 [Source:RefSeq peptide;Acc:NP_001186632]	3.74264	14.8438	-1.98773	down	0.00057

CMTM7	CKLF-like MARVEL transmembrane domain-containing protein 7 [Source:RefSeq peptide;Acc:NP_001007895]	2.0 719 2	8.0 878 1	-1.9647 8	do wn	0.00 057
CCR8	chemokine (C-C motif) receptor 8 [Source:RefSeq peptide;Acc:NP_001026162]	0.0 903 7	1.4 353 8	-3.9894 5	do wn	0.00 057
CX3CR1	chemokine (C-X3-C motif) receptor 1 [Source:HGNC Symbol;Acc:HGNC:2558]	2.5 869 8	6.8 621 1	-1.4073 8	do wn	0.00 057
IGFBP1	insulin-like growth factor-binding protein 1 precursor [Source:RefSeq peptide;Acc:NP_001001294]	20. 247 6	47. 498 5	-1.2301 3	do wn	0.00 057
TPPP	tubulin polymerization promoting protein [Source:HGNC Symbol;Acc:HGNC:24164]	1.0	11.	-3.4878	do	0.00

		166<br>7	405<br>5	1	wn	057
RBM24	RNA-binding protein 24 [Source:RefSeq peptide;Acc:NP_001012881]	0.2<br>745<br>7	1.0<br>448<br>3	-1.9280<br>4	do<br>wn	0.00<br>057
NEDD9	neural precursor cell expressed, developmentally down-regulated 9 [Source:HGNC Symbol;Acc:HGNC:7733]	1.7<br>177<br>4	18.<br>222<br>6	-3.4071<br>4	do<br>wn	0.00<br>057
SSR1	translocon-associated protein subunit alpha precursor [Source:RefSeq peptide;Acc:NP_001006375]	45.<br>939	13.<br>7	1.74555	up	0.00<br>057
IKZF1	DNA-binding protein Ikaros [Source:RefSeq peptide;Acc:NP_990419]	0.3<br>530<br>6	2.4<br>487<br>3	-2.7940<br>4	do<br>wn	0.00<br>057

GABBR2	gamma-aminobutyric acid (GABA) B receptor, 2 [Source:HGNC Symbol;Acc:HGNC:4507]	0.1<br>952<br>6	0.6<br>558<br>5	-1.7479<br>3	do<br>wn	0.00<br>057
FAM65B	protein FAM65B [Source:RefSeq peptide;Acc:NP_001012883]	0.3<br>525<br>4	1.1<br>655<br>8	-1.7252<br>1	do<br>wn	0.00<br>057
CYB5A	Cytochrome b5 [Source:UniProtKB/Swiss-Prot;Acc:P00174]	381<br>.85<br>4	175<br>.36<br>8	1.12263	up	0.00<br>057
PTPN2	tyrosine-protein phosphatase non-receptor type 2 [Source:RefSeq peptide;Acc:NP_001186316]	4.3<br>562<br>2	32.<br>786	-2.9119<br>3	do<br>wn	0.00<br>057
LYN	tyrosine-protein kinase Lyn [Source:RefSeq peptide;Acc:NP_001006390]	2.2	11.	-2.4048	do	0.00

		554	944	9	wn	057
		7	8			
PI15	Peptidase inhibitor 15 [Source:UniProtKB/Swiss-Prot;Acc:Q98ST6]	1.3	0.1	3.66175	up	0.00
		823	092			057
		8	3			
FABP5	fatty acid binding protein 5 (psoriasis-associated) [Source:RefSeq	3.0	11.	-1.9005	do	0.00
	peptide;Acc:NP_001006346]	876	527	3	wn	057
			6			
CHMP4C	chromatin modifying protein 4C [Source:RefSeq peptide;Acc:NP_001186404]	1.4	0.5	1.50356	up	0.00
		519	120			057
		6	8			
LAPTM4	lysosomal protein transmembrane 4 beta [Source:HGNC Symbol;Acc:HGNC:13646]	0.3	4.6	-3.6070	do	0.00
B		777	027	8	wn	057

		2	1			
SQLE	squalene monooxygenase [Source:RefSeq peptide;Acc:NP_001181856]	0.3	2.8	-3.1623	do	0.00
		175	424		wn	057
			3			
MYC	Myc proto-oncogene protein [Source:UniProtKB/Swiss-Prot;Acc:P01109]	5.5	0.8	2.7245	up	0.00
		110	338			057
		1	2			
ENSGAL	Uncharacterized protein [Source:UniProtKB/TrEMBL;Acc:E1BVI2]	0.7	2.3	-1.6943	do	0.00
G000000		189	266	2	wn	057
04904		4	5			
ENSGAL	Uncharacterized protein [Source:UniProtKB/TrEMBL;Acc:F1NUB0]	0.7	7.3	-3.3201	do	0.00
G000000		325	160	7	wn	057
05097			7			

HIGD1A	HIG1 domain family member 1A [Source:RefSeq peptide;Acc:NP_001026128]	87.9525	21.8474	2.00926	up	0.00057
CATHL1	Cathelicidin-1 [Source:UniProtKB/Swiss-Prot;Acc:Q6QLQ5]	0.90022	6.69105	-2.89389	down	0.00057
GARS	glycine--tRNA ligase [Source:RefSeq peptide;Acc:NP_001026681]	27.1884	12.5317	1.11741	up	0.00057
WDR86	WD repeat domain 86 [Source:HGNC Symbol;Acc:HGNC:28020]	0.78954	15.7599	-4.31911	down	0.00057
ENSGAL	Uncharacterized protein [Source:UniProtKB/TrEMBL;Acc:F1NZX3]	3.6	12.	-1.7693	do	0.00

G000000<br>06723		294<br>7	372<br>5	1	wn	057
PITRM1	pitrilysin metallopeptidase 1 [Source:HGNC Symbol;Acc:HGNC:17663]	6.4<br>103<br>9	2.8<br>205<br>2	1.18445	up	0.00<br>057
BAMBI	BMP and activin membrane-bound inhibitor homolog precursor [Source:RefSeq peptide;Acc:NP_001182330]	43.<br>534<br>4	16.<br>744<br>8	1.37844	up	0.00<br>057
PDSS1	prenyl (decaprenyl) diphosphate synthase, subunit 1 [Source:HGNC Symbol;Acc:HGNC:17759]	16.<br>602<br>2	2.7<br>047<br>8	2.61779	up	0.00<br>057
OTUD1	OTU deubiquitinase 1 [Source:HGNC Symbol;Acc:HGNC:27346]	8.7<br>581	25.<br>928	-1.5658<br>5	do<br>wn	0.00<br>057

		2	6			
PDK4	pyruvate dehydrogenase kinase, isozyme 4 [Source:RefSeq peptide;Acc:NP_001186838]	23.2573	164.824	-2.82517	down	0.00057
FAM126A	hyccin [Source:RefSeq peptide;Acc:NP_001026154]	3.08886	8.02457	-1.37735	down	0.00057
HIBADH	3-hydroxyisobutyrate dehydrogenase [Source:RefSeq peptide;Acc:NP_001006362]	69.7032	26.5405	1.39303	up	0.00057
CMTM6	CKLF-like MARVEL transmembrane domain containing 6 [Source:HGNC Symbol;Acc:HGNC:19177]	1.48026	6.22964	-2.0733	down	0.00057

ABHD5	1-acylglycerol-3-phosphate O-acyltransferase ABHD5 [Source:RefSeq peptide;Acc:NP_001265074]	3.7<br>395<br>4	28.<br>121<br>2	-2.9107<br>3	do<br>wn	0.00<br>057
LARS2	leucyl-tRNA synthetase 2 [Source:HGNC Symbol;Acc:HGNC:17095]	6.0<br>003<br>3	2.6<br>751<br>3	1.16543	up	0.00<br>057
CLEC3B	tetranectin precursor [Source:RefSeq peptide;Acc:NP_989997]	7.7<br>61	23.<br>143<br>3	-1.5762<br>8	do<br>wn	0.00<br>057
INHBA	inhibin beta A chain precursor [Source:RefSeq peptide;Acc:NP_990727]	8.6<br>992<br>6	0.4<br>189<br>8	4.37594	up	0.00<br>057
HSBP1L	heat shock factor binding protein 1-like 1 [Source:RefSeq peptide;Acc:NP_001129654]	3.4	14.	-2.0602	do	0.00

1		582<br>3	422<br>7	4	wn	057
KDM1B	lysine (K)-specific demethylase 1B [Source:HGNC Symbol;Acc:HGNC:21577]	1.0<br>066<br>3	2.5<br>624<br>4	-1.3479<br>8	do<br>wn	0.00<br>057
LPCAT1	lysophosphatidylcholine acyltransferase 1 [Source:HGNC Symbol;Acc:HGNC:25718]	2.4<br>051	6.1<br>687<br>5	-1.3588<br>8	do<br>wn	0.00<br>057
NDC80	Kinetochore protein NDC80 homolog [Source:UniProtKB/Swiss-Prot;Acc:Q76I89]	2.4<br>568<br>6	0.5<br>699<br>6	2.1079	up	0.00<br>057
TYMS	thymidylate synthetase [Source:HGNC Symbol;Acc:HGNC:12441]	8.8<br>996	1.1<br>027	3.01269	up	0.00<br>057

		4	2			
PCMTD1	Protein-L-isoaspartate O-methyltransferase domain-containing protein 1 [Source:UniProtKB/Swiss-Prot;Acc:Q5ZMR3]	8.1 569 2	18. 133 6	-1.1525 7	do wn	0.00 057
CYP7A1	cholesterol 7-alpha-monooxygenase [Source:RefSeq peptide;Acc:NP_001001753]	40. 050 6	4.2 240 3	3.24513	up	0.00 057
TOX	thymocyte selection-associated high mobility group box protein TOX [Source:RefSeq peptide;Acc:NP_001026196]	1.1 732 1	0.3 693	1.66761	up	0.00 057
TTPA	tocopherol (alpha) transfer protein [Source:HGNC Symbol;Acc:HGNC:12404]	18. 352 9	151 .59 1	-3.0461	do wn	0.00 057

CDH17	cadherin-17 precursor [Source:RefSeq peptide;Acc:NP_001186424]	0.5<br>585<br>1	1.6<br>971<br>6	-1.6034<br>8	do<br>wn	0.00<br>057
TP53INP<br>1	tumor protein p53-inducible nuclear protein 1 [Source:RefSeq peptide;Acc:NP_001026117]	0.3<br>247<br>8	3.5<br>089<br>7	-3.4335	do<br>wn	0.00<br>057
ANG-1	angiopoietin-1 precursor [Source:RefSeq peptide;Acc:NP_001186376]	0.3<br>668<br>7	1.6<br>237<br>5	-2.1459<br>7	do<br>wn	0.00<br>057
SYBU	syntabulin [Source:HGNC Symbol;Acc:HGNC:26011]	3.2<br>050<br>9	0.5<br>56	2.5272	up	0.00<br>057
RAD21	double-strand-break repair protein rad21 homolog [Source:RefSeq	28.	12.	1.21449	up	0.00

	peptide;Acc:NP_001026121]	093<br>5	106<br>2			057
PYCRL	pyrroline-5-carboxylate reductase-like [Source:HGNC Symbol;Acc:HGNC:25846]	5.5<br>310<br>5	2.2<br>512<br>2	1.29685	up	0.00<br>057
FAM83H	family with sequence similarity 83 member H [Source:HGNC Symbol;Acc:HGNC:24797]	2.5<br>498	1.1<br>592<br>4	1.13721	up	0.00<br>057
NRBP2	nuclear receptor binding protein 2 [Source:HGNC Symbol;Acc:HGNC:19339]	5.2<br>303<br>5	1.6<br>941<br>1	1.62638	up	0.00<br>057
IGSF1	immunoglobulin superfamily member 1 [Source:RefSeq peptide;Acc:NP_001138698]	0.9<br>708	4.3<br>428	-2.1613<br>5	do<br>wn	0.00<br>057

		3	4			
RPN2	dolichyl-diphosphooligosaccharide--protein glycosyltransferase subunit 2 precursor [Source:RefSeq peptide;Acc:NP_001006288]	109.335	43.1372	1.34175	up	0.00057
ADA	adenosine deaminase [Source:RefSeq peptide;Acc:NP_001006290]	2.21485	4.59247	-1.05206	down	0.00057
TPX2	targeting protein for Xklp2 [Source:RefSeq peptide;Acc:NP_989768]	3.0654	0.24224	3.66155	up	0.00057
MMP9	matrix metalloproteinase-9 precursor [Source:RefSeq peptide;Acc:NP_989998]	0.16167	2.77183	-4.09968	down	0.00057

SALL4	sal-like protein 4 [Source:RefSeq peptide;Acc:NP_001074341]	2.2<br>743<br>9	0.6<br>537<br>7	1.79862	up	0.00<br>057
MYBL2	myb-related protein B [Source:RefSeq peptide;Acc:NP_990649]	4.3<br>684<br>7	0.9<br>741<br>6	2.16489	up	0.00<br>057
HNF4alp<br>ha	hepatocyte nuclear factor 4-alpha [Source:RefSeq peptide;Acc:NP_001026026]	21.<br>820<br>7	5.2<br>437<br>9	2.05701	up	0.00<br>057
PPDPF	chromosome 20 open reading frame 149 [Source:RefSeq peptide;Acc:NP_001183966]	183<br>6.9<br>2	286<br>.92	2.67857	up	0.00<br>057
PCMTD2	protein-L-isoaspartate (D-aspartate) O-methyltransferase domain containing 2 [Source:HGNC	4.7	10.	-1.1041	do	0.00

	Symbol;Acc:HGNC:15882]	092	123	4	wn	057
		6	5			
ENSGAL	NA	33.	142	-2.1073	do	0.00
G000000		099	.62	7	wn	057
06756		9	9			
PEPCK	phosphoenolpyruvate carboxykinase, cytosolic [Source:RefSeq peptide;Acc:NP_990802]	14.	32.	-1.1721	do	0.00
		582	859	3	wn	057
		1	9			
RBM38	RNA binding motif protein 38 [Source:HGNC Symbol;Acc:HGNC:15818]	0.7	2.6	-1.8187	do	0.00
		444	262	5	wn	057
		4				
RCC2	regulator of chromosome condensation 2 [Source:HGNC Symbol;Acc:HGNC:30297]	26.	10.	1.26423	up	0.00
		037	84			057

		6				
ACOT7	acyl-CoA thioesterase 7 [Source:HGNC Symbol;Acc:HGNC:24157]	4.6<br>509<br>4	2.2<br>448<br>8	1.05088	up	0.00<br>057
hairy1	transcription factor HES-1 isoform 2 [Source:RefSeq peptide;Acc:NP_989803]	20.<br>420<br>8	6.1<br>688<br>9	1.72696	up	0.00<br>057
SPSB1	splA/ryanodine receptor domain and SOCS box containing 1 [Source:HGNC Symbol;Acc:HGNC:30628]	1.6<br>104<br>7	5.4<br>334<br>6	-1.7543<br>9	do<br>wn	0.00<br>057
APITD1	Centromere protein S [Source:UniProtKB/Swiss-Prot;Acc:E1BSW7]	6.2<br>234<br>7	0.7<br>628<br>7	3.02821	up	0.00<br>057

NBL1	Neuroblastoma suppressor of tumorigenicity 1 [Source:UniProtKB/Swiss-Prot;Acc:Q90YC9]	2.7<br>209<br>6	5.6<br>013<br>4	-1.0416<br>6	do<br>wn	0.00<br>057
WNT4	Protein Wnt-4 [Source:UniProtKB/Swiss-Prot;Acc:P49337]	0.4<br>126<br>1	2.9<br>583<br>5	-2.8419<br>4	do<br>wn	0.00<br>057
IGSF21	immunoglobin superfamily member 21 [Source:HGNC Symbol;Acc:HGNC:28246]	0.3<br>363<br>3	26.<br>699<br>1	-6.3107<br>7	do<br>wn	0.00<br>057
ARHGEF<br>10L	Rho guanine nucleotide exchange factor 10 like [Source:HGNC Symbol;Acc:HGNC:25540]	6.1<br>853<br>3	1.6<br>711	1.88805	up	0.00<br>057
ENSGAL	Uncharacterized protein [Source:UniProtKB/TrEMBL;Acc:E1BRY8]	7.9	1.6	2.29587	up	0.00

G00000001515		4533	1803			057
SAMD11	sterile alpha motif domain-containing protein 11 [Source:RefSeq peptide;Acc:NP_001026048]	0.56239	2.81678	-2.3244	down	0.00057
SRM	spermidine synthase [Source:HGNC Symbol;Acc:HGNC:11296]	12.6599	6.16051	1.03915	up	0.00057
ALDH4A1	delta-1-pyrroline-5-carboxylate dehydrogenase, mitochondrial [Source:RefSeq peptide;Acc:NP_001239033]	71.1568	23.9768	1.56936	up	0.00057
ENSGALG000000	Uncharacterized protein [Source:UniProtKB/TrEMBL;Acc:F1NE21]	1.3842	0.4867	1.50776	up	0.00057

03817		8	9			
C1QA	complement component 1, q subcomponent, A chain [Source:HGNC Symbol;Acc:HGNC:1241]	48.9601	267.935	-2.4522	down	0.00057
YBX1	Nuclease-sensitive element-binding protein 1 [Source:UniProtKB/Swiss-Prot;Acc:Q06066]	492.812	228.505	1.10881	up	0.00057
RASSF2	ras association domain-containing protein 2 [Source:RefSeq peptide;Acc:NP_001026055]	2.78954	15.3806	-2.46301	down	0.00057
tvb	tumor necrosis factor receptor superfamily member 10B precursor [Source:RefSeq peptide;Acc:NP_989446]	4.11222	1.09219	1.91269	up	0.00057

GFRA2	GDNF family receptor alpha-2 precursor [Source:RefSeq peptide;Acc:NP_990432]	41.<br>344<br>4	16.<br>887<br>5	1.29174	up	0.00<br>057
MTHFD2	bifunctional methylenetetrahydrofolate dehydrogenase/cyclohydrolase, mitochondrial [Source:RefSeq peptide;Acc:NP_001026531]	2.0<br>996<br>6	0.8<br>819<br>8	1.25133	up	0.00<br>057
NCAPH	non-SMC condensin I complex subunit H [Source:HGNC Symbol;Acc:HGNC:1112]	4.5<br>443<br>6	0.6<br>902<br>6	2.71887	up	0.00<br>057
SLC20A1	solute carrier family 20 (phosphate transporter), member 1 [Source:HGNC Symbol;Acc:HGNC:10946]	34.<br>834<br>9	5.0<br>592<br>6	2.78354	up	0.00<br>057
OGDH	2-oxoglutarate dehydrogenase, mitochondrial [Source:RefSeq peptide;Acc:NP_001026553]	20.	4.6	2.13032	up	0.00

		3275	4296			057
NUDCD3	NudC domain containing 3 [Source:HGNC Symbol;Acc:HGNC:22208]	13.8454	4.96593	1.47927	up	0.00057
ARHGAP25	rho GTPase-activating protein 25 [Source:RefSeq peptide;Acc:NP_001026054]	1.28156	4.28775	-1.74232	down	0.00057
ENSGALG00000000720	Uncharacterized protein [Source:UniProtKB/TrEMBL;Acc:F1N9J3]	7.5712	19.2907	-1.34931	down	0.00057
SNORA73	Small nucleolar RNA SNORA73 family [Source:RFAM;Acc:RF00045]	50012.	150944	-1.59365	down	0.00057

		5				
RCC1	regulator of chromosome condensation 1 [Source:HGNC Symbol;Acc:HGNC:1913]	3.05804	1.36456	1.16418	up	0.00057
ENSGALG00000028258	Uncharacterized protein [Source:UniProtKB/TrEMBL;Acc:R4GHK4]	0.31687	1.67944	-2.40603	down	0.00057
PTPRU	protein tyrosine phosphatase, receptor type, U [Source:HGNC Symbol;Acc:HGNC:9683]	5.91979	1.48454	1.99553	up	0.00057
IFI27L2	interferon alpha-inducible protein 27-like protein 2 [Source:RefSeq peptide;Acc:NP_001002856]	14.0268	181.797	-3.69607	down	0.00057

MARCKSL1	MARCKS-related protein [Source:RefSeq peptide;Acc:NP_001074187]	3.83327	8.967	-1.22605	down	0.00057
NDUFS5	NADH:ubiquinone oxidoreductase subunit S5 [Source:HGNC Symbol;Acc:HGNC:7712]	61.3289	26.4837	1.21146	up	0.00057
ZC3H12A	zinc finger CCCH-type containing 12A [Source:HGNC Symbol;Acc:HGNC:26259]	3.50337	13.3749	-1.93272	down	0.00057
HEYL	hes-related family bHLH transcription factor with YRPW motif-like [Source:HGNC Symbol;Acc:HGNC:4882]	2.02817	0.43559	2.21912	up	0.00057
HMGCL	hydroxymethylglutaryl-CoA lyase, mitochondrial isoform 1 [Source:RefSeq	98.	150	-3.9313	do	0.00

	peptide;Acc:NP_001185644]	587<br>1	4.1<br>3	9	wn	057
ENSGAL<br>G000000<br>24338	NA	44.<br>037<br>7	11.<br>511	1.93572	up	0.00<br>057
NTM	protein CEPU-1 precursor [Source:RefSeq peptide;Acc:NP_990042]	2.6<br>400<br>2	8.9<br>001<br>2	-1.7532<br>8	do<br>wn	0.00<br>057
ACAD8	isobutyryl-CoA dehydrogenase, mitochondrial [Source:RefSeq peptide;Acc:NP_001264990]	18.<br>487<br>2	6.3<br>478<br>7	1.54218	up	0.00<br>057
IL10R1	interleukin-10 receptor subunit alpha precursor [Source:RefSeq peptide;Acc:NP_001034686]	0.1<br>263	1.7<br>941	-3.8277<br>7	do<br>wn	0.00<br>057

		5	4			
STT3A	STT3A, subunit of the oligosaccharyltransferase complex (catalytic) [Source:HGNC Symbol;Acc:HGNC:6172]	2.2674	0.4306 1	2.3966	up	0.00057
CRTAM	cytotoxic and regulatory T-cell molecule precursor [Source:RefSeq peptide;Acc:NP_001025518]	0.0637 1	2.6910 5	-5.4004 4	down	0.00057
apoAIV	apolipoprotein A-IV precursor [Source:RefSeq peptide;Acc:NP_990269]	723 .41 2	254 3.7 4	-1.8140 6	down	0.00057
HYOU1	hypoxia up-regulated protein 1 precursor [Source:RefSeq peptide;Acc:NP_001006588]	28. 449 6	12. 835 2	1.1483	up	0.00057

ENSGALG00000022570	Uncharacterized protein [Source:UniProtKB/TrEMBL;Acc:H9L369]	82.4023	32.0593	1.36194	up	0.00057
ENSGALG00000024272	NA	2.70178	14.5246	-2.42652	down	0.00057
CHRNB2	neuronal acetylcholine receptor subunit beta-2 precursor [Source:RefSeq peptide;Acc:NP_990144]	3.08537	1.02132	1.59501	up	0.00057
CKS1B	cyclin-dependent kinases regulatory subunit 1 [Source:RefSeq peptide;Acc:NP_001106277]	105.951	9.28133	3.51293	up	0.00057
FLAD1	flavin adenine dinucleotide synthetase 1 [Source:HGNC Symbol;Acc:HGNC:24671]	9.9	3.8	1.3536	up	0.00

		232	830<br>8			057
FDPS	farnesyl diphosphate synthase [Source:HGNC Symbol;Acc:HGNC:3631]	18.<br>826<br>2	7.7<br>179<br>1	1.28646	up	0.00<br>057
MCL1	myeloid cell leukemia sequence 1 (BCL2-related) [Source:RefSeq peptide;Acc:NP_001244212]	4.3<br>039<br>2	37.<br>624<br>7	-3.1279<br>6	do<br>wn	0.00<br>057
TARS2	threonyl-tRNA synthetase 2, mitochondrial (putative) [Source:HGNC Symbol;Acc:HGNC:30740]	17.<br>086<br>7	7.8<br>436<br>1	1.12328	up	0.00<br>057
CTSS	cathepsin S precursor [Source:RefSeq peptide;Acc:NP_001026516]	59.<br>986	441<br>.92	-2.8810<br>9	do<br>wn	0.00<br>057

		5	6			
S100A10	Gallus gallus S100 calcium binding protein A10 (S100A10), mRNA. [Source:RefSeq mRNA;Acc:NM_205506]	6.5 528 1	14. 163 2	-1.1119 6	do wn	0.00 057
MICAL1	NEDD9-interacting protein with calponin homology and LIM domains [Source:RefSeq peptide;Acc:NP_001026747]	0.2 747 3	2.6 423 4	-3.2657 4	do wn	0.00 057
SARS	seryl-tRNA synthetase, cytoplasmic [Source:RefSeq peptide;Acc:NP_001026563]	38. 754 4	9.4 501 1	2.03596	up	0.00 057
RBM15	RNA binding motif protein 15 [Source:HGNC Symbol;Acc:HGNC:14959]	3.6 078 2	1.7 804 6	1.01887	up	0.00 057

SNRPE	Small nuclear ribonucleoprotein E [Source:UniProtKB/Swiss-Prot;Acc:P62303]	16.4944	6.20925	1.40949	up	0.00057
BTG2	BTG family member 2 [Source:HGNC Symbol;Acc:HGNC:1131]	33.9262	131.978	-1.95982	down	0.00057
PIM1	Pim-1 proto-oncogene, serine/threonine kinase [Source:HGNC Symbol;Acc:HGNC:8986]	2.29986	8.11144	-1.81841	down	0.00057
cKv1.1	potassium voltage-gated channel subfamily A member 3 [Source:RefSeq peptide;Acc:NP_001025549]	0.12886	4.96696	-5.26849	down	0.00057
ETNK2	ethanolamine kinase 2 [Source:HGNC Symbol;Acc:HGNC:25575]	29.	2.2	3.75354	up	0.00

		798	093			057
			3			
NUAK2	NUAK family, SNF1-like kinase, 2 [Source:HGNC Symbol;Acc:HGNC:29558]	2.4	0.5	2.17673	up	0.00
		505	42			057
		1				
ENSGAL	Uncharacterized protein [Source:UniProtKB/TrEMBL;Acc:E1C083]	6.4	1.0	2.56132	up	0.00
G000000		033	848			057
00791		9	7			
WDR77	methylosome protein 50 [Source:RefSeq peptide;Acc:NP_001026087]	7.5	3.2	1.18839	up	0.00
		133	967			057
		1	9			
SLC16A1	monocarboxylate transporter 1 [Source:RefSeq peptide;Acc:NP_001006323]	102	42.	1.27802	up	0.00
		.84	409			057

		6	6			
CHIA	acidic chitinase precursor [Source:RefSeq peptide;Acc:NP_989760]	4.80927	356.667	-6.21262	down	0.00057
PHB	Prohibitin [Source:UniProtKB/Swiss-Prot;Acc:P84173]	118.937	53.2833	1.15844	up	0.00057
PPP1R1B	protein phosphatase 1 regulatory inhibitor subunit 1B [Source:HGNC Symbol;Acc:HGNC:9287]	2.14924	0.36791	2.54639	up	0.00057
ERBB2	receptor tyrosine-protein kinase erbB-2 precursor [Source:RefSeq peptide;Acc:NP_001038126]	6.82238	2.50976	1.44273	up	0.00057

THRA	thyroid hormone receptor alpha [Source:RefSeq peptide;Acc:NP_990644]	17.5655	7.21215	1.28424	up	0.00057
IGFBP4	Gallus gallus insulin-like growth factor binding protein 4 (IGFBP4), mRNA. [Source:RefSeq mRNA;Acc:NM_204353]	13.6991	97.2878	-2.82818	down	0.00057
ENSGALG00000023818	NA	6.73021	40.4461	-2.58728	down	0.00057
RAMP2	receptor activity-modifying protein 2 precursor [Source:RefSeq peptide;Acc:NP_001099080]	15.3032	35.4138	-1.21048	down	0.00057
KIF18B	Kinesin-like protein KIF18B [Source:UniProtKB/Swiss-Prot;Acc:Q5ZLK6]	2.0	0.2	3.33243	up	0.00

		4049	0257			057
FZD2	frizzled-2 precursor [Source:RefSeq peptide;Acc:NP_989553]	13.1491	3.45787	1.927	up	0.00057
CDK5RAP3	CDK5 regulatory subunit-associated protein 3 [Source:RefSeq peptide;Acc:NP_001026757]	8.76062	3.85518	1.18424	up	0.00057
MED24	mediator of RNA polymerase II transcription subunit 24 [Source:RefSeq peptide;Acc:NP_001026534]	6.00084	2.45137	1.29157	up	0.00057
TOP2A	DNA topoisomerase 2-alpha [Source:RefSeq peptide;Acc:NP_990122]	16.875	1.0455	4.01251	up	0.00057

			8			
CCR7	C-C chemokine receptor type 7 precursor [Source:RefSeq peptide;Acc:NP_001185681]	0.12951	0.63835	-2.30124	down	0.00057
VAT1	vesicle amine transport 1 [Source:HGNC Symbol;Acc:HGNC:16919]	11.1564	28.3805	-1.34702	down	0.00057
GNA11	guanine nucleotide-binding protein subunit alpha-11 [Source:RefSeq peptide;Acc:NP_989565]	11.4453	5.27349	1.11792	up	0.00057
ACSBG2	long-chain-fatty-acid--CoA ligase ACSBG2 [Source:RefSeq peptide;Acc:NP_001012864]	6.31808	16.451	-1.38062	down	0.00057

CFD	complement factor D (adipsin) [Source:HGNC Symbol;Acc:HGNC:2771]	245.051	908.982	-1.89117	down	0.00057
WDR18	WD repeat-containing protein 18 [Source:RefSeq peptide;Acc:NP_001026107]	3.52959	1.7346	1.0249	up	0.00057
NDUFS7	NADH:ubiquinone oxidoreductase core subunit S7 [Source:HGNC Symbol;Acc:HGNC:7714]	288.003	128.753	1.16147	up	0.00057
TMEM161A	transmembrane protein 161A [Source:HGNC Symbol;Acc:HGNC:26020]	10.7689	3.9433	1.4494	up	0.00057
ELL	RNA polymerase II elongation factor ELL [Source:RefSeq peptide;Acc:NP_001012865]	3.9	8.3	-1.0852	do	0.00

		403	603<br>3	5	wn	057
LSM4	LSM4 homolog, U6 small nuclear RNA and mRNA degradation associated [Source:HGNC Symbol;Acc:HGNC:17259]	11.<br>732<br>1	4.3<br>869<br>5	1.41917	up	0.00<br>057
BTN1A1	butyrophilin subfamily 1 member A1 precursor [Source:RefSeq peptide;Acc:NP_001029989]	1.2<br>886	3.3<br>376<br>5	-1.3730<br>3	do<br>wn	0.00<br>057
ANGPTL<br>4	angiopoietin like 4 [Source:HGNC Symbol;Acc:HGNC:16039]	4.9<br>170<br>3	2.0<br>716<br>3	1.24702	up	0.00<br>057
MYO1F	myosin-If [Source:RefSeq peptide;Acc:NP_990585]	2.1<br>695	9.3<br>754	-2.1114<br>9	do<br>wn	0.00<br>057

		4	2			
ENSGAL<br>G000000<br>21972	Perilipin [Source:UniProtKB/TrEMBL;Acc:H9L1F4]	66.<br>649<br>1	13.<br>577	2.29542	up	0.00<br>057
CNN2	calponin-2 [Source:RefSeq peptide;Acc:NP_001135728]	5.2<br>063<br>7	12.<br>060<br>8	-1.2119<br>7	do<br>wn	0.00<br>057
UHRF1	ubiquitin-like with PHD and ring finger domains 1 [Source:HGNC Symbol;Acc:HGNC:12556]	1.0<br>550<br>8	0.2<br>210<br>7	2.2548	up	0.00<br>057
REL	proto-oncogene c-Rel [Source:RefSeq peptide;Acc:NP_001161198]	2.3<br>235<br>4	6.3<br>759<br>9	-1.4563<br>3	do<br>wn	0.00<br>057

BUB1	mitotic checkpoint serine/threonine-protein kinase BUB1 [Source:RefSeq peptide;Acc:NP_001012888]	4.9<br>138<br>5	0.6<br>365<br>5	2.94851	up	0.00<br>057
XDH	xanthine dehydrogenase/oxidase [Source:RefSeq peptide;Acc:NP_990458]	98.<br>944<br>5	268<br>.58<br>3	-1.4406<br>8	do<br>wn	0.00<br>057
UGP2	UTP--glucose-1-phosphate uridylyltransferase [Source:RefSeq peptide;Acc:NP_989442]	96.<br>637<br>6	713<br>.76<br>8	-2.8848	do<br>wn	0.00<br>057
JAG1	jagged 1 [Source:HGNC Symbol;Acc:HGNC:6188]	1.4<br>606<br>6	3.2<br>099<br>7	-1.1359<br>4	do<br>wn	0.00<br>057
HAO1	hydroxyacid oxidase 1 [Source:RefSeq peptide;Acc:NP_001186371]	34.	16.	1.02838	up	0.00

		5474	9372			057
CST3	Cystatin [Source:UniProtKB/Swiss-Prot;Acc:P01038]	38.7102	138.623	-1.84038	down	0.00057
GNMT	glycine N-methyltransferase [Source:HGNC Symbol;Acc:HGNC:4415]	376.535	113.821	1.72602	up	0.00057
DUSP10	dual specificity protein phosphatase 10 [Source:RefSeq peptide;Acc:NP_001026215]	1.05749	3.14103	-1.57059	down	0.00057
NEK2	serine/threonine-protein kinase Nek2 [Source:RefSeq peptide;Acc:NP_001026221]	5.3989	0.5005	3.43116	up	0.00057

		2	3			
KCNK5	potassium channel, two pore domain subfamily K, member 5 [Source:HGNC Symbol;Acc:HGNC:6280]	4.80548	14.7006	-1.61312	down	0.00057
NFKBIE	nuclear factor of kappa light polypeptide gene enhancer in B-cells inhibitor, epsilon [Source:HGNC Symbol;Acc:HGNC:7799]	0.46459	2.51075	-2.43407	down	0.00057
SIPA1L2	signal-induced proliferation-associated 1 like 2 [Source:HGNC Symbol;Acc:HGNC:23800]	1.74419	5.87056	-1.75094	down	0.00057
MPC1	Gallus gallus brain protein 44-like (BRP44L), mRNA. [Source:RefSeq mRNA;Acc:NM_001031524]	483.249	144.423	1.74246	up	0.00057

RAB32	ras-related protein Rab-32 [Source:RefSeq peptide;Acc:NP_001264601]	1.3<br>993<br>4	2.8<br>961<br>5	-1.0493<br>9	do<br>wn	0.00<br>057
IYD	iodotyrosine deiodinase [Source:HGNC Symbol;Acc:HGNC:21071]	16.<br>870<br>7	4.3<br>539<br>5	1.95413	up	0.00<br>057
SNX9	sorting nexin 9 [Source:HGNC Symbol;Acc:HGNC:14973]	9.8<br>246<br>8	25.<br>120<br>6	-1.3543<br>9	do<br>wn	0.00<br>057
IFNGR1	interferon gamma receptor 1 precursor [Source:RefSeq peptide;Acc:NP_001123859]	10.<br>460<br>8	26.<br>257<br>9	-1.3277<br>6	do<br>wn	0.00<br>057
ALDH8A	aldehyde dehydrogenase 8 family member A1 [Source:HGNC Symbol;Acc:HGNC:15471]	53.	20.	1.36079	up	0.00

1		686<br>2	903<br>7			057
RTN4IP1	reticulon 4 interacting protein 1 [Source:HGNC Symbol;Acc:HGNC:18647]	10.<br>460<br>8	3.0<br>140<br>4	1.79522	up	0.00<br>057
UBE2J1	ubiquitin-conjugating enzyme E2 J1 [Source:RefSeq peptide;Acc:NP_990094]	1.7<br>053<br>5	10.<br>085<br>8	-2.5641<br>8	do<br>wn	0.00<br>057
BORCS6	BLOC-1 related complex subunit 6 [Source:HGNC Symbol;Acc:HGNC:25939]	7.9<br>688<br>7	30.<br>438<br>7	-1.9334<br>6	do<br>wn	0.00<br>057
GSTA	Glutathione S-transferase [Source:UniProtKB/Swiss-Prot;Acc:Q08393]	61.<br>538	165<br>.65	-1.4286<br>5	do<br>wn	0.00<br>057

		1	8			
Sox11	transcription factor SOX-11 [Source:RefSeq peptide;Acc:NP_990518]	3.1	0.8	1.86914	up	0.00
		069	504			057
		7	9			
RSAD2	Uncharacterized protein [Source:UniProtKB/TrEMBL;Acc:F1NLJ6]	0.1	2.8	-4.1195	do	0.00
		613	041	9	wn	057
		2	8			
RRM2	ribonucleotide reductase M2 [Source:HGNC Symbol;Acc:HGNC:10452]	17.	1.1	3.86738	up	0.00
		199	784			057
		6	8			
Lpin1	Uncharacterized protein [Source:UniProtKB/TrEMBL;Acc:E1BWY2]	7.2	55.	-2.9341	do	0.00
		562	458	3	wn	057
		4	9			

cRhoB	rho-related GTP-binding protein RhoB [Source:RefSeq peptide;Acc:NP_990240]	12.<br>917	85.<br>976<br>3	-2.7346<br>7	do<br>wn	0.00<br>057
CAD	carbamoyl-phosphate synthetase 2, aspartate transcarbamylase, and dihydroorotase [Source:HGNC Symbol;Acc:HGNC:1424]	3.4<br>98	1.5<br>957<br>4	1.1323	up	0.00<br>057
EPT1	ethanolaminephosphotransferase 1 [Source:RefSeq peptide;Acc:NP_001026699]	6.4<br>521<br>6	30.<br>410<br>6	-2.2367<br>2	do<br>wn	0.00<br>057
ENSGAL<br>G000000<br>19861	Uncharacterized protein [Source:UniProtKB/TrEMBL;Acc:E1C9B4]	0.3<br>841<br>1	1.5<br>369<br>9	-2.0005	do<br>wn	0.00<br>057
DNAJC2	DnaJ homolog subfamily C member 27 [Source:UniProtKB/Swiss-Prot;Acc:Q6IMM1]	5.3	2.6	1.02297	up	0.00

7		218<br>4	189			057
FDFT1	squalene synthase [Source:RefSeq peptide;Acc:NP_001034383]	3.7<br>233<br>4	7.6<br>416	-1.0372<br>7	do<br>wn	0.00<br>057
GAL9	Gallinacin-9 [Source:UniProtKB/Swiss-Prot;Acc:Q6QLR1]	628<br>0.7	135<br>02.<br>8	-1.1042<br>6	do<br>wn	0.00<br>057
GAL7	Gallinacin-7 [Source:UniProtKB/Swiss-Prot;Acc:Q6QLR2]	0.2<br>118<br>9	2.1<br>569<br>5	-3.3475<br>8	do<br>wn	0.00<br>057
MCM3	DNA replication licensing factor MCM3 [Source:RefSeq peptide;Acc:NP_001006421]	8.0<br>707	0.6<br>077	3.73112	up	0.00<br>057

		8	6			
ABHD12	Monoacylglycerol lipase ABHD12 [Source:UniProtKB/Swiss-Prot;Acc:Q5ZIN0]	7.7893	20.3903	-1.38832	down	0.00057
YPEL5	protein yippee-like 5 [Source:RefSeq peptide;Acc:NP_001007901]	4.1671	9.57278	-1.19989	down	0.00057
WDPCP	WD repeat containing planar cell polarity effector [Source:HGNC Symbol;Acc:HGNC:28027]	0.1698	1.07135	-2.65757	down	0.00057
CNRIP1	CB1 cannabinoid receptor-interacting protein 1 [Source:RefSeq peptide;Acc:NP_001264459]	0.74306	3.68816	-2.31136	down	0.00057

PCSK2	proprotein convertase subtilisin/kexin type 2 [Source:HGNC Symbol;Acc:HGNC:8744]	0.4<br>890<br>1	1.4<br>516<br>8	-1.5697<br>9	do<br>wn	0.00<br>057
BIRC5	survivin isoform 1 [Source:RefSeq peptide;Acc:NP_001012318]	24.<br>644<br>9	4.1<br>052<br>5	2.58575	up	0.00<br>057
PEX6	peroxisomal biogenesis factor 6 [Source:HGNC Symbol;Acc:HGNC:8859]	31.<br>910<br>7	11.<br>232<br>2	1.5064	up	0.00<br>057
DEGS1	Sphingolipid delta(4)-desaturase DES1 [Source:UniProtKB/Swiss-Prot;Acc:Q5F3C1]	12.<br>111<br>1	30.<br>071<br>3	-1.3120<br>6	do<br>wn	0.00<br>057
FLVCR1	feline leukemia virus subgroup C cellular receptor 1 [Source:HGNC	1.0	2.9	-1.5418	do	0.00

	Symbol;Acc:HGNC:24682]	202<br>2	706<br>1	7	wn	057
HAAO	3-hydroxyanthranilate 3,4-dioxygenase [Source:HGNC Symbol;Acc:HGNC:4796]	76.<br>508<br>8	30.<br>144<br>7	1.34372	up	0.00<br>057
ENSGAL<br>G000000<br>11273	Sulfotransferase [Source:UniProtKB/TrEMBL;Acc:E1BRF0]	100<br>.43<br>7	258<br>.22<br>9	-1.3623<br>5	do<br>wn	0.00<br>057
ENSGAL<br>G000000<br>11287	sulfotransferase [Source:RefSeq peptide;Acc:NP_990391]	778<br>.11	76.<br>292<br>8	3.35036	up	0.00<br>057
ACAT2	acetyl-CoA acetyltransferase, cytosolic [Source:RefSeq peptide;Acc:NP_001034376]	73.<br>247	391<br>.03	-2.4164<br>4	do<br>wn	0.00<br>057

		1	2			
HECA	hdc homolog, cell cycle regulator [Source:HGNC Symbol;Acc:HGNC:21041]	7.7	16.	-1.0797	do	0.00
		316	341	4	wn	057
		3	9			
FABP7	Fatty acid-binding protein, brain [Source:UniProtKB/Swiss-Prot;Acc:Q05423]	15.	6.5	1.22364	up	0.00
		340	688			057
		5	5			
KPNA5	karyopherin alpha 5 (importin alpha 6) [Source:HGNC Symbol;Acc:HGNC:6398]	1.8	4.6	-1.3736	do	0.00
		022	700	5	wn	057
		5	9			
PNRC1	proline-rich nuclear receptor coactivator 1 [Source:RefSeq peptide;Acc:NP_001012291]	85.	173	-1.0239	do	0.00
		44	.73		wn	057
			5			

BCKDHB	2-oxoisovalerate dehydrogenase subunit beta, mitochondrial precursor [Source:RefSeq peptide;Acc:NP_989988]	22.1766	10.9108	1.02328	up	0.00057
TINAG	tubulointerstitial nephritis antigen precursor [Source:RefSeq peptide;Acc:NP_001264954]	38.6434	7.08741	2.44689	up	0.00057
FBXO9	F-box only protein 9 [Source:RefSeq peptide;Acc:NP_001006414]	9.13334	19.8521	-1.12007	down	0.00057
CMPK2	cytidine monophosphate (UMP-CMP) kinase 2, mitochondrial [Source:HGNC Symbol;Acc:HGNC:27015]	1.02844	7.04594	-2.77633	down	0.00057
ENSGAL	Uncharacterized protein [Source:UniProtKB/TrEMBL;Acc:F1NKA4]	5.1	1.9	1.42848	up	0.00

G00000016441		6974	2067			057
ENSGALG00000016471	Uncharacterized protein [Source:UniProtKB/TrEMBL;Acc:F1NV37]	1.2049	4.11088	-1.77053	down	0.00057
KRTCAP3	keratinocyte associated protein 3 [Source:HGNC Symbol;Acc:HGNC:28943]	12.4152	3.32411	1.90106	up	0.00057
DNMT3A	DNA (cytosine-5)-methyltransferase 3A [Source:RefSeq peptide;Acc:NP_001020003]	11.9104	3.287	1.85737	up	0.00057
HADHA	trifunctional enzyme subunit alpha, mitochondrial [Source:RefSeq peptide;Acc:NP_990387]	124.12	60.993	1.02506	up	0.00057

		4	6			
GAL8	Gallinacin-8 [Source:UniProtKB/Swiss-Prot;Acc:Q6IV23]	91.	228	-1.3269	do	0.00
		033	.38	8	wn	057
			1			
GAL1	Gallinacin-1 [Source:UniProtKB/Swiss-Prot;Acc:P46156]	0.4	7.6	-4.0760	do	0.00
		529	397	7	wn	057
		6	7			
CENPQ	centromere protein Q [Source:RefSeq peptide;Acc:NP_001038114]	6.2	1.6	1.93765	up	0.00
		527	322			057
		4	2			
RCAN2	regulator of calcineurin 2 [Source:HGNC Symbol;Acc:HGNC:3041]	2.9	1.1	1.39816	up	0.00
		163	065			057
		4				

KIF4A	chromosome-associated kinesin KIF4 [Source:RefSeq peptide;Acc:NP_990306]	11.9687	0.86937	3.78315	up	0.00057
ENSGALG00000020331	Uncharacterized protein [Source:UniProtKB/TrEMBL;Acc:F1NP76]	0.11394	1.81318	-3.99216	down	0.00057
ENSGALG00000004113	Uncharacterized protein [Source:UniProtKB/TrEMBL;Acc:E1BWL9]	0.55325	2.35908	-2.09221	down	0.00057
GPR174	probable G-protein coupled receptor 174 [Source:RefSeq peptide;Acc:NP_001008464]	0.17112	2.13205	-3.63915	down	0.00057
AIFM1	apoptosis-inducing factor 1, mitochondrial [Source:RefSeq peptide;Acc:NP_001007491]	60.	26.	1.20251	up	0.00

		948<br>4	483<br>3			057
TSC22D3	TSC22 domain family protein 3 [Source:RefSeq peptide;Acc:NP_001070702]	48.<br>611<br>6	152<br>.84<br>8	-1.6527<br>2	do<br>wn	0.00<br>057
TIMM8A	translocase of inner mitochondrial membrane 8 homolog A (yeast) [Source:HGNC Symbol;Acc:HGNC:11817]	35.<br>967<br>3	10.<br>83	1.73166	up	0.00<br>057
IL2RG	interleukin 2 receptor subunit gamma [Source:HGNC Symbol;Acc:HGNC:6010]	8.4<br>132<br>5	43.<br>911<br>9	-2.3838<br>8	do<br>wn	0.00<br>057
SLC6A14	solute carrier family 6 (amino acid transporter), member 14 [Source:HGNC Symbol;Acc:HGNC:11047]	0.6<br>854	5.0<br>355	-2.8770<br>2	do<br>wn	0.00<br>057

		6	6			
ENSGALG00000005964	Uncharacterized protein [Source:UniProtKB/TrEMBL;Acc:F1NBN4]	0.47946	3.00696	-2.64883	down	0.00057
CLDN2	claudin-2 [Source:RefSeq peptide;Acc:NP_001264551]	28.198	2.96216	3.25087	up	0.00057
CHRDL1	chordin-like protein 1 precursor [Source:RefSeq peptide;Acc:NP_989502]	0.16368	1.44218	-3.13928	down	0.00057
MSMO1	methylsterol monooxygenase 1 [Source:RefSeq peptide;Acc:NP_001006438]	11.3777	43.7173	-1.94199	down	0.00057

IL15	interleukin 15 [Source:RefSeq peptide;Acc:NP_989902]	3.04489	11.8034	-1.95474	down	0.00057
GRID2	glutamate receptor, ionotropic, delta 2 [Source:HGNC Symbol;Acc:HGNC:4576]	0.06033	1.03104	-4.0951	down	0.00057
ETNPPL	ethanolamine-phosphate phospho-lyase [Source:HGNC Symbol;Acc:HGNC:14404]	181.771	42.0708	2.11123	up	0.00057
EREG	proepiregulin precursor [Source:RefSeq peptide;Acc:NP_001001203]	0.28542	3.16518	-3.47113	down	0.00057
ENSGAL	Uncharacterized protein [Source:UniProtKB/TrEMBL;Acc:E1BS37]	169	390	-1.2062	do	0.00

G00000010979		.311	.669	7	wn	057
CDS1	CDP-diacylglycerol synthase 1 [Source:HGNC Symbol;Acc:HGNC:1800]	0.72396	1.58929	-1.1344	down	0.00057
dCK	deoxycytidine kinase [Source:RefSeq peptide;Acc:NP_001006451]	1.04689	0.23459	2.15788	up	0.00057
CCNA2	cyclin A2 [Source:HGNC Symbol;Acc:HGNC:1578]	9.02226	0.82183	3.45659	up	0.00057
MAD2L1	mitotic spindle assembly checkpoint protein MAD2A [Source:RefSeq peptide;Acc:NP_001264678]	9.1975	3.5318	1.38083	up	0.00057

		8	5			
CAMK2D	calcium/calmodulin-dependent protein kinase type II delta chain [Source:RefSeq peptide;Acc:NP_001034389]	2.16847	5.37956	-1.31081	down	0.00057
ELOVL6	Elongation of very long chain fatty acids protein 6 [Source:UniProtKB/Swiss-Prot;Acc:Q5ZJR8]	4.27352	38.9897	-3.1896	down	0.00057
PDLIM5	PDZ and LIM domain protein 5 [Source:RefSeq peptide;Acc:NP_001026320]	3.2695	6.61891	-1.01752	down	0.00057
DAPP1	dual adaptor of phosphotyrosine and 3-phosphoinositides [Source:RefSeq peptide;Acc:NP_001012951]	1.12166	4.05788	-1.85509	down	0.00057

TEC	tyrosine-protein kinase Tec [Source:RefSeq peptide;Acc:NP_001025543]	0.4<br>932<br>3	1.8<br>216<br>7	-1.8849<br>2	do<br>wn	0.00<br>057
SEL1L3	sel-1 suppressor of lin-12-like 3 (C. elegans) [Source:HGNC Symbol;Acc:HGNC:29108]	11.<br>420<br>5	25.<br>408<br>3	-1.1536<br>7	do<br>wn	0.00<br>057
WDR1	WD repeat-containing protein 1 [Source:RefSeq peptide;Acc:NP_001004402]	26.<br>809<br>7	60.<br>910<br>2	-1.1839<br>3	do<br>wn	0.00<br>057
SLBP	histone RNA hairpin-binding protein [Source:RefSeq peptide;Acc:NP_001004403]	2.0<br>383<br>1	0.7<br>704<br>1	1.40368	up	0.00<br>057
SUCLG1	succinyl-CoA ligase [Source:RefSeq peptide;Acc:NP_001012910]	39.	18.	1.08925	up	0.00

		556 9	592			057
SFXN5	sideroflexin 5 [Source:HGNC Symbol;Acc:HGNC:16073]	5.2 204 6	0.6 835	2.93317	up	0.00 057
EDA2R	tumor necrosis factor receptor superfamily member 27 [Source:RefSeq peptide;Acc:NP_001076829]	1.2 511 9	0.6 166	1.0209	up	0.00 057
GPC3	glypican 3 [Source:HGNC Symbol;Acc:HGNC:4451]	0.4 069 4	2.2 263 9	-2.4518 1	do wn	0.00 057
MOSPD1	motile sperm domain containing 1 [Source:HGNC Symbol;Acc:HGNC:25235]	28. 836	80. 395	-1.4792	do wn	0.00 057

		9	4			
ENSGALG00000007546	Uncharacterized protein [Source:UniProtKB/TrEMBL;Acc:E1BR53]	0.07741	1.7367	-4.48761	down	0.00057
COL4A5	collagen, type IV, alpha 5 [Source:HGNC Symbol;Acc:HGNC:2207]	1.28847	0.27192	2.24442	up	0.00057
SLC25A5	solute carrier family 25 (mitochondrial carrier; adenine nucleotide translocator), member 5 [Source:HGNC Symbol;Acc:HGNC:10991]	100.28	34.3552	1.54543	up	0.00057
TMEM192	transmembrane protein 192 [Source:HGNC Symbol;Acc:HGNC:26775]	3.47762	1.6355	1.08837	up	0.00057

NR3C2	Mineralocorticoid receptor [Source:UniProtKB/Swiss-Prot;Acc:Q8QHI2]	1.4<br>844<br>1	7.8<br>930<br>3	-2.4106<br>9	do<br>wn	0.00<br>057
CCNG2	cyclin G2 [Source:HGNC Symbol;Acc:HGNC:1593]	4.7<br>898<br>6	13.<br>428<br>6	-1.4872<br>6	do<br>wn	0.00<br>057
SLC25A4	ADP/ATP translocase 1 [Source:RefSeq peptide;Acc:NP_001006443]	187<br>.26<br>3	34.<br>304	2.44861	up	0.00<br>057
HMGB2	high mobility group protein B2 [Source:RefSeq peptide;Acc:NP_990817]	9.3<br>100<br>3	2.4<br>519<br>7	1.92484	up	0.00<br>057
ENSGAL	Uncharacterized protein [Source:UniProtKB/TrEMBL;Acc:F1P0D3]	1.7	35.	-4.3627	do	0.00

G000000<br>11190		091<br>5	165<br>2	9	wn	057
HOPX	Homeodomain-only protein [Source:UniProtKB/Swiss-Prot;Acc:Q8JHU0]	1.1<br>324<br>6	5.4<br>653<br>6	-2.2708<br>6	do<br>wn	0.00<br>057
CL2	Ribonuclease CL2 [Source:UniProtKB/Swiss-Prot;Acc:P81476]	295<br>1.3<br>2	135<br>3.6<br>5	1.12451	up	0.00<br>057
NAAA	N-acylethanolamine acid amidase [Source:HGNC Symbol;Acc:HGNC:736]	1.6<br>718<br>9	5.1<br>954<br>6	-1.6357<br>7	do<br>wn	0.00<br>057
IGJ	immunoglobulin J polypeptide, linker protein for immunoglobulin alpha and mu polypeptides precursor [Source:RefSeq peptide;Acc:NP_989594]	0.0<br>143	9.1<br>229	-9.3139<br>2	do<br>wn	0.00<br>057

		3	8			
SYNPO2	synaptopodin 2 [Source:HGNC Symbol;Acc:HGNC:17732]	1.0<br>102<br>7	2.1<br>174<br>5	-1.0675<br>8	do<br>wn	0.00<br>057
ENPEP	glutamyl aminopeptidase (aminopeptidase A) [Source:HGNC Symbol;Acc:HGNC:3355]	1.0<br>317<br>2	41.<br>545<br>8	-5.3315<br>8	do<br>wn	0.00<br>057
ENSGAL<br>G000000<br>12250	Uncharacterized protein [Source:UniProtKB/TrEMBL;Acc:F1NI80]	211<br>.97<br>6	678<br>.29<br>6	-1.6780<br>1	do<br>wn	0.00<br>057
H2AFZ	Histone H2A.Z [Source:UniProtKB/Swiss-Prot;Acc:Q5ZMD6]	56.<br>013<br>5	27.<br>484<br>2	1.02717	up	0.00<br>057

UCH-L1	ubiquitin carboxyl-terminal hydrolase isozyme L1 [Source:RefSeq peptide;Acc:NP_001073681]	0.6 123 2	6.6 850 8	-3.4485 8	do wn	0.00 057
CD38	ADP-ribosyl cyclase 1 [Source:RefSeq peptide;Acc:NP_001188317]	0.9 004 1	8.8 653 5	-3.2995 3	do wn	0.00 057
PPP2R2C	protein phosphatase 2 regulatory subunit B, gamma [Source:HGNC Symbol;Acc:HGNC:9306]	1.0 103 1	0.0 23	5.45674	up	0.00 057
TACC3	transforming, acidic coiled-coil containing protein 3 [Source:RefSeq peptide;Acc:NP_001004429]	11. 884 4	4.8 593 7	1.29022	up	0.00 057
POLR1A	polymerase (RNA) I polypeptide A [Source:HGNC Symbol;Acc:HGNC:17264]	7.8	3.8	1.03412	up	0.00

		858	507			057
		1	4			
RNF103	RING finger protein 103 [Source:RefSeq peptide;Acc:NP_001026340]	5.1	10.	-1.0223	do	0.00
		183	396	4	wn	057
		4	4			
LFABP	fatty acid-binding protein, liver [Source:RefSeq peptide;Acc:NP_989523]	211	5.0	8.70082	up	0.00
		9.2	930			057
		7	5			
NDUFV1	NADH dehydrogenase [Source:RefSeq peptide;Acc:NP_001026754]	119	22.	2.39231	up	0.00
		.64	789			057
		3	3			
ENSGAL	Uncharacterized protein [Source:UniProtKB/TrEMBL;Acc:R4GJX3]	94.	343	-1.8662	do	0.00
G000000		147	.23	1	wn	057

26970		1	6			
RCN1	reticulocalbin 1 [Source:HGNC Symbol;Acc:HGNC:9934]	20.<br>591<br>7	6.4<br>497<br>9	1.67474	up	0.00<br>057
LYVE1	lymphatic vessel endothelial hyaluronic acid receptor 1 [Source:RefSeq peptide;Acc:NP_001186516]	7.3<br>874<br>1	17.<br>108<br>4	-1.2115<br>7	do<br>wn	0.00<br>057
TMEM41<br>B	Transmembrane protein 41B [Source:UniProtKB/Swiss-Prot;Acc:Q5ZIL6]	6.5<br>338<br>4	16.<br>651<br>6	-1.3496<br>6	do<br>wn	0.00<br>057
FTD	ferritin, light polypeptide [Source:RefSeq peptide;Acc:NP_989714]	2.6<br>618<br>1	1.3<br>058<br>9	1.02737	up	0.00<br>057

LDHA	L-lactate dehydrogenase A chain [Source:RefSeq peptide;Acc:NP_990615]	56.<br>153	289<br>.45<br>4	-2.3659	do<br>wn	0.00<br>057
FADS2	fatty acid desaturase 2 [Source:RefSeq peptide;Acc:NP_001153900]	5.0<br>735	24.<br>654<br>3	-2.2807<br>9	do<br>wn	0.00<br>057
MPEG1	macrophage expressed 1 [Source:HGNC Symbol;Acc:HGNC:29619]	7.4<br>030<br>3	58.<br>264<br>1	-2.9764<br>2	do<br>wn	0.00<br>057
CD44	CD44 antigen precursor [Source:RefSeq peptide;Acc:NP_990191]	1.5<br>289<br>6	7.7<br>764<br>9	-2.3465<br>7	do<br>wn	0.00<br>057
CD82	CD82 antigen [Source:RefSeq peptide;Acc:NP_001008470]	13.	36.	-1.4025	do	0.00

		841	591	3	wn	057
		4	8			
CHAC1	cation transport regulator-like protein 1 [Source:RefSeq peptide;Acc:NP_001186585]	4.1	104	-4.6407	do	0.00
		766	.19	1	wn	057
		9				
CHP1	Calcineurin B homologous protein 1 [Source:UniProtKB/Swiss-Prot;Acc:Q5ZM44]	81.	40.	1.01721	up	0.00
		610	321			057
		8	4			
ERH	enhancer of rudimentary homolog [Source:RefSeq peptide;Acc:NP_001006475]	72.	30.	1.23921	up	0.00
		820	847			057
		3				
ENSGAL	Acetyl-coenzyme A synthetase [Source:UniProtKB/TrEMBL;Acc:E1BZT9]	73.	12.	2.49922	up	0.00
G000000		288	962			057

10225		4	7			
FOS	FBJ murine osteosarcoma viral oncogene homolog [Source:HGNC Symbol;Acc:HGNC:3796]	2.3<br>759<br>6	6.5<br>771<br>8	-1.4689<br>6	do<br>wn	0.00<br>057
SLC24A4	solute carrier family 24 (sodium/potassium/calcium exchanger), member 4 [Source:HGNC Symbol;Acc:HGNC:10978]	0.6<br>006<br>2	2.5<br>197<br>1	-2.0687<br>4	do<br>wn	0.00<br>057
RIN3	Ras and Rab interactor 3 [Source:HGNC Symbol;Acc:HGNC:18751]	0.8<br>742<br>9	3.1<br>336<br>6	-1.8416<br>6	do<br>wn	0.00<br>057
HHIPL1	HHIP-like 1 [Source:HGNC Symbol;Acc:HGNC:19710]	2.2<br>495<br>5	4.7<br>645<br>8	-1.0827<br>1	do<br>wn	0.00<br>057

EVL	ena/VASP-like protein [Source:RefSeq peptide;Acc:NP_001006487]	3.5437	9.07306	-1.35633	down	0.00057
AMN	protein amnionless precursor [Source:RefSeq peptide;Acc:NP_001264445]	29.0844	4.76455	2.60983	up	0.00057
WDHD1	WD repeat and HMG-box DNA binding protein 1 [Source:HGNC Symbol;Acc:HGNC:23170]	2.48915	0.52707	2.23959	up	0.00057
GNG2	guanine nucleotide-binding protein G(I)/G(S)/G(O) subunit gamma-2 [Source:RefSeq peptide;Acc:NP_001090997]	0.56422	1.59059	-1.49524	down	0.00057
FEN1	Flap endonuclease 1 [Source:UniProtKB/Swiss-Prot;Acc:Q5ZLN4]	11.	1.5	2.9187	up	0.00

		681	448			057
		7	5			
B4GALN	beta-1,4-N-acetyl-galactosaminyl transferase 4 [Source:HGNC Symbol;Acc:HGNC:26315]	0.9	0.1	2.33678	up	0.00
T4		182	817			057
		3	7			
CYB5R2	NADH-cytochrome b5 reductase 2 [Source:RefSeq peptide;Acc:NP_001258911]	73.	33.	1.11991	up	0.00
		787	951			057
		4	3			
ADM	adrenomedullin [Source:HGNC Symbol;Acc:HGNC:259]	2.2	6.4	-1.5375	do	0.00
		231	538	4	wn	057
		5	1			
ABCC8	ATP binding cassette subfamily C member 8 [Source:HGNC Symbol;Acc:HGNC:59]	2.8	1.0	1.44566	up	0.00
		615	505			057

		5	4			
SLC22A1<br>8	solute carrier family 22 member 18 [Source:HGNC Symbol;Acc:HGNC:10964]	1.5<br>397<br>4	10.<br>457<br>2	-2.7637<br>3	do<br>wn	0.00<br>057
TSPAN4	tetraspanin 4 [Source:HGNC Symbol;Acc:HGNC:11859]	6.9<br>404	18.<br>146<br>2	-1.3865<br>8	do<br>wn	0.00<br>057
CD151	CD151 antigen [Source:RefSeq peptide;Acc:NP_001006472]	12.<br>228<br>2	33.<br>187<br>9	-1.4404<br>4	do<br>wn	0.00<br>057
FADS1	Fatty acid desaturase 1 [Source:UniProtKB/TrEMBL;Acc:E7EDS8]	2.1<br>610<br>2	17.<br>982<br>3	-3.0567<br>9	do<br>wn	0.00<br>057

ABTB2	ankyrin repeat and BTB (POZ) domain containing 2 [Source:HGNC Symbol;Acc:HGNC:23842]	0.3 222 7	1.5 055 7	-2.2239 6	do wn	0.00 057
NDUFS3	NADH dehydrogenase [Source:RefSeq peptide;Acc:NP_001139804]	98. 231	34. 887 8	1.49345	up	0.00 057
THBS1	thrombospondin-1 precursor [Source:RefSeq peptide;Acc:NP_001186382]	3.4 424 5	25. 748	-2.9029 5	do wn	0.00 057
PRKD1	serine/threonine-protein kinase D1 [Source:RefSeq peptide;Acc:NP_001026372]	1.4 154 1	4.1 345 3	-1.5465 1	do wn	0.00 057
CFL2	Cofilin-2 [Source:UniProtKB/Swiss-Prot;Acc:P21566]	7.7	2.9	1.41291	up	0.00

		4247	0772			057
NFKBIA	nuclear factor of kappa light polypeptide gene enhancer in B-cells inhibitor, alpha [Source:HGNC Symbol;Acc:HGNC:7797]	3.50919	43.5814	-3.6345	down	0.00057
FBXO33	F-box protein 33 [Source:HGNC Symbol;Acc:HGNC:19833]	4.28814	8.91355	-1.05565	down	0.00057
FBLN5	fibulin-5 precursor [Source:RefSeq peptide;Acc:NP_001264403]	2.95128	7.95979	-1.43139	down	0.00057
DDX24	DEAD (Asp-Glu-Ala-Asp) box helicase 24 [Source:HGNC Symbol;Acc:HGNC:13266]	3.0468	1.2316	1.3067	up	0.00057

		8	9			
ENSGALG00000020390	Uncharacterized protein [Source:UniProtKB/TrEMBL;Acc:E1C206]	43.2477	157.991	-1.86915	down	0.00057
HSP90AA1	heat shock protein 90kDa alpha family class A member 1 [Source:HGNC Symbol;Acc:HGNC:5253]	116.778	358.327	-1.6175	down	0.00057
BAG5	BAG family molecular chaperone regulator 5 [Source:RefSeq peptide;Acc:NP_001026382]	4.78473	10.9747	-1.19767	down	0.00057
hsp70	heat shock 70 kDa protein [Source:RefSeq peptide;Acc:NP_001006686]	11.2966	204.732	-4.17977	down	0.00057

Metazoa_<br>SRP	Metazoan signal recognition particle RNA [Source:RFAM;Acc:RF00017]	162<br>.74<br>8	55.<br>770<br>4	1.54507	up	0.00<br>057
PYGL	glycogen phosphorylase, liver form [Source:RefSeq peptide;Acc:NP_989723]	37.<br>892<br>4	16.<br>89	1.16574	up	0.00<br>057
ENSGAL<br>G000000<br>02493	Uncharacterized protein [Source:UniProtKB/TrEMBL;Acc:F1P580]	42.<br>971<br>1	132<br>0.0<br>9	-4.9411<br>3	do<br>wn	0.00<br>057
DNAJC1<br>2	dnaJ homolog subfamily C member 12 [Source:RefSeq peptide;Acc:NP_001186459]	2.6<br>561<br>2	24.<br>858	-3.2263<br>2	do<br>wn	0.00<br>057
DDX50	DEAD (Asp-Glu-Ala-Asp) box polypeptide 50 [Source:HGNC Symbol;Acc:HGNC:17906]	10.	4.1	1.39728	up	0.00

		931<br>7	501<br>7			057
P4HA1	prolyl 4-hydroxylase subunit alpha-1 isoform 3 precursor [Source:RefSeq peptide;Acc:NP_001185666]	3.6<br>666<br>9	10.<br>502<br>2	-1.5181<br>4	do<br>wn	0.00<br>057
COMTD<br>1	catechol-O-methyltransferase domain containing 1 [Source:HGNC Symbol;Acc:HGNC:26309]	10.<br>795	27.<br>871	-1.3684	do<br>wn	0.00<br>057
PDLIM1	PDZ and LIM domain 1 [Source:HGNC Symbol;Acc:HGNC:2067]	12.<br>054<br>8	24.<br>631	-1.0308<br>7	do<br>wn	0.00<br>057
ENSGAL<br>G000000<br>23925	Uncharacterized protein [Source:UniProtKB/TrEMBL;Acc:F1P5U6]	12.<br>009<br>5	1.3<br>888	3.11226	up	0.00<br>057

ENSGALG00000005469	cytochrome P-450 2C45 [Source:RefSeq peptide;Acc:NP_001001752]	13.7398	3.63774	1.91725	up	0.00057
ENSGALG00000006384	Uncharacterized protein [Source:UniProtKB/TrEMBL;Acc:F1P3J2]	0.44005	15.9869	-5.1831	down	0.00057
RBP4	Retinol-binding protein 4 [Source:UniProtKB/Swiss-Prot;Acc:P41263]	563.509	1294.9	-1.20034	down	0.00057
CPN1	carboxypeptidase N catalytic chain precursor [Source:RefSeq peptide;Acc:NP_001264183]	154.102	50.3073	1.61504	up	0.00057
GOT1	aspartate aminotransferase, cytoplasmic [Source:RefSeq peptide;Acc:NP_990652]	129	53.	1.26941	up	0.00

		.92	896			057
		5	8			
ARHGA	rho GTPase-activating protein 19 [Source:RefSeq peptide;Acc:NP_001012927]	2.1	0.3	2.52555	up	0.00
P19		704	769			057
		8	6			
NPM3	nucleophosmin/nucleoplasmin 3 [Source:HGNC Symbol;Acc:HGNC:7931]	4.8	1.7	1.4893	up	0.00
		813	386			057
		5	7			
SFXN2	sideroflexin 2 [Source:HGNC Symbol;Acc:HGNC:16086]	22.	6.7	1.73037	up	0.00
		441	632			057
		3	4			
GSTO1	glutathione S-transferase omega-1 [Source:RefSeq peptide;Acc:NP_001264304]	28.	81.	-1.4983	do	0.00
		722	145	4	wn	057

		3	5			
BAG3	BCL2 associated athanogene 3 [Source:HGNC Symbol;Acc:HGNC:939]	1.63876	6.47366	-1.98198	down	0.00057
INPP5F	inositol polyphosphate-5-phosphatase F [Source:HGNC Symbol;Acc:HGNC:17054]	2.48852	5.56439	-1.16094	down	0.00057
HTRA1	HtrA serine peptidase 1 [Source:HGNC Symbol;Acc:HGNC:9476]	9.18387	52.4154	-2.51282	down	0.00057
ACADSB	short/branched chain specific acyl-CoA dehydrogenase, mitochondrial [Source:RefSeq peptide;Acc:NP_001026411]	10.6977	30.6692	-1.51949	down	0.00057

ENSGAL<br>G000000<br>02020	Glutamate dehydrogenase [Source:UniProtKB/TrEMBL;Acc:F1P3F9]	572<br>.27<br>6	230<br>.39<br>1	1.31262	up	0.00<br>057
ANXA11	annexin A11 [Source:RefSeq peptide;Acc:NP_001012921]	5.5<br>845<br>7	20.<br>073<br>9	-1.8458	do<br>wn	0.00<br>057
ANG	Angiogenin [Source:UniProtKB/Swiss-Prot;Acc:P27043]	2.1<br>047<br>9	9.4<br>322<br>7	-2.1639<br>3	do<br>wn	0.00<br>057
RSFR	Ribonuclease homolog [Source:UniProtKB/Swiss-Prot;Acc:P30374]	24.<br>387	194<br>.27<br>5	-2.9939<br>2	do<br>wn	0.00<br>057
ENSGAL	Uncharacterized protein [Source:UniProtKB/TrEMBL;Acc:F1NPK5]	7.6	1.7	2.11337	up	0.00

G00000004511		1094	5894			057
C10orf54	chromosome 10 open reading frame 54 [Source:HGNC Symbol;Acc:HGNC:30085]	2.88979	9.29208	-1.68504	down	0.00057
PLAU	urokinase-type plasminogen activator preproprotein [Source:RefSeq peptide;Acc:NP_990774]	2.87665	27.2423	-3.24338	down	0.00057
ENSGALG00000025721	NA	12.1937	93.8822	-2.94472	down	0.00057
CYP2H1	cytochrome P450 2H1 precursor [Source:RefSeq peptide;Acc:NP_001001616]	108.39	321.02	-1.56638	down	0.00057

		5	5			
LIPA	lipase A, lysosomal acid, cholesterol esterase [Source:HGNC Symbol;Acc:HGNC:6617]	11.<br>469<br>6	78.<br>978<br>4	-2.7836<br>5	do<br>wn	0.00<br>057
PANK1	pantothenate kinase 1 [Source:HGNC Symbol;Acc:HGNC:8598]	52.<br>114<br>6	236<br>.58<br>3	-2.1825<br>9	do<br>wn	0.00<br>057
KIF11	kinesin-like protein KIF11 [Source:RefSeq peptide;Acc:NP_001026401]	5.9<br>046<br>5	0.6<br>502<br>7	3.18274	up	0.00<br>057
PPRC1	peroxisome proliferator-activated receptor gamma, coactivator-related 1 [Source:HGNC Symbol;Acc:HGNC:30025]	2.7<br>293<br>4	0.8<br>262<br>6	1.72389	up	0.00<br>057

PEO1	twinkle protein, mitochondrial precursor [Source:RefSeq peptide;Acc:NP_001026515]	24.3823	7.52057	1.69692	up	0.00057
CYP17A1	steroid 17-alpha-hydroxylase/17,20 lyase precursor [Source:RefSeq peptide;Acc:NP_001001901]	0.52925	1.93568	-1.87081	down	0.00057
CPXM2	carboxypeptidase X (M14 family), member 2 [Source:HGNC Symbol;Acc:HGNC:26977]	0.28429	1.65223	-2.539	down	0.00057
BNIP3	BCL2/adenovirus E1B 19kDa interacting protein 3 [Source:HGNC Symbol;Acc:HGNC:1084]	20.4989	106.527	-2.3776	down	0.00057
FRZB	secreted frizzled-related protein 3 precursor [Source:RefSeq peptide;Acc:NP_990103]	12.	26.	-1.0815	do	0.00

		572	608	9	wn	057
		6	3			
RAB17	RAB17, member RAS oncogene family [Source:HGNC Symbol;Acc:HGNC:16523]	0.4	3.4	-2.9287	do	0.00
		491	204	6	wn	057
		9	1			
SPP2	secreted phosphoprotein 24 [Source:RefSeq peptide;Acc:NP_990499]	248	521	2.25469	up	0.00
		6.6	.06			057
		9	5			
COL18A	collagen, type XVIII, alpha 1 precursor [Source:RefSeq peptide;Acc:NP_989495]	10.	0.6	3.91852	up	0.00
1		110	686			057
		7	4			
CASP18	caspase 18 [Source:RefSeq peptide;Acc:NP_001038154]	1.4	4.7	-1.7025	do	0.00
		457	056	7	wn	057

			6	6			
NDUFS1	NADH-ubiquinone oxidoreductase 75 kDa subunit, mitochondrial peptide;Acc:NP_001006518]	[Source:RefSeq	145.778	69.0886	1.07725	up	0.00057
ATP5G3	ATP synthase lipid-binding protein, mitochondrial isoform 1 peptide;Acc:NP_001264784]	[Source:RefSeq	347.401	147.365	1.2372	up	0.00057
WIPF1	WAS/WASL-interacting protein family member 1 peptide;Acc:NP_001012935]	[Source:RefSeq	1.86411	5.73473	-1.62123	down	0.00057
OLA1	Obg-like ATPase 1 [Source:UniProtKB/Swiss-Prot;Acc:Q5ZM25]		19.1938	9.38639	1.032	up	0.00057

DPP4	dipeptidyl peptidase 4 [Source:RefSeq peptide;Acc:NP_001026426]	1.4 755 6	4.4 683 7	-1.5984 9	do wn	0.00 057
CYP27A 1	cytochrome P450 family 27 subfamily A member 1 [Source:HGNC Symbol;Acc:HGNC:2605]	220 .29 5	107 .56 6	1.03422	up	0.00 057
GPR39	G-protein coupled receptor 39 [Source:RefSeq peptide;Acc:NP_001073574]	3.7 968 7	1.4 808 2	1.35842	up	0.00 057
UPP2	uridine phosphorylase 2 [Source:HGNC Symbol;Acc:HGNC:23061]	54. 396 2	16. 463 4	1.72424	up	0.00 057
FAM171	family with sequence similarity 171 member B [Source:HGNC Symbol;Acc:HGNC:29412]	0.5	0.0	2.63669	up	0.00

B		989	963			057
		6	1			
BARD1	BRCA1-associated RING domain protein 1 [Source:RefSeq peptide;Acc:NP_001026417]	1.6	0.2	2.63405	up	0.00
		28	622			057
			6			
NDUFA1	NADH dehydrogenase [Source:RefSeq peptide;Acc:NP_001026418]	51.	20.	1.35335	up	0.00
0		424	126			057
		6	7			
ITGB2	integrin beta-2 precursor [Source:RefSeq peptide;Acc:NP_990582]	3.5	24.	-2.7967	do	0.00
		204	462	7	wn	057
		3	8			
STK17B	serine/threonine-protein kinase 17B [Source:RefSeq peptide;Acc:NP_001026420]	0.7	3.4	-2.1501	do	0.00
		813	681	7	wn	057

		2	3			
ANKRD44	Serine/threonine-protein phosphatase 6 regulatory ankyrin repeat subunit B [Source:UniProtKB/Swiss-Prot;Acc:Q5F478]	1.50893	5.79543	-1.94139	down	0.00057
CD28	T-cell-specific surface glycoprotein CD28 homolog [Source:UniProtKB/Swiss-Prot;Acc:P31043]	1.52823	3.92813	-1.36197	down	0.00057
CDCA7	cell division cycle associated 7 [Source:HGNC Symbol;Acc:HGNC:14628]	6.93108	1.27805	2.43914	up	0.00057
CYBRD1	cytochrome b reductase 1 [Source:HGNC Symbol;Acc:HGNC:20797]	0.58285	1.59688	-1.45407	down	0.00057

ANKZF1	ankyrin repeat and zinc finger domain containing 1 [Source:HGNC Symbol;Acc:HGNC:25527]	3.54072	1.42906	1.30898	up	0.00057
SLC11A1	Natural resistance-associated macrophage protein 1 [Source:UniProtKB/Swiss-Prot;Acc:P51027]	3.52831	10.6207	-1.58982	down	0.00057
TFCP2L1	transcription factor CP2-like 1 [Source:HGNC Symbol;Acc:HGNC:17925]	2.47272	0.89416	1.46749	up	0.00057
ENSGALG00000012072	Uncharacterized protein [Source:UniProtKB/TrEMBL;Acc:E1BQC5]	6.9947	24.4401	-1.80492	down	0.00057
DTX3L	deltex 3 like, E3 ubiquitin ligase [Source:HGNC Symbol;Acc:HGNC:30323]	4.2	12.	-1.5928	do	0.00

		417	795	8	wn	057
		1	1			
CXCR4	C-X-C chemokine receptor type 4 [Source:RefSeq peptide;Acc:NP_989948]	0.5	8.4	-3.9803	do	0.00
		327	089	5	wn	057
		7	1			
ORC4	origin recognition complex subunit 4 [Source:HGNC Symbol;Acc:HGNC:8490]	6.0	1.3	2.14232	up	0.00
		613	729			057
			8			
KCNT2	potassium channel, sodium activated subfamily T, member 2 [Source:HGNC	0.2	3.7	-3.8168	do	0.00
	Symbol;Acc:HGNC:18866]	639	197	6	wn	057
		5	2			
VCAM1	vascular cell adhesion molecule 1 [Source:HGNC Symbol;Acc:HGNC:12663]	3.8	21.	-2.4446	do	0.00
		960	210	9	wn	057

GTF2B	general transcription factor IIB [Source:HGNC Symbol;Acc:HGNC:4648]	3<br>9.5<br>416<br>5	3<br>19.<br>355<br>2	-1.0204<br>1	do<br>wn	0.00<br>057
CDC20	cell division cycle protein 20 homolog [Source:RefSeq peptide;Acc:NP_001006536]	22.<br>723<br>3	1.4<br>834	3.93719	up	0.00<br>057
PTPRF	protein tyrosine phosphatase, receptor type, F [Source:HGNC Symbol;Acc:HGNC:9670]	13.<br>070<br>3	3.2<br>887<br>3	1.99069	up	0.00<br>057
ENSGAL<br>G000000<br>20700	Uncharacterized protein [Source:UniProtKB/TrEMBL;Acc:F1NE99]	2.8<br>329<br>2	0.8<br>290<br>8	1.77271	up	0.00<br>057

TMEM69	transmembrane protein 69 [Source:HGNC Symbol;Acc:HGNC:28035]	10.849 6	4.4810 6	1.27573	up	0.00057
TSPAN1	tetraspanin-1 [Source:RefSeq peptide;Acc:NP_001264519]	3.9114 5	1.3042 3	1.5845	up	0.00057
UQCRH	cytochrome b-c1 complex subunit 6, mitochondrial [Source:RefSeq peptide;Acc:NP_001264602]	924.79	429.89 6	1.10514	up	0.00057
CMPK1	UMP-CMP kinase [Source:UniProtKB/Swiss-Prot;Acc:Q5ZKE7]	22.141 5	10.699 1	1.04927	up	0.00057
HOOK1	protein Hook homolog 1 [Source:RefSeq peptide;Acc:NP_001006542]	10.	3.7	1.47541	up	0.00

		397	394			057
		9	1			
AK4	adenylate kinase 4 [Source:HGNC Symbol;Acc:HGNC:363]	39.	16.	1.24877	up	0.00
		941	807			057
		6	7			
TNNI3K	serine/threonine-protein kinase TNNI3K [Source:RefSeq peptide;Acc:NP_001191745]	0.6	0.2	1.62158	up	0.00
		442	093			057
		6	7			
ENSGAL	NA	81.	906	-3.4816	do	0.00
G000000		155	.58	9	wn	057
02431		1	4			
RGS2	regulator of G-protein signaling 2 [Source:RefSeq peptide;Acc:NP_989726]	3.3	41.	-3.6354	do	0.00
		019	033	1	wn	057

		7	8			
RGS1	regulator of G-protein signaling 1 [Source:HGNC Symbol;Acc:HGNC:9991]	0.1<br>463	5.3<br>033<br>8	-5.1799<br>1	do<br>wn	0.00<br>057
RGS18	regulator of G-protein signaling 18 [Source:RefSeq peptide;Acc:NP_001244256]	1.6<br>111<br>3	9.4<br>4	-2.5507<br>1	do<br>wn	0.00<br>057
RGS5	regulator of G-protein signaling 5 [Source:HGNC Symbol;Acc:HGNC:10001]	12.<br>591<br>5	88.<br>846	-2.8188<br>5	do<br>wn	0.00<br>057
HSD17B<br>7	3-keto-steroid reductase isoform 1 [Source:RefSeq peptide;Acc:NP_001264435]	0.7<br>291<br>7	5.0<br>699<br>8	-2.7976<br>4	do<br>wn	0.00<br>057

UAP1	UDP-N-acetylglucosamine pyrophosphorylase 1 [Source:HGNC Symbol;Acc:HGNC:12457]	15.3687	6.0189	1.35243	up	0.00057
ENSGALG00000002771	Uncharacterized protein [Source:UniProtKB/TrEMBL;Acc:F1N8J7]	28.6923	66.1219	-1.20447	down	0.00057
CRIP2	cysteine-rich protein 2 [Source:HGNC Symbol;Acc:HGNC:2361]	40.0758	6.48344	2.6279	up	0.00057
ENSGALG00000003022	Dimethylaniline monooxygenase [N-oxide-forming] [Source:UniProtKB/TrEMBL;Acc:E1C7G8]	41.1966	15.8605	1.37708	up	0.00057
PRDX6	peroxiredoxin-6 [Source:RefSeq peptide;Acc:NP_001034418]	93.	208	-1.1639	do	0.00

		043 8	.48 7	8	wn	057
FASLG	tumor necrosis factor ligand superfamily member 6 [Source:RefSeq peptide;Acc:NP_001026730]	0.0 765 4	0.6 272 3	-3.0347	do wn	0.00 057
NUF2	Kinetochore protein Nuf2 [Source:UniProtKB/Swiss-Prot;Acc:Q76I90]	6.9 218 2	0.8 875 5	2.96325	up	0.00 057
FAM129 A	protein Niban [Source:RefSeq peptide;Acc:NP_001012614]	0.3 929 5	1.6 242 5	-2.0473 6	do wn	0.00 057
PALMD	palmdelphin [Source:RefSeq peptide;Acc:NP_001264481]	18. 436	67. 085	-1.8634 4	do wn	0.00 057

		5	9			
PRDX1	Peroxiredoxin-1 [Source:UniProtKB/Swiss-Prot;Acc:P0CB50]	518<br>.92<br>2	235<br>.54<br>6	1.13951	up	0.00<br>057
Rd	Riboflavin-binding protein Riboflavin-binding protein, plasma form Riboflavin-binding protein, yolk major form Riboflavin-binding protein, yolk minor form [Source:UniProtKB/Swiss-Prot;Acc:P02752]	0.7<br>980<br>7	17.<br>439<br>6	-4.4497<br>1	do<br>wn	0.00<br>057
FAAH	fatty acid amide hydrolase [Source:HGNC Symbol;Acc:HGNC:3553]	52.<br>36	25.<br>125<br>5	1.05931	up	0.00<br>057
ORC1	origin recognition complex subunit 1 [Source:RefSeq peptide;Acc:NP_001026457]	1.5<br>871<br>1	0.5<br>215<br>7	1.60548	up	0.00<br>057

DHCR24	24-dehydrocholesterol reductase [Source:RefSeq peptide;Acc:NP_001026459]	1.5<br>703<br>8	6.5<br>926<br>5	-2.0697<br>5	do<br>wn	0.00<br>057
KANK4	KN motif and ankyrin repeat domains 4 [Source:HGNC Symbol;Acc:HGNC:27263]	5.6<br>512<br>6	0.3<br>867<br>8	3.869	up	0.00<br>057
CRYZ	quinone oxidoreductase [Source:RefSeq peptide;Acc:NP_001073231]	7.8<br>502<br>9	1.4<br>489<br>4	2.43775	up	0.00<br>057
SCARNA<br>6	Small Cajal body specific RNA 6 [Source:RFAM;Acc:RF00478]	298<br>.37<br>9	180<br>4.8<br>5	-2.5966<br>6	do<br>wn	0.00<br>057
PFKL	phosphofructokinase, liver [Source:HGNC Symbol;Acc:HGNC:8876]	40.	15.	1.33279	up	0.00

		021	888			057
		8	6			
MOGAT1	monoacylglycerol O-acyltransferase 1 [Source:HGNC Symbol;Acc:HGNC:18210]	9.9	0.4	4.44662	up	0.00
		526	564			057
		2	3			
CPN2	carboxypeptidase N subunit 2 [Source:HGNC Symbol;Acc:HGNC:2313]	0.9	5.3	-2.4920	do	0.00
		547	710	3	wn	057
		4	3			
TFRC	transferrin receptor protein 1 [Source:RefSeq peptide;Acc:NP_990587]	35.	7.3	2.28579	up	0.00
		721	255			057
		5	3			
ENSGAL	Uncharacterized protein [Source:UniProtKB/TrEMBL;Acc:F1NXE6]	2.3	28.	-3.5870	do	0.00
G000000		594	354	8	wn	057

23879		3	8			
PSMD2	26S proteasome non-ATPase regulatory subunit 2 [Source:RefSeq peptide;Acc:NP_001012952]	37.9909	15.7264	1.27246	up	0.00057
AHSG	alpha-2-HS-glycoprotein [Source:HGNC Symbol;Acc:HGNC:349]	2625.3	269.274	3.28533	up	0.00057
FETUB	fetuin-B precursor [Source:RefSeq peptide;Acc:NP_001264337]	309.483	43.2865	2.83787	up	0.00057
RARRES1	ovocalyxin-32 precursor [Source:RefSeq peptide;Acc:NP_989865]	48.9119	108.899	-1.15474	down	0.00057

CP	ceruloplasmin (ferroxidase) [Source:HGNC Symbol;Acc:HGNC:2295]	1.82086	111.7	-5.93887	down	0.00057
KLHL6	kelch-like 6 [Source:RefSeq peptide;Acc:NP_001026474]	1.05283	3.25221	-1.62715	down	0.00057
6	Solute carrier family 25 member 36 [Source:UniProtKB/Swiss-Prot;Acc:Q5ZKP7]	1.13378	4.09879	-1.85406	down	0.00057
FARSB	phenylalanyl-tRNA synthetase beta chain [Source:RefSeq peptide;Acc:NP_001006543]	20.8281	9.62476	1.11371	up	0.00057
PID1	PTB-containing, cubilin and LRP1-interacting protein [Source:RefSeq	9.5	2.2	2.09329	up	0.00

	peptide;Acc:NP_001171615]	949	485			057
		8	3			
OPA1	dynamin-like 120 kDa protein, mitochondrial precursor [Source:RefSeq	17.	6.5	1.40207	up	0.00
	peptide;Acc:NP_001034398]	224	175			057
		6	2			
FXR1	fragile X mental retardation syndrome-related protein 1 [Source:RefSeq	8.0	16.	-1.0475	do	0.00
	peptide;Acc:NP_001012619]	650	670	4	wn	057
		6	5			
USP13	ubiquitin specific peptidase 13 (isopeptidase T-3) [Source:HGNC Symbol;Acc:HGNC:12611]	0.8	0.2	1.64585	up	0.00
		834	823			057
		8	2			
NDUFB5	NADH dehydrogenase [Source:RefSeq peptide;Acc:NP_001006553]	166	73.	1.17841	up	0.00
		.13	405			057

		6	4			
SKIL	ski-like protein [Source:RefSeq peptide;Acc:NP_990505]	1.48162	6.59337	-2.15384	down	0.00057
GFM1	G elongation factor, mitochondrial 1 [Source:HGNC Symbol;Acc:HGNC:13780]	18.0431	6.86131	1.39489	up	0.00057
RACGAP1	Rac GTPase activating protein 1 [Source:HGNC Symbol;Acc:HGNC:9804]	3.37135	0.43189	2.96461	up	0.00057
ENSGALG00000005980	Uncharacterized protein [Source:UniProtKB/TrEMBL;Acc:H9KZC8]	28.862	9.91704	1.54119	up	0.00057

KRT8	keratin 8, type II [Source:HGNC Symbol;Acc:HGNC:6446]	43.6859	16.6656	1.39029	up	0.00057
ATP5B	Gallus gallus ATP synthase, H+ transporting, mitochondrial F1 complex, beta polypeptide (ATP5B), nuclear gene encoding mitochondrial protein, mRNA. [Source:RefSeq mRNA;Acc:NM_001031391]	871.116	208.007	2.06623	up	0.00057
ENSGALG00000024020	Uncharacterized protein [Source:UniProtKB/TrEMBL;Acc:H9L3I5]	13.2773	112.732	-3.08586	down	0.00057
FECH	ferrochelatase, mitochondrial precursor [Source:RefSeq peptide;Acc:NP_989527]	9.4523	4.28698	1.14071	up	0.00057
PSTPIP2	proline-serine-threonine phosphatase interacting protein 2 [Source:HGNC	1.0	4.6	-2.1187	do	0.00

	Symbol;Acc:HGNC:9581]	676<br>2	369<br>6	8	wn	057
Worthingt<br>on	Avidin [Source:UniProtKB/Swiss-Prot;Acc:P02701]	2.3<br>425<br>5	36.<br>825<br>9	-3.9745<br>7	do<br>wn	0.00<br>057
IL7R	interleukin-7 receptor subunit alpha precursor [Source:RefSeq peptide;Acc:NP_001073575]	0.5<br>453<br>9	7.1<br>198<br>8	-3.7064<br>9	do<br>wn	0.00<br>057
C7	complement component 7 [Source:HGNC Symbol;Acc:HGNC:1346]	6.6<br>014<br>3	41.<br>810<br>1	-2.663	do<br>wn	0.00<br>057
FST	Follistatin [Source:UniProtKB/Swiss-Prot;Acc:Q90844]	10.<br>175	31.<br>915	-1.6491<br>8	do<br>wn	0.00<br>057

		4	7			
CENPH	Centromere protein H [Source:UniProtKB/Swiss-Prot;Acc:Q90ZF9]	8.2	1.3	2.62448	up	0.00
		750	419			057
		9	2			
ANKRA2	ankyrin repeat family A protein 2 [Source:RefSeq peptide;Acc:NP_001006565]	4.0	9.5	-1.2433	do	0.00
		463	793	2	wn	057
		3	9			
ZNF366	zinc finger protein 366 [Source:HGNC Symbol;Acc:HGNC:18316]	1.1	3.4	-1.5236	do	0.00
		853	082	5	wn	057
		9	3			
DOCK8	dedicator of cytokinesis 8 [Source:HGNC Symbol;Acc:HGNC:19191]	0.4	2.8	-2.6074	do	0.00
		736	866		wn	057
		9	6			

SLC1A1	solute carrier family 1 (neuronal/epithelial high affinity glutamate transporter, system Xag), member 1 [Source:HGNC Symbol;Acc:HGNC:10939]	3.36419	0.31054	3.43739	up	0.00057
JAK2	tyrosine-protein kinase JAK2 [Source:RefSeq peptide;Acc:NP_001025709]	2.23305	5.98097	-1.42136	down	0.00057
ANXA1	annexin A1 [Source:RefSeq peptide;Acc:NP_996789]	1.26373	3.43891	-1.44426	down	0.00057
CTSL2	cathepsin L1 precursor [Source:RefSeq peptide;Acc:NP_001161481]	163.813	442.584	-1.4339	down	0.00057
NXNL2	nucleoredoxin-like 2 [Source:HGNC Symbol;Acc:HGNC:30482]	0.6	0.1	2.53324	up	0.00

		802<br>6	175<br>2			057
gadd45	growth arrest and DNA damage-inducible protein GADD45 alpha [Source:RefSeq peptide;Acc:NP_001038143]	0.7<br>162	2.9<br>499<br>4	-2.0422<br>4	do<br>wn	0.00<br>057
REEP5	receptor expression-enhancing protein 5 [Source:RefSeq peptide;Acc:NP_001239006]	9.3<br>251<br>5	20.<br>600<br>6	-1.1434<br>8	do<br>wn	0.00<br>057
ST8SIA4	CMP-N-acetylneuraminate-poly-alpha-2,8-sialyltransferase [Source:RefSeq peptide;Acc:NP_989614]	0.3<br>998<br>7	2.2<br>725<br>2	-2.5067<br>1	do<br>wn	0.00<br>057
PHAX	phosphorylated adapter RNA export protein [Source:RefSeq peptide;Acc:NP_001006575]	0.8<br>069	2.0<br>788	-1.3652<br>8	do<br>wn	0.00<br>057

		3	8			
MCTP1	multiple C2 domains, transmembrane 1 [Source:HGNC Symbol;Acc:HGNC:26183]	3.78567	19.1562	-2.33919	down	0.00057
MBLAC2	Metallo-beta-lactamase domain-containing protein 2 [Source:UniProtKB/Swiss-Prot;Acc:Q5F336]	2.56808	5.7708	-1.16808	down	0.00057
CDO1	cysteine dioxygenase type 1 [Source:HGNC Symbol;Acc:HGNC:1795]	21.3741	116.762	-2.44963	down	0.00057
ONECUT2	one cut homeobox 2 [Source:HGNC Symbol;Acc:HGNC:8139]	3.81511	19.2826	-2.3375	down	0.00057

LIPG	lipase, endothelial [Source:HGNC Symbol;Acc:HGNC:6623]	0.4<br>315<br>6	10.<br>03	-4.5386<br>3	do<br>wn	0.00<br>057
ENSGAL<br>G000000<br>05814	Uncharacterized protein [Source:UniProtKB/TrEMBL;Acc:F1NB98]	5.1<br>917<br>7	1.1<br>716<br>6	2.14767	up	0.00<br>057
FANCG	Fanconi anemia, complementation group G [Source:RefSeq peptide;Acc:NP_989709]	1.4<br>582	0.5<br>648<br>5	1.36825	up	0.00<br>057
NPR2	natriuretic peptide receptor 2 [Source:HGNC Symbol;Acc:HGNC:7944]	0.4<br>330<br>3	1.0<br>141	-1.2276<br>6	do<br>wn	0.00<br>057
CAPSL	calcyphosine-like [Source:HGNC Symbol;Acc:HGNC:28375]	4.6	20.	-2.1225	do	0.00

		947<br>2	444<br>1	7	wn	057
RICTOR	RPTOR independent companion of MTOR, complex 2 [Source:HGNC Symbol;Acc:HGNC:28611]	2.0<br>346<br>8	5.0<br>439<br>2	-1.3097<br>4	do<br>wn	0.00<br>057
PLPP1	phospholipid phosphatase 1 [Source:HGNC Symbol;Acc:HGNC:9228]	9.7<br>609<br>8	38.<br>325	-1.9731<br>9	do<br>wn	0.00<br>057
CD180	CD180 molecule [Source:HGNC Symbol;Acc:HGNC:6726]	0.7<br>601<br>3	3.7<br>137<br>7	-2.2885<br>7	do<br>wn	0.00<br>057
SCAMP1	secretory carrier-associated membrane protein 1 [Source:RefSeq peptide;Acc:NP_001264922]	2.4<br>273	5.9<br>637	-1.2968<br>6	do<br>wn	0.00<br>057

		2	5			
GLDC	glycine dehydrogenase [Source:RefSeq peptide;Acc:NP_989653]	139<br>.78<br>5	31.<br>758<br>4	2.138	up	0.00<br>057
ENSGAL<br>G000000<br>28142	Uncharacterized protein [Source:UniProtKB/TrEMBL;Acc:R4GIV6]	0.1<br>226<br>5	0.7<br>615<br>6	-2.6344<br>1	do<br>wn	0.00<br>057
KLF9	Kruppel-like factor 9 [Source:HGNC Symbol;Acc:HGNC:1123]	33.<br>377<br>1	4.3<br>233<br>4	2.94864	up	0.00<br>057
ABHD17<br>B	Alpha/beta hydrolase domain-containing protein 17B [Source:UniProtKB/Swiss-Prot;Acc:Q5ZJ01]	5.2<br>436<br>2	10.<br>994<br>6	-1.0681<br>6	do<br>wn	0.00<br>057

ALDH1A1	retinal dehydrogenase 1 [Source:RefSeq peptide;Acc:NP_989908]	38.4374	78.0425	-1.02175	down	0.00057
ISCA1	Iron-sulfur cluster assembly 1 homolog, mitochondrial [Source:UniProtKB/Swiss-Prot;Acc:Q5ZJ74]	7.9031	25.4797	-1.68886	down	0.00057
LNPEP	leucyl/cystinyl aminopeptidase [Source:HGNC Symbol;Acc:HGNC:6656]	4.46171	15.3801	-1.78539	down	0.00057
LPL	Lipoprotein lipase [Source:UniProtKB/Swiss-Prot;Acc:P11602]	25.5428	0.96791	4.7219	up	0.00057
RFESD	Rieske (Fe-S) domain containing [Source:HGNC Symbol;Acc:HGNC:29587]	0.6	3.5	-2.4718	do	0.00

		380<br>2	394<br>1	3	wn	057
CCDC12<br>5	coiled-coil domain containing 125 [Source:HGNC Symbol;Acc:HGNC:28924]	0.1<br>848	1.0<br>776<br>6	-2.5438<br>6	do<br>wn	0.00<br>057
GNG10	guanine nucleotide-binding protein G(I)/G(S)/G(O) subunit gamma-10 [Source:RefSeq peptide;Acc:NP_001191934]	1.5<br>878<br>7	3.8<br>216<br>1	-1.2670<br>9	do<br>wn	0.00<br>057
SMC2	structural maintenance of chromosomes protein 2 [Source:RefSeq peptide;Acc:NP_990561]	30.<br>034<br>6	3.0<br>82	3.28469	up	0.00<br>057
SVEP1	sushi, von Willebrand factor type A, EGF and pentraxin domain containing 1 [Source:HGNC Symbol;Acc:HGNC:15985]	3.2<br>722	1.0<br>471	1.64381	up	0.00<br>057

			4			
AP3S1	AP-3 complex subunit sigma-1 [Source:RefSeq peptide;Acc:NP_001006586]	12.9701	28.9298	-1.15737	down	0.00057
LOX	protein-lysine 6-oxidase precursor [Source:RefSeq peptide;Acc:NP_990812]	9.58854	3.98169	1.26793	up	0.00057
PPIC	peptidylprolyl isomerase C (cyclophilin C) [Source:HGNC Symbol;Acc:HGNC:9256]	7.752	1.85989	2.05935	up	0.00057
OC3	uncharacterized protein LOC417943 precursor [Source:RefSeq peptide;Acc:NP_001188315]	1.74582	0.36647	2.25215	up	0.00107

CSF2RB	Uncharacterized protein [Source:UniProtKB/TrEMBL;Acc:F1NCG4]	0.3<br>700<br>7	3.8<br>954<br>8	-3.3959<br>5	do<br>wn	0.00<br>107
LANCL3	LanC lantibiotic synthetase component C-like 3 (bacterial) [Source:HGNC Symbol;Acc:HGNC:24767]	2.6<br>883<br>1	6.4<br>552<br>8	-1.2637<br>8	do<br>wn	0.00<br>107
KLHDC4	kelch domain-containing protein 4 [Source:RefSeq peptide;Acc:NP_001025749]	5.9<br>897<br>3	2.1<br>519<br>8	1.47683	up	0.00<br>107
COMT	catechol-O-methyltransferase [Source:HGNC Symbol;Acc:HGNC:2228]	45.<br>757<br>2	16.<br>958<br>7	1.43198	up	0.00<br>107
DMB2	Major histocompatibility complex class II beta chain BMA2, (similar to HLA class II, D beta	1.1	20.	-4.1891	do	0.00

	chain) precursor [Source:RefSeq peptide;Acc:NP_001128638]	239<br>9	502<br>9	3	wn	107
GSN	gelsolin precursor [Source:RefSeq peptide;Acc:NP_990265]	0.3<br>449<br>1	3.8<br>178<br>3	-3.4684<br>4	do<br>wn	0.00<br>107
ATP2A3	sarcoplasmic/endoplasmic reticulum calcium ATPase 3 [Source:RefSeq peptide;Acc:NP_990222]	1.9<br>638<br>8	4.8<br>287<br>7	-1.2979<br>5	do<br>wn	0.00<br>107
SLC43A2	solute carrier family 43 (amino acid system L transporter), member 2 [Source:HGNC Symbol;Acc:HGNC:23087]	7.9<br>860<br>8	25.<br>551<br>3	-1.6778<br>4	do<br>wn	0.00<br>107
ITGA8	integrin alpha-8 precursor [Source:RefSeq peptide;Acc:NP_990619]	2.0<br>983	4.3<br>958	-1.0668<br>9	do<br>wn	0.00<br>107

		6	9			
KAT2B	K(lysine) acetyltransferase 2B [Source:HGNC Symbol;Acc:HGNC:8638]	2.9	6.2	-1.0825	do	0.00
		391	245	7	wn	107
		5	4			
OSBPL3	oxysterol binding protein like 3 [Source:HGNC Symbol;Acc:HGNC:16370]	0.1	1.0	-2.6030	do	0.00
		723	474	7	wn	107
		9	3			
SERPIN	serpin B6 [Source:RefSeq peptide;Acc:NP_001006377]	2.5	6.9	-1.4254	do	0.00
B6		780	244	4	wn	107
		1	4			
FIGNL1	fidgetin-like 1 [Source:HGNC Symbol;Acc:HGNC:13286]	1.8	0.4	2.05063	up	0.00
		119	373			107
		4	6			

DEF6	DEF6, guanine nucleotide exchange factor [Source:HGNC Symbol;Acc:HGNC:2760]	1.48407	3.46595	-1.22369	down	0.00107
CSF3	Myelomonocytic growth factor [Source:UniProtKB/Swiss-Prot;Acc:P13854]	0.31816	1.11098	-1.80399	down	0.00107
CDC6	cell division cycle 6 [Source:HGNC Symbol;Acc:HGNC:1744]	1.73057	0.18146	3.25351	up	0.00107
UBXN6	UBX domain protein 6 [Source:HGNC Symbol;Acc:HGNC:14928]	1.76793	4.18646	-1.24367	down	0.00107
MEMO1	protein MEMO1 [Source:RefSeq peptide;Acc:NP_001182352]	3.8	1.6	1.19482	up	0.00

		514<br>1	824<br>5			107
C3H2ORF43	UPF0554 protein C2orf43 homolog [Source:RefSeq peptide;Acc:NP_001026264]	3.0894<br>4	7.5295<br>2	-1.2852<br>1	down	0.00<br>107
FRYL	FRY like transcription coactivator [Source:HGNC Symbol;Acc:HGNC:29127]	1.7478<br>9	4.5142<br>1	-1.3688<br>6	down	0.00<br>107
CGT	2-hydroxyacylsphingosine 1-beta-galactosyltransferase precursor [Source:RefSeq peptide;Acc:NP_989535]	3.3137<br>3	9.0875<br>5	-1.4554<br>4	down	0.00<br>107
TBC1D19	TBC1 domain family member 19 [Source:HGNC Symbol;Acc:HGNC:25624]	0.3308	0.8720	-1.3981	down	0.00<br>107

		8	4			
DCLRE1A	DNA cross-link repair 1A protein [Source:RefSeq peptide;Acc:NP_001008683]	1.8 696 3	0.9 137 5	1.03288	up	0.00 107
RGL1	ral guanine nucleotide dissociation stimulator-like 1 [Source:RefSeq peptide;Acc:NP_001026442]	4.3 910 8	9.1 180 3	-1.0541 5	down	0.00 107
VAV3	guanine nucleotide exchange factor VAV3 [Source:RefSeq peptide;Acc:NP_996745]	1.6 923	9.0 640 3	-2.4211 6	down	0.00 107
CACYBP	calcyclin binding protein [Source:HGNC Symbol;Acc:HGNC:30423]	9.1 241 3	18. 715 5	-1.0364 7	down	0.00 107

ACSL3	acyl-CoA synthetase long-chain family member 3 [Source:HGNC Symbol;Acc:HGNC:3570]	6.38005	1.2229	2.38327	up	0.00107
TMEM41A	transmembrane protein 41A isoform 1 [Source:RefSeq peptide;Acc:NP_001264615]	9.97232	20.7942	-1.06018	down	0.00107
RINT1	RAD50-interacting protein 1 [Source:RefSeq peptide;Acc:NP_001265046]	1.66099	3.69395	-1.15312	down	0.00153
BIK	BCL2-interacting killer (apoptosis-inducing) [Source:RefSeq peptide;Acc:NP_001264987]	1.12424	2.93053	-1.3822	down	0.00153
scyc1	lymphotactin precursor [Source:RefSeq peptide;Acc:NP_990377]	0.0	10.	-6.8447	do	0.00

		951	94	1	wn	153
		8				
ASMTL	acetylserotonin O-methyltransferase-like [Source:HGNC Symbol;Acc:HGNC:751]	3.2	1.2	1.33207	up	0.00
		098	749			153
		3	4			
CPM	carboxypeptidase M precursor [Source:RefSeq peptide;Acc:NP_001186554]	0.2	0.8	-1.8366	do	0.00
		330	322		wn	153
		2	6			
SYTL2	synaptotagmin like 2 [Source:HGNC Symbol;Acc:HGNC:15585]	0.1	0.7	-2.0875	do	0.00
		812	704	5	wn	153
		7	4			
LPCAT2	lysophosphatidylcholine acyltransferase 2 [Source:RefSeq peptide;Acc:NP_001025739]	1.4	3.3	-1.1869	do	0.00
		605	252	6	wn	153

		3	3			
MAGI1	membrane associated guanylate kinase, WW and PDZ domain containing 1 [Source:HGNC Symbol;Acc:HGNC:946]	7.2 542	2.3 986 2	1.59661	up	0.00 153
TLR4	toll-like receptor 4 precursor [Source:RefSeq peptide;Acc:NP_001025864]	0.2 966 8	2.6 571 7	-3.1629	do wn	0.00 153
CBX4	E3 SUMO-protein ligase CBX4 [Source:RefSeq peptide;Acc:NP_989973]	1.3 427 6	3.8 333 6	-1.5134 1	do wn	0.00 153
ENSGAL G000000 17395	NA	1.3 615 7	0.2 610 4	2.3829	up	0.00 153

MARVELD3	MARVEL domain containing 3 [Source:HGNC Symbol;Acc:HGNC:30525]	0.85542	2.09641	-1.29322	down	0.00153
CATHL2	Cathelicidin-2 [Source:UniProtKB/Swiss-Prot;Acc:Q2IAL7]	1.54598	6.19149	-2.00176	down	0.00153
TNFRSF11B	tumor necrosis factor receptor superfamily member 11B precursor [Source:RefSeq peptide;Acc:NP_001028813]	0.25015	0.85353	-1.77065	down	0.00153
BMP7	bone morphogenetic protein 7 [Source:HGNC Symbol;Acc:HGNC:1074]	0.47027	1.02513	-1.12424	down	0.00153
DBNDD2	dysbindin (dystrobrevin binding protein 1) domain containing 2 [Source:HGNC	6.0	2.9	1.01871	up	0.00

	Symbol;Acc:HGNC:15881]	167<br>4	696<br>1			153
NCLN	nicalin precursor [Source:RefSeq peptide;Acc:NP_001026525]	10.<br>293<br>9	4.7<br>824<br>1	1.10599	up	0.00<br>153
KLF2	Kruppel-like factor 2 [Source:UniProtKB/TrEMBL;Acc:E1C442]	2.3<br>345<br>8	4.9<br>973<br>5	-1.098	do<br>wn	0.00<br>153
FNIP2	folliculin interacting protein 2 [Source:HGNC Symbol;Acc:HGNC:29280]	13.<br>042<br>9	5.6<br>96	1.19524	up	0.00<br>153
PLK4	polo-like kinase 4 [Source:HGNC Symbol;Acc:HGNC:11397]	1.3<br>481	0.2<br>243	2.5874	up	0.00<br>153

ENSGALG00000007355	Uncharacterized protein [Source:UniProtKB/TrEMBL;Acc:E1C272]	5.6672	13.4204	-1.24372	down	0.00153
ENSGALG00000009777	Uncharacterized protein [Source:UniProtKB/TrEMBL;Acc:E1C4F0]	43.9601	5.01626	3.13151	up	0.00153
PYROXD2	pyridine nucleotide-disulphide oxidoreductase domain 2 [Source:HGNC Symbol;Acc:HGNC:23517]	3.73919	1.73757	1.10565	up	0.00153
GLS	glutaminase kidney isoform, mitochondrial precursor [Source:RefSeq peptide;Acc:NP_001026419]	2.27737	5.94009	-1.38311	down	0.00153
PARP9	poly(ADP-ribose) polymerase family member 9 [Source:HGNC Symbol;Acc:HGNC:24118]	3.5	7.2	-1.0320	do	0.00

		296	179	5	wn	153
		7	2			
GFI1	growth factor independent 1 transcription repressor [Source:HGNC Symbol;Acc:HGNC:4237]	0.0	1.0	-3.7603	do	0.00
		757	265	4	wn	153
		6	7			
HBE1	epsilon globin [Source:RefSeq peptide;Acc:NP_001026660]	3.9	0.0	5.45406	up	0.00
		859	909			198
		8	3			
TPN	tapasin isoform 1 precursor [Source:RefSeq peptide;Acc:NP_001029988]	20.	54.	-1.4258	do	0.00
		177	211	4	wn	198
		5	1			
PLCL2	phospholipase C like 2 [Source:HGNC Symbol;Acc:HGNC:9064]	0.2	0.7	-1.5150	do	0.00
		640	545	5	wn	198

		2	9			
DSCC1	DNA replication and sister chromatid cohesion 1 [Source:HGNC Symbol;Acc:HGNC:24453]	2.0<br>290<br>5	0.5<br>501<br>5	1.88292	up	0.00<br>198
MRPL2	mitochondrial ribosomal protein L2 [Source:HGNC Symbol;Acc:HGNC:14056]	38.<br>571<br>1	17.<br>897<br>1	1.1078	up	0.00<br>198
MPP1	55 kDa erythrocyte membrane protein [Source:UniProtKB/Swiss-Prot;Acc:Q5ZJ00]	32.<br>400<br>9	12.<br>074<br>5	1.42407	up	0.00<br>198
ENSGAL<br>G000000<br>20388	Uncharacterized protein [Source:UniProtKB/TrEMBL;Acc:F1NPN5]	8.7<br>842<br>5	199<br>.46<br>1	-4.5050<br>5	do<br>wn	0.00<br>198

HMGCS2	3-hydroxy-3-methylglutaryl-CoA synthase 2 (mitochondrial) [Source:HGNC Symbol;Acc:HGNC:5008]	417.454	165.688	1.33315	up	0.00198
CDC7	cell division cycle 7 [Source:HGNC Symbol;Acc:HGNC:1745]	1.01208	0.09943	3.34745	up	0.00198
LAMP3	lysosome-associated membrane glycoprotein 3 precursor [Source:RefSeq peptide;Acc:NP_001139604]	0.03164	1.91742	-5.92113	down	0.00198
MORC3	MORC family CW-type zinc finger 3 [Source:RefSeq peptide;Acc:NP_001244153]	5.36174	12.2351	-1.19026	down	0.00241
ENSGAL	Cystathionine beta-synthase [Source:UniProtKB/TrEMBL;Acc:E1BYG4]	14.	45.	-1.6476	do	0.00

G000000<br>16196		409<br>3	148<br>1	7	wn	241
MPI	mannose phosphate isomerase [Source:HGNC Symbol;Acc:HGNC:7216]	9.2<br>977<br>1	3.2<br>600<br>7	1.51197	up	0.00<br>241
CSPG4	chondroitin sulfate proteoglycan 4 [Source:HGNC Symbol;Acc:HGNC:2466]	0.7<br>886<br>6	0.1<br>774<br>9	2.15167	up	0.00<br>241
GINS2	GINS complex subunit 2 (Psf2 homolog) [Source:HGNC Symbol;Acc:HGNC:24575]	1.3<br>671<br>2	0.2<br>057<br>8	2.73194	up	0.00<br>241
Ii	CD74 molecule, major histocompatibility complex, class II invariant chain [Source:RefSeq peptide;Acc:NP_001001613]	22.<br>39	548<br>.37	-4.6142<br>3	do<br>wn	0.00<br>241

			2			
LECT2	myeloid protein 1 precursor [Source:RefSeq peptide;Acc:NP_990809]	0.51937	5.57704	-3.42466	down	0.00241
TMEM100	transmembrane protein 100 [Source:HGNC Symbol;Acc:HGNC:25607]	0.62249	1.59365	-1.35621	down	0.00241
AGMO	alkylglycerol monooxygenase [Source:HGNC Symbol;Acc:HGNC:33784]	0.04508	33.0274	-9.5171	down	0.00241
IMPA2	inositol(myo)-1(or 4)-monophosphatase 2 [Source:HGNC Symbol;Acc:HGNC:6051]	6.03611	1.33624	2.17544	up	0.00241

FKBP51	peptidyl-prolyl cis-trans isomerase FKBP5 [Source:RefSeq peptide;Acc:NP_001005431]	93.<br>104<br>8	31.<br>192<br>2	1.57767	up	0.00<br>241
RAB7B	RAB7B, member RAS oncogene family [Source:HGNC Symbol;Acc:HGNC:30513]	1.1<br>597<br>2	0.2<br>573<br>8	2.17182	up	0.00<br>241
ENSGAL<br>G000000<br>20380	Uncharacterized protein [Source:UniProtKB/TrEMBL;Acc:E1C913]	0.2<br>126<br>1	1.6<br>472<br>5	-2.9537<br>6	do<br>wn	0.00<br>241
TMEM53	Gallus gallus transmembrane protein 53 (TMEM53), mRNA. [Source:RefSeq mRNA;Acc:NM_001277580]	6.7<br>017<br>9	2.1<br>350<br>2	1.6503	up	0.00<br>241
NRROS	negative regulator of reactive oxygen species [Source:HGNC Symbol;Acc:HGNC:24613]	1.0	4.0	-1.9977	do	0.00

		099	334		wn	241
		7	7			
GAS2L1	growth arrest specific 2 like 1 [Source:HGNC Symbol;Acc:HGNC:16955]	8.4	2.7	1.63458	up	0.00
		989	372			282
		9	2			
CenpA	Histone H3-like centromeric protein A [Source:UniProtKB/Swiss-Prot;Acc:Q6XXM1]	9.2	1.0	3.16338	up	0.00
		055	274			282
		2	9			
SLC25A3	solute carrier family 25 member 38 [Source:HGNC Symbol;Acc:HGNC:26054]	0.6	2.3	-1.7939	do	0.00
8		657	084	4	wn	282
ENSGAL	Uncharacterized protein [Source:UniProtKB/TrEMBL;Acc:F1NL83]	225	94.	1.24534	up	0.00
G000000		.17	979			282
07863		4	9			

HGSNAT	heparan-alpha-glucosaminide N-acetyltransferase [Source:HGNC Symbol;Acc:HGNC:26527]	9.3<br>654	19.<br>063<br>4	-1.0253<br>9	do<br>wn	0.00<br>282
PDLIM3	PDZ and LIM domain protein 3 [Source:RefSeq peptide;Acc:NP_001001764]	0.4<br>958	1.2<br>026<br>5	-1.2783<br>8	do<br>wn	0.00<br>282
BKJ	Beta-keratin-related protein [Source:UniProtKB/Swiss-Prot;Acc:O13152]	0.4<br>113<br>3	2.0<br>187<br>8	-2.2951<br>2	do<br>wn	0.00<br>282
SLC51A	organic solute transporter subunit alpha [Source:RefSeq peptide;Acc:NP_001264626]	56.<br>177<br>5	25.<br>160<br>9	1.15881	up	0.00<br>282
Worthingt	Avidin [Source:UniProtKB/Swiss-Prot;Acc:P02701]	0.3	6.0	-4.0963	do	0.00

on		546<br>7	665<br>1	1	wn	322
LAMP1	lysosome-associated membrane glycoprotein 1 precursor [Source:RefSeq peptide;Acc:NP_990614]	102<br>.35<br>9	237<br>.84<br>5	-1.2163<br>9	do<br>wn	0.00<br>322
SLC6A2	sodium-dependent noradrenaline transporter [Source:RefSeq peptide;Acc:NP_990047]	0.1<br>033<br>8	0.7<br>553<br>1	-2.8691<br>6	do<br>wn	0.00<br>322
CCNF	cyclin F [Source:HGNC Symbol;Acc:HGNC:1591]	3.5<br>645<br>1	0.7<br>693<br>9	2.21193	up	0.00<br>322
CARD11	caspase recruitment domain-containing protein 11 [Source:RefSeq peptide;Acc:NP_001006161]	0.3<br>608	1.3<br>511	-1.9046<br>2	do<br>wn	0.00<br>322

		8	8			
TFAP4	transcription factor AP-4 (activating enhancer binding protein 4) [Source:HGNC Symbol;Acc:HGNC:11745]	3.5 807	1.3 478 1	1.40963	up	0.00 322
ENSGALG00000008518	Glutamine synthetase [Source:UniProtKB/TrEMBL;Acc:F1P0D2]	4.7 708 5	1.8 960 6	1.33124	up	0.00 322
NR4A3	nuclear receptor subfamily 4 group A member 3 [Source:HGNC Symbol;Acc:HGNC:7982]	0.1 681 8	0.9 258	-2.4606 7	do wn	0.00 322
EPHB2	ephrin type-B receptor 2 precursor [Source:RefSeq peptide;Acc:NP_996834]	7.4 029 7	3.5 148 8	1.07463	up	0.00 322

MCAM	cell surface glycoprotein MUC18 precursor [Source:RefSeq peptide;Acc:NP_001004768]	6.9<br>018<br>7	2.5<br>569<br>1	1.43259	up	0.00<br>322
PIGR	polymeric immunoglobulin receptor precursor [Source:RefSeq peptide;Acc:NP_001038109]	0.1<br>854<br>6	184<br>.53<br>4	-9.9585<br>8	do<br>wn	0.00<br>322
GINS1	DNA replication complex GINS protein PSF1 [Source:RefSeq peptide;Acc:NP_001012621]	6.2<br>413<br>1	0.4<br>546	3.77919	up	0.00<br>322
ABCG8	ATP binding cassette subfamily G member 8 [Source:HGNC Symbol;Acc:HGNC:13887]	6.2<br>470<br>9	0.0<br>666<br>8	6.54969	up	0.00<br>322
RRAGD	Ras-related GTP binding D [Source:HGNC Symbol;Acc:HGNC:19903]	1.6	4.0	-1.2420	do	0.00

		944	079	7	wn	322
		1	2			
DCTD	deoxycytidylate deaminase [Source:RefSeq peptide;Acc:NP_001006444]	16.	2.0	3.04268	up	0.00
		790	376			322
		8	7			
LSP1	lymphocyte-specific protein 1 [Source:RefSeq peptide;Acc:NP_989673]	0.6	4.1	-2.7254	do	0.00
		342	947	7	wn	322
		4	3			
N4BP2L1	NEDD4 binding protein 2-like 1 [Source:HGNC Symbol;Acc:HGNC:25037]	22.	45.	-1.0273	do	0.00
		410	679	6	wn	362
		8	9			
SEMA3G	sema domain, immunoglobulin domain (Ig), short basic domain, secreted, (semaphorin) 3G	0.7	0.2	1.62683	up	0.00
	[Source:HGNC Symbol;Acc:HGNC:30400]	342	377			362

		4	5			
DOCK2	dedicator of cytokinesis 2 [Source:HGNC Symbol;Acc:HGNC:2988]	0.1<br>387<br>3	2.0<br>010<br>9	-3.8504<br>9	do<br>wn	0.00<br>362
ENSGAL<br>G000000<br>00017	Uncharacterized protein [Source:UniProtKB/TrEMBL;Acc:H9KYN4]	6.4<br>475	2.8<br>666<br>8	1.16936	up	0.00<br>362
IMMT	mitochondrial inner membrane protein [Source:RefSeq peptide;Acc:NP_001006462]	20.<br>357<br>9	9.3<br>502<br>5	1.12251	up	0.00<br>362
GDF10	growth differentiation factor 10 [Source:HGNC Symbol;Acc:HGNC:4215]	0.3<br>791<br>3	0.9<br>019<br>2	-1.2503	do<br>wn	0.00<br>362

MDH1B	malate dehydrogenase 1B [Source:HGNC Symbol;Acc:HGNC:17836]	0.71134	0.09056	2.97359	up	0.00362
cat-1	high affinity cationic amino acid transporter 1 [Source:RefSeq peptide;Acc:NP_001138962]	0.99486	0.187	2.41145	up	0.00401
LRP6	LDL receptor related protein 6 [Source:HGNC Symbol;Acc:HGNC:6698]	3.69975	7.64249	-1.04662	down	0.00401
RGS14	regulator of G-protein signaling 14 [Source:HGNC Symbol;Acc:HGNC:9996]	0.84225	1.81996	-1.11159	down	0.00401
ASAP1	ArfGAP with SH3 domain, ankyrin repeat and PH domain 1 [Source:HGNC	0.7	1.9	-1.3055	do	0.00

	Symbol;Acc:HGNC:2720]	918<br>4	573<br>1	9	wn	401
GNPAT	glyceronephosphate O-acyltransferase [Source:HGNC Symbol;Acc:HGNC:4416]	5.7<br>919	2.7<br>104<br>5	1.0955	up	0.00<br>401
TBC1D1<br>4	TBC1 domain family member 14 [Source:RefSeq peptide;Acc:NP_001239243]	2.5<br>955<br>6	5.9<br>929	-1.2072<br>1	do<br>wn	0.00<br>401
BATF	basic leucine zipper transcription factor, ATF-like [Source:HGNC Symbol;Acc:HGNC:958]	0.3<br>540<br>6	1.4<br>648<br>9	-2.0487<br>5	do<br>wn	0.00<br>401
ETV5	ets variant 5 [Source:HGNC Symbol;Acc:HGNC:3494]	1.0<br>462	0.4<br>999	1.06544	up	0.00<br>401

		6	3			
HAVCR1	hepatitis A virus cellular receptor 1 precursor [Source:RefSeq peptide;Acc:NP_001025788]	0.66253	2.93847	-2.149	down	0.00439
C2H3ORF75	elongator complex protein 6 [Source:RefSeq peptide;Acc:NP_001264419]	3.30508	1.17152	1.4963	up	0.00439
TNFRSF18	tumor necrosis factor receptor superfamily member 18 precursor [Source:RefSeq peptide;Acc:NP_001026045]	0.07545	1.56735	-4.37659	down	0.00439
PSMC3IP	PSMC3 interacting protein [Source:HGNC Symbol;Acc:HGNC:17928]	5.89285	2.90862	1.01863	up	0.00439

ISM1	isthmin 1, angiogenesis inhibitor [Source:HGNC Symbol;Acc:HGNC:16213]	0.6<br>464<br>7	0.2<br>732<br>4	1.2424	up	0.00<br>439
BBS5	Bardet-Biedl syndrome 5 protein [Source:RefSeq peptide;Acc:NP_001238960]	1.8<br>023<br>2	0.6<br>669<br>9	1.43412	up	0.00<br>439
TRIM36	tripartite motif containing 36 [Source:HGNC Symbol;Acc:HGNC:16280]	0.0<br>977<br>3	1.0<br>725<br>9	-3.4561<br>4	do<br>wn	0.00<br>439
GPR34	probable G-protein coupled receptor 34 [Source:RefSeq peptide;Acc:NP_001074355]	1.1<br>874<br>9	6.1<br>865<br>5	-2.3812<br>2	do<br>wn	0.00<br>477
XYLT1	xylosyltransferase I [Source:HGNC Symbol;Acc:HGNC:15516]	0.4	1.2	-1.6089	do	0.00

		233	913	3	wn	477
		5	1			
PGPEP1	pyroglutamyl-peptidase I [Source:HGNC Symbol;Acc:HGNC:13568]	21.	9.6	1.13373	up	0.00
		156	417			477
		3	3			
CO6	calcium-activated potassium channel subunit beta-1 [Source:RefSeq	0.2	0.9	-2.0858	do	0.00
	peptide;Acc:NP_989933]	137	072		wn	513
		2	6			
STK32A	serine/threonine-protein kinase 32A [Source:RefSeq peptide;Acc:NP_001020782]	0.2	0.7	-1.3551	do	0.00
		940	521	2	wn	513
		3	9			
ENSGAL	Uncharacterized protein [Source:UniProtKB/TrEMBL;Acc:R4GGP2]	3.0	8.1	-1.4363	do	0.00
G000000		153	602		wn	513

26769		3	4			
PLEKHO<br>1	pleckstrin homology domain-containing family O member 1 [Source:RefSeq peptide;Acc:NP_001026527]	0.5<br>783<br>5	1.6<br>447<br>7	-1.5078<br>8	do<br>wn	0.00<br>513
ARRDC2	arrestin domain containing 2 [Source:HGNC Symbol;Acc:HGNC:25225]	15.<br>737<br>2	7.6<br>999<br>2	1.03126	up	0.00<br>513
SVIP	small VCP/p97-interacting protein [Source:HGNC Symbol;Acc:HGNC:25238]	2.2<br>057<br>5	1.0<br>595<br>3	1.05785	up	0.00<br>513
ENSGAL<br>G000000<br>15461	B6.1 precursor [Source:RefSeq peptide;Acc:NP_990513]	0.1<br>158<br>8	0.7<br>483<br>9	-2.6911<br>2	do<br>wn	0.00<br>549

PHYHD1	phytanoyl-CoA dioxygenase domain containing 1 [Source:HGNC Symbol;Acc:HGNC:23396]	37.6804	11.9818	1.65297	up	0.00549
SLC25A25	solute carrier family 25 (mitochondrial carrier; phosphate carrier), member 25 [Source:HGNC Symbol;Acc:HGNC:20663]	17.9844	45.5243	-1.33989	down	0.00549
ENSGALG00000004637	NA	8.85051	17.9047	-1.01651	down	0.00549
IGF2BP3	Insulin-like growth factor 2 mRNA-binding protein 3 [Source:UniProtKB/Swiss-Prot;Acc:Q5ZLP8]	1.43398	0.29161	2.2979	up	0.00549
SLC52A3	solute carrier family 52 (riboflavin transporter), member 3 [Source:HGNC	4.4	0.9	2.2252	up	0.00

	Symbol;Acc:HGNC:16187]	040	418			549
		7	9			
NFYC	nuclear transcription factor Y subunit gamma [Source:HGNC Symbol;Acc:HGNC:7806]	20.	8.2	1.33487	up	0.00
		857	684			549
		3				
FAM46B	family with sequence similarity 46 member B [Source:HGNC Symbol;Acc:HGNC:28273]	7.0	3.3	1.0859	up	0.00
		909	405			549
		5	2			
SRF	serum response factor [Source:RefSeq peptide;Acc:NP_001239070]	2.4	6.4	-1.4038	do	0.00
		290	275	5	wn	549
		8	1			
k60	interleukin-8 precursor [Source:RefSeq peptide;Acc:NP_990349]	0.1	0.7	-2.2793	do	0.00
		517	366	2	wn	549

		6	9			
PARP8	poly(ADP-ribose) polymerase family member 8 [Source:HGNC Symbol;Acc:HGNC:26124]	0.5335	1.33054	-1.31847	down	0.00549
PSAT1	phosphoserine aminotransferase 1 [Source:HGNC Symbol;Acc:HGNC:19129]	17.3937	53.5047	-1.6211	down	0.00549
ENSGALG00000013546	Uncharacterized protein [Source:UniProtKB/TrEMBL;Acc:F1N918]	0.05298	7.13835	-7.0739	down	0.00549
RRP8	ribosomal RNA processing 8, methyltransferase, homolog (yeast) [Source:HGNC Symbol;Acc:HGNC:29030]	7.31262	3.5517	1.04188	up	0.00583

ANKRD27	ankyrin repeat domain-containing protein 27 [Source:RefSeq peptide;Acc:NP_001129150]	1.21551	2.96424	-1.2861	down	0.00583
CORO6	coronin 6 [Source:HGNC Symbol;Acc:HGNC:21356]	1.53778	0.45002	1.77278	up	0.00583
TET3	tet methylcytosine dioxygenase 3 [Source:HGNC Symbol;Acc:HGNC:28313]	1.70773	0.69745	1.29192	up	0.00583
ASAP3	ArfGAP with SH3 domain, ankyrin repeat and PH domain 3 [Source:HGNC Symbol;Acc:HGNC:14987]	5.58068	2.63273	1.08388	up	0.00583
ENSGAL	NA	0.3	1.1	-1.6155	do	0.00

G000000		797	635	8	wn	583
20796		2	9			
C5	complement component 5 [Source:HGNC Symbol;Acc:HGNC:1331]	10.	45.	-2.0572	do	0.00
		818	025	7	wn	619
		4	7			
ENSGAL	Uncharacterized protein [Source:UniProtKB/TrEMBL;Acc:E1C8F4]	2.7	0.1	4.18565	up	0.00
G000000		509	511			619
03838		1	7			
RNF150	ring finger protein 150 [Source:HGNC Symbol;Acc:HGNC:23138]	1.5	3.9	-1.3615	do	0.00
		325	379	6	wn	619
		1	7			
CTH	cystathionine gamma-lyase [Source:HGNC Symbol;Acc:HGNC:2501]	66.	31.	1.056	up	0.00
		185	832			619

		3	8			
RGMA	repulsive guidance molecule A precursor [Source:RefSeq peptide;Acc:NP_989868]	1.0<br>877<br>3	0.4<br>662<br>2	1.22223	up	0.00<br>651
CCND3	G1/S-specific cyclin-D3 [Source:RefSeq peptide;Acc:NP_001008453]	4.0<br>834<br>1	10.<br>708<br>2	-1.3908<br>7	do<br>wn	0.00<br>651
ENSGAL<br>G000000<br>23180	Uncharacterized protein [Source:UniProtKB/TrEMBL;Acc:E1C925]	50.<br>976<br>7	19.<br>543<br>8	1.38313	up	0.00<br>651
MAST4	microtubule associated serine/threonine kinase family member 4 [Source:HGNC Symbol;Acc:HGNC:19037]	0.4<br>368<br>1	1.1<br>506<br>8	-1.3973<br>9	do<br>wn	0.00<br>651

PLK2	polo-like kinase 2 [Source:HGNC Symbol;Acc:HGNC:19699]	3.0<br>790<br>8	1.0<br>564<br>4	1.54329	up	0.00<br>651
PYROX<br>D1	pyridine nucleotide-disulfide oxidoreductase domain-containing protein 1 [Source:RefSeq peptide;Acc:NP_001264205]	1.4<br>427<br>5	0.4<br>126<br>2	1.80593	up	0.00<br>687
ARHGA<br>P28	Rho GTPase activating protein 28 [Source:HGNC Symbol;Acc:HGNC:25509]	0.8<br>948<br>4	2.2<br>767<br>8	-1.3472<br>9	do<br>wn	0.00<br>687
BLK	BLK proto-oncogene, Src family tyrosine kinase [Source:HGNC Symbol;Acc:HGNC:1057]	0.4<br>559<br>3	1.0<br>652	-1.2242<br>3	do<br>wn	0.00<br>687
CCDC10	coiled-coil domain containing 109B [Source:HGNC Symbol;Acc:HGNC:26076]	1.4	4.2	-1.5411	do	0.00

9B		520<br>4	256<br>9	1	wn	687
ATG12	autophagy related 12 [Source:HGNC Symbol;Acc:HGNC:588]	1.6<br>443<br>6	3.7<br>140<br>8	-1.1754<br>8	do<br>wn	0.00<br>687
TRAIP	TRAF-interacting protein [Source:RefSeq peptide;Acc:NP_001025757]	2.6<br>792<br>5	0.1<br>649<br>5	4.02171	up	0.00<br>721
SUSD3	sushi domain containing 3 [Source:HGNC Symbol;Acc:HGNC:28391]	0.2<br>974	0.7<br>154<br>7	-1.2665<br>1	do<br>wn	0.00<br>721
CREM	Gallus gallus cAMP responsive element modulator (CREM), transcript variant 4, mRNA. [Source:RefSeq mRNA;Acc:NM_204387]	3.1<br>031	12.<br>195	-1.9745<br>6	do<br>wn	0.00<br>721

		6	7			
TYSND1	trypsin domain containing 1 [Source:HGNC Symbol;Acc:HGNC:28531]	10.<br>429<br>9	2.5<br>274<br>7	2.04496	up	0.00<br>721
EP3	prostaglandin E2 receptor EP3 subtype [Source:RefSeq peptide;Acc:NP_001035558]	0.4<br>289<br>7	1.6<br>825	-1.9716<br>5	do<br>wn	0.00<br>721
RFC4	replication factor C subunit 4 [Source:RefSeq peptide;Acc:NP_001006550]	3.1<br>635<br>2	1.4<br>166<br>4	1.15906	up	0.00<br>721
TEL	transcription factor ETV6 [Source:RefSeq peptide;Acc:NP_001186202]	3.3<br>102<br>4	7.0<br>767<br>6	-1.0961<br>5	do<br>wn	0.00<br>754

ASB9	ankyrin repeat and SOCS box protein 9 [Source:RefSeq peptide;Acc:NP_001006262]	1.9 555 6	8.9 295 5	-2.191	do wn	0.00 754
DNAJA4	DnaJ heat shock protein family (Hsp40) member A4 [Source:HGNC Symbol;Acc:HGNC:14885]	1.2 077 6	6.4 044 5	-2.4067 4	do wn	0.00 754
PLA2G1 5	phospholipase A2 group XV [Source:HGNC Symbol;Acc:HGNC:17163]	2.0 952 8	6.3 848 2	-1.6075	do wn	0.00 754
ZBTB16	zinc finger and BTB domain containing 16 [Source:HGNC Symbol;Acc:HGNC:12930]	3.7 430 2	13. 557 4	-1.8568 1	do wn	0.00 754
GPR137	integral membrane protein GPR137B [Source:RefSeq peptide;Acc:NP_001026233]	0.7	5.0	-2.8318	do	0.00

B		073	356		wn	754
			5			
ACOT11	acyl-CoA thioesterase 11 [Source:HGNC Symbol;Acc:HGNC:18156]	6.0	1.6	1.87815	up	0.00
		282	398			754
		6	8			
TSHZ1	teashirt zinc finger homeobox 1 [Source:HGNC Symbol;Acc:HGNC:10669]	0.3	0.8	-1.1659	do	0.00
		700	303	2	wn	788
		6	3			
BMP5	bone morphogenetic protein 5 precursor [Source:RefSeq peptide;Acc:NP_990479]	0.3	1.3	-1.8674	do	0.00
		758	713	6	wn	788
		2	1			
PLK3	polo-like kinase 3 [Source:HGNC Symbol;Acc:HGNC:2154]	0.4	1.0	-1.1562	do	0.00
		802	702	3	wn	788

		2	8			
ZEB1	zinc finger E-box-binding homeobox 1 [Source:RefSeq peptide;Acc:NP_990462]	2.5 685 2	5.1 830 3	-1.0128 6	do wn	0.00 822
SCFD2	sec1 family domain containing 2 [Source:HGNC Symbol;Acc:HGNC:30676]	7.1 009 6	3.1 628 1	1.16681	up	0.00 822
NDUFS8	NADH:ubiquinone oxidoreductase core subunit S8 [Source:HGNC Symbol;Acc:HGNC:7715]	24. 967 1	8.9 625 8	1.47804	up	0.00 822
Metazoa_ SRP	Metazoan signal recognition particle RNA [Source:RFAM;Acc:RF00017]	19. 299 9	1.5 815 4	3.60919	up	0.00 822

EFNA5	ephrin-A5 precursor [Source:RefSeq peptide;Acc:NP_990515]	0.2<br>808<br>4	0.6<br>945<br>5	-1.3063<br>1	do<br>wn	0.00<br>822
MTMR12	myotubularin related protein 12 [Source:HGNC Symbol;Acc:HGNC:18191]	1.2<br>112	2.5<br>160<br>4	-1.0547<br>2	do<br>wn	0.00<br>822
CAMK4	calcium/calmodulin-dependent protein kinase IV [Source:HGNC Symbol;Acc:HGNC:1464]	0.8<br>581	0.1<br>965<br>8	2.12606	up	0.00<br>822
MGST2	microsomal glutathione S-transferase 2 [Source:HGNC Symbol;Acc:HGNC:7063]	0.9<br>112<br>8	3.7<br>844<br>6	-2.0541<br>2	do<br>wn	0.00<br>856
DNA2	DNA replication helicase/nuclease 2 [Source:HGNC Symbol;Acc:HGNC:2939]	2.1	0.0	4.49697	up	0.00

		194	938<br>6			856
ST6GAL<br>1	beta-galactoside alpha-2,6-sialyltransferase 1 [Source:RefSeq peptide;Acc:NP_990572]	14.<br>597<br>9	46.<br>485<br>2	-1.6710<br>1	do<br>wn	0.00<br>856
CD247	T-cell surface glycoprotein CD3 zeta chain precursor [Source:RefSeq peptide;Acc:NP_996762]	0.1<br>243<br>1	4.8<br>025<br>4	-5.2718<br>2	do<br>wn	0.00<br>888
SLC38A2	sodium-coupled neutral amino acid transporter 2 [Source:RefSeq peptide;Acc:NP_001025912]	26.<br>183<br>3	56.<br>786<br>3	-1.1169	do<br>wn	0.00<br>888
MLXIP	MLX interacting protein [Source:HGNC Symbol;Acc:HGNC:17055]	0.2<br>307	0.8<br>645	-1.9058<br>2	do<br>wn	0.00<br>888

		1	3			
ENSGALG00000013033	Uncharacterized protein [Source:UniProtKB/TrEMBL;Acc:E1BXS8]	7.74709	52.4021	-2.7579	down	0.00888
CYR61	Protein CYR61 [Source:UniProtKB/Swiss-Prot;Acc:P19336]	3.2153	7.53647	-1.22893	down	0.00888
SLU7	pre-mRNA-splicing factor SLU7 [Source:RefSeq peptide;Acc:NP_001006146]	2.28036	6.40823	-1.49066	down	0.00918
SRRM1	serine/arginine repetitive matrix protein 1 [Source:RefSeq peptide;Acc:NP_001026665]	10.4252	4.82561	1.11129	up	0.00918

PSIP1	lens epithelium-derived growth factor [Source:RefSeq peptide;Acc:NP_001026781]	4.0<br>694<br>8	1.9<br>311<br>7	1.07537	up	0.00<br>918
IFNAR2	interferon alpha/beta receptor 2 precursor [Source:RefSeq peptide;Acc:NP_990189]	4.6<br>865<br>9	10.<br>736<br>6	-1.1959<br>3	do<br>wn	0.00<br>949
TREM-B<br>1	Triggering receptor expressed on myeloid cells [Source:UniProtKB/TrEMBL;Acc:Q2YHU3]	0.1<br>243<br>5	0.5<br>594<br>7	-2.1696<br>4	do<br>wn	0.00<br>949
RAD54L	RAD54-like (S. cerevisiae) [Source:HGNC Symbol;Acc:HGNC:9826]	1.8<br>146<br>5	0.4<br>376<br>7	2.05177	up	0.00<br>949
ENSGAL	Uncharacterized protein [Source:UniProtKB/TrEMBL;Acc:R4GKS8]	1.4	3.6	-1.3060	do	0.00

G000000		729	419	5	wn	949
27722			5			
ENSGAL	Uncharacterized protein [Source:UniProtKB/TrEMBL;Acc:F1NXQ0]	0.3	4.7	-3.5898	do	0.00
G000000		973	838	5	wn	983
15888						
MITF	microphthalmia-associated transcription factor [Source:RefSeq peptide;Acc:NP_990360]	0.6	2.2	-1.7243	do	0.00
		868	695	5	wn	983
		4	5			
VANGL1	VANGL planar cell polarity protein 1 [Source:HGNC Symbol;Acc:HGNC:15512]	1.8	0.2	2.86653	up	0.01
		281	506			015
		2	6			
CD3D	T-cell surface glycoprotein CD3 delta chain precursor [Source:RefSeq	0.8	35.	-5.3438	do	0.01
	peptide;Acc:NP_990843]	720	416	5	wn	015

		6	3			
BAHCC1	BAH domain and coiled-coil containing 1 [Source:HGNC Symbol;Acc:HGNC:29279]	2.76552	1.20607	1.19724	up	0.01047
EME1	essential meiotic structure-specific endonuclease 1 [Source:HGNC Symbol;Acc:HGNC:24965]	2.24076	0.69043	1.69842	up	0.01047
CARD8	caspase recruitment domain family member 8 [Source:HGNC Symbol;Acc:HGNC:17057]	0.89025	2.53242	-1.50824	down	0.01077
ATPAF2	ATP synthase mitochondrial F1 complex assembly factor 2 [Source:HGNC Symbol;Acc:HGNC:18802]	8.21493	4.02192	1.03036	up	0.0111

BYSL	bystin like [Source:HGNC Symbol;Acc:HGNC:1157]	18.<br>144<br>4	8.8<br>560<br>1	1.0348	up	0.01<br>11
GZMM	granzyme M [Source:HGNC Symbol;Acc:HGNC:4712]	0.1<br>207<br>3	6.9<br>547<br>7	-5.8481<br>8	do<br>wn	0.01<br>11
ST8SIA1	alpha-N-acetylneuraminide alpha-2,8-sialyltransferase [Source:RefSeq peptide;Acc:NP_990339]	0.2<br>358<br>3	0.9<br>006<br>9	-1.9332<br>6	do<br>wn	0.01<br>142
LONRF1	LON peptidase N-terminal domain and ring finger 1 [Source:HGNC Symbol;Acc:HGNC:26302]	0.4<br>369<br>9	1.6<br>575<br>7	-1.9234	do<br>wn	0.01<br>142
GPD2	glycerol-3-phosphate dehydrogenase 2 [Source:HGNC Symbol;Acc:HGNC:4456]	0.8	2.0	-1.2428	do	0.01

		628	420		wn	142
		8	5			
ICOS	inducible T-cell costimulator precursor [Source:RefSeq peptide;Acc:NP_001093758]	0.1	1.9	-3.6689	do	0.01
		510	215	7	wn	142
		7	9			
ENSGAL	Uncharacterized protein [Source:UniProtKB/TrEMBL;Acc:E1BR15]	0.3	5.6	-4.0593	do	0.01
G000000		412	898	4	wn	174
02102		9	8			
ENSGAL	NA	5.6	2.5	1.12579	up	0.01
G000000		566	921			174
12954		7	8			
IL22RA1	interleukin 22 receptor subunit alpha 1 [Source:HGNC Symbol;Acc:HGNC:13700]	0.7	0.3	1.27047	up	0.01
		565	136			174

		9	3			
OBFC1	oligonucleotide/oligosaccharide-binding fold containing 1 [Source:HGNC Symbol;Acc:HGNC:26200]	0.5<br>888	1.3<br>533<br>2	-1.2006<br>6	do<br>wn	0.01<br>174
ATP5A1	ATP synthase subunit alpha [Source:UniProtKB/TrEMBL;Acc:F1NI22]	109<br>.85<br>7	37.<br>117<br>4	1.56546	up	0.01<br>174
ENSGAL<br>G000000<br>19061	Uncharacterized protein [Source:UniProtKB/TrEMBL;Acc:E1C9D9]	0.0<br>504<br>1	11.<br>428<br>4	-7.8247<br>9	do<br>wn	0.01<br>201
CPSF4	cleavage and polyadenylation specificity factor subunit 4 [Source:RefSeq peptide;Acc:NP_001264480]	14.<br>088<br>2	6.2<br>058<br>1	1.18279	up	0.01<br>229

chTC10	rho-related GTP-binding protein RhoQ [Source:RefSeq peptide;Acc:NP_989792]	4.9<br>941<br>7	1.9<br>135<br>9	1.38396	up	0.01<br>229
TSSC4	protein TSSC4 [Source:RefSeq peptide;Acc:NP_001006470]	6.2<br>943<br>2	3.1<br>041<br>3	1.01986	up	0.01<br>229
EPHA3	ephrin type-A receptor 3 precursor [Source:RefSeq peptide;Acc:NP_990761]	0.8<br>108<br>9	0.1<br>565<br>5	2.37291	up	0.01<br>259
RCBTB1	regulator of chromosome condensation (RCC1) and BTB (POZ) domain containing protein 1 [Source:HGNC Symbol;Acc:HGNC:18243]	4.0<br>094<br>7	1.4<br>644<br>6	1.45304	up	0.01<br>287
CREM	Gallus gallus cAMP responsive element modulator (CREM), transcript variant 4, mRNA.	0.1	16.	-7.3577	do	0.01

	[Source:RefSeq mRNA;Acc:NM_204387]	028<br>3	866	9	wn	287
GUCA2B	guanylate cyclase activator 2A (guanylin) precursor [Source:RefSeq peptide;Acc:NP_001183967]	0.8<br>087<br>7	1.7<br>489<br>9	-1.1127<br>2	do<br>wn	0.01<br>287
ARHGEF<br>26	Rho guanine nucleotide exchange factor 26 [Source:HGNC Symbol;Acc:HGNC:24490]	4.2<br>705	1.1<br>298<br>1	1.91832	up	0.01<br>287
LY75	lymphocyte antigen 75 precursor [Source:RefSeq peptide;Acc:NP_001032925]	2.5<br>049<br>3	13.<br>524<br>1	-2.4326<br>9	do<br>wn	0.01<br>316
LMOD1	leiomodin 1 (smooth muscle) [Source:HGNC Symbol;Acc:HGNC:6647]	0.4<br>635	1.0<br>907	-1.2346<br>5	do<br>wn	0.01<br>345

		2	8			
CENPK	Centromere protein K [Source:UniProtKB/Swiss-Prot;Acc:Q1T7C1]	3.7	0.3	3.3956	up	0.01
		241	538			345
		3	7			
DAP3	death associated protein 3 [Source:HGNC Symbol;Acc:HGNC:2673]	31.	10.	1.56911	up	0.01
		595	648			375
		4	2			
TMEM56	transmembrane protein 56 [Source:RefSeq peptide;Acc:NP_001264486]	3.8	0.4	3.01442	up	0.01
		548	770			375
		9	7			
BCOR	BCL6 corepressor [Source:HGNC Symbol;Acc:HGNC:20893]	2.0	0.8	1.34898	up	0.01
		713	131			436
		8	6			

ENSGAL G000000 04798	NA	4.0 264 5	1.6 744 7	1.2658	up	0.01 436
RIC3	RIC3 acetylcholine receptor chaperone [Source:HGNC Symbol;Acc:HGNC:30338]	0.6 147 1	0.2 112 5	1.54095	up	0.01 436
AURKA	aurora kinase A [Source:HGNC Symbol;Acc:HGNC:11393]	9.2 709 4	0.9 398 3	3.30224	up	0.01 464
ENSGAL G000000 16731	Uncharacterized protein [Source:UniProtKB/TrEMBL;Acc:F1NEW6]	2.8 443 3	1.2 919 8	1.13851	up	0.01 464
MSN	moesin [Source:HGNC Symbol;Acc:HGNC:7373]	11.	30.	-1.3895	do	0.01

		568	308		wn	464
		6	4			
COTL1	coactosin-like protein [Source:RefSeq peptide;Acc:NP_001159797]	9.1	25.	-1.4793	do	0.01
		247	441	3	wn	493
		9	7			
MTBP	MDM2 binding protein [Source:HGNC Symbol;Acc:HGNC:7417]	1.2	0.4	1.62808	up	0.01
		779	134			493
		9	5			
SBNO2	strawberry notch homolog 2 (Drosophila) [Source:HGNC Symbol;Acc:HGNC:29158]	0.8	2.7	-1.6229	do	0.01
		824	180	8	wn	493
		6	9			
C19orf25	chromosome 19 open reading frame 25 [Source:HGNC Symbol;Acc:HGNC:26711]	11.	3.9	1.54056	up	0.01
		623	957			521

		9	4			
ENSGAL	Uncharacterized protein [Source:UniProtKB/TrEMBL;Acc:E1BTB4]	0.2	2.1	-3.0639	do	0.01
G000000		607	803	3	wn	521
16572		3	2			
LIMD2	LIM domain-containing protein 2 [Source:RefSeq peptide;Acc:NP_001006330]	0.5	5.2	-3.3352	do	0.01
		190	385		wn	549
		6	1			
FANCL	E3 ubiquitin-protein ligase FANCL [Source:RefSeq peptide;Acc:NP_001029113]	7.1	2.3	1.61287	up	0.01
		357	330			549
		4	2			
FAM134	family with sequence similarity 134 member A [Source:HGNC Symbol;Acc:HGNC:28450]	10.	22.	-1.0821	do	0.01
A		846	965	9	wn	549
		6				

WSCD2	WSC domain containing 2 [Source:HGNC Symbol;Acc:HGNC:29117]	0.1<br>093<br>3	0.8<br>518<br>6	-2.9619<br>7	do<br>wn	0.01<br>577
TMEM21<br>4	transmembrane protein 214 [Source:HGNC Symbol;Acc:HGNC:25983]	7.5<br>402<br>2	2.0<br>607<br>5	1.87143	up	0.01<br>577
TMEM68	transmembrane protein 68 [Source:RefSeq peptide;Acc:NP_001026192]	1.3<br>938<br>6	2.8<br>823<br>7	-1.0481<br>7	do<br>wn	0.01<br>604
DGKH	diacylglycerol kinase, eta [Source:HGNC Symbol;Acc:HGNC:2854]	0.2<br>472<br>2	0.7<br>042<br>1	-1.5101<br>8	do<br>wn	0.01<br>631
CDC45	cell division cycle 45 [Source:HGNC Symbol;Acc:HGNC:1739]	5.2	0.2	4.51392	up	0.01

		385	292			631
		2	9			
ENSGAL	Uncharacterized protein [Source:UniProtKB/TrEMBL;Acc:F1NY84]	0.7	2.3	-1.5328	do	0.01
G000000		964	046	4	wn	631
08598		8	6			
COQ10B	coenzyme Q10B [Source:HGNC Symbol;Acc:HGNC:25819]	13.	96.	-2.8033	do	0.01
		813	428	5	wn	631
		8	3			
THRSP	thyroid hormone responsive [Source:RefSeq peptide;Acc:NP_998742]	4.9	1.5	1.69973	up	0.01
		597	268			687
		9	4			
FOXN4	forkhead box protein N4 [Source:RefSeq peptide;Acc:NP_001076828]	0.0	1.3	-4.2807	do	0.01
		714	879	5	wn	715

		1	6			
DEPDC1<br>B	DEP domain-containing protein 1B [Source:RefSeq peptide;Acc:NP_001006576]	0.9<br>964<br>3	0.1<br>083<br>9	3.20051	up	0.01<br>715
CENPT	Centromere protein T [Source:UniProtKB/Swiss-Prot;Acc:F1NPG5]	2.2<br>578<br>7	0.9<br>643<br>3	1.22737	up	0.01<br>741
FABP3	fatty acid-binding protein, heart [Source:RefSeq peptide;Acc:NP_001026060]	0.7<br>115<br>8	2.7<br>212<br>4	-1.9351<br>6	do<br>wn	0.01<br>741
DHRS7	dehydrogenase/reductase (SDR family) member 7 [Source:HGNC Symbol;Acc:HGNC:21524]	237<br>.85<br>9	100<br>.32<br>8	1.24539	up	0.01<br>741

ENSGAL	Uncharacterized protein [Source:UniProtKB/TrEMBL;Acc:F1N9B0]	13.	5.6	1.24954	up	0.01
G000000		538	941			767
21450		7	3			
ENSGAL	Uncharacterized protein [Source:UniProtKB/TrEMBL;Acc:H9L3V2]	0.3	3.2	-3.2172	do	0.01
G000000		472	298	7	wn	767
26466		9	5			
FABP4	fatty acid-binding protein, adipocyte [Source:RefSeq peptide;Acc:NP_989621]	0.7	2.9	-1.8901	do	0.01
		848	093	9	wn	767
		6	3			
GIF	gastric intrinsic factor [Source:HGNC Symbol;Acc:HGNC:4268]	1.2	36.	-4.8859	do	0.01
		233	170	5	wn	767
		2	7			
INCENP	inner centromere protein [Source:RefSeq peptide;Acc:NP_990661]	2.9	0.4	2.67475	up	0.01

		835	6725			767
LITAF	lipopolysaccharide-induced tumor necrosis factor-alpha factor homolog [Source:RefSeq peptide;Acc:NP_989598]	0.2 137 1	4.5 525	-4.4129 1	do wn	0.01 824
MYBBP1 A	MYB binding protein (P160) 1a [Source:HGNC Symbol;Acc:HGNC:7546]	3.5 642 5	1.3 952 9	1.35304	up	0.01 824
INCENP	inner centromere protein [Source:RefSeq peptide;Acc:NP_990661]	2.4 559 6	0.9 285 8	1.40318	up	0.01 852
MRM1	mitochondrial rRNA methyltransferase 1 [Source:HGNC Symbol;Acc:HGNC:26202]	5.0 863	2.3 794	1.09602	up	0.01 88

		8	3			
LSM1	LSM1 homolog, mRNA degradation associated [Source:HGNC Symbol;Acc:HGNC:20472]	16.<br>006<br>5	7.9<br>808<br>1	1.00405	up	0.01<br>933
RRAS2	ras-related protein R-Ras2 [Source:RefSeq peptide;Acc:NP_001006466]	0.2<br>303<br>4	0.9<br>907<br>4	-2.1047<br>4	do<br>wn	0.01<br>933
ITPA	Inosine triphosphate pyrophosphatase [Source:UniProtKB/Swiss-Prot;Acc:F1NLH9]	10.<br>328	2.8<br>696<br>1	1.84763	up	0.01<br>959
CH25H	cholesterol 25-hydroxylase [Source:RefSeq peptide;Acc:NP_001264283]	0.1<br>076<br>7	1.4<br>165<br>9	-3.7178	do<br>wn	0.01<br>986

PCSK9	proprotein convertase subtilisin/kexin type 9 [Source:HGNC Symbol;Acc:HGNC:20001]	1.1 837 1	2.3 753 7	-1.0048 3	do wn	0.01 986
PHKG1	phosphorylase b kinase gamma catalytic chain, skeletal muscle isoform [Source:RefSeq peptide;Acc:NP_001006217]	26. 425 7	8.2 170 7	1.68524	up	0.02 008
KIF23	kinesin-like protein KIF23 [Source:RefSeq peptide;Acc:NP_001025725]	1.3 899 6	0.0 707 3	4.29662	up	0.02 033
AGPAT2	1-acylglycerol-3-phosphate O-acyltransferase 2 [Source:HGNC Symbol;Acc:HGNC:325]	20. 291 1	4.8 210 3	2.07344	up	0.02 033
HNRNPA	heterogeneous nuclear ribonucleoprotein A3 [Source:HGNC Symbol;Acc:HGNC:24941]	18.	7.3	1.30314	up	0.02

3		021<br>8	032<br>2			033
FOSL2	Gallus gallus FOS-like antigen 2 (FOSL2), mRNA. [Source:RefSeq mRNA;Acc:NM_001045836]	0.1<br>888<br>6	1.1<br>891<br>5	-2.6545<br>1	do<br>wn	0.02<br>054
RRP12	RRP12-like protein [Source:RefSeq peptide;Acc:NP_001012926]	13.<br>187<br>6	3.8<br>767<br>5	1.76626	up	0.02<br>081
F2RL1	proteinase-activated receptor 2 precursor [Source:RefSeq peptide;Acc:NP_001012626]	1.2<br>382<br>1	2.8<br>204<br>2	-1.1876<br>5	do<br>wn	0.02<br>081
NOB1	NIN1/RPN12 binding protein 1 homolog [Source:HGNC Symbol;Acc:HGNC:29540]	15.<br>418	6.9<br>827	1.14281	up	0.02<br>108

		6				
GRAMD3	GRAM domain containing 3 [Source:HGNC Symbol;Acc:HGNC:24911]	1.25581	2.76078	-1.13646	down	0.02108
MMP23B	matrix metallopeptidase 23B [Source:HGNC Symbol;Acc:HGNC:7171]	0.71585	2.00281	-1.48429	down	0.02133
CD40LG	CD40 ligand CD40 ligand, membrane form CD40 ligand, soluble form [Source:UniProtKB/Swiss-Prot;Acc:Q9I8D8]	0.08224	1.63116	-4.30986	down	0.02133
SP5	Sp5 transcription factor [Source:HGNC Symbol;Acc:HGNC:14529]	1.26875	0.4059	1.64421	up	0.02133

CD274	CD274 molecule [Source:HGNC Symbol;Acc:HGNC:17635]	0.33594	2.97411	-3.14618	down	0.02133
MCM4	minichromosome maintenance complex component 4 [Source:HGNC Symbol;Acc:HGNC:6947]	9.09092	1.19569	2.92658	up	0.02158
C14orf80	chromosome 14 open reading frame 80 [Source:HGNC Symbol;Acc:HGNC:20127]	1.18922	0.48198	1.30299	up	0.02158
SLC4A7	solute carrier family 4, sodium bicarbonate cotransporter, member 7 [Source:HGNC Symbol;Acc:HGNC:11033]	0.57302	1.43352	-1.3229	down	0.02214
MYH9	myosin-9 [Source:RefSeq peptide;Acc:NP_990808]	16.	52.	-1.6474	do	0.02

		713<br>5	360<br>2	6	wn	242
SLC6A6	sodium- and chloride-dependent taurine transporter [Source:RefSeq peptide;Acc:NP_001025771]	84.<br>360<br>5	41.<br>727<br>1	1.01558	up	0.02<br>268
ELOVL2	elongation of very long chain fatty acids protein 2 [Source:RefSeq peptide;Acc:NP_001184237]	9.5<br>655<br>9	1.2<br>029	2.99134	up	0.02<br>268
DEGS2	delta(4)-desaturase, sphingolipid 2 [Source:HGNC Symbol;Acc:HGNC:20113]	0.3<br>095<br>9	0.8<br>054<br>1	-1.3793<br>5	do<br>wn	0.02<br>318
GZMA	granzyme A precursor [Source:RefSeq peptide;Acc:NP_989788]	0.0<br>578	7.6<br>959	-7.0569<br>1	do<br>wn	0.02<br>318

			4			
CORO1C	coronin-1C isoform 2 [Source:RefSeq peptide;Acc:NP_001264798]	8.0<br>518<br>7	19.<br>732<br>2	-1.2931<br>6	do<br>wn	0.02<br>344
RHOBTB<br>2	Rho-related BTB domain containing 2 [Source:HGNC Symbol;Acc:HGNC:18756]	1.2<br>630<br>8	0.4<br>847<br>4	1.38168	up	0.02<br>369
DMXL1	Dmx-like 1 [Source:HGNC Symbol;Acc:HGNC:2937]	2.3<br>295<br>8	4.7<br>511<br>6	-1.0282<br>1	do<br>wn	0.02<br>393
P2X1	P2X purinoceptor 1 [Source:RefSeq peptide;Acc:NP_989850]	1.5<br>385<br>9	0.7<br>556	1.02592	up	0.02<br>42

B4GALT3	UDP-Gal:betaGlcNAc beta 1,4- galactosyltransferase, polypeptide 3 [Source:HGNC Symbol;Acc:HGNC:926]	0.87104	2.75768	-1.66265	down	0.02569
SESN1	sestrin 1 [Source:HGNC Symbol;Acc:HGNC:21595]	36.9838	81.188	-1.13437	down	0.02569
GFPT2	glutamine-fructose-6-phosphate transaminase 2 [Source:HGNC Symbol;Acc:HGNC:4242]	0.25266	0.64718	-1.35698	down	0.02593
ENSGALG00000029149	Uncharacterized protein [Source:UniProtKB/TrEMBL;Acc:R4GJK2]	2.21229	0.65407	1.75802	up	0.02593
IRF6	interferon regulatory factor 6 [Source:HGNC Symbol;Acc:HGNC:6121]	3.2	6.6	-1.0307	do	0.02

		713<br>8	836<br>1	3	wn	618
IFT46	intraflagellar transport 46 [Source:HGNC Symbol;Acc:HGNC:26146]	0.9<br>387<br>6	0.4<br>263<br>3	1.13877	up	0.02<br>64
MICALL<br>1	MICAL-like protein 1 [Source:RefSeq peptide;Acc:NP_001188377]	0.8<br>105<br>8	2.0<br>858<br>5	-1.3636	do<br>wn	0.02<br>71
SERPIN<br>B10	Heterochromatin-associated protein MENT [Source:UniProtKB/Swiss-Prot;Acc:O73790]	0.1<br>540<br>7	1.7<br>103	-3.4726<br>3	do<br>wn	0.02<br>71
MBNL3	muscleblind-like 3 isoform 5 [Source:RefSeq peptide;Acc:NP_001156813]	0.2<br>410	1.4<br>407	-2.5796<br>2	do<br>wn	0.02<br>71

		2	9			
C21orf62	chromosome 21 open reading frame 62 [Source:HGNC Symbol;Acc:HGNC:1305]	0.5<br>560<br>8	14.<br>531<br>2	-4.7077<br>2	do<br>wn	0.02<br>804
MCM6	DNA replication licensing factor MCM6 [Source:RefSeq peptide;Acc:NP_001006527]	0.8<br>312<br>5	0.1<br>574<br>2	2.40065	up	0.02<br>85
STOML3	stomatin like 3 [Source:HGNC Symbol;Acc:HGNC:19420]	0.0<br>664<br>9	1.2<br>416<br>7	-4.223	do<br>wn	0.02<br>95
CCL1	chemokine (C-C motif) ligand 1 precursor [Source:RefSeq peptide;Acc:NP_990107]	0.1<br>872<br>7	9.2<br>215<br>1	-5.6218<br>3	do<br>wn	0.02<br>95

CD72	CD72 antigen [Source:RefSeq peptide;Acc:NP_001026617]	0.051	0.85675	-4.07018	down	0.02975
SDC4	Syndecan-4 [Source:UniProtKB/Swiss-Prot;Acc:P49416]	3.94342	8.58429	-1.12225	down	0.03
IL21R	interleukin-21 receptor precursor [Source:RefSeq peptide;Acc:NP_001025811]	0.02017	1.4638	-6.18103	down	0.03027
FAT4	FAT atypical cadherin 4 [Source:HGNC Symbol;Acc:HGNC:23109]	0.53209	1.55239	-1.54475	down	0.03027
PLBD1	phospholipase B domain containing 1 [Source:HGNC Symbol;Acc:HGNC:26215]	0.3	3.7	-3.3420	do	0.03

		650<br>1	012<br>7	3	wn	075
TMEM30<br>C	transmembrane protein 30C [Source:HGNC Symbol;Acc:HGNC:30443]	5.0<br>301<br>6	2.2<br>591<br>1	1.15485	up	0.03<br>095
HNF4bet<br>a	hepatic nuclear factor 4beta [Source:RefSeq peptide;Acc:NP_001025747]	17.<br>306<br>4	3.8<br>831<br>2	2.15602	up	0.03<br>095
SYK	Tyrosine-protein kinase SYK [Source:UniProtKB/Swiss-Prot;Acc:F1N9Y5]	0.1<br>230<br>9	1.2<br>269	-3.3172<br>3	do<br>wn	0.03<br>095
INSIG1	insulin-induced gene 1 protein [Source:RefSeq peptide;Acc:NP_001026137]	3.2<br>546	18.<br>742	-2.5257<br>6	do<br>wn	0.03<br>117

		7	8			
ARHGEF<br>10	Rho guanine nucleotide exchange factor 10 [Source:HGNC Symbol;Acc:HGNC:14103]	1.2<br>323<br>6	0.4<br>039<br>7	1.60909	up	0.03<br>117
ENSGAL<br>G000000<br>23936	Uncharacterized protein [Source:UniProtKB/TrEMBL;Acc:E1BUD0]	7.2<br>027<br>5	21.<br>354<br>3	-1.5679<br>1	do<br>wn	0.03<br>16
DTL	Denticleless protein homolog [Source:UniProtKB/Swiss-Prot;Acc:Q5ZJW8]	1.8<br>866<br>3	0.2<br>102<br>6	3.16559	up	0.03<br>16
THOC1	THO complex 1 [Source:HGNC Symbol;Acc:HGNC:19070]	1.8<br>751<br>7	4.8<br>174<br>5	-1.3612<br>5	do<br>wn	0.03<br>184

CHPT1	Cholinephosphotransferase 1 [Source:UniProtKB/Swiss-Prot;Acc:Q5ZHQ5]	0.2 879 2	1.4 233 1	-2.3055 2	do wn	0.03 205
VPS29	Gallus gallus vacuolar protein sorting 29 homolog (S. cerevisiae) (VPS29), mRNA. [Source:RefSeq mRNA;Acc:NM_001007837]	12. 602 5	6.1 770 7	1.02871	up	0.03 205
FSHR	follicle-stimulating hormone receptor precursor [Source:RefSeq peptide;Acc:NP_990410]	0.6 056 6	1.3 391 9	-1.1447 9	do wn	0.03 205
SPAST	spastin [Source:HGNC Symbol;Acc:HGNC:11233]	0.6 045 3	1.4 870 1	-1.2985 3	do wn	0.03 205
P2RY4	pyrimidinergic receptor P2Y, G-protein coupled, 4 [Source:HGNC Symbol;Acc:HGNC:8542]	26.	10.	1.30244	up	0.03

		087	577			229
		7				
RHBG	Rhesus blood group, B glycoprotein [Source:RefSeq peptide;Acc:NP_989698]	1.1	2.5	-1.1863	do	0.03
		212	517	3	wn	276
		8	3			
USP43	ubiquitin specific peptidase 43 [Source:HGNC Symbol;Acc:HGNC:20072]	0.8	0.3	1.2797	up	0.03
		233	391			297
		8	4			
RNASET	ribonuclease T2 precursor [Source:RefSeq peptide;Acc:NP_001034580]	1.4	3.4	-1.1952	do	0.03
2		971	282	6	wn	297
		3	2			
Lef-1	lymphoid enhancer-binding factor 1 [Source:RefSeq peptide;Acc:NP_990344]	1.5	0.1	3.79641	up	0.03
		680	128			297

		8	6			
KIAA195	KIAA1958 [Source:HGNC Symbol;Acc:HGNC:23427]	5.7	15.	-1.4538	do	0.03
8		401	724	8	wn	297
		1	7			
HMGCS1	hydroxymethylglutaryl-CoA synthase, cytoplasmic [Source:RefSeq peptide;Acc:NP_990742]	107	470	-2.1341	do	0.03
		.07	.00	2	wn	341
		1	7			
ARG2	arginase, type II [Source:RefSeq peptide;Acc:NP_001186633]	4.1	0.4	3.14886	up	0.03
		591	689			363
		5	3			
ENSGAL	Uncharacterized protein [Source:UniProtKB/TrEMBL;Acc:F1N9B4]	10.	23.	-1.2006	do	0.03
G000000		426	964	6	wn	382
08040		3	2			

HEMGN	hemogen [Source:HGNC Symbol;Acc:HGNC:17509]	1.4<br>444	0.5<br>670<br>8	1.34885	up	0.03<br>406
ENSGAL<br>G000000<br>28247	NA	2.0<br>948<br>8	0.6<br>143<br>3	1.76978	up	0.03<br>427
S100A4	S100 calcium binding protein A4 [Source:HGNC Symbol;Acc:HGNC:10494]	0.5<br>819<br>2	2.1<br>982<br>3	-1.9174<br>6	do<br>wn	0.03<br>497
LCAT	lecithin-cholesterol acyltransferase [Source:HGNC Symbol;Acc:HGNC:6522]	32.<br>397<br>3	9.2<br>076<br>3	1.81497	up	0.03<br>566
CRIM1	cysteine-rich motor neuron 1 protein precursor [Source:RefSeq peptide;Acc:NP_989756]	2.2	4.6	-1.0213	do	0.03

		8855	4527	3	wn	566
ACKR1	atypical chemokine receptor 1 (Duffy blood group) [Source:HGNC Symbol;Acc:HGNC:4035]	0.93985	2.0167	-1.1015	down	0.03632
CEP55	centrosomal protein 55kDa [Source:HGNC Symbol;Acc:HGNC:1161]	0.85491	0.07178	3.57415	up	0.03632
DNAJC6	putative tyrosine-protein phosphatase auxilin [Source:RefSeq peptide;Acc:NP_001170886]	2.86041	0.16124	4.14893	up	0.03632
PASK	PAS domain containing serine/threonine kinase [Source:HGNC Symbol;Acc:HGNC:17270]	1.0954	0.1616	2.76063	up	0.03727

		9	5			
GIPR	glucagon receptor precursor [Source:RefSeq peptide;Acc:NP_001094505]	108	44.	1.27747	up	0.03
		.07	582			815
		4	7			
COL4A6	collagen, type IV, alpha 6 [Source:HGNC Symbol;Acc:HGNC:2208]	0.8	0.2	1.59731	up	0.03
		683	869			858
		3	8			
ZC3HC1	nuclear-interacting partner of ALK [Source:RefSeq peptide;Acc:NP_001186366]	2.2	0.7	1.66843	up	0.03
		704	142			899
		4	7			
SLC22A1	solute carrier family 22 (organic cation/carnitine transporter), member 16 [Source:HGNC	0.8	0.0	3.50449	up	0.03
6	Symbol;Acc:HGNC:20302]	098	713			899
		7	6			

ENSGAL G000000 23265	Uncharacterized protein [Source:UniProtKB/TrEMBL;Acc:F1P1P8]	1.1 680 2	0.1 393 8	3.06694	up	0.03 918
ENSGAL G000000 07814	Alkaline phosphatase [Source:UniProtKB/TrEMBL;Acc:E1C0U5]	1.0 596 2	0.3 767 2	1.49199	up	0.03 937
ARL11	ADP ribosylation factor like GTPase 11 [Source:HGNC Symbol;Acc:HGNC:24046]	0.4 749 8	1.0 607 2	-1.1591	do wn	0.03 983
TXNDC9	thioredoxin domain containing 9 [Source:HGNC Symbol;Acc:HGNC:24110]	0.8 616 8	2.2 737 2	-1.3998 4	do wn	0.03 983
MPZL3	myelin protein zero-like protein 3 [Source:RefSeq peptide;Acc:NP_001239214]	0.3	0.7	-1.0903	do	0.04

		595<br>5	655<br>8	5	wn	024
GANC	neutral alpha-glucosidase C [Source:RefSeq peptide;Acc:NP_001186594]	6.3<br>831<br>2	2.7<br>181<br>6	1.23163	up	0.04<br>024
myosin	myosin heavy chain, cardiac muscle isoform [Source:RefSeq peptide;Acc:NP_990097]	1.4<br>714<br>2	0.2<br>638	2.47969	up	0.04<br>071
MASTL	microtubule associated serine/threonine kinase like [Source:HGNC Symbol;Acc:HGNC:19042]	1.5<br>374<br>8	0.1<br>957<br>7	2.9733	up	0.04<br>094
RBFOX2	RNA binding protein, fox-1 homolog (C. elegans) 2 [Source:HGNC Symbol;Acc:HGNC:9906]	3.6<br>821	0.7<br>384	2.31801	up	0.04<br>112

		1	2			
ENSGALG00000028246	Uncharacterized protein [Source:UniProtKB/TrEMBL;Acc:R4GLK9]	30.0457	96.7563	-1.68719	down	0.0413
RAD51	DNA repair protein RAD51 homolog 1 [Source:UniProtKB/Swiss-Prot;Acc:P37383]	2.49369	0.36576	2.76931	up	0.0413
OPRM1	opioid receptor, mu 1 [Source:HGNC Symbol;Acc:HGNC:8156]	0.6977	0.16875	2.04776	up	0.04153
a	Alpha-1,3-mannosyl-glycoprotein 4-beta-N-acetylglucosaminyltransferase A [Source:UniProtKB/Swiss-Prot;Acc:Q5F407]	0.44167	3.68384	-3.06016	down	0.04173

BRCA2	breast cancer 2, early onset [Source:RefSeq peptide;Acc:NP_989607]	4.8 898 1	1.8 293 5	1.41844	up	0.04 173
HABP2	hyaluronan-binding protein 2 preproprotein [Source:RefSeq peptide;Acc:NP_001264308]	17. 228 6	46. 609	-1.4358	do wn	0.04 173
TLE4	transducin-like enhancer protein 4 [Source:RefSeq peptide;Acc:NP_989568]	0.9 559 1	2.0 474 4	-1.0988 8	do wn	0.04 173
TMEM37	transmembrane protein 37 [Source:HGNC Symbol;Acc:HGNC:18216]	3.4 748 5	7.1 065 5	-1.0322	do wn	0.04 214
CD8A	Gallus gallus CD8a molecule (CD8A), mRNA. [Source:RefSeq mRNA;Acc:NM_205235]	0.0	2.6	-6.4357	do	0.04

		309<br>4	783<br>2	3	wn	27
SEMA3A	semaphorin-3A [Source:RefSeq peptide;Acc:NP_990308]	0.7<br>159<br>3	0.3<br>098<br>1	1.20846	up	0.04<br>286
CHPT1	Cholinephosphotransferase 1 [Source:UniProtKB/Swiss-Prot;Acc:Q5ZHQ5]	0.7<br>267<br>8	2.6<br>445<br>7	-1.8634<br>3	do<br>wn	0.04<br>286
C9orf91	chromosome 9 open reading frame 91 [Source:HGNC Symbol;Acc:HGNC:24513]	2.2<br>531<br>9	5.8<br>536<br>1	-1.3773<br>6	do<br>wn	0.04<br>286
FMNL1	Uncharacterized protein [Source:UniProtKB/TrEMBL;Acc:Q5F3R6]	1.4<br>424	4.8<br>291	-1.7432<br>5	do<br>wn	0.04<br>286

		4	2			
SMOC1	SPARC related modular calcium binding 1 [Source:HGNC Symbol;Acc:HGNC:20318]	1.2	0.0	6.18582	up	0.04
		291	168			286
		1	8			
CCL20	C-C motif chemokine 20 precursor [Source:RefSeq peptide;Acc:NP_989769]	0.0	1.4	-3.9064	do	0.04
		934	011	9	wn	286
		4	3			
CZH9OR	vav-like protein C9orf100 homolog [Source:RefSeq peptide;Acc:NP_001182618]	4.0	0.5	2.82433	up	0.04
F100		817	762			389
		5	9			
NRG2	neuregulin 2 [Source:HGNC Symbol;Acc:HGNC:7998]	0.7	3.4	-2.2410	do	0.04
		381	893	1	wn	412
		4	9			

STOML1	stomatin like 1 [Source:HGNC Symbol;Acc:HGNC:14560]	0.3<br>200<br>4	2.5<br>558<br>5	-2.9974<br>8	do<br>wn	0.04<br>431
SDF2	stromal cell-derived factor 2 [Source:HGNC Symbol;Acc:HGNC:10675]	15.<br>466<br>2	6.0<br>651<br>5	1.3505	up	0.04<br>431
WTAP	Wilms tumor 1 associated protein [Source:HGNC Symbol;Acc:HGNC:16846]	4.3<br>815<br>2	15.<br>407<br>4	-1.8141<br>2	do<br>wn	0.04<br>431
CACNB2	calcium channel, voltage-dependent, beta 2 subunit [Source:HGNC Symbol;Acc:HGNC:1402]	0.7<br>371<br>8	0.1<br>839<br>9	2.00243	up	0.04<br>472
SLC37A2	solute carrier family 37 (glucose-6-phosphate transporter), member 2 [Source:HGNC	2.5	5.2	-1.0561	do	0.04

	Symbol;Acc:HGNC:20644]	339<br>6	692<br>1	9	wn	472
PTPN5	protein tyrosine phosphatase, non-receptor type 5 (striatum-enriched) [Source:HGNC Symbol;Acc:HGNC:9657]	0.3<br>741<br>6	1.9<br>498<br>5	-2.3816<br>5	do<br>wn	0.04<br>494
NLN	neurolysin (metallopeptidase M3 family) [Source:HGNC Symbol;Acc:HGNC:16058]	3.4<br>373<br>4	1.5<br>502	1.14884	up	0.04<br>724
ENSGAL<br>G000000<br>23424	NA	0.1<br>166<br>9	0.7<br>494<br>3	-2.6830<br>8	do<br>wn	0.04<br>768
MCPH1	microcephalin [Source:RefSeq peptide;Acc:NP_001098789]	1.2<br>374	0.4<br>407	1.48946	up	0.04<br>813

		1				
CCDC127	coiled-coil domain-containing protein 127 [Source:RefSeq peptide;Acc:NP_001026177]	1.34931	2.8246	-1.06583	down	0.04834
NEIL3	nei-like DNA glycosylase 3 [Source:HGNC Symbol;Acc:HGNC:24573]	0.69642	0.16916	2.04155	up	0.04834
SLC1A4	solute carrier family 1 (glutamate/neutral amino acid transporter), member 4 [Source:HGNC Symbol;Acc:HGNC:10942]	1.08231	2.78811	-1.36518	down	0.04899
MAMLD1	mastermind like domain containing 1 [Source:HGNC Symbol;Acc:HGNC:2568]	1.59918	4.01257	-1.32719	down	0.04899

C2CD3	C2 calcium-dependent domain containing 3 [Source:HGNC Symbol;Acc:HGNC:24564]	1.5<br>275<br>7	0.7<br>584<br>7	1.01006	up	0.04<br>921
SPI1	transcription factor PU.1 [Source:RefSeq peptide;Acc:NP_990354]	0.6<br>354<br>1	3.8<br>900<br>3	-2.6140<br>1	do<br>wn	0.04<br>921

Table S11. Mutual genes between the differentially expressed mRNAs and the target genes of 191 differentially expressed miRNAs which were selected with the criteria of P < 0.05 and fold changes either > 1.5 or < 0.67.

Symbol	gene regulation(immature VS mature liver)	miRNA ID	miRNA regulation(immature VS mature liver)
FAM195A	up	gga-miR-1329-5p	down
FAM195A	up	mmu-mir-5098-p5	up
ARHGAP25	down	PC-5p-479025_5	up
ARHGAP25	down	cin-miR-33_R+4	down
ARHGAP25	down	gga-miR-107-3p_R-1	up
ARHGAP25	down	gga-miR-130b-5p	up
ARHGAP25	down	gga-miR-130b-5p_R-1	up
ARHGAP25	down	gga-miR-130b-5p_R-3	up
ARHGAP25	down	gga-miR-138-5p_R+7	up
ARHGAP25	down	gga-miR-1555-5p	up
ARHGAP25	down	gga-miR-15b-5p_R-1	up
ARHGAP25	down	gga-miR-16c-5p	up
ARHGAP25	down	gga-miR-30a-3p_1ss18GA	up
ARHGAP25	down	gga-miR-30a-3p_2ss10GA20AT	up
ARHGAP25	down	gga-miR-30e-3p_L+1	up
ARHGAP25	down	gga-miR-33-5p	down
ARHGAP25	down	gga-miR-460a-5p	up
ARHGAP25	down	mmu-miR-8094_L+1R+3_1ss3AG	up
SNRPE	up	aca-miR-18a-3p_L+1R-1_1ss12GA	up
BF1	down	gga-miR-125b-5p	down

BF1	down	gga-miR-138-5p_R+7	up
BF1	down	sha-miR-125a_R+2	down
BF1	down	ssa-miR-125a-5p_R+2	down
RASSF2	down	aca-miR-129b-5p_R+1_1ss20TG	up
RASSF2	down	aca-miR-181a_R+3	up
RASSF2	down	gga-let-7c-3p_1ss12TC	up
RASSF2	down	gga-miR-107-5p_R+1	up
RASSF2	down	gga-miR-130b-3p	up
RASSF2	down	gga-miR-130b-5p	up
RASSF2	down	gga-miR-130b-5p_R-1	up
RASSF2	down	gga-miR-130b-5p_R-3	up
RASSF2	down	gga-miR-144-3p_L-1R-2	up
RASSF2	down	gga-miR-146b-3p_L+2	down
RASSF2	down	gga-miR-1552-5p_R+1	up
RASSF2	down	gga-miR-15b-3p_L+1_1ss22AC	up
RASSF2	down	gga-miR-1662_R-3	down
RASSF2	down	gga-miR-181a-5p	up
RASSF2	down	gga-miR-181b-5p_R+1	up
RASSF2	down	gga-miR-222a	up
RASSF2	down	gga-miR-30c-1-3p_R+1	up
RASSF2	down	gga-miR-454-3p	up
RASSF2	down	gga-miR-489-3p_L-2R+1	up
RASSF2	down	gga-mir-147-p3_1	down
RASSF2	down	gga-mir-147-p3_2	down
RASSF2	down	gga-mir-147-p5	down
RASSF2	down	ggo-miR-342_R+1	up

RASSF2	down	oan-miR-19b-1-5p_R+4	up
RASSF2	down	pma-miR-181a-5p_R+3	up
RASSF2	down	tgu-miR-1662_L+1_1ss23TA	down
CAMK4	up	aca-miR-425-5p_R+4	down
CAMK4	up	cin-miR-33_R+4	down
CAMK4	up	gga-let-7b_L-1R-1	down
CAMK4	up	gga-miR-138-5p_R+7	up
CAMK4	up	gga-miR-196-5p_R+2	down
CAMK4	up	gga-miR-2954_R+2	down
CAMK4	up	gga-miR-33-5p	down
CAMK4	up	hsa-miR-425-5p_R+2_1ss20TC	down
BTN1A1	down	gga-miR-135a-2-3p_L-1R+1	down
BTN1A1	down	gga-miR-1796	up
BTN1A1	down	gga-miR-205a_R+1	up
BTN1A1	down	gga-miR-205b	up
BTN1A1	down	gga-miR-460a-3p	up
BTN1A1	down	tni-miR-205_R+2	up
FABP3	down	gga-miR-1716_L-2	up
CHTF8	up	gga-miR-138-5p_R+7	up
CHTF8	up	gga-miR-146a-3p_R-1	down
CHTF8	up	gga-miR-222b-5p_R-4	down
CHTF8	up	gga-miR-383-3p	up
CHTF8	up	gga-miR-6604-5p_1ss7GA	up
ACOT7	up	aca-miR-181a_R+3	up
ACOT7	up	gga-miR-181a-5p	up
ACOT7	up	gga-miR-181b-5p_R+1	up

ACOT7	up	gga-miR-30a-3p_1ss18GA	up
ACOT7	up	gga-miR-30a-3p_2ss10GA20AT	up
ACOT7	up	gga-miR-30e-3p_L+1	up
ACOT7	up	gga-miR-9-5p	down
ACOT7	up	pma-miR-181a-5p_R+3	up
CTSS	down	gga-miR-106-3p_R+1	up
CTSS	down	gga-miR-146a-3p_R-1	down
WNT11	down	aca-miR-129b-5p_R+1_1ss20TG	up
WNT11	down	gga-miR-137-3p_L+1	up
KIF18B	up	gga-miR-138-5p_R+7	up
KIF18B	up	gga-miR-15b-5p_R-1	up
KIF18B	up	gga-miR-1699_L-1R-2	up
KIF18B	up	gga-miR-16c-5p	up
KIF18B	up	gga-miR-1783_R+1	up
KIF18B	up	gga-miR-30c-1-3p_R+1	up
TAT	down	aca-miR-18a-3p_L+1R-1_1ss12GA	up
TAT	down	gga-miR-107-5p_R+1	up
TAT	down	gga-miR-140-3p_L+1R+1_1ss22CA	down
TAT	down	ggo-miR-342_R+1	up
TAT	down	sha-miR-140_R+2	down
PIGR	down	gga-miR-1747-3p	up
PIGR	down	gga-miR-1784-5p	down
PIGR	down	mmu-miR-8094_L+1R+3_1ss3AG	up
UBXN6	down	mmu-miR-8094_L+1R+3_1ss3AG	up
PHB	up	gga-miR-1747-3p	up
SLC25A1	up	aca-miR-150-5p_1ss12TC	down

SLC25A1	up	gga-miR-135a-2-3p_L-1R+1	down
SLC25A1	up	gga-miR-146a-3p_R-1	down
SLC25A1	up	gga-miR-1796	up
SLC25A1	up	gga-miR-181b-1-3p_L+2R-2	up
SLC25A1	up	gga-miR-456-3p_1ss22AT	up
SLC25A1	up	gga-miR-456-3p_R-1	up
SLC25A1	up	gga-miR-6604-5p_1ss7GA	up
SLC25A1	up	hsa-miR-181b-3p_L+1R-1	up
SLC25A1	up	pma-miR-456_R+1_1ss22AC	up
SRRM1	up	aca-miR-18a-3p_L+1R-1_1ss12GA	up
SRRM1	up	gga-miR-106-3p_R+1	up
SRRM1	up	gga-miR-1329-5p	down
SRRM1	up	gga-miR-140-5p_L+1	down
SRRM1	up	gga-miR-1467-3p_L+1	up
SRRM1	up	gga-miR-1559-3p	up
SRRM1	up	gga-miR-17-3p_L-1R+3	up
SRRM1	up	ggo-miR-342_R+1	up
FAM18B1	down	aca-miR-150-5p_1ss12TC	down
FAM18B1	down	gga-miR-106-3p_R+1	up
FAM18B1	down	gga-miR-144-3p_L-1R-2	up
FAM18B1	down	gga-miR-1467-3p_L+1	up
FAM18B1	down	gga-miR-1788-3p_R+1	up
FAM18B1	down	gga-miR-1796	up
FAM18B1	down	gga-miR-456-3p_1ss22AT	up
FAM18B1	down	gga-miR-456-3p_R-1	up
FAM18B1	down	gga-mir-147-p3_1	down

FAM18B1	down	gga-mir-147-p5	down
FAM18B1	down	ggo-miR-342_R+1	up
FAM18B1	down	oha-miR-7-1-3p_1ss10GA	up
FAM18B1	down	pma-miR-456_R+1_1ss22AC	up
FAM18B1	down	tgu-miR-1784-3p_1ss11GA	down
MYBBP1A	up	gga-miR-107-3p_R-1	up
MYBBP1A	up	gga-miR-107-5p_R+1	up
MYBBP1A	up	gga-miR-130b-5p	up
MYBBP1A	up	gga-miR-130b-5p_R-1	up
MYBBP1A	up	gga-miR-130b-5p_R-3	up
MYBBP1A	up	gga-miR-1662_R-3	down
MYBBP1A	up	gga-miR-1784-5p	down
MYBBP1A	up	gga-miR-301a-5p_L+2	up
COX10	up	gga-miR-122-5p	down
COX10	up	gga-miR-140-3p_L+1R+1_1ss22CA	down
COX10	up	gga-miR-142-3p	down
COX10	up	gga-miR-144-3p_L-1R-2	up
COX10	up	gga-miR-1668-3p	up
COX10	up	gga-miR-1788-3p_R+1	up
COX10	up	gga-miR-214_L+1R-3_1ss19GA	down
COX10	up	gga-miR-2954_R+2	down
COX10	up	gga-miR-460a-5p	up
COX10	up	gga-miR-6604-5p_1ss7GA	up
COX10	up	gga-miR-92-5p	up
COX10	up	sha-miR-140_R+2	down
PTPRU	up	gga-miR-107-3p_R-1	up

PTPRU	up	gga-miR-130b-3p	up
PTPRU	up	gga-miR-1555-5p	up
PTPRU	up	gga-miR-383-3p	up
PTPRU	up	gga-miR-454-3p	up
GSN	down	aca-miR-363-3p_R+1	down
GSN	down	oan-miR-363-3p_R+1	up
STOML1	down	cin-miR-33_R+4	down
STOML1	down	gga-miR-33-5p	down
STOML1	down	gga-mir-1609-p3	up
ACAD8	up	gga-miR-1668-3p	up
ACAD8	up	gga-miR-1699_L-1R-2	up
ACAD8	up	gga-miR-6604-5p_1ss7GA	up
ATP2A3	down	gga-miR-2954_R+2	down
NT5DC2	down	gga-miR-458a-3p	down
MYO1F	down	gga-miR-106-3p_R+1	up
MYO1F	down	gga-miR-107-3p_R-1	up
MYO1F	down	gga-miR-15b-5p_R-1	up
MYO1F	down	gga-miR-1668-3p	up
MYO1F	down	gga-miR-16c-5p	up
MYO1F	down	gga-miR-6604-5p_1ss7GA	up
CDC45	up	gga-miR-107-3p_R-1	up
SLC16A1	up	aca-miR-150-5p_1ss12TC	down
SLC16A1	up	gga-miR-106-3p_R+1	up
SLC16A1	up	gga-miR-144-3p_L-1R-2	up
SLC16A1	up	gga-miR-146c-5p_4ss18GA20AG22TG23GT	down

SLC16A1	up	gga-miR-15b-5p_R-1	up
SLC16A1	up	gga-miR-16-1-3p_R-1	up
SLC16A1	up	gga-miR-1668-3p	up
SLC16A1	up	gga-miR-16c-3p_L+1R-1	up
SLC16A1	up	gga-miR-16c-5p	up
SLC16A1	up	gga-miR-1754-5p	up
SLC16A1	up	gga-miR-2131-3p_R+1	up
SLC16A1	up	gga-miR-214_L+1R-3_1ss19GA	down
SLC16A1	up	gga-miR-222b-5p_R-4	down
SLC16A1	up	gga-miR-383-3p	up
SLC16A1	up	gga-miR-429-3p	up
SLC16A1	up	gga-miR-551-3p_R+1	up
SLC16A1	up	xtr-miR-429_R+2	down
ACSBG2	down	aca-miR-363-3p_R+1	down
ACSBG2	down	cin-miR-33_R+4	down
ACSBG2	down	gga-let-7j-3p_L-1R+2	down
ACSBG2	down	gga-miR-106-3p_R+1	up
ACSBG2	down	gga-miR-125b-5p	down
ACSBG2	down	gga-miR-1783_R+1	up
ACSBG2	down	gga-miR-181b-5p_R+1	up
ACSBG2	down	gga-miR-205a_R+1	up
ACSBG2	down	gga-miR-205b	up
ACSBG2	down	gga-miR-33-5p	down
ACSBG2	down	gga-miR-460a-5p	up
ACSBG2	down	gga-miR-9-5p	down
ACSBG2	down	ggo-miR-342_R+1	up

ACSBG2	down	oan-miR-363-3p_R+1	up
ACSBG2	down	sha-miR-125a_R+2	down
ACSBG2	down	ssa-miR-125a-5p_R+2	down
ACSBG2	down	tni-miR-205_R+2	up
GFRA2	up	cin-miR-33_R+4	down
GFRA2	up	gga-miR-138-5p_R+7	up
GFRA2	up	gga-miR-1663-5p_R+2	up
GFRA2	up	gga-miR-33-5p	down
TNFRSF18	down	PC-3p-68190_58	up
TNFRSF18	down	cgr-miR-500	up
TNFRSF18	down	gga-miR-140-5p_L+1	down
TNFRSF18	down	gga-miR-146c-5p_4ss18GA20AG22TG23GT	down
TNFRSF18	down	gga-miR-1602	up
TNFRSF18	down	gga-miR-17-3p_L-1R+3	up
TNFRSF18	down	gga-miR-181b-1-3p_L+2R-2	up
TNFRSF18	down	gga-miR-214_L+1R-3_1ss19GA	down
TNFRSF18	down	gga-miR-460a-3p	up
TNFRSF18	down	ggo-miR-342_R+1	up
TNFRSF18	down	hsa-miR-143-5p	up
TNFRSF18	down	hsa-miR-181b-3p_L+1R-1	up
TNFRSF18	down	hsa-miR-3120-3p_R+1	down
TNFRSF18	down	mmu-miR-500-3p_R-1	up
VAV3	down	csa-let-7d_1ss10TC	up
VAV3	down	gga-let-7a-5p	down
VAV3	down	gga-let-7a-5p_2ss12GT18TC	down

VAV3	down	gga-let-7b_L-1R-1	down
VAV3	down	gga-let-7b_R+2	down
VAV3	down	gga-let-7c-5p_1ss17AG	down
VAV3	down	gga-let-7f-3p_1ss22CT	down
VAV3	down	gga-let-7g-5p_R+1_2ss14TG18CA	down
VAV3	down	gga-let-7k-5p_R+1_1ss16AT	down
VAV3	down	gga-miR-107-3p_R-1	up
VAV3	down	gga-miR-107-5p_R+1	up
VAV3	down	gga-miR-1329-5p	down
VAV3	down	gga-miR-138-5p_R+7	up
VAV3	down	gga-miR-146c-5p_4ss18GA20AG22TG23GT	down
VAV3	down	gga-miR-1662_R-3	down
VAV3	down	gga-miR-17-3p_L-1R+3	up
VAV3	down	gga-miR-1747-3p	up
VAV3	down	gga-miR-1796	up
VAV3	down	gga-miR-196-5p_R+2	down
VAV3	down	gga-miR-205a_R+1	up
VAV3	down	gga-miR-205b	up
VAV3	down	gga-miR-29a-5p_L-1R-2	down
VAV3	down	gga-miR-489-3p_L-2R+1	up
VAV3	down	gga-miR-6548-5p	up
VAV3	down	gga-miR-6710-3p_R+1	up
VAV3	down	gga-miR-9-5p	down
VAV3	down	oha-miR-7-1-3p_1ss10GA	up
VAV3	down	tni-miR-205_R+2	up

HEXA	down	gga-miR-214_L+1R-3_1ss19GA	down
HEXA	down	gga-miR-7460-3p	up
HEXA	down	ggo-miR-342_R+1	up
PMPCA	up	PC-3p-68190_58	up
PMPCA	up	cgr-miR-500	up
PMPCA	up	gga-miR-107-3p_R-1	up
PMPCA	up	gga-miR-125b-3p_R-1_1ss21CT	up
PMPCA	up	gga-miR-15b-5p_R-1	up
PMPCA	up	gga-miR-16-1-3p_R-1	up
PMPCA	up	gga-miR-1668-3p	up
PMPCA	up	gga-miR-16c-5p	up
PMPCA	up	gga-miR-181b-1-3p_L+2R-2	up
PMPCA	up	gga-miR-20b-3p_R-1	up
PMPCA	up	gga-miR-214_L+1R-3_1ss19GA	down
PMPCA	up	gga-miR-222b-5p_R-4	down
PMPCA	up	gga-miR-29a-5p_L-1R-2	down
PMPCA	up	gga-miR-3607-5p_R+1	up
PMPCA	up	gga-miR-490-3p_R+1	up
PMPCA	up	hsa-miR-181b-3p_L+1R-1	up
PMPCA	up	mmu-miR-500-3p_R-1	up
PMPCA	up	oan-miR-19b-1-5p_R+4	up
COMT	up	gga-miR-1559-3p	up
COMT	up	gga-miR-1720-5p	up
COMT	up	gga-miR-2131-3p_R+1	up
COMT	up	gga-miR-30a-3p_1ss18GA	up
COMT	up	gga-miR-30a-3p_2ss10GA20AT	up

COMT	up	gga-miR-30e-3p_L+1	up
COMT	up	gga-miR-455-5p	up
PGAM5	up	gga-miR-1788-3p_R+1	up
PPIB	up	gga-miR-107-5p_R+1	up
RFFL	down	aca-miR-18a-5p	up
RFFL	down	cgr-miR-500	up
RFFL	down	cin-miR-33_R+4	down
RFFL	down	gga-miR-106-3p_R+1	up
RFFL	down	gga-miR-1329-5p	down
RFFL	down	gga-miR-155_R+1	down
RFFL	down	gga-miR-15b-5p_R-1	up
RFFL	down	gga-miR-16c-5p	up
RFFL	down	gga-miR-1729-5p_R-1	down
RFFL	down	gga-miR-21-5p	down
RFFL	down	gga-miR-214_L+1R-3_1ss19GA	down
RFFL	down	gga-miR-216b	up
RFFL	down	gga-miR-222a	up
RFFL	down	gga-miR-301a-5p_L+2	up
RFFL	down	gga-miR-33-5p	down
RFFL	down	gga-miR-460a-5p	up
RFFL	down	gga-miR-6548-5p	up
RFFL	down	gga-miR-6604-5p_1ss7GA	up
RFFL	down	ggo-miR-342_R+1	up
RFFL	down	hsa-miR-21-5p_R+2_1ss1TN	down
RFFL	down	hsa-miR-4443_R+1	up
RFFL	down	mmu-miR-500-3p_R-1	up

DMXL1	down	gga-miR-1559-3p	up
KLHL6	down	gga-miR-383-3p	up
KLHL6	down	mmu-miR-8094_L+1R+3_1ss3AG	up
KLHL6	down	oan-let-7f-1-3p_R+2_1ss8AG	down
AP3S1	down	chi-miR-361-5p	up
AP3S1	down	dre-miR-24_R+2	down
AP3S1	down	efu-miR-361	up
AP3S1	down	gga-let-7j-3p_L-1R+2	down
AP3S1	down	gga-miR-107-3p_R-1	up
AP3S1	down	gga-miR-1329-5p	down
AP3S1	down	gga-miR-1451-3p_R-1	up
AP3S1	down	gga-miR-1788-3p_R+1	up
AP3S1	down	gga-miR-20b-3p_R-1	up
AP3S1	down	gga-miR-22-3p	down
AP3S1	down	gga-miR-222b-5p_R-4	down
AP3S1	down	gga-miR-30a-3p_2ss10GA20AT	up
AP3S1	down	gga-miR-30a-5p_R+2_1ss13CT	up
AP3S1	down	gga-miR-30b-5p	up
AP3S1	down	gga-miR-30c-5p	up
AP3S1	down	gga-miR-30d_R+2	up
AP3S1	down	gga-miR-30e-5p_R+5	up
AP3S1	down	gga-miR-383-3p	up
AP3S1	down	gga-miR-6548-5p	up
AP3S1	down	hsa-miR-3120-3p_R+1	down
AP3S1	down	oan-let-7f-1-3p_R+2_1ss8AG	down
AP3S1	down	ola-miR-126-5p_R+1_1ss1CN	down

AP3S1	down	tgu-miR-30a-5p_R+2	up
ATG12	down	gga-miR-1467-3p_L+1	up
ATG12	down	gga-miR-205a_R+1	up
ATG12	down	gga-miR-205b	up
ATG12	down	gga-miR-2954_R+2	down
ATG12	down	ola-miR-199a-3p_R+1_1ss22AG	down
ATG12	down	tni-miR-205_R+2	up
OAZ2	down	gga-miR-1788-5p	up
HSPA9	up	aca-miR-150-5p_1ss12TC	down
HSPA9	up	chi-miR-361-5p	up
HSPA9	up	cin-miR-33_R+4	down
HSPA9	up	dre-miR-24_R+2	down
HSPA9	up	efu-miR-361	up
HSPA9	up	gga-miR-140-3p_L+1R+1_1ss22CA	down
HSPA9	up	gga-miR-1552-3p_R+1	up
HSPA9	up	gga-miR-1784-5p	down
HSPA9	up	gga-miR-33-5p	down
HSPA9	up	oan-miR-19b-1-5p_R+4	up
HSPA9	up	sha-miR-140_R+2	down
PSPH	down	cin-miR-33_R+4	down
PHKG1	up	gga-miR-1555-5p	up
CHCHD2	up	chi-miR-361-5p	up
CHCHD2	up	efu-miR-361	up
CHCHD2	up	gga-miR-130b-5p	up
CHCHD2	up	gga-miR-130b-5p_R-1	up
CHCHD2	up	gga-miR-130b-5p_R-3	up

CHCHD2	up	gga-miR-1555-5p	up
CHCHD2	up	gga-miR-6548-5p	up
CHCHD2	up	hsa-miR-4443_R+1	up
SPSB1	down	aca-miR-18a-3p_L+1R-1_1ss12GA	up
SPSB1	down	gga-miR-222b-5p_R-4	down
RGS2	down	aca-miR-425-5p_R+4	down
RGS2	down	gga-miR-107-3p_R-1	up
RGS2	down	gga-miR-122-5p	down
RGS2	down	gga-miR-125b-3p_R-1_1ss21CT	up
RGS2	down	gga-miR-1555-5p	up
RGS2	down	gga-miR-429-3p	up
RGS2	down	hsa-miR-425-5p_R+2_1ss20TC	down
RGS2	down	xtr-miR-429_R+2	down
CZH9ORF1<br>00	up	aca-miR-129b-5p_R+1_1ss20TG	up
DEF6	down	gga-miR-107-3p_R-1	up
DEF6	down	gga-miR-1662_R-3	down
DEF6	down	gga-miR-1754-5p	up
DEF6	down	gga-miR-1784-5p	down
DEF6	down	gga-miR-6604-5p_1ss7GA	up
DEF6	down	tgu-miR-1784-3p_1ss11GA	down
ANXA11	down	gga-miR-202-5p_L-1	up
ANXA11	down	gga-miR-22-3p	down
ANXA11	down	gga-miR-6604-5p_1ss7GA	up
ANXA11	down	mmu-miR-8094_L+1R+3_1ss3AG	up
SBNO2	down	dre-miR-24_R+2	down

SBNO2	down	gga-miR-107-3p_R-1	up
SBNO2	down	gga-miR-138-5p_R+7	up
SBNO2	down	gga-miR-1602	up
HSD17B7	down	gga-miR-107-5p_R+1	up
HSD17B7	down	gga-miR-140-3p_L+1R+1_1ss22CA	down
HSD17B7	down	gga-miR-1467-3p_L+1	up
HSD17B7	down	gga-miR-181b-1-3p_L+1R-1	up
HSD17B7	down	gga-miR-222b-5p_R-4	down
HSD17B7	down	gga-miR-29b-3p_R-1	down
HSD17B7	down	gga-miR-29c-3p_L+1R+1_1ss19GA	down
HSD17B7	down	gga-miR-29c-3p_R+1	down
HSD17B7	down	gga-miR-29c-3p_R+1_1ss18GA	down
HSD17B7	down	gga-miR-7460-3p	up
HSD17B7	down	sha-miR-140_R+2	down
HSD17B7	down	ssa-miR-29b-3p_R-1_2ss20TC21TC	down
HSD17B7	down	tni-miR-205_R+2	up
SLC15A4	down	gga-miR-107-3p_R-1	up
SLC15A4	down	gga-miR-130b-3p	up
SLC15A4	down	gga-miR-30a-5p_R+2_1ss13CT	up
SLC15A4	down	gga-miR-30b-5p	up
SLC15A4	down	gga-miR-30c-5p	up
SLC15A4	down	gga-miR-30d_R+2	up
SLC15A4	down	gga-miR-30e-5p_R+5	up
SLC15A4	down	gga-miR-454-3p	up
SLC15A4	down	tgu-miR-30a-5p_R+2	up
LIPG	down	aca-miR-129b-5p_R+1_1ss20TG	up

FRZB	down	aca-miR-138-2-3p_L-2R+3	up
FRZB	down	gga-miR-130b-3p	up
FRZB	down	gga-miR-1329-5p	down
FRZB	down	gga-miR-1699_L-1R-2	up
FRZB	down	gga-miR-454-3p	up
DNAJC12	down	aca-miR-181a_R+3	up
DNAJC12	down	gga-miR-106-3p_R+1	up
DNAJC12	down	gga-miR-144-3p_L-1R-2	up
DNAJC12	down	gga-miR-1559-3p	up
DNAJC12	down	gga-miR-155_R+1	down
DNAJC12	down	gga-miR-1602	up
DNAJC12	down	gga-miR-1796	up
DNAJC12	down	gga-miR-181a-5p	up
DNAJC12	down	gga-miR-181b-5p_R+1	up
DNAJC12	down	gga-miR-429-3p	up
DNAJC12	down	ggo-miR-342_R+1	up
DNAJC12	down	hsa-miR-143-5p	up
DNAJC12	down	pma-miR-181a-5p_R+3	up
DNAJC12	down	xtr-miR-429_R+2	down
VAT1	down	gga-miR-15b-5p_R-1	up
VAT1	down	gga-miR-16c-5p	up
TRAIP	up	gga-miR-107-3p_R-1	up
TRAIP	up	gga-miR-2954_R+2	down
TRAIP	up	gga-miR-30c-1-3p_R+1	up
HMGCS2	up	hsa-miR-4286_R+1	up
CCL20	down	gga-let-7c-3p_1ss12TC	up

CCL20	down	gga-miR-1329-5p	down
CCL20	down	gga-miR-155_R+1	down
CCL20	down	gga-miR-6548-5p	up
SRM	up	aca-miR-425-5p_R+4	down
SRM	up	dre-miR-24_R+2	down
SRM	up	gga-miR-1720-5p	up
SRM	up	gga-miR-200b-5p_L+2	down
SRM	up	gga-miR-223_1ss15AG	down
SRM	up	gga-miR-223_1ss19CT	down
SRM	up	gga-miR-223_R+1	down
SRM	up	gga-miR-383-3p	up
SRM	up	gga-miR-460a-3p	up
SRM	up	hsa-miR-425-5p_R+2_1ss20TC	down
SRM	up	hsa-miR-4443_R+1	up
PRDX6	down	gga-miR-138-5p_R+7	up
PRDX6	down	gga-miR-144-3p_L-1R-2	up
PRDX6	down	gga-miR-1663-5p_R+2	up
PRDX6	down	mmu-miR-8094_L+1R+3_1ss3AG	up
FECH	up	gga-miR-181b-1-3p_L+1R-1	up
FECH	up	gga-miR-181b-1-3p_L+2R-2	up
FECH	up	gga-miR-30a-3p_1ss18GA	up
FECH	up	gga-miR-30a-3p_2ss10GA20AT	up
FECH	up	gga-miR-30e-3p_L+1	up
FECH	up	hsa-miR-181b-3p_L+1R-1	up
RGS14	down	gga-miR-1467-3p_L+1	up
BKJ	down	gga-miR-222b-5p_R-4	down

BKJ	down	odi-miR-7_R+2	down
LITAF	down	gga-miR-205a_R+1	up
LITAF	down	gga-miR-205b	up
LITAF	down	tni-miR-205_R+2	up
KIF20A	up	cin-miR-33_R+4	down
KIF20A	up	gga-miR-142-3p	down
KIF20A	up	gga-miR-1451-3p_R-1	up
KIF20A	up	gga-miR-146c-5p_4ss18GA20AG22T G23GT	down
KIF20A	up	gga-miR-1555-5p	up
KIF20A	up	gga-miR-1731-5p	up
KIF20A	up	gga-miR-1788-5p	up
KIF20A	up	gga-miR-1796	up
KIF20A	up	gga-miR-181b-1-3p_L+2R-2	up
KIF20A	up	gga-miR-200b-5p_L+2	down
KIF20A	up	gga-miR-205a_R+1	up
KIF20A	up	gga-miR-205b	up
KIF20A	up	gga-miR-22-3p	down
KIF20A	up	gga-miR-222b-5p_R-4	down
KIF20A	up	gga-miR-30c-1-3p_R+1	up
KIF20A	up	gga-miR-33-5p	down
KIF20A	up	gga-miR-3607-5p_R+1	up
KIF20A	up	gga-miR-383-3p	up
KIF20A	up	gga-miR-455-5p	up
KIF20A	up	gga-miR-460a-5p	up
KIF20A	up	gga-miR-9-5p	down

KIF20A	up	hsa-miR-181b-3p_L+1R-1	up
KIF20A	up	hsa-miR-21-5p_R+2_1ss1TN	down
KIF20A	up	mmu-miR-8094_L+1R+3_1ss3AG	up
KIF20A	up	tni-miR-205_R+2	up
KIF20A	up	tni-miR-455_L+1_2ss22CT23GT	down
GNA11	up	gga-miR-130b-5p	up
GNA11	up	gga-miR-130b-5p_R-1	up
GNA11	up	gga-miR-130b-5p_R-3	up
ITPA	up	gga-miR-107-3p_R-1	up
ITPA	up	gga-miR-15b-5p_R-1	up
ITPA	up	gga-miR-16c-5p	up
DNAJA4	down	cin-miR-33_R+4	down
DNAJA4	down	gga-miR-137-3p_L+1	up
DNAJA4	down	gga-miR-1559-3p	up
DNAJA4	down	gga-miR-1783_R+1	up
DNAJA4	down	gga-miR-1784-5p	down
DNAJA4	down	gga-miR-33-5p	down
DNAJA4	down	gga-miR-365-2-5p_L-1R+1	up
DNAJA4	down	gga-miR-460a-5p	up
ADAP2	down	gga-miR-181b-1-3p_L+1R-1	up
GBGT1	down	aca-miR-363-5p	up
GBGT1	down	aca-miR-425-5p_R+4	down
GBGT1	down	cgr-miR-326	up
GBGT1	down	gga-miR-140-3p_L+1R+1_1ss22CA	down
GBGT1	down	gga-miR-1602	up
GBGT1	down	gga-miR-1662_R-3	down

GBGT1	down	gga-miR-1754-5p	up
GBGT1	down	gga-miR-181b-1-3p_L+2R-2	up
GBGT1	down	gga-miR-383-3p	up
GBGT1	down	gga-miR-455-5p	up
GBGT1	down	gga-miR-489-3p_L-2R+1	up
GBGT1	down	gga-miR-6548-5p	up
GBGT1	down	gga-miR-6604-5p_1ss7GA	up
GBGT1	down	gga-mir-147-p3_1	down
GBGT1	down	gga-mir-147-p5	down
GBGT1	down	ggo-miR-342_R+1	up
GBGT1	down	hsa-miR-181b-3p_L+1R-1	up
GBGT1	down	hsa-miR-3120-3p_R+1	down
GBGT1	down	hsa-miR-425-5p_R+2_1ss20TC	down
GBGT1	down	sha-miR-140_R+2	down
ELL	down	dre-miR-24_R+2	down
ELL	down	gga-miR-1329-5p	down
ELL	down	gga-miR-144-3p_L-1R-2	up
ELL	down	gga-miR-1555-5p	up
ELL	down	gga-miR-1729-5p_R-1	down
ELL	down	gga-miR-222b-5p_R-4	down
ELL	down	gga-miR-92-5p	up
ELL	down	ggo-miR-342_R+1	up
ELL	down	mmu-miR-8094_L+1R+3_1ss3AG	up
ELL	down	mmu-mir-5098-p5	up
AIMP2	up	gga-miR-20b-3p_R-1	up
AIMP2	up	gga-miR-458a-3p	down

BYSL	up	gga-miR-106-3p_R+1	up
BARD1	up	aca-miR-181a_R+3	up
BARD1	up	aca-miR-363-3p_R+1	down
BARD1	up	gga-miR-107-3p_R-1	up
BARD1	up	gga-miR-1329-5p	down
BARD1	up	gga-miR-1559-3p	up
BARD1	up	gga-miR-155_R+1	down
BARD1	up	gga-miR-1783_R+1	up
BARD1	up	gga-miR-181a-5p	up
BARD1	up	gga-miR-181b-5p_R+1	up
BARD1	up	gga-miR-202-5p_L-1	up
BARD1	up	gga-miR-301a-5p_L+2	up
BARD1	up	oan-miR-363-3p_R+1	up
BARD1	up	pma-miR-181a-5p_R+3	up
CCND3	down	cgr-miR-151-3p	up
CCND3	down	cgr-miR-326	up
CCND3	down	gga-miR-15b-5p_R-1	up
CCND3	down	gga-miR-1602	up
CCND3	down	gga-miR-1662_R-3	down
CCND3	down	gga-miR-16c-5p	up
CCND3	down	gga-miR-1759-3p_L-3	up
ARRDC2	up	gga-miR-1668-3p	up
ARRDC2	up	gga-miR-1716_L-2	up
ARRDC2	up	gga-miR-214_L+1R-3_1ss19GA	down
ARRDC2	up	gga-miR-29b-3p_R-1	down
ARRDC2	up	gga-miR-29c-3p_R+1	down

ARRDC2	up	gga-miR-29c-3p_R+1_1ss18GA	down
ARRDC2	up	gga-mir-1724-p5_1ss21CT	up
ARRDC2	up	hsa-miR-4286_R+1	up
ARRDC2	up	ssa-miR-29b-3p_R-1_2ss20TC21TC	down
SLC6A2	down	gga-let-7j-3p_L-1R+2	down
SLC6A2	down	gga-miR-130b-5p	up
SLC6A2	down	gga-miR-130b-5p_R-1	up
SLC6A2	down	gga-miR-130b-5p_R-3	up
SLC6A2	down	gga-miR-135a-2-3p_L-1R+1	down
SLC6A2	down	gga-miR-1663-5p_R+2	up
SLC6A2	down	gga-miR-1731-5p	up
SLC6A2	down	gga-miR-1783_R+1	up
SLC6A2	down	gga-miR-2131-3p_R+1	up
SLC6A2	down	gga-miR-6548-5p	up
SLC6A2	down	gga-miR-6604-5p_1ss7GA	up
SLC6A2	down	oan-let-7f-1-3p_R+2_1ss8AG	down
LPCAT2	down	aca-miR-181a_R+3	up
LPCAT2	down	gga-miR-1329-5p	down
LPCAT2	down	gga-miR-142-3p	down
LPCAT2	down	gga-miR-1729-5p_R-1	down
LPCAT2	down	gga-miR-181a-5p	up
LPCAT2	down	gga-miR-181b-1-3p_L+1R-1	up
LPCAT2	down	gga-miR-181b-5p_R+1	up
LPCAT2	down	gga-miR-20b-5p	up
LPCAT2	down	gga-miR-2131-3p_R+1	up
LPCAT2	down	gga-miR-3607-5p_R+1	up

LPCAT2	down	gga-miR-454-3p	up
LPCAT2	down	gga-miR-6604-5p_1ss7GA	up
LPCAT2	down	gga-miR-92-5p	up
LPCAT2	down	mmu-miR-8094_L+1R+3_1ss3AG	up
LPCAT2	down	oan-miR-19b-1-5p_R+4	up
LPCAT2	down	odi-miR-7_R+2	down
LPCAT2	down	ola-miR-199a-3p_R+1_1ss22AG	down
LPCAT2	down	pma-miR-181a-5p_R+3	up
KPNA2	up	gga-let-7b_L-1R-1	down
KPNA2	up	gga-miR-196-5p_R+2	down
KPNA2	up	gga-miR-20b-5p	up
KPNA2	up	gga-miR-30a-3p_1ss18GA	up
KPNA2	up	gga-miR-30a-3p_2ss10GA20AT	up
KPNA2	up	gga-miR-30e-3p_L+1	up
KPNA2	up	gga-miR-3607-5p_R+1	up
FEN1	up	aca-miR-150-5p_1ss12TC	down
FEN1	up	gga-miR-106-3p_R+1	up
FEN1	up	gga-miR-137-3p_L+1	up
FEN1	up	gga-miR-1559-3p	up
FEN1	up	gga-miR-1668-3p	up
FEN1	up	gga-miR-181b-1-3p_L+1R-1	up
FEN1	up	gga-miR-301a-5p_L+2	up
FEN1	up	gga-miR-365-2-5p_L-1R+1	up
FEN1	up	gga-miR-6548-5p	up
FEN1	up	odi-miR-7_R+2	down
FEN1	up	ola-miR-126-5p_R+1_1ss1CN	down

PIGS	up	gga-miR-1602	up
PIGS	up	gga-miR-460a-3p	up
DHTKD1	up	gga-miR-181b-1-3p_L+2R-2	up
DHTKD1	up	gga-miR-460a-5p	up
DHTKD1	up	gga-miR-6604-5p_1ss7GA	up
DHTKD1	up	gga-miR-7460-3p	up
DHTKD1	up	hsa-miR-181b-3p_L+1R-1	up
DHTKD1	up	odi-miR-7_R+2	down
SDF2	up	gga-miR-128-1-5p	up
RICTOR	down	gga-miR-144-3p_L-1R-2	up
RICTOR	down	gga-miR-16c-3p_L+1R-1	up
RICTOR	down	gga-miR-2954_R+2	down
RICTOR	down	gga-miR-30a-3p_1ss18GA	up
RICTOR	down	gga-miR-30e-3p_L+1	up
ALDH4A1	up	ola-miR-199a-3p_R+1_1ss22AG	down
TIMD4	down	gga-miR-107-3p_R-1	up
TIMD4	down	gga-miR-107-5p_R+1	up
TIMD4	down	gga-miR-1329-5p	down
TIMD4	down	gga-miR-17-3p_L-1R+3	up
TIMD4	down	gga-miR-1747-3p	up
TIMD4	down	gga-miR-9-5p	down
DBNDD2	up	gga-miR-122-5p	down
DBNDD2	up	gga-miR-15b-5p_R-1	up
DBNDD2	up	gga-miR-16c-5p	up
DBNDD2	up	gga-miR-223_1ss15AG	down
DBNDD2	up	gga-miR-223_1ss19CT	down

DBNDD2	up	gga-miR-223_R+1	down
DBNDD2	up	gga-miR-30c-1-3p_R+1	up
SDC4	down	gga-miR-137-3p_L+1	up
SDC4	down	gga-miR-6604-5p_1ss7GA	up
KLF2	down	gga-miR-107-5p_R+1	up
DNMT3A	up	gga-miR-456-3p_1ss22AT	up
DNMT3A	up	gga-miR-456-3p_R-1	up
DNMT3A	up	gga-miR-6604-5p_1ss7GA	up
DNMT3A	up	pma-miR-456_R+1_1ss22AC	up
DNA2	up	gga-miR-15b-5p_R-1	up
DNA2	up	gga-miR-16c-5p	up
DNA2	up	gga-miR-29b-3p_R-1	down
DNA2	up	gga-miR-29c-3p_R+1	down
DNA2	up	gga-miR-29c-3p_R+1_1ss18GA	down
DNA2	up	gga-miR-30a-5p_R+2_1ss13CT	up
DNA2	up	gga-miR-30b-5p	up
DNA2	up	gga-miR-30c-5p	up
DNA2	up	gga-miR-30d_R+2	up
DNA2	up	gga-miR-30e-5p_R+5	up
DNA2	up	ssa-miR-29b-3p_R-1_2ss20TC21TC	down
DNA2	up	tgu-miR-30a-5p_R+2	up
HMGCL	down	gga-miR-1731-5p	up
HMGCL	down	gga-miR-1759-3p_L-3	up
HMGCL	down	gga-miR-1788-3p_R+1	up
HMGCL	down	gga-miR-200b-5p_L+2	down
HMGCL	down	gga-miR-6548-5p	up

GPR174	down	aca-miR-18a-5p	up
GPR174	down	gga-miR-138-5p_R+7	up
GPR174	down	gga-miR-144-3p_L-1R-2	up
GPR174	down	gga-miR-1555-5p	up
GPR174	down	gga-miR-1602	up
GPR174	down	gga-miR-1788-3p_R+1	up
GPR174	down	gga-miR-6604-5p_1ss7GA	up
UHRF1	up	mmu-mir-5098-p5	up
DDX50	up	gga-let-7j-3p_L-1R+2	down
DDX50	up	gga-miR-107-5p_R+1	up
DDX50	up	gga-miR-1329-5p	down
DDX50	up	gga-miR-429-3p	up
DDX50	up	xtr-miR-429_R+2	down
ADA	down	aca-miR-150-5p_1ss12TC	down
ADA	down	cgr-miR-500	up
ADA	down	gga-miR-125b-3p_R-1_1ss21CT	up
ADA	down	gga-miR-125b-5p	down
ADA	down	gga-miR-140-3p_L+1R+1_1ss22CA	down
ADA	down	gga-miR-140-5p_L+1	down
ADA	down	gga-miR-1784-5p	down
ADA	down	gga-miR-181b-1-3p_L+1R-1	up
ADA	down	gga-miR-202-5p_L-1	up
ADA	down	gga-miR-20b-3p_R-1	up
ADA	down	gga-miR-301a-5p_L+2	up
ADA	down	gga-miR-429-3p	up
ADA	down	hsa-miR-3120-3p_R+1	down

ADA	down	mmu-miR-500-3p_R-1	up
ADA	down	sha-miR-125a_R+2	down
ADA	down	sha-miR-140_R+2	down
ADA	down	ssa-miR-125a-5p_R+2	down
ADA	down	xtr-miR-429_R+2	down
KIF4A	up	gga-miR-1788-3p_R+1	up
KIF4A	up	gga-miR-29b-3p_R-1	down
KIF4A	up	gga-miR-29c-3p_R+1	down
KIF4A	up	gga-miR-29c-3p_R+1_1ss18GA	down
KIF4A	up	gga-miR-30a-5p_R+2_1ss13CT	up
KIF4A	up	gga-miR-30b-5p	up
KIF4A	up	gga-miR-30c-5p	up
KIF4A	up	gga-miR-30d_R+2	up
KIF4A	up	gga-miR-30e-5p_R+5	up
KIF4A	up	gga-miR-383-3p	up
KIF4A	up	gga-miR-490-3p_R+1	up
KIF4A	up	odi-miR-7_R+2	down
KIF4A	up	ssa-miR-29b-3p_R-1_2ss20TC21TC	down
KIF4A	up	tgu-miR-30a-5p_R+2	up
P4HA1	down	gga-miR-122-5p	down
P4HA1	down	gga-miR-140-3p_L+1R+1_1ss22CA	down
P4HA1	down	gga-miR-1747-3p	up
P4HA1	down	gga-miR-1796	up
P4HA1	down	gga-miR-20b-3p_R-1	up
P4HA1	down	rno-miR-122-5p_L+1R+1_1	down
P4HA1	down	rno-miR-122-5p_L+1R+1_2	down

P4HA1	down	sha-miR-140_R+2	down
COL18A1	up	cgr-miR-151-3p	up
COL18A1	up	gga-let-7j-3p_L-1R+2	down
COL18A1	up	gga-miR-140-3p_L+1R+1_1ss22CA	down
COL18A1	up	gga-miR-140-5p_L+1	down
COL18A1	up	gga-miR-29c-3p_L+1R+1_1ss19GA	down
COL18A1	up	hsa-miR-4286_R+1	up
COL18A1	up	sha-miR-140_R+2	down
MAP2K6	up	gga-miR-107-5p_R+1	up
MAP2K6	up	gga-miR-1699_L-1R-2	up
MAP2K6	up	gga-miR-29c-3p_L+1R+1_1ss19GA	down
MAP2K6	up	gga-miR-7460-3p	up
MAP2K6	up	mmu-mir-5098-p5	up
CACYBP	down	gga-miR-30a-5p_R+2_1ss13CT	up
CACYBP	down	gga-miR-30b-5p	up
CACYBP	down	gga-miR-30c-5p	up
CACYBP	down	gga-miR-30d_R+2	up
CACYBP	down	gga-miR-30e-5p_R+5	up
CACYBP	down	tgu-miR-30a-5p_R+2	up
RGS9BP	down	aca-miR-138-2-3p_L-2R+3	up
RGS9BP	down	aca-miR-150-5p_1ss12TC	down
RGS9BP	down	gga-let-7f-3p_1ss22CT	down
RGS9BP	down	gga-miR-125b-3p_R-1_1ss21CT	up
RGS9BP	down	gga-miR-1329-5p	down
RGS9BP	down	gga-miR-1552-5p_R+1	up
RGS9BP	down	gga-miR-1559-3p	up

RGS9BP	down	gga-miR-15b-5p_R-1	up
RGS9BP	down	gga-miR-16c-5p	up
RGS9BP	down	gga-miR-181b-1-3p_L+2R-2	up
RGS9BP	down	gga-miR-202-5p_L-1	up
RGS9BP	down	gga-miR-222b-5p_R-4	down
RGS9BP	down	gga-miR-29a-5p_L-1R-2	down
RGS9BP	down	gga-miR-29b-3p_R-1	down
RGS9BP	down	gga-miR-29c-3p_L+1R+1_1ss19GA	down
RGS9BP	down	gga-miR-29c-3p_R+1	down
RGS9BP	down	gga-miR-29c-3p_R+1_1ss18GA	down
RGS9BP	down	gga-miR-30a-3p_1ss18GA	up
RGS9BP	down	gga-miR-30a-3p_2ss10GA20AT	up
RGS9BP	down	gga-miR-30a-5p_R+2_1ss13CT	up
RGS9BP	down	gga-miR-30b-5p	up
RGS9BP	down	gga-miR-30c-5p	up
RGS9BP	down	gga-miR-30d_R+2	up
RGS9BP	down	gga-miR-30e-3p_L+1	up
RGS9BP	down	gga-miR-30e-5p_R+5	up
RGS9BP	down	gga-miR-3529	up
RGS9BP	down	gga-miR-429-3p	up
RGS9BP	down	gga-miR-6710-3p_R+1	up
RGS9BP	down	hsa-miR-181b-3p_L+1R-1	up
RGS9BP	down	hsa-miR-3120-3p_R+1	down
RGS9BP	down	odi-miR-7_R+2	down
RGS9BP	down	ssa-miR-29b-3p_R-1_2ss20TC21TC	down
RGS9BP	down	tgu-miR-30a-5p_R+2	up

RGS9BP	down	xtr-miR-429_R+2	down
CPSF4	up	gga-miR-130b-3p	up
CPSF4	up	gga-miR-140-3p_L+1R+1_1ss22CA	down
CPSF4	up	gga-miR-15b-5p_R-1	up
CPSF4	up	gga-miR-16c-5p	up
CPSF4	up	gga-miR-181b-1-3p_L+1R-1	up
CPSF4	up	gga-miR-181b-1-3p_L+2R-2	up
CPSF4	up	gga-miR-214_L+1R-3_1ss19GA	down
CPSF4	up	gga-miR-301a-5p_L+2	up
CPSF4	up	gga-miR-30a-3p_1ss18GA	up
CPSF4	up	gga-miR-30a-3p_2ss10GA20AT	up
CPSF4	up	gga-miR-30e-3p_L+1	up
CPSF4	up	gga-miR-454-3p	up
CPSF4	up	hsa-miR-181b-3p_L+1R-1	up
CPSF4	up	sha-miR-140_R+2	down
RGL1	down	aca-miR-181a_R+3	up
RGL1	down	aca-miR-363-3p_R+1	down
RGL1	down	gga-miR-107-5p_R+1	up
RGL1	down	gga-miR-130b-3p	up
RGL1	down	gga-miR-146a-3p_R-1	down
RGL1	down	gga-miR-155_R+1	down
RGL1	down	gga-miR-1784-5p	down
RGL1	down	gga-miR-181a-5p	up
RGL1	down	gga-miR-181b-5p_R+1	up
RGL1	down	gga-miR-20b-3p_R-1	up
RGL1	down	gga-miR-429-3p	up

RGL1	down	gga-miR-454-3p	up
RGL1	down	gga-miR-6604-5p_1ss7GA	up
RGL1	down	mmu-miR-8094_L+1R+3_1ss3AG	up
RGL1	down	oan-miR-363-3p_R+1	up
RGL1	down	pma-miR-181a-5p_R+3	up
RGL1	down	xtr-miR-429_R+2	down
EPHB2	up	gga-miR-22-3p	down
ALDH3A2	up	gga-let-7b_L-1R-1	down
ALDH3A2	up	ggo-miR-342_R+1	up
ALDH3A2	up	mmu-miR-8094_L+1R+3_1ss3AG	up
ALDH3A2	up	ola-miR-126-5p_R+1_1ss1CN	down
WNT4	down	aca-miR-129b-5p_R+1_1ss20TG	up
WNT4	down	gga-miR-222b-5p_R-4	down
FAM129A	down	gga-miR-1329-5p	down
FAM129A	down	gga-miR-1699_L-1R-2	up
FAM129A	down	gga-miR-1731-5p	up
FAM129A	down	gga-miR-1788-3p_R+1	up
FAM129A	down	gga-miR-1788-5p	up
FAM129A	down	gga-miR-429-3p	up
FAM129A	down	xtr-miR-429_R+2	down
WSCD2	down	gga-miR-1329-3p	down
WSCD2	down	gga-miR-15b-5p_R-1	up
WSCD2	down	gga-miR-16c-5p	up
ATPAF2	up	gga-miR-1559-3p	up
ATPAF2	up	gga-miR-1699_L-1R-2	up
C2H3ORF7	up	gga-miR-107-3p_R-1	up

5			
C2H3ORF75	up	gga-miR-1668-3p	up
C2H3ORF75	up	gga-miR-223_1ss15AG	down
C2H3ORF75	up	gga-miR-223_1ss19CT	down
C2H3ORF75	up	gga-miR-223_R+1	down
C2H3ORF75	up	gga-miR-456-3p_1ss22AT	up
C2H3ORF75	up	gga-miR-456-3p_R-1	up
C2H3ORF75	up	pma-miR-456_R+1_1ss22AC	up
MPP1	up	aca-miR-363-3p_R+1	down
MPP1	up	gga-miR-1602	up
MPP1	up	gga-miR-1668-3p	up
MPP1	up	gga-miR-1783_R+1	up
MPP1	up	gga-miR-181a-3p	up
MPP1	up	gga-miR-7460-3p	up
MPP1	up	oan-miR-363-3p_R+1	up
PLAU	down	gga-miR-106-3p_R+1	up
PLAU	down	gga-miR-140-5p_L+1	down
PLAU	down	gga-miR-144-3p_L-1R-2	up
PLAU	down	gga-miR-1559-3p	up

PLAU	down	gga-miR-16-1-3p_R-1	up
PLAU	down	gga-miR-301a-5p_L+2	up
PLAU	down	gga-miR-30a-3p_1ss18GA	up
PLAU	down	gga-miR-30a-3p_2ss10GA20AT	up
PLAU	down	gga-miR-30e-3p_L+1	up
PLAU	down	gga-miR-34a-3p_R-1	up
PLAU	down	gga-miR-458a-3p	down
PLAU	down	gga-miR-460a-5p	up
PLAU	down	hsa-miR-143-5p	up
PLAU	down	rno-miR-122-5p_L+1R+1_1	down
PLAU	down	rno-miR-122-5p_L+1R+1_2	down
ACSL3	up	chi-miR-361-5p	up
ACSL3	up	efu-miR-361	up
ACSL3	up	gga-miR-181b-1-3p_L+2R-2	up
ACSL3	up	hsa-miR-181b-3p_L+1R-1	up
VCAM1	down	aca-miR-181a_R+3	up
VCAM1	down	gga-let-7c-3p_1ss12TC	up
VCAM1	down	gga-miR-1329-5p	down
VCAM1	down	gga-miR-181a-5p	up
VCAM1	down	gga-miR-181b-5p_R+1	up
VCAM1	down	gga-miR-20b-3p_R-1	up
VCAM1	down	gga-miR-383-3p	up
VCAM1	down	gga-miR-429-3p	up
VCAM1	down	hsa-miR-3120-3p_R+1	down
VCAM1	down	oan-let-7f-1-3p_R+2_1ss8AG	down
VCAM1	down	pma-miR-181a-5p_R+3	up

VCAM1	down	xtr-miR-429_R+2	down
SOX8	up	PC-5p-479025_5	up
SOX8	up	gga-miR-205a_R+1	up
SOX8	up	gga-miR-205b	up
SOX8	up	tni-miR-205_R+2	up
CYB5R2	up	aca-miR-18a-5p	up
CYB5R2	up	gga-miR-107-5p_R+1	up
CYB5R2	up	gga-miR-130b-5p	up
CYB5R2	up	gga-miR-130b-5p_R-1	up
CYB5R2	up	gga-miR-130b-5p_R-3	up
CYB5R2	up	gga-miR-138-5p_R+7	up
CYB5R2	up	gga-miR-181b-1-3p_L+1R-1	up
CYB5R2	up	mmu-mir-5098-p5	up
CYB5R2	up	ola-miR-199a-3p_R+1_1ss22AG	down
PPIC	up	aca-miR-363-3p_R+1	down
PPIC	up	gga-miR-122-5p	down
PPIC	up	gga-miR-146b-3p_L+2	down
PPIC	up	oan-miR-363-3p_R+1	up
MRM1	up	gga-miR-138-5p_R+7	up
MRM1	up	gga-miR-15b-5p_R-1	up
MRM1	up	gga-miR-16c-5p	up
MRM1	up	gga-miR-29b-3p_R-1	down
MRM1	up	gga-miR-29c-3p_R+1	down
MRM1	up	gga-miR-29c-3p_R+1_1ss18GA	down
MRM1	up	ssa-miR-29b-3p_R-1_2ss20TC21TC	down
PDLIM1	down	gga-miR-1329-5p	down

PDLIM1	down	gga-miR-16c-5p	up
PDLIM1	down	gga-miR-301a-5p_L+2	up
PDLIM1	down	gga-miR-6548-5p	up
PDLIM1	down	mmu-miR-8094_L+1R+3_1ss3AG	up
PALMD	down	aca-miR-138-2-3p_L-2R+3	up
ARHGEF3	down	aca-miR-181a_R+3	up
ARHGEF3	down	cgr-miR-151-3p	up
ARHGEF3	down	cin-miR-33_R+4	down
ARHGEF3	down	gga-miR-107-5p_R+1	up
ARHGEF3	down	gga-miR-130b-5p	up
ARHGEF3	down	gga-miR-130b-5p_R-1	up
ARHGEF3	down	gga-miR-130b-5p_R-3	up
ARHGEF3	down	gga-miR-140-3p_L+1R+1_1ss22CA	down
ARHGEF3	down	gga-miR-16c-3p_L+1R-1	up
ARHGEF3	down	gga-miR-181a-5p	up
ARHGEF3	down	gga-miR-181b-1-3p_L+1R-1	up
ARHGEF3	down	gga-miR-181b-5p_R+1	up
ARHGEF3	down	gga-miR-20b-3p_R-1	up
ARHGEF3	down	gga-miR-20b-5p	up
ARHGEF3	down	gga-miR-214_L+1R-3_1ss19GA	down
ARHGEF3	down	gga-miR-22-3p	down
ARHGEF3	down	gga-miR-29a-5p_L-1R-2	down
ARHGEF3	down	gga-miR-30a-5p_R+2_1ss13CT	up
ARHGEF3	down	gga-miR-30b-5p	up
ARHGEF3	down	gga-miR-30c-5p	up
ARHGEF3	down	gga-miR-30d_R+2	up

ARHGEF3	down	gga-miR-30e-5p_R+5	up
ARHGEF3	down	gga-miR-33-5p	down
ARHGEF3	down	gga-miR-34a-3p_R-1	up
ARHGEF3	down	gga-miR-429-3p	up
ARHGEF3	down	gga-miR-460a-5p	up
ARHGEF3	down	gga-miR-6710-3p_R+1	up
ARHGEF3	down	mmu-mir-5098-p5	up
ARHGEF3	down	ola-miR-199a-3p_R+1_1ss22AG	down
ARHGEF3	down	pma-miR-181a-5p_R+3	up
ARHGEF3	down	sha-miR-140_R+2	down
ARHGEF3	down	tgu-miR-30a-5p_R+2	up
ARHGEF3	down	xtr-miR-429_R+2	down
ST6GAL1	down	PC-5p-479025_5	up
ST6GAL1	down	aca-miR-363-3p_R+1	down
ST6GAL1	down	gga-miR-1329-5p	down
ST6GAL1	down	gga-miR-20b-5p	up
ST6GAL1	down	gga-miR-22-3p	down
ST6GAL1	down	hsa-miR-143-5p	up
ST6GAL1	down	oan-miR-363-3p_R+1	up
ST6GAL1	down	oha-miR-7-1-3p_1ss10GA	up
TBL3	up	gga-let-7j-3p_L-1R+2	down
TBL3	up	oan-let-7f-1-3p_R+2_1ss8AG	down
LYVE1	down	mmu-miR-8094_L+1R+3_1ss3AG	up
GARS	up	gga-miR-106-3p_R+1	up
GARS	up	gga-miR-1788-3p_R+1	up
HSCB	up	gga-miR-1788-5p	up

HSCB	up	gga-miR-2954_R+2	down
HSCB	up	gga-miR-34a-3p_R-1	up
ITGB1BP3	up	gga-miR-1451-3p_R-1	up
ITGB1BP3	up	gga-miR-460a-3p	up
COX4I1	up	gga-mir-147-p3_2	down
XBP1	down	aca-miR-363-3p_R+1	down
XBP1	down	cin-miR-33_R+4	down
XBP1	down	gga-miR-1699_L-1R-2	up
XBP1	down	gga-miR-1747-3p	up
XBP1	down	gga-miR-205a_R+1	up
XBP1	down	gga-miR-205b	up
XBP1	down	gga-miR-33-5p	down
XBP1	down	gga-miR-456-3p_1ss22AT	up
XBP1	down	gga-miR-456-3p_R-1	up
XBP1	down	oan-miR-363-3p_R+1	up
XBP1	down	pma-miR-456_R+1_1ss22AC	up
XBP1	down	tni-miR-205_R+2	up
TMEM41B	down	PC-3p-68190_58	up
TMEM41B	down	aca-miR-150-5p_1ss12TC	down
TMEM41B	down	chi-miR-361-5p	up
TMEM41B	down	efu-miR-361	up
TMEM41B	down	gga-miR-1559-3p	up
TMEM41B	down	gga-miR-15b-5p_R-1	up
TMEM41B	down	gga-miR-1783_R+1	up
TMEM41B	down	gga-miR-1784-5p	down
TMEM41B	down	gga-miR-214_L+1R-3_1ss19GA	down

TMEM41B	down	gga-miR-30a-5p_R+2_1ss13CT	up
TMEM41B	down	gga-miR-30d_R+2	up
TMEM41B	down	gga-miR-30e-5p_R+5	up
TMEM41B	down	gga-miR-383-3p	up
TMEM41B	down	gga-miR-429-3p	up
TMEM41B	down	gga-miR-6548-5p	up
TMEM41B	down	gga-miR-6604-5p_1ss7GA	up
TMEM41B	down	hsa-miR-3120-3p_R+1	down
TMEM41B	down	oan-miR-19b-1-5p_R+4	up
TMEM41B	down	tgu-miR-30a-5p_R+2	up
TMEM41B	down	xtr-miR-429_R+2	down
MMP11	up	gga-let-7f-3p_1ss22CT	down
MMP11	up	gga-miR-107-3p_R-1	up
MMP11	up	gga-miR-125b-5p	down
MMP11	up	gga-miR-22-3p	down
MMP11	up	gga-miR-29b-3p_R-1	down
MMP11	up	gga-miR-29c-3p_R+1	down
MMP11	up	gga-miR-29c-3p_R+1_1ss18GA	down
MMP11	up	gga-miR-30c-1-3p_R+1	up
MMP11	up	sha-miR-125a_R+2	down
MMP11	up	ssa-miR-125a-5p_R+2	down
MMP11	up	ssa-miR-29b-3p_R-1_2ss20TC21TC	down
RRAS2	down	aca-miR-18a-5p	up
RRAS2	down	bta-miR-450a	up
RRAS2	down	gga-let-7j-3p_L-1R+2	down
RRAS2	down	gga-miR-107-3p_R-1	up

RRAS2	down	gga-miR-140-3p_L+1R+1_1ss22CA	down
RRAS2	down	gga-miR-16-1-3p_R-1	up
RRAS2	down	gga-miR-1729-5p_R-1	down
RRAS2	down	gga-miR-223_1ss15AG	down
RRAS2	down	gga-miR-223_1ss19CT	down
RRAS2	down	gga-miR-223_R+1	down
RRAS2	down	gga-miR-29c-3p_L+1R+1_1ss19GA	down
RRAS2	down	gga-miR-301a-5p_L+2	up
RRAS2	down	gga-miR-30c-1-3p_R+1	up
RRAS2	down	mmu-miR-8094_L+1R+3_1ss3AG	up
RRAS2	down	odi-miR-7_R+2	down
RRAS2	down	sha-miR-140_R+2	down
PASK	up	gga-miR-138-5p_R+7	up
PASK	up	gga-miR-1788-3p_R+1	up
MCM2	up	aca-miR-18a-3p_L+1R-1_1ss12GA	up
MCM2	up	aca-miR-18a-5p	up
MCM2	up	dre-miR-24_R+2	down
MCM2	up	gga-miR-125b-5p	down
MCM2	up	gga-miR-15b-5p_R-1	up
MCM2	up	gga-miR-16c-5p	up
MCM2	up	gga-miR-1720-5p	up
MCM2	up	gga-miR-1729-5p_R-1	down
MCM2	up	gga-miR-222b-5p_R-4	down
MCM2	up	gga-miR-3529	up
MCM2	up	hsa-miR-3120-3p_R+1	down
MCM2	up	sha-miR-125a_R+2	down

MCM2	up	ssa-miR-125a-5p_R+2	down
NDUFAB1	up	gga-miR-107-3p_R-1	up
NDUFAB1	up	gga-miR-15b-5p_R-1	up
NDUFAB1	up	gga-miR-489-3p_L-2R+1	up
GPC3	down	gga-miR-1788-5p	up
PLK1	up	gga-miR-16-1-3p_R-1	up
PLK1	up	gga-miR-222b-5p_R-4	down
PLK1	up	gga-miR-29b-3p_R-1	down
PLK1	up	gga-miR-29c-3p_R+1_1ss18GA	down
PLK1	up	gga-miR-6604-5p_1ss7GA	up
MOSPD1	down	PC-3p-68190_58	up
MOSPD1	down	aca-miR-181a_R+3	up
MOSPD1	down	dre-miR-24_R+2	down
MOSPD1	down	gga-miR-181a-5p	up
MOSPD1	down	gga-miR-181b-1-3p_L+1R-1	up
MOSPD1	down	gga-miR-181b-5p_R+1	up
MOSPD1	down	gga-miR-20b-5p	up
MOSPD1	down	gga-miR-3607-5p_R+1	up
MOSPD1	down	pma-miR-181a-5p_R+3	up
GTF2B	down	gga-miR-1788-5p	up
TPX2	up	gga-miR-214_L+1R-3_1ss19GA	down
TPX2	up	gga-miR-490-3p_R+1	up
TPX2	up	hsa-miR-3120-3p_R+1	down
GINS1	up	aca-miR-18a-5p	up
GINS1	up	gga-miR-1783_R+1	up
GINS1	up	gga-miR-2131-3p_R+1	up

GINS1	up	gga-miR-460a-5p	up
GINS1	up	mmu-miR-8094_L+1R+3_1ss3AG	up
IL21R	down	gga-miR-6604-5p_1ss7GA	up
TGFBI	down	gga-miR-137-3p_L+1	up
TGFBI	down	gga-miR-146c-5p_4ss18GA20AG22TG23GT	down
TGFBI	down	gga-miR-1759-3p_L-3	up
TGFBI	down	gga-miR-3607-5p_R+1	up
TGFBI	down	oha-miR-7-1-3p_1ss10GA	up
DDT	up	PC-5p-479025_5	up
DDT	up	aca-miR-18a-5p	up
DDT	up	gga-miR-130b-3p	up
DDT	up	gga-miR-142-5p_L-2R+1	down
DDT	up	gga-miR-16c-5p	up
DDT	up	gga-miR-214_L+1R-3_1ss19GA	down
DDT	up	gga-miR-30a-3p_1ss18GA	up
DDT	up	gga-miR-30a-3p_2ss10GA20AT	up
DDT	up	gga-miR-30e-3p_L+1	up
DDT	up	gga-miR-6710-3p_R+1	up
DDT	up	hsa-miR-4286_R+1	up
DDT	up	mmu-miR-8094_L+1R+3_1ss3AG	up
IL16	down	gga-let-7j-3p_L-1R+2	down
IL16	down	mmu-mir-5098-p5	up
IL16	down	oan-let-7f-1-3p_R+2_1ss8AG	down
SLC6A6	up	gga-miR-130b-3p	up
SLC6A6	up	gga-miR-137-3p_L+1	up

SLC6A6	up	gga-miR-20b-3p_R-1	up
SLC6A6	up	gga-miR-454-3p	up
SLC6A6	up	gga-miR-490-3p_R+1	up
PANK1	down	gga-miR-107-3p_R-1	up
PANK1	down	gga-miR-144-3p_L-1R-2	up
PANK1	down	gga-miR-15b-5p_R-1	up
PANK1	down	gga-miR-16-1-3p_R-1	up
PANK1	down	gga-miR-16c-5p	up
PANK1	down	oha-miR-7-1-3p_1ss10GA	up
FAH	up	gga-miR-1759-3p_L-3	up
C13H5ORF 15	down	gga-miR-107-3p_R-1	up
C13H5ORF 15	down	gga-miR-1668-3p	up
C13H5ORF 15	down	gga-miR-3607-5p_R+1	up
CRTAM	down	aca-miR-150-5p_1ss12TC	down
CRTAM	down	gga-miR-1668-3p	up
CRTAM	down	gga-miR-1784-5p	down
CRTAM	down	gga-miR-1788-3p_R+1	up
CRTAM	down	gga-miR-301a-5p_L+2	up
CRTAM	down	gga-miR-490-3p_R+1	up
CRTAM	down	gga-mir-147-p3_1	down
CRTAM	down	gga-mir-147-p3_2	down
CRTAM	down	gga-mir-147-p5	down
TSSC4	up	gga-miR-107-3p_R-1	up

TSSC4	up	gga-miR-1555-5p	up
TSSC4	up	gga-miR-20b-3p_R-1	up
TSSC4	up	odi-miR-7_R+2	down
SEMA3A	up	aca-miR-181a_R+3	up
SEMA3A	up	gga-miR-146c-5p_4ss18GA20AG22T G23GT	down
SEMA3A	up	gga-miR-181a-5p	up
SEMA3A	up	gga-miR-181b-5p_R+1	up
SEMA3A	up	gga-miR-30a-5p_R+2_1ss13CT	up
SEMA3A	up	gga-miR-30b-5p	up
SEMA3A	up	gga-miR-30c-5p	up
SEMA3A	up	gga-miR-30d_R+2	up
SEMA3A	up	gga-miR-30e-5p_R+5	up
SEMA3A	up	mmu-miR-8094_L+1R+3_1ss3AG	up
SEMA3A	up	mmu-mir-5098-p5	up
SEMA3A	up	ola-miR-199a-3p_R+1_1ss22AG	down
SEMA3A	up	pma-miR-181a-5p_R+3	up
SEMA3A	up	tgu-miR-30a-5p_R+2	up
ABCC1	down	PC-5p-479025_5	up
ABCC1	down	aca-miR-425-5p_R+4	down
ABCC1	down	gga-miR-107-3p_R-1	up
ABCC1	down	gga-miR-122-5p	down
ABCC1	down	gga-miR-146b-3p_L+2	down
ABCC1	down	gga-miR-15b-5p_R-1	up
ABCC1	down	gga-miR-16c-5p	up
ABCC1	down	gga-miR-1783_R+1	up

ABCC1	down	gga-miR-2131-3p_R+1	up
ABCC1	down	gga-miR-301a-5p_L+2	up
ABCC1	down	gga-miR-30a-3p_1ss18GA	up
ABCC1	down	gga-miR-30a-3p_2ss10GA20AT	up
ABCC1	down	gga-miR-30e-3p_L+1	up
ABCC1	down	hsa-miR-425-5p_R+2_1ss20TC	down
ABCC1	down	mmu-mir-5098-p5	up
ABCC1	down	oan-miR-19b-1-5p_R+4	up
ABCC1	down	tni-miR-455_L+1_2ss22CT23GT	down
TMEM41A	down	gga-miR-140-3p_L+1R+1_1ss22CA	down
TMEM41A	down	gga-miR-30a-3p_1ss18GA	up
TMEM41A	down	gga-miR-30a-3p_2ss10GA20AT	up
TMEM41A	down	gga-miR-30e-3p_L+1	up
TMEM41A	down	sha-miR-140_R+2	down
FANCI	up	aca-miR-18a-5p	up
FANCI	up	gga-miR-15b-5p_R-1	up
FANCI	up	gga-miR-16c-5p	up
FANCI	up	gga-miR-181b-1-3p_L+2R-2	up
FANCI	up	gga-miR-30c-1-3p_R+1	up
FANCI	up	hsa-miR-143-5p	up
FANCI	up	hsa-miR-181b-3p_L+1R-1	up
KIF11	up	aca-miR-138-2-3p_L-2R+3	up
KIF11	up	dre-miR-24_R+2	down
KIF11	up	gga-let-7j-3p_L-1R+2	down
KIF11	up	gga-miR-106-3p_R+1	up
KIF11	up	gga-miR-1329-5p	down

KIF11	up	gga-miR-146c-5p_4ss18GA20AG22TG23GT	down
KIF11	up	gga-miR-17-3p_L-1R+3	up
KIF11	up	gga-miR-222b-5p_R-4	down
KIF11	up	gga-miR-6710-3p_R+1	up
PIK3IP1	down	gga-miR-205b	up
TLR4	down	PC-3p-68190_58	up
TLR4	down	PC-5p-479025_5	up
TLR4	down	gga-miR-106-3p_R+1	up
TLR4	down	gga-miR-107-5p_R+1	up
TLR4	down	gga-miR-142-3p	down
TLR4	down	gga-miR-1699_L-1R-2	up
TLR4	down	gga-miR-181b-1-3p_L+1R-1	up
TLR4	down	gga-miR-22-3p	down
TLR4	down	gga-miR-29b-3p_R-1	down
TLR4	down	gga-miR-29c-3p_R+1	down
TLR4	down	gga-miR-29c-3p_R+1_1ss18GA	down
TLR4	down	gga-miR-6604-5p_1ss7GA	up
TLR4	down	gga-miR-7460-3p	up
TLR4	down	mmu-mir-5098-p5	up
TLR4	down	ssa-miR-29b-3p_R-1_2ss20TC21TC	down
ARRDC4	down	aca-miR-181a_R+3	up
ARRDC4	down	gga-miR-1552-3p_R+1	up
ARRDC4	down	gga-miR-1759-3p_L-3	up
ARRDC4	down	gga-miR-181a-5p	up
ARRDC4	down	gga-miR-181b-5p_R+1	up

ARRDC4	down	gga-miR-30a-3p_1ss18GA	up
ARRDC4	down	gga-miR-30e-3p_L+1	up
ARRDC4	down	pma-miR-181a-5p_R+3	up
PYROXD2	up	gga-miR-383-3p	up
PITRM1	up	gga-miR-15b-5p_R-1	up
PITRM1	up	gga-miR-16c-5p	up
PITRM1	up	gga-miR-222b-5p_R-4	down
PITRM1	up	oha-miR-7-1-3p_1ss10GA	up
CREM	down	aca-miR-150-5p_1ss12TC	down
CREM	down	aca-miR-425-5p_R+4	down
CREM	down	gga-miR-130b-3p	up
CREM	down	gga-miR-130b-5p	up
CREM	down	gga-miR-130b-5p_R-1	up
CREM	down	gga-miR-130b-5p_R-3	up
CREM	down	gga-miR-135a-2-3p_L-1R+1	down
CREM	down	gga-miR-144-3p_L-1R-2	up
CREM	down	gga-miR-20b-5p	up
CREM	down	gga-miR-223_1ss15AG	down
CREM	down	hsa-miR-425-5p_R+2_1ss20TC	down
CPN1	up	gga-miR-1784-5p	down
OPA1	up	gga-miR-460a-5p	up
OPA1	up	gga-miR-489-3p_L-2R+1	up
OPA1	up	mmu-mir-5098-p5	up
FADS2	down	cgr-miR-151-3p	up
FADS2	down	gga-miR-1467-3p_L+1	up
TK1	up	gga-miR-107-3p_R-1	up

TK1	up	gga-miR-1759-3p_L-3	up
TK1	up	gga-miR-214_L+1R-3_1ss19GA	down
TK1	up	gga-miR-223_1ss15AG	down
TK1	up	gga-miR-223_1ss19CT	down
TK1	up	gga-miR-223_R+1	down
TK1	up	gga-miR-455-5p	up
TK1	up	hsa-miR-3120-3p_R+1	down
COX6A1	up	gga-miR-1731-5p	up
ZEB1	down	aca-miR-150-5p_1ss12TC	down
ZEB1	down	gga-miR-1329-5p	down
ZEB1	down	gga-miR-144-3p_L-1R-2	up
ZEB1	down	gga-miR-16-1-3p_R-1	up
ZEB1	down	gga-miR-205a_R+1	up
ZEB1	down	gga-miR-216b	up
ZEB1	down	gga-miR-29a-5p_L-1R-2	down
ZEB1	down	gga-miR-34a-3p_R-1	up
ZEB1	down	gga-miR-3607-5p_R+1	up
ZEB1	down	gga-miR-429-3p	up
ZEB1	down	ola-miR-199a-3p_R+1_1ss22AG	down
ZEB1	down	tni-miR-205_R+2	up
ZEB1	down	xtr-miR-429_R+2	down
CD3D	down	gga-miR-15b-5p_R-1	up
PPRC1	up	dre-miR-24_R+2	down
PPRC1	up	gga-miR-1716_L-2	up
GOT1	up	dre-miR-24_R+2	down
GOT1	up	gga-miR-1788-3p_R+1	up

GOT1	up	gga-miR-6548-5p	up
GOT1	up	gga-miR-9-5p	down
ITGB2	down	csa-let-7d_1ss10TC	up
ITGB2	down	gga-let-7a-5p	down
ITGB2	down	gga-let-7a-5p_2ss12GT18TC	down
ITGB2	down	gga-let-7b_R+2	down
ITGB2	down	gga-let-7c-5p_1ss17AG	down
ITGB2	down	gga-let-7g-5p_R+1_2ss14TG18CA	down
ITGB2	down	gga-let-7k-5p_R+1_1ss16AT	down
ITGB2	down	gga-miR-135a-2-3p_L-1R+1	down
ITGB2	down	gga-miR-1699_L-1R-2	up
ITGB2	down	gga-miR-29b-3p_R-1	down
ITGB2	down	gga-miR-29c-3p_L+1R+1_1ss19GA	down
ITGB2	down	gga-miR-29c-3p_R+1	down
ITGB2	down	gga-miR-29c-3p_R+1_1ss18GA	down
ITGB2	down	gga-miR-30c-1-3p_R+1	up
ITGB2	down	gga-miR-365-2-5p_L-1R+1	up
ITGB2	down	hsa-miR-3120-3p_R+1	down
ITGB2	down	mmu-let-7j_1ss8TG	down
ITGB2	down	ssa-miR-29b-3p_R-1_2ss20TC21TC	down
GLS	down	gga-miR-20b-3p_R-1	up
STK32A	down	cgr-miR-326	up
STK32A	down	gga-miR-135a-2-3p_L-1R+1	down
STK32A	down	gga-miR-138-5p_R+7	up
STK32A	down	gga-miR-214_L+1R-3_1ss19GA	down
RRP12	up	PC-5p-479025_5	up

RRP12	up	aca-miR-150-5p_1ss12TC	down
RRP12	up	gga-miR-140-3p_L+1R+1_1ss22CA	down
RRP12	up	gga-miR-15b-5p_R-1	up
RRP12	up	gga-miR-16c-5p	up
RRP12	up	gga-miR-456-3p_1ss22AT	up
RRP12	up	gga-miR-456-3p_R-1	up
RRP12	up	gga-miR-6604-5p_1ss7GA	up
RRP12	up	ggo-miR-342_R+1	up
RRP12	up	pma-miR-456_R+1_1ss22AC	up
RRP12	up	sha-miR-140_R+2	down
SLC9A3R1	up	aca-miR-150-5p_1ss12TC	down
SLC9A3R1	up	gga-miR-140-3p_L+1R+1_1ss22CA	down
SLC9A3R1	up	gga-miR-1555-5p	up
SLC9A3R1	up	gga-miR-1716_L-2	up
SLC9A3R1	up	gga-miR-1759-3p_L-3	up
SLC9A3R1	up	gga-miR-30b-5p	up
SLC9A3R1	up	gga-miR-30c-5p	up
SLC9A3R1	up	gga-miR-6604-5p_1ss7GA	up
SLC9A3R1	up	gga-mir-1724-p5_1ss21CT	up
SLC9A3R1	up	sha-miR-140_R+2	down
EME1	up	aca-miR-181a_R+3	up
EME1	up	gga-miR-181a-5p	up
EME1	up	gga-miR-181b-5p_R+1	up
EME1	up	gga-miR-2131-3p_R+1	up
EME1	up	gga-mir-1609-p3	up
EME1	up	odi-miR-7_R+2	down

EME1	up	pma-miR-181a-5p_R+3	up
TRAP1	up	aca-miR-18a-3p_L+1R-1_1ss12GA	up
TRAP1	up	gga-miR-107-3p_R-1	up
TRAP1	up	gga-miR-137-3p_L+1	up
TRAP1	up	gga-miR-144-3p_L-1R-2	up
TRAP1	up	gga-miR-1552-3p_R+1	up
TRAP1	up	gga-miR-15b-5p_R-1	up
TRAP1	up	gga-miR-1699_L-1R-2	up
TRAP1	up	gga-miR-16c-5p	up
TRAP1	up	gga-miR-6604-5p_1ss7GA	up
TRAP1	up	hsa-miR-3120-3p_R+1	down
NPM3	up	gga-miR-146a-3p_R-1	down
NPM3	up	gga-miR-1788-3p_R+1	up
NPM3	up	gga-miR-214_L+1R-3_1ss19GA	down
NPM3	up	gga-miR-222a	up
NPM3	up	gga-miR-30a-5p_R+2_1ss13CT	up
NPM3	up	gga-miR-30b-5p	up
NPM3	up	gga-miR-30c-5p	up
NPM3	up	gga-miR-30d_R+2	up
NPM3	up	gga-miR-30e-5p_R+5	up
NPM3	up	gga-miR-383-3p	up
NPM3	up	tgu-miR-30a-5p_R+2	up
SALL4	up	aca-miR-138-2-3p_L-2R+3	up
SALL4	up	aca-miR-181a_R+3	up
SALL4	up	cgr-miR-326	up
SALL4	up	gga-miR-135a-2-3p_L-1R+1	down

SALL4	up	gga-miR-15b-5p_R-1	up
SALL4	up	gga-miR-16c-5p	up
SALL4	up	gga-miR-181a-5p	up
SALL4	up	gga-miR-181b-5p_R+1	up
SALL4	up	gga-miR-200b-5p_L+2	down
SALL4	up	gga-miR-216b	up
SALL4	up	gga-miR-3529	up
SALL4	up	gga-mir-147-p3_1	down
SALL4	up	gga-mir-147-p5	down
SALL4	up	pma-miR-181a-5p_R+3	up
CD44	down	aca-miR-129b-5p_R+1_1ss20TG	up
CD44	down	aca-miR-150-5p_1ss12TC	down
CD44	down	cgr-miR-500	up
CD44	down	gga-miR-125b-3p_R-1_1ss21CT	up
CD44	down	gga-miR-138-5p_R+7	up
CD44	down	gga-miR-1451-3p_R-1	up
CD44	down	gga-miR-1662_R-3	down
CD44	down	gga-miR-2131-3p_R+1	up
CD44	down	gga-miR-3529	up
CD44	down	gga-miR-489-3p_L-2R+1	up
CD44	down	gga-miR-7460-3p	up
CD44	down	mmu-miR-500-3p_R-1	up
CD44	down	tgu-miR-1662_L+1_1ss23TA	down
FANCL	up	gga-miR-106-3p_R+1	up
FANCL	up	gga-miR-144-3p_L-1R-2	up
FANCL	up	gga-miR-1788-5p	up

FANCL	up	gga-miR-490-3p_R+1	up
FANCL	up	mmu-mir-5098-p5	up
FANCL	up	xtr-miR-429_R+2	down
STK17B	down	aca-miR-363-5p	up
STK17B	down	gga-miR-130b-3p	up
STK17B	down	gga-miR-140-3p_L+1R+1_1ss22CA	down
STK17B	down	gga-miR-142-5p_L-2R+1	down
STK17B	down	gga-miR-1663-5p_R+2	up
STK17B	down	gga-miR-181b-1-3p_L+2R-2	up
STK17B	down	gga-miR-20b-5p	up
STK17B	down	gga-miR-223_1ss15AG	down
STK17B	down	gga-miR-223_1ss19CT	down
STK17B	down	gga-miR-223_R+1	down
STK17B	down	gga-miR-29b-3p_R-1	down
STK17B	down	gga-miR-29c-3p_R+1	down
STK17B	down	gga-miR-29c-3p_R+1_1ss18GA	down
STK17B	down	gga-miR-30a-3p_1ss18GA	up
STK17B	down	gga-miR-30a-5p_R+2_1ss13CT	up
STK17B	down	gga-miR-30b-5p	up
STK17B	down	gga-miR-30c-5p	up
STK17B	down	gga-miR-30d_R+2	up
STK17B	down	gga-miR-30e-3p_L+1	up
STK17B	down	gga-miR-30e-5p_R+5	up
STK17B	down	gga-miR-454-3p	up
STK17B	down	gga-miR-460a-5p	up
STK17B	down	hsa-miR-143-5p	up

STK17B	down	hsa-miR-181b-3p_L+1R-1	up
STK17B	down	hsa-miR-3120-3p_R+1	down
STK17B	down	mmu-miR-3964_L+1_1ss6GA	down
STK17B	down	oha-miR-7-1-3p_1ss10GA	up
STK17B	down	sha-miR-140_R+2	down
STK17B	down	ssa-miR-29b-3p_R-1_2ss20TC21TC	down
STK17B	down	tgu-miR-30a-5p_R+2	up
ANKRD44	down	aca-miR-138-2-3p_L-2R+3	up
ANKRD44	down	gga-miR-142-5p_L-2R+1	down
ANKRD44	down	gga-miR-181b-1-3p_L+1R-1	up
ANKRD44	down	mmu-miR-3964_L+1_1ss6GA	down
ANKRD44	down	mmu-mir-5098-p5	up
ZC3HC1	up	gga-miR-15b-5p_R-1	up
ZC3HC1	up	gga-miR-1668-3p	up
ZC3HC1	up	gga-miR-16c-5p	up
ZC3HC1	up	gga-miR-202-5p_L-1	up
CD82	down	PC-5p-479025_5	up
CD82	down	aca-miR-18a-5p	up
CD82	down	chi-miR-361-5p	up
CD82	down	efu-miR-361	up
CD82	down	gga-let-7b_L-1R-1	down
CD82	down	gga-miR-107-5p_R+1	up
CD82	down	gga-miR-15b-5p_R-1	up
CD82	down	gga-miR-1602	up
CD82	down	gga-miR-1663-5p_R+2	up
CD82	down	gga-miR-16c-5p	up

CD82	down	gga-miR-196-5p_R+2	down
CD82	down	gga-miR-200b-5p_L+2	down
CD82	down	gga-miR-222b-5p_R-4	down
CD82	down	gga-miR-456-3p_1ss22AT	up
CD82	down	gga-miR-456-3p_R-1	up
CD82	down	gga-mir-147-p3_2	down
CD82	down	gga-mir-1724-p5_1ss21CT	up
CD82	down	hsa-miR-3120-3p_R+1	down
CD82	down	hsa-miR-4443_R+1	up
CD82	down	pma-miR-456_R+1_1ss22AC	up
COQ10B	down	aca-miR-363-3p_R+1	down
COQ10B	down	mmu-miR-8094_L+1R+3_1ss3AG	up
COQ10B	down	oan-miR-363-3p_R+1	up
CHRDL1	down	aca-miR-138-2-3p_L-2R+3	up
CHRDL1	down	aca-miR-425-5p_R+4	down
CHRDL1	down	cgr-miR-326	up
CHRDL1	down	chi-miR-361-5p	up
CHRDL1	down	efu-miR-361	up
CHRDL1	down	gga-miR-106-3p_R+1	up
CHRDL1	down	gga-miR-107-3p_R-1	up
CHRDL1	down	gga-miR-107-5p_R+1	up
CHRDL1	down	gga-miR-130b-5p	up
CHRDL1	down	gga-miR-130b-5p_R-1	up
CHRDL1	down	gga-miR-130b-5p_R-3	up
CHRDL1	down	gga-miR-1329-5p	down
CHRDL1	down	gga-miR-146a-3p_R-1	down

CHRDL1	down	gga-miR-15b-5p_R-1	up
CHRDL1	down	gga-miR-16c-5p	up
CHRDL1	down	gga-miR-17-3p_L-1R+3	up
CHRDL1	down	gga-miR-1784-5p	down
CHRDL1	down	gga-miR-205a_R+1	up
CHRDL1	down	gga-miR-205b	up
CHRDL1	down	gga-miR-460a-5p	up
CHRDL1	down	gga-miR-489-3p_L-2R+1	up
CHRDL1	down	hsa-miR-425-5p_R+2_1ss20TC	down
CHRDL1	down	hsa-miR-4443_R+1	up
CHRDL1	down	tgu-miR-1784-3p_1ss11GA	down
CHRDL1	down	tni-miR-205_R+2	up
NDUFS3	up	cgr-miR-500	up
NDUFS3	up	gga-miR-107-3p_R-1	up
NDUFS3	up	gga-miR-107-5p_R+1	up
NDUFS3	up	gga-miR-140-3p_L+1R+1_1ss22CA	down
NDUFS3	up	gga-miR-15b-5p_R-1	up
NDUFS3	up	gga-miR-16c-3p_L+1R-1	up
NDUFS3	up	gga-miR-16c-5p	up
NDUFS3	up	gga-miR-205a_R+1	up
NDUFS3	up	gga-miR-205b	up
NDUFS3	up	gga-miR-460a-5p	up
NDUFS3	up	mmu-miR-500-3p_R-1	up
NDUFS3	up	sha-miR-140_R+2	down
NDUFS3	up	tni-miR-205_R+2	up
KIF23	up	gga-miR-107-3p_R-1	up

KIF23	up	gga-miR-20b-5p	up
KIF23	up	gga-miR-29a-5p_L-1R-2	down
KIF23	up	mmu-miR-8094_L+1R+3_1ss3AG	up
SFXN2	up	gga-miR-17-3p_L-1R+3	up
SFXN2	up	gga-miR-1788-3p_R+1	up
SFXN2	up	gga-miR-222b-5p_R-4	down
SFXN2	up	gga-miR-365-2-5p_L-1R+1	up
SFXN2	up	gga-miR-454-3p	up
SFXN2	up	gga-miR-6604-5p_1ss7GA	up
RINT1	down	aca-miR-425-5p_R+4	down
RINT1	down	gga-miR-1552-3p_R+1	up
RINT1	down	gga-miR-1602	up
RINT1	down	gga-miR-1668-3p	up
RINT1	down	gga-miR-205a_R+1	up
RINT1	down	gga-miR-205b	up
RINT1	down	gga-miR-3529	up
RINT1	down	hsa-miR-143-5p	up
RINT1	down	hsa-miR-3120-3p_R+1	down
RINT1	down	hsa-miR-425-5p_R+2_1ss20TC	down
RINT1	down	tni-miR-205_R+2	up
ADAMTS7	up	gga-miR-222b-5p_R-4	down
ADAMTS7	up	gga-miR-29b-3p_R-1	down
ADAMTS7	up	gga-miR-29c-3p_L+1R+1_1ss19GA	down
ADAMTS7	up	gga-miR-29c-3p_R+1	down
ADAMTS7	up	gga-miR-29c-3p_R+1_1ss18GA	down
ADAMTS7	up	gga-miR-383-3p	up

ADAMTS7	up	gga-miR-6604-5p_1ss7GA	up
ADAMTS7	up	oan-miR-19b-1-5p_R+4	up
ADAMTS7	up	ssa-miR-29b-3p_R-1_2ss20TC21TC	down
TRIM36	down	gga-miR-1784-5p	down
BUB1	up	gga-miR-1788-5p	up
BUB1	up	gga-miR-214_L+1R-3_1ss19GA	down
BUB1	up	gga-miR-30a-3p_1ss18GA	up
BUB1	up	gga-miR-30a-3p_2ss10GA20AT	up
BUB1	up	gga-miR-30e-3p_L+1	up
GRAMD3	down	PC-5p-479025_5	up
GRAMD3	down	gga-miR-106-3p_R+1	up
GRAMD3	down	gga-miR-140-3p_L+1R+1_1ss22CA	down
GRAMD3	down	sha-miR-140_R+2	down
COL4A6	up	gga-miR-205a_R+1	up
COL4A6	up	tni-miR-205_R+2	up
NAPEPLD	down	aca-miR-150-5p_1ss12TC	down
NAPEPLD	down	aca-miR-363-5p	up
NAPEPLD	down	gga-miR-137-3p_L+1	up
NAPEPLD	down	gga-miR-144-3p_L-1R-2	up
NAPEPLD	down	gga-miR-146c-5p_4ss18GA20AG22TG23GT	down
NAPEPLD	down	gga-miR-16-1-3p_R-1	up
NAPEPLD	down	gga-miR-1720-5p	up
NAPEPLD	down	gga-miR-20b-5p	up
NAPEPLD	down	gga-miR-30a-5p_R+2_1ss13CT	up
NAPEPLD	down	gga-miR-30e-5p_R+5	up

NAPEPLD	down	gga-miR-490-3p_R+1	up
NAPEPLD	down	gga-miR-6548-5p	up
NAPEPLD	down	tgu-miR-30a-5p_R+2	up
FGL2	down	aca-miR-363-3p_R+1	down
FGL2	down	cin-miR-33_R+4	down
FGL2	down	gga-miR-130b-3p	up
FGL2	down	gga-miR-137-3p_L+1	up
FGL2	down	gga-miR-1662_R-3	down
FGL2	down	gga-miR-1699_L-1R-2	up
FGL2	down	gga-miR-20b-3p_R-1	up
FGL2	down	gga-miR-222b-5p_R-4	down
FGL2	down	gga-miR-29b-3p_R-1	down
FGL2	down	gga-miR-29c-3p_R+1	down
FGL2	down	gga-miR-29c-3p_R+1_1ss18GA	down
FGL2	down	gga-miR-33-5p	down
FGL2	down	gga-miR-429-3p	up
FGL2	down	gga-miR-454-3p	up
FGL2	down	gga-miR-6710-3p_R+1	up
FGL2	down	hsa-miR-3120-3p_R+1	down
FGL2	down	oan-miR-363-3p_R+1	up
FGL2	down	ssa-miR-29b-3p_R-1_2ss20TC21TC	down
FGL2	down	tgu-miR-1662_L+1_1ss23TA	down
FGL2	down	xtr-miR-429_R+2	down
MAN2A2	up	gga-miR-214_L+1R-3_1ss19GA	down
GSTO1	down	gga-miR-489-3p_L-2R+1	up
NET1	down	aca-miR-363-5p	up

NET1	down	dre-miR-24_R+2	down
NET1	down	gga-let-7c-3p_1ss12TC	up
NET1	down	gga-let-7j-3p_L-1R+2	down
NET1	down	gga-miR-1552-3p_R+1	up
NET1	down	gga-miR-22-3p	down
NET1	down	gga-miR-6548-5p	up
NET1	down	gga-miR-7460-3p	up
NET1	down	oan-let-7f-1-3p_R+2_1ss8AG	down
NET1	down	rno-miR-122-5p_L+1R+1_1	down
NET1	down	rno-miR-122-5p_L+1R+1_2	down
SLC38A2	down	gga-miR-106-3p_R+1	up
SLC38A2	down	gga-miR-140-5p_L+1	down
SLC38A2	down	gga-miR-144-3p_L-1R-2	up
SLC38A2	down	gga-miR-1552-3p_R+1	up
SLC38A2	down	gga-miR-1731-5p	up
SLC38A2	down	gga-miR-1759-3p_L-3	up
SLC38A2	down	gga-miR-216b	up
SLC38A2	down	gga-miR-22-3p	down
SLC38A2	down	gga-miR-222a	up
SLC38A2	down	gga-miR-30a-3p_1ss18GA	up
SLC38A2	down	gga-miR-30a-3p_2ss10GA20AT	up
SLC38A2	down	gga-miR-30e-3p_L+1	up
SLC38A2	down	gga-miR-3607-5p_R+1	up
SLC38A2	down	gga-miR-429-3p	up
SLC38A2	down	gga-miR-460a-5p	up
SLC38A2	down	gga-miR-6548-5p	up

SLC38A2	down	gga-miR-6604-5p_1ss7GA	up
SLC38A2	down	mmu-miR-8094_L+1R+3_1ss3AG	up
SLC38A2	down	oan-let-7f-1-3p_R+2_1ss8AG	down
SLC38A2	down	odi-miR-7_R+2	down
SLC38A2	down	tni-miR-455_L+1_2ss22CT23GT	down
SLC38A2	down	xtr-miR-429_R+2	down
ABHD12	up	gga-miR-107-3p_R-1	up
ABHD12	up	gga-miR-130b-3p	up
ABHD12	up	gga-miR-142-3p	down
ABHD12	up	gga-miR-146a-3p_R-1	down
ABHD12	up	gga-miR-15b-5p_R-1	up
ABHD12	up	gga-miR-16c-5p	up
ABHD12	up	gga-miR-1720-5p	up
ABHD12	up	gga-miR-1788-3p_R+1	up
ABHD12	up	gga-miR-20b-3p_R-1	up
ABHD12	up	gga-miR-20b-5p	up
ABHD12	up	gga-miR-216b	up
ABHD12	up	gga-miR-30a-3p_1ss18GA	up
ABHD12	up	gga-miR-30a-3p_2ss10GA20AT	up
ABHD12	up	gga-miR-30e-3p_L+1	up
ABHD12	up	gga-miR-3529	up
ABHD12	up	gga-miR-454-3p	up
ABHD12	up	gga-miR-490-3p_R+1	up
ABHD12	up	gga-miR-6604-5p_1ss7GA	up
PEX6	up	aca-miR-18a-3p_L+1R-1_1ss12GA	up
PEX6	up	gga-miR-214_L+1R-3_1ss19GA	down

PEX6	up	ggo-miR-342_R+1	up
AHSG	up	gga-miR-1668-3p	up
AHSG	up	gga-miR-1796	up
AHSG	up	gga-miR-223_1ss15AG	down
AHSG	up	gga-miR-223_1ss19CT	down
AHSG	up	gga-miR-223_R+1	down
AHSG	up	gga-miR-6548-5p	up
NDUFS1	up	gga-let-7f-3p_1ss22CT	down
NDUFS1	up	gga-miR-140-3p_L+1R+1_1ss22CA	down
NDUFS1	up	gga-miR-1788-5p	up
NDUFS1	up	gga-miR-29a-5p_L-1R-2	down
NDUFS1	up	gga-miR-383-3p	up
NDUFS1	up	hsa-miR-3120-3p_R+1	down
NDUFS1	up	oan-miR-19b-1-5p_R+4	up
NDUFS1	up	sha-miR-140_R+2	down
CYR61	down	aca-miR-18a-5p	up
CYR61	down	gga-miR-20b-3p_R-1	up
CYR61	down	gga-miR-216b	up
CYR61	down	gga-miR-30c-1-3p_R+1	up
CD28	down	gga-miR-181b-1-3p_L+2R-2	up
CD28	down	hsa-miR-181b-3p_L+1R-1	up
CUBN	up	gga-miR-30a-3p_1ss18GA	up
CUBN	up	gga-miR-30a-3p_2ss10GA20AT	up
CUBN	up	gga-miR-30e-3p_L+1	up
CUBN	up	gga-miR-6604-5p_1ss7GA	up
CUBN	up	ola-miR-199a-3p_R+1_1ss22AG	down

ITGA8	down	aca-miR-129b-5p_R+1_1ss20TG	up
ITGA8	down	aca-miR-425-5p_R+4	down
ITGA8	down	cgr-miR-151-3p	up
ITGA8	down	gga-miR-122-5p	down
ITGA8	down	gga-miR-137-3p_L+1	up
ITGA8	down	gga-miR-15b-5p_R-1	up
ITGA8	down	gga-miR-16c-5p	up
ITGA8	down	gga-miR-223_1ss15AG	down
ITGA8	down	gga-miR-223_1ss19CT	down
ITGA8	down	gga-miR-223_R+1	down
ITGA8	down	gga-miR-30a-3p_2ss10GA20AT	up
ITGA8	down	hsa-miR-425-5p_R+2_1ss20TC	down
ITGA8	down	ola-miR-199a-3p_R+1_1ss22AG	down
ITGA8	down	rno-miR-122-5p_L+1R+1_1	down
ITGA8	down	rno-miR-122-5p_L+1R+1_2	down
CNRIP1	down	gga-miR-107-5p_R+1	up
CNRIP1	down	gga-miR-9-5p	down
UGP2	down	PC-3p-68190_58	up
UGP2	down	gga-miR-106-3p_R+1	up
UGP2	down	gga-miR-140-3p_L+1R+1_1ss22CA	down
UGP2	down	gga-miR-144-3p_L-1R-2	up
UGP2	down	gga-miR-155_R+1	down
UGP2	down	gga-miR-1699_L-1R-2	up
UGP2	down	gga-miR-21-5p	down
UGP2	down	gga-miR-214_L+1R-3_1ss19GA	down
UGP2	down	gga-miR-216b	up

UGP2	down	gga-miR-383-3p	up
UGP2	down	gga-miR-429-3p	up
UGP2	down	gga-miR-429-5p_L-1	up
UGP2	down	hsa-miR-21-5p_R+2_1ss1TN	down
UGP2	down	sha-miR-140_R+2	down
UGP2	down	xtr-miR-429_R+2	down
FXR1	down	aca-miR-363-3p_R+1	down
FXR1	down	cgr-miR-151-3p	up
FXR1	down	gga-miR-130b-3p	up
FXR1	down	gga-miR-140-5p_L+1	down
FXR1	down	gga-miR-1559-3p	up
FXR1	down	gga-miR-155_R+1	down
FXR1	down	gga-miR-21-5p	down
FXR1	down	gga-miR-29a-5p_L-1R-2	down
FXR1	down	gga-miR-30a-3p_1ss18GA	up
FXR1	down	gga-miR-30a-3p_2ss10GA20AT	up
FXR1	down	gga-miR-30e-3p_L+1	up
FXR1	down	gga-miR-3607-5p_R+1	up
FXR1	down	gga-miR-429-3p	up
FXR1	down	gga-miR-454-3p	up
FXR1	down	gga-miR-460a-5p	up
FXR1	down	hsa-miR-21-5p_R+2_1ss1TN	down
FXR1	down	oan-miR-363-3p_R+1	up
FXR1	down	ola-miR-199a-3p_R+1_1ss22AG	down
FXR1	down	xtr-miR-429_R+2	down
USP13	up	gga-miR-107-5p_R+1	up

JAG1	down	cgr-miR-500	up
JAG1	down	gga-miR-138-5p_R+7	up
JAG1	down	gga-miR-144-3p_L-1R-2	up
JAG1	down	gga-miR-16-1-3p_R-1	up
JAG1	down	gga-miR-1668-3p	up
JAG1	down	gga-miR-214_L+1R-3_1ss19GA	down
JAG1	down	gga-miR-222b-5p_R-4	down
JAG1	down	gga-miR-301a-5p_L+2	up
JAG1	down	mmu-miR-500-3p_R-1	up
JAG1	down	mmu-mir-5098-p5	up
GANC	up	gga-miR-142-3p	down
GANC	up	gga-miR-1784-5p	down
GANC	up	gga-miR-1788-3p_R+1	up
GANC	up	gga-miR-2131-3p_R+1	up
GANC	up	gga-miR-29a-5p_L-1R-2	down
GANC	up	gga-miR-3529	up
ISM1	up	gga-miR-9-5p	down
YPEL5	down	aca-miR-129b-5p_R+1_1ss20TG	up
YPEL5	down	cgr-miR-326	up
YPEL5	down	gga-miR-125b-3p_R-1_1ss21CT	up
YPEL5	down	gga-miR-130b-3p	up
YPEL5	down	gga-miR-146b-3p_L+2	down
YPEL5	down	gga-miR-1602	up
YPEL5	down	gga-miR-1668-3p	up
YPEL5	down	gga-miR-1759-3p_L-3	up
YPEL5	down	gga-miR-1788-3p_R+1	up

YPEL5	down	gga-miR-181b-1-3p_L+2R-2	up
YPEL5	down	gga-miR-20b-3p_R-1	up
YPEL5	down	gga-miR-29a-5p_L-1R-2	down
YPEL5	down	gga-miR-365-2-5p_L-1R+1	up
YPEL5	down	gga-miR-454-3p	up
YPEL5	down	gga-miR-489-3p_L-2R+1	up
YPEL5	down	hsa-miR-181b-3p_L+1R-1	up
YPEL5	down	hsa-miR-3120-3p_R+1	down
YPEL5	down	mmu-mir-5098-p5	up
ENTPD2	down	gga-miR-125b-5p	down
ENTPD2	down	gga-miR-135a-2-3p_L-1R+1	down
ENTPD2	down	gga-miR-16c-5p	up
ENTPD2	down	gga-miR-1729-5p_R-1	down
ENTPD2	down	gga-miR-1788-5p	up
ENTPD2	down	gga-miR-3529	up
ENTPD2	down	gga-miR-6604-5p_1ss7GA	up
ENTPD2	down	sha-miR-125a_R+2	down
ENTPD2	down	ssa-miR-125a-5p_R+2	down
NDUFB5	up	gga-miR-214_L+1R-3_1ss19GA	down
FSHR	down	gga-miR-106-3p_R+1	up
FSHR	down	gga-miR-1784-5p	down
FSHR	down	gga-miR-455-5p	up
FSHR	down	gga-mir-147-p3_1	down
FSHR	down	gga-mir-147-p5	down
OLA1	up	gga-miR-15b-5p_R-1	up
OLA1	up	gga-miR-16c-5p	up

OLA1	up	gga-miR-181b-1-3p_L+2R-2	up
OLA1	up	hsa-miR-181b-3p_L+1R-1	up
OLA1	up	mmu-mir-5098-p5	up
DEGS1	down	aca-miR-138-2-3p_L-2R+3	up
DEGS1	down	cin-miR-33_R+4	down
DEGS1	down	gga-miR-107-5p_R+1	up
DEGS1	down	gga-miR-146c-5p_4ss18GA20AG22TG23GT	down
DEGS1	down	gga-miR-155_R+1	down
DEGS1	down	gga-miR-1668-3p	up
DEGS1	down	gga-miR-214_L+1R-3_1ss19GA	down
DEGS1	down	gga-miR-33-5p	down
DEGS1	down	gga-miR-383-3p	up
SKIL	down	aca-miR-18a-3p_L+1R-1_1ss12GA	up
SKIL	down	gga-miR-130b-5p	up
SKIL	down	gga-miR-1720-5p	up
SKIL	down	gga-miR-1731-5p	up
SKIL	down	gga-miR-181b-1-3p_L+1R-1	up
SKIL	down	gga-miR-181b-1-3p_L+2R-2	up
SKIL	down	gga-miR-22-3p	down
SKIL	down	gga-miR-460a-5p	up
SKIL	down	hsa-miR-181b-3p_L+1R-1	up
SKIL	down	hsa-miR-4286_R+1	up
SMOC1	up	gga-let-7j-3p_L-1R+2	down
SMOC1	up	gga-miR-130b-3p	up
SMOC1	up	gga-miR-130b-5p	up

SMOC1	up	gga-miR-130b-5p_R-1	up
SMOC1	up	gga-miR-130b-5p_R-3	up
SMOC1	up	gga-miR-144-3p_L-1R-2	up
SMOC1	up	gga-miR-20b-5p	up
SMOC1	up	gga-miR-222a	up
SMOC1	up	gga-miR-454-3p	up
SMOC1	up	oan-let-7f-1-3p_R+2_1ss8AG	down
BAG3	down	PC-5p-479025_5	up
BAG3	down	aca-miR-363-5p	up
BAG3	down	cgr-miR-500	up
BAG3	down	gga-let-7c-3p_1ss12TC	up
BAG3	down	gga-miR-138-5p_R+7	up
BAG3	down	gga-miR-140-3p_L+1R+1_1ss22CA	down
BAG3	down	gga-miR-1559-3p	up
BAG3	down	gga-miR-1663-5p_R+2	up
BAG3	down	gga-miR-1731-5p	up
BAG3	down	gga-miR-1783_R+1	up
BAG3	down	gga-miR-20b-5p	up
BAG3	down	gga-miR-223_1ss15AG	down
BAG3	down	gga-miR-223_1ss19CT	down
BAG3	down	gga-miR-223_R+1	down
BAG3	down	gga-miR-30a-3p_1ss18GA	up
BAG3	down	gga-miR-30a-3p_2ss10GA20AT	up
BAG3	down	gga-miR-30e-3p_L+1	up
BAG3	down	gga-miR-9-5p	down
BAG3	down	hsa-miR-4443_R+1	up

BAG3	down	mmu-miR-500-3p_R-1	up
BAG3	down	sha-miR-140_R+2	down
IFRD1	up	aca-miR-138-2-3p_L-2R+3	up
IFRD1	up	cgr-miR-500	up
IFRD1	up	gga-let-7c-3p_1ss12TC	up
IFRD1	up	gga-let-7j-3p_L-1R+2	down
IFRD1	up	gga-miR-1329-5p	down
IFRD1	up	gga-miR-200b-5p_L+2	down
IFRD1	up	gga-miR-3607-5p_R+1	up
IFRD1	up	gga-miR-429-5p_L-1	up
IFRD1	up	mmu-miR-500-3p_R-1	up
IFRD1	up	oan-let-7f-1-3p_R+2_1ss8AG	down
DUSP10	down	aca-miR-18a-3p_L+1R-1_1ss12GA	up
DUSP10	down	aca-miR-363-3p_R+1	down
DUSP10	down	gga-miR-122-5p	down
DUSP10	down	gga-miR-147_R+1	down
DUSP10	down	gga-miR-1783_R+1	up
DUSP10	down	gga-miR-1784-5p	down
DUSP10	down	gga-miR-1796	up
DUSP10	down	gga-miR-205a_R+1	up
DUSP10	down	gga-miR-205b	up
DUSP10	down	gga-miR-22-3p	down
DUSP10	down	gga-miR-6548-5p	up
DUSP10	down	gga-mir-147-p3_2	down
DUSP10	down	oan-miR-363-3p_R+1	up
DUSP10	down	tni-miR-205_R+2	up

FNIP2	up	gga-miR-2131-3p_R+1	up
FNIP2	up	gga-miR-6710-3p_R+1	up
FNIP2	up	mmu-miR-8094_L+1R+3_1ss3AG	up
MSMO1	down	aca-miR-129b-5p_R+1_1ss20TG	up
MSMO1	down	aca-miR-363-3p_R+1	down
MSMO1	down	gga-miR-106-3p_R+1	up
MSMO1	down	gga-miR-125b-3p_R-1_1ss21CT	up
MSMO1	down	gga-miR-130b-3p	up
MSMO1	down	gga-miR-16-1-3p_R-1	up
MSMO1	down	gga-miR-16c-3p_L+1R-1	up
MSMO1	down	gga-miR-216b	up
MSMO1	down	gga-miR-223_1ss15AG	down
MSMO1	down	gga-miR-223_1ss19CT	down
MSMO1	down	gga-miR-223_R+1	down
MSMO1	down	gga-miR-29a-5p_L-1R-2	down
MSMO1	down	gga-miR-454-3p	up
MSMO1	down	hsa-miR-3120-3p_R+1	down
MSMO1	down	oan-miR-363-3p_R+1	up
RARRES1	down	aca-miR-181a_R+3	up
RARRES1	down	gga-miR-181a-5p	up
RARRES1	down	gga-miR-181b-5p_R+1	up
RARRES1	down	gga-miR-20b-3p_R-1	up
RARRES1	down	gga-miR-30a-3p_1ss18GA	up
RARRES1	down	gga-miR-30e-3p_L+1	up
RARRES1	down	gga-miR-34a-3p_R-1	up
RARRES1	down	pma-miR-181a-5p_R+3	up

THBS1	down	gga-miR-223_1ss15AG	down
THBS1	down	gga-miR-223_R+1	down
THBS1	down	gga-miR-3529	up
THBS1	down	gga-miR-460a-3p	up
THBS1	down	gga-miR-460a-5p	up
THBS1	down	hsa-miR-143-5p	up
GFM1	up	gga-miR-107-3p_R-1	up
GFM1	up	gga-miR-15b-5p_R-1	up
GFM1	up	gga-miR-16c-5p	up
GFM1	up	gga-miR-6710-3p_R+1	up
COL1A2	up	aca-miR-363-3p_R+1	down
COL1A2	up	csa-let-7d_1ss10TC	up
COL1A2	up	gga-let-7a-5p	down
COL1A2	up	gga-let-7a-5p_2ss12GT18TC	down
COL1A2	up	gga-let-7b_L-1R-1	down
COL1A2	up	gga-let-7b_R+2	down
COL1A2	up	gga-let-7c-5p_1ss17AG	down
COL1A2	up	gga-let-7g-5p_R+1_2ss14TG18CA	down
COL1A2	up	gga-let-7k-5p_R+1_1ss16AT	down
COL1A2	up	gga-miR-130b-5p	up
COL1A2	up	gga-miR-130b-5p_R-1	up
COL1A2	up	gga-miR-130b-5p_R-3	up
COL1A2	up	gga-miR-1451-3p_R-1	up
COL1A2	up	gga-miR-1796	up
COL1A2	up	gga-miR-196-5p_R+2	down
COL1A2	up	gga-miR-29b-3p_R-1	down

COL1A2	up	gga-miR-29c-3p_L+1R+1_1ss19GA	down
COL1A2	up	gga-miR-29c-3p_R+1	down
COL1A2	up	gga-miR-29c-3p_R+1_1ss18GA	down
COL1A2	up	mmu-let-7j_1ss8TG	down
COL1A2	up	oan-miR-363-3p_R+1	up
COL1A2	up	ssa-miR-29b-3p_R-1_2ss20TC21TC	down
COL1A2	up	tgu-miR-1662_L+1_1ss23TA	down
ACADSB	down	aca-miR-18a-3p_L+1R-1_1ss12GA	up
ACADSB	down	csa-let-7d_1ss10TC	up
ACADSB	down	gga-let-7a-5p	down
ACADSB	down	gga-let-7a-5p_2ss12GT18TC	down
ACADSB	down	gga-let-7b_R+2	down
ACADSB	down	gga-let-7c-5p_1ss17AG	down
ACADSB	down	gga-let-7g-5p_R+1_2ss14TG18CA	down
ACADSB	down	gga-let-7k-5p_R+1_1ss16AT	down
ACADSB	down	gga-miR-130b-3p	up
ACADSB	down	gga-miR-140-5p_L+1	down
ACADSB	down	gga-miR-142-3p	down
ACADSB	down	gga-miR-1699_L-1R-2	up
ACADSB	down	gga-miR-1788-3p_R+1	up
ACADSB	down	gga-miR-1788-5p	up
ACADSB	down	gga-miR-21-5p	down
ACADSB	down	gga-miR-214_L+1R-3_1ss19GA	down
ACADSB	down	gga-miR-216b	up
ACADSB	down	gga-miR-223_1ss15AG	down
ACADSB	down	gga-miR-223_1ss19CT	down

ACADSB	down	gga-miR-223_R+1	down
ACADSB	down	gga-miR-29a-5p_L-1R-2	down
ACADSB	down	gga-miR-301a-5p_L+2	up
ACADSB	down	gga-miR-3529	up
ACADSB	down	gga-miR-383-3p	up
ACADSB	down	gga-miR-454-3p	up
ACADSB	down	gga-miR-460a-5p	up
ACADSB	down	gga-miR-6548-5p	up
ACADSB	down	hsa-miR-21-5p_R+2_1ss1TN	down
ACADSB	down	hsa-miR-3120-3p_R+1	down
ACADSB	down	mmu-let-7j_1ss8TG	down
ACADSB	down	mmu-mir-5098-p5	up
ACADSB	down	oha-miR-7-1-3p_1ss10GA	up
CPXM2	down	aca-miR-129b-5p_R+1_1ss20TG	up
CPXM2	down	gga-miR-1759-3p_L-3	up
CPXM2	down	gga-miR-92-5p	up
PDK4	down	cgr-miR-500	up
PDK4	down	mmu-miR-500-3p_R-1	up
DTL	up	gga-miR-1783_R+1	up
DTL	up	gga-miR-429-3p	up
DTL	up	rno-miR-122-5p_L+1R+1_1	down
DTL	up	rno-miR-122-5p_L+1R+1_2	down
DTL	up	xtr-miR-429_R+2	down
NEK2	up	gga-miR-1788-5p	up
NEK2	up	gga-miR-30a-3p_1ss18GA	up
NEK2	up	gga-miR-30a-3p_2ss10GA20AT	up

NEK2	up	gga-miR-30e-3p_L+1	up
NEK2	up	gga-miR-3607-5p_R+1	up
NEK2	up	gga-miR-429-3p	up
NEK2	up	xtr-miR-429_R+2	down
CAMK1D	down	gga-miR-29b-3p_R-1	down
CAMK1D	down	gga-miR-29c-3p_R+1	down
CAMK1D	down	gga-miR-29c-3p_R+1_1ss18GA	down
CAMK1D	down	ssa-miR-29b-3p_R-1_2ss20TC21TC	down
HAAO	up	gga-miR-2131-3p_R+1	up
HAAO	up	gga-miR-383-3p	up
HAAO	up	gga-miR-460a-5p	up
HAAO	up	gga-miR-6604-5p_1ss7GA	up
CPM	down	gga-miR-2954_R+2	down
CDC20	up	gga-miR-429-3p	up
CDC20	up	xtr-miR-429_R+2	down
CFL2	up	aca-miR-138-2-3p_L-2R+3	up
CFL2	up	aca-miR-181a_R+3	up
CFL2	up	aca-miR-425-5p_R+4	down
CFL2	up	gga-miR-142-3p	down
CFL2	up	gga-miR-1662_R-3	down
CFL2	up	gga-miR-181a-5p	up
CFL2	up	gga-miR-181b-5p_R+1	up
CFL2	up	gga-miR-20b-3p_R-1	up
CFL2	up	gga-miR-20b-5p	up
CFL2	up	gga-miR-223_1ss15AG	down
CFL2	up	gga-miR-223_1ss19CT	down

CFL2	up	gga-miR-223_R+1	down
CFL2	up	gga-miR-429-3p	up
CFL2	up	hsa-miR-3120-3p_R+1	down
CFL2	up	hsa-miR-425-5p_R+2_1ss20TC	down
CFL2	up	ola-miR-199a-3p_R+1_1ss22AG	down
CFL2	up	pma-miR-181a-5p_R+3	up
CFL2	up	xtr-miR-429_R+2	down
PTPRF	up	dre-miR-24_R+2	down
PTPRF	up	gga-miR-2954_R+2	down
PLK3	down	gga-miR-146a-3p_R-1	down
NFKBIE	down	gga-miR-1329-5p	down
NFKBIE	down	gga-miR-456-3p_1ss22AT	up
NFKBIE	down	gga-miR-456-3p_R-1	up
NFKBIE	down	hsa-miR-143-5p	up
NFKBIE	down	pma-miR-456_R+1_1ss22AC	up
FBXO33	down	gga-miR-140-3p_L+1R+1_1ss22CA	down
FBXO33	down	gga-miR-383-3p	up
FBXO33	down	gga-miR-429-3p	up
FBXO33	down	sha-miR-140_R+2	down
FBXO33	down	xtr-miR-429_R+2	down
GLIPR1L	down	gga-miR-122-5p	down
GLIPR1L	down	gga-miR-1747-3p	up
GLIPR1L	down	gga-miR-181b-1-3p_L+2R-2	up
GLIPR1L	down	gga-miR-222b-5p_R-4	down
GLIPR1L	down	hsa-miR-181b-3p_L+1R-1	up
PRDX1	up	cin-miR-33_R+4	down

PRDX1	up	gga-miR-130b-5p	up
PRDX1	up	gga-miR-130b-5p_R-1	up
PRDX1	up	gga-miR-130b-5p_R-3	up
PRDX1	up	gga-miR-1731-5p	up
PRDX1	up	gga-miR-33-5p	down
PRDX1	up	gga-miR-460a-5p	up
TMEM69	up	oha-miR-7-1-3p_1ss10GA	up
TSPAN1	up	cin-miR-33_R+4	down
TSPAN1	up	gga-miR-107-5p_R+1	up
TSPAN1	up	gga-miR-140-3p_L+1R+1_1ss22CA	down
TSPAN1	up	gga-miR-222b-5p_R-4	down
TSPAN1	up	gga-miR-33-5p	down
TSPAN1	up	sha-miR-140_R+2	down
GRID2	down	gga-miR-1329-5p	down
FAAH	up	gga-miR-30a-3p_1ss18GA	up
FAAH	up	gga-miR-30a-3p_2ss10GA20AT	up
FAAH	up	gga-miR-30a-5p_R+2_1ss13CT	up
FAAH	up	gga-miR-30b-5p	up
FAAH	up	gga-miR-30c-5p	up
FAAH	up	gga-miR-30d_R+2	up
FAAH	up	gga-miR-30e-3p_L+1	up
FAAH	up	gga-miR-30e-5p_R+5	up
FAAH	up	tgu-miR-30a-5p_R+2	up
CP	down	gga-miR-22-3p	down
CMPK1	up	aca-miR-150-5p_1ss12TC	down
CMPK1	up	aca-miR-425-5p_R+4	down

CMPK1	up	gga-miR-106-3p_R+1	up
CMPK1	up	gga-miR-130b-3p	up
CMPK1	up	gga-miR-137-3p_L+1	up
CMPK1	up	gga-miR-146c-5p_4ss18GA20AG22T G23GT	down
CMPK1	up	gga-miR-1668-3p	up
CMPK1	up	gga-miR-16c-5p	up
CMPK1	up	gga-miR-1788-5p	up
CMPK1	up	gga-miR-1796	up
CMPK1	up	gga-miR-202-5p_L-1	up
CMPK1	up	gga-miR-20b-5p	up
CMPK1	up	gga-miR-222b-5p_R-4	down
CMPK1	up	gga-miR-223_1ss15AG	down
CMPK1	up	gga-miR-223_1ss19CT	down
CMPK1	up	gga-miR-223_R+1	down
CMPK1	up	gga-miR-301a-5p_L+2	up
CMPK1	up	gga-miR-383-3p	up
CMPK1	up	gga-miR-454-3p	up
CMPK1	up	gga-miR-6604-5p_1ss7GA	up
CMPK1	up	gga-miR-7460-3p	up
CMPK1	up	hsa-miR-425-5p_R+2_1ss20TC	down
CMPK1	up	mmu-miR-8094_L+1R+3_1ss3AG	up
CMPK1	up	oan-miR-19b-1-5p_R+4	up
CMPK1	up	ola-miR-199a-3p_R+1_1ss22AG	down
CMPK1	up	tgu-miR-1784-3p_1ss11GA	down
SLC25A4	up	gga-miR-106-3p_R+1	up

SLC25A4	up	gga-miR-20b-5p	up
SLC25A4	up	gga-miR-2131-3p_R+1	up
SLC25A4	up	gga-miR-454-3p	up
ORC1	up	gga-miR-20b-5p	up
MEMO1	up	gga-miR-130b-3p	up
MEMO1	up	gga-miR-30a-3p_1ss18GA	up
MEMO1	up	gga-miR-30e-3p_L+1	up
MEMO1	up	gga-miR-454-3p	up
MEMO1	up	gga-miR-456-3p_1ss22AT	up
MEMO1	up	gga-miR-456-3p_R-1	up
MEMO1	up	pma-miR-456_R+1_1ss22AC	up
DCTD	up	gga-miR-1796	up
DCTD	up	gga-miR-181b-1-3p_L+1R-1	up
DCTD	up	gga-miR-181b-1-3p_L+2R-2	up
DCTD	up	gga-miR-429-3p	up
DCTD	up	hsa-miR-181b-3p_L+1R-1	up
DCTD	up	xtr-miR-429_R+2	down
GLCCI1	down	gga-miR-21-5p	down
GLCCI1	down	gga-miR-6710-3p_R+1	up
GLCCI1	down	hsa-miR-21-5p_R+2_1ss1TN	down
NEIL3	up	chi-miR-361-5p	up
NEIL3	up	efu-miR-361	up
NEIL3	up	gga-miR-107-3p_R-1	up
NEIL3	up	gga-miR-144-3p_L-1R-2	up
NEIL3	up	gga-miR-17-3p_L-1R+3	up
NEIL3	up	hsa-miR-143-5p	up

HMGB2	up	gga-miR-15b-5p_R-1	up
HMGB2	up	gga-miR-16c-5p	up
HMGB2	up	gga-miR-181b-1-3p_L+1R-1	up
SCIN	down	aca-miR-181a_R+3	up
SCIN	down	dre-miR-24_R+2	down
SCIN	down	gga-let-7c-3p_1ss12TC	up
SCIN	down	gga-miR-107-3p_R-1	up
SCIN	down	gga-miR-125b-5p	down
SCIN	down	gga-miR-130b-5p	up
SCIN	down	gga-miR-130b-5p_R-1	up
SCIN	down	gga-miR-140-3p_L+1R+1_1ss22CA	down
SCIN	down	gga-miR-140-5p_L+1	down
SCIN	down	gga-miR-146c-5p_4ss18GA20AG22TG23GT	down
SCIN	down	gga-miR-1663-5p_R+2	up
SCIN	down	gga-miR-1668-3p	up
SCIN	down	gga-miR-1784-5p	down
SCIN	down	gga-miR-181a-5p	up
SCIN	down	gga-miR-181b-5p_R+1	up
SCIN	down	gga-miR-205a_R+1	up
SCIN	down	gga-miR-20b-3p_R-1	up
SCIN	down	gga-miR-2131-3p_R+1	up
SCIN	down	gga-miR-222b-5p_R-4	down
SCIN	down	gga-miR-29b-3p_R-1	down
SCIN	down	gga-miR-29c-3p_R+1	down
SCIN	down	gga-miR-29c-3p_R+1_1ss18GA	down

SCIN	down	gga-miR-301a-5p_L+2	up
SCIN	down	gga-miR-456-3p_1ss22AT	up
SCIN	down	gga-miR-456-3p_R-1	up
SCIN	down	gga-miR-489-3p_L-2R+1	up
SCIN	down	hsa-miR-3120-3p_R+1	down
SCIN	down	hsa-miR-4443_R+1	up
SCIN	down	pma-miR-181a-5p_R+3	up
SCIN	down	pma-miR-456_R+1_1ss22AC	up
SCIN	down	sha-miR-125a_R+2	down
SCIN	down	sha-miR-140_R+2	down
SCIN	down	ssa-miR-125a-5p_R+2	down
SCIN	down	ssa-miR-29b-3p_R-1_2ss20TC21TC	down
SCIN	down	tni-miR-205_R+2	up
ACOT11	up	hsa-miR-3120-3p_R+1	down
SLC24A4	down	aca-miR-425-5p_R+4	down
SLC24A4	down	gga-miR-15b-5p_R-1	up
SLC24A4	down	gga-miR-16c-5p	up
SLC24A4	down	gga-miR-20b-5p	up
SLC24A4	down	gga-miR-9-5p	down
SLC24A4	down	hsa-miR-425-5p_R+2_1ss20TC	down
DHCR24	down	aca-miR-18a-3p_L+1R-1_1ss12GA	up
DHCR24	down	gga-let-7c-3p_1ss12TC	up
DHCR24	down	gga-miR-130b-5p	up
DHCR24	down	gga-miR-130b-5p_R-1	up
DHCR24	down	gga-miR-130b-5p_R-3	up
DHCR24	down	gga-miR-1467-3p_L+1	up

DHCR24	down	gga-miR-16-1-3p_R-1	up
DHCR24	down	gga-miR-1759-3p_L-3	up
DHCR24	down	gga-miR-1783_R+1	up
DHCR24	down	gga-miR-181b-1-3p_L+2R-2	up
DHCR24	down	gga-miR-202-5p_L-1	up
DHCR24	down	gga-miR-21-5p	down
DHCR24	down	gga-miR-222b-5p_R-4	down
DHCR24	down	gga-miR-458a-3p	down
DHCR24	down	gga-miR-6710-3p_R+1	up
DHCR24	down	hsa-miR-181b-3p_L+1R-1	up
DHCR24	down	hsa-miR-21-5p_R+2_1ss1TN	down
DHCR24	down	hsa-miR-4443_R+1	up
EREG	down	aca-miR-363-5p	up
EREG	down	gga-miR-130b-5p	up
EREG	down	gga-miR-130b-5p_R-1	up
EREG	down	gga-miR-130b-5p_R-3	up
EREG	down	gga-miR-1663-5p_R+2	up
EREG	down	gga-miR-1720-5p	up
HOOK1	up	gga-miR-1663-5p_R+2	up
HOOK1	up	gga-miR-21-5p	down
HOOK1	up	gga-miR-301a-5p_L+2	up
HOOK1	up	gga-miR-9-5p	down
FAM126A	down	gga-miR-106-3p_R+1	up
FAM126A	down	gga-miR-1552-3p_R+1	up
FAM126A	down	gga-miR-17-3p_L-1R+3	up
FAM126A	down	gga-miR-21-5p	down

FAM126A	down	gga-miR-2131-3p_R+1	up
FAM126A	down	gga-miR-22-3p	down
FAM126A	down	gga-miR-9-5p	down
FAM126A	down	mmu-miR-8094_L+1R+3_1ss3AG	up
KANK4	up	gga-miR-456-3p_1ss22AT	up
KANK4	up	gga-miR-456-3p_R-1	up
KANK4	up	pma-miR-456_R+1_1ss22AC	up
IGF2BP3	up	cgr-miR-151-3p	up
IGF2BP3	up	cgr-miR-500	up
IGF2BP3	up	csa-let-7d_1ss10TC	up
IGF2BP3	up	gga-let-7a-5p	down
IGF2BP3	up	gga-let-7a-5p_2ss12GT18TC	down
IGF2BP3	up	gga-let-7b_L-1R-1	down
IGF2BP3	up	gga-let-7b_R+2	down
IGF2BP3	up	gga-let-7c-5p_1ss17AG	down
IGF2BP3	up	gga-let-7g-5p_R+1_2ss14TG18CA	down
IGF2BP3	up	gga-let-7k-5p_R+1_1ss16AT	down
IGF2BP3	up	gga-miR-107-3p_R-1	up
IGF2BP3	up	gga-miR-142-5p_L-2R+1	down
IGF2BP3	up	gga-miR-16-1-3p_R-1	up
IGF2BP3	up	gga-miR-1729-5p_R-1	down
IGF2BP3	up	gga-miR-196-5p_R+2	down
IGF2BP3	up	gga-miR-29a-5p_L-1R-2	down
IGF2BP3	up	gga-miR-34a-3p_R-1	up
IGF2BP3	up	gga-miR-9-5p	down
IGF2BP3	up	mmu-let-7j_1ss8TG	down

IGF2BP3	up	mmu-miR-3964_L+1_1ss6GA	down
IGF2BP3	up	mmu-miR-500-3p_R-1	up
IGF2BP3	up	mmu-mir-5098-p5	up
IGF2BP3	up	ola-miR-199a-3p_R+1_1ss22AG	down
IGF2BP3	up	tgu-miR-1784-3p_1ss11GA	down
SIPA1L2	down	gga-miR-130b-5p	up
SIPA1L2	down	gga-miR-130b-5p_R-1	up
SIPA1L2	down	gga-miR-130b-5p_R-3	up
SIPA1L2	down	gga-miR-1329-5p	down
SIPA1L2	down	gga-miR-140-3p_L+1R+1_1ss22CA	down
SIPA1L2	down	gga-miR-222a	up
SIPA1L2	down	sha-miR-140_R+2	down
DNAJC6	up	gga-miR-15b-5p_R-1	up
DNAJC6	up	gga-miR-16c-5p	up
DNAJC6	up	hsa-miR-4286_R+1	up
HIBADH	up	gga-miR-106-3p_R+1	up
HIBADH	up	gga-miR-137-3p_L+1	up
HIBADH	up	gga-miR-142-5p_L-2R+1	down
HIBADH	up	gga-miR-1783_R+1	up
HIBADH	up	gga-miR-455-5p	up
HIBADH	up	mmu-miR-3964_L+1_1ss6GA	down
DPP4	down	dre-miR-24_R+2	down
DPP4	down	gga-miR-222b-5p_R-4	down
TAX1BP1	down	mmu-miR-8094_L+1R+3_1ss3AG	up
HHIPL1	down	gga-miR-1759-3p_L-3	up
HHIPL1	down	gga-miR-489-3p_L-2R+1	up

LY75	down	gga-miR-1329-5p	down
LY75	down	gga-miR-1754-5p	up
GNPAT	up	gga-miR-1329-5p	down
EVL	down	gga-miR-15b-5p_R-1	up
EVL	down	gga-miR-16c-5p	up
EVL	down	gga-miR-17-3p_L-1R+3	up
EVL	down	gga-miR-1788-3p_R+1	up
EVL	down	hsa-miR-143-5p	up
CDS1	down	gga-miR-383-3p	up
PLCL2	down	gga-miR-130b-3p	up
PLCL2	down	gga-miR-202-5p_L-1	up
PLCL2	down	gga-miR-454-3p	up
BTG1	down	gga-let-7c-3p_1ss12TC	up
BTG1	down	gga-miR-130b-3p	up
BTG1	down	gga-miR-181b-1-3p_L+1R-1	up
BTG1	down	gga-miR-2131-3p_R+1	up
BTG1	down	gga-miR-22-3p	down
BTG1	down	gga-miR-454-3p	up
KAT2B	down	aca-miR-150-5p_1ss12TC	down
KAT2B	down	aca-miR-181a_R+3	up
KAT2B	down	gga-miR-142-5p_L-2R+1	down
KAT2B	down	gga-miR-181a-5p	up
KAT2B	down	gga-miR-181b-5p_R+1	up
KAT2B	down	gga-miR-20b-5p	up
KAT2B	down	gga-miR-456-3p_1ss22AT	up
KAT2B	down	gga-miR-456-3p_R-1	up

KAT2B	down	gga-miR-460a-5p	up
KAT2B	down	mmu-miR-3964_L+1_1ss6GA	down
KAT2B	down	pma-miR-181a-5p_R+3	up
KAT2B	down	pma-miR-456_R+1_1ss22AC	up
CTH	up	gga-miR-214_L+1R-3_1ss19GA	down
CRYZ	up	aca-miR-18a-5p	up
CRYZ	up	gga-miR-142-5p_L-2R+1	down
CRYZ	up	gga-miR-15b-5p_R-1	up
CRYZ	up	gga-miR-16c-5p	up
CRYZ	up	gga-miR-1784-5p	down
CRYZ	up	gga-miR-1796	up
CRYZ	up	gga-miR-383-3p	up
CRYZ	up	gga-miR-6604-5p_1ss7GA	up
CRYZ	up	hsa-miR-143-5p	up
CRYZ	up	mmu-miR-3964_L+1_1ss6GA	down
CRYZ	up	odi-miR-7_R+2	down
CMTM7	down	gga-miR-214_L+1R-3_1ss19GA	down
SLC25A3	down	aca-miR-425-5p_R+4	down
SLC25A3	down	hsa-miR-425-5p_R+2_1ss20TC	down
BAG5	down	aca-miR-129b-5p_R+1_1ss20TG	up
BAG5	down	aca-miR-181a_R+3	up
BAG5	down	aca-miR-191-5p_R-1	down
BAG5	down	aca-miR-425-5p_R+4	down
BAG5	down	gga-let-7f-3p_1ss22CT	down
BAG5	down	gga-miR-107-5p_R+1	up
BAG5	down	gga-miR-138-5p_R+7	up

BAG5	down	gga-miR-146c-5p_4ss18GA20AG22T G23GT	down
BAG5	down	gga-miR-1559-3p	up
BAG5	down	gga-miR-1663-5p_R+2	up
BAG5	down	gga-miR-1668-3p	up
BAG5	down	gga-miR-1759-3p_L-3	up
BAG5	down	gga-miR-1788-3p_R+1	up
BAG5	down	gga-miR-181a-5p	up
BAG5	down	gga-miR-181b-5p_R+1	up
BAG5	down	gga-miR-214_L+1R-3_1ss19GA	down
BAG5	down	gga-miR-223_1ss15AG	down
BAG5	down	gga-miR-223_1ss19CT	down
BAG5	down	gga-miR-223_R+1	down
BAG5	down	gga-miR-383-3p	up
BAG5	down	gga-miR-458a-3p	down
BAG5	down	gga-miR-460a-3p	up
BAG5	down	gga-miR-460a-5p	up
BAG5	down	hsa-miR-3120-3p_R+1	down
BAG5	down	hsa-miR-425-5p_R+2_1ss20TC	down
BAG5	down	oha-miR-7-1-3p_1ss10GA	up
BAG5	down	pma-miR-181a-5p_R+3	up
BAG5	down	tni-miR-455_L+1_2ss22CT23GT	down
UHRF1BP1 L	up	PC-3p-68190_58	up
UHRF1BP1 L	up	cin-miR-33_R+4	down

UHRF1BP1L	up	gga-miR-29a-5p_L-1R-2	down
UHRF1BP1L	up	gga-miR-30a-3p_1ss18GA	up
UHRF1BP1L	up	gga-miR-30a-3p_2ss10GA20AT	up
UHRF1BP1L	up	gga-miR-30e-3p_L+1	up
UHRF1BP1L	up	gga-miR-33-5p	down
UHRF1BP1L	up	gga-miR-454-3p	up
UHRF1BP1L	up	oha-miR-7-1-3p_1ss10GA	up
ACAT2	down	gga-miR-135a-2-3p_L-1R+1	down
ACAT2	down	gga-miR-140-5p_L+1	down
LARS2	up	gga-miR-130b-5p	up
LARS2	up	gga-miR-130b-5p_R-1	up
LARS2	up	gga-miR-130b-5p_R-3	up
LARS2	up	gga-miR-15b-5p_R-1	up
LARS2	up	gga-miR-16c-5p	up
CCNA2	up	gga-miR-140-3p_L+1R+1_1ss22CA	down
CCNA2	up	gga-miR-1467-3p_L+1	up
CCNA2	up	gga-miR-1663-5p_R+2	up
CCNA2	up	gga-miR-222a	up
CCNA2	up	gga-miR-223_1ss15AG	down

CCNA2	up	gga-miR-383-3p	up
CCNA2	up	gga-miR-460a-5p	up
CCNA2	up	hsa-miR-143-5p	up
CCNA2	up	hsa-miR-3120-3p_R+1	down
CCNA2	up	sha-miR-140_R+2	down
CLEC3B	down	PC-3p-68190_58	up
CLEC3B	down	aca-miR-150-5p_1ss12TC	down
CLEC3B	down	gga-miR-15b-5p_R-1	up
CLEC3B	down	gga-miR-1699_L-1R-2	up
CLEC3B	down	gga-miR-16c-5p	up
CLEC3B	down	gga-miR-1784-5p	down
CLEC3B	down	gga-miR-181b-1-3p_L+1R-1	up
CLEC3B	down	gga-miR-181b-1-3p_L+2R-2	up
CLEC3B	down	gga-miR-21-5p	down
CLEC3B	down	gga-miR-214_L+1R-3_1ss19GA	down
CLEC3B	down	gga-miR-30c-1-3p_R+1	up
CLEC3B	down	gga-miR-6604-5p_1ss7GA	up
CLEC3B	down	gga-miR-7460-3p	up
CLEC3B	down	hsa-miR-181b-3p_L+1R-1	up
CLEC3B	down	hsa-miR-21-5p_R+2_1ss1TN	down
CLEC3B	down	mmu-miR-6412_R-2_1ss15AT	down
DHRS7	up	gga-miR-214_L+1R-3_1ss19GA	down
ACO2	up	aca-miR-363-3p_R+1	down
ACO2	up	gga-miR-1552-3p_R+1	up
ACO2	up	gga-miR-1555-5p	up
ACO2	up	gga-miR-1559-3p	up

ACO2	up	oan-miR-363-3p_R+1	up
CCR8	down	aca-miR-18a-5p	up
CCR8	down	aca-miR-363-3p_R+1	down
CCR8	down	gga-miR-1555-5p	up
CCR8	down	gga-miR-15b-3p_L+1_1ss22AC	up
CCR8	down	gga-miR-1663-5p_R+2	up
CCR8	down	gga-miR-1784-5p	down
CCR8	down	gga-miR-1796	up
CCR8	down	gga-miR-22-3p	down
CCR8	down	gga-miR-2954_R+2	down
CCR8	down	gga-miR-30a-3p_1ss18GA	up
CCR8	down	gga-miR-30a-3p_2ss10GA20AT	up
CCR8	down	gga-miR-30c-1-3p_R+1	up
CCR8	down	gga-miR-30e-3p_L+1	up
CCR8	down	gga-miR-92-5p	up
CCR8	down	oan-miR-363-3p_R+1	up
CCR8	down	rno-miR-122-5p_L+1R+1_1	down
CCR8	down	rno-miR-122-5p_L+1R+1_2	down
MAD2L1	up	PC-5p-479025_5	up
MAD2L1	up	gga-let-7j-3p_L-1R+2	down
MAD2L1	up	gga-miR-1552-3p_R+1	up
MAD2L1	up	gga-miR-1559-3p	up
MAD2L1	up	gga-miR-301a-5p_L+2	up
MAD2L1	up	gga-miR-30c-1-3p_R+1	up
MAD2L1	up	gga-miR-429-3p	up
MAD2L1	up	gga-miR-7460-3p	up

MAD2L1	up	oan-let-7f-1-3p_R+2_1ss8AG	down
MAD2L1	up	xtr-miR-429_R+2	down
CAMK2D	down	aca-miR-181a_R+3	up
CAMK2D	down	chi-miR-361-5p	up
CAMK2D	down	efu-miR-361	up
CAMK2D	down	gga-let-7f-3p_1ss22CT	down
CAMK2D	down	gga-miR-128-1-5p	up
CAMK2D	down	gga-miR-1559-3p	up
CAMK2D	down	gga-miR-1663-5p_R+2	up
CAMK2D	down	gga-miR-1796	up
CAMK2D	down	gga-miR-181a-5p	up
CAMK2D	down	gga-miR-181b-5p_R+1	up
CAMK2D	down	gga-miR-205a_R+1	up
CAMK2D	down	gga-miR-205b	up
CAMK2D	down	gga-miR-22-3p	down
CAMK2D	down	gga-miR-30b-5p	up
CAMK2D	down	gga-miR-30c-5p	up
CAMK2D	down	gga-miR-383-3p	up
CAMK2D	down	gga-miR-460a-3p	up
CAMK2D	down	hsa-miR-3120-3p_R+1	down
CAMK2D	down	mmu-mir-5098-p5	up
CAMK2D	down	pma-miR-181a-5p_R+3	up
CAMK2D	down	tni-miR-205_R+2	up
DTX3L	down	gga-miR-1559-3p	up
DTX3L	down	gga-miR-1796	up
DTX3L	down	gga-miR-20b-5p	up

DTX3L	down	gga-miR-214_L+1R-3_1ss19GA	down
DTX3L	down	gga-miR-223_1ss15AG	down
DTX3L	down	gga-miR-223_1ss19CT	down
DTX3L	down	gga-miR-223_R+1	down
DTX3L	down	gga-miR-460a-5p	up
DTX3L	down	mmu-miR-8094_L+1R+3_1ss3AG	up
ENPEP	down	aca-miR-18a-5p	up
ENPEP	down	gga-miR-125b-5p	down
ENPEP	down	gga-miR-137-3p_L+1	up
ENPEP	down	gga-miR-146b-3p_L+2	down
ENPEP	down	sha-miR-125a_R+2	down
ENPEP	down	ssa-miR-125a-5p_R+2	down
WDHD1	up	aca-miR-129b-5p_R+1_1ss20TG	up
WDHD1	up	gga-miR-1552-3p_R+1	up
WDHD1	up	gga-miR-29b-3p_R-1	down
WDHD1	up	gga-miR-29c-3p_L+1R+1_1ss19GA	down
WDHD1	up	gga-miR-29c-3p_R+1	down
WDHD1	up	gga-miR-29c-3p_R+1_1ss18GA	down
WDHD1	up	ssa-miR-29b-3p_R-1_2ss20TC21TC	down
MICALL1	down	gga-miR-1759-3p_L-3	up
MICALL1	down	gga-miR-1788-3p_R+1	up
RAB32	down	gga-miR-107-3p_R-1	up
RAB32	down	gga-miR-122-5p	down
RAB32	down	gga-miR-146c-5p_4ss18GA20AG22TG23GT	down
RAB32	down	gga-miR-15b-5p_R-1	up

RAB32	down	gga-miR-16c-5p	up
RAB32	down	gga-miR-21-5p	down
RAB32	down	gga-miR-29b-3p_R-1	down
RAB32	down	gga-miR-29c-3p_R+1	down
RAB32	down	gga-miR-29c-3p_R+1_1ss18GA	down
RAB32	down	hsa-miR-21-5p_R+2_1ss1TN	down
CXCR4	down	gga-let-7c-3p_1ss12TC	up
IYD	up	aca-miR-18a-5p	up
IYD	up	cgr-miR-500	up
IYD	up	gga-miR-155_R+1	down
IYD	up	gga-miR-15b-5p_R-1	up
IYD	up	gga-miR-16c-5p	up
IYD	up	gga-miR-2131-3p_R+1	up
IYD	up	gga-miR-30a-5p_R+2_1ss13CT	up
IYD	up	gga-miR-30b-5p	up
IYD	up	gga-miR-30c-5p	up
IYD	up	gga-miR-30d_R+2	up
IYD	up	gga-miR-30e-5p_R+5	up
IYD	up	mmu-miR-500-3p_R-1	up
IYD	up	tgu-miR-30a-5p_R+2	up
MYH9	down	gga-miR-490-3p_R+1	up
MYH9	down	gga-miR-6604-5p_1ss7GA	up
RBFOX2	up	mmu-mir-5098-p5	up
TIMP3	down	gga-miR-1699_L-1R-2	up
TIMP3	down	gga-miR-30c-1-3p_R+1	up
ISCA1	down	PC-3p-68190_58	up

ISCA1	down	aca-miR-363-3p_R+1	down
ISCA1	down	chi-miR-361-5p	up
ISCA1	down	efu-miR-361	up
ISCA1	down	gga-let-7j-3p_L-1R+2	down
ISCA1	down	gga-miR-1329-5p	down
ISCA1	down	gga-miR-140-5p_L+1	down
ISCA1	down	gga-miR-142-5p_L-2R+1	down
ISCA1	down	gga-miR-144-3p_L-1R-2	up
ISCA1	down	gga-miR-1552-3p_R+1	up
ISCA1	down	gga-miR-202-5p_L-1	up
ISCA1	down	gga-miR-2131-3p_R+1	up
ISCA1	down	gga-miR-2954_R+2	down
ISCA1	down	gga-miR-429-5p_L-1	up
ISCA1	down	gga-miR-455-5p	up
ISCA1	down	gga-miR-458a-3p	down
ISCA1	down	gga-miR-6548-5p	up
ISCA1	down	gga-miR-9-5p	down
ISCA1	down	mmu-miR-3964_L+1_1ss6GA	down
ISCA1	down	oan-miR-363-3p_R+1	up
ISCA1	down	odi-miR-7_R+2	down
ISCA1	down	rno-miR-122-5p_L+1R+1_1	down
ISCA1	down	rno-miR-122-5p_L+1R+1_2	down
ISCA1	down	tgu-miR-1784-3p_1ss11GA	down
CTSL2	down	aca-miR-181a_R+3	up
CTSL2	down	gga-miR-181a-5p	up
CTSL2	down	gga-miR-181b-5p_R+1	up

CTSL2	down	pma-miR-181a-5p_R+3	up
PWP1	up	aca-miR-150-5p_1ss12TC	down
PWP1	up	gga-miR-106-3p_R+1	up
PWP1	up	gga-miR-125b-3p_R-1_1ss21CT	up
PWP1	up	gga-miR-130b-5p	up
PWP1	up	gga-miR-130b-5p_R-1	up
PWP1	up	gga-miR-130b-5p_R-3	up
PWP1	up	gga-miR-142-3p	down
PWP1	up	gga-miR-1555-5p	up
PWP1	up	gga-miR-16c-5p	up
PWP1	up	gga-miR-1784-5p	down
PWP1	up	gga-miR-301a-5p_L+2	up
PWP1	up	gga-miR-30a-3p_1ss18GA	up
PWP1	up	gga-miR-30a-3p_2ss10GA20AT	up
PWP1	up	gga-miR-30e-3p_L+1	up
PWP1	up	gga-miR-456-3p_1ss22AT	up
PWP1	up	gga-miR-456-3p_R-1	up
PWP1	up	gga-miR-7460-3p	up
PWP1	up	gga-miR-92-5p	up
PWP1	up	hsa-miR-4286_R+1	up
PWP1	up	pma-miR-456_R+1_1ss22AC	up
RBM24	down	gga-let-7j-3p_L-1R+2	down
RBM24	down	gga-miR-106-3p_R+1	up
RBM24	down	gga-miR-107-3p_R-1	up
RBM24	down	gga-miR-125b-3p_R-1_1ss21CT	up
RBM24	down	gga-miR-144-3p_L-1R-2	up

RBM24	down	gga-miR-1559-3p	up
RBM24	down	gga-miR-1559-5p	up
RBM24	down	gga-miR-15b-5p_R-1	up
RBM24	down	gga-miR-1699_L-1R-2	up
RBM24	down	gga-miR-16c-5p	up
RBM24	down	gga-miR-1729-5p_R-1	down
RBM24	down	gga-miR-222a	up
RBM24	down	gga-miR-383-3p	up
RBM24	down	gga-miR-6604-5p_1ss7GA	up
RBM24	down	gga-mir-147-p3_1	down
RBM24	down	gga-mir-147-p5	down
RBM24	down	oan-miR-19b-1-5p_R+4	up
ELOVL2	up	gga-miR-20b-3p_R-1	up
ELOVL2	up	mmu-mir-5098-p5	up
SSR1	up	gga-miR-15b-5p_R-1	up
SSR1	up	gga-miR-1668-3p	up
SSR1	up	gga-miR-16c-5p	up
SSR1	up	gga-miR-1796	up
SSR1	up	gga-miR-214_L+1R-3_1ss19GA	down
SSR1	up	gga-miR-30a-3p_1ss18GA	up
SSR1	up	gga-miR-30a-3p_2ss10GA20AT	up
SSR1	up	gga-miR-30e-3p_L+1	up
SSR1	up	gga-miR-6710-3p_R+1	up
TRIM24	down	gga-let-7f-3p_1ss22CT	down
TRIM24	down	gga-miR-1329-5p	down
TRIM24	down	gga-miR-16-1-3p_R-1	up

TRIM24	down	gga-miR-9-5p	down
AKR1D1	up	csa-let-7d_1ss10TC	up
AKR1D1	up	gga-let-7a-5p	down
AKR1D1	up	gga-let-7a-5p_2ss12GT18TC	down
AKR1D1	up	gga-let-7b_R+2	down
AKR1D1	up	gga-let-7c-5p_1ss17AG	down
AKR1D1	up	gga-let-7g-5p_R+1_2ss14TG18CA	down
AKR1D1	up	gga-let-7k-5p_R+1_1ss16AT	down
AKR1D1	up	gga-miR-155_R+1	down
AKR1D1	up	gga-miR-22-3p	down
AKR1D1	up	gga-miR-29b-3p_R-1	down
AKR1D1	up	gga-miR-29c-3p_R+1	down
AKR1D1	up	gga-miR-29c-3p_R+1_1ss18GA	down
AKR1D1	up	mmu-let-7j_1ss8TG	down
AKR1D1	up	ssa-miR-29b-3p_R-1_2ss20TC21TC	down
SERPINB6	down	gga-miR-130b-3p	up
SERPINB6	down	gga-miR-1451-3p_R-1	up
SERPINB6	down	gga-miR-1552-3p_R+1	up
SERPINB6	down	gga-miR-1668-3p	up
SERPINB6	down	gga-miR-1788-3p_R+1	up
SERPINB6	down	gga-miR-181b-1-3p_L+1R-1	up
SERPINB6	down	gga-miR-454-3p	up
SERPINB6	down	gga-miR-460a-5p	up
SERPINB6	down	gga-miR-6604-5p_1ss7GA	up
SERPINB6	down	gga-mir-147-p3_1	down
SERPINB6	down	gga-mir-147-p5	down

SERPINB6	down	hsa-miR-3120-3p_R+1	down
SERPINB6	down	mmu-mir-5098-p5	up
SERPINB10	down	aca-miR-129b-5p_R+1_1ss20TG	up
SERPINB10	down	csa-let-7d_1ss10TC	up
SERPINB10	down	gga-let-7a-5p	down
SERPINB10	down	gga-let-7a-5p_2ss12GT18TC	down
SERPINB10	down	gga-let-7b_R+2	down
SERPINB10	down	gga-let-7c-5p_1ss17AG	down
SERPINB10	down	gga-let-7g-5p_R+1_2ss14TG18CA	down
SERPINB10	down	gga-let-7k-5p_R+1_1ss16AT	down
SERPINB10	down	gga-miR-16c-3p_L+1R-1	up
SERPINB10	down	gga-miR-2131-3p_R+1	up
SERPINB10	down	gga-miR-30a-3p_1ss18GA	up
SERPINB10	down	gga-miR-30a-3p_2ss10GA20AT	up

SERPINB1<br>0	down	gga-miR-30e-3p_L+1	up
SERPINB1<br>0	down	gga-miR-92-5p	up
SERPINB1<br>0	down	mmu-let-7j_1ss8TG	down
SERPINB1<br>0	down	mmu-miR-8094_L+1R+3_1ss3AG	up
MCM4	up	gga-miR-107-3p_R-1	up
MCM4	up	gga-miR-205a_R+1	up
MCM4	up	gga-miR-205b	up
MCM4	up	hsa-miR-3120-3p_R+1	down
MCM4	up	tni-miR-205_R+2	up
DENND5B	up	gga-miR-146a-3p_R-1	down
BPGM	up	aca-miR-18a-3p_L+1R-1_1ss12GA	up
BPGM	up	cin-miR-33_R+4	down
BPGM	up	gga-miR-107-3p_R-1	up
BPGM	up	gga-miR-130b-3p	up
BPGM	up	gga-miR-1552-5p_R+1	up
BPGM	up	gga-miR-15b-5p_R-1	up
BPGM	up	gga-miR-16c-5p	up
BPGM	up	gga-miR-1783_R+1	up
BPGM	up	gga-miR-181b-1-3p_L+1R-1	up
BPGM	up	gga-miR-181b-1-3p_L+2R-2	up
BPGM	up	gga-miR-214_L+1R-3_1ss19GA	down
BPGM	up	gga-miR-216b	up

BPGM	up	gga-miR-22-3p	down
BPGM	up	gga-miR-29b-3p_R-1	down
BPGM	up	gga-miR-29c-3p_R+1	down
BPGM	up	gga-miR-29c-3p_R+1_1ss18GA	down
BPGM	up	gga-miR-30a-3p_1ss18GA	up
BPGM	up	gga-miR-30a-3p_2ss10GA20AT	up
BPGM	up	gga-miR-30e-3p_L+1	up
BPGM	up	gga-miR-33-5p	down
BPGM	up	gga-miR-454-3p	up
BPGM	up	gga-miR-460a-5p	up
BPGM	up	gga-miR-490-3p_R+1	up
BPGM	up	hsa-miR-181b-3p_L+1R-1	up
BPGM	up	hsa-miR-3120-3p_R+1	down
BPGM	up	hsa-miR-4286_R+1	up
BPGM	up	oan-miR-19b-1-5p_R+4	up
BPGM	up	ola-miR-199a-3p_R+1_1ss22AG	down
BPGM	up	ssa-miR-29b-3p_R-1_2ss20TC21TC	down
CCDC127	down	aca-miR-129b-5p_R+1_1ss20TG	up
CCDC127	down	aca-miR-363-3p_R+1	down
CCDC127	down	gga-miR-106-3p_R+1	up
CCDC127	down	gga-miR-17-3p_L-1R+3	up
CCDC127	down	gga-miR-1747-3p	up
CCDC127	down	gga-miR-181b-1-3p_L+2R-2	up
CCDC127	down	gga-miR-456-3p_1ss22AT	up
CCDC127	down	gga-miR-456-3p_R-1	up
CCDC127	down	hsa-miR-181b-3p_L+1R-1	up

CCDC127	down	oan-miR-19b-1-5p_R+4	up
CCDC127	down	oan-miR-363-3p_R+1	up
CCDC127	down	odi-miR-7_R+2	down
CCDC127	down	pma-miR-456_R+1_1ss22AC	up
PYROXD1	up	aca-miR-363-3p_R+1	down
PYROXD1	up	gga-miR-1329-5p	down
PYROXD1	up	gga-miR-2954_R+2	down
PYROXD1	up	gga-miR-460a-5p	up
PYROXD1	up	oan-miR-363-3p_R+1	up
PYROXD1	up	tgu-miR-1662_L+1_1ss23TA	down
GABBR2	down	gga-let-7j-3p_L-1R+2	down
GABBR2	down	gga-miR-144-3p_L-1R-2	up
GABBR2	down	gga-miR-222b-5p_R-4	down
GABBR2	down	gga-miR-30a-3p_1ss18GA	up
GABBR2	down	gga-miR-30a-3p_2ss10GA20AT	up
GABBR2	down	gga-miR-30e-3p_L+1	up
GABBR2	down	ggo-miR-342_R+1	up
ST8SIA1	down	aca-miR-150-5p_1ss12TC	down
ST8SIA1	down	gga-miR-216b	up
RHBG	down	gga-miR-107-3p_R-1	up
RHBG	down	gga-miR-138-5p_R+7	up
RHBG	down	gga-miR-1729-5p_R-1	down
RHBG	down	gga-miR-1747-3p	up
RHBG	down	gga-miR-383-3p	up
RHBG	down	gga-miR-6604-5p_1ss7GA	up
NCLN	up	dre-miR-24_R+2	down

NCLN	up	gga-miR-222b-5p_R-4	down
PDLIM3	down	gga-let-7c-3p_1ss12TC	up
PDLIM3	down	gga-miR-107-5p_R+1	up
PDLIM3	down	gga-miR-2131-3p_R+1	up
PDLIM3	down	gga-miR-365-2-5p_L-1R+1	up
PLEKHO1	down	gga-miR-125b-5p	down
PLEKHO1	down	gga-miR-144-3p_L-1R-2	up
PLEKHO1	down	gga-miR-1663-5p_R+2	up
PLEKHO1	down	gga-miR-1668-3p	up
PLEKHO1	down	gga-miR-214_L+1R-3_1ss19GA	down
PLEKHO1	down	gga-miR-29c-3p_L+1R+1_1ss19GA	down
PLEKHO1	down	gga-miR-3607-5p_R+1	up
PLEKHO1	down	hsa-miR-143-5p	up
PLEKHO1	down	hsa-miR-3120-3p_R+1	down
PLEKHO1	down	sha-miR-125a_R+2	down
PLEKHO1	down	ssa-miR-125a-5p_R+2	down
MED24	up	gga-miR-301a-5p_L+2	up
ANKRA2	down	gga-miR-146c-5p_4ss18GA20AG22TG23GT	down
ANKRA2	down	gga-miR-1662_R-3	down
ANKRA2	down	gga-miR-489-3p_L-2R+1	up
ANKRA2	down	gga-miR-6548-5p	up
ANKRA2	down	mmu-miR-8094_L+1R+3_1ss3AG	up
MTHFD2	up	PC-5p-479025_5	up
MTHFD2	up	aca-miR-150-5p_1ss12TC	down
MTHFD2	up	aca-miR-363-3p_R+1	down

MTHFD2	up	gga-let-7c-3p_1ss12TC	up
MTHFD2	up	gga-miR-122-5p	down
MTHFD2	up	gga-miR-222b-5p_R-4	down
MTHFD2	up	oan-miR-363-3p_R+1	up
CYB5A	up	gga-miR-107-5p_R+1	up
CYB5A	up	gga-miR-146a-3p_R-1	down
CYB5A	up	gga-miR-20b-5p	up
CYB5A	up	gga-miR-214_L+1R-3_1ss19GA	down
CYB5A	up	gga-miR-29b-3p_R-1	down
CYB5A	up	gga-miR-29c-3p_R+1	down
CYB5A	up	gga-miR-29c-3p_R+1_1ss18GA	down
CYB5A	up	gga-miR-30a-3p_1ss18GA	up
CYB5A	up	gga-miR-30a-3p_2ss10GA20AT	up
CYB5A	up	gga-miR-30e-3p_L+1	up
CYB5A	up	gga-miR-383-3p	up
CYB5A	up	gga-miR-6548-5p	up
CYB5A	up	gga-mir-147-p3_1	down
CYB5A	up	gga-mir-147-p5	down
CYB5A	up	hsa-miR-3120-3p_R+1	down
CYB5A	up	ssa-miR-29b-3p_R-1_2ss20TC21TC	down
SNX9	down	aca-miR-425-5p_R+4	down
SNX9	down	gga-miR-29c-3p_L+1R+1_1ss19GA	down
SNX9	down	gga-miR-30c-1-3p_R+1	up
SNX9	down	gga-miR-30d_R+2	up
SNX9	down	hsa-miR-425-5p_R+2_1ss20TC	down
SNX9	down	tgu-miR-30a-5p_R+2	up

LONRF1	down	gga-miR-130b-3p	up
LONRF1	down	gga-miR-1329-5p	down
LONRF1	down	gga-miR-1788-3p_R+1	up
LONRF1	down	gga-miR-454-3p	up
LONRF1	down	gga-miR-460a-5p	up
LONRF1	down	hsa-miR-3120-3p_R+1	down
GFPT2	down	gga-miR-365-2-5p_L-1R+1	up
IMPA2	up	gga-miR-1552-3p_R+1	up
IMPA2	up	gga-miR-1559-3p	up
IMPA2	up	gga-miR-216b	up
PTPN2	down	gga-miR-138-5p_R+7	up
PTPN2	down	gga-miR-1552-3p_R+1	up
PTPN2	down	gga-miR-155_R+1	down
PTPN2	down	gga-miR-30a-5p_R+2_1ss13CT	up
PTPN2	down	gga-miR-30b-5p	up
PTPN2	down	gga-miR-30d_R+2	up
PTPN2	down	gga-miR-30e-5p_R+5	up
PTPN2	down	gga-miR-6548-5p	up
PTPN2	down	tgu-miR-30a-5p_R+2	up
SYTL2	down	aca-miR-150-5p_1ss12TC	down
SYTL2	down	gga-miR-20b-5p	up
SYTL2	down	gga-miR-30a-3p_1ss18GA	up
SYTL2	down	gga-miR-30e-3p_L+1	up
SYTL2	down	gga-miR-454-3p	up
FRYL	down	aca-miR-18a-5p	up
FRYL	down	mmu-mir-5098-p5	up

TEC	down	aca-miR-129b-5p_R+1_1ss20TG	up
TEC	down	aca-miR-181a_R+3	up
TEC	down	dre-miR-24_R+2	down
TEC	down	gga-let-7j-3p_L-1R+2	down
TEC	down	gga-miR-107-3p_R-1	up
TEC	down	gga-miR-1329-5p	down
TEC	down	gga-miR-137-3p_L+1	up
TEC	down	gga-miR-15b-5p_R-1	up
TEC	down	gga-miR-16c-5p	up
TEC	down	gga-miR-223_1ss15AG	down
TEC	down	gga-miR-223_1ss19CT	down
TEC	down	gga-miR-223_R+1	down
TEC	down	gga-miR-429-3p	up
TEC	down	gga-miR-6710-3p_R+1	up
TEC	down	pma-miR-181a-5p_R+3	up
TEC	down	xtr-miR-429_R+2	down
BIK	down	gga-miR-137-3p_L+1	up
BIK	down	gga-miR-214_L+1R-3_1ss19GA	down
BIK	down	gga-miR-22-3p	down
BIK	down	gga-miR-490-3p_R+1	up
BIK	down	ggo-miR-342_R+1	up
CORO6	up	dre-miR-24_R+2	down
CORO6	up	gga-miR-144-3p_L-1R-2	up
CORO6	up	gga-miR-429-3p	up
CORO6	up	xtr-miR-429_R+2	down
FAM162A	up	gga-miR-125b-3p_R-1_1ss21CT	up

FAM162A	up	gga-miR-3607-5p_R+1	up
FAM162A	up	mmu-miR-8094_L+1R+3_1ss3AG	up
TAPBPL	down	chi-miR-361-5p	up
TAPBPL	down	efu-miR-361	up
TAPBPL	down	gga-miR-1306-3p_R-1	up
TAPBPL	down	gga-miR-146a-3p_R-1	down
TAPBPL	down	gga-miR-1729-5p_R-1	down
TAPBPL	down	gga-miR-214_L+1R-3_1ss19GA	down
TAPBPL	down	gga-miR-22-3p	down
TAPBPL	down	gga-miR-222b-5p_R-4	down
TAPBPL	down	gga-miR-7460-3p	up
TAPBPL	down	ggo-miR-342_R+1	up
TAPBPL	down	tgu-miR-1662_L+1_1ss23TA	down
GAPDH	up	gga-miR-1759-3p_L-3	up
GAPDH	up	gga-miR-1788-3p_R+1	up
GAPDH	up	gga-miR-181b-1-3p_L+2R-2	up
GAPDH	up	hsa-miR-181b-3p_L+1R-1	up
CD38	down	gga-miR-107-3p_R-1	up
CD38	down	gga-miR-15b-5p_R-1	up
CD38	down	gga-miR-16c-5p	up
CD38	down	gga-miR-222a	up
CD38	down	gga-miR-30a-3p_1ss18GA	up
CD38	down	gga-miR-30e-3p_L+1	up
CD38	down	gga-miR-3607-5p_R+1	up
CD38	down	gga-miR-383-3p	up
TPI1	up	gga-miR-15b-5p_R-1	up

TPI1	up	gga-miR-1602	up
TPI1	up	gga-miR-16c-5p	up
TPI1	up	gga-miR-22-3p	down
TPI1	up	ggo-miR-342_R+1	up
TPI1	up	mmu-miR-8094_L+1R+3_1ss3AG	up
ATN1	up	gga-miR-1467-3p_L+1	up
ATN1	up	gga-miR-17-3p_L-1R+3	up
ATN1	up	gga-miR-1784-5p	down
ATN1	up	gga-miR-200b-5p_L+2	down
PTPN6	down	PC-5p-479025_5	up
PTPN6	down	gga-miR-1467-3p_L+1	up
PTPN6	down	gga-miR-15b-5p_R-1	up
PTPN6	down	gga-miR-1783_R+1	up
PTPN6	down	mmu-miR-8094_L+1R+3_1ss3AG	up
LPCAT3	up	gga-miR-122-5p	down
LPCAT3	up	gga-miR-383-3p	up
LPCAT3	up	hsa-miR-3120-3p_R+1	down
LPCAT3	up	rno-miR-122-5p_L+1R+1_1	down
LPCAT3	up	rno-miR-122-5p_L+1R+1_2	down
CCLI10	down	PC-5p-479025_5	up
CCLI10	down	gga-miR-15b-3p_L+1_1ss22AC	up
CCLI10	down	gga-miR-1747-3p	up
C1S	down	gga-miR-1329-5p	down
C1S	down	gga-miR-1699_L-1R-2	up
C1S	down	oan-let-7f-1-3p_R+2_1ss8AG	down
MBLAC2	down	aca-miR-129b-5p_R+1_1ss20TG	up

MBLAC2	down	aca-miR-181a_R+3	up
MBLAC2	down	aca-miR-18a-3p_L+1R-1_1ss12GA	up
MBLAC2	down	cgr-miR-151-3p	up
MBLAC2	down	cgr-miR-500	up
MBLAC2	down	gga-miR-130b-5p	up
MBLAC2	down	gga-miR-130b-5p_R-1	up
MBLAC2	down	gga-miR-130b-5p_R-3	up
MBLAC2	down	gga-miR-142-5p_L-2R+1	down
MBLAC2	down	gga-miR-15b-5p_R-1	up
MBLAC2	down	gga-miR-1668-3p	up
MBLAC2	down	gga-miR-16c-5p	up
MBLAC2	down	gga-miR-17-3p_L-1R+3	up
MBLAC2	down	gga-miR-1783_R+1	up
MBLAC2	down	gga-miR-1788-5p	up
MBLAC2	down	gga-miR-1796	up
MBLAC2	down	gga-miR-181a-5p	up
MBLAC2	down	gga-miR-181b-5p_R+1	up
MBLAC2	down	gga-miR-21-5p	down
MBLAC2	down	gga-miR-222a	up
MBLAC2	down	gga-miR-490-3p_R+1	up
MBLAC2	down	gga-miR-9-5p	down
MBLAC2	down	hsa-miR-21-5p_R+2_1ss1TN	down
MBLAC2	down	hsa-miR-4443_R+1	up
MBLAC2	down	mmu-miR-3964_L+1_1ss6GA	down
MBLAC2	down	mmu-miR-500-3p_R-1	up
MBLAC2	down	mmu-mir-5098-p5	up

MBLAC2	down	pma-miR-181a-5p_R+3	up
RFESD	down	PC-5p-479025_5	up
RFESD	down	dre-miR-24_R+2	down
RFESD	down	gga-miR-107-5p_R+1	up
RFESD	down	gga-miR-1329-5p	down
RFESD	down	gga-miR-1559-3p	up
RFESD	down	gga-miR-200b-5p_L+2	down
RFESD	down	gga-miR-222b-5p_R-4	down
RFESD	down	gga-miR-2954_R+2	down
RFESD	down	gga-miR-29a-5p_L-1R-2	down
RFESD	down	gga-miR-6548-5p	up
RFESD	down	gga-miR-6604-5p_1ss7GA	up
PHAX	down	gga-miR-222b-5p_R-4	down
DEPDC1B	up	gga-miR-125b-3p_R-1_1ss21CT	up
DEPDC1B	up	gga-miR-130b-3p	up
DEPDC1B	up	gga-miR-144-3p_L-1R-2	up
DEPDC1B	up	gga-miR-1559-3p	up
DEPDC1B	up	gga-miR-16-1-3p_R-1	up
DEPDC1B	up	gga-miR-16c-3p_L+1R-1	up
DEPDC1B	up	gga-miR-214_L+1R-3_1ss19GA	down
DEPDC1B	up	gga-miR-2954_R+2	down
DEPDC1B	up	gga-miR-29b-3p_R-1	down
DEPDC1B	up	gga-miR-29c-3p_R+1	down
DEPDC1B	up	gga-miR-29c-3p_R+1_1ss18GA	down
DEPDC1B	up	gga-miR-429-3p	up
DEPDC1B	up	gga-miR-6548-5p	up

DEPDC1B	up	hsa-miR-3120-3p_R+1	down
DEPDC1B	up	oan-let-7f-1-3p_R+2_1ss8AG	down
DEPDC1B	up	ssa-miR-29b-3p_R-1_2ss20TC21TC	down
DEPDC1B	up	xtr-miR-429_R+2	down
NLN	up	gga-miR-137-3p_L+1	up
NLN	up	gga-miR-2954_R+2	down
C7	down	aca-miR-363-5p	up
C7	down	gga-miR-146a-3p_R-1	down
C7	down	gga-miR-383-3p	up
FABP7	up	hsa-miR-3120-3p_R+1	down
FABP7	up	oan-let-7f-1-3p_R+2_1ss8AG	down
PARP8	down	PC-3p-68190_58	up
PARP8	down	aca-miR-129b-5p_R+1_1ss20TG	up
WDR1	down	gga-let-7j-3p_L-1R+2	down
WDR1	down	gga-miR-106-3p_R+1	up
WDR1	down	gga-miR-125b-3p_R-1_1ss21CT	up
WDR1	down	gga-miR-1552-3p_R+1	up
WDR1	down	gga-miR-155_R+1	down
WDR1	down	gga-miR-15b-5p_R-1	up
WDR1	down	gga-miR-16c-5p	up
WDR1	down	gga-miR-30a-5p_R+2_1ss13CT	up
WDR1	down	gga-miR-30b-5p	up
WDR1	down	gga-miR-30c-5p	up
WDR1	down	gga-miR-30d_R+2	up
WDR1	down	gga-miR-30e-5p_R+5	up
WDR1	down	gga-miR-460a-5p	up

WDR1	down	gga-miR-6548-5p	up
WDR1	down	hsa-miR-143-5p	up
WDR1	down	oan-let-7f-1-3p_R+2_1ss8AG	down
WDR1	down	tgu-miR-1662_L+1_1ss23TA	down
WDR1	down	tgu-miR-1784-3p_1ss11GA	down
WDR1	down	tgu-miR-30a-5p_R+2	up
F2RL1	down	gga-miR-107-3p_R-1	up
F2RL1	down	gga-miR-125b-5p	down
F2RL1	down	gga-miR-1329-5p	down
F2RL1	down	gga-miR-135a-2-3p_L-1R+1	down
F2RL1	down	gga-miR-144-3p_L-1R-2	up
F2RL1	down	gga-miR-1559-3p	up
F2RL1	down	gga-miR-1662_R-3	down
F2RL1	down	gga-miR-17-3p_L-1R+3	up
F2RL1	down	gga-miR-1747-3p	up
F2RL1	down	gga-miR-1783_R+1	up
F2RL1	down	gga-miR-181b-1-3p_L+1R-1	up
F2RL1	down	gga-miR-216b	up
F2RL1	down	gga-miR-22-3p	down
F2RL1	down	gga-miR-223_1ss15AG	down
F2RL1	down	gga-miR-223_1ss19CT	down
F2RL1	down	gga-miR-223_R+1	down
F2RL1	down	gga-miR-2954_R+2	down
F2RL1	down	gga-miR-6548-5p	up
F2RL1	down	gga-miR-6710-3p_R+1	up
F2RL1	down	hsa-miR-3120-3p_R+1	down

F2RL1	down	sha-miR-125a_R+2	down
F2RL1	down	ssa-miR-125a-5p_R+2	down
F2RL1	down	tgu-miR-1662_L+1_1ss23TA	down
JAK2	down	gga-miR-214_L+1R-3_1ss19GA	down
JAK2	down	gga-miR-3607-5p_R+1	up
JAK2	down	gga-miR-383-3p	up
B4GALT3	down	dre-miR-24_R+2	down
B4GALT3	down	gga-let-7a-5p_2ss12GT18TC	down
B4GALT3	down	gga-miR-1329-5p	down
B4GALT3	down	gga-miR-20b-3p_R-1	up
B4GALT3	down	gga-miR-222b-5p_R-4	down
ABHD17B	down	gga-let-7f-3p_1ss22CT	down
ABHD17B	down	gga-let-7j-3p_L-1R+2	down
ABHD17B	down	gga-miR-106-3p_R+1	up
ABHD17B	down	gga-miR-17-3p_L-1R+3	up
ABHD17B	down	gga-miR-1796	up
ABHD17B	down	gga-miR-222a	up
ABHD17B	down	gga-miR-429-3p	up
ABHD17B	down	hsa-miR-143-5p	up
ABHD17B	down	xtr-miR-429_R+2	down
PCMTD1	down	chi-miR-361-5p	up
PCMTD1	down	cin-miR-33_R+4	down
PCMTD1	down	efu-miR-361	up
PCMTD1	down	gga-miR-107-3p_R-1	up
PCMTD1	down	gga-miR-125b-3p_R-1_1ss21CT	up
PCMTD1	down	gga-miR-130b-5p	up

PCMTD1	down	gga-miR-130b-5p_R-1	up
PCMTD1	down	gga-miR-130b-5p_R-3	up
PCMTD1	down	gga-miR-1559-3p	up
PCMTD1	down	gga-miR-16-1-3p_R-1	up
PCMTD1	down	gga-miR-1783_R+1	up
PCMTD1	down	gga-miR-1796	up
PCMTD1	down	gga-miR-20b-5p	up
PCMTD1	down	gga-miR-21-5p	down
PCMTD1	down	gga-miR-2131-3p_R+1	up
PCMTD1	down	gga-miR-222a	up
PCMTD1	down	gga-miR-30a-3p_1ss18GA	up
PCMTD1	down	gga-miR-30a-3p_2ss10GA20AT	up
PCMTD1	down	gga-miR-30e-3p_L+1	up
PCMTD1	down	gga-miR-34a-3p_R-1	up
PCMTD1	down	gga-miR-429-3p	up
PCMTD1	down	gga-miR-6548-5p	up
PCMTD1	down	hsa-miR-21-5p_R+2_1ss1TN	down
PCMTD1	down	mmu-miR-8094_L+1R+3_1ss3AG	up
PCMTD1	down	oan-miR-19b-1-5p_R+4	up
PCMTD1	down	xtr-miR-429_R+2	down
TMEM68	down	aca-miR-181a_R+3	up
TMEM68	down	aca-miR-363-5p	up
TMEM68	down	cgr-miR-500	up
TMEM68	down	gga-let-7f-3p_1ss22CT	down
TMEM68	down	gga-let-7j-3p_L-1R+2	down
TMEM68	down	gga-miR-122-5p	down

TMEM68	down	gga-miR-1329-5p	down
TMEM68	down	gga-miR-140-5p_L+1	down
TMEM68	down	gga-miR-1555-5p	up
TMEM68	down	gga-miR-16-1-3p_R-1	up
TMEM68	down	gga-miR-17-3p_L-1R+3	up
TMEM68	down	gga-miR-1796	up
TMEM68	down	gga-miR-181a-5p	up
TMEM68	down	gga-miR-181b-1-3p_L+1R-1	up
TMEM68	down	gga-miR-181b-5p_R+1	up
TMEM68	down	gga-miR-205a_R+1	up
TMEM68	down	gga-miR-205b	up
TMEM68	down	gga-miR-29b-3p_R-1	down
TMEM68	down	gga-miR-29c-3p_R+1	down
TMEM68	down	gga-miR-29c-3p_R+1_1ss18GA	down
TMEM68	down	gga-miR-429-5p_L-1	up
TMEM68	down	gga-miR-490-3p_R+1	up
TMEM68	down	hsa-miR-143-5p	up
TMEM68	down	mmu-miR-500-3p_R-1	up
TMEM68	down	oha-miR-7-1-3p_1ss10GA	up
TMEM68	down	pma-miR-181a-5p_R+3	up
TMEM68	down	ssa-miR-29b-3p_R-1_2ss20TC21TC	down
TMEM68	down	tgu-miR-1784-3p_1ss11GA	down
TMEM68	down	tni-miR-205_R+2	up
ALCAM	down	aca-miR-18a-5p	up
ALCAM	down	gga-miR-1662_R-3	down
ALCAM	down	gga-miR-1729-5p_R-1	down

ALCAM	down	gga-miR-1788-3p_R+1	up
ALCAM	down	gga-miR-181b-1-3p_L+1R-1	up
ALCAM	down	gga-miR-222b-5p_R-4	down
ALCAM	down	gga-miR-3523	up
ALCAM	down	gga-miR-6548-5p	up
ALCAM	down	gga-miR-7460-3p	up
ALCAM	down	gga-miR-92-5p	up
ALCAM	down	gga-mir-147-p3_2	down
ALCAM	down	hsa-miR-3120-3p_R+1	down
ALCAM	down	mmu-mir-5098-p5	up
ALCAM	down	oan-miR-19b-1-5p_R+4	up
KIAA1524	up	aca-miR-138-2-3p_L-2R+3	up
KIAA1524	up	aca-miR-181a_R+3	up
KIAA1524	up	aca-miR-425-5p_R+4	down
KIAA1524	up	gga-let-7c-3p_1ss12TC	up
KIAA1524	up	gga-miR-146b-3p_L+2	down
KIAA1524	up	gga-miR-1796	up
KIAA1524	up	gga-miR-181a-5p	up
KIAA1524	up	gga-miR-181b-5p_R+1	up
KIAA1524	up	gga-miR-20b-5p	up
KIAA1524	up	gga-miR-6548-5p	up
KIAA1524	up	hsa-miR-425-5p_R+2_1ss20TC	down
KIAA1524	up	pma-miR-181a-5p_R+3	up
CYP7A1	up	gga-miR-107-3p_R-1	up
CYP7A1	up	gga-miR-1559-3p	up
CYP7A1	up	gga-miR-15b-5p_R-1	up

CYP7A1	up	gga-miR-1662_R-3	down
CYP7A1	up	gga-miR-16c-5p	up
CYP7A1	up	gga-miR-489-3p_L-2R+1	up
CYP7A1	up	tgu-miR-1662_L+1_1ss23TA	down
CD247	down	gga-let-7f-3p_1ss22CT	down
CD247	down	gga-miR-140-3p_L+1R+1_1ss22CA	down
CD247	down	gga-miR-1754-5p	up
CD247	down	gga-miR-1788-5p	up
CD247	down	gga-miR-2131-3p_R+1	up
CD247	down	gga-miR-460a-5p	up
CD247	down	gga-miR-9-5p	down
CD247	down	sha-miR-140_R+2	down
PPP2R2C	up	PC-3p-68190_58	up
PPP2R2C	up	gga-miR-17-3p_L-1R+3	up
PPP2R2C	up	gga-miR-456-3p_1ss22AT	up
PPP2R2C	up	gga-miR-456-3p_R-1	up
PPP2R2C	up	pma-miR-456_R+1_1ss22AC	up
TACC3	up	gga-miR-130b-3p	up
SLBP	up	aca-miR-425-5p_R+4	down
SLBP	up	gga-miR-1451-3p_R-1	up
SLBP	up	gga-miR-1662_R-3	down
SLBP	up	gga-miR-1788-3p_R+1	up
SLBP	up	gga-miR-1796	up
SLBP	up	gga-miR-460a-5p	up
SLBP	up	gga-mir-147-p3_2	down
SLBP	up	hsa-miR-3120-3p_R+1	down

SLBP	up	hsa-miR-425-5p_R+2_1ss20TC	down
SLBP	up	mmu-mir-5098-p5	up
SLBP	up	tgu-miR-1662_L+1_1ss23TA	down
FABP5	down	aca-miR-363-3p_R+1	down
FABP5	down	gga-miR-122-5p	down
FABP5	down	gga-miR-1559-3p	up
FABP5	down	gga-miR-16-1-3p_R-1	up
FABP5	down	gga-miR-20b-5p	up
FABP5	down	gga-miR-456-3p_1ss22AT	up
FABP5	down	gga-miR-456-3p_R-1	up
FABP5	down	oan-miR-363-3p_R+1	up
FABP5	down	pma-miR-456_R+1_1ss22AC	up
FABP5	down	rno-miR-122-5p_L+1R+1_1	down
FABP5	down	rno-miR-122-5p_L+1R+1_2	down
UBE2J1	down	gga-miR-1729-5p_R-1	down
UBE2J1	down	gga-miR-222a	up
UBE2J1	down	gga-miR-30a-5p_R+2_1ss13CT	up
UBE2J1	down	gga-miR-30b-5p	up
UBE2J1	down	gga-miR-30c-5p	up
UBE2J1	down	gga-miR-30e-5p_R+5	up
UBE2J1	down	gga-miR-34a-3p_R-1	up
UBE2J1	down	gga-miR-458a-3p	down
UBE2J1	down	gga-miR-460a-5p	up
UBE2J1	down	ggo-miR-342_R+1	up
UBE2J1	down	tgu-miR-30a-5p_R+2	up
PNRC1	down	gga-miR-125b-3p_R-1_1ss21CT	up

PNRC1	down	gga-miR-130b-3p	up
PNRC1	down	gga-miR-1784-5p	down
PNRC1	down	gga-miR-34a-3p_R-1	up
PNRC1	down	gga-miR-454-3p	up
PNRC1	down	mmu-mir-5098-p5	up
IMMT	up	aca-miR-363-5p	up
IMMT	up	gga-miR-130b-3p	up
IMMT	up	gga-miR-6548-5p	up
CD8A	down	gga-miR-122-5p	down
CD8A	down	gga-miR-15b-5p_R-1	up
CD8A	down	gga-miR-1668-3p	up
CD8A	down	gga-miR-16c-5p	up
CD8A	down	gga-miR-1784-5p	down
CD8A	down	gga-miR-92-5p	up
IFNAR1	down	gga-miR-106-3p_R+1	up
IFNAR1	down	gga-miR-181b-1-3p_L+2R-2	up
IFNAR1	down	gga-miR-20b-5p	up
IFNAR1	down	hsa-miR-181b-3p_L+1R-1	up
CDH17	down	gga-miR-106-3p_R+1	up
CDH17	down	gga-miR-17-3p_L-1R+3	up
CDH17	down	gga-miR-22-3p	down
CDH17	down	gga-miR-365-2-5p_L-1R+1	up
CDH17	down	hsa-miR-143-5p	up
TP53INP1	down	aca-miR-150-5p_1ss12TC	down
TP53INP1	down	aca-miR-18a-3p_L+1R-1_1ss12GA	up
TP53INP1	down	cgr-miR-151-3p	up

TP53INP1	down	cgr-miR-326	up
TP53INP1	down	gga-miR-107-5p_R+1	up
TP53INP1	down	gga-miR-1306-3p_R-1	up
TP53INP1	down	gga-miR-144-3p_L-1R-2	up
TP53INP1	down	gga-miR-16-1-3p_R-1	up
TP53INP1	down	gga-miR-1668-3p	up
TP53INP1	down	gga-miR-17-3p_L-1R+3	up
TP53INP1	down	gga-miR-205a_R+1	up
TP53INP1	down	gga-miR-205b	up
TP53INP1	down	gga-miR-20b-5p	up
TP53INP1	down	gga-miR-21-5p	down
TP53INP1	down	gga-miR-22-3p	down
TP53INP1	down	gga-miR-30a-5p_R+2_1ss13CT	up
TP53INP1	down	gga-miR-30c-5p	up
TP53INP1	down	gga-miR-30d_R+2	up
TP53INP1	down	gga-miR-30e-5p_R+5	up
TP53INP1	down	gga-miR-6604-5p_1ss7GA	up
TP53INP1	down	ggo-miR-342_R+1	up
TP53INP1	down	hsa-miR-143-5p	up
TP53INP1	down	hsa-miR-21-5p_R+2_1ss1TN	down
TP53INP1	down	hsa-miR-4443_R+1	up
TP53INP1	down	mmu-miR-8094_L+1R+3_1ss3AG	up
TP53INP1	down	odi-miR-7_R+2	down
TP53INP1	down	oha-miR-7-1-3p_1ss10GA	up
TP53INP1	down	rno-miR-122-5p_L+1R+1_1	down
TP53INP1	down	rno-miR-122-5p_L+1R+1_2	down

TP53INP1	down	tgu-miR-1784-3p_1ss11GA	down
TP53INP1	down	tgu-miR-30a-5p_R+2	up
TP53INP1	down	tni-miR-205_R+2	up
MORC3	down	aca-miR-129b-5p_R+1_1ss20TG	up
MORC3	down	aca-miR-18a-3p_L+1R-1_1ss12GA	up
MORC3	down	aca-miR-191-5p_R-1	down
MORC3	down	cin-miR-33_R+4	down
MORC3	down	gga-let-7f-3p_1ss22CT	down
MORC3	down	gga-miR-125b-3p_R-1_1ss21CT	up
MORC3	down	gga-miR-130b-3p	up
MORC3	down	gga-miR-142-5p_L-2R+1	down
MORC3	down	gga-miR-146a-3p_R-1	down
MORC3	down	gga-miR-155_R+1	down
MORC3	down	gga-miR-16-1-3p_R-1	up
MORC3	down	gga-miR-1602	up
MORC3	down	gga-miR-1668-3p	up
MORC3	down	gga-miR-1759-3p_L-3	up
MORC3	down	gga-miR-200b-5p_L+2	down
MORC3	down	gga-miR-33-5p	down
MORC3	down	gga-miR-34a-3p_R-1	up
MORC3	down	gga-miR-3523	up
MORC3	down	gga-miR-383-3p	up
MORC3	down	gga-miR-429-5p_L-1	up
MORC3	down	gga-miR-454-3p	up
MORC3	down	gga-miR-6710-3p_R+1	up
MORC3	down	gga-miR-7460-3p	up

MORC3	down	hsa-miR-3120-3p_R+1	down
MORC3	down	mmu-miR-3964_L+1_1ss6GA	down
SFXN5	up	cgr-miR-326	up
SFXN5	up	gga-miR-17-3p_L-1R+3	up
SFXN5	up	gga-miR-1759-3p_L-3	up
SFXN5	up	gga-miR-223_1ss15AG	down
SFXN5	up	gga-miR-223_1ss19CT	down
SFXN5	up	gga-miR-223_R+1	down
SFXN5	up	hsa-miR-143-5p	up
RAD21	up	aca-miR-181a_R+3	up
RAD21	up	aca-miR-363-3p_R+1	down
RAD21	up	cgr-miR-500	up
RAD21	up	gga-let-7f-3p_1ss22CT	down
RAD21	up	gga-miR-106-3p_R+1	up
RAD21	up	gga-miR-122-5p	down
RAD21	up	gga-miR-146b-3p_L+2	down
RAD21	up	gga-miR-17-3p_L-1R+3	up
RAD21	up	gga-miR-1784-5p	down
RAD21	up	gga-miR-181a-5p	up
RAD21	up	gga-miR-181b-5p_R+1	up
RAD21	up	gga-miR-21-5p	down
RAD21	up	gga-miR-22-3p	down
RAD21	up	gga-miR-30a-3p_1ss18GA	up
RAD21	up	gga-miR-30e-3p_L+1	up
RAD21	up	gga-miR-365-2-5p_L-1R+1	up
RAD21	up	gga-miR-458a-3p	down

RAD21	up	hsa-miR-21-5p_R+2_1ss1TN	down
RAD21	up	mmu-miR-500-3p_R-1	up
RAD21	up	oan-let-7f-1-3p_R+2_1ss8AG	down
RAD21	up	oan-miR-363-3p_R+1	up
RAD21	up	pma-miR-181a-5p_R+3	up
SIK1	down	aca-miR-363-3p_R+1	down
SIK1	down	cin-miR-33_R+4	down
SIK1	down	gga-miR-107-5p_R+1	up
SIK1	down	gga-miR-130b-3p	up
SIK1	down	gga-miR-130b-5p	up
SIK1	down	gga-miR-130b-5p_R-1	up
SIK1	down	gga-miR-130b-5p_R-3	up
SIK1	down	gga-miR-137-3p_L+1	up
SIK1	down	gga-miR-142-3p	down
SIK1	down	gga-miR-1668-3p	up
SIK1	down	gga-miR-16c-5p	up
SIK1	down	gga-miR-1759-3p_L-3	up
SIK1	down	gga-miR-1784-5p	down
SIK1	down	gga-miR-1788-5p	up
SIK1	down	gga-miR-20b-3p_R-1	up
SIK1	down	gga-miR-20b-5p	up
SIK1	down	gga-miR-214_L+1R-3_1ss19GA	down
SIK1	down	gga-miR-223_R+1	down
SIK1	down	gga-miR-30a-3p_1ss18GA	up
SIK1	down	gga-miR-30a-3p_2ss10GA20AT	up
SIK1	down	gga-miR-30e-3p_L+1	up

SIK1	down	gga-miR-33-5p	down
SIK1	down	gga-miR-3607-5p_R+1	up
SIK1	down	gga-miR-454-3p	up
SIK1	down	gga-miR-9-5p	down
SIK1	down	hsa-miR-3120-3p_R+1	down
SIK1	down	oan-miR-363-3p_R+1	up
MAOA	down	PC-5p-479025_5	up
MAOA	down	aca-miR-363-5p	up
MAOA	down	gga-miR-15b-5p_R-1	up
MAOA	down	gga-miR-16c-5p	up
MAOA	down	gga-miR-181b-1-3p_L+1R-1	up
MAOA	down	gga-miR-20b-3p_R-1	up
MAOA	down	gga-miR-216b	up
MAOA	down	gga-mir-147-p3_1	down
MAOA	down	gga-mir-147-p5	down
MAOA	down	hsa-miR-3120-3p_R+1	down
MAOA	down	oan-miR-19b-1-5p_R+4	up
TINAG	up	gga-let-7j-3p_L-1R+2	down
TINAG	up	gga-miR-107-3p_R-1	up
TINAG	up	gga-miR-144-3p_L-1R-2	up
TINAG	up	gga-miR-15b-5p_R-1	up
TINAG	up	gga-miR-16c-5p	up
TINAG	up	gga-miR-6548-5p	up
TINAG	up	gga-mir-147-p3_1	down
TINAG	up	gga-mir-147-p5	down
ASAP1	down	gga-miR-144-3p_L-1R-2	up

ASAP1	down	gga-miR-1720-5p	up
ASAP1	down	gga-miR-1788-3p_R+1	up
ASAP1	down	gga-miR-214_L+1R-3_1ss19GA	down
ASAP1	down	gga-miR-429-3p	up
ASAP1	down	xtr-miR-429_R+2	down
POLA1	up	gga-miR-15b-5p_R-1	up
POLA1	up	gga-miR-16c-5p	up
POLA1	up	gga-miR-1784-5p	down
POLA1	up	gga-miR-181b-1-3p_L+2R-2	up
POLA1	up	hsa-miR-181b-3p_L+1R-1	up
FBXO9	down	aca-miR-18a-3p_L+1R-1_1ss12GA	up
FBXO9	down	cgr-miR-500	up
FBXO9	down	gga-miR-107-3p_R-1	up
FBXO9	down	gga-miR-130b-5p	up
FBXO9	down	gga-miR-130b-5p_R-1	up
FBXO9	down	gga-miR-130b-5p_R-3	up
FBXO9	down	gga-miR-1451-3p_R-1	up
FBXO9	down	gga-miR-146a-3p_R-1	down
FBXO9	down	gga-miR-15b-5p_R-1	up
FBXO9	down	gga-miR-16c-5p	up
FBXO9	down	gga-miR-1759-3p_L-3	up
FBXO9	down	gga-miR-202-5p_L-1	up
FBXO9	down	gga-miR-214_L+1R-3_1ss19GA	down
FBXO9	down	gga-miR-3607-5p_R+1	up
FBXO9	down	gga-miR-383-3p	up
FBXO9	down	gga-miR-458a-3p	down

FBXO9	down	gga-miR-460a-5p	up
FBXO9	down	mmu-miR-500-3p_R-1	up
FBXO9	down	tgu-miR-1662_L+1_1ss23TA	down
GSTA	down	gga-miR-140-3p_L+1R+1_1ss22CA	down
GSTA	down	gga-miR-17-3p_L-1R+3	up
GSTA	down	gga-miR-20b-3p_R-1	up
GSTA	down	gga-miR-21-5p	down
GSTA	down	gga-miR-34a-3p_R-1	up
GSTA	down	gga-miR-6604-5p_1ss7GA	up
GSTA	down	hsa-miR-3120-3p_R+1	down
GSTA	down	mmu-miR-8094_L+1R+3_1ss3AG	up
GSTA	down	sha-miR-140_R+2	down
DSCC1	up	aca-miR-181a_R+3	up
DSCC1	up	gga-miR-1731-5p	up
DSCC1	up	gga-miR-181a-5p	up
DSCC1	up	gga-miR-181b-5p_R+1	up
DSCC1	up	gga-miR-2131-3p_R+1	up
DSCC1	up	gga-miR-30a-5p_R+2_1ss13CT	up
DSCC1	up	gga-miR-30d_R+2	up
DSCC1	up	gga-miR-30e-5p_R+5	up
DSCC1	up	pma-miR-181a-5p_R+3	up
DSCC1	up	tgu-miR-30a-5p_R+2	up
PDHA1	up	aca-miR-181a_R+3	up
PDHA1	up	gga-miR-1796	up
PDHA1	up	gga-miR-181a-5p	up
PDHA1	up	gga-miR-181b-5p_R+1	up

PDHA1	up	hsa-miR-3120-3p_R+1	down
PDHA1	up	pma-miR-181a-5p_R+3	up
KRTCAP3	up	gga-miR-29b-3p_R-1	down
KRTCAP3	up	gga-miR-29c-3p_R+1	down
KRTCAP3	up	gga-miR-29c-3p_R+1_1ss18GA	down
KRTCAP3	up	gga-miR-383-3p	up
KRTCAP3	up	gga-miR-456-3p_1ss22AT	up
KRTCAP3	up	gga-miR-456-3p_R-1	up
KRTCAP3	up	gga-miR-6548-5p	up
KRTCAP3	up	hsa-miR-4286_R+1	up
KRTCAP3	up	pma-miR-456_R+1_1ss22AC	up
KRTCAP3	up	ssa-miR-29b-3p_R-1_2ss20TC21TC	down
TMEM214	up	gga-miR-1759-3p_L-3	up
REPS2	up	PC-5p-479025_5	up
REPS2	up	aca-miR-129b-5p_R+1_1ss20TG	up
REPS2	up	aca-miR-138-2-3p_L-2R+3	up
REPS2	up	gga-let-7j-3p_L-1R+2	down
REPS2	up	gga-miR-155_R+1	down
REPS2	up	gga-miR-1699_L-1R-2	up
REPS2	up	gga-miR-16c-5p	up
REPS2	up	gga-miR-1788-3p_R+1	up
REPS2	up	gga-miR-181b-1-3p_L+2R-2	up
REPS2	up	gga-miR-222a	up
REPS2	up	gga-miR-29b-3p_R-1	down
REPS2	up	gga-miR-29c-3p_L+1R+1_1ss19GA	down
REPS2	up	gga-miR-29c-3p_R+1	down

REPS2	up	gga-miR-29c-3p_R+1_1ss18GA	down
REPS2	up	gga-miR-456-3p_1ss22AT	up
REPS2	up	gga-miR-456-3p_R-1	up
REPS2	up	gga-miR-460a-5p	up
REPS2	up	hsa-miR-181b-3p_L+1R-1	up
REPS2	up	mmu-miR-8094_L+1R+3_1ss3AG	up
REPS2	up	oha-miR-7-1-3p_1ss10GA	up
REPS2	up	pma-miR-456_R+1_1ss22AC	up
REPS2	up	ssa-miR-29b-3p_R-1_2ss20TC21TC	down
ASB9	down	gga-miR-125b-3p_R-1_1ss21CT	up
ASB9	down	gga-miR-1796	up
ASB9	down	gga-miR-29a-5p_L-1R-2	down
ASB9	down	tni-miR-455_L+1_2ss22CT23GT	down
BLK	down	hsa-miR-4443_R+1	up
FDFT1	down	aca-miR-18a-3p_L+1R-1_1ss12GA	up
FDFT1	down	gga-miR-146c-5p_4ss18GA20AG22TG23GT	down
FDFT1	down	gga-miR-16c-5p	up
FDFT1	down	gga-miR-1729-5p_R-1	down
FDFT1	down	gga-miR-365-2-5p_L-1R+1	up
FDFT1	down	gga-miR-6548-5p	up
FDFT1	down	ola-miR-199a-3p_R+1_1ss22AG	down
MCM3	up	cgr-miR-326	up
MCM3	up	gga-miR-107-5p_R+1	up
MCM3	up	gga-miR-1759-3p_L-3	up
MCM3	up	gga-miR-1788-5p	up

MCM3	up	gga-miR-181b-1-3p_L+1R-1	up
MCM3	up	gga-miR-20b-5p	up
MCM3	up	gga-miR-6548-5p	up
MCM3	up	gga-miR-9-5p	down
RGN	down	PC-3p-68190_58	up
RGN	down	gga-miR-223_1ss15AG	down
RGN	down	gga-miR-223_1ss19CT	down
RGN	down	gga-miR-223_R+1	down
SLC9A7	down	aca-miR-181a_R+3	up
SLC9A7	down	gga-miR-181a-5p	up
SLC9A7	down	gga-miR-181b-5p_R+1	up
SLC9A7	down	hsa-miR-4286_R+1	up
SLC9A7	down	pma-miR-181a-5p_R+3	up
LYG2	down	gga-miR-1788-3p_R+1	up
LYG2	down	hsa-miR-3120-3p_R+1	down
LYG2	down	mmu-mir-5098-p5	up
CHST10	down	PC-3p-68190_58	up
CHST10	down	aca-miR-18a-3p_L+1R-1_1ss12GA	up
CHST10	down	gga-miR-106-3p_R+1	up
CHST10	down	gga-miR-107-5p_R+1	up
CHST10	down	gga-miR-16c-5p	up
CHST10	down	gga-miR-17-3p_L-1R+3	up
CHST10	down	gga-miR-1731-5p	up
CHST10	down	gga-miR-29a-5p_L-1R-2	down
CHST10	down	gga-miR-30a-3p_1ss18GA	up
CHST10	down	gga-miR-30a-3p_2ss10GA20AT	up

CHST10	down	gga-miR-30a-5p_R+2_1ss13CT	up
CHST10	down	gga-miR-30d_R+2	up
CHST10	down	gga-miR-30e-3p_L+1	up
CHST10	down	gga-miR-30e-5p_R+5	up
CHST10	down	odi-miR-7_R+2	down
CHST10	down	ola-miR-199a-3p_R+1_1ss22AG	down
CHST10	down	tgu-miR-30a-5p_R+2	up
CHST10	down	tni-miR-455_L+1_2ss22CT23GT	down
IL1RL1	down	gga-miR-15b-5p_R-1	up
IL1RL1	down	gga-miR-16c-5p	up
IL1RL1	down	gga-miR-1788-3p_R+1	up
RASA3	down	gga-miR-142-5p_L-2R+1	down
RASA3	down	gga-miR-20b-5p	up
RASA3	down	gga-miR-222a	up
RASA3	down	mmu-miR-3964_L+1_1ss6GA	down
RASA3	down	oan-miR-19b-1-5p_R+4	up
GAS6	down	aca-miR-181a_R+3	up
GAS6	down	gga-miR-181a-5p	up
GAS6	down	gga-miR-181b-5p_R+1	up
GAS6	down	pma-miR-181a-5p_R+3	up
IRG1	down	aca-miR-138-2-3p_L-2R+3	up
IRG1	down	gga-miR-107-3p_R-1	up
IRG1	down	gga-miR-122-5p	down
IRG1	down	gga-miR-15b-5p_R-1	up
IRG1	down	gga-miR-16c-5p	up
IRG1	down	gga-miR-202-5p_L-1	up

IRG1	down	gga-miR-30a-3p_1ss18GA	up
IRG1	down	gga-miR-30e-3p_L+1	up
IRG1	down	gga-miR-34a-3p_R-1	up
IRG1	down	gga-miR-3607-5p_R+1	up
IRG1	down	gga-miR-429-5p_L-1	up
IRG1	down	gga-miR-6604-5p_1ss7GA	up
IRG1	down	gga-miR-9-5p	down
IRG1	down	gga-miR-92-5p	up
IRG1	down	hsa-miR-143-5p	up
IRG1	down	mmu-miR-8094_L+1R+3_1ss3AG	up
IRG1	down	mmu-mir-5098-p5	up
IRG1	down	rno-miR-122-5p_L+1R+1_1	down
IRG1	down	rno-miR-122-5p_L+1R+1_2	down
UCHL3	up	gga-miR-3607-5p_R+1	up
DIS3	up	mmu-mir-5098-p5	up
LCP1	down	gga-miR-130b-5p	up
LCP1	down	gga-miR-130b-5p_R-1	up
LCP1	down	gga-miR-130b-5p_R-3	up
LCP1	down	gga-miR-137-3p_L+1	up
LCP1	down	gga-miR-1747-3p	up
LCP1	down	gga-miR-214_L+1R-3_1ss19GA	down
LCP1	down	gga-miR-383-3p	up
LCP1	down	gga-miR-429-5p_L-1	up
LCP1	down	gga-miR-454-3p	up
LCP1	down	gga-miR-460a-5p	up
LCP1	down	gga-miR-9-5p	down

SUCLA2	down	cgr-miR-500	up
SUCLA2	down	gga-let-7f-3p_1ss22CT	down
SUCLA2	down	gga-miR-106-3p_R+1	up
SUCLA2	down	gga-miR-144-3p_L-1R-2	up
SUCLA2	down	mmu-miR-500-3p_R-1	up
RCBTB1	up	aca-miR-129b-5p_R+1_1ss20TG	up
RCBTB1	up	cgr-miR-326	up
RCBTB1	up	cin-miR-33_R+4	down
RCBTB1	up	gga-miR-1559-3p	up
RCBTB1	up	gga-miR-1602	up
RCBTB1	up	gga-miR-214_L+1R-3_1ss19GA	down
RCBTB1	up	gga-miR-33-5p	down
RCBTB1	up	gga-miR-460a-3p	up
CKAP2	up	aca-miR-191-5p_R-1	down
CKAP2	up	gga-miR-106-3p_R+1	up
CKAP2	up	gga-miR-181b-1-3p_L+1R-1	up
CKAP2	up	gga-miR-6548-5p	up
N4BP2L1	down	gga-miR-6604-5p_1ss7GA	up
SLC46A3	down	aca-miR-425-5p_R+4	down
SLC46A3	down	gga-miR-107-3p_R-1	up
SLC46A3	down	gga-miR-130b-3p	up
SLC46A3	down	gga-miR-15b-5p_R-1	up
SLC46A3	down	gga-miR-16c-5p	up
SLC46A3	down	gga-miR-1783_R+1	up
SLC46A3	down	gga-miR-205a_R+1	up
SLC46A3	down	gga-miR-205b	up

SLC46A3	down	gga-miR-20b-5p	up
SLC46A3	down	gga-miR-21-5p	down
SLC46A3	down	gga-miR-214_L+1R-3_1ss19GA	down
SLC46A3	down	gga-miR-222b-5p_R-4	down
SLC46A3	down	gga-miR-29a-5p_L-1R-2	down
SLC46A3	down	gga-miR-34a-3p_R-1	up
SLC46A3	down	gga-miR-383-3p	up
SLC46A3	down	gga-miR-454-3p	up
SLC46A3	down	gga-miR-456-3p_1ss22AT	up
SLC46A3	down	gga-miR-456-3p_R-1	up
SLC46A3	down	hsa-miR-21-5p_R+2_1ss1TN	down
SLC46A3	down	hsa-miR-3120-3p_R+1	down
SLC46A3	down	hsa-miR-425-5p_R+2_1ss20TC	down
SLC46A3	down	hsa-miR-4286_R+1	up
SLC46A3	down	mmu-miR-8094_L+1R+3_1ss3AG	up
SLC46A3	down	oan-miR-19b-1-5p_R+4	up
SLC46A3	down	ola-miR-199a-3p_R+1_1ss22AG	down
SLC46A3	down	pma-miR-456_R+1_1ss22AC	up
SLC46A3	down	rno-miR-122-5p_L+1R+1_1	down
SLC46A3	down	rno-miR-122-5p_L+1R+1_2	down
SLC46A3	down	tni-miR-205_R+2	up
CEP57	down	aca-miR-150-5p_1ss12TC	down
CEP57	down	gga-miR-214_L+1R-3_1ss19GA	down
CEP57	down	ola-miR-199a-3p_R+1_1ss22AG	down
RRM1	up	gga-let-7f-3p_1ss22CT	down
RRM1	up	gga-miR-1329-5p	down

RRM1	up	gga-miR-1720-5p	up
RRM1	up	gga-miR-383-3p	up
RRM1	up	hsa-miR-3120-3p_R+1	down
UQCRH	up	gga-miR-140-3p_L+1R+1_1ss22CA	down
UQCRH	up	gga-miR-1552-3p_R+1	up
UQCRH	up	gga-miR-222b-5p_R-4	down
UQCRH	up	sha-miR-140_R+2	down
ARL6IP5	down	gga-miR-122-5p	down
ARL6IP5	down	gga-miR-137-3p_L+1	up
ARL6IP5	down	gga-miR-1559-3p	up
ARL6IP5	down	gga-miR-1663-5p_R+2	up
ARL6IP5	down	gga-miR-1720-5p	up
ARL6IP5	down	gga-miR-205a_R+1	up
ARL6IP5	down	gga-miR-205b	up
ARL6IP5	down	gga-miR-21-5p	down
ARL6IP5	down	gga-miR-29a-5p_L-1R-2	down
ARL6IP5	down	gga-miR-30a-5p_R+2_1ss13CT	up
ARL6IP5	down	gga-miR-30b-5p	up
ARL6IP5	down	gga-miR-30c-5p	up
ARL6IP5	down	gga-miR-30d_R+2	up
ARL6IP5	down	gga-miR-30e-5p_R+5	up
ARL6IP5	down	gga-miR-383-3p	up
ARL6IP5	down	gga-miR-429-3p	up
ARL6IP5	down	hsa-miR-143-5p	up
ARL6IP5	down	hsa-miR-21-5p_R+2_1ss1TN	down
ARL6IP5	down	ola-miR-126-5p_R+1_1ss1CN	down

ARL6IP5	down	rno-miR-122-5p_L+1R+1_1	down
ARL6IP5	down	rno-miR-122-5p_L+1R+1_2	down
ARL6IP5	down	tgu-miR-30a-5p_R+2	up
ARL6IP5	down	tni-miR-205_R+2	up
ARL6IP5	down	xtr-miR-429_R+2	down
H2AFZ	up	gga-let-7c-3p_1ss12TC	up
H2AFZ	up	gga-miR-107-5p_R+1	up
H2AFZ	up	gga-miR-16c-3p_L+1R-1	up
H2AFZ	up	gga-mir-147-p3_2	down
COTL1	down	PC-3p-68190_58	up
COTL1	down	gga-miR-140-3p_L+1R+1_1ss22CA	down
COTL1	down	gga-miR-1668-3p	up
COTL1	down	gga-miR-22-3p	down
COTL1	down	mmu-miR-6412_R-2_1ss15AT	down
COTL1	down	sha-miR-140_R+2	down
NTM	down	gga-miR-1759-3p_L-3	up
GZMA	down	gga-miR-155_R+1	down
GZMA	down	gga-miR-1668-3p	up
GZMA	down	gga-miR-458a-3p	down
GZMA	down	mmu-miR-8094_L+1R+3_1ss3AG	up
LYN	down	cin-miR-33_R+4	down
LYN	down	gga-miR-106-3p_R+1	up
LYN	down	gga-miR-130b-5p	up
LYN	down	gga-miR-130b-5p_R-1	up
LYN	down	gga-miR-130b-5p_R-3	up
LYN	down	gga-miR-146c-5p_4ss18GA20AG22T	down

		G23GT	
LYN	down	gga-miR-1552-3p_R+1	up
LYN	down	gga-miR-15b-5p_R-1	up
LYN	down	gga-miR-16-1-3p_R-1	up
LYN	down	gga-miR-16c-5p	up
LYN	down	gga-miR-1784-5p	down
LYN	down	gga-miR-181a-3p	up
LYN	down	gga-miR-181b-1-3p_L+1R-1	up
LYN	down	gga-miR-214_L+1R-3_1ss19GA	down
LYN	down	gga-miR-33-5p	down
LYN	down	gga-miR-460a-5p	up
DPT	down	PC-3p-68190_58	up
DPT	down	gga-miR-1467-3p_L+1	up
DPT	down	gga-miR-1699_L-1R-2	up
DPT	down	gga-miR-1788-3p_R+1	up
DPT	down	gga-miR-222a	up
DPT	down	hsa-miR-4443_R+1	up
DPT	down	odi-miR-7_R+2	down
DPT	down	rno-miR-122-5p_L+1R+1_1	down
DPT	down	rno-miR-122-5p_L+1R+1_2	down
PACSIN2	down	aca-miR-181a_R+3	up
PACSIN2	down	cin-miR-33_R+4	down
PACSIN2	down	gga-let-7c-3p_1ss12TC	up
PACSIN2	down	gga-miR-1329-5p	down
PACSIN2	down	gga-miR-16-1-3p_R-1	up
PACSIN2	down	gga-miR-181a-5p	up

PACSIN2	down	gga-miR-181b-5p_R+1	up
PACSIN2	down	gga-miR-20b-3p_R-1	up
PACSIN2	down	gga-miR-30a-3p_1ss18GA	up
PACSIN2	down	gga-miR-30a-3p_2ss10GA20AT	up
PACSIN2	down	gga-miR-30e-3p_L+1	up
PACSIN2	down	gga-miR-33-5p	down
PACSIN2	down	gga-miR-429-3p	up
PACSIN2	down	gga-miR-455-5p	up
PACSIN2	down	gga-miR-6548-5p	up
PACSIN2	down	pma-miR-181a-5p_R+3	up
PACSIN2	down	xtr-miR-429_R+2	down
ABHD5	down	gga-miR-107-5p_R+1	up
ABHD5	down	gga-miR-140-5p_L+1	down
ABHD5	down	gga-miR-460a-5p	up
HIGD1A	up	gga-miR-146a-3p_R-1	down
HIGD1A	up	gga-miR-1559-3p	up
HIGD1A	up	gga-miR-200b-5p_L+2	down
HIGD1A	up	gga-miR-456-3p_1ss22AT	up
HIGD1A	up	gga-miR-456-3p_R-1	up
HIGD1A	up	gga-miR-489-3p_L-2R+1	up
HIGD1A	up	hsa-miR-4443_R+1	up
HIGD1A	up	mmu-miR-8094_L+1R+3_1ss3AG	up
HIGD1A	up	pma-miR-456_R+1_1ss22AC	up
MICAL1	down	gga-miR-107-3p_R-1	up
MICAL1	down	gga-miR-1559-5p	up
MICAL1	down	gga-miR-17-3p_L-1R+3	up

MICAL1	down	gga-miR-22-3p	down
MICAL1	down	gga-miR-455-5p	up
GAL7	down	aca-miR-129b-5p_R+1_1ss20TG	up
GAL7	down	rno-miR-122-5p_L+1R+1_1	down
GAL7	down	rno-miR-122-5p_L+1R+1_2	down
C3H2ORF43	down	aca-miR-129b-5p_R+1_1ss20TG	up
C3H2ORF43	down	aca-miR-363-5p	up
C3H2ORF43	down	gga-miR-125b-3p_R-1_1ss21CT	up
C3H2ORF43	down	gga-miR-137-3p_L+1	up
C3H2ORF43	down	gga-miR-142-3p	down
C3H2ORF43	down	gga-miR-1451-3p_R-1	up
C3H2ORF43	down	gga-miR-1552-3p_R+1	up
C3H2ORF43	down	gga-miR-1747-3p	up
C3H2ORF43	down	gga-miR-30a-5p_R+2_1ss13CT	up
C3H2ORF43	down	gga-miR-30b-5p	up
C3H2ORF4	down	gga-miR-30c-5p	up

3			
C3H2ORF43	down	gga-miR-30d_R+2	up
C3H2ORF43	down	gga-miR-30e-5p_R+5	up
C3H2ORF43	down	gga-miR-6548-5p	up
C3H2ORF43	down	gga-miR-9-5p	down
C3H2ORF43	down	ggo-miR-342_R+1	up
C3H2ORF43	down	hsa-miR-3120-3p_R+1	down
C3H2ORF43	down	hsa-miR-4286_R+1	up
C3H2ORF43	down	mmu-miR-8094_L+1R+3_1ss3AG	up
C3H2ORF43	down	mmu-mir-5098-p5	up
C3H2ORF43	down	oha-miR-7-1-3p_1ss10GA	up
C3H2ORF43	down	ola-miR-199a-3p_R+1_1ss22AG	down
C3H2ORF43	down	tgu-miR-30a-5p_R+2	up
MCPH1	up	aca-miR-129b-5p_R+1_1ss20TG	up

MCPH1	up	gga-miR-1788-5p	up
MCPH1	up	gga-miR-202-5p_L-1	up
MCPH1	up	mmu-miR-8094_L+1R+3_1ss3AG	up
IFNGR1	down	aca-miR-181a_R+3	up
IFNGR1	down	chi-miR-361-5p	up
IFNGR1	down	efu-miR-361	up
IFNGR1	down	gga-miR-1329-5p	down
IFNGR1	down	gga-miR-1559-3p	up
IFNGR1	down	gga-miR-1784-5p	down
IFNGR1	down	gga-miR-181a-5p	up
IFNGR1	down	gga-miR-181b-5p_R+1	up
IFNGR1	down	gga-miR-216b	up
IFNGR1	down	pma-miR-181a-5p_R+3	up
SUCLG1	up	aca-miR-129b-5p_R+1_1ss20TG	up
SUCLG1	up	aca-miR-18a-5p	up
SUCLG1	up	aca-miR-363-5p	up
SUCLG1	up	cgr-miR-326	up
SUCLG1	up	gga-miR-125b-3p_R-1_1ss21CT	up
SUCLG1	up	gga-miR-1329-5p	down
SUCLG1	up	gga-miR-138-5p_R+7	up
SUCLG1	up	gga-miR-1451-3p_R-1	up
SUCLG1	up	gga-miR-17-3p_L-1R+3	up
SUCLG1	up	gga-miR-1788-3p_R+1	up
SUCLG1	up	gga-miR-3607-5p_R+1	up
SUCLG1	up	gga-miR-489-3p_L-2R+1	up
SUCLG1	up	gga-miR-6548-5p	up

SUCLG1	up	gga-miR-6604-5p_1ss7GA	up
SUCLG1	up	gga-miR-9-5p	down
SUCLG1	up	hsa-miR-143-5p	up
SUCLG1	up	rno-miR-122-5p_L+1R+1_1	down
SUCLG1	up	rno-miR-122-5p_L+1R+1_2	down
CHP1	up	PC-5p-479025_5	up
CHP1	up	cgr-miR-500	up
CHP1	up	gga-miR-106-3p_R+1	up
CHP1	up	gga-miR-1329-5p	down
CHP1	up	gga-miR-155_R+1	down
CHP1	up	gga-miR-181b-1-3p_L+2R-2	up
CHP1	up	gga-miR-205a_R+1	up
CHP1	up	gga-miR-30b-5p	up
CHP1	up	gga-miR-30c-5p	up
CHP1	up	gga-miR-455-5p	up
CHP1	up	gga-mir-147-p3_1	down
CHP1	up	gga-mir-147-p5	down
CHP1	up	hsa-miR-181b-3p_L+1R-1	up
CHP1	up	mmu-miR-500-3p_R-1	up
CHP1	up	tni-miR-205_R+2	up
INCENP	up	aca-miR-150-5p_1ss12TC	down
INCENP	up	aca-miR-18a-3p_L+1R-1_1ss12GA	up
INCENP	up	cgr-miR-326	up
INCENP	up	cgr-miR-500	up
INCENP	up	gga-let-7c-3p_1ss12TC	up
INCENP	up	gga-miR-106-3p_R+1	up

INCENP	up	gga-miR-130b-3p	up
INCENP	up	gga-miR-137-3p_L+1	up
INCENP	up	gga-miR-140-3p_L+1R+1_1ss22CA	down
INCENP	up	gga-miR-142-3p	down
INCENP	up	gga-miR-1451-3p_R-1	up
INCENP	up	gga-miR-146b-3p_L+2	down
INCENP	up	gga-miR-1559-3p	up
INCENP	up	gga-miR-15b-3p_L+1_1ss22AC	up
INCENP	up	gga-miR-1788-5p	up
INCENP	up	gga-miR-1796	up
INCENP	up	gga-miR-21-5p	down
INCENP	up	gga-miR-22-3p	down
INCENP	up	gga-miR-222b-5p_R-4	down
INCENP	up	gga-miR-429-3p	up
INCENP	up	gga-miR-489-3p_L-2R+1	up
INCENP	up	gga-miR-6548-5p	up
INCENP	up	hsa-miR-143-5p	up
INCENP	up	hsa-miR-21-5p_R+2_1ss1TN	down
INCENP	up	hsa-miR-3120-3p_R+1	down
INCENP	up	mmu-miR-500-3p_R-1	up
INCENP	up	mmu-miR-8094_L+1R+3_1ss3AG	up
INCENP	up	ola-miR-199a-3p_R+1_1ss22AG	down
INCENP	up	sha-miR-140_R+2	down
INCENP	up	tgu-miR-1662_L+1_1ss23TA	down
INCENP	up	xtr-miR-429_R+2	down
RAD51	up	gga-miR-6548-5p	up

SYK	down	PC-5p-479025_5	up
SYK	down	aca-miR-138-2-3p_L-2R+3	up
SYK	down	gga-miR-1796	up
SYK	down	gga-miR-2954_R+2	down
SYK	down	gga-miR-458a-3p	down
SYK	down	gga-miR-9-5p	down
SYK	down	ggo-miR-342_R+1	up
CENPH	up	gga-miR-1555-5p	up
CENPH	up	gga-miR-460a-3p	up
CENPH	up	hsa-miR-143-5p	up
CENPH	up	mmu-miR-8094_L+1R+3_1ss3AG	up
MCM6	up	aca-miR-138-2-3p_L-2R+3	up
MCM6	up	gga-miR-125b-3p_R-1_1ss21CT	up
MCM6	up	gga-miR-130b-3p	up
MCM6	up	gga-miR-2131-3p_R+1	up
MCM6	up	gga-miR-222b-5p_R-4	down
MCM6	up	gga-miR-30a-3p_1ss18GA	up
MCM6	up	gga-miR-30a-3p_2ss10GA20AT	up
MCM6	up	gga-miR-30a-5p_R+2_1ss13CT	up
MCM6	up	gga-miR-30b-5p	up
MCM6	up	gga-miR-30c-5p	up
MCM6	up	gga-miR-30d_R+2	up
MCM6	up	gga-miR-30e-3p_L+1	up
MCM6	up	gga-miR-30e-5p_R+5	up
MCM6	up	gga-miR-429-3p	up
MCM6	up	gga-miR-454-3p	up

MCM6	up	hsa-miR-3120-3p_R+1	down
MCM6	up	oan-let-7f-1-3p_R+2_1ss8AG	down
MCM6	up	odi-miR-7_R+2	down
MCM6	up	oha-miR-7-1-3p_1ss10GA	up
MCM6	up	rno-miR-122-5p_L+1R+1_1	down
MCM6	up	rno-miR-122-5p_L+1R+1_2	down
MCM6	up	tgu-miR-30a-5p_R+2	up
MCM6	up	xtr-miR-429_R+2	down
DCLRE1A	up	PC-5p-479025_5	up
DCLRE1A	up	gga-let-7b_L-1R-1	down
DCLRE1A	up	gga-miR-196-5p_R+2	down
DCLRE1A	up	gga-miR-21-5p	down
DCLRE1A	up	gga-miR-30a-5p_R+2_1ss13CT	up
DCLRE1A	up	gga-miR-30e-5p_R+5	up
DCLRE1A	up	gga-miR-490-3p_R+1	up
DCLRE1A	up	gga-mir-147-p3_1	down
DCLRE1A	up	gga-mir-147-p5	down
DCLRE1A	up	ola-miR-199a-3p_R+1_1ss22AG	down
DCLRE1A	up	tgu-miR-30a-5p_R+2	up
HABP2	down	gga-let-7a-5p	down
HABP2	down	gga-let-7a-5p_2ss12GT18TC	down
HABP2	down	gga-let-7b_R+2	down
HABP2	down	gga-let-7c-5p_1ss17AG	down
HABP2	down	gga-let-7g-5p_R+1_2ss14TG18CA	down
HABP2	down	gga-let-7k-5p_R+1_1ss16AT	down
HABP2	down	gga-miR-155_R+1	down

HABP2	down	gga-miR-181b-1-3p_L+1R-1	up
HABP2	down	gga-miR-200b-5p_L+2	down
HABP2	down	gga-miR-214_L+1R-3_1ss19GA	down
HABP2	down	gga-miR-383-3p	up
HABP2	down	ggo-miR-342_R+1	up
HABP2	down	hsa-miR-4443_R+1	up
HABP2	down	mmu-let-7j_1ss8TG	down
HABP2	down	tni-miR-205_R+2	up
ARHGAP19	up	cgr-miR-326	up
ARHGAP19	up	gga-miR-106-3p_R+1	up
ARHGAP19	up	gga-miR-107-3p_R-1	up
ARHGAP19	up	gga-miR-128-1-5p	up
ARHGAP19	up	gga-miR-140-5p_L+1	down
ARHGAP19	up	gga-miR-15b-5p_R-1	up
ARHGAP19	up	gga-miR-1699_L-1R-2	up
ARHGAP19	up	gga-miR-16c-5p	up
ARHGAP19	up	gga-miR-1759-3p_L-3	up
ARHGAP19	up	gga-miR-181b-1-3p_L+2R-2	up
ARHGAP19	up	gga-miR-383-3p	up
ARHGAP19	up	hsa-miR-181b-3p_L+1R-1	up
AURKA	up	ola-miR-199a-3p_R+1_1ss22AG	down
NDUFA10	up	dre-miR-24_R+2	down
NDUFA10	up	gga-miR-140-3p_L+1R+1_1ss22CA	down
NDUFA10	up	gga-miR-1602	up
NDUFA10	up	gga-miR-1668-3p	up
NDUFA10	up	gga-miR-1783_R+1	up

NDUFA10	up	gga-miR-1784-5p	down
NDUFA10	up	gga-miR-205a_R+1	up
NDUFA10	up	gga-miR-205b	up
NDUFA10	up	gga-miR-20b-3p_R-1	up
NDUFA10	up	gga-miR-30c-1-3p_R+1	up
NDUFA10	up	gga-miR-365-2-5p_L-1R+1	up
NDUFA10	up	hsa-miR-3120-3p_R+1	down
NDUFA10	up	mmu-mir-5098-p5	up
NDUFA10	up	sha-miR-140_R+2	down
NDUFA10	up	tni-miR-205_R+2	up
RSFR	down	gga-miR-107-3p_R-1	up
RSFR	down	gga-miR-15b-5p_R-1	up
RSFR	down	gga-miR-16c-5p	up
RSFR	down	mmu-mir-5098-p5	up
RGS1	down	gga-miR-1731-5p	up
RGS1	down	gga-miR-458a-3p	down
RGS1	down	gga-miR-460a-5p	up
RGS1	down	tgu-miR-1662_L+1_1ss23TA	down
STARD5	down	gga-miR-138-5p_R+7	up
STARD5	down	gga-miR-7460-3p	up
MPZL3	down	gga-miR-130b-5p	up
MPZL3	down	gga-miR-130b-5p_R-1	up
MPZL3	down	gga-miR-130b-5p_R-3	up
MPZL3	down	gga-miR-16c-3p_L+1R-1	up
MPZL3	down	gga-miR-30a-3p_2ss10GA20AT	up
MPZL3	down	gga-miR-34a-3p_R-1	up

NDUFA8	up	ggo-miR-342_R+1	up
NDUFA8	up	ola-miR-199a-3p_R+1_1ss22AG	down
MARCKSL1	down	gga-miR-1716_L-2	up
MARCKSL1	down	gga-miR-1720-5p	up
MARCKSL1	down	gga-miR-1783_R+1	up
MARCKSL1	down	mmu-mir-5098-p5	up
NFYC	up	gga-miR-130b-5p	up
NFYC	up	gga-miR-130b-5p_R-1	up
NFYC	up	gga-miR-130b-5p_R-3	up
NFYC	up	gga-miR-1663-5p_R+2	up
NFYC	up	gga-miR-1747-3p	up
NFYC	up	gga-miR-458a-3p	down
IFI27L2	down	gga-miR-106-3p_R+1	up
BIRC2	down	PC-5p-479025_5	up
BIRC2	down	gga-miR-16c-5p	up
BIRC2	down	gga-miR-29b-3p_R-1	down
BIRC2	down	gga-miR-29c-3p_R+1	down
BIRC2	down	gga-miR-29c-3p_R+1_1ss18GA	down
BIRC2	down	gga-miR-6548-5p	up
BIRC2	down	gga-miR-6710-3p_R+1	up
BIRC2	down	odi-miR-7_R+2	down
BIRC2	down	ssa-miR-29b-3p_R-1_2ss20TC21TC	down
PI15	up	gga-miR-107-5p_R+1	up
CENPQ	up	gga-miR-107-5p_R+1	up
CENPQ	up	gga-miR-15b-5p_R-1	up
CENPQ	up	gga-miR-16c-5p	up

CENPQ	up	gga-miR-3607-5p_R+1	up
CENPQ	up	gga-miR-460a-5p	up
CENPQ	up	gga-miR-6548-5p	up
CENPQ	up	hsa-miR-4443_R+1	up
GAL8	dowm	gga-miR-1663-5p_R+2	up
GAL8	dowm	gga-miR-17-3p_L-1R+3	up
GAL8	dowm	gga-miR-1784-5p	down
GAL8	dowm	gga-miR-200b-5p_L+2	down
GAL8	dowm	gga-miR-3607-5p_R+1	up
GAL9	dowm	gga-miR-1602	up
GAL9	dowm	gga-miR-29a-5p_L-1R-2	down
VWF	down	gga-miR-1663-5p_R+2	up
SMC2	up	gga-miR-1796	up
SMC2	up	gga-miR-6604-5p_1ss7GA	up
PDLIM5	down	aca-miR-129b-5p_R+1_1ss20TG	up
PDLIM5	down	aca-miR-18a-5p	up
PDLIM5	down	aca-miR-363-5p	up
PDLIM5	down	cgr-miR-500	up
PDLIM5	down	cin-miR-33_R+4	down
PDLIM5	down	gga-miR-140-3p_L+1R+1_1ss22CA	down
PDLIM5	down	gga-miR-142-5p_L-2R+1	down
PDLIM5	down	gga-miR-146a-3p_R-1	down
PDLIM5	down	gga-miR-15b-5p_R-1	up
PDLIM5	down	gga-miR-16c-3p_L+1R-1	up
PDLIM5	down	gga-miR-16c-5p	up
PDLIM5	down	gga-miR-1747-3p	up

PDLIM5	down	gga-miR-1759-3p_L-3	up
PDLIM5	down	gga-miR-1783_R+1	up
PDLIM5	down	gga-miR-1784-5p	down
PDLIM5	down	gga-miR-1796	up
PDLIM5	down	gga-miR-181b-1-3p_L+1R-1	up
PDLIM5	down	gga-miR-20b-5p	up
PDLIM5	down	gga-miR-214_L+1R-3_1ss19GA	down
PDLIM5	down	gga-miR-30a-3p_1ss18GA	up
PDLIM5	down	gga-miR-30a-3p_2ss10GA20AT	up
PDLIM5	down	gga-miR-30e-3p_L+1	up
PDLIM5	down	gga-miR-33-5p	down
PDLIM5	down	gga-miR-365-2-5p_L-1R+1	up
PDLIM5	down	gga-miR-383-3p	up
PDLIM5	down	gga-miR-458a-3p	down
PDLIM5	down	hsa-miR-143-5p	up
PDLIM5	down	hsa-miR-3120-3p_R+1	down
PDLIM5	down	mmu-miR-500-3p_R-1	up
PDLIM5	down	mmu-mir-5098-p5	up
PDLIM5	down	odi-miR-7_R+2	down
PDLIM5	down	sha-miR-140_R+2	down
RNASET2	down	chi-miR-361-5p	up
RNASET2	down	efu-miR-361	up
RNASET2	down	gga-miR-106-3p_R+1	up
RNASET2	down	gga-miR-130b-5p	up
RNASET2	down	gga-miR-130b-5p_R-1	up
RNASET2	down	gga-miR-130b-5p_R-3	up

RNASET2	down	gga-miR-137-3p_L+1	up
RNASET2	down	gga-miR-155_R+1	down
RNASET2	down	gga-miR-16-1-3p_R-1	up
RNASET2	down	gga-miR-29a-5p_L-1R-2	down
RNASET2	down	gga-miR-6710-3p_R+1	up
HMOX1	down	aca-miR-129b-5p_R+1_1ss20TG	up
HMOX1	down	gga-miR-140-3p_L+1R+1_1ss22CA	down
HMOX1	down	gga-miR-1668-3p	up
HMOX1	down	sha-miR-140_R+2	down
LPL	up	gga-miR-137-3p_L+1	up
LPL	up	gga-miR-15b-5p_R-1	up
LPL	up	gga-miR-16c-5p	up
LPL	up	gga-miR-1796	up
LPL	up	gga-miR-2954_R+2	down
LPL	up	gga-miR-458a-3p	down
LPL	up	hsa-miR-143-5p	up
NCF4	down	cgr-miR-151-3p	up
NCF4	down	gga-let-7c-3p_1ss12TC	up
NCF4	down	gga-miR-17-3p_L-1R+3	up
NCF4	down	gga-miR-21-5p	down
NCF4	down	oan-miR-19b-1-5p_R+4	up
RAC2	down	gga-miR-181b-1-3p_L+2R-2	up
RAC2	down	gga-miR-6548-5p	up
RAC2	down	hsa-miR-181b-3p_L+1R-1	up
REEP5	down	cgr-miR-326	up
REEP5	down	cin-miR-33_R+4	down

REEP5	down	gga-miR-1329-5p	down
REEP5	down	gga-miR-1559-3p	up
REEP5	down	gga-miR-2131-3p_R+1	up
REEP5	down	gga-miR-2954_R+2	down
REEP5	down	gga-miR-33-5p	down
REEP5	down	gga-miR-6548-5p	up
REEP5	down	gga-miR-92-5p	up
REEP5	down	hsa-miR-3120-3p_R+1	down
REEP5	down	ola-miR-126-5p_R+1_1ss1CN	down
HOPX	down	aca-miR-181a_R+3	up
HOPX	down	dre-miR-24_R+2	down
HOPX	down	gga-miR-181a-5p	up
HOPX	down	gga-miR-181b-5p_R+1	up
HOPX	down	gga-miR-222b-5p_R-4	down
HOPX	down	gga-miR-489-3p_L-2R+1	up
HOPX	down	gga-miR-9-5p	down
HOPX	down	pma-miR-181a-5p_R+3	up
CRIM1	down	PC-5p-479025_5	up
CRIM1	down	aca-miR-150-5p_1ss12TC	down
CRIM1	down	gga-miR-223_1ss15AG	down
CRIM1	down	gga-miR-223_1ss19CT	down
CRIM1	down	gga-miR-223_R+1	down
CRIM1	down	gga-miR-9-5p	down
GLDC	up	aca-miR-138-2-3p_L-2R+3	up
GLDC	up	gga-miR-106-3p_R+1	up
GLDC	up	gga-miR-144-3p_L-1R-2	up

GLDC	up	gga-miR-1467-3p_L+1	up
GLDC	up	gga-miR-1747-3p	up
GLDC	up	gga-miR-429-3p	up
GLDC	up	ola-miR-199a-3p_R+1_1ss22AG	down
GLDC	up	xtr-miR-429_R+2	down
RAD54L	up	aca-miR-18a-3p_L+1R-1_1ss12GA	up
RAD54L	up	gga-miR-16c-5p	up
RAD54L	up	gga-miR-1747-3p	up
RAD54L	up	gga-miR-181b-1-3p_L+2R-2	up
RAD54L	up	gga-miR-214_L+1R-3_1ss19GA	down
RAD54L	up	gga-miR-383-3p	up
RAD54L	up	hsa-miR-181b-3p_L+1R-1	up
RAD54L	up	oha-miR-7-1-3p_1ss10GA	up
WIPF1	down	cin-miR-33_R+4	down
WIPF1	down	gga-let-7j-3p_L-1R+2	down
WIPF1	down	gga-miR-106-3p_R+1	up
WIPF1	down	gga-miR-107-3p_R-1	up
WIPF1	down	gga-miR-130b-5p	up
WIPF1	down	gga-miR-130b-5p_R-1	up
WIPF1	down	gga-miR-130b-5p_R-3	up
WIPF1	down	gga-miR-144-3p_L-1R-2	up
WIPF1	down	gga-miR-1555-5p	up
WIPF1	down	gga-miR-16-1-3p_R-1	up
WIPF1	down	gga-miR-1796	up
WIPF1	down	gga-miR-30a-5p_R+2_1ss13CT	up
WIPF1	down	gga-miR-30b-5p	up

WIPF1	down	gga-miR-30c-5p	up
WIPF1	down	gga-miR-30d_R+2	up
WIPF1	down	gga-miR-30e-5p_R+5	up
WIPF1	down	gga-miR-33-5p	down
WIPF1	down	gga-miR-429-3p	up
WIPF1	down	gga-miR-460a-5p	up
WIPF1	down	gga-mir-147-p3_1	down
WIPF1	down	gga-mir-147-p5	down
WIPF1	down	ggo-miR-342_R+1	up
WIPF1	down	hsa-miR-3120-3p_R+1	down
WIPF1	down	tgu-miR-30a-5p_R+2	up
WIPF1	down	xtr-miR-429_R+2	down
GATM	up	aca-miR-181a_R+3	up
GATM	up	csa-let-7d_1ss10TC	up
GATM	up	gga-let-7a-5p	down
GATM	up	gga-let-7a-5p_2ss12GT18TC	down
GATM	up	gga-let-7b_R+2	down
GATM	up	gga-let-7c-5p_1ss17AG	down
GATM	up	gga-let-7g-5p_R+1_2ss14TG18CA	down
GATM	up	gga-let-7k-5p_R+1_1ss16AT	down
GATM	up	gga-miR-1663-5p_R+2	up
GATM	up	gga-miR-1796	up
GATM	up	gga-miR-181a-5p	up
GATM	up	gga-miR-181b-5p_R+1	up
GATM	up	gga-miR-22-3p	down
GATM	up	gga-miR-30a-5p_R+2_1ss13CT	up

GATM	up	gga-miR-30b-5p	up
GATM	up	gga-miR-30c-5p	up
GATM	up	gga-miR-30d_R+2	up
GATM	up	gga-miR-30e-5p_R+5	up
GATM	up	gga-miR-489-3p_L-2R+1	up
GATM	up	mmu-let-7j_1ss8TG	down
GATM	up	mmu-mir-5098-p5	up
GATM	up	oha-miR-7-1-3p_1ss10GA	up
GATM	up	pma-miR-181a-5p_R+3	up
GATM	up	tgu-miR-30a-5p_R+2	up
DOCK4	down	gga-miR-20b-5p	up
HNF4beta	up	gga-miR-1783_R+1	up
HNF4beta	up	gga-miR-181b-1-3p_L+2R-2	up
HNF4beta	up	hsa-miR-181b-3p_L+1R-1	up
CD151	down	PC-3p-68190_58	up
CD151	down	aca-miR-129b-5p_R+1_1ss20TG	up
CD151	down	dre-miR-24_R+2	down
CD151	down	gga-miR-130b-5p	up
CD151	down	gga-miR-130b-5p_R-1	up
CD151	down	gga-miR-130b-5p_R-3	up
CD151	down	gga-miR-1467-3p_L+1	up
CD151	down	gga-miR-1668-3p	up
CD151	down	gga-miR-1783_R+1	up
CD151	down	gga-miR-1784-5p	down
CD151	down	gga-miR-222b-5p_R-4	down
CD151	down	gga-miR-29a-5p_L-1R-2	down

CD151	down	hsa-miR-4443_R+1	up
CD151	down	oan-miR-19b-1-5p_R+4	up
ARPP19	up	cin-miR-33_R+4	down
ARPP19	up	gga-miR-15b-5p_R-1	up
ARPP19	up	gga-miR-16c-5p	up
ARPP19	up	gga-miR-1729-5p_R-1	down
ARPP19	up	gga-miR-20b-3p_R-1	up
ARPP19	up	gga-miR-33-5p	down
BIRC5	up	aca-miR-129b-5p_R+1_1ss20TG	up
BIRC5	up	cgr-miR-500	up
BIRC5	up	gga-miR-383-3p	up
BIRC5	up	mmu-miR-500-3p_R-1	up
BIRC5	up	oan-miR-19b-1-5p_R+4	up
CASP18	down	ggo-miR-342_R+1	up
LSP1	down	cin-miR-33_R+4	down
LSP1	down	gga-miR-107-3p_R-1	up
LSP1	down	gga-miR-33-5p	down
YBX1	up	gga-miR-137-3p_L+1	up
YBX1	up	hsa-miR-3120-3p_R+1	down
CD72	down	gga-miR-1552-3p_R+1	up
CD72	down	gga-miR-20b-3p_R-1	up
LDHA	down	gga-let-7c-3p_1ss12TC	up
LDHA	down	gga-miR-106-3p_R+1	up
LDHA	down	gga-miR-130b-3p	up
LDHA	down	gga-miR-1329-5p	down
LDHA	down	gga-miR-1467-3p_L+1	up

LDHA	down	gga-miR-17-3p_L-1R+3	up
LDHA	down	gga-miR-20b-5p	up
LDHA	down	gga-miR-454-3p	up
LDHA	down	gga-miR-9-5p	down
NBL1	down	gga-miR-106-3p_R+1	up
NBL1	down	gga-miR-1602	up
NBL1	down	gga-miR-1699_L-1R-2	up
NBL1	down	gga-miR-20b-5p	up
NBL1	down	gga-miR-6548-5p	up
NBL1	down	gga-mir-1609-p3	up
NBL1	down	oan-miR-19b-1-5p_R+4	up
NBL1	down	ola-miR-126-5p_R+1_1ss1CN	down
NUF2	up	aca-miR-129b-5p_R+1_1ss20TG	up
NUF2	up	gga-mir-147-p3_1	down
NUF2	up	gga-mir-147-p5	down
CYP1A4	up	gga-miR-15b-5p_R-1	up
CYP1A4	up	gga-miR-16c-5p	up
CYP2H1	down	aca-miR-363-3p_R+1	down
CYP2H1	down	cgr-miR-326	up
CYP2H1	down	gga-let-7c-3p_1ss12TC	up
CYP2H1	down	gga-miR-1329-5p	down
CYP2H1	down	gga-miR-146a-3p_R-1	down
CYP2H1	down	gga-miR-1668-3p	up
CYP2H1	down	gga-miR-1784-5p	down
CYP2H1	down	gga-miR-20b-3p_R-1	up
CYP2H1	down	gga-miR-222b-5p_R-4	down

CYP2H1	down	gga-miR-456-3p_1ss22AT	up
CYP2H1	down	gga-miR-456-3p_R-1	up
CYP2H1	down	oan-miR-363-3p_R+1	up
CYP2H1	down	pma-miR-456_R+1_1ss22AC	up
IRF6	down	gga-miR-1329-5p	down
REL	down	aca-miR-181a_R+3	up
REL	down	gga-miR-106-3p_R+1	up
REL	down	gga-miR-107-5p_R+1	up
REL	down	gga-miR-130b-5p	up
REL	down	gga-miR-130b-5p_R-1	up
REL	down	gga-miR-130b-5p_R-3	up
REL	down	gga-miR-144-3p_L-1R-2	up
REL	down	gga-miR-146b-3p_L+2	down
REL	down	gga-miR-146c-5p_4ss18GA20AG22T G23GT	down
REL	down	gga-miR-16-1-3p_R-1	up
REL	down	gga-miR-1668-3p	up
REL	down	gga-miR-16c-3p_L+1R-1	up
REL	down	gga-miR-17-3p_L-1R+3	up
REL	down	gga-miR-1729-5p_R-1	down
REL	down	gga-miR-1784-5p	down
REL	down	gga-miR-1796	up
REL	down	gga-miR-460a-5p	up
REL	down	ggo-miR-342_R+1	up
REL	down	hsa-miR-143-5p	up
REL	down	hsa-miR-4443_R+1	up

REL	down	pma-miR-181a-5p_R+3	up
LIMD2	down	dre-miR-24_R+2	down
LIMD2	down	gga-miR-1754-5p	up
LIMD2	down	gga-miR-1783_R+1	up
LIMD2	down	gga-miR-222b-5p_R-4	down
LIMD2	down	gga-miR-301a-5p_L+2	up
LIMD2	down	hsa-miR-4286_R+1	up
LIMK1	up	gga-miR-128-1-5p	up
LIMK1	up	gga-miR-147_R+1	down
RCC2	up	gga-let-7b_L-1R-1	down
RCC2	up	gga-miR-147_R+1	down
RCC2	up	gga-miR-2954_R+2	down
RCC2	up	gga-miR-29c-3p_L+1R+1_1ss19GA	down
RCC2	up	gga-miR-455-5p	up
RCC2	up	hsa-miR-3120-3p_R+1	down
RCC2	up	oan-miR-19b-1-5p_R+4	up
ARHGEF10L	up	csa-let-7d_1ss10TC	up
ARHGEF10L	up	gga-let-7a-5p	down
ARHGEF10L	up	gga-let-7a-5p_2ss12GT18TC	down
ARHGEF10L	up	gga-let-7b_R+2	down
ARHGEF10L	up	gga-let-7c-5p_1ss17AG	down

ARHGEF10 L	up	gga-let-7g-5p_R+1_2ss14TG18CA	down
ARHGEF10 L	up	gga-let-7k-5p_R+1_1ss16AT	down
ARHGEF10 L	up	mmu-let-7j_1ss8TG	down
NUDT1	up	gga-miR-1784-5p	down
NUDT1	up	gga-miR-6548-5p	up
ANG	down	PC-3p-68190_58	up
ANG	down	gga-miR-107-3p_R-1	up
ANG	down	gga-miR-15b-5p_R-1	up
ANG	down	gga-miR-16c-5p	up
ANG	down	gga-miR-365-2-5p_L-1R+1	up
ANG	down	mmu-mir-5098-p5	up
SLU7	down	cgr-miR-151-3p	up
SLU7	down	cin-miR-33_R+4	down
SLU7	down	gga-let-7c-3p_1ss12TC	up
SLU7	down	gga-miR-1329-5p	down
SLU7	down	gga-miR-1467-3p_L+1	up
SLU7	down	gga-miR-17-3p_L-1R+3	up
SLU7	down	gga-miR-30a-5p_R+2_1ss13CT	up
SLU7	down	gga-miR-30d_R+2	up
SLU7	down	gga-miR-30e-5p_R+5	up
SLU7	down	gga-miR-33-5p	down
SLU7	down	gga-miR-34a-3p_R-1	up
SLU7	down	gga-miR-460a-5p	up

SLU7	down	gga-miR-490-3p_R+1	up
SLU7	down	tgu-miR-30a-5p_R+2	up
PFKL	up	gga-miR-128-1-5p	up
PFKL	up	gga-miR-138-5p_R+7	up
PFKL	up	gga-miR-15b-5p_R-1	up
PFKL	up	gga-miR-16c-5p	up
PFKL	up	gga-miR-6604-5p_1ss7GA	up
PFKL	up	gga-mir-147-p3_2	down
APITD1	up	aca-miR-18a-5p	up
APITD1	up	gga-miR-222b-5p_R-4	down
APITD1	up	oha-miR-7-1-3p_1ss10GA	up
HSBP1L1	down	aca-miR-425-5p_R+4	down
HSBP1L1	down	gga-miR-125b-5p	down
HSBP1L1	down	gga-miR-1759-3p_L-3	up
HSBP1L1	down	gga-miR-181b-1-3p_L+1R-1	up
HSBP1L1	down	gga-miR-30a-5p_R+2_1ss13CT	up
HSBP1L1	down	gga-miR-30b-5p	up
HSBP1L1	down	gga-miR-30c-5p	up
HSBP1L1	down	gga-miR-30d_R+2	up
HSBP1L1	down	gga-miR-30e-5p_R+5	up
HSBP1L1	down	hsa-miR-425-5p_R+2_1ss20TC	down
HSBP1L1	down	sha-miR-125a_R+2	down
HSBP1L1	down	ssa-miR-125a-5p_R+2	down
HSBP1L1	down	tgu-miR-30a-5p_R+2	up

Table S12. Intersection target differentially expressed genes of 191 differentially expressed miRNAs (which were selected with the criteria of P < 0.05 and fold changes either > 1.5 or < 0.67.) in mRNA levels.

differently expressed mRNAs	gene regulation(immature VS mature liver)	miRNA ID	miRNA regulation(immature VS mature liver)
ABCC1	down	gga-miR-107-3p_R-1	up
ABCC1	down	gga-miR-15b-5p_R-1	up
ABCC1	down	gga-miR-16c-5p	up
ABCC1	down	gga-miR-1783_R+1	up
ABCC1	down	gga-miR-2131-3p_R+1	up
ABCC1	down	gga-miR-301a-5p_L+2	up
ABCC1	down	gga-miR-30a-3p_1ss18GA	up
ABCC1	down	gga-miR-30a-3p_2ss10GA20AT	up
ABCC1	down	gga-miR-30e-3p_L+1	up
ABCC1	down	mmu-mir-5098-p5	up
ABCC1	down	oan-miR-19b-1-5p_R+4	up
ABCC1	down	PC-5p-479025_5	up
ABHD12	up	gga-miR-142-3p	down
ABHD12	up	gga-miR-146a-3p_R-1	down
ABHD17B	down	gga-miR-106-3p_R+1	up
ABHD17B	down	gga-miR-17-3p_L-1R+3	up
ABHD17B	down	gga-miR-1796	up
ABHD17B	down	gga-miR-222a	up
ABHD17B	down	gga-miR-429-3p	up

ABHD17B	down	hsa-miR-143-5p	up
ABHD5	down	gga-miR-107-5p_R+1	up
ABHD5	down	gga-miR-460a-5p	up
ACADSB	down	aca-miR-18a-3p_L+1R-1_1ss12GA	up
ACADSB	down	csa-let-7d_1ss10TC	up
ACADSB	down	gga-miR-130b-3p	up
ACADSB	down	gga-miR-1699_L-1R-2	up
ACADSB	down	gga-miR-1788-3p_R+1	up
ACADSB	down	gga-miR-1788-5p	up
ACADSB	down	gga-miR-216b	up
ACADSB	down	gga-miR-301a-5p_L+2	up
ACADSB	down	gga-miR-3529	up
ACADSB	down	gga-miR-383-3p	up
ACADSB	down	gga-miR-454-3p	up
ACADSB	down	gga-miR-460a-5p	up
ACADSB	down	gga-miR-6548-5p	up
ACADSB	down	mmu-mir-5098-p5	up
ACADSB	down	oha-miR-7-1-3p_1ss10GA	up
ACO2	up	aca-miR-363-3p_R+1	down
ACOT11	up	hsa-miR-3120-3p_R+1	down
ACOT7	up	gga-miR-9-5p	down
ACSBG2	down	gga-miR-106-3p_R+1	up
ACSBG2	down	gga-miR-1783_R+1	up
ACSBG2	down	gga-miR-181b-5p_R+1	up
ACSBG2	down	gga-miR-205a_R+1	up
ACSBG2	down	gga-miR-205b	up

ACSBG2	down	gga-miR-460a-5p	up
ACSBG2	down	ggo-miR-342_R+1	up
ACSBG2	down	oan-miR-363-3p_R+1	up
ACSBG2	down	tni-miR-205_R+2	up
ADA	down	cgr-miR-500	up
ADA	down	gga-miR-125b-3p_R-1_1ss21CT	up
ADA	down	gga-miR-181b-1-3p_L+1R-1	up
ADA	down	gga-miR-202-5p_L-1	up
ADA	down	gga-miR-20b-3p_R-1	up
ADA	down	gga-miR-301a-5p_L+2	up
ADA	down	gga-miR-429-3p	up
ADA	down	mmu-miR-500-3p_R-1	up
ADAMTS7	up	gga-miR-222b-5p_R-4	down
ADAMTS7	up	gga-miR-29b-3p_R-1	down
ADAMTS7	up	gga-miR-29c-3p_L+1R+1_1ss19GA	down
ADAMTS7	up	gga-miR-29c-3p_R+1	down
ADAMTS7	up	gga-miR-29c-3p_R+1_1ss18GA	down
ADAMTS7	up	ssa-miR-29b-3p_R-1_2ss20TC21TC	down
ADAP2	down	gga-miR-181b-1-3p_L+1R-1	up
AHSG	up	gga-miR-223_1ss15AG	down
AHSG	up	gga-miR-223_1ss19CT	down
AHSG	up	gga-miR-223_R+1	down
AIMP2	up	gga-miR-458a-3p	down
AKR1D1	up	gga-let-7a-5p	down
AKR1D1	up	gga-let-7a-5p_2ss12GT18TC	down
AKR1D1	up	gga-let-7b_R+2	down

AKR1D1	up	gga-let-7c-5p_1ss17AG	down
AKR1D1	up	gga-let-7g-5p_R+1_2ss14TG18CA	down
AKR1D1	up	gga-let-7k-5p_R+1_1ss16AT	down
AKR1D1	up	gga-miR-155_R+1	down
AKR1D1	up	gga-miR-22-3p	down
AKR1D1	up	gga-miR-29b-3p_R-1	down
AKR1D1	up	gga-miR-29c-3p_R+1	down
AKR1D1	up	gga-miR-29c-3p_R+1_1ss18GA	down
AKR1D1	up	mmu-let-7j_1ss8TG	down
AKR1D1	up	ssa-miR-29b-3p_R-1_2ss20TC21TC	down
ALCAM	down	aca-miR-18a-5p	up
ALCAM	down	gga-miR-1788-3p_R+1	up
ALCAM	down	gga-miR-181b-1-3p_L+1R-1	up
ALCAM	down	gga-miR-3523	up
ALCAM	down	gga-miR-6548-5p	up
ALCAM	down	gga-miR-7460-3p	up
ALCAM	down	gga-miR-92-5p	up
ALCAM	down	mmu-mir-5098-p5	up
ALCAM	down	oan-miR-19b-1-5p_R+4	up
ALDH3A2	up	gga-let-7b_L-1R-1	down
ALDH3A2	up	ola-miR-126-5p_R+1_1ss1CN	down
ALDH4A1	up	ola-miR-199a-3p_R+1_1ss22AG	down
ANG	down	gga-miR-107-3p_R-1	up
ANG	down	gga-miR-15b-5p_R-1	up
ANG	down	gga-miR-16c-5p	up
ANG	down	gga-miR-365-2-5p_L-1R+1	up

ANG	down	mmu-mir-5098-p5	up
ANG	down	PC-3p-68190_58	up
ANKRA2	down	gga-miR-489-3p_L-2R+1	up
ANKRA2	down	gga-miR-6548-5p	up
ANKRA2	down	mmu-miR-8094_L+1R+3_1ss3AG	up
ANKRD44	down	aca-miR-138-2-3p_L-2R+3	up
ANKRD44	down	gga-miR-181b-1-3p_L+1R-1	up
ANKRD44	down	mmu-mir-5098-p5	up
ANXA11	down	gga-miR-202-5p_L-1	up
ANXA11	down	gga-miR-6604-5p_1ss7GA	up
ANXA11	down	mmu-miR-8094_L+1R+3_1ss3AG	up
AP3S1	down	chi-miR-361-5p	up
AP3S1	down	efu-miR-361	up
AP3S1	down	gga-miR-107-3p_R-1	up
AP3S1	down	gga-miR-1451-3p_R-1	up
AP3S1	down	gga-miR-1788-3p_R+1	up
AP3S1	down	gga-miR-20b-3p_R-1	up
AP3S1	down	gga-miR-30a-3p_2ss10GA20AT	up
AP3S1	down	gga-miR-30a-5p_R+2_1ss13CT	up
AP3S1	down	gga-miR-30b-5p	up
AP3S1	down	gga-miR-30c-5p	up
AP3S1	down	gga-miR-30d_R+2	up
AP3S1	down	gga-miR-30e-5p_R+5	up
AP3S1	down	gga-miR-383-3p	up
AP3S1	down	gga-miR-6548-5p	up
AP3S1	down	tgu-miR-30a-5p_R+2	up

APITD1	up	gga-miR-222b-5p_R-4	down
ARHGAP19	up	gga-miR-140-5p_L+1	down
ARHGAP25	down	gga-miR-107-3p_R-1	up
ARHGAP25	down	gga-miR-130b-5p	up
ARHGAP25	down	gga-miR-130b-5p_R-1	up
ARHGAP25	down	gga-miR-130b-5p_R-3	up
ARHGAP25	down	gga-miR-138-5p_R+7	up
ARHGAP25	down	gga-miR-1555-5p	up
ARHGAP25	down	gga-miR-15b-5p_R-1	up
ARHGAP25	down	gga-miR-16c-5p	up
ARHGAP25	down	gga-miR-30a-3p_1ss18GA	up
ARHGAP25	down	gga-miR-30a-3p_2ss10GA20AT	up
ARHGAP25	down	gga-miR-30e-3p_L+1	up
ARHGAP25	down	gga-miR-460a-5p	up
ARHGAP25	down	mmu-miR-8094_L+1R+3_1ss3AG	up
ARHGAP25	down	PC-5p-479025_5	up
ARHGEF10L	up	gga-let-7a-5p	down
ARHGEF10L	up	gga-let-7a-5p_2ss12GT18TC	down
ARHGEF10L	up	gga-let-7b_R+2	down
ARHGEF10L	up	gga-let-7c-5p_1ss17AG	down
ARHGEF10L	up	gga-let-7g-5p_R+1_2ss14TG18CA	down
ARHGEF10L	up	gga-let-7k-5p_R+1_1ss16AT	down
ARHGEF10L	up	mmu-let-7j_1ss8TG	down
ARHGEF3	down	aca-miR-181a_R+3	up
ARHGEF3	down	cgr-miR-151-3p	up
ARHGEF3	down	gga-miR-107-5p_R+1	up

ARHGEF3	down	gga-miR-130b-5p	up
ARHGEF3	down	gga-miR-130b-5p_R-1	up
ARHGEF3	down	gga-miR-130b-5p_R-3	up
ARHGEF3	down	gga-miR-16c-3p_L+1R-1	up
ARHGEF3	down	gga-miR-181a-5p	up
ARHGEF3	down	gga-miR-181b-1-3p_L+1R-1	up
ARHGEF3	down	gga-miR-181b-5p_R+1	up
ARHGEF3	down	gga-miR-20b-3p_R-1	up
ARHGEF3	down	gga-miR-20b-5p	up
ARHGEF3	down	gga-miR-30a-5p_R+2_1ss13CT	up
ARHGEF3	down	gga-miR-30b-5p	up
ARHGEF3	down	gga-miR-30c-5p	up
ARHGEF3	down	gga-miR-30d_R+2	up
ARHGEF3	down	gga-miR-30e-5p_R+5	up
ARHGEF3	down	gga-miR-34a-3p_R-1	up
ARHGEF3	down	gga-miR-429-3p	up
ARHGEF3	down	gga-miR-460a-5p	up
ARHGEF3	down	gga-miR-6710-3p_R+1	up
ARHGEF3	down	mmu-mir-5098-p5	up
ARHGEF3	down	pma-miR-181a-5p_R+3	up
ARHGEF3	down	tgu-miR-30a-5p_R+2	up
ARL6IP5	down	gga-miR-137-3p_L+1	up
ARL6IP5	down	gga-miR-1559-3p	up
ARL6IP5	down	gga-miR-1663-5p_R+2	up
ARL6IP5	down	gga-miR-1720-5p	up
ARL6IP5	down	gga-miR-205a_R+1	up

ARL6IP5	down	gga-miR-205b	up
ARL6IP5	down	gga-miR-30a-5p_R+2_1ss13CT	up
ARL6IP5	down	gga-miR-30b-5p	up
ARL6IP5	down	gga-miR-30c-5p	up
ARL6IP5	down	gga-miR-30d_R+2	up
ARL6IP5	down	gga-miR-30e-5p_R+5	up
ARL6IP5	down	gga-miR-383-3p	up
ARL6IP5	down	gga-miR-429-3p	up
ARL6IP5	down	hsa-miR-143-5p	up
ARL6IP5	down	tgu-miR-30a-5p_R+2	up
ARL6IP5	down	tni-miR-205_R+2	up
ARPP19	up	cin-miR-33_R+4	down
ARPP19	up	gga-miR-1729-5p_R-1	down
ARPP19	up	gga-miR-33-5p	down
ARRDC2	up	gga-miR-214_L+1R-3_1ss19GA	down
ARRDC2	up	gga-miR-29b-3p_R-1	down
ARRDC2	up	gga-miR-29c-3p_R+1	down
ARRDC2	up	gga-miR-29c-3p_R+1_1ss18GA	down
ARRDC2	up	ssa-miR-29b-3p_R-1_2ss20TC21TC	down
ARRDC4	down	aca-miR-181a_R+3	up
ARRDC4	down	gga-miR-1552-3p_R+1	up
ARRDC4	down	gga-miR-1759-3p_L-3	up
ARRDC4	down	gga-miR-181a-5p	up
ARRDC4	down	gga-miR-181b-5p_R+1	up
ARRDC4	down	gga-miR-30a-3p_1ss18GA	up
ARRDC4	down	gga-miR-30e-3p_L+1	up

ARRDC4	down	pma-miR-181a-5p_R+3	up
ASAP1	down	gga-miR-144-3p_L-1R-2	up
ASAP1	down	gga-miR-1720-5p	up
ASAP1	down	gga-miR-1788-3p_R+1	up
ASAP1	down	gga-miR-429-3p	up
ASB9	down	gga-miR-125b-3p_R-1_1ss21CT	up
ASB9	down	gga-miR-1796	up
ATG12	down	gga-miR-1467-3p_L+1	up
ATG12	down	gga-miR-205a_R+1	up
ATG12	down	gga-miR-205b	up
ATG12	down	tni-miR-205_R+2	up
ATN1	up	gga-miR-1784-5p	down
ATN1	up	gga-miR-200b-5p_L+2	down
AURKA	up	ola-miR-199a-3p_R+1_1ss22AG	down
B4GALT3	down	gga-miR-20b-3p_R-1	up
BAG3	down	aca-miR-363-5p	up
BAG3	down	cgr-miR-500	up
BAG3	down	gga-let-7c-3p_1ss12TC	up
BAG3	down	gga-miR-138-5p_R+7	up
BAG3	down	gga-miR-1559-3p	up
BAG3	down	gga-miR-1663-5p_R+2	up
BAG3	down	gga-miR-1731-5p	up
BAG3	down	gga-miR-1783_R+1	up
BAG3	down	gga-miR-20b-5p	up
BAG3	down	gga-miR-30a-3p_1ss18GA	up
BAG3	down	gga-miR-30a-3p_2ss10GA20AT	up

BAG3	down	gga-miR-30e-3p_L+1	up
BAG3	down	hsa-miR-4443_R+1	up
BAG3	down	mmu-miR-500-3p_R-1	up
BAG3	down	PC-5p-479025_5	up
BAG5	down	aca-miR-129b-5p_R+1_1ss20TG	up
BAG5	down	aca-miR-181a_R+3	up
BAG5	down	gga-miR-107-5p_R+1	up
BAG5	down	gga-miR-138-5p_R+7	up
BAG5	down	gga-miR-1559-3p	up
BAG5	down	gga-miR-1663-5p_R+2	up
BAG5	down	gga-miR-1668-3p	up
BAG5	down	gga-miR-1759-3p_L-3	up
BAG5	down	gga-miR-1788-3p_R+1	up
BAG5	down	gga-miR-181a-5p	up
BAG5	down	gga-miR-181b-5p_R+1	up
BAG5	down	gga-miR-383-3p	up
BAG5	down	gga-miR-460a-3p	up
BAG5	down	gga-miR-460a-5p	up
BAG5	down	oha-miR-7-1-3p_1ss10GA	up
BAG5	down	pma-miR-181a-5p_R+3	up
BARD1	up	aca-miR-363-3p_R+1	down
BARD1	up	gga-miR-1329-5p	down
BARD1	up	gga-miR-155_R+1	down
BF1	down	gga-miR-138-5p_R+7	up
BIK	down	gga-miR-137-3p_L+1	up
BIK	down	gga-miR-490-3p_R+1	up

BIK	down	ggo-miR-342_R+1	up
BIRC2	down	gga-miR-16c-5p	up
BIRC2	down	gga-miR-6548-5p	up
BIRC2	down	gga-miR-6710-3p_R+1	up
BIRC2	down	PC-5p-479025_5	up
BLK	down	hsa-miR-4443_R+1	up
BPGM	up	cin-miR-33_R+4	down
BPGM	up	gga-miR-214_L+1R-3_1ss19GA	down
BPGM	up	gga-miR-22-3p	down
BPGM	up	gga-miR-29b-3p_R-1	down
BPGM	up	gga-miR-29c-3p_R+1	down
BPGM	up	gga-miR-29c-3p_R+1_1ss18GA	down
BPGM	up	gga-miR-33-5p	down
BPGM	up	hsa-miR-3120-3p_R+1	down
BPGM	up	ola-miR-199a-3p_R+1_1ss22AG	down
BPGM	up	ssa-miR-29b-3p_R-1_2ss20TC21TC	down
BTG1	down	gga-let-7c-3p_1ss12TC	up
BTG1	down	gga-miR-130b-3p	up
BTG1	down	gga-miR-181b-1-3p_L+1R-1	up
BTG1	down	gga-miR-2131-3p_R+1	up
BTG1	down	gga-miR-454-3p	up
BTN1A1	down	gga-miR-1796	up
BTN1A1	down	gga-miR-205a_R+1	up
BTN1A1	down	gga-miR-205b	up
BTN1A1	down	gga-miR-460a-3p	up
BTN1A1	down	tni-miR-205_R+2	up

BUB1	up	gga-miR-214_L+1R-3_1ss19GA	down
C13H5ORF15	down	gga-miR-107-3p_R-1	up
C13H5ORF15	down	gga-miR-1668-3p	up
C13H5ORF15	down	gga-miR-3607-5p_R+1	up
C1S	down	gga-miR-1699_L-1R-2	up
C2H3ORF75	up	gga-miR-223_1ss15AG	down
C2H3ORF75	up	gga-miR-223_1ss19CT	down
C2H3ORF75	up	gga-miR-223_R+1	down
C3H2ORF43	down	aca-miR-129b-5p_R+1_1ss20TG	up
C3H2ORF43	down	aca-miR-363-5p	up
C3H2ORF43	down	gga-miR-125b-3p_R-1_1ss21CT	up
C3H2ORF43	down	gga-miR-137-3p_L+1	up
C3H2ORF43	down	gga-miR-1451-3p_R-1	up
C3H2ORF43	down	gga-miR-1552-3p_R+1	up
C3H2ORF43	down	gga-miR-1747-3p	up
C3H2ORF43	down	gga-miR-30a-5p_R+2_1ss13CT	up
C3H2ORF43	down	gga-miR-30b-5p	up
C3H2ORF43	down	gga-miR-30c-5p	up
C3H2ORF43	down	gga-miR-30d_R+2	up
C3H2ORF43	down	gga-miR-30e-5p_R+5	up
C3H2ORF43	down	gga-miR-6548-5p	up
C3H2ORF43	down	ggo-miR-342_R+1	up
C3H2ORF43	down	hsa-miR-4286_R+1	up
C3H2ORF43	down	mmu-mir-5098-p5	up
C3H2ORF43	down	mmu-miR-8094_L+1R+3_1ss3AG	up
C3H2ORF43	down	oha-miR-7-1-3p_1ss10GA	up

C3H2ORF43	down	tgu-miR-30a-5p_R+2	up
C7	down	aca-miR-363-5p	up
C7	down	gga-miR-383-3p	up
CACYBP	down	gga-miR-30a-5p_R+2_1ss13CT	up
CACYBP	down	gga-miR-30b-5p	up
CACYBP	down	gga-miR-30c-5p	up
CACYBP	down	gga-miR-30d_R+2	up
CACYBP	down	gga-miR-30e-5p_R+5	up
CACYBP	down	tgu-miR-30a-5p_R+2	up
CAMK2D	down	aca-miR-181a_R+3	up
CAMK2D	down	chi-miR-361-5p	up
CAMK2D	down	efu-miR-361	up
CAMK2D	down	gga-miR-128-1-5p	up
CAMK2D	down	gga-miR-1559-3p	up
CAMK2D	down	gga-miR-1663-5p_R+2	up
CAMK2D	down	gga-miR-1796	up
CAMK2D	down	gga-miR-181a-5p	up
CAMK2D	down	gga-miR-181b-5p_R+1	up
CAMK2D	down	gga-miR-205a_R+1	up
CAMK2D	down	gga-miR-205b	up
CAMK2D	down	gga-miR-30b-5p	up
CAMK2D	down	gga-miR-30c-5p	up
CAMK2D	down	gga-miR-383-3p	up
CAMK2D	down	gga-miR-460a-3p	up
CAMK2D	down	mmu-mir-5098-p5	up
CAMK2D	down	pma-miR-181a-5p_R+3	up

CAMK2D	down	tni-miR-205_R+2	up
CAMK4	up	aca-miR-425-5p_R+4	down
CAMK4	up	cin-miR-33_R+4	down
CAMK4	up	gga-let-7b_L-1R-1	down
CAMK4	up	gga-miR-196-5p_R+2	down
CAMK4	up	gga-miR-2954_R+2	down
CAMK4	up	gga-miR-33-5p	down
CAMK4	up	hsa-miR-425-5p_R+2_1ss20TC	down
CASP18	down	ggo-miR-342_R+1	up
CCDC127	down	aca-miR-129b-5p_R+1_1ss20TG	up
CCDC127	down	gga-miR-106-3p_R+1	up
CCDC127	down	gga-miR-17-3p_L-1R+3	up
CCDC127	down	gga-miR-1747-3p	up
CCDC127	down	gga-miR-181b-1-3p_L+2R-2	up
CCDC127	down	gga-miR-456-3p_1ss22AT	up
CCDC127	down	gga-miR-456-3p_R-1	up
CCDC127	down	hsa-miR-181b-3p_L+1R-1	up
CCDC127	down	oan-miR-19b-1-5p_R+4	up
CCDC127	down	oan-miR-363-3p_R+1	up
CCDC127	down	pma-miR-456_R+1_1ss22AC	up
CCL20	down	gga-let-7c-3p_1ss12TC	up
CCL20	down	gga-miR-6548-5p	up
CCLI10	down	gga-miR-15b-3p_L+1_1ss22AC	up
CCLI10	down	gga-miR-1747-3p	up
CCLI10	down	PC-5p-479025_5	up
CCNA2	up	gga-miR-140-3p_L+1R+1_1ss22CA	down

CCNA2	up	gga-miR-223_1ss15AG	down
CCNA2	up	hsa-miR-3120-3p_R+1	down
CCNA2	up	sha-miR-140_R+2	down
CCND3	down	cgr-miR-151-3p	up
CCND3	down	cgr-miR-326	up
CCND3	down	gga-miR-15b-5p_R-1	up
CCND3	down	gga-miR-1602	up
CCND3	down	gga-miR-16c-5p	up
CCND3	down	gga-miR-1759-3p_L-3	up
CCR8	down	aca-miR-18a-5p	up
CCR8	down	gga-miR-1555-5p	up
CCR8	down	gga-miR-15b-3p_L+1_1ss22AC	up
CCR8	down	gga-miR-1663-5p_R+2	up
CCR8	down	gga-miR-1796	up
CCR8	down	gga-miR-30a-3p_1ss18GA	up
CCR8	down	gga-miR-30a-3p_2ss10GA20AT	up
CCR8	down	gga-miR-30c-1-3p_R+1	up
CCR8	down	gga-miR-30e-3p_L+1	up
CCR8	down	gga-miR-92-5p	up
CCR8	down	oan-miR-363-3p_R+1	up
CD151	down	aca-miR-129b-5p_R+1_1ss20TG	up
CD151	down	gga-miR-130b-5p	up
CD151	down	gga-miR-130b-5p_R-1	up
CD151	down	gga-miR-130b-5p_R-3	up
CD151	down	gga-miR-1467-3p_L+1	up
CD151	down	gga-miR-1668-3p	up

CD151	down	gga-miR-1783_R+1	up
CD151	down	hsa-miR-4443_R+1	up
CD151	down	oan-miR-19b-1-5p_R+4	up
CD151	down	PC-3p-68190_58	up
CD247	down	gga-miR-1754-5p	up
CD247	down	gga-miR-1788-5p	up
CD247	down	gga-miR-2131-3p_R+1	up
CD247	down	gga-miR-460a-5p	up
CD28	down	gga-miR-181b-1-3p_L+2R-2	up
CD28	down	hsa-miR-181b-3p_L+1R-1	up
CD38	down	gga-miR-107-3p_R-1	up
CD38	down	gga-miR-15b-5p_R-1	up
CD38	down	gga-miR-16c-5p	up
CD38	down	gga-miR-222a	up
CD38	down	gga-miR-30a-3p_1ss18GA	up
CD38	down	gga-miR-30e-3p_L+1	up
CD38	down	gga-miR-3607-5p_R+1	up
CD38	down	gga-miR-383-3p	up
CD3D	down	gga-miR-15b-5p_R-1	up
CD44	down	aca-miR-129b-5p_R+1_1ss20TG	up
CD44	down	cgr-miR-500	up
CD44	down	gga-miR-125b-3p_R-1_1ss21CT	up
CD44	down	gga-miR-138-5p_R+7	up
CD44	down	gga-miR-1451-3p_R-1	up
CD44	down	gga-miR-2131-3p_R+1	up
CD44	down	gga-miR-3529	up

CD44	down	gga-miR-489-3p_L-2R+1	up
CD44	down	gga-miR-7460-3p	up
CD44	down	mmu-miR-500-3p_R-1	up
CD72	down	gga-miR-1552-3p_R+1	up
CD72	down	gga-miR-20b-3p_R-1	up
CD82	down	aca-miR-18a-5p	up
CD82	down	chi-miR-361-5p	up
CD82	down	efu-miR-361	up
CD82	down	gga-miR-107-5p_R+1	up
CD82	down	gga-miR-15b-5p_R-1	up
CD82	down	gga-miR-1602	up
CD82	down	gga-miR-1663-5p_R+2	up
CD82	down	gga-miR-16c-5p	up
CD82	down	gga-mir-1724-p5_1ss21CT	up
CD82	down	gga-miR-456-3p_1ss22AT	up
CD82	down	gga-miR-456-3p_R-1	up
CD82	down	hsa-miR-4443_R+1	up
CD82	down	PC-5p-479025_5	up
CD82	down	pma-miR-456_R+1_1ss22AC	up
CD8A	down	gga-miR-15b-5p_R-1	up
CD8A	down	gga-miR-1668-3p	up
CD8A	down	gga-miR-16c-5p	up
CD8A	down	gga-miR-92-5p	up
CDC20	up	xtr-miR-429_R+2	down
CDH17	down	gga-miR-106-3p_R+1	up
CDH17	down	gga-miR-17-3p_L-1R+3	up

CDH17	down	gga-miR-365-2-5p_L-1R+1	up
CDH17	down	hsa-miR-143-5p	up
CDS1	down	gga-miR-383-3p	up
CFL2	up	aca-miR-425-5p_R+4	down
CFL2	up	gga-miR-142-3p	down
CFL2	up	gga-miR-1662_R-3	down
CFL2	up	gga-miR-223_1ss15AG	down
CFL2	up	gga-miR-223_1ss19CT	down
CFL2	up	gga-miR-223_R+1	down
CFL2	up	hsa-miR-3120-3p_R+1	down
CFL2	up	hsa-miR-425-5p_R+2_1ss20TC	down
CFL2	up	ola-miR-199a-3p_R+1_1ss22AG	down
CFL2	up	xtr-miR-429_R+2	down
CHP1	up	gga-miR-1329-5p	down
CHP1	up	gga-mir-147-p3_1	down
CHP1	up	gga-mir-147-p5	down
CHP1	up	gga-miR-155_R+1	down
CHRDL1	down	aca-miR-138-2-3p_L-2R+3	up
CHRDL1	down	cgr-miR-326	up
CHRDL1	down	chi-miR-361-5p	up
CHRDL1	down	efu-miR-361	up
CHRDL1	down	gga-miR-106-3p_R+1	up
CHRDL1	down	gga-miR-107-3p_R-1	up
CHRDL1	down	gga-miR-107-5p_R+1	up
CHRDL1	down	gga-miR-130b-5p	up
CHRDL1	down	gga-miR-130b-5p_R-1	up

CHRDL1	down	gga-miR-130b-5p_R-3	up
CHRDL1	down	gga-miR-15b-5p_R-1	up
CHRDL1	down	gga-miR-16c-5p	up
CHRDL1	down	gga-miR-17-3p_L-1R+3	up
CHRDL1	down	gga-miR-205a_R+1	up
CHRDL1	down	gga-miR-205b	up
CHRDL1	down	gga-miR-460a-5p	up
CHRDL1	down	gga-miR-489-3p_L-2R+1	up
CHRDL1	down	hsa-miR-4443_R+1	up
CHRDL1	down	tni-miR-205_R+2	up
CHST10	down	aca-miR-18a-3p_L+1R-1_1ss12GA	up
CHST10	down	gga-miR-106-3p_R+1	up
CHST10	down	gga-miR-107-5p_R+1	up
CHST10	down	gga-miR-16c-5p	up
CHST10	down	gga-miR-1731-5p	up
CHST10	down	gga-miR-17-3p_L-1R+3	up
CHST10	down	gga-miR-30a-3p_1ss18GA	up
CHST10	down	gga-miR-30a-3p_2ss10GA20AT	up
CHST10	down	gga-miR-30a-5p_R+2_1ss13CT	up
CHST10	down	gga-miR-30d_R+2	up
CHST10	down	gga-miR-30e-3p_L+1	up
CHST10	down	gga-miR-30e-5p_R+5	up
CHST10	down	PC-3p-68190_58	up
CHST10	down	tgu-miR-30a-5p_R+2	up
CHTF8	up	gga-miR-146a-3p_R-1	down
CHTF8	up	gga-miR-222b-5p_R-4	down

CKAP2	up	aca-miR-191-5p_R-1	down
CLEC3B	down	gga-miR-15b-5p_R-1	up
CLEC3B	down	gga-miR-1699_L-1R-2	up
CLEC3B	down	gga-miR-16c-5p	up
CLEC3B	down	gga-miR-181b-1-3p_L+1R-1	up
CLEC3B	down	gga-miR-181b-1-3p_L+2R-2	up
CLEC3B	down	gga-miR-30c-1-3p_R+1	up
CLEC3B	down	gga-miR-6604-5p_1ss7GA	up
CLEC3B	down	gga-miR-7460-3p	up
CLEC3B	down	hsa-miR-181b-3p_L+1R-1	up
CLEC3B	down	PC-3p-68190_58	up
CMPK1	up	aca-miR-150-5p_1ss12TC	down
CMPK1	up	aca-miR-425-5p_R+4	down
CMPK1	up	gga-miR-146c-5p_4ss18GA20AG22TG23GT	down
CMPK1	up	gga-miR-222b-5p_R-4	down
CMPK1	up	gga-miR-223_1ss15AG	down
CMPK1	up	gga-miR-223_1ss19CT	down
CMPK1	up	gga-miR-223_R+1	down
CMPK1	up	hsa-miR-425-5p_R+2_1ss20TC	down
CMPK1	up	ola-miR-199a-3p_R+1_1ss22AG	down
CMPK1	up	tgu-miR-1784-3p_1ss11GA	down
CNRIP1	down	gga-miR-107-5p_R+1	up
COL18A1	up	gga-let-7j-3p_L-1R+2	down
COL18A1	up	gga-miR-140-3p_L+1R+1_1ss22CA	down
COL18A1	up	gga-miR-140-5p_L+1	down
COL18A1	up	gga-miR-29c-3p_L+1R+1_1ss19GA	down

COL18A1	up	sha-miR-140_R+2	down
COL1A2	up	aca-miR-363-3p_R+1	down
COL1A2	up	gga-let-7a-5p	down
COL1A2	up	gga-let-7a-5p_2ss12GT18TC	down
COL1A2	up	gga-let-7b_L-1R-1	down
COL1A2	up	gga-let-7b_R+2	down
COL1A2	up	gga-let-7c-5p_1ss17AG	down
COL1A2	up	gga-let-7g-5p_R+1_2ss14TG18CA	down
COL1A2	up	gga-let-7k-5p_R+1_1ss16AT	down
COL1A2	up	gga-miR-196-5p_R+2	down
COL1A2	up	gga-miR-29b-3p_R-1	down
COL1A2	up	gga-miR-29c-3p_L+1R+1_1ss19GA	down
COL1A2	up	gga-miR-29c-3p_R+1	down
COL1A2	up	gga-miR-29c-3p_R+1_1ss18GA	down
COL1A2	up	mmu-let-7j_1ss8TG	down
COL1A2	up	ssa-miR-29b-3p_R-1_2ss20TC21TC	down
COL1A2	up	tgu-miR-1662_L+1_1ss23TA	down
COQ10B	down	mmu-miR-8094_L+1R+3_1ss3AG	up
COQ10B	down	oan-miR-363-3p_R+1	up
CORO6	up	dre-miR-24_R+2	down
CORO6	up	xtr-miR-429_R+2	down
COTL1	down	gga-miR-1668-3p	up
COTL1	down	PC-3p-68190_58	up
COX10	up	gga-miR-122-5p	down
COX10	up	gga-miR-140-3p_L+1R+1_1ss22CA	down
COX10	up	gga-miR-142-3p	down

COX10	up	gga-miR-214_L+1R-3_1ss19GA	down
COX10	up	gga-miR-2954_R+2	down
COX10	up	sha-miR-140_R+2	down
COX4I1	up	gga-mir-147-p3_2	down
CPN1	up	gga-miR-1784-5p	down
CPSF4	up	gga-miR-140-3p_L+1R+1_1ss22CA	down
CPSF4	up	gga-miR-214_L+1R-3_1ss19GA	down
CPSF4	up	sha-miR-140_R+2	down
CPXM2	down	aca-miR-129b-5p_R+1_1ss20TG	up
CPXM2	down	gga-miR-1759-3p_L-3	up
CPXM2	down	gga-miR-92-5p	up
CREM	down	gga-miR-130b-3p	up
CREM	down	gga-miR-130b-5p	up
CREM	down	gga-miR-130b-5p_R-1	up
CREM	down	gga-miR-130b-5p_R-3	up
CREM	down	gga-miR-144-3p_L-1R-2	up
CREM	down	gga-miR-20b-5p	up
CRIM1	down	PC-5p-479025_5	up
CRTAM	down	gga-miR-1668-3p	up
CRTAM	down	gga-miR-1788-3p_R+1	up
CRTAM	down	gga-miR-301a-5p_L+2	up
CRTAM	down	gga-miR-490-3p_R+1	up
CRYZ	up	gga-miR-142-5p_L-2R+1	down
CRYZ	up	gga-miR-1784-5p	down
CRYZ	up	mmu-miR-3964_L+1_1ss6GA	down
CRYZ	up	odi-miR-7_R+2	down

CTH	up	gga-miR-214_L+1R-3_1ss19GA	down
CTSL2	down	aca-miR-181a_R+3	up
CTSL2	down	gga-miR-181a-5p	up
CTSL2	down	gga-miR-181b-5p_R+1	up
CTSL2	down	pma-miR-181a-5p_R+3	up
CTSS	down	gga-miR-106-3p_R+1	up
CUBN	up	ola-miR-199a-3p_R+1_1ss22AG	down
CXCR4	down	gga-let-7c-3p_1ss12TC	up
CYB5A	up	gga-miR-146a-3p_R-1	down
CYB5A	up	gga-mir-147-p3_1	down
CYB5A	up	gga-mir-147-p5	down
CYB5A	up	gga-miR-214_L+1R-3_1ss19GA	down
CYB5A	up	gga-miR-29b-3p_R-1	down
CYB5A	up	gga-miR-29c-3p_R+1	down
CYB5A	up	gga-miR-29c-3p_R+1_1ss18GA	down
CYB5A	up	hsa-miR-3120-3p_R+1	down
CYB5A	up	ssa-miR-29b-3p_R-1_2ss20TC21TC	down
CYB5R2	up	ola-miR-199a-3p_R+1_1ss22AG	down
CYP2H1	down	cgr-miR-326	up
CYP2H1	down	gga-let-7c-3p_1ss12TC	up
CYP2H1	down	gga-miR-1668-3p	up
CYP2H1	down	gga-miR-20b-3p_R-1	up
CYP2H1	down	gga-miR-456-3p_1ss22AT	up
CYP2H1	down	gga-miR-456-3p_R-1	up
CYP2H1	down	oan-miR-363-3p_R+1	up
CYP2H1	down	pma-miR-456_R+1_1ss22AC	up

CYP7A1	up	gga-miR-1662_R-3	down
CYP7A1	up	tgu-miR-1662_L+1_1ss23TA	down
CYR61	down	aca-miR-18a-5p	up
CYR61	down	gga-miR-20b-3p_R-1	up
CYR61	down	gga-miR-216b	up
CYR61	down	gga-miR-30c-1-3p_R+1	up
DBNDD2	up	gga-miR-122-5p	down
DBNDD2	up	gga-miR-223_1ss15AG	down
DBNDD2	up	gga-miR-223_1ss19CT	down
DBNDD2	up	gga-miR-223_R+1	down
DCLRE1A	up	gga-let-7b_L-1R-1	down
DCLRE1A	up	gga-mir-147-p3_1	down
DCLRE1A	up	gga-mir-147-p5	down
DCLRE1A	up	gga-miR-196-5p_R+2	down
DCLRE1A	up	gga-miR-21-5p	down
DCLRE1A	up	ola-miR-199a-3p_R+1_1ss22AG	down
DCTD	up	xtr-miR-429_R+2	down
DDT	up	gga-miR-142-5p_L-2R+1	down
DDT	up	gga-miR-214_L+1R-3_1ss19GA	down
DDX50	up	gga-let-7j-3p_L-1R+2	down
DDX50	up	gga-miR-1329-5p	down
DDX50	up	xtr-miR-429_R+2	down
DEF6	down	gga-miR-107-3p_R-1	up
DEF6	down	gga-miR-1754-5p	up
DEF6	down	gga-miR-6604-5p_1ss7GA	up
DEGS1	down	aca-miR-138-2-3p_L-2R+3	up

DEGS1	down	gga-miR-107-5p_R+1	up
DEGS1	down	gga-miR-1668-3p	up
DEGS1	down	gga-miR-383-3p	up
DENND5B	up	gga-miR-146a-3p_R-1	down
DEPDC1B	up	gga-miR-214_L+1R-3_1ss19GA	down
DEPDC1B	up	gga-miR-2954_R+2	down
DEPDC1B	up	gga-miR-29b-3p_R-1	down
DEPDC1B	up	gga-miR-29c-3p_R+1	down
DEPDC1B	up	gga-miR-29c-3p_R+1_1ss18GA	down
DEPDC1B	up	hsa-miR-3120-3p_R+1	down
DEPDC1B	up	oan-let-7f-1-3p_R+2_1ss8AG	down
DEPDC1B	up	ssa-miR-29b-3p_R-1_2ss20TC21TC	down
DEPDC1B	up	xtr-miR-429_R+2	down
DHCR24	down	aca-miR-18a-3p_L+1R-1_1ss12GA	up
DHCR24	down	gga-let-7c-3p_1ss12TC	up
DHCR24	down	gga-miR-130b-5p	up
DHCR24	down	gga-miR-130b-5p_R-1	up
DHCR24	down	gga-miR-130b-5p_R-3	up
DHCR24	down	gga-miR-1467-3p_L+1	up
DHCR24	down	gga-miR-16-1-3p_R-1	up
DHCR24	down	gga-miR-1759-3p_L-3	up
DHCR24	down	gga-miR-1783_R+1	up
DHCR24	down	gga-miR-181b-1-3p_L+2R-2	up
DHCR24	down	gga-miR-202-5p_L-1	up
DHCR24	down	gga-miR-6710-3p_R+1	up
DHCR24	down	hsa-miR-181b-3p_L+1R-1	up

DHCR24	down	hsa-miR-4443_R+1	up
DHRS7	up	gga-miR-214_L+1R-3_1ss19GA	down
DHTKD1	up	odi-miR-7_R+2	down
DMXL1	down	gga-miR-1559-3p	up
DNA2	up	gga-miR-29b-3p_R-1	down
DNA2	up	gga-miR-29c-3p_R+1	down
DNA2	up	gga-miR-29c-3p_R+1_1ss18GA	down
DNA2	up	ssa-miR-29b-3p_R-1_2ss20TC21TC	down
DNAJA4	down	gga-miR-137-3p_L+1	up
DNAJA4	down	gga-miR-1559-3p	up
DNAJA4	down	gga-miR-1783_R+1	up
DNAJA4	down	gga-miR-365-2-5p_L-1R+1	up
DNAJA4	down	gga-miR-460a-5p	up
DNAJC12	down	aca-miR-181a_R+3	up
DNAJC12	down	gga-miR-106-3p_R+1	up
DNAJC12	down	gga-miR-144-3p_L-1R-2	up
DNAJC12	down	gga-miR-1559-3p	up
DNAJC12	down	gga-miR-1602	up
DNAJC12	down	gga-miR-1796	up
DNAJC12	down	gga-miR-181a-5p	up
DNAJC12	down	gga-miR-181b-5p_R+1	up
DNAJC12	down	gga-miR-429-3p	up
DNAJC12	down	ggo-miR-342_R+1	up
DNAJC12	down	hsa-miR-143-5p	up
DNAJC12	down	pma-miR-181a-5p_R+3	up
DOCK4	down	gga-miR-20b-5p	up

DPT	down	gga-miR-1467-3p_L+1	up
DPT	down	gga-miR-1699_L-1R-2	up
DPT	down	gga-miR-1788-3p_R+1	up
DPT	down	gga-miR-222a	up
DPT	down	hsa-miR-4443_R+1	up
DPT	down	PC-3p-68190_58	up
DTL	up	rno-miR-122-5p_L+1R+1_1	down
DTL	up	rno-miR-122-5p_L+1R+1_2	down
DTL	up	xtr-miR-429_R+2	down
DTX3L	down	gga-miR-1559-3p	up
DTX3L	down	gga-miR-1796	up
DTX3L	down	gga-miR-20b-5p	up
DTX3L	down	gga-miR-460a-5p	up
DTX3L	down	mmu-miR-8094_L+1R+3_1ss3AG	up
DUSP10	down	aca-miR-18a-3p_L+1R-1_1ss12GA	up
DUSP10	down	gga-miR-1783_R+1	up
DUSP10	down	gga-miR-1796	up
DUSP10	down	gga-miR-205a_R+1	up
DUSP10	down	gga-miR-205b	up
DUSP10	down	gga-miR-6548-5p	up
DUSP10	down	oan-miR-363-3p_R+1	up
DUSP10	down	tni-miR-205_R+2	up
ELL	down	gga-miR-144-3p_L-1R-2	up
ELL	down	gga-miR-1555-5p	up
ELL	down	gga-miR-92-5p	up
ELL	down	ggo-miR-342_R+1	up

ELL	down	mmu-mir-5098-p5	up
ELL	down	mmu-miR-8094_L+1R+3_1ss3AG	up
EME1	up	odi-miR-7_R+2	down
ENPEP	down	aca-miR-18a-5p	up
ENPEP	down	gga-miR-137-3p_L+1	up
ENTPD2	down	gga-miR-16c-5p	up
ENTPD2	down	gga-miR-1788-5p	up
ENTPD2	down	gga-miR-3529	up
ENTPD2	down	gga-miR-6604-5p_1ss7GA	up
EPHB2	up	gga-miR-22-3p	down
EREG	down	aca-miR-363-5p	up
EREG	down	gga-miR-130b-5p	up
EREG	down	gga-miR-130b-5p_R-1	up
EREG	down	gga-miR-130b-5p_R-3	up
EREG	down	gga-miR-1663-5p_R+2	up
EREG	down	gga-miR-1720-5p	up
EVL	down	gga-miR-15b-5p_R-1	up
EVL	down	gga-miR-16c-5p	up
EVL	down	gga-miR-17-3p_L-1R+3	up
EVL	down	gga-miR-1788-3p_R+1	up
EVL	down	hsa-miR-143-5p	up
F2RL1	down	gga-miR-107-3p_R-1	up
F2RL1	down	gga-miR-144-3p_L-1R-2	up
F2RL1	down	gga-miR-1559-3p	up
F2RL1	down	gga-miR-17-3p_L-1R+3	up
F2RL1	down	gga-miR-1747-3p	up

F2RL1	down	gga-miR-1783_R+1	up
F2RL1	down	gga-miR-181b-1-3p_L+1R-1	up
F2RL1	down	gga-miR-216b	up
F2RL1	down	gga-miR-6548-5p	up
F2RL1	down	gga-miR-6710-3p_R+1	up
FABP3	down	gga-miR-1716_L-2	up
FABP5	down	gga-miR-1559-3p	up
FABP5	down	gga-miR-16-1-3p_R-1	up
FABP5	down	gga-miR-20b-5p	up
FABP5	down	gga-miR-456-3p_1ss22AT	up
FABP5	down	gga-miR-456-3p_R-1	up
FABP5	down	oan-miR-363-3p_R+1	up
FABP5	down	pma-miR-456_R+1_1ss22AC	up
FABP7	up	hsa-miR-3120-3p_R+1	down
FABP7	up	oan-let-7f-1-3p_R+2_1ss8AG	down
FADS2	down	cgr-miR-151-3p	up
FADS2	down	gga-miR-1467-3p_L+1	up
FAM126A	down	gga-miR-106-3p_R+1	up
FAM126A	down	gga-miR-1552-3p_R+1	up
FAM126A	down	gga-miR-17-3p_L-1R+3	up
FAM126A	down	gga-miR-2131-3p_R+1	up
FAM126A	down	mmu-miR-8094_L+1R+3_1ss3AG	up
FAM129A	down	gga-miR-1699_L-1R-2	up
FAM129A	down	gga-miR-1731-5p	up
FAM129A	down	gga-miR-1788-3p_R+1	up
FAM129A	down	gga-miR-1788-5p	up

FAM129A	down	gga-miR-429-3p	up
FAM18B1	down	gga-miR-106-3p_R+1	up
FAM18B1	down	gga-miR-144-3p_L-1R-2	up
FAM18B1	down	gga-miR-1467-3p_L+1	up
FAM18B1	down	gga-miR-1788-3p_R+1	up
FAM18B1	down	gga-miR-1796	up
FAM18B1	down	gga-miR-456-3p_1ss22AT	up
FAM18B1	down	gga-miR-456-3p_R-1	up
FAM18B1	down	ggo-miR-342_R+1	up
FAM18B1	down	oha-miR-7-1-3p_1ss10GA	up
FAM18B1	down	pma-miR-456_R+1_1ss22AC	up
FAM195A	up	gga-miR-1329-5p	down
FANCL	up	xtr-miR-429_R+2	down
FBXO33	down	gga-miR-383-3p	up
FBXO33	down	gga-miR-429-3p	up
FBXO9	down	aca-miR-18a-3p_L+1R-1_1ss12GA	up
FBXO9	down	cgr-miR-500	up
FBXO9	down	gga-miR-107-3p_R-1	up
FBXO9	down	gga-miR-130b-5p	up
FBXO9	down	gga-miR-130b-5p_R-1	up
FBXO9	down	gga-miR-130b-5p_R-3	up
FBXO9	down	gga-miR-1451-3p_R-1	up
FBXO9	down	gga-miR-15b-5p_R-1	up
FBXO9	down	gga-miR-16c-5p	up
FBXO9	down	gga-miR-1759-3p_L-3	up
FBXO9	down	gga-miR-202-5p_L-1	up

FBXO9	down	gga-miR-3607-5p_R+1	up
FBXO9	down	gga-miR-383-3p	up
FBXO9	down	gga-miR-460a-5p	up
FBXO9	down	mmu-miR-500-3p_R-1	up
FDFT1	down	aca-miR-18a-3p_L+1R-1_1ss12GA	up
FDFT1	down	gga-miR-16c-5p	up
FDFT1	down	gga-miR-365-2-5p_L-1R+1	up
FDFT1	down	gga-miR-6548-5p	up
FEN1	up	aca-miR-150-5p_1ss12TC	down
FEN1	up	odi-miR-7_R+2	down
FEN1	up	ola-miR-126-5p_R+1_1ss1CN	down
FGL2	down	gga-miR-130b-3p	up
FGL2	down	gga-miR-137-3p_L+1	up
FGL2	down	gga-miR-1699_L-1R-2	up
FGL2	down	gga-miR-20b-3p_R-1	up
FGL2	down	gga-miR-429-3p	up
FGL2	down	gga-miR-454-3p	up
FGL2	down	gga-miR-6710-3p_R+1	up
FGL2	down	oan-miR-363-3p_R+1	up
FRYL	down	aca-miR-18a-5p	up
FRYL	down	mmu-mir-5098-p5	up
FRZB	down	aca-miR-138-2-3p_L-2R+3	up
FRZB	down	gga-miR-130b-3p	up
FRZB	down	gga-miR-1699_L-1R-2	up
FRZB	down	gga-miR-454-3p	up
FSHR	down	gga-miR-106-3p_R+1	up

FSHR	down	gga-miR-455-5p	up
FXR1	down	cgr-miR-151-3p	up
FXR1	down	gga-miR-130b-3p	up
FXR1	down	gga-miR-1559-3p	up
FXR1	down	gga-miR-30a-3p_1ss18GA	up
FXR1	down	gga-miR-30a-3p_2ss10GA20AT	up
FXR1	down	gga-miR-30e-3p_L+1	up
FXR1	down	gga-miR-3607-5p_R+1	up
FXR1	down	gga-miR-429-3p	up
FXR1	down	gga-miR-454-3p	up
FXR1	down	gga-miR-460a-5p	up
FXR1	down	oan-miR-363-3p_R+1	up
GABBR2	down	gga-miR-144-3p_L-1R-2	up
GABBR2	down	gga-miR-30a-3p_1ss18GA	up
GABBR2	down	gga-miR-30a-3p_2ss10GA20AT	up
GABBR2	down	gga-miR-30e-3p_L+1	up
GABBR2	down	ggo-miR-342_R+1	up
GAL7	down	aca-miR-129b-5p_R+1_1ss20TG	up
GAL8	down	gga-miR-1663-5p_R+2	up
GAL8	down	gga-miR-17-3p_L-1R+3	up
GAL8	down	gga-miR-3607-5p_R+1	up
GAL9	down	gga-miR-1602	up
GANC	up	gga-miR-142-3p	down
GANC	up	gga-miR-1784-5p	down
GANC	up	gga-miR-29a-5p_L-1R-2	down
GAS6	down	aca-miR-181a_R+3	up

GAS6	down	gga-miR-181a-5p	up
GAS6	down	gga-miR-181b-5p_R+1	up
GAS6	down	pma-miR-181a-5p_R+3	up
GATM	up	gga-let-7a-5p	down
GATM	up	gga-let-7a-5p_2ss12GT18TC	down
GATM	up	gga-let-7b_R+2	down
GATM	up	gga-let-7c-5p_1ss17AG	down
GATM	up	gga-let-7g-5p_R+1_2ss14TG18CA	down
GATM	up	gga-let-7k-5p_R+1_1ss16AT	down
GATM	up	gga-miR-22-3p	down
GATM	up	mmu-let-7j_1ss8TG	down
GBGT1	down	aca-miR-363-5p	up
GBGT1	down	cgr-miR-326	up
GBGT1	down	gga-miR-1602	up
GBGT1	down	gga-miR-1754-5p	up
GBGT1	down	gga-miR-181b-1-3p_L+2R-2	up
GBGT1	down	gga-miR-383-3p	up
GBGT1	down	gga-miR-455-5p	up
GBGT1	down	gga-miR-489-3p_L-2R+1	up
GBGT1	down	gga-miR-6548-5p	up
GBGT1	down	gga-miR-6604-5p_1ss7GA	up
GBGT1	down	ggo-miR-342_R+1	up
GBGT1	down	hsa-miR-181b-3p_L+1R-1	up
GFPT2	down	gga-miR-365-2-5p_L-1R+1	up
GFRA2	up	cin-miR-33_R+4	down
GFRA2	up	gga-miR-33-5p	down

GLCCI1	down	gga-miR-6710-3p_R+1	up
GLDC	up	ola-miR-199a-3p_R+1_1ss22AG	down
GLDC	up	xtr-miR-429_R+2	down
GLIPR1L	down	gga-miR-1747-3p	up
GLIPR1L	down	gga-miR-181b-1-3p_L+2R-2	up
GLIPR1L	down	hsa-miR-181b-3p_L+1R-1	up
GLS	down	gga-miR-20b-3p_R-1	up
GNPAT	up	gga-miR-1329-5p	down
GOT1	up	dre-miR-24_R+2	down
GOT1	up	gga-miR-9-5p	down
GPC3	down	gga-miR-1788-5p	up
GPR174	down	aca-miR-18a-5p	up
GPR174	down	gga-miR-138-5p_R+7	up
GPR174	down	gga-miR-144-3p_L-1R-2	up
GPR174	down	gga-miR-1555-5p	up
GPR174	down	gga-miR-1602	up
GPR174	down	gga-miR-1788-3p_R+1	up
GPR174	down	gga-miR-6604-5p_1ss7GA	up
GRAMD3	down	gga-miR-106-3p_R+1	up
GRAMD3	down	PC-5p-479025_5	up
GSN	down	oan-miR-363-3p_R+1	up
GSTA	down	gga-miR-17-3p_L-1R+3	up
GSTA	down	gga-miR-20b-3p_R-1	up
GSTA	down	gga-miR-34a-3p_R-1	up
GSTA	down	gga-miR-6604-5p_1ss7GA	up
GSTA	down	mmu-miR-8094_L+1R+3_1ss3AG	up

GSTO1	down	gga-miR-489-3p_L-2R+1	up
GTF2B	down	gga-miR-1788-5p	up
GZMA	down	gga-miR-1668-3p	up
GZMA	down	mmu-miR-8094_L+1R+3_1ss3AG	up
H2AFZ	up	gga-mir-147-p3_2	down
HABP2	down	gga-miR-181b-1-3p_L+1R-1	up
HABP2	down	gga-miR-383-3p	up
HABP2	down	ggo-miR-342_R+1	up
HABP2	down	hsa-miR-4443_R+1	up
HABP2	down	tni-miR-205_R+2	up
HEXA	down	gga-miR-7460-3p	up
HEXA	down	ggo-miR-342_R+1	up
HHIPL1	down	gga-miR-1759-3p_L-3	up
HHIPL1	down	gga-miR-489-3p_L-2R+1	up
HIBADH	up	gga-miR-142-5p_L-2R+1	down
HIBADH	up	mmu-miR-3964_L+1_1ss6GA	down
HIGD1A	up	gga-miR-146a-3p_R-1	down
HIGD1A	up	gga-miR-200b-5p_L+2	down
HMGCL	down	gga-miR-1731-5p	up
HMGCL	down	gga-miR-1759-3p_L-3	up
HMGCL	down	gga-miR-1788-3p_R+1	up
HMGCL	down	gga-miR-6548-5p	up
HMOX1	down	aca-miR-129b-5p_R+1_1ss20TG	up
HMOX1	down	gga-miR-1668-3p	up
HOOK1	up	gga-miR-21-5p	down
HOOK1	up	gga-miR-9-5p	down

HOPX	down	aca-miR-181a_R+3	up
HOPX	down	gga-miR-181a-5p	up
HOPX	down	gga-miR-181b-5p_R+1	up
HOPX	down	gga-miR-489-3p_L-2R+1	up
HOPX	down	pma-miR-181a-5p_R+3	up
HSBP1L1	down	gga-miR-1759-3p_L-3	up
HSBP1L1	down	gga-miR-181b-1-3p_L+1R-1	up
HSBP1L1	down	gga-miR-30a-5p_R+2_1ss13CT	up
HSBP1L1	down	gga-miR-30b-5p	up
HSBP1L1	down	gga-miR-30c-5p	up
HSBP1L1	down	gga-miR-30d_R+2	up
HSBP1L1	down	gga-miR-30e-5p_R+5	up
HSBP1L1	down	tgu-miR-30a-5p_R+2	up
HSCB	up	gga-miR-2954_R+2	down
HSD17B7	down	gga-miR-107-5p_R+1	up
HSD17B7	down	gga-miR-1467-3p_L+1	up
HSD17B7	down	gga-miR-181b-1-3p_L+1R-1	up
HSD17B7	down	gga-miR-7460-3p	up
HSD17B7	down	tni-miR-205_R+2	up
HSPA9	up	aca-miR-150-5p_1ss12TC	down
HSPA9	up	cin-miR-33_R+4	down
HSPA9	up	dre-miR-24_R+2	down
HSPA9	up	gga-miR-140-3p_L+1R+1_1ss22CA	down
HSPA9	up	gga-miR-1784-5p	down
HSPA9	up	gga-miR-33-5p	down
HSPA9	up	sha-miR-140_R+2	down

IFI27L2	down	gga-miR-106-3p_R+1	up
IFNAR1	down	gga-miR-106-3p_R+1	up
IFNAR1	down	gga-miR-181b-1-3p_L+2R-2	up
IFNAR1	down	gga-miR-20b-5p	up
IFNAR1	down	hsa-miR-181b-3p_L+1R-1	up
IFNGR1	down	aca-miR-181a_R+3	up
IFNGR1	down	chi-miR-361-5p	up
IFNGR1	down	efu-miR-361	up
IFNGR1	down	gga-miR-1559-3p	up
IFNGR1	down	gga-miR-181a-5p	up
IFNGR1	down	gga-miR-181b-5p_R+1	up
IFNGR1	down	gga-miR-216b	up
IFNGR1	down	pma-miR-181a-5p_R+3	up
IFRD1	up	gga-let-7j-3p_L-1R+2	down
IFRD1	up	gga-miR-1329-5p	down
IFRD1	up	gga-miR-200b-5p_L+2	down
IFRD1	up	oan-let-7f-1-3p_R+2_1ss8AG	down
IGF2BP3	up	gga-let-7a-5p	down
IGF2BP3	up	gga-let-7a-5p_2ss12GT18TC	down
IGF2BP3	up	gga-let-7b_L-1R-1	down
IGF2BP3	up	gga-let-7b_R+2	down
IGF2BP3	up	gga-let-7c-5p_1ss17AG	down
IGF2BP3	up	gga-let-7g-5p_R+1_2ss14TG18CA	down
IGF2BP3	up	gga-let-7k-5p_R+1_1ss16AT	down
IGF2BP3	up	gga-miR-142-5p_L-2R+1	down
IGF2BP3	up	gga-miR-1729-5p_R-1	down

IGF2BP3	up	gga-miR-196-5p_R+2	down
IGF2BP3	up	gga-miR-29a-5p_L-1R-2	down
IGF2BP3	up	gga-miR-9-5p	down
IGF2BP3	up	mmu-let-7j_1ss8TG	down
IGF2BP3	up	mmu-miR-3964_L+1_1ss6GA	down
IGF2BP3	up	ola-miR-199a-3p_R+1_1ss22AG	down
IGF2BP3	up	tgu-miR-1784-3p_1ss11GA	down
IL16	down	mmu-mir-5098-p5	up
IL1RL1	down	gga-miR-15b-5p_R-1	up
IL1RL1	down	gga-miR-16c-5p	up
IL1RL1	down	gga-miR-1788-3p_R+1	up
IL21R	down	gga-miR-6604-5p_1ss7GA	up
INCENP	up	aca-miR-150-5p_1ss12TC	down
INCENP	up	gga-miR-140-3p_L+1R+1_1ss22CA	down
INCENP	up	gga-miR-142-3p	down
INCENP	up	gga-miR-146b-3p_L+2	down
INCENP	up	gga-miR-21-5p	down
INCENP	up	gga-miR-222b-5p_R-4	down
INCENP	up	gga-miR-22-3p	down
INCENP	up	hsa-miR-21-5p_R+2_1ss1TN	down
INCENP	up	hsa-miR-3120-3p_R+1	down
INCENP	up	ola-miR-199a-3p_R+1_1ss22AG	down
INCENP	up	sha-miR-140_R+2	down
INCENP	up	tgu-miR-1662_L+1_1ss23TA	down
INCENP	up	xtr-miR-429_R+2	down
IRG1	down	aca-miR-138-2-3p_L-2R+3	up

IRG1	down	gga-miR-107-3p_R-1	up
IRG1	down	gga-miR-15b-5p_R-1	up
IRG1	down	gga-miR-16c-5p	up
IRG1	down	gga-miR-202-5p_L-1	up
IRG1	down	gga-miR-30a-3p_1ss18GA	up
IRG1	down	gga-miR-30e-3p_L+1	up
IRG1	down	gga-miR-34a-3p_R-1	up
IRG1	down	gga-miR-3607-5p_R+1	up
IRG1	down	gga-miR-429-5p_L-1	up
IRG1	down	gga-miR-6604-5p_1ss7GA	up
IRG1	down	gga-miR-92-5p	up
IRG1	down	hsa-miR-143-5p	up
IRG1	down	mmu-mir-5098-p5	up
IRG1	down	mmu-miR-8094_L+1R+3_1ss3AG	up
ISCA1	down	chi-miR-361-5p	up
ISCA1	down	efu-miR-361	up
ISCA1	down	gga-miR-144-3p_L-1R-2	up
ISCA1	down	gga-miR-1552-3p_R+1	up
ISCA1	down	gga-miR-202-5p_L-1	up
ISCA1	down	gga-miR-2131-3p_R+1	up
ISCA1	down	gga-miR-429-5p_L-1	up
ISCA1	down	gga-miR-455-5p	up
ISCA1	down	gga-miR-6548-5p	up
ISCA1	down	oan-miR-363-3p_R+1	up
ISCA1	down	PC-3p-68190_58	up
ISM1	up	gga-miR-9-5p	down

ITGA8	down	aca-miR-129b-5p_R+1_1ss20TG	up
ITGA8	down	cgr-miR-151-3p	up
ITGA8	down	gga-miR-137-3p_L+1	up
ITGA8	down	gga-miR-15b-5p_R-1	up
ITGA8	down	gga-miR-16c-5p	up
ITGA8	down	gga-miR-30a-3p_2ss10GA20AT	up
ITGB2	down	csa-let-7d_1ss10TC	up
ITGB2	down	gga-miR-1699_L-1R-2	up
ITGB2	down	gga-miR-30c-1-3p_R+1	up
ITGB2	down	gga-miR-365-2-5p_L-1R+1	up
IYD	up	gga-miR-155_R+1	down
JAG1	down	cgr-miR-500	up
JAG1	down	gga-miR-138-5p_R+7	up
JAG1	down	gga-miR-144-3p_L-1R-2	up
JAG1	down	gga-miR-16-1-3p_R-1	up
JAG1	down	gga-miR-1668-3p	up
JAG1	down	gga-miR-301a-5p_L+2	up
JAG1	down	mmu-miR-500-3p_R-1	up
JAG1	down	mmu-mir-5098-p5	up
JAK2	down	gga-miR-3607-5p_R+1	up
JAK2	down	gga-miR-383-3p	up
KAT2B	down	aca-miR-181a_R+3	up
KAT2B	down	gga-miR-181a-5p	up
KAT2B	down	gga-miR-181b-5p_R+1	up
KAT2B	down	gga-miR-20b-5p	up
KAT2B	down	gga-miR-456-3p_1ss22AT	up

KAT2B	down	gga-miR-456-3p_R-1	up
KAT2B	down	gga-miR-460a-5p	up
KAT2B	down	pma-miR-181a-5p_R+3	up
KAT2B	down	pma-miR-456_R+1_1ss22AC	up
KIAA1524	up	aca-miR-425-5p_R+4	down
KIAA1524	up	gga-miR-146b-3p_L+2	down
KIAA1524	up	hsa-miR-425-5p_R+2_1ss20TC	down
KIF11	up	dre-miR-24_R+2	down
KIF11	up	gga-let-7j-3p_L-1R+2	down
KIF11	up	gga-miR-1329-5p	down
KIF11	up	gga-miR-146c-5p_4ss18GA20AG22TG23GT	down
KIF11	up	gga-miR-222b-5p_R-4	down
KIF20A	up	cin-miR-33_R+4	down
KIF20A	up	gga-miR-142-3p	down
KIF20A	up	gga-miR-146c-5p_4ss18GA20AG22TG23GT	down
KIF20A	up	gga-miR-200b-5p_L+2	down
KIF20A	up	gga-miR-222b-5p_R-4	down
KIF20A	up	gga-miR-22-3p	down
KIF20A	up	gga-miR-33-5p	down
KIF20A	up	gga-miR-9-5p	down
KIF20A	up	hsa-miR-21-5p_R+2_1ss1TN	down
KIF20A	up	tni-miR-455_L+1_2ss22CT23GT	down
KIF23	up	gga-miR-29a-5p_L-1R-2	down
KIF4A	up	gga-miR-29b-3p_R-1	down
KIF4A	up	gga-miR-29c-3p_R+1	down
KIF4A	up	gga-miR-29c-3p_R+1_1ss18GA	down

KIF4A	up	odi-miR-7_R+2	down
KIF4A	up	ssa-miR-29b-3p_R-1_2ss20TC21TC	down
KLF2	down	gga-miR-107-5p_R+1	up
KLHL6	down	gga-miR-383-3p	up
KLHL6	down	mmu-miR-8094_L+1R+3_1ss3AG	up
KPNA2	up	gga-let-7b_L-1R-1	down
KPNA2	up	gga-miR-196-5p_R+2	down
KRTCAP3	up	gga-miR-29b-3p_R-1	down
KRTCAP3	up	gga-miR-29c-3p_R+1	down
KRTCAP3	up	gga-miR-29c-3p_R+1_1ss18GA	down
KRTCAP3	up	ssa-miR-29b-3p_R-1_2ss20TC21TC	down
LCP1	down	gga-miR-130b-5p	up
LCP1	down	gga-miR-130b-5p_R-1	up
LCP1	down	gga-miR-130b-5p_R-3	up
LCP1	down	gga-miR-137-3p_L+1	up
LCP1	down	gga-miR-1747-3p	up
LCP1	down	gga-miR-383-3p	up
LCP1	down	gga-miR-429-5p_L-1	up
LCP1	down	gga-miR-454-3p	up
LCP1	down	gga-miR-460a-5p	up
LDHA	down	gga-let-7c-3p_1ss12TC	up
LDHA	down	gga-miR-106-3p_R+1	up
LDHA	down	gga-miR-130b-3p	up
LDHA	down	gga-miR-1467-3p_L+1	up
LDHA	down	gga-miR-17-3p_L-1R+3	up
LDHA	down	gga-miR-20b-5p	up

LDHA	down	gga-miR-454-3p	up
LIMD2	down	gga-miR-1754-5p	up
LIMD2	down	gga-miR-1783_R+1	up
LIMD2	down	gga-miR-301a-5p_L+2	up
LIMD2	down	hsa-miR-4286_R+1	up
LIMK1	up	gga-miR-147_R+1	down
LIPG	down	aca-miR-129b-5p_R+1_1ss20TG	up
LITAF	down	gga-miR-205a_R+1	up
LITAF	down	gga-miR-205b	up
LITAF	down	tni-miR-205_R+2	up
LONRF1	down	gga-miR-130b-3p	up
LONRF1	down	gga-miR-1788-3p_R+1	up
LONRF1	down	gga-miR-454-3p	up
LONRF1	down	gga-miR-460a-5p	up
LPCAT2	down	aca-miR-181a_R+3	up
LPCAT2	down	gga-miR-181a-5p	up
LPCAT2	down	gga-miR-181b-1-3p_L+1R-1	up
LPCAT2	down	gga-miR-181b-5p_R+1	up
LPCAT2	down	gga-miR-20b-5p	up
LPCAT2	down	gga-miR-2131-3p_R+1	up
LPCAT2	down	gga-miR-3607-5p_R+1	up
LPCAT2	down	gga-miR-454-3p	up
LPCAT2	down	gga-miR-6604-5p_1ss7GA	up
LPCAT2	down	gga-miR-92-5p	up
LPCAT2	down	mmu-miR-8094_L+1R+3_1ss3AG	up
LPCAT2	down	oan-miR-19b-1-5p_R+4	up

LPCAT2	down	pma-miR-181a-5p_R+3	up
LPCAT3	up	gga-miR-122-5p	down
LPCAT3	up	hsa-miR-3120-3p_R+1	down
LPCAT3	up	rno-miR-122-5p_L+1R+1_1	down
LPCAT3	up	rno-miR-122-5p_L+1R+1_2	down
LPL	up	gga-miR-2954_R+2	down
LPL	up	gga-miR-458a-3p	down
LSP1	down	gga-miR-107-3p_R-1	up
LY75	down	gga-miR-1754-5p	up
LYG2	down	gga-miR-1788-3p_R+1	up
LYG2	down	mmu-mir-5098-p5	up
LYN	down	gga-miR-106-3p_R+1	up
LYN	down	gga-miR-130b-5p	up
LYN	down	gga-miR-130b-5p_R-1	up
LYN	down	gga-miR-130b-5p_R-3	up
LYN	down	gga-miR-1552-3p_R+1	up
LYN	down	gga-miR-15b-5p_R-1	up
LYN	down	gga-miR-16-1-3p_R-1	up
LYN	down	gga-miR-16c-5p	up
LYN	down	gga-miR-181a-3p	up
LYN	down	gga-miR-181b-1-3p_L+1R-1	up
LYN	down	gga-miR-460a-5p	up
LYVE1	down	mmu-miR-8094_L+1R+3_1ss3AG	up
MAD2L1	up	gga-let-7j-3p_L-1R+2	down
MAD2L1	up	oan-let-7f-1-3p_R+2_1ss8AG	down
MAD2L1	up	xtr-miR-429_R+2	down

MAN2A2	up	gga-miR-214_L+1R-3_1ss19GA	down
MAOA	down	aca-miR-363-5p	up
MAOA	down	gga-miR-15b-5p_R-1	up
MAOA	down	gga-miR-16c-5p	up
MAOA	down	gga-miR-181b-1-3p_L+1R-1	up
MAOA	down	gga-miR-20b-3p_R-1	up
MAOA	down	gga-miR-216b	up
MAOA	down	oan-miR-19b-1-5p_R+4	up
MAOA	down	PC-5p-479025_5	up
MAP2K6	up	gga-miR-29c-3p_L+1R+1_1ss19GA	down
MARCKSL1	down	gga-miR-1716_L-2	up
MARCKSL1	down	gga-miR-1720-5p	up
MARCKSL1	down	gga-miR-1783_R+1	up
MARCKSL1	down	mmu-mir-5098-p5	up
MBLAC2	down	aca-miR-129b-5p_R+1_1ss20TG	up
MBLAC2	down	aca-miR-181a_R+3	up
MBLAC2	down	aca-miR-18a-3p_L+1R-1_1ss12GA	up
MBLAC2	down	cgr-miR-151-3p	up
MBLAC2	down	cgr-miR-500	up
MBLAC2	down	gga-miR-130b-5p	up
MBLAC2	down	gga-miR-130b-5p_R-1	up
MBLAC2	down	gga-miR-130b-5p_R-3	up
MBLAC2	down	gga-miR-15b-5p_R-1	up
MBLAC2	down	gga-miR-1668-3p	up
MBLAC2	down	gga-miR-16c-5p	up
MBLAC2	down	gga-miR-17-3p_L-1R+3	up

MBLAC2	down	gga-miR-1783_R+1	up
MBLAC2	down	gga-miR-1788-5p	up
MBLAC2	down	gga-miR-1796	up
MBLAC2	down	gga-miR-181a-5p	up
MBLAC2	down	gga-miR-181b-5p_R+1	up
MBLAC2	down	gga-miR-222a	up
MBLAC2	down	gga-miR-490-3p_R+1	up
MBLAC2	down	hsa-miR-4443_R+1	up
MBLAC2	down	mmu-miR-500-3p_R-1	up
MBLAC2	down	mmu-mir-5098-p5	up
MBLAC2	down	pma-miR-181a-5p_R+3	up
MCM2	up	dre-miR-24_R+2	down
MCM2	up	gga-miR-125b-5p	down
MCM2	up	gga-miR-1729-5p_R-1	down
MCM2	up	gga-miR-222b-5p_R-4	down
MCM2	up	hsa-miR-3120-3p_R+1	down
MCM2	up	sha-miR-125a_R+2	down
MCM2	up	ssa-miR-125a-5p_R+2	down
MCM3	up	gga-miR-9-5p	down
MCM4	up	hsa-miR-3120-3p_R+1	down
MCM6	up	gga-miR-222b-5p_R-4	down
MCM6	up	hsa-miR-3120-3p_R+1	down
MCM6	up	oan-let-7f-1-3p_R+2_1ss8AG	down
MCM6	up	odi-miR-7_R+2	down
MCM6	up	rno-miR-122-5p_L+1R+1_1	down
MCM6	up	rno-miR-122-5p_L+1R+1_2	down

MCM6	up	xtr-miR-429_R+2	down
MICAL1	down	gga-miR-107-3p_R-1	up
MICAL1	down	gga-miR-1559-5p	up
MICAL1	down	gga-miR-17-3p_L-1R+3	up
MICAL1	down	gga-miR-455-5p	up
MICALL1	down	gga-miR-1759-3p_L-3	up
MICALL1	down	gga-miR-1788-3p_R+1	up
MMP11	up	gga-let-7f-3p_1ss22CT	down
MMP11	up	gga-miR-125b-5p	down
MMP11	up	gga-miR-22-3p	down
MMP11	up	gga-miR-29b-3p_R-1	down
MMP11	up	gga-miR-29c-3p_R+1	down
MMP11	up	gga-miR-29c-3p_R+1_1ss18GA	down
MMP11	up	sha-miR-125a_R+2	down
MMP11	up	ssa-miR-125a-5p_R+2	down
MMP11	up	ssa-miR-29b-3p_R-1_2ss20TC21TC	down
MORC3	down	aca-miR-129b-5p_R+1_1ss20TG	up
MORC3	down	aca-miR-18a-3p_L+1R-1_1ss12GA	up
MORC3	down	gga-miR-125b-3p_R-1_1ss21CT	up
MORC3	down	gga-miR-130b-3p	up
MORC3	down	gga-miR-1602	up
MORC3	down	gga-miR-16-1-3p_R-1	up
MORC3	down	gga-miR-1668-3p	up
MORC3	down	gga-miR-1759-3p_L-3	up
MORC3	down	gga-miR-34a-3p_R-1	up
MORC3	down	gga-miR-3523	up

MORC3	down	gga-miR-383-3p	up
MORC3	down	gga-miR-429-5p_L-1	up
MORC3	down	gga-miR-454-3p	up
MORC3	down	gga-miR-6710-3p_R+1	up
MORC3	down	gga-miR-7460-3p	up
MOSPD1	down	aca-miR-181a_R+3	up
MOSPD1	down	gga-miR-181a-5p	up
MOSPD1	down	gga-miR-181b-1-3p_L+1R-1	up
MOSPD1	down	gga-miR-181b-5p_R+1	up
MOSPD1	down	gga-miR-20b-5p	up
MOSPD1	down	gga-miR-3607-5p_R+1	up
MOSPD1	down	PC-3p-68190_58	up
MOSPD1	down	pma-miR-181a-5p_R+3	up
MPP1	up	aca-miR-363-3p_R+1	down
MPZL3	down	gga-miR-130b-5p	up
MPZL3	down	gga-miR-130b-5p_R-1	up
MPZL3	down	gga-miR-130b-5p_R-3	up
MPZL3	down	gga-miR-16c-3p_L+1R-1	up
MPZL3	down	gga-miR-30a-3p_2ss10GA20AT	up
MPZL3	down	gga-miR-34a-3p_R-1	up
MRM1	up	gga-miR-29b-3p_R-1	down
MRM1	up	gga-miR-29c-3p_R+1	down
MRM1	up	gga-miR-29c-3p_R+1_1ss18GA	down
MRM1	up	ssa-miR-29b-3p_R-1_2ss20TC21TC	down
MSMO1	down	aca-miR-129b-5p_R+1_1ss20TG	up
MSMO1	down	gga-miR-106-3p_R+1	up

MSMO1	down	gga-miR-125b-3p_R-1_1ss21CT	up
MSMO1	down	gga-miR-130b-3p	up
MSMO1	down	gga-miR-16-1-3p_R-1	up
MSMO1	down	gga-miR-16c-3p_L+1R-1	up
MSMO1	down	gga-miR-216b	up
MSMO1	down	gga-miR-454-3p	up
MSMO1	down	oan-miR-363-3p_R+1	up
MTHFD2	up	aca-miR-150-5p_1ss12TC	down
MTHFD2	up	aca-miR-363-3p_R+1	down
MTHFD2	up	gga-miR-122-5p	down
MTHFD2	up	gga-miR-222b-5p_R-4	down
MYBBP1A	up	gga-miR-1662_R-3	down
MYBBP1A	up	gga-miR-1784-5p	down
MYH9	down	gga-miR-490-3p_R+1	up
MYH9	down	gga-miR-6604-5p_1ss7GA	up
MYO1F	down	gga-miR-106-3p_R+1	up
MYO1F	down	gga-miR-107-3p_R-1	up
MYO1F	down	gga-miR-15b-5p_R-1	up
MYO1F	down	gga-miR-1668-3p	up
MYO1F	down	gga-miR-16c-5p	up
MYO1F	down	gga-miR-6604-5p_1ss7GA	up
N4BP2L1	down	gga-miR-6604-5p_1ss7GA	up
NAPEPLD	down	aca-miR-363-5p	up
NAPEPLD	down	gga-miR-137-3p_L+1	up
NAPEPLD	down	gga-miR-144-3p_L-1R-2	up
NAPEPLD	down	gga-miR-16-1-3p_R-1	up

NAPEPLD	down	gga-miR-1720-5p	up
NAPEPLD	down	gga-miR-20b-5p	up
NAPEPLD	down	gga-miR-30a-5p_R+2_1ss13CT	up
NAPEPLD	down	gga-miR-30e-5p_R+5	up
NAPEPLD	down	gga-miR-490-3p_R+1	up
NAPEPLD	down	gga-miR-6548-5p	up
NAPEPLD	down	tgu-miR-30a-5p_R+2	up
NBL1	down	gga-miR-106-3p_R+1	up
NBL1	down	gga-miR-1602	up
NBL1	down	gga-mir-1609-p3	up
NBL1	down	gga-miR-1699_L-1R-2	up
NBL1	down	gga-miR-20b-5p	up
NBL1	down	gga-miR-6548-5p	up
NBL1	down	oan-miR-19b-1-5p_R+4	up
NCF4	down	cgr-miR-151-3p	up
NCF4	down	gga-let-7c-3p_1ss12TC	up
NCF4	down	gga-miR-17-3p_L-1R+3	up
NCF4	down	oan-miR-19b-1-5p_R+4	up
NCLN	up	dre-miR-24_R+2	down
NCLN	up	gga-miR-222b-5p_R-4	down
NDUFA10	up	dre-miR-24_R+2	down
NDUFA10	up	gga-miR-140-3p_L+1R+1_1ss22CA	down
NDUFA10	up	gga-miR-1784-5p	down
NDUFA10	up	hsa-miR-3120-3p_R+1	down
NDUFA10	up	sha-miR-140_R+2	down
NDUFA8	up	ola-miR-199a-3p_R+1_1ss22AG	down

NDUFB5	up	gga-miR-214_L+1R-3_1ss19GA	down
NDUFS1	up	gga-let-7f-3p_1ss22CT	down
NDUFS1	up	gga-miR-140-3p_L+1R+1_1ss22CA	down
NDUFS1	up	gga-miR-29a-5p_L-1R-2	down
NDUFS1	up	hsa-miR-3120-3p_R+1	down
NDUFS1	up	sha-miR-140_R+2	down
NDUFS3	up	gga-miR-140-3p_L+1R+1_1ss22CA	down
NDUFS3	up	sha-miR-140_R+2	down
NEK2	up	xtr-miR-429_R+2	down
NET1	down	aca-miR-363-5p	up
NET1	down	gga-let-7c-3p_1ss12TC	up
NET1	down	gga-miR-1552-3p_R+1	up
NET1	down	gga-miR-6548-5p	up
NET1	down	gga-miR-7460-3p	up
NFKBIE	down	gga-miR-456-3p_1ss22AT	up
NFKBIE	down	gga-miR-456-3p_R-1	up
NFKBIE	down	hsa-miR-143-5p	up
NFKBIE	down	pma-miR-456_R+1_1ss22AC	up
NFYC	up	gga-miR-458a-3p	down
NLN	up	gga-miR-2954_R+2	down
NPM3	up	gga-miR-146a-3p_R-1	down
NPM3	up	gga-miR-214_L+1R-3_1ss19GA	down
NTM	down	gga-miR-1759-3p_L-3	up
NUDT1	up	gga-miR-1784-5p	down
NUF2	up	gga-mir-147-p3_1	down
NUF2	up	gga-mir-147-p5	down

OAZ2	down	gga-miR-1788-5p	up
P4HA1	down	gga-miR-1747-3p	up
P4HA1	down	gga-miR-1796	up
P4HA1	down	gga-miR-20b-3p_R-1	up
PACSIN2	down	aca-miR-181a_R+3	up
PACSIN2	down	gga-let-7c-3p_1ss12TC	up
PACSIN2	down	gga-miR-16-1-3p_R-1	up
PACSIN2	down	gga-miR-181a-5p	up
PACSIN2	down	gga-miR-181b-5p_R+1	up
PACSIN2	down	gga-miR-20b-3p_R-1	up
PACSIN2	down	gga-miR-30a-3p_1ss18GA	up
PACSIN2	down	gga-miR-30a-3p_2ss10GA20AT	up
PACSIN2	down	gga-miR-30e-3p_L+1	up
PACSIN2	down	gga-miR-429-3p	up
PACSIN2	down	gga-miR-455-5p	up
PACSIN2	down	gga-miR-6548-5p	up
PACSIN2	down	pma-miR-181a-5p_R+3	up
PALMD	down	aca-miR-138-2-3p_L-2R+3	up
PANK1	down	gga-miR-107-3p_R-1	up
PANK1	down	gga-miR-144-3p_L-1R-2	up
PANK1	down	gga-miR-15b-5p_R-1	up
PANK1	down	gga-miR-16-1-3p_R-1	up
PANK1	down	gga-miR-16c-5p	up
PANK1	down	oha-miR-7-1-3p_1ss10GA	up
PARP8	down	aca-miR-129b-5p_R+1_1ss20TG	up
PARP8	down	PC-3p-68190_58	up

PCMTD1	down	chi-miR-361-5p	up
PCMTD1	down	efu-miR-361	up
PCMTD1	down	gga-miR-107-3p_R-1	up
PCMTD1	down	gga-miR-125b-3p_R-1_1ss21CT	up
PCMTD1	down	gga-miR-130b-5p	up
PCMTD1	down	gga-miR-130b-5p_R-1	up
PCMTD1	down	gga-miR-130b-5p_R-3	up
PCMTD1	down	gga-miR-1559-3p	up
PCMTD1	down	gga-miR-16-1-3p_R-1	up
PCMTD1	down	gga-miR-1783_R+1	up
PCMTD1	down	gga-miR-1796	up
PCMTD1	down	gga-miR-20b-5p	up
PCMTD1	down	gga-miR-2131-3p_R+1	up
PCMTD1	down	gga-miR-222a	up
PCMTD1	down	gga-miR-30a-3p_1ss18GA	up
PCMTD1	down	gga-miR-30a-3p_2ss10GA20AT	up
PCMTD1	down	gga-miR-30e-3p_L+1	up
PCMTD1	down	gga-miR-34a-3p_R-1	up
PCMTD1	down	gga-miR-429-3p	up
PCMTD1	down	gga-miR-6548-5p	up
PCMTD1	down	mmu-miR-8094_L+1R+3_1ss3AG	up
PCMTD1	down	oan-miR-19b-1-5p_R+4	up
PDHA1	up	hsa-miR-3120-3p_R+1	down
PDK4	down	cgr-miR-500	up
PDK4	down	mmu-miR-500-3p_R-1	up
PDLIM1	down	gga-miR-16c-5p	up

PDLIM1	down	gga-miR-301a-5p_L+2	up
PDLIM1	down	gga-miR-6548-5p	up
PDLIM1	down	mmu-miR-8094_L+1R+3_1ss3AG	up
PDLIM3	down	gga-let-7c-3p_1ss12TC	up
PDLIM3	down	gga-miR-107-5p_R+1	up
PDLIM3	down	gga-miR-2131-3p_R+1	up
PDLIM3	down	gga-miR-365-2-5p_L-1R+1	up
PDLIM5	down	aca-miR-129b-5p_R+1_1ss20TG	up
PDLIM5	down	aca-miR-18a-5p	up
PDLIM5	down	aca-miR-363-5p	up
PDLIM5	down	cgr-miR-500	up
PDLIM5	down	gga-miR-15b-5p_R-1	up
PDLIM5	down	gga-miR-16c-3p_L+1R-1	up
PDLIM5	down	gga-miR-16c-5p	up
PDLIM5	down	gga-miR-1747-3p	up
PDLIM5	down	gga-miR-1759-3p_L-3	up
PDLIM5	down	gga-miR-1783_R+1	up
PDLIM5	down	gga-miR-1796	up
PDLIM5	down	gga-miR-181b-1-3p_L+1R-1	up
PDLIM5	down	gga-miR-20b-5p	up
PDLIM5	down	gga-miR-30a-3p_1ss18GA	up
PDLIM5	down	gga-miR-30a-3p_2ss10GA20AT	up
PDLIM5	down	gga-miR-30e-3p_L+1	up
PDLIM5	down	gga-miR-365-2-5p_L-1R+1	up
PDLIM5	down	gga-miR-383-3p	up
PDLIM5	down	hsa-miR-143-5p	up

PDLIM5	down	mmu-miR-500-3p_R-1	up
PDLIM5	down	mmu-mir-5098-p5	up
PEX6	up	gga-miR-214_L+1R-3_1ss19GA	down
PFKL	up	gga-mir-147-p3_2	down
PIGR	down	gga-miR-1747-3p	up
PIGR	down	mmu-miR-8094_L+1R+3_1ss3AG	up
PIK3IP1	down	gga-miR-205b	up
PITRM1	up	gga-miR-222b-5p_R-4	down
PLAU	down	gga-miR-106-3p_R+1	up
PLAU	down	gga-miR-144-3p_L-1R-2	up
PLAU	down	gga-miR-1559-3p	up
PLAU	down	gga-miR-16-1-3p_R-1	up
PLAU	down	gga-miR-301a-5p_L+2	up
PLAU	down	gga-miR-30a-3p_1ss18GA	up
PLAU	down	gga-miR-30a-3p_2ss10GA20AT	up
PLAU	down	gga-miR-30e-3p_L+1	up
PLAU	down	gga-miR-34a-3p_R-1	up
PLAU	down	gga-miR-460a-5p	up
PLAU	down	hsa-miR-143-5p	up
PLCL2	down	gga-miR-130b-3p	up
PLCL2	down	gga-miR-202-5p_L-1	up
PLCL2	down	gga-miR-454-3p	up
PLEKHO1	down	gga-miR-144-3p_L-1R-2	up
PLEKHO1	down	gga-miR-1663-5p_R+2	up
PLEKHO1	down	gga-miR-1668-3p	up
PLEKHO1	down	gga-miR-3607-5p_R+1	up

PLEKHO1	down	hsa-miR-143-5p	up
PLK1	up	gga-miR-222b-5p_R-4	down
PLK1	up	gga-miR-29b-3p_R-1	down
PLK1	up	gga-miR-29c-3p_R+1_1ss18GA	down
PMPCA	up	gga-miR-214_L+1R-3_1ss19GA	down
PMPCA	up	gga-miR-222b-5p_R-4	down
PMPCA	up	gga-miR-29a-5p_L-1R-2	down
PNRC1	down	gga-miR-125b-3p_R-1_1ss21CT	up
PNRC1	down	gga-miR-130b-3p	up
PNRC1	down	gga-miR-34a-3p_R-1	up
PNRC1	down	gga-miR-454-3p	up
PNRC1	down	mmu-mir-5098-p5	up
POLA1	up	gga-miR-1784-5p	down
PPIC	up	aca-miR-363-3p_R+1	down
PPIC	up	gga-miR-122-5p	down
PPIC	up	gga-miR-146b-3p_L+2	down
PPRC1	up	dre-miR-24_R+2	down
PRDX1	up	cin-miR-33_R+4	down
PRDX1	up	gga-miR-33-5p	down
PRDX6	down	gga-miR-138-5p_R+7	up
PRDX6	down	gga-miR-144-3p_L-1R-2	up
PRDX6	down	gga-miR-1663-5p_R+2	up
PRDX6	down	mmu-miR-8094_L+1R+3_1ss3AG	up
PTPN2	down	gga-miR-138-5p_R+7	up
PTPN2	down	gga-miR-1552-3p_R+1	up
PTPN2	down	gga-miR-30a-5p_R+2_1ss13CT	up

PTPN2	down	gga-miR-30b-5p	up
PTPN2	down	gga-miR-30d_R+2	up
PTPN2	down	gga-miR-30e-5p_R+5	up
PTPN2	down	gga-miR-6548-5p	up
PTPN2	down	tgu-miR-30a-5p_R+2	up
PTPN6	down	gga-miR-1467-3p_L+1	up
PTPN6	down	gga-miR-15b-5p_R-1	up
PTPN6	down	gga-miR-1783_R+1	up
PTPN6	down	mmu-miR-8094_L+1R+3_1ss3AG	up
PTPN6	down	PC-5p-479025_5	up
PTPRF	up	dre-miR-24_R+2	down
PTPRF	up	gga-miR-2954_R+2	down
PWP1	up	aca-miR-150-5p_1ss12TC	down
PWP1	up	gga-miR-142-3p	down
PWP1	up	gga-miR-1784-5p	down
PYROXD1	up	aca-miR-363-3p_R+1	down
PYROXD1	up	gga-miR-1329-5p	down
PYROXD1	up	gga-miR-2954_R+2	down
PYROXD1	up	tgu-miR-1662_L+1_1ss23TA	down
RAB32	down	gga-miR-107-3p_R-1	up
RAB32	down	gga-miR-15b-5p_R-1	up
RAB32	down	gga-miR-16c-5p	up
RAC2	down	gga-miR-181b-1-3p_L+2R-2	up
RAC2	down	gga-miR-6548-5p	up
RAC2	down	hsa-miR-181b-3p_L+1R-1	up
RAD21	up	aca-miR-363-3p_R+1	down

RAD21	up	gga-let-7f-3p_1ss22CT	down
RAD21	up	gga-miR-122-5p	down
RAD21	up	gga-miR-146b-3p_L+2	down
RAD21	up	gga-miR-1784-5p	down
RAD21	up	gga-miR-21-5p	down
RAD21	up	gga-miR-22-3p	down
RAD21	up	gga-miR-458a-3p	down
RAD21	up	hsa-miR-21-5p_R+2_1ss1TN	down
RAD21	up	oan-let-7f-1-3p_R+2_1ss8AG	down
RAD54L	up	gga-miR-214_L+1R-3_1ss19GA	down
RARRES1	down	aca-miR-181a_R+3	up
RARRES1	down	gga-miR-181a-5p	up
RARRES1	down	gga-miR-181b-5p_R+1	up
RARRES1	down	gga-miR-20b-3p_R-1	up
RARRES1	down	gga-miR-30a-3p_1ss18GA	up
RARRES1	down	gga-miR-30e-3p_L+1	up
RARRES1	down	gga-miR-34a-3p_R-1	up
RARRES1	down	pma-miR-181a-5p_R+3	up
RASA3	down	gga-miR-20b-5p	up
RASA3	down	gga-miR-222a	up
RASA3	down	oan-miR-19b-1-5p_R+4	up
RASSF2	down	aca-miR-129b-5p_R+1_1ss20TG	up
RASSF2	down	aca-miR-181a_R+3	up
RASSF2	down	gga-let-7c-3p_1ss12TC	up
RASSF2	down	gga-miR-107-5p_R+1	up
RASSF2	down	gga-miR-130b-3p	up

RASSF2	down	gga-miR-130b-5p	up
RASSF2	down	gga-miR-130b-5p_R-1	up
RASSF2	down	gga-miR-130b-5p_R-3	up
RASSF2	down	gga-miR-144-3p_L-1R-2	up
RASSF2	down	gga-miR-1552-5p_R+1	up
RASSF2	down	gga-miR-15b-3p_L+1_1ss22AC	up
RASSF2	down	gga-miR-181a-5p	up
RASSF2	down	gga-miR-181b-5p_R+1	up
RASSF2	down	gga-miR-222a	up
RASSF2	down	gga-miR-30c-1-3p_R+1	up
RASSF2	down	gga-miR-454-3p	up
RASSF2	down	gga-miR-489-3p_L-2R+1	up
RASSF2	down	ggo-miR-342_R+1	up
RASSF2	down	oan-miR-19b-1-5p_R+4	up
RASSF2	down	pma-miR-181a-5p_R+3	up
RBM24	down	gga-miR-106-3p_R+1	up
RBM24	down	gga-miR-107-3p_R-1	up
RBM24	down	gga-miR-125b-3p_R-1_1ss21CT	up
RBM24	down	gga-miR-144-3p_L-1R-2	up
RBM24	down	gga-miR-1559-3p	up
RBM24	down	gga-miR-1559-5p	up
RBM24	down	gga-miR-15b-5p_R-1	up
RBM24	down	gga-miR-1699_L-1R-2	up
RBM24	down	gga-miR-16c-5p	up
RBM24	down	gga-miR-222a	up
RBM24	down	gga-miR-383-3p	up

RBM24	down	gga-miR-6604-5p_1ss7GA	up
RBM24	down	oan-miR-19b-1-5p_R+4	up
RCBTB1	up	cin-miR-33_R+4	down
RCBTB1	up	gga-miR-214_L+1R-3_1ss19GA	down
RCBTB1	up	gga-miR-33-5p	down
RCC2	up	gga-let-7b_L-1R-1	down
RCC2	up	gga-miR-147_R+1	down
RCC2	up	gga-miR-2954_R+2	down
RCC2	up	gga-miR-29c-3p_L+1R+1_1ss19GA	down
RCC2	up	hsa-miR-3120-3p_R+1	down
REEP5	down	cgr-miR-326	up
REEP5	down	gga-miR-1559-3p	up
REEP5	down	gga-miR-2131-3p_R+1	up
REEP5	down	gga-miR-6548-5p	up
REEP5	down	gga-miR-92-5p	up
REL	down	aca-miR-181a_R+3	up
REL	down	gga-miR-106-3p_R+1	up
REL	down	gga-miR-107-5p_R+1	up
REL	down	gga-miR-130b-5p	up
REL	down	gga-miR-130b-5p_R-1	up
REL	down	gga-miR-130b-5p_R-3	up
REL	down	gga-miR-144-3p_L-1R-2	up
REL	down	gga-miR-16-1-3p_R-1	up
REL	down	gga-miR-1668-3p	up
REL	down	gga-miR-16c-3p_L+1R-1	up
REL	down	gga-miR-17-3p_L-1R+3	up

REL	down	gga-miR-1796	up
REL	down	gga-miR-460a-5p	up
REL	down	ggo-miR-342_R+1	up
REL	down	hsa-miR-143-5p	up
REL	down	hsa-miR-4443_R+1	up
REL	down	pma-miR-181a-5p_R+3	up
REPS2	up	gga-let-7j-3p_L-1R+2	down
REPS2	up	gga-miR-155_R+1	down
REPS2	up	gga-miR-29b-3p_R-1	down
REPS2	up	gga-miR-29c-3p_L+1R+1_1ss19GA	down
REPS2	up	gga-miR-29c-3p_R+1	down
REPS2	up	gga-miR-29c-3p_R+1_1ss18GA	down
REPS2	up	ssa-miR-29b-3p_R-1_2ss20TC21TC	down
RFESD	down	gga-miR-107-5p_R+1	up
RFESD	down	gga-miR-1559-3p	up
RFESD	down	gga-miR-6548-5p	up
RFESD	down	gga-miR-6604-5p_1ss7GA	up
RFESD	down	PC-5p-479025_5	up
RFFL	down	aca-miR-18a-5p	up
RFFL	down	cgr-miR-500	up
RFFL	down	gga-miR-106-3p_R+1	up
RFFL	down	gga-miR-15b-5p_R-1	up
RFFL	down	gga-miR-16c-5p	up
RFFL	down	gga-miR-216b	up
RFFL	down	gga-miR-222a	up
RFFL	down	gga-miR-301a-5p_L+2	up

RFFL	down	gga-miR-460a-5p	up
RFFL	down	gga-miR-6548-5p	up
RFFL	down	gga-miR-6604-5p_1ss7GA	up
RFFL	down	ggo-miR-342_R+1	up
RFFL	down	hsa-miR-4443_R+1	up
RFFL	down	mmu-miR-500-3p_R-1	up
RGL1	down	aca-miR-181a_R+3	up
RGL1	down	gga-miR-107-5p_R+1	up
RGL1	down	gga-miR-130b-3p	up
RGL1	down	gga-miR-181a-5p	up
RGL1	down	gga-miR-181b-5p_R+1	up
RGL1	down	gga-miR-20b-3p_R-1	up
RGL1	down	gga-miR-429-3p	up
RGL1	down	gga-miR-454-3p	up
RGL1	down	gga-miR-6604-5p_1ss7GA	up
RGL1	down	mmu-miR-8094_L+1R+3_1ss3AG	up
RGL1	down	oan-miR-363-3p_R+1	up
RGL1	down	pma-miR-181a-5p_R+3	up
RGN	down	PC-3p-68190_58	up
RGS1	down	gga-miR-1731-5p	up
RGS1	down	gga-miR-460a-5p	up
RGS14	down	gga-miR-1467-3p_L+1	up
RGS2	down	gga-miR-107-3p_R-1	up
RGS2	down	gga-miR-125b-3p_R-1_1ss21CT	up
RGS2	down	gga-miR-1555-5p	up
RGS2	down	gga-miR-429-3p	up

RGS9BP	down	aca-miR-138-2-3p_L-2R+3	up
RGS9BP	down	gga-miR-125b-3p_R-1_1ss21CT	up
RGS9BP	down	gga-miR-1552-5p_R+1	up
RGS9BP	down	gga-miR-1559-3p	up
RGS9BP	down	gga-miR-15b-5p_R-1	up
RGS9BP	down	gga-miR-16c-5p	up
RGS9BP	down	gga-miR-181b-1-3p_L+2R-2	up
RGS9BP	down	gga-miR-202-5p_L-1	up
RGS9BP	down	gga-miR-30a-3p_1ss18GA	up
RGS9BP	down	gga-miR-30a-3p_2ss10GA20AT	up
RGS9BP	down	gga-miR-30a-5p_R+2_1ss13CT	up
RGS9BP	down	gga-miR-30b-5p	up
RGS9BP	down	gga-miR-30c-5p	up
RGS9BP	down	gga-miR-30d_R+2	up
RGS9BP	down	gga-miR-30e-3p_L+1	up
RGS9BP	down	gga-miR-30e-5p_R+5	up
RGS9BP	down	gga-miR-3529	up
RGS9BP	down	gga-miR-429-3p	up
RGS9BP	down	gga-miR-6710-3p_R+1	up
RGS9BP	down	hsa-miR-181b-3p_L+1R-1	up
RGS9BP	down	tgu-miR-30a-5p_R+2	up
RHBG	down	gga-miR-107-3p_R-1	up
RHBG	down	gga-miR-138-5p_R+7	up
RHBG	down	gga-miR-1747-3p	up
RHBG	down	gga-miR-383-3p	up
RHBG	down	gga-miR-6604-5p_1ss7GA	up

RICTOR	down	gga-miR-144-3p_L-1R-2	up
RICTOR	down	gga-miR-16c-3p_L+1R-1	up
RICTOR	down	gga-miR-30a-3p_1ss18GA	up
RICTOR	down	gga-miR-30e-3p_L+1	up
RINT1	down	gga-miR-1552-3p_R+1	up
RINT1	down	gga-miR-1602	up
RINT1	down	gga-miR-1668-3p	up
RINT1	down	gga-miR-205a_R+1	up
RINT1	down	gga-miR-205b	up
RINT1	down	gga-miR-3529	up
RINT1	down	hsa-miR-143-5p	up
RINT1	down	tni-miR-205_R+2	up
RNASET2	down	chi-miR-361-5p	up
RNASET2	down	efu-miR-361	up
RNASET2	down	gga-miR-106-3p_R+1	up
RNASET2	down	gga-miR-130b-5p	up
RNASET2	down	gga-miR-130b-5p_R-1	up
RNASET2	down	gga-miR-130b-5p_R-3	up
RNASET2	down	gga-miR-137-3p_L+1	up
RNASET2	down	gga-miR-16-1-3p_R-1	up
RNASET2	down	gga-miR-6710-3p_R+1	up
RRAS2	down	aca-miR-18a-5p	up
RRAS2	down	bta-miR-450a	up
RRAS2	down	gga-miR-107-3p_R-1	up
RRAS2	down	gga-miR-16-1-3p_R-1	up
RRAS2	down	gga-miR-301a-5p_L+2	up

RRAS2	down	gga-miR-30c-1-3p_R+1	up
RRAS2	down	mmu-miR-8094_L+1R+3_1ss3AG	up
RRM1	up	gga-let-7f-3p_1ss22CT	down
RRM1	up	gga-miR-1329-5p	down
RRM1	up	hsa-miR-3120-3p_R+1	down
RRP12	up	aca-miR-150-5p_1ss12TC	down
RRP12	up	gga-miR-140-3p_L+1R+1_1ss22CA	down
RRP12	up	sha-miR-140_R+2	down
RSFR	down	gga-miR-107-3p_R-1	up
RSFR	down	gga-miR-15b-5p_R-1	up
RSFR	down	gga-miR-16c-5p	up
RSFR	down	mmu-mir-5098-p5	up
SALL4	up	gga-miR-135a-2-3p_L-1R+1	down
SALL4	up	gga-mir-147-p3_1	down
SALL4	up	gga-mir-147-p5	down
SALL4	up	gga-miR-200b-5p_L+2	down
SBNO2	down	gga-miR-107-3p_R-1	up
SBNO2	down	gga-miR-138-5p_R+7	up
SBNO2	down	gga-miR-1602	up
SCIN	down	aca-miR-181a_R+3	up
SCIN	down	gga-let-7c-3p_1ss12TC	up
SCIN	down	gga-miR-107-3p_R-1	up
SCIN	down	gga-miR-130b-5p	up
SCIN	down	gga-miR-130b-5p_R-1	up
SCIN	down	gga-miR-1663-5p_R+2	up
SCIN	down	gga-miR-1668-3p	up

SCIN	down	gga-miR-181a-5p	up
SCIN	down	gga-miR-181b-5p_R+1	up
SCIN	down	gga-miR-205a_R+1	up
SCIN	down	gga-miR-20b-3p_R-1	up
SCIN	down	gga-miR-2131-3p_R+1	up
SCIN	down	gga-miR-301a-5p_L+2	up
SCIN	down	gga-miR-456-3p_1ss22AT	up
SCIN	down	gga-miR-456-3p_R-1	up
SCIN	down	gga-miR-489-3p_L-2R+1	up
SCIN	down	hsa-miR-4443_R+1	up
SCIN	down	pma-miR-181a-5p_R+3	up
SCIN	down	pma-miR-456_R+1_1ss22AC	up
SCIN	down	tni-miR-205_R+2	up
SDC4	down	gga-miR-137-3p_L+1	up
SDC4	down	gga-miR-6604-5p_1ss7GA	up
SEMA3A	up	gga-miR-146c-5p_4ss18GA20AG22TG23GT	down
SEMA3A	up	ola-miR-199a-3p_R+1_1ss22AG	down
SERPINB10	down	aca-miR-129b-5p_R+1_1ss20TG	up
SERPINB10	down	csa-let-7d_1ss10TC	up
SERPINB10	down	gga-miR-16c-3p_L+1R-1	up
SERPINB10	down	gga-miR-2131-3p_R+1	up
SERPINB10	down	gga-miR-30a-3p_1ss18GA	up
SERPINB10	down	gga-miR-30a-3p_2ss10GA20AT	up
SERPINB10	down	gga-miR-30e-3p_L+1	up
SERPINB10	down	gga-miR-92-5p	up
SERPINB10	down	mmu-miR-8094_L+1R+3_1ss3AG	up

SERPINB6	down	gga-miR-130b-3p	up
SERPINB6	down	gga-miR-1451-3p_R-1	up
SERPINB6	down	gga-miR-1552-3p_R+1	up
SERPINB6	down	gga-miR-1668-3p	up
SERPINB6	down	gga-miR-1788-3p_R+1	up
SERPINB6	down	gga-miR-181b-1-3p_L+1R-1	up
SERPINB6	down	gga-miR-454-3p	up
SERPINB6	down	gga-miR-460a-5p	up
SERPINB6	down	gga-miR-6604-5p_1ss7GA	up
SERPINB6	down	mmu-mir-5098-p5	up
SFXN2	up	gga-miR-222b-5p_R-4	down
SFXN5	up	gga-miR-223_1ss15AG	down
SFXN5	up	gga-miR-223_1ss19CT	down
SFXN5	up	gga-miR-223_R+1	down
SIK1	down	gga-miR-107-5p_R+1	up
SIK1	down	gga-miR-130b-3p	up
SIK1	down	gga-miR-130b-5p	up
SIK1	down	gga-miR-130b-5p_R-1	up
SIK1	down	gga-miR-130b-5p_R-3	up
SIK1	down	gga-miR-137-3p_L+1	up
SIK1	down	gga-miR-1668-3p	up
SIK1	down	gga-miR-16c-5p	up
SIK1	down	gga-miR-1759-3p_L-3	up
SIK1	down	gga-miR-1788-5p	up
SIK1	down	gga-miR-20b-3p_R-1	up
SIK1	down	gga-miR-20b-5p	up

SIK1	down	gga-miR-30a-3p_1ss18GA	up
SIK1	down	gga-miR-30a-3p_2ss10GA20AT	up
SIK1	down	gga-miR-30e-3p_L+1	up
SIK1	down	gga-miR-3607-5p_R+1	up
SIK1	down	gga-miR-454-3p	up
SIK1	down	oan-miR-363-3p_R+1	up
SIPA1L2	down	gga-miR-130b-5p	up
SIPA1L2	down	gga-miR-130b-5p_R-1	up
SIPA1L2	down	gga-miR-130b-5p_R-3	up
SIPA1L2	down	gga-miR-222a	up
SKIL	down	aca-miR-18a-3p_L+1R-1_1ss12GA	up
SKIL	down	gga-miR-130b-5p	up
SKIL	down	gga-miR-1720-5p	up
SKIL	down	gga-miR-1731-5p	up
SKIL	down	gga-miR-181b-1-3p_L+1R-1	up
SKIL	down	gga-miR-181b-1-3p_L+2R-2	up
SKIL	down	gga-miR-460a-5p	up
SKIL	down	hsa-miR-181b-3p_L+1R-1	up
SKIL	down	hsa-miR-4286_R+1	up
SLBP	up	aca-miR-425-5p_R+4	down
SLBP	up	gga-mir-147-p3_2	down
SLBP	up	gga-miR-1662_R-3	down
SLBP	up	hsa-miR-3120-3p_R+1	down
SLBP	up	hsa-miR-425-5p_R+2_1ss20TC	down
SLBP	up	tgu-miR-1662_L+1_1ss23TA	down
SLC15A4	down	gga-miR-107-3p_R-1	up

SLC15A4	down	gga-miR-130b-3p	up
SLC15A4	down	gga-miR-30a-5p_R+2_1ss13CT	up
SLC15A4	down	gga-miR-30b-5p	up
SLC15A4	down	gga-miR-30c-5p	up
SLC15A4	down	gga-miR-30d_R+2	up
SLC15A4	down	gga-miR-30e-5p_R+5	up
SLC15A4	down	gga-miR-454-3p	up
SLC15A4	down	tgu-miR-30a-5p_R+2	up
SLC16A1	up	aca-miR-150-5p_1ss12TC	down
SLC16A1	up	gga-miR-146c-5p_4ss18GA20AG22TG23GT	down
SLC16A1	up	gga-miR-214_L+1R-3_1ss19GA	down
SLC16A1	up	gga-miR-222b-5p_R-4	down
SLC16A1	up	xtr-miR-429_R+2	down
SLC24A4	down	gga-miR-15b-5p_R-1	up
SLC24A4	down	gga-miR-16c-5p	up
SLC24A4	down	gga-miR-20b-5p	up
SLC25A1	up	aca-miR-150-5p_1ss12TC	down
SLC25A1	up	gga-miR-135a-2-3p_L-1R+1	down
SLC25A1	up	gga-miR-146a-3p_R-1	down
SLC38A2	down	gga-miR-106-3p_R+1	up
SLC38A2	down	gga-miR-144-3p_L-1R-2	up
SLC38A2	down	gga-miR-1552-3p_R+1	up
SLC38A2	down	gga-miR-1731-5p	up
SLC38A2	down	gga-miR-1759-3p_L-3	up
SLC38A2	down	gga-miR-216b	up
SLC38A2	down	gga-miR-222a	up

SLC38A2	down	gga-miR-30a-3p_1ss18GA	up
SLC38A2	down	gga-miR-30a-3p_2ss10GA20AT	up
SLC38A2	down	gga-miR-30e-3p_L+1	up
SLC38A2	down	gga-miR-3607-5p_R+1	up
SLC38A2	down	gga-miR-429-3p	up
SLC38A2	down	gga-miR-460a-5p	up
SLC38A2	down	gga-miR-6548-5p	up
SLC38A2	down	gga-miR-6604-5p_1ss7GA	up
SLC38A2	down	mmu-miR-8094_L+1R+3_1ss3AG	up
SLC46A3	down	gga-miR-107-3p_R-1	up
SLC46A3	down	gga-miR-130b-3p	up
SLC46A3	down	gga-miR-15b-5p_R-1	up
SLC46A3	down	gga-miR-16c-5p	up
SLC46A3	down	gga-miR-1783_R+1	up
SLC46A3	down	gga-miR-205a_R+1	up
SLC46A3	down	gga-miR-205b	up
SLC46A3	down	gga-miR-20b-5p	up
SLC46A3	down	gga-miR-34a-3p_R-1	up
SLC46A3	down	gga-miR-383-3p	up
SLC46A3	down	gga-miR-454-3p	up
SLC46A3	down	gga-miR-456-3p_1ss22AT	up
SLC46A3	down	gga-miR-456-3p_R-1	up
SLC46A3	down	hsa-miR-4286_R+1	up
SLC46A3	down	mmu-miR-8094_L+1R+3_1ss3AG	up
SLC46A3	down	oan-miR-19b-1-5p_R+4	up
SLC46A3	down	pma-miR-456_R+1_1ss22AC	up

SLC46A3	down	tni-miR-205_R+2	up
SLC6A2	down	gga-miR-130b-5p	up
SLC6A2	down	gga-miR-130b-5p_R-1	up
SLC6A2	down	gga-miR-130b-5p_R-3	up
SLC6A2	down	gga-miR-1663-5p_R+2	up
SLC6A2	down	gga-miR-1731-5p	up
SLC6A2	down	gga-miR-1783_R+1	up
SLC6A2	down	gga-miR-2131-3p_R+1	up
SLC6A2	down	gga-miR-6548-5p	up
SLC6A2	down	gga-miR-6604-5p_1ss7GA	up
SLC9A3R1	up	aca-miR-150-5p_1ss12TC	down
SLC9A3R1	up	gga-miR-140-3p_L+1R+1_1ss22CA	down
SLC9A3R1	up	sha-miR-140_R+2	down
SLC9A7	down	aca-miR-181a_R+3	up
SLC9A7	down	gga-miR-181a-5p	up
SLC9A7	down	gga-miR-181b-5p_R+1	up
SLC9A7	down	hsa-miR-4286_R+1	up
SLC9A7	down	pma-miR-181a-5p_R+3	up
SLU7	down	cgr-miR-151-3p	up
SLU7	down	gga-let-7c-3p_1ss12TC	up
SLU7	down	gga-miR-1467-3p_L+1	up
SLU7	down	gga-miR-17-3p_L-1R+3	up
SLU7	down	gga-miR-30a-5p_R+2_1ss13CT	up
SLU7	down	gga-miR-30d_R+2	up
SLU7	down	gga-miR-30e-5p_R+5	up
SLU7	down	gga-miR-34a-3p_R-1	up

SLU7	down	gga-miR-460a-5p	up
SLU7	down	gga-miR-490-3p_R+1	up
SLU7	down	tgu-miR-30a-5p_R+2	up
SMOC1	up	gga-let-7j-3p_L-1R+2	down
SMOC1	up	oan-let-7f-1-3p_R+2_1ss8AG	down
SNX9	down	gga-miR-30c-1-3p_R+1	up
SNX9	down	gga-miR-30d_R+2	up
SNX9	down	tgu-miR-30a-5p_R+2	up
SPSB1	down	aca-miR-18a-3p_L+1R-1_1ss12GA	up
SRM	up	aca-miR-425-5p_R+4	down
SRM	up	dre-miR-24_R+2	down
SRM	up	gga-miR-200b-5p_L+2	down
SRM	up	gga-miR-223_1ss15AG	down
SRM	up	gga-miR-223_1ss19CT	down
SRM	up	gga-miR-223_R+1	down
SRM	up	hsa-miR-425-5p_R+2_1ss20TC	down
SRRM1	up	gga-miR-1329-5p	down
SRRM1	up	gga-miR-140-5p_L+1	down
SSR1	up	gga-miR-214_L+1R-3_1ss19GA	down
ST6GAL1	down	gga-miR-20b-5p	up
ST6GAL1	down	hsa-miR-143-5p	up
ST6GAL1	down	oan-miR-363-3p_R+1	up
ST6GAL1	down	oha-miR-7-1-3p_1ss10GA	up
ST6GAL1	down	PC-5p-479025_5	up
ST8SIA1	down	gga-miR-216b	up
STARD5	down	gga-miR-138-5p_R+7	up

STARD5	down	gga-miR-7460-3p	up
STK17B	down	aca-miR-363-5p	up
STK17B	down	gga-miR-130b-3p	up
STK17B	down	gga-miR-1663-5p_R+2	up
STK17B	down	gga-miR-181b-1-3p_L+2R-2	up
STK17B	down	gga-miR-20b-5p	up
STK17B	down	gga-miR-30a-3p_1ss18GA	up
STK17B	down	gga-miR-30a-5p_R+2_1ss13CT	up
STK17B	down	gga-miR-30b-5p	up
STK17B	down	gga-miR-30c-5p	up
STK17B	down	gga-miR-30d_R+2	up
STK17B	down	gga-miR-30e-3p_L+1	up
STK17B	down	gga-miR-30e-5p_R+5	up
STK17B	down	gga-miR-454-3p	up
STK17B	down	gga-miR-460a-5p	up
STK17B	down	hsa-miR-143-5p	up
STK17B	down	hsa-miR-181b-3p_L+1R-1	up
STK17B	down	oha-miR-7-1-3p_1ss10GA	up
STK17B	down	tgu-miR-30a-5p_R+2	up
STK32A	down	cgr-miR-326	up
STK32A	down	gga-miR-138-5p_R+7	up
STOML1	down	gga-mir-1609-p3	up
SUCLA2	down	cgr-miR-500	up
SUCLA2	down	gga-miR-106-3p_R+1	up
SUCLA2	down	gga-miR-144-3p_L-1R-2	up
SUCLA2	down	mmu-miR-500-3p_R-1	up

SUCLG1	up	gga-miR-1329-5p	down
SUCLG1	up	gga-miR-9-5p	down
SUCLG1	up	rno-miR-122-5p_L+1R+1_1	down
SUCLG1	up	rno-miR-122-5p_L+1R+1_2	down
SYK	down	aca-miR-138-2-3p_L-2R+3	up
SYK	down	gga-miR-1796	up
SYK	down	ggo-miR-342_R+1	up
SYK	down	PC-5p-479025_5	up
SYTL2	down	gga-miR-20b-5p	up
SYTL2	down	gga-miR-30a-3p_1ss18GA	up
SYTL2	down	gga-miR-30e-3p_L+1	up
SYTL2	down	gga-miR-454-3p	up
TAPBPL	down	chi-miR-361-5p	up
TAPBPL	down	efu-miR-361	up
TAPBPL	down	gga-miR-1306-3p_R-1	up
TAPBPL	down	gga-miR-7460-3p	up
TAPBPL	down	ggo-miR-342_R+1	up
TAT	down	aca-miR-18a-3p_L+1R-1_1ss12GA	up
TAT	down	gga-miR-107-5p_R+1	up
TAT	down	ggo-miR-342_R+1	up
TAX1BP1	down	mmu-miR-8094_L+1R+3_1ss3AG	up
TBL3	up	gga-let-7j-3p_L-1R+2	down
TBL3	up	oan-let-7f-1-3p_R+2_1ss8AG	down
TEC	down	aca-miR-129b-5p_R+1_1ss20TG	up
TEC	down	aca-miR-181a_R+3	up
TEC	down	gga-miR-107-3p_R-1	up

TEC	down	gga-miR-137-3p_L+1	up
TEC	down	gga-miR-15b-5p_R-1	up
TEC	down	gga-miR-16c-5p	up
TEC	down	gga-miR-429-3p	up
TEC	down	gga-miR-6710-3p_R+1	up
TEC	down	pma-miR-181a-5p_R+3	up
TGFBI	down	gga-miR-137-3p_L+1	up
TGFBI	down	gga-miR-1759-3p_L-3	up
TGFBI	down	gga-miR-3607-5p_R+1	up
TGFBI	down	oha-miR-7-1-3p_1ss10GA	up
THBS1	down	gga-miR-3529	up
THBS1	down	gga-miR-460a-3p	up
THBS1	down	gga-miR-460a-5p	up
THBS1	down	hsa-miR-143-5p	up
TIMD4	down	gga-miR-107-3p_R-1	up
TIMD4	down	gga-miR-107-5p_R+1	up
TIMD4	down	gga-miR-17-3p_L-1R+3	up
TIMD4	down	gga-miR-1747-3p	up
TIMP3	down	gga-miR-1699_L-1R-2	up
TIMP3	down	gga-miR-30c-1-3p_R+1	up
TINAG	up	gga-let-7j-3p_L-1R+2	down
TINAG	up	gga-mir-147-p3_1	down
TINAG	up	gga-mir-147-p5	down
TK1	up	gga-miR-214_L+1R-3_1ss19GA	down
TK1	up	gga-miR-223_1ss15AG	down
TK1	up	gga-miR-223_1ss19CT	down

TK1	up	gga-miR-223_R+1	down
TK1	up	hsa-miR-3120-3p_R+1	down
TLR4	down	gga-miR-106-3p_R+1	up
TLR4	down	gga-miR-107-5p_R+1	up
TLR4	down	gga-miR-1699_L-1R-2	up
TLR4	down	gga-miR-181b-1-3p_L+1R-1	up
TLR4	down	gga-miR-6604-5p_1ss7GA	up
TLR4	down	gga-miR-7460-3p	up
TLR4	down	mmu-mir-5098-p5	up
TLR4	down	PC-3p-68190_58	up
TLR4	down	PC-5p-479025_5	up
TMEM41A	down	gga-miR-30a-3p_1ss18GA	up
TMEM41A	down	gga-miR-30a-3p_2ss10GA20AT	up
TMEM41A	down	gga-miR-30e-3p_L+1	up
TMEM41B	down	chi-miR-361-5p	up
TMEM41B	down	efu-miR-361	up
TMEM41B	down	gga-miR-1559-3p	up
TMEM41B	down	gga-miR-15b-5p_R-1	up
TMEM41B	down	gga-miR-1783_R+1	up
TMEM41B	down	gga-miR-30a-5p_R+2_1ss13CT	up
TMEM41B	down	gga-miR-30d_R+2	up
TMEM41B	down	gga-miR-30e-5p_R+5	up
TMEM41B	down	gga-miR-383-3p	up
TMEM41B	down	gga-miR-429-3p	up
TMEM41B	down	gga-miR-6548-5p	up
TMEM41B	down	gga-miR-6604-5p_1ss7GA	up

TMEM41B	down	oan-miR-19b-1-5p_R+4	up
TMEM41B	down	PC-3p-68190_58	up
TMEM41B	down	tgu-miR-30a-5p_R+2	up
TMEM68	down	aca-miR-181a_R+3	up
TMEM68	down	aca-miR-363-5p	up
TMEM68	down	cgr-miR-500	up
TMEM68	down	gga-miR-1555-5p	up
TMEM68	down	gga-miR-16-1-3p_R-1	up
TMEM68	down	gga-miR-17-3p_L-1R+3	up
TMEM68	down	gga-miR-1796	up
TMEM68	down	gga-miR-181a-5p	up
TMEM68	down	gga-miR-181b-1-3p_L+1R-1	up
TMEM68	down	gga-miR-181b-5p_R+1	up
TMEM68	down	gga-miR-205a_R+1	up
TMEM68	down	gga-miR-205b	up
TMEM68	down	gga-miR-429-5p_L-1	up
TMEM68	down	gga-miR-490-3p_R+1	up
TMEM68	down	hsa-miR-143-5p	up
TMEM68	down	mmu-miR-500-3p_R-1	up
TMEM68	down	oha-miR-7-1-3p_1ss10GA	up
TMEM68	down	pma-miR-181a-5p_R+3	up
TMEM68	down	tni-miR-205_R+2	up
TNFRSF18	down	cgr-miR-500	up
TNFRSF18	down	gga-miR-1602	up
TNFRSF18	down	gga-miR-17-3p_L-1R+3	up
TNFRSF18	down	gga-miR-181b-1-3p_L+2R-2	up

TNFRSF18	down	gga-miR-460a-3p	up
TNFRSF18	down	ggo-miR-342_R+1	up
TNFRSF18	down	hsa-miR-143-5p	up
TNFRSF18	down	hsa-miR-181b-3p_L+1R-1	up
TNFRSF18	down	mmu-miR-500-3p_R-1	up
TNFRSF18	down	PC-3p-68190_58	up
TP53INP1	down	aca-miR-18a-3p_L+1R-1_1ss12GA	up
TP53INP1	down	cgr-miR-151-3p	up
TP53INP1	down	cgr-miR-326	up
TP53INP1	down	gga-miR-107-5p_R+1	up
TP53INP1	down	gga-miR-1306-3p_R-1	up
TP53INP1	down	gga-miR-144-3p_L-1R-2	up
TP53INP1	down	gga-miR-16-1-3p_R-1	up
TP53INP1	down	gga-miR-1668-3p	up
TP53INP1	down	gga-miR-17-3p_L-1R+3	up
TP53INP1	down	gga-miR-205a_R+1	up
TP53INP1	down	gga-miR-205b	up
TP53INP1	down	gga-miR-20b-5p	up
TP53INP1	down	gga-miR-30a-5p_R+2_1ss13CT	up
TP53INP1	down	gga-miR-30c-5p	up
TP53INP1	down	gga-miR-30d_R+2	up
TP53INP1	down	gga-miR-30e-5p_R+5	up
TP53INP1	down	gga-miR-6604-5p_1ss7GA	up
TP53INP1	down	ggo-miR-342_R+1	up
TP53INP1	down	hsa-miR-143-5p	up
TP53INP1	down	hsa-miR-4443_R+1	up

TP53INP1	down	mmu-miR-8094_L+1R+3_1ss3AG	up
TP53INP1	down	oha-miR-7-1-3p_1ss10GA	up
TP53INP1	down	tgu-miR-30a-5p_R+2	up
TP53INP1	down	tni-miR-205_R+2	up
TPI1	up	gga-miR-22-3p	down
TPX2	up	gga-miR-214_L+1R-3_1ss19GA	down
TPX2	up	hsa-miR-3120-3p_R+1	down
TRAIP	up	gga-miR-2954_R+2	down
TRAP1	up	hsa-miR-3120-3p_R+1	down
TRIM24	down	gga-miR-16-1-3p_R-1	up
TSPAN1	up	cin-miR-33_R+4	down
TSPAN1	up	gga-miR-140-3p_L+1R+1_1ss22CA	down
TSPAN1	up	gga-miR-222b-5p_R-4	down
TSPAN1	up	gga-miR-33-5p	down
TSPAN1	up	sha-miR-140_R+2	down
TSSC4	up	odi-miR-7_R+2	down
UBE2J1	down	gga-miR-222a	up
UBE2J1	down	gga-miR-30a-5p_R+2_1ss13CT	up
UBE2J1	down	gga-miR-30b-5p	up
UBE2J1	down	gga-miR-30c-5p	up
UBE2J1	down	gga-miR-30e-5p_R+5	up
UBE2J1	down	gga-miR-34a-3p_R-1	up
UBE2J1	down	gga-miR-460a-5p	up
UBE2J1	down	ggo-miR-342_R+1	up
UBE2J1	down	tgu-miR-30a-5p_R+2	up
UBXN6	down	mmu-miR-8094_L+1R+3_1ss3AG	up

UGP2	down	gga-miR-106-3p_R+1	up
UGP2	down	gga-miR-144-3p_L-1R-2	up
UGP2	down	gga-miR-1699_L-1R-2	up
UGP2	down	gga-miR-216b	up
UGP2	down	gga-miR-383-3p	up
UGP2	down	gga-miR-429-3p	up
UGP2	down	gga-miR-429-5p_L-1	up
UGP2	down	PC-3p-68190_58	up
UHRF1BP1L	up	cin-miR-33_R+4	down
UHRF1BP1L	up	gga-miR-29a-5p_L-1R-2	down
UHRF1BP1L	up	gga-miR-33-5p	down
UQCRH	up	gga-miR-140-3p_L+1R+1_1ss22CA	down
UQCRH	up	gga-miR-222b-5p_R-4	down
UQCRH	up	sha-miR-140_R+2	down
VAT1	down	gga-miR-15b-5p_R-1	up
VAT1	down	gga-miR-16c-5p	up
VAV3	down	csa-let-7d_1ss10TC	up
VAV3	down	gga-miR-107-3p_R-1	up
VAV3	down	gga-miR-107-5p_R+1	up
VAV3	down	gga-miR-138-5p_R+7	up
VAV3	down	gga-miR-17-3p_L-1R+3	up
VAV3	down	gga-miR-1747-3p	up
VAV3	down	gga-miR-1796	up
VAV3	down	gga-miR-205a_R+1	up
VAV3	down	gga-miR-205b	up
VAV3	down	gga-miR-489-3p_L-2R+1	up

VAV3	down	gga-miR-6548-5p	up
VAV3	down	gga-miR-6710-3p_R+1	up
VAV3	down	oha-miR-7-1-3p_1ss10GA	up
VAV3	down	tni-miR-205_R+2	up
VCAM1	down	aca-miR-181a_R+3	up
VCAM1	down	gga-let-7c-3p_1ss12TC	up
VCAM1	down	gga-miR-181a-5p	up
VCAM1	down	gga-miR-181b-5p_R+1	up
VCAM1	down	gga-miR-20b-3p_R-1	up
VCAM1	down	gga-miR-383-3p	up
VCAM1	down	gga-miR-429-3p	up
VCAM1	down	pma-miR-181a-5p_R+3	up
VWF	down	gga-miR-1663-5p_R+2	up
WDHD1	up	gga-miR-29b-3p_R-1	down
WDHD1	up	gga-miR-29c-3p_L+1R+1_1ss19GA	down
WDHD1	up	gga-miR-29c-3p_R+1	down
WDHD1	up	gga-miR-29c-3p_R+1_1ss18GA	down
WDHD1	up	ssa-miR-29b-3p_R-1_2ss20TC21TC	down
WDR1	down	gga-miR-106-3p_R+1	up
WDR1	down	gga-miR-125b-3p_R-1_1ss21CT	up
WDR1	down	gga-miR-1552-3p_R+1	up
WDR1	down	gga-miR-15b-5p_R-1	up
WDR1	down	gga-miR-16c-5p	up
WDR1	down	gga-miR-30a-5p_R+2_1ss13CT	up
WDR1	down	gga-miR-30b-5p	up
WDR1	down	gga-miR-30c-5p	up

WDR1	down	gga-miR-30d_R+2	up
WDR1	down	gga-miR-30e-5p_R+5	up
WDR1	down	gga-miR-460a-5p	up
WDR1	down	gga-miR-6548-5p	up
WDR1	down	hsa-miR-143-5p	up
WDR1	down	tgu-miR-30a-5p_R+2	up
WIPF1	down	gga-miR-106-3p_R+1	up
WIPF1	down	gga-miR-107-3p_R-1	up
WIPF1	down	gga-miR-130b-5p	up
WIPF1	down	gga-miR-130b-5p_R-1	up
WIPF1	down	gga-miR-130b-5p_R-3	up
WIPF1	down	gga-miR-144-3p_L-1R-2	up
WIPF1	down	gga-miR-1555-5p	up
WIPF1	down	gga-miR-16-1-3p_R-1	up
WIPF1	down	gga-miR-1796	up
WIPF1	down	gga-miR-30a-5p_R+2_1ss13CT	up
WIPF1	down	gga-miR-30b-5p	up
WIPF1	down	gga-miR-30c-5p	up
WIPF1	down	gga-miR-30d_R+2	up
WIPF1	down	gga-miR-30e-5p_R+5	up
WIPF1	down	gga-miR-429-3p	up
WIPF1	down	gga-miR-460a-5p	up
WIPF1	down	ggo-miR-342_R+1	up
WIPF1	down	tgu-miR-30a-5p_R+2	up
WNT11	down	aca-miR-129b-5p_R+1_1ss20TG	up
WNT11	down	gga-miR-137-3p_L+1	up

WNT4	down	aca-miR-129b-5p_R+1_1ss20TG	up
WSCD2	down	gga-miR-15b-5p_R-1	up
WSCD2	down	gga-miR-16c-5p	up
XBP1	down	gga-miR-1699_L-1R-2	up
XBP1	down	gga-miR-1747-3p	up
XBP1	down	gga-miR-205a_R+1	up
XBP1	down	gga-miR-205b	up
XBP1	down	gga-miR-456-3p_1ss22AT	up
XBP1	down	gga-miR-456-3p_R-1	up
XBP1	down	oan-miR-363-3p_R+1	up
XBP1	down	pma-miR-456_R+1_1ss22AC	up
XBP1	down	tni-miR-205_R+2	up
YBX1	up	hsa-miR-3120-3p_R+1	down
YPEL5	down	aca-miR-129b-5p_R+1_1ss20TG	up
YPEL5	down	cgr-miR-326	up
YPEL5	down	gga-miR-125b-3p_R-1_1ss21CT	up
YPEL5	down	gga-miR-130b-3p	up
YPEL5	down	gga-miR-1602	up
YPEL5	down	gga-miR-1668-3p	up
YPEL5	down	gga-miR-1759-3p_L-3	up
YPEL5	down	gga-miR-1788-3p_R+1	up
YPEL5	down	gga-miR-181b-1-3p_L+2R-2	up
YPEL5	down	gga-miR-20b-3p_R-1	up
YPEL5	down	gga-miR-365-2-5p_L-1R+1	up
YPEL5	down	gga-miR-454-3p	up
YPEL5	down	gga-miR-489-3p_L-2R+1	up

YPEL5	down	hsa-miR-181b-3p_L+1R-1	up
YPEL5	down	mmu-mir-5098-p5	up
ZEB1	down	gga-miR-144-3p_L-1R-2	up
ZEB1	down	gga-miR-16-1-3p_R-1	up
ZEB1	down	gga-miR-205a_R+1	up
ZEB1	down	gga-miR-216b	up
ZEB1	down	gga-miR-34a-3p_R-1	up
ZEB1	down	gga-miR-3607-5p_R+1	up
ZEB1	down	gga-miR-429-3p	up
ZEB1	down	tni-miR-205_R+2	up

Table S13. Mutual genes between the differentially expressed mRNAs and the target genes of 32 differentially expressed miRNAs which were selected with the criteria of FDR < 0.05 and fold changes either > 1.5 or < 0.67.

Symbol	gene regulation(immature VS mature liver)	miRNA ID	miRNA regulation(immature VS mature liver)
ARHGAP25	down	cin-miR-33_R+4	down
ARHGAP25	down	gga-miR-107-3p_R-1	up
ARHGAP25	down	gga-miR-130b-5p_R-1	up
SNRPE	up	aca-miR-18a-3p_L+1R-1_1ss12GA	up
BF1	down	gga-miR-125b-5p	down
RASSF2	down	gga-miR-130b-5p_R-1	up
RASSF2	down	gga-miR-181b-5p_R+1	up
RASSF2	down	gga-miR-489-3p_L-2R+1	up
CAMK4	up	cin-miR-33_R+4	down
CHTF8	up	gga-miR-146a-3p_R-1	down
ACOT7	up	gga-miR-181b-5p_R+1	up
CTSS	down	gga-miR-106-3p_R+1	up
CTSS	down	gga-miR-146a-3p_R-1	down
TAT	down	aca-miR-18a-3p_L+1R-1_1ss12GA	up
SLC25A1	up	gga-miR-146a-3p_R-1	down
SLC25A1	up	gga-miR-456-3p_1ss22AT	up
SLC25A1	up	gga-miR-456-3p_R-1	up
SLC25A1	up	pma-miR-456_R+1_1ss22AC	up
SRRM1	up	aca-miR-18a-3p_L+1R-1_1ss12GA	up

SRRM1	up	gga-miR-106-3p_R+1	up
SRRM1	up	gga-miR-1559-3p	up
FAM18B1	down	gga-miR-106-3p_R+1	up
FAM18B1	down	gga-miR-456-3p_1ss22AT	up
FAM18B1	down	gga-miR-456-3p_R-1	up
FAM18B1	down	pma-miR-456_R+1_1ss22AC	up
MYBBP1A	up	gga-miR-107-3p_R-1	up
MYBBP1A	up	gga-miR-130b-5p_R-1	up
MYBBP1A	up	gga-miR-301a-5p_L+2	up
PTPRU	up	gga-miR-107-3p_R-1	up
GSN	down	aca-miR-363-3p_R+1	up
GSN	down	oan-miR-363-3p_R+1	up
STOML1	down	cin-miR-33_R+4	down
NT5DC2	down	gga-miR-458a-3p	down
MYO1F	down	gga-miR-106-3p_R+1	up
MYO1F	down	gga-miR-107-3p_R-1	up
CDC45	up	gga-miR-107-3p_R-1	up
SLC16A1	up	gga-miR-106-3p_R+1	up
ACSBG2	down	aca-miR-363-3p_R+1	up
ACSBG2	down	cin-miR-33_R+4	down
ACSBG2	down	gga-let-7j-3p_L-1R+2	down
ACSBG2	down	gga-miR-106-3p_R+1	up
ACSBG2	down	gga-miR-125b-5p	down
ACSBG2	down	gga-miR-181b-5p_R+1	up
ACSBG2	down	oan-miR-363-3p_R+1	up
GFRA2	up	cin-miR-33_R+4	down

TNFRSF18	down	PC-3p-68190_58	up
VAV3	down	gga-let-7b_R+2	down
VAV3	down	gga-miR-107-3p_R-1	up
VAV3	down	gga-miR-489-3p_L-2R+1	up
VAV3	down	gga-miR-6710-3p_R+1	up
PMPCA	up	PC-3p-68190_58	up
PMPCA	up	gga-miR-107-3p_R-1	up
COMT	up	gga-miR-1559-3p	up
RFFL	down	cin-miR-33_R+4	down
RFFL	down	gga-miR-106-3p_R+1	up
RFFL	down	gga-miR-1729-5p_R-1	down
RFFL	down	gga-miR-301a-5p_L+2	up
DMXL1	down	gga-miR-1559-3p	up
AP3S1	down	gga-let-7j-3p_L-1R+2	down
AP3S1	down	gga-miR-107-3p_R-1	up
AP3S1	down	gga-miR-1451-3p_R-1	up
HSPA9	up	cin-miR-33_R+4	down
PSPH	down	cin-miR-33_R+4	down
CHCHD2	up	gga-miR-130b-5p_R-1	up
SPSB1	down	aca-miR-18a-3p_L+1R-1_1ss12GA	up
RGS2	down	gga-miR-107-3p_R-1	up
DEF6	down	gga-miR-107-3p_R-1	up
ANXA11	down	gga-miR-202-5p_L-1	up
SBNO2	down	gga-miR-107-3p_R-1	up
SLC15A4	down	gga-miR-107-3p_R-1	up
DNAJC12	down	gga-miR-106-3p_R+1	up

DNAJC12	down	gga-miR-1559-3p	up
DNAJC12	down	gga-miR-181b-5p_R+1	up
TRAIP	up	gga-miR-107-3p_R-1	up
KIF20A	up	cin-miR-33_R+4	down
KIF20A	up	gga-miR-1451-3p_R-1	up
GNA11	up	gga-miR-130b-5p_R-1	up
ITPA	up	gga-miR-107-3p_R-1	up
DNAJA4	down	cin-miR-33_R+4	down
DNAJA4	down	gga-miR-1559-3p	up
GBGT1	down	aca-miR-363-5p	up
GBGT1	down	gga-miR-489-3p_L-2R+1	up
ELL	down	gga-miR-1729-5p_R-1	down
AIMP2	up	gga-miR-458a-3p	down
BYSL	up	gga-miR-106-3p_R+1	up
BARD1	up	aca-miR-363-3p_R+1	up
BARD1	up	gga-miR-107-3p_R-1	up
BARD1	up	gga-miR-1559-3p	up
BARD1	up	gga-miR-181b-5p_R+1	up
BARD1	up	gga-miR-202-5p_L-1	up
BARD1	up	gga-miR-301a-5p_L+2	up
BARD1	up	oan-miR-363-3p_R+1	up
SLC6A2	down	gga-let-7j-3p_L-1R+2	down
SLC6A2	down	gga-miR-130b-5p_R-1	up
LPCAT2	down	gga-miR-1729-5p_R-1	down
LPCAT2	down	gga-miR-181b-5p_R+1	up
LPCAT2	down	gga-miR-20b-5p	up

KPNA2	up	gga-miR-20b-5p	up
FEN1	up	gga-miR-106-3p_R+1	up
FEN1	up	gga-miR-1559-3p	up
FEN1	up	gga-miR-301a-5p_L+2	up
SDF2	up	gga-miR-128-1-5p	up
TIMD4	down	gga-miR-107-3p_R-1	up
DNMT3A	up	gga-miR-456-3p_1ss22AT	up
DNMT3A	up	gga-miR-456-3p_R-1	up
DNMT3A	up	pma-miR-456_R+1_1ss22AC	up
DDX50	up	gga-let-7j-3p_L-1R+2	down
ADA	down	gga-miR-125b-5p	down
ADA	down	gga-miR-202-5p_L-1	up
ADA	down	gga-miR-301a-5p_L+2	up
P4HA1	down	rno-miR-122-5p_L+1R+1_1	down
P4HA1	down	rno-miR-122-5p_L+1R+1_2	down
COL18A1	up	gga-let-7j-3p_L-1R+2	down
RGS9BP	down	gga-miR-1559-3p	up
RGS9BP	down	gga-miR-202-5p_L-1	up
RGS9BP	down	gga-miR-3529	up
RGS9BP	down	gga-miR-6710-3p_R+1	up
CPSF4	up	gga-miR-301a-5p_L+2	up
RGL1	down	aca-miR-363-3p_R+1	up
RGL1	down	gga-miR-146a-3p_R-1	down
RGL1	down	gga-miR-181b-5p_R+1	up
RGL1	down	oan-miR-363-3p_R+1	up
ATPAF2	up	gga-miR-1559-3p	up

C2H3ORF75	up	gga-miR-107-3p_R-1	up
C2H3ORF75	up	gga-miR-456-3p_1ss22AT	up
C2H3ORF75	up	gga-miR-456-3p_R-1	up
C2H3ORF75	up	pma-miR-456_R+1_1ss22AC	up
MPP1	up	aca-miR-363-3p_R+1	up
MPP1	up	oan-miR-363-3p_R+1	up
PLAU	down	gga-miR-106-3p_R+1	up
PLAU	down	gga-miR-1559-3p	up
PLAU	down	gga-miR-301a-5p_L+2	up
PLAU	down	gga-miR-458a-3p	down
PLAU	down	rno-miR-122-5p_L+1R+1_1	down
PLAU	down	rno-miR-122-5p_L+1R+1_2	down
VCAM1	down	gga-miR-181b-5p_R+1	up
CYB5R2	up	gga-miR-130b-5p_R-1	up
PPIC	up	aca-miR-363-3p_R+1	up
PPIC	up	oan-miR-363-3p_R+1	up
PDLIM1	down	gga-miR-301a-5p_L+2	up
ARHGEF3	down	cin-miR-33_R+4	down
ARHGEF3	down	gga-miR-130b-5p_R-1	up
ARHGEF3	down	gga-miR-181b-5p_R+1	up
ARHGEF3	down	gga-miR-20b-5p	up
ARHGEF3	down	gga-miR-6710-3p_R+1	up
ST6GAL1	down	aca-miR-363-3p_R+1	up
ST6GAL1	down	gga-miR-20b-5p	up
ST6GAL1	down	oan-miR-363-3p_R+1	up
TBL3	up	gga-let-7j-3p_L-1R+2	down

GARS	up	gga-miR-106-3p_R+1	up
ITGB1BP3	up	gga-miR-1451-3p_R-1	up
XBP1	down	aca-miR-363-3p_R+1	up
XBP1	down	cin-miR-33_R+4	down
XBP1	down	gga-miR-456-3p_1ss22AT	up
XBP1	down	gga-miR-456-3p_R-1	up
XBP1	down	oan-miR-363-3p_R+1	up
XBP1	down	pma-miR-456_R+1_1ss22AC	up
TMEM41B	down	PC-3p-68190_58	up
TMEM41B	down	gga-miR-1559-3p	up
MMP11	up	gga-miR-107-3p_R-1	up
MMP11	up	gga-miR-125b-5p	down
RRAS2	down	gga-let-7j-3p_L-1R+2	down
RRAS2	down	gga-miR-107-3p_R-1	up
RRAS2	down	gga-miR-1729-5p_R-1	down
RRAS2	down	gga-miR-301a-5p_L+2	up
MCM2	up	aca-miR-18a-3p_L+1R-1_1ss12GA	up
MCM2	up	gga-miR-125b-5p	down
MCM2	up	gga-miR-1729-5p_R-1	down
MCM2	up	gga-miR-3529	up
NDUFAB1	up	gga-miR-107-3p_R-1	up
NDUFAB1	up	gga-miR-489-3p_L-2R+1	up
MOSPD1	down	PC-3p-68190_58	up
MOSPD1	down	gga-miR-181b-5p_R+1	up
MOSPD1	down	gga-miR-20b-5p	up
DDT	up	gga-miR-6710-3p_R+1	up

IL16	down	gga-let-7j-3p_L-1R+2	down
PANK1	down	gga-miR-107-3p_R-1	up
C13H5ORF15	down	gga-miR-107-3p_R-1	up
CRTAM	down	gga-miR-301a-5p_L+2	up
TSSC4	up	gga-miR-107-3p_R-1	up
SEMA3A	up	gga-miR-181b-5p_R+1	up
ABCC1	down	gga-miR-107-3p_R-1	up
ABCC1	down	gga-miR-301a-5p_L+2	up
KIF11	up	gga-let-7j-3p_L-1R+2	down
KIF11	up	gga-miR-106-3p_R+1	up
KIF11	up	gga-miR-6710-3p_R+1	up
TLR4	down	PC-3p-68190_58	up
TLR4	down	gga-miR-106-3p_R+1	up
ARRDC4	down	gga-miR-181b-5p_R+1	up
CREM	down	gga-miR-130b-5p_R-1	up
CREM	down	gga-miR-20b-5p	up
OPA1	up	gga-miR-489-3p_L-2R+1	up
TK1	up	gga-miR-107-3p_R-1	up
ITGB2	down	gga-let-7b_R+2	down
ITGB2	down	mmu-let-7j_1ss8TG	down
RRP12	up	gga-miR-456-3p_1ss22AT	up
RRP12	up	gga-miR-456-3p_R-1	up
RRP12	up	pma-miR-456_R+1_1ss22AC	up
EME1	up	gga-miR-181b-5p_R+1	up
TRAP1	up	aca-miR-18a-3p_L+1R-1_1ss12GA	up
TRAP1	up	gga-miR-107-3p_R-1	up

NPM3	up	gga-miR-146a-3p_R-1	down
SALL4	up	gga-miR-181b-5p_R+1	up
SALL4	up	gga-miR-3529	up
CD44	down	gga-miR-1451-3p_R-1	up
CD44	down	gga-miR-3529	up
CD44	down	gga-miR-489-3p_L-2R+1	up
FANCL	up	gga-miR-106-3p_R+1	up
STK17B	down	aca-miR-363-5p	up
STK17B	down	gga-miR-20b-5p	up
ZC3HC1	up	gga-miR-202-5p_L-1	up
CD82	down	gga-miR-456-3p_1ss22AT	up
CD82	down	gga-miR-456-3p_R-1	up
CD82	down	pma-miR-456_R+1_1ss22AC	up
COQ10B	down	aca-miR-363-3p_R+1	up
COQ10B	down	oan-miR-363-3p_R+1	up
CHRDL1	down	gga-miR-106-3p_R+1	up
CHRDL1	down	gga-miR-107-3p_R-1	up
CHRDL1	down	gga-miR-130b-5p_R-1	up
CHRDL1	down	gga-miR-146a-3p_R-1	down
CHRDL1	down	gga-miR-489-3p_L-2R+1	up
NDUFS3	up	gga-miR-107-3p_R-1	up
KIF23	up	gga-miR-107-3p_R-1	up
KIF23	up	gga-miR-20b-5p	up
RINT1	down	gga-miR-3529	up
GRAMD3	down	gga-miR-106-3p_R+1	up
NAPEPLD	down	aca-miR-363-5p	up

NAPEPLD	down	gga-miR-20b-5p	up
FGL2	down	aca-miR-363-3p_R+1	up
FGL2	down	cin-miR-33_R+4	down
FGL2	down	gga-miR-6710-3p_R+1	up
FGL2	down	oan-miR-363-3p_R+1	up
GSTO1	down	gga-miR-489-3p_L-2R+1	up
NET1	down	aca-miR-363-5p	up
NET1	down	gga-let-7j-3p_L-1R+2	down
NET1	down	rno-miR-122-5p_L+1R+1_1	down
NET1	down	rno-miR-122-5p_L+1R+1_2	down
SLC38A2	down	gga-miR-106-3p_R+1	up
ABHD12	up	gga-miR-107-3p_R-1	up
ABHD12	up	gga-miR-146a-3p_R-1	down
ABHD12	up	gga-miR-20b-5p	up
ABHD12	up	gga-miR-3529	up
PEX6	up	aca-miR-18a-3p_L+1R-1_1ss12GA	up
ITGA8	down	rno-miR-122-5p_L+1R+1_1	down
ITGA8	down	rno-miR-122-5p_L+1R+1_2	down
UGP2	down	PC-3p-68190_58	up
UGP2	down	gga-miR-106-3p_R+1	up
FXR1	down	aca-miR-363-3p_R+1	up
FXR1	down	gga-miR-1559-3p	up
FXR1	down	oan-miR-363-3p_R+1	up
JAG1	down	gga-miR-301a-5p_L+2	up
GANC	up	gga-miR-3529	up
YPEL5	down	gga-miR-489-3p_L-2R+1	up

ENTPD2	down	gga-miR-125b-5p	down
ENTPD2	down	gga-miR-1729-5p_R-1	down
ENTPD2	down	gga-miR-3529	up
FSHR	down	gga-miR-106-3p_R+1	up
DEGS1	down	cin-miR-33_R+4	down
SKIL	down	aca-miR-18a-3p_L+1R-1_1ss12GA	up
SMOC1	up	gga-let-7j-3p_L-1R+2	down
SMOC1	up	gga-miR-130b-5p_R-1	up
SMOC1	up	gga-miR-20b-5p	up
BAG3	down	aca-miR-363-5p	up
BAG3	down	gga-miR-1559-3p	up
BAG3	down	gga-miR-20b-5p	up
IFRD1	up	gga-let-7j-3p_L-1R+2	down
DUSP10	down	aca-miR-18a-3p_L+1R-1_1ss12GA	up
DUSP10	down	aca-miR-363-3p_R+1	up
DUSP10	down	oan-miR-363-3p_R+1	up
FNIP2	up	gga-miR-6710-3p_R+1	up
MSMO1	down	aca-miR-363-3p_R+1	up
MSMO1	down	gga-miR-106-3p_R+1	up
MSMO1	down	oan-miR-363-3p_R+1	up
RARRES1	down	gga-miR-181b-5p_R+1	up
THBS1	down	gga-miR-3529	up
GFM1	up	gga-miR-107-3p_R-1	up
GFM1	up	gga-miR-6710-3p_R+1	up
COL1A2	up	aca-miR-363-3p_R+1	up
COL1A2	up	gga-let-7b_R+2	down

COL1A2	up	gga-miR-130b-5p_R-1	up
COL1A2	up	gga-miR-1451-3p_R-1	up
COL1A2	up	mmu-let-7j_1ss8TG	down
COL1A2	up	oan-miR-363-3p_R+1	up
ACADSB	down	aca-miR-18a-3p_L+1R-1_1ss12GA	up
ACADSB	down	gga-let-7b_R+2	down
ACADSB	down	gga-miR-301a-5p_L+2	up
ACADSB	down	gga-miR-3529	up
ACADSB	down	mmu-let-7j_1ss8TG	down
DTL	up	rno-miR-122-5p_L+1R+1_1	down
DTL	up	rno-miR-122-5p_L+1R+1_2	down
CFL2	up	gga-miR-181b-5p_R+1	up
CFL2	up	gga-miR-20b-5p	up
PLK3	down	gga-miR-146a-3p_R-1	down
NFKBIE	down	gga-miR-456-3p_1ss22AT	up
NFKBIE	down	gga-miR-456-3p_R-1	up
NFKBIE	down	pma-miR-456_R+1_1ss22AC	up
PRDX1	up	cin-miR-33_R+4	down
PRDX1	up	gga-miR-130b-5p_R-1	up
TSPAN1	up	cin-miR-33_R+4	down
CMPK1	up	gga-miR-106-3p_R+1	up
CMPK1	up	gga-miR-202-5p_L-1	up
CMPK1	up	gga-miR-20b-5p	up
CMPK1	up	gga-miR-301a-5p_L+2	up
SLC25A4	up	gga-miR-106-3p_R+1	up
SLC25A4	up	gga-miR-20b-5p	up

ORC1	up	gga-miR-20b-5p	up
MEMO1	up	gga-miR-456-3p_1ss22AT	up
MEMO1	up	gga-miR-456-3p_R-1	up
MEMO1	up	pma-miR-456_R+1_1ss22AC	up
GLCCI1	down	gga-miR-6710-3p_R+1	up
NEIL3	up	gga-miR-107-3p_R-1	up
SCIN	down	gga-miR-107-3p_R-1	up
SCIN	down	gga-miR-125b-5p	down
SCIN	down	gga-miR-130b-5p_R-1	up
SCIN	down	gga-miR-181b-5p_R+1	up
SCIN	down	gga-miR-301a-5p_L+2	up
SCIN	down	gga-miR-456-3p_1ss22AT	up
SCIN	down	gga-miR-456-3p_R-1	up
SCIN	down	gga-miR-489-3p_L-2R+1	up
SCIN	down	pma-miR-456_R+1_1ss22AC	up
SLC24A4	down	gga-miR-20b-5p	up
DHCR24	down	aca-miR-18a-3p_L+1R-1_1ss12GA	up
DHCR24	down	gga-miR-130b-5p_R-1	up
DHCR24	down	gga-miR-202-5p_L-1	up
DHCR24	down	gga-miR-458a-3p	down
DHCR24	down	gga-miR-6710-3p_R+1	up
EREG	down	aca-miR-363-5p	up
EREG	down	gga-miR-130b-5p_R-1	up
HOOK1	up	gga-miR-301a-5p_L+2	up
FAM126A	down	gga-miR-106-3p_R+1	up
KANK4	up	gga-miR-456-3p_1ss22AT	up

KANK4	up	gga-miR-456-3p_R-1	up
KANK4	up	pma-miR-456_R+1_1ss22AC	up
IGF2BP3	up	gga-let-7b_R+2	down
IGF2BP3	up	gga-miR-107-3p_R-1	up
IGF2BP3	up	gga-miR-1729-5p_R-1	down
IGF2BP3	up	mmu-let-7j_1ss8TG	down
SIPA1L2	down	gga-miR-130b-5p_R-1	up
HIBADH	up	gga-miR-106-3p_R+1	up
HHIPL1	down	gga-miR-489-3p_L-2R+1	up
PLCL2	down	gga-miR-202-5p_L-1	up
KAT2B	down	gga-miR-181b-5p_R+1	up
KAT2B	down	gga-miR-20b-5p	up
KAT2B	down	gga-miR-456-3p_1ss22AT	up
KAT2B	down	gga-miR-456-3p_R-1	up
KAT2B	down	pma-miR-456_R+1_1ss22AC	up
BAG5	down	aca-miR-191-5p_R-1	down
BAG5	down	gga-miR-1559-3p	up
BAG5	down	gga-miR-181b-5p_R+1	up
BAG5	down	gga-miR-458a-3p	down
UHRF1BP1L	up	PC-3p-68190_58	up
UHRF1BP1L	up	cin-miR-33_R+4	down
LARS2	up	gga-miR-130b-5p_R-1	up
CLEC3B	down	PC-3p-68190_58	up
ACO2	up	aca-miR-363-3p_R+1	up
ACO2	up	gga-miR-1559-3p	up
ACO2	up	oan-miR-363-3p_R+1	up

CCR8	down	aca-miR-363-3p_R+1	up
CCR8	down	oan-miR-363-3p_R+1	up
CCR8	down	rno-miR-122-5p_L+1R+1_1	down
CCR8	down	rno-miR-122-5p_L+1R+1_2	down
MAD2L1	up	gga-let-7j-3p_L-1R+2	down
MAD2L1	up	gga-miR-1559-3p	up
MAD2L1	up	gga-miR-301a-5p_L+2	up
CAMK2D	down	gga-miR-128-1-5p	up
CAMK2D	down	gga-miR-1559-3p	up
CAMK2D	down	gga-miR-181b-5p_R+1	up
DTX3L	down	gga-miR-1559-3p	up
DTX3L	down	gga-miR-20b-5p	up
ENPEP	down	gga-miR-125b-5p	down
RAB32	down	gga-miR-107-3p_R-1	up
ISCA1	down	PC-3p-68190_58	up
ISCA1	down	aca-miR-363-3p_R+1	up
ISCA1	down	gga-let-7j-3p_L-1R+2	down
ISCA1	down	gga-miR-202-5p_L-1	up
ISCA1	down	gga-miR-458a-3p	down
ISCA1	down	oan-miR-363-3p_R+1	up
ISCA1	down	rno-miR-122-5p_L+1R+1_1	down
ISCA1	down	rno-miR-122-5p_L+1R+1_2	down
CTSL2	down	gga-miR-181b-5p_R+1	up
PWP1	up	gga-miR-106-3p_R+1	up
PWP1	up	gga-miR-130b-5p_R-1	up
PWP1	up	gga-miR-301a-5p_L+2	up

PWP1	up	gga-miR-456-3p_1ss22AT	up
PWP1	up	gga-miR-456-3p_R-1	up
PWP1	up	pma-miR-456_R+1_1ss22AC	up
RBM24	down	gga-let-7j-3p_L-1R+2	down
RBM24	down	gga-miR-106-3p_R+1	up
RBM24	down	gga-miR-107-3p_R-1	up
RBM24	down	gga-miR-1559-3p	up
RBM24	down	gga-miR-1729-5p_R-1	down
SSR1	up	gga-miR-6710-3p_R+1	up
AKR1D1	up	gga-let-7b_R+2	down
AKR1D1	up	mmu-let-7j_1ss8TG	down
SERPINB6	down	gga-miR-1451-3p_R-1	up
SERPINB10	down	gga-let-7b_R+2	down
SERPINB10	down	mmu-let-7j_1ss8TG	down
MCM4	up	gga-miR-107-3p_R-1	up
DENND5B	up	gga-miR-146a-3p_R-1	down
BPGM	up	aca-miR-18a-3p_L+1R-1_1ss12GA	up
BPGM	up	cin-miR-33_R+4	down
BPGM	up	gga-miR-107-3p_R-1	up
CCDC127	down	aca-miR-363-3p_R+1	up
CCDC127	down	gga-miR-106-3p_R+1	up
CCDC127	down	gga-miR-456-3p_1ss22AT	up
CCDC127	down	gga-miR-456-3p_R-1	up
CCDC127	down	oan-miR-363-3p_R+1	up
CCDC127	down	pma-miR-456_R+1_1ss22AC	up
PYROXD1	up	aca-miR-363-3p_R+1	up

PYROXD1	up	oan-miR-363-3p_R+1	up
GABBR2	down	gga-let-7j-3p_L-1R+2	down
RHBG	down	gga-miR-107-3p_R-1	up
RHBG	down	gga-miR-1729-5p_R-1	down
PLEKHO1	down	gga-miR-125b-5p	down
MED24	up	gga-miR-301a-5p_L+2	up
ANKRA2	down	gga-miR-489-3p_L-2R+1	up
MTHFD2	up	aca-miR-363-3p_R+1	up
MTHFD2	up	oan-miR-363-3p_R+1	up
CYB5A	up	gga-miR-146a-3p_R-1	down
CYB5A	up	gga-miR-20b-5p	up
IMPA2	up	gga-miR-1559-3p	up
SYTL2	down	gga-miR-20b-5p	up
TEC	down	gga-let-7j-3p_L-1R+2	down
TEC	down	gga-miR-107-3p_R-1	up
TEC	down	gga-miR-6710-3p_R+1	up
TAPBPL	down	gga-miR-146a-3p_R-1	down
TAPBPL	down	gga-miR-1729-5p_R-1	down
CD38	down	gga-miR-107-3p_R-1	up
LPCAT3	up	rno-miR-122-5p_L+1R+1_1	down
LPCAT3	up	rno-miR-122-5p_L+1R+1_2	down
MBLAC2	down	aca-miR-18a-3p_L+1R-1_1ss12GA	up
MBLAC2	down	gga-miR-130b-5p_R-1	up
MBLAC2	down	gga-miR-181b-5p_R+1	up
RFESD	down	gga-miR-1559-3p	up
DEPDC1B	up	gga-miR-1559-3p	up

C7	down	aca-miR-363-5p	up
C7	down	gga-miR-146a-3p_R-1	down
PARP8	down	PC-3p-68190_58	up
WDR1	down	gga-let-7j-3p_L-1R+2	down
WDR1	down	gga-miR-106-3p_R+1	up
F2RL1	down	gga-miR-107-3p_R-1	up
F2RL1	down	gga-miR-125b-5p	down
F2RL1	down	gga-miR-1559-3p	up
F2RL1	down	gga-miR-6710-3p_R+1	up
ABHD17B	down	gga-let-7j-3p_L-1R+2	down
ABHD17B	down	gga-miR-106-3p_R+1	up
PCMTD1	down	cin-miR-33_R+4	down
PCMTD1	down	gga-miR-107-3p_R-1	up
PCMTD1	down	gga-miR-130b-5p_R-1	up
PCMTD1	down	gga-miR-1559-3p	up
PCMTD1	down	gga-miR-20b-5p	up
TMEM68	down	aca-miR-363-5p	up
TMEM68	down	gga-let-7j-3p_L-1R+2	down
TMEM68	down	gga-miR-181b-5p_R+1	up
ALCAM	down	gga-miR-1729-5p_R-1	down
KIAA1524	up	gga-miR-181b-5p_R+1	up
KIAA1524	up	gga-miR-20b-5p	up
CYP7A1	up	gga-miR-107-3p_R-1	up
CYP7A1	up	gga-miR-1559-3p	up
CYP7A1	up	gga-miR-489-3p_L-2R+1	up
PPP2R2C	up	PC-3p-68190_58	up

PPP2R2C	up	gga-miR-456-3p_1ss22AT	up
PPP2R2C	up	gga-miR-456-3p_R-1	up
PPP2R2C	up	pma-miR-456_R+1_1ss22AC	up
SLBP	up	gga-miR-1451-3p_R-1	up
FABP5	down	aca-miR-363-3p_R+1	up
FABP5	down	gga-miR-1559-3p	up
FABP5	down	gga-miR-20b-5p	up
FABP5	down	gga-miR-456-3p_1ss22AT	up
FABP5	down	gga-miR-456-3p_R-1	up
FABP5	down	oan-miR-363-3p_R+1	up
FABP5	down	pma-miR-456_R+1_1ss22AC	up
FABP5	down	rno-miR-122-5p_L+1R+1_1	down
FABP5	down	rno-miR-122-5p_L+1R+1_2	down
UBE2J1	down	gga-miR-1729-5p_R-1	down
UBE2J1	down	gga-miR-458a-3p	down
IMMT	up	aca-miR-363-5p	up
IFNAR1	down	gga-miR-106-3p_R+1	up
IFNAR1	down	gga-miR-20b-5p	up
CDH17	down	gga-miR-106-3p_R+1	up
TP53INP1	down	aca-miR-18a-3p_L+1R-1_1ss12GA	up
TP53INP1	down	gga-miR-20b-5p	up
TP53INP1	down	rno-miR-122-5p_L+1R+1_1	down
TP53INP1	down	rno-miR-122-5p_L+1R+1_2	down
MORC3	down	aca-miR-18a-3p_L+1R-1_1ss12GA	up
MORC3	down	aca-miR-191-5p_R-1	down
MORC3	down	cin-miR-33_R+4	down

MORC3	down	gga-miR-146a-3p_R-1	down
MORC3	down	gga-miR-6710-3p_R+1	up
RAD21	up	aca-miR-363-3p_R+1	up
RAD21	up	gga-miR-106-3p_R+1	up
RAD21	up	gga-miR-181b-5p_R+1	up
RAD21	up	gga-miR-458a-3p	down
RAD21	up	oan-miR-363-3p_R+1	up
SIK1	down	aca-miR-363-3p_R+1	up
SIK1	down	cin-miR-33_R+4	down
SIK1	down	gga-miR-130b-5p_R-1	up
SIK1	down	gga-miR-20b-5p	up
SIK1	down	oan-miR-363-3p_R+1	up
MAOA	down	aca-miR-363-5p	up
TINAG	up	gga-let-7j-3p_L-1R+2	down
TINAG	up	gga-miR-107-3p_R-1	up
FBXO9	down	aca-miR-18a-3p_L+1R-1_1ss12GA	up
FBXO9	down	gga-miR-107-3p_R-1	up
FBXO9	down	gga-miR-130b-5p_R-1	up
FBXO9	down	gga-miR-1451-3p_R-1	up
FBXO9	down	gga-miR-146a-3p_R-1	down
FBXO9	down	gga-miR-202-5p_L-1	up
FBXO9	down	gga-miR-458a-3p	down
DSCC1	up	gga-miR-181b-5p_R+1	up
PDHA1	up	gga-miR-181b-5p_R+1	up
KRTCAP3	up	gga-miR-456-3p_1ss22AT	up
KRTCAP3	up	gga-miR-456-3p_R-1	up

KRTCAP3	up	pma-miR-456_R+1_1ss22AC	up
REPS2	up	gga-let-7j-3p_L-1R+2	down
REPS2	up	gga-miR-456-3p_1ss22AT	up
REPS2	up	gga-miR-456-3p_R-1	up
REPS2	up	pma-miR-456_R+1_1ss22AC	up
FDFT1	down	aca-miR-18a-3p_L+1R-1_1ss12GA	up
FDFT1	down	gga-miR-1729-5p_R-1	down
MCM3	up	gga-miR-20b-5p	up
RGN	down	PC-3p-68190_58	up
SLC9A7	down	gga-miR-181b-5p_R+1	up
CHST10	down	PC-3p-68190_58	up
CHST10	down	aca-miR-18a-3p_L+1R-1_1ss12GA	up
CHST10	down	gga-miR-106-3p_R+1	up
RASA3	down	gga-miR-20b-5p	up
GAS6	down	gga-miR-181b-5p_R+1	up
IRG1	down	gga-miR-107-3p_R-1	up
IRG1	down	gga-miR-202-5p_L-1	up
IRG1	down	rno-miR-122-5p_L+1R+1_1	down
IRG1	down	rno-miR-122-5p_L+1R+1_2	down
LCP1	down	gga-miR-130b-5p_R-1	up
SUCLA2	down	gga-miR-106-3p_R+1	up
RCBTB1	up	cin-miR-33_R+4	down
RCBTB1	up	gga-miR-1559-3p	up
CKAP2	up	aca-miR-191-5p_R-1	down
CKAP2	up	gga-miR-106-3p_R+1	up
SLC46A3	down	gga-miR-107-3p_R-1	up

SLC46A3	down	gga-miR-20b-5p	up
SLC46A3	down	gga-miR-456-3p_1ss22AT	up
SLC46A3	down	gga-miR-456-3p_R-1	up
SLC46A3	down	pma-miR-456_R+1_1ss22AC	up
SLC46A3	down	rno-miR-122-5p_L+1R+1_1	down
SLC46A3	down	rno-miR-122-5p_L+1R+1_2	down
ARL6IP5	down	gga-miR-1559-3p	up
ARL6IP5	down	rno-miR-122-5p_L+1R+1_1	down
ARL6IP5	down	rno-miR-122-5p_L+1R+1_2	down
COTL1	down	PC-3p-68190_58	up
GZMA	down	gga-miR-458a-3p	down
LYN	down	cin-miR-33_R+4	down
LYN	down	gga-miR-106-3p_R+1	up
LYN	down	gga-miR-130b-5p_R-1	up
DPT	down	PC-3p-68190_58	up
DPT	down	rno-miR-122-5p_L+1R+1_1	down
DPT	down	rno-miR-122-5p_L+1R+1_2	down
PACSIN2	down	cin-miR-33_R+4	down
PACSIN2	down	gga-miR-181b-5p_R+1	up
HIGD1A	up	gga-miR-146a-3p_R-1	down
HIGD1A	up	gga-miR-1559-3p	up
HIGD1A	up	gga-miR-456-3p_1ss22AT	up
HIGD1A	up	gga-miR-456-3p_R-1	up
HIGD1A	up	gga-miR-489-3p_L-2R+1	up
HIGD1A	up	pma-miR-456_R+1_1ss22AC	up
MICAL1	down	gga-miR-107-3p_R-1	up

GAL7	down	rno-miR-122-5p_L+1R+1_1	down
GAL7	down	rno-miR-122-5p_L+1R+1_2	down
C3H2ORF43	down	aca-miR-363-5p	up
C3H2ORF43	down	gga-miR-1451-3p_R-1	up
MCPH1	up	gga-miR-202-5p_L-1	up
IFNGR1	down	gga-miR-1559-3p	up
IFNGR1	down	gga-miR-181b-5p_R+1	up
SUCLG1	up	aca-miR-363-5p	up
SUCLG1	up	gga-miR-1451-3p_R-1	up
SUCLG1	up	gga-miR-489-3p_L-2R+1	up
SUCLG1	up	rno-miR-122-5p_L+1R+1_1	down
SUCLG1	up	rno-miR-122-5p_L+1R+1_2	down
CHP1	up	gga-miR-106-3p_R+1	up
INCENP	up	aca-miR-18a-3p_L+1R-1_1ss12GA	up
INCENP	up	gga-miR-106-3p_R+1	up
INCENP	up	gga-miR-1451-3p_R-1	up
INCENP	up	gga-miR-1559-3p	up
INCENP	up	gga-miR-489-3p_L-2R+1	up
SYK	down	gga-miR-458a-3p	down
MCM6	up	rno-miR-122-5p_L+1R+1_1	down
MCM6	up	rno-miR-122-5p_L+1R+1_2	down
HABP2	down	gga-let-7b_R+2	down
HABP2	down	mmu-let-7j_1ss8TG	down
ARHGAP19	up	gga-miR-106-3p_R+1	up
ARHGAP19	up	gga-miR-107-3p_R-1	up
ARHGAP19	up	gga-miR-128-1-5p	up

RSFR	down	gga-miR-107-3p_R-1	up
RGS1	down	gga-miR-458a-3p	down
MPZL3	down	gga-miR-130b-5p_R-1	up
NFYC	up	gga-miR-130b-5p_R-1	up
NFYC	up	gga-miR-458a-3p	down
IFI27L2	down	gga-miR-106-3p_R+1	up
BIRC2	down	gga-miR-6710-3p_R+1	up
PDLIM5	down	aca-miR-363-5p	up
PDLIM5	down	cin-miR-33_R+4	down
PDLIM5	down	gga-miR-146a-3p_R-1	down
PDLIM5	down	gga-miR-20b-5p	up
PDLIM5	down	gga-miR-458a-3p	down
RNASET2	down	gga-miR-106-3p_R+1	up
RNASET2	down	gga-miR-130b-5p_R-1	up
RNASET2	down	gga-miR-6710-3p_R+1	up
LPL	up	gga-miR-458a-3p	down
REEP5	down	cin-miR-33_R+4	down
REEP5	down	gga-miR-1559-3p	up
HOPX	down	gga-miR-181b-5p_R+1	up
HOPX	down	gga-miR-489-3p_L-2R+1	up
GLDC	up	gga-miR-106-3p_R+1	up
RAD54L	up	aca-miR-18a-3p_L+1R-1_1ss12GA	up
WIPF1	down	cin-miR-33_R+4	down
WIPF1	down	gga-let-7j-3p_L-1R+2	down
WIPF1	down	gga-miR-106-3p_R+1	up
WIPF1	down	gga-miR-107-3p_R-1	up

WIPF1	down	gga-miR-130b-5p_R-1	up
GATM	up	gga-let-7b_R+2	down
GATM	up	gga-miR-181b-5p_R+1	up
GATM	up	gga-miR-489-3p_L-2R+1	up
GATM	up	mmu-let-7j_1ss8TG	down
DOCK4	down	gga-miR-20b-5p	up
CD151	down	PC-3p-68190_58	up
CD151	down	gga-miR-130b-5p_R-1	up
ARPP19	up	cin-miR-33_R+4	down
ARPP19	up	gga-miR-1729-5p_R-1	down
LSP1	down	cin-miR-33_R+4	down
LSP1	down	gga-miR-107-3p_R-1	up
LDHA	down	gga-miR-106-3p_R+1	up
LDHA	down	gga-miR-20b-5p	up
NBL1	down	gga-miR-106-3p_R+1	up
NBL1	down	gga-miR-20b-5p	up
CYP2H1	down	aca-miR-363-3p_R+1	up
CYP2H1	down	gga-miR-146a-3p_R-1	down
CYP2H1	down	gga-miR-456-3p_1ss22AT	up
CYP2H1	down	gga-miR-456-3p_R-1	up
CYP2H1	down	oan-miR-363-3p_R+1	up
CYP2H1	down	pma-miR-456_R+1_1ss22AC	up
REL	down	gga-miR-106-3p_R+1	up
REL	down	gga-miR-130b-5p_R-1	up
REL	down	gga-miR-1729-5p_R-1	down
LIMD2	down	gga-miR-301a-5p_L+2	up

LIMK1	up	gga-miR-128-1-5p	up
ARHGEF10L	up	gga-let-7b_R+2	down
ARHGEF10L	up	mmu-let-7j_1ss8TG	down
ANG	down	PC-3p-68190_58	up
ANG	down	gga-miR-107-3p_R-1	up
SLU7	down	cin-miR-33_R+4	down
PFKL	up	gga-miR-128-1-5p	up
HSBP1L1	down	gga-miR-125b-5p	down

Table S14. Intersection target differentially expressed genes of 32 differentially expressed miRNAs (which were selected with the criteria of FDR < 0.05 and fold changes either > 1.5 or < 0.67.) in mRNA levels.

Symbol	gene regulation(immature VS mature liver)	miRNA ID	miRNA regulation(immature VS mature liver)
ARHGAP25	down	gga-miR-107-3p_R-1	up
ARHGAP25	down	gga-miR-130b-5p_R-1	up
RASSF2	down	gga-miR-130b-5p_R-1	up
RASSF2	down	gga-miR-181b-5p_R+1	up
RASSF2	down	gga-miR-489-3p_L-2R+1	up
CTSS	down	gga-miR-106-3p_R+1	up
TAT	down	aca-miR-18a-3p_L+1R-1_1ss12GA	up
FAM18B1	down	gga-miR-106-3p_R+1	up
FAM18B1	down	gga-miR-456-3p_1ss22AT	up
FAM18B1	down	gga-miR-456-3p_R-1	up
FAM18B1	down	pma-miR-456_R+1_1ss22AC	up
GSN	down	aca-miR-363-3p_R+1	up
GSN	down	oan-miR-363-3p_R+1	up
MYO1F	down	gga-miR-106-3p_R+1	up
MYO1F	down	gga-miR-107-3p_R-1	up
ACSBG2	down	aca-miR-363-3p_R+1	up
ACSBG2	down	gga-miR-106-3p_R+1	up
ACSBG2	down	gga-miR-181b-5p_R+1	up
ACSBG2	down	oan-miR-363-3p_R+1	up

TNFRSF18	down	PC-3p-68190_58	up
VAV3	down	gga-miR-107-3p_R-1	up
VAV3	down	gga-miR-489-3p_L-2R+1	up
VAV3	down	gga-miR-6710-3p_R+1	up
RFFL	down	gga-miR-106-3p_R+1	up
RFFL	down	gga-miR-301a-5p_L+2	up
DMXL1	down	gga-miR-1559-3p	up
AP3S1	down	gga-miR-107-3p_R-1	up
AP3S1	down	gga-miR-1451-3p_R-1	up
SPSB1	down	aca-miR-18a-3p_L+1R-1_1ss12GA	up
RGS2	down	gga-miR-107-3p_R-1	up
DEF6	down	gga-miR-107-3p_R-1	up
ANXA11	down	gga-miR-202-5p_L-1	up
SBNO2	down	gga-miR-107-3p_R-1	up
SLC15A4	down	gga-miR-107-3p_R-1	up
DNAJC12	down	gga-miR-106-3p_R+1	up
DNAJC12	down	gga-miR-1559-3p	up
DNAJC12	down	gga-miR-181b-5p_R+1	up
DNAJA4	down	gga-miR-1559-3p	up
GBGT1	down	aca-miR-363-5p	up
GBGT1	down	gga-miR-489-3p_L-2R+1	up
SLC6A2	down	gga-miR-130b-5p_R-1	up
LPCAT2	down	gga-miR-181b-5p_R+1	up
LPCAT2	down	gga-miR-20b-5p	up
TIMD4	down	gga-miR-107-3p_R-1	up
ADA	down	gga-miR-202-5p_L-1	up

ADA	down	gga-miR-301a-5p_L+2	up
RGS9BP	down	gga-miR-1559-3p	up
RGS9BP	down	gga-miR-202-5p_L-1	up
RGS9BP	down	gga-miR-3529	up
RGS9BP	down	gga-miR-6710-3p_R+1	up
RGL1	down	aca-miR-363-3p_R+1	up
RGL1	down	gga-miR-181b-5p_R+1	up
RGL1	down	oan-miR-363-3p_R+1	up
PLAU	down	gga-miR-106-3p_R+1	up
PLAU	down	gga-miR-1559-3p	up
PLAU	down	gga-miR-301a-5p_L+2	up
VCAM1	down	gga-miR-181b-5p_R+1	up
PDLIM1	down	gga-miR-301a-5p_L+2	up
ARHGEF3	down	gga-miR-130b-5p_R-1	up
ARHGEF3	down	gga-miR-181b-5p_R+1	up
ARHGEF3	down	gga-miR-20b-5p	up
ARHGEF3	down	gga-miR-6710-3p_R+1	up
ST6GAL1	down	aca-miR-363-3p_R+1	up
ST6GAL1	down	gga-miR-20b-5p	up
ST6GAL1	down	oan-miR-363-3p_R+1	up
XBP1	down	aca-miR-363-3p_R+1	up
XBP1	down	gga-miR-456-3p_1ss22AT	up
XBP1	down	gga-miR-456-3p_R-1	up
XBP1	down	oan-miR-363-3p_R+1	up
XBP1	down	pma-miR-456_R+1_1ss22AC	up
TMEM41B	down	PC-3p-68190_58	up

TMEM41B	down	gga-miR-1559-3p	up
RRAS2	down	gga-miR-107-3p_R-1	up
RRAS2	down	gga-miR-301a-5p_L+2	up
MOSPD1	down	PC-3p-68190_58	up
MOSPD1	down	gga-miR-181b-5p_R+1	up
MOSPD1	down	gga-miR-20b-5p	up
PANK1	down	gga-miR-107-3p_R-1	up
C13H5ORF15	down	gga-miR-107-3p_R-1	up
CRTAM	down	gga-miR-301a-5p_L+2	up
ABCC1	down	gga-miR-107-3p_R-1	up
ABCC1	down	gga-miR-301a-5p_L+2	up
TLR4	down	PC-3p-68190_58	up
TLR4	down	gga-miR-106-3p_R+1	up
ARRDC4	down	gga-miR-181b-5p_R+1	up
CREM	down	gga-miR-130b-5p_R-1	up
CREM	down	gga-miR-20b-5p	up
CD44	down	gga-miR-1451-3p_R-1	up
CD44	down	gga-miR-3529	up
CD44	down	gga-miR-489-3p_L-2R+1	up
STK17B	down	aca-miR-363-5p	up
STK17B	down	gga-miR-20b-5p	up
CD82	down	gga-miR-456-3p_1ss22AT	up
CD82	down	gga-miR-456-3p_R-1	up
CD82	down	pma-miR-456_R+1_1ss22AC	up
COQ10B	down	aca-miR-363-3p_R+1	up
COQ10B	down	oan-miR-363-3p_R+1	up

CHRDL1	down	gga-miR-106-3p_R+1	up
CHRDL1	down	gga-miR-107-3p_R-1	up
CHRDL1	down	gga-miR-130b-5p_R-1	up
CHRDL1	down	gga-miR-489-3p_L-2R+1	up
RINT1	down	gga-miR-3529	up
GRAMD3	down	gga-miR-106-3p_R+1	up
NAPEPLD	down	aca-miR-363-5p	up
NAPEPLD	down	gga-miR-20b-5p	up
FGL2	down	aca-miR-363-3p_R+1	up
FGL2	down	gga-miR-6710-3p_R+1	up
FGL2	down	oan-miR-363-3p_R+1	up
GSTO1	down	gga-miR-489-3p_L-2R+1	up
NET1	down	aca-miR-363-5p	up
SLC38A2	down	gga-miR-106-3p_R+1	up
UGP2	down	PC-3p-68190_58	up
UGP2	down	gga-miR-106-3p_R+1	up
FXR1	down	aca-miR-363-3p_R+1	up
FXR1	down	gga-miR-1559-3p	up
FXR1	down	oan-miR-363-3p_R+1	up
JAG1	down	gga-miR-301a-5p_L+2	up
YPEL5	down	gga-miR-489-3p_L-2R+1	up
ENTPD2	down	gga-miR-3529	up
FSHR	down	gga-miR-106-3p_R+1	up
SKIL	down	aca-miR-18a-3p_L+1R-1_1ss12GA	up
BAG3	down	aca-miR-363-5p	up
BAG3	down	gga-miR-1559-3p	up

BAG3	down	gga-miR-20b-5p	up
DUSP10	down	aca-miR-18a-3p_L+1R-1_1ss12GA	up
DUSP10	down	aca-miR-363-3p_R+1	up
DUSP10	down	oan-miR-363-3p_R+1	up
MSMO1	down	aca-miR-363-3p_R+1	up
MSMO1	down	gga-miR-106-3p_R+1	up
MSMO1	down	oan-miR-363-3p_R+1	up
RARRES1	down	gga-miR-181b-5p_R+1	up
THBS1	down	gga-miR-3529	up
ACADSB	down	aca-miR-18a-3p_L+1R-1_1ss12GA	up
ACADSB	down	gga-miR-301a-5p_L+2	up
ACADSB	down	gga-miR-3529	up
NFKBIE	down	gga-miR-456-3p_1ss22AT	up
NFKBIE	down	gga-miR-456-3p_R-1	up
NFKBIE	down	pma-miR-456_R+1_1ss22AC	up
GLCCI1	down	gga-miR-6710-3p_R+1	up
SCIN	down	gga-miR-107-3p_R-1	up
SCIN	down	gga-miR-130b-5p_R-1	up
SCIN	down	gga-miR-181b-5p_R+1	up
SCIN	down	gga-miR-301a-5p_L+2	up
SCIN	down	gga-miR-456-3p_1ss22AT	up
SCIN	down	gga-miR-456-3p_R-1	up
SCIN	down	gga-miR-489-3p_L-2R+1	up
SCIN	down	pma-miR-456_R+1_1ss22AC	up
SLC24A4	down	gga-miR-20b-5p	up
DHCR24	down	aca-miR-18a-3p_L+1R-1_1ss12GA	up

DHCR24	down	gga-miR-130b-5p_R-1	up
DHCR24	down	gga-miR-202-5p_L-1	up
DHCR24	down	gga-miR-6710-3p_R+1	up
EREG	down	aca-miR-363-5p	up
EREG	down	gga-miR-130b-5p_R-1	up
FAM126A	down	gga-miR-106-3p_R+1	up
SIPA1L2	down	gga-miR-130b-5p_R-1	up
HHIPL1	down	gga-miR-489-3p_L-2R+1	up
PLCL2	down	gga-miR-202-5p_L-1	up
KAT2B	down	gga-miR-181b-5p_R+1	up
KAT2B	down	gga-miR-20b-5p	up
KAT2B	down	gga-miR-456-3p_1ss22AT	up
KAT2B	down	gga-miR-456-3p_R-1	up
KAT2B	down	pma-miR-456_R+1_1ss22AC	up
BAG5	down	gga-miR-1559-3p	up
BAG5	down	gga-miR-181b-5p_R+1	up
CLEC3B	down	PC-3p-68190_58	up
CCR8	down	aca-miR-363-3p_R+1	up
CCR8	down	oan-miR-363-3p_R+1	up
CAMK2D	down	gga-miR-128-1-5p	up
CAMK2D	down	gga-miR-1559-3p	up
CAMK2D	down	gga-miR-181b-5p_R+1	up
DTX3L	down	gga-miR-1559-3p	up
DTX3L	down	gga-miR-20b-5p	up
RAB32	down	gga-miR-107-3p_R-1	up
ISCA1	down	PC-3p-68190_58	up

ISCA1	down	aca-miR-363-3p_R+1	up
ISCA1	down	gga-miR-202-5p_L-1	up
ISCA1	down	oan-miR-363-3p_R+1	up
CTSL2	down	gga-miR-181b-5p_R+1	up
RBM24	down	gga-miR-106-3p_R+1	up
RBM24	down	gga-miR-107-3p_R-1	up
RBM24	down	gga-miR-1559-3p	up
SERPINB6	down	gga-miR-1451-3p_R-1	up
CCDC127	down	aca-miR-363-3p_R+1	up
CCDC127	down	gga-miR-106-3p_R+1	up
CCDC127	down	gga-miR-456-3p_1ss22AT	up
CCDC127	down	gga-miR-456-3p_R-1	up
CCDC127	down	oan-miR-363-3p_R+1	up
CCDC127	down	pma-miR-456_R+1_1ss22AC	up
RHBG	down	gga-miR-107-3p_R-1	up
ANKRA2	down	gga-miR-489-3p_L-2R+1	up
SYTL2	down	gga-miR-20b-5p	up
TEC	down	gga-miR-107-3p_R-1	up
TEC	down	gga-miR-6710-3p_R+1	up
CD38	down	gga-miR-107-3p_R-1	up
MBLAC2	down	aca-miR-18a-3p_L+1R-1_1ss12GA	up
MBLAC2	down	gga-miR-130b-5p_R-1	up
MBLAC2	down	gga-miR-181b-5p_R+1	up
RFESD	down	gga-miR-1559-3p	up
C7	down	aca-miR-363-5p	up
PARP8	down	PC-3p-68190_58	up

WDR1	down	gga-miR-106-3p_R+1	up
F2RL1	down	gga-miR-107-3p_R-1	up
F2RL1	down	gga-miR-1559-3p	up
F2RL1	down	gga-miR-6710-3p_R+1	up
ABHD17B	down	gga-miR-106-3p_R+1	up
PCMTD1	down	gga-miR-107-3p_R-1	up
PCMTD1	down	gga-miR-130b-5p_R-1	up
PCMTD1	down	gga-miR-1559-3p	up
PCMTD1	down	gga-miR-20b-5p	up
TMEM68	down	aca-miR-363-5p	up
TMEM68	down	gga-miR-181b-5p_R+1	up
FABP5	down	aca-miR-363-3p_R+1	up
FABP5	down	gga-miR-1559-3p	up
FABP5	down	gga-miR-20b-5p	up
FABP5	down	gga-miR-456-3p_1ss22AT	up
FABP5	down	gga-miR-456-3p_R-1	up
FABP5	down	oan-miR-363-3p_R+1	up
FABP5	down	pma-miR-456_R+1_1ss22AC	up
IFNAR1	down	gga-miR-106-3p_R+1	up
IFNAR1	down	gga-miR-20b-5p	up
CDH17	down	gga-miR-106-3p_R+1	up
TP53INP1	down	aca-miR-18a-3p_L+1R-1_1ss12GA	up
TP53INP1	down	gga-miR-20b-5p	up
MORC3	down	aca-miR-18a-3p_L+1R-1_1ss12GA	up
MORC3	down	gga-miR-6710-3p_R+1	up
SIK1	down	aca-miR-363-3p_R+1	up

SIK1	down	gga-miR-130b-5p_R-1	up
SIK1	down	gga-miR-20b-5p	up
SIK1	down	oan-miR-363-3p_R+1	up
MAOA	down	aca-miR-363-5p	up
FBXO9	down	aca-miR-18a-3p_L+1R-1_1ss12GA	up
FBXO9	down	gga-miR-107-3p_R-1	up
FBXO9	down	gga-miR-130b-5p_R-1	up
FBXO9	down	gga-miR-1451-3p_R-1	up
FBXO9	down	gga-miR-202-5p_L-1	up
FDFT1	down	aca-miR-18a-3p_L+1R-1_1ss12GA	up
RGN	down	PC-3p-68190_58	up
SLC9A7	down	gga-miR-181b-5p_R+1	up
CHST10	down	PC-3p-68190_58	up
CHST10	down	aca-miR-18a-3p_L+1R-1_1ss12GA	up
CHST10	down	gga-miR-106-3p_R+1	up
RASA3	down	gga-miR-20b-5p	up
GAS6	down	gga-miR-181b-5p_R+1	up
IRG1	down	gga-miR-107-3p_R-1	up
IRG1	down	gga-miR-202-5p_L-1	up
LCP1	down	gga-miR-130b-5p_R-1	up
SUCLA2	down	gga-miR-106-3p_R+1	up
SLC46A3	down	gga-miR-107-3p_R-1	up
SLC46A3	down	gga-miR-20b-5p	up
SLC46A3	down	gga-miR-456-3p_1ss22AT	up
SLC46A3	down	gga-miR-456-3p_R-1	up
SLC46A3	down	pma-miR-456_R+1_1ss22AC	up

ARL6IP5	down	gga-miR-1559-3p	up
COTL1	down	PC-3p-68190_58	up
LYN	down	gga-miR-106-3p_R+1	up
LYN	down	gga-miR-130b-5p_R-1	up
DPT	down	PC-3p-68190_58	up
PACSIN2	down	gga-miR-181b-5p_R+1	up
MICAL1	down	gga-miR-107-3p_R-1	up
C3H2ORF43	down	aca-miR-363-5p	up
C3H2ORF43	down	gga-miR-1451-3p_R-1	up
IFNGR1	down	gga-miR-1559-3p	up
IFNGR1	down	gga-miR-181b-5p_R+1	up
RSFR	down	gga-miR-107-3p_R-1	up
MPZL3	down	gga-miR-130b-5p_R-1	up
IFI27L2	down	gga-miR-106-3p_R+1	up
BIRC2	down	gga-miR-6710-3p_R+1	up
PDLIM5	down	aca-miR-363-5p	up
PDLIM5	down	gga-miR-20b-5p	up
RNASET2	down	gga-miR-106-3p_R+1	up
RNASET2	down	gga-miR-130b-5p_R-1	up
RNASET2	down	gga-miR-6710-3p_R+1	up
REEP5	down	gga-miR-1559-3p	up
HOPX	down	gga-miR-181b-5p_R+1	up
HOPX	down	gga-miR-489-3p_L-2R+1	up
WIPF1	down	gga-miR-106-3p_R+1	up
WIPF1	down	gga-miR-107-3p_R-1	up
WIPF1	down	gga-miR-130b-5p_R-1	up

DOCK4	down	gga-miR-20b-5p	up
CD151	down	PC-3p-68190_58	up
CD151	down	gga-miR-130b-5p_R-1	up
LSP1	down	gga-miR-107-3p_R-1	up
LDHA	down	gga-miR-106-3p_R+1	up
LDHA	down	gga-miR-20b-5p	up
NBL1	down	gga-miR-106-3p_R+1	up
NBL1	down	gga-miR-20b-5p	up
CYP2H1	down	aca-miR-363-3p_R+1	up
CYP2H1	down	gga-miR-456-3p_1ss22AT	up
CYP2H1	down	gga-miR-456-3p_R-1	up
CYP2H1	down	oan-miR-363-3p_R+1	up
CYP2H1	down	pma-miR-456_R+1_1ss22AC	up
REL	down	gga-miR-106-3p_R+1	up
REL	down	gga-miR-130b-5p_R-1	up
LIMD2	down	gga-miR-301a-5p_L+2	up
ANG	down	PC-3p-68190_58	up
ANG	down	gga-miR-107-3p_R-1	up
CAMK4	up	cin-miR-33_R+4	down
CHTF8	up	gga-miR-146a-3p_R-1	down
SLC25A1	up	gga-miR-146a-3p_R-1	down
GFRA2	up	cin-miR-33_R+4	down
HSPA9	up	cin-miR-33_R+4	down
KIF20A	up	cin-miR-33_R+4	down
AIMP2	up	gga-miR-458a-3p	down
DDX50	up	gga-let-7j-3p_L-1R+2	down

COL18A1	up	gga-let-7j-3p_L-1R+2	down
TBL3	up	gga-let-7j-3p_L-1R+2	down
MMP11	up	gga-miR-125b-5p	down
MCM2	up	gga-miR-125b-5p	down
MCM2	up	gga-miR-1729-5p_R-1	down
KIF11	up	gga-let-7j-3p_L-1R+2	down
NPM3	up	gga-miR-146a-3p_R-1	down
ABHD12	up	gga-miR-146a-3p_R-1	down
SMOC1	up	gga-let-7j-3p_L-1R+2	down
IFRD1	up	gga-let-7j-3p_L-1R+2	down
COL1A2	up	gga-let-7b_R+2	down
COL1A2	up	mmu-let-7j_1ss8TG	down
DTL	up	rno-miR-122-5p_L+1R+1_1	down
DTL	up	rno-miR-122-5p_L+1R+1_2	down
PRDX1	up	cin-miR-33_R+4	down
TSPAN1	up	cin-miR-33_R+4	down
IGF2BP3	up	gga-let-7b_R+2	down
IGF2BP3	up	gga-miR-1729-5p_R-1	down
IGF2BP3	up	mmu-let-7j_1ss8TG	down
UHRF1BP1L	up	cin-miR-33_R+4	down
MAD2L1	up	gga-let-7j-3p_L-1R+2	down
AKR1D1	up	gga-let-7b_R+2	down
AKR1D1	up	mmu-let-7j_1ss8TG	down
DENND5B	up	gga-miR-146a-3p_R-1	down
BPGM	up	cin-miR-33_R+4	down
CYB5A	up	gga-miR-146a-3p_R-1	down

LPCAT3	up	rno-miR-122-5p_L+1R+1_1	down
LPCAT3	up	rno-miR-122-5p_L+1R+1_2	down
RAD21	up	gga-miR-458a-3p	down
TINAG	up	gga-let-7j-3p_L-1R+2	down
REPS2	up	gga-let-7j-3p_L-1R+2	down
RCBTB1	up	cin-miR-33_R+4	down
CKAP2	up	aca-miR-191-5p_R-1	down
HIGD1A	up	gga-miR-146a-3p_R-1	down
SUCLG1	up	rno-miR-122-5p_L+1R+1_1	down
SUCLG1	up	rno-miR-122-5p_L+1R+1_2	down
MCM6	up	rno-miR-122-5p_L+1R+1_1	down
MCM6	up	rno-miR-122-5p_L+1R+1_2	down
NFYC	up	gga-miR-458a-3p	down
LPL	up	gga-miR-458a-3p	down
GATM	up	gga-let-7b_R+2	down
GATM	up	mmu-let-7j_1ss8TG	down
ARPP19	up	cin-miR-33_R+4	down
ARPP19	up	gga-miR-1729-5p_R-1	down

Table S15. Quality control parameters of the miRNA sequencing and mRNA sequencing in the present study.

Item		Mature liver			postnatal liver		
		AC1	AC2	AC3	YC1	YC2	YC3
mRNA sequencing	Q20 (%)	99.5	99.52	99.49	99.53	99.45	99.5
	Q30 (%)	95.19	95.2	94.57	95.44	94.47	94.68
	GC content (%)	51	52.5	50	50	51	50
miRNA sequencing	Q20 (%)	98.95	99.05	99.07	99.01	99.04	98.94
	Q30 (%)	97.96	98.1	98.25	98.01	98.21	97.96
	GC content (%)	53.23	52.26	50.76	52.36	51.24	52.53

Table S16. Primer sequences for PCR reactions in luciferase reporter study.

Gene name	Forward primer	Reverse primer
IGFB3-3'UTR	ccgCTCGAGCCTTGTGGCTTAGGGTCTGG	attGCGGCCGCAAGAAGGGGAAACACTGCCC
RAD21-3'UTR	ccgCTCGAGTTTATGAAGCGGGCGTATG	attGCGGCCGCAAATGGGACTTCCTTTGCA

Figure S1.

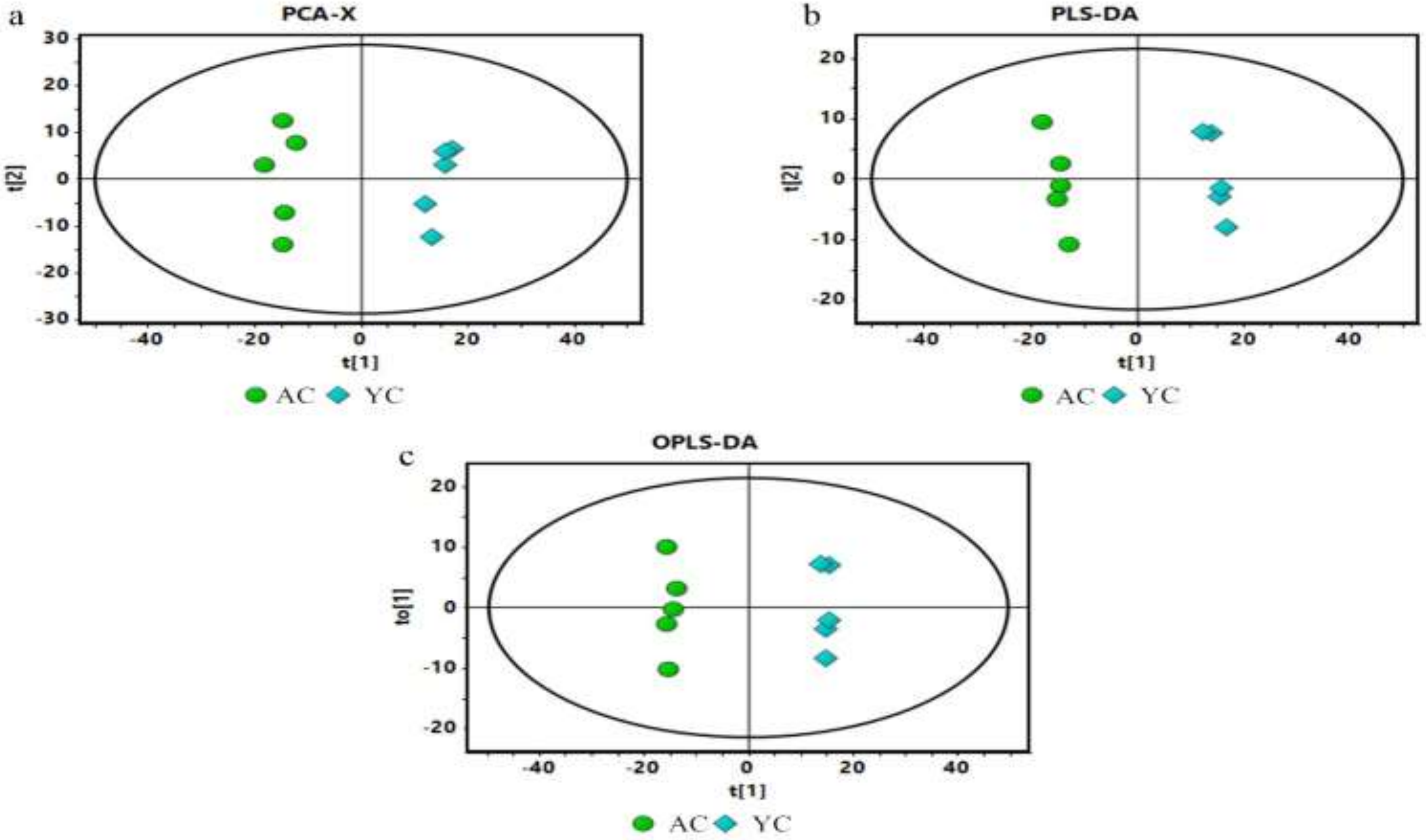

Figure S2.

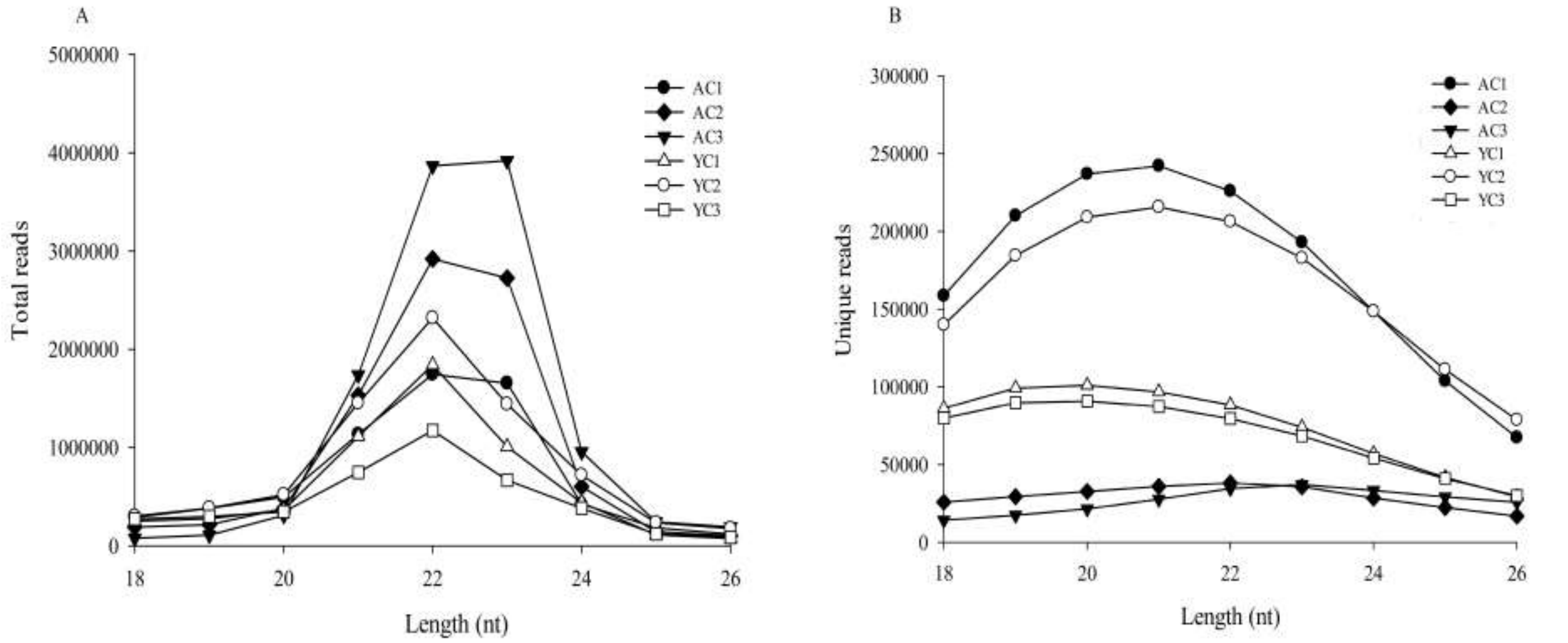
A
Total reads
5000000
4000000
3000000
2000000
1000000
0
18
20
22
24
26
Length (nt)
AC1
AC2
AC3
YC1
YC2
YC3
B
Unique reads
300000
250000
200000
150000
100000
50000
0
18
20
22
24
26
Length (nt)
AC1
AC2
AC3
YC1
YC2
YC3

Figure S3.

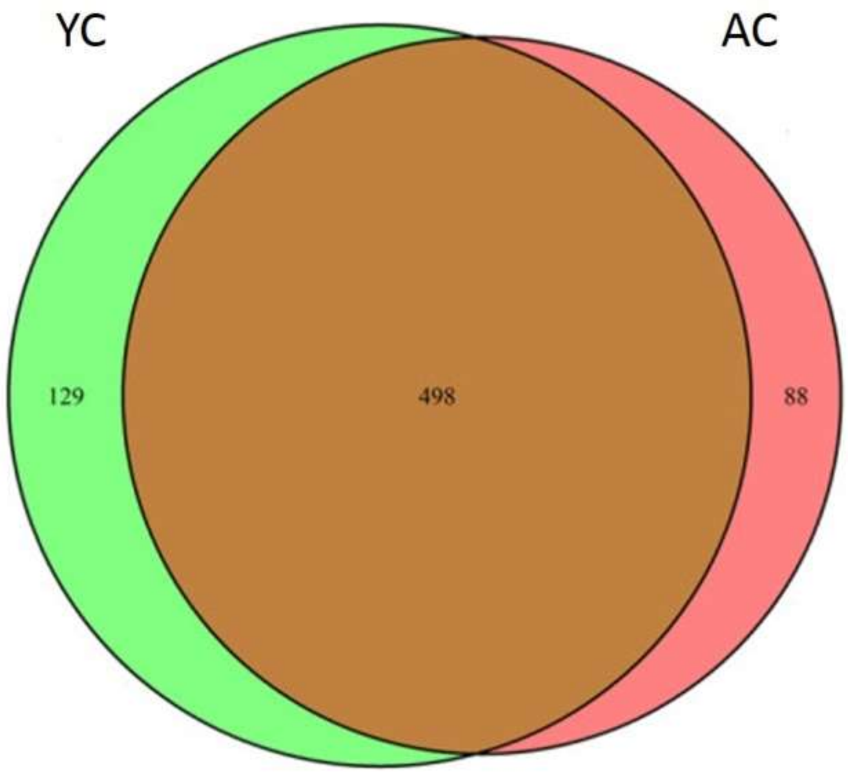

Supplement: Supplementary file 1 — Supplementary Information [file 41598_2017_18674_MOESM1_ESM.pdf]
